# Blood pressure, plasma proteins, and cardiovascular diseases: a network Mendelian randomization and observational study

**DOI:** 10.1093/eurheartj/ehaf725

**Published:** 2025-10-09

**Authors:** Devendra Meena, Jingxian Huang, Alexander Smith, James Yarmolinsky, Siwei Wu, Fotios Koskeridis, Yi-Hsuan Ko, Marie-Joe Dib, Charalabos Antonatos, Yiannis Vasilopoulos, Xinzhu Yu, Georg W Otto, Dipender Gill, Manuel Mayr, Paul Elliott, Abbas Dehghan, Ioanna Tzoulaki

**Affiliations:** Department of Epidemiology and Biostatistics, School of Public Health, Imperial College London, White City Campus, 90 Wood Lane, London W12 0BZ, UK; Department of Epidemiology and Biostatistics, School of Public Health, Imperial College London, White City Campus, 90 Wood Lane, London W12 0BZ, UK; Department of Epidemiology and Biostatistics, School of Public Health, Imperial College London, White City Campus, 90 Wood Lane, London W12 0BZ, UK; Department of Epidemiology and Biostatistics, School of Public Health, Imperial College London, White City Campus, 90 Wood Lane, London W12 0BZ, UK; Department of Epidemiology and Biostatistics, School of Public Health, Imperial College London, White City Campus, 90 Wood Lane, London W12 0BZ, UK; Department of Epidemiology and Biostatistics, School of Public Health, Imperial College London, White City Campus, 90 Wood Lane, London W12 0BZ, UK; Department of Epidemiology and Biostatistics, School of Public Health, Imperial College London, White City Campus, 90 Wood Lane, London W12 0BZ, UK; Division of Cardiovascular Medicine, Hospital of the University of Pennsylvania, Philadelphia, PA, USA; Laboratory of Genetics, Section of Genetics, Cell Biology and Development, Department of Biology, University of Patras, Patras, Greece; Laboratory of Genetics, Section of Genetics, Cell Biology and Development, Department of Biology, University of Patras, Patras, Greece; Department of Epidemiology and Biostatistics, School of Public Health, Imperial College London, White City Campus, 90 Wood Lane, London W12 0BZ, UK; Department of Epidemiology and Biostatistics, School of Public Health, Imperial College London, White City Campus, 90 Wood Lane, London W12 0BZ, UK; UK Dementia Research Institute at Imperial College London, White City Campus, 86 Wood Lane, London W12 0BZ, UK; Department of Epidemiology and Biostatistics, School of Public Health, Imperial College London, White City Campus, 90 Wood Lane, London W12 0BZ, UK; National Heart & Lung Institute, Imperial College London, London, UK; British Heart Foundation Centre of Research Excellence, Imperial College London, London SW7 2AZ, UK; Department of Epidemiology and Biostatistics, School of Public Health, Imperial College London, White City Campus, 90 Wood Lane, London W12 0BZ, UK; UK Dementia Research Institute at Imperial College London, White City Campus, 86 Wood Lane, London W12 0BZ, UK; Department of Epidemiology and Biostatistics, School of Public Health, Imperial College London, White City Campus, 90 Wood Lane, London W12 0BZ, UK; UK Dementia Research Institute at Imperial College London, White City Campus, 86 Wood Lane, London W12 0BZ, UK; British Heart Foundation Centre of Research Excellence, Imperial College London, London SW7 2AZ, UK; Department of Epidemiology and Biostatistics, School of Public Health, Imperial College London, White City Campus, 90 Wood Lane, London W12 0BZ, UK; UK Dementia Research Institute at Imperial College London, White City Campus, 86 Wood Lane, London W12 0BZ, UK; British Heart Foundation Centre of Research Excellence, Imperial College London, London SW7 2AZ, UK; Centre for Systems Biology, Biomedical Research Foundation, Academy of Athens, 4 Soranou Ephessiou, Athens 115 27, Greece

**Keywords:** Proteomics, Blood pressure, Mendelian randomization, Cardiovascular disease

## Abstract

**Background and Aims:**

The biological pathways leading to elevated blood pressure (BP) and subsequent cardiovascular diseases (CVDs) remain incompletely understood. Investigating the proteomic landscape of BP and its overlap with CVD could provide critical insights into the molecular determinants and pathways involved in BP regulation and its subsequent effect on CVD.

**Methods:**

A proteome-wide Mendelian randomization (MR) study was conducted by leveraging genetic instruments from 2007 plasma proteins to assess their causal effects on BP (systolic and diastolic BP). Proteins showing strong associations with BP were further analyzed for potential causal effects on coronary artery disease (CAD) and stroke subtypes. Network MR was performed to estimate the proportion of CVD risk mediated through BP. Bayesian colocalization was applied to determine whether identified associations share common causal variants. Observational associations were examined in UK Biobank participants to assess associations between proteins, BP, and incident CVD events using linear regression and Cox proportional hazard models.

**Results:**

Proteome-wide MR identified 242 proteins associated with BP, of which 48 were also linked to CAD or stroke, with four (ACOX1, FGF5, FURIN, MST1) also supported by genetic colocalization analyses (FDR 5% and PP ≥70%). Genetically predicted FURIN and FGF5 were strongly associated with BP and stroke risk, while ACOX1, FGF5, and MST1 exhibited potential causal effects on CAD. Network MR suggested that a substantial proportion of their effect on CAD and stroke (30.5%–77.2%) was mediated through BP regulation. Observational analyses further supported these findings.

**Conclusions:**

This study identifies key plasma proteins with potential causal roles in BP regulation and CVD risk, highlighting BP as a major mediator of their effects on CAD and stroke. These findings provide novel insights into the molecular mechanisms underlying hypertension-related CVD and identify promising protein targets for further investigation.

## Structured Graphical Abstract

**Structured Graphical Abstract:**
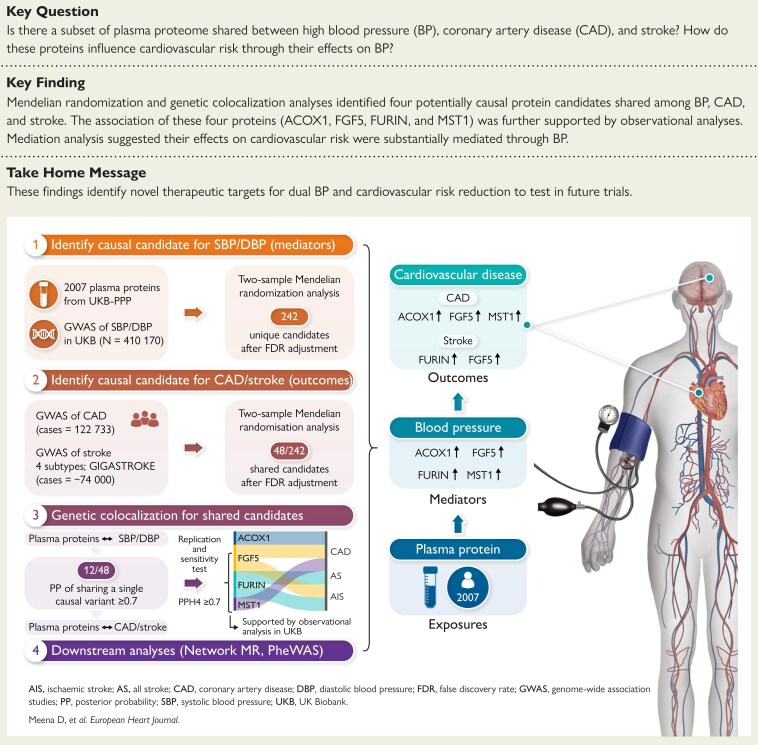
Blood pressure, circulating plasma proteins, and cardiovascular diseases. AIS, all ischaemic strokes; AS, all strokes; CAD, coronary artery disease; DBP, diastolic blood pressure; FDR, false discovery rate; GWAS, genome-wide association studies; MR, Mendelian randomization; PP, posterior probability; PPP, Pharma Proteomics Project; SBP, systolic blood pressure; UKB, UK Biobank.

**See the editorial comment for this article ‘Identification of plasma protein mediators of blood pressure and cardiovascular disease: what are the next steps?’, by P. B. Munroe *et al*, https://doi.org/10.1093/eurheartj/ehaf530**.

## Introduction

Elevated blood pressure (BP) is a leading modifiable risk factor for premature death, cardiovascular disease (CVD), and all-cause mortality, affecting an estimated 1.28 billion adults aged 30–79 years worldwide.^1,2^ Even small increases in BP are associated with a higher risk of CVD; for instance, each 10 mmHg increase in systolic blood pressure (SBP) is linked to a 45% higher risk of coronary artery disease (CAD) and ∼65% higher risk of stroke.^3,4^

Substantial epidemiological evidence highlights shared lifestyle and genetic risk factors between BP and CVD. However, the molecular pathways linking elevated BP and its progression toward CVDs remain incompletely understood. Elucidating these pathways is crucial for identifying new therapeutic targets and developing more effective strategies to mitigate the burden of BP-related CVD. Proteomics, the rapidly evolving field of studying proteins at large scale, offers a powerful approach to uncovering these pathways by identifying molecular targets that may serve as key regulators of cellular signalling and function and represent a major source of therapeutic targets for many diseases.^5,6^ In the context of BP, they may act as mediators of high BP (e.g. through vasoconstriction or vascular remodelling) or as plasma-based markers of cellular dysfunction linked to hypertensive physiology. Investigating the proteomic landscape of BP and its overlap with CVD could, therefore, provide critical insights into the molecular determinants and pathways involved in BP regulation and its subsequent effect on CVDs^7–10.^

In this study, we systematically evaluated the proteomic determinants of BP through a comprehensive proteome-wide Mendelian randomization (MR) analysis. MR leverages genetic variants as instrumental variables (IVs) to assess potential causal relationships between exposures (e.g. protein abundance) and outcomes (e.g. BP and CVD). This approach is analogous to randomized controlled trials, as genetic variants are randomly allocated at conception, helping to mitigate confounding and reverse causation.^11^ Recent large-scale proteogenomic studies have identified thousands of genetic variants associated with protein abundance (protein quantitative trait loci, or pQTLs), offering a unique opportunity to apply MR to investigate the causal roles of plasma-based markers in disease pathways.^12^ To further investigate whether the plasma-based proteomic determinants of BP are also associated with CVDs, we extended our analysis to include CAD and stroke and performed network MR to assess the potential mediating role of BP in linking BP-associated proteins to CVDs. Extensive downstream analyses, including Bayesian colocalization, observational analysis using Cox regression models, and phenome-wide association studies (PheWAS), were applied to substantiate the robustness of our findings and their relevance to other outcomes. Overall, our work prioritizes plasma-based biomarkers for BP-related CVDs, enhances the understanding of their molecular underpinnings, and informs the development of improved prevention and treatment strategies to reduce the CVD burden through BP management.

## Methods

**Figure 1:**
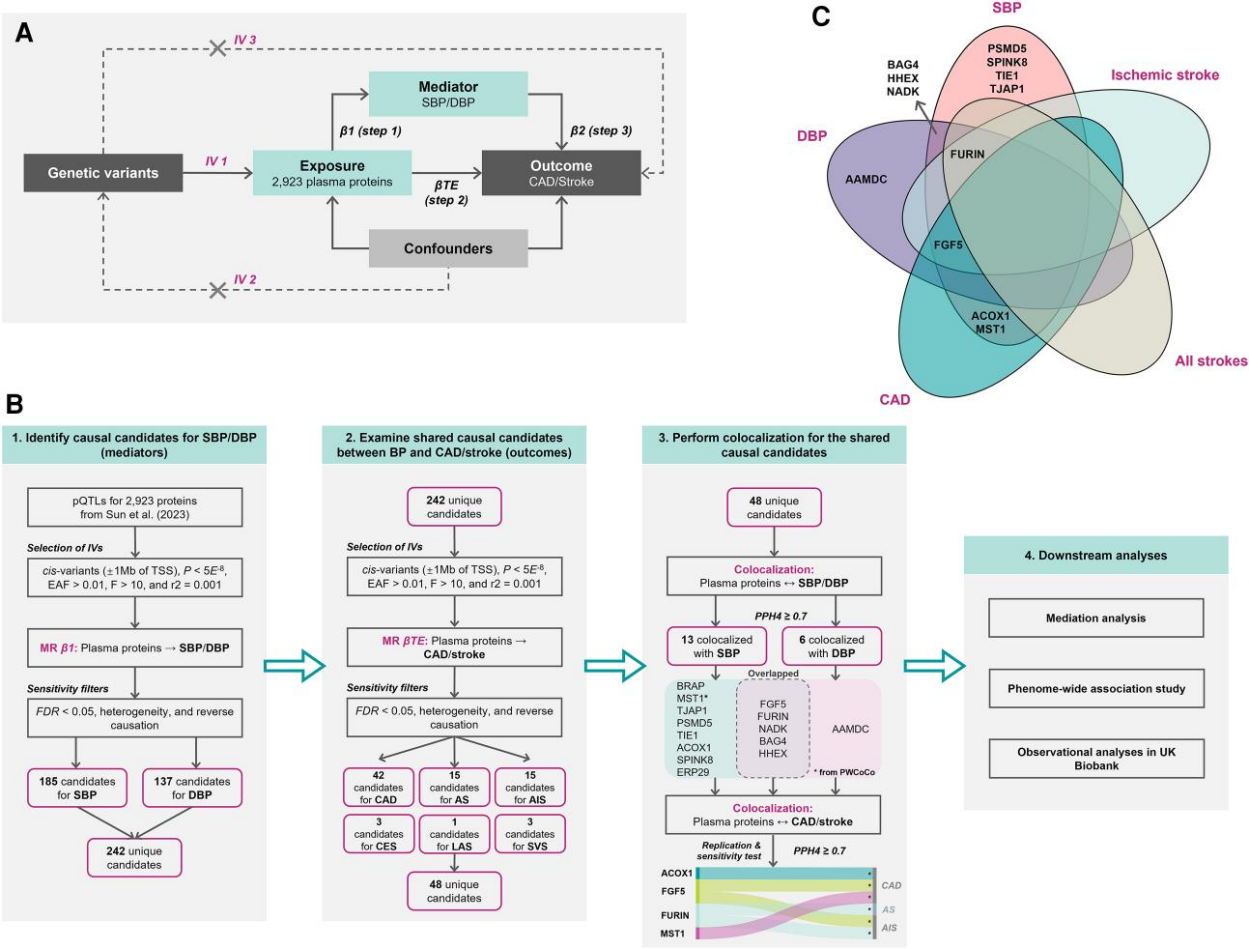
Study design. (*A*) Conceptual framework of Mendelian randomization. (*B*) Overview of the study design and main findings. (*C*) Venn diagram showing colocalized BP-associated proteins shared with CAD and stroke. AIS, all ischaemic strokes; AS, all strokes; CAD, coronary artery disease; CES, cardioembolic stroke; LAS, large artery stroke; SVS, small vessel stroke

As outlined in *Figure 1*, we began with a proteome-wide MR analysis using *cis*-pQTLs for 2923 plasma proteins to assess their potential causal effects on SBP and DBP. Genetically predicted proteins showing strong associations with BP were then evaluated for potential causal effects on CAD and stroke subtypes using the same MR framework. For genetically predicted proteins associated with both BP and CVD outcomes, Bayesian colocalization was performed to assess whether shared genetic variants underlie these associations. Proteins with strong MR and colocalization evidence were carried forward for network MR to estimate the proportion of CVD risk mediated by BP. We triangulated these results with observational analyses in UK Biobank (UKB) participants, examining the associations between protein levels, BP medication use, BP levels, and incident CVD events. Finally, PheWAS analyses were conducted for the lead genetic variants to assess pleiotropy across a broad range of traits.

### Proteome-wide Mendelian randomization

#### Genome-wide association study data sources for proteins, blood pressure, and cardiovascular disease outcomes

Genetic variants associated with 2923 unique plasma proteins in up to ∼54 219 participants, primarily of European ancestry, were obtained from publicly available genome-wide association study (GWAS) data through the UKB Pharma Proteomics Project (UKB-PPP).^13^ UKB-PPP is a precompetitive consortium of 13 biopharmaceutical companies funding the generation of blood-based proteomic data in a subset of UKB participant. Summary statistics of GWAS for all stroke (AS; *N* = 73 652 cases and 1 234 808 controls) and four subtypes including ischaemic stroke (AIS; *N* = 62 100 cases and 1 234 808 controls), cardioembolic stroke (CES; *N* = 10 804 cases and 1 234 808 controls), large artery stroke (LAS; *N* = 6399 cases and 1 234 808 controls), and small vessel stroke (SVS; *N* = 6811 cases and 1 234 808 controls) were obtained from the GIGASTROKE consortium.^14^ Genome-wide association studies data for CAD (*N* = 122 733 cases and *N* = 547 261 controls) were obtained from the meta-analysis by van der Harst *et al*.^15^ Further details of all GWAS datasets used in this analysis can be found in Supplementary data online, *Table S1*.

Previous BP GWAS^16,17^ included the UKB data and adjusted for body mass index (BMI) as covariate in the GWAS model, which may introduce bias due to sample overlap^18^ and collider bias in MR,^19^ respectively. To address these, we performed a GWAS of SBP [mean (SD) = 141.09 (20.64); mmHg] and DBP [mean (SD) = 84.28 (11.24); mmHg] in up to 410 170 Europeans from the UKB excluding the subsample of UKB-PPP [mean age (SD) = 56.7 (8.01); *N* = ∼54 219]. Briefly, UKB is a prospective cohort study from the UK, which contains >500 000 volunteers between 40 and 69 years of age at inclusion. The study design, sample characteristics and genome-wide genotype data have been described previously.^20^ Following informed consent, participants completed a standardized questionnaire on life course exposures, medical history and treatments, and underwent a standardized portfolio of phenotypic tests including two BP measurements taken seated after a 2 min rest using an appropriate cuff and an Omron HEM-7015IT digital BP monitor. A manual sphygmomanometer was used if the standard automated device could not be employed. Two traits (SBP and DBP) were analysed as described previously.^16^ In brief, the mean SBP and DBP values were calculated from two automated (*N* = 418 755) or two manual (*N* = 25 888) blood pressure measurements. For individuals with one manual and one automated blood pressure measurement (*N* = 13 521), the mean of these two values was used. For individuals with only one available BP measurement (*N* = 413), we used the single value. After calculating BP measurements, we adjusted for medication use by adding 15 and 10 mmHg to SBP and DBP,^16,17,21,22^ respectively, for individuals reported to be taking BP-lowering medication (*n* = 94 289). The GWAS (without BMI adjustment) was performed using BOLT-LMM software,^23^ and quality control filters were applied as described previously.^16^ Next, we systematically compared our BP GWAS with the one performed by Evangelou *et al*.^16^ to further validate our findings. We calculated the genetic correlation^24^ for SBP and DBP between the two GWAS datasets and observed a strong positive correlation for both traits: SBP (rg = 0.95, se = 0.004) and DBP (rg = 0.91, se = 0.005). These rg estimates demonstrate that the effect estimates from our BP GWAS (excluding the UKB-PPP cohort) were highly correlated with those reported in the existing BP GWAS. The studies used in our analysis were approved by their respective institutional review boards, and informed consent was provided by all participants.

#### Selection of *cis-*pQTLs as genetic instruments and Mendelian randomization analyses

To obtain genetic IVs for the 2923 plasma proteins,^13^ we extracted *cis*-acting biallelic single nucleotide polymorphisms (SNPs) (located within ±1 megabase [mb] of the corresponding gene encoding the protein; defined as *cis*-pQTL) and minor allele frequency (MAF) > 0.01. Genetic instruments were further filtered by the strength of association (*P* < 5 × 10^−8^) and clumped at a pairwise linkage disequilibrium (LD) threshold of r^2^ < 0.001 and a window of 10 000 kilobases (kb) using the TwoSampleMR R package.^25,26^ For all MR and subsequent downstream sensitivity analyses, we utilized a randomly selected reference panel of 10 000 individuals of European ancestry from the UKB for generating instruments and 1000 Genomes European ancestry individuals (Phase 3) for clumping.^27^ This reference panel provides a representative sample of the underlying population, given the substantial overlap between the GWAS we utilized and UKB participants, enabling precise LD estimation.

For MR causal estimates to be valid, the following assumptions must be met: the genetic instruments (i) are strongly associated with the exposure, (ii) are not associated with any potential confounder of the exposure–outcome association, and (iii) do not affect the outcome independently of the exposure. The inverse-variance weighted (IVW) method (for ≥2 IVs) or the Wald ratio method (for <2 IVs) was used as the primary approach for all MR analyses. Briefly, the Wald ratio method estimates the causal effect by taking the ratio of SNPs effect on outcome (BP and CVD outcomes) to SNPs effect on exposure (protein abundance), while the IVW method performs a weighted linear regression on the variant-specific ratio estimates by inverse-variance weighting and has higher statistical power when all IVs used are valid instruments.^26^ All SNPs effects were derived from GWAS summary statistics described in the previous section. For traits instrumented by >3 IVs, we performed Cochran's Q test to detect the presence of heterogeneity. Benjamini–Hochberg false discovery rate (FDR) correction was applied and an FDR-corrected *P* < .05 was considered statistically significant.^28^ For continuous outcomes, beta estimates were obtained directly from the MR estimates, while for dichotomous outcomes, odds ratios were derived by exponentiating the MR estimates. For significant associations with no evidence of heterogeneity (FDR-corrected *P* < .05, Cochran's Q > 0.05), we included additional criteria to increase the reliability of the instruments and comply with the MR assumptions. We therefore excluded proteins from downstream analyses when (i) bidirectional MR^29^ or Steiger filtering approach^30^ showed evidence for reverse causality (i.e. genetic predisposition to outcome has a putative causal effect on the protein) and (ii) F-statistic was lower than 10 from the MR analysis to avoid potential weak instrument bias.^31^

#### Network Mendelian randomization and proportion mediated

To investigate the potential mediating effects of BP on the protein-CAD/stroke associations, we applied a two-step MR strategy (network MR).^32^ Specifically, we performed two-sample *cis*- and univariable MR analyses to investigate the potential causal effect of proteins on BP (β_1_), the potential causal effect of BP on CAD/stroke (β_2_), and the potential causal effect of proteins on CAD/stroke (β_TE_). The proportion mediated was then estimated as β_1_ × β_2_/β_TE_^32^ and the standard error and 95% confidence interval (95% CI) were estimated using the delta method.^32^ We verified that β_1_, β_2_, and β_TE_ were in the same effect direction to avoid inconsistent mediation.^33^

### Genetic colocalization

To investigate whether the genetic associations between proteins, BP, and CVD outcomes share the same causal variants and are not confounded by LD, we employed a Bayesian colocalization approach.^34^ The colocalization analysis was performed on the pre-defined *cis-*region (i.e. ±1mb) of the corresponding coding gene, with low frequency and rare variants (MAF <0.01) excluded. Priors were set as default that any SNP within the colocalization window was exclusively associated with the two traits with the probability of 1 × 10^−4^ and associated with both traits with the probability of 1 × 10^−5^.^35^ Each configuration of the two traits could be assigned to one of the hypotheses; hypothesis 0: no association, hypothesis 1 and 2: only one trait is associated, hypothesis 3: both traits are associated but with different causal variants, and hypothesis 4: both traits are associated and share the same causal variant. A colocalization posterior probability of hypothesis 4 (PP) higher than 70% was considered evidence that the 2 traits colocalize in the region.^36^ The colocalization was conducted using the ‘coloc’^35^ R packages.

Furthermore, we used multi-trait colocalization to examine colocalization across the plasma protein level, BP, and CVD.^37^ The window for the multi-trait colocalization was set the same as the conventional colocalization method mentioned previously. Default priors were set so that any variant within the colocalization window had a 1 × 10⁻⁴ probability of being associated with one trait, 1 × 10⁻⁶ for two traits, and 1 × 10⁻⁷ for all three traits.^37^ The multi-trait colocalization PP >70% was considered as strong evidence of colocalization, whereas the colocalization PP > 50% was considered suggestive evidence of colocalization.^36^ The multi-trait colocalization analysis was conducted using the ‘moloc’ R package.^37^

For gene dense regions (i.e. proteins whose cognate genes are in close proximity), we performed PairWise Conditional and Colocalisation (PWCoCo)^38^ to address the single causal variant assumption of colocalization and examine independent colocalization of non-primary signals. These regions were defined among protein candidates associated with both BP and CVD outcomes as those where a gene’s ± 500 kb window overlaps with the ±500 kb window of another gene on either side of the coding region (N = 22). Additionally, to further mitigate bias due to pleiotropy, we cross-referenced the genotype-tissue expression (GTEx)^39^ and Open Targets Genetics portal^40^ for proteins which we prioritized and we determined whether the *cis*-pQTLs for these proteins (i.e. the variants used as genetic instruments for MR) were also *cis*-eQTLs (i.e. whether the same genetic variant affects the cognate gene’s expression) for the corresponding gene. Following this, pQTLs were excluded if they were identified as pleiotropic with no evidence of being an eQLT.

### Protein–protein interactions

To identify BP relevant pathways and to summarize proteins associated with BP and CVD in functional networks, we submitted genetically predicted proteins associated with both BP and CVD outcomes from the proteome-wide MR to the Search Tool for the Retrieval of Interacting Genes (STRING) database, version 12.0 to construct protein-protein interaction (PPI) networks.^41^ In the STRING database, only experimentally validated interactions reaching a confidence threshold of 0.4 were considered. We next identified communities present in the derived network using the Leiden clustering algorithm.^42^ Enrichment analysis of proteins included in each community was conducted with Enrichr,^43^ using an FDR-corrected *P* < .05 to declare significant enrichment results.

### Observational analyses

We triangulated MR findings with observational evidence obtained from UKB. To examine associations between BP treatment and plasma proteins with evidence of causality from MR analysis on BP, we leveraged the proteomics data from UKB individuals with hypertension (hypertension diagnosis prior to blood sample collection or by the use of BP medication at collection). Hypertension was defined based on the CALIBER code list for hypertension in general practitioner records or hospital episode statistics.^44^ Individual protein levels were adjusted by age and sex, and the residuals were rank inverse normalized. T-tests were used to compare adjusted protein levels between hypertensive individuals with and without prescribed BP medication. To isolate the effect of BP medication, we conducted sensitivity analysis excluding individuals taking medication for high cholesterol and diabetes. False discovery rate correction was applied, and an FDR-corrected *P* < .05 was considered statistically significant.

We also assessed the association between each of the proteins that showed strong evidence of causality and colocalization for BP and CVD with BP levels (cross-sectional analysis) and incident CVD (longitudinal analysis). To study the association between protein levels and BP, we used separate linear regression analyses with standardized levels of each protein of interest as the predictor and BP measurements as the outcome. Standardized beta coefficients for the association between protein levels and SBP are reported. Cox proportional hazard models were used to calculate hazard ratios between standardized protein levels and time-to-event of composite CVD (CAD and stroke), as well as stroke and CAD separately. For both linear regressions and Cox regressions, we ran sequential models adjusted for (a) age and genetic sex; (b) Townsend deprivation index, body mass index, smoking status; (c) LDL and HDL cholesterol, diabetes and high cholesterol medication. Separate models were run for SBP and DBP. Diagnosis of CAD and stroke was defined by patient linked general practitioner records (readcode), hospital episode statistics (ICD-10) and death records (ICD-10) within the June 2023 UKB release using the CALIBER code list^44^ for stroke and CAD code list from Patel *et al*.^45^ Participant follow-up began at the date of blood sample collection and time-to-event was set at whichever occurred first; the first instance of diagnosis, death of the participant, or censoring date (June 2023). Participants with prevalent CVD at baseline were excluded from Cox regression analyses.

### Phenome-wide association study

We conducted PheWAS analyses to assess associations linked to lead *cis*-pQTLs across various traits beyond cardiovascular disease and to identify potential opposing effects with other phenotypes. We conducted these analyses in the UKB with participants of European ancestry for the lead *cis*-pQTLs of proteins that showed strong evidence of causality (FDR <0.05) and colocalization (PP >70%). One individual from each pair of relatives (kinship coefficient >0.0884) was randomly excluded. The final sample consisted of 424 439 individuals. Cases and controls were defined using the International Classification of Diseases 9th (ICD-9) or 10th (ICD-10) Revision codes, derived from the inpatient Hospital Episode Statistics (HES) records, and translated into phecodes according to the PheWAS R package.^46^ Controls were identified as individuals without records of the respective phecode. We restricted our analysis to phecodes with at least 200 cases, as suggested previously.^47^ Logistic regression models were employed, adjusting for age, sex, and the first four genetic principal components.

#### Software

All statistical analyses were performed with R open-source software (version 4.3.2).^48^ Data harmonization, clumping, and primary MR were conducted with TwoSampleMR package (version 0.5.8).^25^ We used ‘coloc’ package (version 5.2.3)^34^ for conventional colocalization and ‘moloc’ for multi-trait colocalization.^37^ Genetic correlation was estimated using LDSC.^24^ Region genomic plots for conventional colocalization were created with the locuscomparer package (version 1.0.0)^49^ and gassocplot package (version 1.0; https://github.com/jrs95/gassocplot).

## Results

Of the 2 923 measured proteins, *cis*-region genetic association summary statistics (*cis*-pQTLs; *P* < 5 × 10^−8^) were available for 2 007 circulating proteins after removal of proteins falling within the major histocompatibility complex region (due to the complex LD in this region; *N* = 34).

### Genetically predicted proteins associated with blood pressure and cardiovascular disease

**Figure 2:**
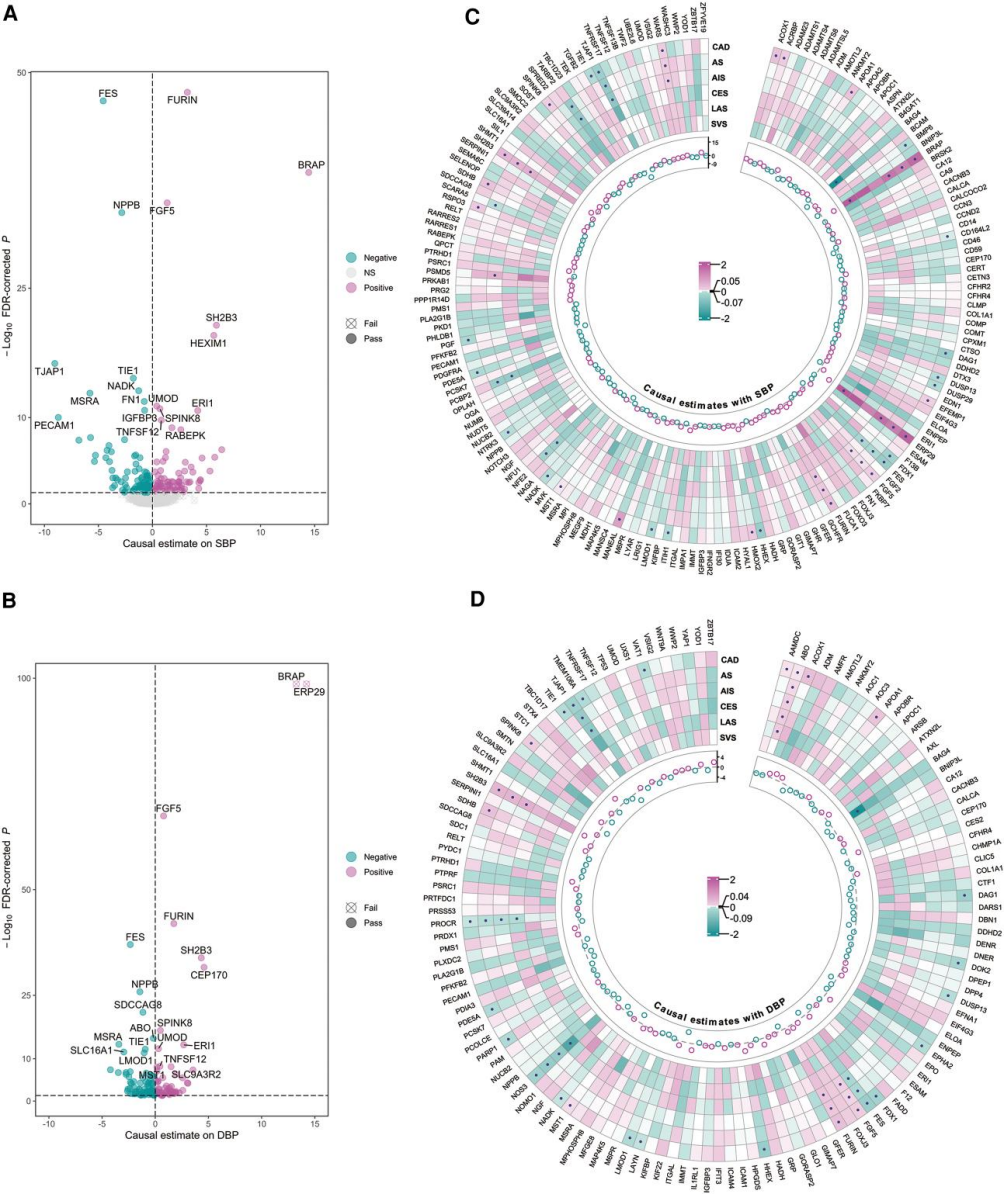
Plasma protein as potential causal candidates for systolic blood pressure (SBP) and diastolic blood pressure (DBP) and their effects on CAD and stroke outcomes. Volcano plots illustrating the potential causal effect (IVW method or Wald ratio method) of each genetically predicted protein on (*A*) SBP; (*B*) DBP. The top 20 candidates ranked by FDR-corrected *P* < .05 are labelled in the volcano plots; (*C*) Heatmap showing the proteins that passed sensitivity filters for SBP and their potential causal effect on cardiovascular outcomes; and (*D*) Heatmap showing the proteins that passed sensitivity filters for DBP and their potential causal effect on cardiovascular outcomes. Dots in the cell represent a protein-outcome association that passes the sensitivity filters (FDR < 0.05, Cochran's Q > 0.05, no reverse causation). Effect size on BP measures are in mmHg. AIS, all ischaemic strokes; AS, all strokes; CAD, coronary artery disease; CES, cardioembolic stroke; LAS, large artery stroke; SVS, small vessel stroke

Proteome-wide *cis*-MR analysis identified 242 genetically predicted proteins associated with SBP or DBP (185 for SBP, 137 for DBP; FDR < 0.05) with no evidence of heterogeneity (Cochran's Q > 0.05) or evidence for reverse causality as assessed by bidirectional MR or Steiger filtering (see Supplementary data online, *Tables S2–S4*, *Figure 2*).

Of the 242 BP-associated genetically predicted proteins, 48 (19.8%) were also associated with either CAD (42 proteins) or stroke (17 proteins) using the same criteria. Of these, 37 showed consistent direction of effects between BP and CVD traits (*Figure 2C* and *D*; Supplementary data online, *Figures S1–S10*; Supplementary data online, *Tables S5–S17*). In protein–protein interaction analyses, these proteins highlighted crosstalk between pathways including STK signalling, angiopoietin signalling, extracellular matrix (ECM) remodelling and angiogenesis (see Supplementary data online, *Figure S11*; Supplementary data online, *Table S18).*

**Figure 3:**
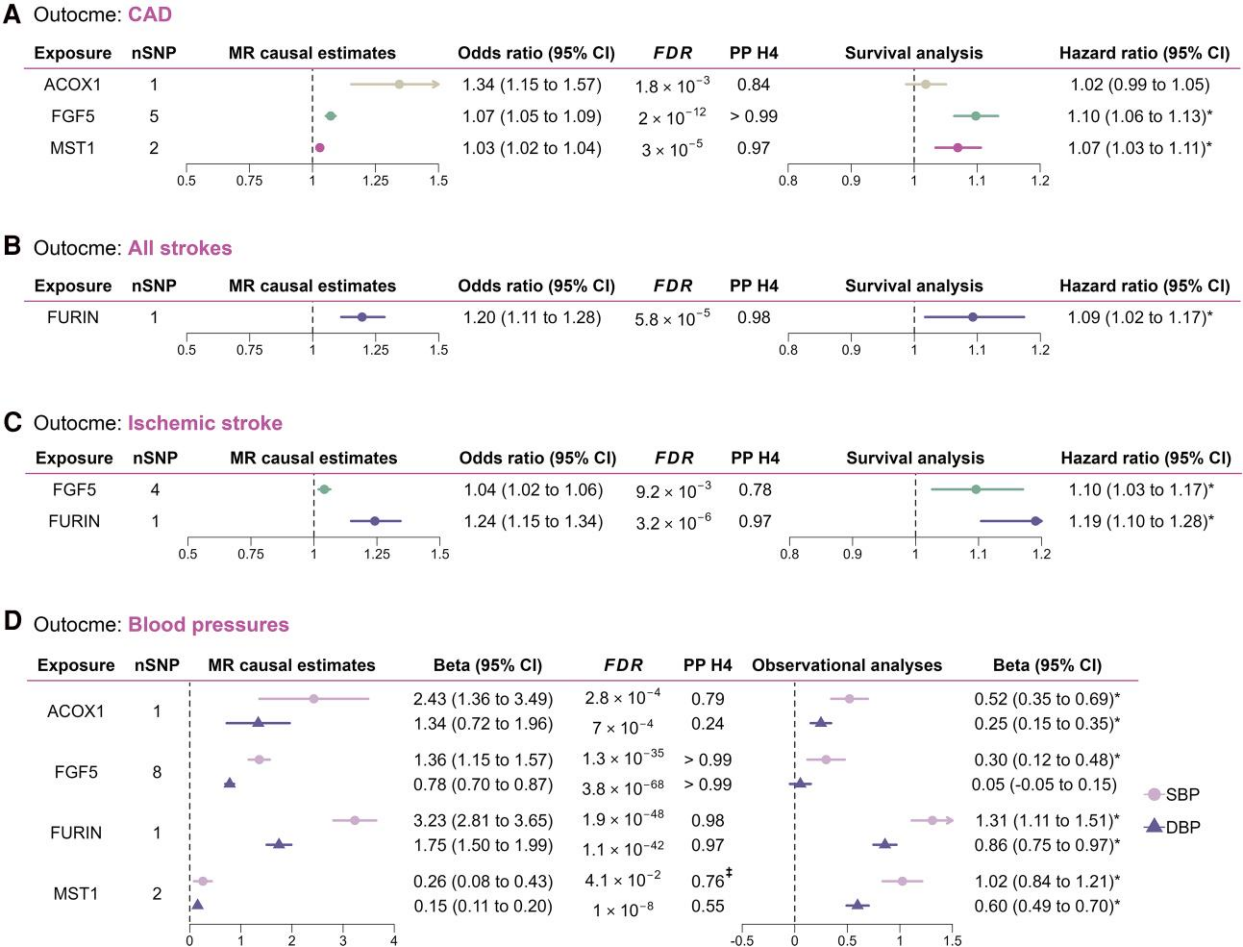
Mendelian randomisation analyses for the effect of genetically predicted plasma protein levels on cardiovascular outcomes and blood pressure measures. Forest plots illustrating the effect of prioritized plasma proteins (FDR-corrected *P* < .05 and PPH4 ≥ 70%) on (*A–C*) cardiovascular outcomes; and (*D*) blood pressure. Odds ratio refers to the genetically predicted effects of plasma protein levels on outcomes of interest, estimated from MR analysis. Effect size on BP measures are in mmHg. Hazard ratio (HR) was estimated from the Cox proportional hazard models in the observational analysis; ‡Represents a colocalized signal from the PWCoCo analysis; *Represents significantly associated proteins in the observational analysis

Subsequent colocalization analysis for those 48 proteins highlighted 13 proteins with strong evidence of sharing a single causal variant with SBP or DBP (PP ≥ 70%; Supplementary data online, *Table S19*). Of these, 5 proteins (ACOX1, BRAP, ERP29, FGF5, and FURIN) also showed strong colocalization with CAD and/or stroke and all had concordant direction of effects for BP and CAD and/or stroke (see Supplementary data online, *Figures S12 and S13*; Supplementary data online, *Table S20*). For gene dense regions (see Methods), we also performed PWCoCo which highlighted a conditionally independent signal (rs34484573) for MST1 with strong evidence of colocalization with SBP (PP = 76.4%) and CAD (PP = 96.5%; *Figure 3*; Supplementary data online, *Table S21*). Lead *cis*-pQTLs of these 6 proteins (including MST1) with robust colocalization evidence were also *cis*-eQTLs for cognate gene (GTEx) except for BRAP and ERP29, indicating that the genetic effect on protein abundance is at least partially mediated through changes in gene expression at the mRNA level (see Supplementary data online, *Table S22*). BRAP and ERP29 were excluded from downstream analysis. Multi-trait colocalization results for these signals for BP and CVD outcomes are outlined in Supplementary data online, *Figures S14*–S18 and Supplementary data online, *Table S23*. For example, FGF5 and ACOX1 showed strong evidence of colocalization with BP and CAD (PP > 95% and PP > 70%, respectively), while FURIN with BP and AS (PP > 79%). The SNPs being utilized as IVs in the proteome-wide MR analyses for the 4 prioritized proteins are listed in Supplementary data online, *Table S24*.

### Replication of blood pressure–associated proteins in ICBP consortium

We next sought to replicate the MR results of the 4 proteins in independent data from ICBP. Among them, the potentially causal effects of 3 prioritized proteins (ACOX1, FGF5, and FURIN) were replicated using an independent BP GWAS (ICBP consortium with *N* = 299 024 for DBP and *n* = 287 245 for SBP).^50^ MST1 showed comparable effect estimate and consistent direction of effect, but did not reach nominal significance (*P* = .06) (see Supplementary data online, *Figure S19*).

### Mediation analysis

**Figure 4:**
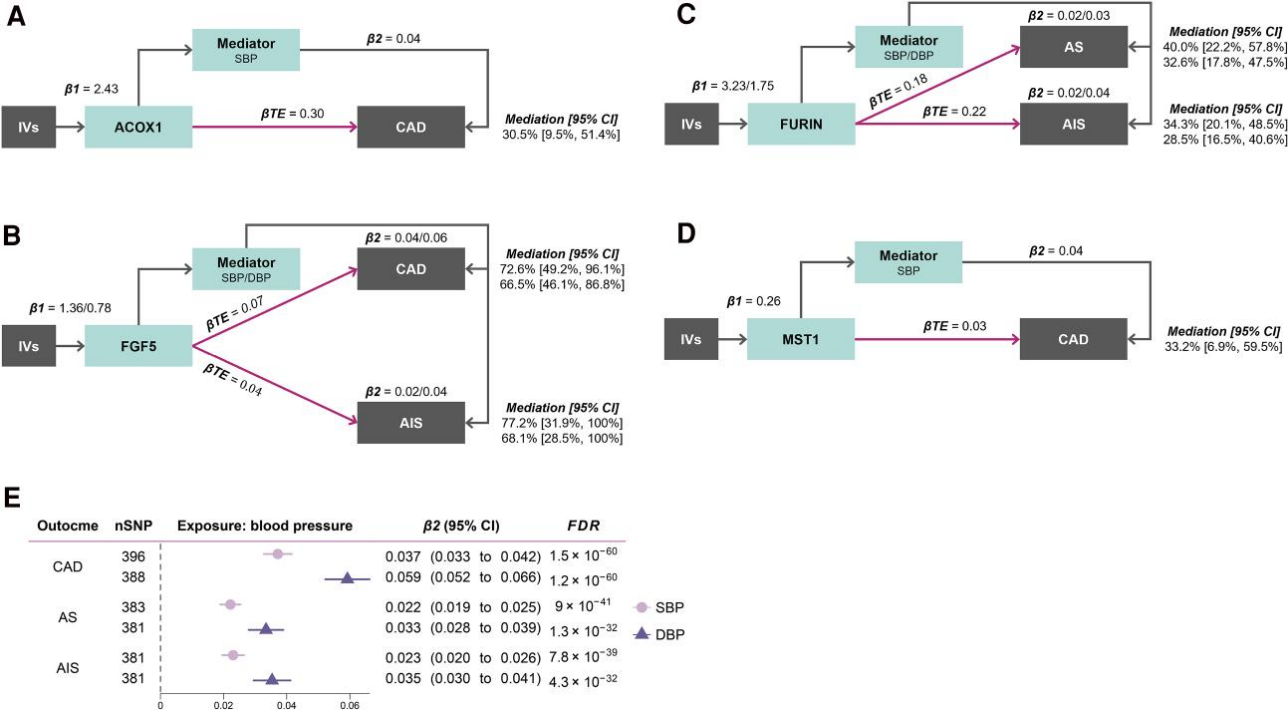
Mediation analyses showing estimated proportion of βTE from plasma proteins on CAD and stroke mediated through BP. (*A*) ACOX1 effect on CAD potentially mediated by SBP. (*B*) FGF5 effect on CVDs potentially mediated by SBP and DBP. (*C*) FURIN effect on CVDs potentially mediated by SBP and DBP. (*D*) MST1 effect on CAD potentially mediated by SBP. All effects were estimated from the MR analysis. (*E*) Effect of BP on CVDs from the network MR. Effect estimates for βTE were in logOR units. Proportion mediated was estimated as β1 × β2/βTE. 95% confidence intervals were derived using the delta method

The four proteins (ACOX1, FGF5, FURIN, and MST1) that showed strong evidence of sharing a single causal variant through colocalization with both BP and CVDs in the previous step (β_1_ and β_TE_) were carried forward for the mediation analysis. Higher genetically predicted SBP and DBP were associated with a higher risk of CAD and stroke (β_2;_  Supplementary data online, *Figures S20*–*S22*; Supplementary data online, *Table S25*). Subsequent network MR (β_1_ × β_2_/β_TE_) suggested that genetically predicted SBP and DBP potentially mediate the effect of proteins on CAD and stroke. For example, we estimated that 72.6% [95% CI = (49.2%, 96.1%)] of the effect of FGF5 on CAD may be mediated through SBP (see Supplementary data online, *Table S26*; *Figure 4*), and 77.2% [95% CI = (31.9%, 100%)] of the effect of FGF5 on AIS mediated through SBP.

### Triangulation with observational data

**Figure 5:**
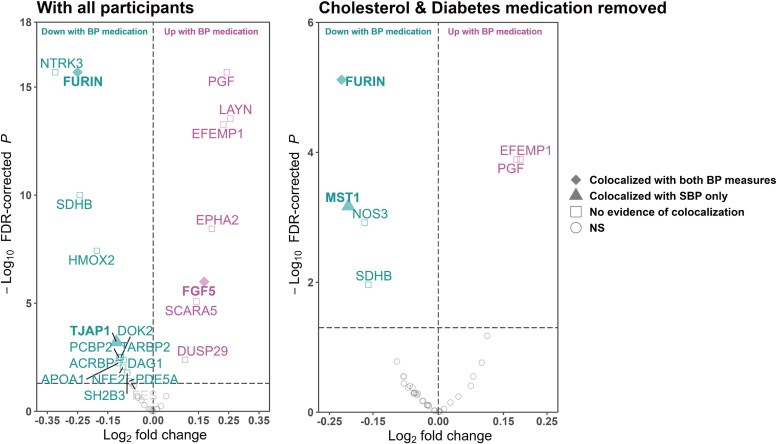
Volcano plots comparing circulating protein levels in hypertensive individuals within UK Biobank separated by BP medication usage. Candidates ranked by FDR-corrected *P* < .05 are coloured and labelled in the volcano plots

In the comparative analysis of 35 shared proteins with consistent direction of effect between the BP and CVD traits identified through the MR analysis, we found that 21 protein levels were associated with BP treatment (*Figure 5*). Regarding the four prioritized proteins, individuals on BP medications had significantly lower circulating levels of FURIN and MST1, compared to individuals with hypertension but not on BP medication, even after excluding individuals with diabetes or lipid-lowering medications (see Supplementary data online, *Table S27*; *Figure 5*).

Observational analysis using linear regression models revealed that all four proteins with robust evidence of causality and colocalization for BP and CVD were significantly associated with baseline SBP in models adjusted for multiple potential confounders (*Figure 3D*; Supplementary data online, *Table S28*). Also, in Cox proportional hazard models, all proteins were associated with CVD outcomes in minimally adjusted models (age and sex) and of them FGF5, FURIN and MST1 were associated with higher risk of composite CVD in subsequent models including a range of cardiovascular risk factors (*Figure 3A–C*, see Supplementary data online, *Tables S29* and *S30* for CVD subtypes).

### Phenome-wide association studies for genetic variants driving Mendelian randomization and colocalization

We also performed a PheWAS across 845 phenotypes, focusing on the lead *cis*-pQTLs of the four proteins prioritized in colocalization analysis. All examined lead *cis*-pQTL showed associations with a range of cardiovascular phenotypes and cardiovascular risk factors without evidence for detrimental or adverse effects in other examined phenotypes (see Supplementary data online, *Figures S23* and *S24* and Supplementary data online, *Table S31*).

## Discussion

Here, we present a comprehensive framework triangulating genetic and observational analyses highlighting the proteomic landscape of BP and its link to CVD. Through proteome-wide MR and Bayesian colocalization analysis, we prioritized 12 circulating plasma proteins with a potential causal role in BP regulation. Among these, genetically predicted levels of four proteins—ACOX1, FGF5, FURIN, and MST1—also demonstrated evidence of potential causal associations with CAD and/or stroke, supported by observational analysis. Mediation analyses further indicated that BP acts as a critical intermediary, with a substantial proportion of the effect of these proteins on CAD and/or stroke mediated through BP levels (*Structured Graphical Abstract*).

Our findings highlight acyl-CoA oxidase 1 (ACOX1) as a protein consistently associated with both BP and CAD in genetic and observational analysis, though the observational associations were attenuated after adjusting for other cardiovascular risk factors. ACOX1 is an enzyme primarily responsible for the oxidation of very-long-chain fatty acids in the peroxisome, and recent animal and human data supports its role in obesity, lipid metabolism, and insulin resistance.^51^ A liver-specific knockout of *ACOX1* has been reported to promote resistance to diet-induced obesity, inflammation, and insulin resistance, further reinforcing its metabolic significance.^51^ Additionally, common genetic variants in *ACOX1* have been associated with SBP^52^ and lipid levels,^53^ aligning with our findings. Collectively, evidence suggest that ACOX1 likely plays a pivotal role in cardiovascular pathology, with its effects partly mediated through SBP regulation.

FURIN is a peptidase that activates key proteins involved in inflammation, vascular remodelling, and lipid metabolism.^54,55^ Growing evidence supports FURIN’s role in CVD, with support from observational, proteogenomic, and basic science studies.^56,57^ FURIN is expressed in arterial tissue, and cis-pQTL SNPs have been linked to coronary heart disease (CHD) and cardiovascular risk factors, further implicating its role in vascular health.^57^ Here we provide additional evidence supporting a potential causal association between FURIN and stroke, with approximately 40% of its effect mediated through BP regulation. Notably, these associations were also observed in a range of observational analyses in agreement with previous evidence.^58^ FURIN is currently being explored as a drug target, with several phase 1 and 2 clinical trials investigating FURIN inhibitors for cancer immunotherapy showing promising efficacy and a good safety profile.^59^ Our PheWAS analysis supports the safety of targeting FURIN, though no clinical trials have yet investigated its therapeutic potential for CVD. The present evidence and insights from previous trials may further inform the development of furin-targeted treatments and therapeutic interventions for CVD.

*FGF5* (fibroblast growth factor 5) is a well-established hypertension susceptibility gene, with genetic variants linked to elevated SBP, DBP, and stroke.^60,61^ Our findings further support a potential causal link between increased plasma FGF5 levels, BP, and subsequent CHD and ischaemic stroke, aligning with previous MR studies and reinforcing observational evidence on FGF5 plasma levels. Fibroblast growth factors have been extensively studied as potential therapeutic targets for cardiovascular disease, primarily due to their metabolic and angiogenic effects. However, FGF5 has received comparatively less attention. While non-BP-related mechanisms, such as cardiovascular remodelling, have been proposed to explain FGF5’s role in CVD,^62^ our findings suggest that a significant proportion of its effect on CHD and ischaemic stroke is mediated through BP regulation.

Macrophage stimulating 1 (MST1), a key component of the Hippo pathway, was highlighted through MR, colocalization, and observational analysis. MST1 activation has been implicated in dilated cardiomyopathy, cardiomyocyte death following ischaemic injury, and inhibition of cardiac growth.^63,64^ Experimental studies further suggest that *MST1* knockdown reduces atherosclerotic plaque formation and improves metabolic health, including protection against non-alcoholic fatty liver disease.^65,66^ Our study provides evidence of a potentially causal effect of MST1 on BP, a link that has been little explored. Additionally, our findings further suggest that MST1’s effect on CAD may be mediated by its detrimental influence on BP levels, reinforcing its role as a potential contributor to vascular dysfunction. Although MST1-targeting drugs remain in the preclinical stage, inhibitors have shown potential in suppressing MST1 activity.^67^ Given its broad physiological impact, further research is needed to clarify MST1’s therapeutic potential in cardiovascular health.

### Strengths and limitations

Our study has several strengths. First, to the best of our knowledge, this is one of the first studies to examine the potential mediating role of BP in the relationship between plasma proteins and CVDs, utilizing large-scale genomics data. Second, we have integrated evidence from MR, colocalization, observational analysis, and PheWAS analyses to strengthen the robustness of our findings. Some limitations require careful consideration in interpretation of our findings. First, the summary-level data sources used are primarily from participants of European ancestry, which limits the generalizability of our findings to other populations. Second, several *cis*-pQTLs used in the MR analysis could be protein-altering variants or in high linkage disequilibrium with these variants, potentially leading to an aptamer binding effect. Among the proteins we prioritized, we noted that *cis*-pQTLs used as genetic instruments for ACOX1 were in high LD (r² > 0.8; 1000 genomes reference panel) with missense or stop-gained variants. However, for ACOX1, the lead *cis*-pQTL (rs10852766) is also a *cis*-eQTL (eQTLGen, Open Target Genetics), indicating that it affects *ACOX1* expression levels independent of aptamer binding. This may imply that potential causal associations between ACOX1 and CAD may still hold true. Third, despite efforts to avoid sample overlap, some participants from the CAD GWAS may have also contributed to the UKB-PPP, potentially leading to attenuated causal effect estimates. We mitigated this bias by consistently using genetic instruments with F-statistics >10 throughout the study.

In summary, our study provides strong evidence for shared proteomic signatures associated with BP, CAD, and stroke outcomes, highlighting proteins that may affect cardiovascular risk through modifiable risk factors such as BP. Our findings underscore the critical role of BP as a mediator in the relationship between circulating proteins and cardiovascular outcomes, particularly for FGF5, FURIN, ACOX1, and MST1. These proteins represent promising targets for further research, with potential implications for novel therapeutic interventions aimed at BP regulation and CVD prevention. Further research should aim at validating those findings and exploring mechanistic pathways to aid the translational potential of those discoveries.

## Supplementary Material

ehaf725_Supplementary_Data

## Declarations

### Data Availability

The UKBPPP data can be downloaded from http://ukb-ppp.gwas.eu. GWAS summary statistics for stroke and its subtypes for Europeans can be obtained from the GWAS catalogue with accession number GCST90104534-GCST90104563. GWAS summary statistics for CAD can be downloaded from the GWAS catalogue with accession number GCST005194. The constructed instrumental variables and code used for MR analyses will be made available at GitHub (https://github.com/Jingxian-Huang/bp_mediation_pp_cvd).
