# Supplementary material for: Blood pressure, plasma proteins, and cardiovascular diseases: a network Mendelian randomization and observational study
Source: Eur Heart J. 2025 Oct 9;47(3):331–42. doi: 10.1093/eurheartj/ehaf725 (PMC12807568; doi:10.1093/eurheartj/ehaf725)
Supplement: ehaf725_Supplementary_Data [file ehaf725_supplementary_data.zip › Supplementary_Figures.docx]

**SUPPLEMENTARY INFORMATION**

**Blood pressure, plasma proteins and cardiovascular diseases:**

**a network Mendelian randomisation and observational study**

Devendra Meena^1, ‡^, Jingxian Huang^1, ‡^, Alexander Smith^1^, James Yarmolinsky^1^, Siwei Wu^1^, Fotios Koskeridis^1^, Yi-Hsuan Ko^1^, Marie-Joe Dib^2^, Charalabos Antonatos^3^, Yiannis Vasilopoulos^3^, Xinzhu Yu^1^, Georg W. Otto^1,4^, Dipender Gill^1^, Manuel Mayr^5,6^, Paul Elliott^1,4^, Abbas Dehghan^1,4, 6^ *, Ioanna Tzoulaki^1, 4,6,7^ *

**Affiliations**

^1^ Department of Epidemiology and Biostatistics, School of Public Health, Imperial College London, London, UK

^2^ Division of Cardiovascular Medicine, Hospital of the University of Pennsylvania, Philadelphia, USA

^3^ Laboratory of Genetics, Section of Genetics, Cell Biology and Development, Department of Biology, University of Patras, Patras, Greece

^4^ UK Dementia Research Institute at Imperial College London, London, UK

^5^ National Heart & Lung Institute, Imperial College London, London, UK

^6^ British Heart Foundation Centre of Research Excellence, Imperial College London, London, UK

^7^ Centre for Systems Biology, Biomedical Research Foundation, Academy of Athens, Athens, Greece


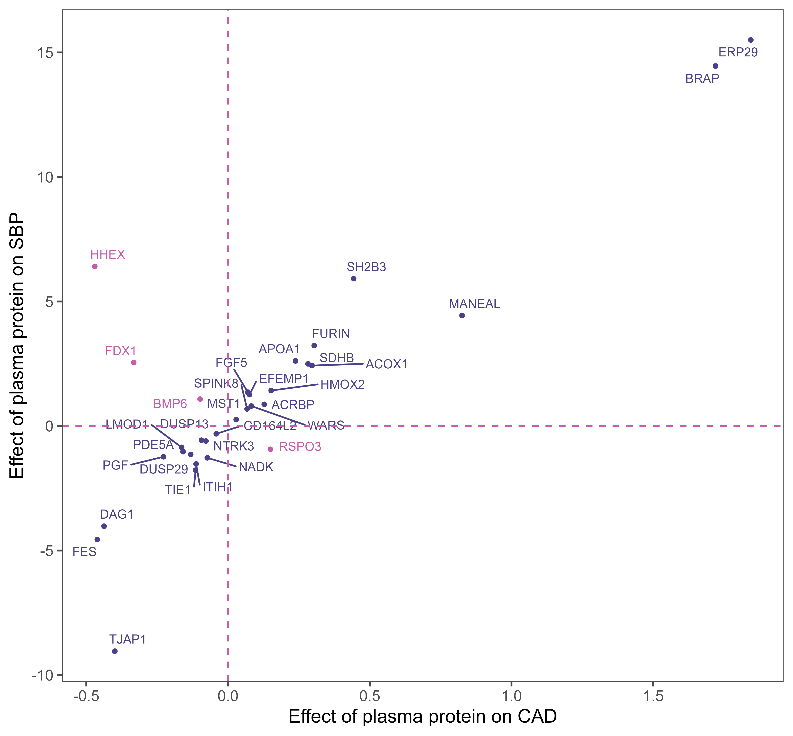

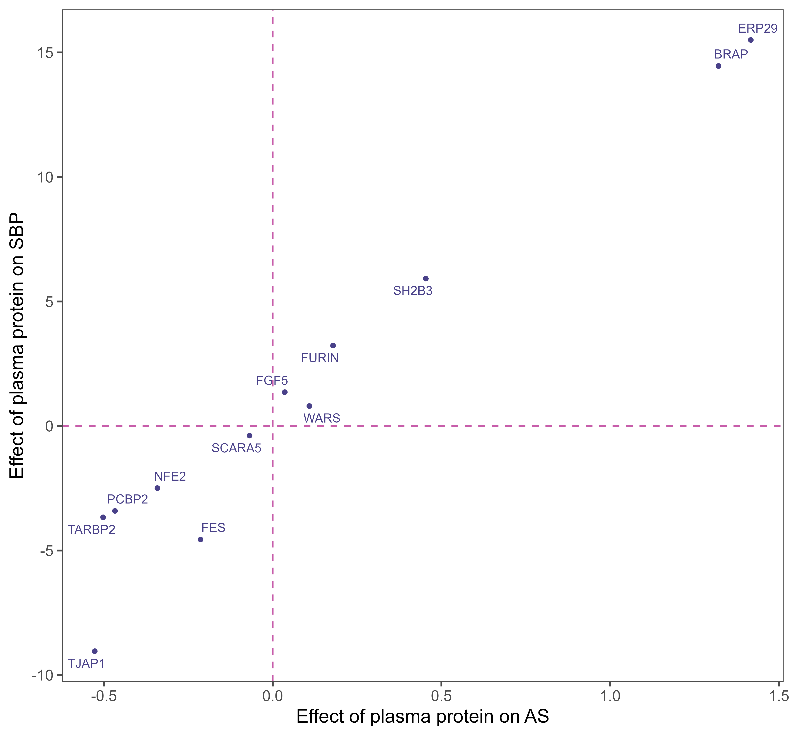


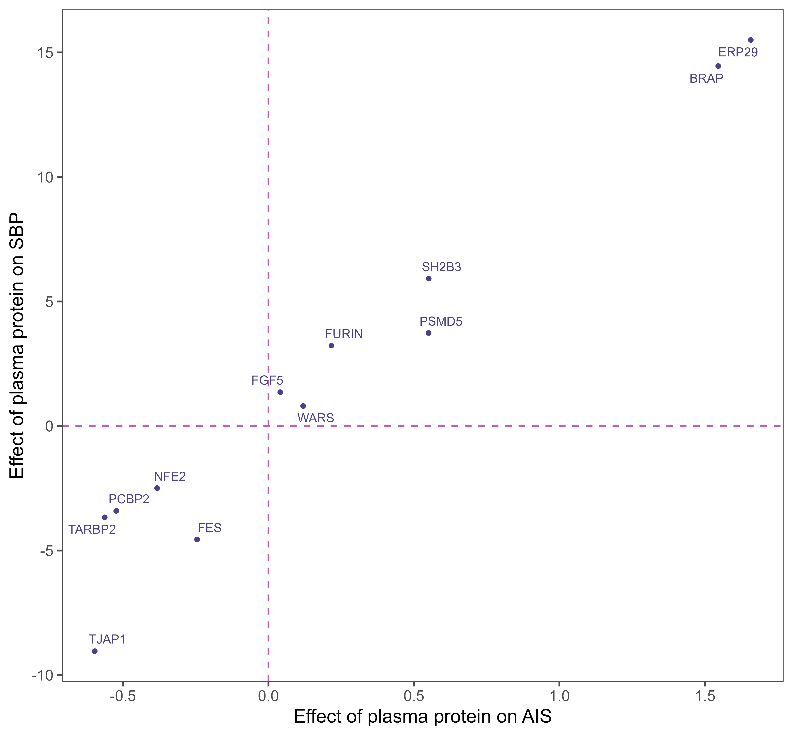

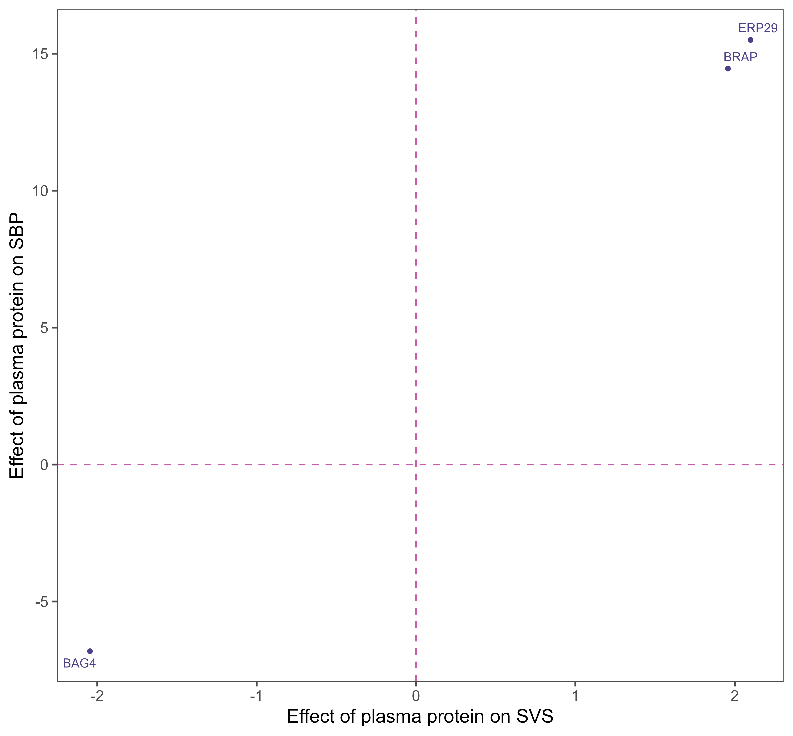


**Supplementary Figure 1.** Scatter plot showing the effect of SBP-associated proteins on CAD and or stroke outcomes. No SBP-associated proteins were significantly associated with CES and LAS. All estimates refer to inverse variance weighted (number of genetic instruments ≥ 2) or Wald ratio (number of genetic instruments < 2).


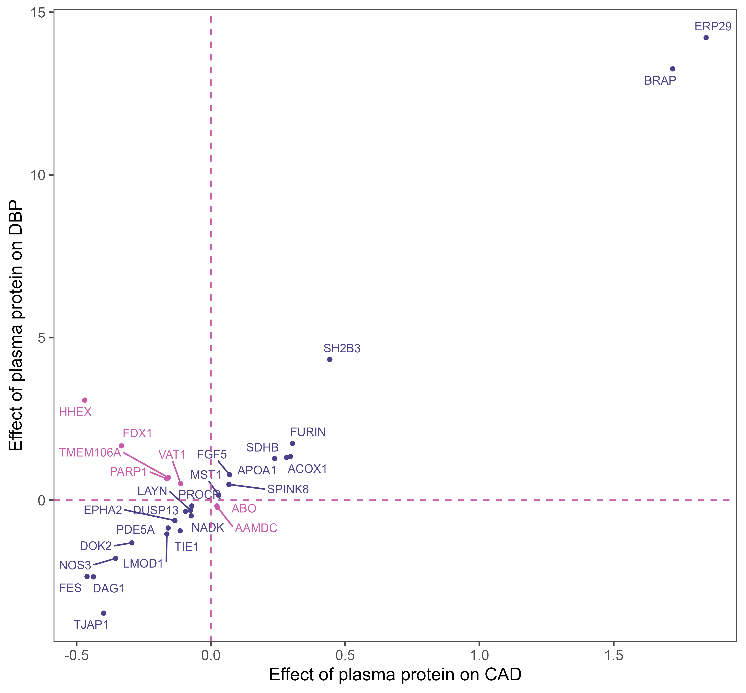

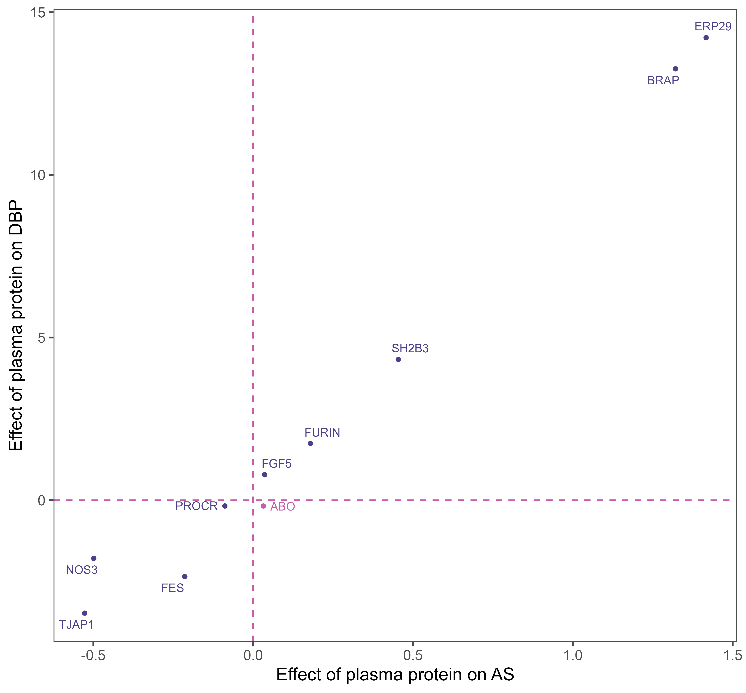

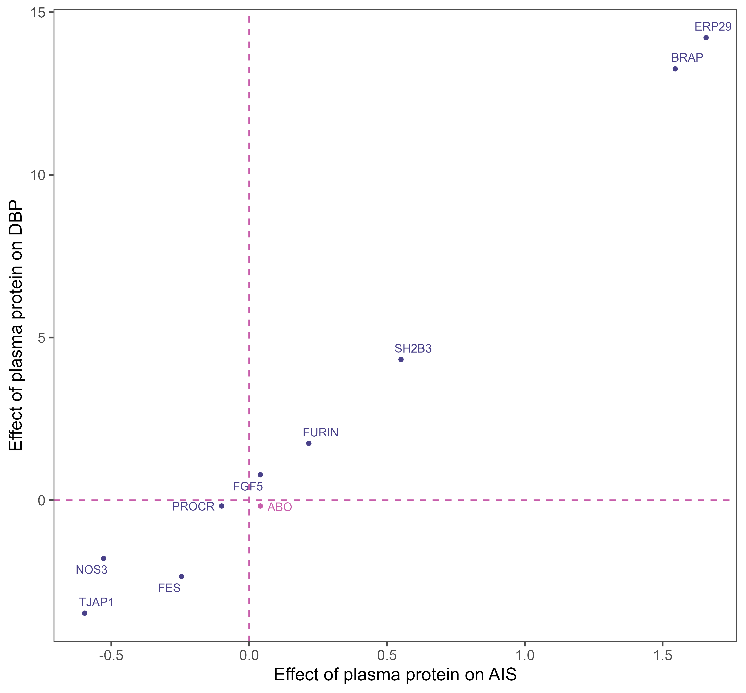

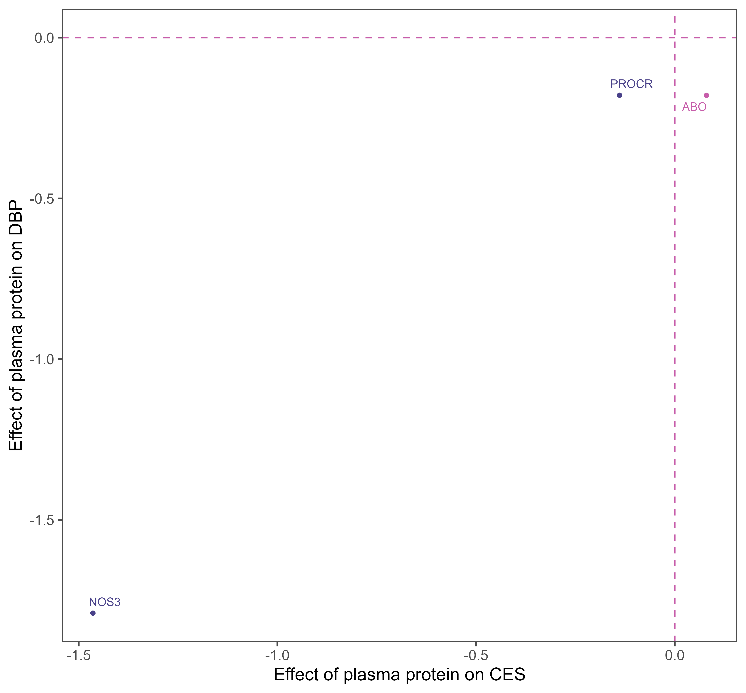


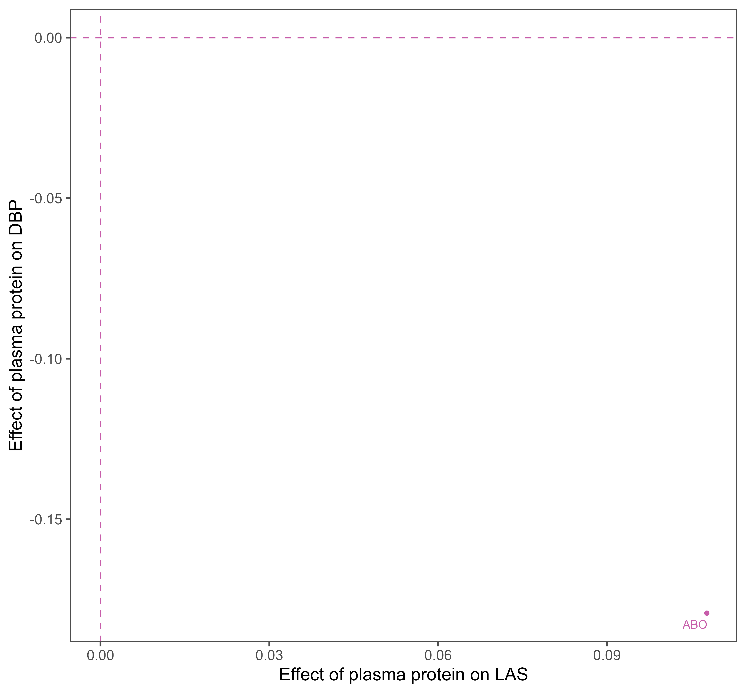

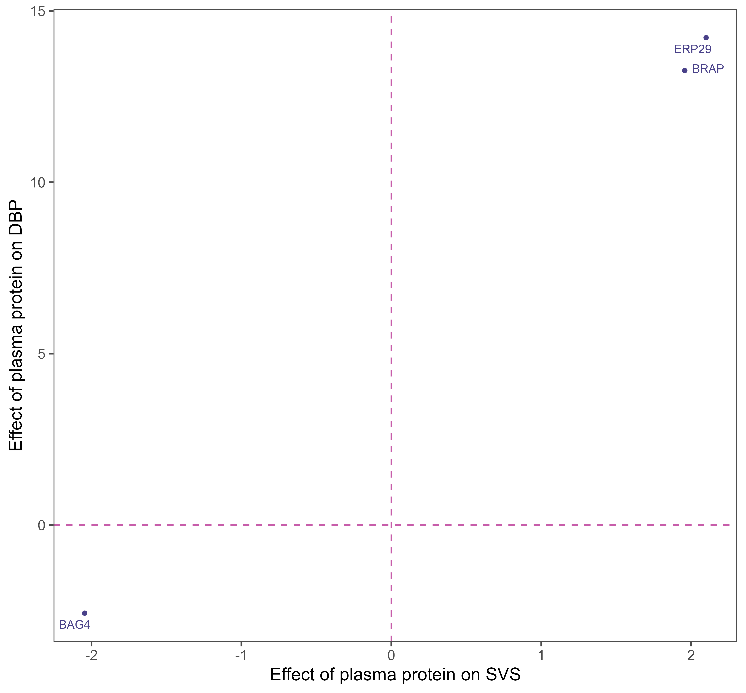


**Supplementary Figure 2.** Scatter plot showing the effet of DBP-associated proteins on CAD or stroke outcomes. All estimates refer to inverse variance weighted (number of genetic instruments ≥ 2) or Wald ratio (unmber of genetic instruments < 2).


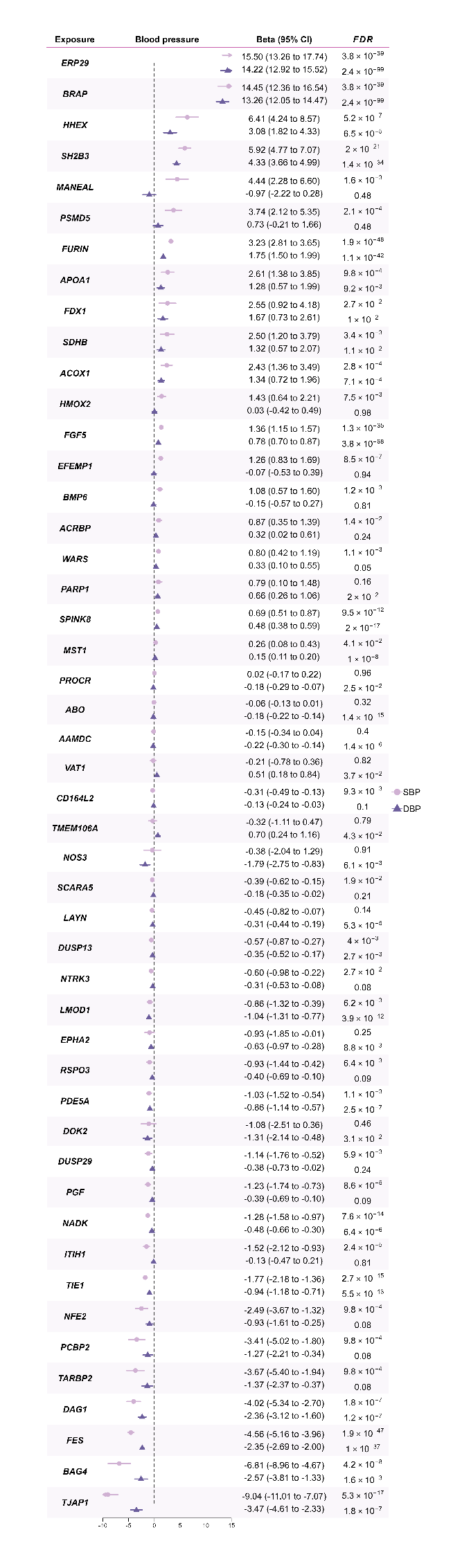


**Supplementary Figure 3.** Forest plot showing the 48 BP-associated plasma proteins that were also significantly associated with at least one CAD or stroke outcome. All estimates refer to inverse variance weighted (nSNP ≥ 2) or Wald ratio (nSNP < 2).


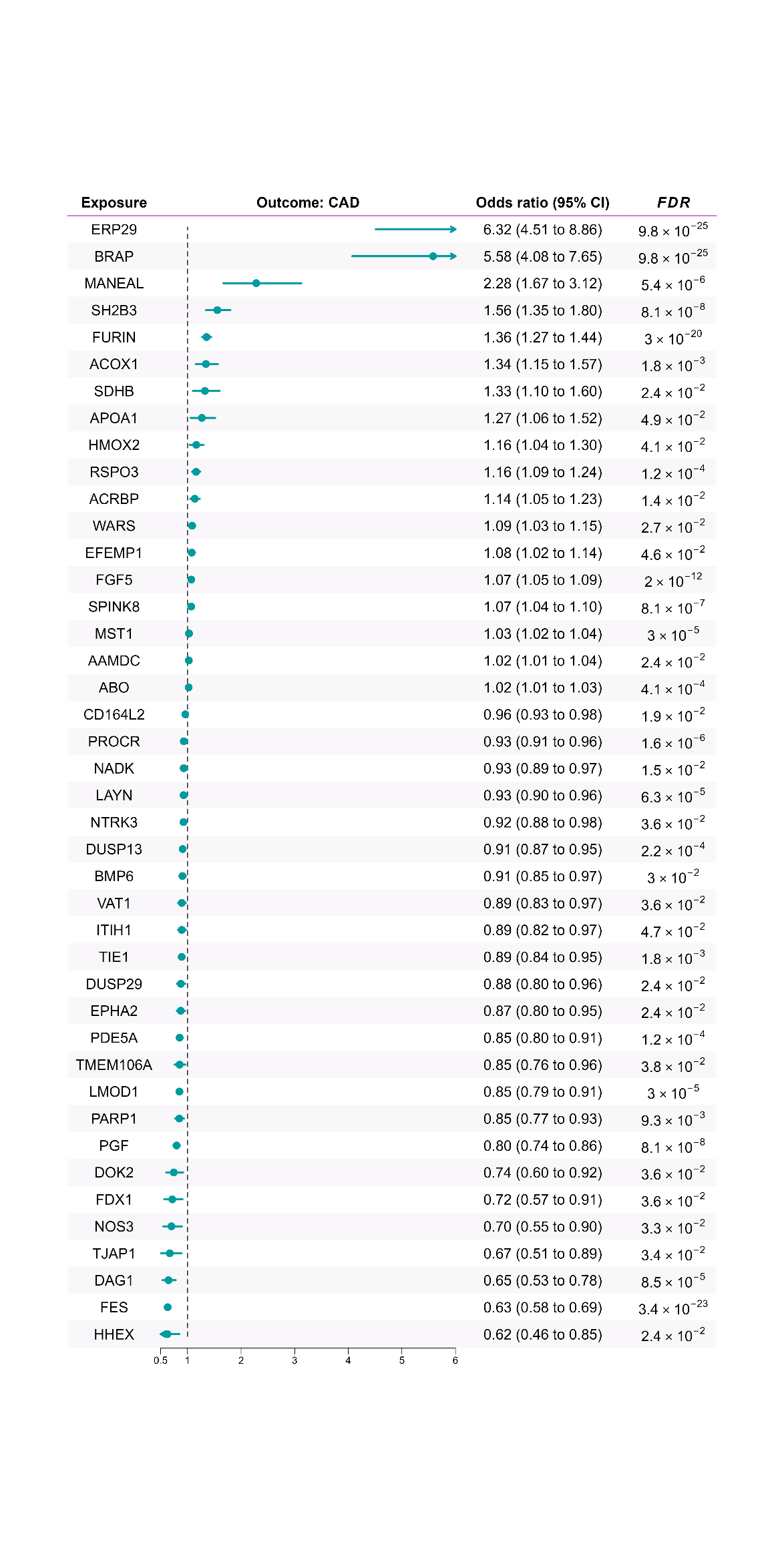


**Supplementary Figure 4.** Forest plot showing the 42 BP-associated plasma proteins that were also significantly associated with CAD. All estimates refer to inverse variance weighted (number of genetic instruments ≥ 2) or Wald ratio (number of genetic instruments < 2).


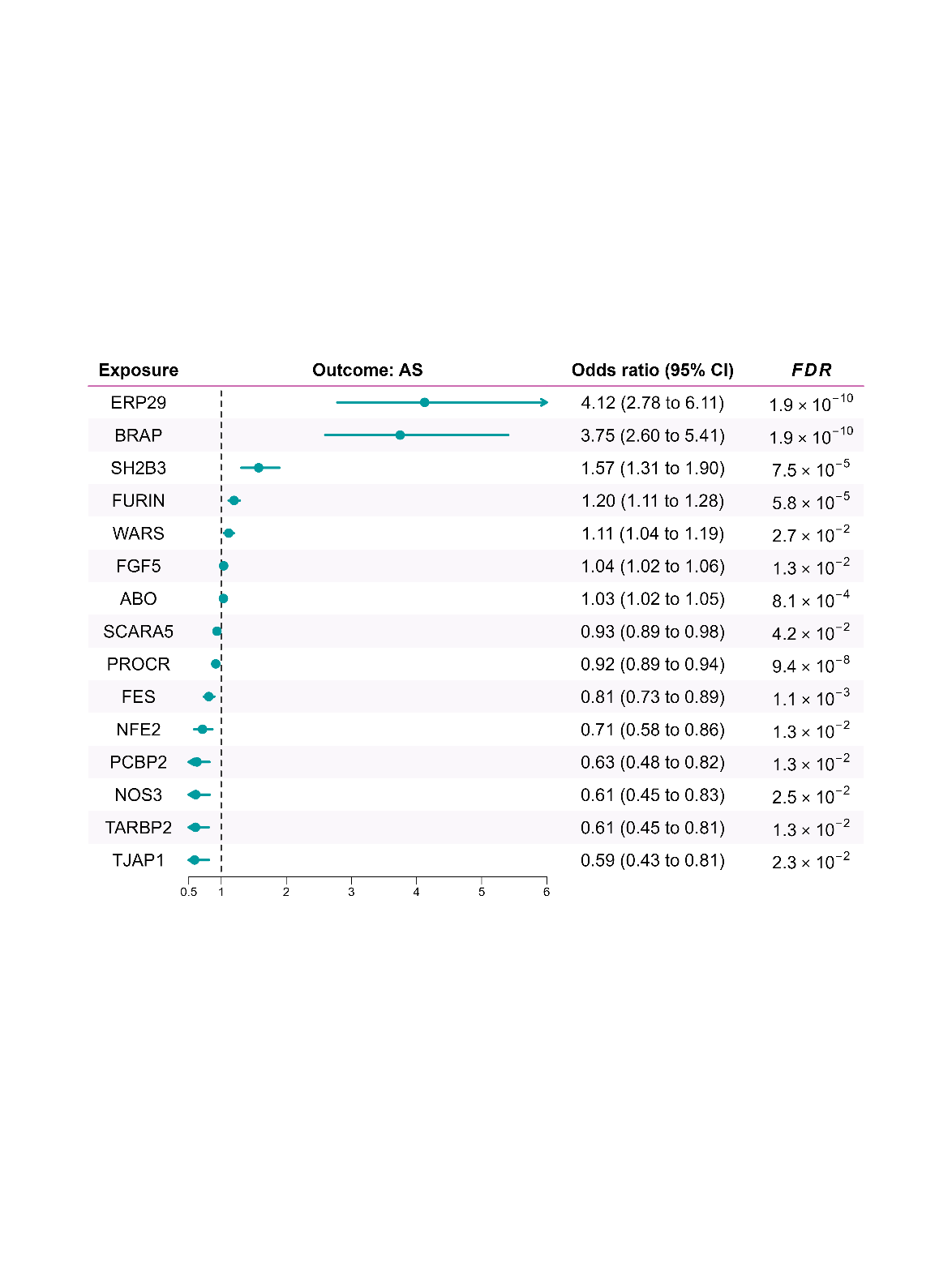


**Supplementary Figure 5.** Forest plot showing the 15 BP-associated plasma proteins that were also significantly associated with AS. All estimates refer to inverse variance weighted (number of genetic instruments ≥ 2) or Wald ratio (number of genetic instruments < 2).


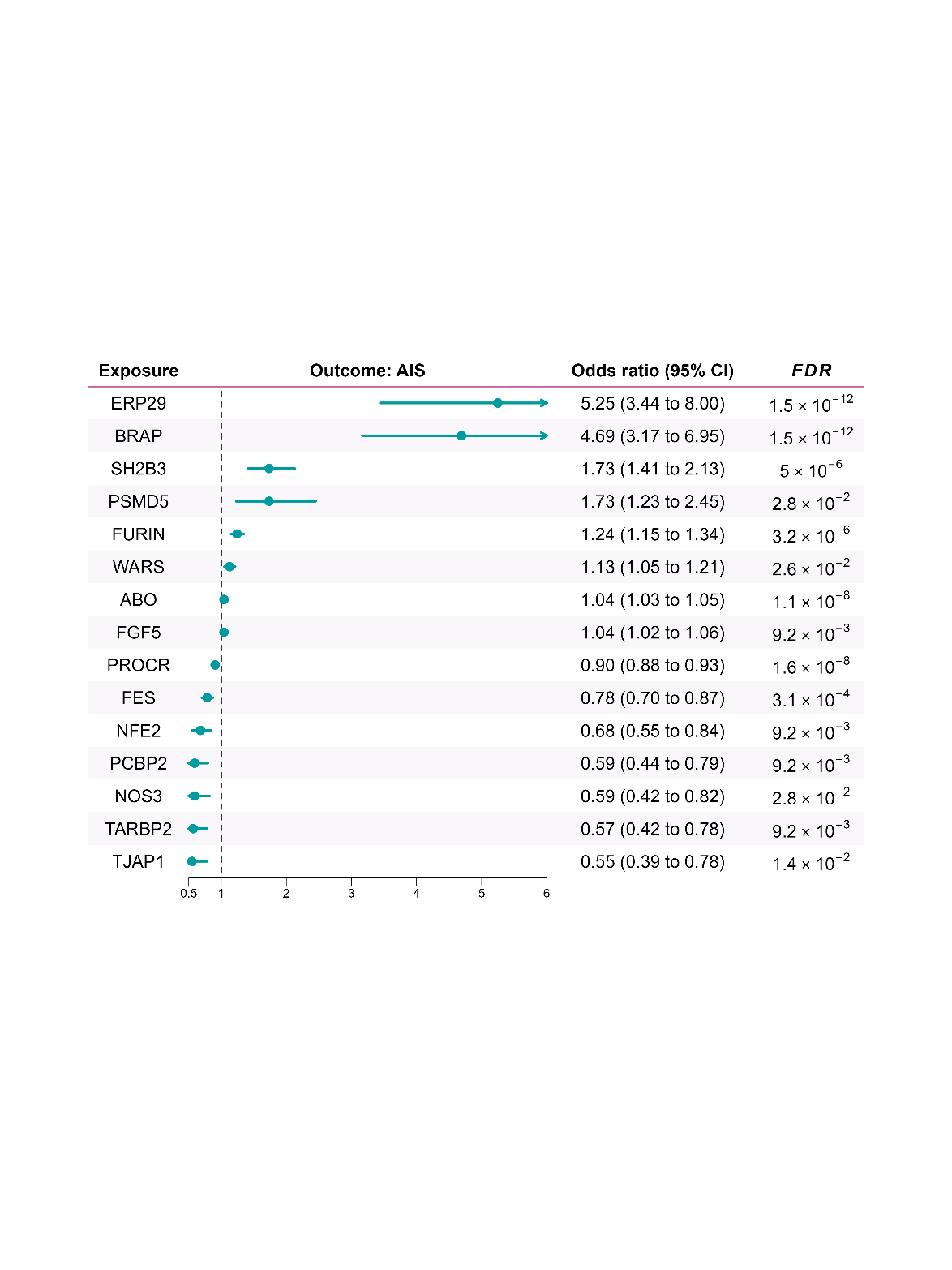


**Supplementary Figure 6.** Forest plot showing the 15 BP-associated plasma proteins that were also significantly associated with AIS. All estimates refer to inverse variance weighted (number of genetic instruments ≥ 2) or Wald ratio (number of genetic instruments < 2).


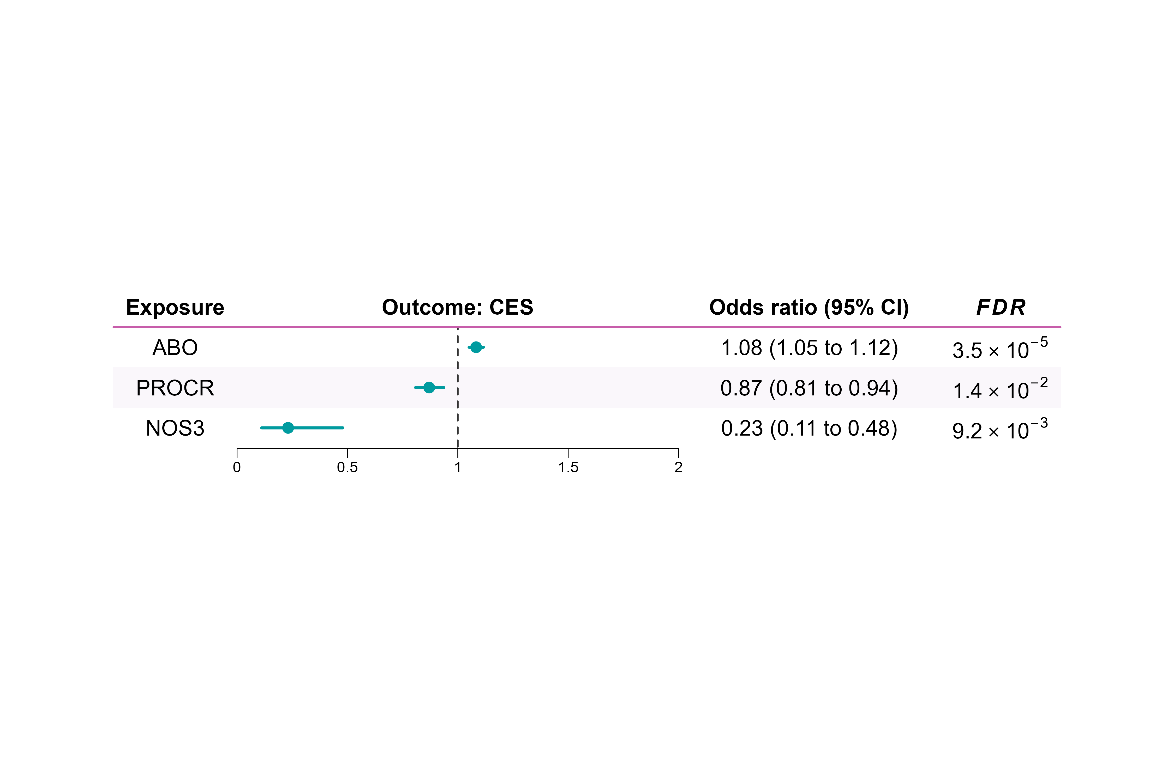


**Supplementary Figure 7.** Forest plot showing the 3 BP-associated plasma proteins that were also significantly associated with CES. All estimates refer to inverse variance weighted (number of genetic instruments ≥ 2) or Wald ratio (number of genetic instruments < 2).


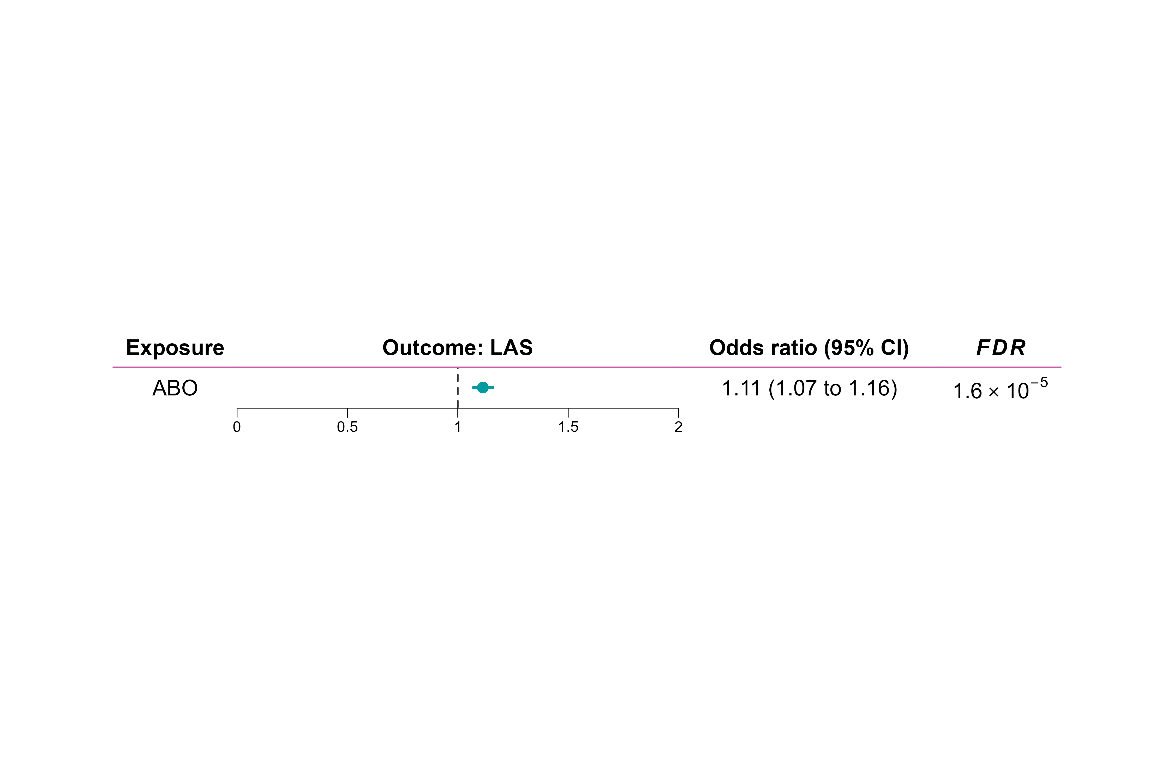


**Supplementary Figure 8.** Forest plot showing the 1 BP-associated plasma protein that was also significantly associated with LAS. All estimates refer to inverse variance weighted (number of genetic instruments ≥ 2) or Wald ratio (number of genetic instruments < 2).


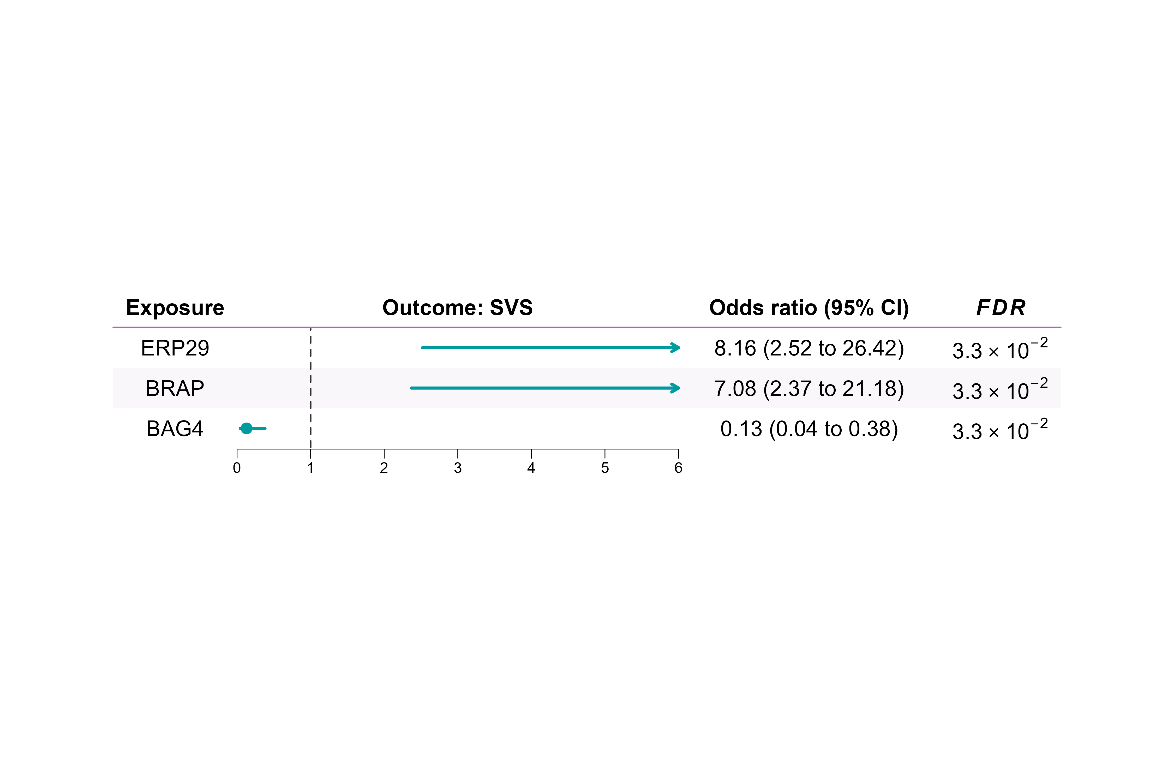


**Supplementary Figure 9.** Forest plot showing the 3 BP-associated plasma proteins that were also significantly associated with SVS. All estimates refer to inverse variance weighted (number of genetic instruments ≥ 2) or Wald ratio (number of genetic instruments < 2).


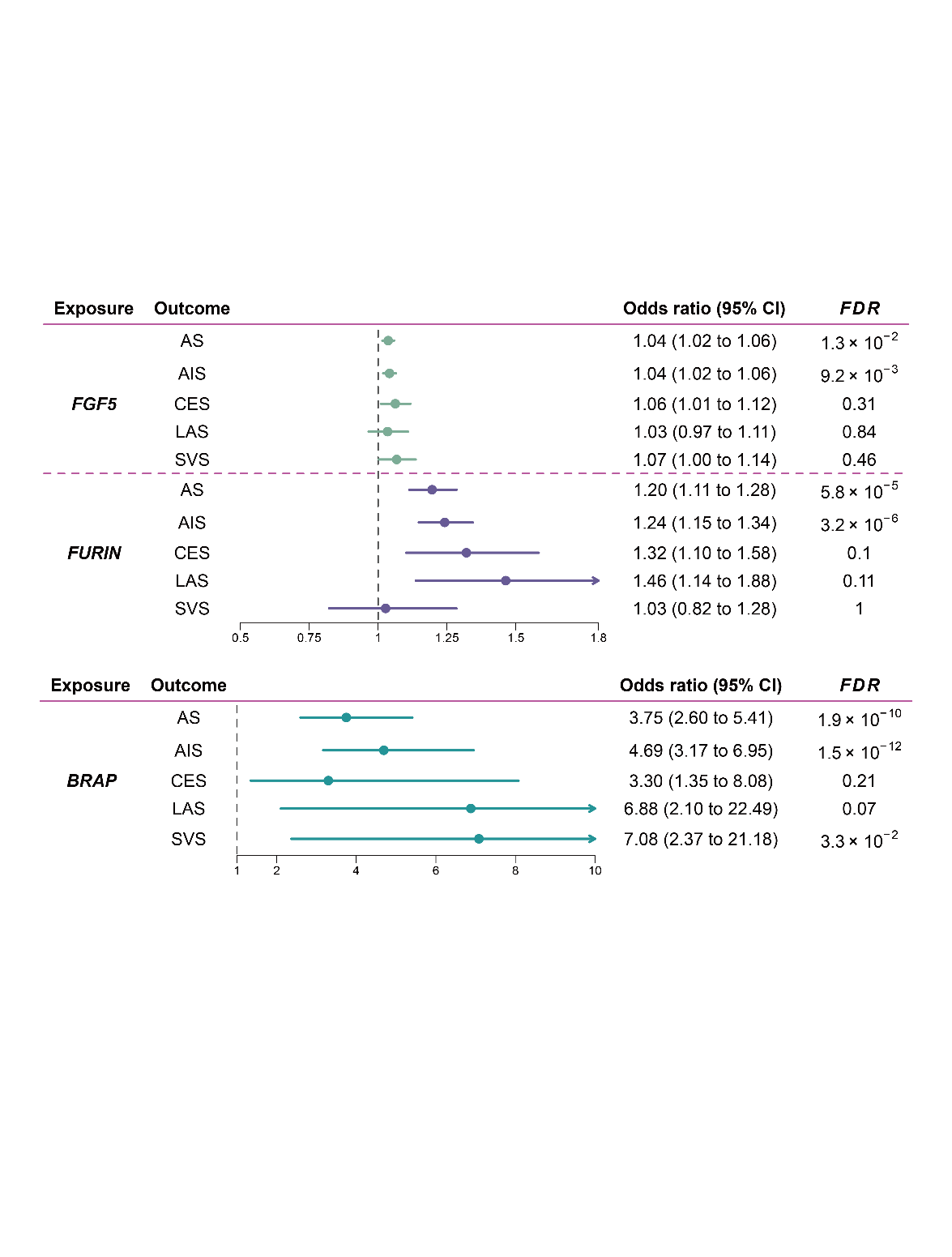


**Supplementary Figure 10.** Forest plot showing the effects of 3 BP-associated plasma proteins (FGF5, FURIN, and BRAP) on different stroke subtypes. All estimates refer to inverse variance weighted (number of genetic instruments ≥ 2) or Wald ratio (number of genetic instruments < 2).


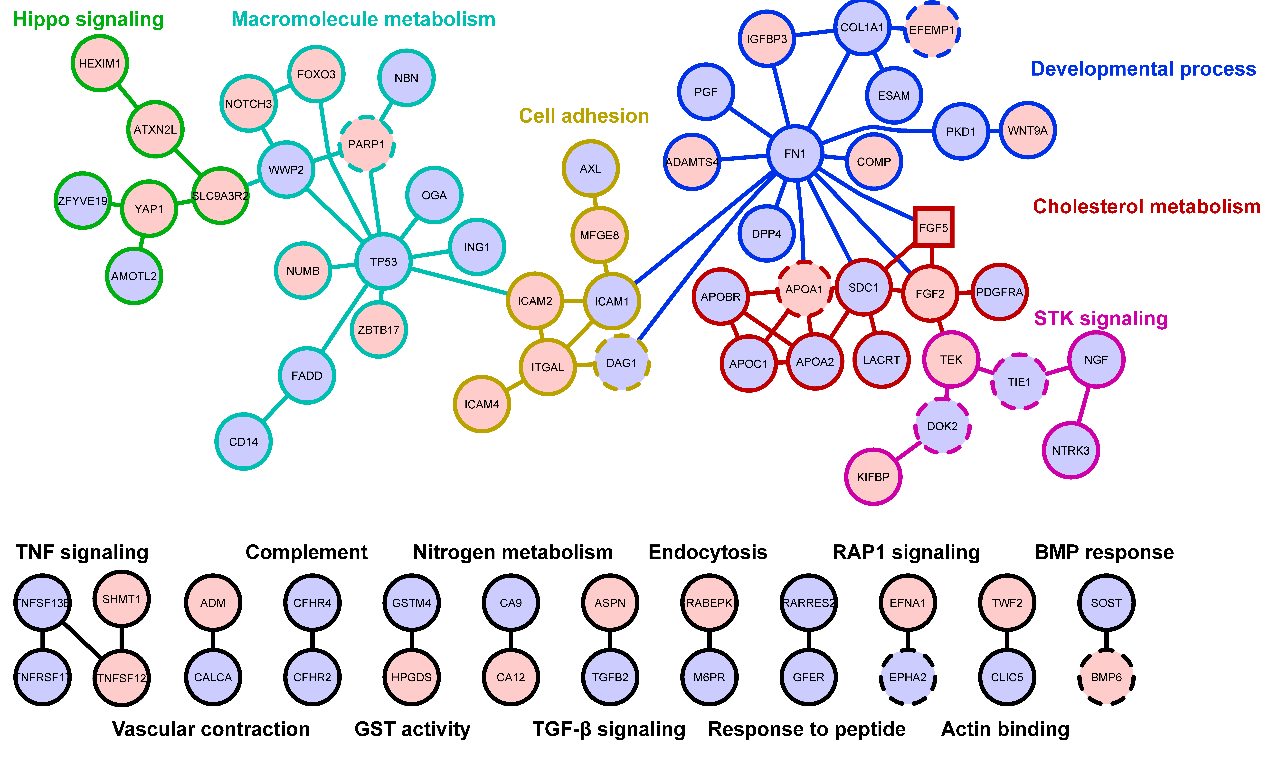


**Supplementary Figure 11.** Protein-protein interactions (PPIs) between genetically predicted proteins associated with BP. Each node corresponds to a protein. Red color denotes positive beta values, while blue color denotes negative beta values. Discrete color scale was applied for each identified community through the Leiden algorithm. Abbreviations: STK, serine/threonine kinase; TNF, tumor necrosis factor; GST, glutathione S-transferase; TGF-β, transforming growth factor beta; BMP, bone morphogenic factor.


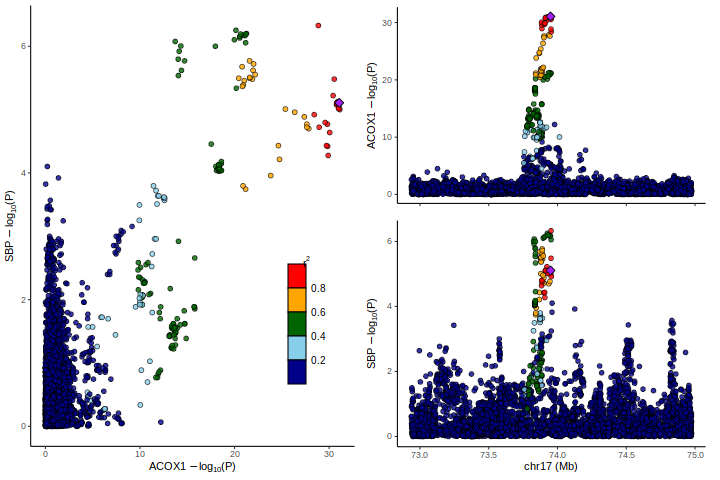

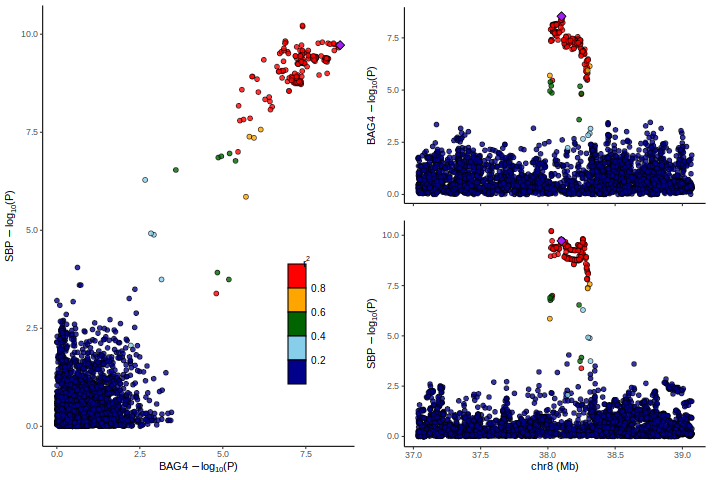

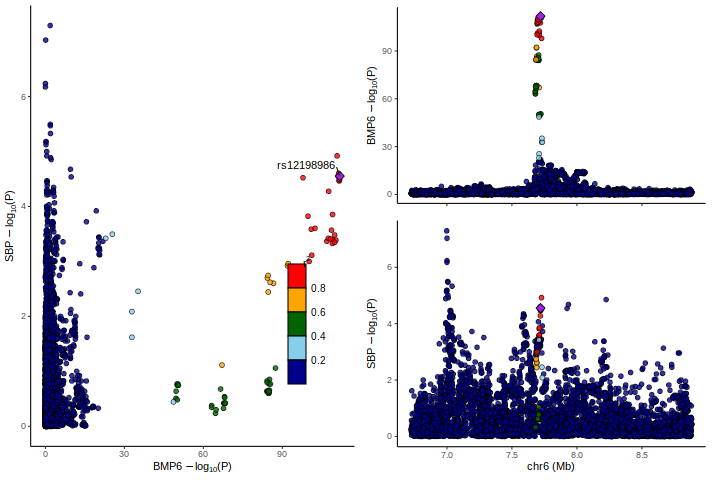

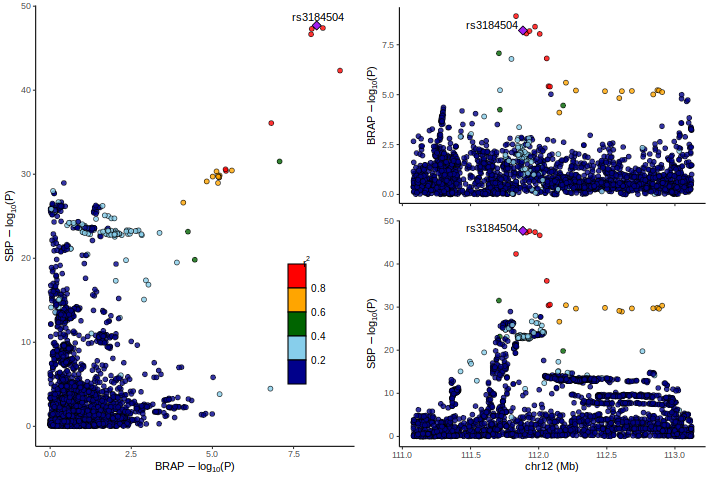

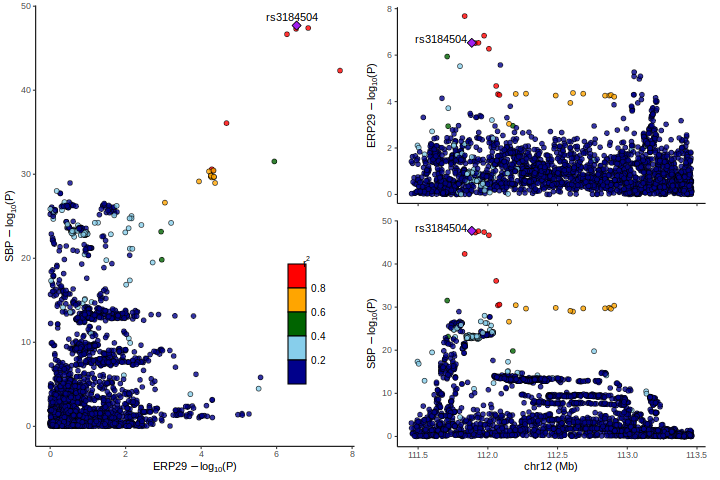

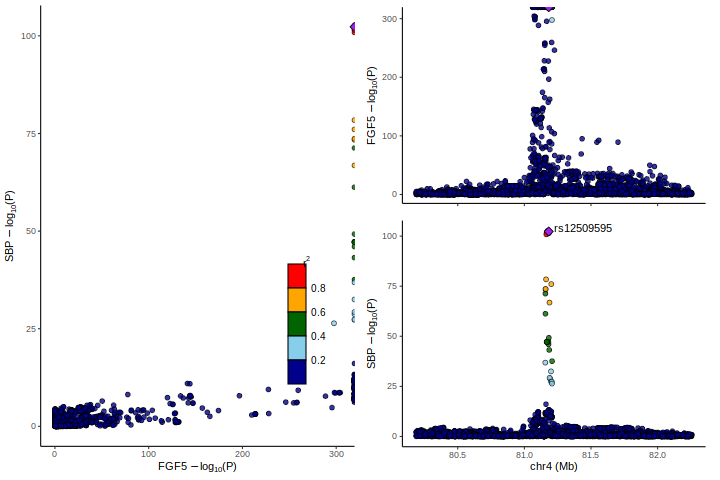

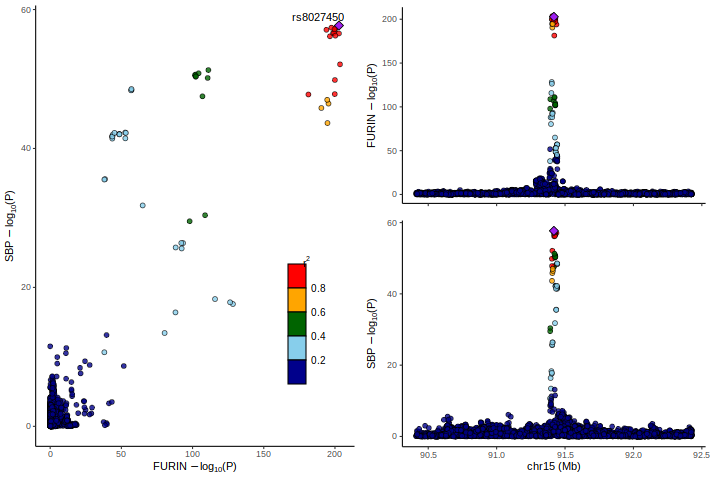

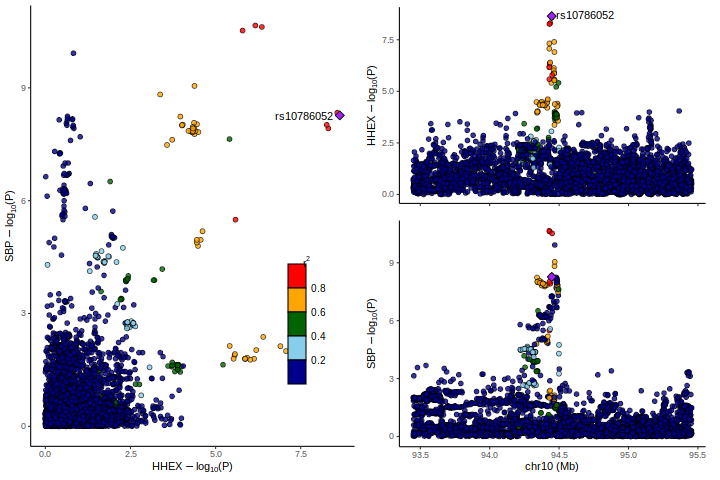

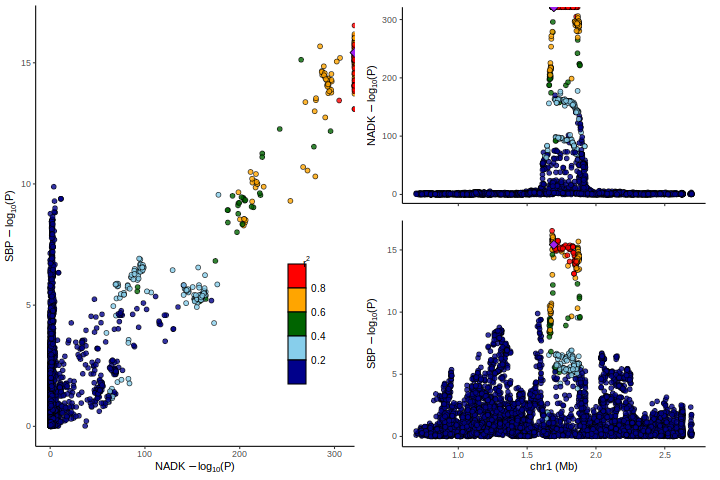

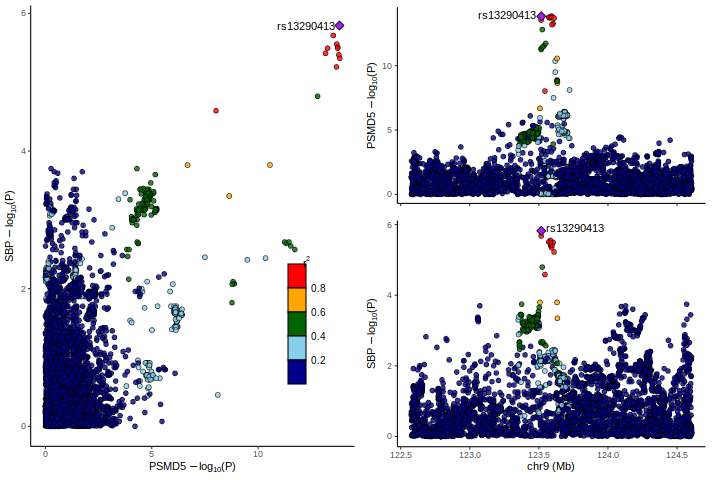


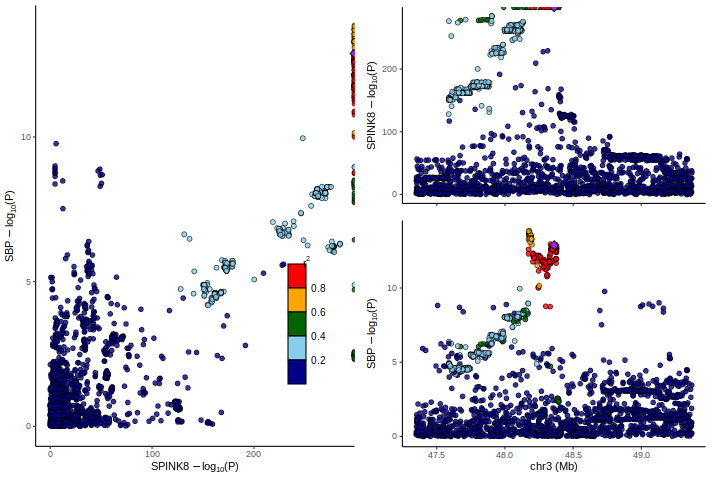

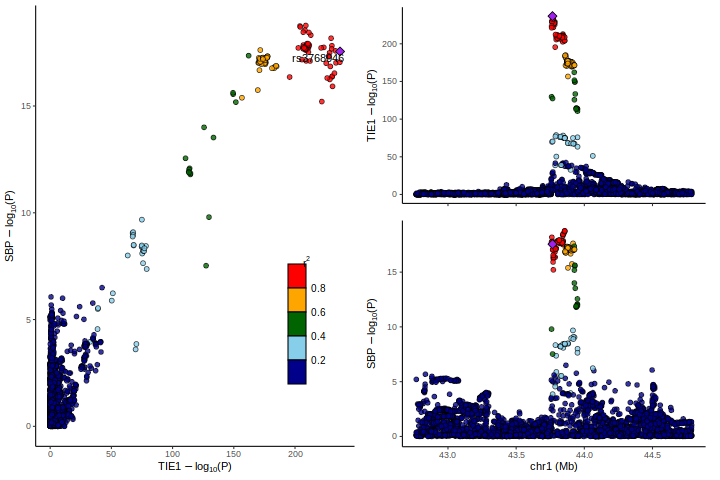

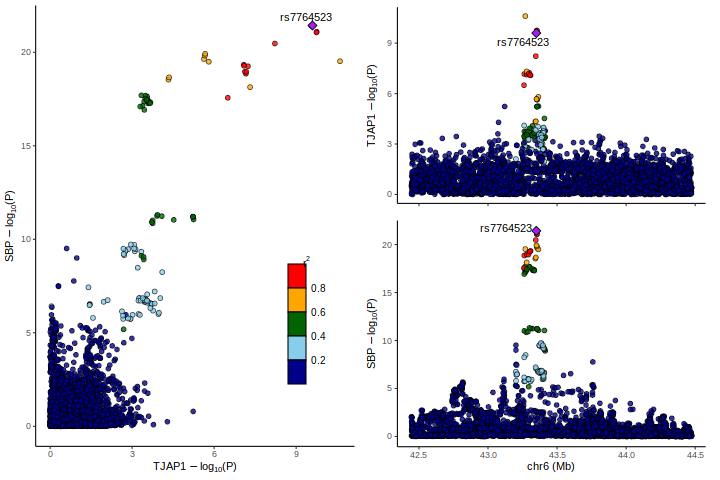


**Supplementary Figure 12.** Region genomic plots showing the results of colocalization analysis for common causal candidates between SBP and CAD/stroke.


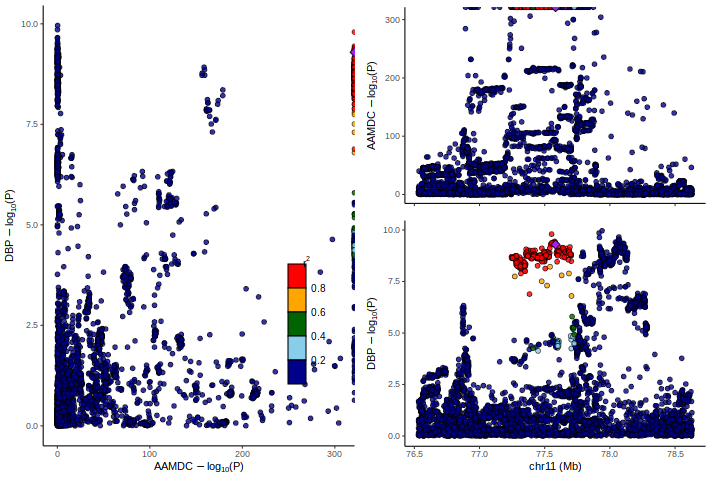

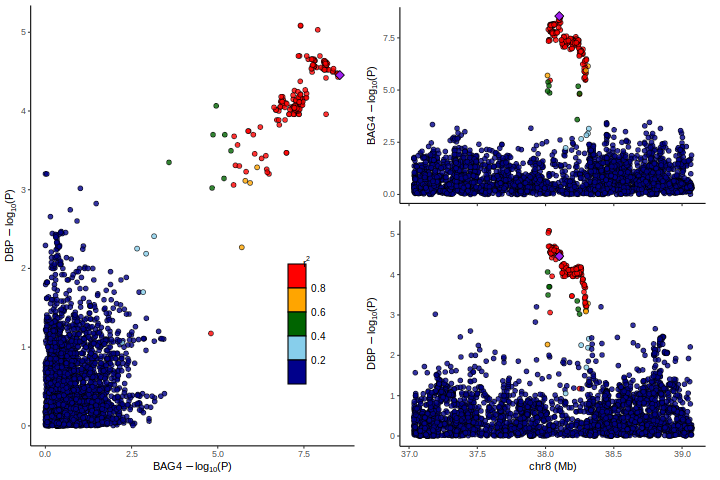


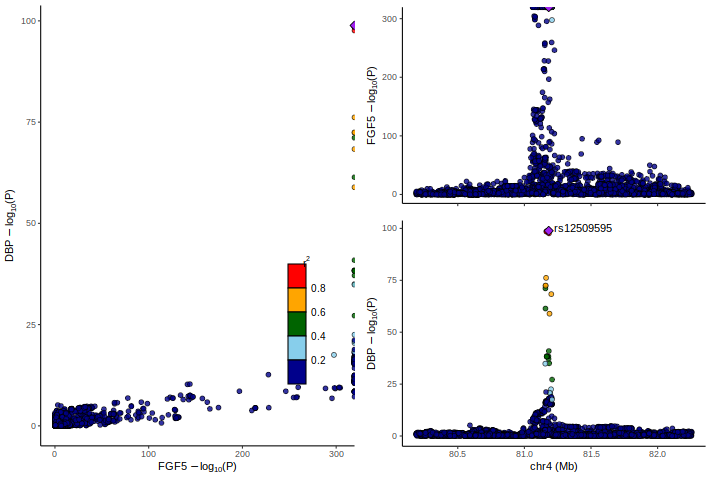

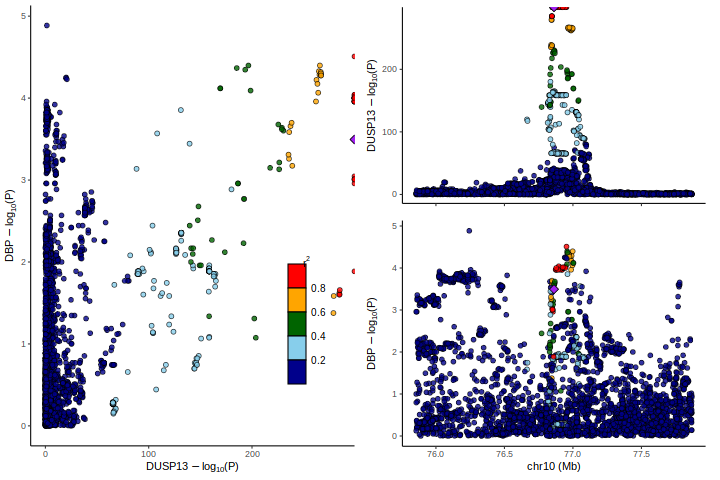


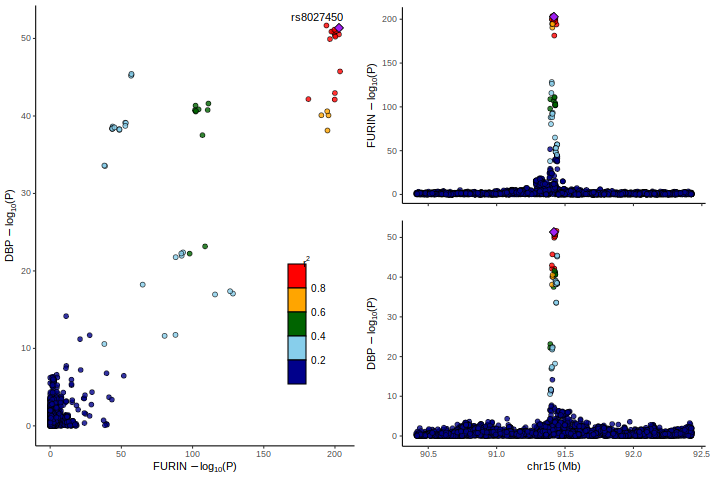

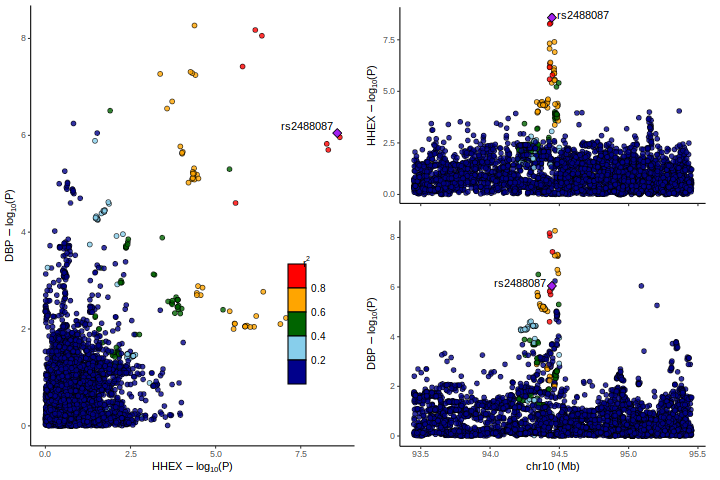


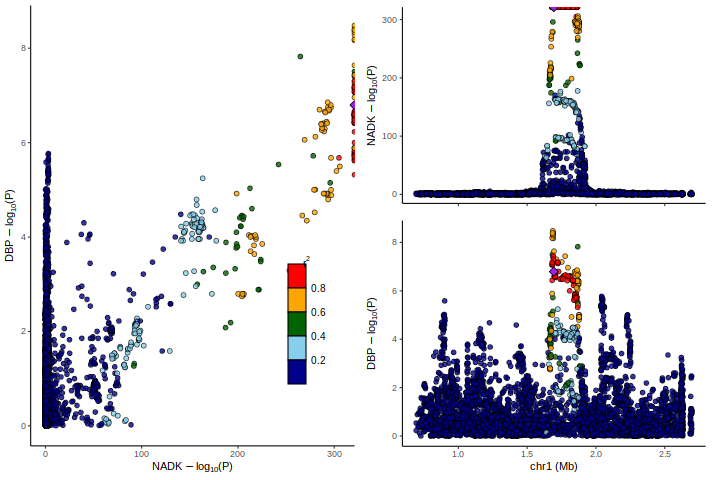

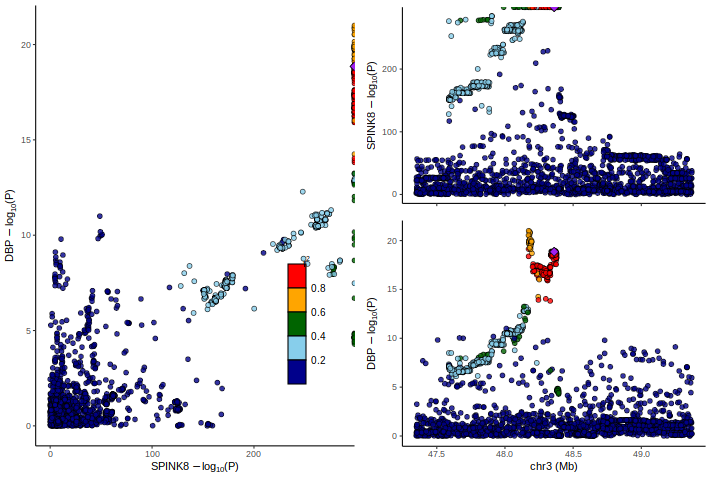


**Supplementary Figure 13.** Region genomic plots showing the results of colocalization analysis for common causal candidates between DBP and CAD/stroke.


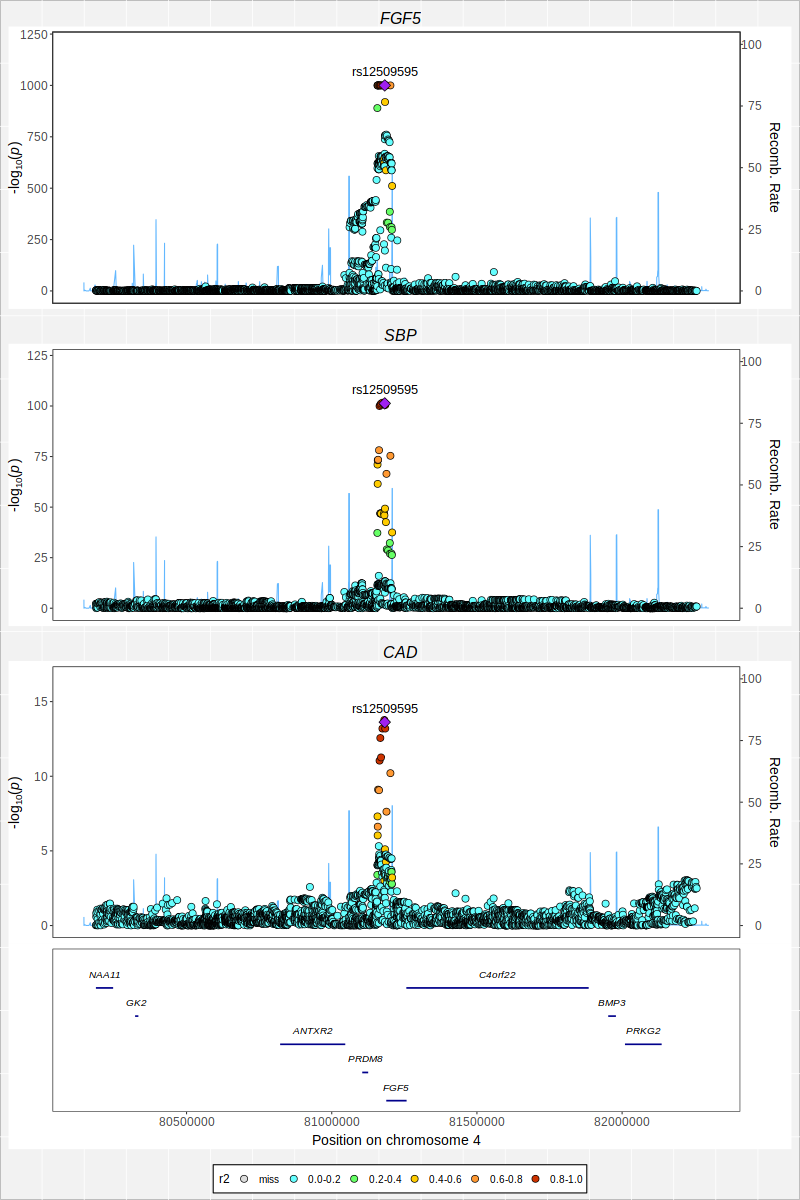


**Supplementary Figure 14.** Region genomic plots showing the results of multi-trait colocalization analysis for common causal candidates between FGF5, SBP, and CAD.


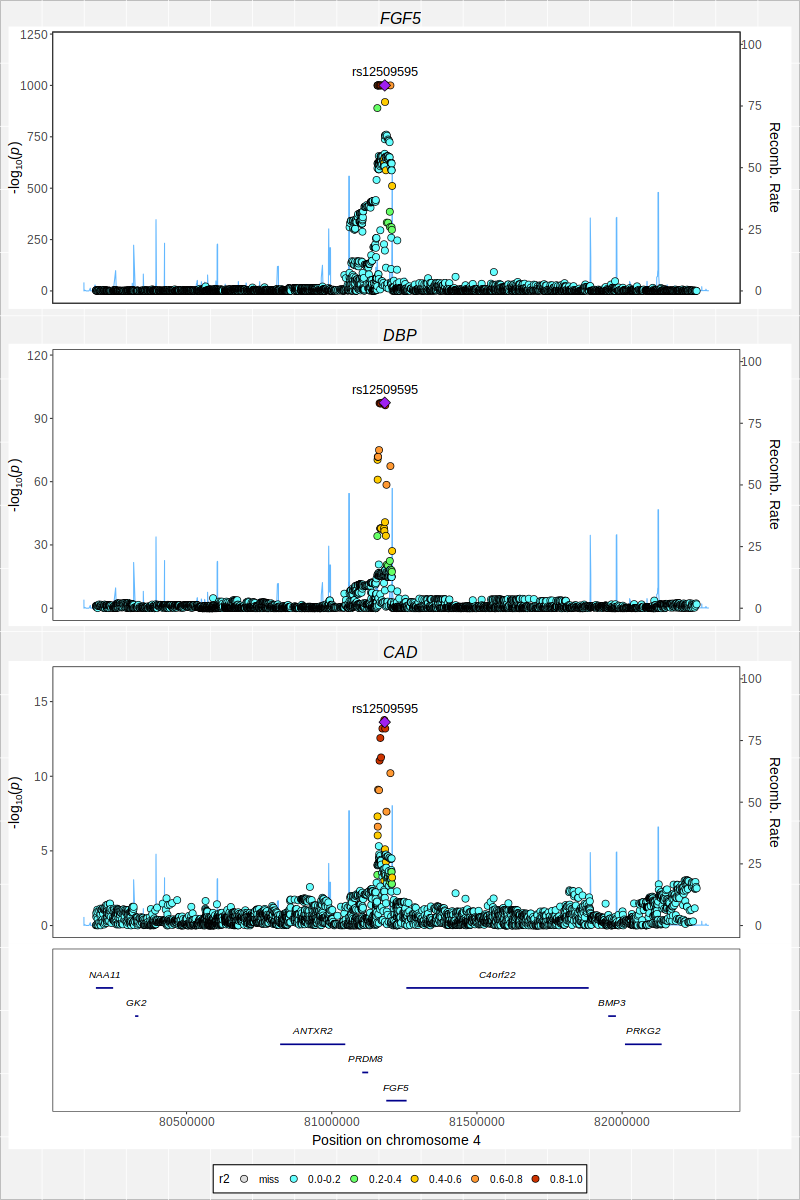


**Supplementary Figure 15.** Region genomic plots showing the results of multi-trait colocalization analysis for common causal candidates between FGF5, DBP, and CAD.


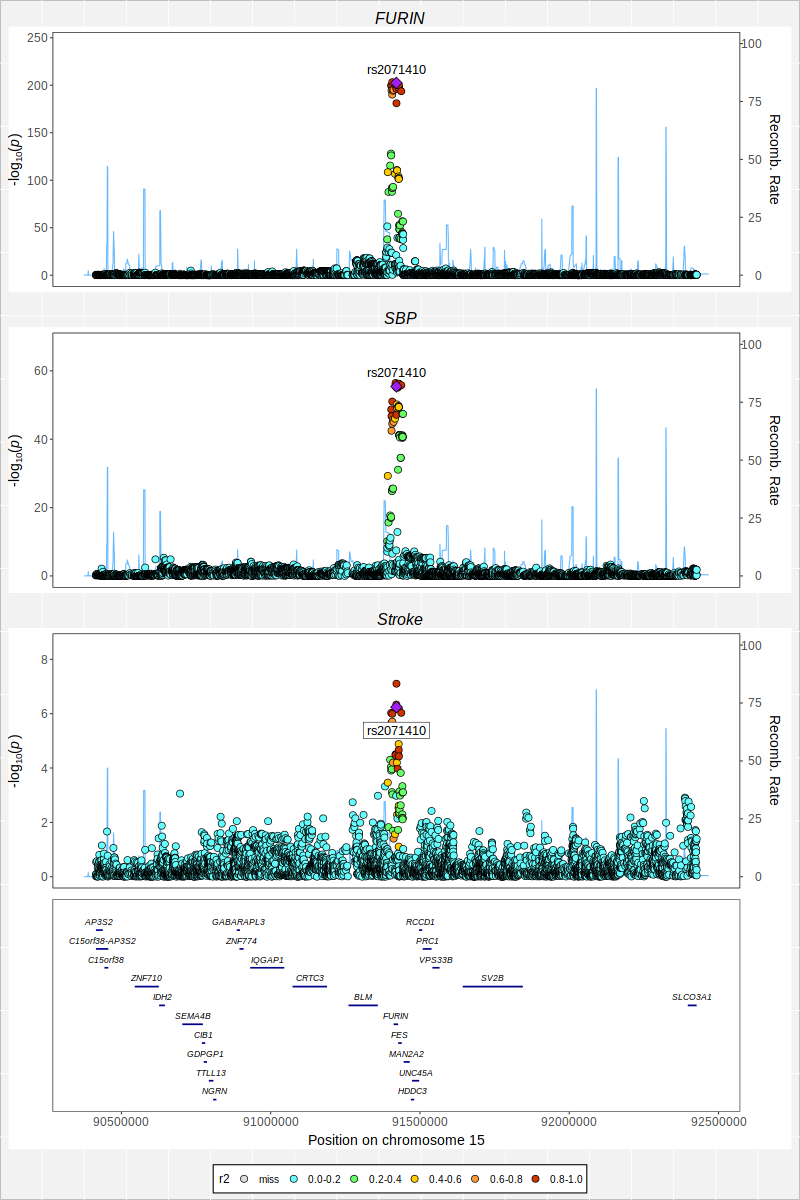


**Supplementary Figure 16.** Region genomic plots showing the results of multi-trait colocalization analysis for common causal candidates between FURIN, SBP, and all strokes.


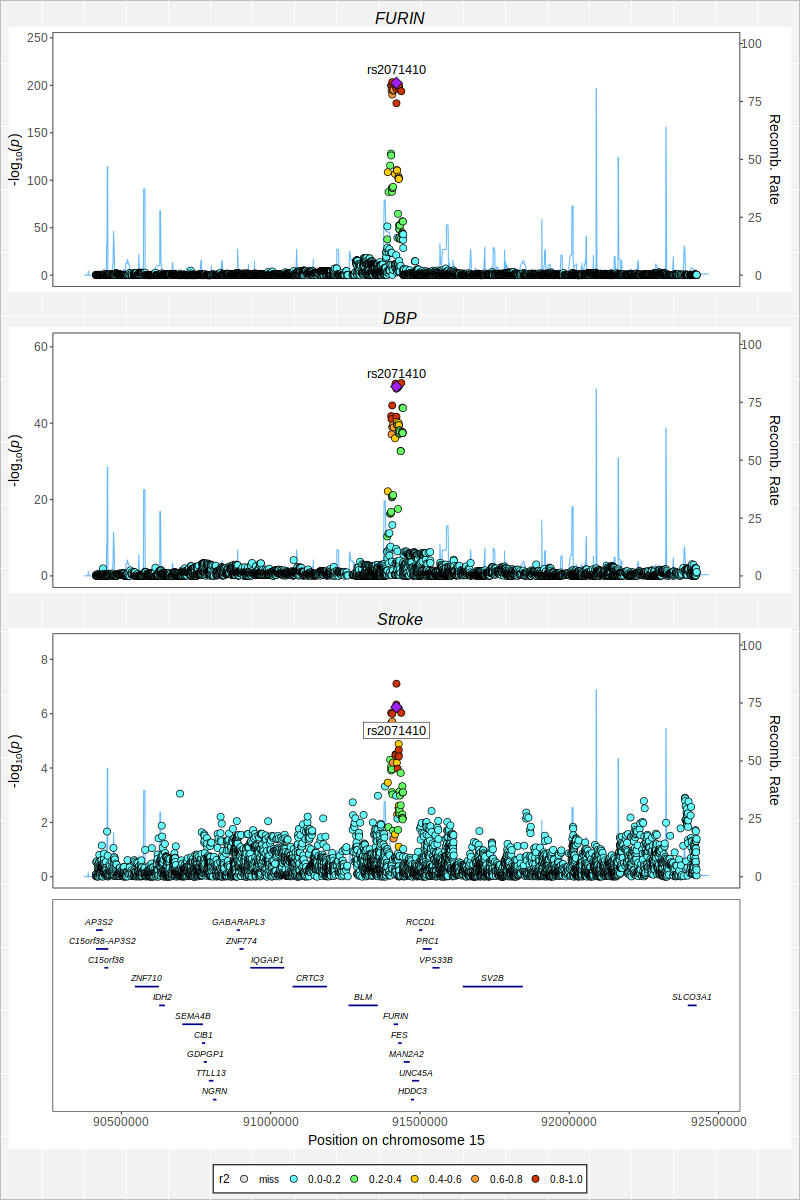


**Supplementary Figure 17.** Region genomic plots showing the results of multi-trait colocalization analysis for common causal candidates between FURIN, DBP, and all strokes.


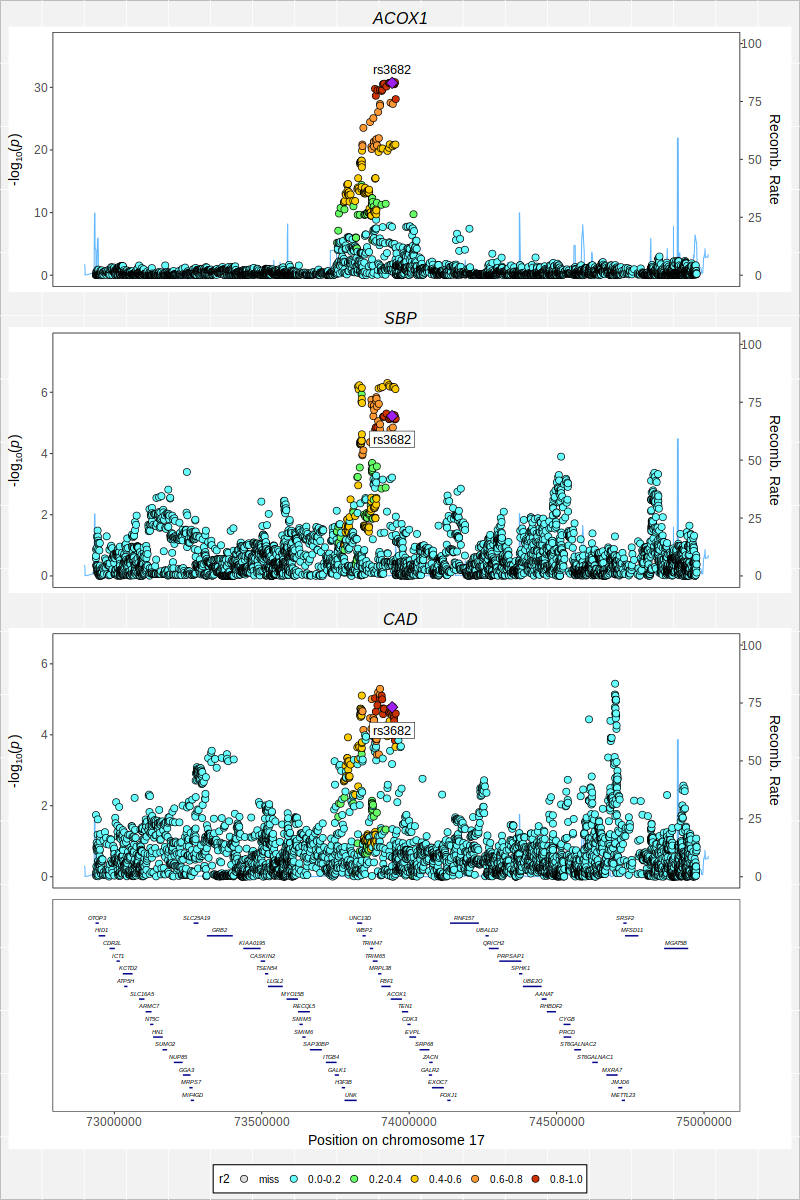


**Supplementary Figure 18.** Region genomic plots showing the results of multi-trait colocalization analysis for common causal candidates between ACOX1, SBP, and CAD.


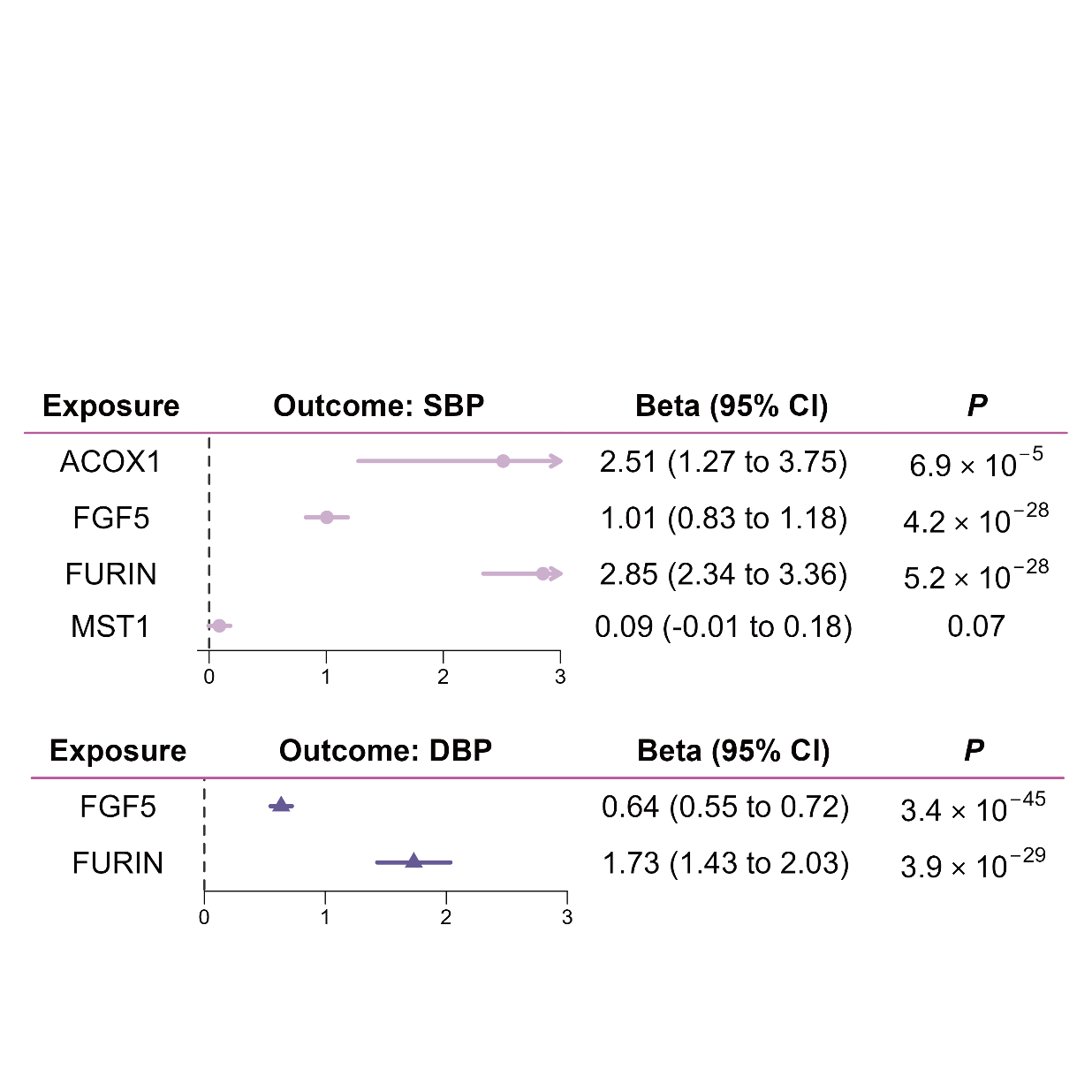


**Supplementary Figure 19.** Forest plot showing the replication of top BP-associated proteins in ICBP consortium. All estimates refer to inverse variance weighted (nSNP ≥ 2) or Wald ratio (nSNP < 2).


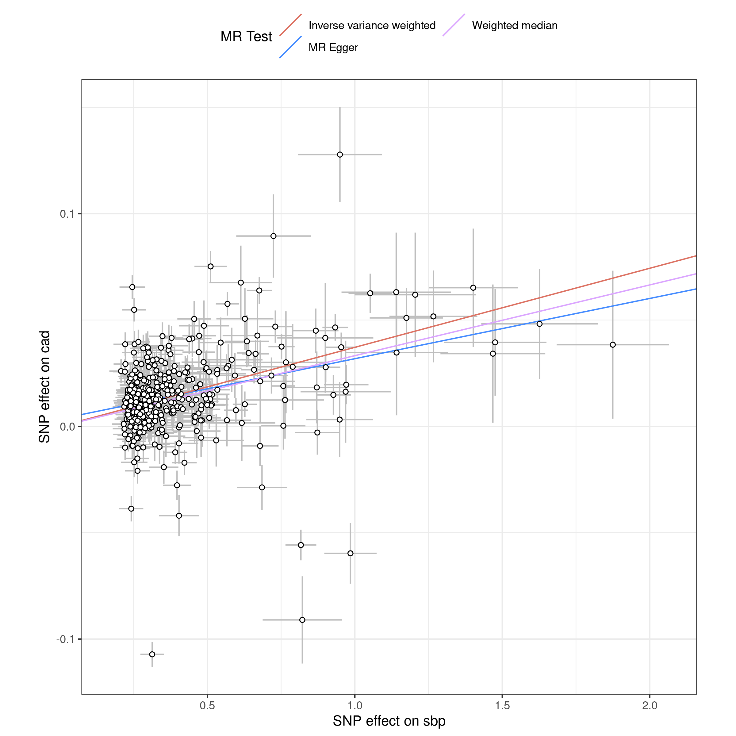

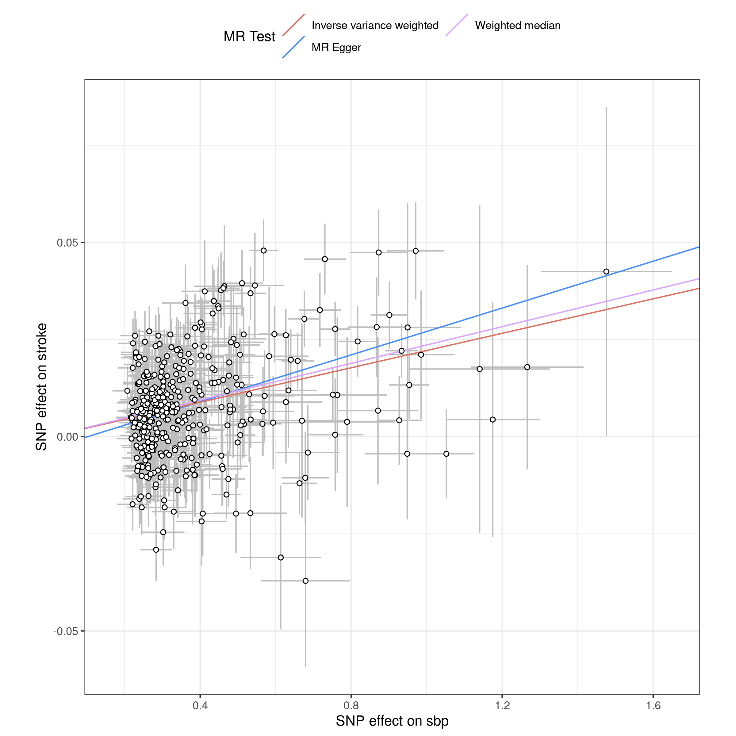


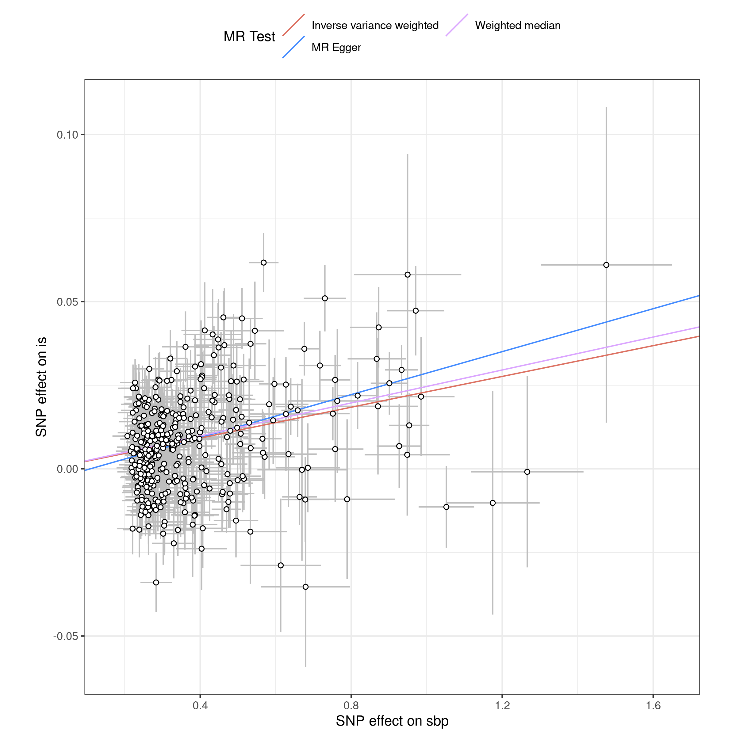

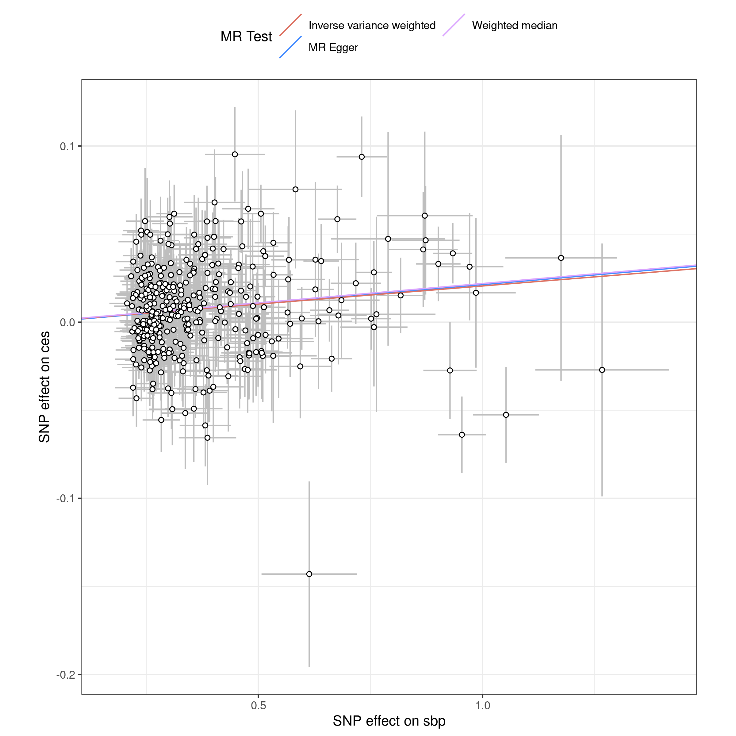


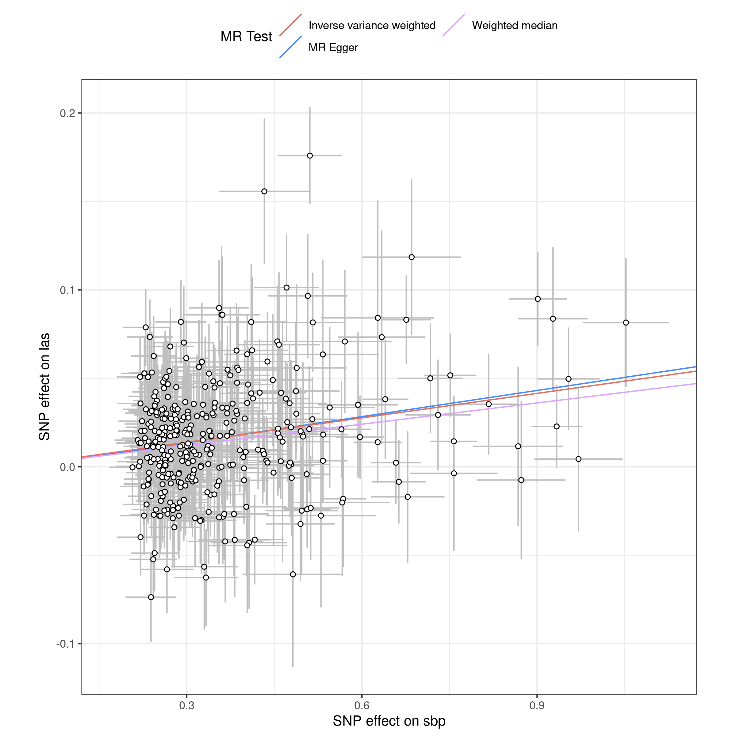

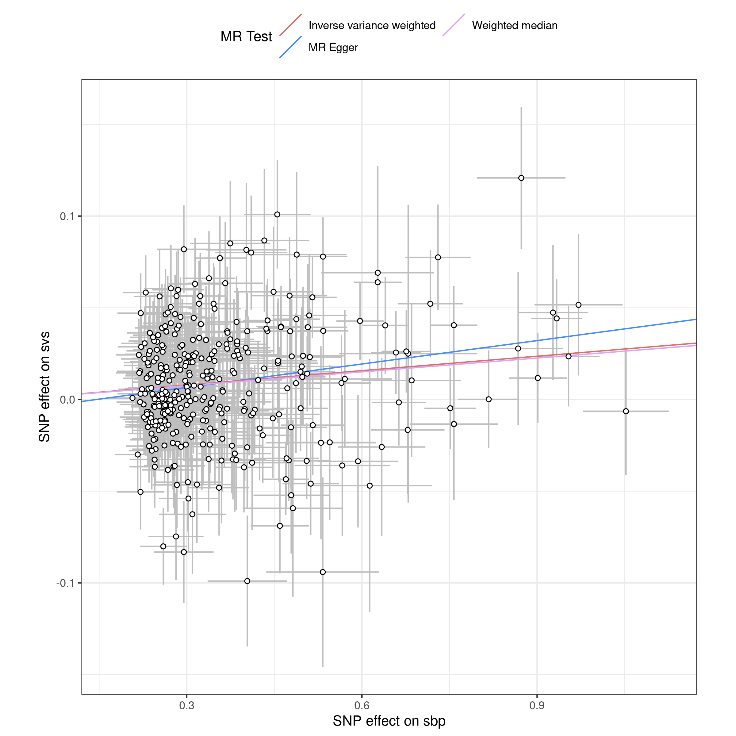


**Supplementary Figure 20.** Scatter plots generated from MR analyses for the effect of SBP on CAD, AS, AIS, CES, LAS, and SVS.


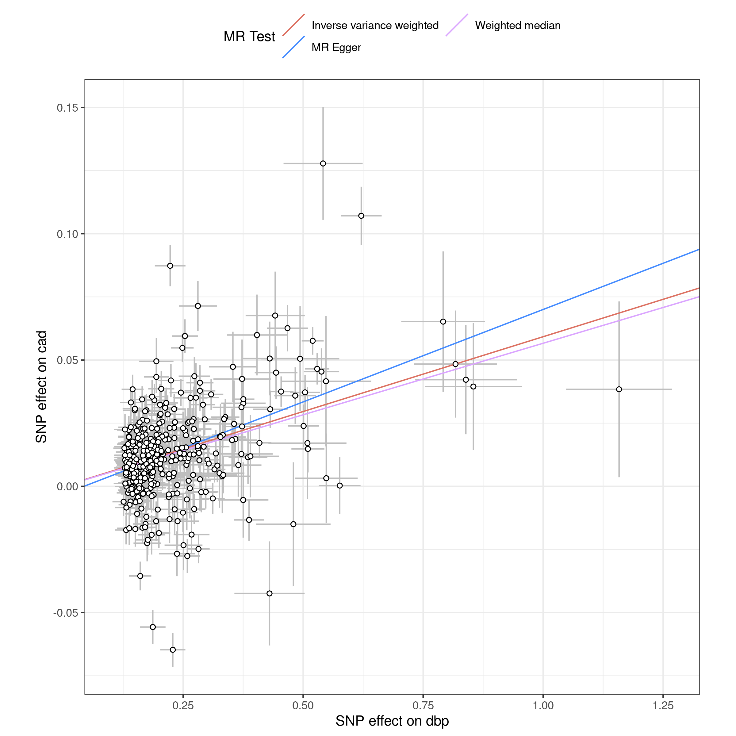

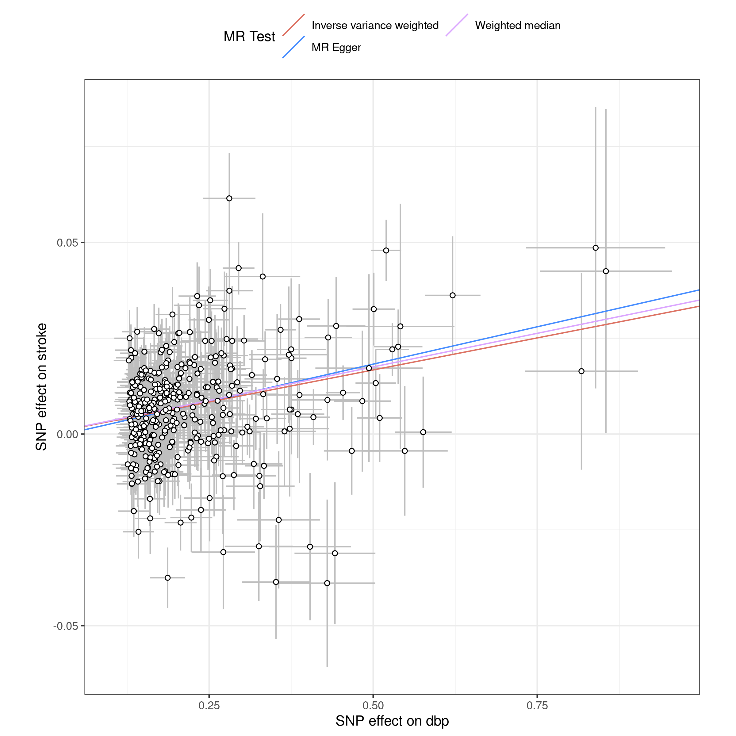


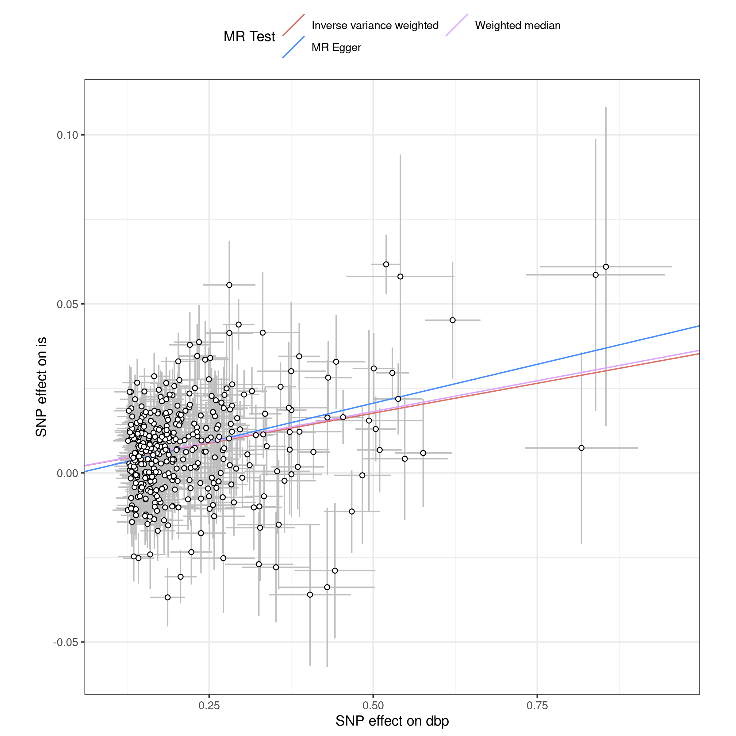

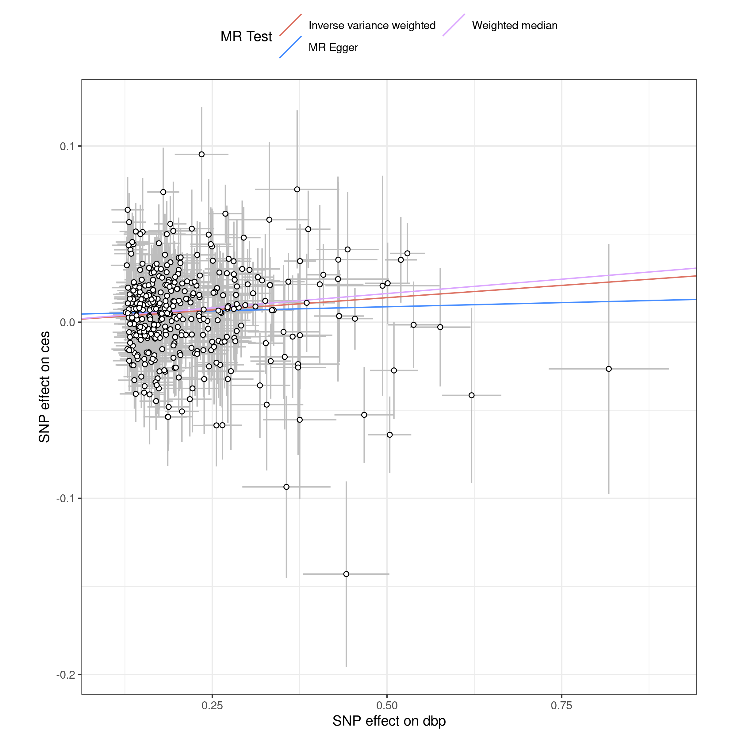


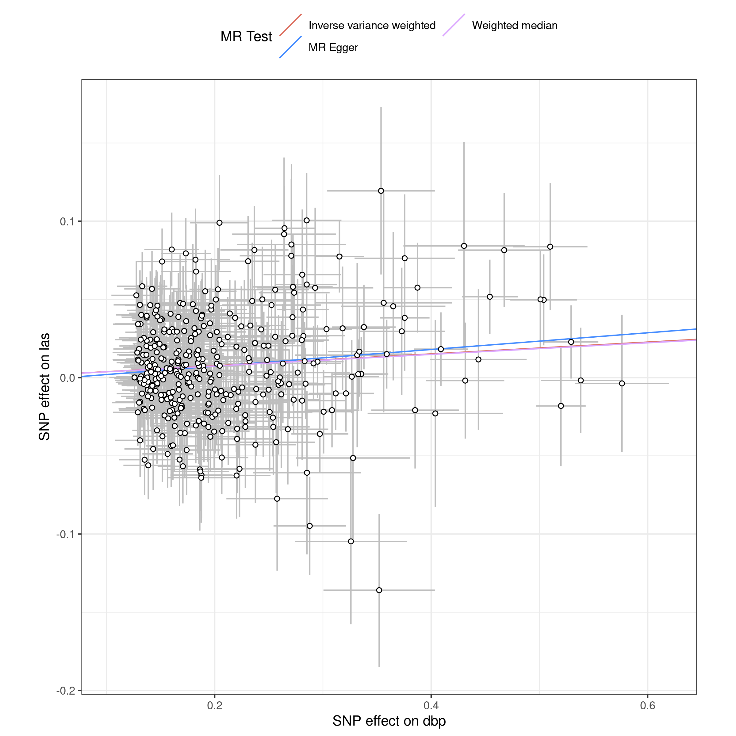

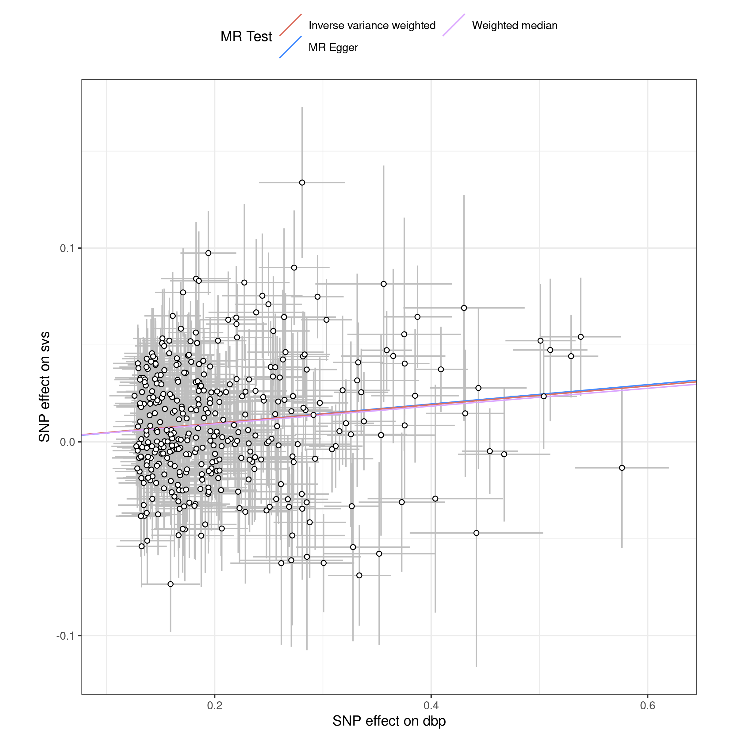


**Supplementary Figure 21.** Scatter plots generated from MR analyses for the effect of DBP on CAD, AS, AIS, CES, LAS, and SVS.


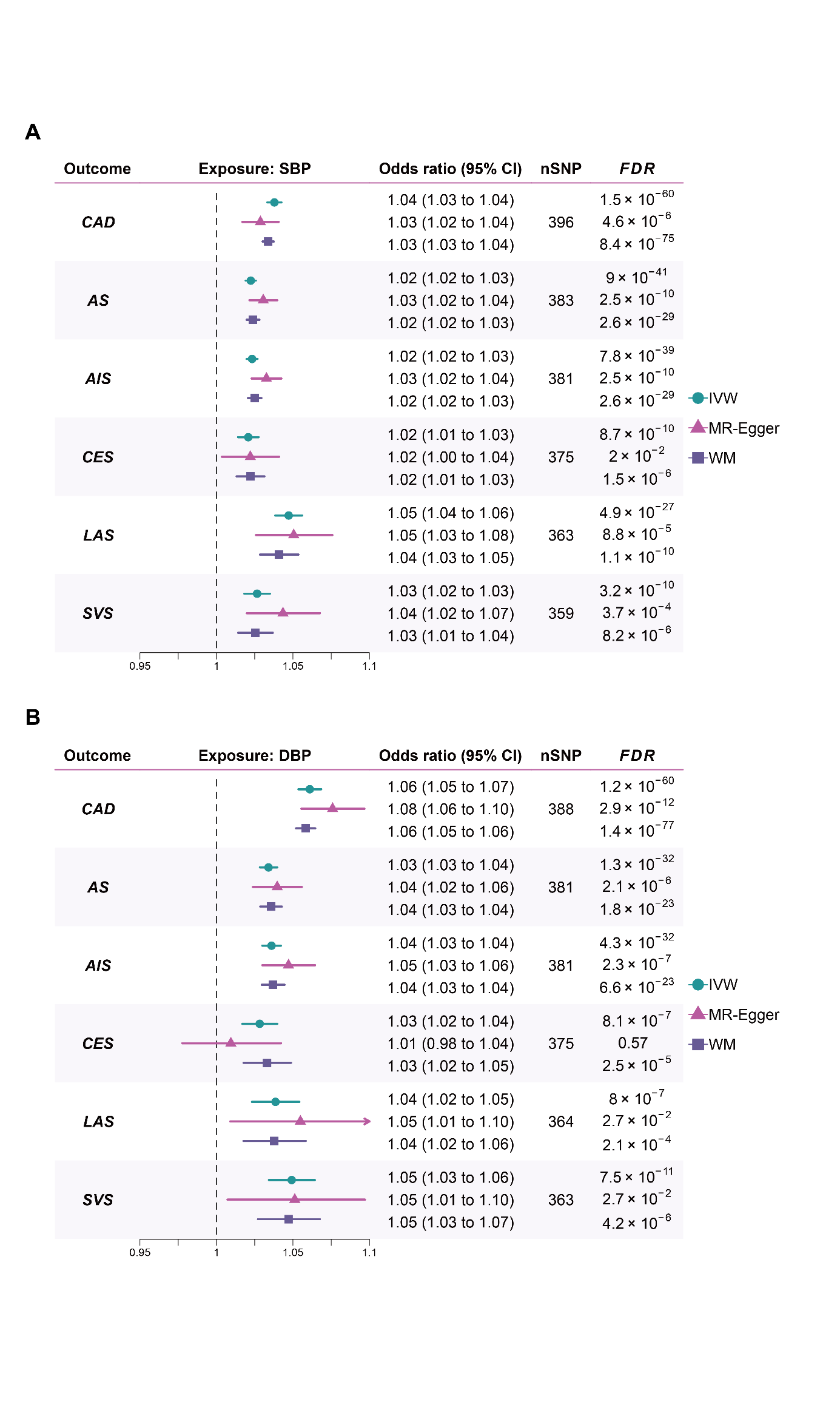


**Supplementary Figure 22.** MR analyses for the effect of blood pressure on cardiovascular outcomes.

**A.** Forest plots illustrating the effect of systolic blood pressure on cardiovascular outcomes; and **B.** diastolic blood pressure on cardiovascular outcomes.


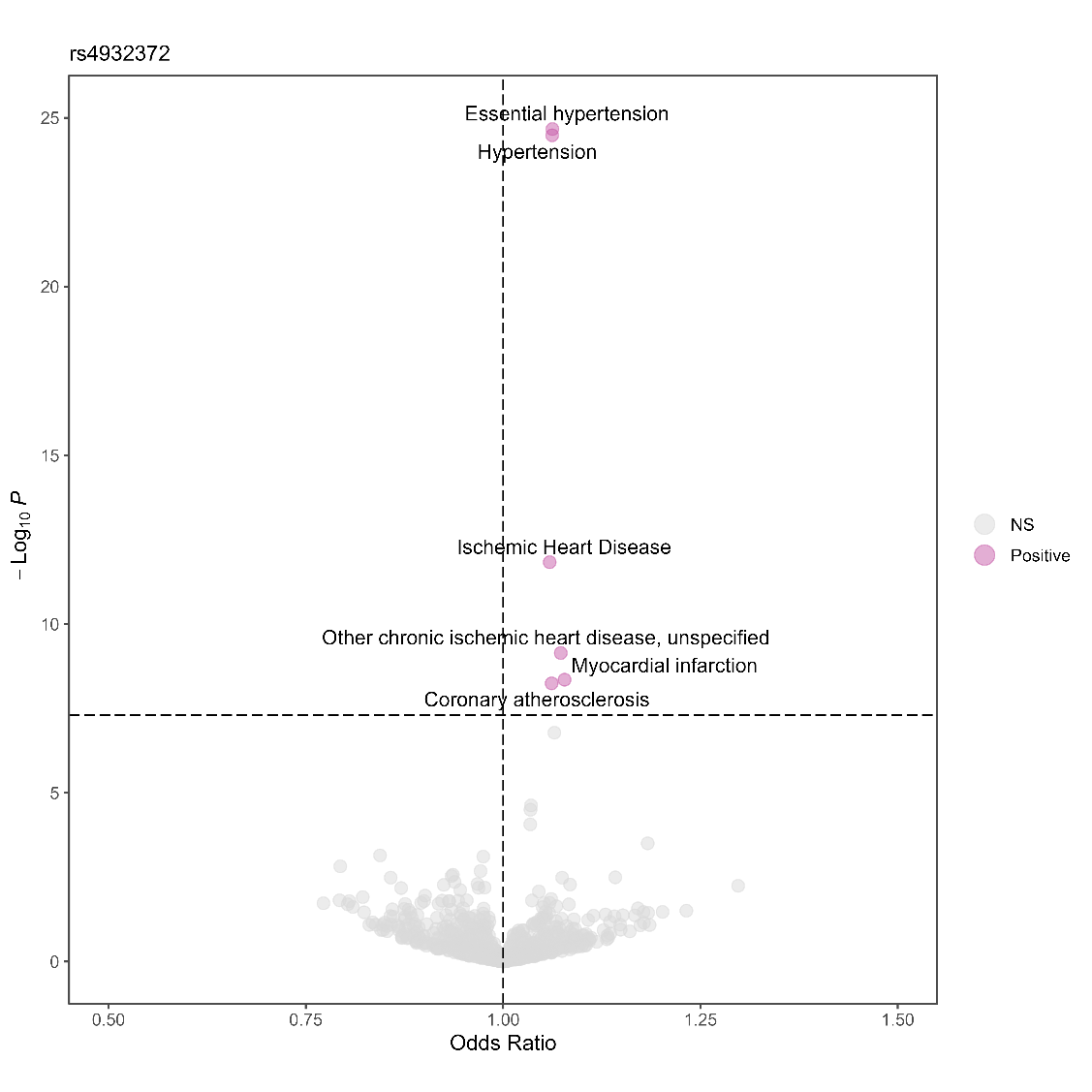


**Supplementary Figure 23.** Volcano plots showing phenotypes associated with FURIN polymorphism at rs4932372 from PheWAS analysis. Coloured points represent phenotypes reach p < 5e-8.


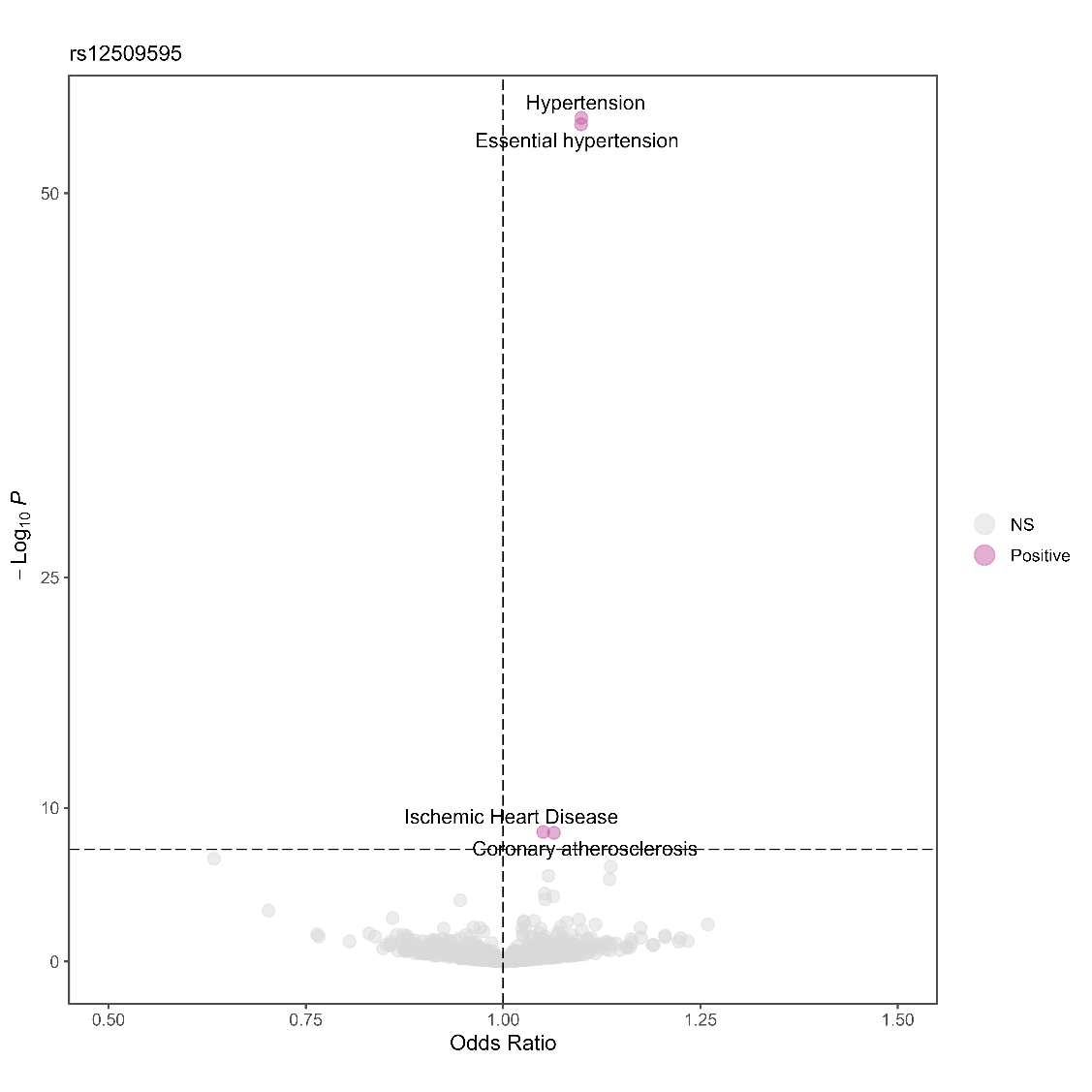


**Supplementary Figure 24.** Volcano plots showing phenotypes associated with FGF5 polymorphism at rs12509595 from PheWAS analysis. Coloured points represent phenotypes reach p < 5e-8.
