# Supplementary material for: Blood pressure, plasma proteins, and cardiovascular diseases: a network Mendelian randomization and observational study
Source: Eur Heart J. 2025 Oct 9;47(3):331–42. doi: 10.1093/eurheartj/ehaf725 (PMC12807568; doi:10.1093/eurheartj/ehaf725)
Supplement: ehaf725_Supplementary_Data [file ehaf725_supplementary_data.zip › Supplementary_Tables.pdf]

Blood pressure, circulating plasma proteins and cardiovascular diseases: A network Mendelian randomisation and observational study

Sheet number	Sheet name	Table
1	Index	Index
2	ST1	ST1: Data sources used in the MR analysis.
3	ST2	ST2: MR causal estimates for plasma proteins on systolic blood pressure.
4	ST3	ST3: MR causal estimates for plasma proteins on diastolic blood pressure.
5	ST4	ST4: MR causal estimates for blood pressure measures on plasma proteins.
6	ST5	ST5: MR causal estimates for SBP-associated proteins on CAD.
7	ST6	ST6: MR causal estimates for DBP-associated proteins on CAD.
8	ST7	ST7: MR causal estimates for SBP-associated proteins on all stroke.
9	ST8	ST8: MR causal estimates for DBP-associated proteins on all stroke.
10	ST9	ST9: MR causal estimates for SBP-associated proteins on ischemic stroke.
11	ST10	ST10: MR causal estimates for DBP-associated proteins on ischemic stroke.
12	ST11	ST11: MR causal estimates for SBP-associated proteins on small vessel stroke.
13	ST12	ST12: MR causal estimates for DBP-associated proteins on small vessel stroke.
14	ST13	ST13: MR causal estimates for SBP-associated proteins on cardioembolic stroke.
15	ST14	ST14: MR causal estimates for DBP-associated proteins on cardioembolic stroke.
16	ST15	ST15: MR causal estimates for SBP-associated proteins on large artery stroke.
17	ST16	ST16: MR causal estimates for DBP-associated proteins on large artery stroke.
18	ST17	ST17: MR causal estimates for CAD and stroke on BP-associated proteins.
19	ST18	ST18: Protein-Protein interactions and respective scores derived from the STRING v12.0 database.
20	ST19	ST19: Bayesian colocalization analysis with BP for shared causal proteins between CVDs and BP.
21	ST20	ST20: Bayesian colocalization analysis with CVDs for 13 proteins shared between BP and CVDs.
22	ST21	ST21: PairWise Conditional and Colocalisation (PWCoCo) for 22 BP-associated proteins with overlapped genomic windows.
23	ST22	ST22: Lookup of cis-pQTLs for the prioritised protein in publicly available eQTL databases.
24	ST23	ST23: Multi-trait colocalization analysis for shared causal proteins between CVDs and BP.
25	ST24	ST24; Genetic variants used in Mendelian randomization to explore the causal relation of the prioritized plasma proteins (ACOX1, FGF5, FURIN and MST1) with BP and CVDs.
26	ST25	ST25: MR causal estimates for blood pressure measures on CAD and stroke.
27	ST26	ST26: Table showing components of mediation analysis and proportion mediated.
28	ST27	ST27: Comparative analysis of protein levels in hypertensive individuals within UK Biobank separated by BP medication usage.
29	ST28	ST28: Effects of plasma proteins on BP in observational analysis using linear regression models.
30	ST29	ST29: Effects of plasma proteins on CAD and stroke in observational analysis using Cox proportional hazard models.
31	ST30	ST30: Effects of plasma proteins on composite CVD (CAD and stroke) in observational analysis using Cox proportional hazard models.
32	ST31	ST31: PheWAS results for the lead cis-pQTLs of the 4 proteins prioritized (ACOX1, FGF5, FURIN, and MST1).

ST1; Data sources used in the MR analysis.

Category	Phenotype	No of participants	Ancestry	Consortium/cohort	Author	PMID
Exposure	Circulating plasma proteins	54,219	European	UKBPPP	Sun et al, 2023	37794186
Mediator	Blood pressure	410,170	European	UKB	In-house data	NA
Outcome	Coronary artery disease	122,733 cases and 547,261 controls	European	UKB + CARDIoGRAMplusC4D	Harst et al, 2018	29212778
Outcome	Stroke	73,652 cases and 1,234,808 controls	European	GIGASTROKE	Mishra et al, 2022	36180795
Outcome	Ischemic stroke	62,100 cases and 1,234,808 controls	European	GIGASTROKE	Mishra et al, 2022	36180795
Outcome	Cardioembolic stroke	10,804 cases and 1,234,808 controls	European	GIGASTROKE	Mishra et al, 2022	36180795
Outcome	Large artery stroke	6,399 cases and 1,234,808 controls	European	GIGASTROKE	Mishra et al, 2022	36180795
Outcome	Small vessel stroke	6,811 cases and 1,234,808 controls	European	GIGASTROKE	Mishra et al, 2022	36180795

ST2; MR causal estimates for plasma proteins on systolic blood pressure.

Causal candidates prioritized for SBP were marked as "Yes" in column "Prioritized". Effect of plasma protein levels on blood pressure is in mmHg unit.

										Steiger filtering					
**Exposure**	**Outocme**	**Method**	**nsnp**	**Beta**	**SE**	**P-value**	**Cochran's Q P-value**	**FDR-corrected P-value**	**Prioritized**	**nsnp**	**Beta**	**SE**	**P-value**	**Cochran's Q P-value**	**FDR-corrected P-value**
A1BG	SBP	Inverse variance weighted	5	0.269	0.370	0.467	0.035	0.818	No	5	0.269	0.370	0.467	0.035	0.818
AAMDC	SBP	Inverse variance weighted	3	-0.151	0.095	0.112	0.077	0.397	No	3	-0.151	0.095	0.112	0.077	0.397
AARSD1	SBP	Wald ratio	1	0.246	0.334	0.462	-	0.817	No	1	0.246	0.334	0.462	-	0.817
ABHD14B	SBP	Inverse variance weighted	2	0.094	0.144	0.515	-	0.846	No	2	0.094	0.144	0.515	-	0.846
ABL1	SBP	Wald ratio	1	-1.708	0.935	0.068	-	0.302	No	1	-1.708	0.935	0.068	-	0.302
ABO	SBP	Inverse variance weighted	9	-0.064	0.036	0.074	0.869	0.318	No	9	-0.064	0.036	0.074	0.869	0.318
ACAA1	SBP	Inverse variance weighted	2	0.073	0.343	0.832	-	0.966	No	2	0.073	0.343	0.832	-	0.966
ACADM	SBP	Wald ratio	1	-0.565	0.303	0.062	-	0.292	No	1	-0.565	0.303	0.062	-	0.292
ACADSB	SBP	Inverse variance weighted	4	-0.097	0.268	0.717	0.094	0.937	No	4	-0.097	0.268	0.717	0.094	0.937
ACAN	SBP	Inverse variance weighted	2	0.538	0.343	0.116	-	0.407	No	2	0.538	0.343	0.116	-	0.407
ACE	SBP	Inverse variance weighted	4	0.200	0.271	0.460	0.000	0.816	No	4	0.200	0.271	0.460	0.000	0.816
ACHE	SBP	Inverse variance weighted	6	0.167	0.118	0.158	0.848	0.492	No	6	0.167	0.118	0.158	0.848	0.492
ACOT13	SBP	Inverse variance weighted	2	-0.829	0.519	0.110	-	0.392	No	2	-0.829	0.519	0.110	-	0.392
ACOX1	SBP	Wald ratio	1	2.428	0.543	0.000	-	0.000	Yes	1	2.428	0.543	0.000	-	0.000
ACP1	SBP	Inverse variance weighted	3	-0.155	0.211	0.463	0.022	0.817	No	3	-0.155	0.211	0.463	0.022	0.817
ACP5	SBP	Inverse variance weighted	6	-0.020	0.202	0.922	0.005	0.982	No	6	-0.020	0.202	0.922	0.005	0.982
ACP6	SBP	Inverse variance weighted	8	-0.099	0.048	0.038	0.210	0.222	No	8	-0.099	0.048	0.038	0.210	0.222
ACRBP	SBP	Wald ratio	1	0.871	0.264	0.001	-	0.014	Yes	1	0.871	0.264	0.001	-	0.014
ACRV1	SBP	Wald ratio	1	0.207	0.699	0.767	-	0.950	No	1	0.207	0.699	0.767	-	0.950
ACTA2	SBP	Wald ratio	1	0.266	0.766	0.729	-	0.943	No	1	0.266	0.766	0.729	-	0.943
ACVRL1	SBP	Inverse variance weighted	6	-0.240	0.129	0.062	0.783	0.292	No	6	-0.240	0.129	0.062	0.783	0.292
ACY1	SBP	Inverse variance weighted	2	-0.534	0.325	0.100	-	0.376	No	2	-0.534	0.325	0.100	-	0.376
ACY3	SBP	Inverse variance weighted	4	-0.043	0.145	0.768	0.769	0.950	No	4	-0.043	0.145	0.768	0.769	0.950
ACYP1	SBP	Inverse variance weighted	3	-0.241	0.146	0.099	0.777	0.372	No	3	-0.241	0.146	0.099	0.777	0.372
ADA	SBP	Inverse variance weighted	7	0.130	0.111	0.239	0.001	0.620	No	7	0.130	0.111	0.239	0.001	0.620
ADA2	SBP	Inverse variance weighted	3	0.049	0.145	0.733	0.437	0.943	No	3	0.049	0.145	0.733	0.437	0.943
ADAM12	SBP	Inverse variance weighted	3	-0.124	0.216	0.566	0.413	0.870	No	3	-0.124	0.216	0.566	0.413	0.870
ADAM15	SBP	Inverse variance weighted	11	-0.015	0.054	0.783	0.324	0.953	No	11	-0.015	0.054	0.783	0.324	0.953
ADAM22	SBP	Inverse variance weighted	5	0.140	0.173	0.418	0.747	0.790	No	5	0.140	0.173	0.418	0.747	0.790
ADAM23	SBP	Inverse variance weighted	9	-0.193	0.067	0.004	0.283	0.044	Yes	9	-0.193	0.067	0.004	0.283	0.044
ADAM8	SBP	Inverse variance weighted	8	0.095	0.081	0.243	0.708	0.625	No	8	0.095	0.081	0.243	0.708	0.625
ADAM9	SBP	Inverse variance weighted	5	0.393	0.398	0.324	0.060	0.705	No	5	0.393	0.398	0.324	0.060	0.705
ADAMTS1	SBP	Wald ratio	1	2.660	0.874	0.002	-	0.029	Yes	1	2.660	0.874	0.002	-	0.029
ADAMTS13	SBP	Inverse variance weighted	6	0.008	0.094	0.930	0.618	0.983	No	6	0.008	0.094	0.930	0.618	0.983
ADAMTS15	SBP	Inverse variance weighted	3	-0.478	1.036	0.645	0.000	0.904	No	3	-0.478	1.036	0.645	0.000	0.904
ADAMTS16	SBP	Inverse variance weighted	5	-0.682	0.421	0.105	0.004	0.384	No	5	-0.682	0.421	0.105	0.004	0.384
ADAMTS4	SBP	Wald ratio	1	0.990	0.335	0.003	-	0.037	Yes	1	0.990	0.335	0.003	-	0.037
ADAMTS8	SBP	Inverse variance weighted	7	-0.484	0.112	0.000	0.053	0.001	Yes	7	-0.484	0.112	0.000	0.053	0.001
ADAMTSL2	SBP	Inverse variance weighted	4	0.249	0.314	0.429	0.379	0.790	No	4	0.249	0.314	0.429	0.379	0.790
ADAMTSL4	SBP	Inverse variance weighted	3	-0.667	0.246	0.007	0.224	0.066	No	3	-0.667	0.246	0.007	0.224	0.066
ADAMTSL5	SBP	Inverse variance weighted	5	-0.428	0.102	0.000	0.812	0.001	Yes	5	-0.428	0.102	0.000	0.812	0.001
ADD1	SBP	Wald ratio	1	-0.189	0.283	0.505	-	0.842	No	1	-0.189	0.283	0.505	-	0.842
ADGRB3	SBP	Inverse variance weighted	12	0.012	0.064	0.849	0.006	0.967	No	12	0.012	0.064	0.849	0.006	0.967
ADGRD1	SBP	Inverse variance weighted	8	0.117	0.117	0.317	0.140	0.699	No	8	0.117	0.117	0.317	0.140	0.699
ADGRE1	SBP	Inverse variance weighted	5	0.001	0.200	0.998	0.038	0.999	No	5	0.001	0.200	0.998	0.038	0.999
ADGRE2	SBP	Inverse variance weighted	4	0.315	0.166	0.058	0.319	0.283	No	4	0.315	0.166	0.058	0.319	0.283
ADGRE5	SBP	Inverse variance weighted	4	0.192	0.252	0.445	0.108	0.801	No	4	0.192	0.252	0.445	0.108	0.801
ADGRG1	SBP	Inverse variance weighted	4	-0.420	0.395	0.287	0.727	0.675	No	4	-0.420	0.395	0.287	0.727	0.675
ADH1B	SBP	Inverse variance weighted	2	3.878	2.478	0.118	-	0.408	No	2	3.878	2.478	0.118	-	0.408
ADH4	SBP	Wald ratio	1	-0.798	0.311	0.010	-	0.090	No	1	-0.798	0.311	0.010	-	0.090
ADIPOQ	SBP	Inverse variance weighted	8	-0.423	0.169	0.012	0.519	0.105	No	8	-0.423	0.169	0.012	0.519	0.105
ADM	SBP	Wald ratio	1	4.295	1.131	0.000	-	0.003	Yes	1	4.295	1.131	0.000	-	0.003
AFAP1	SBP	Inverse variance weighted	6	0.109	0.040	0.006	0.570	0.061	No	6	0.109	0.040	0.006	0.570	0.061
AFM	SBP	Inverse variance weighted	4	-0.460	0.429	0.283	0.120	0.671	No	4	-0.460	0.429	0.283	0.120	0.671
AFP	SBP	Inverse variance weighted	2	0.171	0.219	0.434	-	0.794	No	2	0.171	0.219	0.434	-	0.794
AGR2	SBP	Wald ratio	1	-0.041	0.395	0.917	-	0.982	No	1	-0.041	0.395	0.917	-	0.982

ST2; MR causal estimates for plasma proteins on systolic blood pressure.

Causal candidates prioritized for SBP were marked as "Yes" in column "Prioritized". Effect of plasma protein levels on blood pressure is in mmHg unit.

										Steiger filtering					
Exposure	Outocme	Method	nsnp	Beta	SE	P-value	Cochran's Q P-value	FDR-corrected P-value	Prioritized	nsnp	Beta	SE	P-value	Cochran's Q P-value	FDR-corrected P-value
AGR3	SBP	Wald ratio	1	-1.511	1.087	0.165	-	0.501	No	1	-1.511	1.087	0.165	-	0.501
AGRN	SBP	Inverse variance weighted	3	0.130	0.112	0.247	0.637	0.628	No	3	0.130	0.112	0.247	0.637	0.628
AGRP	SBP	Inverse variance weighted	5	0.250	0.343	0.466	0.835	0.817	No	5	0.250	0.343	0.466	0.835	0.817
AGT	SBP	Inverse variance weighted	4	0.142	0.083	0.089	0.673	0.353	No	4	0.142	0.083	0.089	0.673	0.353
AGXT	SBP	Inverse variance weighted	3	0.151	0.143	0.289	0.977	0.675	No	3	0.151	0.143	0.289	0.977	0.675
AHCY	SBP	Inverse variance weighted	2	0.028	0.115	0.809	-	0.961	No	2	0.028	0.115	0.809	-	0.961
AH-K	SBP	Wald ratio	1	-0.399	0.243	0.101	-	0.376	No	1	-0.399	0.243	0.101	-	0.376
AH-K2	SBP	Inverse variance weighted	3	0.403	0.270	0.135	0.132	0.443	No	3	0.403	0.270	0.135	0.132	0.443
AHSG	SBP	Inverse variance weighted	6	-0.103	0.058	0.078	0.678	0.327	No	6	-0.103	0.058	0.078	0.678	0.327
AHSP	SBP	Wald ratio	1	0.286	0.975	0.770	-	0.950	No	1	0.286	0.975	0.770	-	0.950
AIF1L	SBP	Inverse variance weighted	2	0.276	0.147	0.061	-	0.288	No	2	0.276	0.147	0.061	-	0.288
AK1	SBP	Wald ratio	1	-0.911	0.906	0.315	-	0.696	No	1	-0.911	0.906	0.315	-	0.696
AK2	SBP	Wald ratio	1	0.239	1.019	0.815	-	0.963	No	1	0.239	1.019	0.815	-	0.963
AKAP12	SBP	Inverse variance weighted	2	0.126	0.677	0.852	-	0.967	No	2	0.126	0.677	0.852	-	0.967
AKR1B1	SBP	Inverse variance weighted	2	0.520	0.273	0.057	-	0.280	No	2	0.520	0.273	0.057	-	0.280
AKR1C4	SBP	Wald ratio	1	0.235	0.732	0.749	-	0.946	No	1	0.235	0.732	0.749	-	0.946
AKR7L	SBP	Inverse variance weighted	2	-0.053	0.205	0.798	-	0.958	No	2	-0.053	0.205	0.798	-	0.958
AKT3	SBP	Wald ratio	1	-0.549	0.728	0.451	-	0.807	No	1	-0.549	0.728	0.451	-	0.807
ALCAM	SBP	Inverse variance weighted	3	-0.129	0.252	0.607	0.510	0.887	No	3	-0.129	0.252	0.607	0.510	0.887
ALDH1A1	SBP	Wald ratio	1	-1.058	0.498	0.034	-	0.207	No	1	-1.058	0.498	0.034	-	0.207
ALDH3A1	SBP	Inverse variance weighted	2	0.061	0.129	0.637	-	0.901	No	2	0.061	0.129	0.637	-	0.901
ALDH5A1	SBP	Inverse variance weighted	2	0.255	0.251	0.311	-	0.696	No	2	0.255	0.251	0.311	-	0.696
ALPP	SBP	Inverse variance weighted	4	0.063	0.082	0.444	0.610	0.801	No	4	0.063	0.082	0.444	0.610	0.801
AMBN	SBP	Wald ratio	1	0.949	0.597	0.112	-	0.397	No	1	0.949	0.597	0.112	-	0.397
AMBP	SBP	Inverse variance weighted	4	0.029	0.284	0.919	0.513	0.982	No	4	0.029	0.284	0.919	0.513	0.982
AMFR	SBP	Wald ratio	1	1.837	0.933	0.049	-	0.256	No	1	1.837	0.933	0.049	-	0.256
AMIGO1	SBP	Wald ratio	1	-0.342	0.935	0.714	-	0.934	No	1	-0.342	0.935	0.714	-	0.934
AMIGO2	SBP	Inverse variance weighted	3	-0.184	0.282	0.515	0.198	0.847	No	3	-0.184	0.282	0.515	0.198	0.847
AMN	SBP	Inverse variance weighted	6	0.039	0.072	0.590	0.164	0.879	No	6	0.039	0.072	0.590	0.164	0.879
AMOTL2	SBP	Wald ratio	1	-3.977	0.772	0.000	-	0.000	Yes	1	-3.977	0.772	0.000	-	0.000
AMPD3	SBP	Inverse variance weighted	7	-0.439	0.319	0.169	0.001	0.510	No	7	-0.439	0.319	0.169	0.001	0.510
AMY2A	SBP	Inverse variance weighted	15	-0.015	0.086	0.866	0.331	0.971	No	15	-0.015	0.086	0.866	0.331	0.971
AMY2B	SBP	Inverse variance weighted	15	-0.018	0.083	0.824	0.309	0.966	No	15	-0.018	0.083	0.824	0.309	0.966
ANG	SBP	Inverse variance weighted	5	0.155	0.089	0.083	0.597	0.337	No	5	0.155	0.089	0.083	0.597	0.337
ANGPT1	SBP	Wald ratio	1	-1.087	0.752	0.149	-	0.472	No	1	-1.087	0.752	0.149	-	0.472
ANGPT2	SBP	Inverse variance weighted	3	-0.132	0.209	0.528	0.451	0.855	No	3	-0.132	0.209	0.528	0.451	0.855
ANGPTL1	SBP	Inverse variance weighted	5	-0.054	0.167	0.748	0.092	0.946	No	5	-0.054	0.167	0.748	0.092	0.946
ANGPTL2	SBP	Wald ratio	1	-1.532	0.724	0.034	-	0.209	No	1	-1.532	0.724	0.034	-	0.209
ANGPTL3	SBP	Inverse variance weighted	2	-0.032	0.147	0.829	-	0.966	No	2	-0.032	0.147	0.829	-	0.966
ANGPTL4	SBP	Inverse variance weighted	2	-0.470	0.239	0.049	-	0.256	No	2	-0.470	0.239	0.049	-	0.256
ANGPTL7	SBP	Inverse variance weighted	3	0.320	0.281	0.255	0.005	0.638	No	3	0.320	0.281	0.255	0.005	0.638
ANKMY2	SBP	Wald ratio	1	-1.679	0.521	0.001	-	0.018	Yes	1	-1.679	0.521	0.001	-	0.018
ANKRD54	SBP	Wald ratio	1	0.889	0.591	0.132	-	0.437	No	1	0.889	0.591	0.132	-	0.437
ANPEP	SBP	Inverse variance weighted	12	0.200	0.097	0.040	0.179	0.225	No	12	0.200	0.097	0.040	0.179	0.225
ANXA1	SBP	Wald ratio	1	-1.003	0.920	0.276	-	0.662	No	1	-1.003	0.920	0.276	-	0.662
ANXA10	SBP	Wald ratio	1	1.297	0.878	0.140	-	0.455	No	1	1.297	0.878	0.140	-	0.455
ANXA11	SBP	Wald ratio	1	-1.194	0.654	0.068	-	0.302	No	1	-1.194	0.654	0.068	-	0.302
ANXA2	SBP	Inverse variance weighted	4	0.068	0.163	0.676	0.012	0.920	No	4	0.068	0.163	0.676	0.012	0.920
ANXA3	SBP	Inverse variance weighted	2	-0.430	0.538	0.425	-	0.790	No	2	-0.430	0.538	0.425	-	0.790
ANXA4	SBP	Inverse variance weighted	2	0.671	0.688	0.330	-	0.711	No	2	0.671	0.688	0.330	-	0.711
ANXA5	SBP	Wald ratio	1	0.631	0.395	0.110	-	0.392	No	1	0.631	0.395	0.110	-	0.392
AOC1	SBP	Inverse variance weighted	3	-0.484	0.255	0.058	0.035	0.283	No	3	-0.484	0.255	0.058	0.035	0.283
AOC3	SBP	Inverse variance weighted	5	0.141	0.176	0.425	0.000	0.790	No	5	0.141	0.176	0.425	0.000	0.790
AP1G2	SBP	Inverse variance weighted	2	0.349	1.095	0.750	-	0.946	No	2	0.349	1.095	0.750	-	0.946
AP3B1	SBP	Wald ratio	1	-0.136	0.762	0.859	-	0.969	No	1	-0.136	0.762	0.859	-	0.969
APBB1IP	SBP	Wald ratio	1	0.227	0.186	0.223	-	0.600	No	1	0.227	0.186	0.223	-	0.600

ST2; MR causal estimates for plasma proteins on systolic blood pressure.

Causal candidates prioritized for SBP were marked as "Yes" in column "Prioritized". Effect of plasma protein levels on blood pressure is in mmHg unit.

										Steiger filtering					
**Exposure**	**Outocme**	**Method**	**nsnp**	**Beta**	**SE**	**P-value**	**Cochran's Q P-value**	**FDR-corrected P-value**	**Prioritized**	**nsnp**	**Beta**	**SE**	**P-value**	**Cochran's Q P-value**	**FDR-corrected P-value**
APCS	SBP	Inverse variance weighted	4	-0.166	0.340	0.626	0.033	0.897	No	4	-0.166	0.340	0.626	0.033	0.897
APEX1	SBP	Inverse variance weighted	3	0.165	0.127	0.192	0.196	0.553	No	3	0.165	0.127	0.192	0.196	0.553
APOA1	SBP	Wald ratio	1	2.613	0.629	0.000	-	0.001	Yes	1	2.613	0.629	0.000	-	0.001
APOA2	SBP	Wald ratio	1	-1.841	0.627	0.003	-	0.039	Yes	1	-1.841	0.627	0.003	-	0.039
APOA4	SBP	Wald ratio	1	0.085	0.292	0.771	-	0.952	No	1	0.085	0.292	0.771	-	0.952
APOBR	SBP	Inverse variance weighted	8	-0.166	0.053	0.002	0.034	0.022	Yes	8	-0.166	0.053	0.002	0.034	0.022
APOC1	SBP	Inverse variance weighted	2	-1.463	0.469	0.002	-	0.024	Yes	2	-1.463	0.469	0.002	-	0.024
APOD	SBP	Inverse variance weighted	2	-0.531	0.201	0.008	-	0.077	No	2	-0.531	0.201	0.008	-	0.077
APOE	SBP	Inverse variance weighted	9	-0.183	0.091	0.045	0.002	0.241	No	9	-0.183	0.091	0.045	0.002	0.241
APOF	SBP	Inverse variance weighted	3	-0.101	0.219	0.644	0.771	0.904	No	3	-0.101	0.219	0.644	0.771	0.904
APOH	SBP	Inverse variance weighted	5	0.129	0.142	0.363	0.001	0.747	No	5	0.129	0.142	0.363	0.001	0.747
APOL1	SBP	Wald ratio	1	-0.422	0.165	0.011	-	0.093	No	1	-0.422	0.165	0.011	-	0.093
APP	SBP	Wald ratio	1	0.436	0.604	0.471	-	0.821	No	1	0.436	0.604	0.471	-	0.821
APPL2	SBP	Wald ratio	1	-0.121	0.312	0.698	-	0.925	No	1	-0.121	0.312	0.698	-	0.925
APRT	SBP	Inverse variance weighted	2	1.179	0.607	0.052	-	0.263	No	2	1.179	0.607	0.052	-	0.263
AREG	SBP	Wald ratio	1	0.084	0.263	0.750	-	0.946	No	1	0.084	0.263	0.750	-	0.946
ARFIP1	SBP	Wald ratio	1	-0.046	0.259	0.859	-	0.969	No	1	-0.046	0.259	0.859	-	0.969
ARG1	SBP	Inverse variance weighted	3	-0.108	0.247	0.663	0.247	0.915	No	3	-0.108	0.247	0.663	0.247	0.915
ARG2	SBP	Wald ratio	1	-0.206	0.767	0.788	-	0.955	No	1	-0.206	0.767	0.788	-	0.955
ARHGAP25	SBP	Inverse variance weighted	2	-0.161	0.865	0.852	-	0.967	No	2	-0.161	0.865	0.852	-	0.967
ARHGAP45	SBP	Wald ratio	1	1.170	0.559	0.036	-	0.216	No	1	1.170	0.559	0.036	-	0.216
ARHGEF10	SBP	Inverse variance weighted	6	0.022	0.126	0.859	0.270	0.969	No	6	0.022	0.126	0.859	0.270	0.969
ARHGEF12	SBP	Wald ratio	1	-0.692	1.131	0.541	-	0.858	No	1	-0.692	1.131	0.541	-	0.858
ARHGEF5	SBP	Inverse variance weighted	13	0.013	0.111	0.907	0.083	0.980	No	13	0.013	0.111	0.907	0.083	0.980
ARL2BP	SBP	Wald ratio	1	-0.280	0.149	0.060	-	0.288	No	1	-0.280	0.149	0.060	-	0.288
ARSA	SBP	Inverse variance weighted	6	-0.087	0.084	0.300	0.140	0.688	No	6	-0.087	0.084	0.300	0.140	0.688
ARSB	SBP	Inverse variance weighted	5	0.293	0.120	0.014	0.377	0.114	No	5	0.293	0.120	0.014	0.377	0.114
ART3	SBP	Inverse variance weighted	5	-0.064	0.170	0.707	0.289	0.931	No	5	-0.064	0.170	0.707	0.289	0.931
ART5	SBP	Inverse variance weighted	2	0.051	0.233	0.826	-	0.966	No	2	0.051	0.233	0.826	-	0.966
ASAH1	SBP	Inverse variance weighted	6	0.030	0.091	0.743	0.307	0.945	No	6	0.030	0.091	0.743	0.307	0.945
ASAH2	SBP	Inverse variance weighted	8	-0.024	0.066	0.722	0.255	0.939	No	8	-0.024	0.066	0.722	0.255	0.939
ASGR1	SBP	Wald ratio	1	-0.487	0.268	0.069	-	0.306	No	1	-0.487	0.268	0.069	-	0.306
ASGR2	SBP	Inverse variance weighted	5	0.119	0.274	0.664	0.002	0.916	No	5	0.119	0.274	0.664	0.002	0.916
ASPN	SBP	Inverse variance weighted	2	0.565	0.175	0.001	-	0.018	Yes	2	0.565	0.175	0.001	-	0.018
ASPSCR1	SBP	Wald ratio	1	1.963	1.203	0.103	-	0.379	No	1	1.963	1.203	0.103	-	0.379
ASRGL1	SBP	Inverse variance weighted	3	-0.094	0.199	0.637	0.048	0.901	No	3	-0.094	0.199	0.637	0.048	0.901
ASS1	SBP	Wald ratio	1	-2.223	1.228	0.070	-	0.309	No	1	-2.223	1.228	0.070	-	0.309
ATG16L1	SBP	Wald ratio	1	-0.084	1.044	0.936	-	0.984	No	1	-0.084	1.044	0.936	-	0.984
ATOX1	SBP	Wald ratio	1	-0.249	0.552	0.652	-	0.908	No	1	-0.249	0.552	0.652	-	0.908
ATP1B1	SBP	Wald ratio	1	-0.814	0.997	0.414	-	0.787	No	1	-0.814	0.997	0.414	-	0.787
ATP5IF1	SBP	Wald ratio	1	-0.982	0.378	0.009	-	0.085	No	1	-0.982	0.378	0.009	-	0.085
ATRAID	SBP	Inverse variance weighted	3	-0.272	0.254	0.284	0.056	0.672	No	3	-0.272	0.254	0.284	0.056	0.672
ATRN	SBP	Inverse variance weighted	5	0.147	0.066	0.026	0.608	0.169	No	5	0.147	0.066	0.026	0.608	0.169
ATXN10	SBP	Wald ratio	1	0.722	0.454	0.112	-	0.397	No	1	0.722	0.454	0.112	-	0.397
ATXN2L	SBP	Wald ratio	1	2.865	0.989	0.004	-	0.043	Yes	1	2.865	0.989	0.004	-	0.043
ATXN3	SBP	Inverse variance weighted	4	-0.059	0.222	0.790	0.046	0.955	No	4	-0.059	0.222	0.790	0.046	0.955
AXIN1	SBP	Wald ratio	1	-0.572	0.895	0.523	-	0.853	No	1	-0.572	0.895	0.523	-	0.853
AXL	SBP	Inverse variance weighted	2	-0.397	0.226	0.079	-	0.328	No	2	-0.397	0.226	0.079	-	0.328
AZU1	SBP	Inverse variance weighted	3	-0.140	0.277	0.612	0.128	0.890	No	3	-0.140	0.277	0.612	0.128	0.890
B3GNT7	SBP	Inverse variance weighted	6	-0.121	0.062	0.050	0.800	0.257	No	6	-0.121	0.062	0.050	0.800	0.257
B4GALT1	SBP	Wald ratio	1	-0.182	0.206	0.376	-	0.758	No	1	-0.182	0.206	0.376	-	0.758
B4GAT1	SBP	Inverse variance weighted	3	-0.542	0.155	0.000	0.693	0.008	Yes	3	-0.542	0.155	0.000	0.693	0.008
BACH1	SBP	Wald ratio	1	2.015	0.876	0.021	-	0.147	No	1	2.015	0.876	0.021	-	0.147
BAG3	SBP	Inverse variance weighted	2	0.176	0.534	0.742	-	0.945	No	2	0.176	0.534	0.742	-	0.945
BAG4	SBP	Wald ratio	1	-6.813	1.096	0.000	-	0.000	Yes	1	-6.813	1.096	0.000	-	0.000
BAIAP2	SBP	Wald ratio	1	0.047	0.607	0.939	-	0.984	No	1	0.047	0.607	0.939	-	0.984

ST2; MR causal estimates for plasma proteins on systolic blood pressure.

Causal candidates prioritized for SBP were marked as "Yes" in column "Prioritized". Effect of plasma protein levels on blood pressure is in mmHg unit.

										Steiger filtering					
**Exposure**	**Outocme**	**Method**	**nsnp**	**Beta**	**SE**	**P-value**	**Cochran's Q P-value**	**FDR-corrected P-value**	**Prioritized**	**nsnp**	**Beta**	**SE**	**P-value**	**Cochran's Q P-value**	**FDR-corrected P-value**
BAMBI	SBP	Wald ratio	1	-2.394	0.942	0.011	-	0.096	No	1	-2.394	0.942	0.011	-	0.096
BANK1	SBP	Inverse variance weighted	4	-0.131	0.486	0.788	0.035	0.955	No	4	-0.131	0.486	0.788	0.035	0.955
BAP18	SBP	Wald ratio	1	-0.302	0.764	0.693	-	0.924	No	1	-0.302	0.764	0.693	-	0.924
BCAM	SBP	Inverse variance weighted	5	0.751	0.161	0.000	0.381	0.000	Yes	5	0.751	0.161	0.000	0.381	0.000
BCAN	SBP	Inverse variance weighted	2	0.180	0.179	0.313	-	0.696	No	2	0.180	0.179	0.313	-	0.696
BCAT1	SBP	Inverse variance weighted	6	-0.095	0.121	0.435	0.472	0.795	No	6	-0.095	0.121	0.435	0.472	0.795
BCHE	SBP	Inverse variance weighted	7	-0.187	0.077	0.016	0.633	0.121	No	7	-0.187	0.077	0.016	0.633	0.121
BCL2	SBP	Wald ratio	1	-1.244	0.819	0.129	-	0.433	No	1	-1.244	0.819	0.129	-	0.433
BCL2L15	SBP	Inverse variance weighted	2	-0.414	0.403	0.304	-	0.692	No	2	-0.414	0.403	0.304	-	0.692
BCR	SBP	Wald ratio	1	0.017	0.696	0.980	-	0.996	No	1	0.017	0.696	0.980	-	0.996
BDNF	SBP	Inverse variance weighted	2	0.735	0.568	0.196	-	0.559	No	2	0.735	0.568	0.196	-	0.559
BGLAP	SBP	Inverse variance weighted	2	-1.111	0.799	0.164	-	0.501	No	2	-1.111	0.799	0.164	-	0.501
BID	SBP	Inverse variance weighted	2	1.000	0.743	0.179	-	0.530	No	2	1.000	0.743	0.179	-	0.530
BIN2	SBP	Wald ratio	1	0.254	0.360	0.480	-	0.831	No	1	0.254	0.360	0.480	-	0.831
BLMH	SBP	Inverse variance weighted	4	0.009	0.071	0.901	0.505	0.978	No	4	0.009	0.071	0.901	0.505	0.978
BLNK	SBP	Wald ratio	1	0.624	0.734	0.395	-	0.772	No	1	0.624	0.734	0.395	-	0.772
BMP10	SBP	Inverse variance weighted	2	-0.115	0.087	0.183	-	0.539	No	2	-0.115	0.087	0.183	-	0.539
BMP4	SBP	Wald ratio	1	0.241	1.069	0.821	-	0.966	No	1	0.241	1.069	0.821	-	0.966
BMP6	SBP	Inverse variance weighted	3	1.081	0.263	0.000	0.619	0.001	Yes	3	1.081	0.263	0.000	0.619	0.001
BMPER	SBP	Inverse variance weighted	7	0.157	0.170	0.356	0.466	0.739	No	7	0.157	0.170	0.356	0.466	0.739
BNIP3L	SBP	Wald ratio	1	-3.744	0.802	0.000	-	0.000	Yes	1	-3.744	0.802	0.000	-	0.000
BOC	SBP	Inverse variance weighted	3	0.025	0.577	0.966	0.002	0.993	No	3	0.025	0.577	0.966	0.002	0.993
BOLA1	SBP	Wald ratio	1	0.727	0.378	0.054	-	0.270	No	1	0.727	0.378	0.054	-	0.270
BPIFA2	SBP	Inverse variance weighted	5	0.218	0.216	0.313	0.905	0.696	No	5	0.218	0.216	0.313	0.905	0.696
BPIFB1	SBP	Inverse variance weighted	6	0.049	0.193	0.800	0.000	0.959	No	6	0.049	0.193	0.800	0.000	0.959
BPIFB2	SBP	Inverse variance weighted	3	0.006	0.174	0.971	0.180	0.994	No	3	0.006	0.174	0.971	0.180	0.994
BRAP	SBP	Wald ratio	1	14.453	1.066	0.000	-	0.000	Yes	1	14.453	1.066	0.000	-	0.000
BRSK2	SBP	Inverse variance weighted	4	-0.261	0.074	0.000	0.767	0.008	Yes	4	-0.261	0.074	0.000	0.767	0.008
BSG	SBP	Wald ratio	1	-0.069	0.315	0.827	-	0.966	No	1	-0.069	0.315	0.827	-	0.966
BST1	SBP	Inverse variance weighted	9	0.018	0.051	0.731	0.075	0.943	No	9	0.018	0.051	0.731	0.075	0.943
BST2	SBP	Inverse variance weighted	3	-0.523	0.262	0.046	0.779	0.245	No	3	-0.523	0.262	0.046	0.779	0.245
BTC	SBP	Inverse variance weighted	5	-0.053	0.086	0.536	0.533	0.858	No	5	-0.053	0.086	0.536	0.533	0.858
BTD	SBP	Inverse variance weighted	24	-0.062	0.056	0.275	0.930	0.661	No	24	-0.062	0.056	0.275	0.930	0.661
BTN1A1	SBP	Inverse variance weighted	2	-0.238	0.925	0.797	-	0.958	No	2	-0.238	0.925	0.797	-	0.958
BTN2A1	SBP	Inverse variance weighted	5	0.367	0.145	0.012	0.309	0.100	No	5	0.367	0.145	0.012	0.309	0.100
BTN3A2	SBP	Inverse variance weighted	9	-0.129	0.118	0.274	0.000	0.661	No	9	-0.129	0.118	0.274	0.000	0.661
C19orf12	SBP	Wald ratio	1	-0.378	0.762	0.620	-	0.894	No	1	-0.378	0.762	0.620	-	0.894
C1QA	SBP	Inverse variance weighted	3	0.185	0.111	0.097	0.495	0.369	No	3	0.185	0.111	0.097	0.495	0.369
C1QL2	SBP	Inverse variance weighted	4	-0.162	0.233	0.486	0.068	0.836	No	4	-0.162	0.233	0.486	0.068	0.836
C1QTNF1	SBP	Inverse variance weighted	2	-0.280	0.224	0.211	-	0.583	No	2	-0.280	0.224	0.211	-	0.583
C1QTNF5	SBP	Wald ratio	1	0.676	0.363	0.063	-	0.292	No	1	0.676	0.363	0.063	-	0.292
C1QTNF6	SBP	Wald ratio	1	-0.027	0.594	0.964	-	0.993	No	1	-0.027	0.594	0.964	-	0.993
C1QTNF9	SBP	Inverse variance weighted	11	-0.054	0.052	0.299	0.137	0.688	No	11	-0.054	0.052	0.299	0.137	0.688
C1R	SBP	Inverse variance weighted	2	0.145	0.297	0.624	-	0.897	No	2	0.145	0.297	0.624	-	0.897
C1RL	SBP	Inverse variance weighted	12	0.094	0.102	0.355	0.031	0.739	No	12	0.094	0.102	0.355	0.031	0.739
C1S	SBP	Inverse variance weighted	2	0.130	0.100	0.194	-	0.556	No	2	0.130	0.100	0.194	-	0.556
C2CD2L	SBP	Wald ratio	1	-0.148	0.758	0.846	-	0.967	No	1	-0.148	0.758	0.846	-	0.967
C2orf69	SBP	Wald ratio	1	0.703	0.445	0.114	-	0.403	No	1	0.703	0.445	0.114	-	0.403
C3	SBP	Wald ratio	1	0.164	0.364	0.652	-	0.908	No	1	0.164	0.364	0.652	-	0.908
C4BPB	SBP	Inverse variance weighted	3	0.170	0.290	0.557	0.110	0.865	No	3	0.170	0.290	0.557	0.110	0.865
C5	SBP	Wald ratio	1	0.340	0.278	0.220	-	0.597	No	1	0.340	0.278	0.220	-	0.597
C7	SBP	Inverse variance weighted	8	-0.027	0.065	0.682	0.438	0.922	No	8	-0.027	0.065	0.682	0.438	0.922
C7orf50	SBP	Inverse variance weighted	4	-0.251	0.233	0.280	0.009	0.670	No	4	-0.251	0.233	0.280	0.009	0.670
C8B	SBP	Inverse variance weighted	4	-0.051	0.064	0.429	0.456	0.790	No	4	-0.051	0.064	0.429	0.456	0.790
C9	SBP	Inverse variance weighted	3	0.525	0.446	0.239	0.258	0.620	No	3	0.525	0.446	0.239	0.258	0.620
CA1	SBP	Wald ratio	1	1.034	0.488	0.034	-	0.208	No	1	1.034	0.488	0.034	-	0.208

ST2; MR causal estimates for plasma proteins on systolic blood pressure.

Causal candidates prioritized for SBP were marked as "Yes" in column "Prioritized". Effect of plasma protein levels on blood pressure is in mmHg unit.

										Steiger filtering					
**Exposure**	**Outocme**	**Method**	**nsnp**	**Beta**	**SE**	**P-value**	**Cochran's Q P-value**	**FDR-corrected P-value**	**Prioritized**	**nsnp**	**Beta**	**SE**	**P-value**	**Cochran's Q P-value**	**FDR-corrected P-value**
CA11	SBP	Wald ratio	1	-0.097	0.701	0.890	-	0.975	No	1	-0.097	0.701	0.890	-	0.975
CA12	SBP	Inverse variance weighted	3	0.484	0.150	0.001	0.311	0.018	Yes	3	0.484	0.150	0.001	0.311	0.018
CA13	SBP	Inverse variance weighted	2	0.046	0.209	0.825	-	0.966	No	2	0.046	0.209	0.825	-	0.966
CA14	SBP	Inverse variance weighted	3	0.282	0.321	0.379	0.432	0.760	No	3	0.282	0.321	0.379	0.432	0.760
CA2	SBP	Wald ratio	1	-0.013	0.407	0.975	-	0.995	No	1	-0.013	0.407	0.975	-	0.995
CA3	SBP	Inverse variance weighted	3	-0.256	0.305	0.402	0.209	0.779	No	3	-0.256	0.305	0.402	0.209	0.779
CA4	SBP	Inverse variance weighted	5	-0.223	0.311	0.474	0.000	0.826	No	5	-0.223	0.311	0.474	0.000	0.826
CA5A	SBP	Inverse variance weighted	5	0.017	0.075	0.824	0.183	0.966	No	5	0.017	0.075	0.824	0.183	0.966
CA6	SBP	Inverse variance weighted	7	-0.050	0.063	0.432	0.326	0.793	No	7	-0.050	0.063	0.432	0.326	0.793
CA9	SBP	Inverse variance weighted	2	-0.991	0.308	0.001	-	0.019	Yes	2	-0.991	0.308	0.001	-	0.019
CACNB3	SBP	Wald ratio	1	1.160	0.384	0.003	-	0.032	Yes	1	1.160	0.384	0.003	-	0.032
CACYBP	SBP	Wald ratio	1	0.240	0.350	0.493	-	0.839	No	1	0.240	0.350	0.493	-	0.839
CALB1	SBP	Inverse variance weighted	2	0.451	0.359	0.210	-	0.579	No	2	0.451	0.359	0.210	-	0.579
CALB2	SBP	Wald ratio	1	2.332	0.653	0.000	-	0.007	Yes	1	2.332	0.653	0.000	-	0.007
CALCA	SBP	Wald ratio	1	-1.251	0.386	0.001	-	0.017	Yes	1	-1.251	0.386	0.001	-	0.017
CALCB	SBP	Inverse variance weighted	3	0.303	0.289	0.294	0.532	0.683	No	3	0.303	0.289	0.294	0.532	0.683
CALCOCO1	SBP	Wald ratio	1	0.432	0.415	0.297	-	0.688	No	1	0.432	0.415	0.297	-	0.688
CALCOCO2	SBP	Wald ratio	1	3.575	0.736	0.000	-	0.000	Yes	1	3.575	0.736	0.000	-	0.000
CAMKK1	SBP	Wald ratio	1	0.083	0.152	0.586	-	0.879	No	1	0.083	0.152	0.586	-	0.879
CANT1	SBP	Inverse variance weighted	2	0.403	0.582	0.489	-	0.836	No	2	0.403	0.582	0.489	-	0.836
CAPG	SBP	Inverse variance weighted	7	0.083	0.049	0.091	0.391	0.356	No	7	0.083	0.049	0.091	0.391	0.356
CAPN3	SBP	Wald ratio	1	-0.490	1.070	0.647	-	0.904	No	1	-0.490	1.070	0.647	-	0.904
CAPS	SBP	Inverse variance weighted	4	0.004	0.410	0.993	0.195	0.997	No	4	0.004	0.410	0.993	0.195	0.997
CARHSP1	SBP	Wald ratio	1	0.399	0.487	0.412	-	0.785	No	1	0.399	0.487	0.412	-	0.785
CASP1	SBP	Wald ratio	1	-1.012	0.877	0.249	-	0.629	No	1	-1.012	0.877	0.249	-	0.629
CASP10	SBP	Wald ratio	1	0.051	0.219	0.816	-	0.964	No	1	0.051	0.219	0.816	-	0.964
CASP3	SBP	Wald ratio	1	-0.108	0.341	0.750	-	0.946	No	1	-0.108	0.341	0.750	-	0.946
CASP7	SBP	Inverse variance weighted	2	0.022	1.739	0.990	-	0.997	No	2	0.022	1.739	0.990	-	0.997
CASP8	SBP	Inverse variance weighted	2	-0.093	0.246	0.705	-	0.930	No	2	-0.093	0.246	0.705	-	0.930
CASP9	SBP	Wald ratio	1	1.294	0.488	0.008	-	0.076	No	1	1.294	0.488	0.008	-	0.076
CAT	SBP	Inverse variance weighted	3	-0.152	0.310	0.623	0.137	0.896	No	3	-0.152	0.310	0.623	0.137	0.896
CBLIF	SBP	Wald ratio	1	0.361	0.622	0.561	-	0.868	No	1	0.361	0.622	0.561	-	0.868
CBLN4	SBP	Inverse variance weighted	5	0.016	0.135	0.907	0.523	0.980	No	5	0.016	0.135	0.907	0.523	0.980
CBS	SBP	Inverse variance weighted	2	0.019	0.320	0.952	-	0.988	No	2	0.019	0.320	0.952	-	0.988
CC2D1A	SBP	Inverse variance weighted	2	0.023	0.901	0.980	-	0.996	No	2	0.023	0.901	0.980	-	0.996
CCDC134	SBP	Wald ratio	1	1.123	0.676	0.097	-	0.369	No	1	1.123	0.676	0.097	-	0.369
CCDC50	SBP	Inverse variance weighted	2	-0.074	0.271	0.784	-	0.954	No	2	-0.074	0.271	0.784	-	0.954
CCDC80	SBP	Inverse variance weighted	2	0.271	0.562	0.629	-	0.897	No	2	0.271	0.562	0.629	-	0.897
CCER2	SBP	Wald ratio	1	-1.405	1.014	0.166	-	0.502	No	1	-1.405	1.014	0.166	-	0.502
CCL11	SBP	Wald ratio	1	-0.557	0.425	0.190	-	0.551	No	1	-0.557	0.425	0.190	-	0.551
CCL13	SBP	Inverse variance weighted	4	-0.026	0.160	0.870	0.816	0.971	No	4	-0.026	0.160	0.870	0.816	0.971
CCL14	SBP	Inverse variance weighted	2	0.048	0.085	0.572	-	0.874	No	2	0.048	0.085	0.572	-	0.874
CCL15	SBP	Inverse variance weighted	5	-0.007	0.051	0.895	0.423	0.977	No	5	-0.007	0.051	0.895	0.423	0.977
CCL16	SBP	Inverse variance weighted	5	0.024	0.056	0.673	0.611	0.919	No	5	0.024	0.056	0.673	0.611	0.919
CCL17	SBP	Inverse variance weighted	2	-0.037	0.174	0.831	-	0.966	No	2	-0.037	0.174	0.831	-	0.966
CCL18	SBP	Inverse variance weighted	6	0.056	0.082	0.495	0.799	0.839	No	6	0.056	0.082	0.495	0.799	0.839
CCL19	SBP	Wald ratio	1	0.033	0.568	0.954	-	0.989	No	1	0.033	0.568	0.954	-	0.989
CCL2	SBP	Wald ratio	1	-0.967	0.677	0.153	-	0.483	No	1	-0.967	0.677	0.153	-	0.483
CCL20	SBP	Wald ratio	1	-0.097	0.470	0.836	-	0.966	No	1	-0.097	0.470	0.836	-	0.966
CCL21	SBP	Inverse variance weighted	2	0.145	0.540	0.788	-	0.955	No	2	0.145	0.540	0.788	-	0.955
CCL22	SBP	Inverse variance weighted	6	0.259	0.217	0.234	0.686	0.617	No	6	0.259	0.217	0.234	0.686	0.617
CCL23	SBP	Inverse variance weighted	6	-0.002	0.152	0.992	0.059	0.997	No	6	-0.002	0.152	0.992	0.059	0.997
CCL24	SBP	Inverse variance weighted	10	-0.010	0.058	0.869	0.087	0.971	No	10	-0.010	0.058	0.869	0.087	0.971
CCL25	SBP	Inverse variance weighted	4	-0.011	0.101	0.917	0.033	0.982	No	4	-0.011	0.101	0.917	0.033	0.982
CCL26	SBP	Inverse variance weighted	3	0.191	0.352	0.587	0.825	0.879	No	3	0.191	0.352	0.587	0.825	0.879
CCL27	SBP	Inverse variance weighted	2	0.305	0.283	0.280	-	0.670	No	2	0.305	0.283	0.280	-	0.670

ST2; MR causal estimates for plasma proteins on systolic blood pressure.

Causal candidates prioritized for SBP were marked as "Yes" in column "Prioritized". Effect of plasma protein levels on blood pressure is in mmHg unit.

										Steiger filtering					
**Exposure**	**Outcocme**	**Method**	**nsnp**	**Beta**	**SE**	**P-value**	**Cochran's Q P-value**	**FDR-corrected P-value**	**Prioritized**	**nsnp**	**Beta**	**SE**	**P-value**	**Cochran's Q P-value**	**FDR-corrected P-value**
CCL28	SBP	Inverse variance weighted	2	-0.567	1.198	0.636	-	0.900	No	2	-0.567	1.198	0.636	-	0.900
CCL3	SBP	Inverse variance weighted	4	-0.033	0.313	0.915	0.000	0.982	No	4	-0.033	0.313	0.915	0.000	0.982
CCL4	SBP	Inverse variance weighted	8	-0.103	0.124	0.409	0.786	0.785	No	8	-0.103	0.124	0.409	0.786	0.785
CCL5	SBP	Wald ratio	1	0.024	0.222	0.916	-	0.982	No	1	0.024	0.222	0.916	-	0.982
CCL7	SBP	Inverse variance weighted	2	0.390	0.702	0.578	-	0.877	No	2	0.390	0.702	0.578	-	0.877
CCL8	SBP	Inverse variance weighted	4	-0.058	0.074	0.430	0.091	0.791	No	4	-0.058	0.074	0.430	0.091	0.791
CCN1	SBP	Inverse variance weighted	3	-0.362	0.521	0.487	0.045	0.836	No	3	-0.362	0.521	0.487	0.045	0.836
CCN2	SBP	Inverse variance weighted	2	-0.106	0.223	0.633	-	0.899	No	2	-0.106	0.223	0.633	-	0.899
CCN3	SBP	Inverse variance weighted	4	-1.159	0.216	0.000	0.143	0.000	Yes	4	-1.159	0.216	0.000	0.143	0.000
CCN4	SBP	Inverse variance weighted	9	-0.007	0.074	0.929	0.653	0.983	No	9	-0.007	0.074	0.929	0.653	0.983
CCN5	SBP	Inverse variance weighted	3	-0.334	0.278	0.228	0.931	0.611	No	3	-0.334	0.278	0.228	0.931	0.611
CCND2	SBP	Wald ratio	1	-5.733	0.904	0.000	-	0.000	Yes	1	-5.733	0.904	0.000	-	0.000
CCS	SBP	Wald ratio	1	0.139	0.085	0.101	-	0.376	No	1	0.139	0.085	0.101	-	0.376
CD101	SBP	Inverse variance weighted	23	-0.050	0.050	0.314	0.735	0.696	No	23	-0.050	0.050	0.314	0.735	0.696
CD109	SBP	Inverse variance weighted	7	-0.049	0.073	0.501	0.027	0.839	No	7	-0.049	0.073	0.501	0.027	0.839
CD14	SBP	Wald ratio	1	-0.535	0.150	0.000	-	0.007	Yes	1	-0.535	0.150	0.000	-	0.007
CD160	SBP	Wald ratio	1	0.659	0.681	0.333	-	0.716	No	1	0.659	0.681	0.333	-	0.716
CD163	SBP	Wald ratio	1	-0.022	0.373	0.953	-	0.988	No	1	-0.022	0.373	0.953	-	0.988
CD164	SBP	Inverse variance weighted	3	0.552	0.410	0.178	0.081	0.529	No	3	0.552	0.410	0.178	0.081	0.529
CD164L2	SBP	Wald ratio	1	-0.312	0.091	0.001	-	0.009	Yes	1	-0.312	0.091	0.001	-	0.009
CD177	SBP	Inverse variance weighted	16	0.029	0.070	0.683	0.095	0.922	No	16	0.029	0.070	0.683	0.095	0.922
CD1C	SBP	Inverse variance weighted	2	-0.173	0.377	0.647	-	0.904	No	2	-0.173	0.377	0.647	-	0.904
CD2	SBP	Wald ratio	1	1.025	0.876	0.242	-	0.623	No	1	1.025	0.876	0.242	-	0.623
CD200	SBP	Inverse variance weighted	5	-0.205	0.208	0.324	0.693	0.705	No	5	-0.205	0.208	0.324	0.693	0.705
CD200R1	SBP	Inverse variance weighted	21	-0.017	0.046	0.719	0.872	0.938	No	21	-0.017	0.046	0.719	0.872	0.938
CD207	SBP	Inverse variance weighted	8	0.002	0.098	0.981	0.169	0.996	No	8	0.002	0.098	0.981	0.169	0.996
CD209	SBP	Inverse variance weighted	6	0.222	0.102	0.029	0.642	0.186	No	6	0.222	0.102	0.029	0.642	0.186
CD22	SBP	Inverse variance weighted	2	-0.339	0.162	0.037	-	0.217	No	2	-0.339	0.162	0.037	-	0.217
CD226	SBP	Inverse variance weighted	2	-0.082	0.141	0.561	-	0.868	No	2	-0.082	0.141	0.561	-	0.868
CD244	SBP	Inverse variance weighted	4	-0.008	0.157	0.962	0.160	0.991	No	4	-0.008	0.157	0.962	0.160	0.991
CD248	SBP	Inverse variance weighted	3	0.814	0.508	0.109	0.205	0.392	No	3	0.814	0.508	0.109	0.205	0.392
CD27	SBP	Inverse variance weighted	2	0.024	0.359	0.947	-	0.984	No	2	0.024	0.359	0.947	-	0.984
CD274	SBP	Inverse variance weighted	5	-0.038	0.116	0.747	0.419	0.946	No	5	-0.038	0.116	0.747	0.419	0.946
CD276	SBP	Inverse variance weighted	3	-0.094	0.108	0.383	0.011	0.763	No	3	-0.094	0.108	0.383	0.011	0.763
CD28	SBP	Inverse variance weighted	3	0.165	0.676	0.807	0.000	0.961	No	3	0.165	0.676	0.807	0.000	0.961
CD2AP	SBP	Inverse variance weighted	2	-0.255	0.142	0.072	-	0.312	No	2	-0.255	0.142	0.072	-	0.312
CD300A	SBP	Inverse variance weighted	2	0.308	0.306	0.314	-	0.696	No	2	0.308	0.306	0.314	-	0.696
CD300C	SBP	Inverse variance weighted	4	-0.137	0.188	0.466	0.005	0.817	No	4	-0.137	0.188	0.466	0.005	0.817
CD300E	SBP	Inverse variance weighted	3	-0.076	0.112	0.501	0.291	0.839	No	3	-0.076	0.112	0.501	0.291	0.839
CD300LF	SBP	Inverse variance weighted	11	-0.005	0.055	0.934	0.044	0.984	No	11	-0.005	0.055	0.934	0.044	0.984
CD300LG	SBP	Inverse variance weighted	2	-0.014	0.107	0.894	-	0.977	No	2	-0.014	0.107	0.894	-	0.977
CD302	SBP	Inverse variance weighted	3	-0.199	0.220	0.366	0.101	0.750	No	3	-0.199	0.220	0.366	0.101	0.750
CD33	SBP	Inverse variance weighted	10	0.048	0.048	0.314	0.031	0.696	No	10	0.048	0.048	0.314	0.031	0.696
CD34	SBP	Wald ratio	1	0.647	0.571	0.257	-	0.641	No	1	0.647	0.571	0.257	-	0.641
CD36	SBP	Inverse variance weighted	2	-0.036	0.171	0.832	-	0.966	No	2	-0.036	0.171	0.832	-	0.966
CD38	SBP	Inverse variance weighted	4	0.108	0.129	0.402	0.246	0.779	No	4	0.108	0.129	0.402	0.246	0.779
CD4	SBP	Inverse variance weighted	2	0.060	0.188	0.751	-	0.946	No	2	0.060	0.188	0.751	-	0.946
CD40	SBP	Wald ratio	1	0.098	0.109	0.368	-	0.751	No	1	0.098	0.109	0.368	-	0.751
CD46	SBP	Wald ratio	1	-2.322	0.509	0.000	-	0.000	Yes	1	-2.322	0.509	0.000	-	0.000
CD48	SBP	Inverse variance weighted	7	0.004	0.060	0.946	0.848	0.984	No	7	0.004	0.060	0.946	0.848	0.984
CD5	SBP	Inverse variance weighted	2	-0.126	0.466	0.786	-	0.955	No	2	-0.126	0.466	0.786	-	0.955
CD55	SBP	Inverse variance weighted	3	-0.283	0.252	0.261	0.002	0.644	No	3	-0.283	0.252	0.261	0.002	0.644
CD58	SBP	Inverse variance weighted	2	-0.551	0.234	0.018	-	0.133	No	2	-0.551	0.234	0.018	-	0.133
CD59	SBP	Inverse variance weighted	2	-0.615	0.184	0.001	-	0.013	Yes	2	-0.615	0.184	0.001	-	0.013
CD5L	SBP	Inverse variance weighted	4	-0.152	0.267	0.569	0.115	0.872	No	4	-0.152	0.267	0.569	0.115	0.872
CD6	SBP	Inverse variance weighted	6	-0.048	0.066	0.469	0.138	0.819	No	6	-0.048	0.066	0.469	0.138	0.819

ST2; MR causal estimates for plasma proteins on systolic blood pressure.

Causal candidates prioritized for SBP were marked as "Yes" in column "Prioritized". Effect of plasma protein levels on blood pressure is in mmHg unit.

										Steiger filtering					
Exposure	Outocme	Method	nsnp	Beta	SE	P-value	Cochran's Q P-value	FDR-corrected P-value	Prioritized	nsnp	Beta	SE	P-value	Cochran's Q P-value	FDR-corrected P-value
CD63	SBP	Wald ratio	1	1.263	1.086	0.245	-	0.626	No	1	1.263	1.086	0.245	-	0.626
CD69	SBP	Wald ratio	1	0.476	0.774	0.539	-	0.858	No	1	0.476	0.774	0.539	-	0.858
CD7	SBP	Inverse variance weighted	3	-0.071	0.111	0.520	0.221	0.850	No	3	-0.071	0.111	0.520	0.221	0.850
CD70	SBP	Inverse variance weighted	6	-0.103	0.274	0.707	0.000	0.931	No	6	-0.103	0.274	0.707	0.000	0.931
CD72	SBP	Inverse variance weighted	2	0.118	0.341	0.729	-	0.943	No	2	0.118	0.341	0.729	-	0.943
CD74	SBP	Wald ratio	1	0.053	0.745	0.943	-	0.984	No	1	0.053	0.745	0.943	-	0.984
CD79B	SBP	Wald ratio	1	-0.202	0.253	0.425	-	0.790	No	1	-0.202	0.253	0.425	-	0.790
CD80	SBP	Inverse variance weighted	7	0.004	0.133	0.978	0.086	0.996	No	7	0.004	0.133	0.978	0.086	0.996
CD83	SBP	Inverse variance weighted	2	-0.123	0.238	0.606	-	0.887	No	2	-0.123	0.238	0.606	-	0.887
CD84	SBP	Wald ratio	1	0.422	0.342	0.217	-	0.590	No	1	0.422	0.342	0.217	-	0.590
CD86	SBP	Inverse variance weighted	4	-0.049	0.278	0.861	0.626	0.969	No	4	-0.049	0.278	0.861	0.626	0.969
CD8A	SBP	Inverse variance weighted	2	0.231	0.245	0.345	-	0.728	No	2	0.231	0.245	0.345	-	0.728
CD93	SBP	Wald ratio	1	2.521	1.062	0.018	-	0.130	No	1	2.521	1.062	0.018	-	0.130
CDA	SBP	Inverse variance weighted	5	0.004	0.172	0.980	0.001	0.996	No	5	0.004	0.172	0.980	0.001	0.996
CDC27	SBP	Wald ratio	1	0.679	0.505	0.179	-	0.530	No	1	0.679	0.505	0.179	-	0.530
CDCP1	SBP	Inverse variance weighted	5	0.094	0.179	0.599	0.251	0.883	No	5	0.094	0.179	0.599	0.251	0.883
CDH1	SBP	Wald ratio	1	-1.100	0.867	0.205	-	0.572	No	1	-1.100	0.867	0.205	-	0.572
CDH15	SBP	Inverse variance weighted	7	-0.080	0.118	0.500	0.599	0.839	No	7	-0.080	0.118	0.500	0.599	0.839
CDH17	SBP	Inverse variance weighted	6	-0.270	0.270	0.318	0.135	0.701	No	6	-0.270	0.270	0.318	0.135	0.701
CDH2	SBP	Inverse variance weighted	3	0.720	0.374	0.054	0.641	0.270	No	3	0.720	0.374	0.054	0.641	0.270
CDH23	SBP	Inverse variance weighted	2	0.613	0.293	0.036	-	0.216	No	2	0.613	0.293	0.036	-	0.216
CDH3	SBP	Inverse variance weighted	2	-0.023	0.468	0.960	-	0.991	No	2	-0.023	0.468	0.960	-	0.991
CDH5	SBP	Inverse variance weighted	2	-0.327	0.208	0.116	-	0.407	No	2	-0.327	0.208	0.116	-	0.407
CDH6	SBP	Inverse variance weighted	6	-0.085	0.107	0.425	0.006	0.790	No	6	-0.085	0.107	0.425	0.006	0.790
CDHR1	SBP	Inverse variance weighted	5	0.030	0.146	0.839	0.596	0.967	No	5	0.030	0.146	0.839	0.596	0.967
CDHR5	SBP	Inverse variance weighted	7	0.080	0.121	0.510	0.022	0.844	No	7	0.080	0.121	0.510	0.022	0.844
CDKN1A	SBP	Inverse variance weighted	3	0.815	0.504	0.106	0.020	0.386	No	3	0.815	0.504	0.106	0.020	0.386
CDNF	SBP	Inverse variance weighted	4	0.063	0.111	0.566	0.269	0.870	No	4	0.063	0.111	0.566	0.269	0.870
CDON	SBP	Inverse variance weighted	4	-0.261	0.311	0.401	0.263	0.779	No	4	-0.261	0.311	0.401	0.263	0.779
CEACAM1	SBP	Inverse variance weighted	4	-0.242	0.133	0.068	0.648	0.302	No	4	-0.242	0.133	0.068	0.648	0.302
CEACAM16	SBP	Inverse variance weighted	7	0.211	0.107	0.048	0.304	0.254	No	7	0.211	0.107	0.048	0.304	0.254
CEACAM19	SBP	Inverse variance weighted	3	-0.281	0.192	0.142	0.544	0.458	No	3	-0.281	0.192	0.142	0.544	0.458
CEACAM20	SBP	Inverse variance weighted	3	0.200	0.250	0.425	0.464	0.790	No	3	0.200	0.250	0.425	0.464	0.790
CEACAM21	SBP	Inverse variance weighted	8	0.000	0.039	0.991	0.410	0.997	No	8	0.000	0.039	0.991	0.410	0.997
CEACAM5	SBP	Inverse variance weighted	8	0.082	0.127	0.518	0.083	0.849	No	8	0.082	0.127	0.518	0.083	0.849
CEACAM6	SBP	Wald ratio	1	0.198	0.337	0.557	-	0.865	No	1	0.198	0.337	0.557	-	0.865
CEACAM8	SBP	Inverse variance weighted	3	-0.154	0.417	0.712	0.119	0.933	No	3	-0.154	0.417	0.712	0.119	0.933
CEBPB	SBP	Wald ratio	1	-1.090	0.744	0.143	-	0.460	No	1	-1.090	0.744	0.143	-	0.460
CELA2A	SBP	Inverse variance weighted	2	0.323	0.539	0.549	-	0.862	No	2	0.323	0.539	0.549	-	0.862
CELA3A	SBP	Inverse variance weighted	3	-0.199	0.321	0.535	0.047	0.858	No	3	-0.199	0.321	0.535	0.047	0.858
CELSR2	SBP	Inverse variance weighted	5	-0.135	0.149	0.367	0.063	0.750	No	5	-0.135	0.149	0.367	0.063	0.750
CEMIP2	SBP	Inverse variance weighted	3	0.346	0.251	0.168	0.636	0.507	No	3	0.346	0.251	0.168	0.636	0.507
CEND1	SBP	Wald ratio	1	-2.842	1.101	0.010	-	0.087	No	1	-2.842	1.101	0.010	-	0.087
CENPF	SBP	Wald ratio	1	-0.361	0.676	0.593	-	0.880	No	1	-0.361	0.676	0.593	-	0.880
CEP112	SBP	Inverse variance weighted	2	-0.365	0.165	0.027	-	0.177	No	2	-0.365	0.165	0.027	-	0.177
CEP152	SBP	Wald ratio	1	0.331	0.852	0.698	-	0.925	No	1	0.331	0.852	0.698	-	0.925
CEP170	SBP	Wald ratio	1	2.973	0.644	0.000	-	0.000	Yes	1	2.973	0.644	0.000	-	0.000
CEP20	SBP	Inverse variance weighted	2	-0.284	0.365	0.436	-	0.796	No	2	-0.284	0.365	0.436	-	0.796
CEP43	SBP	Wald ratio	1	0.642	0.537	0.232	-	0.615	No	1	0.642	0.537	0.232	-	0.615
CEP85	SBP	Wald ratio	1	-0.419	0.571	0.462	-	0.817	No	1	-0.419	0.571	0.462	-	0.817
CERT	SBP	Wald ratio	1	-2.507	0.840	0.003	-	0.034	Yes	1	-2.507	0.840	0.003	-	0.034
CES1	SBP	Inverse variance weighted	2	-0.024	0.079	0.755	-	0.950	No	2	-0.024	0.079	0.755	-	0.950
CES2	SBP	Inverse variance weighted	2	-1.435	0.616	0.020	-	0.140	No	2	-1.435	0.616	0.020	-	0.140
CES3	SBP	Inverse variance weighted	7	0.083	0.172	0.630	0.304	0.897	No	7	0.083	0.172	0.630	0.304	0.897
CETN3	SBP	Wald ratio	1	0.877	0.250	0.000	-	0.008	Yes	1	0.877	0.250	0.000	-	0.008
CFD	SBP	Inverse variance weighted	4	-0.095	0.291	0.744	0.142	0.946	No	4	-0.095	0.291	0.744	0.142	0.946

ST2; MR causal estimates for plasma proteins on systolic blood pressure.

Causal candidates prioritized for SBP were marked as "Yes" in column "Prioritized". Effect of plasma protein levels on blood pressure is in mmHg unit.

										Steiger filtering					
**Exposure**	**Outcocme**	**Method**	**nsnp**	**Beta**	**SE**	**P-value**	**Cochran's Q P-value**	**FDR-corrected P-value**	**Prioritized**	**nsnp**	**Beta**	**SE**	**P-value**	**Cochran's Q P-value**	**FDR-corrected P-value**
CFH	SBP	Inverse variance weighted	2	0.002	0.114	0.986	-	0.997	No	2	0.002	0.114	0.986	-	0.997
CFHR2	SBP	Inverse variance weighted	10	-0.212	0.067	0.002	0.012	0.021	Yes	10	-0.212	0.067	0.002	0.012	0.021
CFHR4	SBP	Inverse variance weighted	8	-0.269	0.062	0.000	0.222	0.001	Yes	8	-0.269	0.062	0.000	0.222	0.001
CFHR5	SBP	Inverse variance weighted	3	-0.176	0.164	0.281	0.015	0.670	No	3	-0.176	0.164	0.281	0.015	0.670
CFI	SBP	Inverse variance weighted	3	0.242	0.190	0.204	0.994	0.572	No	3	0.242	0.190	0.204	0.994	0.572
CGA	SBP	Wald ratio	1	0.671	1.087	0.537	-	0.858	No	1	0.671	1.087	0.537	-	0.858
CGREF1	SBP	Inverse variance weighted	8	0.078	0.102	0.445	0.047	0.801	No	8	0.078	0.102	0.445	0.047	0.801
CHAC2	SBP	Wald ratio	1	0.036	0.286	0.901	-	0.978	No	1	0.036	0.286	0.901	-	0.978
CHAD	SBP	Inverse variance weighted	2	-0.230	0.646	0.721	-	0.939	No	2	-0.230	0.646	0.721	-	0.939
CHCHD10	SBP	Inverse variance weighted	2	-1.048	0.534	0.050	-	0.256	No	2	-1.048	0.534	0.050	-	0.256
CHCHD6	SBP	Wald ratio	1	-0.112	0.179	0.532	-	0.858	No	1	-0.112	0.179	0.532	-	0.858
CHGA	SBP	Inverse variance weighted	2	-0.038	0.261	0.884	-	0.974	No	2	-0.038	0.261	0.884	-	0.974
CHGB	SBP	Inverse variance weighted	4	-0.072	0.068	0.286	0.881	0.675	No	4	-0.072	0.068	0.286	0.881	0.675
CHI3L1	SBP	Inverse variance weighted	8	-0.040	0.094	0.670	0.086	0.918	No	8	-0.040	0.094	0.670	0.086	0.918
CHIT1	SBP	Inverse variance weighted	11	-0.047	0.044	0.283	0.807	0.671	No	11	-0.047	0.044	0.283	0.807	0.671
CHL1	SBP	Inverse variance weighted	6	0.136	0.206	0.510	0.005	0.844	No	6	0.136	0.206	0.510	0.005	0.844
CHMP1A	SBP	Inverse variance weighted	2	1.292	0.805	0.109	-	0.392	No	2	1.292	0.805	0.109	-	0.392
CHMP6	SBP	Inverse variance weighted	2	0.083	0.255	0.746	-	0.946	No	2	0.083	0.255	0.746	-	0.946
CHRDL2	SBP	Inverse variance weighted	5	0.274	0.128	0.032	0.507	0.198	No	5	0.274	0.128	0.032	0.507	0.198
CIAPIN1	SBP	Wald ratio	1	-0.422	0.839	0.615	-	0.891	No	1	-0.422	0.839	0.615	-	0.891
CILP	SBP	Wald ratio	1	-0.261	0.143	0.067	-	0.301	No	1	-0.261	0.143	0.067	-	0.301
CIT	SBP	Inverse variance weighted	3	-0.393	0.490	0.423	0.661	0.790	No	3	-0.393	0.490	0.423	0.661	0.790
CKAP4	SBP	Inverse variance weighted	4	0.050	0.252	0.844	0.218	0.967	No	4	0.050	0.252	0.844	0.218	0.967
CLC	SBP	Wald ratio	1	-0.880	1.095	0.422	-	0.790	No	1	-0.880	1.095	0.422	-	0.790
CLEC10A	SBP	Inverse variance weighted	5	-0.015	0.069	0.831	0.390	0.966	No	5	-0.015	0.069	0.831	0.390	0.966
CLEC11A	SBP	Inverse variance weighted	5	-0.180	0.140	0.198	0.396	0.563	No	5	-0.180	0.140	0.198	0.396	0.563
CLEC12A	SBP	Inverse variance weighted	2	0.029	0.129	0.820	-	0.966	No	2	0.029	0.129	0.820	-	0.966
CLEC14A	SBP	Wald ratio	1	-0.012	0.521	0.981	-	0.996	No	1	-0.012	0.521	0.981	-	0.996
CLEC1A	SBP	Inverse variance weighted	5	-0.120	0.147	0.415	0.268	0.788	No	5	-0.120	0.147	0.415	0.268	0.788
CLEC1B	SBP	Inverse variance weighted	2	0.091	0.269	0.734	-	0.943	No	2	0.091	0.269	0.734	-	0.943
CLEC3B	SBP	Wald ratio	1	0.167	0.198	0.399	-	0.777	No	1	0.167	0.198	0.399	-	0.777
CLEC4A	SBP	Inverse variance weighted	6	0.079	0.080	0.320	0.953	0.702	No	6	0.079	0.080	0.320	0.953	0.702
CLEC4C	SBP	Inverse variance weighted	10	0.057	0.056	0.306	0.169	0.693	No	10	0.057	0.056	0.306	0.169	0.693
CLEC4D	SBP	Inverse variance weighted	8	-0.215	0.082	0.009	0.372	0.080	No	8	-0.215	0.082	0.009	0.372	0.080
CLEC4G	SBP	Inverse variance weighted	3	0.262	0.330	0.427	0.045	0.790	No	3	0.262	0.330	0.427	0.045	0.790
CLEC4M	SBP	Inverse variance weighted	4	0.140	0.124	0.261	0.171	0.643	No	4	0.140	0.124	0.261	0.171	0.643
CLEC5A	SBP	Inverse variance weighted	3	0.273	0.329	0.407	0.004	0.784	No	3	0.273	0.329	0.407	0.004	0.784
CLEC6A	SBP	Inverse variance weighted	3	0.083	0.076	0.275	0.369	0.661	No	3	0.083	0.076	0.275	0.369	0.661
CLEC7A	SBP	Inverse variance weighted	12	0.061	0.047	0.192	0.342	0.553	No	12	0.061	0.047	0.192	0.342	0.553
CLGN	SBP	Inverse variance weighted	12	0.128	0.061	0.036	0.287	0.213	No	12	0.128	0.061	0.036	0.287	0.213
CLIC5	SBP	Wald ratio	1	-0.884	0.333	0.008	-	0.075	No	1	-0.884	0.333	0.008	-	0.075
CLIP2	SBP	Wald ratio	1	0.073	0.352	0.836	-	0.966	No	1	0.073	0.352	0.836	-	0.966
CLMP	SBP	Inverse variance weighted	3	0.520	0.120	0.000	0.754	0.001	Yes	3	0.520	0.120	0.000	0.754	0.001
CLPP	SBP	Wald ratio	1	1.374	1.143	0.229	-	0.612	No	1	1.374	1.143	0.229	-	0.612
CLPS	SBP	Inverse variance weighted	5	0.015	0.155	0.922	0.001	0.982	No	5	0.015	0.155	0.922	0.001	0.982
CLSTN2	SBP	Inverse variance weighted	7	-0.128	0.099	0.193	0.371	0.554	No	7	-0.128	0.099	0.193	0.371	0.554
CLSTN3	SBP	Inverse variance weighted	4	-0.023	0.119	0.849	0.110	0.967	No	4	-0.023	0.119	0.849	0.110	0.967
CLU	SBP	Inverse variance weighted	3	-0.302	0.390	0.438	0.318	0.798	No	3	-0.302	0.390	0.438	0.318	0.798
CLUL1	SBP	Inverse variance weighted	7	0.040	0.097	0.676	0.045	0.920	No	7	0.040	0.097	0.676	0.045	0.920
CMC1	SBP	Wald ratio	1	-0.700	0.563	0.214	-	0.585	No	1	-0.700	0.563	0.214	-	0.585
CNDP1	SBP	Inverse variance weighted	4	-0.022	0.167	0.897	0.101	0.977	No	4	-0.022	0.167	0.897	0.101	0.977
CNP	SBP	Wald ratio	1	-0.943	0.492	0.055	-	0.274	No	1	-0.943	0.492	0.055	-	0.274
CNPY4	SBP	Inverse variance weighted	2	0.068	0.552	0.902	-	0.978	No	2	0.068	0.552	0.902	-	0.978
CNTN1	SBP	Inverse variance weighted	5	0.101	0.201	0.617	0.224	0.892	No	5	0.101	0.201	0.617	0.224	0.892
CNTN2	SBP	Inverse variance weighted	11	0.046	0.048	0.340	0.527	0.724	No	11	0.046	0.048	0.340	0.527	0.724
CNTN3	SBP	Inverse variance weighted	7	0.024	0.131	0.854	0.463	0.967	No	7	0.024	0.131	0.854	0.463	0.967

ST2; MR causal estimates for plasma proteins on systolic blood pressure.

Causal candidates prioritized for SBP were marked as "Yes" in column "Prioritized". Effect of plasma protein levels on blood pressure is in mmHg unit.

										Steiger filtering					
**Exposure**	**Outocme**	**Method**	**nsnp**	**Beta**	**SE**	**P-value**	**Cochran's Q P-value**	**FDR-corrected P-value**	**Prioritized**	**nsnp**	**Beta**	**SE**	**P-value**	**Cochran's Q P-value**	**FDR-corrected P-value**
CNTN4	SBP	Inverse variance weighted	13	0.091	0.125	0.465	0.043	0.817	No	13	0.091	0.125	0.465	0.043	0.817
CNTN5	SBP	Inverse variance weighted	11	0.152	0.174	0.383	0.000	0.763	No	11	0.152	0.174	0.383	0.000	0.763
CNT-P2	SBP	Inverse variance weighted	7	0.147	0.082	0.072	0.304	0.313	No	7	0.147	0.082	0.072	0.304	0.313
CNT-P4	SBP	Wald ratio	1	0.483	0.713	0.498	-	0.839	No	1	0.483	0.713	0.498	-	0.839
COCH	SBP	Inverse variance weighted	5	-0.141	0.145	0.332	0.167	0.714	No	5	-0.141	0.145	0.332	0.167	0.714
COL15A1	SBP	Inverse variance weighted	3	-0.373	0.699	0.594	0.000	0.880	No	3	-0.373	0.699	0.594	0.000	0.880
COL18A1	SBP	Inverse variance weighted	3	0.048	0.706	0.946	0.000	0.984	No	3	0.048	0.706	0.946	0.000	0.984
COL1A1	SBP	Wald ratio	1	-2.768	0.889	0.002	-	0.025	Yes	1	-2.768	0.889	0.002	-	0.025
COL24A1	SBP	Inverse variance weighted	3	-0.251	0.229	0.273	0.636	0.660	No	3	-0.251	0.229	0.273	0.636	0.660
COL28A1	SBP	Inverse variance weighted	9	0.122	0.052	0.020	0.719	0.140	No	9	0.122	0.052	0.020	0.719	0.140
COL2A1	SBP	Inverse variance weighted	7	0.124	0.145	0.392	0.000	0.770	No	7	0.124	0.145	0.392	0.000	0.770
COL3A1	SBP	Inverse variance weighted	2	0.162	0.653	0.804	-	0.959	No	2	0.162	0.653	0.804	-	0.959
COL4A1	SBP	Inverse variance weighted	3	0.356	0.212	0.092	0.507	0.359	No	3	0.356	0.212	0.092	0.507	0.359
COL5A1	SBP	Inverse variance weighted	7	0.028	0.184	0.880	0.103	0.974	No	7	0.028	0.184	0.880	0.103	0.974
COL6A3	SBP	Inverse variance weighted	2	1.051	0.407	0.010	-	0.087	No	2	1.051	0.407	0.010	-	0.087
COL9A1	SBP	Inverse variance weighted	3	-0.125	0.223	0.574	0.442	0.875	No	3	-0.125	0.223	0.574	0.442	0.875
COLEC12	SBP	Inverse variance weighted	5	0.118	0.272	0.665	0.130	0.916	No	5	0.118	0.272	0.665	0.130	0.916
COMMD1	SBP	Wald ratio	1	-0.633	0.226	0.005	-	0.054	No	1	-0.633	0.226	0.005	-	0.054
COMP	SBP	Inverse variance weighted	4	0.979	0.189	0.000	0.398	0.000	Yes	4	0.979	0.189	0.000	0.398	0.000
COMT	SBP	Inverse variance weighted	2	-0.560	0.124	0.000	-	0.000	Yes	2	-0.560	0.124	0.000	-	0.000
COQ7	SBP	Wald ratio	1	-0.476	0.316	0.132	-	0.437	No	1	-0.476	0.316	0.132	-	0.437
CPA1	SBP	Inverse variance weighted	2	-0.277	0.928	0.766	-	0.950	No	2	-0.277	0.928	0.766	-	0.950
CPA2	SBP	Inverse variance weighted	4	-0.272	0.209	0.193	0.006	0.554	No	4	-0.272	0.209	0.193	0.006	0.554
CPA4	SBP	Inverse variance weighted	7	0.189	0.109	0.083	0.037	0.337	No	7	0.189	0.109	0.083	0.037	0.337
CPB1	SBP	Wald ratio	1	-0.553	0.368	0.133	-	0.438	No	1	-0.553	0.368	0.133	-	0.438
CPB2	SBP	Inverse variance weighted	6	0.063	0.066	0.342	0.712	0.725	No	6	0.063	0.066	0.342	0.712	0.725
CPE	SBP	Inverse variance weighted	3	-0.033	0.167	0.845	0.394	0.967	No	3	-0.033	0.167	0.845	0.394	0.967
CPM	SBP	Inverse variance weighted	5	0.160	0.398	0.687	0.011	0.922	No	5	0.160	0.398	0.687	0.011	0.922
CPOX	SBP	Inverse variance weighted	3	0.625	0.302	0.038	0.392	0.222	No	3	0.625	0.302	0.038	0.392	0.222
CPPED1	SBP	Inverse variance weighted	11	0.003	0.087	0.972	0.021	0.994	No	11	0.003	0.087	0.972	0.021	0.994
CPQ	SBP	Inverse variance weighted	13	-0.112	0.091	0.219	0.599	0.594	No	13	-0.112	0.091	0.219	0.599	0.594
CPTP	SBP	Wald ratio	1	-5.276	0.958	0.000	-	0.000	Yes	1	-5.276	0.958	0.000	-	0.000
CPVL	SBP	Inverse variance weighted	9	0.027	0.055	0.631	0.972	0.898	No	9	0.027	0.055	0.631	0.972	0.898
CPXM1	SBP	Inverse variance weighted	3	-0.418	0.147	0.004	0.205	0.047	Yes	3	-0.418	0.147	0.004	0.205	0.047
CPXM2	SBP	Inverse variance weighted	5	-0.119	0.088	0.174	0.351	0.520	No	5	-0.119	0.088	0.174	0.351	0.520
CR1	SBP	Inverse variance weighted	6	0.021	0.178	0.907	0.001	0.980	No	6	0.021	0.178	0.907	0.001	0.980
CR2	SBP	Inverse variance weighted	4	0.426	0.456	0.351	0.001	0.733	No	4	0.426	0.456	0.351	0.001	0.733
CRACR2A	SBP	Inverse variance weighted	4	0.000	0.214	0.999	0.992	0.999	No	4	0.000	0.214	0.999	0.992	0.999
CRADD	SBP	Wald ratio	1	-0.008	0.562	0.989	-	0.997	No	1	-0.008	0.562	0.989	-	0.997
CREG1	SBP	Wald ratio	1	0.022	0.224	0.920	-	0.982	No	1	0.022	0.224	0.920	-	0.982
CRELD1	SBP	Inverse variance weighted	8	0.033	0.055	0.545	0.920	0.860	No	8	0.033	0.055	0.545	0.920	0.860
CRELD2	SBP	Inverse variance weighted	8	0.017	0.249	0.947	0.000	0.984	No	8	0.017	0.249	0.947	0.000	0.984
CRH	SBP	Inverse variance weighted	4	-0.077	0.159	0.627	0.348	0.897	No	4	-0.077	0.159	0.627	0.348	0.897
CRHBP	SBP	Inverse variance weighted	3	-0.028	0.072	0.693	0.453	0.924	No	3	-0.028	0.072	0.693	0.453	0.924
CRIM1	SBP	Inverse variance weighted	2	0.149	1.086	0.891	-	0.975	No	2	0.149	1.086	0.891	-	0.975
CRIP2	SBP	Inverse variance weighted	2	-0.062	0.290	0.831	-	0.966	No	2	-0.062	0.290	0.831	-	0.966
CRISP2	SBP	Inverse variance weighted	6	0.029	0.147	0.844	0.001	0.967	No	6	0.029	0.147	0.844	0.001	0.967
CRISP3	SBP	Inverse variance weighted	4	0.192	0.192	0.315	0.000	0.696	No	4	0.192	0.192	0.315	0.000	0.696
CRNN	SBP	Inverse variance weighted	7	-0.030	0.062	0.630	0.820	0.897	No	7	-0.030	0.062	0.630	0.820	0.897
CRTAC1	SBP	Inverse variance weighted	8	0.023	0.073	0.751	0.096	0.946	No	8	0.023	0.073	0.751	0.096	0.946
CRTAM	SBP	Inverse variance weighted	5	-0.259	0.204	0.204	0.032	0.572	No	5	-0.259	0.204	0.204	0.032	0.572
CRYBB1	SBP	Wald ratio	1	0.107	0.260	0.682	-	0.922	No	1	0.107	0.260	0.682	-	0.922
CRYBB2	SBP	Inverse variance weighted	2	-1.154	0.709	0.104	-	0.382	No	2	-1.154	0.709	0.104	-	0.382
CRYGD	SBP	Inverse variance weighted	10	-0.101	0.057	0.075	0.476	0.320	No	10	-0.101	0.057	0.075	0.476	0.320
CRYM	SBP	Inverse variance weighted	3	0.100	0.161	0.535	0.110	0.858	No	3	0.100	0.161	0.535	0.110	0.858
CRYZL1	SBP	Wald ratio	1	-0.480	0.306	0.117	-	0.407	No	1	-0.480	0.306	0.117	-	0.407

ST2; MR causal estimates for plasma proteins on systolic blood pressure.

Causal candidates prioritized for SBP were marked as "Yes" in column "Prioritized". Effect of plasma protein levels on blood pressure is in mmHg unit.

										Steiger filtering					
**Exposure**	**Outocme**	**Method**	**nsnp**	**Beta**	**SE**	**P-value**	**Cochran's Q P-value**	**FDR-corrected P-value**	**Prioritized**	**nsnp**	**Beta**	**SE**	**P-value**	**Cochran's Q P-value**	**FDR-corrected P-value**
CSF1	SBP	Inverse variance weighted	3	0.562	0.210	0.007	0.642	0.071	No	3	0.562	0.210	0.007	0.642	0.071
CSF1R	SBP	Inverse variance weighted	4	-0.064	0.157	0.683	0.370	0.922	No	4	-0.064	0.157	0.683	0.370	0.922
CSF2	SBP	Inverse variance weighted	2	-0.229	0.201	0.253	-	0.635	No	2	-0.229	0.201	0.253	-	0.635
CSF2RB	SBP	Inverse variance weighted	8	0.004	0.038	0.915	0.332	0.982	No	8	0.004	0.038	0.915	0.332	0.982
CSF3	SBP	Wald ratio	1	0.652	0.526	0.215	-	0.588	No	1	0.652	0.526	0.215	-	0.588
CSF3R	SBP	Inverse variance weighted	8	-0.031	0.116	0.790	0.507	0.955	No	8	-0.031	0.116	0.790	0.507	0.955
CSPG4	SBP	Inverse variance weighted	3	-0.676	0.349	0.053	0.593	0.264	No	3	-0.676	0.349	0.053	0.593	0.264
CST1	SBP	Inverse variance weighted	5	0.057	0.111	0.611	0.040	0.890	No	5	0.057	0.111	0.611	0.040	0.890
CST3	SBP	Inverse variance weighted	2	-0.052	0.283	0.855	-	0.968	No	2	-0.052	0.283	0.855	-	0.968
CST5	SBP	Inverse variance weighted	7	-0.127	0.071	0.076	0.110	0.321	No	7	-0.127	0.071	0.076	0.110	0.321
CST6	SBP	Inverse variance weighted	2	0.678	0.709	0.339	-	0.723	No	2	0.678	0.709	0.339	-	0.723
CST7	SBP	Inverse variance weighted	9	0.008	0.050	0.872	0.505	0.972	No	9	0.008	0.050	0.872	0.505	0.972
CSTB	SBP	Inverse variance weighted	5	0.020	0.109	0.853	0.005	0.967	No	5	0.020	0.109	0.853	0.005	0.967
CTBS	SBP	Inverse variance weighted	3	0.045	0.107	0.674	0.129	0.919	No	3	0.045	0.107	0.674	0.129	0.919
CTF1	SBP	Wald ratio	1	-0.726	0.818	0.375	-	0.757	No	1	-0.726	0.818	0.375	-	0.757
CTHRC1	SBP	Wald ratio	1	1.302	0.749	0.082	-	0.336	No	1	1.302	0.749	0.082	-	0.336
CTRB1	SBP	Inverse variance weighted	6	-0.114	0.076	0.132	0.893	0.437	No	6	-0.114	0.076	0.132	0.893	0.437
CTRC	SBP	Inverse variance weighted	5	0.432	0.176	0.014	0.058	0.112	No	5	0.432	0.176	0.014	0.058	0.112
CTRL	SBP	Wald ratio	1	0.097	0.559	0.862	-	0.970	No	1	0.097	0.559	0.862	-	0.970
CTSB	SBP	Inverse variance weighted	3	-0.408	0.251	0.104	0.000	0.382	No	3	-0.408	0.251	0.104	0.000	0.382
CTSC	SBP	Inverse variance weighted	7	0.025	0.064	0.697	0.881	0.925	No	7	0.025	0.064	0.697	0.881	0.925
CTSD	SBP	Inverse variance weighted	5	0.028	0.114	0.809	0.532	0.961	No	5	0.028	0.114	0.809	0.532	0.961
CTSE	SBP	Inverse variance weighted	4	0.118	0.114	0.300	0.089	0.688	No	4	0.118	0.114	0.300	0.089	0.688
CTSF	SBP	Wald ratio	1	0.204	0.214	0.341	-	0.725	No	1	0.204	0.214	0.341	-	0.725
CTSH	SBP	Inverse variance weighted	12	0.075	0.065	0.250	0.027	0.630	No	12	0.075	0.065	0.250	0.027	0.630
CTSO	SBP	Inverse variance weighted	3	-0.570	0.194	0.003	0.747	0.039	Yes	3	-0.570	0.194	0.003	0.747	0.039
CTSS	SBP	Inverse variance weighted	4	0.195	0.187	0.298	0.003	0.688	No	4	0.195	0.187	0.298	0.003	0.688
CTSV	SBP	Inverse variance weighted	3	-0.016	0.185	0.932	0.476	0.984	No	3	-0.016	0.185	0.932	0.476	0.984
CTSZ	SBP	Inverse variance weighted	2	0.313	0.152	0.040	-	0.225	No	2	0.313	0.152	0.040	-	0.225
CWC15	SBP	Wald ratio	1	-0.600	0.676	0.374	-	0.756	No	1	-0.600	0.676	0.374	-	0.756
CX3CL1	SBP	Wald ratio	1	0.219	0.193	0.257	-	0.641	No	1	0.219	0.193	0.257	-	0.641
CXADR	SBP	Inverse variance weighted	2	-0.265	0.335	0.429	-	0.790	No	2	-0.265	0.335	0.429	-	0.790
CXCL1	SBP	Inverse variance weighted	5	-0.025	0.104	0.809	0.058	0.961	No	5	-0.025	0.104	0.809	0.058	0.961
CXCL10	SBP	Inverse variance weighted	3	-0.955	0.687	0.164	0.086	0.501	No	3	-0.955	0.687	0.164	0.086	0.501
CXCL11	SBP	Inverse variance weighted	3	0.234	0.198	0.237	0.174	0.619	No	3	0.234	0.198	0.237	0.174	0.619
CXCL12	SBP	Inverse variance weighted	2	-0.015	0.512	0.977	-	0.996	No	2	-0.015	0.512	0.977	-	0.996
CXCL13	SBP	Wald ratio	1	-0.116	0.800	0.884	-	0.974	No	1	-0.116	0.800	0.884	-	0.974
CXCL14	SBP	Inverse variance weighted	2	0.096	0.520	0.853	-	0.967	No	2	0.096	0.520	0.853	-	0.967
CXCL16	SBP	Inverse variance weighted	4	0.258	0.341	0.449	0.508	0.805	No	4	0.258	0.341	0.449	0.508	0.805
CXCL17	SBP	Inverse variance weighted	2	0.863	0.517	0.095	-	0.367	No	2	0.863	0.517	0.095	-	0.367
CXCL5	SBP	Inverse variance weighted	3	-0.066	0.105	0.531	0.783	0.858	No	3	-0.066	0.105	0.531	0.783	0.858
CXCL6	SBP	Inverse variance weighted	4	0.094	0.073	0.194	0.777	0.555	No	4	0.094	0.073	0.194	0.777	0.555
CXCL8	SBP	Inverse variance weighted	2	-0.023	0.455	0.959	-	0.991	No	2	-0.023	0.455	0.959	-	0.991
CXCL9	SBP	Wald ratio	1	-0.015	0.302	0.960	-	0.991	No	1	-0.015	0.302	0.960	-	0.991
CYB5A	SBP	Wald ratio	1	-0.833	1.136	0.464	-	0.817	No	1	-0.833	1.136	0.464	-	0.817
CYB5R2	SBP	Inverse variance weighted	6	-0.028	0.134	0.834	0.505	0.966	No	6	-0.028	0.134	0.834	0.505	0.966
CYTL1	SBP	Inverse variance weighted	6	0.009	0.098	0.927	0.110	0.983	No	6	0.009	0.098	0.927	0.110	0.983
DAG1	SBP	Wald ratio	1	-4.022	0.672	0.000	-	0.000	Yes	1	-4.022	0.672	0.000	-	0.000
DAPK2	SBP	Inverse variance weighted	2	-0.045	0.104	0.662	-	0.914	No	2	-0.045	0.104	0.662	-	0.914
DAPP1	SBP	Wald ratio	1	-0.047	0.401	0.906	-	0.980	No	1	-0.047	0.401	0.906	-	0.980
DARS1	SBP	Wald ratio	1	-1.221	0.646	0.059	-	0.283	No	1	-1.221	0.646	0.059	-	0.283
DBH	SBP	Inverse variance weighted	12	0.008	0.052	0.878	0.035	0.974	No	12	0.008	0.052	0.878	0.035	0.974
DBI	SBP	Inverse variance weighted	2	0.048	0.150	0.752	-	0.946	No	2	0.048	0.150	0.752	-	0.946
DBN1	SBP	Wald ratio	1	-1.374	0.781	0.078	-	0.327	No	1	-1.374	0.781	0.078	-	0.327
DBNL	SBP	Wald ratio	1	0.770	0.619	0.213	-	0.585	No	1	0.770	0.619	0.213	-	0.585
DCBLD2	SBP	Inverse variance weighted	3	-0.155	0.122	0.203	0.790	0.572	No	3	-0.155	0.122	0.203	0.790	0.572

ST2; MR causal estimates for plasma proteins on systolic blood pressure.

Causal candidates prioritized for SBP were marked as "Yes" in column "Prioritized". Effect of plasma protein levels on blood pressure is in mmHg unit.

										Steiger filtering					
**Exposure**	**Outocme**	**Method**	**nsnp**	**Beta**	**SE**	**P-value**	**Cochran's Q P-value**	**FDR-corrected P-value**	**Prioritized**	**nsnp**	**Beta**	**SE**	**P-value**	**Cochran's Q P-value**	**FDR-corrected P-value**
DCC	SBP	Inverse variance weighted	2	-0.137	0.171	0.424	-	0.790	No	2	-0.137	0.171	0.424	-	0.790
DCN	SBP	Wald ratio	1	-0.909	0.775	0.241	-	0.622	No	1	-0.909	0.775	0.241	-	0.622
DCTD	SBP	Inverse variance weighted	2	-0.516	0.587	0.379	-	0.760	No	2	-0.516	0.587	0.379	-	0.760
DCTPP1	SBP	Inverse variance weighted	2	-0.160	0.359	0.655	-	0.912	No	2	-0.160	0.359	0.655	-	0.912
DCXR	SBP	Wald ratio	1	0.184	0.568	0.747	-	0.946	No	1	0.184	0.568	0.747	-	0.946
DDAH1	SBP	Inverse variance weighted	4	0.470	0.225	0.037	0.708	0.217	No	4	0.470	0.225	0.037	0.708	0.217
DDC	SBP	Inverse variance weighted	3	-0.044	0.107	0.679	0.457	0.922	No	3	-0.044	0.107	0.679	0.457	0.922
DDHD2	SBP	Wald ratio	1	-2.586	0.414	0.000	-	0.000	Yes	1	-2.586	0.414	0.000	-	0.000
DDI2	SBP	Wald ratio	1	2.058	0.872	0.018	-	0.133	No	1	2.058	0.872	0.018	-	0.133
DDT	SBP	Wald ratio	1	-0.369	0.208	0.076	-	0.323	No	1	-0.369	0.208	0.076	-	0.323
DDX58	SBP	Inverse variance weighted	4	0.128	0.314	0.684	0.002	0.922	No	4	0.128	0.314	0.684	0.002	0.922
DECR1	SBP	Inverse variance weighted	2	0.734	0.616	0.233	-	0.617	No	2	0.734	0.616	0.233	-	0.617
DENR	SBP	Wald ratio	1	-0.178	1.198	0.882	-	0.974	No	1	-0.178	1.198	0.882	-	0.974
DGCR6	SBP	Wald ratio	1	-0.120	0.330	0.715	-	0.934	No	1	-0.120	0.330	0.715	-	0.934
DGKA	SBP	Wald ratio	1	-0.449	1.152	0.697	-	0.925	No	1	-0.449	1.152	0.697	-	0.925
DHRS4L2	SBP	Inverse variance weighted	5	-0.130	0.206	0.529	0.040	0.855	No	5	-0.130	0.206	0.529	0.040	0.855
DIABLO	SBP	Wald ratio	1	-1.353	1.160	0.243	-	0.625	No	1	-1.353	1.160	0.243	-	0.625
DKK1	SBP	Inverse variance weighted	3	-0.372	0.310	0.230	0.820	0.613	No	3	-0.372	0.310	0.230	0.820	0.613
DKK3	SBP	Inverse variance weighted	3	-0.196	0.104	0.059	0.483	0.283	No	3	-0.196	0.104	0.059	0.483	0.283
DKK4	SBP	Inverse variance weighted	2	0.297	0.164	0.070	-	0.309	No	2	0.297	0.164	0.070	-	0.309
DKKL1	SBP	Inverse variance weighted	16	-0.028	0.054	0.611	0.002	0.890	No	16	-0.028	0.054	0.611	0.002	0.890
DLK1	SBP	Inverse variance weighted	6	0.242	0.121	0.046	0.077	0.246	No	6	0.242	0.121	0.046	0.077	0.246
DLL1	SBP	Inverse variance weighted	3	-0.693	0.336	0.039	0.132	0.224	No	3	-0.693	0.336	0.039	0.132	0.224
DMP1	SBP	Wald ratio	1	0.144	0.823	0.861	-	0.969	No	1	0.144	0.823	0.861	-	0.969
D-JA4	SBP	Inverse variance weighted	3	0.154	0.283	0.585	0.712	0.879	No	3	0.154	0.283	0.585	0.712	0.879
D-JB1	SBP	Wald ratio	1	0.177	0.797	0.825	-	0.966	No	1	0.177	0.797	0.825	-	0.966
D-JB14	SBP	Wald ratio	1	0.157	0.957	0.870	-	0.971	No	1	0.157	0.957	0.870	-	0.971
D-JB2	SBP	Wald ratio	1	-0.930	1.224	0.447	-	0.803	No	1	-0.930	1.224	0.447	-	0.803
D-JB6	SBP	Wald ratio	1	-0.126	0.211	0.551	-	0.864	No	1	-0.126	0.211	0.551	-	0.864
D-JC9	SBP	Wald ratio	1	4.283	1.315	0.001	-	0.017	Yes	1	4.283	1.315	0.001	-	0.017
DNER	SBP	Inverse variance weighted	5	-0.353	0.166	0.034	0.107	0.207	No	5	-0.353	0.166	0.034	0.107	0.207
DNM1	SBP	Inverse variance weighted	3	-0.100	0.154	0.514	0.613	0.846	No	3	-0.100	0.154	0.514	0.613	0.846
DNMBP	SBP	Inverse variance weighted	2	-0.194	0.252	0.441	-	0.801	No	2	-0.194	0.252	0.441	-	0.801
DNPEP	SBP	Inverse variance weighted	2	-0.165	0.414	0.690	-	0.923	No	2	-0.165	0.414	0.690	-	0.923
DNPH1	SBP	Inverse variance weighted	2	0.192	1.246	0.878	-	0.974	No	2	0.192	1.246	0.878	-	0.974
DOC2B	SBP	Wald ratio	1	1.082	0.670	0.106	-	0.388	No	1	1.082	0.670	0.106	-	0.388
DOK2	SBP	Wald ratio	1	-1.077	0.732	0.142	-	0.458	No	1	-1.077	0.732	0.142	-	0.458
DPEP1	SBP	Inverse variance weighted	8	-0.107	0.085	0.208	0.005	0.578	No	8	-0.107	0.085	0.208	0.005	0.578
DPEP2	SBP	Inverse variance weighted	7	-0.041	0.218	0.849	0.452	0.967	No	7	-0.041	0.218	0.849	0.452	0.967
DPP10	SBP	Inverse variance weighted	3	0.112	0.395	0.776	0.000	0.953	No	3	0.112	0.395	0.776	0.000	0.953
DPP4	SBP	Inverse variance weighted	2	-0.601	0.448	0.180	-	0.532	No	2	-0.601	0.448	0.180	-	0.532
DPP6	SBP	Inverse variance weighted	3	-0.117	0.222	0.598	0.146	0.883	No	3	-0.117	0.222	0.598	0.146	0.883
DPP7	SBP	Wald ratio	1	-0.666	0.635	0.294	-	0.684	No	1	-0.666	0.635	0.294	-	0.684
DPT	SBP	Inverse variance weighted	6	-0.100	0.110	0.364	0.920	0.747	No	6	-0.100	0.110	0.364	0.920	0.747
DPY30	SBP	Wald ratio	1	-1.549	0.704	0.028	-	0.180	No	1	-1.549	0.704	0.028	-	0.180
DRAXIN	SBP	Inverse variance weighted	5	0.127	0.109	0.244	0.600	0.625	No	5	0.127	0.109	0.244	0.600	0.625
DSC2	SBP	Inverse variance weighted	2	-0.037	0.183	0.842	-	0.967	No	2	-0.037	0.183	0.842	-	0.967
DSCAM	SBP	Inverse variance weighted	7	-0.057	0.094	0.541	0.817	0.858	No	7	-0.057	0.094	0.541	0.817	0.858
DSG2	SBP	Inverse variance weighted	4	-0.161	0.263	0.541	0.065	0.858	No	4	-0.161	0.263	0.541	0.065	0.858
DSG3	SBP	Inverse variance weighted	4	0.040	0.164	0.806	0.583	0.961	No	4	0.040	0.164	0.806	0.583	0.961
DSG4	SBP	Wald ratio	1	0.834	0.786	0.289	-	0.675	No	1	0.834	0.786	0.289	-	0.675
DTD1	SBP	Wald ratio	1	-0.713	0.262	0.006	-	0.065	No	1	-0.713	0.262	0.006	-	0.065
DTNB	SBP	Wald ratio	1	0.233	1.183	0.844	-	0.967	No	1	0.233	1.183	0.844	-	0.967
DTX3	SBP	Wald ratio	1	1.594	0.453	0.000	-	0.008	Yes	1	1.594	0.453	0.000	-	0.008
DTYMK	SBP	Inverse variance weighted	2	0.131	0.606	0.829	-	0.966	No	2	0.131	0.606	0.829	-	0.966
DUSP13	SBP	Wald ratio	1	-0.570	0.153	0.000	-	0.004	Yes	1	-0.570	0.153	0.000	-	0.004

ST2; MR causal estimates for plasma proteins on systolic blood pressure.

Causal candidates prioritized for SBP were marked as "Yes" in column "Prioritized". Effect of plasma protein levels on blood pressure is in mmHg unit.

										Steiger filtering					
**Exposure**	**Outocme**	**Method**	**nsnp**	**Beta**	**SE**	**P-value**	**Cochran's Q P-value**	**FDR-corrected P-value**	**Prioritized**	**nsnp**	**Beta**	**SE**	**P-value**	**Cochran's Q P-value**	**FDR-corrected P-value**
DUSP29	SBP	Wald ratio	1	-1.140	0.315	0.000	-	0.006	Yes	1	-1.140	0.315	0.000	-	0.006
DYNLT1	SBP	Wald ratio	1	-0.595	0.500	0.234	-	0.617	No	1	-0.595	0.500	0.234	-	0.617
EBAG9	SBP	Wald ratio	1	1.054	0.620	0.089	-	0.353	No	1	1.054	0.620	0.089	-	0.353
ECE1	SBP	Inverse variance weighted	2	0.258	0.901	0.774	-	0.952	No	2	0.258	0.901	0.774	-	0.952
ECHDC3	SBP	Inverse variance weighted	8	-0.070	0.070	0.319	0.417	0.701	No	8	-0.070	0.070	0.319	0.417	0.701
ECHS1	SBP	Inverse variance weighted	2	0.145	0.189	0.442	-	0.801	No	2	0.145	0.189	0.442	-	0.801
ECI2	SBP	Inverse variance weighted	2	-0.099	0.147	0.498	-	0.839	No	2	-0.099	0.147	0.498	-	0.839
ECM1	SBP	Inverse variance weighted	3	-0.033	0.079	0.680	0.174	0.922	No	3	-0.033	0.079	0.680	0.174	0.922
EDAR	SBP	Inverse variance weighted	7	-0.036	0.165	0.828	0.001	0.966	No	7	-0.036	0.165	0.828	0.001	0.966
EDDM3B	SBP	Wald ratio	1	-0.051	0.259	0.845	-	0.967	No	1	-0.051	0.259	0.845	-	0.967
EDIL3	SBP	Inverse variance weighted	2	-0.850	0.435	0.051	-	0.261	No	2	-0.850	0.435	0.051	-	0.261
EDN1	SBP	Wald ratio	1	0.683	0.182	0.000	-	0.004	Yes	1	0.683	0.182	0.000	-	0.004
EFCAB14	SBP	Inverse variance weighted	2	-1.720	0.791	0.030	-	0.188	No	2	-1.720	0.791	0.030	-	0.188
EFEMP1	SBP	Inverse variance weighted	2	1.260	0.221	0.000	-	0.000	Yes	2	1.260	0.221	0.000	-	0.000
EFHD1	SBP	Inverse variance weighted	5	0.374	0.364	0.304	0.008	0.692	No	5	0.374	0.364	0.304	0.008	0.692
EF-1	SBP	Wald ratio	1	0.276	0.128	0.031	-	0.192	No	1	0.276	0.128	0.031	-	0.192
EF-4	SBP	Wald ratio	1	0.517	0.746	0.489	-	0.836	No	1	0.517	0.746	0.489	-	0.836
EGF	SBP	Wald ratio	1	0.697	0.319	0.029	-	0.185	No	1	0.697	0.319	0.029	-	0.185
EGFL7	SBP	Inverse variance weighted	4	-0.222	0.220	0.313	0.960	0.696	No	4	-0.222	0.220	0.313	0.960	0.696
EGFLAM	SBP	Inverse variance weighted	5	-0.066	0.063	0.298	0.610	0.688	No	5	-0.066	0.063	0.298	0.610	0.688
EGFR	SBP	Inverse variance weighted	4	-0.054	0.237	0.818	0.280	0.965	No	4	-0.054	0.237	0.818	0.280	0.965
EGLN1	SBP	Inverse variance weighted	2	0.044	0.638	0.945	-	0.984	No	2	0.044	0.638	0.945	-	0.984
EHBP1	SBP	Wald ratio	1	-0.345	0.236	0.145	-	0.465	No	1	-0.345	0.236	0.145	-	0.465
EHD3	SBP	Wald ratio	1	-0.268	0.231	0.246	-	0.628	No	1	-0.268	0.231	0.246	-	0.628
EIF2AK2	SBP	Wald ratio	1	-0.692	0.266	0.009	-	0.085	No	1	-0.692	0.266	0.009	-	0.085
EIF2AK3	SBP	Wald ratio	1	0.229	0.347	0.509	-	0.844	No	1	0.229	0.347	0.509	-	0.844
EIF4G3	SBP	Wald ratio	1	1.620	0.526	0.002	-	0.027	Yes	1	1.620	0.526	0.002	-	0.027
EIF5	SBP	Wald ratio	1	-1.028	0.634	0.105	-	0.385	No	1	-1.028	0.634	0.105	-	0.385
ELAC1	SBP	Inverse variance weighted	2	0.162	0.409	0.692	-	0.924	No	2	0.162	0.409	0.692	-	0.924
ELN	SBP	Inverse variance weighted	2	1.369	1.291	0.289	-	0.675	No	2	1.369	1.291	0.289	-	0.675
ELOA	SBP	Wald ratio	1	1.910	0.500	0.000	-	0.003	Yes	1	1.910	0.500	0.000	-	0.003
E-H	SBP	Wald ratio	1	0.827	0.381	0.030	-	0.189	No	1	0.827	0.381	0.030	-	0.189
ENDOU	SBP	Inverse variance weighted	3	0.279	0.129	0.030	0.622	0.191	No	3	0.279	0.129	0.030	0.622	0.191
ENG	SBP	Inverse variance weighted	2	-0.265	0.157	0.092	-	0.359	No	2	-0.265	0.157	0.092	-	0.359
ENO1	SBP	Inverse variance weighted	2	-0.011	0.533	0.983	-	0.996	No	2	-0.011	0.533	0.983	-	0.996
ENO2	SBP	Inverse variance weighted	2	-0.502	0.285	0.078	-	0.326	No	2	-0.502	0.285	0.078	-	0.326
ENO3	SBP	Wald ratio	1	1.105	0.445	0.013	-	0.109	No	1	1.105	0.445	0.013	-	0.109
ENPEP	SBP	Inverse variance weighted	2	-4.206	0.730	0.000	-	0.000	Yes	2	-4.206	0.730	0.000	-	0.000
ENPP2	SBP	Inverse variance weighted	5	0.064	0.278	0.819	0.014	0.966	No	5	0.064	0.278	0.819	0.014	0.966
ENPP5	SBP	Inverse variance weighted	15	-0.041	0.049	0.406	0.455	0.784	No	15	-0.041	0.049	0.406	0.455	0.784
ENPP6	SBP	Inverse variance weighted	15	-0.148	0.118	0.210	0.346	0.579	No	15	-0.148	0.118	0.210	0.346	0.579
ENPP7	SBP	Inverse variance weighted	5	0.097	0.064	0.132	0.063	0.437	No	5	0.097	0.064	0.132	0.063	0.437
ENSA	SBP	Wald ratio	1	-0.339	0.830	0.683	-	0.922	No	1	-0.339	0.830	0.683	-	0.922
ENTPD2	SBP	Wald ratio	1	-0.619	0.226	0.006	-	0.062	No	1	-0.619	0.226	0.006	-	0.062
ENTPD5	SBP	Inverse variance weighted	8	-0.029	0.115	0.804	0.076	0.959	No	8	-0.029	0.115	0.804	0.076	0.959
ENTPD6	SBP	Inverse variance weighted	24	0.148	0.062	0.016	0.144	0.121	No	24	0.148	0.062	0.016	0.144	0.121
ENTR1	SBP	Inverse variance weighted	2	-0.105	0.158	0.505	-	0.842	No	2	-0.105	0.158	0.505	-	0.842
EPCAM	SBP	Wald ratio	1	-0.568	1.024	0.579	-	0.877	No	1	-0.568	1.024	0.579	-	0.877
EPGN	SBP	Wald ratio	1	-1.429	0.892	0.109	-	0.392	No	1	-1.429	0.892	0.109	-	0.392
EPHA1	SBP	Inverse variance weighted	3	-0.169	0.463	0.715	0.003	0.934	No	3	-0.169	0.463	0.715	0.003	0.934
EPHA2	SBP	Inverse variance weighted	2	-0.929	0.471	0.048	-	0.254	No	2	-0.929	0.471	0.048	-	0.254
EPHA4	SBP	Inverse variance weighted	4	0.158	0.232	0.496	0.470	0.839	No	4	0.158	0.232	0.496	0.470	0.839
EPHB6	SBP	Inverse variance weighted	2	0.134	0.144	0.350	-	0.733	No	2	0.134	0.144	0.350	-	0.733
EPHX2	SBP	Wald ratio	1	-0.103	0.405	0.800	-	0.959	No	1	-0.103	0.405	0.800	-	0.959
EPO	SBP	Wald ratio	1	0.861	0.536	0.108	-	0.392	No	1	0.861	0.536	0.108	-	0.392
EPPK1	SBP	Inverse variance weighted	13	0.016	0.099	0.871	0.254	0.971	No	13	0.016	0.099	0.871	0.254	0.971

ST2; MR causal estimates for plasma proteins on systolic blood pressure.

Causal candidates prioritized for SBP were marked as "Yes" in column "Prioritized". Effect of plasma protein levels on blood pressure is in mmHg unit.

										**Steiger filtering**					
**Exposure**	**Outocme**	**Method**	**nsnp**	**Beta**	**SE**	**P-value**	**Cochran's Q P-value**	**FDR-corrected P-value**	**Prioritized**	**nsnp**	**Beta**	**SE**	**P-value**	**Cochran's Q P-value**	**FDR-corrected P-value**
EPS8L2	SBP	Inverse variance weighted	2	-0.082	0.135	0.543	-	0.858	No	2	-0.082	0.135	0.543	-	0.858
ERBB2	SBP	Inverse variance weighted	7	0.069	0.235	0.768	0.119	0.950	No	7	0.069	0.235	0.768	0.119	0.950
ERBB3	SBP	Inverse variance weighted	4	0.107	0.284	0.706	0.041	0.931	No	4	0.107	0.284	0.706	0.041	0.931
ERBB4	SBP	Inverse variance weighted	6	-0.246	0.310	0.428	0.002	0.790	No	6	-0.246	0.310	0.428	0.002	0.790
ERC2	SBP	Wald ratio	1	0.113	0.743	0.879	-	0.974	No	1	0.113	0.743	0.879	-	0.974
EREG	SBP	Wald ratio	1	-0.677	0.435	0.119	-	0.413	No	1	-0.677	0.435	0.119	-	0.413
ERI1	SBP	Wald ratio	1	4.186	0.565	0.000	-	0.000	Yes	1	4.186	0.565	0.000	-	0.000
ERMAP	SBP	Inverse variance weighted	2	-0.085	0.574	0.882	-	0.974	No	2	-0.085	0.574	0.882	-	0.974
ERN1	SBP	Wald ratio	1	0.889	0.361	0.014	-	0.112	No	1	0.889	0.361	0.014	-	0.112
ERP29	SBP	Wald ratio	1	15.499	1.144	0.000	-	0.000	Yes	1	15.499	1.144	0.000	-	0.000
ERP44	SBP	Wald ratio	1	1.008	0.906	0.266	-	0.650	No	1	1.008	0.906	0.266	-	0.650
ESAM	SBP	Wald ratio	1	-1.139	0.327	0.000	-	0.008	Yes	1	-1.139	0.327	0.000	-	0.008
ESM1	SBP	Inverse variance weighted	2	0.451	0.191	0.018	-	0.133	No	2	0.451	0.191	0.018	-	0.133
ESYT2	SBP	Inverse variance weighted	3	-0.267	0.268	0.320	0.711	0.702	No	3	-0.267	0.268	0.320	0.711	0.702
EVI5	SBP	Wald ratio	1	-0.534	0.308	0.083	-	0.337	No	1	-0.534	0.308	0.083	-	0.337
EXTL1	SBP	Wald ratio	1	0.063	0.106	0.553	-	0.865	No	1	0.063	0.106	0.553	-	0.865
EZR	SBP	Inverse variance weighted	2	0.513	0.387	0.185	-	0.539	No	2	0.513	0.387	0.185	-	0.539
F10	SBP	Inverse variance weighted	2	0.250	0.196	0.203	-	0.572	No	2	0.250	0.196	0.203	-	0.572
F11	SBP	Inverse variance weighted	4	-0.077	0.123	0.534	0.200	0.858	No	4	-0.077	0.123	0.534	0.200	0.858
F11R	SBP	Wald ratio	1	1.412	0.720	0.050	-	0.257	No	1	1.412	0.720	0.050	-	0.257
F12	SBP	Inverse variance weighted	6	0.044	0.050	0.384	0.132	0.763	No	6	0.044	0.050	0.384	0.132	0.763
F13B	SBP	Inverse variance weighted	6	-0.508	0.117	0.000	0.186	0.001	Yes	6	-0.508	0.117	0.000	0.186	0.001
F2	SBP	Inverse variance weighted	4	-0.128	1.121	0.909	0.000	0.981	No	4	-0.128	1.121	0.909	0.000	0.981
F2R	SBP	Inverse variance weighted	2	-0.063	0.356	0.860	-	0.969	No	2	-0.063	0.356	0.860	-	0.969
F3	SBP	Inverse variance weighted	5	-0.356	0.188	0.058	0.751	0.283	No	5	-0.356	0.188	0.058	0.751	0.283
F7	SBP	Inverse variance weighted	5	0.212	0.159	0.183	0.003	0.539	No	5	0.212	0.159	0.183	0.003	0.539
FABP1	SBP	Wald ratio	1	-0.207	0.186	0.266	-	0.650	No	1	-0.207	0.186	0.266	-	0.650
FABP2	SBP	Inverse variance weighted	2	-0.258	0.195	0.185	-	0.539	No	2	-0.258	0.195	0.185	-	0.539
FABP3	SBP	Wald ratio	1	-0.031	0.502	0.951	-	0.987	No	1	-0.031	0.502	0.951	-	0.987
FABP4	SBP	Wald ratio	1	0.287	0.468	0.540	-	0.858	No	1	0.287	0.468	0.540	-	0.858
FABP5	SBP	Wald ratio	1	0.411	0.364	0.259	-	0.642	No	1	0.411	0.364	0.259	-	0.642
FABP6	SBP	Wald ratio	1	-0.134	0.304	0.660	-	0.914	No	1	-0.134	0.304	0.660	-	0.914
FABP9	SBP	Inverse variance weighted	2	0.006	1.033	0.996	-	0.998	No	2	0.006	1.033	0.996	-	0.998
FADD	SBP	Wald ratio	1	-0.906	0.504	0.072	-	0.313	No	1	-0.906	0.504	0.072	-	0.313
FAM13A	SBP	Inverse variance weighted	3	-0.176	0.454	0.698	0.024	0.925	No	3	-0.176	0.454	0.698	0.024	0.925
FAM171B	SBP	Wald ratio	1	0.241	0.117	0.040	-	0.225	No	1	0.241	0.117	0.040	-	0.225
FAM172A	SBP	Inverse variance weighted	2	0.598	0.335	0.074	-	0.318	No	2	0.598	0.335	0.074	-	0.318
FAM20A	SBP	Inverse variance weighted	5	-0.077	0.248	0.757	0.042	0.950	No	5	-0.077	0.248	0.757	0.042	0.950
FAM3B	SBP	Inverse variance weighted	7	0.205	0.073	0.005	0.903	0.051	No	7	0.205	0.073	0.005	0.903	0.051
FAM3C	SBP	Wald ratio	1	-0.796	0.427	0.063	-	0.292	No	1	-0.796	0.427	0.063	-	0.292
FAM3D	SBP	Inverse variance weighted	3	-0.150	0.190	0.429	0.120	0.790	No	3	-0.150	0.190	0.429	0.120	0.790
FAP	SBP	Inverse variance weighted	3	0.112	0.169	0.507	0.823	0.842	No	3	0.112	0.169	0.507	0.823	0.842
FARSA	SBP	Wald ratio	1	-0.693	0.862	0.421	-	0.790	No	1	-0.693	0.862	0.421	-	0.790
FAS	SBP	Inverse variance weighted	3	-0.385	0.163	0.018	0.334	0.133	No	3	-0.385	0.163	0.018	0.334	0.133
FASLG	SBP	Wald ratio	1	-0.009	0.529	0.987	-	0.997	No	1	-0.009	0.529	0.987	-	0.997
FBLN2	SBP	Inverse variance weighted	5	-0.069	0.257	0.787	0.028	0.955	No	5	-0.069	0.257	0.787	0.028	0.955
FBN2	SBP	Inverse variance weighted	2	0.217	0.155	0.162	-	0.499	No	2	0.217	0.155	0.162	-	0.499
FBP1	SBP	Inverse variance weighted	3	0.188	0.129	0.147	0.463	0.468	No	3	0.188	0.129	0.147	0.463	0.468
FCAMR	SBP	Inverse variance weighted	3	0.032	0.117	0.788	0.727	0.955	No	3	0.032	0.117	0.788	0.727	0.955
FCAR	SBP	Inverse variance weighted	8	-0.007	0.056	0.896	0.341	0.977	No	8	-0.007	0.056	0.896	0.341	0.977
FCER1A	SBP	Inverse variance weighted	2	-0.818	0.587	0.163	-	0.500	No	2	-0.818	0.587	0.163	-	0.500
FCER2	SBP	Inverse variance weighted	3	0.205	0.139	0.140	0.250	0.455	No	3	0.205	0.139	0.140	0.250	0.455
FCGR2A	SBP	Inverse variance weighted	16	0.010	0.041	0.806	0.824	0.961	No	16	0.010	0.041	0.806	0.824	0.961
FCGR2B	SBP	Inverse variance weighted	4	-0.141	0.085	0.097	0.876	0.369	No	4	-0.141	0.085	0.097	0.876	0.369
FCGR3B	SBP	Inverse variance weighted	9	-0.016	0.047	0.738	0.565	0.945	No	9	-0.016	0.047	0.738	0.565	0.945
FCN1	SBP	Inverse variance weighted	4	0.260	0.205	0.205	0.003	0.572	No	4	0.260	0.205	0.205	0.003	0.572

ST2; MR causal estimates for plasma proteins on systolic blood pressure.

Causal candidates prioritized for SBP were marked as "Yes" in column "Prioritized". Effect of plasma protein levels on blood pressure is in mmHg unit.

										Steiger filtering					
**Exposure**	**Outcocme**	**Method**	**nsnp**	**Beta**	**SE**	**P-value**	**Cochran's Q P-value**	**FDR-corrected P-value**	**Prioritized**	**nsnp**	**Beta**	**SE**	**P-value**	**Cochran's Q P-value**	**FDR-corrected P-value**
FCN2	SBP	Inverse variance weighted	9	0.031	0.063	0.620	0.362	0.894	No	9	0.031	0.063	0.620	0.362	0.894
FCRL1	SBP	Inverse variance weighted	3	0.195	0.282	0.489	0.002	0.836	No	3	0.195	0.282	0.489	0.002	0.836
FCRL2	SBP	Inverse variance weighted	5	0.074	0.065	0.256	0.465	0.639	No	5	0.074	0.065	0.256	0.465	0.639
FCRL3	SBP	Inverse variance weighted	9	0.023	0.041	0.575	0.693	0.875	No	9	0.023	0.041	0.575	0.693	0.875
FCRL5	SBP	Inverse variance weighted	8	-0.057	0.094	0.542	0.588	0.858	No	8	-0.057	0.094	0.542	0.588	0.858
FCRL6	SBP	Inverse variance weighted	6	-0.026	0.068	0.703	0.216	0.928	No	6	-0.026	0.068	0.703	0.216	0.928
FCRLB	SBP	Inverse variance weighted	11	-0.014	0.054	0.793	0.691	0.956	No	11	-0.014	0.054	0.793	0.691	0.956
FDX1	SBP	Wald ratio	1	2.550	0.829	0.002	-	0.027	Yes	1	2.550	0.829	0.002	-	0.027
FES	SBP	Wald ratio	1	-4.557	0.305	0.000	-	0.000	Yes	1	-4.557	0.305	0.000	-	0.000
FETUB	SBP	Inverse variance weighted	11	0.202	0.085	0.018	0.886	0.131	No	11	0.202	0.085	0.018	0.886	0.131
FGA	SBP	Inverse variance weighted	3	-0.305	0.340	0.369	0.272	0.751	No	3	-0.305	0.340	0.369	0.272	0.751
FGF2	SBP	Inverse variance weighted	3	0.174	0.059	0.003	0.558	0.039	Yes	3	0.174	0.059	0.003	0.558	0.039
FGF21	SBP	Inverse variance weighted	2	0.275	0.598	0.645	-	0.904	No	2	0.275	0.598	0.645	-	0.904
FGF23	SBP	Inverse variance weighted	3	-0.063	0.716	0.930	0.024	0.983	No	3	-0.063	0.716	0.930	0.024	0.983
FGF5	SBP	Inverse variance weighted	8	1.360	0.105	0.000	0.009	0.000	Yes	8	1.360	0.105	0.000	0.009	0.000
FGFBP1	SBP	Inverse variance weighted	3	0.143	0.264	0.589	0.385	0.879	No	3	0.143	0.264	0.589	0.385	0.879
FGFBP2	SBP	Inverse variance weighted	5	0.074	0.106	0.487	0.098	0.836	No	5	0.074	0.106	0.487	0.098	0.836
FGFBP3	SBP	Inverse variance weighted	2	-0.108	0.152	0.477	-	0.829	No	2	-0.108	0.152	0.477	-	0.829
FGFR2	SBP	Inverse variance weighted	4	-0.273	0.731	0.709	0.000	0.931	No	4	-0.273	0.731	0.709	0.000	0.931
FGFR4	SBP	Inverse variance weighted	11	-0.017	0.057	0.762	0.057	0.950	No	11	-0.017	0.057	0.762	0.057	0.950
FGL1	SBP	Inverse variance weighted	7	0.026	0.072	0.720	0.239	0.938	No	7	0.026	0.072	0.720	0.239	0.938
FGR	SBP	Inverse variance weighted	3	1.401	0.624	0.025	0.059	0.164	No	3	1.401	0.624	0.025	0.059	0.164
FHIT	SBP	Inverse variance weighted	2	-0.008	0.751	0.991	-	0.997	No	2	-0.008	0.751	0.991	-	0.997
FIS1	SBP	Wald ratio	1	-0.542	0.248	0.029	-	0.184	No	1	-0.542	0.248	0.029	-	0.184
FKBP1B	SBP	Inverse variance weighted	2	0.109	0.652	0.867	-	0.971	No	2	0.109	0.652	0.867	-	0.971
FKBP4	SBP	Wald ratio	1	-1.394	0.755	0.065	-	0.297	No	1	-1.394	0.755	0.065	-	0.297
FKBP5	SBP	Wald ratio	1	-0.063	0.207	0.762	-	0.950	No	1	-0.063	0.207	0.762	-	0.950
FKBP7	SBP	Wald ratio	1	-1.835	0.602	0.002	-	0.029	Yes	1	-1.835	0.602	0.002	-	0.029
FLRT2	SBP	Inverse variance weighted	6	-0.129	0.095	0.174	0.180	0.519	No	6	-0.129	0.095	0.174	0.180	0.519
FLT1	SBP	Wald ratio	1	0.104	0.900	0.908	-	0.980	No	1	0.104	0.900	0.908	-	0.980
FLT3	SBP	Inverse variance weighted	2	-0.718	0.413	0.082	-	0.336	No	2	-0.718	0.413	0.082	-	0.336
FLT3LG	SBP	Inverse variance weighted	2	-0.707	0.756	0.350	-	0.733	No	2	-0.707	0.756	0.350	-	0.733
FLT4	SBP	Inverse variance weighted	3	-0.243	0.226	0.284	0.072	0.672	No	3	-0.243	0.226	0.284	0.072	0.672
FN1	SBP	Inverse variance weighted	2	-0.761	0.098	0.000	-	0.000	Yes	2	-0.761	0.098	0.000	-	0.000
FNDC1	SBP	Inverse variance weighted	7	-0.154	0.138	0.264	0.049	0.649	No	7	-0.154	0.138	0.264	0.049	0.649
FNTA	SBP	Wald ratio	1	2.326	0.829	0.005	-	0.053	No	1	2.326	0.829	0.005	-	0.053
FOLH1	SBP	Wald ratio	1	-0.120	0.458	0.793	-	0.956	No	1	-0.120	0.458	0.793	-	0.956
FOLR1	SBP	Inverse variance weighted	3	0.273	0.395	0.489	0.293	0.836	No	3	0.273	0.395	0.489	0.293	0.836
FOLR2	SBP	Inverse variance weighted	2	0.553	0.339	0.103	-	0.379	No	2	0.553	0.339	0.103	-	0.379
FOLR3	SBP	Inverse variance weighted	11	-0.033	0.071	0.646	0.027	0.904	No	11	-0.033	0.071	0.646	0.027	0.904
FOXJ3	SBP	Wald ratio	1	3.036	0.594	0.000	-	0.000	Yes	1	3.036	0.594	0.000	-	0.000
FOXO1	SBP	Wald ratio	1	0.635	0.356	0.075	-	0.318	No	1	0.635	0.356	0.075	-	0.318
FOXO3	SBP	Wald ratio	1	4.423	1.120	0.000	-	0.002	Yes	1	4.423	1.120	0.000	-	0.002
FRZB	SBP	Inverse variance weighted	6	0.118	0.120	0.327	0.063	0.709	No	6	0.118	0.120	0.327	0.063	0.709
FSHB	SBP	Wald ratio	1	0.721	0.384	0.060	-	0.288	No	1	0.721	0.384	0.060	-	0.288
FST	SBP	Inverse variance weighted	2	-0.310	0.652	0.634	-	0.900	No	2	-0.310	0.652	0.634	-	0.900
FSTL1	SBP	Inverse variance weighted	2	0.019	0.232	0.934	-	0.984	No	2	0.019	0.232	0.934	-	0.984
FSTL3	SBP	Inverse variance weighted	2	-1.109	1.298	0.393	-	0.770	No	2	-1.109	1.298	0.393	-	0.770
FTCD	SBP	Inverse variance weighted	3	0.169	0.944	0.858	0.017	0.969	No	3	0.169	0.944	0.858	0.017	0.969
FUCA1	SBP	Inverse variance weighted	3	0.410	0.119	0.001	0.304	0.009	Yes	3	0.410	0.119	0.001	0.304	0.009
FUOM	SBP	Inverse variance weighted	5	-0.309	0.198	0.120	0.519	0.414	No	5	-0.309	0.198	0.120	0.519	0.414
FURIN	SBP	Wald ratio	1	3.229	0.213	0.000	-	0.000	Yes	1	3.229	0.213	0.000	-	0.000
FUT1	SBP	Wald ratio	1	0.911	0.946	0.336	-	0.719	No	1	0.911	0.946	0.336	-	0.719
FUT8	SBP	Inverse variance weighted	9	0.026	0.051	0.618	0.839	0.892	No	9	0.026	0.051	0.618	0.839	0.892
FXN	SBP	Wald ratio	1	1.294	0.529	0.014	-	0.114	No	1	1.294	0.529	0.014	-	0.114
FXYD5	SBP	Wald ratio	1	0.002	0.125	0.985	-	0.997	No	1	0.002	0.125	0.985	-	0.997

ST2; MR causal estimates for plasma proteins on systolic blood pressure.

Causal candidates prioritized for SBP were marked as "Yes" in column "Prioritized". Effect of plasma protein levels on blood pressure is in mmHg unit.

										Steiger filtering					
Exposure	Outocme	Method	nsnp	Beta	SE	P-value	Cochran's Q P-value	FDR-corrected P-value	Prioritized	nsnp	Beta	SE	P-value	Cochran's Q P-value	FDR-corrected P-value
GAL	SBP	Inverse variance weighted	4	0.404	0.954	0.672	0.000	0.918	No	4	0.404	0.954	0.672	0.000	0.918
GALNT10	SBP	Inverse variance weighted	3	0.116	0.103	0.260	0.491	0.642	No	3	0.116	0.103	0.260	0.491	0.642
GALNT2	SBP	Inverse variance weighted	4	0.231	0.141	0.101	0.267	0.376	No	4	0.231	0.141	0.101	0.267	0.376
GALNT3	SBP	Inverse variance weighted	4	0.067	0.109	0.538	0.408	0.858	No	4	0.067	0.109	0.538	0.408	0.858
GALNT5	SBP	Inverse variance weighted	6	0.184	0.115	0.110	0.921	0.392	No	6	0.184	0.115	0.110	0.921	0.392
GALNT7	SBP	Wald ratio	1	-0.442	0.646	0.494	-	0.839	No	1	-0.442	0.646	0.494	-	0.839
GAMT	SBP	Wald ratio	1	1.723	1.207	0.154	-	0.484	No	1	1.723	1.207	0.154	-	0.484
GART	SBP	Wald ratio	1	-0.035	0.367	0.925	-	0.983	No	1	-0.035	0.367	0.925	-	0.983
GAS2	SBP	Wald ratio	1	-0.142	0.367	0.699	-	0.925	No	1	-0.142	0.367	0.699	-	0.925
GAS6	SBP	Inverse variance weighted	2	-0.357	0.188	0.058	-	0.282	No	2	-0.357	0.188	0.058	-	0.282
GASK1A	SBP	Inverse variance weighted	9	0.059	0.054	0.268	0.814	0.653	No	9	0.059	0.054	0.268	0.814	0.653
GAST	SBP	Wald ratio	1	0.533	1.139	0.640	-	0.901	No	1	0.533	1.139	0.640	-	0.901
GATD3	SBP	Wald ratio	1	-0.090	0.380	0.813	-	0.963	No	1	-0.090	0.380	0.813	-	0.963
GBP1	SBP	Inverse variance weighted	6	0.281	0.135	0.038	0.046	0.221	No	6	0.281	0.135	0.038	0.046	0.221
GBP2	SBP	Inverse variance weighted	2	1.215	0.496	0.014	-	0.113	No	2	1.215	0.496	0.014	-	0.113
GBP4	SBP	Inverse variance weighted	7	-1.265	0.521	0.015	0.075	0.117	No	7	-1.265	0.521	0.015	0.075	0.117
GC	SBP	Inverse variance weighted	10	0.012	0.043	0.781	0.160	0.953	No	10	0.012	0.043	0.781	0.160	0.953
GCHFR	SBP	Wald ratio	1	1.379	0.359	0.000	-	0.003	Yes	1	1.379	0.359	0.000	-	0.003
GCLM	SBP	Wald ratio	1	1.201	0.744	0.106	-	0.388	No	1	1.201	0.744	0.106	-	0.388
GCNT1	SBP	Inverse variance weighted	8	0.036	0.129	0.777	0.018	0.953	No	8	0.036	0.129	0.777	0.018	0.953
GDF15	SBP	Inverse variance weighted	6	0.123	0.143	0.390	0.675	0.770	No	6	0.123	0.143	0.390	0.675	0.770
GDNF	SBP	Inverse variance weighted	4	-0.208	0.214	0.330	0.117	0.712	No	4	-0.208	0.214	0.330	0.117	0.712
GFAP	SBP	Inverse variance weighted	3	0.834	0.664	0.209	0.045	0.579	No	3	0.834	0.664	0.209	0.045	0.579
GFER	SBP	Wald ratio	1	-2.517	0.694	0.000	-	0.006	Yes	1	-2.517	0.694	0.000	-	0.006
GFRA1	SBP	Inverse variance weighted	5	0.268	0.312	0.391	0.007	0.770	No	5	0.268	0.312	0.391	0.007	0.770
GFRA2	SBP	Inverse variance weighted	7	-0.096	0.137	0.481	0.189	0.831	No	7	-0.096	0.137	0.481	0.189	0.831
GFRA3	SBP	Inverse variance weighted	2	-0.268	0.536	0.617	-	0.892	No	2	-0.268	0.536	0.617	-	0.892
GFRAL	SBP	Inverse variance weighted	7	-0.035	0.119	0.769	0.149	0.950	No	7	-0.035	0.119	0.769	0.149	0.950
GGACT	SBP	Inverse variance weighted	2	-0.089	0.088	0.312	-	0.696	No	2	-0.089	0.088	0.312	-	0.696
GGCT	SBP	Wald ratio	1	0.013	0.377	0.972	-	0.994	No	1	0.013	0.377	0.972	-	0.994
GGH	SBP	Inverse variance weighted	21	0.071	0.103	0.492	0.123	0.838	No	21	0.071	0.103	0.492	0.123	0.838
GGT1	SBP	Inverse variance weighted	6	-0.197	0.244	0.420	0.021	0.790	No	6	-0.197	0.244	0.420	0.021	0.790
GGT5	SBP	Inverse variance weighted	5	-0.071	0.131	0.588	0.861	0.879	No	5	-0.071	0.131	0.588	0.861	0.879
GHR	SBP	Inverse variance weighted	10	0.193	0.068	0.004	0.326	0.048	Yes	10	0.193	0.068	0.004	0.326	0.048
GHRL	SBP	Inverse variance weighted	2	0.090	0.271	0.741	-	0.945	No	2	0.090	0.271	0.741	-	0.945
GIMAP7	SBP	Inverse variance weighted	4	0.396	0.077	0.000	0.638	0.000	Yes	4	0.396	0.077	0.000	0.638	0.000
GIMAP8	SBP	Inverse variance weighted	2	-0.121	0.215	0.574	-	0.875	No	2	-0.121	0.215	0.574	-	0.875
GIP	SBP	Wald ratio	1	-1.304	0.985	0.185	-	0.539	No	1	-1.304	0.985	0.185	-	0.539
GIPC2	SBP	Inverse variance weighted	2	-0.283	0.161	0.078	-	0.327	No	2	-0.283	0.161	0.078	-	0.327
GIPC3	SBP	Inverse variance weighted	2	-0.183	0.147	0.213	-	0.585	No	2	-0.183	0.147	0.213	-	0.585
GIT1	SBP	Inverse variance weighted	2	1.707	0.531	0.001	-	0.019	Yes	2	1.707	0.531	0.001	-	0.019
GKN1	SBP	Wald ratio	1	0.306	1.043	0.769	-	0.950	No	1	0.306	1.043	0.769	-	0.950
GLB1	SBP	Wald ratio	1	-0.076	0.184	0.681	-	0.922	No	1	-0.076	0.184	0.681	-	0.922
GLO1	SBP	Inverse variance weighted	2	0.121	0.120	0.313	-	0.696	No	2	0.121	0.120	0.313	-	0.696
GLRX	SBP	Inverse variance weighted	7	-0.032	0.088	0.719	0.176	0.938	No	7	-0.032	0.088	0.719	0.176	0.938
GLRX5	SBP	Inverse variance weighted	2	-0.358	0.419	0.392	-	0.770	No	2	-0.358	0.419	0.392	-	0.770
GLT8D2	SBP	Wald ratio	1	0.071	0.232	0.760	-	0.950	No	1	0.071	0.232	0.760	-	0.950
GM2A	SBP	Inverse variance weighted	6	-0.048	0.061	0.430	0.923	0.791	No	6	-0.048	0.061	0.430	0.923	0.791
GMFG	SBP	Wald ratio	1	-0.284	0.619	0.646	-	0.904	No	1	-0.284	0.619	0.646	-	0.904
GMPR	SBP	Inverse variance weighted	4	0.393	0.348	0.259	0.002	0.642	No	4	0.393	0.348	0.259	0.002	0.642
GMPR2	SBP	Inverse variance weighted	3	-0.753	1.157	0.515	0.001	0.846	No	3	-0.753	1.157	0.515	0.001	0.846
G-S	SBP	Wald ratio	1	0.852	0.543	0.117	-	0.407	No	1	0.852	0.543	0.117	-	0.407
GNLY	SBP	Inverse variance weighted	5	0.026	0.073	0.727	0.272	0.943	No	5	0.026	0.073	0.727	0.272	0.943
GNPDA1	SBP	Inverse variance weighted	3	0.472	0.855	0.581	0.004	0.878	No	3	0.472	0.855	0.581	0.004	0.878
GNPDA2	SBP	Inverse variance weighted	4	-0.124	0.212	0.557	0.145	0.865	No	4	-0.124	0.212	0.557	0.145	0.865
GOLGA3	SBP	Inverse variance weighted	2	0.759	0.356	0.033	-	0.203	No	2	0.759	0.356	0.033	-	0.203

ST2; MR causal estimates for plasma proteins on systolic blood pressure.

Causal candidates prioritized for SBP were marked as "Yes" in column "Prioritized". Effect of plasma protein levels on blood pressure is in mmHg unit.

										Steiger filtering					
**Exposure**	**Outocme**	**Method**	**nsnp**	**Beta**	**SE**	**P-value**	**Cochran's Q P-value**	**FDR-corrected P-value**	**Prioritized**	**nsnp**	**Beta**	**SE**	**P-value**	**Cochran's Q P-value**	**FDR-corrected P-value**
GOLM2	SBP	Inverse variance weighted	2	-0.766	1.145	0.504	-	0.840	No	2	-0.766	1.145	0.504	-	0.840
GORASP2	SBP	Wald ratio	1	3.142	0.817	0.000	-	0.003	Yes	1	3.142	0.817	0.000	-	0.003
GOT1	SBP	Wald ratio	1	-0.645	1.187	0.587	-	0.879	No	1	-0.645	1.187	0.587	-	0.879
GP1BA	SBP	Inverse variance weighted	2	-0.433	0.309	0.160	-	0.496	No	2	-0.433	0.309	0.160	-	0.496
GP1BB	SBP	Inverse variance weighted	2	-0.952	0.669	0.155	-	0.485	No	2	-0.952	0.669	0.155	-	0.485
GP2	SBP	Inverse variance weighted	2	0.835	1.073	0.436	-	0.796	No	2	0.835	1.073	0.436	-	0.796
GP5	SBP	Wald ratio	1	0.187	0.327	0.568	-	0.871	No	1	0.187	0.327	0.568	-	0.871
GP6	SBP	Wald ratio	1	0.018	0.135	0.892	-	0.975	No	1	0.018	0.135	0.892	-	0.975
GPA33	SBP	Inverse variance weighted	3	-0.156	0.271	0.564	0.198	0.870	No	3	-0.156	0.271	0.564	0.198	0.870
GPC1	SBP	Inverse variance weighted	3	-0.030	0.118	0.797	0.600	0.958	No	3	-0.030	0.118	0.797	0.600	0.958
GPC5	SBP	Inverse variance weighted	12	0.001	0.048	0.988	0.439	0.997	No	12	0.001	0.048	0.988	0.439	0.997
GPD1	SBP	Inverse variance weighted	2	-0.258	0.629	0.682	-	0.922	No	2	-0.258	0.629	0.682	-	0.922
GPHA2	SBP	Inverse variance weighted	4	-0.384	0.273	0.159	0.062	0.493	No	4	-0.384	0.273	0.159	0.062	0.493
GPIHBP1	SBP	Inverse variance weighted	2	-0.092	0.181	0.612	-	0.890	No	2	-0.092	0.181	0.612	-	0.890
GPNMB	SBP	Inverse variance weighted	7	0.127	0.154	0.412	0.015	0.785	No	7	0.127	0.154	0.412	0.015	0.785
GPR158	SBP	Wald ratio	1	0.160	1.000	0.873	-	0.972	No	1	0.160	1.000	0.873	-	0.972
GPR15L	SBP	Inverse variance weighted	4	0.020	0.179	0.913	0.925	0.982	No	4	0.020	0.179	0.913	0.925	0.982
GPR37	SBP	Inverse variance weighted	6	-0.048	0.056	0.391	0.671	0.770	No	6	-0.048	0.056	0.391	0.671	0.770
GPRC5C	SBP	Wald ratio	1	-0.396	0.343	0.248	-	0.629	No	1	-0.396	0.343	0.248	-	0.629
GRAP2	SBP	Wald ratio	1	0.842	0.626	0.179	-	0.530	No	1	0.842	0.626	0.179	-	0.530
GRHPR	SBP	Inverse variance weighted	2	0.036	0.260	0.889	-	0.975	No	2	0.036	0.260	0.889	-	0.975
GRIK2	SBP	Inverse variance weighted	7	0.020	0.148	0.893	0.689	0.977	No	7	0.020	0.148	0.893	0.689	0.977
GRK5	SBP	Wald ratio	1	0.100	0.155	0.520	-	0.850	No	1	0.100	0.155	0.520	-	0.850
GRN	SBP	Wald ratio	1	0.192	0.179	0.282	-	0.670	No	1	0.192	0.179	0.282	-	0.670
GRP	SBP	Inverse variance weighted	4	-0.341	0.118	0.004	0.667	0.043	Yes	4	-0.341	0.118	0.004	0.667	0.043
GRPEL1	SBP	Wald ratio	1	-1.887	0.840	0.025	-	0.164	No	1	-1.887	0.840	0.025	-	0.164
GSN	SBP	Inverse variance weighted	6	-0.132	0.244	0.587	0.621	0.879	No	6	-0.132	0.244	0.587	0.621	0.879
GSR	SBP	Inverse variance weighted	2	-0.650	0.371	0.080	-	0.330	No	2	-0.650	0.371	0.080	-	0.330
GSTA1	SBP	Inverse variance weighted	2	-0.139	0.124	0.263	-	0.645	No	2	-0.139	0.124	0.263	-	0.645
GSTA3	SBP	Inverse variance weighted	2	-0.140	0.126	0.265	-	0.649	No	2	-0.140	0.126	0.265	-	0.649
GSTM4	SBP	Inverse variance weighted	2	-0.249	0.103	0.016	-	0.120	No	2	-0.249	0.103	0.016	-	0.120
GSTP1	SBP	Inverse variance weighted	2	0.267	0.256	0.297	-	0.688	No	2	0.267	0.256	0.297	-	0.688
GSTT2B	SBP	Inverse variance weighted	15	-0.061	0.046	0.188	0.092	0.546	No	15	-0.061	0.046	0.188	0.092	0.546
GUCA2A	SBP	Inverse variance weighted	4	0.455	0.330	0.168	0.080	0.507	No	4	0.455	0.330	0.168	0.080	0.507
GUSB	SBP	Inverse variance weighted	3	0.405	0.262	0.122	0.986	0.418	No	3	0.405	0.262	0.122	0.986	0.418
GYS1	SBP	Wald ratio	1	-1.253	0.943	0.184	-	0.539	No	1	-1.253	0.943	0.184	-	0.539
GZMA	SBP	Inverse variance weighted	2	1.531	0.561	0.006	-	0.064	No	2	1.531	0.561	0.006	-	0.064
GZMB	SBP	Inverse variance weighted	2	0.119	0.127	0.350	-	0.733	No	2	0.119	0.127	0.350	-	0.733
GZMH	SBP	Inverse variance weighted	5	0.146	0.215	0.497	0.219	0.839	No	5	0.146	0.215	0.497	0.219	0.839
HADH	SBP	Wald ratio	1	2.155	0.748	0.004	-	0.044	Yes	1	2.155	0.748	0.004	-	0.044
HAGH	SBP	Inverse variance weighted	2	-0.127	0.423	0.764	-	0.950	No	2	-0.127	0.423	0.764	-	0.950
HAVCR1	SBP	Inverse variance weighted	6	0.013	0.181	0.941	0.000	0.984	No	6	0.013	0.181	0.941	0.000	0.984
HAVCR2	SBP	Inverse variance weighted	3	0.035	0.382	0.927	0.412	0.983	No	3	0.035	0.382	0.927	0.412	0.983
HBEGF	SBP	Inverse variance weighted	2	-0.229	1.183	0.847	-	0.967	No	2	-0.229	1.183	0.847	-	0.967
HBQ1	SBP	Inverse variance weighted	3	0.032	0.117	0.786	0.289	0.955	No	3	0.032	0.117	0.786	0.289	0.955
HBZ	SBP	Inverse variance weighted	11	-0.101	0.041	0.013	0.374	0.111	No	11	-0.101	0.041	0.013	0.374	0.111
HCLS1	SBP	Inverse variance weighted	2	-0.435	0.615	0.480	-	0.831	No	2	-0.435	0.615	0.480	-	0.831
HDDC2	SBP	Inverse variance weighted	6	0.097	0.074	0.189	0.142	0.549	No	6	0.097	0.074	0.189	0.142	0.549
HDGF	SBP	Inverse variance weighted	10	-0.082	0.045	0.072	0.107	0.312	No	10	-0.082	0.045	0.072	0.107	0.312
HDGFL2	SBP	Wald ratio	1	0.765	0.848	0.367	-	0.750	No	1	0.765	0.848	0.367	-	0.750
HEBP1	SBP	Wald ratio	1	-0.818	0.354	0.021	-	0.143	No	1	-0.818	0.354	0.021	-	0.143
HEG1	SBP	Inverse variance weighted	3	0.696	0.303	0.021	0.570	0.147	No	3	0.696	0.303	0.021	0.570	0.147
HEPACAM2	SBP	Wald ratio	1	-0.935	1.084	0.388	-	0.769	No	1	-0.935	1.084	0.388	-	0.769
HEXIM1	SBP	Wald ratio	1	5.674	0.579	0.000	-	0.000	Yes	1	5.674	0.579	0.000	-	0.000
HGF	SBP	Inverse variance weighted	2	0.457	0.404	0.258	-	0.641	No	2	0.457	0.404	0.258	-	0.641
HGFAC	SBP	Inverse variance weighted	6	-0.081	0.234	0.730	0.000	0.943	No	6	-0.081	0.234	0.730	0.000	0.943

ST2; MR causal estimates for plasma proteins on systolic blood pressure.

Causal candidates prioritized for SBP were marked as "Yes" in column "Prioritized". Effect of plasma protein levels on blood pressure is in mmHg unit.

										Steiger filtering					
**Exposure**	**Outocme**	**Method**	**nsnp**	**Beta**	**SE**	**P-value**	**Cochran's Q P-value**	**FDR-corrected P-value**	**Prioritized**	**nsnp**	**Beta**	**SE**	**P-value**	**Cochran's Q P-value**	**FDR-corrected P-value**
HHEX	SBP	Wald ratio	1	6.408	1.106	0.000	-	0.000	Yes	1	6.408	1.106	0.000	-	0.000
HIP1	SBP	Wald ratio	1	-0.635	0.943	0.501	-	0.839	No	1	-0.635	0.943	0.501	-	0.839
HIP1R	SBP	Inverse variance weighted	2	0.488	1.017	0.631	-	0.898	No	2	0.488	1.017	0.631	-	0.898
HJV	SBP	Wald ratio	1	-0.025	0.270	0.926	-	0.983	No	1	-0.025	0.270	0.926	-	0.983
HMBS	SBP	Inverse variance weighted	3	0.468	0.227	0.040	0.993	0.225	No	3	0.468	0.227	0.040	0.993	0.225
HMCN2	SBP	Inverse variance weighted	10	-0.024	0.087	0.783	0.036	0.953	No	10	-0.024	0.087	0.783	0.036	0.953
HMOX1	SBP	Inverse variance weighted	3	-0.105	0.621	0.866	0.118	0.971	No	3	-0.105	0.621	0.866	0.118	0.971
HMOX2	SBP	Wald ratio	1	1.425	0.403	0.000	-	0.008	Yes	1	1.425	0.403	0.000	-	0.008
HNMT	SBP	Inverse variance weighted	4	-0.058	0.106	0.580	0.014	0.878	No	4	-0.058	0.106	0.580	0.014	0.878
HPCAL1	SBP	Inverse variance weighted	2	-0.537	0.405	0.185	-	0.539	No	2	-0.537	0.405	0.185	-	0.539
HPGDS	SBP	Inverse variance weighted	7	0.259	0.125	0.039	0.034	0.225	No	7	0.259	0.125	0.039	0.034	0.225
HPSE	SBP	Wald ratio	1	0.247	0.262	0.345	-	0.728	No	1	0.247	0.262	0.345	-	0.728
HRC	SBP	Inverse variance weighted	2	0.401	0.413	0.331	-	0.713	No	2	0.401	0.413	0.331	-	0.713
HRG	SBP	Inverse variance weighted	4	-0.008	0.179	0.966	0.004	0.993	No	4	-0.008	0.179	0.966	0.004	0.993
HS1BP3	SBP	Inverse variance weighted	3	-1.008	0.477	0.035	0.245	0.211	No	3	-1.008	0.477	0.035	0.245	0.211
HS3ST3B1	SBP	Inverse variance weighted	6	-0.062	0.153	0.686	0.041	0.922	No	6	-0.062	0.153	0.686	0.041	0.922
HS6ST1	SBP	Inverse variance weighted	3	-0.032	0.170	0.848	0.813	0.967	No	3	-0.032	0.170	0.848	0.813	0.967
HSBP1	SBP	Inverse variance weighted	6	-0.014	0.083	0.868	0.132	0.971	No	6	-0.014	0.083	0.868	0.132	0.971
HSD17B14	SBP	Inverse variance weighted	13	-0.033	0.057	0.566	0.248	0.870	No	13	-0.033	0.057	0.566	0.248	0.870
HSDL2	SBP	Inverse variance weighted	6	0.103	0.068	0.128	0.555	0.433	No	6	0.103	0.068	0.128	0.555	0.433
HSPA2	SBP	Inverse variance weighted	2	0.196	0.712	0.783	-	0.953	No	2	0.196	0.712	0.783	-	0.953
HSPB1	SBP	Inverse variance weighted	5	-0.019	0.092	0.834	0.324	0.966	No	5	-0.019	0.092	0.834	0.324	0.966
HSPB6	SBP	Wald ratio	1	-0.618	1.206	0.608	-	0.888	No	1	-0.618	1.206	0.608	-	0.888
HSPG2	SBP	Inverse variance weighted	3	-0.130	0.545	0.811	0.004	0.963	No	3	-0.130	0.545	0.811	0.004	0.963
HYAL1	SBP	Inverse variance weighted	2	0.551	0.145	0.000	-	0.003	Yes	2	0.551	0.145	0.000	-	0.003
HYOU1	SBP	Inverse variance weighted	2	0.467	0.228	0.041	-	0.227	No	2	0.467	0.228	0.041	-	0.227
ICAM1	SBP	Inverse variance weighted	6	-0.258	0.098	0.008	0.381	0.077	No	6	-0.258	0.098	0.008	0.381	0.077
ICAM2	SBP	Wald ratio	1	2.273	0.753	0.003	-	0.032	Yes	1	2.273	0.753	0.003	-	0.032
ICAM3	SBP	Inverse variance weighted	4	-0.119	0.188	0.526	0.096	0.855	No	4	-0.119	0.188	0.526	0.096	0.855
ICAM4	SBP	Wald ratio	1	0.845	0.372	0.023	-	0.156	No	1	0.845	0.372	0.023	-	0.156
ICAM5	SBP	Inverse variance weighted	7	-0.065	0.084	0.441	0.038	0.801	No	7	-0.065	0.084	0.441	0.038	0.801
ICOSLG	SBP	Inverse variance weighted	5	-0.025	0.088	0.774	0.670	0.952	No	5	-0.025	0.088	0.774	0.670	0.952
IDO1	SBP	Inverse variance weighted	4	-0.209	0.219	0.341	0.074	0.725	No	4	-0.209	0.219	0.341	0.074	0.725
IDUA	SBP	Inverse variance weighted	8	-0.267	0.071	0.000	0.447	0.003	Yes	8	-0.267	0.071	0.000	0.447	0.003
IFI30	SBP	Inverse variance weighted	2	-0.389	0.095	0.000	-	0.001	Yes	2	-0.389	0.095	0.000	-	0.001
IFIT3	SBP	Wald ratio	1	-0.807	0.392	0.040	-	0.225	No	1	-0.807	0.392	0.040	-	0.225
IF-R1	SBP	Inverse variance weighted	6	0.189	0.097	0.052	0.797	0.263	No	6	0.189	0.097	0.052	0.797	0.263
IFNGR1	SBP	Wald ratio	1	0.304	0.239	0.204	-	0.572	No	1	0.304	0.239	0.204	-	0.572
IFNGR2	SBP	Inverse variance weighted	12	0.143	0.045	0.001	0.247	0.019	Yes	12	0.143	0.045	0.001	0.247	0.019
IFNL1	SBP	Wald ratio	1	1.209	0.733	0.099	-	0.372	No	1	1.209	0.733	0.099	-	0.372
IFNLR1	SBP	Inverse variance weighted	4	0.004	0.114	0.971	0.993	0.994	No	4	0.004	0.114	0.971	0.993	0.994
IGDCC4	SBP	Inverse variance weighted	11	-0.217	0.141	0.122	0.208	0.418	No	11	-0.217	0.141	0.122	0.208	0.418
IGF1R	SBP	Inverse variance weighted	3	-0.641	0.407	0.115	0.173	0.405	No	3	-0.641	0.407	0.115	0.173	0.405
IGF2R	SBP	Inverse variance weighted	9	-0.154	0.093	0.099	0.403	0.372	No	9	-0.154	0.093	0.099	0.403	0.372
IGFBP1	SBP	Wald ratio	1	-1.376	0.799	0.085	-	0.341	No	1	-1.376	0.799	0.085	-	0.341
IGFBP2	SBP	Inverse variance weighted	2	-0.168	0.608	0.782	-	0.953	No	2	-0.168	0.608	0.782	-	0.953
IGFBP3	SBP	Inverse variance weighted	4	-0.726	0.098	0.000	0.281	0.000	Yes	4	-0.726	0.098	0.000	0.281	0.000
IGFBP4	SBP	Wald ratio	1	0.166	0.859	0.847	-	0.967	No	1	0.166	0.859	0.847	-	0.967
IGFBP6	SBP	Wald ratio	1	1.003	0.506	0.047	-	0.251	No	1	1.003	0.506	0.047	-	0.251
IGFBP7	SBP	Inverse variance weighted	3	-0.209	0.197	0.289	0.063	0.675	No	3	-0.209	0.197	0.289	0.063	0.675
IGFBPL1	SBP	Inverse variance weighted	8	-0.055	0.081	0.499	0.629	0.839	No	8	-0.055	0.081	0.499	0.629	0.839
IGLC2	SBP	Inverse variance weighted	4	1.016	0.482	0.035	0.011	0.212	No	4	1.016	0.482	0.035	0.011	0.212
IGSF21	SBP	Inverse variance weighted	4	-0.107	0.221	0.626	0.612	0.897	No	4	-0.107	0.221	0.626	0.612	0.897
IGSF3	SBP	Inverse variance weighted	2	0.666	0.385	0.083	-	0.337	No	2	0.666	0.385	0.083	-	0.337
IGSF8	SBP	Wald ratio	1	-0.242	0.317	0.446	-	0.802	No	1	-0.242	0.317	0.446	-	0.802
IGSF9	SBP	Inverse variance weighted	5	0.084	0.215	0.695	0.069	0.925	No	5	0.084	0.215	0.695	0.069	0.925

ST2; MR causal estimates for plasma proteins on systolic blood pressure.

Causal candidates prioritized for SBP were marked as "Yes" in column "Prioritized". Effect of plasma protein levels on blood pressure is in mmHg unit.

										Steiger filtering					
**Exposure**	**Outocme**	**Method**	**nsnp**	**Beta**	**SE**	**P-value**	**Cochran's Q P-value**	**FDR-corrected P-value**	**Prioritized**	**nsnp**	**Beta**	**SE**	**P-value**	**Cochran's Q P-value**	**FDR-corrected P-value**
IL10	SBP	Wald ratio	1	-0.091	0.400	0.821	-	0.966	No	1	-0.091	0.400	0.821	-	0.966
IL10RA	SBP	Wald ratio	1	0.063	0.289	0.828	-	0.966	No	1	0.063	0.289	0.828	-	0.966
IL10RB	SBP	Inverse variance weighted	4	-0.061	0.178	0.732	0.001	0.943	No	4	-0.061	0.178	0.732	0.001	0.943
IL12RB1	SBP	Inverse variance weighted	2	0.025	0.230	0.912	-	0.982	No	2	0.025	0.230	0.912	-	0.982
IL15RA	SBP	Inverse variance weighted	3	-0.056	0.160	0.727	0.628	0.943	No	3	-0.056	0.160	0.727	0.628	0.943
IL16	SBP	Inverse variance weighted	2	0.050	0.080	0.535	-	0.858	No	2	0.050	0.080	0.535	-	0.858
IL17C	SBP	Inverse variance weighted	2	-0.886	0.322	0.006	-	0.062	No	2	-0.886	0.322	0.006	-	0.062
IL17D	SBP	Inverse variance weighted	4	-0.063	0.159	0.692	0.118	0.924	No	4	-0.063	0.159	0.692	0.118	0.924
IL17F	SBP	Inverse variance weighted	3	0.317	0.268	0.237	0.246	0.619	No	3	0.317	0.268	0.237	0.246	0.619
IL17RA	SBP	Inverse variance weighted	8	0.041	0.045	0.362	0.530	0.746	No	8	0.041	0.045	0.362	0.530	0.746
IL17RB	SBP	Inverse variance weighted	8	-0.146	0.117	0.213	0.000	0.585	No	8	-0.146	0.117	0.213	0.000	0.585
IL18	SBP	Inverse variance weighted	2	0.150	0.146	0.306	-	0.693	No	2	0.150	0.146	0.306	-	0.693
IL18BP	SBP	Wald ratio	1	-0.514	0.948	0.588	-	0.879	No	1	-0.514	0.948	0.588	-	0.879
IL18R1	SBP	Inverse variance weighted	11	0.076	0.051	0.137	0.977	0.446	No	11	0.076	0.051	0.137	0.977	0.446
IL19	SBP	Inverse variance weighted	2	-0.070	0.131	0.590	-	0.879	No	2	-0.070	0.131	0.590	-	0.879
IL1B	SBP	Wald ratio	1	0.319	1.148	0.781	-	0.953	No	1	0.319	1.148	0.781	-	0.953
IL1R1	SBP	Inverse variance weighted	3	-0.092	0.190	0.628	0.600	0.897	No	3	-0.092	0.190	0.628	0.600	0.897
IL1R2	SBP	Inverse variance weighted	4	0.049	0.075	0.517	0.410	0.849	No	4	0.049	0.075	0.517	0.410	0.849
IL1RAP	SBP	Inverse variance weighted	8	-0.023	0.051	0.660	0.076	0.914	No	8	-0.023	0.051	0.660	0.076	0.914
IL1RL1	SBP	Inverse variance weighted	8	0.157	0.070	0.026	0.143	0.168	No	8	0.157	0.070	0.026	0.143	0.168
IL1RL2	SBP	Inverse variance weighted	5	-0.050	0.114	0.661	0.171	0.914	No	5	-0.050	0.114	0.661	0.171	0.914
IL1RN	SBP	Inverse variance weighted	3	-0.193	0.152	0.205	0.466	0.572	No	3	-0.193	0.152	0.205	0.466	0.572
IL20	SBP	Wald ratio	1	-0.817	0.965	0.397	-	0.775	No	1	-0.817	0.965	0.397	-	0.775
IL20RB	SBP	Wald ratio	1	0.350	0.353	0.322	-	0.704	No	1	0.350	0.353	0.322	-	0.704
IL22	SBP	Inverse variance weighted	2	0.125	0.419	0.765	-	0.950	No	2	0.125	0.419	0.765	-	0.950
IL22RA1	SBP	Inverse variance weighted	2	0.126	0.156	0.420	-	0.790	No	2	0.126	0.156	0.420	-	0.790
IL2RA	SBP	Inverse variance weighted	8	0.058	0.069	0.407	0.577	0.784	No	8	0.058	0.069	0.407	0.577	0.784
IL31RA	SBP	Inverse variance weighted	7	0.063	0.085	0.459	0.125	0.814	No	7	0.063	0.085	0.459	0.125	0.814
IL32	SBP	Inverse variance weighted	4	0.188	0.160	0.240	0.326	0.620	No	4	0.188	0.160	0.240	0.326	0.620
IL34	SBP	Inverse variance weighted	6	0.029	0.154	0.852	0.000	0.967	No	6	0.029	0.154	0.852	0.000	0.967
IL36A	SBP	Wald ratio	1	0.075	0.818	0.927	-	0.983	No	1	0.075	0.818	0.927	-	0.983
IL36G	SBP	Inverse variance weighted	2	0.394	0.743	0.597	-	0.881	No	2	0.394	0.743	0.597	-	0.881
IL4R	SBP	Inverse variance weighted	2	0.051	0.270	0.850	-	0.967	No	2	0.051	0.270	0.850	-	0.967
IL5RA	SBP	Inverse variance weighted	7	-0.084	0.093	0.366	0.966	0.750	No	7	-0.084	0.093	0.366	0.966	0.750
IL6R	SBP	Inverse variance weighted	9	0.056	0.049	0.248	0.068	0.629	No	9	0.056	0.049	0.248	0.068	0.629
IL6ST	SBP	Inverse variance weighted	2	-0.407	0.226	0.072	-	0.312	No	2	-0.407	0.226	0.072	-	0.312
IL7	SBP	Inverse variance weighted	2	0.075	0.508	0.883	-	0.974	No	2	0.075	0.508	0.883	-	0.974
IL7R	SBP	Inverse variance weighted	6	-0.043	0.055	0.434	0.271	0.795	No	6	-0.043	0.055	0.434	0.271	0.795
IMMT	SBP	Wald ratio	1	-1.367	0.346	0.000	-	0.002	Yes	1	-1.367	0.346	0.000	-	0.002
IMPA1	SBP	Wald ratio	1	0.684	0.207	0.001	-	0.014	Yes	1	0.684	0.207	0.001	-	0.014
ING1	SBP	Wald ratio	1	-4.314	0.804	0.000	-	0.000	Yes	1	-4.314	0.804	0.000	-	0.000
INHBB	SBP	Inverse variance weighted	2	0.141	0.155	0.363	-	0.747	No	2	0.141	0.155	0.363	-	0.747
INHBC	SBP	Inverse variance weighted	4	0.202	0.109	0.064	0.063	0.297	No	4	0.202	0.109	0.064	0.063	0.297
INPP1	SBP	Wald ratio	1	-0.191	0.540	0.724	-	0.941	No	1	-0.191	0.540	0.724	-	0.941
INPP5D	SBP	Inverse variance weighted	2	0.571	0.234	0.015	-	0.115	No	2	0.571	0.234	0.015	-	0.115
INSL3	SBP	Wald ratio	1	0.392	1.401	0.779	-	0.953	No	1	0.392	1.401	0.779	-	0.953
INSL4	SBP	Wald ratio	1	0.199	0.238	0.401	-	0.779	No	1	0.199	0.238	0.401	-	0.779
INSL5	SBP	Inverse variance weighted	2	-0.385	0.225	0.088	-	0.350	No	2	-0.385	0.225	0.088	-	0.350
INSR	SBP	Wald ratio	1	-2.115	0.773	0.006	-	0.063	No	1	-2.115	0.773	0.006	-	0.063
IPCEF1	SBP	Inverse variance weighted	2	0.556	0.420	0.185	-	0.539	No	2	0.556	0.420	0.185	-	0.539
IQGAP2	SBP	Wald ratio	1	-0.352	0.296	0.234	-	0.617	No	1	-0.352	0.296	0.234	-	0.617
IRAK4	SBP	Wald ratio	1	-0.080	0.635	0.900	-	0.978	No	1	-0.080	0.635	0.900	-	0.978
ISLR2	SBP	Inverse variance weighted	2	-0.472	0.192	0.014	-	0.113	No	2	-0.472	0.192	0.014	-	0.113
ISM1	SBP	Inverse variance weighted	5	0.070	0.153	0.647	0.862	0.904	No	5	0.070	0.153	0.647	0.862	0.904
IST1	SBP	Wald ratio	1	0.933	0.507	0.066	-	0.299	No	1	0.933	0.507	0.066	-	0.299
ITGA11	SBP	Inverse variance weighted	5	-0.087	0.326	0.790	0.001	0.955	No	5	-0.087	0.326	0.790	0.001	0.955

ST2; MR causal estimates for plasma proteins on systolic blood pressure.
Causal candidates prioritized for SBP were marked as "Yes" in column "Prioritized". Effect of plasma protein levels on blood pressure is in mmHg unit.

										Steiger filtering					
Exposure	Outocme	Method	nsnp	Beta	SE	P-value	Cochran's Q P-value	FDR-corrected P-value	Prioritized	nsnp	Beta	SE	P-value	Cochran's Q P-value	FDR-corrected P-value
ITGA2	SBP	Inverse variance weighted	5	0.089	0.126	0.481	0.006	0.831	No	5	0.089	0.126	0.481	0.006	0.831
ITGA5	SBP	Wald ratio	1	0.536	0.794	0.499	-	0.839	No	1	0.536	0.794	0.499	-	0.839
ITGA6	SBP	Inverse variance weighted	4	-0.188	0.240	0.433	0.127	0.794	No	4	-0.188	0.240	0.433	0.127	0.794
ITGAL	SBP	Wald ratio	1	5.321	1.151	0.000	-	0.000	Yes	1	5.321	1.151	0.000	-	0.000
ITGAM	SBP	Wald ratio	1	-0.172	0.270	0.524	-	0.854	No	1	-0.172	0.270	0.524	-	0.854
ITGAV	SBP	Inverse variance weighted	3	-0.159	0.672	0.813	0.000	0.963	No	3	-0.159	0.672	0.813	0.000	0.963
ITGAX	SBP	Wald ratio	1	2.241	0.959	0.019	-	0.140	No	1	2.241	0.959	0.019	-	0.140
ITGB2	SBP	Wald ratio	1	0.058	0.352	0.869	-	0.971	No	1	0.058	0.352	0.869	-	0.971
ITGB5	SBP	Inverse variance weighted	2	-1.175	2.861	0.681	-	0.922	No	2	-1.175	2.861	0.681	-	0.922
ITGB6	SBP	Inverse variance weighted	3	0.009	0.142	0.950	0.679	0.986	No	3	0.009	0.142	0.950	0.679	0.986
ITGB7	SBP	Inverse variance weighted	2	-0.004	0.613	0.994	-	0.997	No	2	-0.004	0.613	0.994	-	0.997
ITGBL1	SBP	Inverse variance weighted	5	-0.036	0.152	0.815	0.123	0.963	No	5	-0.036	0.152	0.815	0.123	0.963
ITIH1	SBP	Wald ratio	1	-1.522	0.303	0.000	-	0.000	Yes	1	-1.522	0.303	0.000	-	0.000
ITIH3	SBP	Inverse variance weighted	5	0.573	0.232	0.013	0.014	0.111	No	5	0.573	0.232	0.013	0.014	0.111
ITIH4	SBP	Inverse variance weighted	4	0.727	0.319	0.023	0.005	0.154	No	4	0.727	0.319	0.023	0.005	0.154
ITIH5	SBP	Wald ratio	1	0.112	0.398	0.778	-	0.953	No	1	0.112	0.398	0.778	-	0.953
ITPA	SBP	Inverse variance weighted	7	-0.010	0.065	0.874	0.249	0.972	No	7	-0.010	0.065	0.874	0.249	0.972
ITPR1	SBP	Wald ratio	1	0.188	0.423	0.656	-	0.912	No	1	0.188	0.423	0.656	-	0.912
JAM2	SBP	Inverse variance weighted	2	-0.831	0.519	0.109	-	0.392	No	2	-0.831	0.519	0.109	-	0.392
JAM3	SBP	Inverse variance weighted	2	0.994	0.869	0.253	-	0.635	No	2	0.994	0.869	0.253	-	0.635
JPT2	SBP	Wald ratio	1	-0.800	0.885	0.366	-	0.750	No	1	-0.800	0.885	0.366	-	0.750
KAZALD1	SBP	Inverse variance weighted	5	-0.133	0.108	0.216	0.010	0.589	No	5	-0.133	0.108	0.216	0.010	0.589
KCTD5	SBP	Wald ratio	1	-2.170	0.920	0.018	-	0.133	No	1	-2.170	0.920	0.018	-	0.133
KDR	SBP	Inverse variance weighted	7	-0.030	0.121	0.802	0.014	0.959	No	7	-0.030	0.121	0.802	0.014	0.959
KEL	SBP	Inverse variance weighted	6	0.048	0.168	0.774	0.024	0.952	No	6	0.048	0.168	0.774	0.024	0.952
KHK	SBP	Inverse variance weighted	3	-0.018	0.177	0.920	0.027	0.982	No	3	-0.018	0.177	0.920	0.027	0.982
KIAA0319	SBP	Inverse variance weighted	5	-0.339	0.193	0.079	0.059	0.329	No	5	-0.339	0.193	0.079	0.059	0.329
KIF1C	SBP	Wald ratio	1	1.389	0.916	0.129	-	0.433	No	1	1.389	0.916	0.129	-	0.433
KIF22	SBP	Wald ratio	1	-1.018	1.165	0.382	-	0.763	No	1	-1.018	1.165	0.382	-	0.763
KIFBP	SBP	Wald ratio	1	2.978	0.931	0.001	-	0.019	Yes	1	2.978	0.931	0.001	-	0.019
KIR2DL2	SBP	Inverse variance weighted	5	-0.059	0.069	0.390	0.749	0.770	No	5	-0.059	0.069	0.390	0.749	0.770
KIR2DL3	SBP	Inverse variance weighted	4	-0.020	0.126	0.875	0.192	0.972	No	4	-0.020	0.126	0.875	0.192	0.972
KIR2DS4	SBP	Inverse variance weighted	8	-0.089	0.036	0.013	0.939	0.111	No	8	-0.089	0.036	0.013	0.939	0.111
KIR3DL1	SBP	Inverse variance weighted	8	0.031	0.041	0.447	0.839	0.803	No	8	0.031	0.041	0.447	0.839	0.803
KIR3DL2	SBP	Wald ratio	1	-0.101	0.319	0.752	-	0.946	No	1	-0.101	0.319	0.752	-	0.946
KIRREL2	SBP	Inverse variance weighted	6	0.129	0.197	0.514	0.629	0.846	No	6	0.129	0.197	0.514	0.629	0.846
KIT	SBP	Inverse variance weighted	2	-0.395	1.888	0.834	-	0.966	No	2	-0.395	1.888	0.834	-	0.966
KITLG	SBP	Wald ratio	1	-1.611	0.724	0.026	-	0.170	No	1	-1.611	0.724	0.026	-	0.170
KLB	SBP	Inverse variance weighted	5	0.086	0.054	0.111	0.653	0.395	No	5	0.086	0.054	0.111	0.653	0.395
KLHL41	SBP	Inverse variance weighted	2	1.101	0.739	0.136	-	0.446	No	2	1.101	0.739	0.136	-	0.446
KLK1	SBP	Inverse variance weighted	4	-0.179	0.166	0.282	0.073	0.670	No	4	-0.179	0.166	0.282	0.073	0.670
KLK10	SBP	Inverse variance weighted	6	0.010	0.071	0.889	0.350	0.975	No	6	0.010	0.071	0.889	0.350	0.975
KLK11	SBP	Inverse variance weighted	3	-0.010	0.096	0.920	0.489	0.982	No	3	-0.010	0.096	0.920	0.489	0.982
KLK12	SBP	Inverse variance weighted	10	-0.012	0.038	0.742	0.281	0.945	No	10	-0.012	0.038	0.742	0.281	0.945
KLK13	SBP	Inverse variance weighted	5	0.008	0.119	0.946	0.118	0.984	No	5	0.008	0.119	0.946	0.118	0.984
KLK14	SBP	Inverse variance weighted	5	-0.090	0.103	0.382	0.257	0.763	No	5	-0.090	0.103	0.382	0.257	0.763
KLK15	SBP	Inverse variance weighted	9	0.033	0.052	0.523	0.218	0.853	No	9	0.033	0.052	0.523	0.218	0.853
KLK3	SBP	Inverse variance weighted	2	1.243	0.702	0.077	-	0.323	No	2	1.243	0.702	0.077	-	0.323
KLK4	SBP	Inverse variance weighted	6	0.014	0.115	0.906	0.197	0.980	No	6	0.014	0.115	0.906	0.197	0.980
KLK6	SBP	Wald ratio	1	0.227	0.221	0.304	-	0.692	No	1	0.227	0.221	0.304	-	0.692
KLK7	SBP	Inverse variance weighted	4	-0.004	0.116	0.974	0.180	0.995	No	4	-0.004	0.116	0.974	0.180	0.995
KLK8	SBP	Inverse variance weighted	7	0.165	0.090	0.067	0.232	0.301	No	7	0.165	0.090	0.067	0.232	0.301
KLKB1	SBP	Inverse variance weighted	2	-0.205	0.115	0.074	-	0.318	No	2	-0.205	0.115	0.074	-	0.318
KLRB1	SBP	Inverse variance weighted	6	0.191	0.408	0.640	0.013	0.901	No	6	0.191	0.408	0.640	0.013	0.901
KLRD1	SBP	Inverse variance weighted	4	0.086	0.136	0.526	0.095	0.855	No	4	0.086	0.136	0.526	0.095	0.855
KLRF1	SBP	Inverse variance weighted	4	0.054	0.276	0.844	0.057	0.967	No	4	0.054	0.276	0.844	0.057	0.967

ST2; MR causal estimates for plasma proteins on systolic blood pressure.

Causal candidates prioritized for SBP were marked as "Yes" in column "Prioritized". Effect of plasma protein levels on blood pressure is in mmHg unit.

										Steiger filtering					
**Exposure**	**Outcocme**	**Method**	**nsnp**	**Beta**	**SE**	**P-value**	**Cochran's Q P-value**	**FDR-corrected P-value**	**Prioritized**	**nsnp**	**Beta**	**SE**	**P-value**	**Cochran's Q P-value**	**FDR-corrected P-value**
KLRK1	SBP	Inverse variance weighted	4	-0.137	0.082	0.096	0.497	0.369	No	4	-0.137	0.082	0.096	0.497	0.369
KRT18	SBP	Wald ratio	1	0.169	0.959	0.860	-	0.969	No	1	0.169	0.959	0.860	-	0.969
KRT19	SBP	Wald ratio	1	0.185	0.275	0.501	-	0.839	No	1	0.185	0.275	0.501	-	0.839
KRT5	SBP	Inverse variance weighted	2	-0.398	0.672	0.554	-	0.865	No	2	-0.398	0.672	0.554	-	0.865
KYAT1	SBP	Wald ratio	1	0.128	0.309	0.678	-	0.922	No	1	0.128	0.309	0.678	-	0.922
KYNU	SBP	Inverse variance weighted	5	0.097	0.148	0.514	0.373	0.846	No	5	0.097	0.148	0.514	0.373	0.846
LACRT	SBP	Wald ratio	1	-1.765	0.587	0.003	-	0.033	Yes	1	-1.765	0.587	0.003	-	0.033
LACTB2	SBP	Inverse variance weighted	2	-0.159	0.260	0.541	-	0.858	No	2	-0.159	0.260	0.541	-	0.858
LAG3	SBP	Inverse variance weighted	2	0.112	0.230	0.627	-	0.897	No	2	0.112	0.230	0.627	-	0.897
LAIR1	SBP	Inverse variance weighted	7	-0.067	0.071	0.349	0.810	0.733	No	7	-0.067	0.071	0.349	0.810	0.733
LAIR2	SBP	Inverse variance weighted	13	0.052	0.058	0.370	0.248	0.751	No	13	0.052	0.058	0.370	0.248	0.751
LAMA4	SBP	Wald ratio	1	-0.538	0.252	0.033	-	0.203	No	1	-0.538	0.252	0.033	-	0.203
LAMB1	SBP	Inverse variance weighted	7	0.022	0.118	0.855	0.001	0.968	No	7	0.022	0.118	0.855	0.001	0.968
LAMP1	SBP	Wald ratio	1	-0.115	0.729	0.875	-	0.972	No	1	-0.115	0.729	0.875	-	0.972
LAMP3	SBP	Wald ratio	1	-0.135	0.185	0.466	-	0.817	No	1	-0.135	0.185	0.466	-	0.817
LAP3	SBP	Wald ratio	1	-0.500	0.739	0.499	-	0.839	No	1	-0.500	0.739	0.499	-	0.839
LAT	SBP	Wald ratio	1	1.922	0.942	0.041	-	0.228	No	1	1.922	0.942	0.041	-	0.228
LAT2	SBP	Wald ratio	1	1.021	1.119	0.362	-	0.746	No	1	1.021	1.119	0.362	-	0.746
LATS1	SBP	Wald ratio	1	0.989	0.691	0.152	-	0.481	No	1	0.989	0.691	0.152	-	0.481
LAYN	SBP	Inverse variance weighted	3	-0.447	0.192	0.020	0.049	0.142	No	3	-0.447	0.192	0.020	0.049	0.142
LBP	SBP	Inverse variance weighted	7	0.095	0.080	0.233	0.086	0.617	No	7	0.095	0.080	0.233	0.086	0.617
LBR	SBP	Wald ratio	1	-0.979	1.066	0.358	-	0.742	No	1	-0.979	1.066	0.358	-	0.742
LCAT	SBP	Inverse variance weighted	2	-0.192	0.319	0.547	-	0.862	No	2	-0.192	0.319	0.547	-	0.862
LCN15	SBP	Wald ratio	1	0.137	0.073	0.062	-	0.292	No	1	0.137	0.073	0.062	-	0.292
LCN2	SBP	Wald ratio	1	-1.097	0.627	0.080	-	0.331	No	1	-1.097	0.627	0.080	-	0.331
LCP1	SBP	Inverse variance weighted	5	0.007	0.065	0.916	0.101	0.982	No	5	0.007	0.065	0.916	0.101	0.982
LDLR	SBP	Wald ratio	1	-0.835	1.013	0.410	-	0.785	No	1	-0.835	1.013	0.410	-	0.785
LDLRAP1	SBP	Wald ratio	1	0.559	0.582	0.336	-	0.720	No	1	0.559	0.582	0.336	-	0.720
LECT2	SBP	Inverse variance weighted	7	0.056	0.051	0.268	0.591	0.653	No	7	0.056	0.051	0.268	0.591	0.653
LEFTY2	SBP	Inverse variance weighted	8	-0.019	0.072	0.793	0.118	0.956	No	8	-0.019	0.072	0.793	0.118	0.956
LEG1	SBP	Wald ratio	1	0.186	0.108	0.085	-	0.341	No	1	0.186	0.108	0.085	-	0.341
LEP	SBP	Wald ratio	1	2.531	1.074	0.018	-	0.133	No	1	2.531	1.074	0.018	-	0.133
LEPR	SBP	Inverse variance weighted	2	0.011	0.587	0.985	-	0.997	No	2	0.011	0.587	0.985	-	0.997
LGALS1	SBP	Inverse variance weighted	2	-0.218	0.205	0.287	-	0.675	No	2	-0.218	0.205	0.287	-	0.675
LGALS3	SBP	Inverse variance weighted	4	-0.073	0.074	0.324	0.499	0.705	No	4	-0.073	0.074	0.324	0.499	0.705
LGALS3BP	SBP	Inverse variance weighted	6	-0.166	0.221	0.454	0.679	0.810	No	6	-0.166	0.221	0.454	0.679	0.810
LGALS4	SBP	Inverse variance weighted	3	-0.192	0.555	0.730	0.043	0.943	No	3	-0.192	0.555	0.730	0.043	0.943
LGALS8	SBP	Wald ratio	1	-0.227	0.149	0.128	-	0.432	No	1	-0.227	0.149	0.128	-	0.432
LGALS9	SBP	Inverse variance weighted	2	0.046	0.212	0.829	-	0.966	No	2	0.046	0.212	0.829	-	0.966
LGMN	SBP	Inverse variance weighted	11	0.038	0.129	0.770	0.707	0.950	No	11	0.038	0.129	0.770	0.707	0.950
LHB	SBP	Wald ratio	1	-1.186	0.817	0.147	-	0.468	No	1	-1.186	0.817	0.147	-	0.468
LHPP	SBP	Inverse variance weighted	8	-0.060	0.114	0.601	0.252	0.884	No	8	-0.060	0.114	0.601	0.252	0.884
LIF	SBP	Wald ratio	1	-0.409	0.900	0.650	-	0.907	No	1	-0.409	0.900	0.650	-	0.907
LIFR	SBP	Inverse variance weighted	3	0.189	0.221	0.393	0.904	0.770	No	3	0.189	0.221	0.393	0.904	0.770
LILRA2	SBP	Inverse variance weighted	7	0.076	0.075	0.309	0.225	0.696	No	7	0.076	0.075	0.309	0.225	0.696
LILRA3	SBP	Inverse variance weighted	8	-0.014	0.047	0.770	0.162	0.950	No	8	-0.014	0.047	0.770	0.162	0.950
LILRA4	SBP	Wald ratio	1	-1.470	1.125	0.191	-	0.553	No	1	-1.470	1.125	0.191	-	0.553
LILRA5	SBP	Inverse variance weighted	4	-0.295	0.121	0.014	0.301	0.114	No	4	-0.295	0.121	0.014	0.301	0.114
LILRA6	SBP	Inverse variance weighted	13	0.029	0.061	0.640	0.075	0.901	No	13	0.029	0.061	0.640	0.075	0.901
LILRB1	SBP	Inverse variance weighted	8	-0.067	0.108	0.535	0.002	0.858	No	8	-0.067	0.108	0.535	0.002	0.858
LILRB2	SBP	Inverse variance weighted	8	0.041	0.043	0.338	0.378	0.723	No	8	0.041	0.043	0.338	0.378	0.723
LILRB4	SBP	Inverse variance weighted	8	-0.065	0.146	0.657	0.398	0.912	No	8	-0.065	0.146	0.657	0.398	0.912
LILRB5	SBP	Inverse variance weighted	11	0.040	0.033	0.231	0.834	0.615	No	11	0.040	0.033	0.231	0.834	0.615
LIPF	SBP	Inverse variance weighted	2	0.092	0.289	0.751	-	0.946	No	2	0.092	0.289	0.751	-	0.946
LMNB1	SBP	Wald ratio	1	0.826	1.078	0.443	-	0.801	No	1	0.826	1.078	0.443	-	0.801
LMNB2	SBP	Wald ratio	1	-1.142	0.961	0.235	-	0.618	No	1	-1.142	0.961	0.235	-	0.618

ST2; MR causal estimates for plasma proteins on systolic blood pressure.

Causal candidates prioritized for SBP were marked as "Yes" in column "Prioritized". Effect of plasma protein levels on blood pressure is in mmHg unit.

										Steiger filtering					
**Exposure**	**Outocme**	**Method**	**nsnp**	**Beta**	**SE**	**P-value**	**Cochran's Q P-value**	**FDR-corrected P-value**	**Prioritized**	**nsnp**	**Beta**	**SE**	**P-value**	**Cochran's Q P-value**	**FDR-corrected P-value**
LMOD1	SBP	Wald ratio	1	-0.856	0.238	0.000	-	0.006	Yes	1	-0.856	0.238	0.000	-	0.006
LPA	SBP	Inverse variance weighted	19	0.152	0.083	0.066	0.019	0.299	No	19	0.152	0.083	0.066	0.019	0.299
LPO	SBP	Inverse variance weighted	2	0.186	0.164	0.256	-	0.639	No	2	0.186	0.164	0.256	-	0.639
LRCH4	SBP	Wald ratio	1	-0.006	0.645	0.992	-	0.997	No	1	-0.006	0.645	0.992	-	0.997
LRIG1	SBP	Inverse variance weighted	10	0.229	0.050	0.000	0.688	0.000	Yes	10	0.229	0.050	0.000	0.688	0.000
LRIG3	SBP	Wald ratio	1	-0.259	0.514	0.614	-	0.890	No	1	-0.259	0.514	0.614	-	0.890
LRP11	SBP	Inverse variance weighted	7	-0.123	0.087	0.158	0.126	0.492	No	7	-0.123	0.087	0.158	0.126	0.492
LRRC37A2	SBP	Inverse variance weighted	6	-0.223	0.086	0.009	0.001	0.085	No	6	-0.223	0.086	0.009	0.001	0.085
LRRFIP1	SBP	Wald ratio	1	1.174	0.632	0.063	-	0.292	No	1	1.174	0.632	0.063	-	0.292
LRRN1	SBP	Inverse variance weighted	6	0.039	0.051	0.441	0.493	0.801	No	6	0.039	0.051	0.441	0.493	0.801
LRTM2	SBP	Inverse variance weighted	2	0.214	0.260	0.410	-	0.785	No	2	0.214	0.260	0.410	-	0.785
LSP1	SBP	Inverse variance weighted	3	2.126	1.267	0.093	0.000	0.362	No	3	2.126	1.267	0.093	0.000	0.362
LTA4H	SBP	Inverse variance weighted	2	-0.624	0.415	0.132	-	0.437	No	2	-0.624	0.415	0.132	-	0.437
LTBP2	SBP	Inverse variance weighted	2	-1.848	0.990	0.062	-	0.292	No	2	-1.848	0.990	0.062	-	0.292
LTBP3	SBP	Inverse variance weighted	6	-0.035	0.209	0.867	0.000	0.971	No	6	-0.035	0.209	0.867	0.000	0.971
LTBR	SBP	Inverse variance weighted	4	-0.199	0.172	0.249	0.188	0.629	No	4	-0.199	0.172	0.249	0.188	0.629
LUZP2	SBP	Inverse variance weighted	6	-0.128	0.146	0.381	0.347	0.762	No	6	-0.128	0.146	0.381	0.347	0.762
LXN	SBP	Inverse variance weighted	2	-0.842	0.563	0.135	-	0.443	No	2	-0.842	0.563	0.135	-	0.443
LY6D	SBP	Inverse variance weighted	5	0.130	0.241	0.589	0.000	0.879	No	5	0.130	0.241	0.589	0.000	0.879
LY75	SBP	Inverse variance weighted	7	0.021	0.040	0.609	0.590	0.888	No	7	0.021	0.040	0.609	0.590	0.888
LY9	SBP	Inverse variance weighted	5	-0.002	0.083	0.980	0.333	0.996	No	5	-0.002	0.083	0.980	0.333	0.996
LY96	SBP	Inverse variance weighted	2	0.464	0.606	0.444	-	0.801	No	2	0.464	0.606	0.444	-	0.801
LYAR	SBP	Wald ratio	1	2.683	0.817	0.001	-	0.015	Yes	1	2.683	0.817	0.001	-	0.015
LYN	SBP	Wald ratio	1	-1.442	1.138	0.205	-	0.572	No	1	-1.442	1.138	0.205	-	0.572
LYPD3	SBP	Inverse variance weighted	4	-0.146	0.117	0.213	0.748	0.585	No	4	-0.146	0.117	0.213	0.748	0.585
LYPD8	SBP	Inverse variance weighted	8	0.102	0.100	0.308	0.066	0.696	No	8	0.102	0.100	0.308	0.066	0.696
LYSMD3	SBP	Wald ratio	1	1.318	0.779	0.090	-	0.356	No	1	1.318	0.779	0.090	-	0.356
LYVE1	SBP	Inverse variance weighted	5	0.358	0.572	0.531	0.000	0.858	No	5	0.358	0.572	0.531	0.000	0.858
LYZL2	SBP	Inverse variance weighted	2	0.180	0.200	0.369	-	0.751	No	2	0.180	0.200	0.369	-	0.751
LZTFL1	SBP	Wald ratio	1	-0.170	0.279	0.543	-	0.858	No	1	-0.170	0.279	0.543	-	0.858
M6PR	SBP	Wald ratio	1	-0.951	0.226	0.000	-	0.001	Yes	1	-0.951	0.226	0.000	-	0.001
MAD1L1	SBP	Inverse variance weighted	2	-0.161	0.840	0.848	-	0.967	No	2	-0.161	0.840	0.848	-	0.967
MAMDC2	SBP	Inverse variance weighted	3	-0.095	0.387	0.806	0.026	0.961	No	3	-0.095	0.387	0.806	0.026	0.961
MAMDC4	SBP	Inverse variance weighted	3	0.189	0.230	0.411	0.512	0.785	No	3	0.189	0.230	0.411	0.512	0.785
MAN1A2	SBP	Inverse variance weighted	4	0.230	0.190	0.226	0.555	0.605	No	4	0.230	0.190	0.226	0.555	0.605
MAN2B2	SBP	Inverse variance weighted	6	0.089	0.116	0.443	0.422	0.801	No	6	0.089	0.116	0.443	0.422	0.801
MANEAL	SBP	Wald ratio	1	4.436	1.102	0.000	-	0.002	Yes	1	4.436	1.102	0.000	-	0.002
MANF	SBP	Wald ratio	1	-1.320	0.590	0.025	-	0.165	No	1	-1.320	0.590	0.025	-	0.165
MANSC1	SBP	Wald ratio	1	-0.977	1.011	0.334	-	0.716	No	1	-0.977	1.011	0.334	-	0.716
MANSC4	SBP	Inverse variance weighted	5	-0.192	0.050	0.000	0.408	0.003	Yes	5	-0.192	0.050	0.000	0.408	0.003
MAP2	SBP	Inverse variance weighted	2	0.239	0.366	0.514	-	0.846	No	2	0.239	0.366	0.514	-	0.846
MAP2K1	SBP	Wald ratio	1	-0.839	0.780	0.282	-	0.670	No	1	-0.839	0.780	0.282	-	0.670
MAP4K5	SBP	Wald ratio	1	1.201	0.223	0.000	-	0.000	Yes	1	1.201	0.223	0.000	-	0.000
MAPK13	SBP	Wald ratio	1	1.149	0.745	0.123	-	0.418	No	1	1.149	0.745	0.123	-	0.418
MAPK9	SBP	Inverse variance weighted	2	-0.396	0.844	0.639	-	0.901	No	2	-0.396	0.844	0.639	-	0.901
MAPKAPK2	SBP	Inverse variance weighted	3	-0.087	0.255	0.733	0.568	0.943	No	3	-0.087	0.255	0.733	0.568	0.943
MARCO	SBP	Inverse variance weighted	3	0.272	0.158	0.084	0.520	0.338	No	3	0.272	0.158	0.084	0.520	0.338
MASP1	SBP	Inverse variance weighted	3	-0.383	0.197	0.051	0.901	0.262	No	3	-0.383	0.197	0.051	0.901	0.262
MATN2	SBP	Inverse variance weighted	3	0.026	0.101	0.793	0.955	0.956	No	3	0.026	0.101	0.793	0.955	0.956
MATN3	SBP	Inverse variance weighted	6	0.048	0.072	0.507	0.690	0.842	No	6	0.048	0.072	0.507	0.690	0.842
MAVS	SBP	Wald ratio	1	0.548	0.473	0.247	-	0.628	No	1	0.548	0.473	0.247	-	0.628
MAX	SBP	Wald ratio	1	0.923	0.482	0.056	-	0.275	No	1	0.923	0.482	0.056	-	0.275
MB	SBP	Wald ratio	1	-1.294	1.368	0.344	-	0.728	No	1	-1.294	1.368	0.344	-	0.728
MBL2	SBP	Inverse variance weighted	12	0.027	0.044	0.540	0.961	0.858	No	12	0.027	0.044	0.540	0.961	0.858
MCAM	SBP	Inverse variance weighted	3	0.042	0.254	0.869	0.450	0.971	No	3	0.042	0.254	0.869	0.450	0.971
MCEE	SBP	Wald ratio	1	0.354	0.213	0.098	-	0.369	No	1	0.354	0.213	0.098	-	0.369

ST2; MR causal estimates for plasma proteins on systolic blood pressure.

Causal candidates prioritized for SBP were marked as "Yes" in column "Prioritized". Effect of plasma protein levels on blood pressure is in mmHg unit.

										Steiger filtering					
Exposure	Outocme	Method	nsnp	Beta	SE	P-value	Cochran's Q P-value	FDR-corrected P-value	Prioritized	nsnp	Beta	SE	P-value	Cochran's Q P-value	FDR-corrected P-value
MCEMP1	SBP	Inverse variance weighted	3	-0.137	0.215	0.525	0.221	0.854	No	3	-0.137	0.215	0.525	0.221	0.854
MCFD2	SBP	Wald ratio	1	-0.482	0.299	0.107	-	0.390	No	1	-0.482	0.299	0.107	-	0.390
MDGA1	SBP	Inverse variance weighted	13	-0.036	0.036	0.314	0.728	0.696	No	13	-0.036	0.036	0.314	0.728	0.696
MDH1	SBP	Inverse variance weighted	2	-1.489	0.434	0.001	-	0.010	Yes	2	-1.489	0.434	0.001	-	0.010
MDK	SBP	Inverse variance weighted	3	0.464	0.330	0.159	0.661	0.494	No	3	0.464	0.330	0.159	0.661	0.494
MDM1	SBP	Wald ratio	1	-0.270	0.223	0.225	-	0.603	No	1	-0.270	0.223	0.225	-	0.603
MECR	SBP	Wald ratio	1	-0.552	0.534	0.301	-	0.688	No	1	-0.552	0.534	0.301	-	0.688
MEGF10	SBP	Inverse variance weighted	4	0.141	0.077	0.067	0.556	0.302	No	4	0.141	0.077	0.067	0.556	0.302
MEGF11	SBP	Inverse variance weighted	2	0.458	0.263	0.082	-	0.336	No	2	0.458	0.263	0.082	-	0.336
MEGF9	SBP	Wald ratio	1	-0.445	0.132	0.001	-	0.012	Yes	1	-0.445	0.132	0.001	-	0.012
MELTF	SBP	Inverse variance weighted	14	0.042	0.099	0.671	0.173	0.918	No	14	0.042	0.099	0.671	0.173	0.918
MENT	SBP	Inverse variance weighted	3	0.861	1.146	0.453	0.024	0.809	No	3	0.861	1.146	0.453	0.024	0.809
MEP1A	SBP	Inverse variance weighted	3	-0.206	0.199	0.300	0.547	0.688	No	3	-0.206	0.199	0.300	0.547	0.688
MEP1B	SBP	Inverse variance weighted	10	0.043	0.036	0.237	0.960	0.619	No	10	0.043	0.036	0.237	0.960	0.619
MEPE	SBP	Inverse variance weighted	2	0.183	0.304	0.548	-	0.862	No	2	0.183	0.304	0.548	-	0.862
MERTK	SBP	Inverse variance weighted	3	0.149	0.153	0.329	0.528	0.711	No	3	0.149	0.153	0.329	0.528	0.711
MET	SBP	Inverse variance weighted	2	0.967	0.908	0.287	-	0.675	No	2	0.967	0.908	0.287	-	0.675
METAP1D	SBP	Wald ratio	1	1.644	0.875	0.060	-	0.288	No	1	1.644	0.875	0.060	-	0.288
METAP2	SBP	Wald ratio	1	0.113	0.762	0.882	-	0.974	No	1	0.113	0.762	0.882	-	0.974
MFAP4	SBP	Inverse variance weighted	5	0.152	0.279	0.586	0.034	0.879	No	5	0.152	0.279	0.586	0.034	0.879
MFAP5	SBP	Inverse variance weighted	3	0.137	0.252	0.587	0.197	0.879	No	3	0.137	0.252	0.587	0.197	0.879
MFGE8	SBP	Inverse variance weighted	2	0.175	0.113	0.122	-	0.418	No	2	0.175	0.113	0.122	-	0.418
MGLL	SBP	Wald ratio	1	1.386	0.612	0.024	-	0.158	No	1	1.386	0.612	0.024	-	0.158
MGMT	SBP	Inverse variance weighted	5	-0.054	0.133	0.685	0.105	0.922	No	5	-0.054	0.133	0.685	0.105	0.922
MIA	SBP	Inverse variance weighted	5	0.036	0.054	0.510	0.427	0.844	No	5	0.036	0.054	0.510	0.427	0.844
MICALL2	SBP	Inverse variance weighted	2	0.121	0.731	0.869	-	0.971	No	2	0.121	0.731	0.869	-	0.971
MIF	SBP	Wald ratio	1	-0.844	0.362	0.020	-	0.140	No	1	-0.844	0.362	0.020	-	0.140
MILR1	SBP	Inverse variance weighted	23	-0.056	0.113	0.622	0.003	0.895	No	23	-0.056	0.113	0.622	0.003	0.895
MINDY1	SBP	Inverse variance weighted	2	0.477	0.229	0.037	-	0.217	No	2	0.477	0.229	0.037	-	0.217
MINK1	SBP	Wald ratio	1	-0.411	0.771	0.595	-	0.880	No	1	-0.411	0.771	0.595	-	0.880
MITD1	SBP	Wald ratio	1	-0.524	0.565	0.354	-	0.737	No	1	-0.524	0.565	0.354	-	0.737
MLN	SBP	Inverse variance weighted	4	0.099	0.175	0.572	0.011	0.874	No	4	0.099	0.175	0.572	0.011	0.874
MME	SBP	Wald ratio	1	1.092	0.720	0.129	-	0.433	No	1	1.092	0.720	0.129	-	0.433
MMP1	SBP	Inverse variance weighted	10	0.019	0.096	0.841	0.162	0.967	No	10	0.019	0.096	0.841	0.162	0.967
MMP10	SBP	Inverse variance weighted	6	0.114	0.159	0.475	0.080	0.826	No	6	0.114	0.159	0.475	0.080	0.826
MMP12	SBP	Inverse variance weighted	7	-0.107	0.079	0.175	0.591	0.522	No	7	-0.107	0.079	0.175	0.591	0.522
MMP13	SBP	Wald ratio	1	-1.213	0.952	0.202	-	0.572	No	1	-1.213	0.952	0.202	-	0.572
MMP3	SBP	Inverse variance weighted	7	-0.178	0.427	0.677	0.052	0.920	No	7	-0.178	0.427	0.677	0.052	0.920
MMP7	SBP	Inverse variance weighted	2	0.043	0.293	0.884	-	0.974	No	2	0.043	0.293	0.884	-	0.974
MMP8	SBP	Inverse variance weighted	15	-0.008	0.103	0.937	0.189	0.984	No	15	-0.008	0.103	0.937	0.189	0.984
MMP9	SBP	Wald ratio	1	0.030	0.287	0.917	-	0.982	No	1	0.030	0.287	0.917	-	0.982
MMUT	SBP	Wald ratio	1	0.957	0.411	0.020	-	0.140	No	1	0.957	0.411	0.020	-	0.140
MNDA	SBP	Wald ratio	1	-0.174	1.685	0.918	-	0.982	No	1	-0.174	1.685	0.918	-	0.982
MOCS2	SBP	Inverse variance weighted	7	0.005	0.182	0.978	0.000	0.996	No	7	0.005	0.182	0.978	0.000	0.996
MPHOSPH8	SBP	Wald ratio	1	2.060	0.664	0.002	-	0.025	Yes	1	2.060	0.664	0.002	-	0.025
MPI	SBP	Inverse variance weighted	3	-1.554	0.281	0.000	0.101	0.000	Yes	3	-1.554	0.281	0.000	0.101	0.000
MPO	SBP	Inverse variance weighted	4	0.073	0.142	0.607	0.410	0.887	No	4	0.073	0.142	0.607	0.410	0.887
MRC1	SBP	Inverse variance weighted	5	-0.171	0.402	0.670	0.000	0.918	No	5	-0.171	0.402	0.670	0.000	0.918
MRI1	SBP	Inverse variance weighted	5	-0.076	0.247	0.758	0.912	0.950	No	5	-0.076	0.247	0.758	0.912	0.950
MRPL28	SBP	Wald ratio	1	-1.701	1.061	0.109	-	0.392	No	1	-1.701	1.061	0.109	-	0.392
MSLN	SBP	Inverse variance weighted	8	-0.024	0.118	0.840	0.003	0.967	No	8	-0.024	0.118	0.840	0.003	0.967
MSMB	SBP	Inverse variance weighted	8	-0.032	0.038	0.395	0.475	0.772	No	8	-0.032	0.038	0.395	0.475	0.772
MSR1	SBP	Inverse variance weighted	3	0.204	0.105	0.052	0.362	0.263	No	3	0.204	0.105	0.052	0.362	0.263
MSRA	SBP	Wald ratio	1	-5.791	0.720	0.000	-	0.000	Yes	1	-5.791	0.720	0.000	-	0.000
MST1	SBP	Inverse variance weighted	2	0.257	0.088	0.004	-	0.041	Yes	2	0.257	0.088	0.004	-	0.041
MSTN	SBP	Wald ratio	1	2.203	0.825	0.008	-	0.073	No	1	2.203	0.825	0.008	-	0.073

ST2; MR causal estimates for plasma proteins on systolic blood pressure.

Causal candidates prioritized for SBP were marked as "Yes" in column "Prioritized". Effect of plasma protein levels on blood pressure is in mmHg unit.

										Steiger filtering					
Exposure	Outocme	Method	nsnp	Beta	SE	P-value	Cochran's Q P-value	FDR-corrected P-value	Prioritized	nsnp	Beta	SE	P-value	Cochran's Q P-value	FDR-corrected P-value
MTHFD2	SBP	Wald ratio	1	0.259	0.496	0.601	-	0.884	No	1	0.259	0.496	0.601	-	0.884
MTHFSD	SBP	Inverse variance weighted	2	0.162	0.107	0.132	-	0.437	No	2	0.162	0.107	0.132	-	0.437
MTIF3	SBP	Inverse variance weighted	2	-0.314	0.389	0.420	-	0.790	No	2	-0.314	0.389	0.420	-	0.790
MTR	SBP	Wald ratio	1	0.143	1.085	0.895	-	0.977	No	1	0.143	1.085	0.895	-	0.977
MTSS1	SBP	Inverse variance weighted	2	0.853	0.669	0.203	-	0.572	No	2	0.853	0.669	0.203	-	0.572
MTSS2	SBP	Inverse variance weighted	2	-0.240	0.565	0.671	-	0.918	No	2	-0.240	0.565	0.671	-	0.918
MTUS1	SBP	Inverse variance weighted	3	-0.379	0.227	0.094	0.264	0.364	No	3	-0.379	0.227	0.094	0.264	0.364
MUC13	SBP	Inverse variance weighted	4	0.257	0.177	0.146	0.464	0.468	No	4	0.257	0.177	0.146	0.464	0.468
MUC16	SBP	Wald ratio	1	-1.424	0.635	0.025	-	0.164	No	1	-1.424	0.635	0.025	-	0.164
MUC2	SBP	Inverse variance weighted	2	-0.150	0.169	0.374	-	0.756	No	2	-0.150	0.169	0.374	-	0.756
MVK	SBP	Wald ratio	1	-1.613	0.507	0.001	-	0.020	Yes	1	-1.613	0.507	0.001	-	0.020
MXRA8	SBP	Wald ratio	1	4.588	0.859	0.000	-	0.000	Yes	1	4.588	0.859	0.000	-	0.000
MYBPC2	SBP	Wald ratio	1	-0.041	0.808	0.959	-	0.991	No	1	-0.041	0.808	0.959	-	0.991
MYDGF	SBP	Wald ratio	1	0.386	0.479	0.420	-	0.790	No	1	0.386	0.479	0.420	-	0.790
MYO9B	SBP	Inverse variance weighted	2	3.513	1.294	0.007	-	0.066	No	2	3.513	1.294	0.007	-	0.066
MYOC	SBP	Inverse variance weighted	3	0.125	0.140	0.373	0.418	0.755	No	3	0.125	0.140	0.373	0.418	0.755
MYOM2	SBP	Inverse variance weighted	2	0.860	0.723	0.234	-	0.617	No	2	0.860	0.723	0.234	-	0.617
MYOM3	SBP	Wald ratio	1	0.508	0.557	0.362	-	0.746	No	1	0.508	0.557	0.362	-	0.746
MZB1	SBP	Wald ratio	1	-0.499	0.423	0.238	-	0.620	No	1	-0.499	0.423	0.238	-	0.620
NAA80	SBP	Wald ratio	1	1.155	0.481	0.016	-	0.123	No	1	1.155	0.481	0.016	-	0.123
NAAA	SBP	Inverse variance weighted	9	-0.028	0.070	0.692	0.351	0.924	No	9	-0.028	0.070	0.692	0.351	0.924
NADK	SBP	Wald ratio	1	-1.277	0.157	0.000	-	0.000	Yes	1	-1.277	0.157	0.000	-	0.000
NAGA	SBP	Inverse variance weighted	2	1.129	0.377	0.003	-	0.034	Yes	2	1.129	0.377	0.003	-	0.034
NAGK	SBP	Inverse variance weighted	2	-0.007	0.184	0.971	-	0.994	No	2	-0.007	0.184	0.971	-	0.994
NAGPA	SBP	Inverse variance weighted	3	0.046	0.300	0.877	0.002	0.974	No	3	0.046	0.300	0.877	0.002	0.974
NAMPT	SBP	Wald ratio	1	1.336	0.977	0.171	-	0.514	No	1	1.336	0.977	0.171	-	0.514
NAP1L4	SBP	Wald ratio	1	-0.288	0.187	0.122	-	0.418	No	1	-0.288	0.187	0.122	-	0.418
NAPRT	SBP	Inverse variance weighted	3	0.034	0.194	0.860	0.083	0.969	No	3	0.034	0.194	0.860	0.083	0.969
NBL1	SBP	Inverse variance weighted	2	0.004	0.661	0.995	-	0.998	No	2	0.004	0.661	0.995	-	0.998
NBN	SBP	Wald ratio	1	-3.508	0.992	0.000	-	0.008	Yes	1	-3.508	0.992	0.000	-	0.008
NCAM1	SBP	Inverse variance weighted	6	0.377	0.139	0.007	0.177	0.065	No	6	0.377	0.139	0.007	0.177	0.065
NCAM2	SBP	Inverse variance weighted	8	0.094	0.067	0.162	0.559	0.498	No	8	0.094	0.067	0.162	0.559	0.498
NCAN	SBP	Inverse variance weighted	3	-0.914	0.366	0.013	0.128	0.106	No	3	-0.914	0.366	0.013	0.128	0.106
NCF2	SBP	Wald ratio	1	-0.301	0.364	0.408	-	0.785	No	1	-0.301	0.364	0.408	-	0.785
NCR1	SBP	Inverse variance weighted	3	-0.038	0.159	0.810	0.817	0.961	No	3	-0.038	0.159	0.810	0.817	0.961
NCR3LG1	SBP	Inverse variance weighted	4	-0.863	0.278	0.002	0.001	0.025	No	4	-0.863	0.278	0.002	0.001	0.025
NCS1	SBP	Inverse variance weighted	3	-0.686	0.336	0.041	0.336	0.228	No	3	-0.686	0.336	0.041	0.336	0.228
NECTIN2	SBP	Inverse variance weighted	3	0.431	0.225	0.056	0.420	0.275	No	3	0.431	0.225	0.056	0.420	0.275
NECTIN4	SBP	Wald ratio	1	0.270	0.142	0.057	-	0.282	No	1	0.270	0.142	0.057	-	0.282
NELL1	SBP	Inverse variance weighted	9	0.197	0.109	0.070	0.852	0.309	No	9	0.197	0.109	0.070	0.852	0.309
NELL2	SBP	Inverse variance weighted	2	-0.132	0.210	0.528	-	0.855	No	2	-0.132	0.210	0.528	-	0.855
NEO1	SBP	Wald ratio	1	0.240	0.599	0.689	-	0.922	No	1	0.240	0.599	0.689	-	0.922
NEXN	SBP	Wald ratio	1	1.342	0.523	0.010	-	0.090	No	1	1.342	0.523	0.010	-	0.090
NFASC	SBP	Inverse variance weighted	7	-0.278	0.168	0.098	0.014	0.371	No	7	-0.278	0.168	0.098	0.014	0.371
NFATC1	SBP	Inverse variance weighted	2	-0.401	0.289	0.166	-	0.502	No	2	-0.401	0.289	0.166	-	0.502
NFE2	SBP	Wald ratio	1	-2.492	0.600	0.000	-	0.001	Yes	1	-2.492	0.600	0.000	-	0.001
NFKB1	SBP	Wald ratio	1	0.300	0.347	0.387	-	0.768	No	1	0.300	0.347	0.387	-	0.768
NFKBIE	SBP	Wald ratio	1	0.009	0.184	0.959	-	0.991	No	1	0.009	0.184	0.959	-	0.991
NFU1	SBP	Wald ratio	1	-0.962	0.332	0.004	-	0.043	Yes	1	-0.962	0.332	0.004	-	0.043
NGF	SBP	Wald ratio	1	-3.694	1.110	0.001	-	0.014	Yes	1	-3.694	1.110	0.001	-	0.014
NHLRC3	SBP	Inverse variance weighted	8	0.013	0.137	0.925	0.776	0.983	No	8	0.013	0.137	0.925	0.776	0.983
NID1	SBP	Inverse variance weighted	8	-0.040	0.208	0.847	0.830	0.967	No	8	-0.040	0.208	0.847	0.830	0.967
NID2	SBP	Inverse variance weighted	5	0.043	0.068	0.529	0.612	0.855	No	5	0.043	0.068	0.529	0.612	0.855
NINJ1	SBP	Inverse variance weighted	2	0.119	0.556	0.830	-	0.966	No	2	0.119	0.556	0.830	-	0.966
NIT1	SBP	Wald ratio	1	-0.312	0.170	0.066	-	0.299	No	1	-0.312	0.170	0.066	-	0.299
NIT2	SBP	Wald ratio	1	-1.360	0.561	0.015	-	0.118	No	1	-1.360	0.561	0.015	-	0.118

ST2; MR causal estimates for plasma proteins on systolic blood pressure.

Causal candidates prioritized for SBP were marked as "Yes" in column "Prioritized". Effect of plasma protein levels on blood pressure is in mmHg unit.

										Steiger filtering					
Exposure	Outocme	Method	nsnp	Beta	SE	P-value	Cochran's Q P-value	FDR-corrected P-value	Prioritized	nsnp	Beta	SE	P-value	Cochran's Q P-value	FDR-corrected P-value
NME3	SBP	Inverse variance weighted	3	-0.390	0.256	0.128	0.524	0.432	No	3	-0.390	0.256	0.128	0.524	0.432
NMI	SBP	Wald ratio	1	0.134	0.088	0.129	-	0.433	No	1	0.134	0.088	0.129	-	0.433
NM-T1	SBP	Inverse variance weighted	2	-0.109	0.296	0.712	-	0.933	No	2	-0.109	0.296	0.712	-	0.933
NOMO1	SBP	Wald ratio	1	0.848	0.409	0.038	-	0.221	No	1	0.848	0.409	0.038	-	0.221
NOS1	SBP	Inverse variance weighted	3	0.231	0.282	0.412	0.882	0.785	No	3	0.231	0.282	0.412	0.882	0.785
NOS2	SBP	Inverse variance weighted	2	-0.327	0.392	0.404	-	0.783	No	2	-0.327	0.392	0.404	-	0.783
NOS3	SBP	Wald ratio	1	-0.375	0.849	0.659	-	0.913	No	1	-0.375	0.849	0.659	-	0.913
NOTCH1	SBP	Inverse variance weighted	2	-1.190	0.651	0.068	-	0.302	No	2	-1.190	0.651	0.068	-	0.302
NOTCH2	SBP	Inverse variance weighted	3	-0.882	0.481	0.067	0.114	0.301	No	3	-0.882	0.481	0.067	0.114	0.301
NOTCH3	SBP	Inverse variance weighted	4	1.485	0.384	0.000	0.444	0.003	Yes	4	1.485	0.384	0.000	0.444	0.003
NPC2	SBP	Inverse variance weighted	3	-0.018	0.223	0.937	0.726	0.984	No	3	-0.018	0.223	0.937	0.726	0.984
NPHS1	SBP	Inverse variance weighted	7	0.002	0.109	0.987	0.138	0.997	No	7	0.002	0.109	0.987	0.138	0.997
NPL	SBP	Inverse variance weighted	3	0.173	0.278	0.533	0.034	0.858	No	3	0.173	0.278	0.533	0.034	0.858
NPPB	SBP	Wald ratio	1	-2.838	0.223	0.000	-	0.000	Yes	1	-2.838	0.223	0.000	-	0.000
NPPC	SBP	Inverse variance weighted	2	-0.632	0.715	0.377	-	0.758	No	2	-0.632	0.715	0.377	-	0.758
NPTX1	SBP	Inverse variance weighted	6	-0.228	0.082	0.005	0.352	0.057	No	6	-0.228	0.082	0.005	0.352	0.057
NPTXR	SBP	Inverse variance weighted	4	0.010	0.142	0.943	0.007	0.984	No	4	0.010	0.142	0.943	0.007	0.984
NPY	SBP	Wald ratio	1	-0.514	0.815	0.528	-	0.855	No	1	-0.514	0.815	0.528	-	0.855
NRCAM	SBP	Inverse variance weighted	3	0.044	0.140	0.751	0.990	0.946	No	3	0.044	0.140	0.751	0.990	0.946
NRP1	SBP	Inverse variance weighted	6	-0.119	0.193	0.538	0.037	0.858	No	6	-0.119	0.193	0.538	0.037	0.858
NRP2	SBP	Inverse variance weighted	5	-0.017	0.174	0.921	0.736	0.982	No	5	-0.017	0.174	0.921	0.736	0.982
NRTN	SBP	Wald ratio	1	-0.538	0.649	0.407	-	0.784	No	1	-0.538	0.649	0.407	-	0.784
NSFL1C	SBP	Inverse variance weighted	2	-0.143	0.461	0.756	-	0.950	No	2	-0.143	0.461	0.756	-	0.950
NT5C	SBP	Inverse variance weighted	4	-0.047	0.076	0.538	0.349	0.858	No	4	-0.047	0.076	0.538	0.349	0.858
NT5C3A	SBP	Wald ratio	1	0.356	0.373	0.341	-	0.725	No	1	0.356	0.373	0.341	-	0.725
NT5E	SBP	Inverse variance weighted	5	0.232	0.115	0.044	0.023	0.239	No	5	0.232	0.115	0.044	0.023	0.239
NTF3	SBP	Inverse variance weighted	4	0.696	0.337	0.039	0.083	0.224	No	4	0.696	0.337	0.039	0.083	0.224
NTF4	SBP	Wald ratio	1	-1.438	1.034	0.164	-	0.501	No	1	-1.438	1.034	0.164	-	0.501
NTRK2	SBP	Inverse variance weighted	2	-0.700	0.706	0.321	-	0.703	No	2	-0.700	0.706	0.321	-	0.703
NTRK3	SBP	Inverse variance weighted	2	-0.602	0.196	0.002	-	0.027	Yes	2	-0.602	0.196	0.002	-	0.027
NUB1	SBP	Inverse variance weighted	2	-0.560	0.422	0.184	-	0.539	No	2	-0.560	0.422	0.184	-	0.539
NUCB2	SBP	Inverse variance weighted	2	-0.707	0.155	0.000	-	0.000	Yes	2	-0.707	0.155	0.000	-	0.000
NUDT15	SBP	Wald ratio	1	1.078	1.140	0.344	-	0.728	No	1	1.078	1.140	0.344	-	0.728
NUDT16	SBP	Wald ratio	1	-0.095	0.155	0.541	-	0.858	No	1	-0.095	0.155	0.541	-	0.858
NUDT2	SBP	Inverse variance weighted	3	-0.492	0.212	0.020	0.142	0.142	No	3	-0.492	0.212	0.020	0.142	0.142
NUDT5	SBP	Wald ratio	1	2.452	0.705	0.001	-	0.009	Yes	1	2.452	0.705	0.001	-	0.009
NUMB	SBP	Wald ratio	1	1.803	0.464	0.000	-	0.003	Yes	1	1.803	0.464	0.000	-	0.003
NXPH3	SBP	Inverse variance weighted	3	-0.082	0.211	0.697	0.867	0.925	No	3	-0.082	0.211	0.697	0.867	0.925
OBP2B	SBP	Inverse variance weighted	6	0.090	0.070	0.195	0.886	0.556	No	6	0.090	0.070	0.195	0.886	0.556
OCLN	SBP	Inverse variance weighted	2	-0.700	0.420	0.096	-	0.367	No	2	-0.700	0.420	0.096	-	0.367
ODAM	SBP	Inverse variance weighted	10	-0.044	0.127	0.732	0.853	0.943	No	10	-0.044	0.127	0.732	0.853	0.943
OGA	SBP	Wald ratio	1	-1.613	0.351	0.000	-	0.000	Yes	1	-1.613	0.351	0.000	-	0.000
OGFR	SBP	Inverse variance weighted	3	0.205	0.547	0.708	0.165	0.931	No	3	0.205	0.547	0.708	0.165	0.931
OGN	SBP	Inverse variance weighted	3	-0.275	0.135	0.043	0.023	0.232	No	3	-0.275	0.135	0.043	0.023	0.232
OLFM4	SBP	Inverse variance weighted	9	0.038	0.064	0.549	0.103	0.862	No	9	0.038	0.064	0.549	0.103	0.862
OLR1	SBP	Inverse variance weighted	2	0.761	0.547	0.164	-	0.501	No	2	0.761	0.547	0.164	-	0.501
OMD	SBP	Inverse variance weighted	2	-0.533	2.836	0.851	-	0.967	No	2	-0.533	2.836	0.851	-	0.967
OMG	SBP	Wald ratio	1	-0.877	0.446	0.049	-	0.256	No	1	-0.877	0.446	0.049	-	0.256
OMP	SBP	Wald ratio	1	-0.116	0.238	0.625	-	0.897	No	1	-0.116	0.238	0.625	-	0.897
OPLAH	SBP	Wald ratio	1	-0.840	0.220	0.000	-	0.003	Yes	1	-0.840	0.220	0.000	-	0.003
OPTC	SBP	Inverse variance weighted	4	0.397	0.261	0.128	0.254	0.433	No	4	0.397	0.261	0.128	0.254	0.433
ORM1	SBP	Inverse variance weighted	9	0.060	0.114	0.600	0.203	0.884	No	9	0.060	0.114	0.600	0.203	0.884
OSCAR	SBP	Inverse variance weighted	6	-0.106	0.080	0.184	0.340	0.539	No	6	-0.106	0.080	0.184	0.340	0.539
OSM	SBP	Wald ratio	1	0.371	0.632	0.557	-	0.865	No	1	0.371	0.632	0.557	-	0.865
OSMR	SBP	Inverse variance weighted	9	-0.117	0.067	0.082	0.411	0.335	No	9	-0.117	0.067	0.082	0.411	0.335
OSTN	SBP	Inverse variance weighted	2	-0.285	0.482	0.554	-	0.865	No	2	-0.285	0.482	0.554	-	0.865

ST2; MR causal estimates for plasma proteins on systolic blood pressure.

Causal candidates prioritized for SBP were marked as "Yes" in column "Prioritized". Effect of plasma protein levels on blood pressure is in mmHg unit.

										Steiger filtering					
Exposure	Outocme	Method	nsnp	Beta	SE	P-value	Cochran's Q P-value	FDR-corrected P-value	Prioritized	nsnp	Beta	SE	P-value	Cochran's Q P-value	FDR-corrected P-value
OTOA	SBP	Wald ratio	1	-1.327	0.743	0.074	-	0.318	No	1	-1.327	0.743	0.074	-	0.318
OTUD6B	SBP	Wald ratio	1	-5.367	1.038	0.000	-	0.000	Yes	1	-5.367	1.038	0.000	-	0.000
OXCT1	SBP	Wald ratio	1	0.100	0.864	0.907	-	0.980	No	1	0.100	0.864	0.907	-	0.980
OXT	SBP	Inverse variance weighted	5	-0.016	0.111	0.888	0.050	0.975	No	5	-0.016	0.111	0.888	0.050	0.975
P4HB	SBP	Inverse variance weighted	2	-0.254	0.774	0.743	-	0.945	No	2	-0.254	0.774	0.743	-	0.945
PACS2	SBP	Wald ratio	1	0.203	0.247	0.412	-	0.785	No	1	0.203	0.247	0.412	-	0.785
PADI2	SBP	Wald ratio	1	0.024	0.317	0.940	-	0.984	No	1	0.024	0.317	0.940	-	0.984
PADI4	SBP	Inverse variance weighted	3	-0.085	0.152	0.575	0.922	0.875	No	3	-0.085	0.152	0.575	0.922	0.875
PAEP	SBP	Inverse variance weighted	6	0.012	0.081	0.880	0.064	0.974	No	6	0.012	0.081	0.880	0.064	0.974
PAFAH2	SBP	Inverse variance weighted	2	-2.034	1.017	0.046	-	0.243	No	2	-2.034	1.017	0.046	-	0.243
PAG1	SBP	Inverse variance weighted	2	-0.134	0.642	0.835	-	0.966	No	2	-0.134	0.642	0.835	-	0.966
PALM	SBP	Inverse variance weighted	3	-0.040	0.450	0.930	0.028	0.983	No	3	-0.040	0.450	0.930	0.028	0.983
PALM2	SBP	Inverse variance weighted	4	0.346	0.259	0.182	0.165	0.538	No	4	0.346	0.259	0.182	0.165	0.538
PAM	SBP	Inverse variance weighted	16	-0.070	0.061	0.249	0.502	0.630	No	16	-0.070	0.061	0.249	0.502	0.630
PAMR1	SBP	Inverse variance weighted	4	0.088	0.118	0.457	0.158	0.813	No	4	0.088	0.118	0.457	0.158	0.813
PAPPA	SBP	Inverse variance weighted	2	-0.504	1.553	0.745	-	0.946	No	2	-0.504	1.553	0.745	-	0.946
PARD3	SBP	Wald ratio	1	0.366	0.623	0.557	-	0.865	No	1	0.366	0.623	0.557	-	0.865
PARK7	SBP	Wald ratio	1	0.103	0.254	0.685	-	0.922	No	1	0.103	0.254	0.685	-	0.922
PARP1	SBP	Wald ratio	1	0.788	0.351	0.025	-	0.164	No	1	0.788	0.351	0.025	-	0.164
PBK	SBP	Wald ratio	1	0.568	0.864	0.511	-	0.845	No	1	0.568	0.864	0.511	-	0.845
PBLD	SBP	Inverse variance weighted	2	0.202	0.080	0.012	-	0.104	No	2	0.202	0.080	0.012	-	0.104
PBXIP1	SBP	Wald ratio	1	-1.049	0.824	0.203	-	0.572	No	1	-1.049	0.824	0.203	-	0.572
PCBD1	SBP	Wald ratio	1	-0.146	0.221	0.508	-	0.844	No	1	-0.146	0.221	0.508	-	0.844
PCBP2	SBP	Wald ratio	1	-3.408	0.821	0.000	-	0.001	Yes	1	-3.408	0.821	0.000	-	0.001
PCDH1	SBP	Wald ratio	1	-0.623	0.512	0.224	-	0.601	No	1	-0.623	0.512	0.224	-	0.601
PCDH12	SBP	Inverse variance weighted	4	-0.191	0.170	0.260	0.099	0.642	No	4	-0.191	0.170	0.260	0.099	0.642
PCDH17	SBP	Inverse variance weighted	3	-0.293	0.763	0.701	0.029	0.926	No	3	-0.293	0.763	0.701	0.029	0.926
PCDH7	SBP	Wald ratio	1	0.365	0.369	0.324	-	0.705	No	1	0.365	0.369	0.324	-	0.705
PCDH9	SBP	Inverse variance weighted	5	0.224	0.134	0.095	0.667	0.367	No	5	0.224	0.134	0.095	0.667	0.367
PCDHB15	SBP	Inverse variance weighted	7	0.197	0.140	0.160	0.076	0.496	No	7	0.197	0.140	0.160	0.076	0.496
PCOLCE	SBP	Wald ratio	1	0.337	0.241	0.163	-	0.499	No	1	0.337	0.241	0.163	-	0.499
PCSK7	SBP	Inverse variance weighted	2	-0.419	0.125	0.001	-	0.012	Yes	2	-0.419	0.125	0.001	-	0.012
PCSK9	SBP	Inverse variance weighted	3	-0.013	0.120	0.913	0.364	0.982	No	3	-0.013	0.120	0.913	0.364	0.982
PDCD1	SBP	Inverse variance weighted	2	0.426	0.163	0.009	-	0.081	No	2	0.426	0.163	0.009	-	0.081
PDCD1LG2	SBP	Inverse variance weighted	11	0.023	0.066	0.730	0.744	0.943	No	11	0.023	0.066	0.730	0.744	0.943
PDCD5	SBP	Inverse variance weighted	4	-0.111	0.103	0.281	0.968	0.670	No	4	-0.111	0.103	0.281	0.968	0.670
PDCD6	SBP	Inverse variance weighted	7	-0.131	0.061	0.031	0.023	0.192	No	7	-0.131	0.061	0.031	0.023	0.192
PDE5A	SBP	Inverse variance weighted	2	-1.031	0.250	0.000	-	0.001	Yes	2	-1.031	0.250	0.000	-	0.001
PDGFA	SBP	Inverse variance weighted	3	-0.439	0.721	0.543	0.205	0.858	No	3	-0.439	0.721	0.543	0.205	0.858
PDGFC	SBP	Inverse variance weighted	2	-0.779	0.424	0.066	-	0.299	No	2	-0.779	0.424	0.066	-	0.299
PDGFRA	SBP	Inverse variance weighted	3	-0.525	0.145	0.000	0.944	0.006	Yes	3	-0.525	0.145	0.000	0.944	0.006
PDGFRB	SBP	Inverse variance weighted	13	0.374	0.265	0.158	0.004	0.492	No	13	0.374	0.265	0.158	0.004	0.492
PDIA3	SBP	Wald ratio	1	-1.659	0.795	0.037	-	0.217	No	1	-1.659	0.795	0.037	-	0.217
PDIA4	SBP	Wald ratio	1	0.276	0.462	0.550	-	0.863	No	1	0.276	0.462	0.550	-	0.863
PDIA5	SBP	Inverse variance weighted	2	-0.218	0.370	0.556	-	0.865	No	2	-0.218	0.370	0.556	-	0.865
PDLIM5	SBP	Wald ratio	1	1.344	0.982	0.171	-	0.512	No	1	1.344	0.982	0.171	-	0.512
PDLIM7	SBP	Wald ratio	1	1.832	1.005	0.068	-	0.304	No	1	1.832	1.005	0.068	-	0.304
PDZD2	SBP	Inverse variance weighted	3	0.085	0.163	0.602	0.517	0.885	No	3	0.085	0.163	0.602	0.517	0.885
PDZK1	SBP	Wald ratio	1	0.575	0.346	0.096	-	0.369	No	1	0.575	0.346	0.096	-	0.369
PEAR1	SBP	Inverse variance weighted	4	0.436	0.281	0.121	0.066	0.415	No	4	0.436	0.281	0.121	0.066	0.415
PEBP1	SBP	Inverse variance weighted	2	-0.112	0.360	0.756	-	0.950	No	2	-0.112	0.360	0.756	-	0.950
PECAM1	SBP	Wald ratio	1	-8.714	1.219	0.000	-	0.000	Yes	1	-8.714	1.219	0.000	-	0.000
PECR	SBP	Inverse variance weighted	2	-0.028	0.141	0.843	-	0.967	No	2	-0.028	0.141	0.843	-	0.967
PENK	SBP	Inverse variance weighted	4	0.146	0.252	0.562	0.008	0.868	No	4	0.146	0.252	0.562	0.008	0.868
PEPD	SBP	Inverse variance weighted	3	0.220	0.111	0.048	0.644	0.254	No	3	0.220	0.111	0.048	0.644	0.254
PER3	SBP	Inverse variance weighted	4	-0.148	0.265	0.576	0.000	0.876	No	4	-0.148	0.265	0.576	0.000	0.876

ST2; MR causal estimates for plasma proteins on systolic blood pressure.

Causal candidates prioritized for SBP were marked as "Yes" in column "Prioritized". Effect of plasma protein levels on blood pressure is in mmHg unit.

										Steiger filtering					
**Exposure**	**Outcome**	**Method**	**nsnp**	**Beta**	**SE**	**P-value**	**Cochran's Q P-value**	**FDR-corrected P-value**	**Prioritized**	**nsnp**	**Beta**	**SE**	**P-value**	**Cochran's Q P-value**	**FDR-corrected P-value**
PF4	SBP	Wald ratio	1	-0.004	0.516	0.994	-	0.997	No	1	-0.004	0.516	0.994	-	0.997
PFKFB2	SBP	Wald ratio	1	-0.658	0.212	0.002	-	0.025	Yes	1	-0.658	0.212	0.002	-	0.025
PGD	SBP	Wald ratio	1	-0.034	0.160	0.834	-	0.966	No	1	-0.034	0.160	0.834	-	0.966
PGF	SBP	Inverse variance weighted	2	-1.234	0.259	0.000	-	0.000	Yes	2	-1.234	0.259	0.000	-	0.000
PGLYRP1	SBP	Wald ratio	1	0.084	0.149	0.575	-	0.875	No	1	0.084	0.149	0.575	-	0.875
PGLYRP2	SBP	Inverse variance weighted	11	0.068	0.041	0.101	0.744	0.376	No	11	0.068	0.041	0.101	0.744	0.376
PGLYRP4	SBP	Inverse variance weighted	4	0.092	0.090	0.306	0.703	0.693	No	4	0.092	0.090	0.306	0.703	0.693
PHACTR2	SBP	Wald ratio	1	0.327	0.200	0.101	-	0.376	No	1	0.327	0.200	0.101	-	0.376
PHLDB1	SBP	Wald ratio	1	-2.593	0.906	0.004	-	0.046	Yes	1	-2.593	0.906	0.004	-	0.046
PHYKPL	SBP	Inverse variance weighted	7	-0.078	0.184	0.673	0.941	0.919	No	7	-0.078	0.184	0.673	0.941	0.919
PI16	SBP	Inverse variance weighted	6	-0.068	0.154	0.661	0.343	0.914	No	6	-0.068	0.154	0.661	0.343	0.914
PI3	SBP	Wald ratio	1	0.345	0.149	0.021	-	0.142	No	1	0.345	0.149	0.021	-	0.142
PIBF1	SBP	Wald ratio	1	1.372	0.673	0.041	-	0.229	No	1	1.372	0.673	0.041	-	0.229
PIGR	SBP	Inverse variance weighted	3	0.125	0.231	0.588	0.832	0.879	No	3	0.125	0.231	0.588	0.832	0.879
PIK3AP1	SBP	Inverse variance weighted	4	0.413	0.182	0.023	0.581	0.156	No	4	0.413	0.182	0.023	0.581	0.156
PIK3IP1	SBP	Inverse variance weighted	3	-0.085	0.149	0.567	0.635	0.871	No	3	-0.085	0.149	0.567	0.635	0.871
PIKFYVE	SBP	Wald ratio	1	-2.117	0.966	0.028	-	0.182	No	1	-2.117	0.966	0.028	-	0.182
PILRA	SBP	Inverse variance weighted	8	0.031	0.043	0.471	0.415	0.821	No	8	0.031	0.043	0.471	0.415	0.821
PILRB	SBP	Inverse variance weighted	11	0.028	0.037	0.456	0.653	0.812	No	11	0.028	0.037	0.456	0.653	0.812
PINLYP	SBP	Inverse variance weighted	5	0.032	0.070	0.644	0.114	0.904	No	5	0.032	0.070	0.644	0.114	0.904
PKD1	SBP	Inverse variance weighted	5	-0.748	0.259	0.004	0.019	0.043	Yes	5	-0.748	0.259	0.004	0.019	0.043
PKD2	SBP	Wald ratio	1	-0.215	0.442	0.626	-	0.897	No	1	-0.215	0.442	0.626	-	0.897
PKLR	SBP	Inverse variance weighted	2	0.050	0.470	0.916	-	0.982	No	2	0.050	0.470	0.916	-	0.982
PKN3	SBP	Wald ratio	1	-0.702	0.271	0.009	-	0.085	No	1	-0.702	0.271	0.009	-	0.085
PLA2G10	SBP	Inverse variance weighted	2	0.172	0.325	0.595	-	0.880	No	2	0.172	0.325	0.595	-	0.880
PLA2G15	SBP	Inverse variance weighted	3	0.351	0.176	0.045	0.324	0.243	No	3	0.351	0.176	0.045	0.324	0.243
PLA2G1B	SBP	Wald ratio	1	-3.250	0.808	0.000	-	0.002	Yes	1	-3.250	0.808	0.000	-	0.002
PLA2G2A	SBP	Inverse variance weighted	5	-0.080	0.067	0.232	0.372	0.615	No	5	-0.080	0.067	0.232	0.372	0.615
PLA2G4A	SBP	Inverse variance weighted	2	-0.573	0.832	0.491	-	0.837	No	2	-0.573	0.832	0.491	-	0.837
PLA2G7	SBP	Inverse variance weighted	2	0.358	0.970	0.712	-	0.933	No	2	0.358	0.970	0.712	-	0.933
PLAT	SBP	Wald ratio	1	0.394	0.438	0.368	-	0.751	No	1	0.394	0.438	0.368	-	0.751
PLAU	SBP	Inverse variance weighted	4	0.796	0.629	0.205	0.017	0.572	No	4	0.796	0.629	0.205	0.017	0.572
PLAUR	SBP	Inverse variance weighted	3	0.232	0.269	0.387	0.367	0.768	No	3	0.232	0.269	0.387	0.367	0.768
PLB1	SBP	Inverse variance weighted	10	-0.063	0.069	0.355	0.022	0.739	No	10	-0.063	0.069	0.355	0.022	0.739
PLCB2	SBP	Wald ratio	1	-0.027	0.285	0.925	-	0.983	No	1	-0.027	0.285	0.925	-	0.983
PLEKHO1	SBP	Wald ratio	1	0.352	0.816	0.666	-	0.916	No	1	0.352	0.816	0.666	-	0.916
PLG	SBP	Inverse variance weighted	18	0.072	0.106	0.498	0.076	0.839	No	18	0.072	0.106	0.498	0.076	0.839
PLIN3	SBP	Wald ratio	1	-0.980	0.549	0.074	-	0.318	No	1	-0.980	0.549	0.074	-	0.318
PLPBP	SBP	Wald ratio	1	-0.016	0.991	0.987	-	0.997	No	1	-0.016	0.991	0.987	-	0.997
PLSCR3	SBP	Wald ratio	1	0.841	0.713	0.238	-	0.620	No	1	0.841	0.713	0.238	-	0.620
PLTP	SBP	Inverse variance weighted	5	0.057	0.142	0.688	0.074	0.922	No	5	0.057	0.142	0.688	0.074	0.922
PLXDC1	SBP	Inverse variance weighted	16	0.125	0.107	0.243	0.656	0.625	No	16	0.125	0.107	0.243	0.656	0.625
PLXDC2	SBP	Inverse variance weighted	3	-0.587	0.584	0.315	0.119	0.696	No	3	-0.587	0.584	0.315	0.119	0.696
PLX-4	SBP	Wald ratio	1	-0.036	0.477	0.939	-	0.984	No	1	-0.036	0.477	0.939	-	0.984
PLXNB2	SBP	Inverse variance weighted	6	-0.078	0.076	0.301	0.116	0.689	No	6	-0.078	0.076	0.301	0.116	0.689
PM20D1	SBP	Inverse variance weighted	8	-0.012	0.048	0.797	0.595	0.958	No	8	-0.012	0.048	0.797	0.595	0.958
PMM2	SBP	Inverse variance weighted	5	0.286	0.195	0.142	0.331	0.458	No	5	0.286	0.195	0.142	0.331	0.458
PMS1	SBP	Wald ratio	1	3.344	0.886	0.000	-	0.003	Yes	1	3.344	0.886	0.000	-	0.003
PMVK	SBP	Wald ratio	1	-1.063	0.731	0.146	-	0.467	No	1	-1.063	0.731	0.146	-	0.467
PNLIP	SBP	Wald ratio	1	0.011	1.192	0.993	-	0.997	No	1	0.011	1.192	0.993	-	0.997
PNLIPRP1	SBP	Inverse variance weighted	2	-0.014	0.179	0.939	-	0.984	No	2	-0.014	0.179	0.939	-	0.984
PNLIPRP2	SBP	Inverse variance weighted	11	-0.008	0.036	0.815	0.377	0.963	No	11	-0.008	0.036	0.815	0.377	0.963
PNMA1	SBP	Inverse variance weighted	2	0.345	0.516	0.504	-	0.840	No	2	0.345	0.516	0.504	-	0.840
PODXL	SBP	Inverse variance weighted	2	0.097	0.481	0.841	-	0.967	No	2	0.097	0.481	0.841	-	0.967
PODXL2	SBP	Inverse variance weighted	3	-0.214	0.148	0.147	0.430	0.468	No	3	-0.214	0.148	0.147	0.430	0.468
POMC	SBP	Wald ratio	1	0.354	0.567	0.533	-	0.858	No	1	0.354	0.567	0.533	-	0.858

ST2; MR causal estimates for plasma proteins on systolic blood pressure.

Causal candidates prioritized for SBP were marked as "Yes" in column "Prioritized". Effect of plasma protein levels on blood pressure is in mmHg unit.

										Steiger filtering					
**Exposure**	**Outocme**	**Method**	**nsnp**	**Beta**	**SE**	**P-value**	**Cochran's Q P-value**	**FDR-corrected P-value**	**Prioritized**	**nsnp**	**Beta**	**SE**	**P-value**	**Cochran's Q P-value**	**FDR-corrected P-value**
PON1	SBP	Inverse variance weighted	10	0.042	0.044	0.344	0.762	0.728	No	10	0.042	0.044	0.344	0.762	0.728
PON2	SBP	Inverse variance weighted	10	-0.002	0.059	0.978	0.153	0.996	No	10	-0.002	0.059	0.978	0.153	0.996
PON3	SBP	Inverse variance weighted	16	-0.045	0.100	0.655	0.534	0.912	No	16	-0.045	0.100	0.655	0.534	0.912
POSTN	SBP	Inverse variance weighted	3	-0.060	0.213	0.778	0.167	0.953	No	3	-0.060	0.213	0.778	0.167	0.953
PPCDC	SBP	Inverse variance weighted	8	0.179	0.311	0.564	0.000	0.870	No	8	0.179	0.311	0.564	0.000	0.870
PPIE	SBP	Wald ratio	1	-1.059	0.796	0.183	-	0.539	No	1	-1.059	0.796	0.183	-	0.539
PPL	SBP	Inverse variance weighted	3	0.252	0.473	0.594	0.242	0.880	No	3	0.252	0.473	0.594	0.242	0.880
PPM1F	SBP	Wald ratio	1	1.076	0.740	0.146	-	0.467	No	1	1.076	0.740	0.146	-	0.467
PPME1	SBP	Wald ratio	1	2.173	0.886	0.014	-	0.113	No	1	2.173	0.886	0.014	-	0.113
PPP1R12A	SBP	Wald ratio	1	2.854	1.202	0.018	-	0.130	No	1	2.854	1.202	0.018	-	0.130
PPP1R14A	SBP	Inverse variance weighted	2	0.468	0.202	0.021	-	0.142	No	2	0.468	0.202	0.021	-	0.142
PPP1R14D	SBP	Wald ratio	1	2.677	0.750	0.000	-	0.007	Yes	1	2.677	0.750	0.000	-	0.007
PPP1R9B	SBP	Wald ratio	1	0.026	1.010	0.980	-	0.996	No	1	0.026	1.010	0.980	-	0.996
PPP2R5A	SBP	Wald ratio	1	0.095	0.441	0.830	-	0.966	No	1	0.095	0.441	0.830	-	0.966
PRAP1	SBP	Inverse variance weighted	5	-0.016	0.097	0.869	0.796	0.971	No	5	-0.016	0.097	0.869	0.796	0.971
PRCP	SBP	Inverse variance weighted	2	0.342	0.582	0.556	-	0.865	No	2	0.342	0.582	0.556	-	0.865
PRDX1	SBP	Wald ratio	1	-0.433	0.639	0.498	-	0.839	No	1	-0.433	0.639	0.498	-	0.839
PRDX2	SBP	Inverse variance weighted	2	0.126	0.210	0.549	-	0.862	No	2	0.126	0.210	0.549	-	0.862
PRDX3	SBP	Wald ratio	1	-0.194	0.654	0.766	-	0.950	No	1	-0.194	0.654	0.766	-	0.950
PRDX5	SBP	Inverse variance weighted	2	0.224	1.561	0.886	-	0.974	No	2	0.224	1.561	0.886	-	0.974
PRDX6	SBP	Inverse variance weighted	2	-0.284	0.289	0.325	-	0.706	No	2	-0.284	0.289	0.325	-	0.706
PRELP	SBP	Inverse variance weighted	4	-0.080	0.085	0.349	0.680	0.733	No	4	-0.080	0.085	0.349	0.680	0.733
PRG2	SBP	Wald ratio	1	1.153	0.403	0.004	-	0.046	Yes	1	1.153	0.403	0.004	-	0.046
PRG3	SBP	Inverse variance weighted	2	0.092	0.302	0.761	-	0.950	No	2	0.092	0.302	0.761	-	0.950
PRKAB1	SBP	Wald ratio	1	0.848	0.229	0.000	-	0.004	Yes	1	0.848	0.229	0.000	-	0.004
PRKAR2A	SBP	Wald ratio	1	2.055	0.831	0.013	-	0.111	No	1	2.055	0.831	0.013	-	0.111
PRKD2	SBP	Wald ratio	1	0.073	0.963	0.940	-	0.984	No	1	0.073	0.963	0.940	-	0.984
PRKG1	SBP	Inverse variance weighted	3	-0.669	0.546	0.221	0.001	0.597	No	3	-0.669	0.546	0.221	0.001	0.597
PRND	SBP	Inverse variance weighted	5	0.004	0.180	0.982	0.240	0.996	No	5	0.004	0.180	0.982	0.240	0.996
PROC	SBP	Inverse variance weighted	2	0.255	0.203	0.209	-	0.579	No	2	0.255	0.203	0.209	-	0.579
PROCR	SBP	Wald ratio	1	0.024	0.098	0.803	-	0.959	No	1	0.024	0.098	0.803	-	0.959
PROK1	SBP	Inverse variance weighted	6	0.059	0.099	0.553	0.514	0.865	No	6	0.059	0.099	0.553	0.514	0.865
PROS1	SBP	Inverse variance weighted	5	0.465	0.323	0.149	0.593	0.473	No	5	0.465	0.323	0.149	0.593	0.473
PRR4	SBP	Inverse variance weighted	6	0.104	0.051	0.043	0.672	0.232	No	6	0.104	0.051	0.043	0.672	0.232
PRRT3	SBP	Inverse variance weighted	3	-0.103	0.200	0.606	0.569	0.887	No	3	-0.103	0.200	0.606	0.569	0.887
PRSS2	SBP	Wald ratio	1	-0.503	0.291	0.084	-	0.338	No	1	-0.503	0.291	0.084	-	0.338
PRSS22	SBP	Inverse variance weighted	2	0.088	0.208	0.671	-	0.918	No	2	0.088	0.208	0.671	-	0.918
PRSS27	SBP	Inverse variance weighted	3	-0.449	0.200	0.025	0.748	0.165	No	3	-0.449	0.200	0.025	0.748	0.165
PRSS53	SBP	Inverse variance weighted	10	0.091	0.047	0.051	0.980	0.262	No	10	0.091	0.047	0.051	0.980	0.262
PRSS8	SBP	Inverse variance weighted	2	0.994	0.358	0.006	-	0.058	No	2	0.994	0.358	0.006	-	0.058
PRTFDC1	SBP	Inverse variance weighted	2	-0.058	0.197	0.767	-	0.950	No	2	-0.058	0.197	0.767	-	0.950
PRTG	SBP	Inverse variance weighted	6	-0.025	0.091	0.782	0.827	0.953	No	6	-0.025	0.091	0.782	0.827	0.953
PRTN3	SBP	Inverse variance weighted	7	-0.043	0.103	0.676	0.227	0.920	No	7	-0.043	0.103	0.676	0.227	0.920
PSAP	SBP	Inverse variance weighted	3	0.480	0.192	0.012	0.493	0.105	No	3	0.480	0.192	0.012	0.493	0.105
PSAPL1	SBP	Inverse variance weighted	5	0.042	0.079	0.595	0.295	0.880	No	5	0.042	0.079	0.595	0.295	0.880
PSCA	SBP	Inverse variance weighted	18	-0.027	0.038	0.482	0.201	0.831	No	18	-0.027	0.038	0.482	0.201	0.831
PSG1	SBP	Inverse variance weighted	10	-0.004	0.048	0.939	0.550	0.984	No	10	-0.004	0.048	0.939	0.550	0.984
PSMD5	SBP	Wald ratio	1	3.735	0.823	0.000	-	0.000	Yes	1	3.735	0.823	0.000	-	0.000
PSMD9	SBP	Wald ratio	1	-0.864	0.217	0.000	-	0.002	No	1	-0.864	0.217	0.000	-	0.002
PSME1	SBP	Wald ratio	1	-0.140	0.454	0.758	-	0.950	No	1	-0.140	0.454	0.758	-	0.950
PSME2	SBP	Wald ratio	1	-0.126	0.411	0.758	-	0.950	No	1	-0.126	0.411	0.758	-	0.950
PSMG4	SBP	Inverse variance weighted	2	-0.166	0.273	0.544	-	0.858	No	2	-0.166	0.273	0.544	-	0.858
PSRC1	SBP	Inverse variance weighted	2	-0.764	0.203	0.000	-	0.003	Yes	2	-0.764	0.203	0.000	-	0.003
PSTPIP2	SBP	Inverse variance weighted	2	-0.059	0.257	0.817	-	0.965	No	2	-0.059	0.257	0.817	-	0.965
PTGES2	SBP	Wald ratio	1	1.281	0.514	0.013	-	0.106	No	1	1.281	0.514	0.013	-	0.106
PTGR1	SBP	Inverse variance weighted	9	-0.063	0.123	0.605	0.068	0.887	No	9	-0.063	0.123	0.605	0.068	0.887

ST2; MR causal estimates for plasma proteins on systolic blood pressure.

Causal candidates prioritized for SBP were marked as "Yes" in column "Prioritized". Effect of plasma protein levels on blood pressure is in mmHg unit.

										Steiger filtering					
Exposure	Outocme	Method	nsnp	Beta	SE	P-value	Cochran's Q P-value	FDR-corrected P-value	Prioritized	nsnp	Beta	SE	P-value	Cochran's Q P-value	FDR-corrected P-value
PTH1R	SBP	Inverse variance weighted	9	-0.145	0.472	0.759	0.000	0.950	No	9	-0.145	0.472	0.759	0.000	0.950
PTK7	SBP	Wald ratio	1	1.399	0.694	0.044	-	0.237	No	1	1.399	0.694	0.044	-	0.237
PTN	SBP	Wald ratio	1	-0.201	0.558	0.719	-	0.938	No	1	-0.201	0.558	0.719	-	0.938
PTPRB	SBP	Inverse variance weighted	5	-0.047	0.105	0.658	0.265	0.913	No	5	-0.047	0.105	0.658	0.265	0.913
PTPRC	SBP	Inverse variance weighted	4	-0.214	0.255	0.401	0.145	0.779	No	4	-0.214	0.255	0.401	0.145	0.779
PTPRF	SBP	Wald ratio	1	1.397	0.508	0.006	-	0.061	No	1	1.397	0.508	0.006	-	0.061
PTPRH	SBP	Inverse variance weighted	6	0.071	0.063	0.258	0.474	0.641	No	6	0.071	0.063	0.258	0.474	0.641
PTPRK	SBP	Wald ratio	1	0.471	0.452	0.298	-	0.688	No	1	0.471	0.452	0.298	-	0.688
PTPRM	SBP	Inverse variance weighted	5	1.142	0.469	0.015	0.114	0.115	No	5	1.142	0.469	0.015	0.114	0.115
PTPRN2	SBP	Inverse variance weighted	2	0.609	0.443	0.169	-	0.510	No	2	0.609	0.443	0.169	-	0.510
PTPRS	SBP	Inverse variance weighted	9	-0.136	0.269	0.613	0.000	0.890	No	9	-0.136	0.269	0.613	0.000	0.890
PTPRZ1	SBP	Inverse variance weighted	2	-0.170	0.499	0.734	-	0.943	No	2	-0.170	0.499	0.734	-	0.943
PTRHD1	SBP	Wald ratio	1	5.759	1.066	0.000	-	0.000	Yes	1	5.759	1.066	0.000	-	0.000
PTS	SBP	Wald ratio	1	0.147	0.197	0.455	-	0.811	No	1	0.147	0.197	0.455	-	0.811
PTX3	SBP	Inverse variance weighted	2	0.604	0.546	0.268	-	0.653	No	2	0.604	0.546	0.268	-	0.653
PVALB	SBP	Inverse variance weighted	7	-0.014	0.050	0.780	0.805	0.953	No	7	-0.014	0.050	0.780	0.805	0.953
PVR	SBP	Inverse variance weighted	7	0.045	0.081	0.578	0.060	0.877	No	7	0.045	0.081	0.578	0.060	0.877
PXDNL	SBP	Inverse variance weighted	5	-0.097	0.195	0.621	0.206	0.894	No	5	-0.097	0.195	0.621	0.206	0.894
PXN	SBP	Inverse variance weighted	2	-0.189	0.152	0.215	-	0.588	No	2	-0.189	0.152	0.215	-	0.588
PYDC1	SBP	Inverse variance weighted	3	-0.161	0.139	0.246	0.884	0.628	No	3	-0.161	0.139	0.246	0.884	0.628
PYY	SBP	Inverse variance weighted	2	0.256	0.174	0.141	-	0.456	No	2	0.256	0.174	0.141	-	0.456
PZP	SBP	Inverse variance weighted	7	-0.197	0.111	0.076	0.880	0.323	No	7	-0.197	0.111	0.076	0.880	0.323
QDPR	SBP	Inverse variance weighted	2	0.031	0.616	0.959	-	0.991	No	2	0.031	0.616	0.959	-	0.991
QPCT	SBP	Inverse variance weighted	4	0.325	0.111	0.003	0.602	0.039	Yes	4	0.325	0.111	0.003	0.602	0.039
QSOX1	SBP	Inverse variance weighted	4	-0.059	0.102	0.562	0.049	0.868	No	4	-0.059	0.102	0.562	0.049	0.868
RAB11FIP3	SBP	Inverse variance weighted	2	1.563	0.611	0.011	-	0.092	No	2	1.563	0.611	0.011	-	0.092
RAB2B	SBP	Wald ratio	1	0.185	0.251	0.463	-	0.817	No	1	0.185	0.251	0.463	-	0.817
RAB44	SBP	Inverse variance weighted	2	-0.786	0.337	0.020	-	0.140	No	2	-0.786	0.337	0.020	-	0.140
RAB6A	SBP	Wald ratio	1	0.707	0.822	0.390	-	0.770	No	1	0.707	0.822	0.390	-	0.770
RABEP1	SBP	Wald ratio	1	-0.535	0.515	0.299	-	0.688	No	1	-0.535	0.515	0.299	-	0.688
RABEPK	SBP	Wald ratio	1	1.803	0.267	0.000	-	0.000	Yes	1	1.803	0.267	0.000	-	0.000
RABGAP1L	SBP	Wald ratio	1	-1.044	0.639	0.102	-	0.378	No	1	-1.044	0.639	0.102	-	0.378
RAD23B	SBP	Wald ratio	1	-1.093	0.698	0.117	-	0.408	No	1	-1.093	0.698	0.117	-	0.408
RALB	SBP	Wald ratio	1	0.242	0.357	0.498	-	0.839	No	1	0.242	0.357	0.498	-	0.839
RALY	SBP	Wald ratio	1	0.978	0.566	0.084	-	0.339	No	1	0.978	0.566	0.084	-	0.339
RANBP1	SBP	Wald ratio	1	-2.400	0.720	0.001	-	0.013	Yes	1	-2.400	0.720	0.001	-	0.013
RARRES1	SBP	Inverse variance weighted	5	0.283	0.086	0.001	0.592	0.014	Yes	5	0.283	0.086	0.001	0.592	0.014
RARRES2	SBP	Wald ratio	1	-0.625	0.209	0.003	-	0.034	Yes	1	-0.625	0.209	0.003	-	0.034
RASSF2	SBP	Inverse variance weighted	2	-0.101	0.352	0.774	-	0.952	No	2	-0.101	0.352	0.774	-	0.952
RBKS	SBP	Inverse variance weighted	4	0.034	0.158	0.830	0.169	0.966	No	4	0.034	0.158	0.830	0.169	0.966
RBM17	SBP	Wald ratio	1	-0.200	0.289	0.489	-	0.836	No	1	-0.200	0.289	0.489	-	0.836
RBM19	SBP	Wald ratio	1	-0.297	1.064	0.780	-	0.953	No	1	-0.297	1.064	0.780	-	0.953
RBP1	SBP	Wald ratio	1	0.203	0.420	0.629	-	0.897	No	1	0.203	0.420	0.629	-	0.897
RBP2	SBP	Wald ratio	1	1.082	0.582	0.063	-	0.292	No	1	1.082	0.582	0.063	-	0.292
RBP5	SBP	Inverse variance weighted	2	-0.280	0.133	0.035	-	0.212	No	2	-0.280	0.133	0.035	-	0.212
RBP7	SBP	Wald ratio	1	0.122	0.163	0.453	-	0.809	No	1	0.122	0.163	0.453	-	0.809
RBPMS2	SBP	Inverse variance weighted	2	0.934	1.715	0.586	-	0.879	No	2	0.934	1.715	0.586	-	0.879
RECK	SBP	Inverse variance weighted	5	-0.203	0.381	0.594	0.000	0.880	No	5	-0.203	0.381	0.594	0.000	0.880
REEP4	SBP	Inverse variance weighted	2	0.293	0.337	0.385	-	0.765	No	2	0.293	0.337	0.385	-	0.765
REG1A	SBP	Inverse variance weighted	3	-0.128	0.151	0.397	0.498	0.775	No	3	-0.128	0.151	0.397	0.498	0.775
REG1B	SBP	Inverse variance weighted	3	-0.083	0.142	0.559	0.226	0.866	No	3	-0.083	0.142	0.559	0.226	0.866
REG3A	SBP	Inverse variance weighted	6	-0.051	0.322	0.874	0.001	0.972	No	6	-0.051	0.322	0.874	0.001	0.972
REG3G	SBP	Inverse variance weighted	4	0.174	0.155	0.262	0.640	0.645	No	4	0.174	0.155	0.262	0.640	0.645
REG4	SBP	Inverse variance weighted	4	-0.179	0.390	0.646	0.002	0.904	No	4	-0.179	0.390	0.646	0.002	0.904
RELT	SBP	Inverse variance weighted	2	-0.554	0.131	0.000	-	0.001	Yes	2	-0.554	0.131	0.000	-	0.001
REN	SBP	Inverse variance weighted	3	0.178	0.321	0.578	0.536	0.877	No	3	0.178	0.321	0.578	0.536	0.877

ST2; MR causal estimates for plasma proteins on systolic blood pressure.

Causal candidates prioritized for SBP were marked as "Yes" in column "Prioritized". Effect of plasma protein levels on blood pressure is in mmHg unit.

										Steiger filtering					
**Exposure**	**Outocme**	**Method**	**nsnp**	**Beta**	**SE**	**P-value**	**Cochran's Q P-value**	**FDR-corrected P-value**	**Prioritized**	**nsnp**	**Beta**	**SE**	**P-value**	**Cochran's Q P-value**	**FDR-corrected P-value**
RET	SBP	Inverse variance weighted	4	0.095	0.107	0.372	0.535	0.755	No	4	0.095	0.107	0.372	0.535	0.755
RETN	SBP	Inverse variance weighted	7	-0.084	0.195	0.666	0.002	0.916	No	7	-0.084	0.195	0.666	0.002	0.916
RGCC	SBP	Wald ratio	1	1.183	0.969	0.222	-	0.600	No	1	1.183	0.969	0.222	-	0.600
RGMA	SBP	Inverse variance weighted	8	-0.165	0.150	0.273	0.233	0.660	No	8	-0.165	0.150	0.273	0.233	0.660
RGMB	SBP	Inverse variance weighted	4	-0.656	0.422	0.121	0.005	0.415	No	4	-0.656	0.422	0.121	0.005	0.415
RHOC	SBP	Wald ratio	1	0.028	0.525	0.958	-	0.991	No	1	0.028	0.525	0.958	-	0.991
RIDA	SBP	Inverse variance weighted	2	-0.319	0.327	0.329	-	0.711	No	2	-0.319	0.327	0.329	-	0.711
RILP	SBP	Inverse variance weighted	3	0.180	0.549	0.742	0.925	0.945	No	3	0.180	0.549	0.742	0.925	0.945
RILPL2	SBP	Inverse variance weighted	2	-0.364	0.456	0.424	-	0.790	No	2	-0.364	0.456	0.424	-	0.790
RLN2	SBP	Inverse variance weighted	6	0.047	0.324	0.886	0.097	0.974	No	6	0.047	0.324	0.886	0.097	0.974
R-SE1	SBP	Inverse variance weighted	4	0.156	0.193	0.418	0.612	0.789	No	4	0.156	0.193	0.418	0.612	0.789
R-SE10	SBP	Inverse variance weighted	10	0.053	0.058	0.356	0.186	0.740	No	10	0.053	0.058	0.356	0.186	0.740
R-SE3	SBP	Inverse variance weighted	6	0.015	0.144	0.918	0.938	0.982	No	6	0.015	0.144	0.918	0.938	0.982
R-SE4	SBP	Inverse variance weighted	4	-0.041	0.139	0.767	0.307	0.950	No	4	-0.041	0.139	0.767	0.307	0.950
R-SE6	SBP	Inverse variance weighted	3	0.007	0.096	0.940	0.929	0.984	No	3	0.007	0.096	0.940	0.929	0.984
R-SEH2A	SBP	Wald ratio	1	0.211	0.376	0.576	-	0.875	No	1	0.211	0.376	0.576	-	0.875
R-SET2	SBP	Inverse variance weighted	4	-0.115	0.105	0.274	0.287	0.661	No	4	-0.115	0.105	0.274	0.287	0.661
RNF149	SBP	Wald ratio	1	0.089	0.151	0.554	-	0.865	No	1	0.089	0.151	0.554	-	0.865
RNF43	SBP	Wald ratio	1	-0.143	0.362	0.693	-	0.924	No	1	-0.143	0.362	0.693	-	0.924
ROBO1	SBP	Wald ratio	1	0.156	0.209	0.456	-	0.811	No	1	0.156	0.209	0.456	-	0.811
ROBO4	SBP	Wald ratio	1	-0.286	0.428	0.503	-	0.840	No	1	-0.286	0.428	0.503	-	0.840
ROR1	SBP	Inverse variance weighted	6	-0.095	0.148	0.518	0.323	0.849	No	6	-0.095	0.148	0.518	0.323	0.849
RPA2	SBP	Wald ratio	1	-0.855	0.375	0.023	-	0.153	No	1	-0.855	0.375	0.023	-	0.153
RPE	SBP	Wald ratio	1	-1.807	0.758	0.017	-	0.128	No	1	-1.807	0.758	0.017	-	0.128
RPL14	SBP	Wald ratio	1	0.244	0.692	0.725	-	0.941	No	1	0.244	0.692	0.725	-	0.941
RRM2	SBP	Wald ratio	1	0.101	0.822	0.902	-	0.978	No	1	0.101	0.822	0.902	-	0.978
RRM2B	SBP	Inverse variance weighted	2	-0.026	0.735	0.972	-	0.994	No	2	-0.026	0.735	0.972	-	0.994
RSPO1	SBP	Inverse variance weighted	3	-0.103	0.258	0.689	0.089	0.922	No	3	-0.103	0.258	0.689	0.089	0.922
RSPO3	SBP	Wald ratio	1	-0.933	0.260	0.000	-	0.006	Yes	1	-0.933	0.260	0.000	-	0.006
RTBDN	SBP	Inverse variance weighted	2	-0.361	0.755	0.633	-	0.899	No	2	-0.361	0.755	0.633	-	0.899
RTN4IP1	SBP	Wald ratio	1	0.033	0.415	0.937	-	0.984	No	1	0.033	0.415	0.937	-	0.984
RTN4R	SBP	Inverse variance weighted	3	-0.258	0.133	0.052	0.299	0.263	No	3	-0.258	0.133	0.052	0.299	0.263
RWDD1	SBP	Wald ratio	1	0.347	0.355	0.328	-	0.709	No	1	0.347	0.355	0.328	-	0.709
S100A11	SBP	Wald ratio	1	-0.372	0.320	0.245	-	0.626	No	1	-0.372	0.320	0.245	-	0.626
S100A12	SBP	Inverse variance weighted	2	0.041	0.176	0.814	-	0.963	No	2	0.041	0.176	0.814	-	0.963
S100A13	SBP	Inverse variance weighted	5	0.137	0.168	0.413	0.511	0.785	No	5	0.137	0.168	0.413	0.511	0.785
S100A3	SBP	Wald ratio	1	0.264	0.874	0.763	-	0.950	No	1	0.264	0.874	0.763	-	0.950
S100A4	SBP	Inverse variance weighted	2	0.619	0.230	0.007	-	0.068	No	2	0.619	0.230	0.007	-	0.068
S100P	SBP	Inverse variance weighted	2	0.239	0.252	0.342	-	0.725	No	2	0.239	0.252	0.342	-	0.725
SAA4	SBP	Inverse variance weighted	10	0.108	0.135	0.423	0.068	0.790	No	10	0.108	0.135	0.423	0.068	0.790
SAMD9L	SBP	Inverse variance weighted	2	0.006	0.203	0.977	-	0.996	No	2	0.006	0.203	0.977	-	0.996
SARG	SBP	Wald ratio	1	-0.421	0.641	0.512	-	0.845	No	1	-0.421	0.641	0.512	-	0.845
SAT2	SBP	Inverse variance weighted	3	0.071	0.090	0.429	0.175	0.790	No	3	0.071	0.090	0.429	0.175	0.790
SBSN	SBP	Inverse variance weighted	4	-0.084	0.123	0.496	0.059	0.839	No	4	-0.084	0.123	0.496	0.059	0.839
SCAMP3	SBP	Wald ratio	1	-0.177	0.567	0.755	-	0.950	No	1	-0.177	0.567	0.755	-	0.950
SCARA5	SBP	Inverse variance weighted	4	-0.386	0.120	0.001	0.461	0.019	Yes	4	-0.386	0.120	0.001	0.461	0.019
SCARB2	SBP	Inverse variance weighted	4	0.096	0.454	0.832	0.000	0.966	No	4	0.096	0.454	0.832	0.000	0.966
SCARF1	SBP	Inverse variance weighted	4	0.277	0.100	0.006	0.558	0.058	No	4	0.277	0.100	0.006	0.558	0.058
SCARF2	SBP	Inverse variance weighted	2	0.027	0.359	0.941	-	0.984	No	2	0.027	0.359	0.941	-	0.984
SCG2	SBP	Wald ratio	1	-1.739	1.048	0.097	-	0.369	No	1	-1.739	1.048	0.097	-	0.369
SCG3	SBP	Inverse variance weighted	8	-0.167	0.064	0.009	0.811	0.080	No	8	-0.167	0.064	0.009	0.811	0.080
SCGB1A1	SBP	Inverse variance weighted	4	-0.042	0.124	0.737	0.352	0.945	No	4	-0.042	0.124	0.737	0.352	0.945
SCGB3A1	SBP	Inverse variance weighted	2	-0.474	0.810	0.558	-	0.866	No	2	-0.474	0.810	0.558	-	0.866
SCGB3A2	SBP	Inverse variance weighted	3	-0.023	0.185	0.901	0.960	0.978	No	3	-0.023	0.185	0.901	0.960	0.978
SCGN	SBP	Wald ratio	1	0.632	0.491	0.198	-	0.563	No	1	0.632	0.491	0.198	-	0.563
SCLY	SBP	Inverse variance weighted	2	-0.027	0.216	0.899	-	0.978	No	2	-0.027	0.216	0.899	-	0.978

ST2; MR causal estimates for plasma proteins on systolic blood pressure.

Causal candidates prioritized for SBP were marked as "Yes" in column "Prioritized". Effect of plasma protein levels on blood pressure is in mmHg unit.

										Steiger filtering					
**Exposure**	**Outocme**	**Method**	**nsnp**	**Beta**	**SE**	**P-value**	**Cochran's Q P-value**	**FDR-corrected P-value**	**Prioritized**	**nsnp**	**Beta**	**SE**	**P-value**	**Cochran's Q P-value**	**FDR-corrected P-value**
SCN4B	SBP	Inverse variance weighted	8	-0.156	0.070	0.027	0.336	0.172	No	8	-0.156	0.070	0.027	0.336	0.172
SCP2	SBP	Wald ratio	1	-0.894	0.869	0.304	-	0.692	No	1	-0.894	0.869	0.304	-	0.692
SCPEP1	SBP	Inverse variance weighted	3	-0.072	0.127	0.570	0.549	0.872	No	3	-0.072	0.127	0.570	0.549	0.872
SCRG1	SBP	Inverse variance weighted	2	0.327	0.625	0.601	-	0.884	No	2	0.327	0.625	0.601	-	0.884
SCRN1	SBP	Inverse variance weighted	3	0.729	0.319	0.022	0.540	0.152	No	3	0.729	0.319	0.022	0.540	0.152
SCT	SBP	Wald ratio	1	-0.679	0.925	0.463	-	0.817	No	1	-0.679	0.925	0.463	-	0.817
SDC1	SBP	Inverse variance weighted	2	-0.475	0.333	0.154	-	0.484	No	2	-0.475	0.333	0.154	-	0.484
SDC4	SBP	Inverse variance weighted	2	0.161	0.295	0.586	-	0.879	No	2	0.161	0.295	0.586	-	0.879
SDCCAG8	SBP	Wald ratio	1	-0.685	0.198	0.001	-	0.009	Yes	1	-0.685	0.198	0.001	-	0.009
SDHB	SBP	Wald ratio	1	2.496	0.661	0.000	-	0.003	Yes	1	2.496	0.661	0.000	-	0.003
SDK2	SBP	Inverse variance weighted	4	0.063	0.122	0.607	0.078	0.887	No	4	0.063	0.122	0.607	0.078	0.887
SEC31A	SBP	Wald ratio	1	0.393	0.391	0.315	-	0.696	No	1	0.393	0.391	0.315	-	0.696
SEL1L	SBP	Wald ratio	1	0.265	0.137	0.054	-	0.269	No	1	0.265	0.137	0.054	-	0.269
SELE	SBP	Inverse variance weighted	2	0.474	0.390	0.223	-	0.601	No	2	0.474	0.390	0.223	-	0.601
SELENOP	SBP	Wald ratio	1	1.468	0.407	0.000	-	0.006	Yes	1	1.468	0.407	0.000	-	0.006
SELL	SBP	Inverse variance weighted	4	-0.148	0.095	0.119	0.427	0.413	No	4	-0.148	0.095	0.119	0.427	0.413
SELP	SBP	Inverse variance weighted	2	-0.365	0.146	0.013	-	0.106	No	2	-0.365	0.146	0.013	-	0.106
SELPLG	SBP	Inverse variance weighted	2	0.069	0.087	0.428	-	0.790	No	2	0.069	0.087	0.428	-	0.790
SEMA3F	SBP	Inverse variance weighted	2	-0.301	0.274	0.272	-	0.660	No	2	-0.301	0.274	0.272	-	0.660
SEMA4D	SBP	Inverse variance weighted	2	-0.540	0.940	0.566	-	0.870	No	2	-0.540	0.940	0.566	-	0.870
SEMA6C	SBP	Wald ratio	1	-1.370	0.465	0.003	-	0.038	Yes	1	-1.370	0.465	0.003	-	0.038
SEMA7A	SBP	Inverse variance weighted	2	-0.697	0.710	0.326	-	0.708	No	2	-0.697	0.710	0.326	-	0.708
SEPTIN3	SBP	Wald ratio	1	0.378	1.121	0.736	-	0.944	No	1	0.378	1.121	0.736	-	0.944
SEPTIN8	SBP	Inverse variance weighted	2	-0.432	0.489	0.377	-	0.758	No	2	-0.432	0.489	0.377	-	0.758
SEPTIN9	SBP	Wald ratio	1	0.142	0.432	0.741	-	0.945	No	1	0.142	0.432	0.741	-	0.945
SERPI-1	SBP	Inverse variance weighted	2	1.254	0.527	0.017	-	0.130	No	2	1.254	0.527	0.017	-	0.130
SERPI-11	SBP	Inverse variance weighted	9	0.017	0.078	0.829	0.128	0.966	No	9	0.017	0.078	0.829	0.128	0.966
SERPI-12	SBP	Inverse variance weighted	6	0.082	0.063	0.191	0.277	0.551	No	6	0.082	0.063	0.191	0.277	0.551
SERPI-3	SBP	Inverse variance weighted	3	0.104	0.111	0.351	0.994	0.733	No	3	0.104	0.111	0.351	0.994	0.733
SERPI-4	SBP	Inverse variance weighted	6	0.027	0.119	0.823	0.024	0.966	No	6	0.027	0.119	0.823	0.024	0.966
SERPI-5	SBP	Inverse variance weighted	4	0.254	0.473	0.591	0.000	0.880	No	4	0.254	0.473	0.591	0.000	0.880
SERPI-6	SBP	Inverse variance weighted	4	0.368	0.183	0.044	0.698	0.238	No	4	0.368	0.183	0.044	0.698	0.238
SERPI-9	SBP	Inverse variance weighted	6	0.064	0.068	0.345	0.458	0.728	No	6	0.064	0.068	0.345	0.458	0.728
SERPINB1	SBP	Inverse variance weighted	2	0.312	0.349	0.373	-	0.755	No	2	0.312	0.349	0.373	-	0.755
SERPINB5	SBP	Wald ratio	1	-0.788	0.689	0.253	-	0.635	No	1	-0.788	0.689	0.253	-	0.635
SERPINB6	SBP	Wald ratio	1	0.019	0.250	0.940	-	0.984	No	1	0.019	0.250	0.940	-	0.984
SERPINB8	SBP	Inverse variance weighted	5	-0.020	0.080	0.801	0.040	0.959	No	5	-0.020	0.080	0.801	0.040	0.959
SERPINB9	SBP	Inverse variance weighted	2	-0.399	0.315	0.205	-	0.572	No	2	-0.399	0.315	0.205	-	0.572
SERPINC1	SBP	Wald ratio	1	-0.690	0.722	0.339	-	0.723	No	1	-0.690	0.722	0.339	-	0.723
SERPIND1	SBP	Inverse variance weighted	2	0.210	1.443	0.884	-	0.974	No	2	0.210	1.443	0.884	-	0.974
SERPINE1	SBP	Inverse variance weighted	2	0.122	1.395	0.930	-	0.983	No	2	0.122	1.395	0.930	-	0.983
SERPINE2	SBP	Inverse variance weighted	6	-0.014	0.073	0.849	0.854	0.967	No	6	-0.014	0.073	0.849	0.854	0.967
SERPINF1	SBP	Inverse variance weighted	3	-0.277	0.141	0.050	0.381	0.257	No	3	-0.277	0.141	0.050	0.381	0.257
SERPINF2	SBP	Inverse variance weighted	2	0.147	0.233	0.528	-	0.855	No	2	0.147	0.233	0.528	-	0.855
SERPING1	SBP	Inverse variance weighted	4	0.433	0.531	0.415	0.000	0.788	No	4	0.433	0.531	0.415	0.000	0.788
SERPINH1	SBP	Inverse variance weighted	2	-0.719	0.898	0.423	-	0.790	No	2	-0.719	0.898	0.423	-	0.790
SERPINI1	SBP	Inverse variance weighted	8	0.449	0.115	0.000	0.203	0.002	Yes	8	0.449	0.115	0.000	0.203	0.002
SERPINI2	SBP	Inverse variance weighted	3	0.318	0.132	0.016	0.282	0.122	No	3	0.318	0.132	0.016	0.282	0.122
SESTD1	SBP	Wald ratio	1	-0.187	0.404	0.644	-	0.904	No	1	-0.187	0.404	0.644	-	0.904
SETMAR	SBP	Inverse variance weighted	4	0.007	0.295	0.982	0.027	0.996	No	4	0.007	0.295	0.982	0.027	0.996
SEZ6	SBP	Wald ratio	1	-0.023	0.688	0.973	-	0.994	No	1	-0.023	0.688	0.973	-	0.994
SEZ6L	SBP	Inverse variance weighted	4	0.187	0.305	0.541	0.079	0.858	No	4	0.187	0.305	0.541	0.079	0.858
SEZ6L2	SBP	Inverse variance weighted	3	0.576	0.487	0.237	0.018	0.619	No	3	0.576	0.487	0.237	0.018	0.619
SF3B4	SBP	Wald ratio	1	1.382	0.793	0.081	-	0.335	No	1	1.382	0.793	0.081	-	0.335
SFRP1	SBP	Inverse variance weighted	4	-0.006	0.153	0.966	0.729	0.993	No	4	-0.006	0.153	0.966	0.729	0.993
SFRP4	SBP	Inverse variance weighted	3	0.073	0.188	0.697	0.665	0.925	No	3	0.073	0.188	0.697	0.665	0.925

ST2; MR causal estimates for plasma proteins on systolic blood pressure.

Causal candidates prioritized for SBP were marked as "Yes" in column "Prioritized". Effect of plasma protein levels on blood pressure is in mmHg unit.

										Steiger filtering					
**Exposure**	**Outocme**	**Method**	**nsnp**	**Beta**	**SE**	**P-value**	**Cochran's Q P-value**	**FDR-corrected P-value**	**Prioritized**	**nsnp**	**Beta**	**SE**	**P-value**	**Cochran's Q P-value**	**FDR-corrected P-value**
SFTPA1	SBP	Inverse variance weighted	2	-0.294	0.174	0.091	-	0.356	No	2	-0.294	0.174	0.091	-	0.356
SFTPA2	SBP	Inverse variance weighted	6	0.030	0.120	0.801	0.001	0.959	No	6	0.030	0.120	0.801	0.001	0.959
SFTPD	SBP	Inverse variance weighted	9	-0.086	0.082	0.299	0.007	0.688	No	9	-0.086	0.082	0.299	0.007	0.688
SGSH	SBP	Inverse variance weighted	9	-0.010	0.055	0.861	0.169	0.969	No	9	-0.010	0.055	0.861	0.169	0.969
SH2B3	SBP	Wald ratio	1	5.918	0.587	0.000	-	0.000	Yes	1	5.918	0.587	0.000	-	0.000
SH3BGRL2	SBP	Wald ratio	1	1.871	1.097	0.088	-	0.350	No	1	1.871	1.097	0.088	-	0.350
SH3BP1	SBP	Inverse variance weighted	3	0.234	0.284	0.409	0.054	0.785	No	3	0.234	0.284	0.409	0.054	0.785
SH3GLB2	SBP	Wald ratio	1	0.258	0.149	0.083	-	0.337	No	1	0.258	0.149	0.083	-	0.337
SHBG	SBP	Inverse variance weighted	3	0.211	0.150	0.161	0.304	0.496	No	3	0.211	0.150	0.161	0.304	0.496
SHH	SBP	Inverse variance weighted	2	0.419	1.605	0.794	-	0.956	No	2	0.419	1.605	0.794	-	0.956
SHISA5	SBP	Inverse variance weighted	3	0.150	0.541	0.782	0.025	0.953	No	3	0.150	0.541	0.782	0.025	0.953
SHMT1	SBP	Inverse variance weighted	6	0.220	0.074	0.003	0.117	0.034	Yes	6	0.220	0.074	0.003	0.117	0.034
SHPK	SBP	Wald ratio	1	0.210	0.545	0.700	-	0.926	No	1	0.210	0.545	0.700	-	0.926
SIAE	SBP	Inverse variance weighted	3	-0.023	0.256	0.927	0.158	0.983	No	3	-0.023	0.256	0.927	0.158	0.983
SIGLEC1	SBP	Inverse variance weighted	6	-0.017	0.091	0.853	0.970	0.967	No	6	-0.017	0.091	0.853	0.970	0.967
SIGLEC10	SBP	Inverse variance weighted	7	-0.067	0.078	0.393	0.919	0.770	No	7	-0.067	0.078	0.393	0.919	0.770
SIGLEC15	SBP	Wald ratio	1	-0.355	1.071	0.740	-	0.945	No	1	-0.355	1.071	0.740	-	0.945
SIGLEC5	SBP	Inverse variance weighted	13	-0.098	0.052	0.061	0.383	0.288	No	13	-0.098	0.052	0.061	0.383	0.288
SIGLEC6	SBP	Inverse variance weighted	11	0.088	0.083	0.289	0.303	0.675	No	11	0.088	0.083	0.289	0.303	0.675
SIGLEC7	SBP	Inverse variance weighted	5	0.018	0.128	0.889	0.309	0.975	No	5	0.018	0.128	0.889	0.309	0.975
SIGLEC8	SBP	Inverse variance weighted	8	-0.063	0.091	0.490	0.302	0.837	No	8	-0.063	0.091	0.490	0.302	0.837
SIGLEC9	SBP	Inverse variance weighted	6	0.038	0.069	0.586	0.839	0.879	No	6	0.038	0.069	0.586	0.839	0.879
SIL1	SBP	Wald ratio	1	2.142	0.694	0.002	-	0.026	Yes	1	2.142	0.694	0.002	-	0.026
SIRPA	SBP	Inverse variance weighted	10	-0.060	0.040	0.132	0.137	0.437	No	10	-0.060	0.040	0.132	0.137	0.437
SIRPB1	SBP	Inverse variance weighted	4	0.012	0.127	0.924	0.001	0.983	No	4	0.012	0.127	0.924	0.001	0.983
SIRT2	SBP	Wald ratio	1	-0.132	0.965	0.892	-	0.975	No	1	-0.132	0.965	0.892	-	0.975
SIRT5	SBP	Wald ratio	1	-0.199	0.988	0.840	-	0.967	No	1	-0.199	0.988	0.840	-	0.967
SKAP1	SBP	Wald ratio	1	-0.837	0.832	0.314	-	0.696	No	1	-0.837	0.832	0.314	-	0.696
SLA2	SBP	Wald ratio	1	0.642	0.689	0.351	-	0.733	No	1	0.642	0.689	0.351	-	0.733
SLAMF1	SBP	Inverse variance weighted	2	0.202	0.349	0.562	-	0.868	No	2	0.202	0.349	0.562	-	0.868
SLAMF6	SBP	Inverse variance weighted	2	0.423	0.454	0.351	-	0.733	No	2	0.423	0.454	0.351	-	0.733
SLAMF7	SBP	Inverse variance weighted	7	0.030	0.062	0.628	0.991	0.897	No	7	0.030	0.062	0.628	0.991	0.897
SLAMF8	SBP	Inverse variance weighted	6	0.013	0.051	0.804	0.245	0.959	No	6	0.013	0.051	0.804	0.245	0.959
SLC16A1	SBP	Wald ratio	1	-3.805	0.669	0.000	-	0.000	Yes	1	-3.805	0.669	0.000	-	0.000
SLC27A4	SBP	Wald ratio	1	1.649	0.677	0.015	-	0.115	No	1	1.649	0.677	0.015	-	0.115
SLC39A14	SBP	Wald ratio	1	1.379	0.438	0.002	-	0.022	Yes	1	1.379	0.438	0.002	-	0.022
SLC39A5	SBP	Inverse variance weighted	2	0.096	0.757	0.899	-	0.978	No	2	0.096	0.757	0.899	-	0.978
SLC4A1	SBP	Wald ratio	1	-0.786	0.663	0.236	-	0.619	No	1	-0.786	0.663	0.236	-	0.619
SLC9A3R1	SBP	Wald ratio	1	-0.804	0.431	0.062	-	0.292	No	1	-0.804	0.431	0.062	-	0.292
SLC9A3R2	SBP	Wald ratio	1	2.625	0.394	0.000	-	0.000	Yes	1	2.625	0.394	0.000	-	0.000
SLIT2	SBP	Inverse variance weighted	2	-1.634	0.607	0.007	-	0.068	No	2	-1.634	0.607	0.007	-	0.068
SLITRK1	SBP	Wald ratio	1	0.024	0.336	0.943	-	0.984	No	1	0.024	0.336	0.943	-	0.984
SLITRK6	SBP	Inverse variance weighted	7	-0.163	0.100	0.102	0.451	0.378	No	7	-0.163	0.100	0.102	0.451	0.378
SLMAP	SBP	Inverse variance weighted	2	0.152	1.078	0.888	-	0.975	No	2	0.152	1.078	0.888	-	0.975
SLURP1	SBP	Inverse variance weighted	2	0.035	0.100	0.725	-	0.942	No	2	0.035	0.100	0.725	-	0.942
SMAD1	SBP	Wald ratio	1	-0.006	0.919	0.994	-	0.997	No	1	-0.006	0.919	0.994	-	0.997
SMAD3	SBP	Wald ratio	1	-0.807	0.397	0.042	-	0.230	No	1	-0.807	0.397	0.042	-	0.230
SMAD5	SBP	Wald ratio	1	-2.131	1.014	0.036	-	0.213	No	1	-2.131	1.014	0.036	-	0.213
SMARCA2	SBP	Wald ratio	1	1.457	1.024	0.155	-	0.485	No	1	1.457	1.024	0.155	-	0.485
SMOC1	SBP	Inverse variance weighted	4	-0.386	0.383	0.314	0.000	0.696	No	4	-0.386	0.383	0.314	0.000	0.696
SMOC2	SBP	Inverse variance weighted	6	-0.353	0.104	0.001	0.848	0.011	Yes	6	-0.353	0.104	0.001	0.848	0.011
SMPD1	SBP	Inverse variance weighted	2	-0.095	0.099	0.339	-	0.723	No	2	-0.095	0.099	0.339	-	0.723
SMPD3	SBP	Wald ratio	1	-1.809	0.918	0.049	-	0.256	No	1	-1.809	0.918	0.049	-	0.256
SMPDL3A	SBP	Inverse variance weighted	9	0.130	0.074	0.078	0.060	0.327	No	9	0.130	0.074	0.078	0.060	0.327
SMTN	SBP	Wald ratio	1	-2.058	1.008	0.041	-	0.228	No	1	-2.058	1.008	0.041	-	0.228
S-P25	SBP	Inverse variance weighted	2	-0.083	0.329	0.800	-	0.959	No	2	-0.083	0.329	0.800	-	0.959

ST2; MR causal estimates for plasma proteins on systolic blood pressure.

Causal candidates prioritized for SBP were marked as "Yes" in column "Prioritized". Effect of plasma protein levels on blood pressure is in mmHg unit.

										Steiger filtering					
**Exposure**	**Outcome**	**Method**	**nsnp**	**Beta**	**SE**	**P-value**	**Cochran's Q P-value**	**FDR-corrected P-value**	**Prioritized**	**nsnp**	**Beta**	**SE**	**P-value**	**Cochran's Q P-value**	**FDR-corrected P-value**
S-P29	SBP	Wald ratio	1	-0.521	0.571	0.361	-	0.746	No	1	-0.521	0.571	0.361	-	0.746
SNCA	SBP	Wald ratio	1	2.384	0.882	0.007	-	0.067	No	1	2.384	0.882	0.007	-	0.067
SNCG	SBP	Inverse variance weighted	8	0.035	0.081	0.669	0.001	0.918	No	8	0.035	0.081	0.669	0.001	0.918
SNED1	SBP	Inverse variance weighted	3	-0.013	0.261	0.960	0.400	0.991	No	3	-0.013	0.261	0.960	0.400	0.991
SNX15	SBP	Inverse variance weighted	2	-0.027	0.315	0.931	-	0.983	No	2	-0.027	0.315	0.931	-	0.983
SNX18	SBP	Wald ratio	1	-0.323	0.573	0.573	-	0.875	No	1	-0.323	0.573	0.573	-	0.875
SNX9	SBP	Inverse variance weighted	3	-0.430	0.568	0.449	0.140	0.805	No	3	-0.430	0.568	0.449	0.140	0.805
SOD1	SBP	Wald ratio	1	0.548	1.000	0.583	-	0.879	No	1	0.548	1.000	0.583	-	0.879
SOD2	SBP	Inverse variance weighted	3	0.018	0.245	0.942	0.902	0.984	No	3	0.018	0.245	0.942	0.902	0.984
SOD3	SBP	Inverse variance weighted	7	0.052	0.078	0.506	0.754	0.842	No	7	0.052	0.078	0.506	0.754	0.842
SORBS1	SBP	Inverse variance weighted	2	1.273	2.984	0.670	-	0.918	No	2	1.273	2.984	0.670	-	0.918
SORCS2	SBP	Inverse variance weighted	8	-0.159	0.101	0.116	0.239	0.407	No	8	-0.159	0.101	0.116	0.239	0.407
SORD	SBP	Inverse variance weighted	2	-0.398	0.566	0.482	-	0.831	No	2	-0.398	0.566	0.482	-	0.831
SORT1	SBP	Inverse variance weighted	2	-0.515	0.209	0.014	-	0.111	No	2	-0.515	0.209	0.014	-	0.111
SOST	SBP	Wald ratio	1	-2.902	0.750	0.000	-	0.003	Yes	1	-2.902	0.750	0.000	-	0.003
SPAG1	SBP	Inverse variance weighted	7	-0.051	0.062	0.407	0.270	0.784	No	7	-0.051	0.062	0.407	0.270	0.784
SPARC	SBP	Inverse variance weighted	2	-0.280	0.599	0.640	-	0.901	No	2	-0.280	0.599	0.640	-	0.901
SPARCL1	SBP	Inverse variance weighted	5	-0.070	0.091	0.443	0.045	0.801	No	5	-0.070	0.091	0.443	0.045	0.801
SPESP1	SBP	Inverse variance weighted	2	-0.362	0.319	0.256	-	0.639	No	2	-0.362	0.319	0.256	-	0.639
SPINK1	SBP	Inverse variance weighted	5	0.053	0.098	0.587	0.681	0.879	No	5	0.053	0.098	0.587	0.681	0.879
SPINK2	SBP	Inverse variance weighted	11	-0.049	0.092	0.592	0.338	0.880	No	11	-0.049	0.092	0.592	0.338	0.880
SPINK4	SBP	Inverse variance weighted	9	-0.070	0.066	0.290	0.092	0.675	No	9	-0.070	0.066	0.290	0.092	0.675
SPINK5	SBP	Inverse variance weighted	5	0.051	0.095	0.591	0.385	0.880	No	5	0.051	0.095	0.591	0.385	0.880
SPINK6	SBP	Inverse variance weighted	3	-0.230	0.233	0.324	0.012	0.705	No	3	-0.230	0.233	0.324	0.012	0.705
SPINK8	SBP	Wald ratio	1	0.686	0.092	0.000	-	0.000	Yes	1	0.686	0.092	0.000	-	0.000
SPINT1	SBP	Inverse variance weighted	5	0.088	0.237	0.710	0.006	0.932	No	5	0.088	0.237	0.710	0.006	0.932
SPINT2	SBP	Inverse variance weighted	2	0.516	0.252	0.040	-	0.225	No	2	0.516	0.252	0.040	-	0.225
SPINT3	SBP	Inverse variance weighted	2	-0.550	0.701	0.433	-	0.794	No	2	-0.550	0.701	0.433	-	0.794
SPOCK1	SBP	Inverse variance weighted	3	0.000	0.371	0.999	0.171	0.999	No	3	0.000	0.371	0.999	0.171	0.999
SPON1	SBP	Inverse variance weighted	4	0.177	0.128	0.168	0.524	0.507	No	4	0.177	0.128	0.168	0.524	0.507
SPON2	SBP	Inverse variance weighted	4	0.044	0.240	0.853	0.195	0.967	No	4	0.044	0.240	0.853	0.195	0.967
SPP1	SBP	Inverse variance weighted	4	-0.260	0.304	0.392	0.834	0.770	No	4	-0.260	0.304	0.392	0.834	0.770
SPRED2	SBP	Wald ratio	1	2.452	0.631	0.000	-	0.003	Yes	1	2.452	0.631	0.000	-	0.003
SPRING1	SBP	Wald ratio	1	1.137	1.071	0.289	-	0.675	No	1	1.137	1.071	0.289	-	0.675
SPRR3	SBP	Inverse variance weighted	2	-0.058	0.168	0.731	-	0.943	No	2	-0.058	0.168	0.731	-	0.943
SPRY2	SBP	Inverse variance weighted	2	-1.054	0.388	0.007	-	0.066	No	2	-1.054	0.388	0.007	-	0.066
SPTLC1	SBP	Inverse variance weighted	2	-0.069	0.229	0.762	-	0.950	No	2	-0.069	0.229	0.762	-	0.950
SRP14	SBP	Wald ratio	1	-0.898	0.823	0.275	-	0.662	No	1	-0.898	0.823	0.275	-	0.662
SSC4D	SBP	Inverse variance weighted	5	0.123	0.111	0.268	0.764	0.653	No	5	0.123	0.111	0.268	0.764	0.653
SSC5D	SBP	Inverse variance weighted	4	0.011	0.090	0.906	0.340	0.980	No	4	0.011	0.090	0.906	0.340	0.980
SSH3	SBP	Wald ratio	1	-0.473	1.172	0.687	-	0.922	No	1	-0.473	1.172	0.687	-	0.922
ST3GAL1	SBP	Inverse variance weighted	2	-0.112	0.164	0.496	-	0.839	No	2	-0.112	0.164	0.496	-	0.839
ST6GAL1	SBP	Inverse variance weighted	3	-0.230	0.317	0.467	0.113	0.818	No	3	-0.230	0.317	0.467	0.113	0.818
ST8SIA1	SBP	Wald ratio	1	0.450	0.947	0.635	-	0.900	No	1	0.450	0.947	0.635	-	0.900
STAB2	SBP	Inverse variance weighted	11	0.183	0.088	0.038	0.521	0.221	No	11	0.183	0.088	0.038	0.521	0.221
STAMBP	SBP	Wald ratio	1	0.270	0.805	0.738	-	0.945	No	1	0.270	0.805	0.738	-	0.945
STAT2	SBP	Inverse variance weighted	2	-0.030	0.243	0.900	-	0.978	No	2	-0.030	0.243	0.900	-	0.978
STC1	SBP	Wald ratio	1	-1.234	0.736	0.094	-	0.363	No	1	-1.234	0.736	0.094	-	0.363
STC2	SBP	Inverse variance weighted	2	0.540	0.505	0.284	-	0.672	No	2	0.540	0.505	0.284	-	0.672
STK4	SBP	Wald ratio	1	-0.590	0.728	0.418	-	0.789	No	1	-0.590	0.728	0.418	-	0.789
STX16	SBP	Inverse variance weighted	2	0.282	0.562	0.616	-	0.892	No	2	0.282	0.562	0.616	-	0.892
STX4	SBP	Wald ratio	1	2.066	0.984	0.036	-	0.214	No	1	2.066	0.984	0.036	-	0.214
STX7	SBP	Inverse variance weighted	6	-0.132	0.086	0.123	0.151	0.419	No	6	-0.132	0.086	0.123	0.151	0.419
STX8	SBP	Wald ratio	1	-0.533	0.463	0.249	-	0.629	No	1	-0.533	0.463	0.249	-	0.629
STXBP1	SBP	Wald ratio	1	0.554	0.733	0.450	-	0.805	No	1	0.554	0.733	0.450	-	0.805
SUGP1	SBP	Wald ratio	1	0.253	0.166	0.129	-	0.433	No	1	0.253	0.166	0.129	-	0.433

ST2; MR causal estimates for plasma proteins on systolic blood pressure.

Causal candidates prioritized for SBP were marked as "Yes" in column "Prioritized". Effect of plasma protein levels on blood pressure is in mmHg unit.

										Steiger filtering					
Exposure	Outocme	Method	nsnp	Beta	SE	P-value	Cochran's Q P-value	FDR-corrected P-value	Prioritized	nsnp	Beta	SE	P-value	Cochran's Q P-value	FDR-corrected P-value
SULT1A1	SBP	Inverse variance weighted	4	-1.054	0.616	0.087	0.011	0.348	No	4	-1.054	0.616	0.087	0.011	0.348
SULT2A1	SBP	Inverse variance weighted	2	0.174	0.142	0.222	-	0.599	No	2	0.174	0.142	0.222	-	0.599
SUMF2	SBP	Inverse variance weighted	7	0.091	0.114	0.425	0.131	0.790	No	7	0.091	0.114	0.425	0.131	0.790
SUSD1	SBP	Inverse variance weighted	2	0.013	0.350	0.971	-	0.994	No	2	0.013	0.350	0.971	-	0.994
SUSD2	SBP	Inverse variance weighted	9	-0.099	0.122	0.417	0.009	0.789	No	9	-0.099	0.122	0.417	0.009	0.789
SUSD4	SBP	Inverse variance weighted	2	-0.008	0.106	0.942	-	0.984	No	2	-0.008	0.106	0.942	-	0.984
SUSD5	SBP	Inverse variance weighted	7	-0.064	0.088	0.464	0.437	0.817	No	7	-0.064	0.088	0.464	0.437	0.817
SV2A	SBP	Wald ratio	1	0.390	0.251	0.121	-	0.416	No	1	0.390	0.251	0.121	-	0.416
SWAP70	SBP	Inverse variance weighted	2	-0.846	1.909	0.658	-	0.913	No	2	-0.846	1.909	0.658	-	0.913
TAB2	SBP	Wald ratio	1	-0.193	1.029	0.851	-	0.967	No	1	-0.193	1.029	0.851	-	0.967
TACC3	SBP	Inverse variance weighted	2	-0.615	0.300	0.040	-	0.226	No	2	-0.615	0.300	0.040	-	0.226
TACSTD2	SBP	Inverse variance weighted	6	-0.005	0.086	0.954	0.041	0.989	No	6	-0.005	0.086	0.954	0.041	0.989
TAFA5	SBP	Inverse variance weighted	8	0.155	0.200	0.438	0.139	0.798	No	8	0.155	0.200	0.438	0.139	0.798
TALDO1	SBP	Inverse variance weighted	4	0.860	0.477	0.071	0.374	0.312	No	4	0.860	0.477	0.071	0.374	0.312
TARBP2	SBP	Wald ratio	1	-3.665	0.883	0.000	-	0.001	Yes	1	-3.665	0.883	0.000	-	0.001
TBC1D17	SBP	Inverse variance weighted	2	-0.264	0.101	0.009	-	0.082	No	2	-0.264	0.101	0.009	-	0.082
TBC1D23	SBP	Wald ratio	1	-0.782	0.262	0.003	-	0.034	Yes	1	-0.782	0.262	0.003	-	0.034
TBC1D5	SBP	Wald ratio	1	1.592	1.356	0.240	-	0.622	No	1	1.592	1.356	0.240	-	0.622
TBCA	SBP	Wald ratio	1	0.252	0.306	0.410	-	0.785	No	1	0.252	0.306	0.410	-	0.785
TBCB	SBP	Wald ratio	1	-0.387	0.766	0.614	-	0.890	No	1	-0.387	0.766	0.614	-	0.890
TBCC	SBP	Wald ratio	1	0.497	0.291	0.088	-	0.350	No	1	0.497	0.291	0.088	-	0.350
TCL1A	SBP	Inverse variance weighted	3	0.252	0.178	0.157	0.197	0.492	No	3	0.252	0.178	0.157	0.197	0.492
TCN1	SBP	Inverse variance weighted	11	0.133	0.077	0.084	0.730	0.339	No	11	0.133	0.077	0.084	0.730	0.339
TCN2	SBP	Inverse variance weighted	11	-0.113	0.175	0.519	0.000	0.849	No	11	-0.113	0.175	0.519	0.000	0.849
TCOF1	SBP	Wald ratio	1	1.706	1.140	0.135	-	0.443	No	1	1.706	1.140	0.135	-	0.443
TCTN3	SBP	Inverse variance weighted	11	0.081	0.079	0.304	0.062	0.692	No	11	0.081	0.079	0.304	0.062	0.692
TDGF1	SBP	Inverse variance weighted	7	0.001	0.049	0.986	0.066	0.997	No	7	0.001	0.049	0.986	0.066	0.997
TDP1	SBP	Inverse variance weighted	3	-0.340	0.755	0.652	0.059	0.909	No	3	-0.340	0.755	0.652	0.059	0.909
TDRKH	SBP	Wald ratio	1	0.077	0.125	0.541	-	0.858	No	1	0.077	0.125	0.541	-	0.858
TEF	SBP	Wald ratio	1	0.438	0.632	0.488	-	0.836	No	1	0.438	0.632	0.488	-	0.836
TEK	SBP	Inverse variance weighted	13	0.284	0.080	0.000	0.318	0.008	Yes	13	0.284	0.080	0.000	0.318	0.008
TEX101	SBP	Wald ratio	1	-0.142	0.172	0.411	-	0.785	No	1	-0.142	0.172	0.411	-	0.785
TF	SBP	Inverse variance weighted	2	0.102	0.125	0.416	-	0.788	No	2	0.102	0.125	0.416	-	0.788
TFF1	SBP	Inverse variance weighted	2	0.176	0.222	0.429	-	0.790	No	2	0.176	0.222	0.429	-	0.790
TFF2	SBP	Inverse variance weighted	2	-0.353	0.315	0.263	-	0.645	No	2	-0.353	0.315	0.263	-	0.645
TFF3	SBP	Inverse variance weighted	4	0.162	0.251	0.519	0.324	0.849	No	4	0.162	0.251	0.519	0.324	0.849
TFPI	SBP	Inverse variance weighted	3	-0.286	0.194	0.141	0.070	0.458	No	3	-0.286	0.194	0.141	0.070	0.458
TFPI2	SBP	Inverse variance weighted	6	0.141	0.304	0.642	0.062	0.904	No	6	0.141	0.304	0.642	0.062	0.904
TFRC	SBP	Inverse variance weighted	3	0.159	0.077	0.040	0.622	0.225	No	3	0.159	0.077	0.040	0.622	0.225
TG	SBP	Inverse variance weighted	2	-0.069	0.473	0.884	-	0.974	No	2	-0.069	0.473	0.884	-	0.974
TGFA	SBP	Inverse variance weighted	2	0.099	0.326	0.760	-	0.950	No	2	0.099	0.326	0.760	-	0.950
TGFB1	SBP	Inverse variance weighted	2	0.399	0.215	0.063	-	0.292	No	2	0.399	0.215	0.063	-	0.292
TGFB2	SBP	Wald ratio	1	-0.897	0.300	0.003	-	0.034	Yes	1	-0.897	0.300	0.003	-	0.034
TGFBI	SBP	Inverse variance weighted	7	-0.019	0.082	0.812	0.223	0.963	No	7	-0.019	0.082	0.812	0.223	0.963
TGFBR1	SBP	Wald ratio	1	-0.302	0.904	0.738	-	0.945	No	1	-0.302	0.904	0.738	-	0.945
TGFBR2	SBP	Inverse variance weighted	4	0.214	1.272	0.866	0.000	0.971	No	4	0.214	1.272	0.866	0.000	0.971
TGFBR3	SBP	Inverse variance weighted	3	-0.006	0.402	0.988	0.076	0.997	No	3	-0.006	0.402	0.988	0.076	0.997
TGM2	SBP	Inverse variance weighted	2	-0.479	0.233	0.040	-	0.225	No	2	-0.479	0.233	0.040	-	0.225
TGOLN2	SBP	Inverse variance weighted	8	-0.144	0.063	0.023	0.262	0.154	No	8	-0.144	0.063	0.023	0.262	0.154
THBD	SBP	Inverse variance weighted	3	-0.553	0.231	0.016	0.342	0.123	No	3	-0.553	0.231	0.016	0.342	0.123
THBS2	SBP	Inverse variance weighted	4	0.231	0.228	0.311	0.001	0.696	No	4	0.231	0.228	0.311	0.001	0.696
THBS4	SBP	Inverse variance weighted	3	-0.343	0.238	0.149	0.100	0.473	No	3	-0.343	0.238	0.149	0.100	0.473
THOP1	SBP	Inverse variance weighted	2	-0.228	0.163	0.162	-	0.499	No	2	-0.228	0.163	0.162	-	0.499
THPO	SBP	Inverse variance weighted	2	0.364	0.607	0.549	-	0.862	No	2	0.364	0.607	0.549	-	0.862
THSD1	SBP	Inverse variance weighted	2	-0.347	0.486	0.475	-	0.826	No	2	-0.347	0.486	0.475	-	0.826
THTPA	SBP	Wald ratio	1	0.188	0.092	0.042	-	0.230	No	1	0.188	0.092	0.042	-	0.230

ST2; MR causal estimates for plasma proteins on systolic blood pressure.

Causal candidates prioritized for SBP were marked as "Yes" in column "Prioritized". Effect of plasma protein levels on blood pressure is in mmHg unit.

										Steiger filtering					
**Exposure**	**Outcome**	**Method**	**nsnp**	**Beta**	**SE**	**P-value**	**Cochran's Q P-value**	**FDR-corrected P-value**	**Prioritized**	**nsnp**	**Beta**	**SE**	**P-value**	**Cochran's Q P-value**	**FDR-corrected P-value**
THY1	SBP	Inverse variance weighted	7	0.262	0.109	0.016	0.745	0.123	No	7	0.262	0.109	0.016	0.745	0.123
TIE1	SBP	Wald ratio	1	-1.771	0.207	0.000	-	0.000	Yes	1	-1.771	0.207	0.000	-	0.000
TIGAR	SBP	Wald ratio	1	-1.266	0.749	0.091	-	0.356	No	1	-1.266	0.749	0.091	-	0.356
TIGIT	SBP	Wald ratio	1	-0.411	0.465	0.377	-	0.758	No	1	-0.411	0.465	0.377	-	0.758
TIMD4	SBP	Inverse variance weighted	3	-0.152	0.211	0.469	0.204	0.820	No	3	-0.152	0.211	0.469	0.204	0.820
TIMM10	SBP	Wald ratio	1	0.225	0.221	0.308	-	0.696	No	1	0.225	0.221	0.308	-	0.696
TIMP2	SBP	Inverse variance weighted	2	-0.046	1.177	0.969	-	0.994	No	2	-0.046	1.177	0.969	-	0.994
TIMP3	SBP	Inverse variance weighted	6	0.003	0.066	0.968	0.599	0.994	No	6	0.003	0.066	0.968	0.599	0.994
TIMP4	SBP	Inverse variance weighted	4	0.032	0.114	0.777	0.547	0.953	No	4	0.032	0.114	0.777	0.547	0.953
TI-GL1	SBP	Wald ratio	1	-1.112	0.566	0.049	-	0.256	No	1	-1.112	0.566	0.049	-	0.256
TJAP1	SBP	Wald ratio	1	-9.039	1.005	0.000	-	0.000	Yes	1	-9.039	1.005	0.000	-	0.000
TK1	SBP	Wald ratio	1	0.553	0.548	0.313	-	0.696	No	1	0.553	0.548	0.313	-	0.696
TLR1	SBP	Inverse variance weighted	2	-0.256	0.097	0.008	-	0.077	No	2	-0.256	0.097	0.008	-	0.077
TLR3	SBP	Inverse variance weighted	11	0.046	0.038	0.226	0.417	0.605	No	11	0.046	0.038	0.226	0.417	0.605
TLR4	SBP	Inverse variance weighted	2	-0.347	0.272	0.202	-	0.572	No	2	-0.347	0.272	0.202	-	0.572
TMED8	SBP	Wald ratio	1	-1.136	0.586	0.053	-	0.264	No	1	-1.136	0.586	0.053	-	0.264
TMEM106A	SBP	Wald ratio	1	-0.322	0.404	0.426	-	0.790	No	1	-0.322	0.404	0.426	-	0.790
TMEM132A	SBP	Inverse variance weighted	5	0.031	0.065	0.630	0.810	0.897	No	5	0.031	0.065	0.630	0.810	0.897
TMEM25	SBP	Inverse variance weighted	7	0.041	0.103	0.690	0.835	0.923	No	7	0.041	0.103	0.690	0.835	0.923
TMPRSS11D	SBP	Inverse variance weighted	3	-0.394	0.452	0.384	0.081	0.763	No	3	-0.394	0.452	0.384	0.081	0.763
TMPRSS15	SBP	Inverse variance weighted	2	0.334	0.337	0.322	-	0.704	No	2	0.334	0.337	0.322	-	0.704
TMPRSS5	SBP	Inverse variance weighted	9	0.061	0.052	0.236	0.632	0.619	No	9	0.061	0.052	0.236	0.632	0.619
TMSB10	SBP	Wald ratio	1	-1.951	1.112	0.079	-	0.329	No	1	-1.951	1.112	0.079	-	0.329
TNC	SBP	Inverse variance weighted	5	0.296	0.147	0.044	0.631	0.239	No	5	0.296	0.147	0.044	0.631	0.239
TNFAIP2	SBP	Inverse variance weighted	2	-1.288	0.495	0.009	-	0.085	No	2	-1.288	0.495	0.009	-	0.085
TNFAIP8	SBP	Wald ratio	1	0.950	0.532	0.074	-	0.318	No	1	0.950	0.532	0.074	-	0.318
TNFAIP8L2	SBP	Wald ratio	1	0.166	0.764	0.828	-	0.966	No	1	0.166	0.764	0.828	-	0.966
TNFRSF10A	SBP	Inverse variance weighted	6	-0.018	0.124	0.885	0.035	0.974	No	6	-0.018	0.124	0.885	0.035	0.974
TNFRSF10B	SBP	Inverse variance weighted	5	-0.018	0.117	0.878	0.620	0.974	No	5	-0.018	0.117	0.878	0.620	0.974
TNFRSF10C	SBP	Inverse variance weighted	7	-0.038	0.143	0.789	0.458	0.955	No	7	-0.038	0.143	0.789	0.458	0.955
TNFRSF11A	SBP	Inverse variance weighted	4	0.038	0.149	0.800	0.014	0.959	No	4	0.038	0.149	0.800	0.014	0.959
TNFRSF11B	SBP	Inverse variance weighted	5	0.407	0.409	0.320	0.001	0.702	No	5	0.407	0.409	0.320	0.001	0.702
TNFRSF12A	SBP	Inverse variance weighted	2	0.607	0.554	0.273	-	0.660	No	2	0.607	0.554	0.273	-	0.660
TNFRSF13B	SBP	Inverse variance weighted	3	-0.171	0.330	0.604	0.926	0.887	No	3	-0.171	0.330	0.604	0.926	0.887
TNFRSF13C	SBP	Inverse variance weighted	5	0.006	0.094	0.947	0.268	0.984	No	5	0.006	0.094	0.947	0.268	0.984
TNFRSF14	SBP	Wald ratio	1	0.648	0.348	0.063	-	0.292	No	1	0.648	0.348	0.063	-	0.292
TNFRSF17	SBP	Wald ratio	1	-1.665	0.304	0.000	-	0.000	Yes	1	-1.665	0.304	0.000	-	0.000
TNFRSF19	SBP	Inverse variance weighted	4	0.323	0.219	0.140	0.166	0.456	No	4	0.323	0.219	0.140	0.166	0.456
TNFRSF1A	SBP	Wald ratio	1	-0.200	0.528	0.705	-	0.930	No	1	-0.200	0.528	0.705	-	0.930
TNFRSF1B	SBP	Wald ratio	1	-0.400	0.265	0.131	-	0.437	No	1	-0.400	0.265	0.131	-	0.437
TNFRSF21	SBP	Inverse variance weighted	2	-0.342	0.464	0.462	-	0.817	No	2	-0.342	0.464	0.462	-	0.817
TNFRSF4	SBP	Inverse variance weighted	3	0.405	0.997	0.685	0.000	0.922	No	3	0.405	0.997	0.685	0.000	0.922
TNFRSF6B	SBP	Inverse variance weighted	5	-0.287	0.272	0.291	0.001	0.678	No	5	-0.287	0.272	0.291	0.001	0.678
TNFRSF8	SBP	Inverse variance weighted	3	0.493	0.476	0.301	0.000	0.688	No	3	0.493	0.476	0.301	0.000	0.688
TNFRSF9	SBP	Wald ratio	1	-0.185	0.275	0.502	-	0.840	No	1	-0.185	0.275	0.502	-	0.840
TNFSF10	SBP	Inverse variance weighted	3	0.094	0.233	0.685	0.295	0.922	No	3	0.094	0.233	0.685	0.295	0.922
TNFSF11	SBP	Wald ratio	1	0.175	0.346	0.613	-	0.890	No	1	0.175	0.346	0.613	-	0.890
TNFSF12	SBP	Inverse variance weighted	2	0.816	0.116	0.000	-	0.000	Yes	2	0.816	0.116	0.000	-	0.000
TNFSF13	SBP	Inverse variance weighted	4	-0.406	0.526	0.441	0.000	0.801	No	4	-0.406	0.526	0.441	0.000	0.801
TNFSF13B	SBP	Inverse variance weighted	6	-0.466	0.164	0.004	0.401	0.048	Yes	6	-0.466	0.164	0.004	0.401	0.048
TNFSF14	SBP	Inverse variance weighted	6	-0.042	0.201	0.833	0.054	0.966	No	6	-0.042	0.201	0.833	0.054	0.966
TNFSF8	SBP	Inverse variance weighted	2	0.034	0.176	0.845	-	0.967	No	2	0.034	0.176	0.845	-	0.967
TNIP1	SBP	Wald ratio	1	-1.520	0.951	0.110	-	0.392	No	1	-1.520	0.951	0.110	-	0.392
TNN	SBP	Inverse variance weighted	6	-0.005	0.051	0.922	0.253	0.982	No	6	-0.005	0.051	0.922	0.253	0.982
TNR	SBP	Inverse variance weighted	4	-0.231	0.198	0.245	0.016	0.626	No	4	-0.231	0.198	0.245	0.016	0.626
TOP1	SBP	Wald ratio	1	0.692	1.363	0.612	-	0.890	No	1	0.692	1.363	0.612	-	0.890

ST2; MR causal estimates for plasma proteins on systolic blood pressure.

Causal candidates prioritized for SBP were marked as "Yes" in column "Prioritized". Effect of plasma protein levels on blood pressure is in mmHg unit.

										Steiger filtering					
**Exposure**	**Outocme**	**Method**	**nsnp**	**Beta**	**SE**	**P-value**	**Cochran's Q P-value**	**FDR-corrected P-value**	**Prioritized**	**nsnp**	**Beta**	**SE**	**P-value**	**Cochran's Q P-value**	**FDR-corrected P-value**
TOP2B	SBP	Wald ratio	1	0.782	0.526	0.137	-	0.446	No	1	0.782	0.526	0.137	-	0.446
TOR1AIP1	SBP	Inverse variance weighted	4	0.179	0.071	0.012	0.185	0.102	No	4	0.179	0.071	0.012	0.185	0.102
TP53	SBP	Wald ratio	1	-0.510	0.717	0.477	-	0.828	No	1	-0.510	0.717	0.477	-	0.828
TP53I3	SBP	Inverse variance weighted	2	-0.111	0.090	0.217	-	0.590	No	2	-0.111	0.090	0.217	-	0.590
TPK1	SBP	Inverse variance weighted	7	-0.246	0.133	0.065	0.136	0.297	No	7	-0.246	0.133	0.065	0.136	0.297
TPMT	SBP	Inverse variance weighted	3	-0.031	0.097	0.750	0.247	0.946	No	3	-0.031	0.097	0.750	0.247	0.946
TPP1	SBP	Inverse variance weighted	3	-0.220	0.547	0.688	0.004	0.922	No	3	-0.220	0.547	0.688	0.004	0.922
TPPP3	SBP	Wald ratio	1	-0.999	0.622	0.108	-	0.392	No	1	-0.999	0.622	0.108	-	0.392
TPR	SBP	Wald ratio	1	2.120	0.870	0.015	-	0.115	No	1	2.120	0.870	0.015	-	0.115
TPSAB1	SBP	Inverse variance weighted	12	-0.092	0.120	0.445	0.018	0.801	No	12	-0.092	0.120	0.445	0.018	0.801
TPSD1	SBP	Inverse variance weighted	4	-0.138	0.195	0.480	0.077	0.831	No	4	-0.138	0.195	0.480	0.077	0.831
TREH	SBP	Inverse variance weighted	10	0.007	0.071	0.919	0.004	0.982	No	10	0.007	0.071	0.919	0.004	0.982
TREM2	SBP	Inverse variance weighted	2	0.229	0.758	0.763	-	0.950	No	2	0.229	0.758	0.763	-	0.950
TREML2	SBP	Inverse variance weighted	4	-0.057	0.193	0.768	0.130	0.950	No	4	-0.057	0.193	0.768	0.130	0.950
TRIM21	SBP	Inverse variance weighted	2	-0.457	1.071	0.670	-	0.918	No	2	-0.457	1.071	0.670	-	0.918
TRIM24	SBP	Wald ratio	1	0.080	1.033	0.938	-	0.984	No	1	0.080	1.033	0.938	-	0.984
TRIM25	SBP	Wald ratio	1	0.229	0.459	0.618	-	0.892	No	1	0.229	0.459	0.618	-	0.892
TRIM5	SBP	Inverse variance weighted	3	0.168	0.183	0.358	0.124	0.742	No	3	0.168	0.183	0.358	0.124	0.742
TRIM58	SBP	Inverse variance weighted	2	0.010	0.223	0.966	-	0.993	No	2	0.010	0.223	0.966	-	0.993
TSC22D1	SBP	Wald ratio	1	0.651	0.717	0.363	-	0.747	No	1	0.651	0.717	0.363	-	0.747
TSPAN1	SBP	Wald ratio	1	0.750	0.900	0.405	-	0.783	No	1	0.750	0.900	0.405	-	0.783
TSPAN15	SBP	Wald ratio	1	-0.821	0.665	0.217	-	0.590	No	1	-0.821	0.665	0.217	-	0.590
TSPAN8	SBP	Inverse variance weighted	3	-0.038	0.089	0.671	0.319	0.918	No	3	-0.038	0.089	0.671	0.319	0.918
TSPYL1	SBP	Inverse variance weighted	2	0.118	0.348	0.734	-	0.943	No	2	0.118	0.348	0.734	-	0.943
TST	SBP	Inverse variance weighted	2	-0.376	0.319	0.239	-	0.620	No	2	-0.376	0.319	0.239	-	0.620
TTF2	SBP	Inverse variance weighted	2	0.169	0.167	0.312	-	0.696	No	2	0.169	0.167	0.312	-	0.696
TTN	SBP	Wald ratio	1	0.190	0.989	0.847	-	0.967	No	1	0.190	0.989	0.847	-	0.967
TTR	SBP	Wald ratio	1	-0.158	0.330	0.632	-	0.898	No	1	-0.158	0.330	0.632	-	0.898
TWF2	SBP	Wald ratio	1	2.378	0.618	0.000	-	0.003	Yes	1	2.378	0.618	0.000	-	0.003
TXN	SBP	Inverse variance weighted	2	0.095	0.630	0.880	-	0.974	No	2	0.095	0.630	0.880	-	0.974
TXNDC15	SBP	Inverse variance weighted	10	0.039	0.058	0.502	0.194	0.840	No	10	0.039	0.058	0.502	0.194	0.840
TXNDC5	SBP	Inverse variance weighted	2	0.620	0.395	0.116	-	0.407	No	2	0.620	0.395	0.116	-	0.407
TXNDC9	SBP	Wald ratio	1	-0.985	0.789	0.212	-	0.583	No	1	-0.985	0.789	0.212	-	0.583
TXNRD1	SBP	Wald ratio	1	1.198	0.924	0.195	-	0.556	No	1	1.198	0.924	0.195	-	0.556
TYMP	SBP	Wald ratio	1	0.366	0.218	0.093	-	0.361	No	1	0.366	0.218	0.093	-	0.361
TYRO3	SBP	Inverse variance weighted	8	0.207	0.084	0.014	0.102	0.111	No	8	0.207	0.084	0.014	0.102	0.111
TYRP1	SBP	Wald ratio	1	1.010	0.534	0.059	-	0.283	No	1	1.010	0.534	0.059	-	0.283
UBAC1	SBP	Wald ratio	1	-0.541	0.630	0.390	-	0.770	No	1	-0.541	0.630	0.390	-	0.770
UBE2L6	SBP	Inverse variance weighted	2	-0.716	0.203	0.000	-	0.008	Yes	2	-0.716	0.203	0.000	-	0.008
ULBP2	SBP	Inverse variance weighted	8	0.058	0.083	0.486	0.315	0.836	No	8	0.058	0.083	0.486	0.315	0.836
UMOD	SBP	Inverse variance weighted	10	0.389	0.051	0.000	0.755	0.000	Yes	10	0.389	0.051	0.000	0.755	0.000
UNC5D	SBP	Inverse variance weighted	2	0.469	0.795	0.555	-	0.865	No	2	0.469	0.795	0.555	-	0.865
UNG	SBP	Wald ratio	1	-0.310	0.931	0.739	-	0.945	No	1	-0.310	0.931	0.739	-	0.945
UPB1	SBP	Wald ratio	1	0.600	1.051	0.568	-	0.871	No	1	0.600	1.051	0.568	-	0.871
UROD	SBP	Wald ratio	1	0.175	0.094	0.063	-	0.292	No	1	0.175	0.094	0.063	-	0.292
UROS	SBP	Wald ratio	1	0.706	0.361	0.051	-	0.259	No	1	0.706	0.361	0.051	-	0.259
USP28	SBP	Inverse variance weighted	2	-1.772	0.948	0.061	-	0.292	No	2	-1.772	0.948	0.061	-	0.292
USP8	SBP	Wald ratio	1	-1.225	0.529	0.021	-	0.142	No	1	-1.225	0.529	0.021	-	0.142
UXS1	SBP	Inverse variance weighted	2	-0.812	0.378	0.032	-	0.197	No	2	-0.812	0.378	0.032	-	0.197
VAMP5	SBP	Inverse variance weighted	2	0.538	0.766	0.482	-	0.831	No	2	0.538	0.766	0.482	-	0.831
VAMP8	SBP	Inverse variance weighted	2	0.170	0.363	0.639	-	0.901	No	2	0.170	0.363	0.639	-	0.901
VASH1	SBP	Inverse variance weighted	2	0.067	0.387	0.863	-	0.970	No	2	0.067	0.387	0.863	-	0.970
VASN	SBP	Inverse variance weighted	11	0.314	0.148	0.034	0.001	0.207	No	11	0.314	0.148	0.034	0.001	0.207
VAT1	SBP	Wald ratio	1	-0.212	0.290	0.465	-	0.817	No	1	-0.212	0.290	0.465	-	0.817
VAV3	SBP	Inverse variance weighted	2	0.061	0.573	0.916	-	0.982	No	2	0.061	0.573	0.916	-	0.982
VCAM1	SBP	Inverse variance weighted	2	-0.315	0.426	0.460	-	0.816	No	2	-0.315	0.426	0.460	-	0.816

ST2; MR causal estimates for plasma proteins on systolic blood pressure.

Causal candidates prioritized for SBP were marked as "Yes" in column "Prioritized". Effect of plasma protein levels on blood pressure is in mmHg unit.

										Steiger filtering					
**Exposure**	**Outocme**	**Method**	**nsnp**	**Beta**	**SE**	**P-value**	**Cochran's Q P-value**	**FDR-corrected P-value**	**Prioritized**	**nsnp**	**Beta**	**SE**	**P-value**	**Cochran's Q P-value**	**FDR-corrected P-value**
VCAN	SBP	Inverse variance weighted	3	-0.115	0.106	0.281	0.047	0.670	No	3	-0.115	0.106	0.281	0.047	0.670
VCPKMT	SBP	Inverse variance weighted	2	-0.940	0.588	0.110	-	0.392	No	2	-0.940	0.588	0.110	-	0.392
VEGFA	SBP	Inverse variance weighted	8	-0.005	0.057	0.935	0.731	0.984	No	8	-0.005	0.057	0.935	0.731	0.984
VEGFB	SBP	Inverse variance weighted	2	-0.995	0.367	0.007	-	0.066	No	2	-0.995	0.367	0.007	-	0.066
VEGFC	SBP	Inverse variance weighted	2	0.723	0.427	0.090	-	0.356	No	2	0.723	0.427	0.090	-	0.356
VGF	SBP	Wald ratio	1	-0.907	0.661	0.170	-	0.511	No	1	-0.907	0.661	0.170	-	0.511
VIT	SBP	Inverse variance weighted	7	-0.106	0.105	0.313	0.114	0.696	No	7	-0.106	0.105	0.313	0.114	0.696
VMO1	SBP	Inverse variance weighted	15	0.096	0.051	0.059	0.456	0.286	No	15	0.096	0.051	0.059	0.456	0.286
VNN1	SBP	Inverse variance weighted	8	-0.048	0.042	0.250	0.854	0.630	No	8	-0.048	0.042	0.250	0.854	0.630
VNN2	SBP	Inverse variance weighted	6	-0.002	0.061	0.970	0.581	0.994	No	6	-0.002	0.061	0.970	0.581	0.994
VPS4B	SBP	Wald ratio	1	0.227	0.888	0.798	-	0.958	No	1	0.227	0.888	0.798	-	0.958
VSIG10	SBP	Inverse variance weighted	4	0.118	0.065	0.069	0.231	0.306	No	4	0.118	0.065	0.069	0.231	0.306
VSIG10L	SBP	Wald ratio	1	-0.001	0.189	0.997	-	0.999	No	1	-0.001	0.189	0.997	-	0.999
VSIG2	SBP	Inverse variance weighted	2	0.761	0.217	0.000	-	0.008	Yes	2	0.761	0.217	0.000	-	0.008
VSIR	SBP	Inverse variance weighted	3	-0.122	0.140	0.382	0.272	0.763	No	3	-0.122	0.140	0.382	0.272	0.763
VSNL1	SBP	Inverse variance weighted	2	-0.163	0.581	0.779	-	0.953	No	2	-0.163	0.581	0.779	-	0.953
VSTM1	SBP	Inverse variance weighted	7	0.006	0.050	0.897	0.571	0.977	No	7	0.006	0.050	0.897	0.571	0.977
VSTM2L	SBP	Inverse variance weighted	2	0.190	0.357	0.594	-	0.880	No	2	0.190	0.357	0.594	-	0.880
VTCN1	SBP	Inverse variance weighted	2	-0.058	0.498	0.907	-	0.980	No	2	-0.058	0.498	0.907	-	0.980
VTI1A	SBP	Wald ratio	1	0.603	0.682	0.377	-	0.758	No	1	0.603	0.682	0.377	-	0.758
VWA1	SBP	Inverse variance weighted	4	0.114	0.303	0.707	0.000	0.931	No	4	0.114	0.303	0.707	0.000	0.931
VWC2	SBP	Inverse variance weighted	5	0.022	0.212	0.917	0.656	0.982	No	5	0.022	0.212	0.917	0.656	0.982
VWC2L	SBP	Inverse variance weighted	2	-0.603	0.425	0.156	-	0.487	No	2	-0.603	0.425	0.156	-	0.487
VWF	SBP	Inverse variance weighted	2	0.142	0.381	0.709	-	0.931	No	2	0.142	0.381	0.709	-	0.931
WARS	SBP	Wald ratio	1	0.803	0.195	0.000	-	0.001	Yes	1	0.803	0.195	0.000	-	0.001
WASF1	SBP	Inverse variance weighted	3	0.007	0.134	0.958	0.937	0.991	No	3	0.007	0.134	0.958	0.937	0.991
WASHC3	SBP	Wald ratio	1	1.847	0.534	0.001	-	0.009	Yes	1	1.847	0.534	0.001	-	0.009
WFDC1	SBP	Inverse variance weighted	2	-0.007	0.148	0.964	-	0.993	No	2	-0.007	0.148	0.964	-	0.993
WFDC12	SBP	Inverse variance weighted	6	0.047	0.087	0.589	0.699	0.879	No	6	0.047	0.087	0.589	0.699	0.879
WFDC2	SBP	Inverse variance weighted	2	1.908	1.068	0.074	-	0.318	No	2	1.908	1.068	0.074	-	0.318
WFIKKN1	SBP	Inverse variance weighted	3	0.135	0.396	0.732	0.002	0.943	No	3	0.135	0.396	0.732	0.002	0.943
WFIKKN2	SBP	Inverse variance weighted	4	-0.131	0.071	0.065	0.264	0.297	No	4	-0.131	0.071	0.065	0.264	0.297
WIF1	SBP	Inverse variance weighted	3	-0.215	0.203	0.290	0.613	0.675	No	3	-0.215	0.203	0.290	0.613	0.675
WNT9A	SBP	Inverse variance weighted	2	0.721	0.293	0.014	-	0.111	No	2	0.721	0.293	0.014	-	0.111
WWP2	SBP	Wald ratio	1	-0.728	0.256	0.005	-	0.048	Yes	1	-0.728	0.256	0.005	-	0.048
XCL1	SBP	Inverse variance weighted	5	-0.025	0.068	0.708	0.257	0.931	No	5	-0.025	0.068	0.708	0.257	0.931
XRCC4	SBP	Wald ratio	1	0.186	0.648	0.774	-	0.952	No	1	0.186	0.648	0.774	-	0.952
YAP1	SBP	Wald ratio	1	-1.055	0.627	0.092	-	0.359	No	1	-1.055	0.627	0.092	-	0.359
YES1	SBP	Wald ratio	1	-1.122	0.554	0.043	-	0.233	No	1	-1.122	0.554	0.043	-	0.233
YJU2	SBP	Wald ratio	1	0.686	0.761	0.367	-	0.750	No	1	0.686	0.761	0.367	-	0.750
YOD1	SBP	Wald ratio	1	-1.278	0.368	0.001	-	0.009	Yes	1	-1.278	0.368	0.001	-	0.009
ZBP1	SBP	Inverse variance weighted	3	0.063	0.146	0.665	0.270	0.916	No	3	0.063	0.146	0.665	0.270	0.916
ZBTB16	SBP	Inverse variance weighted	3	0.177	0.115	0.124	0.363	0.422	No	3	0.177	0.115	0.124	0.363	0.422
ZBTB17	SBP	Wald ratio	1	3.473	0.905	0.000	-	0.003	Yes	1	3.473	0.905	0.000	-	0.003
ZFYVE19	SBP	Wald ratio	1	-0.392	0.112	0.000	-	0.008	Yes	1	-0.392	0.112	0.000	-	0.008
ZP3	SBP	Inverse variance weighted	14	-0.027	0.039	0.486	0.265	0.836	No	14	-0.027	0.039	0.486	0.265	0.836
ZPR1	SBP	Wald ratio	1	0.789	0.732	0.282	-	0.670	No	1	0.789	0.732	0.282	-	0.670

nsnp = number of single nucleotide polymorphisms; SE = standard error; Cochran's Q P-value = p-value from Cochran's Q test assessing heterogeneity; FDR=false discovery rate

ST3: MR causal estimates for plasma proteins on diastolic blood pressure.

Causal candidates prioritized for DBP were marked as "Yes" in column "Prioritized". Effect of plasma protein levels on blood pressure is in mmHg unit.

										Steiger filtering					
Exposure	Outocme	Method	nsnp	Beta	SE	P-value	Cochran's Q P-value	FDR-corrected P-value	Prioritized	nsnp	Beta	SE	P-value	Cochran's Q P-value	FDR-corrected P-value
A1BG	DBP	Inverse variance weighted	5	0.218	0.212	0.303	0.039	0.693	No	5	0.218	0.212	0.303	0.039	0.694
AAMDC	DBP	Inverse variance weighted	3	-0.219	0.039	0.000	0.275	0.000	Yes	3	-0.219	0.039	0.000	0.275	0.000
AARSD1	DBP	Wald ratio	1	-0.492	0.194	0.011	-	0.103	No	1	-0.492	0.194	0.011	-	0.104
ABHD14B	DBP	Inverse variance weighted	2	0.073	0.084	0.379	-	0.751	No	2	0.073	0.084	0.379	-	0.752
ABL1	DBP	Wald ratio	1	-1.177	0.541	0.030	-	0.198	No	1	-1.177	0.541	0.030	-	0.199
ABO	DBP	Inverse variance weighted	9	-0.179	0.021	0.000	0.639	0.000	Yes	9	-0.179	0.021	0.000	0.639	0.000
ACAA1	DBP	Inverse variance weighted	2	-0.132	0.199	0.508	-	0.830	No	2	-0.132	0.199	0.508	-	0.831
ACADM	DBP	Wald ratio	1	-0.217	0.175	0.215	-	0.593	No	1	-0.217	0.175	0.215	-	0.594
ACADSB	DBP	Inverse variance weighted	4	-0.059	0.106	0.581	0.432	0.870	No	4	-0.059	0.106	0.581	0.432	0.870
ACAN	DBP	Inverse variance weighted	2	-0.137	0.198	0.489	-	0.823	No	2	-0.137	0.198	0.489	-	0.823
ACE	DBP	Inverse variance weighted	4	0.136	0.091	0.133	0.017	0.484	No	4	0.136	0.091	0.133	0.017	0.485
ACHE	DBP	Inverse variance weighted	6	0.277	0.104	0.008	0.043	0.083	No	6	0.277	0.104	0.008	0.043	0.084
ACOT13	DBP	Inverse variance weighted	2	-0.401	0.301	0.183	-	0.555	No	2	-0.401	0.301	0.183	-	0.556
ACOX1	DBP	Wald ratio	1	1.339	0.314	0.000	-	0.001	Yes	1	1.339	0.314	0.000	-	0.001
ACP1	DBP	Inverse variance weighted	3	0.207	0.111	0.062	0.043	0.307	No	3	0.207	0.111	0.062	0.043	0.308
ACP5	DBP	Inverse variance weighted	6	0.033	0.139	0.810	0.000	0.955	No	6	0.033	0.139	0.810	0.000	0.955
ACP6	DBP	Inverse variance weighted	8	-0.053	0.031	0.087	0.096	0.388	No	8	-0.053	0.031	0.087	0.096	0.389
ACRBP	DBP	Wald ratio	1	0.315	0.153	0.039	-	0.235	No	1	0.315	0.153	0.039	-	0.237
ACRV1	DBP	Wald ratio	1	0.070	0.405	0.864	-	0.976	No	1	0.070	0.405	0.864	-	0.976
ACTA2	DBP	Wald ratio	1	-0.371	0.444	0.403	-	0.770	No	1	-0.371	0.444	0.403	-	0.771
ACVRL1	DBP	Inverse variance weighted	6	-0.134	0.088	0.126	0.227	0.477	No	6	-0.134	0.088	0.126	0.227	0.478
ACY1	DBP	Inverse variance weighted	2	-0.159	0.188	0.399	-	0.769	No	2	-0.159	0.188	0.399	-	0.769
ACY3	DBP	Inverse variance weighted	4	-0.054	0.146	0.713	0.028	0.931	No	4	-0.054	0.146	0.713	0.028	0.932
ACYP1	DBP	Inverse variance weighted	3	-0.044	0.147	0.765	0.048	0.938	No	3	-0.044	0.147	0.765	0.048	0.939
ADA	DBP	Inverse variance weighted	7	0.019	0.049	0.697	0.050	0.931	No	7	0.019	0.049	0.697	0.050	0.931
ADA2	DBP	Inverse variance weighted	3	0.036	0.084	0.672	0.663	0.914	No	3	0.036	0.084	0.672	0.663	0.914
ADAM12	DBP	Inverse variance weighted	3	-0.101	0.125	0.419	0.889	0.778	No	3	-0.101	0.125	0.419	0.889	0.779
ADAM15	DBP	Inverse variance weighted	11	0.090	0.032	0.005	0.288	0.060	No	11	0.090	0.032	0.005	0.288	0.061
ADAM22	DBP	Inverse variance weighted	5	0.132	0.100	0.189	0.985	0.562	No	5	0.132	0.100	0.189	0.985	0.563
ADAM23	DBP	Inverse variance weighted	9	-0.056	0.052	0.274	0.028	0.665	No	9	-0.056	0.052	0.274	0.028	0.666
ADAM8	DBP	Inverse variance weighted	8	0.058	0.047	0.215	0.580	0.593	No	8	0.058	0.047	0.215	0.580	0.594
ADAM9	DBP	Inverse variance weighted	5	0.218	0.165	0.187	0.326	0.561	No	5	0.218	0.165	0.187	0.326	0.562
ADAMTS1	DBP	Wald ratio	1	0.825	0.506	0.103	-	0.421	No	1	0.825	0.506	0.103	-	0.423
ADAMTS13	DBP	Inverse variance weighted	6	0.044	0.057	0.443	0.352	0.796	No	6	0.044	0.057	0.443	0.352	0.796
ADAMTS15	DBP	Inverse variance weighted	3	-0.235	0.270	0.383	0.115	0.754	No	3	-0.235	0.270	0.383	0.115	0.754
ADAMTS16	DBP	Inverse variance weighted	5	-0.213	0.235	0.363	0.007	0.741	No	5	-0.213	0.235	0.363	0.007	0.742
ADAMTS4	DBP	Wald ratio	1	0.450	0.194	0.020	-	0.151	No	1	0.450	0.194	0.020	-	0.152
ADAMTS8	DBP	Inverse variance weighted	7	0.105	0.045	0.020	0.417	0.149	No	7	0.105	0.045	0.020	0.417	0.150
ADAMTSL2	DBP	Inverse variance weighted	4	0.116	0.272	0.670	0.075	0.914	No	4	0.116	0.272	0.670	0.075	0.914
ADAMTSL4	DBP	Inverse variance weighted	3	0.110	0.152	0.467	0.183	0.808	No	3	0.110	0.152	0.467	0.183	0.809
ADAMTSL5	DBP	Inverse variance weighted	5	-0.148	0.059	0.013	0.694	0.113	No	5	-0.148	0.059	0.013	0.694	0.114
ADD1	DBP	Wald ratio	1	-0.342	0.164	0.037	-	0.230	No	1	-0.342	0.164	0.037	-	0.231
ADGRB3	DBP	Inverse variance weighted	12	0.016	0.031	0.597	0.067	0.879	No	12	0.016	0.031	0.597	0.067	0.879
ADGRD1	DBP	Inverse variance weighted	8	0.065	0.054	0.233	0.749	0.620	No	8	0.065	0.054	0.233	0.749	0.621
ADGRE1	DBP	Inverse variance weighted	5	0.014	0.090	0.875	0.187	0.980	No	5	0.014	0.090	0.875	0.187	0.980
ADGRE2	DBP	Inverse variance weighted	4	-0.115	0.089	0.198	0.392	0.568	No	4	-0.115	0.089	0.198	0.392	0.569
ADGRE5	DBP	Inverse variance weighted	4	0.026	0.102	0.797	0.424	0.948	No	4	0.026	0.102	0.797	0.424	0.948
ADGRG1	DBP	Inverse variance weighted	4	-0.149	0.228	0.513	0.448	0.832	No	4	-0.149	0.228	0.513	0.448	0.832
ADH1B	DBP	Inverse variance weighted	2	1.082	0.507	0.033	-	0.211	No	2	1.082	0.507	0.033	-	0.212
ADH4	DBP	Wald ratio	1	-0.237	0.180	0.188	-	0.561	No	1	-0.237	0.180	0.188	-	0.562
ADIPOQ	DBP	Inverse variance weighted	8	-0.097	0.125	0.438	0.118	0.793	No	8	-0.097	0.125	0.438	0.118	0.794
ADM	DBP	Wald ratio	1	2.265	0.655	0.001	-	0.011	Yes	1	2.265	0.655	0.001	-	0.011
AFAP1	DBP	Inverse variance weighted	6	0.018	0.023	0.442	0.766	0.796	No	6	0.018	0.023	0.442	0.766	0.796
AFM	DBP	Inverse variance weighted	4	-0.060	0.254	0.813	0.106	0.956	No	4	-0.060	0.254	0.813	0.106	0.957
AFP	DBP	Inverse variance weighted	2	0.056	0.330	0.865	-	0.976	No	2	0.056	0.330	0.865	-	0.976
AGR2	DBP	Wald ratio	1	-0.093	0.229	0.685	-	0.920	No	1	-0.093	0.229	0.685	-	0.920

ST3: MR causal estimates for plasma proteins on diastolic blood pressure.

Causal candidates prioritized for DBP were marked as "Yes" in column "Prioritized". Effect of plasma protein levels on blood pressure is in mmHg unit.

										Steiger filtering					
Exposure	Outocme	Method	nsnp	Beta	SE	P-value	Cochran's Q P-value	FDR-corrected P-value	Prioritized	nsnp	Beta	SE	P-value	Cochran's Q P-value	FDR-corrected P-value
AGR3	DBP	Wald ratio	1	-0.238	0.630	0.705	-	0.931	No	1	-0.238	0.630	0.705	-	0.932
AGRN	DBP	Inverse variance weighted	3	0.091	0.065	0.161	0.867	0.521	No	3	0.091	0.065	0.161	0.867	0.522
AGRP	DBP	Inverse variance weighted	5	-0.072	0.199	0.715	0.794	0.931	No	5	-0.072	0.199	0.715	0.794	0.932
AGT	DBP	Inverse variance weighted	4	0.063	0.048	0.195	0.582	0.567	No	4	0.063	0.048	0.195	0.582	0.568
AGXT	DBP	Inverse variance weighted	3	-0.052	0.090	0.565	0.301	0.860	No	3	-0.052	0.090	0.565	0.301	0.860
AHCY	DBP	Inverse variance weighted	2	-0.066	0.066	0.319	-	0.710	No	2	-0.066	0.066	0.319	-	0.711
AH-K	DBP	Wald ratio	1	0.059	0.141	0.674	-	0.914	No	1	0.059	0.141	0.674	-	0.914
AH-K2	DBP	Inverse variance weighted	3	0.021	0.113	0.852	0.349	0.973	No	3	0.021	0.113	0.852	0.349	0.973
AHSG	DBP	Inverse variance weighted	6	-0.043	0.034	0.209	0.637	0.586	No	6	-0.043	0.034	0.209	0.637	0.587
AHSP	DBP	Wald ratio	1	0.706	0.565	0.211	-	0.589	No	1	0.706	0.565	0.211	-	0.590
AIF1L	DBP	Inverse variance weighted	2	-0.038	0.125	0.760	-	0.938	No	2	-0.038	0.125	0.760	-	0.938
AK1	DBP	Wald ratio	1	-0.109	0.525	0.835	-	0.964	No	1	-0.109	0.525	0.835	-	0.964
AK2	DBP	Wald ratio	1	-0.864	0.589	0.143	-	0.492	No	1	-0.864	0.589	0.143	-	0.493
AKAP12	DBP	Inverse variance weighted	2	0.365	0.392	0.352	-	0.732	No	2	0.365	0.392	0.352	-	0.733
AKR1B1	DBP	Inverse variance weighted	2	0.301	0.154	0.051	-	0.272	No	2	0.301	0.154	0.051	-	0.273
AKR1C4	DBP	Wald ratio	1	0.252	0.424	0.553	-	0.854	No	1	0.252	0.424	0.553	-	0.855
AKR7L	DBP	Inverse variance weighted	2	-0.015	0.216	0.944	-	0.989	No	2	-0.015	0.216	0.944	-	0.989
AKT3	DBP	Wald ratio	1	-0.376	0.421	0.372	-	0.749	No	1	-0.376	0.421	0.372	-	0.750
ALCAM	DBP	Inverse variance weighted	3	0.017	0.146	0.905	0.386	0.983	No	3	0.017	0.146	0.905	0.386	0.983
ALDH1A1	DBP	Wald ratio	1	-0.738	0.288	0.010	-	0.100	No	1	-0.738	0.288	0.010	-	0.101
ALDH3A1	DBP	Inverse variance weighted	2	-0.008	0.074	0.914	-	0.983	No	2	-0.008	0.074	0.914	-	0.983
ALDH5A1	DBP	Inverse variance weighted	2	0.196	0.152	0.197	-	0.567	No	2	0.196	0.152	0.197	-	0.568
ALPP	DBP	Inverse variance weighted	4	0.066	0.073	0.366	0.071	0.743	No	4	0.066	0.073	0.366	0.071	0.744
AMBN	DBP	Wald ratio	1	0.523	0.346	0.130	-	0.480	No	1	0.523	0.346	0.130	-	0.482
AMBP	DBP	Inverse variance weighted	4	0.277	0.293	0.345	0.023	0.725	No	4	0.277	0.293	0.345	0.023	0.726
AMFR	DBP	Wald ratio	1	1.895	0.540	0.000	-	0.009	Yes	1	1.895	0.540	0.000	-	0.009
AMIGO1	DBP	Wald ratio	1	-0.602	0.541	0.266	-	0.652	No	1	-0.602	0.541	0.266	-	0.653
AMIGO2	DBP	Inverse variance weighted	3	-0.016	0.191	0.935	0.110	0.986	No	3	-0.016	0.191	0.935	0.110	0.986
AMN	DBP	Inverse variance weighted	6	-0.030	0.055	0.581	0.020	0.870	No	6	-0.030	0.055	0.581	0.020	0.870
AMOTL2	DBP	Wald ratio	1	-2.475	0.447	0.000	-	0.000	Yes	1	-2.475	0.447	0.000	-	0.000
AMPD3	DBP	Inverse variance weighted	7	-0.378	0.182	0.038	0.002	0.235	No	7	-0.378	0.182	0.038	0.002	0.236
AMY2A	DBP	Inverse variance weighted	15	-0.024	0.047	0.616	0.876	0.890	No	15	-0.024	0.047	0.616	0.876	0.891
AMY2B	DBP	Inverse variance weighted	15	-0.042	0.045	0.345	0.684	0.725	No	15	-0.042	0.045	0.345	0.684	0.726
ANG	DBP	Inverse variance weighted	5	-0.030	0.079	0.706	0.050	0.931	No	5	-0.030	0.079	0.706	0.050	0.932
ANGPT1	DBP	Wald ratio	1	-0.847	0.436	0.052	-	0.275	No	1	-0.847	0.436	0.052	-	0.276
ANGPT2	DBP	Inverse variance weighted	3	-0.237	0.316	0.454	0.001	0.800	No	3	-0.237	0.316	0.454	0.001	0.801
ANGPTL1	DBP	Inverse variance weighted	5	-0.099	0.069	0.150	0.606	0.498	No	5	-0.099	0.069	0.150	0.606	0.499
ANGPTL2	DBP	Wald ratio	1	-0.627	0.419	0.134	-	0.485	No	1	-0.627	0.419	0.134	-	0.486
ANGPTL3	DBP	Inverse variance weighted	2	0.066	0.085	0.440	-	0.794	No	2	0.066	0.085	0.440	-	0.795
ANGPTL4	DBP	Inverse variance weighted	2	-0.217	0.138	0.115	-	0.456	No	2	-0.217	0.138	0.115	-	0.458
ANGPTL7	DBP	Inverse variance weighted	3	0.208	0.078	0.008	0.291	0.085	No	3	0.208	0.078	0.008	0.291	0.085
ANKMY2	DBP	Wald ratio	1	-0.975	0.302	0.001	-	0.020	Yes	1	-0.975	0.302	0.001	-	0.021
ANKRD54	DBP	Wald ratio	1	0.813	0.342	0.017	-	0.139	No	1	0.813	0.342	0.017	-	0.140
ANPEP	DBP	Inverse variance weighted	12	0.053	0.063	0.393	0.069	0.767	No	12	0.053	0.063	0.393	0.069	0.768
ANXA1	DBP	Wald ratio	1	-0.687	0.533	0.197	-	0.567	No	1	-0.687	0.533	0.197	-	0.568
ANXA10	DBP	Wald ratio	1	0.538	0.509	0.291	-	0.681	No	1	0.538	0.509	0.291	-	0.681
ANXA11	DBP	Wald ratio	1	-0.780	0.379	0.039	-	0.237	No	1	-0.780	0.379	0.039	-	0.238
ANXA2	DBP	Inverse variance weighted	4	0.042	0.093	0.653	0.014	0.904	No	4	0.042	0.093	0.653	0.014	0.905
ANXA3	DBP	Inverse variance weighted	2	0.018	0.290	0.950	-	0.992	No	2	0.018	0.290	0.950	-	0.992
ANXA4	DBP	Inverse variance weighted	2	0.580	0.398	0.145	-	0.492	No	2	0.580	0.398	0.145	-	0.493
ANXA5	DBP	Wald ratio	1	-0.157	0.229	0.492	-	0.823	No	1	-0.157	0.229	0.492	-	0.824
AOC1	DBP	Inverse variance weighted	3	-0.383	0.081	0.000	0.574	0.000	Yes	3	-0.383	0.081	0.000	0.574	0.000
AOC3	DBP	Inverse variance weighted	5	-0.163	0.053	0.002	0.140	0.032	Yes	5	-0.163	0.053	0.002	0.140	0.033
AP1G2	DBP	Inverse variance weighted	2	0.310	0.603	0.607	-	0.884	No	2	0.310	0.603	0.607	-	0.885
AP3B1	DBP	Wald ratio	1	0.177	0.442	0.689	-	0.924	No	1	0.177	0.442	0.689	-	0.925
APBB1IP	DBP	Wald ratio	1	0.231	0.108	0.032	-	0.208	No	1	0.231	0.108	0.032	-	0.209

ST3: MR causal estimates for plasma proteins on diastolic blood pressure.

Causal candidates prioritized for DBP were marked as "Yes" in column "Prioritized". Effect of plasma protein levels on blood pressure is in mmHg unit.

										Steiger filtering					
Exposure	Outocme	Method	nsnp	Beta	SE	P-value	Cochran's Q P-value	FDR-corrected P-value	Prioritized	nsnp	Beta	SE	P-value	Cochran's Q P-value	FDR-corrected P-value
APCS	DBP	Inverse variance weighted	4	0.013	0.115	0.913	0.495	0.983	No	4	0.013	0.115	0.913	0.495	0.983
APEX1	DBP	Inverse variance weighted	3	0.068	0.061	0.264	0.327	0.652	No	3	0.068	0.061	0.264	0.327	0.653
APOA1	DBP	Wald ratio	1	1.280	0.365	0.000	-	0.009	Yes	1	1.280	0.365	0.000	-	0.009
APOA2	DBP	Wald ratio	1	-1.047	0.363	0.004	-	0.053	No	1	-1.047	0.363	0.004	-	0.053
APOA4	DBP	Wald ratio	1	-0.014	0.169	0.934	-	0.986	No	1	-0.014	0.169	0.934	-	0.986
APOBR	DBP	Inverse variance weighted	8	-0.095	0.024	0.000	0.201	0.003	Yes	8	-0.095	0.024	0.000	0.201	0.003
APOC1	DBP	Inverse variance weighted	2	-0.971	0.271	0.000	-	0.007	Yes	2	-0.971	0.271	0.000	-	0.008
APOD	DBP	Inverse variance weighted	2	-0.281	0.116	0.016	-	0.130	No	2	-0.281	0.116	0.016	-	0.131
APOE	DBP	Inverse variance weighted	9	0.060	0.062	0.332	0.000	0.720	No	9	0.060	0.062	0.332	0.000	0.721
APOF	DBP	Inverse variance weighted	3	-0.083	0.127	0.510	0.670	0.831	No	3	-0.083	0.127	0.510	0.670	0.832
APOH	DBP	Inverse variance weighted	5	-0.043	0.070	0.539	0.007	0.847	No	5	-0.043	0.070	0.539	0.007	0.848
APOL1	DBP	Wald ratio	1	-0.248	0.096	0.009	-	0.095	No	1	-0.248	0.096	0.009	-	0.096
APP	DBP	Wald ratio	1	0.308	0.350	0.378	-	0.751	No	1	0.308	0.350	0.378	-	0.752
APPL2	DBP	Wald ratio	1	-0.097	0.181	0.590	-	0.875	No	1	-0.097	0.181	0.590	-	0.876
APRT	DBP	Inverse variance weighted	2	0.665	0.294	0.024	-	0.169	No	2	0.665	0.294	0.024	-	0.170
AREG	DBP	Wald ratio	1	0.027	0.152	0.861	-	0.976	No	1	0.027	0.152	0.861	-	0.976
ARFIP1	DBP	Wald ratio	1	-0.118	0.150	0.434	-	0.793	No	1	-0.118	0.150	0.434	-	0.794
ARG1	DBP	Inverse variance weighted	3	-0.017	0.185	0.926	0.097	0.986	No	3	-0.017	0.185	0.926	0.097	0.986
ARG2	DBP	Wald ratio	1	-0.011	0.444	0.980	-	0.999	No	1	-0.011	0.444	0.980	-	0.999
ARHGAP25	DBP	Inverse variance weighted	2	0.077	0.493	0.876	-	0.980	No	2	0.077	0.493	0.876	-	0.980
ARHGAP45	DBP	Wald ratio	1	0.672	0.323	0.038	-	0.231	No	1	0.672	0.323	0.038	-	0.232
ARHGEF10	DBP	Inverse variance weighted	6	0.001	0.065	0.988	0.844	1.000	No	6	0.001	0.065	0.988	0.844	1.000
ARHGEF12	DBP	Wald ratio	1	0.070	0.655	0.915	-	0.983	No	1	0.070	0.655	0.915	-	0.983
ARHGEF5	DBP	Inverse variance weighted	13	-0.004	0.057	0.940	0.225	0.988	No	13	-0.004	0.057	0.940	0.225	0.988
ARL2BP	DBP	Wald ratio	1	-0.097	0.086	0.259	-	0.648	No	1	-0.097	0.086	0.259	-	0.649
ARSA	DBP	Inverse variance weighted	6	-0.053	0.047	0.253	0.173	0.640	No	6	-0.053	0.047	0.253	0.173	0.641
ARSB	DBP	Inverse variance weighted	5	0.351	0.104	0.001	0.050	0.013	Yes	5	0.351	0.104	0.001	0.050	0.014
ART3	DBP	Inverse variance weighted	5	0.058	0.097	0.552	0.308	0.854	No	5	0.058	0.097	0.552	0.308	0.855
ART5	DBP	Inverse variance weighted	2	0.176	0.123	0.151	-	0.498	No	2	0.176	0.123	0.151	-	0.499
ASAH1	DBP	Inverse variance weighted	6	-0.074	0.085	0.385	0.008	0.755	No	6	-0.074	0.085	0.385	0.008	0.755
ASAH2	DBP	Inverse variance weighted	8	-0.034	0.051	0.496	0.029	0.825	No	8	-0.034	0.051	0.496	0.029	0.826
ASGR1	DBP	Wald ratio	1	-0.394	0.155	0.011	-	0.103	No	1	-0.394	0.155	0.011	-	0.104
ASGR2	DBP	Inverse variance weighted	5	0.153	0.149	0.305	0.005	0.693	No	5	0.153	0.149	0.305	0.005	0.694
ASPN	DBP	Inverse variance weighted	2	0.083	0.101	0.413	-	0.777	No	2	0.083	0.101	0.413	-	0.778
ASPSCR1	DBP	Wald ratio	1	1.474	0.697	0.034	-	0.218	No	1	1.474	0.697	0.034	-	0.219
ASRGL1	DBP	Inverse variance weighted	3	-0.080	0.139	0.564	0.012	0.859	No	3	-0.080	0.139	0.564	0.012	0.860
ASS1	DBP	Wald ratio	1	-0.959	0.711	0.177	-	0.550	No	1	-0.959	0.711	0.177	-	0.551
ATG16L1	DBP	Wald ratio	1	0.457	0.604	0.449	-	0.799	No	1	0.457	0.604	0.449	-	0.800
ATOX1	DBP	Wald ratio	1	0.328	0.320	0.305	-	0.693	No	1	0.328	0.320	0.305	-	0.694
ATP1B1	DBP	Wald ratio	1	-0.249	0.577	0.665	-	0.911	No	1	-0.249	0.577	0.665	-	0.911
ATP5IF1	DBP	Wald ratio	1	-0.152	0.219	0.488	-	0.822	No	1	-0.152	0.219	0.488	-	0.822
ATRAID	DBP	Inverse variance weighted	3	-0.127	0.139	0.361	0.076	0.738	No	3	-0.127	0.139	0.361	0.076	0.739
ATRN	DBP	Inverse variance weighted	5	0.070	0.038	0.067	0.697	0.325	No	5	0.070	0.038	0.067	0.697	0.326
ATXN10	DBP	Wald ratio	1	0.668	0.263	0.011	-	0.103	No	1	0.668	0.263	0.011	-	0.104
ATXN2L	DBP	Wald ratio	1	1.970	0.573	0.001	-	0.011	Yes	1	1.970	0.573	0.001	-	0.011
ATXN3	DBP	Inverse variance weighted	4	-0.177	0.196	0.365	0.000	0.743	No	4	-0.177	0.196	0.365	0.000	0.744
AXIN1	DBP	Wald ratio	1	-0.441	0.518	0.395	-	0.767	No	1	-0.441	0.518	0.395	-	0.768
AXL	DBP	Inverse variance weighted	2	-0.440	0.131	0.001	-	0.014	Yes	2	-0.440	0.131	0.001	-	0.014
AZU1	DBP	Inverse variance weighted	3	-0.121	0.257	0.638	0.005	0.899	No	3	-0.121	0.257	0.638	0.005	0.899
B3GNT7	DBP	Inverse variance weighted	6	0.063	0.043	0.140	0.206	0.492	No	6	0.063	0.043	0.140	0.206	0.493
B4GALT1	DBP	Wald ratio	1	-0.117	0.119	0.327	-	0.717	No	1	-0.117	0.119	0.327	-	0.718
B4GAT1	DBP	Inverse variance weighted	3	-0.195	0.244	0.425	0.001	0.787	No	3	-0.195	0.244	0.425	0.001	0.787
BACH1	DBP	Wald ratio	1	-0.104	0.507	0.838	-	0.966	No	1	-0.104	0.507	0.838	-	0.966
BAG3	DBP	Inverse variance weighted	2	0.075	0.551	0.892	-	0.982	No	2	0.075	0.551	0.892	-	0.982
BAG4	DBP	Wald ratio	1	-2.571	0.635	0.000	-	0.002	Yes	1	-2.571	0.635	0.000	-	0.002
BAIAP2	DBP	Wald ratio	1	0.071	0.351	0.840	-	0.966	No	1	0.071	0.351	0.840	-	0.967

ST3: MR causal estimates for plasma proteins on diastolic blood pressure.

Causal candidates prioritized for DBP were marked as "Yes" in column "Prioritized". Effect of plasma protein levels on blood pressure is in mmHg unit.

										Steiger filtering					
Exposure	Outocme	Method	nsnp	Beta	SE	P-value	Cochran's Q P-value	FDR-corrected P-value	Prioritized	nsnp	Beta	SE	P-value	Cochran's Q P-value	FDR-corrected P-value
BAMBI	DBP	Wald ratio	1	-0.430	0.546	0.430	-	0.791	No	1	-0.430	0.546	0.430	-	0.792
BANK1	DBP	Inverse variance weighted	4	-0.217	0.391	0.579	0.001	0.870	No	4	-0.217	0.391	0.579	0.001	0.870
BAP18	DBP	Wald ratio	1	0.390	0.442	0.378	-	0.751	No	1	0.390	0.442	0.378	-	0.752
BCAM	DBP	Inverse variance weighted	5	0.190	0.116	0.103	0.164	0.421	No	5	0.190	0.116	0.103	0.164	0.423
BCAN	DBP	Inverse variance weighted	2	0.020	0.103	0.848	-	0.971	No	2	0.020	0.103	0.848	-	0.971
BCAT1	DBP	Inverse variance weighted	6	-0.073	0.070	0.301	0.430	0.692	No	6	-0.073	0.070	0.301	0.430	0.693
BCHE	DBP	Inverse variance weighted	7	-0.084	0.045	0.062	0.895	0.309	No	7	-0.084	0.045	0.062	0.895	0.311
BCL2	DBP	Wald ratio	1	-0.219	0.474	0.644	-	0.901	No	1	-0.219	0.474	0.644	-	0.902
BCL2L15	DBP	Inverse variance weighted	2	-0.337	0.206	0.103	-	0.421	No	2	-0.337	0.206	0.103	-	0.423
BCR	DBP	Wald ratio	1	-0.146	0.403	0.718	-	0.931	No	1	-0.146	0.403	0.718	-	0.932
BDNF	DBP	Inverse variance weighted	2	0.308	0.329	0.350	-	0.731	No	2	0.308	0.329	0.350	-	0.732
BGLAP	DBP	Inverse variance weighted	2	-0.870	0.438	0.047	-	0.261	No	2	-0.870	0.438	0.047	-	0.262
BID	DBP	Inverse variance weighted	2	0.952	0.430	0.027	-	0.187	No	2	0.952	0.430	0.027	-	0.188
BIN2	DBP	Wald ratio	1	0.382	0.208	0.066	-	0.323	No	1	0.382	0.208	0.066	-	0.325
BLMH	DBP	Inverse variance weighted	4	0.022	0.049	0.655	0.240	0.905	No	4	0.022	0.049	0.655	0.240	0.906
BLNK	DBP	Wald ratio	1	0.101	0.425	0.813	-	0.956	No	1	0.101	0.425	0.813	-	0.957
BMP10	DBP	Inverse variance weighted	2	-0.050	0.076	0.512	-	0.832	No	2	-0.050	0.076	0.512	-	0.832
BMP4	DBP	Wald ratio	1	1.239	0.619	0.045	-	0.255	No	1	1.239	0.619	0.045	-	0.256
BMP6	DBP	Inverse variance weighted	3	-0.152	0.214	0.479	0.140	0.813	No	3	-0.152	0.214	0.479	0.140	0.813
BMPER	DBP	Inverse variance weighted	7	0.115	0.099	0.246	0.976	0.632	No	7	0.115	0.099	0.246	0.976	0.633
BNIP3L	DBP	Wald ratio	1	-2.004	0.464	0.000	-	0.001	Yes	1	-2.004	0.464	0.000	-	0.001
BOC	DBP	Inverse variance weighted	3	0.060	0.245	0.806	0.033	0.954	No	3	0.060	0.245	0.806	0.033	0.954
BOLA1	DBP	Wald ratio	1	0.372	0.219	0.089	-	0.392	No	1	0.372	0.219	0.089	-	0.393
BPIFA2	DBP	Inverse variance weighted	5	-0.037	0.125	0.767	0.813	0.938	No	5	-0.037	0.125	0.767	0.813	0.939
BPIFB1	DBP	Inverse variance weighted	6	0.003	0.102	0.978	0.001	0.999	No	6	0.003	0.102	0.978	0.001	0.999
BPIFB2	DBP	Inverse variance weighted	3	-0.078	0.117	0.508	0.099	0.830	No	3	-0.078	0.117	0.508	0.099	0.831
BRAP	DBP	Wald ratio	1	13.260	0.617	0.000	-	0.000	No	-	-	-	-	-	-
BRSK2	DBP	Inverse variance weighted	4	-0.117	0.043	0.007	0.446	0.080	No	4	-0.117	0.043	0.007	0.446	0.081
BSG	DBP	Wald ratio	1	0.072	0.182	0.694	-	0.929	No	1	0.072	0.182	0.694	-	0.929
BST1	DBP	Inverse variance weighted	9	-0.006	0.032	0.862	0.031	0.976	No	9	-0.006	0.032	0.862	0.031	0.976
BST2	DBP	Inverse variance weighted	3	-0.291	0.152	0.055	0.901	0.287	No	3	-0.291	0.152	0.055	0.901	0.288
BTC	DBP	Inverse variance weighted	5	-0.001	0.050	0.984	0.692	1.000	No	5	-0.001	0.050	0.984	0.692	1.000
BTD	DBP	Inverse variance weighted	24	0.031	0.036	0.393	0.192	0.767	No	24	0.031	0.036	0.393	0.192	0.768
BTN1A1	DBP	Inverse variance weighted	2	-0.504	0.232	0.030	-	0.198	No	2	-0.504	0.232	0.030	-	0.199
BTN2A1	DBP	Inverse variance weighted	5	-0.322	0.153	0.035	0.003	0.221	No	5	-0.322	0.153	0.035	0.003	0.223
BTN3A2	DBP	Inverse variance weighted	9	0.160	0.096	0.096	0.000	0.404	No	9	0.160	0.096	0.096	0.000	0.405
C19orf12	DBP	Wald ratio	1	-0.610	0.441	0.166	-	0.531	No	1	-0.610	0.441	0.166	-	0.533
C1QA	DBP	Inverse variance weighted	3	0.125	0.064	0.052	0.709	0.276	No	3	0.125	0.064	0.052	0.709	0.277
C1QL2	DBP	Inverse variance weighted	4	-0.006	0.116	0.956	0.153	0.992	No	4	-0.006	0.116	0.956	0.153	0.992
C1QTNF1	DBP	Inverse variance weighted	2	-0.068	0.099	0.492	-	0.823	No	2	-0.068	0.099	0.492	-	0.824
C1QTNF5	DBP	Wald ratio	1	0.303	0.211	0.150	-	0.498	No	1	0.303	0.211	0.150	-	0.499
C1QTNF6	DBP	Wald ratio	1	-0.187	0.344	0.587	-	0.875	No	1	-0.187	0.344	0.587	-	0.875
C1QTNF9	DBP	Inverse variance weighted	11	-0.059	0.030	0.046	0.168	0.257	No	11	-0.059	0.030	0.046	0.168	0.258
C1R	DBP	Inverse variance weighted	2	0.085	0.100	0.395	-	0.767	No	2	0.085	0.100	0.395	-	0.768
C1RL	DBP	Inverse variance weighted	12	0.047	0.043	0.277	0.405	0.670	No	12	0.047	0.043	0.277	0.405	0.671
C1S	DBP	Inverse variance weighted	2	0.071	0.051	0.167	-	0.532	No	2	0.071	0.051	0.167	-	0.533
C2CD2L	DBP	Wald ratio	1	0.119	0.439	0.786	-	0.942	No	1	0.119	0.439	0.786	-	0.942
C2orf69	DBP	Wald ratio	1	0.246	0.258	0.340	-	0.724	No	1	0.246	0.258	0.340	-	0.724
C3	DBP	Wald ratio	1	0.055	0.211	0.795	-	0.947	No	1	0.055	0.211	0.795	-	0.947
C4BPB	DBP	Inverse variance weighted	3	0.002	0.113	0.983	0.986	1.000	No	3	0.002	0.113	0.983	0.986	1.000
C5	DBP	Wald ratio	1	-0.292	0.161	0.070	-	0.333	No	1	-0.292	0.161	0.070	-	0.334
C7	DBP	Inverse variance weighted	8	-0.035	0.047	0.454	0.144	0.800	No	8	-0.035	0.047	0.454	0.144	0.801
C7orf50	DBP	Inverse variance weighted	4	-0.061	0.165	0.712	0.001	0.931	No	4	-0.061	0.165	0.712	0.001	0.932
C8B	DBP	Inverse variance weighted	4	-0.046	0.037	0.212	0.674	0.589	No	4	-0.046	0.037	0.212	0.674	0.590
C9	DBP	Inverse variance weighted	3	0.204	0.222	0.358	0.667	0.737	No	3	0.204	0.222	0.358	0.667	0.738
CA1	DBP	Wald ratio	1	0.505	0.283	0.074	-	0.346	No	1	0.505	0.283	0.074	-	0.347

ST3: MR causal estimates for plasma proteins on diastolic blood pressure.

Causal candidates prioritized for DBP were marked as "Yes" in column "Prioritized". Effect of plasma protein levels on blood pressure is in mmHg unit.

										Steiger filtering					
Exposure	Outocme	Method	nsnp	Beta	SE	P-value	Cochran's Q P-value	FDR-corrected P-value	Prioritized	nsnp	Beta	SE	P-value	Cochran's Q P-value	FDR-corrected P-value
CA11	DBP	Wald ratio	1	0.022	0.406	0.957	-	0.992	No	1	0.022	0.406	0.957	-	0.992
CA12	DBP	Inverse variance weighted	3	0.332	0.080	0.000	0.588	0.001	Yes	3	0.332	0.080	0.000	0.588	0.001
CA13	DBP	Inverse variance weighted	2	0.029	0.101	0.778	-	0.939	No	2	0.029	0.101	0.778	-	0.939
CA14	DBP	Inverse variance weighted	3	0.109	0.186	0.559	0.420	0.857	No	3	0.109	0.186	0.559	0.420	0.857
CA2	DBP	Wald ratio	1	-0.048	0.236	0.838	-	0.966	No	1	-0.048	0.236	0.838	-	0.966
CA3	DBP	Inverse variance weighted	3	-0.141	0.222	0.525	0.084	0.841	No	3	-0.141	0.222	0.525	0.084	0.842
CA4	DBP	Inverse variance weighted	5	-0.097	0.127	0.443	0.006	0.796	No	5	-0.097	0.127	0.443	0.006	0.796
CA5A	DBP	Inverse variance weighted	5	-0.008	0.040	0.840	0.266	0.966	No	5	-0.008	0.040	0.840	0.266	0.967
CA6	DBP	Inverse variance weighted	7	-0.057	0.034	0.095	0.798	0.402	No	7	-0.057	0.034	0.095	0.798	0.403
CA9	DBP	Inverse variance weighted	2	-0.482	0.179	0.007	-	0.081	No	2	-0.482	0.179	0.007	-	0.082
CACNB3	DBP	Wald ratio	1	0.649	0.223	0.004	-	0.050	Yes	1	0.649	0.223	0.004	-	0.050
CACYBP	DBP	Wald ratio	1	-0.118	0.202	0.562	-	0.859	No	1	-0.118	0.202	0.562	-	0.859
CALB1	DBP	Inverse variance weighted	2	0.079	0.379	0.834	-	0.963	No	2	0.079	0.379	0.834	-	0.964
CALB2	DBP	Wald ratio	1	1.239	0.378	0.001	-	0.018	Yes	1	1.239	0.378	0.001	-	0.018
CALCA	DBP	Wald ratio	1	-0.779	0.224	0.000	-	0.010	Yes	1	-0.779	0.224	0.000	-	0.010
CALCB	DBP	Inverse variance weighted	3	0.177	0.167	0.289	0.558	0.679	No	3	0.177	0.167	0.289	0.558	0.680
CALCOCO1	DBP	Wald ratio	1	0.174	0.240	0.468	-	0.810	No	1	0.174	0.240	0.468	-	0.811
CALCOCO2	DBP	Wald ratio	1	0.909	0.426	0.033	-	0.211	No	1	0.909	0.426	0.033	-	0.212
CAMKK1	DBP	Wald ratio	1	-0.129	0.088	0.146	-	0.492	No	1	-0.129	0.088	0.146	-	0.493
CANT1	DBP	Inverse variance weighted	2	0.276	0.297	0.354	-	0.734	No	2	0.276	0.297	0.354	-	0.735
CAPG	DBP	Inverse variance weighted	7	-0.032	0.028	0.247	0.893	0.632	No	7	-0.032	0.028	0.247	0.893	0.633
CAPN3	DBP	Wald ratio	1	0.567	0.620	0.360	-	0.737	No	1	0.567	0.620	0.360	-	0.738
CAPS	DBP	Inverse variance weighted	4	0.061	0.189	0.748	0.507	0.938	No	4	0.061	0.189	0.748	0.507	0.938
CARHSP1	DBP	Wald ratio	1	0.166	0.282	0.556	-	0.855	No	1	0.166	0.282	0.556	-	0.855
CASP1	DBP	Wald ratio	1	-0.590	0.508	0.245	-	0.632	No	1	-0.590	0.508	0.245	-	0.633
CASP10	DBP	Wald ratio	1	0.009	0.127	0.943	-	0.989	No	1	0.009	0.127	0.943	-	0.989
CASP3	DBP	Wald ratio	1	-0.127	0.197	0.520	-	0.838	No	1	-0.127	0.197	0.520	-	0.838
CASP7	DBP	Inverse variance weighted	2	-0.198	1.433	0.890	-	0.982	No	2	-0.198	1.433	0.890	-	0.982
CASP8	DBP	Inverse variance weighted	2	0.111	0.135	0.413	-	0.777	No	2	0.111	0.135	0.413	-	0.778
CASP9	DBP	Wald ratio	1	0.141	0.283	0.618	-	0.891	No	1	0.141	0.283	0.618	-	0.891
CAT	DBP	Inverse variance weighted	3	-0.056	0.127	0.659	0.623	0.908	No	3	-0.056	0.127	0.659	0.623	0.908
CBLIF	DBP	Wald ratio	1	0.642	0.360	0.075	-	0.347	No	1	0.642	0.360	0.075	-	0.348
CBLN4	DBP	Inverse variance weighted	5	-0.001	0.078	0.989	0.917	1.000	No	5	-0.001	0.078	0.989	0.917	1.000
CBS	DBP	Inverse variance weighted	2	0.089	0.185	0.630	-	0.892	No	2	0.089	0.185	0.630	-	0.892
CC2D1A	DBP	Inverse variance weighted	2	0.240	0.516	0.642	-	0.900	No	2	0.240	0.516	0.642	-	0.900
CCDC134	DBP	Wald ratio	1	1.109	0.392	0.005	-	0.060	No	1	1.109	0.392	0.005	-	0.061
CCDC50	DBP	Inverse variance weighted	2	-0.068	0.147	0.645	-	0.901	No	2	-0.068	0.147	0.645	-	0.902
CCDC80	DBP	Inverse variance weighted	2	-0.448	0.325	0.168	-	0.533	No	2	-0.448	0.325	0.168	-	0.534
CCER2	DBP	Wald ratio	1	-0.859	0.587	0.143	-	0.492	No	1	-0.859	0.587	0.143	-	0.493
CCL11	DBP	Wald ratio	1	-0.128	0.246	0.603	-	0.882	No	1	-0.128	0.246	0.603	-	0.882
CCL13	DBP	Inverse variance weighted	4	0.016	0.092	0.866	0.942	0.976	No	4	0.016	0.092	0.866	0.942	0.976
CCL14	DBP	Inverse variance weighted	2	0.003	0.049	0.947	-	0.991	No	2	0.003	0.049	0.947	-	0.991
CCL15	DBP	Inverse variance weighted	5	-0.023	0.034	0.493	0.249	0.823	No	5	-0.023	0.034	0.493	0.249	0.824
CCL16	DBP	Inverse variance weighted	5	0.028	0.033	0.396	0.838	0.767	No	5	0.028	0.033	0.396	0.838	0.768
CCL17	DBP	Inverse variance weighted	2	0.040	0.101	0.694	-	0.929	No	2	0.040	0.101	0.694	-	0.929
CCL18	DBP	Inverse variance weighted	6	0.043	0.048	0.366	0.638	0.743	No	6	0.043	0.048	0.366	0.638	0.744
CCL19	DBP	Wald ratio	1	-0.248	0.329	0.451	-	0.799	No	1	-0.248	0.329	0.451	-	0.800
CCL2	DBP	Wald ratio	1	-0.265	0.392	0.500	-	0.829	No	1	-0.265	0.392	0.500	-	0.829
CCL20	DBP	Wald ratio	1	-0.157	0.272	0.564	-	0.859	No	1	-0.157	0.272	0.564	-	0.860
CCL21	DBP	Inverse variance weighted	2	-0.029	0.201	0.886	-	0.980	No	2	-0.029	0.201	0.886	-	0.980
CCL22	DBP	Inverse variance weighted	6	0.129	0.126	0.307	0.519	0.695	No	6	0.129	0.126	0.307	0.519	0.696
CCL23	DBP	Inverse variance weighted	6	0.044	0.090	0.623	0.050	0.891	No	6	0.044	0.090	0.623	0.050	0.892
CCL24	DBP	Inverse variance weighted	10	-0.023	0.034	0.503	0.073	0.829	No	10	-0.023	0.034	0.503	0.073	0.830
CCL25	DBP	Inverse variance weighted	4	-0.026	0.052	0.619	0.071	0.891	No	4	-0.026	0.052	0.619	0.071	0.891
CCL26	DBP	Inverse variance weighted	3	-0.099	0.204	0.626	0.664	0.891	No	3	-0.099	0.204	0.626	0.664	0.892
CCL27	DBP	Inverse variance weighted	2	0.166	0.323	0.608	-	0.884	No	2	0.166	0.323	0.608	-	0.885

ST3: MR causal estimates for plasma proteins on diastolic blood pressure.

Causal candidates prioritized for DBP were marked as "Yes" in column "Prioritized". Effect of plasma protein levels on blood pressure is in mmHg unit.

										Steiger filtering					
Exposure	Outocme	Method	nsnp	Beta	SE	P-value	Cochran's Q P-value	FDR-corrected P-value	Prioritized	nsnp	Beta	SE	P-value	Cochran's Q P-value	FDR-corrected P-value
CCL28	DBP	Inverse variance weighted	2	0.084	0.337	0.803	-	0.952	No	2	0.084	0.337	0.803	-	0.952
CCL3	DBP	Inverse variance weighted	4	-0.062	0.192	0.748	0.000	0.938	No	4	-0.062	0.192	0.748	0.000	0.938
CCL4	DBP	Inverse variance weighted	8	-0.030	0.072	0.673	0.730	0.914	No	8	-0.030	0.072	0.673	0.730	0.914
CCL5	DBP	Wald ratio	1	0.015	0.129	0.910	-	0.983	No	1	0.015	0.129	0.910	-	0.983
CCL7	DBP	Inverse variance weighted	2	0.065	0.222	0.771	-	0.938	No	2	0.065	0.222	0.771	-	0.939
CCL8	DBP	Inverse variance weighted	4	-0.024	0.031	0.433	0.326	0.793	No	4	-0.024	0.031	0.433	0.326	0.794
CCN1	DBP	Inverse variance weighted	3	0.121	0.175	0.487	0.352	0.822	No	3	0.121	0.175	0.487	0.352	0.822
CCN2	DBP	Inverse variance weighted	2	0.069	0.079	0.381	-	0.753	No	2	0.069	0.079	0.381	-	0.754
CCN3	DBP	Inverse variance weighted	4	0.686	0.223	0.002	0.001	0.031	No	4	0.686	0.223	0.002	0.001	0.032
CCN4	DBP	Inverse variance weighted	9	-0.011	0.043	0.795	0.912	0.947	No	9	-0.011	0.043	0.795	0.912	0.947
CCN5	DBP	Inverse variance weighted	3	-0.071	0.240	0.768	0.107	0.938	No	3	-0.071	0.240	0.768	0.107	0.939
CCND2	DBP	Wald ratio	1	-0.399	0.523	0.446	-	0.797	No	1	-0.399	0.523	0.446	-	0.798
CCS	DBP	Wald ratio	1	0.046	0.049	0.344	-	0.724	No	1	0.046	0.049	0.344	-	0.725
CD101	DBP	Inverse variance weighted	23	-0.049	0.029	0.087	0.868	0.388	No	23	-0.049	0.029	0.087	0.868	0.389
CD109	DBP	Inverse variance weighted	7	-0.014	0.028	0.628	0.392	0.892	No	7	-0.014	0.028	0.628	0.392	0.892
CD14	DBP	Wald ratio	1	-0.213	0.087	0.014	-	0.120	No	1	-0.213	0.087	0.014	-	0.121
CD160	DBP	Wald ratio	1	0.082	0.394	0.835	-	0.964	No	1	0.082	0.394	0.835	-	0.964
CD163	DBP	Wald ratio	1	-0.140	0.216	0.515	-	0.833	No	1	-0.140	0.216	0.515	-	0.833
CD164	DBP	Inverse variance weighted	3	0.184	0.482	0.703	0.000	0.931	No	3	0.184	0.482	0.703	0.000	0.932
CD164L2	DBP	Wald ratio	1	-0.135	0.052	0.010	-	0.098	No	1	-0.135	0.052	0.010	-	0.099
CD177	DBP	Inverse variance weighted	16	0.015	0.048	0.747	0.008	0.938	No	16	0.015	0.048	0.747	0.008	0.938
CD1C	DBP	Inverse variance weighted	2	-0.046	0.218	0.831	-	0.963	No	2	-0.046	0.218	0.831	-	0.963
CD2	DBP	Wald ratio	1	0.553	0.507	0.275	-	0.665	No	1	0.553	0.507	0.275	-	0.666
CD200	DBP	Inverse variance weighted	5	-0.205	0.162	0.206	0.123	0.583	No	5	-0.205	0.162	0.206	0.123	0.584
CD200R1	DBP	Inverse variance weighted	21	0.000	0.027	0.992	0.833	1.000	No	21	0.000	0.027	0.992	0.833	1.000
CD207	DBP	Inverse variance weighted	8	-0.029	0.052	0.571	0.277	0.865	No	8	-0.029	0.052	0.571	0.277	0.866
CD209	DBP	Inverse variance weighted	6	0.098	0.059	0.096	0.619	0.404	No	6	0.098	0.059	0.096	0.619	0.405
CD22	DBP	Inverse variance weighted	2	-0.226	0.094	0.016	-	0.133	No	2	-0.226	0.094	0.016	-	0.134
CD226	DBP	Inverse variance weighted	2	-0.026	0.082	0.753	-	0.938	No	2	-0.026	0.082	0.753	-	0.938
CD244	DBP	Inverse variance weighted	4	-0.025	0.080	0.751	0.263	0.938	No	4	-0.025	0.080	0.751	0.263	0.938
CD248	DBP	Inverse variance weighted	3	0.291	0.279	0.297	0.240	0.688	No	3	0.291	0.279	0.297	0.240	0.689
CD27	DBP	Inverse variance weighted	2	-0.076	0.197	0.697	-	0.931	No	2	-0.076	0.197	0.697	-	0.931
CD274	DBP	Inverse variance weighted	5	-0.055	0.074	0.460	0.301	0.805	No	5	-0.055	0.074	0.460	0.301	0.805
CD276	DBP	Inverse variance weighted	3	-0.016	0.076	0.830	0.001	0.963	No	3	-0.016	0.076	0.830	0.001	0.963
CD28	DBP	Inverse variance weighted	3	0.157	0.119	0.186	0.668	0.559	No	3	0.157	0.119	0.186	0.668	0.560
CD2AP	DBP	Inverse variance weighted	2	-0.161	0.082	0.049	-	0.265	No	2	-0.161	0.082	0.049	-	0.266
CD300A	DBP	Inverse variance weighted	2	0.221	0.153	0.150	-	0.498	No	2	0.221	0.153	0.150	-	0.499
CD300C	DBP	Inverse variance weighted	4	-0.083	0.100	0.408	0.013	0.774	No	4	-0.083	0.100	0.408	0.013	0.775
CD300E	DBP	Inverse variance weighted	3	-0.026	0.081	0.748	0.151	0.938	No	3	-0.026	0.081	0.748	0.151	0.938
CD300LF	DBP	Inverse variance weighted	11	0.019	0.023	0.416	0.623	0.778	No	11	0.019	0.023	0.416	0.623	0.779
CD300LG	DBP	Inverse variance weighted	2	-0.026	0.080	0.745	-	0.938	No	2	-0.026	0.080	0.745	-	0.938
CD302	DBP	Inverse variance weighted	3	-0.026	0.153	0.866	0.037	0.976	No	3	-0.026	0.153	0.866	0.037	0.976
CD33	DBP	Inverse variance weighted	10	0.025	0.021	0.230	0.327	0.614	No	10	0.025	0.021	0.230	0.327	0.615
CD34	DBP	Wald ratio	1	-0.526	0.330	0.111	-	0.445	No	1	-0.526	0.330	0.111	-	0.446
CD36	DBP	Inverse variance weighted	2	-0.172	0.135	0.201	-	0.577	No	2	-0.172	0.135	0.201	-	0.578
CD38	DBP	Inverse variance weighted	4	0.046	0.074	0.533	0.253	0.844	No	4	0.046	0.074	0.533	0.253	0.844
CD4	DBP	Inverse variance weighted	2	0.084	0.100	0.400	-	0.769	No	2	0.084	0.100	0.400	-	0.769
CD40	DBP	Wald ratio	1	0.026	0.063	0.683	-	0.920	No	1	0.026	0.063	0.683	-	0.920
CD46	DBP	Wald ratio	1	0.128	0.294	0.664	-	0.911	No	1	0.128	0.294	0.664	-	0.911
CD48	DBP	Inverse variance weighted	7	-0.015	0.047	0.753	0.092	0.938	No	7	-0.015	0.047	0.753	0.092	0.938
CD5	DBP	Inverse variance weighted	2	-0.157	0.264	0.553	-	0.854	No	2	-0.157	0.264	0.553	-	0.855
CD55	DBP	Inverse variance weighted	3	-0.025	0.212	0.906	0.000	0.983	No	3	-0.025	0.212	0.906	0.000	0.983
CD58	DBP	Inverse variance weighted	2	-0.282	0.135	0.037	-	0.230	No	2	-0.282	0.135	0.037	-	0.231
CD59	DBP	Inverse variance weighted	2	-0.206	0.136	0.131	-	0.481	No	2	-0.206	0.136	0.131	-	0.482
CD5L	DBP	Inverse variance weighted	4	-0.049	0.110	0.659	0.469	0.908	No	4	-0.049	0.110	0.659	0.469	0.908
CD6	DBP	Inverse variance weighted	6	-0.049	0.037	0.182	0.167	0.555	No	6	-0.049	0.037	0.182	0.167	0.556

ST3: MR causal estimates for plasma proteins on diastolic blood pressure.

Causal candidates prioritized for DBP were marked as "Yes" in column "Prioritized". Effect of plasma protein levels on blood pressure is in mmHg unit.

										Steiger filtering					
Exposure	Outocme	Method	nsnp	Beta	SE	P-value	Cochran's Q P-value	FDR-corrected P-value	Prioritized	nsnp	Beta	SE	P-value	Cochran's Q P-value	FDR-corrected P-value
CD63	DBP	Wald ratio	1	0.380	0.629	0.545	-	0.850	No	1	0.380	0.629	0.545	-	0.851
CD69	DBP	Wald ratio	1	0.218	0.448	0.627	-	0.892	No	1	0.218	0.448	0.627	-	0.892
CD7	DBP	Inverse variance weighted	3	-0.054	0.072	0.449	0.150	0.799	No	3	-0.054	0.072	0.449	0.150	0.800
CD70	DBP	Inverse variance weighted	6	-0.025	0.130	0.846	0.005	0.969	No	6	-0.025	0.130	0.846	0.005	0.969
CD72	DBP	Inverse variance weighted	2	-0.040	0.161	0.804	-	0.952	No	2	-0.040	0.161	0.804	-	0.953
CD74	DBP	Wald ratio	1	-0.334	0.431	0.439	-	0.794	No	1	-0.334	0.431	0.439	-	0.795
CD79B	DBP	Wald ratio	1	-0.190	0.147	0.195	-	0.567	No	1	-0.190	0.147	0.195	-	0.568
CD80	DBP	Inverse variance weighted	7	0.047	0.070	0.503	0.172	0.829	No	7	0.047	0.070	0.503	0.172	0.830
CD83	DBP	Inverse variance weighted	2	-0.152	0.138	0.271	-	0.660	No	2	-0.152	0.138	0.271	-	0.661
CD84	DBP	Wald ratio	1	0.073	0.198	0.712	-	0.931	No	1	0.073	0.198	0.712	-	0.932
CD86	DBP	Inverse variance weighted	4	0.046	0.214	0.829	0.152	0.963	No	4	0.046	0.214	0.829	0.152	0.963
CD8A	DBP	Inverse variance weighted	2	-0.019	0.088	0.833	-	0.963	No	2	-0.019	0.088	0.833	-	0.964
CD93	DBP	Wald ratio	1	0.491	0.615	0.424	-	0.787	No	1	0.491	0.615	0.424	-	0.787
CDA	DBP	Inverse variance weighted	5	0.005	0.104	0.964	0.001	0.993	No	5	0.005	0.104	0.964	0.001	0.993
CDC27	DBP	Wald ratio	1	0.464	0.292	0.112	-	0.447	No	1	0.464	0.292	0.112	-	0.448
CDCP1	DBP	Inverse variance weighted	5	0.000	0.171	1.000	0.006	1.000	No	5	0.000	0.171	1.000	0.006	1.000
CDH1	DBP	Wald ratio	1	-0.076	0.502	0.880	-	0.980	No	1	-0.076	0.502	0.880	-	0.980
CDH15	DBP	Inverse variance weighted	7	-0.046	0.096	0.633	0.064	0.895	No	7	-0.046	0.096	0.633	0.064	0.895
CDH17	DBP	Inverse variance weighted	6	-0.149	0.180	0.406	0.050	0.773	No	6	-0.149	0.180	0.406	0.050	0.773
CDH2	DBP	Inverse variance weighted	3	0.443	0.391	0.257	0.038	0.645	No	3	0.443	0.391	0.257	0.038	0.646
CDH23	DBP	Inverse variance weighted	2	0.277	0.170	0.102	-	0.421	No	2	0.277	0.170	0.102	-	0.423
CDH3	DBP	Inverse variance weighted	2	0.437	0.285	0.126	-	0.477	No	2	0.437	0.285	0.126	-	0.478
CDH5	DBP	Inverse variance weighted	2	-0.238	0.121	0.048	-	0.261	No	2	-0.238	0.121	0.048	-	0.262
CDH6	DBP	Inverse variance weighted	6	-0.049	0.056	0.383	0.020	0.754	No	6	-0.049	0.056	0.383	0.020	0.754
CDHR1	DBP	Inverse variance weighted	5	0.034	0.109	0.757	0.154	0.938	No	5	0.034	0.109	0.757	0.154	0.938
CDHR5	DBP	Inverse variance weighted	7	0.020	0.104	0.845	0.000	0.969	No	7	0.020	0.104	0.845	0.000	0.969
CDKN1A	DBP	Inverse variance weighted	3	-0.068	0.363	0.852	0.002	0.973	No	3	-0.068	0.363	0.852	0.002	0.973
CDNF	DBP	Inverse variance weighted	4	-0.007	0.063	0.908	0.278	0.983	No	4	-0.007	0.063	0.908	0.278	0.983
CDON	DBP	Inverse variance weighted	4	-0.096	0.156	0.537	0.630	0.846	No	4	-0.096	0.156	0.537	0.630	0.847
CEACAM1	DBP	Inverse variance weighted	4	-0.188	0.077	0.014	0.906	0.122	No	4	-0.188	0.077	0.014	0.906	0.123
CEACAM16	DBP	Inverse variance weighted	7	0.099	0.074	0.182	0.107	0.555	No	7	0.099	0.074	0.182	0.107	0.556
CEACAM19	DBP	Inverse variance weighted	3	-0.106	0.111	0.340	0.632	0.724	No	3	-0.106	0.111	0.340	0.632	0.724
CEACAM20	DBP	Inverse variance weighted	3	0.109	0.145	0.453	0.723	0.800	No	3	0.109	0.145	0.453	0.723	0.801
CEACAM21	DBP	Inverse variance weighted	8	-0.032	0.022	0.143	0.538	0.492	No	8	-0.032	0.022	0.143	0.538	0.493
CEACAM5	DBP	Inverse variance weighted	8	0.042	0.055	0.447	0.652	0.798	No	8	0.042	0.055	0.447	0.652	0.799
CEACAM6	DBP	Wald ratio	1	0.237	0.195	0.226	-	0.607	No	1	0.237	0.195	0.226	-	0.608
CEACAM8	DBP	Inverse variance weighted	3	-0.005	0.313	0.988	0.028	1.000	No	3	-0.005	0.313	0.988	0.028	1.000
CEBPB	DBP	Wald ratio	1	-0.211	0.431	0.625	-	0.891	No	1	-0.211	0.431	0.625	-	0.892
CELA2A	DBP	Inverse variance weighted	2	0.091	0.182	0.619	-	0.891	No	2	0.091	0.182	0.619	-	0.891
CELA3A	DBP	Inverse variance weighted	3	-0.166	0.186	0.373	0.046	0.749	No	3	-0.166	0.186	0.373	0.046	0.750
CELSR2	DBP	Inverse variance weighted	5	0.030	0.084	0.725	0.074	0.931	No	5	0.030	0.084	0.725	0.074	0.932
CEMIP2	DBP	Inverse variance weighted	3	0.243	0.145	0.094	0.774	0.401	No	3	0.243	0.145	0.094	0.774	0.402
CEND1	DBP	Wald ratio	1	0.863	0.638	0.176	-	0.547	No	1	0.863	0.638	0.176	-	0.548
CENPF	DBP	Wald ratio	1	-0.510	0.391	0.193	-	0.566	No	1	-0.510	0.391	0.193	-	0.567
CEP112	DBP	Inverse variance weighted	2	-0.248	0.096	0.010	-	0.095	No	2	-0.248	0.096	0.010	-	0.096
CEP152	DBP	Wald ratio	1	0.323	0.493	0.512	-	0.832	No	1	0.323	0.493	0.512	-	0.832
CEP170	DBP	Wald ratio	1	4.587	0.373	0.000	-	0.000	Yes	1	4.587	0.373	0.000	-	0.000
CEP20	DBP	Inverse variance weighted	2	-0.435	0.449	0.333	-	0.720	No	2	-0.435	0.449	0.333	-	0.721
CEP43	DBP	Wald ratio	1	0.221	0.311	0.476	-	0.812	No	1	0.221	0.311	0.476	-	0.813
CEP85	DBP	Wald ratio	1	0.478	0.330	0.148	-	0.495	No	1	0.478	0.330	0.148	-	0.496
CERT	DBP	Wald ratio	1	-0.264	0.486	0.588	-	0.875	No	1	-0.264	0.486	0.588	-	0.875
CES1	DBP	Inverse variance weighted	2	-0.028	0.046	0.543	-	0.850	No	2	-0.028	0.046	0.543	-	0.851
CES2	DBP	Inverse variance weighted	2	-0.993	0.312	0.001	-	0.024	Yes	2	-0.993	0.312	0.001	-	0.024
CES3	DBP	Inverse variance weighted	7	0.009	0.091	0.921	0.546	0.986	No	7	0.009	0.091	0.921	0.546	0.986
CETN3	DBP	Wald ratio	1	0.154	0.145	0.288	-	0.679	No	1	0.154	0.145	0.288	-	0.680
CFD	DBP	Inverse variance weighted	4	-0.017	0.125	0.889	0.510	0.981	No	4	-0.017	0.125	0.889	0.510	0.981

ST3: MR causal estimates for plasma proteins on diastolic blood pressure.

Causal candidates prioritized for DBP were marked as "Yes" in column "Prioritized". Effect of plasma protein levels on blood pressure is in mmHg unit.

										Steiger filtering					
Exposure	Outocme	Method	nsnp	Beta	SE	P-value	Cochran's Q P-value	FDR-corrected P-value	Prioritized	nsnp	Beta	SE	P-value	Cochran's Q P-value	FDR-corrected P-value
CFH	DBP	Inverse variance weighted	2	0.089	0.066	0.176	-	0.547	No	2	0.089	0.066	0.176	-	0.549
CFHR2	DBP	Inverse variance weighted	10	-0.059	0.043	0.167	0.002	0.532	No	10	-0.059	0.043	0.167	0.002	0.533
CFHR4	DBP	Inverse variance weighted	8	-0.109	0.037	0.003	0.185	0.049	Yes	8	-0.109	0.037	0.003	0.185	0.050
CFHR5	DBP	Inverse variance weighted	3	-0.085	0.115	0.461	0.002	0.806	No	3	-0.085	0.115	0.461	0.002	0.807
CFI	DBP	Inverse variance weighted	3	-0.068	0.110	0.538	0.933	0.847	No	3	-0.068	0.110	0.538	0.933	0.847
CGA	DBP	Wald ratio	1	-1.115	0.629	0.076	-	0.351	No	1	-1.115	0.629	0.076	-	0.352
CGREF1	DBP	Inverse variance weighted	8	0.040	0.064	0.530	0.019	0.843	No	8	0.040	0.064	0.530	0.019	0.844
CHAC2	DBP	Wald ratio	1	-0.376	0.166	0.023	-	0.165	No	1	-0.376	0.166	0.023	-	0.167
CHAD	DBP	Inverse variance weighted	2	-0.057	0.265	0.829	-	0.963	No	2	-0.057	0.265	0.829	-	0.963
CHCHD10	DBP	Inverse variance weighted	2	-0.302	0.309	0.329	-	0.717	No	2	-0.302	0.309	0.329	-	0.718
CHCHD6	DBP	Wald ratio	1	-0.071	0.104	0.494	-	0.825	No	1	-0.071	0.104	0.494	-	0.825
CHGA	DBP	Inverse variance weighted	2	-0.001	0.252	0.998	-	1.000	No	2	-0.001	0.252	0.998	-	1.000
CHGB	DBP	Inverse variance weighted	4	-0.020	0.039	0.619	0.714	0.891	No	4	-0.020	0.039	0.619	0.714	0.891
CHI3L1	DBP	Inverse variance weighted	8	-0.056	0.043	0.194	0.352	0.567	No	8	-0.056	0.043	0.194	0.352	0.568
CHIT1	DBP	Inverse variance weighted	11	-0.021	0.025	0.411	0.539	0.777	No	11	-0.021	0.025	0.411	0.539	0.778
CHL1	DBP	Inverse variance weighted	6	0.126	0.091	0.164	0.083	0.527	No	6	0.126	0.091	0.164	0.083	0.528
CHMP1A	DBP	Inverse variance weighted	2	1.595	0.432	0.000	-	0.005	Yes	2	1.595	0.432	0.000	-	0.005
CHMP6	DBP	Inverse variance weighted	2	0.126	0.373	0.735	-	0.938	No	2	0.126	0.373	0.735	-	0.938
CHRDL2	DBP	Inverse variance weighted	5	0.200	0.074	0.007	0.551	0.080	No	5	0.200	0.074	0.007	0.551	0.081
CIAPIN1	DBP	Wald ratio	1	-0.312	0.486	0.521	-	0.839	No	1	-0.312	0.486	0.521	-	0.839
CILP	DBP	Wald ratio	1	-0.107	0.082	0.195	-	0.567	No	1	-0.107	0.082	0.195	-	0.568
CIT	DBP	Inverse variance weighted	3	-0.048	0.284	0.867	0.842	0.976	No	3	-0.048	0.284	0.867	0.842	0.976
CKAP4	DBP	Inverse variance weighted	4	0.115	0.154	0.453	0.177	0.800	No	4	0.115	0.154	0.453	0.177	0.801
CLC	DBP	Wald ratio	1	-0.401	0.634	0.527	-	0.841	No	1	-0.401	0.634	0.527	-	0.842
CLEC10A	DBP	Inverse variance weighted	5	-0.068	0.053	0.205	0.117	0.583	No	5	-0.068	0.053	0.205	0.117	0.584
CLEC11A	DBP	Inverse variance weighted	5	-0.009	0.080	0.915	0.968	0.983	No	5	-0.009	0.080	0.915	0.968	0.983
CLEC12A	DBP	Inverse variance weighted	2	0.051	0.052	0.328	-	0.717	No	2	0.051	0.052	0.328	-	0.718
CLEC14A	DBP	Wald ratio	1	0.785	0.302	0.009	-	0.094	No	1	0.785	0.302	0.009	-	0.095
CLEC1A	DBP	Inverse variance weighted	5	-0.017	0.075	0.815	0.440	0.957	No	5	-0.017	0.075	0.815	0.440	0.957
CLEC1B	DBP	Inverse variance weighted	2	-0.001	0.156	0.996	-	1.000	No	2	-0.001	0.156	0.996	-	1.000
CLEC3B	DBP	Wald ratio	1	0.163	0.114	0.155	-	0.509	No	1	0.163	0.114	0.155	-	0.510
CLEC4A	DBP	Inverse variance weighted	6	0.094	0.046	0.042	0.946	0.247	No	6	0.094	0.046	0.042	0.946	0.249
CLEC4C	DBP	Inverse variance weighted	10	0.024	0.044	0.589	0.005	0.875	No	10	0.024	0.044	0.589	0.005	0.876
CLEC4D	DBP	Inverse variance weighted	8	-0.044	0.046	0.338	0.554	0.724	No	8	-0.044	0.046	0.338	0.554	0.724
CLEC4G	DBP	Inverse variance weighted	3	0.103	0.212	0.625	0.022	0.891	No	3	0.103	0.212	0.625	0.022	0.892
CLEC4M	DBP	Inverse variance weighted	4	0.070	0.064	0.274	0.268	0.665	No	4	0.070	0.064	0.274	0.268	0.666
CLEC5A	DBP	Inverse variance weighted	3	0.234	0.152	0.124	0.028	0.475	No	3	0.234	0.152	0.124	0.028	0.477
CLEC6A	DBP	Inverse variance weighted	3	0.000	0.065	0.999	0.110	1.000	No	3	0.000	0.065	0.999	0.110	1.000
CLEC7A	DBP	Inverse variance weighted	12	0.019	0.026	0.471	0.896	0.811	No	12	0.019	0.026	0.471	0.896	0.812
CLGN	DBP	Inverse variance weighted	12	0.083	0.037	0.026	0.209	0.180	No	12	0.083	0.037	0.026	0.209	0.181
CLIC5	DBP	Wald ratio	1	-0.598	0.193	0.002	-	0.030	Yes	1	-0.598	0.193	0.002	-	0.030
CLIP2	DBP	Wald ratio	1	0.133	0.204	0.514	-	0.832	No	1	0.133	0.204	0.514	-	0.832
CLMP	DBP	Inverse variance weighted	3	0.173	0.070	0.013	0.886	0.113	No	3	0.173	0.070	0.013	0.886	0.114
CLPP	DBP	Wald ratio	1	1.304	0.662	0.049	-	0.264	No	1	1.304	0.662	0.049	-	0.265
CLPS	DBP	Inverse variance weighted	5	0.057	0.087	0.514	0.001	0.832	No	5	0.057	0.087	0.514	0.001	0.832
CLSTN2	DBP	Inverse variance weighted	7	-0.028	0.055	0.606	0.754	0.883	No	7	-0.028	0.055	0.606	0.754	0.884
CLSTN3	DBP	Inverse variance weighted	4	0.046	0.066	0.490	0.135	0.823	No	4	0.046	0.066	0.490	0.135	0.823
CLU	DBP	Inverse variance weighted	3	0.113	0.330	0.731	0.086	0.934	No	3	0.113	0.330	0.731	0.086	0.934
CLUL1	DBP	Inverse variance weighted	7	0.011	0.038	0.767	0.734	0.938	No	7	0.011	0.038	0.767	0.734	0.939
CMC1	DBP	Wald ratio	1	-0.169	0.326	0.605	-	0.883	No	1	-0.169	0.326	0.605	-	0.883
CNDP1	DBP	Inverse variance weighted	4	-0.016	0.104	0.880	0.065	0.980	No	4	-0.016	0.104	0.880	0.065	0.980
CNP	DBP	Wald ratio	1	0.025	0.285	0.930	-	0.986	No	1	0.025	0.285	0.930	-	0.986
CNPY4	DBP	Inverse variance weighted	2	-0.148	0.306	0.628	-	0.892	No	2	-0.148	0.306	0.628	-	0.892
CNTN1	DBP	Inverse variance weighted	5	0.126	0.176	0.473	0.012	0.811	No	5	0.126	0.176	0.473	0.012	0.812
CNTN2	DBP	Inverse variance weighted	11	0.058	0.028	0.037	0.563	0.230	No	11	0.058	0.028	0.037	0.563	0.231
CNTN3	DBP	Inverse variance weighted	7	-0.014	0.076	0.854	0.920	0.973	No	7	-0.014	0.076	0.854	0.920	0.973

ST3: MR causal estimates for plasma proteins on diastolic blood pressure.

Causal candidates prioritized for DBP were marked as "Yes" in column "Prioritized". Effect of plasma protein levels on blood pressure is in mmHg unit.

										Steiger filtering					
Exposure	Outocme	Method	nsnp	Beta	SE	P-value	Cochran's Q P-value	FDR-corrected P-value	Prioritized	nsnp	Beta	SE	P-value	Cochran's Q P-value	FDR-corrected P-value
CNTN4	DBP	Inverse variance weighted	13	0.078	0.075	0.299	0.028	0.691	No	13	0.078	0.075	0.299	0.028	0.692
CNTN5	DBP	Inverse variance weighted	11	0.121	0.103	0.241	0.000	0.627	No	11	0.121	0.103	0.241	0.000	0.628
CNT-P2	DBP	Inverse variance weighted	7	0.077	0.043	0.077	0.737	0.352	No	7	0.077	0.043	0.077	0.737	0.353
CNT-P4	DBP	Wald ratio	1	0.813	0.413	0.049	-	0.264	No	1	0.813	0.413	0.049	-	0.265
COCH	DBP	Inverse variance weighted	5	-0.099	0.120	0.408	0.011	0.774	No	5	-0.099	0.120	0.408	0.011	0.775
COL15A1	DBP	Inverse variance weighted	3	0.422	0.175	0.016	0.086	0.132	No	3	0.422	0.175	0.016	0.086	0.133
COL18A1	DBP	Inverse variance weighted	3	0.072	0.266	0.787	0.019	0.943	No	3	0.072	0.266	0.787	0.019	0.943
COL1A1	DBP	Wald ratio	1	-1.881	0.515	0.000	-	0.006	Yes	1	-1.881	0.515	0.000	-	0.006
COL24A1	DBP	Inverse variance weighted	3	-0.131	0.132	0.322	0.733	0.713	No	3	-0.131	0.132	0.322	0.733	0.714
COL28A1	DBP	Inverse variance weighted	9	0.060	0.030	0.049	0.596	0.264	No	9	0.060	0.030	0.049	0.596	0.265
COL2A1	DBP	Inverse variance weighted	7	0.070	0.073	0.338	0.000	0.724	No	7	0.070	0.073	0.338	0.000	0.724
COL3A1	DBP	Inverse variance weighted	2	0.421	0.378	0.265	-	0.652	No	2	0.421	0.378	0.265	-	0.653
COL4A1	DBP	Inverse variance weighted	3	0.203	0.242	0.402	0.020	0.770	No	3	0.203	0.242	0.402	0.020	0.771
COL5A1	DBP	Inverse variance weighted	7	-0.134	0.129	0.296	0.017	0.686	No	7	-0.134	0.129	0.296	0.017	0.687
COL6A3	DBP	Inverse variance weighted	2	-0.440	0.235	0.062	-	0.307	No	2	-0.440	0.235	0.062	-	0.308
COL9A1	DBP	Inverse variance weighted	3	0.087	0.173	0.616	0.167	0.890	No	3	0.087	0.173	0.616	0.167	0.891
COLEC12	DBP	Inverse variance weighted	5	0.016	0.173	0.928	0.073	0.986	No	5	0.016	0.173	0.928	0.073	0.986
COMMD1	DBP	Wald ratio	1	-0.203	0.131	0.121	-	0.472	No	1	-0.203	0.131	0.121	-	0.474
COMP	DBP	Inverse variance weighted	4	0.164	0.112	0.141	0.373	0.492	No	4	0.164	0.112	0.141	0.373	0.493
COMT	DBP	Inverse variance weighted	2	-0.136	0.051	0.007	-	0.083	No	2	-0.136	0.051	0.007	-	0.084
COQ7	DBP	Wald ratio	1	-0.432	0.183	0.018	-	0.140	No	1	-0.432	0.183	0.018	-	0.141
CPA1	DBP	Inverse variance weighted	2	-0.179	0.437	0.683	-	0.920	No	2	-0.179	0.437	0.683	-	0.920
CPA2	DBP	Inverse variance weighted	4	-0.207	0.162	0.202	0.000	0.579	No	4	-0.207	0.162	0.202	0.000	0.580
CPA4	DBP	Inverse variance weighted	7	0.144	0.076	0.056	0.004	0.289	No	7	0.144	0.076	0.056	0.004	0.290
CPB1	DBP	Wald ratio	1	-0.380	0.213	0.074	-	0.347	No	1	-0.380	0.213	0.074	-	0.348
CPB2	DBP	Inverse variance weighted	6	0.054	0.038	0.156	0.793	0.509	No	6	0.054	0.038	0.156	0.793	0.510
CPE	DBP	Inverse variance weighted	3	-0.030	0.097	0.760	0.869	0.938	No	3	-0.030	0.097	0.760	0.869	0.938
CPM	DBP	Inverse variance weighted	5	0.130	0.201	0.519	0.040	0.837	No	5	0.130	0.201	0.519	0.040	0.837
CPOX	DBP	Inverse variance weighted	3	0.331	0.230	0.151	0.177	0.498	No	3	0.331	0.230	0.151	0.177	0.499
CPPED1	DBP	Inverse variance weighted	11	0.075	0.037	0.041	0.340	0.241	No	11	0.075	0.037	0.041	0.340	0.242
CPQ	DBP	Inverse variance weighted	13	0.017	0.053	0.744	0.578	0.938	No	13	0.017	0.053	0.744	0.578	0.938
CPTP	DBP	Wald ratio	1	-1.040	0.554	0.061	-	0.303	No	1	-1.040	0.554	0.061	-	0.304
CPVL	DBP	Inverse variance weighted	9	-0.009	0.032	0.782	0.696	0.939	No	9	-0.009	0.032	0.782	0.696	0.940
CPXM1	DBP	Inverse variance weighted	3	0.043	0.067	0.523	0.432	0.839	No	3	0.043	0.067	0.523	0.432	0.840
CPXM2	DBP	Inverse variance weighted	5	-0.104	0.048	0.032	0.773	0.206	No	5	-0.104	0.048	0.032	0.773	0.207
CR1	DBP	Inverse variance weighted	6	-0.027	0.087	0.757	0.011	0.938	No	6	-0.027	0.087	0.757	0.011	0.938
CR2	DBP	Inverse variance weighted	4	-0.023	0.155	0.880	0.124	0.980	No	4	-0.023	0.155	0.880	0.124	0.980
CRACR2A	DBP	Inverse variance weighted	4	0.155	0.124	0.210	0.998	0.588	No	4	0.155	0.124	0.210	0.998	0.590
CRADD	DBP	Wald ratio	1	0.280	0.325	0.390	-	0.764	No	1	0.280	0.325	0.390	-	0.764
CREG1	DBP	Wald ratio	1	0.024	0.130	0.853	-	0.973	No	1	0.024	0.130	0.853	-	0.973
CRELD1	DBP	Inverse variance weighted	8	0.045	0.033	0.171	0.367	0.537	No	8	0.045	0.033	0.171	0.367	0.538
CRELD2	DBP	Inverse variance weighted	8	0.027	0.098	0.779	0.006	0.939	No	8	0.027	0.098	0.779	0.006	0.939
CRH	DBP	Inverse variance weighted	4	-0.095	0.088	0.278	0.605	0.670	No	4	-0.095	0.088	0.278	0.605	0.671
CRHBP	DBP	Inverse variance weighted	3	0.015	0.042	0.713	0.555	0.931	No	3	0.015	0.042	0.713	0.555	0.932
CRIM1	DBP	Inverse variance weighted	2	0.272	0.941	0.772	-	0.938	No	2	0.272	0.941	0.772	-	0.939
CRIP2	DBP	Inverse variance weighted	2	0.272	0.168	0.105	-	0.426	No	2	0.272	0.168	0.105	-	0.428
CRISP2	DBP	Inverse variance weighted	6	-0.040	0.054	0.456	0.146	0.802	No	6	-0.040	0.054	0.456	0.146	0.802
CRISP3	DBP	Inverse variance weighted	4	0.120	0.083	0.151	0.004	0.498	No	4	0.120	0.083	0.151	0.004	0.499
CRNN	DBP	Inverse variance weighted	7	-0.038	0.036	0.284	0.989	0.676	No	7	-0.038	0.036	0.284	0.989	0.677
CRTAC1	DBP	Inverse variance weighted	8	-0.016	0.032	0.617	0.467	0.891	No	8	-0.016	0.032	0.617	0.467	0.891
CRTAM	DBP	Inverse variance weighted	5	-0.113	0.127	0.376	0.016	0.751	No	5	-0.113	0.127	0.376	0.016	0.752
CRYBB1	DBP	Wald ratio	1	-0.010	0.151	0.949	-	0.992	No	1	-0.010	0.151	0.949	-	0.992
CRYBB2	DBP	Inverse variance weighted	2	-1.006	0.411	0.014	-	0.123	No	2	-1.006	0.411	0.014	-	0.124
CRYGD	DBP	Inverse variance weighted	10	-0.016	0.035	0.652	0.344	0.903	No	10	-0.016	0.035	0.652	0.344	0.903
CRYM	DBP	Inverse variance weighted	3	0.040	0.063	0.524	0.473	0.840	No	3	0.040	0.063	0.524	0.473	0.840
CRYZL1	DBP	Wald ratio	1	-0.195	0.177	0.270	-	0.660	No	1	-0.195	0.177	0.270	-	0.660

ST3: MR causal estimates for plasma proteins on diastolic blood pressure.

Causal candidates prioritized for DBP were marked as "Yes" in column "Prioritized". Effect of plasma protein levels on blood pressure is in mmHg unit.

										Steiger filtering					
**Exposure**	**Outocme**	**Method**	**nsnp**	**Beta**	**SE**	**P-value**	**Cochran's Q P-value**	**FDR-corrected P-value**	**Prioritized**	**nsnp**	**Beta**	**SE**	**P-value**	**Cochran's Q P-value**	**FDR-corrected P-value**
CSF1	DBP	Inverse variance weighted	3	-0.018	0.171	0.918	0.137	0.985	No	3	-0.018	0.171	0.918	0.137	0.985
CSF1R	DBP	Inverse variance weighted	4	-0.098	0.089	0.269	0.389	0.657	No	4	-0.098	0.089	0.269	0.389	0.658
CSF2	DBP	Inverse variance weighted	2	0.113	0.373	0.762	-	0.938	No	2	0.113	0.373	0.762	-	0.939
CSF2RB	DBP	Inverse variance weighted	8	0.009	0.021	0.666	0.564	0.911	No	8	0.009	0.021	0.666	0.564	0.911
CSF3	DBP	Wald ratio	1	0.179	0.304	0.555	-	0.854	No	1	0.179	0.304	0.555	-	0.855
CSF3R	DBP	Inverse variance weighted	8	-0.010	0.067	0.887	0.508	0.980	No	8	-0.010	0.067	0.887	0.508	0.980
CSPG4	DBP	Inverse variance weighted	3	0.083	0.202	0.681	0.663	0.918	No	3	0.083	0.202	0.681	0.663	0.918
CST1	DBP	Inverse variance weighted	5	-0.020	0.054	0.706	0.129	0.931	No	5	-0.020	0.054	0.706	0.129	0.932
CST3	DBP	Inverse variance weighted	2	0.096	0.086	0.264	-	0.652	No	2	0.096	0.086	0.264	-	0.653
CST5	DBP	Inverse variance weighted	7	-0.084	0.037	0.025	0.211	0.172	No	7	-0.084	0.037	0.025	0.211	0.173
CST6	DBP	Inverse variance weighted	2	0.395	0.379	0.297	-	0.688	No	2	0.395	0.379	0.297	-	0.689
CST7	DBP	Inverse variance weighted	9	0.007	0.030	0.824	0.389	0.962	No	9	0.007	0.030	0.824	0.389	0.962
CSTB	DBP	Inverse variance weighted	5	0.040	0.068	0.553	0.002	0.854	No	5	0.040	0.068	0.553	0.002	0.855
CTBS	DBP	Inverse variance weighted	3	0.033	0.056	0.555	0.194	0.854	No	3	0.033	0.056	0.555	0.194	0.855
CTF1	DBP	Wald ratio	1	-2.742	0.474	0.000	-	0.000	Yes	1	-2.742	0.474	0.000	-	0.000
CTHRC1	DBP	Wald ratio	1	-0.498	0.434	0.251	-	0.637	No	1	-0.498	0.434	0.251	-	0.638
CTRB1	DBP	Inverse variance weighted	6	-0.027	0.044	0.532	0.497	0.844	No	6	-0.027	0.044	0.532	0.497	0.844
CTRC	DBP	Inverse variance weighted	5	0.134	0.088	0.127	0.149	0.477	No	5	0.134	0.088	0.127	0.149	0.479
CTRL	DBP	Wald ratio	1	0.470	0.324	0.146	-	0.492	No	1	0.470	0.324	0.146	-	0.494
CTSB	DBP	Inverse variance weighted	3	-0.211	0.137	0.124	0.000	0.475	No	3	-0.211	0.137	0.124	0.000	0.477
CTSC	DBP	Inverse variance weighted	7	-0.043	0.039	0.265	0.365	0.652	No	7	-0.043	0.039	0.265	0.365	0.653
CTSD	DBP	Inverse variance weighted	5	0.077	0.072	0.285	0.306	0.677	No	5	0.077	0.072	0.285	0.306	0.678
CTSE	DBP	Inverse variance weighted	4	0.052	0.107	0.625	0.001	0.891	No	4	0.052	0.107	0.625	0.001	0.892
CTSF	DBP	Wald ratio	1	-0.122	0.124	0.325	-	0.716	No	1	-0.122	0.124	0.325	-	0.717
CTSH	DBP	Inverse variance weighted	12	-0.091	0.037	0.015	0.032	0.124	No	12	-0.091	0.037	0.015	0.032	0.125
CTSO	DBP	Inverse variance weighted	3	-0.292	0.112	0.009	0.991	0.095	No	3	-0.292	0.112	0.009	0.991	0.096
CTSS	DBP	Inverse variance weighted	4	0.012	0.050	0.807	0.711	0.954	No	4	0.012	0.050	0.807	0.711	0.954
CTSV	DBP	Inverse variance weighted	3	-0.164	0.107	0.126	0.885	0.477	No	3	-0.164	0.107	0.126	0.885	0.478
CTSZ	DBP	Inverse variance weighted	2	0.223	0.088	0.011	-	0.103	No	2	0.223	0.088	0.011	-	0.104
CWC15	DBP	Wald ratio	1	0.044	0.391	0.911	-	0.983	No	1	0.044	0.391	0.911	-	0.983
CX3CL1	DBP	Wald ratio	1	0.226	0.112	0.044	-	0.255	No	1	0.226	0.112	0.044	-	0.256
CXADR	DBP	Inverse variance weighted	2	-0.135	0.194	0.487	-	0.822	No	2	-0.135	0.194	0.487	-	0.822
CXCL1	DBP	Inverse variance weighted	5	-0.073	0.062	0.237	0.050	0.624	No	5	-0.073	0.062	0.237	0.050	0.625
CXCL10	DBP	Inverse variance weighted	3	-0.594	0.254	0.019	0.666	0.148	No	3	-0.594	0.254	0.019	0.666	0.148
CXCL11	DBP	Inverse variance weighted	3	0.050	0.087	0.562	0.376	0.859	No	3	0.050	0.087	0.562	0.376	0.859
CXCL12	DBP	Inverse variance weighted	2	0.102	0.145	0.482	-	0.816	No	2	0.102	0.145	0.482	-	0.817
CXCL13	DBP	Wald ratio	1	-0.249	0.463	0.591	-	0.876	No	1	-0.249	0.463	0.591	-	0.876
CXCL14	DBP	Inverse variance weighted	2	0.442	0.301	0.142	-	0.492	No	2	0.442	0.301	0.142	-	0.493
CXCL16	DBP	Inverse variance weighted	4	-0.237	0.244	0.331	0.205	0.719	No	4	-0.237	0.244	0.331	0.205	0.720
CXCL17	DBP	Inverse variance weighted	2	0.697	0.299	0.020	-	0.149	No	2	0.697	0.299	0.020	-	0.150
CXCL5	DBP	Inverse variance weighted	3	0.077	0.061	0.208	0.603	0.585	No	3	0.077	0.061	0.208	0.603	0.586
CXCL6	DBP	Inverse variance weighted	4	0.059	0.042	0.162	0.728	0.524	No	4	0.059	0.042	0.162	0.728	0.526
CXCL8	DBP	Inverse variance weighted	2	0.202	0.264	0.444	-	0.796	No	2	0.202	0.264	0.444	-	0.797
CXCL9	DBP	Wald ratio	1	-0.076	0.175	0.664	-	0.911	No	1	-0.076	0.175	0.664	-	0.911
CYB5A	DBP	Wald ratio	1	0.189	0.658	0.774	-	0.938	No	1	0.189	0.658	0.774	-	0.939
CYB5R2	DBP	Inverse variance weighted	6	-0.021	0.084	0.808	0.312	0.954	No	6	-0.021	0.084	0.808	0.312	0.954
CYTL1	DBP	Inverse variance weighted	6	0.049	0.042	0.247	0.463	0.632	No	6	0.049	0.042	0.247	0.463	0.633
DAG1	DBP	Wald ratio	1	-2.358	0.389	0.000	-	0.000	Yes	1	-2.358	0.389	0.000	-	0.000
DAPK2	DBP	Inverse variance weighted	2	-0.016	0.060	0.787	-	0.943	No	2	-0.016	0.060	0.787	-	0.943
DAPP1	DBP	Wald ratio	1	-0.070	0.232	0.764	-	0.938	No	1	-0.070	0.232	0.764	-	0.939
DARS1	DBP	Wald ratio	1	-1.501	0.374	0.000	-	0.002	Yes	1	-1.501	0.374	0.000	-	0.002
DBH	DBP	Inverse variance weighted	12	0.032	0.041	0.430	0.000	0.791	No	12	0.032	0.041	0.430	0.000	0.792
DBI	DBP	Inverse variance weighted	2	0.010	0.088	0.905	-	0.983	No	2	0.010	0.088	0.905	-	0.983
DBN1	DBP	Wald ratio	1	-1.341	0.452	0.003	-	0.043	Yes	1	-1.341	0.452	0.003	-	0.044
DBNL	DBP	Wald ratio	1	0.397	0.359	0.268	-	0.656	No	1	0.397	0.359	0.268	-	0.657
DCBLD2	DBP	Inverse variance weighted	3	-0.047	0.072	0.513	0.350	0.832	No	3	-0.047	0.072	0.513	0.350	0.832

ST3: MR causal estimates for plasma proteins on diastolic blood pressure.

Causal candidates prioritized for DBP were marked as "Yes" in column "Prioritized". Effect of plasma protein levels on blood pressure is in mmHg unit.

										Steiger filtering					
**Exposure**	**Outocme**	**Method**	**nsnp**	**Beta**	**SE**	**P-value**	**Cochran's Q P-value**	**FDR-corrected P-value**	**Prioritized**	**nsnp**	**Beta**	**SE**	**P-value**	**Cochran's Q P-value**	**FDR-corrected P-value**
DCC	DBP	Inverse variance weighted	2	0.001	0.099	0.995	-	1.000	No	2	0.001	0.099	0.995	-	1.000
DCN	DBP	Wald ratio	1	-0.671	0.448	0.134	-	0.485	No	1	-0.671	0.448	0.134	-	0.486
DCTD	DBP	Inverse variance weighted	2	-0.159	0.340	0.640	-	0.900	No	2	-0.159	0.340	0.640	-	0.900
DCTPP1	DBP	Inverse variance weighted	2	-0.203	0.208	0.328	-	0.717	No	2	-0.203	0.208	0.328	-	0.718
DCXR	DBP	Wald ratio	1	0.447	0.329	0.174	-	0.543	No	1	0.447	0.329	0.174	-	0.544
DDAH1	DBP	Inverse variance weighted	4	0.041	0.191	0.832	0.092	0.963	No	4	0.041	0.191	0.832	0.092	0.963
DDC	DBP	Inverse variance weighted	3	-0.066	0.062	0.289	0.988	0.679	No	3	-0.066	0.062	0.289	0.988	0.680
DDHD2	DBP	Wald ratio	1	-0.984	0.240	0.000	-	0.001	Yes	1	-0.984	0.240	0.000	-	0.001
DDI2	DBP	Wald ratio	1	0.563	0.505	0.264	-	0.652	No	1	0.563	0.505	0.264	-	0.653
DDT	DBP	Wald ratio	1	-0.073	0.121	0.546	-	0.850	No	1	-0.073	0.121	0.546	-	0.851
DDX58	DBP	Inverse variance weighted	4	0.107	0.137	0.434	0.036	0.793	No	4	0.107	0.137	0.434	0.036	0.794
DECR1	DBP	Inverse variance weighted	2	0.404	0.350	0.250	-	0.635	No	2	0.404	0.350	0.250	-	0.636
DENR	DBP	Wald ratio	1	-2.613	0.693	0.000	-	0.004	Yes	1	-2.613	0.693	0.000	-	0.004
DGCR6	DBP	Wald ratio	1	0.127	0.191	0.506	-	0.829	No	1	0.127	0.191	0.506	-	0.830
DGKA	DBP	Wald ratio	1	-0.321	0.667	0.630	-	0.892	No	1	-0.321	0.667	0.630	-	0.892
DHRS4L2	DBP	Inverse variance weighted	5	-0.092	0.075	0.221	0.691	0.600	No	5	-0.092	0.075	0.221	0.691	0.601
DIABLO	DBP	Wald ratio	1	0.097	0.671	0.885	-	0.980	No	1	0.097	0.671	0.885	-	0.980
DKK1	DBP	Inverse variance weighted	3	-0.334	0.236	0.157	0.178	0.512	No	3	-0.334	0.236	0.157	0.178	0.513
DKK3	DBP	Inverse variance weighted	3	-0.076	0.121	0.526	0.018	0.841	No	3	-0.076	0.121	0.526	0.018	0.842
DKK4	DBP	Inverse variance weighted	2	0.037	0.109	0.737	-	0.938	No	2	0.037	0.109	0.737	-	0.938
DKKL1	DBP	Inverse variance weighted	16	0.002	0.021	0.904	0.549	0.983	No	16	0.002	0.021	0.904	0.549	0.983
DLK1	DBP	Inverse variance weighted	6	0.072	0.079	0.363	0.028	0.741	No	6	0.072	0.079	0.363	0.028	0.742
DLL1	DBP	Inverse variance weighted	3	-0.278	0.140	0.047	0.349	0.261	No	3	-0.278	0.140	0.047	0.349	0.262
DMP1	DBP	Wald ratio	1	-0.217	0.477	0.649	-	0.902	No	1	-0.217	0.477	0.649	-	0.902
D-JA4	DBP	Inverse variance weighted	3	0.085	0.164	0.602	0.765	0.881	No	3	0.085	0.164	0.602	0.765	0.881
D-JB1	DBP	Wald ratio	1	-0.273	0.461	0.553	-	0.854	No	1	-0.273	0.461	0.553	-	0.855
D-JB14	DBP	Wald ratio	1	-0.660	0.554	0.234	-	0.620	No	1	-0.660	0.554	0.234	-	0.621
D-JB2	DBP	Wald ratio	1	0.575	0.708	0.417	-	0.778	No	1	0.575	0.708	0.417	-	0.779
D-JB6	DBP	Wald ratio	1	-0.195	0.122	0.111	-	0.445	No	1	-0.195	0.122	0.111	-	0.446
D-JC9	DBP	Wald ratio	1	2.046	0.761	0.007	-	0.083	No	1	2.046	0.761	0.007	-	0.084
DNER	DBP	Inverse variance weighted	5	-0.274	0.086	0.001	0.193	0.024	Yes	5	-0.274	0.086	0.001	0.193	0.024
DNM1	DBP	Inverse variance weighted	3	-0.023	0.089	0.795	0.858	0.947	No	3	-0.023	0.089	0.795	0.858	0.947
DNMBP	DBP	Inverse variance weighted	2	-0.086	0.146	0.557	-	0.856	No	2	-0.086	0.146	0.557	-	0.856
DNPEP	DBP	Inverse variance weighted	2	-0.316	0.240	0.187	-	0.561	No	2	-0.316	0.240	0.187	-	0.562
DNPH1	DBP	Inverse variance weighted	2	0.081	0.572	0.887	-	0.980	No	2	0.081	0.572	0.887	-	0.980
DOC2B	DBP	Wald ratio	1	0.477	0.388	0.219	-	0.599	No	1	0.477	0.388	0.219	-	0.600
DOK2	DBP	Wald ratio	1	-1.311	0.424	0.002	-	0.031	Yes	1	-1.311	0.424	0.002	-	0.031
DPEP1	DBP	Inverse variance weighted	8	-0.199	0.042	0.000	0.039	0.000	Yes	8	-0.199	0.042	0.000	0.039	0.000
DPEP2	DBP	Inverse variance weighted	7	0.212	0.131	0.105	0.377	0.426	No	7	0.212	0.131	0.105	0.377	0.427
DPP10	DBP	Inverse variance weighted	3	0.004	0.205	0.984	0.001	1.000	No	3	0.004	0.205	0.984	0.001	1.000
DPP4	DBP	Inverse variance weighted	2	-0.399	0.108	0.000	-	0.005	Yes	2	-0.399	0.108	0.000	-	0.005
DPP6	DBP	Inverse variance weighted	3	0.088	0.115	0.443	0.213	0.796	No	3	0.088	0.115	0.443	0.213	0.796
DPP7	DBP	Wald ratio	1	-0.034	0.368	0.927	-	0.986	No	1	-0.034	0.368	0.927	-	0.986
DPT	DBP	Inverse variance weighted	6	-0.093	0.064	0.145	0.791	0.492	No	6	-0.093	0.064	0.145	0.791	0.493
DPY30	DBP	Wald ratio	1	-0.937	0.408	0.022	-	0.158	No	1	-0.937	0.408	0.022	-	0.159
DRAXIN	DBP	Inverse variance weighted	5	0.069	0.065	0.283	0.375	0.676	No	5	0.069	0.065	0.283	0.375	0.677
DSC2	DBP	Inverse variance weighted	2	0.035	0.106	0.743	-	0.938	No	2	0.035	0.106	0.743	-	0.938
DSCAM	DBP	Inverse variance weighted	7	-0.070	0.054	0.197	0.509	0.567	No	7	-0.070	0.054	0.197	0.509	0.568
DSG2	DBP	Inverse variance weighted	4	-0.014	0.206	0.947	0.004	0.991	No	4	-0.014	0.206	0.947	0.004	0.991
DSG3	DBP	Inverse variance weighted	4	-0.002	0.117	0.986	0.204	1.000	No	4	-0.002	0.117	0.986	0.204	1.000
DSG4	DBP	Wald ratio	1	0.444	0.455	0.330	-	0.718	No	1	0.444	0.455	0.330	-	0.719
DTD1	DBP	Wald ratio	1	-0.141	0.152	0.350	-	0.731	No	1	-0.141	0.152	0.350	-	0.732
DTNB	DBP	Wald ratio	1	0.599	0.685	0.381	-	0.753	No	1	0.599	0.685	0.381	-	0.754
DTX3	DBP	Wald ratio	1	0.718	0.262	0.006	-	0.076	No	1	0.718	0.262	0.006	-	0.077
DTYMK	DBP	Inverse variance weighted	2	0.019	0.351	0.957	-	0.992	No	2	0.019	0.351	0.957	-	0.992
DUSP13	DBP	Wald ratio	1	-0.346	0.089	0.000	-	0.003	Yes	1	-0.346	0.089	0.000	-	0.003

ST3: MR causal estimates for plasma proteins on diastolic blood pressure.

Causal candidates prioritized for DBP were marked as "Yes" in column "Prioritized". Effect of plasma protein levels on blood pressure is in mmHg unit.

										Steiger filtering					
**Exposure**	**Outocme**	**Method**	**nsnp**	**Beta**	**SE**	**P-value**	**Cochran's Q P-value**	**FDR-corrected P-value**	**Prioritized**	**nsnp**	**Beta**	**SE**	**P-value**	**Cochran's Q P-value**	**FDR-corrected P-value**
DUSP29	DBP	Wald ratio	1	-0.376	0.182	0.039	-	0.237	No	1	-0.376	0.182	0.039	-	0.238
DYNLT1	DBP	Wald ratio	1	0.027	0.289	0.925	-	0.986	No	1	0.027	0.289	0.925	-	0.986
EBAG9	DBP	Wald ratio	1	0.034	0.359	0.924	-	0.986	No	1	0.034	0.359	0.924	-	0.986
ECE1	DBP	Inverse variance weighted	2	0.096	0.629	0.878	-	0.980	No	2	0.096	0.629	0.878	-	0.980
ECHDC3	DBP	Inverse variance weighted	8	-0.074	0.040	0.066	0.625	0.323	No	8	-0.074	0.040	0.066	0.625	0.325
ECHS1	DBP	Inverse variance weighted	2	0.014	0.115	0.900	-	0.983	No	2	0.014	0.115	0.900	-	0.983
ECI2	DBP	Inverse variance weighted	2	-0.051	0.104	0.622	-	0.891	No	2	-0.051	0.104	0.622	-	0.892
ECM1	DBP	Inverse variance weighted	3	0.053	0.040	0.190	0.258	0.563	No	3	0.053	0.040	0.190	0.258	0.564
EDAR	DBP	Inverse variance weighted	7	0.006	0.072	0.936	0.053	0.986	No	7	0.006	0.072	0.936	0.053	0.986
EDDM3B	DBP	Wald ratio	1	-0.019	0.150	0.900	-	0.983	No	1	-0.019	0.150	0.900	-	0.983
EDIL3	DBP	Inverse variance weighted	2	-0.334	0.298	0.263	-	0.652	No	2	-0.334	0.298	0.263	-	0.653
EDN1	DBP	Wald ratio	1	0.157	0.105	0.137	-	0.490	No	1	0.157	0.105	0.137	-	0.491
EFCAB14	DBP	Inverse variance weighted	2	-0.686	0.342	0.045	-	0.255	No	2	-0.686	0.342	0.045	-	0.256
EFEMP1	DBP	Inverse variance weighted	2	-0.072	0.236	0.759	-	0.938	No	2	-0.072	0.236	0.759	-	0.938
EFHD1	DBP	Inverse variance weighted	5	0.177	0.197	0.370	0.016	0.747	No	5	0.177	0.197	0.370	0.016	0.748
EF-1	DBP	Wald ratio	1	0.252	0.074	0.001	-	0.012	Yes	1	0.252	0.074	0.001	-	0.012
EF-4	DBP	Wald ratio	1	0.227	0.432	0.600	-	0.880	No	1	0.227	0.432	0.600	-	0.880
EGF	DBP	Wald ratio	1	0.192	0.185	0.300	-	0.691	No	1	0.192	0.185	0.300	-	0.692
EGFL7	DBP	Inverse variance weighted	4	-0.104	0.173	0.546	0.136	0.851	No	4	-0.104	0.173	0.546	0.136	0.851
EGFLAM	DBP	Inverse variance weighted	5	-0.046	0.037	0.206	0.618	0.583	No	5	-0.046	0.037	0.206	0.618	0.584
EGFR	DBP	Inverse variance weighted	4	-0.191	0.124	0.123	0.370	0.475	No	4	-0.191	0.124	0.123	0.370	0.477
EGLN1	DBP	Inverse variance weighted	2	0.175	0.172	0.309	-	0.698	No	2	0.175	0.172	0.309	-	0.699
EHBP1	DBP	Wald ratio	1	-0.115	0.137	0.400	-	0.769	No	1	-0.115	0.137	0.400	-	0.769
EHD3	DBP	Wald ratio	1	-0.287	0.134	0.032	-	0.206	No	1	-0.287	0.134	0.032	-	0.207
EIF2AK2	DBP	Wald ratio	1	-0.225	0.154	0.144	-	0.492	No	1	-0.225	0.154	0.144	-	0.493
EIF2AK3	DBP	Wald ratio	1	0.421	0.201	0.036	-	0.223	No	1	0.421	0.201	0.036	-	0.225
EIF4G3	DBP	Wald ratio	1	1.248	0.305	0.000	-	0.001	Yes	1	1.248	0.305	0.000	-	0.001
EIF5	DBP	Wald ratio	1	-0.446	0.367	0.224	-	0.605	No	1	-0.446	0.367	0.224	-	0.606
ELAC1	DBP	Inverse variance weighted	2	0.236	0.238	0.322	-	0.713	No	2	0.236	0.238	0.322	-	0.714
ELN	DBP	Inverse variance weighted	2	0.212	0.271	0.435	-	0.793	No	2	0.212	0.271	0.435	-	0.794
ELOA	DBP	Wald ratio	1	1.087	0.289	0.000	-	0.004	Yes	1	1.087	0.289	0.000	-	0.005
E-H	DBP	Wald ratio	1	0.096	0.221	0.664	-	0.911	No	1	0.096	0.221	0.664	-	0.911
ENDOU	DBP	Inverse variance weighted	3	0.130	0.115	0.256	0.095	0.643	No	3	0.130	0.115	0.256	0.095	0.644
ENG	DBP	Inverse variance weighted	2	-0.092	0.093	0.325	-	0.717	No	2	-0.092	0.093	0.325	-	0.718
ENO1	DBP	Inverse variance weighted	2	-0.360	0.309	0.243	-	0.629	No	2	-0.360	0.309	0.243	-	0.630
ENO2	DBP	Inverse variance weighted	2	0.058	0.165	0.724	-	0.931	No	2	0.058	0.165	0.724	-	0.932
ENO3	DBP	Wald ratio	1	0.245	0.258	0.342	-	0.724	No	1	0.245	0.258	0.342	-	0.725
ENPEP	DBP	Inverse variance weighted	2	-2.334	0.473	0.000	-	0.000	Yes	2	-2.334	0.473	0.000	-	0.000
ENPP2	DBP	Inverse variance weighted	5	0.009	0.123	0.943	0.124	0.989	No	5	0.009	0.123	0.943	0.124	0.989
ENPP5	DBP	Inverse variance weighted	15	-0.030	0.029	0.313	0.382	0.703	No	15	-0.030	0.029	0.313	0.382	0.704
ENPP6	DBP	Inverse variance weighted	15	-0.084	0.065	0.193	0.609	0.567	No	15	-0.084	0.065	0.193	0.609	0.568
ENPP7	DBP	Inverse variance weighted	5	0.052	0.035	0.138	0.090	0.492	No	5	0.052	0.035	0.138	0.090	0.493
ENSA	DBP	Wald ratio	1	-0.400	0.480	0.405	-	0.772	No	1	-0.400	0.480	0.405	-	0.773
ENTPD2	DBP	Wald ratio	1	-0.312	0.131	0.017	-	0.137	No	1	-0.312	0.131	0.017	-	0.138
ENTPD5	DBP	Inverse variance weighted	8	-0.024	0.049	0.620	0.740	0.891	No	8	-0.024	0.049	0.620	0.740	0.891
ENTPD6	DBP	Inverse variance weighted	24	0.076	0.038	0.044	0.059	0.255	No	24	0.076	0.038	0.044	0.059	0.256
ENTR1	DBP	Inverse variance weighted	2	-0.281	0.186	0.130	-	0.480	No	2	-0.281	0.186	0.130	-	0.481
EPCAM	DBP	Wald ratio	1	-0.694	0.593	0.242	-	0.627	No	1	-0.694	0.593	0.242	-	0.628
EPGN	DBP	Wald ratio	1	-0.647	0.517	0.211	-	0.589	No	1	-0.647	0.517	0.211	-	0.590
EPHA1	DBP	Inverse variance weighted	3	-0.045	0.231	0.845	0.013	0.969	No	3	-0.045	0.231	0.845	0.013	0.969
EPHA2	DBP	Inverse variance weighted	2	-0.626	0.177	0.000	-	0.009	Yes	2	-0.626	0.177	0.000	-	0.009
EPHA4	DBP	Inverse variance weighted	4	-0.020	0.179	0.910	0.148	0.983	No	4	-0.020	0.179	0.910	0.148	0.983
EPHB6	DBP	Inverse variance weighted	2	0.040	0.083	0.635	-	0.897	No	2	0.040	0.083	0.635	-	0.897
EPHX2	DBP	Wald ratio	1	-0.078	0.235	0.740	-	0.938	No	1	-0.078	0.235	0.740	-	0.938
EPO	DBP	Wald ratio	1	-1.095	0.311	0.000	-	0.009	Yes	1	-1.095	0.311	0.000	-	0.009
EPPK1	DBP	Inverse variance weighted	13	-0.073	0.051	0.156	0.468	0.510	No	13	-0.073	0.051	0.156	0.468	0.511

ST3: MR causal estimates for plasma proteins on diastolic blood pressure.

Causal candidates prioritized for DBP were marked as "Yes" in column "Prioritized". Effect of plasma protein levels on blood pressure is in mmHg unit.

										Steiger filtering					
Exposure	Outocme	Method	nsnp	Beta	SE	P-value	Cochran's Q P-value	FDR-corrected P-value	Prioritized	nsnp	Beta	SE	P-value	Cochran's Q P-value	FDR-corrected P-value
EPS8L2	DBP	Inverse variance weighted	2	-0.113	0.078	0.150	-	0.498	No	2	-0.113	0.078	0.150	-	0.499
ERBB2	DBP	Inverse variance weighted	7	0.119	0.147	0.418	0.066	0.778	No	7	0.119	0.147	0.418	0.066	0.779
ERBB3	DBP	Inverse variance weighted	4	0.093	0.206	0.651	0.005	0.903	No	4	0.093	0.206	0.651	0.005	0.903
ERBB4	DBP	Inverse variance weighted	6	-0.208	0.155	0.179	0.015	0.551	No	6	-0.208	0.155	0.179	0.015	0.552
ERC2	DBP	Wald ratio	1	-0.079	0.430	0.855	-	0.973	No	1	-0.079	0.430	0.855	-	0.973
EREG	DBP	Wald ratio	1	0.025	0.252	0.919	-	0.985	No	1	0.025	0.252	0.919	-	0.985
ERI1	DBP	Wald ratio	1	2.671	0.327	0.000	-	0.000	Yes	1	2.671	0.327	0.000	-	0.000
ERMAP	DBP	Inverse variance weighted	2	-0.072	0.299	0.810	-	0.955	No	2	-0.072	0.299	0.810	-	0.955
ERN1	DBP	Wald ratio	1	0.451	0.209	0.031	-	0.205	No	1	0.451	0.209	0.031	-	0.207
ERP29	DBP	Wald ratio	1	14.220	0.662	0.000	-	0.000	No	-	-	-	-	-	-
ERP44	DBP	Wald ratio	1	1.306	0.525	0.013	-	0.113	No	1	1.306	0.525	0.013	-	0.114
ESAM	DBP	Wald ratio	1	-0.686	0.189	0.000	-	0.006	Yes	1	-0.686	0.189	0.000	-	0.007
ESM1	DBP	Inverse variance weighted	2	0.107	0.115	0.351	-	0.731	No	2	0.107	0.115	0.351	-	0.732
ESYT2	DBP	Inverse variance weighted	3	-0.247	0.157	0.116	0.359	0.457	No	3	-0.247	0.157	0.116	0.359	0.459
EVI5	DBP	Wald ratio	1	-0.290	0.178	0.104	-	0.424	No	1	-0.290	0.178	0.104	-	0.425
EXTL1	DBP	Wald ratio	1	-0.059	0.061	0.333	-	0.720	No	1	-0.059	0.061	0.333	-	0.721
EZR	DBP	Inverse variance weighted	2	0.183	0.224	0.415	-	0.778	No	2	0.183	0.224	0.415	-	0.779
F10	DBP	Inverse variance weighted	2	0.006	0.155	0.969	-	0.996	No	2	0.006	0.155	0.969	-	0.996
F11	DBP	Inverse variance weighted	4	0.065	0.057	0.260	0.602	0.648	No	4	0.065	0.057	0.260	0.602	0.649
F11R	DBP	Wald ratio	1	0.448	0.417	0.282	-	0.676	No	1	0.448	0.417	0.282	-	0.676
F12	DBP	Inverse variance weighted	6	0.118	0.023	0.000	0.377	0.000	Yes	6	0.118	0.023	0.000	0.377	0.000
F13B	DBP	Inverse variance weighted	6	-0.190	0.095	0.045	0.012	0.255	No	6	-0.190	0.095	0.045	0.012	0.256
F2	DBP	Inverse variance weighted	4	-0.178	0.680	0.794	0.000	0.947	No	4	-0.178	0.680	0.794	0.000	0.947
F2R	DBP	Inverse variance weighted	2	-0.173	0.206	0.401	-	0.769	No	2	-0.173	0.206	0.401	-	0.769
F3	DBP	Inverse variance weighted	5	-0.190	0.111	0.088	0.384	0.389	No	5	-0.190	0.111	0.088	0.384	0.390
F7	DBP	Inverse variance weighted	5	0.114	0.097	0.241	0.002	0.627	No	5	0.114	0.097	0.241	0.002	0.628
FABP1	DBP	Wald ratio	1	-0.164	0.108	0.128	-	0.480	No	1	-0.164	0.108	0.128	-	0.481
FABP2	DBP	Inverse variance weighted	2	-0.362	0.127	0.004	-	0.058	No	2	-0.362	0.127	0.004	-	0.059
FABP3	DBP	Wald ratio	1	-0.252	0.291	0.386	-	0.757	No	1	-0.252	0.291	0.386	-	0.758
FABP4	DBP	Wald ratio	1	0.394	0.271	0.146	-	0.492	No	1	0.394	0.271	0.146	-	0.493
FABP5	DBP	Wald ratio	1	0.138	0.211	0.514	-	0.832	No	1	0.138	0.211	0.514	-	0.832
FABP6	DBP	Wald ratio	1	-0.204	0.176	0.247	-	0.632	No	1	-0.204	0.176	0.247	-	0.633
FABP9	DBP	Inverse variance weighted	2	-0.088	0.443	0.843	-	0.969	No	2	-0.088	0.443	0.843	-	0.969
FADD	DBP	Wald ratio	1	-1.409	0.292	0.000	-	0.000	Yes	1	-1.409	0.292	0.000	-	0.000
FAM13A	DBP	Inverse variance weighted	3	0.048	0.136	0.723	0.385	0.931	No	3	0.048	0.136	0.723	0.385	0.932
FAM171B	DBP	Wald ratio	1	0.076	0.068	0.258	-	0.647	No	1	0.076	0.068	0.258	-	0.648
FAM172A	DBP	Inverse variance weighted	2	0.301	0.344	0.382	-	0.753	No	2	0.301	0.344	0.382	-	0.754
FAM20A	DBP	Inverse variance weighted	5	0.152	0.099	0.123	0.324	0.475	No	5	0.152	0.099	0.123	0.324	0.477
FAM3B	DBP	Inverse variance weighted	7	0.099	0.042	0.018	0.778	0.140	No	7	0.099	0.042	0.018	0.778	0.141
FAM3C	DBP	Wald ratio	1	-0.430	0.247	0.082	-	0.372	No	1	-0.430	0.247	0.082	-	0.373
FAM3D	DBP	Inverse variance weighted	3	-0.031	0.140	0.823	0.032	0.961	No	3	-0.031	0.140	0.823	0.032	0.961
FAP	DBP	Inverse variance weighted	3	0.069	0.098	0.478	0.828	0.813	No	3	0.069	0.098	0.478	0.828	0.813
FARSA	DBP	Wald ratio	1	-0.316	0.499	0.527	-	0.841	No	1	-0.316	0.499	0.527	-	0.842
FAS	DBP	Inverse variance weighted	3	-0.097	0.090	0.284	0.714	0.676	No	3	-0.097	0.090	0.284	0.714	0.677
FASLG	DBP	Wald ratio	1	0.329	0.306	0.282	-	0.675	No	1	0.329	0.306	0.282	-	0.676
FBLN2	DBP	Inverse variance weighted	5	0.055	0.150	0.717	0.025	0.931	No	5	0.055	0.150	0.717	0.025	0.932
FBN2	DBP	Inverse variance weighted	2	0.186	0.078	0.017	-	0.138	No	2	0.186	0.078	0.017	-	0.139
FBP1	DBP	Inverse variance weighted	3	0.021	0.075	0.776	0.922	0.938	No	3	0.021	0.075	0.776	0.922	0.939
FCAMR	DBP	Inverse variance weighted	3	-0.001	0.068	0.985	0.960	1.000	No	3	-0.001	0.068	0.985	0.960	1.000
FCAR	DBP	Inverse variance weighted	8	0.003	0.030	0.923	0.710	0.986	No	8	0.003	0.030	0.923	0.710	0.986
FCER1A	DBP	Inverse variance weighted	2	-0.372	0.221	0.093	-	0.400	No	2	-0.372	0.221	0.093	-	0.401
FCER2	DBP	Inverse variance weighted	3	0.151	0.074	0.040	0.314	0.239	No	3	0.151	0.074	0.040	0.314	0.241
FCGR2A	DBP	Inverse variance weighted	16	-0.012	0.024	0.604	0.842	0.882	No	16	-0.012	0.024	0.604	0.842	0.882
FCGR2B	DBP	Inverse variance weighted	4	-0.050	0.049	0.315	0.614	0.705	No	4	-0.050	0.049	0.315	0.614	0.706
FCGR3B	DBP	Inverse variance weighted	9	-0.018	0.029	0.528	0.352	0.843	No	9	-0.018	0.029	0.528	0.352	0.844
FCN1	DBP	Inverse variance weighted	4	0.098	0.068	0.147	0.216	0.495	No	4	0.098	0.068	0.147	0.216	0.496

ST3: MR causal estimates for plasma proteins on diastolic blood pressure.

Causal candidates prioritized for DBP were marked as "Yes" in column "Prioritized". Effect of plasma protein levels on blood pressure is in mmHg unit.

										Steiger filtering					
**Exposure**	**Outocme**	**Method**	**nsnp**	**Beta**	**SE**	**P-value**	**Cochran's Q P-value**	**FDR-corrected P-value**	**Prioritized**	**nsnp**	**Beta**	**SE**	**P-value**	**Cochran's Q P-value**	**FDR-corrected P-value**
FCN2	DBP	Inverse variance weighted	9	0.038	0.035	0.274	0.774	0.665	No	9	0.038	0.035	0.274	0.774	0.666
FCRL1	DBP	Inverse variance weighted	3	0.089	0.161	0.579	0.002	0.870	No	3	0.089	0.161	0.579	0.002	0.870
FCRL2	DBP	Inverse variance weighted	5	0.013	0.038	0.740	0.700	0.938	No	5	0.013	0.038	0.740	0.700	0.938
FCRL3	DBP	Inverse variance weighted	9	0.019	0.026	0.457	0.302	0.802	No	9	0.019	0.026	0.457	0.302	0.803
FCRL5	DBP	Inverse variance weighted	8	0.041	0.060	0.498	0.288	0.827	No	8	0.041	0.060	0.498	0.288	0.827
FCRL6	DBP	Inverse variance weighted	6	-0.003	0.036	0.927	0.299	0.986	No	6	-0.003	0.036	0.927	0.299	0.986
FCRLB	DBP	Inverse variance weighted	11	-0.010	0.031	0.757	0.474	0.938	No	11	-0.010	0.031	0.757	0.474	0.938
FDX1	DBP	Wald ratio	1	1.670	0.481	0.001	-	0.010	Yes	1	1.670	0.481	0.001	-	0.010
FES	DBP	Wald ratio	1	-2.348	0.177	0.000	-	0.000	Yes	1	-2.348	0.177	0.000	-	0.000
FETUB	DBP	Inverse variance weighted	11	0.061	0.049	0.213	0.833	0.590	No	11	0.061	0.049	0.213	0.833	0.591
FGA	DBP	Inverse variance weighted	3	0.077	0.172	0.655	0.459	0.905	No	3	0.077	0.172	0.655	0.459	0.906
FGF2	DBP	Inverse variance weighted	3	0.045	0.034	0.188	0.574	0.561	No	3	0.045	0.034	0.188	0.574	0.562
FGF21	DBP	Inverse variance weighted	2	0.111	0.173	0.522	-	0.839	No	2	0.111	0.173	0.522	-	0.839
FGF23	DBP	Inverse variance weighted	3	0.045	0.761	0.952	0.000	0.992	No	3	0.045	0.761	0.952	0.000	0.992
FGF5	DBP	Inverse variance weighted	8	0.781	0.044	0.000	0.201	0.000	Yes	8	0.781	0.044	0.000	0.201	0.000
FGFBP1	DBP	Inverse variance weighted	3	-0.020	0.172	0.909	0.282	0.983	No	3	-0.020	0.172	0.909	0.282	0.983
FGFBP2	DBP	Inverse variance weighted	5	0.010	0.057	0.861	0.145	0.976	No	5	0.010	0.057	0.861	0.145	0.976
FGFBP3	DBP	Inverse variance weighted	2	-0.105	0.072	0.145	-	0.492	No	2	-0.105	0.072	0.145	-	0.493
FGFR2	DBP	Inverse variance weighted	4	-0.259	0.437	0.553	0.000	0.854	No	4	-0.259	0.437	0.553	0.000	0.855
FGFR4	DBP	Inverse variance weighted	11	-0.002	0.044	0.956	0.000	0.992	No	11	-0.002	0.044	0.956	0.000	0.992
FGL1	DBP	Inverse variance weighted	7	-0.071	0.047	0.131	0.125	0.481	No	7	-0.071	0.047	0.131	0.125	0.482
FGR	DBP	Inverse variance weighted	3	0.561	0.237	0.018	0.297	0.140	No	3	0.561	0.237	0.018	0.297	0.141
FHIT	DBP	Inverse variance weighted	2	0.089	0.334	0.789	-	0.944	No	2	0.089	0.334	0.789	-	0.944
FIS1	DBP	Wald ratio	1	-0.105	0.144	0.464	-	0.807	No	1	-0.105	0.144	0.464	-	0.808
FKBP1B	DBP	Inverse variance weighted	2	0.033	0.221	0.880	-	0.980	No	2	0.033	0.221	0.880	-	0.980
FKBP4	DBP	Wald ratio	1	-0.277	0.437	0.526	-	0.841	No	1	-0.277	0.437	0.526	-	0.842
FKBP5	DBP	Wald ratio	1	-0.275	0.120	0.022	-	0.160	No	1	-0.275	0.120	0.022	-	0.161
FKBP7	DBP	Wald ratio	1	-0.373	0.349	0.284	-	0.676	No	1	-0.373	0.349	0.284	-	0.677
FLRT2	DBP	Inverse variance weighted	6	-0.012	0.095	0.895	0.000	0.982	No	6	-0.012	0.095	0.895	0.000	0.982
FLT1	DBP	Wald ratio	1	0.685	0.521	0.188	-	0.561	No	1	0.685	0.521	0.188	-	0.562
FLT3	DBP	Inverse variance weighted	2	-0.155	0.499	0.756	-	0.938	No	2	-0.155	0.499	0.756	-	0.938
FLT3LG	DBP	Inverse variance weighted	2	-0.315	0.438	0.471	-	0.811	No	2	-0.315	0.438	0.471	-	0.812
FLT4	DBP	Inverse variance weighted	3	-0.171	0.166	0.305	0.015	0.693	No	3	-0.171	0.166	0.305	0.015	0.694
FN1	DBP	Inverse variance weighted	2	0.005	0.115	0.963	-	0.993	No	2	0.005	0.115	0.963	-	0.993
FNDC1	DBP	Inverse variance weighted	7	-0.067	0.069	0.328	0.155	0.717	No	7	-0.067	0.069	0.328	0.155	0.718
FNTA	DBP	Wald ratio	1	1.233	0.480	0.010	-	0.099	No	1	1.233	0.480	0.010	-	0.100
FOLH1	DBP	Wald ratio	1	-0.003	0.265	0.991	-	1.000	No	1	-0.003	0.265	0.991	-	1.000
FOLR1	DBP	Inverse variance weighted	3	0.241	0.254	0.343	0.221	0.724	No	3	0.241	0.254	0.343	0.221	0.725
FOLR2	DBP	Inverse variance weighted	2	0.187	0.196	0.340	-	0.724	No	2	0.187	0.196	0.340	-	0.724
FOLR3	DBP	Inverse variance weighted	11	0.002	0.058	0.974	0.000	0.998	No	11	0.002	0.058	0.974	0.000	0.998
FOXJ3	DBP	Wald ratio	1	1.519	0.344	0.000	-	0.000	Yes	1	1.519	0.344	0.000	-	0.000
FOXO1	DBP	Wald ratio	1	0.315	0.206	0.126	-	0.477	No	1	0.315	0.206	0.126	-	0.478
FOXO3	DBP	Wald ratio	1	1.449	0.648	0.025	-	0.177	No	1	1.449	0.648	0.025	-	0.178
FRZB	DBP	Inverse variance weighted	6	0.051	0.082	0.534	0.013	0.845	No	6	0.051	0.082	0.534	0.013	0.845
FSHB	DBP	Wald ratio	1	0.202	0.222	0.363	-	0.741	No	1	0.202	0.222	0.363	-	0.742
FST	DBP	Inverse variance weighted	2	-0.260	0.322	0.419	-	0.778	No	2	-0.260	0.322	0.419	-	0.779
FSTL1	DBP	Inverse variance weighted	2	0.196	0.134	0.145	-	0.492	No	2	0.196	0.134	0.145	-	0.493
FSTL3	DBP	Inverse variance weighted	2	-0.072	0.281	0.797	-	0.948	No	2	-0.072	0.281	0.797	-	0.948
FTCD	DBP	Inverse variance weighted	3	0.410	0.270	0.129	0.432	0.480	No	3	0.410	0.270	0.129	0.432	0.481
FUCA1	DBP	Inverse variance weighted	3	0.254	0.107	0.018	0.054	0.140	No	3	0.254	0.107	0.018	0.054	0.141
FUOM	DBP	Inverse variance weighted	5	-0.173	0.115	0.132	0.822	0.484	No	5	-0.173	0.115	0.132	0.822	0.485
FURIN	DBP	Wald ratio	1	1.746	0.123	0.000	-	0.000	Yes	1	1.746	0.123	0.000	-	0.000
FUT1	DBP	Wald ratio	1	0.752	0.548	0.170	-	0.535	No	1	0.752	0.548	0.170	-	0.537
FUT8	DBP	Inverse variance weighted	9	0.073	0.030	0.014	0.469	0.122	No	9	0.073	0.030	0.014	0.469	0.123
FXN	DBP	Wald ratio	1	0.090	0.306	0.768	-	0.938	No	1	0.090	0.306	0.768	-	0.939
FXYD5	DBP	Wald ratio	1	-0.022	0.072	0.759	-	0.938	No	1	-0.022	0.072	0.759	-	0.938

ST3: MR causal estimates for plasma proteins on diastolic blood pressure.

Causal candidates prioritized for DBP were marked as "Yes" in column "Prioritized". Effect of plasma protein levels on blood pressure is in mmHg unit.

										Steiger filtering					
Exposure	Outocme	Method	nsnp	Beta	SE	P-value	Cochran's Q P-value	FDR-corrected P-value	Prioritized	nsnp	Beta	SE	P-value	Cochran's Q P-value	FDR-corrected P-value
GAL	DBP	Inverse variance weighted	4	0.447	0.667	0.503	0.000	0.829	No	4	0.447	0.667	0.503	0.000	0.830
GALNT10	DBP	Inverse variance weighted	3	0.127	0.107	0.235	0.039	0.620	No	3	0.127	0.107	0.235	0.039	0.621
GALNT2	DBP	Inverse variance weighted	4	0.087	0.097	0.370	0.135	0.746	No	4	0.087	0.097	0.370	0.135	0.747
GALNT3	DBP	Inverse variance weighted	4	0.045	0.083	0.583	0.157	0.870	No	4	0.045	0.083	0.583	0.157	0.871
GALNT5	DBP	Inverse variance weighted	6	0.066	0.067	0.319	0.997	0.710	No	6	0.066	0.067	0.319	0.997	0.711
GALNT7	DBP	Wald ratio	1	-0.103	0.374	0.782	-	0.939	No	1	-0.103	0.374	0.782	-	0.940
GAMT	DBP	Wald ratio	1	0.194	0.699	0.781	-	0.939	No	1	0.194	0.699	0.781	-	0.940
GART	DBP	Wald ratio	1	0.171	0.213	0.421	-	0.782	No	1	0.171	0.213	0.421	-	0.782
GAS2	DBP	Wald ratio	1	0.080	0.213	0.707	-	0.931	No	1	0.080	0.213	0.707	-	0.932
GAS6	DBP	Inverse variance weighted	2	-0.219	0.102	0.031	-	0.206	No	2	-0.219	0.102	0.031	-	0.207
GASK1A	DBP	Inverse variance weighted	9	-0.037	0.031	0.238	0.603	0.624	No	9	-0.037	0.031	0.238	0.603	0.625
GAST	DBP	Wald ratio	1	1.325	0.660	0.045	-	0.255	No	1	1.325	0.660	0.045	-	0.256
GATD3	DBP	Wald ratio	1	-0.029	0.220	0.896	-	0.982	No	1	-0.029	0.220	0.896	-	0.982
GBP1	DBP	Inverse variance weighted	6	0.176	0.066	0.007	0.157	0.083	No	6	0.176	0.066	0.007	0.157	0.084
GBP2	DBP	Inverse variance weighted	2	0.759	0.200	0.000	-	0.004	No	2	0.759	0.200	0.000	-	0.004
GBP4	DBP	Inverse variance weighted	7	-0.410	0.218	0.060	0.564	0.300	No	7	-0.410	0.218	0.060	0.564	0.301
GC	DBP	Inverse variance weighted	10	-0.018	0.023	0.434	0.268	0.793	No	10	-0.018	0.023	0.434	0.268	0.794
GCHFR	DBP	Wald ratio	1	0.486	0.208	0.019	-	0.148	No	1	0.486	0.208	0.019	-	0.148
GCLM	DBP	Wald ratio	1	0.817	0.430	0.057	-	0.293	No	1	0.817	0.430	0.057	-	0.294
GCNT1	DBP	Inverse variance weighted	8	0.030	0.083	0.720	0.004	0.931	No	8	0.030	0.083	0.720	0.004	0.932
GDF15	DBP	Inverse variance weighted	6	0.088	0.083	0.286	0.985	0.677	No	6	0.088	0.083	0.286	0.985	0.678
GDNF	DBP	Inverse variance weighted	4	-0.210	0.088	0.017	0.438	0.139	No	4	-0.210	0.088	0.017	0.438	0.140
GFAP	DBP	Inverse variance weighted	3	0.321	0.219	0.142	0.673	0.492	No	3	0.321	0.219	0.142	0.673	0.493
GFER	DBP	Wald ratio	1	-2.019	0.402	0.000	-	0.000	Yes	1	-2.019	0.402	0.000	-	0.000
GFRA1	DBP	Inverse variance weighted	5	0.130	0.135	0.334	0.096	0.722	No	5	0.130	0.135	0.334	0.096	0.722
GFRA2	DBP	Inverse variance weighted	7	0.056	0.076	0.465	0.230	0.807	No	7	0.056	0.076	0.465	0.230	0.808
GFRA3	DBP	Inverse variance weighted	2	0.126	0.326	0.700	-	0.931	No	2	0.126	0.326	0.700	-	0.932
GFRAL	DBP	Inverse variance weighted	7	-0.021	0.055	0.709	0.688	0.931	No	7	-0.021	0.055	0.709	0.688	0.932
GGACT	DBP	Inverse variance weighted	2	-0.034	0.051	0.501	-	0.829	No	2	-0.034	0.051	0.501	-	0.830
GGCT	DBP	Wald ratio	1	0.277	0.218	0.205	-	0.583	No	1	0.277	0.218	0.205	-	0.584
GGH	DBP	Inverse variance weighted	21	0.081	0.060	0.181	0.106	0.554	No	21	0.081	0.060	0.181	0.106	0.555
GGT1	DBP	Inverse variance weighted	6	-0.056	0.087	0.522	0.681	0.839	No	6	-0.056	0.087	0.522	0.681	0.839
GGT5	DBP	Inverse variance weighted	5	0.000	0.076	1.000	0.517	1.000	No	5	0.000	0.076	1.000	0.517	1.000
GHR	DBP	Inverse variance weighted	10	0.077	0.037	0.035	0.477	0.221	No	10	0.077	0.037	0.035	0.477	0.222
GHRL	DBP	Inverse variance weighted	2	0.008	0.157	0.959	-	0.993	No	2	0.008	0.157	0.959	-	0.993
GIMAP7	DBP	Inverse variance weighted	4	0.221	0.045	0.000	0.863	0.000	Yes	4	0.221	0.045	0.000	0.863	0.000
GIMAP8	DBP	Inverse variance weighted	2	-0.054	0.122	0.662	-	0.909	No	2	-0.054	0.122	0.662	-	0.910
GIP	DBP	Wald ratio	1	-0.588	0.570	0.302	-	0.693	No	1	-0.588	0.570	0.302	-	0.693
GIPC2	DBP	Inverse variance weighted	2	-0.169	0.093	0.069	-	0.332	No	2	-0.169	0.093	0.069	-	0.333
GIPC3	DBP	Inverse variance weighted	2	-0.157	0.085	0.064	-	0.315	No	2	-0.157	0.085	0.064	-	0.316
GIT1	DBP	Inverse variance weighted	2	1.391	0.542	0.010	-	0.099	No	2	1.391	0.542	0.010	-	0.100
GKN1	DBP	Wald ratio	1	-0.576	0.604	0.341	-	0.724	No	1	-0.576	0.604	0.341	-	0.724
GLB1	DBP	Wald ratio	1	-0.010	0.107	0.923	-	0.986	No	1	-0.010	0.107	0.923	-	0.986
GLO1	DBP	Inverse variance weighted	2	0.228	0.069	0.001	-	0.018	Yes	2	0.228	0.069	0.001	-	0.018
GLRX	DBP	Inverse variance weighted	7	0.000	0.053	0.994	0.145	1.000	No	7	0.000	0.053	0.994	0.145	1.000
GLRX5	DBP	Inverse variance weighted	2	-0.070	0.197	0.723	-	0.931	No	2	-0.070	0.197	0.723	-	0.932
GLT8D2	DBP	Wald ratio	1	-0.202	0.134	0.133	-	0.484	No	1	-0.202	0.134	0.133	-	0.485
GM2A	DBP	Inverse variance weighted	6	-0.027	0.035	0.450	0.605	0.799	No	6	-0.027	0.035	0.450	0.605	0.800
GMFG	DBP	Wald ratio	1	-0.251	0.358	0.483	-	0.817	No	1	-0.251	0.358	0.483	-	0.818
GMPR	DBP	Inverse variance weighted	4	-0.061	0.144	0.673	0.054	0.914	No	4	-0.061	0.144	0.673	0.054	0.914
GMPR2	DBP	Inverse variance weighted	3	0.000	0.601	1.000	0.004	1.000	No	3	0.000	0.601	1.000	0.004	1.000
G-S	DBP	Wald ratio	1	0.036	0.314	0.909	-	0.983	No	1	0.036	0.314	0.909	-	0.983
GNLY	DBP	Inverse variance weighted	5	-0.082	0.054	0.124	0.084	0.475	No	5	-0.082	0.054	0.124	0.084	0.477
GNPDA1	DBP	Inverse variance weighted	3	0.340	0.354	0.336	0.062	0.724	No	3	0.340	0.354	0.336	0.062	0.724
GNPDA2	DBP	Inverse variance weighted	4	-0.077	0.092	0.406	0.383	0.772	No	4	-0.077	0.092	0.406	0.383	0.773
GOLGA3	DBP	Inverse variance weighted	2	0.479	0.206	0.020	-	0.149	No	2	0.479	0.206	0.020	-	0.150

ST3: MR causal estimates for plasma proteins on diastolic blood pressure.

Causal candidates prioritized for DBP were marked as "Yes" in column "Prioritized". Effect of plasma protein levels on blood pressure is in mmHg unit.

										Steiger filtering					
**Exposure**	**Outocme**	**Method**	**nsnp**	**Beta**	**SE**	**P-value**	**Cochran's Q P-value**	**FDR-corrected P-value**	**Prioritized**	**nsnp**	**Beta**	**SE**	**P-value**	**Cochran's Q P-value**	**FDR-corrected P-value**
GOLM2	DBP	Inverse variance weighted	2	-0.494	0.644	0.443	-	0.796	No	2	-0.494	0.644	0.443	-	0.796
GORASP2	DBP	Wald ratio	1	2.568	0.473	0.000	-	0.000	Yes	1	2.568	0.473	0.000	-	0.000
GOT1	DBP	Wald ratio	1	-1.192	0.687	0.083	-	0.373	No	1	-1.192	0.687	0.083	-	0.375
GP1BA	DBP	Inverse variance weighted	2	0.008	0.179	0.965	-	0.994	No	2	0.008	0.179	0.965	-	0.994
GP1BB	DBP	Inverse variance weighted	2	-0.135	0.760	0.859	-	0.976	No	2	-0.135	0.760	0.859	-	0.976
GP2	DBP	Inverse variance weighted	2	0.573	0.873	0.511	-	0.832	No	2	0.573	0.873	0.511	-	0.832
GP5	DBP	Wald ratio	1	0.369	0.189	0.051	-	0.272	No	1	0.369	0.189	0.051	-	0.273
GP6	DBP	Wald ratio	1	-0.012	0.078	0.881	-	0.980	No	1	-0.012	0.078	0.881	-	0.980
GPA33	DBP	Inverse variance weighted	3	-0.155	0.181	0.390	0.117	0.764	No	3	-0.155	0.181	0.390	0.117	0.764
GPC1	DBP	Inverse variance weighted	3	0.000	0.068	0.996	0.975	1.000	No	3	0.000	0.068	0.996	0.975	1.000
GPC5	DBP	Inverse variance weighted	12	-0.027	0.030	0.378	0.289	0.751	No	12	-0.027	0.030	0.378	0.289	0.752
GPD1	DBP	Inverse variance weighted	2	0.107	0.364	0.769	-	0.938	No	2	0.107	0.364	0.769	-	0.939
GPHA2	DBP	Inverse variance weighted	4	-0.202	0.301	0.502	0.000	0.829	No	4	-0.202	0.301	0.502	0.000	0.830
GPIHBP1	DBP	Inverse variance weighted	2	-0.055	0.088	0.530	-	0.843	No	2	-0.055	0.088	0.530	-	0.844
GPNMB	DBP	Inverse variance weighted	7	0.031	0.081	0.704	0.043	0.931	No	7	0.031	0.081	0.704	0.043	0.932
GPR158	DBP	Wald ratio	1	-0.050	0.579	0.931	-	0.986	No	1	-0.050	0.579	0.931	-	0.986
GPR15L	DBP	Inverse variance weighted	4	-0.052	0.104	0.618	0.453	0.891	No	4	-0.052	0.104	0.618	0.453	0.891
GPR37	DBP	Inverse variance weighted	6	-0.051	0.033	0.118	0.779	0.459	No	6	-0.051	0.033	0.118	0.779	0.461
GPRC5C	DBP	Wald ratio	1	-0.150	0.198	0.448	-	0.799	No	1	-0.150	0.198	0.448	-	0.800
GRAP2	DBP	Wald ratio	1	-0.555	0.362	0.126	-	0.477	No	1	-0.555	0.362	0.126	-	0.478
GRHPR	DBP	Inverse variance weighted	2	0.054	0.184	0.770	-	0.938	No	2	0.054	0.184	0.770	-	0.939
GRIK2	DBP	Inverse variance weighted	7	0.026	0.086	0.762	0.937	0.938	No	7	0.026	0.086	0.762	0.937	0.939
GRK5	DBP	Wald ratio	1	0.053	0.090	0.558	-	0.857	No	1	0.053	0.090	0.558	-	0.857
GRN	DBP	Wald ratio	1	0.120	0.103	0.245	-	0.631	No	1	0.120	0.103	0.245	-	0.632
GRP	DBP	Inverse variance weighted	4	-0.304	0.083	0.000	0.220	0.005	Yes	4	-0.304	0.083	0.000	0.220	0.006
GRPEL1	DBP	Wald ratio	1	-1.240	0.487	0.011	-	0.102	No	1	-1.240	0.487	0.011	-	0.103
GSN	DBP	Inverse variance weighted	6	-0.018	0.210	0.931	0.051	0.986	No	6	-0.018	0.210	0.931	0.051	0.986
GSR	DBP	Inverse variance weighted	2	-0.152	0.195	0.436	-	0.793	No	2	-0.152	0.195	0.436	-	0.794
GSTA1	DBP	Inverse variance weighted	2	-0.105	0.072	0.143	-	0.492	No	2	-0.105	0.072	0.143	-	0.493
GSTA3	DBP	Inverse variance weighted	2	-0.106	0.073	0.145	-	0.492	No	2	-0.106	0.073	0.145	-	0.493
GSTM4	DBP	Inverse variance weighted	2	-0.338	0.060	0.000	-	0.000	Yes	2	-0.338	0.060	0.000	-	0.000
GSTP1	DBP	Inverse variance weighted	2	0.114	0.120	0.342	-	0.724	No	2	0.114	0.120	0.342	-	0.725
GSTT2B	DBP	Inverse variance weighted	15	-0.005	0.022	0.828	0.491	0.963	No	15	-0.005	0.022	0.828	0.491	0.963
GUCA2A	DBP	Inverse variance weighted	4	0.338	0.368	0.358	0.000	0.737	No	4	0.338	0.368	0.358	0.000	0.738
GUSB	DBP	Inverse variance weighted	3	0.277	0.152	0.068	0.405	0.327	No	3	0.277	0.152	0.068	0.405	0.328
GYS1	DBP	Wald ratio	1	-0.305	0.546	0.577	-	0.869	No	1	-0.305	0.546	0.577	-	0.870
GZMA	DBP	Inverse variance weighted	2	1.030	0.486	0.034	-	0.217	No	2	1.030	0.486	0.034	-	0.218
GZMB	DBP	Inverse variance weighted	2	0.007	0.074	0.927	-	0.986	No	2	0.007	0.074	0.927	-	0.986
GZMH	DBP	Inverse variance weighted	5	0.016	0.104	0.876	0.507	0.980	No	5	0.016	0.104	0.876	0.507	0.980
HADH	DBP	Wald ratio	1	1.340	0.433	0.002	-	0.031	Yes	1	1.340	0.433	0.002	-	0.031
HAGH	DBP	Inverse variance weighted	2	0.141	0.224	0.529	-	0.843	No	2	0.141	0.224	0.529	-	0.844
HAVCR1	DBP	Inverse variance weighted	6	0.030	0.085	0.722	0.007	0.931	No	6	0.030	0.085	0.722	0.007	0.932
HAVCR2	DBP	Inverse variance weighted	3	0.290	0.221	0.191	0.743	0.563	No	3	0.290	0.221	0.191	0.743	0.565
HBEGF	DBP	Inverse variance weighted	2	-0.443	0.371	0.233	-	0.620	No	2	-0.443	0.371	0.233	-	0.621
HBQ1	DBP	Inverse variance weighted	3	-0.032	0.106	0.765	0.049	0.938	No	3	-0.032	0.106	0.765	0.049	0.939
HBZ	DBP	Inverse variance weighted	11	-0.038	0.023	0.106	0.386	0.427	No	11	-0.038	0.023	0.106	0.386	0.428
HCLS1	DBP	Inverse variance weighted	2	0.222	0.264	0.400	-	0.769	No	2	0.222	0.264	0.400	-	0.769
HDDC2	DBP	Inverse variance weighted	6	-0.011	0.045	0.811	0.112	0.956	No	6	-0.011	0.045	0.811	0.112	0.956
HDGF	DBP	Inverse variance weighted	10	-0.036	0.027	0.186	0.070	0.559	No	10	-0.036	0.027	0.186	0.070	0.560
HDGFL2	DBP	Wald ratio	1	1.078	0.491	0.028	-	0.190	No	1	1.078	0.491	0.028	-	0.191
HEBP1	DBP	Wald ratio	1	-0.484	0.205	0.018	-	0.140	No	1	-0.484	0.205	0.018	-	0.141
HEG1	DBP	Inverse variance weighted	3	-0.024	0.232	0.917	0.173	0.984	No	3	-0.024	0.232	0.917	0.173	0.984
HEPACAM2	DBP	Wald ratio	1	-1.699	0.628	0.007	-	0.080	No	1	-1.699	0.628	0.007	-	0.081
HEXIM1	DBP	Wald ratio	1	1.945	0.336	0.000	-	0.000	Yes	1	1.945	0.336	0.000	-	0.000
HGF	DBP	Inverse variance weighted	2	0.085	0.234	0.716	-	0.931	No	2	0.085	0.234	0.716	-	0.932
HGFAC	DBP	Inverse variance weighted	6	-0.151	0.091	0.099	0.001	0.413	No	6	-0.151	0.091	0.099	0.001	0.414

ST3: MR causal estimates for plasma proteins on diastolic blood pressure.

Causal candidates prioritized for DBP were marked as "Yes" in column "Prioritized". Effect of plasma protein levels on blood pressure is in mmHg unit.

										Steiger filtering					
Exposure	Outocme	Method	nsnp	Beta	SE	P-value	Cochran's Q P-value	FDR-corrected P-value	Prioritized	nsnp	Beta	SE	P-value	Cochran's Q P-value	FDR-corrected P-value
HHEX	DBP	Wald ratio	1	3.075	0.640	0.000	-	0.000	Yes	1	3.075	0.640	0.000	-	0.000
HIP1	DBP	Wald ratio	1	0.199	0.546	0.715	-	0.931	No	1	0.199	0.546	0.715	-	0.932
HIP1R	DBP	Inverse variance weighted	2	-0.053	1.467	0.971	-	0.998	No	2	-0.053	1.467	0.971	-	0.998
HJV	DBP	Wald ratio	1	0.019	0.156	0.902	-	0.983	No	1	0.019	0.156	0.902	-	0.983
HMBS	DBP	Inverse variance weighted	3	0.261	0.132	0.048	0.643	0.261	No	3	0.261	0.132	0.048	0.643	0.262
HMCN2	DBP	Inverse variance weighted	10	-0.070	0.051	0.165	0.031	0.529	No	10	-0.070	0.051	0.165	0.031	0.530
HMOX1	DBP	Inverse variance weighted	3	0.039	0.671	0.954	0.001	0.992	No	3	0.039	0.671	0.954	0.001	0.992
HMOX2	DBP	Wald ratio	1	0.034	0.233	0.886	-	0.980	No	1	0.034	0.233	0.886	-	0.980
HNMT	DBP	Inverse variance weighted	4	-0.066	0.055	0.233	0.034	0.620	No	4	-0.066	0.055	0.233	0.034	0.621
HPCAL1	DBP	Inverse variance weighted	2	-0.203	0.443	0.647	-	0.902	No	2	-0.203	0.443	0.647	-	0.902
HPGDS	DBP	Inverse variance weighted	7	0.205	0.061	0.001	0.137	0.015	Yes	7	0.205	0.061	0.001	0.137	0.016
HPSE	DBP	Wald ratio	1	0.289	0.152	0.057	-	0.290	No	1	0.289	0.152	0.057	-	0.291
HRC	DBP	Inverse variance weighted	2	-0.066	0.218	0.763	-	0.938	No	2	-0.066	0.218	0.763	-	0.939
HRG	DBP	Inverse variance weighted	4	0.077	0.085	0.365	0.030	0.743	No	4	0.077	0.085	0.365	0.030	0.744
HS1BP3	DBP	Inverse variance weighted	3	-0.317	0.314	0.313	0.163	0.703	No	3	-0.317	0.314	0.313	0.163	0.704
HS3ST3B1	DBP	Inverse variance weighted	6	-0.168	0.093	0.072	0.025	0.338	No	6	-0.168	0.093	0.072	0.025	0.340
HS6ST1	DBP	Inverse variance weighted	3	-0.069	0.098	0.481	0.542	0.814	No	3	-0.069	0.098	0.481	0.542	0.815
HSBP1	DBP	Inverse variance weighted	6	0.013	0.037	0.725	0.721	0.931	No	6	0.013	0.037	0.725	0.721	0.932
HSD17B14	DBP	Inverse variance weighted	13	0.016	0.030	0.592	0.890	0.876	No	13	0.016	0.030	0.592	0.890	0.876
HSDL2	DBP	Inverse variance weighted	6	0.103	0.039	0.009	0.791	0.089	No	6	0.103	0.039	0.009	0.791	0.090
HSPA2	DBP	Inverse variance weighted	2	-0.101	0.329	0.760	-	0.938	No	2	-0.101	0.329	0.760	-	0.938
HSPB1	DBP	Inverse variance weighted	5	-0.019	0.049	0.701	0.463	0.931	No	5	-0.019	0.049	0.701	0.463	0.932
HSPB6	DBP	Wald ratio	1	0.543	0.698	0.437	-	0.793	No	1	0.543	0.698	0.437	-	0.794
HSPG2	DBP	Inverse variance weighted	3	-0.010	0.335	0.975	0.002	0.998	No	3	-0.010	0.335	0.975	0.002	0.998
HYAL1	DBP	Inverse variance weighted	2	0.235	0.084	0.005	-	0.066	No	2	0.235	0.084	0.005	-	0.067
HYOU1	DBP	Inverse variance weighted	2	0.064	0.132	0.629	-	0.892	No	2	0.064	0.132	0.629	-	0.892
ICAM1	DBP	Inverse variance weighted	6	-0.233	0.058	0.000	0.355	0.002	Yes	6	-0.233	0.058	0.000	0.355	0.002
ICAM2	DBP	Wald ratio	1	1.030	0.436	0.018	-	0.140	No	1	1.030	0.436	0.018	-	0.141
ICAM3	DBP	Inverse variance weighted	4	-0.099	0.092	0.282	0.209	0.675	No	4	-0.099	0.092	0.282	0.209	0.676
ICAM4	DBP	Wald ratio	1	0.907	0.216	0.000	-	0.001	Yes	1	0.907	0.216	0.000	-	0.001
ICAM5	DBP	Inverse variance weighted	7	-0.029	0.043	0.504	0.114	0.829	No	7	-0.029	0.043	0.504	0.114	0.830
ICOSLG	DBP	Inverse variance weighted	5	-0.021	0.064	0.742	0.173	0.938	No	5	-0.021	0.064	0.742	0.173	0.938
IDO1	DBP	Inverse variance weighted	4	-0.089	0.083	0.288	0.457	0.679	No	4	-0.089	0.083	0.288	0.457	0.680
IDUA	DBP	Inverse variance weighted	8	-0.104	0.041	0.011	0.912	0.101	No	8	-0.104	0.041	0.011	0.912	0.102
IFI30	DBP	Inverse variance weighted	2	-0.227	0.175	0.196	-	0.567	No	2	-0.227	0.175	0.196	-	0.568
IFIT3	DBP	Wald ratio	1	-0.772	0.227	0.001	-	0.013	Yes	1	-0.772	0.227	0.001	-	0.013
IF-R1	DBP	Inverse variance weighted	6	0.143	0.056	0.011	0.881	0.104	No	6	0.143	0.056	0.011	0.881	0.105
IFNGR1	DBP	Wald ratio	1	0.162	0.139	0.243	-	0.627	No	1	0.162	0.139	0.243	-	0.628
IFNGR2	DBP	Inverse variance weighted	12	0.112	0.049	0.023	0.000	0.165	No	12	0.112	0.049	0.023	0.000	0.166
IFNL1	DBP	Wald ratio	1	0.237	0.424	0.577	-	0.869	No	1	0.237	0.424	0.577	-	0.870
IFNLR1	DBP	Inverse variance weighted	4	-0.033	0.066	0.615	0.709	0.890	No	4	-0.033	0.066	0.615	0.709	0.891
IGDCC4	DBP	Inverse variance weighted	11	-0.045	0.089	0.615	0.104	0.890	No	11	-0.045	0.089	0.615	0.104	0.891
IGF1R	DBP	Inverse variance weighted	3	-0.431	0.366	0.238	0.015	0.624	No	3	-0.431	0.366	0.238	0.015	0.625
IGF2R	DBP	Inverse variance weighted	9	-0.035	0.074	0.639	0.050	0.900	No	9	-0.035	0.074	0.639	0.050	0.900
IGFBP1	DBP	Wald ratio	1	0.224	0.463	0.628	-	0.892	No	1	0.224	0.463	0.628	-	0.892
IGFBP2	DBP	Inverse variance weighted	2	0.250	0.352	0.477	-	0.813	No	2	0.250	0.352	0.477	-	0.813
IGFBP3	DBP	Inverse variance weighted	4	0.320	0.080	0.000	0.054	0.002	Yes	4	0.320	0.080	0.000	0.054	0.002
IGFBP4	DBP	Wald ratio	1	0.156	0.498	0.753	-	0.938	No	1	0.156	0.498	0.753	-	0.938
IGFBP6	DBP	Wald ratio	1	0.548	0.293	0.061	-	0.307	No	1	0.548	0.293	0.061	-	0.308
IGFBP7	DBP	Inverse variance weighted	3	-0.263	0.138	0.056	0.018	0.289	No	3	-0.263	0.138	0.056	0.018	0.290
IGFBPL1	DBP	Inverse variance weighted	8	-0.019	0.052	0.719	0.277	0.931	No	8	-0.019	0.052	0.719	0.277	0.932
IGLC2	DBP	Inverse variance weighted	4	0.439	0.229	0.055	0.059	0.287	No	4	0.439	0.229	0.055	0.059	0.288
IGSF21	DBP	Inverse variance weighted	4	-0.008	0.162	0.960	0.184	0.993	No	4	-0.008	0.162	0.960	0.184	0.993
IGSF3	DBP	Inverse variance weighted	2	0.499	0.296	0.092	-	0.399	No	2	0.499	0.296	0.092	-	0.400
IGSF8	DBP	Wald ratio	1	-0.165	0.184	0.369	-	0.746	No	1	-0.165	0.184	0.369	-	0.747
IGSF9	DBP	Inverse variance weighted	5	0.064	0.130	0.624	0.049	0.891	No	5	0.064	0.130	0.624	0.049	0.892

ST3: MR causal estimates for plasma proteins on diastolic blood pressure.

Causal candidates prioritized for DBP were marked as "Yes" in column "Prioritized". Effect of plasma protein levels on blood pressure is in mmHg unit.

										Steiger filtering					
Exposure	Outocme	Method	nsnp	Beta	SE	P-value	Cochran's Q P-value	FDR-corrected P-value	Prioritized	nsnp	Beta	SE	P-value	Cochran's Q P-value	FDR-corrected P-value
IL10	DBP	Wald ratio	1	-0.263	0.231	0.255	-	0.642	No	1	-0.263	0.231	0.255	-	0.643
IL10RA	DBP	Wald ratio	1	0.005	0.168	0.976	-	0.998	No	1	0.005	0.168	0.976	-	0.998
IL10RB	DBP	Inverse variance weighted	4	0.037	0.117	0.752	0.000	0.938	No	4	0.037	0.117	0.752	0.000	0.938
IL12RB1	DBP	Inverse variance weighted	2	-0.067	0.181	0.710	-	0.931	No	2	-0.067	0.181	0.710	-	0.932
IL15RA	DBP	Inverse variance weighted	3	-0.014	0.093	0.877	0.689	0.980	No	3	-0.014	0.093	0.877	0.689	0.980
IL16	DBP	Inverse variance weighted	2	0.019	0.145	0.897	-	0.982	No	2	0.019	0.145	0.897	-	0.982
IL17C	DBP	Inverse variance weighted	2	-0.508	0.187	0.006	-	0.078	No	2	-0.508	0.187	0.006	-	0.078
IL17D	DBP	Inverse variance weighted	4	0.010	0.066	0.884	0.593	0.980	No	4	0.010	0.066	0.884	0.593	0.980
IL17F	DBP	Inverse variance weighted	3	0.206	0.212	0.331	0.073	0.719	No	3	0.206	0.212	0.331	0.073	0.720
IL17RA	DBP	Inverse variance weighted	8	0.002	0.026	0.944	0.497	0.989	No	8	0.002	0.026	0.944	0.497	0.989
IL17RB	DBP	Inverse variance weighted	8	-0.061	0.054	0.258	0.001	0.646	No	8	-0.061	0.054	0.258	0.001	0.647
IL18	DBP	Inverse variance weighted	2	-0.098	0.079	0.212	-	0.589	No	2	-0.098	0.079	0.212	-	0.590
IL18BP	DBP	Wald ratio	1	0.170	0.549	0.757	-	0.938	No	1	0.170	0.549	0.757	-	0.938
IL18R1	DBP	Inverse variance weighted	11	-0.004	0.030	0.897	0.495	0.982	No	11	-0.004	0.030	0.897	0.495	0.982
IL19	DBP	Inverse variance weighted	2	-0.016	0.081	0.845	-	0.969	No	2	-0.016	0.081	0.845	-	0.969
IL1B	DBP	Wald ratio	1	-0.718	0.664	0.280	-	0.673	No	1	-0.718	0.664	0.280	-	0.674
IL1R1	DBP	Inverse variance weighted	3	0.020	0.124	0.871	0.279	0.977	No	3	0.020	0.124	0.871	0.279	0.977
IL1R2	DBP	Inverse variance weighted	4	0.047	0.056	0.399	0.177	0.769	No	4	0.047	0.056	0.399	0.177	0.769
IL1RAP	DBP	Inverse variance weighted	8	0.003	0.032	0.914	0.042	0.983	No	8	0.003	0.032	0.914	0.042	0.983
IL1RL1	DBP	Inverse variance weighted	8	0.121	0.033	0.000	0.776	0.005	Yes	8	0.121	0.033	0.000	0.776	0.005
IL1RL2	DBP	Inverse variance weighted	5	-0.050	0.052	0.334	0.631	0.722	No	5	-0.050	0.052	0.334	0.631	0.723
IL1RN	DBP	Inverse variance weighted	3	-0.067	0.088	0.449	0.921	0.799	No	3	-0.067	0.088	0.449	0.921	0.800
IL20	DBP	Wald ratio	1	-0.347	0.558	0.535	-	0.845	No	1	-0.347	0.558	0.535	-	0.845
IL20RB	DBP	Wald ratio	1	0.158	0.205	0.440	-	0.794	No	1	0.158	0.205	0.440	-	0.795
IL22	DBP	Inverse variance weighted	2	-0.004	0.242	0.988	-	1.000	No	2	-0.004	0.242	0.988	-	1.000
IL22RA1	DBP	Inverse variance weighted	2	0.106	0.090	0.242	-	0.627	No	2	0.106	0.090	0.242	-	0.628
IL2RA	DBP	Inverse variance weighted	8	0.023	0.040	0.571	0.452	0.865	No	8	0.023	0.040	0.571	0.452	0.866
IL31RA	DBP	Inverse variance weighted	7	0.021	0.057	0.715	0.033	0.931	No	7	0.021	0.057	0.715	0.033	0.932
IL32	DBP	Inverse variance weighted	4	0.173	0.086	0.046	0.403	0.255	No	4	0.173	0.086	0.046	0.403	0.256
IL34	DBP	Inverse variance weighted	6	-0.018	0.065	0.777	0.001	0.939	No	6	-0.018	0.065	0.777	0.001	0.939
IL36A	DBP	Wald ratio	1	0.488	0.474	0.303	-	0.693	No	1	0.488	0.474	0.303	-	0.693
IL36G	DBP	Inverse variance weighted	2	0.343	0.826	0.678	-	0.918	No	2	0.343	0.826	0.678	-	0.918
IL4R	DBP	Inverse variance weighted	2	0.053	0.099	0.590	-	0.875	No	2	0.053	0.099	0.590	-	0.876
IL5RA	DBP	Inverse variance weighted	7	-0.071	0.054	0.188	0.836	0.561	No	7	-0.071	0.054	0.188	0.836	0.562
IL6R	DBP	Inverse variance weighted	9	-0.012	0.035	0.725	0.005	0.931	No	9	-0.012	0.035	0.725	0.005	0.932
IL6ST	DBP	Inverse variance weighted	2	-0.174	0.131	0.183	-	0.555	No	2	-0.174	0.131	0.183	-	0.556
IL7	DBP	Inverse variance weighted	2	0.077	0.325	0.813	-	0.956	No	2	0.077	0.325	0.813	-	0.957
IL7R	DBP	Inverse variance weighted	6	-0.034	0.029	0.238	0.398	0.624	No	6	-0.034	0.029	0.238	0.398	0.625
IMMT	DBP	Wald ratio	1	-0.739	0.200	0.000	-	0.005	Yes	1	-0.739	0.200	0.000	-	0.005
IMPA1	DBP	Wald ratio	1	0.307	0.120	0.010	-	0.100	No	1	0.307	0.120	0.010	-	0.101
ING1	DBP	Wald ratio	1	-1.426	0.465	0.002	-	0.032	Yes	1	-1.426	0.465	0.002	-	0.033
INHBB	DBP	Inverse variance weighted	2	0.135	0.092	0.139	-	0.492	No	2	0.135	0.092	0.139	-	0.493
INHBC	DBP	Inverse variance weighted	4	0.055	0.058	0.339	0.108	0.724	No	4	0.055	0.058	0.339	0.108	0.724
INPP1	DBP	Wald ratio	1	-0.833	0.313	0.008	-	0.084	No	1	-0.833	0.313	0.008	-	0.085
INPP5D	DBP	Inverse variance weighted	2	0.148	0.135	0.275	-	0.665	No	2	0.148	0.135	0.275	-	0.666
INSL3	DBP	Wald ratio	1	1.434	0.811	0.077	-	0.352	No	1	1.434	0.811	0.077	-	0.353
INSL4	DBP	Wald ratio	1	0.109	0.138	0.429	-	0.790	No	1	0.109	0.138	0.429	-	0.791
INSL5	DBP	Inverse variance weighted	2	-0.107	0.148	0.471	-	0.811	No	2	-0.107	0.148	0.471	-	0.812
INSR	DBP	Wald ratio	1	-1.193	0.447	0.008	-	0.084	No	1	-1.193	0.447	0.008	-	0.084
IPCEF1	DBP	Inverse variance weighted	2	-0.135	0.508	0.791	-	0.945	No	2	-0.135	0.508	0.791	-	0.945
IQGAP2	DBP	Wald ratio	1	-0.191	0.171	0.265	-	0.652	No	1	-0.191	0.171	0.265	-	0.653
IRAK4	DBP	Wald ratio	1	-0.027	0.367	0.941	-	0.989	No	1	-0.027	0.367	0.941	-	0.989
ISLR2	DBP	Inverse variance weighted	2	-0.217	0.122	0.075	-	0.348	No	2	-0.217	0.122	0.075	-	0.350
ISM1	DBP	Inverse variance weighted	5	0.111	0.088	0.210	0.742	0.588	No	5	0.111	0.088	0.210	0.742	0.589
IST1	DBP	Wald ratio	1	0.202	0.293	0.490	-	0.823	No	1	0.202	0.293	0.490	-	0.823
ITGA11	DBP	Inverse variance weighted	5	-0.037	0.087	0.672	0.550	0.914	No	5	-0.037	0.087	0.672	0.550	0.914

ST3: MR causal estimates for plasma proteins on diastolic blood pressure.

Causal candidates prioritized for DBP were marked as "Yes" in column "Prioritized". Effect of plasma protein levels on blood pressure is in mmHg unit.

										Steiger filtering					
Exposure	Outocme	Method	nsnp	Beta	SE	P-value	Cochran's Q P-value	FDR-corrected P-value	Prioritized	nsnp	Beta	SE	P-value	Cochran's Q P-value	FDR-corrected P-value
ITGA2	DBP	Inverse variance weighted	5	0.067	0.054	0.217	0.094	0.595	No	5	0.067	0.054	0.217	0.094	0.597
ITGA5	DBP	Wald ratio	1	0.131	0.460	0.775	-	0.938	No	1	0.131	0.460	0.775	-	0.939
ITGA6	DBP	Inverse variance weighted	4	-0.004	0.150	0.976	0.085	0.998	No	4	-0.004	0.150	0.976	0.085	0.998
ITGAL	DBP	Wald ratio	1	2.585	0.667	0.000	-	0.003	Yes	1	2.585	0.667	0.000	-	0.003
ITGAM	DBP	Wald ratio	1	-0.441	0.156	0.005	-	0.061	No	1	-0.441	0.156	0.005	-	0.062
ITGAV	DBP	Inverse variance weighted	3	0.027	0.252	0.915	0.038	0.983	No	3	0.027	0.252	0.915	0.038	0.983
ITGAX	DBP	Wald ratio	1	0.522	0.555	0.347	-	0.729	No	1	0.522	0.555	0.347	-	0.730
ITGB2	DBP	Wald ratio	1	-0.122	0.204	0.550	-	0.854	No	1	-0.122	0.204	0.550	-	0.855
ITGB5	DBP	Inverse variance weighted	2	-0.779	1.439	0.589	-	0.875	No	2	-0.779	1.439	0.589	-	0.876
ITGB6	DBP	Inverse variance weighted	3	0.018	0.082	0.827	0.918	0.963	No	3	0.018	0.082	0.827	0.918	0.963
ITGB7	DBP	Inverse variance weighted	2	0.144	0.317	0.648	-	0.902	No	2	0.144	0.317	0.648	-	0.902
ITGBL1	DBP	Inverse variance weighted	5	0.017	0.065	0.794	0.424	0.947	No	5	0.017	0.065	0.794	0.424	0.947
ITIH1	DBP	Wald ratio	1	-0.129	0.176	0.462	-	0.807	No	1	-0.129	0.176	0.462	-	0.807
ITIH3	DBP	Inverse variance weighted	5	0.040	0.107	0.708	0.092	0.931	No	5	0.040	0.107	0.708	0.092	0.932
ITIH4	DBP	Inverse variance weighted	4	0.090	0.240	0.708	0.000	0.931	No	4	0.090	0.240	0.708	0.000	0.932
ITIH5	DBP	Wald ratio	1	0.203	0.230	0.378	-	0.751	No	1	0.203	0.230	0.378	-	0.752
ITPA	DBP	Inverse variance weighted	7	-0.030	0.033	0.359	0.917	0.737	No	7	-0.030	0.033	0.359	0.917	0.738
ITPR1	DBP	Wald ratio	1	-0.027	0.245	0.911	-	0.983	No	1	-0.027	0.245	0.911	-	0.983
JAM2	DBP	Inverse variance weighted	2	-0.070	0.301	0.816	-	0.957	No	2	-0.070	0.301	0.816	-	0.957
JAM3	DBP	Inverse variance weighted	2	0.180	0.222	0.417	-	0.778	No	2	0.180	0.222	0.417	-	0.779
JPT2	DBP	Wald ratio	1	-0.398	0.512	0.437	-	0.793	No	1	-0.398	0.512	0.437	-	0.794
KAZALD1	DBP	Inverse variance weighted	5	-0.070	0.044	0.116	0.153	0.457	No	5	-0.070	0.044	0.116	0.153	0.458
KCTD5	DBP	Wald ratio	1	-1.069	0.533	0.045	-	0.255	No	1	-1.069	0.533	0.045	-	0.256
KDR	DBP	Inverse variance weighted	7	0.021	0.055	0.706	0.125	0.931	No	7	0.021	0.055	0.706	0.125	0.932
KEL	DBP	Inverse variance weighted	6	0.152	0.097	0.120	0.024	0.467	No	6	0.152	0.097	0.120	0.024	0.468
KHK	DBP	Inverse variance weighted	3	-0.007	0.054	0.894	0.615	0.982	No	3	-0.007	0.054	0.894	0.615	0.982
KIAA0319	DBP	Inverse variance weighted	5	-0.148	0.101	0.143	0.115	0.492	No	5	-0.148	0.101	0.143	0.115	0.493
KIF1C	DBP	Wald ratio	1	0.058	0.531	0.913	-	0.983	No	1	0.058	0.531	0.913	-	0.983
KIF22	DBP	Wald ratio	1	-4.214	0.675	0.000	-	0.000	Yes	1	-4.214	0.675	0.000	-	0.000
KIFBP	DBP	Wald ratio	1	1.756	0.539	0.001	-	0.019	Yes	1	1.756	0.539	0.001	-	0.020
KIR2DL2	DBP	Inverse variance weighted	5	-0.049	0.040	0.215	0.909	0.593	No	5	-0.049	0.040	0.215	0.909	0.594
KIR2DL3	DBP	Inverse variance weighted	4	-0.087	0.074	0.237	0.185	0.624	No	4	-0.087	0.074	0.237	0.185	0.625
KIR2DS4	DBP	Inverse variance weighted	8	-0.038	0.021	0.069	0.554	0.332	No	8	-0.038	0.021	0.069	0.554	0.333
KIR3DL1	DBP	Inverse variance weighted	8	0.032	0.024	0.173	0.552	0.542	No	8	0.032	0.024	0.173	0.552	0.543
KIR3DL2	DBP	Wald ratio	1	-0.015	0.185	0.936	-	0.986	No	1	-0.015	0.185	0.936	-	0.986
KIRREL2	DBP	Inverse variance weighted	6	0.160	0.114	0.160	0.894	0.520	No	6	0.160	0.114	0.160	0.894	0.522
KIT	DBP	Inverse variance weighted	2	-0.010	1.613	0.995	-	1.000	No	2	-0.010	1.613	0.995	-	1.000
KITLG	DBP	Wald ratio	1	-0.624	0.419	0.137	-	0.490	No	1	-0.624	0.419	0.137	-	0.491
KLB	DBP	Inverse variance weighted	5	0.074	0.034	0.030	0.308	0.198	No	5	0.074	0.034	0.030	0.308	0.199
KLHL41	DBP	Inverse variance weighted	2	0.647	0.431	0.133	-	0.484	No	2	0.647	0.431	0.133	-	0.485
KLK1	DBP	Inverse variance weighted	4	-0.156	0.132	0.239	0.004	0.624	No	4	-0.156	0.132	0.239	0.004	0.625
KLK10	DBP	Inverse variance weighted	6	-0.004	0.039	0.913	0.598	0.983	No	6	-0.004	0.039	0.913	0.598	0.983
KLK11	DBP	Inverse variance weighted	3	-0.004	0.070	0.954	0.202	0.992	No	3	-0.004	0.070	0.954	0.202	0.992
KLK12	DBP	Inverse variance weighted	10	-0.006	0.020	0.775	0.650	0.938	No	10	-0.006	0.020	0.775	0.650	0.939
KLK13	DBP	Inverse variance weighted	5	0.025	0.051	0.624	0.403	0.891	No	5	0.025	0.051	0.624	0.403	0.892
KLK14	DBP	Inverse variance weighted	5	0.000	0.052	0.997	0.596	1.000	No	5	0.000	0.052	0.997	0.596	1.000
KLK15	DBP	Inverse variance weighted	9	0.004	0.026	0.868	0.676	0.977	No	9	0.004	0.026	0.868	0.676	0.977
KLK3	DBP	Inverse variance weighted	2	0.787	0.406	0.053	-	0.279	No	2	0.787	0.406	0.053	-	0.280
KLK4	DBP	Inverse variance weighted	6	0.074	0.058	0.207	0.344	0.585	No	6	0.074	0.058	0.207	0.344	0.586
KLK6	DBP	Wald ratio	1	0.096	0.128	0.454	-	0.800	No	1	0.096	0.128	0.454	-	0.801
KLK7	DBP	Inverse variance weighted	4	-0.016	0.053	0.756	0.503	0.938	No	4	-0.016	0.053	0.756	0.503	0.938
KLK8	DBP	Inverse variance weighted	7	0.054	0.056	0.339	0.151	0.724	No	7	0.054	0.056	0.339	0.151	0.724
KLKB1	DBP	Inverse variance weighted	2	-0.045	0.066	0.497	-	0.826	No	2	-0.045	0.066	0.497	-	0.827
KLRB1	DBP	Inverse variance weighted	6	0.130	0.193	0.502	0.086	0.829	No	6	0.130	0.193	0.502	0.086	0.830
KLRD1	DBP	Inverse variance weighted	4	0.058	0.054	0.286	0.425	0.677	No	4	0.058	0.054	0.286	0.425	0.678
KLRF1	DBP	Inverse variance weighted	4	0.061	0.133	0.645	0.156	0.901	No	4	0.061	0.133	0.645	0.156	0.902

ST3: MR causal estimates for plasma proteins on diastolic blood pressure.

Causal candidates prioritized for DBP were marked as "Yes" in column "Prioritized". Effect of plasma protein levels on blood pressure is in mmHg unit.

										Steiger filtering					
**Exposure**	**Outocme**	**Method**	**nsnp**	**Beta**	**SE**	**P-value**	**Cochran's Q P-value**	**FDR-corrected P-value**	**Prioritized**	**nsnp**	**Beta**	**SE**	**P-value**	**Cochran's Q P-value**	**FDR-corrected P-value**
KLRK1	DBP	Inverse variance weighted	4	-0.092	0.048	0.054	0.722	0.282	No	4	-0.092	0.048	0.054	0.722	0.283
KRT18	DBP	Wald ratio	1	0.313	0.555	0.572	-	0.866	No	1	0.313	0.555	0.572	-	0.866
KRT19	DBP	Wald ratio	1	0.306	0.159	0.055	-	0.287	No	1	0.306	0.159	0.055	-	0.288
KRT5	DBP	Inverse variance weighted	2	-0.043	0.238	0.856	-	0.974	No	2	-0.043	0.238	0.856	-	0.974
KYAT1	DBP	Wald ratio	1	0.031	0.179	0.864	-	0.976	No	1	0.031	0.179	0.864	-	0.976
KYNU	DBP	Inverse variance weighted	5	-0.056	0.083	0.504	0.413	0.829	No	5	-0.056	0.083	0.504	0.413	0.830
LACRT	DBP	Wald ratio	1	-1.022	0.340	0.003	-	0.039	Yes	1	-1.022	0.340	0.003	-	0.039
LACTB2	DBP	Inverse variance weighted	2	0.103	0.150	0.493	-	0.824	No	2	0.103	0.150	0.493	-	0.824
LAG3	DBP	Inverse variance weighted	2	-0.055	0.133	0.679	-	0.918	No	2	-0.055	0.133	0.679	-	0.918
LAIR1	DBP	Inverse variance weighted	7	-0.020	0.048	0.680	0.226	0.918	No	7	-0.020	0.048	0.680	0.226	0.918
LAIR2	DBP	Inverse variance weighted	13	0.030	0.030	0.329	0.598	0.717	No	13	0.030	0.030	0.329	0.598	0.718
LAMA4	DBP	Wald ratio	1	0.046	0.146	0.754	-	0.938	No	1	0.046	0.146	0.754	-	0.938
LAMB1	DBP	Inverse variance weighted	7	0.027	0.058	0.636	0.010	0.897	No	7	0.027	0.058	0.636	0.010	0.898
LAMP1	DBP	Wald ratio	1	0.825	0.422	0.051	-	0.270	No	1	0.825	0.422	0.051	-	0.271
LAMP3	DBP	Wald ratio	1	-0.040	0.107	0.710	-	0.931	No	1	-0.040	0.107	0.710	-	0.932
LAP3	DBP	Wald ratio	1	-0.757	0.428	0.077	-	0.352	No	1	-0.757	0.428	0.077	-	0.353
LAT	DBP	Wald ratio	1	1.241	0.545	0.023	-	0.165	No	1	1.241	0.545	0.023	-	0.166
LAT2	DBP	Wald ratio	1	-0.005	0.648	0.994	-	1.000	No	1	-0.005	0.648	0.994	-	1.000
LATS1	DBP	Wald ratio	1	-0.412	0.400	0.303	-	0.693	No	1	-0.412	0.400	0.303	-	0.694
LAYN	DBP	Inverse variance weighted	3	-0.312	0.064	0.000	0.802	0.000	Yes	3	-0.312	0.064	0.000	0.802	0.000
LBP	DBP	Inverse variance weighted	7	-0.001	0.037	0.970	0.314	0.997	No	7	-0.001	0.037	0.970	0.314	0.997
LBR	DBP	Wald ratio	1	-0.205	0.617	0.739	-	0.938	No	1	-0.205	0.617	0.739	-	0.938
LCAT	DBP	Inverse variance weighted	2	0.397	0.185	0.032	-	0.208	No	2	0.397	0.185	0.032	-	0.209
LCN15	DBP	Wald ratio	1	-0.012	0.042	0.782	-	0.939	No	1	-0.012	0.042	0.782	-	0.940
LCN2	DBP	Wald ratio	1	-0.460	0.363	0.206	-	0.583	No	1	-0.460	0.363	0.206	-	0.584
LCP1	DBP	Inverse variance weighted	5	-0.004	0.035	0.911	0.158	0.983	No	5	-0.004	0.035	0.911	0.158	0.983
LDLR	DBP	Wald ratio	1	-0.907	0.586	0.122	-	0.473	No	1	-0.907	0.586	0.122	-	0.475
LDLRAP1	DBP	Wald ratio	1	0.241	0.337	0.474	-	0.811	No	1	0.241	0.337	0.474	-	0.812
LECT2	DBP	Inverse variance weighted	7	-0.011	0.039	0.773	0.092	0.938	No	7	-0.011	0.039	0.773	0.092	0.939
LEFTY2	DBP	Inverse variance weighted	8	0.000	0.063	0.994	0.000	1.000	No	8	0.000	0.063	0.994	0.000	1.000
LEG1	DBP	Wald ratio	1	0.066	0.063	0.290	-	0.680	No	1	0.066	0.063	0.290	-	0.681
LEP	DBP	Wald ratio	1	1.259	0.622	0.043	-	0.252	No	1	1.259	0.622	0.043	-	0.253
LEPR	DBP	Inverse variance weighted	2	0.123	0.386	0.749	-	0.938	No	2	0.123	0.386	0.749	-	0.938
LGALS1	DBP	Inverse variance weighted	2	-0.078	0.085	0.360	-	0.737	No	2	-0.078	0.085	0.360	-	0.738
LGALS3	DBP	Inverse variance weighted	4	0.072	0.043	0.095	0.511	0.402	No	4	0.072	0.043	0.095	0.511	0.403
LGALS3BP	DBP	Inverse variance weighted	6	-0.170	0.128	0.186	0.593	0.559	No	6	-0.170	0.128	0.186	0.593	0.560
LGALS4	DBP	Inverse variance weighted	3	0.067	0.399	0.866	0.008	0.976	No	3	0.067	0.399	0.866	0.008	0.976
LGALS8	DBP	Wald ratio	1	-0.021	0.086	0.807	-	0.954	No	1	-0.021	0.086	0.807	-	0.954
LGALS9	DBP	Inverse variance weighted	2	-0.020	0.120	0.870	-	0.977	No	2	-0.020	0.120	0.870	-	0.977
LGMN	DBP	Inverse variance weighted	11	0.115	0.094	0.223	0.101	0.603	No	11	0.115	0.094	0.223	0.101	0.604
LHB	DBP	Wald ratio	1	-0.292	0.473	0.537	-	0.846	No	1	-0.292	0.473	0.537	-	0.847
LHPP	DBP	Inverse variance weighted	8	-0.006	0.058	0.911	0.523	0.983	No	8	-0.006	0.058	0.911	0.523	0.983
LIF	DBP	Wald ratio	1	-0.115	0.521	0.826	-	0.963	No	1	-0.115	0.521	0.826	-	0.963
LIFR	DBP	Inverse variance weighted	3	-0.109	0.128	0.393	0.951	0.767	No	3	-0.109	0.128	0.393	0.951	0.768
LILRA2	DBP	Inverse variance weighted	7	0.048	0.044	0.284	0.198	0.676	No	7	0.048	0.044	0.284	0.198	0.677
LILRA3	DBP	Inverse variance weighted	8	-0.031	0.022	0.168	0.476	0.533	No	8	-0.031	0.022	0.168	0.476	0.534
LILRA4	DBP	Wald ratio	1	-0.236	0.652	0.717	-	0.931	No	1	-0.236	0.652	0.717	-	0.932
LILRA5	DBP	Inverse variance weighted	4	-0.184	0.063	0.004	0.461	0.050	No	4	-0.184	0.063	0.004	0.461	0.051
LILRA6	DBP	Inverse variance weighted	13	0.032	0.042	0.454	0.006	0.800	No	13	0.032	0.042	0.454	0.006	0.801
LILRB1	DBP	Inverse variance weighted	8	-0.076	0.040	0.060	0.238	0.301	No	8	-0.076	0.040	0.060	0.238	0.302
LILRB2	DBP	Inverse variance weighted	8	-0.006	0.031	0.854	0.111	0.973	No	8	-0.006	0.031	0.854	0.111	0.973
LILRB4	DBP	Inverse variance weighted	8	0.004	0.086	0.964	0.376	0.993	No	8	0.004	0.086	0.964	0.376	0.993
LILRB5	DBP	Inverse variance weighted	11	0.004	0.019	0.850	0.872	0.972	No	11	0.004	0.019	0.850	0.872	0.973
LIPF	DBP	Inverse variance weighted	2	-0.011	0.084	0.892	-	0.982	No	2	-0.011	0.084	0.892	-	0.982
LMNB1	DBP	Wald ratio	1	0.414	0.624	0.507	-	0.830	No	1	0.414	0.624	0.507	-	0.831
LMNB2	DBP	Wald ratio	1	-1.441	0.556	0.010	-	0.095	No	1	-1.441	0.556	0.010	-	0.096

ST3: MR causal estimates for plasma proteins on diastolic blood pressure.

Causal candidates prioritized for DBP were marked as "Yes" in column "Prioritized". Effect of plasma protein levels on blood pressure is in mmHg unit.

										Steiger filtering					
Exposure	Outocme	Method	nsnp	Beta	SE	P-value	Cochran's Q P-value	FDR-corrected P-value	Prioritized	nsnp	Beta	SE	P-value	Cochran's Q P-value	FDR-corrected P-value
LMOD1	DBP	Wald ratio	1	-1.043	0.137	0.000	-	0.000	Yes	1	-1.043	0.137	0.000	-	0.000
LPA	DBP	Inverse variance weighted	19	0.082	0.036	0.021	0.559	0.158	No	19	0.082	0.036	0.021	0.559	0.159
LPO	DBP	Inverse variance weighted	2	0.141	0.095	0.138	-	0.491	No	2	0.141	0.095	0.138	-	0.492
LRCH4	DBP	Wald ratio	1	-0.202	0.374	0.589	-	0.875	No	1	-0.202	0.374	0.589	-	0.876
LRIG1	DBP	Inverse variance weighted	10	-0.032	0.037	0.395	0.092	0.767	No	10	-0.032	0.037	0.395	0.092	0.768
LRIG3	DBP	Wald ratio	1	-0.211	0.297	0.478	-	0.813	No	1	-0.211	0.297	0.478	-	0.813
LRP11	DBP	Inverse variance weighted	7	0.017	0.043	0.697	0.293	0.931	No	7	0.017	0.043	0.697	0.293	0.931
LRRC37A2	DBP	Inverse variance weighted	6	-0.092	0.041	0.023	0.016	0.165	No	6	-0.092	0.041	0.023	0.016	0.167
LRRFIP1	DBP	Wald ratio	1	-0.881	0.366	0.016	-	0.132	No	1	-0.881	0.366	0.016	-	0.133
LRRN1	DBP	Inverse variance weighted	6	0.003	0.032	0.929	0.321	0.986	No	6	0.003	0.032	0.929	0.321	0.986
LRTM2	DBP	Inverse variance weighted	2	0.055	0.150	0.712	-	0.931	No	2	0.055	0.150	0.712	-	0.932
LSP1	DBP	Inverse variance weighted	3	0.978	0.588	0.096	0.000	0.404	No	3	0.978	0.588	0.096	0.000	0.405
LTA4H	DBP	Inverse variance weighted	2	-0.366	0.240	0.127	-	0.477	No	2	-0.366	0.240	0.127	-	0.479
LTBP2	DBP	Inverse variance weighted	2	-0.494	0.397	0.213	-	0.590	No	2	-0.494	0.397	0.213	-	0.591
LTBP3	DBP	Inverse variance weighted	6	0.069	0.114	0.544	0.000	0.850	No	6	0.069	0.114	0.544	0.000	0.851
LTBR	DBP	Inverse variance weighted	4	-0.063	0.079	0.426	0.885	0.788	No	4	-0.063	0.079	0.426	0.885	0.788
LUZP2	DBP	Inverse variance weighted	6	-0.070	0.080	0.384	0.482	0.754	No	6	-0.070	0.080	0.384	0.482	0.755
LXN	DBP	Inverse variance weighted	2	-0.578	0.445	0.194	-	0.567	No	2	-0.578	0.445	0.194	-	0.568
LY6D	DBP	Inverse variance weighted	5	0.017	0.104	0.871	0.003	0.977	No	5	0.017	0.104	0.871	0.003	0.977
LY75	DBP	Inverse variance weighted	7	0.003	0.027	0.897	0.251	0.982	No	7	0.003	0.027	0.897	0.251	0.982
LY9	DBP	Inverse variance weighted	5	0.005	0.055	0.930	0.198	0.986	No	5	0.005	0.055	0.930	0.198	0.986
LY96	DBP	Inverse variance weighted	2	0.322	0.283	0.255	-	0.642	No	2	0.322	0.283	0.255	-	0.643
LYAR	DBP	Wald ratio	1	0.951	0.473	0.045	-	0.255	No	1	0.951	0.473	0.045	-	0.256
LYN	DBP	Wald ratio	1	-1.254	0.659	0.057	-	0.293	No	1	-1.254	0.659	0.057	-	0.294
LYPD3	DBP	Inverse variance weighted	4	-0.118	0.068	0.082	0.987	0.369	No	4	-0.118	0.068	0.082	0.987	0.370
LYPD8	DBP	Inverse variance weighted	8	0.020	0.052	0.704	0.152	0.931	No	8	0.020	0.052	0.704	0.152	0.932
LYSMD3	DBP	Wald ratio	1	0.280	0.451	0.534	-	0.845	No	1	0.280	0.451	0.534	-	0.845
LYVE1	DBP	Inverse variance weighted	5	0.089	0.316	0.777	0.000	0.939	No	5	0.089	0.316	0.777	0.000	0.939
LYZL2	DBP	Inverse variance weighted	2	0.027	0.116	0.816	-	0.957	No	2	0.027	0.116	0.816	-	0.957
LZTFL1	DBP	Wald ratio	1	-0.256	0.162	0.112	-	0.447	No	1	-0.256	0.162	0.112	-	0.448
M6PR	DBP	Wald ratio	1	-0.577	0.131	0.000	-	0.000	Yes	1	-0.577	0.131	0.000	-	0.000
MAD1L1	DBP	Inverse variance weighted	2	-0.028	0.356	0.937	-	0.986	No	2	-0.028	0.356	0.937	-	0.986
MAMDC2	DBP	Inverse variance weighted	3	0.172	0.117	0.142	0.413	0.492	No	3	0.172	0.117	0.142	0.413	0.493
MAMDC4	DBP	Inverse variance weighted	3	0.109	0.133	0.412	0.933	0.777	No	3	0.109	0.133	0.412	0.933	0.778
MAN1A2	DBP	Inverse variance weighted	4	0.167	0.145	0.251	0.155	0.637	No	4	0.167	0.145	0.251	0.155	0.638
MAN2B2	DBP	Inverse variance weighted	6	0.037	0.067	0.581	0.672	0.870	No	6	0.037	0.067	0.581	0.672	0.870
MANEAL	DBP	Wald ratio	1	-0.973	0.638	0.127	-	0.477	No	1	-0.973	0.638	0.127	-	0.479
MANF	DBP	Wald ratio	1	-0.912	0.341	0.008	-	0.083	No	1	-0.912	0.341	0.008	-	0.084
MANSC1	DBP	Wald ratio	1	-0.313	0.585	0.593	-	0.876	No	1	-0.313	0.585	0.593	-	0.876
MANSC4	DBP	Inverse variance weighted	5	0.033	0.029	0.250	0.719	0.637	No	5	0.033	0.029	0.250	0.719	0.638
MAP2	DBP	Inverse variance weighted	2	0.225	0.212	0.289	-	0.679	No	2	0.225	0.212	0.289	-	0.680
MAP2K1	DBP	Wald ratio	1	-0.574	0.451	0.203	-	0.580	No	1	-0.574	0.451	0.203	-	0.581
MAP4K5	DBP	Wald ratio	1	0.704	0.129	0.000	-	0.000	Yes	1	0.704	0.129	0.000	-	0.000
MAPK13	DBP	Wald ratio	1	0.719	0.431	0.095	-	0.402	No	1	0.719	0.431	0.095	-	0.403
MAPK9	DBP	Inverse variance weighted	2	-0.438	0.305	0.151	-	0.498	No	2	-0.438	0.305	0.151	-	0.499
MAPKAPK2	DBP	Inverse variance weighted	3	-0.119	0.148	0.419	0.607	0.778	No	3	-0.119	0.148	0.419	0.607	0.779
MARCO	DBP	Inverse variance weighted	3	0.149	0.091	0.103	0.910	0.421	No	3	0.149	0.091	0.103	0.910	0.423
MASP1	DBP	Inverse variance weighted	3	-0.302	0.172	0.080	0.101	0.361	No	3	-0.302	0.172	0.080	0.101	0.363
MATN2	DBP	Inverse variance weighted	3	0.017	0.058	0.772	0.568	0.938	No	3	0.017	0.058	0.772	0.568	0.939
MATN3	DBP	Inverse variance weighted	6	0.043	0.042	0.302	0.732	0.693	No	6	0.043	0.042	0.302	0.732	0.693
MAVS	DBP	Wald ratio	1	0.374	0.274	0.172	-	0.540	No	1	0.374	0.274	0.172	-	0.541
MAX	DBP	Wald ratio	1	0.470	0.279	0.092	-	0.399	No	1	0.470	0.279	0.092	-	0.400
MB	DBP	Wald ratio	1	0.958	0.792	0.226	-	0.607	No	1	0.958	0.792	0.226	-	0.608
MBL2	DBP	Inverse variance weighted	12	0.004	0.025	0.885	0.851	0.980	No	12	0.004	0.025	0.885	0.851	0.980
MCAM	DBP	Inverse variance weighted	3	-0.121	0.332	0.715	0.006	0.931	No	3	-0.121	0.332	0.715	0.006	0.932
MCEE	DBP	Wald ratio	1	-0.101	0.123	0.412	-	0.777	No	1	-0.101	0.123	0.412	-	0.778

ST3: MR causal estimates for plasma proteins on diastolic blood pressure.

Causal candidates prioritized for DBP were marked as "Yes" in column "Prioritized". Effect of plasma protein levels on blood pressure is in mmHg unit.

										Steiger filtering					
**Exposure**	**Outcome**	**Method**	**nsnp**	**Beta**	**SE**	**P-value**	**Cochran's Q P-value**	**FDR-corrected P-value**	**Prioritized**	**nsnp**	**Beta**	**SE**	**P-value**	**Cochran's Q P-value**	**FDR-corrected P-value**
MCEMP1	DBP	Inverse variance weighted	3	0.008	0.101	0.934	0.416	0.986	No	3	0.008	0.101	0.934	0.416	0.986
MCFD2	DBP	Wald ratio	1	0.061	0.173	0.726	-	0.931	No	1	0.061	0.173	0.726	-	0.932
MDGA1	DBP	Inverse variance weighted	13	-0.038	0.021	0.071	0.692	0.336	No	13	-0.038	0.021	0.071	0.692	0.337
MDH1	DBP	Inverse variance weighted	2	-0.407	0.251	0.105	-	0.426	No	2	-0.407	0.251	0.105	-	0.427
MDK	DBP	Inverse variance weighted	3	0.064	0.220	0.770	0.265	0.938	No	3	0.064	0.220	0.770	0.265	0.939
MDM1	DBP	Wald ratio	1	0.067	0.129	0.603	-	0.881	No	1	0.067	0.129	0.603	-	0.882
MECR	DBP	Wald ratio	1	-0.731	0.309	0.018	-	0.140	No	1	-0.731	0.309	0.018	-	0.141
MEGF10	DBP	Inverse variance weighted	4	0.092	0.045	0.039	0.693	0.237	No	4	0.092	0.045	0.039	0.693	0.238
MEGF11	DBP	Inverse variance weighted	2	0.352	0.152	0.021	-	0.154	No	2	0.352	0.152	0.021	-	0.155
MEGF9	DBP	Wald ratio	1	-0.010	0.076	0.895	-	0.982	No	1	-0.010	0.076	0.895	-	0.982
MELTF	DBP	Inverse variance weighted	14	0.018	0.060	0.759	0.123	0.938	No	14	0.018	0.060	0.759	0.123	0.938
MENT	DBP	Inverse variance weighted	3	0.266	0.343	0.437	0.872	0.793	No	3	0.266	0.343	0.437	0.872	0.794
MEP1A	DBP	Inverse variance weighted	3	-0.155	0.163	0.340	0.135	0.724	No	3	-0.155	0.163	0.340	0.135	0.724
MEP1B	DBP	Inverse variance weighted	10	-0.008	0.021	0.717	0.845	0.931	No	10	-0.008	0.021	0.717	0.845	0.932
MEPE	DBP	Inverse variance weighted	2	0.174	0.188	0.354	-	0.734	No	2	0.174	0.188	0.354	-	0.735
MERTK	DBP	Inverse variance weighted	3	0.155	0.088	0.080	0.431	0.361	No	3	0.155	0.088	0.080	0.431	0.363
MET	DBP	Inverse variance weighted	2	0.225	0.252	0.372	-	0.749	No	2	0.225	0.252	0.372	-	0.750
METAP1D	DBP	Wald ratio	1	0.793	0.507	0.117	-	0.459	No	1	0.793	0.507	0.117	-	0.461
METAP2	DBP	Wald ratio	1	-0.623	0.441	0.158	-	0.513	No	1	-0.623	0.441	0.158	-	0.514
MFAP4	DBP	Inverse variance weighted	5	0.169	0.121	0.163	0.213	0.525	No	5	0.169	0.121	0.163	0.213	0.526
MFAP5	DBP	Inverse variance weighted	3	0.100	0.114	0.381	0.761	0.753	No	3	0.100	0.114	0.381	0.761	0.754
MFGE8	DBP	Inverse variance weighted	2	0.227	0.066	0.001	-	0.010	Yes	2	0.227	0.066	0.001	-	0.010
MGLL	DBP	Wald ratio	1	0.727	0.355	0.040	-	0.239	No	1	0.727	0.355	0.040	-	0.241
MGMT	DBP	Inverse variance weighted	5	0.110	0.056	0.047	0.545	0.261	No	5	0.110	0.056	0.047	0.545	0.262
MIA	DBP	Inverse variance weighted	5	0.027	0.065	0.680	0.002	0.918	No	5	0.027	0.065	0.680	0.002	0.918
MICALL2	DBP	Inverse variance weighted	2	-0.148	0.497	0.765	-	0.938	No	2	-0.148	0.497	0.765	-	0.939
MIF	DBP	Wald ratio	1	-0.197	0.210	0.348	-	0.729	No	1	-0.197	0.210	0.348	-	0.730
MILR1	DBP	Inverse variance weighted	23	0.007	0.057	0.899	0.054	0.983	No	23	0.007	0.057	0.899	0.054	0.983
MINDY1	DBP	Inverse variance weighted	2	0.100	0.173	0.563	-	0.859	No	2	0.100	0.173	0.563	-	0.859
MINK1	DBP	Wald ratio	1	-0.202	0.447	0.651	-	0.903	No	1	-0.202	0.447	0.651	-	0.903
MITD1	DBP	Wald ratio	1	-0.433	0.327	0.186	-	0.559	No	1	-0.433	0.327	0.186	-	0.560
MLN	DBP	Inverse variance weighted	4	0.055	0.119	0.645	0.002	0.901	No	4	0.055	0.119	0.645	0.002	0.902
MME	DBP	Wald ratio	1	0.637	0.417	0.127	-	0.477	No	1	0.637	0.417	0.127	-	0.479
MMP1	DBP	Inverse variance weighted	10	0.041	0.056	0.469	0.147	0.810	No	10	0.041	0.056	0.469	0.147	0.811
MMP10	DBP	Inverse variance weighted	6	-0.011	0.075	0.878	0.264	0.980	No	6	-0.011	0.075	0.878	0.264	0.980
MMP12	DBP	Inverse variance weighted	7	-0.051	0.046	0.264	0.465	0.652	No	7	-0.051	0.046	0.264	0.465	0.653
MMP13	DBP	Wald ratio	1	-0.921	0.551	0.095	-	0.402	No	1	-0.921	0.551	0.095	-	0.403
MMP3	DBP	Inverse variance weighted	7	-0.004	0.172	0.983	0.526	1.000	No	7	-0.004	0.172	0.983	0.526	1.000
MMP7	DBP	Inverse variance weighted	2	0.035	0.240	0.885	-	0.980	No	2	0.035	0.240	0.885	-	0.980
MMP8	DBP	Inverse variance weighted	15	0.035	0.054	0.520	0.384	0.838	No	15	0.035	0.054	0.520	0.384	0.838
MMP9	DBP	Wald ratio	1	-0.039	0.166	0.817	-	0.957	No	1	-0.039	0.166	0.817	-	0.958
MMUT	DBP	Wald ratio	1	0.088	0.238	0.712	-	0.931	No	1	0.088	0.238	0.712	-	0.932
MNDA	DBP	Wald ratio	1	-1.023	0.975	0.294	-	0.683	No	1	-1.023	0.975	0.294	-	0.684
MOCS2	DBP	Inverse variance weighted	7	0.003	0.103	0.979	0.000	0.999	No	7	0.003	0.103	0.979	0.000	0.999
MPHOSPH8	DBP	Wald ratio	1	1.273	0.385	0.001	-	0.017	Yes	1	1.273	0.385	0.001	-	0.017
MPI	DBP	Inverse variance weighted	3	-1.236	0.374	0.001	0.000	0.017	No	3	-1.236	0.374	0.001	0.000	0.017
MPO	DBP	Inverse variance weighted	4	-0.007	0.136	0.958	0.043	0.992	No	4	-0.007	0.136	0.958	0.043	0.992
MRC1	DBP	Inverse variance weighted	5	-0.201	0.200	0.313	0.001	0.703	No	5	-0.201	0.200	0.313	0.001	0.704
MRI1	DBP	Inverse variance weighted	5	-0.126	0.172	0.464	0.218	0.807	No	5	-0.126	0.172	0.464	0.218	0.808
MRPL28	DBP	Wald ratio	1	-1.229	0.615	0.045	-	0.255	No	1	-1.229	0.615	0.045	-	0.256
MSLN	DBP	Inverse variance weighted	8	0.046	0.064	0.475	0.008	0.811	No	8	0.046	0.064	0.475	0.008	0.812
MSMB	DBP	Inverse variance weighted	8	-0.059	0.041	0.147	0.001	0.493	No	8	-0.059	0.041	0.147	0.001	0.495
MSR1	DBP	Inverse variance weighted	3	0.166	0.060	0.006	0.712	0.074	No	3	0.166	0.060	0.006	0.712	0.075
MSRA	DBP	Wald ratio	1	-3.423	0.417	0.000	-	0.000	Yes	1	-3.423	0.417	0.000	-	0.000
MST1	DBP	Inverse variance weighted	2	0.154	0.024	0.000	-	0.000	Yes	2	0.154	0.024	0.000	-	0.000
MSTN	DBP	Wald ratio	1	0.325	0.478	0.497	-	0.826	No	1	0.325	0.478	0.497	-	0.827

ST3: MR causal estimates for plasma proteins on diastolic blood pressure.

Causal candidates prioritized for DBP were marked as "Yes" in column "Prioritized". Effect of plasma protein levels on blood pressure is in mmHg unit.

										Steiger filtering					
Exposure	Outocme	Method	nsnp	Beta	SE	P-value	Cochran's Q P-value	FDR-corrected P-value	Prioritized	nsnp	Beta	SE	P-value	Cochran's Q P-value	FDR-corrected P-value
MTHFD2	DBP	Wald ratio	1	0.099	0.287	0.729	-	0.933	No	1	0.099	0.287	0.729	-	0.934
MTHFSD	DBP	Inverse variance weighted	2	0.085	0.049	0.087	-	0.388	No	2	0.085	0.049	0.087	-	0.389
MTIF3	DBP	Inverse variance weighted	2	-0.158	0.461	0.732	-	0.935	No	2	-0.158	0.461	0.732	-	0.935
MTR	DBP	Wald ratio	1	0.234	0.628	0.710	-	0.931	No	1	0.234	0.628	0.710	-	0.932
MTSS1	DBP	Inverse variance weighted	2	0.786	0.388	0.043	-	0.251	No	2	0.786	0.388	0.043	-	0.252
MTSS2	DBP	Inverse variance weighted	2	-0.001	0.449	0.998	-	1.000	No	2	-0.001	0.449	0.998	-	1.000
MTUS1	DBP	Inverse variance weighted	3	-0.026	0.151	0.861	0.172	0.976	No	3	-0.026	0.151	0.861	0.172	0.976
MUC13	DBP	Inverse variance weighted	4	0.140	0.102	0.173	0.766	0.543	No	4	0.140	0.102	0.173	0.766	0.544
MUC16	DBP	Wald ratio	1	-0.879	0.367	0.017	-	0.136	No	1	-0.879	0.367	0.017	-	0.137
MUC2	DBP	Inverse variance weighted	2	-0.118	0.098	0.227	-	0.609	No	2	-0.118	0.098	0.227	-	0.610
MVK	DBP	Wald ratio	1	-0.780	0.294	0.008	-	0.085	No	1	-0.780	0.294	0.008	-	0.085
MXRA8	DBP	Wald ratio	1	0.768	0.497	0.122	-	0.473	No	1	0.768	0.497	0.122	-	0.475
MYBPC2	DBP	Wald ratio	1	0.392	0.468	0.402	-	0.770	No	1	0.392	0.468	0.402	-	0.770
MYDGF	DBP	Wald ratio	1	0.367	0.277	0.185	-	0.559	No	1	0.367	0.277	0.185	-	0.560
MYO9B	DBP	Inverse variance weighted	2	2.468	1.086	0.023	-	0.165	No	2	2.468	1.086	0.023	-	0.166
MYOC	DBP	Inverse variance weighted	3	-0.016	0.095	0.865	0.255	0.976	No	3	-0.016	0.095	0.865	0.255	0.976
MYOM2	DBP	Inverse variance weighted	2	0.787	0.362	0.030	-	0.198	No	2	0.787	0.362	0.030	-	0.199
MYOM3	DBP	Wald ratio	1	-0.147	0.322	0.648	-	0.902	No	1	-0.147	0.322	0.648	-	0.902
MZB1	DBP	Wald ratio	1	-0.253	0.245	0.302	-	0.693	No	1	-0.253	0.245	0.302	-	0.693
NAA80	DBP	Wald ratio	1	0.676	0.279	0.015	-	0.128	No	1	0.676	0.279	0.015	-	0.129
NAAA	DBP	Inverse variance weighted	9	-0.040	0.039	0.302	0.645	0.693	No	9	-0.040	0.039	0.302	0.645	0.693
NADK	DBP	Wald ratio	1	-0.481	0.091	0.000	-	0.000	Yes	1	-0.481	0.091	0.000	-	0.000
NAGA	DBP	Inverse variance weighted	2	0.667	0.302	0.027	-	0.187	No	2	0.667	0.302	0.027	-	0.188
NAGK	DBP	Inverse variance weighted	2	0.201	0.119	0.093	-	0.400	No	2	0.201	0.119	0.093	-	0.401
NAGPA	DBP	Inverse variance weighted	3	0.023	0.200	0.907	0.000	0.983	No	3	0.023	0.200	0.907	0.000	0.983
NAMPT	DBP	Wald ratio	1	0.463	0.566	0.413	-	0.777	No	1	0.463	0.566	0.413	-	0.778
NAP1L4	DBP	Wald ratio	1	-0.107	0.108	0.323	-	0.714	No	1	-0.107	0.108	0.323	-	0.715
NAPRT	DBP	Inverse variance weighted	3	0.023	0.094	0.804	0.175	0.952	No	3	0.023	0.094	0.804	0.175	0.953
NBL1	DBP	Inverse variance weighted	2	-0.002	0.376	0.996	-	1.000	No	2	-0.002	0.376	0.996	-	1.000
NBN	DBP	Wald ratio	1	-2.389	0.574	0.000	-	0.001	Yes	1	-2.389	0.574	0.000	-	0.001
NCAM1	DBP	Inverse variance weighted	6	0.125	0.081	0.125	0.165	0.477	No	6	0.125	0.081	0.125	0.165	0.478
NCAM2	DBP	Inverse variance weighted	8	0.021	0.039	0.598	0.540	0.879	No	8	0.021	0.039	0.598	0.540	0.880
NCAN	DBP	Inverse variance weighted	3	-0.818	0.308	0.008	0.013	0.085	No	3	-0.818	0.308	0.008	0.013	0.085
NCF2	DBP	Wald ratio	1	-0.578	0.211	0.006	-	0.076	No	1	-0.578	0.211	0.006	-	0.076
NCR1	DBP	Inverse variance weighted	3	-0.045	0.092	0.624	0.822	0.891	No	3	-0.045	0.092	0.624	0.822	0.892
NCR3LG1	DBP	Inverse variance weighted	4	-0.210	0.139	0.129	0.005	0.480	No	4	-0.210	0.139	0.129	0.005	0.481
NCS1	DBP	Inverse variance weighted	3	-0.329	0.371	0.376	0.019	0.751	No	3	-0.329	0.371	0.376	0.019	0.752
NECTIN2	DBP	Inverse variance weighted	3	0.110	0.130	0.398	0.866	0.769	No	3	0.110	0.130	0.398	0.866	0.769
NECTIN4	DBP	Wald ratio	1	0.039	0.082	0.632	-	0.894	No	1	0.039	0.082	0.632	-	0.895
NELL1	DBP	Inverse variance weighted	9	0.142	0.063	0.025	0.649	0.172	No	9	0.142	0.063	0.025	0.649	0.173
NELL2	DBP	Inverse variance weighted	2	-0.023	0.121	0.852	-	0.973	No	2	-0.023	0.121	0.852	-	0.973
NEO1	DBP	Wald ratio	1	-0.845	0.347	0.015	-	0.125	No	1	-0.845	0.347	0.015	-	0.126
NEXN	DBP	Wald ratio	1	0.006	0.302	0.985	-	1.000	No	1	0.006	0.302	0.985	-	1.000
NFASC	DBP	Inverse variance weighted	7	-0.140	0.096	0.142	0.017	0.492	No	7	-0.140	0.096	0.142	0.017	0.493
NFATC1	DBP	Inverse variance weighted	2	-0.283	0.168	0.091	-	0.396	No	2	-0.283	0.168	0.091	-	0.398
NFE2	DBP	Wald ratio	1	-0.932	0.347	0.007	-	0.083	No	1	-0.932	0.347	0.007	-	0.084
NFKB1	DBP	Wald ratio	1	-0.171	0.201	0.394	-	0.767	No	1	-0.171	0.201	0.394	-	0.768
NFKBIE	DBP	Wald ratio	1	-0.011	0.107	0.915	-	0.983	No	1	-0.011	0.107	0.915	-	0.983
NFU1	DBP	Wald ratio	1	-0.452	0.192	0.019	-	0.143	No	1	-0.452	0.192	0.019	-	0.144
NGF	DBP	Wald ratio	1	-2.082	0.642	0.001	-	0.020	Yes	1	-2.082	0.642	0.001	-	0.020
NHLRC3	DBP	Inverse variance weighted	8	-0.006	0.094	0.953	0.199	0.992	No	8	-0.006	0.094	0.953	0.199	0.992
NID1	DBP	Inverse variance weighted	8	-0.116	0.121	0.336	0.944	0.724	No	8	-0.116	0.121	0.336	0.944	0.724
NID2	DBP	Inverse variance weighted	5	0.031	0.049	0.524	0.185	0.840	No	5	0.031	0.049	0.524	0.185	0.840
NINJ1	DBP	Inverse variance weighted	2	0.070	0.324	0.830	-	0.963	No	2	0.070	0.324	0.830	-	0.963
NIT1	DBP	Wald ratio	1	-0.129	0.098	0.189	-	0.561	No	1	-0.129	0.098	0.189	-	0.562
NIT2	DBP	Wald ratio	1	-0.095	0.325	0.770	-	0.938	No	1	-0.095	0.325	0.770	-	0.939

ST3: MR causal estimates for plasma proteins on diastolic blood pressure.

Causal candidates prioritized for DBP were marked as "Yes" in column "Prioritized". Effect of plasma protein levels on blood pressure is in mmHg unit.

										Steiger filtering					
Exposure	Outocme	Method	nsnp	Beta	SE	P-value	Cochran's Q P-value	FDR-corrected P-value	Prioritized	nsnp	Beta	SE	P-value	Cochran's Q P-value	FDR-corrected P-value
NME3	DBP	Inverse variance weighted	3	-0.199	0.148	0.178	0.536	0.550	No	3	-0.199	0.148	0.178	0.536	0.552
NMI	DBP	Wald ratio	1	0.019	0.051	0.712	-	0.931	No	1	0.019	0.051	0.712	-	0.932
NM-T1	DBP	Inverse variance weighted	2	-0.044	0.347	0.899	-	0.983	No	2	-0.044	0.347	0.899	-	0.983
NOMO1	DBP	Wald ratio	1	0.707	0.237	0.003	-	0.040	Yes	1	0.707	0.237	0.003	-	0.041
NOS1	DBP	Inverse variance weighted	3	0.170	0.207	0.413	0.198	0.777	No	3	0.170	0.207	0.413	0.198	0.778
NOS2	DBP	Inverse variance weighted	2	-0.017	0.227	0.939	-	0.988	No	2	-0.017	0.227	0.939	-	0.988
NOS3	DBP	Wald ratio	1	-1.790	0.492	0.000	-	0.006	Yes	1	-1.790	0.492	0.000	-	0.006
NOTCH1	DBP	Inverse variance weighted	2	-0.302	0.658	0.646	-	0.901	No	2	-0.302	0.658	0.646	-	0.902
NOTCH2	DBP	Inverse variance weighted	3	-0.243	0.193	0.208	0.352	0.586	No	3	-0.243	0.193	0.208	0.352	0.587
NOTCH3	DBP	Inverse variance weighted	4	0.331	0.271	0.221	0.217	0.600	No	4	0.331	0.271	0.221	0.217	0.601
NPC2	DBP	Inverse variance weighted	3	-0.230	0.129	0.075	0.979	0.349	No	3	-0.230	0.129	0.075	0.979	0.350
NPHS1	DBP	Inverse variance weighted	7	-0.026	0.066	0.690	0.105	0.925	No	7	-0.026	0.066	0.690	0.105	0.925
NPL	DBP	Inverse variance weighted	3	0.047	0.107	0.660	0.222	0.909	No	3	0.047	0.107	0.660	0.222	0.909
NPPB	DBP	Wald ratio	1	-1.443	0.129	0.000	-	0.000	Yes	1	-1.443	0.129	0.000	-	0.000
NPPC	DBP	Inverse variance weighted	2	-0.614	0.569	0.281	-	0.674	No	2	-0.614	0.569	0.281	-	0.675
NPTX1	DBP	Inverse variance weighted	6	-0.159	0.067	0.018	0.050	0.140	No	6	-0.159	0.067	0.018	0.050	0.141
NPTXR	DBP	Inverse variance weighted	4	0.033	0.061	0.592	0.083	0.876	No	4	0.033	0.061	0.592	0.083	0.876
NPY	DBP	Wald ratio	1	-1.342	0.472	0.004	-	0.060	No	1	-1.342	0.472	0.004	-	0.060
NRCAM	DBP	Inverse variance weighted	3	0.042	0.081	0.602	0.729	0.881	No	3	0.042	0.081	0.602	0.729	0.881
NRP1	DBP	Inverse variance weighted	6	0.047	0.097	0.624	0.116	0.891	No	6	0.047	0.097	0.624	0.116	0.892
NRP2	DBP	Inverse variance weighted	5	-0.047	0.153	0.756	0.057	0.938	No	5	-0.047	0.153	0.756	0.057	0.938
NRTN	DBP	Wald ratio	1	-0.115	0.376	0.761	-	0.938	No	1	-0.115	0.376	0.761	-	0.939
NSFL1C	DBP	Inverse variance weighted	2	0.188	0.267	0.481	-	0.814	No	2	0.188	0.267	0.481	-	0.815
NT5C	DBP	Inverse variance weighted	4	0.007	0.042	0.868	0.598	0.977	No	4	0.007	0.042	0.868	0.598	0.977
NT5C3A	DBP	Wald ratio	1	0.359	0.216	0.096	-	0.404	No	1	0.359	0.216	0.096	-	0.405
NT5E	DBP	Inverse variance weighted	5	0.150	0.088	0.088	0.001	0.388	No	5	0.150	0.088	0.088	0.001	0.390
NTF3	DBP	Inverse variance weighted	4	0.528	0.192	0.006	0.089	0.076	No	4	0.528	0.192	0.006	0.089	0.076
NTF4	DBP	Wald ratio	1	-1.096	0.599	0.067	-	0.325	No	1	-1.096	0.599	0.067	-	0.326
NTRK2	DBP	Inverse variance weighted	2	-0.578	0.580	0.319	-	0.710	No	2	-0.578	0.580	0.319	-	0.711
NTRK3	DBP	Inverse variance weighted	2	-0.306	0.113	0.007	-	0.081	No	2	-0.306	0.113	0.007	-	0.082
NUB1	DBP	Inverse variance weighted	2	-0.243	0.535	0.650	-	0.902	No	2	-0.243	0.535	0.650	-	0.902
NUCB2	DBP	Inverse variance weighted	2	-0.209	0.057	0.000	-	0.005	Yes	2	-0.209	0.057	0.000	-	0.005
NUDT15	DBP	Wald ratio	1	0.515	0.660	0.435	-	0.793	No	1	0.515	0.660	0.435	-	0.794
NUDT16	DBP	Wald ratio	1	-0.206	0.090	0.022	-	0.160	No	1	-0.206	0.090	0.022	-	0.161
NUDT2	DBP	Inverse variance weighted	3	-0.330	0.159	0.037	0.038	0.231	No	3	-0.330	0.159	0.037	0.038	0.232
NUDT5	DBP	Wald ratio	1	0.860	0.408	0.035	-	0.221	No	1	0.860	0.408	0.035	-	0.222
NUMB	DBP	Wald ratio	1	0.155	0.269	0.565	-	0.860	No	1	0.155	0.269	0.565	-	0.860
NXPH3	DBP	Inverse variance weighted	3	-0.014	0.172	0.934	0.140	0.986	No	3	-0.014	0.172	0.934	0.140	0.986
OBP2B	DBP	Inverse variance weighted	6	0.007	0.128	0.958	0.000	0.992	No	6	0.007	0.128	0.958	0.000	0.992
OCLN	DBP	Inverse variance weighted	2	-0.366	0.280	0.192	-	0.566	No	2	-0.366	0.280	0.192	-	0.567
ODAM	DBP	Inverse variance weighted	10	-0.102	0.082	0.217	0.262	0.595	No	10	-0.102	0.082	0.217	0.262	0.597
OGA	DBP	Wald ratio	1	-0.276	0.203	0.174	-	0.543	No	1	-0.276	0.203	0.174	-	0.544
OGFR	DBP	Inverse variance weighted	3	0.182	0.236	0.441	0.567	0.795	No	3	0.182	0.236	0.441	0.567	0.796
OGN	DBP	Inverse variance weighted	3	-0.045	0.080	0.573	0.020	0.866	No	3	-0.045	0.080	0.573	0.020	0.866
OLFM4	DBP	Inverse variance weighted	9	0.025	0.033	0.452	0.230	0.800	No	9	0.025	0.033	0.452	0.230	0.800
OLR1	DBP	Inverse variance weighted	2	0.205	0.244	0.400	-	0.769	No	2	0.205	0.244	0.400	-	0.769
OMD	DBP	Inverse variance weighted	2	0.488	1.182	0.680	-	0.918	No	2	0.488	1.182	0.680	-	0.918
OMG	DBP	Wald ratio	1	-0.508	0.259	0.049	-	0.265	No	1	-0.508	0.259	0.049	-	0.266
OMP	DBP	Wald ratio	1	-0.162	0.138	0.241	-	0.627	No	1	-0.162	0.138	0.241	-	0.628
OPLAH	DBP	Wald ratio	1	-0.355	0.127	0.005	-	0.067	No	1	-0.355	0.127	0.005	-	0.068
OPTC	DBP	Inverse variance weighted	4	0.150	0.130	0.248	0.724	0.634	No	4	0.150	0.130	0.248	0.724	0.635
ORM1	DBP	Inverse variance weighted	9	-0.061	0.058	0.294	0.393	0.683	No	9	-0.061	0.058	0.294	0.393	0.684
OSCAR	DBP	Inverse variance weighted	6	-0.033	0.051	0.513	0.230	0.832	No	6	-0.033	0.051	0.513	0.230	0.832
OSM	DBP	Wald ratio	1	0.457	0.366	0.212	-	0.589	No	1	0.457	0.366	0.212	-	0.590
OSMR	DBP	Inverse variance weighted	9	-0.068	0.054	0.206	0.045	0.583	No	9	-0.068	0.054	0.206	0.045	0.584
OSTN	DBP	Inverse variance weighted	2	0.179	0.353	0.612	-	0.888	No	2	0.179	0.353	0.612	-	0.888

ST3: MR causal estimates for plasma proteins on diastolic blood pressure.

Causal candidates prioritized for DBP were marked as "Yes" in column "Prioritized". Effect of plasma protein levels on blood pressure is in mmHg unit.

										Steiger filtering					
Exposure	Outocme	Method	nsnp	Beta	SE	P-value	Cochran's Q P-value	FDR-corrected P-value	Prioritized	nsnp	Beta	SE	P-value	Cochran's Q P-value	FDR-corrected P-value
OTOA	DBP	Wald ratio	1	-0.890	0.430	0.039	-	0.235	No	1	-0.890	0.430	0.039	-	0.237
OTUD6B	DBP	Wald ratio	1	-0.427	0.601	0.478	-	0.813	No	1	-0.427	0.601	0.478	-	0.813
OXCT1	DBP	Wald ratio	1	0.105	0.500	0.834	-	0.963	No	1	0.105	0.500	0.834	-	0.964
OXT	DBP	Inverse variance weighted	5	-0.024	0.042	0.562	0.514	0.859	No	5	-0.024	0.042	0.562	0.514	0.859
P4HB	DBP	Inverse variance weighted	2	-0.326	0.710	0.646	-	0.901	No	2	-0.326	0.710	0.646	-	0.902
PACS2	DBP	Wald ratio	1	-0.288	0.143	0.044	-	0.255	No	1	-0.288	0.143	0.044	-	0.256
PADI2	DBP	Wald ratio	1	0.183	0.183	0.319	-	0.710	No	1	0.183	0.183	0.319	-	0.711
PADI4	DBP	Inverse variance weighted	3	0.033	0.100	0.744	0.276	0.938	No	3	0.033	0.100	0.744	0.276	0.938
PAEP	DBP	Inverse variance weighted	6	-0.021	0.038	0.578	0.238	0.869	No	6	-0.021	0.038	0.578	0.238	0.870
PAFAH2	DBP	Inverse variance weighted	2	-0.231	0.797	0.772	-	0.938	No	2	-0.231	0.797	0.772	-	0.939
PAG1	DBP	Inverse variance weighted	2	-0.185	0.362	0.610	-	0.886	No	2	-0.185	0.362	0.610	-	0.887
PALM	DBP	Inverse variance weighted	3	0.232	0.138	0.094	0.524	0.401	No	3	0.232	0.138	0.094	0.524	0.402
PALM2	DBP	Inverse variance weighted	4	0.335	0.115	0.004	0.645	0.050	No	4	0.335	0.115	0.004	0.645	0.051
PAM	DBP	Inverse variance weighted	16	-0.111	0.035	0.002	0.699	0.025	Yes	16	-0.111	0.035	0.002	0.699	0.026
PAMR1	DBP	Inverse variance weighted	4	0.000	0.063	0.997	0.222	1.000	No	4	0.000	0.063	0.997	0.222	1.000
PAPPA	DBP	Inverse variance weighted	2	-0.508	0.763	0.506	-	0.829	No	2	-0.508	0.763	0.506	-	0.830
PARD3	DBP	Wald ratio	1	-0.135	0.361	0.707	-	0.931	No	1	-0.135	0.361	0.707	-	0.932
PARK7	DBP	Wald ratio	1	-0.065	0.147	0.657	-	0.906	No	1	-0.065	0.147	0.657	-	0.906
PARP1	DBP	Wald ratio	1	0.659	0.203	0.001	-	0.020	Yes	1	0.659	0.203	0.001	-	0.020
PBK	DBP	Wald ratio	1	-0.149	0.501	0.765	-	0.938	No	1	-0.149	0.501	0.765	-	0.939
PBLD	DBP	Inverse variance weighted	2	0.024	0.047	0.605	-	0.883	No	2	0.024	0.047	0.605	-	0.883
PBXIP1	DBP	Wald ratio	1	0.423	0.477	0.376	-	0.751	No	1	0.423	0.477	0.376	-	0.752
PCBD1	DBP	Wald ratio	1	-0.177	0.128	0.166	-	0.530	No	1	-0.177	0.128	0.166	-	0.531
PCBP2	DBP	Wald ratio	1	-1.274	0.475	0.007	-	0.083	No	1	-1.274	0.475	0.007	-	0.084
PCDH1	DBP	Wald ratio	1	0.197	0.297	0.506	-	0.829	No	1	0.197	0.297	0.506	-	0.830
PCDH12	DBP	Inverse variance weighted	4	-0.033	0.086	0.706	0.182	0.931	No	4	-0.033	0.086	0.706	0.182	0.932
PCDH17	DBP	Inverse variance weighted	3	0.070	0.513	0.892	0.009	0.982	No	3	0.070	0.513	0.892	0.009	0.982
PCDH7	DBP	Wald ratio	1	0.217	0.214	0.310	-	0.699	No	1	0.217	0.214	0.310	-	0.700
PCDH9	DBP	Inverse variance weighted	5	0.098	0.078	0.210	0.953	0.588	No	5	0.098	0.078	0.210	0.953	0.589
PCDHB15	DBP	Inverse variance weighted	7	0.002	0.070	0.979	0.210	0.999	No	7	0.002	0.070	0.979	0.210	0.999
PCOLCE	DBP	Wald ratio	1	-0.516	0.140	0.000	-	0.005	Yes	1	-0.516	0.140	0.000	-	0.005
PCSK7	DBP	Inverse variance weighted	2	-0.222	0.072	0.002	-	0.032	Yes	2	-0.222	0.072	0.002	-	0.033
PCSK9	DBP	Inverse variance weighted	3	-0.158	0.075	0.034	0.310	0.218	No	3	-0.158	0.075	0.034	0.310	0.219
PDCD1	DBP	Inverse variance weighted	2	0.171	0.094	0.069	-	0.332	No	2	0.171	0.094	0.069	-	0.333
PDCD1LG2	DBP	Inverse variance weighted	11	-0.001	0.038	0.975	0.950	0.998	No	11	-0.001	0.038	0.975	0.950	0.998
PDCD5	DBP	Inverse variance weighted	4	0.055	0.060	0.359	0.642	0.737	No	4	0.055	0.060	0.359	0.642	0.738
PDCD6	DBP	Inverse variance weighted	7	-0.041	0.037	0.259	0.015	0.648	No	7	-0.041	0.037	0.259	0.015	0.649
PDE5A	DBP	Inverse variance weighted	2	-0.856	0.145	0.000	-	0.000	Yes	2	-0.856	0.145	0.000	-	0.000
PDGFA	DBP	Inverse variance weighted	3	0.116	0.738	0.876	0.007	0.980	No	3	0.116	0.738	0.876	0.007	0.980
PDGFC	DBP	Inverse variance weighted	2	-0.229	0.245	0.351	-	0.731	No	2	-0.229	0.245	0.351	-	0.732
PDGFRA	DBP	Inverse variance weighted	3	0.082	0.084	0.329	0.400	0.717	No	3	0.082	0.084	0.329	0.400	0.718
PDGFRB	DBP	Inverse variance weighted	13	0.235	0.136	0.085	0.031	0.377	No	13	0.235	0.136	0.085	0.031	0.378
PDIA3	DBP	Wald ratio	1	-2.749	0.460	0.000	-	0.000	Yes	1	-2.749	0.460	0.000	-	0.000
PDIA4	DBP	Wald ratio	1	0.025	0.267	0.927	-	0.986	No	1	0.025	0.267	0.927	-	0.986
PDIA5	DBP	Inverse variance weighted	2	-0.053	0.124	0.667	-	0.911	No	2	-0.053	0.124	0.667	-	0.912
PDLIM5	DBP	Wald ratio	1	-1.264	0.569	0.026	-	0.181	No	1	-1.264	0.569	0.026	-	0.182
PDLIM7	DBP	Wald ratio	1	-0.010	0.582	0.986	-	1.000	No	1	-0.010	0.582	0.986	-	1.000
PDZD2	DBP	Inverse variance weighted	3	0.063	0.105	0.547	0.295	0.851	No	3	0.063	0.105	0.547	0.295	0.851
PDZK1	DBP	Wald ratio	1	0.526	0.200	0.009	-	0.089	No	1	0.526	0.200	0.009	-	0.090
PEAR1	DBP	Inverse variance weighted	4	0.193	0.209	0.356	0.007	0.737	No	4	0.193	0.209	0.356	0.007	0.737
PEBP1	DBP	Inverse variance weighted	2	0.142	0.208	0.496	-	0.825	No	2	0.142	0.208	0.496	-	0.826
PECAM1	DBP	Wald ratio	1	-2.334	0.706	0.001	-	0.017	Yes	1	-2.334	0.706	0.001	-	0.017
PECR	DBP	Inverse variance weighted	2	-0.135	0.082	0.098	-	0.408	No	2	-0.135	0.082	0.098	-	0.409
PENK	DBP	Inverse variance weighted	4	0.162	0.145	0.265	0.009	0.652	No	4	0.162	0.145	0.265	0.009	0.653
PEPD	DBP	Inverse variance weighted	3	0.122	0.064	0.058	0.629	0.295	No	3	0.122	0.064	0.058	0.629	0.296
PER3	DBP	Inverse variance weighted	4	-0.090	0.211	0.671	0.000	0.914	No	4	-0.090	0.211	0.671	0.000	0.914

ST3: MR causal estimates for plasma proteins on diastolic blood pressure.
Causal candidates prioritized for DBP were marked as "Yes" in column "Prioritized". Effect of plasma protein levels on blood pressure is in mmHg unit.

										Steiger filtering					
Exposure	Outocme	Method	nsnp	Beta	SE	P-value	Cochran's Q P-value	FDR-corrected P-value	Prioritized	nsnp	Beta	SE	P-value	Cochran's Q P-value	FDR-corrected P-value
PF4	DBP	Wald ratio	1	0.169	0.299	0.572	-	0.865	No	1	0.169	0.299	0.572	-	0.866
PFKFB2	DBP	Wald ratio	1	-0.599	0.123	0.000	-	0.000	Yes	1	-0.599	0.123	0.000	-	0.000
PGD	DBP	Wald ratio	1	-0.138	0.093	0.138	-	0.491	No	1	-0.138	0.093	0.138	-	0.492
PGF	DBP	Inverse variance weighted	2	-0.391	0.150	0.009	-	0.094	No	2	-0.391	0.150	0.009	-	0.095
PGLYRP1	DBP	Wald ratio	1	0.067	0.086	0.438	-	0.793	No	1	0.067	0.086	0.438	-	0.794
PGLYRP2	DBP	Inverse variance weighted	11	-0.019	0.031	0.533	0.087	0.844	No	11	-0.019	0.031	0.533	0.087	0.844
PGLYRP4	DBP	Inverse variance weighted	4	0.001	0.052	0.989	0.967	1.000	No	4	0.001	0.052	0.989	0.967	1.000
PHACTR2	DBP	Wald ratio	1	0.055	0.116	0.632	-	0.894	No	1	0.055	0.116	0.632	-	0.895
PHLDB1	DBP	Wald ratio	1	-0.086	0.525	0.870	-	0.977	No	1	-0.086	0.525	0.870	-	0.977
PHYKPL	DBP	Inverse variance weighted	7	-0.088	0.107	0.412	0.801	0.777	No	7	-0.088	0.107	0.412	0.801	0.778
PI16	DBP	Inverse variance weighted	6	0.313	0.138	0.023	0.020	0.165	No	6	0.313	0.138	0.023	0.020	0.166
PI3	DBP	Wald ratio	1	0.068	0.086	0.427	-	0.788	No	1	0.068	0.086	0.427	-	0.789
PIBF1	DBP	Wald ratio	1	-0.460	0.390	0.238	-	0.624	No	1	-0.460	0.390	0.238	-	0.625
PIGR	DBP	Inverse variance weighted	3	0.022	0.133	0.871	0.850	0.977	No	3	0.022	0.133	0.871	0.850	0.977
PIK3AP1	DBP	Inverse variance weighted	4	0.209	0.105	0.048	0.532	0.261	No	4	0.209	0.105	0.048	0.532	0.262
PIK3IP1	DBP	Inverse variance weighted	3	-0.101	0.127	0.430	0.112	0.791	No	3	-0.101	0.127	0.430	0.112	0.792
PIKFYVE	DBP	Wald ratio	1	-1.391	0.559	0.013	-	0.113	No	1	-1.391	0.559	0.013	-	0.114
PILRA	DBP	Inverse variance weighted	8	-0.062	0.025	0.012	0.983	0.110	No	8	-0.062	0.025	0.012	0.983	0.111
PILRB	DBP	Inverse variance weighted	11	-0.055	0.021	0.011	0.870	0.101	No	11	-0.055	0.021	0.011	0.870	0.102
PINLYP	DBP	Inverse variance weighted	5	-0.008	0.030	0.775	0.613	0.938	No	5	-0.008	0.030	0.775	0.613	0.939
PKD1	DBP	Inverse variance weighted	5	-0.327	0.123	0.008	0.093	0.085	No	5	-0.327	0.123	0.008	0.093	0.085
PKD2	DBP	Wald ratio	1	0.165	0.256	0.518	-	0.837	No	1	0.165	0.256	0.518	-	0.837
PKLR	DBP	Inverse variance weighted	2	0.164	0.135	0.224	-	0.604	No	2	0.164	0.135	0.224	-	0.605
PKN3	DBP	Wald ratio	1	-0.176	0.157	0.261	-	0.651	No	1	-0.176	0.157	0.261	-	0.652
PLA2G10	DBP	Inverse variance weighted	2	0.263	0.360	0.465	-	0.807	No	2	0.263	0.360	0.465	-	0.808
PLA2G15	DBP	Inverse variance weighted	3	0.267	0.148	0.072	0.091	0.340	No	3	0.267	0.148	0.072	0.091	0.341
PLA2G1B	DBP	Wald ratio	1	-2.238	0.468	0.000	-	0.000	Yes	1	-2.238	0.468	0.000	-	0.000
PLA2G2A	DBP	Inverse variance weighted	5	-0.020	0.050	0.686	0.132	0.920	No	5	-0.020	0.050	0.686	0.132	0.921
PLA2G4A	DBP	Inverse variance weighted	2	-0.281	0.568	0.621	-	0.891	No	2	-0.281	0.568	0.621	-	0.892
PLA2G7	DBP	Inverse variance weighted	2	0.039	0.539	0.942	-	0.989	No	2	0.039	0.539	0.942	-	0.989
PLAT	DBP	Wald ratio	1	-0.072	0.254	0.775	-	0.938	No	1	-0.072	0.254	0.775	-	0.939
PLAU	DBP	Inverse variance weighted	4	0.095	0.276	0.730	0.119	0.933	No	4	0.095	0.276	0.730	0.119	0.934
PLAUR	DBP	Inverse variance weighted	3	0.121	0.155	0.438	0.748	0.793	No	3	0.121	0.155	0.438	0.748	0.794
PLB1	DBP	Inverse variance weighted	10	-0.039	0.033	0.235	0.141	0.620	No	10	-0.039	0.033	0.235	0.141	0.621
PLCB2	DBP	Wald ratio	1	-0.061	0.165	0.710	-	0.931	No	1	-0.061	0.165	0.710	-	0.932
PLEKHO1	DBP	Wald ratio	1	-0.266	0.472	0.573	-	0.866	No	1	-0.266	0.472	0.573	-	0.866
PLG	DBP	Inverse variance weighted	18	0.064	0.068	0.341	0.019	0.724	No	18	0.064	0.068	0.341	0.019	0.724
PLIN3	DBP	Wald ratio	1	-0.588	0.318	0.064	-	0.315	No	1	-0.588	0.318	0.064	-	0.316
PLPBP	DBP	Wald ratio	1	-0.653	0.574	0.255	-	0.642	No	1	-0.653	0.574	0.255	-	0.643
PLSCR3	DBP	Wald ratio	1	0.508	0.413	0.219	-	0.598	No	1	0.508	0.413	0.219	-	0.599
PLTP	DBP	Inverse variance weighted	5	0.022	0.074	0.770	0.142	0.938	No	5	0.022	0.074	0.770	0.142	0.939
PLXDC1	DBP	Inverse variance weighted	16	0.047	0.075	0.538	0.103	0.846	No	16	0.047	0.075	0.538	0.103	0.847
PLXDC2	DBP	Inverse variance weighted	3	1.041	0.232	0.000	0.649	0.000	Yes	3	1.041	0.232	0.000	0.649	0.000
PLX-4	DBP	Wald ratio	1	-0.165	0.276	0.551	-	0.854	No	1	-0.165	0.276	0.551	-	0.855
PLXNB2	DBP	Inverse variance weighted	6	-0.009	0.040	0.823	0.181	0.961	No	6	-0.009	0.040	0.823	0.181	0.961
PM20D1	DBP	Inverse variance weighted	8	-0.010	0.028	0.724	0.497	0.931	No	8	-0.010	0.028	0.724	0.497	0.932
PMM2	DBP	Inverse variance weighted	5	0.178	0.105	0.090	0.525	0.394	No	5	0.178	0.105	0.090	0.525	0.395
PMS1	DBP	Wald ratio	1	1.787	0.513	0.000	-	0.010	Yes	1	1.787	0.513	0.000	-	0.010
PMVK	DBP	Wald ratio	1	0.308	0.423	0.466	-	0.808	No	1	0.308	0.423	0.466	-	0.809
PNLIP	DBP	Wald ratio	1	0.559	0.691	0.419	-	0.778	No	1	0.559	0.691	0.419	-	0.779
PNLIPRP1	DBP	Inverse variance weighted	2	0.087	0.104	0.405	-	0.772	No	2	0.087	0.104	0.405	-	0.773
PNLIPRP2	DBP	Inverse variance weighted	11	-0.021	0.020	0.293	0.548	0.683	No	11	-0.021	0.020	0.293	0.548	0.684
PNMA1	DBP	Inverse variance weighted	2	0.468	0.299	0.118	-	0.459	No	2	0.468	0.299	0.118	-	0.461
PODXL	DBP	Inverse variance weighted	2	-0.007	0.444	0.988	-	1.000	No	2	-0.007	0.444	0.988	-	1.000
PODXL2	DBP	Inverse variance weighted	3	-0.124	0.222	0.577	0.001	0.869	No	3	-0.124	0.222	0.577	0.001	0.870
POMC	DBP	Wald ratio	1	-0.723	0.328	0.028	-	0.189	No	1	-0.723	0.328	0.028	-	0.190

ST3: MR causal estimates for plasma proteins on diastolic blood pressure.

Causal candidates prioritized for DBP were marked as "Yes" in column "Prioritized". Effect of plasma protein levels on blood pressure is in mmHg unit.

										Steiger filtering					
Exposure	Outocme	Method	nsnp	Beta	SE	P-value	Cochran's Q P-value	FDR-corrected P-value	Prioritized	nsnp	Beta	SE	P-value	Cochran's Q P-value	FDR-corrected P-value
PON1	DBP	Inverse variance weighted	10	-0.018	0.026	0.473	0.640	0.811	No	10	-0.018	0.026	0.473	0.640	0.812
PON2	DBP	Inverse variance weighted	10	0.065	0.040	0.101	0.037	0.416	No	10	0.065	0.040	0.101	0.037	0.417
PON3	DBP	Inverse variance weighted	16	0.012	0.058	0.831	0.869	0.963	No	16	0.012	0.058	0.831	0.869	0.963
POSTN	DBP	Inverse variance weighted	3	0.184	0.139	0.184	0.105	0.559	No	3	0.184	0.139	0.184	0.105	0.560
PPCDC	DBP	Inverse variance weighted	8	0.198	0.182	0.276	0.000	0.668	No	8	0.198	0.182	0.276	0.000	0.669
PPIE	DBP	Wald ratio	1	-0.874	0.460	0.058	-	0.293	No	1	-0.874	0.460	0.058	-	0.294
PPL	DBP	Inverse variance weighted	3	0.599	0.230	0.009	0.712	0.094	No	3	0.599	0.230	0.009	0.712	0.095
PPM1F	DBP	Wald ratio	1	0.395	0.429	0.357	-	0.737	No	1	0.395	0.429	0.357	-	0.738
PPME1	DBP	Wald ratio	1	0.680	0.513	0.185	-	0.559	No	1	0.680	0.513	0.185	-	0.560
PPP1R12A	DBP	Wald ratio	1	0.774	0.696	0.266	-	0.653	No	1	0.774	0.696	0.266	-	0.654
PPP1R14A	DBP	Inverse variance weighted	2	0.125	0.117	0.286	-	0.677	No	2	0.125	0.117	0.286	-	0.678
PPP1R14D	DBP	Wald ratio	1	0.696	0.434	0.109	-	0.437	No	1	0.696	0.434	0.109	-	0.438
PPP1R9B	DBP	Wald ratio	1	1.435	0.585	0.014	-	0.122	No	1	1.435	0.585	0.014	-	0.123
PPP2R5A	DBP	Wald ratio	1	0.427	0.255	0.095	-	0.402	No	1	0.427	0.255	0.095	-	0.403
PRAP1	DBP	Inverse variance weighted	5	-0.048	0.069	0.493	0.192	0.823	No	5	-0.048	0.069	0.493	0.192	0.824
PRCP	DBP	Inverse variance weighted	2	0.028	0.337	0.934	-	0.986	No	2	0.028	0.337	0.934	-	0.986
PRDX1	DBP	Wald ratio	1	-1.497	0.370	0.000	-	0.002	Yes	1	-1.497	0.370	0.000	-	0.002
PRDX2	DBP	Inverse variance weighted	2	0.097	0.122	0.428	-	0.790	No	2	0.097	0.122	0.428	-	0.790
PRDX3	DBP	Wald ratio	1	0.154	0.379	0.684	-	0.920	No	1	0.154	0.379	0.684	-	0.920
PRDX5	DBP	Inverse variance weighted	2	0.643	0.612	0.293	-	0.683	No	2	0.643	0.612	0.293	-	0.684
PRDX6	DBP	Inverse variance weighted	2	-0.125	0.167	0.456	-	0.802	No	2	-0.125	0.167	0.456	-	0.802
PRELP	DBP	Inverse variance weighted	4	-0.038	0.049	0.436	0.652	0.793	No	4	-0.038	0.049	0.436	0.652	0.794
PRG2	DBP	Wald ratio	1	0.464	0.233	0.047	-	0.259	No	1	0.464	0.233	0.047	-	0.260
PRG3	DBP	Inverse variance weighted	2	-0.007	0.203	0.974	-	0.998	No	2	-0.007	0.203	0.974	-	0.998
PRKAB1	DBP	Wald ratio	1	0.252	0.133	0.057	-	0.293	No	1	0.252	0.133	0.057	-	0.294
PRKAR2A	DBP	Wald ratio	1	0.624	0.481	0.194	-	0.567	No	1	0.624	0.481	0.194	-	0.568
PRKD2	DBP	Wald ratio	1	0.502	0.557	0.368	-	0.744	No	1	0.502	0.557	0.368	-	0.745
PRKG1	DBP	Inverse variance weighted	3	-0.212	0.287	0.459	0.002	0.805	No	3	-0.212	0.287	0.459	0.002	0.805
PRND	DBP	Inverse variance weighted	5	0.059	0.089	0.506	0.591	0.829	No	5	0.059	0.089	0.506	0.591	0.830
PROC	DBP	Inverse variance weighted	2	0.005	0.185	0.980	-	0.999	No	2	0.005	0.185	0.980	-	0.999
PROCR	DBP	Wald ratio	1	-0.179	0.057	0.002	-	0.025	Yes	1	-0.179	0.057	0.002	-	0.026
PROK1	DBP	Inverse variance weighted	6	-0.037	0.069	0.594	0.201	0.876	No	6	-0.037	0.069	0.594	0.201	0.876
PROS1	DBP	Inverse variance weighted	5	0.001	0.192	0.996	0.377	1.000	No	5	0.001	0.192	0.996	0.377	1.000
PRR4	DBP	Inverse variance weighted	6	0.083	0.030	0.005	0.951	0.066	No	6	0.083	0.030	0.005	0.951	0.067
PRRT3	DBP	Inverse variance weighted	3	-0.137	0.116	0.234	0.508	0.620	No	3	-0.137	0.116	0.234	0.508	0.621
PRSS2	DBP	Wald ratio	1	-0.122	0.169	0.469	-	0.810	No	1	-0.122	0.169	0.469	-	0.811
PRSS22	DBP	Inverse variance weighted	2	0.073	0.211	0.730	-	0.933	No	2	0.073	0.211	0.730	-	0.934
PRSS27	DBP	Inverse variance weighted	3	-0.200	0.123	0.105	0.324	0.426	No	3	-0.200	0.123	0.105	0.324	0.427
PRSS53	DBP	Inverse variance weighted	10	0.165	0.027	0.000	0.891	0.000	Yes	10	0.165	0.027	0.000	0.891	0.000
PRSS8	DBP	Inverse variance weighted	2	1.248	0.757	0.099	-	0.413	No	2	1.248	0.757	0.099	-	0.414
PRTFDC1	DBP	Inverse variance weighted	2	-0.360	0.114	0.002	-	0.026	Yes	2	-0.360	0.114	0.002	-	0.026
PRTG	DBP	Inverse variance weighted	6	-0.068	0.053	0.197	0.796	0.567	No	6	-0.068	0.053	0.197	0.796	0.568
PRTN3	DBP	Inverse variance weighted	7	-0.084	0.069	0.225	0.093	0.606	No	7	-0.084	0.069	0.225	0.093	0.607
PSAP	DBP	Inverse variance weighted	3	0.210	0.111	0.058	0.952	0.295	No	3	0.210	0.111	0.058	0.952	0.296
PSAPL1	DBP	Inverse variance weighted	5	0.006	0.041	0.893	0.834	0.982	No	5	0.006	0.041	0.893	0.834	0.982
PSCA	DBP	Inverse variance weighted	18	-0.040	0.022	0.066	0.215	0.323	No	18	-0.040	0.022	0.066	0.215	0.325
PSG1	DBP	Inverse variance weighted	10	-0.025	0.031	0.418	0.293	0.778	No	10	-0.025	0.031	0.418	0.293	0.779
PSMD5	DBP	Wald ratio	1	0.725	0.477	0.128	-	0.480	No	1	0.725	0.477	0.128	-	0.481
PSMD9	DBP	Wald ratio	1	-0.387	0.126	0.002	-	0.031	No	1	-0.387	0.126	0.002	-	0.032
PSME1	DBP	Wald ratio	1	0.362	0.263	0.169	-	0.533	No	1	0.362	0.263	0.169	-	0.534
PSME2	DBP	Wald ratio	1	0.328	0.238	0.169	-	0.533	No	1	0.328	0.238	0.169	-	0.534
PSMG4	DBP	Inverse variance weighted	2	0.112	0.158	0.477	-	0.813	No	2	0.112	0.158	0.477	-	0.813
PSRC1	DBP	Inverse variance weighted	2	-0.442	0.117	0.000	-	0.004	Yes	2	-0.442	0.117	0.000	-	0.004
PSTPIP2	DBP	Inverse variance weighted	2	0.061	0.383	0.874	-	0.980	No	2	0.061	0.383	0.874	-	0.980
PTGES2	DBP	Wald ratio	1	0.614	0.297	0.039	-	0.236	No	1	0.614	0.297	0.039	-	0.237
PTGR1	DBP	Inverse variance weighted	9	-0.062	0.069	0.367	0.086	0.743	No	9	-0.062	0.069	0.367	0.086	0.744

ST3: MR causal estimates for plasma proteins on diastolic blood pressure.

Causal candidates prioritized for DBP were marked as "Yes" in column "Prioritized". Effect of plasma protein levels on blood pressure is in mmHg unit.

										Steiger filtering					
Exposure	Outocme	Method	nsnp	Beta	SE	P-value	Cochran's Q P-value	FDR-corrected P-value	Prioritized	nsnp	Beta	SE	P-value	Cochran's Q P-value	FDR-corrected P-value
PTH1R	DBP	Inverse variance weighted	9	-0.020	0.272	0.940	0.000	0.988	No	9	-0.020	0.272	0.940	0.000	0.988
PTK7	DBP	Wald ratio	1	0.382	0.402	0.341	-	0.724	No	1	0.382	0.402	0.341	-	0.724
PTN	DBP	Wald ratio	1	-0.059	0.323	0.854	-	0.973	No	1	-0.059	0.323	0.854	-	0.973
PTPRB	DBP	Inverse variance weighted	5	-0.005	0.062	0.932	0.254	0.986	No	5	-0.005	0.062	0.932	0.254	0.986
PTPRC	DBP	Inverse variance weighted	4	-0.108	0.113	0.338	0.369	0.724	No	4	-0.108	0.113	0.338	0.369	0.724
PTPRF	DBP	Wald ratio	1	1.067	0.294	0.000	-	0.006	Yes	1	1.067	0.294	0.000	-	0.006
PTPRH	DBP	Inverse variance weighted	6	0.043	0.036	0.236	0.477	0.621	No	6	0.043	0.036	0.236	0.477	0.622
PTPRK	DBP	Wald ratio	1	0.464	0.262	0.076	-	0.351	No	1	0.464	0.262	0.076	-	0.352
PTPRM	DBP	Inverse variance weighted	5	0.327	0.199	0.099	0.800	0.413	No	5	0.327	0.199	0.099	0.800	0.414
PTPRN2	DBP	Inverse variance weighted	2	0.360	0.295	0.223	-	0.603	No	2	0.360	0.295	0.223	-	0.604
PTPRS	DBP	Inverse variance weighted	9	-0.092	0.148	0.532	0.000	0.844	No	9	-0.092	0.148	0.532	0.000	0.844
PTPRZ1	DBP	Inverse variance weighted	2	-0.026	0.193	0.895	-	0.982	No	2	-0.026	0.193	0.895	-	0.982
PTRHD1	DBP	Wald ratio	1	3.011	0.617	0.000	-	0.000	Yes	1	3.011	0.617	0.000	-	0.000
PTS	DBP	Wald ratio	1	-0.120	0.114	0.291	-	0.682	No	1	-0.120	0.114	0.291	-	0.683
PTX3	DBP	Inverse variance weighted	2	0.638	0.429	0.137	-	0.490	No	2	0.638	0.429	0.137	-	0.491
PVALB	DBP	Inverse variance weighted	7	0.006	0.029	0.827	0.675	0.963	No	7	0.006	0.029	0.827	0.675	0.963
PVR	DBP	Inverse variance weighted	7	-0.019	0.034	0.581	0.392	0.870	No	7	-0.019	0.034	0.581	0.392	0.870
PXDNL	DBP	Inverse variance weighted	5	0.024	0.093	0.800	0.460	0.950	No	5	0.024	0.093	0.800	0.460	0.950
PXN	DBP	Inverse variance weighted	2	-0.128	0.096	0.180	-	0.554	No	2	-0.128	0.096	0.180	-	0.555
PYDC1	DBP	Inverse variance weighted	3	-0.313	0.080	0.000	0.641	0.003	Yes	3	-0.313	0.080	0.000	0.641	0.003
PYY	DBP	Inverse variance weighted	2	-0.053	0.236	0.823	-	0.961	No	2	-0.053	0.236	0.823	-	0.961
PZP	DBP	Inverse variance weighted	7	-0.020	0.064	0.755	0.853	0.938	No	7	-0.020	0.064	0.755	0.853	0.938
QDPR	DBP	Inverse variance weighted	2	0.007	0.339	0.983	-	1.000	No	2	0.007	0.339	0.983	-	1.000
QPCT	DBP	Inverse variance weighted	4	0.258	0.091	0.005	0.108	0.060	No	4	0.258	0.091	0.005	0.108	0.061
QSOX1	DBP	Inverse variance weighted	4	0.003	0.062	0.959	0.035	0.993	No	4	0.003	0.062	0.959	0.035	0.993
RAB11FIP3	DBP	Inverse variance weighted	2	0.596	0.393	0.130	-	0.480	No	2	0.596	0.393	0.130	-	0.481
RAB2B	DBP	Wald ratio	1	0.089	0.146	0.542	-	0.850	No	1	0.089	0.146	0.542	-	0.851
RAB44	DBP	Inverse variance weighted	2	0.062	0.195	0.752	-	0.938	No	2	0.062	0.195	0.752	-	0.938
RAB6A	DBP	Wald ratio	1	0.695	0.476	0.144	-	0.492	No	1	0.695	0.476	0.144	-	0.493
RABEP1	DBP	Wald ratio	1	-0.174	0.298	0.560	-	0.858	No	1	-0.174	0.298	0.560	-	0.858
RABEPK	DBP	Wald ratio	1	0.118	0.155	0.446	-	0.797	No	1	0.118	0.155	0.446	-	0.798
RABGAP1L	DBP	Wald ratio	1	-0.219	0.370	0.554	-	0.854	No	1	-0.219	0.370	0.554	-	0.855
RAD23B	DBP	Wald ratio	1	-0.410	0.404	0.310	-	0.700	No	1	-0.410	0.404	0.310	-	0.701
RALB	DBP	Wald ratio	1	-0.109	0.207	0.600	-	0.880	No	1	-0.109	0.207	0.600	-	0.880
RALY	DBP	Wald ratio	1	0.355	0.328	0.278	-	0.670	No	1	0.355	0.328	0.278	-	0.671
RANBP1	DBP	Wald ratio	1	-0.773	0.417	0.064	-	0.313	No	1	-0.773	0.417	0.064	-	0.315
RARRES1	DBP	Inverse variance weighted	5	0.134	0.050	0.007	0.904	0.080	No	5	0.134	0.050	0.007	0.904	0.081
RARRES2	DBP	Wald ratio	1	-0.233	0.121	0.054	-	0.282	No	1	-0.233	0.121	0.054	-	0.283
RASSF2	DBP	Inverse variance weighted	2	0.095	0.204	0.641	-	0.900	No	2	0.095	0.204	0.641	-	0.900
RBKS	DBP	Inverse variance weighted	4	-0.051	0.077	0.508	0.310	0.830	No	4	-0.051	0.077	0.508	0.310	0.831
RBM17	DBP	Wald ratio	1	-0.178	0.167	0.288	-	0.679	No	1	-0.178	0.167	0.288	-	0.680
RBM19	DBP	Wald ratio	1	-0.587	0.616	0.340	-	0.724	No	1	-0.587	0.616	0.340	-	0.724
RBP1	DBP	Wald ratio	1	-0.089	0.243	0.713	-	0.931	No	1	-0.089	0.243	0.713	-	0.932
RBP2	DBP	Wald ratio	1	0.366	0.337	0.278	-	0.670	No	1	0.366	0.337	0.278	-	0.671
RBP5	DBP	Inverse variance weighted	2	-0.150	0.102	0.140	-	0.492	No	2	-0.150	0.102	0.140	-	0.493
RBP7	DBP	Wald ratio	1	0.154	0.094	0.103	-	0.422	No	1	0.154	0.094	0.103	-	0.423
RBPMS2	DBP	Inverse variance weighted	2	-0.258	0.636	0.685	-	0.920	No	2	-0.258	0.636	0.685	-	0.920
RECK	DBP	Inverse variance weighted	5	-0.084	0.219	0.702	0.000	0.931	No	5	-0.084	0.219	0.702	0.000	0.932
REEP4	DBP	Inverse variance weighted	2	0.327	0.137	0.017	-	0.136	No	2	0.327	0.137	0.017	-	0.137
REG1A	DBP	Inverse variance weighted	3	-0.109	0.087	0.213	0.400	0.590	No	3	-0.109	0.087	0.213	0.400	0.591
REG1B	DBP	Inverse variance weighted	3	-0.064	0.121	0.595	0.040	0.876	No	3	-0.064	0.121	0.595	0.040	0.876
REG3A	DBP	Inverse variance weighted	6	-0.140	0.190	0.462	0.001	0.807	No	6	-0.140	0.190	0.462	0.001	0.807
REG3G	DBP	Inverse variance weighted	4	0.099	0.090	0.269	0.530	0.657	No	4	0.099	0.090	0.269	0.530	0.658
REG4	DBP	Inverse variance weighted	4	0.040	0.203	0.843	0.008	0.969	No	4	0.040	0.203	0.843	0.008	0.969
RELT	DBP	Inverse variance weighted	2	-0.430	0.076	0.000	-	0.000	Yes	2	-0.430	0.076	0.000	-	0.000
REN	DBP	Inverse variance weighted	3	0.045	0.265	0.866	0.130	0.976	No	3	0.045	0.265	0.866	0.130	0.976

ST3: MR causal estimates for plasma proteins on diastolic blood pressure.

Causal candidates prioritized for DBP were marked as "Yes" in column "Prioritized". Effect of plasma protein levels on blood pressure is in mmHg unit.

										Steiger filtering					
Exposure	Outocme	Method	nsnp	Beta	SE	P-value	Cochran's Q P-value	FDR-corrected P-value	Prioritized	nsnp	Beta	SE	P-value	Cochran's Q P-value	FDR-corrected P-value
RET	DBP	Inverse variance weighted	4	0.010	0.078	0.899	0.188	0.983	No	4	0.010	0.078	0.899	0.188	0.983
RETN	DBP	Inverse variance weighted	7	-0.021	0.089	0.815	0.049	0.957	No	7	-0.021	0.089	0.815	0.049	0.957
RGCC	DBP	Wald ratio	1	1.074	0.561	0.056	-	0.289	No	1	1.074	0.561	0.056	-	0.290
RGMA	DBP	Inverse variance weighted	8	0.099	0.112	0.379	0.030	0.751	No	8	0.099	0.112	0.379	0.030	0.752
RGMB	DBP	Inverse variance weighted	4	-0.268	0.215	0.211	0.019	0.589	No	4	-0.268	0.215	0.211	0.019	0.590
RHOC	DBP	Wald ratio	1	0.138	0.304	0.650	-	0.902	No	1	0.138	0.304	0.650	-	0.902
RIDA	DBP	Inverse variance weighted	2	-0.069	0.113	0.544	-	0.850	No	2	-0.069	0.113	0.544	-	0.851
RILP	DBP	Inverse variance weighted	3	-0.182	0.318	0.567	0.795	0.863	No	3	-0.182	0.318	0.567	0.795	0.863
RILPL2	DBP	Inverse variance weighted	2	0.316	0.442	0.474	-	0.811	No	2	0.316	0.442	0.474	-	0.812
RLN2	DBP	Inverse variance weighted	6	-0.011	0.137	0.936	0.486	0.986	No	6	-0.011	0.137	0.936	0.486	0.986
R-SE1	DBP	Inverse variance weighted	4	0.099	0.112	0.374	0.787	0.750	No	4	0.099	0.112	0.374	0.787	0.751
R-SE10	DBP	Inverse variance weighted	10	0.038	0.036	0.293	0.097	0.683	No	10	0.038	0.036	0.293	0.097	0.684
R-SE3	DBP	Inverse variance weighted	6	0.111	0.091	0.224	0.310	0.605	No	6	0.111	0.091	0.224	0.310	0.606
R-SE4	DBP	Inverse variance weighted	4	-0.057	0.073	0.435	0.611	0.793	No	4	-0.057	0.073	0.435	0.611	0.794
R-SE6	DBP	Inverse variance weighted	3	-0.028	0.056	0.614	0.676	0.890	No	3	-0.028	0.056	0.614	0.676	0.890
R-SEH2A	DBP	Wald ratio	1	0.263	0.218	0.227	-	0.607	No	1	0.263	0.218	0.227	-	0.608
R-SET2	DBP	Inverse variance weighted	4	-0.087	0.054	0.108	0.867	0.432	No	4	-0.087	0.054	0.108	0.867	0.434
RNF149	DBP	Wald ratio	1	0.066	0.088	0.450	-	0.799	No	1	0.066	0.088	0.450	-	0.800
RNF43	DBP	Wald ratio	1	-0.127	0.210	0.544	-	0.850	No	1	-0.127	0.210	0.544	-	0.851
ROBO1	DBP	Wald ratio	1	0.155	0.121	0.203	-	0.579	No	1	0.155	0.121	0.203	-	0.580
ROBO4	DBP	Wald ratio	1	-0.169	0.248	0.495	-	0.825	No	1	-0.169	0.248	0.495	-	0.826
ROR1	DBP	Inverse variance weighted	6	-0.092	0.079	0.246	0.757	0.632	No	6	-0.092	0.079	0.246	0.757	0.633
RPA2	DBP	Wald ratio	1	-0.553	0.217	0.011	-	0.101	No	1	-0.553	0.217	0.011	-	0.102
RPE	DBP	Wald ratio	1	-0.314	0.439	0.474	-	0.811	No	1	-0.314	0.439	0.474	-	0.812
RPL14	DBP	Wald ratio	1	-0.019	0.401	0.963	-	0.993	No	1	-0.019	0.401	0.963	-	0.993
RRM2	DBP	Wald ratio	1	0.227	0.476	0.634	-	0.895	No	1	0.227	0.476	0.634	-	0.895
RRM2B	DBP	Inverse variance weighted	2	-0.162	0.438	0.712	-	0.931	No	2	-0.162	0.438	0.712	-	0.932
RSPO1	DBP	Inverse variance weighted	3	-0.062	0.113	0.585	0.251	0.873	No	3	-0.062	0.113	0.585	0.251	0.873
RSPO3	DBP	Wald ratio	1	-0.398	0.150	0.008	-	0.086	No	1	-0.398	0.150	0.008	-	0.087
RTBDN	DBP	Inverse variance weighted	2	-0.332	0.230	0.148	-	0.495	No	2	-0.332	0.230	0.148	-	0.496
RTN4IP1	DBP	Wald ratio	1	-0.036	0.240	0.881	-	0.980	No	1	-0.036	0.240	0.881	-	0.980
RTN4R	DBP	Inverse variance weighted	3	-0.042	0.101	0.678	0.123	0.918	No	3	-0.042	0.101	0.678	0.123	0.918
RWDD1	DBP	Wald ratio	1	-0.040	0.205	0.846	-	0.969	No	1	-0.040	0.205	0.846	-	0.969
S100A11	DBP	Wald ratio	1	-0.311	0.185	0.093	-	0.400	No	1	-0.311	0.185	0.093	-	0.401
S100A12	DBP	Inverse variance weighted	2	-0.036	0.102	0.722	-	0.931	No	2	-0.036	0.102	0.722	-	0.932
S100A13	DBP	Inverse variance weighted	5	0.145	0.097	0.136	0.643	0.489	No	5	0.145	0.097	0.136	0.643	0.490
S100A3	DBP	Wald ratio	1	0.156	0.506	0.757	-	0.938	No	1	0.156	0.506	0.757	-	0.938
S100A4	DBP	Inverse variance weighted	2	0.200	0.154	0.196	-	0.567	No	2	0.200	0.154	0.196	-	0.568
S100P	DBP	Inverse variance weighted	2	0.076	0.102	0.453	-	0.800	No	2	0.076	0.102	0.453	-	0.801
SAA4	DBP	Inverse variance weighted	10	0.061	0.068	0.366	0.214	0.743	No	10	0.061	0.068	0.366	0.214	0.744
SAMD9L	DBP	Inverse variance weighted	2	0.062	0.085	0.464	-	0.807	No	2	0.062	0.085	0.464	-	0.808
SARG	DBP	Wald ratio	1	-0.414	0.371	0.265	-	0.652	No	1	-0.414	0.371	0.265	-	0.653
SAT2	DBP	Inverse variance weighted	3	0.205	0.151	0.174	0.000	0.543	No	3	0.205	0.151	0.174	0.000	0.544
SBSN	DBP	Inverse variance weighted	4	-0.056	0.093	0.547	0.005	0.851	No	4	-0.056	0.093	0.547	0.005	0.852
SCAMP3	DBP	Wald ratio	1	0.268	0.328	0.415	-	0.778	No	1	0.268	0.328	0.415	-	0.779
SCARA5	DBP	Inverse variance weighted	4	-0.181	0.084	0.031	0.221	0.206	No	4	-0.181	0.084	0.031	0.221	0.207
SCARB2	DBP	Inverse variance weighted	4	-0.024	0.207	0.906	0.003	0.983	No	4	-0.024	0.207	0.906	0.003	0.983
SCARF1	DBP	Inverse variance weighted	4	0.126	0.061	0.038	0.347	0.231	No	4	0.126	0.061	0.038	0.347	0.232
SCARF2	DBP	Inverse variance weighted	2	0.088	0.245	0.719	-	0.931	No	2	0.088	0.245	0.719	-	0.932
SCG2	DBP	Wald ratio	1	-1.051	0.607	0.083	-	0.374	No	1	-1.051	0.607	0.083	-	0.376
SCG3	DBP	Inverse variance weighted	8	-0.086	0.037	0.020	0.648	0.149	No	8	-0.086	0.037	0.020	0.648	0.150
SCGB1A1	DBP	Inverse variance weighted	4	0.106	0.069	0.125	0.427	0.477	No	4	0.106	0.069	0.125	0.427	0.478
SCGB3A1	DBP	Inverse variance weighted	2	-0.323	0.365	0.375	-	0.751	No	2	-0.323	0.365	0.375	-	0.752
SCGB3A2	DBP	Inverse variance weighted	3	0.023	0.143	0.872	0.168	0.978	No	3	0.023	0.143	0.872	0.168	0.978
SCGN	DBP	Wald ratio	1	0.623	0.284	0.028	-	0.192	No	1	0.623	0.284	0.028	-	0.193
SCLY	DBP	Inverse variance weighted	2	0.051	0.125	0.685	-	0.920	No	2	0.051	0.125	0.685	-	0.920

ST3: MR causal estimates for plasma proteins on diastolic blood pressure.

Causal candidates prioritized for DBP were marked as "Yes" in column "Prioritized". Effect of plasma protein levels on blood pressure is in mmHg unit.

										Steiger filtering					
Exposure	Outocme	Method	nsnp	Beta	SE	P-value	Cochran's Q P-value	FDR-corrected P-value	Prioritized	nsnp	Beta	SE	P-value	Cochran's Q P-value	FDR-corrected P-value
SCN4B	DBP	Inverse variance weighted	8	-0.065	0.038	0.089	0.469	0.392	No	8	-0.065	0.038	0.089	0.469	0.393
SCP2	DBP	Wald ratio	1	-0.672	0.503	0.181	-	0.555	No	1	-0.672	0.503	0.181	-	0.556
SCPEP1	DBP	Inverse variance weighted	3	-0.099	0.073	0.179	0.427	0.550	No	3	-0.099	0.073	0.179	0.427	0.552
SCRG1	DBP	Inverse variance weighted	2	0.258	0.210	0.220	-	0.599	No	2	0.258	0.210	0.220	-	0.600
SCRN1	DBP	Inverse variance weighted	3	0.295	0.240	0.220	0.185	0.599	No	3	0.295	0.240	0.220	0.185	0.600
SCT	DBP	Wald ratio	1	-0.494	0.536	0.356	-	0.737	No	1	-0.494	0.536	0.356	-	0.737
SDC1	DBP	Inverse variance weighted	2	-0.716	0.159	0.000	-	0.000	Yes	2	-0.716	0.159	0.000	-	0.000
SDC4	DBP	Inverse variance weighted	2	-0.007	0.188	0.968	-	0.996	No	2	-0.007	0.188	0.968	-	0.996
SDCCAG8	DBP	Wald ratio	1	-1.158	0.114	0.000	-	0.000	Yes	1	-1.158	0.114	0.000	-	0.000
SDHB	DBP	Wald ratio	1	1.316	0.382	0.001	-	0.011	Yes	1	1.316	0.382	0.001	-	0.011
SDK2	DBP	Inverse variance weighted	4	0.124	0.047	0.008	0.463	0.087	No	4	0.124	0.047	0.008	0.463	0.088
SEC31A	DBP	Wald ratio	1	-0.081	0.226	0.722	-	0.931	No	1	-0.081	0.226	0.722	-	0.932
SEL1L	DBP	Wald ratio	1	0.025	0.080	0.752	-	0.938	No	1	0.025	0.080	0.752	-	0.938
SELE	DBP	Inverse variance weighted	2	0.514	0.225	0.023	-	0.164	No	2	0.514	0.225	0.023	-	0.165
SELENOP	DBP	Wald ratio	1	0.519	0.236	0.028	-	0.189	No	1	0.519	0.236	0.028	-	0.190
SELL	DBP	Inverse variance weighted	4	-0.078	0.060	0.192	0.307	0.566	No	4	-0.078	0.060	0.192	0.307	0.567
SELP	DBP	Inverse variance weighted	2	-0.114	0.085	0.178	-	0.550	No	2	-0.114	0.085	0.178	-	0.552
SELPLG	DBP	Inverse variance weighted	2	0.046	0.050	0.360	-	0.737	No	2	0.046	0.050	0.360	-	0.738
SEMA3F	DBP	Inverse variance weighted	2	-0.185	0.283	0.513	-	0.832	No	2	-0.185	0.283	0.513	-	0.832
SEMA4D	DBP	Inverse variance weighted	2	-0.406	0.512	0.427	-	0.789	No	2	-0.406	0.512	0.427	-	0.789
SEMA6C	DBP	Wald ratio	1	-0.683	0.269	0.011	-	0.103	No	1	-0.683	0.269	0.011	-	0.104
SEMA7A	DBP	Inverse variance weighted	2	-0.573	0.559	0.305	-	0.693	No	2	-0.573	0.559	0.305	-	0.694
SEPTIN3	DBP	Wald ratio	1	0.654	0.649	0.314	-	0.703	No	1	0.654	0.649	0.314	-	0.704
SEPTIN8	DBP	Inverse variance weighted	2	-0.472	0.230	0.040	-	0.239	No	2	-0.472	0.230	0.040	-	0.241
SEPTIN9	DBP	Wald ratio	1	0.042	0.250	0.866	-	0.976	No	1	0.042	0.250	0.866	-	0.976
SERPI-1	DBP	Inverse variance weighted	2	0.547	0.259	0.035	-	0.221	No	2	0.547	0.259	0.035	-	0.222
SERPI-11	DBP	Inverse variance weighted	9	-0.044	0.059	0.458	0.006	0.803	No	9	-0.044	0.059	0.458	0.006	0.803
SERPI-12	DBP	Inverse variance weighted	6	0.057	0.032	0.079	0.523	0.359	No	6	0.057	0.032	0.079	0.523	0.361
SERPI-3	DBP	Inverse variance weighted	3	0.017	0.064	0.793	0.826	0.947	No	3	0.017	0.064	0.793	0.826	0.947
SERPI-4	DBP	Inverse variance weighted	6	0.010	0.070	0.885	0.020	0.980	No	6	0.010	0.070	0.885	0.020	0.980
SERPI-5	DBP	Inverse variance weighted	4	0.071	0.300	0.814	0.000	0.957	No	4	0.071	0.300	0.814	0.000	0.957
SERPI-6	DBP	Inverse variance weighted	4	0.257	0.106	0.015	0.746	0.128	No	4	0.257	0.106	0.015	0.746	0.129
SERPI-9	DBP	Inverse variance weighted	6	0.096	0.039	0.014	0.445	0.123	No	6	0.096	0.039	0.014	0.445	0.124
SERPINB1	DBP	Inverse variance weighted	2	0.467	0.202	0.021	-	0.154	No	2	0.467	0.202	0.021	-	0.155
SERPINB5	DBP	Wald ratio	1	0.012	0.399	0.975	-	0.998	No	1	0.012	0.399	0.975	-	0.998
SERPINB6	DBP	Wald ratio	1	-0.008	0.145	0.957	-	0.992	No	1	-0.008	0.145	0.957	-	0.992
SERPINB8	DBP	Inverse variance weighted	5	-0.020	0.043	0.641	0.079	0.900	No	5	-0.020	0.043	0.641	0.079	0.900
SERPINB9	DBP	Inverse variance weighted	2	0.050	0.183	0.783	-	0.940	No	2	0.050	0.183	0.783	-	0.940
SERPINC1	DBP	Wald ratio	1	0.019	0.418	0.963	-	0.993	No	1	0.019	0.418	0.963	-	0.993
SERPIND1	DBP	Inverse variance weighted	2	0.225	0.750	0.764	-	0.938	No	2	0.225	0.750	0.764	-	0.939
SERPINE1	DBP	Inverse variance weighted	2	0.168	0.241	0.485	-	0.819	No	2	0.168	0.241	0.485	-	0.820
SERPINE2	DBP	Inverse variance weighted	6	-0.003	0.054	0.960	0.147	0.993	No	6	-0.003	0.054	0.960	0.147	0.993
SERPINF1	DBP	Inverse variance weighted	3	-0.242	0.084	0.004	0.353	0.052	No	3	-0.242	0.084	0.004	0.353	0.052
SERPINF2	DBP	Inverse variance weighted	2	-0.010	0.135	0.943	-	0.989	No	2	-0.010	0.135	0.943	-	0.989
SERPING1	DBP	Inverse variance weighted	4	0.037	0.106	0.726	0.262	0.931	No	4	0.037	0.106	0.726	0.262	0.932
SERPINH1	DBP	Inverse variance weighted	2	-0.168	0.384	0.661	-	0.909	No	2	-0.168	0.384	0.661	-	0.910
SERPINI1	DBP	Inverse variance weighted	8	0.285	0.056	0.000	0.686	0.000	Yes	8	0.285	0.056	0.000	0.686	0.000
SERPINI2	DBP	Inverse variance weighted	3	0.116	0.079	0.141	0.261	0.492	No	3	0.116	0.079	0.141	0.261	0.493
SESTD1	DBP	Wald ratio	1	-0.001	0.234	0.997	-	1.000	No	1	-0.001	0.234	0.997	-	1.000
SETMAR	DBP	Inverse variance weighted	4	0.067	0.157	0.672	0.052	0.914	No	4	0.067	0.157	0.672	0.052	0.914
SEZ6	DBP	Wald ratio	1	-0.182	0.399	0.648	-	0.902	No	1	-0.182	0.399	0.648	-	0.902
SEZ6L	DBP	Inverse variance weighted	4	0.048	0.127	0.707	0.317	0.931	No	4	0.048	0.127	0.707	0.317	0.932
SEZ6L2	DBP	Inverse variance weighted	3	0.262	0.357	0.463	0.002	0.807	No	3	0.262	0.357	0.463	0.002	0.808
SF3B4	DBP	Wald ratio	1	0.689	0.459	0.133	-	0.484	No	1	0.689	0.459	0.133	-	0.485
SFRP1	DBP	Inverse variance weighted	4	-0.091	0.088	0.305	0.935	0.693	No	4	-0.091	0.088	0.305	0.935	0.694
SFRP4	DBP	Inverse variance weighted	3	0.057	0.109	0.600	0.411	0.880	No	3	0.057	0.109	0.600	0.411	0.880

ST3: MR causal estimates for plasma proteins on diastolic blood pressure.

Causal candidates prioritized for DBP were marked as "Yes" in column "Prioritized". Effect of plasma protein levels on blood pressure is in mmHg unit.

										Steiger filtering					
Exposure	Outocme	Method	nsnp	Beta	SE	P-value	Cochran's Q P-value	FDR-corrected P-value	Prioritized	nsnp	Beta	SE	P-value	Cochran's Q P-value	FDR-corrected P-value
SFTPA1	DBP	Inverse variance weighted	2	-0.059	0.101	0.555	-	0.854	No	2	-0.059	0.101	0.555	-	0.855
SFTPA2	DBP	Inverse variance weighted	6	-0.022	0.039	0.570	0.283	0.865	No	6	-0.022	0.039	0.570	0.283	0.866
SFTPD	DBP	Inverse variance weighted	9	-0.015	0.045	0.743	0.015	0.938	No	9	-0.015	0.045	0.743	0.015	0.938
SGSH	DBP	Inverse variance weighted	9	0.019	0.027	0.488	0.409	0.822	No	9	0.019	0.027	0.488	0.409	0.823
SH2B3	DBP	Wald ratio	1	4.325	0.340	0.000	-	0.000	Yes	1	4.325	0.340	0.000	-	0.000
SH3BGRL2	DBP	Wald ratio	1	1.130	0.635	0.075	-	0.348	No	1	1.130	0.635	0.075	-	0.350
SH3BP1	DBP	Inverse variance weighted	3	0.142	0.096	0.140	0.538	0.492	No	3	0.142	0.096	0.140	0.538	0.493
SH3GLB2	DBP	Wald ratio	1	0.125	0.086	0.145	-	0.492	No	1	0.125	0.086	0.145	-	0.493
SHBG	DBP	Inverse variance weighted	3	0.237	0.297	0.425	0.000	0.787	No	3	0.237	0.297	0.425	0.000	0.787
SHH	DBP	Inverse variance weighted	2	0.247	0.986	0.802	-	0.952	No	2	0.247	0.986	0.802	-	0.952
SHISA5	DBP	Inverse variance weighted	3	0.128	0.287	0.657	0.046	0.906	No	3	0.128	0.287	0.657	0.046	0.906
SHMT1	DBP	Inverse variance weighted	6	0.120	0.032	0.000	0.440	0.005	Yes	6	0.120	0.032	0.000	0.440	0.005
SHPK	DBP	Wald ratio	1	0.083	0.316	0.792	-	0.946	No	1	0.083	0.316	0.792	-	0.947
SIAE	DBP	Inverse variance weighted	3	-0.043	0.139	0.757	0.197	0.938	No	3	-0.043	0.139	0.757	0.197	0.938
SIGLEC1	DBP	Inverse variance weighted	6	-0.028	0.053	0.593	0.885	0.876	No	6	-0.028	0.053	0.593	0.885	0.876
SIGLEC10	DBP	Inverse variance weighted	7	0.004	0.045	0.928	0.678	0.986	No	7	0.004	0.045	0.928	0.678	0.986
SIGLEC15	DBP	Wald ratio	1	0.391	0.620	0.529	-	0.843	No	1	0.391	0.620	0.529	-	0.844
SIGLEC5	DBP	Inverse variance weighted	13	-0.047	0.029	0.107	0.717	0.431	No	13	-0.047	0.029	0.107	0.717	0.432
SIGLEC6	DBP	Inverse variance weighted	11	0.056	0.049	0.249	0.273	0.635	No	11	0.056	0.049	0.249	0.273	0.636
SIGLEC7	DBP	Inverse variance weighted	5	-0.011	0.096	0.909	0.093	0.983	No	5	-0.011	0.096	0.909	0.093	0.983
SIGLEC8	DBP	Inverse variance weighted	8	-0.003	0.049	0.957	0.416	0.992	No	8	-0.003	0.049	0.957	0.416	0.992
SIGLEC9	DBP	Inverse variance weighted	6	0.002	0.040	0.952	0.675	0.992	No	6	0.002	0.040	0.952	0.675	0.992
SIL1	DBP	Wald ratio	1	0.979	0.402	0.015	-	0.125	No	1	0.979	0.402	0.015	-	0.126
SIRPA	DBP	Inverse variance weighted	10	-0.034	0.019	0.068	0.765	0.328	No	10	-0.034	0.019	0.068	0.765	0.329
SIRPB1	DBP	Inverse variance weighted	4	0.006	0.050	0.906	0.061	0.983	No	4	0.006	0.050	0.906	0.061	0.983
SIRT2	DBP	Wald ratio	1	-0.689	0.559	0.217	-	0.596	No	1	-0.689	0.559	0.217	-	0.597
SIRT5	DBP	Wald ratio	1	0.651	0.572	0.255	-	0.642	No	1	0.651	0.572	0.255	-	0.643
SKAP1	DBP	Wald ratio	1	-0.908	0.482	0.060	-	0.300	No	1	-0.908	0.482	0.060	-	0.301
SLA2	DBP	Wald ratio	1	0.090	0.399	0.822	-	0.961	No	1	0.090	0.399	0.822	-	0.961
SLAMF1	DBP	Inverse variance weighted	2	0.377	0.279	0.177	-	0.550	No	2	0.377	0.279	0.177	-	0.551
SLAMF6	DBP	Inverse variance weighted	2	0.246	0.140	0.080	-	0.361	No	2	0.246	0.140	0.080	-	0.363
SLAMF7	DBP	Inverse variance weighted	7	0.008	0.037	0.820	0.391	0.961	No	7	0.008	0.037	0.820	0.391	0.961
SLAMF8	DBP	Inverse variance weighted	6	-0.030	0.025	0.240	0.482	0.626	No	6	-0.030	0.025	0.240	0.482	0.627
SLC16A1	DBP	Wald ratio	1	-2.963	0.387	0.000	-	0.000	Yes	1	-2.963	0.387	0.000	-	0.000
SLC27A4	DBP	Wald ratio	1	1.010	0.392	0.010	-	0.098	No	1	1.010	0.392	0.010	-	0.098
SLC39A14	DBP	Wald ratio	1	0.521	0.254	0.040	-	0.239	No	1	0.521	0.254	0.040	-	0.241
SLC39A5	DBP	Inverse variance weighted	2	-0.310	0.397	0.434	-	0.793	No	2	-0.310	0.397	0.434	-	0.794
SLC4A1	DBP	Wald ratio	1	0.111	0.384	0.773	-	0.938	No	1	0.111	0.384	0.773	-	0.939
SLC9A3R1	DBP	Wald ratio	1	-0.477	0.249	0.056	-	0.289	No	1	-0.477	0.249	0.056	-	0.290
SLC9A3R2	DBP	Wald ratio	1	1.487	0.228	0.000	-	0.000	Yes	1	1.487	0.228	0.000	-	0.000
SLIT2	DBP	Inverse variance weighted	2	-0.286	0.351	0.416	-	0.778	No	2	-0.286	0.351	0.416	-	0.779
SLITRK1	DBP	Wald ratio	1	0.195	0.194	0.316	-	0.706	No	1	0.195	0.194	0.316	-	0.707
SLITRK6	DBP	Inverse variance weighted	7	-0.058	0.070	0.405	0.181	0.772	No	7	-0.058	0.070	0.405	0.181	0.773
SLMAP	DBP	Inverse variance weighted	2	-0.553	1.430	0.699	-	0.931	No	2	-0.553	1.430	0.699	-	0.932
SLURP1	DBP	Inverse variance weighted	2	-0.035	0.058	0.546	-	0.850	No	2	-0.035	0.058	0.546	-	0.851
SMAD1	DBP	Wald ratio	1	-1.340	0.532	0.012	-	0.107	No	1	-1.340	0.532	0.012	-	0.108
SMAD3	DBP	Wald ratio	1	-0.196	0.230	0.395	-	0.767	No	1	-0.196	0.230	0.395	-	0.768
SMAD5	DBP	Wald ratio	1	-0.127	0.588	0.829	-	0.963	No	1	-0.127	0.588	0.829	-	0.963
SMARCA2	DBP	Wald ratio	1	0.816	0.593	0.169	-	0.533	No	1	0.816	0.593	0.169	-	0.534
SMOC1	DBP	Inverse variance weighted	4	-0.282	0.197	0.152	0.000	0.500	No	4	-0.282	0.197	0.152	0.000	0.501
SMOC2	DBP	Inverse variance weighted	6	-0.062	0.060	0.307	0.994	0.696	No	6	-0.062	0.060	0.307	0.994	0.696
SMPD1	DBP	Inverse variance weighted	2	-0.050	0.057	0.383	-	0.754	No	2	-0.050	0.057	0.383	-	0.754
SMPD3	DBP	Wald ratio	1	-1.377	0.532	0.010	-	0.095	No	1	-1.377	0.532	0.010	-	0.096
SMPDL3A	DBP	Inverse variance weighted	9	0.085	0.056	0.130	0.001	0.480	No	9	0.085	0.056	0.130	0.001	0.481
SMTN	DBP	Wald ratio	1	-1.884	0.583	0.001	-	0.020	Yes	1	-1.884	0.583	0.001	-	0.021
S-P25	DBP	Inverse variance weighted	2	0.249	0.190	0.190	-	0.563	No	2	0.249	0.190	0.190	-	0.564

ST3: MR causal estimates for plasma proteins on diastolic blood pressure.

Causal candidates prioritized for DBP were marked as "Yes" in column "Prioritized". Effect of plasma protein levels on blood pressure is in mmHg unit.

										Steiger filtering					
**Exposure**	**Outcome**	**Method**	**nsnp**	**Beta**	**SE**	**P-value**	**Cochran's Q P-value**	**FDR-corrected P-value**	**Prioritized**	**nsnp**	**Beta**	**SE**	**P-value**	**Cochran's Q P-value**	**FDR-corrected P-value**
S-P29	DBP	Wald ratio	1	-0.279	0.330	0.399	-	0.769	No	1	-0.279	0.330	0.399	-	0.769
SNCA	DBP	Wald ratio	1	0.590	0.511	0.248	-	0.634	No	1	0.590	0.511	0.248	-	0.635
SNCG	DBP	Inverse variance weighted	8	0.012	0.025	0.640	0.882	0.900	No	8	0.012	0.025	0.640	0.882	0.900
SNED1	DBP	Inverse variance weighted	3	-0.233	0.237	0.324	0.087	0.715	No	3	-0.233	0.237	0.324	0.087	0.715
SNX15	DBP	Inverse variance weighted	2	-0.018	0.086	0.834	-	0.963	No	2	-0.018	0.086	0.834	-	0.964
SNX18	DBP	Wald ratio	1	-0.108	0.332	0.744	-	0.938	No	1	-0.108	0.332	0.744	-	0.938
SNX9	DBP	Inverse variance weighted	3	-0.361	0.388	0.352	0.065	0.732	No	3	-0.361	0.388	0.352	0.065	0.733
SOD1	DBP	Wald ratio	1	0.453	0.579	0.434	-	0.793	No	1	0.453	0.579	0.434	-	0.794
SOD2	DBP	Inverse variance weighted	3	0.051	0.174	0.768	0.223	0.938	No	3	0.051	0.174	0.768	0.223	0.939
SOD3	DBP	Inverse variance weighted	7	0.093	0.045	0.041	0.610	0.240	No	7	0.093	0.045	0.041	0.610	0.242
SORBS1	DBP	Inverse variance weighted	2	0.334	0.930	0.720	-	0.931	No	2	0.334	0.930	0.720	-	0.932
SORCS2	DBP	Inverse variance weighted	8	-0.133	0.060	0.028	0.201	0.189	No	8	-0.133	0.060	0.028	0.201	0.190
SORD	DBP	Inverse variance weighted	2	-0.327	0.152	0.031	-	0.206	No	2	-0.327	0.152	0.031	-	0.207
SORT1	DBP	Inverse variance weighted	2	-0.153	0.282	0.587	-	0.875	No	2	-0.153	0.282	0.587	-	0.875
SOST	DBP	Wald ratio	1	-0.412	0.434	0.343	-	0.724	No	1	-0.412	0.434	0.343	-	0.725
SPAG1	DBP	Inverse variance weighted	7	-0.048	0.032	0.131	0.631	0.481	No	7	-0.048	0.032	0.131	0.631	0.482
SPARC	DBP	Inverse variance weighted	2	-0.310	0.347	0.372	-	0.749	No	2	-0.310	0.347	0.372	-	0.750
SPARCL1	DBP	Inverse variance weighted	5	-0.098	0.034	0.004	0.651	0.050	No	5	-0.098	0.034	0.004	0.651	0.051
SPESP1	DBP	Inverse variance weighted	2	-0.136	0.184	0.460	-	0.805	No	2	-0.136	0.184	0.460	-	0.805
SPINK1	DBP	Inverse variance weighted	5	0.066	0.069	0.340	0.205	0.724	No	5	0.066	0.069	0.340	0.205	0.724
SPINK2	DBP	Inverse variance weighted	11	-0.025	0.051	0.624	0.415	0.891	No	11	-0.025	0.051	0.624	0.415	0.892
SPINK4	DBP	Inverse variance weighted	9	-0.005	0.049	0.922	0.003	0.986	No	9	-0.005	0.049	0.922	0.003	0.986
SPINK5	DBP	Inverse variance weighted	5	-0.020	0.090	0.821	0.025	0.961	No	5	-0.020	0.090	0.821	0.025	0.961
SPINK6	DBP	Inverse variance weighted	3	-0.143	0.099	0.149	0.092	0.496	No	3	-0.143	0.099	0.149	0.092	0.497
SPINK8	DBP	Wald ratio	1	0.482	0.053	0.000	-	0.000	Yes	1	0.482	0.053	0.000	-	0.000
SPINT1	DBP	Inverse variance weighted	5	-0.006	0.133	0.964	0.009	0.993	No	5	-0.006	0.133	0.964	0.009	0.993
SPINT2	DBP	Inverse variance weighted	2	0.154	0.146	0.289	-	0.679	No	2	0.154	0.146	0.289	-	0.680
SPINT3	DBP	Inverse variance weighted	2	-0.565	0.228	0.013	-	0.117	No	2	-0.565	0.228	0.013	-	0.117
SPOCK1	DBP	Inverse variance weighted	3	-0.060	0.208	0.773	0.191	0.938	No	3	-0.060	0.208	0.773	0.191	0.939
SPON1	DBP	Inverse variance weighted	4	0.309	0.118	0.009	0.054	0.092	No	4	0.309	0.118	0.009	0.054	0.093
SPON2	DBP	Inverse variance weighted	4	0.052	0.111	0.641	0.726	0.900	No	4	0.052	0.111	0.641	0.726	0.900
SPP1	DBP	Inverse variance weighted	4	-0.032	0.176	0.855	0.969	0.973	No	4	-0.032	0.176	0.855	0.969	0.973
SPRED2	DBP	Wald ratio	1	0.941	0.365	0.010	-	0.098	No	1	0.941	0.365	0.010	-	0.098
SPRING1	DBP	Wald ratio	1	-0.016	0.620	0.980	-	0.999	No	1	-0.016	0.620	0.980	-	0.999
SPRR3	DBP	Inverse variance weighted	2	0.065	0.097	0.506	-	0.829	No	2	0.065	0.097	0.506	-	0.830
SPRY2	DBP	Inverse variance weighted	2	-0.188	0.225	0.403	-	0.770	No	2	-0.188	0.225	0.403	-	0.771
SPTLC1	DBP	Inverse variance weighted	2	0.014	0.130	0.916	-	0.983	No	2	0.014	0.130	0.916	-	0.983
SRP14	DBP	Wald ratio	1	-0.731	0.476	0.125	-	0.477	No	1	-0.731	0.476	0.125	-	0.478
SSC4D	DBP	Inverse variance weighted	5	0.040	0.066	0.540	0.383	0.848	No	5	0.040	0.066	0.540	0.383	0.848
SSC5D	DBP	Inverse variance weighted	4	0.049	0.049	0.320	0.448	0.710	No	4	0.049	0.049	0.320	0.448	0.711
SSH3	DBP	Wald ratio	1	0.973	0.679	0.152	-	0.499	No	1	0.973	0.679	0.152	-	0.500
ST3GAL1	DBP	Inverse variance weighted	2	0.047	0.095	0.619	-	0.891	No	2	0.047	0.095	0.619	-	0.891
ST6GAL1	DBP	Inverse variance weighted	3	-0.175	0.177	0.322	0.131	0.713	No	3	-0.175	0.177	0.322	0.131	0.714
ST8SIA1	DBP	Wald ratio	1	-0.196	0.548	0.720	-	0.931	No	1	-0.196	0.548	0.720	-	0.932
STAB2	DBP	Inverse variance weighted	11	0.149	0.062	0.017	0.133	0.137	No	11	0.149	0.062	0.017	0.133	0.138
STAMBP	DBP	Wald ratio	1	-0.101	0.466	0.829	-	0.963	No	1	-0.101	0.466	0.829	-	0.963
STAT2	DBP	Inverse variance weighted	2	0.207	0.141	0.142	-	0.492	No	2	0.207	0.141	0.142	-	0.493
STC1	DBP	Wald ratio	1	-1.553	0.427	0.000	-	0.006	Yes	1	-1.553	0.427	0.000	-	0.006
STC2	DBP	Inverse variance weighted	2	0.242	0.292	0.408	-	0.774	No	2	0.242	0.292	0.408	-	0.775
STK4	DBP	Wald ratio	1	-0.182	0.421	0.665	-	0.911	No	1	-0.182	0.421	0.665	-	0.911
STX16	DBP	Inverse variance weighted	2	0.478	0.325	0.142	-	0.492	No	2	0.478	0.325	0.142	-	0.493
STX4	DBP	Wald ratio	1	3.547	0.570	0.000	-	0.000	Yes	1	3.547	0.570	0.000	-	0.000
STX7	DBP	Inverse variance weighted	6	-0.035	0.056	0.532	0.066	0.844	No	6	-0.035	0.056	0.532	0.066	0.844
STX8	DBP	Wald ratio	1	-0.236	0.268	0.379	-	0.751	No	1	-0.236	0.268	0.379	-	0.752
STXBP1	DBP	Wald ratio	1	0.563	0.425	0.185	-	0.559	No	1	0.563	0.425	0.185	-	0.560
SUGP1	DBP	Wald ratio	1	-0.097	0.096	0.313	-	0.703	No	1	-0.097	0.096	0.313	-	0.704

ST3: MR causal estimates for plasma proteins on diastolic blood pressure.

Causal candidates prioritized for DBP were marked as "Yes" in column "Prioritized". Effect of plasma protein levels on blood pressure is in mmHg unit.

										Steiger filtering					
Exposure	Outocme	Method	nsnp	Beta	SE	P-value	Cochran's Q P-value	FDR-corrected P-value	Prioritized	nsnp	Beta	SE	P-value	Cochran's Q P-value	FDR-corrected P-value
SULT1A1	DBP	Inverse variance weighted	4	-0.574	0.452	0.204	0.000	0.582	No	4	-0.574	0.452	0.204	0.000	0.583
SULT2A1	DBP	Inverse variance weighted	2	0.165	0.082	0.045	-	0.255	No	2	0.165	0.082	0.045	-	0.256
SUMF2	DBP	Inverse variance weighted	7	0.019	0.061	0.760	0.206	0.938	No	7	0.019	0.061	0.760	0.206	0.938
SUSD1	DBP	Inverse variance weighted	2	-0.054	0.161	0.737	-	0.938	No	2	-0.054	0.161	0.737	-	0.938
SUSD2	DBP	Inverse variance weighted	9	-0.049	0.049	0.314	0.280	0.703	No	9	-0.049	0.049	0.314	0.280	0.704
SUSD4	DBP	Inverse variance weighted	2	0.058	0.061	0.348	-	0.729	No	2	0.058	0.061	0.348	-	0.730
SUSD5	DBP	Inverse variance weighted	7	-0.002	0.073	0.973	0.055	0.998	No	7	-0.002	0.073	0.973	0.055	0.998
SV2A	DBP	Wald ratio	1	0.206	0.145	0.157	-	0.512	No	1	0.206	0.145	0.157	-	0.513
SWAP70	DBP	Inverse variance weighted	2	-0.643	1.063	0.546	-	0.850	No	2	-0.643	1.063	0.546	-	0.851
TAB2	DBP	Wald ratio	1	0.556	0.595	0.350	-	0.731	No	1	0.556	0.595	0.350	-	0.732
TACC3	DBP	Inverse variance weighted	2	-0.292	0.120	0.015	-	0.125	No	2	-0.292	0.120	0.015	-	0.126
TACSTD2	DBP	Inverse variance weighted	6	-0.024	0.033	0.473	0.608	0.811	No	6	-0.024	0.033	0.473	0.608	0.812
TAFA5	DBP	Inverse variance weighted	8	0.039	0.143	0.785	0.019	0.942	No	8	0.039	0.143	0.785	0.019	0.942
TALDO1	DBP	Inverse variance weighted	4	0.412	0.393	0.293	0.099	0.683	No	4	0.412	0.393	0.293	0.099	0.684
TARBP2	DBP	Wald ratio	1	-1.371	0.511	0.007	-	0.083	No	1	-1.371	0.511	0.007	-	0.084
TBC1D17	DBP	Inverse variance weighted	2	-0.219	0.059	0.000	-	0.005	Yes	2	-0.219	0.059	0.000	-	0.005
TBC1D23	DBP	Wald ratio	1	-0.101	0.152	0.505	-	0.829	No	1	-0.101	0.152	0.505	-	0.830
TBC1D5	DBP	Wald ratio	1	0.436	0.785	0.579	-	0.870	No	1	0.436	0.785	0.579	-	0.870
TBCA	DBP	Wald ratio	1	0.093	0.177	0.601	-	0.881	No	1	0.093	0.177	0.601	-	0.881
TBCB	DBP	Wald ratio	1	0.124	0.444	0.780	-	0.939	No	1	0.124	0.444	0.780	-	0.939
TBCC	DBP	Wald ratio	1	0.165	0.169	0.328	-	0.717	No	1	0.165	0.169	0.328	-	0.718
TCL1A	DBP	Inverse variance weighted	3	0.108	0.095	0.255	0.253	0.642	No	3	0.108	0.095	0.255	0.253	0.643
TCN1	DBP	Inverse variance weighted	11	0.029	0.057	0.611	0.087	0.887	No	11	0.029	0.057	0.611	0.087	0.887
TCN2	DBP	Inverse variance weighted	11	-0.039	0.070	0.582	0.003	0.870	No	11	-0.039	0.070	0.582	0.003	0.870
TCOF1	DBP	Wald ratio	1	1.540	0.661	0.020	-	0.149	No	1	1.540	0.661	0.020	-	0.150
TCTN3	DBP	Inverse variance weighted	11	0.016	0.040	0.695	0.200	0.929	No	11	0.016	0.040	0.695	0.200	0.929
TDGF1	DBP	Inverse variance weighted	7	0.011	0.044	0.804	0.000	0.952	No	7	0.011	0.044	0.804	0.000	0.953
TDP1	DBP	Inverse variance weighted	3	0.022	0.305	0.943	0.252	0.989	No	3	0.022	0.305	0.943	0.252	0.989
TDRKH	DBP	Wald ratio	1	0.024	0.073	0.737	-	0.938	No	1	0.024	0.073	0.737	-	0.938
TEF	DBP	Wald ratio	1	0.447	0.366	0.221	-	0.600	No	1	0.447	0.366	0.221	-	0.601
TEK	DBP	Inverse variance weighted	13	0.121	0.055	0.029	0.076	0.192	No	13	0.121	0.055	0.029	0.076	0.194
TEX101	DBP	Wald ratio	1	-0.033	0.100	0.739	-	0.938	No	1	-0.033	0.100	0.739	-	0.938
TF	DBP	Inverse variance weighted	2	0.066	0.075	0.374	-	0.750	No	2	0.066	0.075	0.374	-	0.751
TFF1	DBP	Inverse variance weighted	2	0.162	0.094	0.084	-	0.376	No	2	0.162	0.094	0.084	-	0.377
TFF2	DBP	Inverse variance weighted	2	-0.223	0.385	0.563	-	0.859	No	2	-0.223	0.385	0.563	-	0.859
TFF3	DBP	Inverse variance weighted	4	0.097	0.174	0.577	0.171	0.869	No	4	0.097	0.174	0.577	0.171	0.870
TFPI	DBP	Inverse variance weighted	3	-0.140	0.153	0.359	0.008	0.737	No	3	-0.140	0.153	0.359	0.008	0.738
TFPI2	DBP	Inverse variance weighted	6	0.105	0.234	0.654	0.002	0.905	No	6	0.105	0.234	0.654	0.002	0.905
TFRC	DBP	Inverse variance weighted	3	0.032	0.045	0.475	0.775	0.811	No	3	0.032	0.045	0.475	0.775	0.812
TG	DBP	Inverse variance weighted	2	-0.224	0.274	0.414	-	0.777	No	2	-0.224	0.274	0.414	-	0.778
TGFA	DBP	Inverse variance weighted	2	-0.020	0.189	0.915	-	0.983	No	2	-0.020	0.189	0.915	-	0.983
TGFB1	DBP	Inverse variance weighted	2	0.499	0.278	0.072	-	0.340	No	2	0.499	0.278	0.072	-	0.341
TGFB2	DBP	Wald ratio	1	-0.276	0.174	0.112	-	0.447	No	1	-0.276	0.174	0.112	-	0.448
TGFBI	DBP	Inverse variance weighted	7	0.045	0.045	0.316	0.287	0.706	No	7	0.045	0.045	0.316	0.287	0.707
TGFBR1	DBP	Wald ratio	1	0.325	0.524	0.535	-	0.845	No	1	0.325	0.524	0.535	-	0.845
TGFBR2	DBP	Inverse variance weighted	4	0.370	0.867	0.670	0.000	0.914	No	4	0.370	0.867	0.670	0.000	0.914
TGFBR3	DBP	Inverse variance weighted	3	0.144	0.354	0.684	0.003	0.920	No	3	0.144	0.354	0.684	0.003	0.920
TGM2	DBP	Inverse variance weighted	2	-0.361	0.135	0.007	-	0.083	No	2	-0.361	0.135	0.007	-	0.084
TGOLN2	DBP	Inverse variance weighted	8	0.003	0.032	0.919	0.851	0.985	No	8	0.003	0.032	0.919	0.851	0.985
THBD	DBP	Inverse variance weighted	3	-0.217	0.129	0.092	0.380	0.399	No	3	-0.217	0.129	0.092	0.380	0.400
THBS2	DBP	Inverse variance weighted	4	-0.205	0.096	0.033	0.038	0.211	No	4	-0.205	0.096	0.033	0.038	0.212
THBS4	DBP	Inverse variance weighted	3	0.064	0.091	0.481	0.414	0.814	No	3	0.064	0.091	0.481	0.414	0.815
THOP1	DBP	Inverse variance weighted	2	-0.323	0.130	0.013	-	0.113	No	2	-0.323	0.130	0.013	-	0.114
THPO	DBP	Inverse variance weighted	2	-0.174	0.327	0.594	-	0.876	No	2	-0.174	0.327	0.594	-	0.876
THSD1	DBP	Inverse variance weighted	2	-0.320	0.232	0.168	-	0.533	No	2	-0.320	0.232	0.168	-	0.534
THTPA	DBP	Wald ratio	1	0.135	0.053	0.012	-	0.106	No	1	0.135	0.053	0.012	-	0.107

ST3: MR causal estimates for plasma proteins on diastolic blood pressure.

Causal candidates prioritized for DBP were marked as "Yes" in column "Prioritized". Effect of plasma protein levels on blood pressure is in mmHg unit.

										Steiger filtering					
**Exposure**	**Outocme**	**Method**	**nsnp**	**Beta**	**SE**	**P-value**	**Cochran's Q P-value**	**FDR-corrected P-value**	**Prioritized**	**nsnp**	**Beta**	**SE**	**P-value**	**Cochran's Q P-value**	**FDR-corrected P-value**
THY1	DBP	Inverse variance weighted	7	0.048	0.063	0.446	0.757	0.797	No	7	0.048	0.063	0.446	0.757	0.798
TIE1	DBP	Wald ratio	1	-0.942	0.120	0.000	-	0.000	Yes	1	-0.942	0.120	0.000	-	0.000
TIGAR	DBP	Wald ratio	1	-0.036	0.434	0.934	-	0.986	No	1	-0.036	0.434	0.934	-	0.986
TIGIT	DBP	Wald ratio	1	-0.093	0.269	0.729	-	0.933	No	1	-0.093	0.269	0.729	-	0.934
TIMD4	DBP	Inverse variance weighted	3	0.075	0.098	0.445	0.358	0.797	No	3	0.075	0.098	0.445	0.358	0.798
TIMM10	DBP	Wald ratio	1	-0.065	0.128	0.610	-	0.886	No	1	-0.065	0.128	0.610	-	0.887
TIMP2	DBP	Inverse variance weighted	2	-0.029	0.478	0.951	-	0.992	No	2	-0.029	0.478	0.951	-	0.992
TIMP3	DBP	Inverse variance weighted	6	0.035	0.038	0.360	0.640	0.737	No	6	0.035	0.038	0.360	0.640	0.738
TIMP4	DBP	Inverse variance weighted	4	-0.105	0.101	0.301	0.071	0.692	No	4	-0.105	0.101	0.301	0.071	0.693
TI-GL1	DBP	Wald ratio	1	-0.895	0.327	0.006	-	0.076	No	1	-0.895	0.327	0.006	-	0.077
TJAP1	DBP	Wald ratio	1	-3.473	0.582	0.000	-	0.000	Yes	1	-3.473	0.582	0.000	-	0.000
TK1	DBP	Wald ratio	1	0.227	0.317	0.475	-	0.811	No	1	0.227	0.317	0.475	-	0.812
TLR1	DBP	Inverse variance weighted	2	-0.086	0.058	0.134	-	0.485	No	2	-0.086	0.058	0.134	-	0.486
TLR3	DBP	Inverse variance weighted	11	-0.003	0.030	0.926	0.050	0.986	No	11	-0.003	0.030	0.926	0.050	0.986
TLR4	DBP	Inverse variance weighted	2	-0.029	0.238	0.903	-	0.983	No	2	-0.029	0.238	0.903	-	0.983
TMED8	DBP	Wald ratio	1	-0.113	0.339	0.738	-	0.938	No	1	-0.113	0.339	0.738	-	0.938
TMEM106A	DBP	Wald ratio	1	0.696	0.234	0.003	-	0.043	Yes	1	0.696	0.234	0.003	-	0.043
TMEM132A	DBP	Inverse variance weighted	5	-0.002	0.038	0.963	0.736	0.993	No	5	-0.002	0.038	0.963	0.736	0.993
TMEM25	DBP	Inverse variance weighted	7	-0.017	0.059	0.775	0.891	0.938	No	7	-0.017	0.059	0.775	0.891	0.939
TMPRSS11D	DBP	Inverse variance weighted	3	-0.048	0.282	0.865	0.054	0.976	No	3	-0.048	0.282	0.865	0.054	0.976
TMPRSS15	DBP	Inverse variance weighted	2	0.119	0.195	0.542	-	0.850	No	2	0.119	0.195	0.542	-	0.851
TMPRSS5	DBP	Inverse variance weighted	9	0.088	0.032	0.006	0.337	0.072	No	9	0.088	0.032	0.006	0.337	0.073
TMSB10	DBP	Wald ratio	1	-0.581	0.644	0.367	-	0.743	No	1	-0.581	0.644	0.367	-	0.744
TNC	DBP	Inverse variance weighted	5	0.208	0.124	0.093	0.078	0.400	No	5	0.208	0.124	0.093	0.078	0.401
TNFAIP2	DBP	Inverse variance weighted	2	-0.452	0.287	0.115	-	0.456	No	2	-0.452	0.287	0.115	-	0.457
TNFAIP8	DBP	Wald ratio	1	0.566	0.308	0.067	-	0.323	No	1	0.566	0.308	0.067	-	0.325
TNFAIP8L2	DBP	Wald ratio	1	0.165	0.442	0.709	-	0.931	No	1	0.165	0.442	0.709	-	0.932
TNFRSF10A	DBP	Inverse variance weighted	6	0.043	0.072	0.553	0.033	0.854	No	6	0.043	0.072	0.553	0.033	0.855
TNFRSF10B	DBP	Inverse variance weighted	5	-0.023	0.067	0.730	0.978	0.933	No	5	-0.023	0.067	0.730	0.978	0.934
TNFRSF10C	DBP	Inverse variance weighted	7	-0.152	0.102	0.138	0.166	0.491	No	7	-0.152	0.102	0.138	0.166	0.492
TNFRSF11A	DBP	Inverse variance weighted	4	-0.014	0.052	0.788	0.272	0.943	No	4	-0.014	0.052	0.788	0.272	0.944
TNFRSF11B	DBP	Inverse variance weighted	5	-0.365	0.137	0.008	0.181	0.084	No	5	-0.365	0.137	0.008	0.181	0.084
TNFRSF12A	DBP	Inverse variance weighted	2	-0.042	0.321	0.895	-	0.982	No	2	-0.042	0.321	0.895	-	0.982
TNFRSF13B	DBP	Inverse variance weighted	3	0.106	0.191	0.580	0.439	0.870	No	3	0.106	0.191	0.580	0.439	0.870
TNFRSF13C	DBP	Inverse variance weighted	5	0.003	0.048	0.958	0.419	0.992	No	5	0.003	0.048	0.958	0.419	0.992
TNFRSF14	DBP	Wald ratio	1	0.402	0.201	0.046	-	0.255	No	1	0.402	0.201	0.046	-	0.256
TNFRSF17	DBP	Wald ratio	1	-0.881	0.176	0.000	-	0.000	Yes	1	-0.881	0.176	0.000	-	0.000
TNFRSF19	DBP	Inverse variance weighted	4	0.048	0.149	0.747	0.073	0.938	No	4	0.048	0.149	0.747	0.073	0.938
TNFRSF1A	DBP	Wald ratio	1	-0.086	0.306	0.779	-	0.939	No	1	-0.086	0.306	0.779	-	0.939
TNFRSF1B	DBP	Wald ratio	1	-0.030	0.153	0.846	-	0.969	No	1	-0.030	0.153	0.846	-	0.969
TNFRSF21	DBP	Inverse variance weighted	2	-0.169	0.405	0.676	-	0.917	No	2	-0.169	0.405	0.676	-	0.917
TNFRSF4	DBP	Inverse variance weighted	3	-0.170	0.479	0.723	0.000	0.931	No	3	-0.170	0.479	0.723	0.000	0.932
TNFRSF6B	DBP	Inverse variance weighted	5	-0.258	0.153	0.093	0.001	0.400	No	5	-0.258	0.153	0.093	0.001	0.401
TNFRSF8	DBP	Inverse variance weighted	3	0.294	0.286	0.304	0.000	0.693	No	3	0.294	0.286	0.304	0.000	0.694
TNFRSF9	DBP	Wald ratio	1	0.091	0.159	0.568	-	0.863	No	1	0.091	0.159	0.568	-	0.863
TNFSF10	DBP	Inverse variance weighted	3	0.334	0.122	0.006	0.506	0.076	No	3	0.334	0.122	0.006	0.506	0.077
TNFSF11	DBP	Wald ratio	1	0.046	0.201	0.819	-	0.960	No	1	0.046	0.201	0.819	-	0.960
TNFSF12	DBP	Inverse variance weighted	2	0.440	0.067	0.000	-	0.000	Yes	2	0.440	0.067	0.000	-	0.000
TNFSF13	DBP	Inverse variance weighted	4	-0.223	0.235	0.341	0.000	0.724	No	4	-0.223	0.235	0.341	0.000	0.724
TNFSF13B	DBP	Inverse variance weighted	6	-0.162	0.094	0.084	0.995	0.376	No	6	-0.162	0.094	0.084	0.995	0.377
TNFSF14	DBP	Inverse variance weighted	6	-0.042	0.092	0.649	0.236	0.902	No	6	-0.042	0.092	0.649	0.236	0.902
TNFSF8	DBP	Inverse variance weighted	2	0.043	0.102	0.670	-	0.914	No	2	0.043	0.102	0.670	-	0.914
TNIP1	DBP	Wald ratio	1	-1.300	0.551	0.018	-	0.140	No	1	-1.300	0.551	0.018	-	0.141
TNN	DBP	Inverse variance weighted	6	0.014	0.041	0.736	0.026	0.938	No	6	0.014	0.041	0.736	0.026	0.938
TNR	DBP	Inverse variance weighted	4	-0.139	0.141	0.326	0.001	0.717	No	4	-0.139	0.141	0.326	0.001	0.718
TOP1	DBP	Wald ratio	1	0.250	0.789	0.751	-	0.938	No	1	0.250	0.789	0.751	-	0.938

ST3: MR causal estimates for plasma proteins on diastolic blood pressure.

Causal candidates prioritized for DBP were marked as "Yes" in column "Prioritized". Effect of plasma protein levels on blood pressure is in mmHg unit.

										Steiger filtering					
Exposure	Outcome	Method	nsnp	Beta	SE	P-value	Cochran's Q P-value	FDR-corrected P-value	Prioritized	nsnp	Beta	SE	P-value	Cochran's Q P-value	FDR-corrected P-value
TOP2B	DBP	Wald ratio	1	0.505	0.304	0.097	-	0.406	No	1	0.505	0.304	0.097	-	0.408
TOR1AIP1	DBP	Inverse variance weighted	4	0.051	0.036	0.163	0.289	0.525	No	4	0.051	0.036	0.163	0.289	0.526
TP53	DBP	Wald ratio	1	-1.317	0.415	0.002	-	0.024	Yes	1	-1.317	0.415	0.002	-	0.025
TP53I3	DBP	Inverse variance weighted	2	-0.007	0.078	0.923	-	0.986	No	2	-0.007	0.078	0.923	-	0.986
TPK1	DBP	Inverse variance weighted	7	-0.107	0.071	0.132	0.221	0.484	No	7	-0.107	0.071	0.132	0.221	0.485
TPMT	DBP	Inverse variance weighted	3	0.002	0.086	0.981	0.038	1.000	No	3	0.002	0.086	0.981	0.038	1.000
TPP1	DBP	Inverse variance weighted	3	0.070	0.218	0.748	0.075	0.938	No	3	0.070	0.218	0.748	0.075	0.938
TPPP3	DBP	Wald ratio	1	-0.608	0.360	0.091	-	0.399	No	1	-0.608	0.360	0.091	-	0.400
TPR	DBP	Wald ratio	1	1.004	0.504	0.046	-	0.257	No	1	1.004	0.504	0.046	-	0.258
TPSAB1	DBP	Inverse variance weighted	12	-0.047	0.055	0.395	0.208	0.767	No	12	-0.047	0.055	0.395	0.208	0.768
TPSD1	DBP	Inverse variance weighted	4	-0.054	0.102	0.593	0.136	0.876	No	4	-0.054	0.102	0.593	0.136	0.876
TREH	DBP	Inverse variance weighted	10	-0.012	0.037	0.741	0.019	0.938	No	10	-0.012	0.037	0.741	0.019	0.938
TREM2	DBP	Inverse variance weighted	2	-0.027	0.215	0.899	-	0.983	No	2	-0.027	0.215	0.899	-	0.983
TREML2	DBP	Inverse variance weighted	4	-0.034	0.122	0.780	0.081	0.939	No	4	-0.034	0.122	0.780	0.081	0.940
TRIM21	DBP	Inverse variance weighted	2	-0.024	0.673	0.972	-	0.998	No	2	-0.024	0.673	0.972	-	0.998
TRIM24	DBP	Wald ratio	1	0.525	0.598	0.380	-	0.753	No	1	0.525	0.598	0.380	-	0.753
TRIM25	DBP	Wald ratio	1	0.178	0.266	0.504	-	0.829	No	1	0.178	0.266	0.504	-	0.830
TRIM5	DBP	Inverse variance weighted	3	0.165	0.073	0.024	0.475	0.170	No	3	0.165	0.073	0.024	0.475	0.171
TRIM58	DBP	Inverse variance weighted	2	0.007	0.154	0.962	-	0.993	No	2	0.007	0.154	0.962	-	0.993
TSC22D1	DBP	Wald ratio	1	0.629	0.415	0.130	-	0.480	No	1	0.629	0.415	0.130	-	0.481
TSPAN1	DBP	Wald ratio	1	0.545	0.521	0.296	-	0.686	No	1	0.545	0.521	0.296	-	0.687
TSPAN15	DBP	Wald ratio	1	-0.570	0.385	0.139	-	0.492	No	1	-0.570	0.385	0.139	-	0.493
TSPAN8	DBP	Inverse variance weighted	3	-0.089	0.048	0.063	0.521	0.312	No	3	-0.089	0.048	0.063	0.521	0.313
TSPYL1	DBP	Inverse variance weighted	2	-0.029	0.201	0.886	-	0.980	No	2	-0.029	0.201	0.886	-	0.980
TST	DBP	Inverse variance weighted	2	0.002	0.185	0.992	-	1.000	No	2	0.002	0.185	0.992	-	1.000
TTF2	DBP	Inverse variance weighted	2	0.022	0.068	0.751	-	0.938	No	2	0.022	0.068	0.751	-	0.938
TTN	DBP	Wald ratio	1	-0.236	0.573	0.681	-	0.918	No	1	-0.236	0.573	0.681	-	0.918
TTR	DBP	Wald ratio	1	-0.113	0.191	0.554	-	0.854	No	1	-0.113	0.191	0.554	-	0.855
TWF2	DBP	Wald ratio	1	-0.536	0.358	0.134	-	0.485	No	1	-0.536	0.358	0.134	-	0.486
TXN	DBP	Inverse variance weighted	2	-0.252	0.365	0.490	-	0.823	No	2	-0.252	0.365	0.490	-	0.823
TXNDC15	DBP	Inverse variance weighted	10	-0.010	0.033	0.753	0.202	0.938	No	10	-0.010	0.033	0.753	0.202	0.938
TXNDC5	DBP	Inverse variance weighted	2	0.460	0.228	0.044	-	0.255	No	2	0.460	0.228	0.044	-	0.256
TXNDC9	DBP	Wald ratio	1	-0.665	0.457	0.145	-	0.492	No	1	-0.665	0.457	0.145	-	0.493
TXNRD1	DBP	Wald ratio	1	0.093	0.535	0.862	-	0.976	No	1	0.093	0.535	0.862	-	0.976
TYMP	DBP	Wald ratio	1	0.333	0.126	0.008	-	0.087	No	1	0.333	0.126	0.008	-	0.088
TYRO3	DBP	Inverse variance weighted	8	0.171	0.062	0.006	0.007	0.072	No	8	0.171	0.062	0.006	0.007	0.072
TYRP1	DBP	Wald ratio	1	0.612	0.309	0.048	-	0.261	No	1	0.612	0.309	0.048	-	0.262
UBAC1	DBP	Wald ratio	1	-0.345	0.365	0.344	-	0.724	No	1	-0.345	0.365	0.344	-	0.725
UBE2L6	DBP	Inverse variance weighted	2	0.025	0.099	0.798	-	0.948	No	2	0.025	0.099	0.798	-	0.948
ULBP2	DBP	Inverse variance weighted	8	-0.036	0.050	0.471	0.249	0.811	No	8	-0.036	0.050	0.471	0.249	0.812
UMOD	DBP	Inverse variance weighted	10	0.303	0.038	0.000	0.088	0.000	Yes	10	0.303	0.038	0.000	0.088	0.000
UNC5D	DBP	Inverse variance weighted	2	0.104	0.743	0.888	-	0.981	No	2	0.104	0.743	0.888	-	0.981
UNG	DBP	Wald ratio	1	-0.477	0.539	0.376	-	0.751	No	1	-0.477	0.539	0.376	-	0.752
UPB1	DBP	Wald ratio	1	0.170	0.609	0.780	-	0.939	No	1	0.170	0.609	0.780	-	0.939
UROD	DBP	Wald ratio	1	-0.025	0.054	0.642	-	0.900	No	1	-0.025	0.054	0.642	-	0.900
UROS	DBP	Wald ratio	1	0.378	0.209	0.071	-	0.336	No	1	0.378	0.209	0.071	-	0.337
USP28	DBP	Inverse variance weighted	2	-0.922	0.688	0.180	-	0.554	No	2	-0.922	0.688	0.180	-	0.555
USP8	DBP	Wald ratio	1	0.260	0.306	0.396	-	0.767	No	1	0.260	0.306	0.396	-	0.768
UXS1	DBP	Inverse variance weighted	2	-0.892	0.259	0.001	-	0.011	Yes	2	-0.892	0.259	0.001	-	0.011
VAMP5	DBP	Inverse variance weighted	2	-0.036	0.382	0.925	-	0.986	No	2	-0.036	0.382	0.925	-	0.986
VAMP8	DBP	Inverse variance weighted	2	0.190	0.145	0.191	-	0.564	No	2	0.190	0.145	0.191	-	0.565
VASH1	DBP	Inverse variance weighted	2	0.001	0.199	0.994	-	1.000	No	2	0.001	0.199	0.994	-	1.000
VASN	DBP	Inverse variance weighted	11	0.066	0.060	0.268	0.127	0.656	No	11	0.066	0.060	0.268	0.127	0.657
VAT1	DBP	Wald ratio	1	0.507	0.168	0.002	-	0.037	Yes	1	0.507	0.168	0.002	-	0.037
VAV3	DBP	Inverse variance weighted	2	0.635	0.518	0.220	-	0.599	No	2	0.635	0.518	0.220	-	0.600
VCAM1	DBP	Inverse variance weighted	2	-0.488	0.247	0.048	-	0.261	No	2	-0.488	0.247	0.048	-	0.262

ST3: MR causal estimates for plasma proteins on diastolic blood pressure.

Causal candidates prioritized for DBP were marked as "Yes" in column "Prioritized". Effect of plasma protein levels on blood pressure is in mmHg unit.

										Steiger filtering					
Exposure	Outocme	Method	nsnp	Beta	SE	P-value	Cochran's Q P-value	FDR-corrected P-value	Prioritized	nsnp	Beta	SE	P-value	Cochran's Q P-value	FDR-corrected P-value
VCAN	DBP	Inverse variance weighted	3	-0.063	0.035	0.073	0.516	0.344	No	3	-0.063	0.035	0.073	0.516	0.346
VCPKMT	DBP	Inverse variance weighted	2	-0.326	0.340	0.339	-	0.724	No	2	-0.326	0.340	0.339	-	0.724
VEGFA	DBP	Inverse variance weighted	8	-0.067	0.048	0.163	0.038	0.525	No	8	-0.067	0.048	0.163	0.038	0.526
VEGFB	DBP	Inverse variance weighted	2	-0.263	0.514	0.608	-	0.885	No	2	-0.263	0.514	0.608	-	0.885
VEGFC	DBP	Inverse variance weighted	2	0.387	0.247	0.117	-	0.459	No	2	0.387	0.247	0.117	-	0.461
VGF	DBP	Wald ratio	1	-0.769	0.383	0.045	-	0.255	No	1	-0.769	0.383	0.045	-	0.256
VIT	DBP	Inverse variance weighted	7	0.017	0.081	0.832	0.005	0.963	No	7	0.017	0.081	0.832	0.005	0.963
VMO1	DBP	Inverse variance weighted	15	0.035	0.030	0.242	0.450	0.627	No	15	0.035	0.030	0.242	0.450	0.628
VNN1	DBP	Inverse variance weighted	8	-0.030	0.027	0.253	0.305	0.640	No	8	-0.030	0.027	0.253	0.305	0.641
VNN2	DBP	Inverse variance weighted	6	0.055	0.042	0.197	0.200	0.567	No	6	0.055	0.042	0.197	0.200	0.568
VPS4B	DBP	Wald ratio	1	0.031	0.514	0.952	-	0.992	No	1	0.031	0.514	0.952	-	0.992
VSIG10	DBP	Inverse variance weighted	4	0.060	0.042	0.155	0.148	0.509	No	4	0.060	0.042	0.155	0.148	0.510
VSIG10L	DBP	Wald ratio	1	0.090	0.110	0.413	-	0.777	No	1	0.090	0.110	0.413	-	0.778
VSIG2	DBP	Inverse variance weighted	2	0.447	0.126	0.000	-	0.008	Yes	2	0.447	0.126	0.000	-	0.008
VSIR	DBP	Inverse variance weighted	3	-0.009	0.071	0.897	0.696	0.982	No	3	-0.009	0.071	0.897	0.696	0.982
VSNL1	DBP	Inverse variance weighted	2	-0.135	0.385	0.726	-	0.932	No	2	-0.135	0.385	0.726	-	0.932
VSTM1	DBP	Inverse variance weighted	7	0.030	0.040	0.451	0.078	0.800	No	7	0.030	0.040	0.451	0.078	0.800
VSTM2L	DBP	Inverse variance weighted	2	0.095	0.154	0.537	-	0.846	No	2	0.095	0.154	0.537	-	0.847
VTCN1	DBP	Inverse variance weighted	2	-0.166	0.310	0.593	-	0.876	No	2	-0.166	0.310	0.593	-	0.876
VTI1A	DBP	Wald ratio	1	0.370	0.395	0.348	-	0.729	No	1	0.370	0.395	0.348	-	0.730
VWA1	DBP	Inverse variance weighted	4	0.127	0.098	0.194	0.023	0.567	No	4	0.127	0.098	0.194	0.023	0.568
VWC2	DBP	Inverse variance weighted	5	0.060	0.123	0.626	0.788	0.891	No	5	0.060	0.123	0.626	0.788	0.892
VWC2L	DBP	Inverse variance weighted	2	-0.432	0.360	0.230	-	0.614	No	2	-0.432	0.360	0.230	-	0.615
VWF	DBP	Inverse variance weighted	2	0.018	0.221	0.934	-	0.986	No	2	0.018	0.221	0.934	-	0.986
WARS	DBP	Wald ratio	1	0.326	0.113	0.004	-	0.053	No	1	0.326	0.113	0.004	-	0.054
WASF1	DBP	Inverse variance weighted	3	0.034	0.078	0.661	0.612	0.909	No	3	0.034	0.078	0.661	0.612	0.910
WASHC3	DBP	Wald ratio	1	0.558	0.309	0.071	-	0.338	No	1	0.558	0.309	0.071	-	0.339
WFDC1	DBP	Inverse variance weighted	2	-0.021	0.086	0.809	-	0.955	No	2	-0.021	0.086	0.809	-	0.955
WFDC12	DBP	Inverse variance weighted	6	0.038	0.052	0.464	0.373	0.807	No	6	0.038	0.052	0.464	0.373	0.808
WFDC2	DBP	Inverse variance weighted	2	0.441	0.642	0.492	-	0.823	No	2	0.441	0.642	0.492	-	0.824
WFIKKN1	DBP	Inverse variance weighted	3	0.150	0.264	0.571	0.000	0.865	No	3	0.150	0.264	0.571	0.000	0.866
WFIKKN2	DBP	Inverse variance weighted	4	-0.046	0.070	0.514	0.009	0.832	No	4	-0.046	0.070	0.514	0.009	0.832
WIF1	DBP	Inverse variance weighted	3	-0.022	0.117	0.853	0.618	0.973	No	3	-0.022	0.117	0.853	0.618	0.973
WNT9A	DBP	Inverse variance weighted	2	0.591	0.169	0.000	-	0.010	Yes	2	0.591	0.169	0.000	-	0.010
WWP2	DBP	Wald ratio	1	-0.705	0.148	0.000	-	0.000	Yes	1	-0.705	0.148	0.000	-	0.000
XCL1	DBP	Inverse variance weighted	5	0.016	0.045	0.726	0.135	0.931	No	5	0.016	0.045	0.726	0.135	0.932
XRCC4	DBP	Wald ratio	1	-0.162	0.375	0.666	-	0.911	No	1	-0.162	0.375	0.666	-	0.911
YAP1	DBP	Wald ratio	1	1.385	0.363	0.000	-	0.004	Yes	1	1.385	0.363	0.000	-	0.004
YES1	DBP	Wald ratio	1	-0.382	0.321	0.233	-	0.620	No	1	-0.382	0.321	0.233	-	0.621
YJU2	DBP	Wald ratio	1	-0.068	0.441	0.878	-	0.980	No	1	-0.068	0.441	0.878	-	0.980
YOD1	DBP	Wald ratio	1	-1.155	0.213	0.000	-	0.000	Yes	1	-1.155	0.213	0.000	-	0.000
ZBP1	DBP	Inverse variance weighted	3	-0.019	0.128	0.885	0.049	0.980	No	3	-0.019	0.128	0.885	0.049	0.980
ZBTB16	DBP	Inverse variance weighted	3	0.065	0.066	0.329	0.541	0.717	No	3	0.065	0.066	0.329	0.541	0.718
ZBTB17	DBP	Wald ratio	1	1.930	0.524	0.000	-	0.005	Yes	1	1.930	0.524	0.000	-	0.005
ZFYVE19	DBP	Wald ratio	1	-0.102	0.065	0.116	-	0.457	No	1	-0.102	0.065	0.116	-	0.458
ZP3	DBP	Inverse variance weighted	14	-0.041	0.021	0.045	0.424	0.255	No	14	-0.041	0.021	0.045	0.424	0.256
ZPR1	DBP	Wald ratio	1	0.617	0.424	0.146	-	0.492	No	1	0.617	0.424	0.146	-	0.493

nsnp = number of single nucleotide polymorphisms; SE = standard error; Cochran's Q P-value = p-value from Cochran's Q test assessing heterogeneity; FDR=false discovery rate

ST4: MR causal estimates for blood pressure measures on plasma proteins.

All estimates are from inverse variance weighted method (nsnps >1) or Wald-ratio method (nsnps = 1). Effect of blood pressure on plasma protein levels is in standard deviation unit.

Outcome	Exposure	nsnp	Beta	SE	P-value	FDR-corrected P-value	FDR-corrected Cochran's Q P-value
AAMDC	DBP	500	-0.003	0.007	0.648	0.811	0.000
ABO	DBP	500	0.000	0.001	0.984	0.996	0.371
ACADM	DBP	500	0.003	0.002	0.088	0.313	0.710
ACOX1	DBP	500	-0.001	0.002	0.523	0.757	0.057
ADCYAP1R1	DBP	500	-0.002	0.002	0.215	0.511	0.065
ADM	DBP	500	0.008	0.002	0.000	0.002	0.000
AGRP	DBP	500	0.006	0.002	0.001	0.015	0.000
AMFR	DBP	500	0.001	0.002	0.561	0.778	0.098
AMOTL2	DBP	500	0.001	0.002	0.596	0.788	0.016
ANKMY2	DBP	500	0.002	0.002	0.347	0.606	0.000
AOC1	DBP	500	0.000	0.002	0.850	0.919	0.045
AOC3	DBP	500	0.001	0.002	0.420	0.684	0.000
APOA1	DBP	500	-0.004	0.002	0.012	0.100	0.000
APOBR	DBP	500	0.000	0.001	0.769	0.880	0.000
APOC1	DBP	500	-0.009	0.002	0.000	0.001	0.000
ARHGEF12	DBP	500	0.002	0.002	0.277	0.548	0.002
ARSB	DBP	500	0.001	0.002	0.717	0.864	0.000
ATXN2L	DBP	500	0.003	0.002	0.135	0.414	0.001
AXL	DBP	500	0.009	0.002	0.000	0.001	0.000
BAG4	DBP	500	0.002	0.002	0.193	0.504	0.000
BNIP3L	DBP	500	0.004	0.002	0.038	0.200	0.002
BRAP	DBP	500	0.002	0.002	0.204	0.509	0.000
CA12	DBP	500	0.006	0.002	0.009	0.079	0.000
CACNB3	DBP	500	0.001	0.002	0.532	0.757	0.000
CALB2	DBP	500	0.004	0.002	0.037	0.200	0.000
CALCA	DBP	500	0.004	0.002	0.013	0.100	0.000
CCN3	DBP	500	0.008	0.003	0.008	0.078	0.000
CEP170	DBP	500	0.002	0.002	0.188	0.500	0.000
CES2	DBP	500	0.000	0.002	0.995	0.996	0.000
CFHR4	DBP	500	-0.001	0.001	0.399	0.662	0.000
CHMP1A	DBP	500	0.002	0.002	0.251	0.546	0.000
CLIC5	DBP	500	0.002	0.002	0.236	0.531	0.003
COL1A1	DBP	500	0.000	0.002	0.984	0.996	0.000
CTF1	DBP	500	-0.001	0.002	0.535	0.757	0.000
DAG1	DBP	500	0.002	0.002	0.380	0.646	0.000
DARS1	DBP	500	0.002	0.002	0.298	0.569	0.000
DBN1	DBP	500	-0.001	0.002	0.737	0.869	0.004
DDHD2	DBP	500	0.002	0.002	0.333	0.587	0.000
DENR	DBP	500	0.001	0.002	0.768	0.880	0.001
DNER	DBP	500	-0.004	0.002	0.101	0.345	0.000
DOK2	DBP	500	0.001	0.002	0.570	0.778	0.000
DPEP1	DBP	500	-0.006	0.006	0.274	0.548	0.000
DPP4	DBP	500	-0.001	0.002	0.591	0.788	0.000

ST4: MR causal estimates for blood pressure measures on plasma proteins.
All estimates are from inverse variance weighted method (nsnps >1) or Wald-ratio method (nsnps = 1). Effect of blood pressure on plasma protein levels is in standard deviation unit.

Outcome	Exposure	nsnp	Beta	SE	P-value	FDR-corrected P-value	FDR-corrected Cochran's Q P-value
DUSP13	DBP	500	-0.002	0.002	0.178	0.493	0.000
EFNA1	DBP	500	0.005	0.002	0.017	0.122	0.000
EIF4G3	DBP	500	0.001	0.002	0.475	0.727	0.022
ELOA	DBP	500	0.004	0.002	0.023	0.135	0.000
ENPEP	DBP	500	-0.001	0.002	0.426	0.684	0.017
EPHA2	DBP	500	0.001	0.002	0.524	0.757	0.000
EPO	DBP	500	0.003	0.002	0.212	0.511	0.000
ERI1	DBP	500	-0.001	0.002	0.657	0.816	0.060
ERP29	DBP	500	0.001	0.002	0.615	0.788	0.000
ESAM	DBP	500	0.001	0.002	0.684	0.832	0.000
F12	DBP	500	0.002	0.001	0.223	0.515	0.000
FADD	DBP	500	0.001	0.002	0.479	0.727	0.000
FDX1	DBP	500	0.003	0.002	0.136	0.414	0.004
FES	DBP	500	-0.003	0.002	0.180	0.493	0.000
FGF12	DBP	500	0.000	0.002	0.815	0.902	0.191
FGF5	DBP	500	0.017	0.006	0.007	0.077	0.000
FOXJ3	DBP	500	0.002	0.002	0.284	0.548	0.033
FURIN	DBP	500	0.010	0.003	0.000	0.003	0.000
GBP2	DBP	500	0.005	0.002	0.001	0.017	0.071
GFER	DBP	500	0.001	0.002	0.526	0.757	0.000
GIMAP7	DBP	500	-0.002	0.002	0.222	0.515	0.000
GLO1	DBP	500	0.001	0.002	0.502	0.755	0.000
GORASP2	DBP	500	0.000	0.002	0.821	0.902	0.000
GRPEL1	DBP	500	0.003	0.002	0.118	0.379	0.000
GRP	DBP	500	0.005	0.002	0.013	0.100	0.000
GSTM4	DBP	500	-0.004	0.005	0.360	0.622	0.000
HADH	DBP	500	0.000	0.002	0.889	0.952	0.299
HEXIM1	DBP	500	0.003	0.002	0.088	0.313	0.000
HHEX	DBP	500	0.003	0.002	0.158	0.451	0.000
HPGDS	DBP	500	0.001	0.002	0.614	0.788	0.000
ICAM1	DBP	500	0.008	0.002	0.000	0.001	0.000
ICAM4	DBP	500	0.001	0.002	0.566	0.778	0.000
IFIT3	DBP	500	0.002	0.002	0.231	0.525	0.000
IGFBP3	DBP	500	0.009	0.005	0.079	0.312	0.000
IL1RL1	DBP	500	0.000	0.002	0.825	0.902	0.000
IMMT	DBP	500	0.005	0.002	0.004	0.043	0.001
ING1	DBP	500	0.003	0.002	0.078	0.312	0.006
ITGAL	DBP	500	0.005	0.002	0.020	0.125	0.000
KIF22	DBP	500	0.000	0.002	0.924	0.957	0.000
KIFBP	DBP	500	0.002	0.002	0.263	0.546	0.000
LACRT	DBP	500	-0.003	0.002	0.067	0.288	0.000
LAYN	DBP	500	-0.001	0.003	0.758	0.880	0.000
LILRA5	DBP	500	0.007	0.002	0.001	0.017	0.000

ST4: MR causal estimates for blood pressure measures on plasma proteins.

All estimates are from inverse variance weighted method (nsnps >1) or Wald-ratio method (nsnps = 1). Effect of blood pressure on plasma protein levels is in standard deviation unit.

Outcome	Exposure	nsnp	Beta	SE	P-value	FDR-corrected P-value	FDR-corrected Cochran's Q P-value
LMOD1	DBP	500	0.002	0.002	0.276	0.548	0.000
LMOD1	DBP	500	0.004	0.002	0.019	0.125	0.000
LMOD1	DBP	500	0.002	0.002	0.202	0.509	0.000
LMOD1	DBP	500	0.004	0.002	0.048	0.232	0.000
M6PR	DBP	500	0.001	0.002	0.681	0.832	0.144
MAP4K5	DBP	500	0.001	0.002	0.446	0.703	0.000
MFGE8	DBP	500	0.004	0.002	0.066	0.288	0.000
MPHOSPH8	DBP	500	0.000	0.002	0.852	0.919	0.000
MPI	DBP	500	-0.002	0.002	0.473	0.727	0.000
MSRA	DBP	500	0.001	0.002	0.778	0.880	0.000
MST1	DBP	500	0.003	0.001	0.056	0.263	0.000
NADK	DBP	500	0.001	0.003	0.753	0.880	0.000
NBN	DBP	500	0.005	0.002	0.010	0.090	0.000
NGF	DBP	500	0.001	0.002	0.386	0.646	0.176
NGFR	DBP	500	0.003	0.002	0.116	0.379	0.275
NOMO1	DBP	500	0.004	0.002	0.082	0.312	0.000
NOS3	DBP	500	0.007	0.002	0.000	0.003	0.000
NPPB	DBP	500	0.003	0.002	0.243	0.539	0.000
NUCB2	DBP	500	0.003	0.002	0.084	0.313	0.000
PALM2	DBP	500	0.006	0.002	0.001	0.011	0.000
PAM	DBP	500	0.001	0.003	0.824	0.902	0.000
PAMR1	DBP	500	0.006	0.002	0.003	0.036	0.000
PARP1	DBP	500	0.003	0.002	0.048	0.232	0.000
PCOLCE	DBP	501	0.007	0.003	0.008	0.078	0.001
PCSK7	DBP	500	0.003	0.002	0.118	0.379	0.036
PDE5A	DBP	500	0.000	0.002	0.940	0.963	0.000
PDIA3	DBP	500	-0.002	0.002	0.284	0.548	0.001
PECAM1	DBP	500	0.002	0.002	0.275	0.548	0.000
PFKFB2	DBP	500	0.000	0.003	0.900	0.952	0.000
PLA2G1B	DBP	500	0.000	0.002	0.925	0.957	0.000
PLXDC2	DBP	500	-0.003	0.002	0.189	0.500	0.000
PMS1	DBP	500	0.002	0.002	0.206	0.509	0.057
PRDX1	DBP	501	0.002	0.002	0.155	0.451	0.021
PROCR	DBP	500	0.000	0.002	0.773	0.880	0.189
PRSS53	DBP	500	0.005	0.005	0.306	0.577	0.000
PRTFDC1	DBP	500	0.001	0.002	0.645	0.811	0.000
PSMD9	DBP	500	0.005	0.002	0.002	0.028	0.071
PSRC1	DBP	500	0.001	0.002	0.472	0.727	0.015
PTPRF	DBP	500	0.001	0.002	0.685	0.832	0.000
PTRHD1	DBP	500	0.002	0.002	0.259	0.546	0.000
PYDC1	DBP	500	0.002	0.002	0.260	0.546	0.000
RELT	DBP	500	0.002	0.004	0.522	0.757	0.000
SDC1	DBP	500	0.003	0.003	0.319	0.577	0.000

ST4: MR causal estimates for blood pressure measures on plasma proteins.
All estimates are from inverse variance weighted method (nsnps >1) or Wald-ratio method (nsnps = 1). Effect of blood pressure on plasma protein levels is in standard deviation unit.

Outcome	Exposure	nsnp	Beta	SE	P-value	FDR-corrected P-value	FDR-corrected Cochran's Q P-value
SDCCAG8	DBP	500	0.000	0.002	0.928	0.957	0.000
SDHB	DBP	500	-0.003	0.002	0.141	0.420	0.000
SERPING1	DBP	500	0.001	0.002	0.606	0.788	0.000
SERPINI1	DBP	500	0.000	0.005	0.913	0.957	0.000
SH2B3	DBP	500	0.001	0.002	0.544	0.762	0.000
SHMT1	DBP	500	0.005	0.005	0.320	0.577	0.000
SLC16A1	DBP	500	0.004	0.002	0.060	0.273	0.000
SLC9A3R2	DBP	500	0.007	0.002	0.000	0.002	0.000
SMTN	DBP	500	0.002	0.002	0.160	0.451	0.019
SPARCL1	DBP	500	0.001	0.002	0.633	0.804	0.000
SPINK8	DBP	500	0.007	0.004	0.075	0.312	0.000
SPRING1	DBP	500	0.000	0.002	0.899	0.952	0.477
STC1	DBP	500	0.002	0.002	0.311	0.577	0.000
STX4	DBP	500	0.002	0.002	0.208	0.509	0.002
SYAP1	DBP	500	0.001	0.002	0.446	0.703	0.000
TBC1D17	DBP	500	0.002	0.002	0.259	0.546	0.018
TIE1	DBP	500	-0.001	0.003	0.799	0.898	0.000
TJAP1	DBP	500	0.002	0.002	0.320	0.577	0.000
TMEM106A	DBP	500	0.001	0.002	0.597	0.788	0.000
TNFRSF12A	DBP	500	0.004	0.002	0.038	0.200	0.000
TNFRSF17	DBP	500	-0.001	0.002	0.511	0.757	0.000
TNFSF12	DBP	500	-0.002	0.002	0.424	0.684	0.000
TP53BP1	DBP	500	-0.001	0.002	0.726	0.866	0.000
TP53I3	DBP	500	0.003	0.002	0.090	0.313	0.000
TP53INP1	DBP	500	-0.004	0.002	0.020	0.125	0.507
TP53	DBP	500	0.002	0.002	0.386	0.646	0.004
UMOD	DBP	500	0.004	0.007	0.608	0.788	0.000
UXS1	DBP	500	0.001	0.002	0.729	0.866	0.000
VAT1	DBP	500	0.000	0.002	0.996	0.996	0.000
VSIG2	DBP	500	0.004	0.002	0.039	0.200	0.000
WNT9A	DBP	500	0.002	0.002	0.330	0.587	0.000
WWP2	DBP	500	0.001	0.002	0.574	0.778	0.000
YAP1	DBP	500	0.004	0.002	0.022	0.135	0.000
YOD1	DBP	500	0.003	0.002	0.134	0.414	0.000
ZBTB17	DBP	500	0.003	0.002	0.082	0.312	0.000
ACADM	SBP	509	0.002	0.001	0.046	0.181	0.024
ACOX1	SBP	509	0.000	0.001	0.682	0.782	0.031
ACRBP	SBP	509	0.002	0.001	0.026	0.173	0.000
ADAM23	SBP	509	0.002	0.001	0.053	0.181	0.000
ADAMTS13	SBP	509	0.002	0.001	0.120	0.259	0.000
ADAMTS15	SBP	509	0.002	0.001	0.135	0.273	0.000
ADAMTS16	SBP	509	0.001	0.001	0.355	0.479	0.000
ADAMTS1	SBP	509	0.002	0.001	0.033	0.173	0.000

ST4: MR causal estimates for blood pressure measures on plasma proteins.

All estimates are from inverse variance weighted method (nsnps >1) or Wald-ratio method (nsnps = 1). Effect of blood pressure on plasma protein levels is in standard deviation unit.

Outcome	Exposure	nsnp	Beta	SE	P-value	FDR-corrected P-value	FDR-corrected Cochran's Q P-value
ADAMTS4	SBP	509	0.002	0.001	0.090	0.232	0.023
ADAMTS8	SBP	509	-0.001	0.002	0.681	0.782	0.000
ADAMTSL5	SBP	509	0.001	0.001	0.444	0.554	0.000
ADM	SBP	509	0.004	0.001	0.001	0.012	0.000
AGRP	SBP	509	0.003	0.001	0.008	0.078	0.000
AMOTL2	SBP	509	0.001	0.001	0.164	0.301	0.007
ANKMY2	SBP	509	0.001	0.001	0.173	0.302	0.000
APOA1	SBP	509	-0.001	0.001	0.193	0.319	0.000
APOA2	SBP	509	0.000	0.001	0.861	0.911	0.000
APOBR	SBP	509	0.001	0.001	0.192	0.319	0.000
APOC1	SBP	509	-0.005	0.001	0.000	0.008	0.000
ASPN	SBP	509	-0.002	0.001	0.115	0.255	0.000
ATXN2L	SBP	509	0.002	0.001	0.035	0.173	0.000
B4GAT1	SBP	509	-0.001	0.001	0.343	0.475	0.000
BAG4	SBP	509	0.002	0.001	0.040	0.173	0.000
BCAM	SBP	509	0.002	0.001	0.040	0.173	0.000
BMP6	SBP	509	0.001	0.001	0.335	0.466	0.000
BNIP3L	SBP	509	0.002	0.001	0.126	0.266	0.000
BRAP	SBP	509	0.002	0.001	0.066	0.193	0.000
BRSK2	SBP	509	0.000	0.001	0.839	0.901	0.457
CA12	SBP	509	0.002	0.001	0.065	0.193	0.000
CA9	SBP	509	-0.002	0.001	0.113	0.252	0.000
CACNB3	SBP	509	0.002	0.001	0.022	0.161	0.000
CALB2	SBP	509	0.003	0.001	0.006	0.076	0.000
CALCA	SBP	509	0.004	0.001	0.000	0.008	0.000
CALCOCO2	SBP	509	0.003	0.001	0.015	0.129	0.000
CCN3	SBP	509	0.002	0.001	0.231	0.360	0.000
CCND2	SBP	509	0.001	0.001	0.167	0.301	0.063
CD14	SBP	509	0.000	0.001	0.998	0.998	0.000
CD164L2	SBP	509	-0.002	0.001	0.039	0.173	0.156
CD46	SBP	509	0.002	0.001	0.038	0.173	0.000
CD59	SBP	509	0.002	0.001	0.064	0.193	0.000
CEP170	SBP	509	0.001	0.001	0.160	0.301	0.000
CERT	SBP	509	0.002	0.001	0.067	0.193	0.000
CETN3	SBP	509	0.002	0.001	0.151	0.289	0.000
CFHR2	SBP	509	0.000	0.001	0.844	0.901	0.000
CFHR4	SBP	509	-0.001	0.001	0.279	0.411	0.000
CLMP	SBP	509	0.001	0.001	0.276	0.410	0.000
COL1A1	SBP	509	-0.001	0.001	0.300	0.435	0.000
COMP	SBP	509	-0.002	0.001	0.188	0.319	0.000
COMT	SBP	509	0.001	0.002	0.595	0.712	0.000
CPTP	SBP	509	0.000	0.001	0.863	0.911	0.008
CPXM1	SBP	509	0.000	0.001	0.819	0.901	0.000

ST4: MR causal estimates for blood pressure measures on plasma proteins.

All estimates are from inverse variance weighted method (nsnps >1) or Wald-ratio method (nsnps = 1). Effect of blood pressure on plasma protein levels is in standard deviation unit.

Outcome	Exposure	nsnp	Beta	SE	P-value	FDR-corrected P-value	FDR-corrected Cochran's Q P-value
CTSO	SBP	509	0.002	0.001	0.139	0.277	0.000
DAG1	SBP	509	0.001	0.001	0.397	0.517	0.000
DDHD2	SBP	509	0.001	0.001	0.218	0.348	0.000
DNAJC9	SBP	509	0.002	0.001	0.037	0.173	0.001
DTX3	SBP	509	0.002	0.001	0.031	0.173	0.000
DUSP13	SBP	509	-0.003	0.001	0.007	0.077	0.000
DUSP29	SBP	509	0.001	0.001	0.306	0.441	0.005
EDN1	SBP	509	0.003	0.001	0.019	0.146	0.000
EFEMP1	SBP	509	0.001	0.001	0.646	0.756	0.000
EIF4G3	SBP	509	0.001	0.001	0.237	0.360	0.000
ELOA	SBP	509	0.003	0.001	0.019	0.146	0.000
ENPEP	SBP	509	0.000	0.001	0.839	0.901	0.009
ERI1	SBP	509	0.001	0.001	0.256	0.383	0.011
ERP29	SBP	509	0.002	0.001	0.075	0.207	0.000
ESAM	SBP	509	0.001	0.001	0.430	0.544	0.000
F13B	SBP	509	-0.001	0.001	0.499	0.612	0.000
FDX1	SBP	509	0.001	0.001	0.411	0.532	0.147
FES	SBP	509	-0.002	0.001	0.191	0.319	0.000
FGF20	SBP	509	0.000	0.001	0.980	0.985	0.725
FGF21	SBP	509	0.004	0.001	0.001	0.012	0.000
FGF23	SBP	509	0.005	0.001	0.000	0.000	0.000
FGF2	SBP	509	0.002	0.001	0.037	0.173	0.000
FGF5	SBP	509	0.010	0.004	0.004	0.051	0.000
FKBP7	SBP	509	0.002	0.001	0.057	0.183	0.027
FN1	SBP	509	-0.002	0.003	0.430	0.544	0.000
FOXJ3	SBP	509	0.002	0.001	0.058	0.183	0.000
FOXO3	SBP	509	0.001	0.001	0.310	0.441	0.038
FUCA1	SBP	509	0.001	0.001	0.088	0.232	0.000
FURIN	SBP	509	0.006	0.002	0.000	0.008	0.000
GCHFR	SBP	509	0.002	0.001	0.050	0.181	0.000
GFER	SBP	509	0.001	0.001	0.363	0.483	0.000
GHRHR	SBP	509	0.000	0.001	0.702	0.795	0.827
GHRL	SBP	509	-0.002	0.001	0.142	0.279	0.000
GHR	SBP	509	0.004	0.001	0.000	0.008	0.000
GIMAP7	SBP	509	0.000	0.001	0.643	0.756	0.000
GIT1	SBP	509	0.002	0.001	0.135	0.273	0.000
GORASP2	SBP	509	0.001	0.001	0.190	0.319	0.000
GRPEL1	SBP	509	0.002	0.001	0.103	0.248	0.000
GRP	SBP	509	0.004	0.001	0.009	0.082	0.000
HADH	SBP	509	-0.001	0.001	0.609	0.725	0.037
HEXIM1	SBP	509	0.003	0.001	0.007	0.077	0.000
HHEX	SBP	509	0.003	0.001	0.025	0.173	0.000
HMOX2	SBP	509	0.001	0.001	0.310	0.441	0.000

ST4: MR causal estimates for blood pressure measures on plasma proteins.

All estimates are from inverse variance weighted method (nsnps >1) or Wald-ratio method (nsnps = 1). Effect of blood pressure on plasma protein levels is in standard deviation unit.

Outcome	Exposure	nsnp	Beta	SE	P-value	FDR-corrected P-value	FDR-corrected Cochran's Q P-value
HYAL1	SBP	509	0.002	0.001	0.037	0.173	0.000
ICAM2	SBP	509	0.002	0.001	0.063	0.193	0.000
IDUA	SBP	509	0.000	0.001	0.884	0.925	0.000
IFI30	SBP	509	0.004	0.001	0.002	0.032	0.000
IFNGR2	SBP	509	0.003	0.002	0.118	0.259	0.000
IGFBP3	SBP	509	0.003	0.001	0.003	0.038	0.000
IMMT	SBP	509	0.003	0.001	0.031	0.173	0.000
IMPA1	SBP	509	0.001	0.001	0.181	0.313	0.000
ING1	SBP	509	0.002	0.001	0.073	0.202	0.000
ITGAL	SBP	509	0.002	0.001	0.058	0.183	0.000
ITIH1	SBP	509	0.000	0.001	0.966	0.985	0.676
KIFBP	SBP	509	0.002	0.001	0.103	0.248	0.000
LACRT	SBP	509	-0.001	0.001	0.555	0.677	0.000
LMOD1	SBP	509	0.002	0.001	0.052	0.181	0.000
LMOD1	SBP	509	0.003	0.001	0.002	0.032	0.000
LMOD1	SBP	509	0.001	0.001	0.170	0.301	0.000
LMOD1	SBP	509	0.001	0.001	0.162	0.301	0.000
LRIG1	SBP	509	0.001	0.001	0.426	0.544	0.000
LYAR	SBP	509	0.000	0.001	0.890	0.926	0.009
M6PR	SBP	509	0.002	0.001	0.040	0.173	0.038
MANEAL	SBP	509	0.001	0.001	0.205	0.335	0.038
MANSC4	SBP	509	0.000	0.001	0.976	0.985	0.001
MAP4K5	SBP	509	0.002	0.001	0.144	0.280	0.000
MDH1	SBP	509	0.002	0.001	0.040	0.173	0.000
MEGF9	SBP	509	0.002	0.001	0.110	0.251	0.000
MPHOSPH8	SBP	509	0.001	0.001	0.171	0.301	0.000
MPI	SBP	509	0.001	0.001	0.561	0.680	0.000
MSRA	SBP	509	0.001	0.001	0.183	0.314	0.000
MST1	SBP	509	0.003	0.002	0.113	0.252	0.000
MVK	SBP	509	0.006	0.001	0.000	0.000	0.000
MXRA8	SBP	509	-0.004	0.001	0.001	0.012	0.000
NADK	SBP	509	0.003	0.002	0.123	0.262	0.000
NAGA	SBP	509	0.002	0.001	0.089	0.232	0.000
NBN	SBP	509	0.002	0.001	0.040	0.173	0.000
NCR3LG1	SBP	509	-0.001	0.002	0.568	0.685	0.000
NFE2	SBP	509	0.003	0.001	0.007	0.076	0.000
NFU1	SBP	509	0.002	0.001	0.043	0.180	0.000
NGF	SBP	509	0.001	0.001	0.329	0.461	0.023
NGFR	SBP	509	0.001	0.001	0.237	0.360	0.573
NOTCH3	SBP	509	-0.002	0.001	0.171	0.301	0.000
NPPB	SBP	509	0.003	0.001	0.030	0.173	0.000
NTRK3	SBP	509	-0.002	0.001	0.170	0.301	0.000
NUCB2	SBP	509	0.001	0.002	0.683	0.782	9.4e-323

ST4: MR causal estimates for blood pressure measures on plasma proteins.

All estimates are from inverse variance weighted method (nsnps >1) or Wald-ratio method (nsnps = 1). Effect of blood pressure on plasma protein levels is in standard deviation unit.

Outcome	Exposure	nsnp	Beta	SE	P-value	FDR-corrected P-value	FDR-corrected Cochran's Q P-value
NUDT5	SBP	509	0.002	0.001	0.048	0.181	0.000
NUMB	SBP	509	0.002	0.001	0.036	0.173	0.000
OGA	SBP	509	0.002	0.001	0.069	0.197	0.000
OPLAH	SBP	509	0.002	0.001	0.018	0.145	0.000
OTUD6B	SBP	509	0.002	0.001	0.105	0.248	0.000
PCBP2	SBP	509	0.002	0.001	0.079	0.215	0.000
PCSK7	SBP	509	0.000	0.002	0.839	0.901	0.000
PDE5A	SBP	509	0.001	0.001	0.469	0.581	0.000
PDGFRA	SBP	509	0.000	0.001	0.975	0.985	0.000
PECAM1	SBP	509	0.002	0.001	0.107	0.248	0.000
PFKFB2	SBP	509	0.002	0.001	0.031	0.173	0.000
PGF	SBP	509	0.004	0.001	0.000	0.008	0.000
PHLDB1	SBP	509	0.002	0.001	0.047	0.181	0.000
PKD1	SBP	509	0.001	0.001	0.215	0.348	0.000
PLA2G1B	SBP	509	-0.001	0.001	0.325	0.458	0.000
PMS1	SBP	509	0.001	0.001	0.471	0.581	0.441
PMVK	SBP	509	0.002	0.001	0.095	0.239	0.000
PPP1R14D	SBP	509	0.001	0.001	0.414	0.533	0.004
PRG2	SBP	509	0.001	0.001	0.643	0.756	0.000
PRKAB1	SBP	509	0.002	0.001	0.054	0.181	0.001
PSMD5	SBP	509	0.000	0.001	0.842	0.901	0.682
PSMD9	SBP	509	0.001	0.001	0.140	0.277	0.038
PSRC1	SBP	509	0.001	0.001	0.356	0.479	0.000
PTRHD1	SBP	509	0.002	0.001	0.132	0.273	0.000
QPCT	SBP	509	0.001	0.001	0.219	0.348	0.000
RABEPK	SBP	509	0.001	0.001	0.246	0.369	0.191
RANBP1	SBP	509	0.001	0.001	0.238	0.360	0.000
RARRES1	SBP	509	0.002	0.001	0.073	0.202	0.000
RARRES2	SBP	509	0.002	0.001	0.036	0.173	0.000
RELT	SBP	509	0.000	0.002	0.811	0.901	0.000
RSPO3	SBP	509	-0.002	0.001	0.148	0.285	0.000
SCARA5	SBP	509	0.000	0.001	0.722	0.813	0.000
SDCCAG8	SBP	509	0.002	0.001	0.106	0.248	0.003
SDHB	SBP	509	0.000	0.001	0.929	0.952	0.000
SELENOP	SBP	509	0.000	0.001	0.835	0.901	0.007
SEMA6C	SBP	509	0.001	0.001	0.366	0.483	0.053
SERPING1	SBP	509	0.002	0.001	0.059	0.183	0.000
SERPINI1	SBP	509	0.000	0.003	0.884	0.925	0.000
SH2B3	SBP	509	0.002	0.001	0.106	0.248	0.000
SHMT1	SBP	509	0.004	0.003	0.218	0.348	0.000
SIL1	SBP	509	0.001	0.001	0.353	0.479	0.000
SLC16A1	SBP	509	0.002	0.001	0.053	0.181	0.002
SLC39A14	SBP	509	0.000	0.001	0.809	0.901	0.000

ST4: MR causal estimates for blood pressure measures on plasma proteins.

All estimates are from inverse variance weighted method (nsnps >1) or Wald-ratio method (nsnps = 1). Effect of blood pressure on plasma protein levels is in standard deviation unit.

Outcome	Exposure	nsnp	Beta	SE	P-value	FDR-corrected P-value	FDR-corrected Cochran's Q P-value
SLC9A3R2	SBP	509	0.006	0.001	0.000	0.000	0.000
SMOC2	SBP	509	0.001	0.001	0.359	0.480	0.000
SOST	SBP	509	0.000	0.001	0.691	0.787	0.000
SPINK8	SBP	509	0.001	0.002	0.441	0.554	0.000
SPRED2	SBP	509	0.002	0.001	0.047	0.181	0.182
SPRING1	SBP	509	-0.001	0.001	0.171	0.301	0.325
TARBP2	SBP	509	0.002	0.001	0.099	0.246	0.000
TBC1D23	SBP	509	0.002	0.001	0.093	0.236	0.000
TEK	SBP	509	0.002	0.001	0.133	0.273	0.000
TGFB2	SBP	509	0.000	0.001	0.909	0.941	0.241
TIE1	SBP	509	-0.001	0.002	0.353	0.479	0.000
TJAP1	SBP	509	0.001	0.001	0.298	0.435	0.000
TNFRSF13B	SBP	509	0.001	0.001	0.393	0.515	0.000
TNFRSF17	SBP	509	-0.001	0.001	0.669	0.779	0.000
TNFSF12	SBP	509	-0.003	0.001	0.050	0.181	0.000
TNFSF13B	SBP	509	0.002	0.001	0.122	0.261	0.000
TWF2	SBP	509	0.002	0.001	0.056	0.183	0.000
UBE2L6	SBP	509	0.002	0.001	0.087	0.232	0.000
UMOD	SBP	509	0.000	0.004	0.926	0.952	0.000
VSIG2	SBP	509	0.000	0.001	0.830	0.901	0.000
WARS	SBP	509	0.005	0.001	0.000	0.000	0.000
WASHC3	SBP	509	0.002	0.001	0.015	0.129	0.000
WWP2	SBP	509	0.002	0.001	0.049	0.181	0.000
YOD1	SBP	509	0.001	0.001	0.234	0.360	0.035
ZBTB17	SBP	509	0.002	0.001	0.048	0.181	0.000
ZFYVE19	SBP	509	0.001	0.001	0.233	0.360	0.000

nsnp = number of single nucleotide polymorphisms; SE = standard error; Cochran's Q P-value = p-value from Cochran's Q test assessing heterogeneity; FDR=false discovery rate

ST5: MR causal estimates for SBP-associated proteins on CAD.

Causal candidates prioritized for CAD were marked as "Yes" in column "Prioritized". All estimates are from inverse variance weighted method (nsnp >1) or Wald-ratio method (nsnp = 1). CAD, coronary artery disease.

										Steiger filtering					
Exposure	Outocme	nsnp	Beta	SE	P-value	Odds ratio	FDR-corrected P-value	FDR-corrected Cochran's Q P-value	Prioritized	nsnp	Beta	SE	P-value	Cochran's Q P-value	FDR-corrected P-value
ACOX1	CAD	1	0.296	0.078	0.000	1.345	0.002	-	Yes	1	0.296	0.078	0.000	-	0.002
ACRBP	CAD	1	0.128	0.040	0.001	1.136	0.014	-	Yes	1	0.128	0.040	0.001	-	0.014
ADAM23	CAD	5	-0.017	0.009	0.058	0.983	0.200	0.795	No	5	-0.017	0.009	0.058	0.455	0.200
ADAMTS1	CAD	1	-0.175	0.129	0.176	0.839	0.451	-	No	1	-0.175	0.129	0.176	-	0.451
ADAMTS4	CAD	1	0.015	0.049	0.756	1.015	0.905	-	No	1	0.015	0.049	0.756	-	0.905
ADAMTS8	CAD	7	0.003	0.017	0.865	1.003	0.950	0.156	No	7	0.003	0.017	0.865	0.031	0.950
ADAMTSL5	CAD	2	-0.008	0.016	0.607	0.992	0.823	-	No	2	-0.008	0.016	0.607	-	0.823
ADM	CAD	1	0.334	0.161	0.038	1.397	0.149	-	No	1	0.334	0.161	0.038	-	0.149
AMOTL2	CAD	1	-0.202	0.112	0.072	0.817	0.242	-	No	1	-0.202	0.112	0.072	-	0.242
ANKMY2	CAD	1	-0.059	0.077	0.444	0.943	0.754	-	No	1	-0.059	0.077	0.444	-	0.754
APOA1	CAD	1	0.238	0.091	0.009	1.269	0.049	-	Yes	1	0.238	0.091	0.009	-	0.049
APOA2	CAD	1	-0.051	0.091	0.576	0.950	0.823	-	No	1	-0.051	0.091	0.576	-	0.823
APOBR	CAD	3	0.000	0.010	0.986	1.000	0.999	0.156	No	3	0.000	0.010	0.986	0.035	0.999
APOC1	CAD	2	-0.018	0.222	0.937	0.982	0.997	-	No	2	-0.018	0.222	0.937	-	0.997
ASPN	CAD	2	-0.010	0.025	0.693	0.990	0.885	-	No	2	-0.010	0.025	0.693	-	0.885
ATXN2L	CAD	1	0.143	0.148	0.333	1.154	0.616	-	No	1	0.143	0.148	0.333	-	0.616
B4GAT1	CAD	3	-0.039	0.080	0.624	0.962	0.823	0.000	No	3	-0.039	0.080	0.624	0.000	0.823
BAG4	CAD	1	-0.324	0.160	0.042	0.723	0.158	-	No	1	-0.324	0.160	0.042	-	0.158
BCAM	CAD	5	0.103	0.048	0.031	1.109	0.128	0.063	No	5	0.103	0.048	0.031	0.004	0.128
BMP6	CAD	3	-0.099	0.034	0.004	0.906	0.030	0.995	Yes	3	-0.099	0.034	0.004	0.894	0.030
BNIP3L	CAD	1	-0.148	0.113	0.190	0.862	0.472	-	No	1	-0.148	0.113	0.190	-	0.472
BRAP	CAD	1	1.720	0.160	0.000	5.582	0.000	-	Yes	1	1.720	0.160	0.000	-	0.000
BRSK2	CAD	1	0.244	0.118	0.039	1.276	0.149	-	No	1	0.244	0.118	0.039	-	0.149
CA12	CAD	2	0.047	0.020	0.017	1.048	0.078	-	No	2	0.047	0.020	0.017	-	0.078
CA9	CAD	2	0.022	0.041	0.584	1.023	0.823	-	No	2	0.022	0.041	0.584	-	0.823
CACNB3	CAD	1	0.028	0.055	0.612	1.028	0.823	-	No	1	0.028	0.055	0.612	-	0.823
CALCA	CAD	1	-0.052	0.050	0.299	0.950	0.580	-	No	1	-0.052	0.050	0.299	-	0.580
CALCOCO2	CAD	1	0.121	0.103	0.242	1.129	0.542	-	No	1	0.121	0.103	0.242	-	0.542
CCN3	CAD	4	-0.014	0.026	0.588	0.986	0.823	0.551	No	4	-0.014	0.026	0.588	0.257	0.823
CCND2	CAD	1	-0.096	0.142	0.497	0.908	0.782	-	No	1	-0.096	0.142	0.497	-	0.782
CD14	CAD	1	-0.003	0.021	0.904	0.997	0.984	-	No	1	-0.003	0.021	0.904	-	0.984
CD164L2	CAD	1	-0.042	0.013	0.002	0.959	0.019	-	Yes	1	-0.042	0.013	0.002	-	0.019
CD46	CAD	1	-0.137	0.074	0.063	0.872	0.213	-	No	1	-0.137	0.074	0.063	-	0.213
CD59	CAD	2	-0.029	0.026	0.264	0.971	0.560	-	No	2	-0.029	0.026	0.264	-	0.560
CEP170	CAD	1	-0.021	0.092	0.816	0.979	0.921	-	No	1	-0.021	0.092	0.816	-	0.921
CERT	CAD	1	-0.117	0.117	0.317	0.890	0.596	-	No	1	-0.117	0.117	0.317	-	0.596
CETN3	CAD	1	0.014	0.037	0.712	1.014	0.894	-	No	1	0.014	0.037	0.712	-	0.894
CFHR2	CAD	8	0.012	0.009	0.189	1.012	0.472	0.156	No	8	0.012	0.009	0.189	0.032	0.472
CFHR4	CAD	8	0.007	0.012	0.575	1.007	0.823	0.105	No	8	0.007	0.012	0.575	0.014	0.823
CLMP	CAD	2	0.020	0.018	0.257	1.020	0.553	-	No	2	0.020	0.018	0.257	-	0.553
COL1A1	CAD	1	0.039	0.129	0.763	1.040	0.907	-	No	1	0.039	0.129	0.763	-	0.907
COMP	CAD	3	0.006	0.068	0.926	1.006	0.996	0.092	No	3	0.006	0.068	0.926	0.010	0.996
COMT	CAD	2	-0.045	0.040	0.255	0.956	0.553	-	No	2	-0.045	0.040	0.255	-	0.553
CPXM1	CAD	2	-0.022	0.017	0.200	0.978	0.486	-	No	2	-0.022	0.017	0.200	-	0.486
CTSO	CAD	1	-0.001	0.033	0.978	0.999	0.999	-	No	1	-0.001	0.033	0.978	-	0.999
DAG1	CAD	1	-0.437	0.096	0.000	0.646	0.000	-	Yes	1	-0.437	0.096	0.000	-	0.000
DDHD2	CAD	1	-0.119	0.055	0.030	0.888	0.128	-	No	1	-0.119	0.055	0.030	-	0.128
DTX3	CAD	1	0.117	0.066	0.076	1.125	0.246	-	No	1	0.117	0.066	0.076	-	0.246
DUSP13	CAD	1	-0.094	0.022	0.000	0.910	0.000	-	Yes	1	-0.094	0.022	0.000	-	0.000
DUSP29	CAD	1	-0.132	0.044	0.003	0.877	0.024	-	Yes	1	-0.132	0.044	0.003	-	0.024
EDN1	CAD	1	-0.004	0.023	0.868	0.996	0.950	-	No	1	-0.004	0.023	0.868	-	0.950
EFEMP1	CAD	2	0.076	0.029	0.008	1.079	0.046	-	Yes	2	0.076	0.029	0.008	-	0.046
EIF4G3	CAD	1	0.104	0.075	0.164	1.109	0.430	-	No	1	0.104	0.075	0.164	-	0.430
ELOA	CAD	1	-0.004	0.072	0.959	0.996	0.999	-	No	1	-0.004	0.072	0.959	-	0.999
ENPEP	CAD	2	-0.125	0.084	0.135	0.882	0.377	-	No	2	-0.125	0.084	0.135	-	0.377
ERI1	CAD	1	-0.028	0.083	0.730	0.972	0.897	-	No	1	-0.028	0.083	0.730	-	0.897

ST5: MR causal estimates for SBP-associated proteins on CAD.

Causal candidates prioritized for CAD were marked as "Yes" in column "Prioritized". All estimates are from inverse variance weighted method (nsnp >1) or Wald-ratio method (nsnp = 1). CAD, coronary artery disease.

										Steiger filtering					
Exposure	Outcme	nsnp	Beta	SE	P-value	Odds ratio	FDR-corrected P-value	FDR-corrected Cochran's Q P-value	Prioritized	nsnp	Beta	SE	P-value	Cochran's Q P-value	FDR-corrected P-value
ERP29	CAD	1	1.844	0.172	0.000	6.323	0.000	-	Yes	1	1.844	0.172	0.000	-	0.000
ESAM	CAD	1	0.034	0.047	0.476	1.034	0.782	-	No	1	0.034	0.047	0.476	-	0.782
F13B	CAD	5	0.026	0.013	0.051	1.026	0.181	0.895	No	5	0.026	0.013	0.051	0.639	0.181
FDX1	CAD	1	-0.333	0.120	0.005	0.717	0.036	-	Yes	1	-0.333	0.120	0.005	-	0.036
FES	CAD	1	-0.461	0.045	0.000	0.630	0.000	-	Yes	1	-0.461	0.045	0.000	-	0.000
FGF2	CAD	3	0.001	0.011	0.927	1.001	0.996	0.452	No	3	0.001	0.011	0.927	0.181	0.996
FGF5	CAD	5	0.070	0.009	0.000	1.072	0.000	0.895	Yes	5	0.070	0.009	0.000	0.648	0.000
FKBP7	CAD	1	0.023	0.083	0.783	1.023	0.911	-	No	1	0.023	0.083	0.783	-	0.911
FN1	CAD	2	-0.099	0.045	0.027	0.906	0.120	-	No	2	-0.099	0.045	0.027	-	0.120
FOXJ3	CAD	1	0.073	0.091	0.421	1.076	0.742	-	No	1	0.073	0.091	0.421	-	0.742
FOXO3	CAD	1	-0.075	0.168	0.656	0.928	0.856	-	No	1	-0.075	0.168	0.656	-	0.856
FUCA1	CAD	2	-0.014	0.016	0.376	0.986	0.684	-	No	2	-0.014	0.016	0.376	-	0.684
FURIN	CAD	1	0.304	0.032	0.000	1.355	0.000	-	Yes	1	0.304	0.032	0.000	-	0.000
GCHFR	CAD	1	-0.015	0.051	0.766	0.985	0.907	-	No	1	-0.015	0.051	0.766	-	0.907
GFER	CAD	1	0.000	0.105	1.000	1.000	1.000	-	No	1	0.000	0.105	1.000	-	1.000
GHR	CAD	6	-0.002	0.008	0.773	0.998	0.911	0.795	No	6	-0.002	0.008	0.773	0.407	0.911
GIMAP7	CAD	3	0.020	0.012	0.089	1.020	0.277	0.672	No	3	0.020	0.012	0.089	0.329	0.277
GIT1	CAD	2	0.303	0.283	0.284	1.354	0.576	-	No	2	0.303	0.283	0.284	-	0.576
GORASP2	CAD	1	-0.040	0.122	0.747	0.961	0.903	-	No	1	-0.040	0.122	0.747	-	0.903
GRP	CAD	5	0.016	0.030	0.599	1.016	0.823	0.067	No	5	0.016	0.030	0.599	0.006	0.823
HADH	CAD	1	0.072	0.105	0.493	1.075	0.782	-	No	1	0.072	0.105	0.493	-	0.782
HHEX	CAD	1	-0.470	0.157	0.003	0.625	0.024	-	Yes	1	-0.470	0.157	0.003	-	0.024
HMOX2	CAD	1	0.152	0.056	0.007	1.164	0.041	-	Yes	1	0.152	0.056	0.007	-	0.041
HYAL1	CAD	2	0.078	0.039	0.049	1.081	0.178	-	No	2	0.078	0.039	0.049	-	0.178
ICAM2	CAD	1	0.134	0.106	0.207	1.144	0.497	-	No	1	0.134	0.106	0.207	-	0.497
IDUA	CAD	5	-0.027	0.011	0.013	0.974	0.066	0.795	No	5	-0.027	0.011	0.013	0.445	0.066
IFI30	CAD	2	-0.027	0.039	0.483	0.973	0.782	-	No	2	-0.027	0.039	0.483	-	0.782
IFNGR2	CAD	9	0.010	0.017	0.556	1.010	0.823	0.000	No	9	0.010	0.017	0.556	0.000	0.823
IGFBP3	CAD	4	0.031	0.021	0.144	1.031	0.392	0.156	No	4	0.031	0.021	0.144	0.032	0.392
IMMT	CAD	1	-0.072	0.050	0.150	0.931	0.404	-	No	1	-0.072	0.050	0.150	-	0.404
IMPA1	CAD	1	0.041	0.029	0.163	1.041	0.430	-	No	1	0.041	0.029	0.163	-	0.430
ITGAL	CAD	1	-0.002	0.181	0.991	0.998	1.000	-	No	1	-0.002	0.181	0.991	-	1.000
ITIH1	CAD	1	-0.113	0.043	0.008	0.893	0.047	-	Yes	1	-0.113	0.043	0.008	-	0.047
KIFBP	CAD	1	-0.004	0.135	0.976	0.996	0.999	-	No	1	-0.004	0.135	0.976	-	0.999
LMOD1	CAD	1	-0.164	0.034	0.000	0.849	0.000	-	Yes	1	-0.164	0.034	0.000	-	0.000
LRIG1	CAD	7	0.012	0.007	0.093	1.012	0.287	0.995	No	7	0.012	0.007	0.093	0.928	0.287
LYAR	CAD	1	-0.030	0.121	0.803	0.970	0.921	-	No	1	-0.030	0.121	0.803	-	0.921
M6PR	CAD	1	-0.009	0.027	0.752	0.991	0.904	-	No	1	-0.009	0.027	0.752	-	0.904
MANEAL	CAD	1	0.826	0.160	0.000	2.283	0.000	-	Yes	1	0.826	0.160	0.000	-	0.000
MANSC4	CAD	4	-0.004	0.007	0.582	0.996	0.823	0.995	No	4	-0.004	0.007	0.582	0.974	0.823
MAP4K5	CAD	1	-0.002	0.032	0.955	0.998	0.999	-	No	1	-0.002	0.032	0.955	-	0.999
MDH1	CAD	2	0.250	0.114	0.028	1.284	0.120	-	No	2	0.250	0.114	0.028	-	0.120
MEGF9	CAD	1	0.030	0.019	0.112	1.030	0.331	-	No	1	0.030	0.019	0.112	-	0.331
MPHOSPH8	CAD	1	0.105	0.097	0.280	1.110	0.573	-	No	1	0.105	0.097	0.280	-	0.573
MPI	CAD	2	-0.029	0.027	0.298	0.972	0.580	-	No	2	-0.029	0.027	0.298	-	0.580
MSRA	CAD	1	0.021	0.106	0.839	1.022	0.935	-	No	1	0.021	0.106	0.839	-	0.935
MST1	CAD	2	0.029	0.006	0.000	1.029	0.000	-	Yes	2	0.029	0.006	0.000	-	0.000
MVK	CAD	1	-0.032	0.065	0.627	0.969	0.823	-	No	1	-0.032	0.065	0.627	-	0.823
NADK	CAD	1	-0.073	0.023	0.002	0.929	0.015	-	Yes	1	-0.073	0.023	0.002	-	0.015
NAGA	CAD	2	0.078	0.038	0.041	1.082	0.154	-	No	2	0.078	0.038	0.041	-	0.154
NFE2	CAD	1	0.067	0.086	0.440	1.069	0.754	-	No	1	0.067	0.086	0.440	-	0.754
NFU1	CAD	1	0.027	0.052	0.598	1.028	0.823	-	No	1	0.027	0.052	0.598	-	0.823
NGF	CAD	1	0.110	0.163	0.501	1.116	0.782	-	No	1	0.110	0.163	0.501	-	0.782
NOTCH3	CAD	3	0.060	0.082	0.464	1.062	0.782	0.995	No	3	0.060	0.082	0.464	0.800	0.782
NPPB	CAD	1	-0.021	0.032	0.512	0.979	0.789	-	No	1	-0.021	0.032	0.512	-	0.789
NTRK3	CAD	2	-0.078	0.028	0.006	0.925	0.036	-	Yes	2	-0.078	0.028	0.006	-	0.036

ST5: MR causal estimates for SBP-associated proteins on CAD.

Causal candidates prioritized for CAD were marked as "Yes" in column "Prioritized". All estimates are from inverse variance weighted method (nsnp >1) or Wald-ratio method (nsnp = 1). CAD, coronary artery disease.

										Steiger filtering					
Exposure	Outocme	nsnp	Beta	SE	P-value	Odds ratio	FDR-corrected P-value	FDR-corrected Cochran's Q P-value	Prioritized	nsnp	Beta	SE	P-value	Cochran's Q P-value	FDR-corrected P-value
NUCB2	CAD	2	-0.013	0.021	0.520	0.987	0.791	-	No	2	-0.013	0.021	0.520	-	0.791
NUDT5	CAD	1	0.125	0.091	0.170	1.133	0.441	-	No	1	0.125	0.091	0.170	-	0.441
NUMB	CAD	1	0.040	0.068	0.558	1.041	0.823	-	No	1	0.040	0.068	0.558	-	0.823
OGA	CAD	1	0.002	0.052	0.975	1.002	0.999	-	No	1	0.002	0.052	0.975	-	0.999
OPLAH	CAD	1	0.022	0.032	0.501	1.022	0.782	-	No	1	0.022	0.032	0.501	-	0.782
PCBP2	CAD	1	0.091	0.118	0.440	1.095	0.754	-	No	1	0.091	0.118	0.440	-	0.754
PCSK7	CAD	2	0.022	0.057	0.701	1.022	0.886	-	No	2	0.022	0.057	0.701	-	0.886
PDE5A	CAD	2	-0.159	0.036	0.000	0.853	0.000	-	Yes	2	-0.159	0.036	0.000	-	0.000
PDGFRA	CAD	3	-0.028	0.024	0.234	0.972	0.531	0.543	No	3	-0.028	0.024	0.234	0.241	0.531
PECAM1	CAD	1	-0.125	0.176	0.478	0.882	0.782	-	No	1	-0.125	0.176	0.478	-	0.782
PFKFB2	CAD	1	0.011	0.030	0.721	1.011	0.894	-	No	1	0.011	0.030	0.721	-	0.894
PGF	CAD	1	-0.228	0.038	0.000	0.796	0.000	-	Yes	1	-0.228	0.038	0.000	-	0.000
PHLDB1	CAD	1	-0.180	0.117	0.124	0.835	0.358	-	No	1	-0.180	0.117	0.124	-	0.358
PKD1	CAD	1	-0.062	0.113	0.582	0.940	0.823	-	No	1	-0.062	0.113	0.582	-	0.823
PLA2G1B	CAD	1	-0.241	0.114	0.034	0.786	0.137	-	No	1	-0.241	0.114	0.034	-	0.137
PMS1	CAD	1	0.062	0.128	0.627	1.064	0.823	-	No	1	0.062	0.128	0.627	-	0.823
PPP1R14D	CAD	1	-0.089	0.107	0.405	0.914	0.719	-	No	1	-0.089	0.107	0.405	-	0.719
PRG2	CAD	1	-0.012	0.060	0.841	0.988	0.935	-	No	1	-0.012	0.060	0.841	-	0.935
PRKAB1	CAD	1	0.008	0.033	0.804	1.008	0.921	-	No	1	0.008	0.033	0.804	-	0.921
PSMD5	CAD	1	-0.010	0.119	0.931	0.990	0.996	-	No	1	-0.010	0.119	0.931	-	0.996
PSRC1	CAD	2	0.061	0.031	0.050	1.063	0.181	-	No	2	0.061	0.031	0.050	-	0.181
PTRHD1	CAD	1	0.036	0.150	0.812	1.036	0.921	-	No	1	0.036	0.150	0.812	-	0.921
QPCT	CAD	3	0.030	0.014	0.028	1.030	0.120	0.995	No	3	0.030	0.014	0.028	0.973	0.120
RABEPK	CAD	1	-0.021	0.039	0.600	0.980	0.823	-	No	1	-0.021	0.039	0.600	-	0.823
RARRES1	CAD	5	0.014	0.012	0.258	1.014	0.553	0.810	No	5	0.014	0.012	0.258	0.522	0.553
RARRES2	CAD	1	0.044	0.029	0.134	1.045	0.376	-	No	1	0.044	0.029	0.134	-	0.376
RELT	CAD	2	-0.005	0.019	0.785	0.995	0.911	-	No	2	-0.005	0.019	0.785	-	0.911
RSPO3	CAD	1	0.149	0.034	0.000	1.161	0.000	-	Yes	1	0.149	0.034	0.000	-	0.000
SCARA5	CAD	4	-0.009	0.017	0.602	0.991	0.823	0.795	No	4	-0.009	0.017	0.602	0.459	0.823
SDCCAG8	CAD	1	0.071	0.028	0.012	1.073	0.061	-	No	1	0.071	0.028	0.012	-	0.061
SDHB	CAD	1	0.282	0.095	0.003	1.326	0.024	-	Yes	1	0.282	0.095	0.003	-	0.024
SELENOP	CAD	1	-0.064	0.057	0.267	0.938	0.561	-	No	1	-0.064	0.057	0.267	-	0.561
SEMA6C	CAD	1	-0.072	0.066	0.276	0.931	0.569	-	No	1	-0.072	0.066	0.276	-	0.569
SERPINI1	CAD	6	0.015	0.014	0.307	1.015	0.591	0.995	No	6	0.015	0.014	0.307	0.932	0.591
SH2B3	CAD	1	0.443	0.074	0.000	1.557	0.000	-	Yes	1	0.443	0.074	0.000	-	0.000
SHMT1	CAD	4	0.005	0.013	0.717	1.005	0.894	0.163	No	4	0.005	0.013	0.717	0.040	0.894
SIL1	CAD	1	-0.003	0.100	0.973	0.997	0.999	-	No	1	-0.003	0.100	0.973	-	0.999
SLC16A1	CAD	1	-0.245	0.096	0.011	0.783	0.059	-	No	1	-0.245	0.096	0.011	-	0.059
SLC39A14	CAD	1	0.117	0.061	0.057	1.124	0.199	-	No	1	0.117	0.061	0.057	-	0.199
SLC9A3R2	CAD	1	0.143	0.058	0.014	1.154	0.068	-	No	1	0.143	0.058	0.014	-	0.068
SMOC2	CAD	5	-0.010	0.015	0.524	0.990	0.791	0.895	No	5	-0.010	0.015	0.524	0.676	0.791
SOST	CAD	1	-0.121	0.115	0.292	0.886	0.580	-	No	1	-0.121	0.115	0.292	-	0.580
SPINK8	CAD	1	0.067	0.012	0.000	1.070	0.000	-	Yes	1	0.067	0.012	0.000	-	0.000
SPRED2	CAD	1	0.046	0.092	0.617	1.047	0.823	-	No	1	0.046	0.092	0.617	-	0.823
TARBP2	CAD	1	0.098	0.127	0.440	1.103	0.754	-	No	1	0.098	0.127	0.440	-	0.754
TBC1D23	CAD	1	-0.093	0.037	0.012	0.912	0.061	-	No	1	-0.093	0.037	0.012	-	0.061
TEK	CAD	11	0.015	0.012	0.211	1.015	0.499	0.895	No	11	0.015	0.012	0.211	0.666	0.499
TGFB2	CAD	1	0.015	0.040	0.702	1.015	0.886	-	No	1	0.015	0.040	0.702	-	0.886
TIE1	CAD	1	-0.114	0.030	0.000	0.892	0.002	-	Yes	1	-0.114	0.030	0.000	-	0.002
TJAP1	CAD	1	-0.399	0.142	0.005	0.671	0.034	-	Yes	1	-0.399	0.142	0.005	-	0.034
TNFRSF17	CAD	1	-0.070	0.043	0.106	0.932	0.322	-	No	1	-0.070	0.043	0.106	-	0.322
TNFSF12	CAD	2	-0.021	0.025	0.392	0.979	0.707	-	No	2	-0.021	0.025	0.392	-	0.707
TNFSF13B	CAD	5	-0.050	0.047	0.291	0.951	0.580	0.995	No	5	-0.050	0.047	0.291	0.860	0.580
TWF2	CAD	1	0.061	0.088	0.486	1.063	0.782	-	No	1	0.061	0.088	0.486	-	0.782
UBE2L6	CAD	2	-0.055	0.025	0.027	0.946	0.120	-	No	2	-0.055	0.025	0.027	-	0.120
UMOD	CAD	10	-0.002	0.007	0.806	0.998	0.921	0.819	No	10	-0.002	0.007	0.806	0.546	0.921

ST5: MR causal estimates for SBP-associated proteins on CAD.

Causal candidates prioritized for CAD were marked as "Yes" in column "Prioritized". All estimates are from inverse variance weighted method (nsnp >1) or Wald-ratio method (nsnp = 1). CAD, coronary artery disease.

										Steiger filtering					
Exposure	Outocme	nsnp	Beta	SE	P-value	Odds ratio	FDR-corrected P-value	FDR-corrected Cochran's Q P-value	Prioritized	nsnp	Beta	SE	P-value	Cochran's Q P-value	FDR-corrected P-value
VSIG2	CAD	1	-0.023	0.032	0.476	0.978	0.782	-	No	1	-0.023	0.032	0.476	-	0.782
WARS	CAD	1	0.082	0.028	0.003	1.085	0.027	-	Yes	1	0.082	0.028	0.003	-	0.027
WASHC3	CAD	1	-0.110	0.069	0.114	0.896	0.333	-	No	1	-0.110	0.069	0.114	-	0.333
WWP2	CAD	1	-0.045	0.038	0.234	0.956	0.531	-	No	1	-0.045	0.038	0.234	-	0.531
YOD1	CAD	1	0.023	0.053	0.661	1.023	0.858	-	No	1	0.023	0.053	0.661	-	0.858
ZBTB17	CAD	1	0.273	0.128	0.034	1.313	0.136	-	No	1	0.273	0.128	0.034	-	0.136
ZFYVE19	CAD	1	0.000	0.016	0.986	1.000	0.999	-	No	1	0.000	0.016	0.986	-	0.999

nsnp = number of single nucleotide polymorphisms; SE = standard error; Cochran's Q P-value = p-value from Cochran's Q test assessing heterogeneity; FDR=false discovery rate

ST6: MR causal estimates for DBP-associated proteins on CAD.

Causal candidates prioritized for CAD were marked as "Yes" in column "Prioritized". All estimates are from inverse variance weighted method (nspp >1) or Wald-ratio method (nspp = 1). CAD, coronary artery disease.

										Steiger filtering					
Exposure	Outcme	nsnp	Beta	SE	P-value	Odds ratio	FDR-corrected P-value	FDR-corrected Cochran's Q P-value	Prioritized	nsnp	Beta	SE	P-value	Cochran's Q P-value	FDR-corrected P-value
AAMDC	CAD	2	0.025	0.008	0.003	1.025	0.024	-	Yes	2	0.025	0.008	0.003	-	0.024
ABO	CAD	8	0.022	0.005	0.000	1.022	0.000	0.995	Yes	8	0.022	0.005	0.000	0.833	0.000
ACOX1	CAD	1	0.296	0.078	0.000	1.345	0.002	-	Yes	1	0.296	0.078	0.000	-	0.002
ADM	CAD	1	0.334	0.161	0.038	1.397	0.149	-	No	1	0.334	0.161	0.038	-	0.149
AMFR	CAD	1	0.204	0.134	0.130	1.226	0.370	-	No	1	0.204	0.134	0.130	-	0.370
AMOTL2	CAD	1	-0.202	0.112	0.072	0.817	0.242	-	No	1	-0.202	0.112	0.072	-	0.242
ANKMY2	CAD	1	-0.059	0.077	0.444	0.943	0.754	-	No	1	-0.059	0.077	0.444	-	0.754
AOC1	CAD	3	-0.051	0.029	0.078	0.950	0.250	0.429	No	3	-0.051	0.029	0.078	0.143	0.250
AOC3	CAD	3	-0.018	0.011	0.085	0.982	0.270	0.810	No	3	-0.018	0.011	0.085	0.508	0.270
APOA1	CAD	1	0.238	0.091	0.009	1.269	0.049	-	Yes	1	0.238	0.091	0.009	-	0.049
APOBR	CAD	3	0.000	0.010	0.986	1.000	0.999	0.156	No	3	0.000	0.010	0.986	0.035	0.999
APOC1	CAD	2	-0.018	0.222	0.937	0.982	0.997	-	No	2	-0.018	0.222	0.937	-	0.997
ARSB	CAD	4	-0.011	0.017	0.506	0.989	0.784	0.810	No	4	-0.011	0.017	0.506	0.510	0.784
ATXN2L	CAD	1	0.143	0.148	0.333	1.154	0.616	-	No	1	0.143	0.148	0.333	-	0.616
AXL	CAD	2	0.002	0.080	0.978	1.002	0.999	-	No	2	0.002	0.080	0.978	-	0.999
BAG4	CAD	1	-0.324	0.160	0.042	0.723	0.158	-	No	1	-0.324	0.160	0.042	-	0.158
BNIP3L	CAD	1	-0.148	0.113	0.190	0.862	0.472	-	No	1	-0.148	0.113	0.190	-	0.472
CA12	CAD	2	0.047	0.020	0.017	1.048	0.078	-	No	2	0.047	0.020	0.017	-	0.078
CACNB3	CAD	1	0.028	0.055	0.612	1.028	0.823	-	No	1	0.028	0.055	0.612	-	0.823
CALCA	CAD	1	-0.052	0.050	0.299	0.950	0.580	-	No	1	-0.052	0.050	0.299	-	0.580
CEP170	CAD	1	-0.021	0.092	0.816	0.979	0.921	-	No	1	-0.021	0.092	0.816	-	0.921
CES2	CAD	2	-0.072	0.072	0.321	0.931	0.597	-	No	2	-0.072	0.072	0.321	-	0.597
CFHR4	CAD	8	0.007	0.012	0.575	1.007	0.823	0.105	No	8	0.007	0.012	0.575	0.014	0.823
CHMP1A	CAD	1	-0.013	0.057	0.817	0.987	0.921	-	No	1	-0.013	0.057	0.817	-	0.921
CLIC5	CAD	1	-0.019	0.048	0.684	0.981	0.878	-	No	1	-0.019	0.048	0.684	-	0.878
COL1A1	CAD	1	0.039	0.129	0.763	1.040	0.907	-	No	1	0.039	0.129	0.763	-	0.907
CTF1	CAD	1	-0.057	0.117	0.623	0.944	0.823	-	No	1	-0.057	0.117	0.623	-	0.823
DAG1	CAD	1	-0.437	0.096	0.000	0.646	0.000	-	Yes	1	-0.437	0.096	0.000	-	0.000
DARS1	CAD	1	-0.084	0.083	0.311	0.919	0.593	-	No	1	-0.084	0.083	0.311	-	0.593
DBN1	CAD	1	0.012	0.112	0.916	1.012	0.994	-	No	1	0.012	0.112	0.916	-	0.994
DDHD2	CAD	1	-0.119	0.055	0.030	0.888	0.128	-	No	1	-0.119	0.055	0.030	-	0.128
DENR	CAD	1	-0.373	0.164	0.022	0.689	0.104	-	No	1	-0.373	0.164	0.022	-	0.104
DNER	CAD	5	-0.028	0.023	0.216	0.972	0.501	0.447	No	5	-0.028	0.023	0.216	0.159	0.501
DOK2	CAD	1	-0.294	0.107	0.006	0.745	0.036	-	Yes	1	-0.294	0.107	0.006	-	0.036
DPEP1	CAD	4	-0.023	0.036	0.521	0.977	0.791	0.995	No	4	-0.023	0.036	0.521	0.995	0.791
DPP4	CAD	2	-0.018	0.036	0.627	0.982	0.823	-	No	2	-0.018	0.036	0.627	-	0.823
DUSP13	CAD	1	-0.094	0.022	0.000	0.910	0.000	-	Yes	1	-0.094	0.022	0.000	-	0.000
EFNA1	CAD	1	0.021	0.018	0.254	1.021	0.553	-	No	1	0.021	0.018	0.254	-	0.553
EIF4G3	CAD	1	0.104	0.075	0.164	1.109	0.430	-	No	1	0.104	0.075	0.164	-	0.430
ELOA	CAD	1	-0.004	0.072	0.959	0.996	0.999	-	No	1	-0.004	0.072	0.959	-	0.999
ENPEP	CAD	2	-0.125	0.084	0.135	0.882	0.377	-	No	2	-0.125	0.084	0.135	-	0.377
EPHA2	CAD	2	-0.134	0.044	0.003	0.875	0.024	-	Yes	2	-0.134	0.044	0.003	-	0.024
EPO	CAD	1	0.026	0.078	0.735	1.027	0.898	-	No	1	0.026	0.078	0.735	-	0.898
ERI1	CAD	1	-0.028	0.083	0.730	0.972	0.897	-	No	1	-0.028	0.083	0.730	-	0.897
ESAM	CAD	1	0.034	0.047	0.476	1.034	0.782	-	No	1	0.034	0.047	0.476	-	0.782
F12	CAD	6	-0.007	0.007	0.345	0.993	0.633	0.452	No	6	-0.007	0.007	0.345	0.172	0.633
FADD	CAD	1	0.176	0.072	0.015	1.193	0.072	-	No	1	0.176	0.072	0.015	-	0.072
FDX1	CAD	1	-0.333	0.120	0.005	0.717	0.036	-	Yes	1	-0.333	0.120	0.005	-	0.036
FES	CAD	1	-0.461	0.045	0.000	0.630	0.000	-	Yes	1	-0.461	0.045	0.000	-	0.000
FGF5	CAD	5	0.070	0.009	0.000	1.072	0.000	0.895	Yes	5	0.070	0.009	0.000	0.648	0.000
FOXJ3	CAD	1	0.073	0.091	0.421	1.076	0.742	-	No	1	0.073	0.091	0.421	-	0.742
FURIN	CAD	1	0.304	0.032	0.000	1.355	0.000	-	Yes	1	0.304	0.032	0.000	-	0.000
GFER	CAD	1	0.000	0.105	1.000	1.000	1.000	-	No	1	0.000	0.105	1.000	-	1.000
GIMAP7	CAD	3	0.020	0.012	0.089	1.020	0.277	0.672	No	3	0.020	0.012	0.089	0.329	0.277
GLO1	CAD	2	0.032	0.025	0.212	1.032	0.499	-	No	2	0.032	0.025	0.212	-	0.499
GORASP2	CAD	1	-0.040	0.122	0.747	0.961	0.903	-	No	1	-0.040	0.122	0.747	-	0.903

ST6: MR causal estimates for DBP-associated proteins on CAD.

Causal candidates prioritized for CAD were marked as "Yes" in column "Prioritized". All estimates are from inverse variance weighted method (nsnp >1) or Wald-ratio method (nsnp = 1). CAD, coronary artery disease.

										Steiger filtering					
Exposure	Outcme	nsnp	Beta	SE	P-value	Odds ratio	FDR-corrected P-value	FDR-corrected Cochran's Q P-value	Prioritized	nsnp	Beta	SE	P-value	Cochran's Q P-value	FDR-corrected P-value
GRP	CAD	5	0.016	0.030	0.599	1.016	0.823	0.067	No	5	0.016	0.030	0.599	0.006	0.823
HADH	CAD	1	0.072	0.105	0.493	1.075	0.782	-	No	1	0.072	0.105	0.493	-	0.782
HHEX	CAD	1	-0.470	0.157	0.003	0.625	0.024	-	Yes	1	-0.470	0.157	0.003	-	0.024
HPGDS	CAD	7	0.016	0.016	0.313	1.016	0.593	0.286	No	7	0.016	0.016	0.313	0.089	0.593
ICAM1	CAD	5	0.000	0.017	0.997	1.000	1.000	0.524	No	5	0.000	0.017	0.997	0.221	1.000
ICAM4	CAD	1	-0.011	0.054	0.839	0.989	0.935	-	No	1	-0.011	0.054	0.839	-	0.935
IFIT3	CAD	1	0.101	0.056	0.074	1.106	0.244	-	No	1	0.101	0.056	0.074	-	0.244
IGFBP3	CAD	4	0.031	0.021	0.144	1.031	0.392	0.156	No	4	0.031	0.021	0.144	0.032	0.392
IL1RL1	CAD	7	0.016	0.012	0.178	1.016	0.451	0.171	No	7	0.016	0.012	0.178	0.046	0.451
IMMT	CAD	1	-0.072	0.050	0.150	0.931	0.404	-	No	1	-0.072	0.050	0.150	-	0.404
ITGAL	CAD	1	-0.002	0.181	0.991	0.998	1.000	-	No	1	-0.002	0.181	0.991	-	1.000
KIF22	CAD	1	0.205	0.166	0.217	1.228	0.501	-	No	1	0.205	0.166	0.217	-	0.501
KIFBP	CAD	1	-0.004	0.135	0.976	0.996	0.999	-	No	1	-0.004	0.135	0.976	-	0.999
LAYN	CAD	2	-0.075	0.016	0.000	0.927	0.000	-	Yes	2	-0.075	0.016	0.000	-	0.000
LMOD1	CAD	1	-0.164	0.034	0.000	0.849	0.000	-	Yes	1	-0.164	0.034	0.000	-	0.000
M6PR	CAD	1	-0.009	0.027	0.752	0.991	0.904	-	No	1	-0.009	0.027	0.752	-	0.904
MAP4K5	CAD	1	-0.002	0.032	0.955	0.998	0.999	-	No	1	-0.002	0.032	0.955	-	0.999
MFGE8	CAD	2	0.017	0.016	0.296	1.017	0.580	-	No	2	0.017	0.016	0.296	-	0.580
MPHOSPH8	CAD	1	0.105	0.097	0.280	1.110	0.573	-	No	1	0.105	0.097	0.280	-	0.573
MSRA	CAD	1	0.021	0.106	0.839	1.022	0.935	-	No	1	0.021	0.106	0.839	-	0.935
MST1	CAD	2	0.029	0.006	0.000	1.029	0.000	-	Yes	2	0.029	0.006	0.000	-	0.000
NADK	CAD	1	-0.073	0.023	0.002	0.929	0.015	-	Yes	1	-0.073	0.023	0.002	-	0.015
NGF	CAD	1	0.110	0.163	0.501	1.116	0.782	-	No	1	0.110	0.163	0.501	-	0.782
NOMO1	CAD	1	0.042	0.060	0.491	1.042	0.782	-	No	1	0.042	0.060	0.491	-	0.782
NOS3	CAD	1	-0.355	0.125	0.005	0.701	0.033	-	Yes	1	-0.355	0.125	0.005	-	0.033
NPPB	CAD	1	-0.021	0.032	0.512	0.979	0.789	-	No	1	-0.021	0.032	0.512	-	0.789
NUCB2	CAD	2	-0.013	0.021	0.520	0.987	0.791	-	No	2	-0.013	0.021	0.520	-	0.791
PAM	CAD	12	0.017	0.011	0.142	1.017	0.389	0.995	No	12	0.017	0.011	0.142	0.926	0.389
PARP1	CAD	1	-0.165	0.049	0.001	0.848	0.009	-	Yes	1	-0.165	0.049	0.001	-	0.009
PCOLCE	CAD	1	-0.006	0.035	0.865	0.994	0.950	-	No	1	-0.006	0.035	0.865	-	0.950
PCSK7	CAD	2	0.022	0.057	0.701	1.022	0.886	-	No	2	0.022	0.057	0.701	-	0.886
PDE5A	CAD	2	-0.159	0.036	0.000	0.853	0.000	-	Yes	2	-0.159	0.036	0.000	-	0.000
PDIA3	CAD	1	0.031	0.112	0.784	1.031	0.911	-	No	1	0.031	0.112	0.784	-	0.911
PECAM1	CAD	1	-0.125	0.176	0.478	0.882	0.782	-	No	1	-0.125	0.176	0.478	-	0.782
PFKFB2	CAD	1	0.011	0.030	0.721	1.011	0.894	-	No	1	0.011	0.030	0.721	-	0.894
PLA2G1B	CAD	1	-0.241	0.114	0.034	0.786	0.137	-	No	1	-0.241	0.114	0.034	-	0.137
PLXDC2	CAD	3	-0.043	0.057	0.444	0.958	0.754	0.995	No	3	-0.043	0.057	0.444	0.912	0.754
PMS1	CAD	1	0.062	0.128	0.627	1.064	0.823	-	No	1	0.062	0.128	0.627	-	0.823
PRDX1	CAD	1	-0.107	0.094	0.252	0.898	0.553	-	No	1	-0.107	0.094	0.252	-	0.553
PROCR	CAD	1	-0.071	0.013	0.000	0.931	0.000	-	Yes	1	-0.071	0.013	0.000	-	0.000
PRSS53	CAD	4	0.007	0.011	0.528	1.007	0.792	0.187	No	4	0.007	0.011	0.528	0.054	0.792
PRTFDC1	CAD	2	-0.002	0.031	0.956	0.998	0.999	-	No	2	-0.002	0.031	0.956	-	0.999
PSRC1	CAD	2	0.061	0.031	0.050	1.063	0.181	-	No	2	0.061	0.031	0.050	-	0.181
PTPRF	CAD	1	-0.112	0.070	0.112	0.894	0.331	-	No	1	-0.112	0.070	0.112	-	0.331
PTRHD1	CAD	1	0.036	0.150	0.812	1.036	0.921	-	No	1	0.036	0.150	0.812	-	0.921
PYDC1	CAD	1	-0.022	0.020	0.271	0.978	0.565	-	No	1	-0.022	0.020	0.271	-	0.565
RELT	CAD	2	-0.005	0.019	0.785	0.995	0.911	-	No	2	-0.005	0.019	0.785	-	0.911
SDC1	CAD	2	-0.035	0.069	0.612	0.966	0.823	-	No	2	-0.035	0.069	0.612	-	0.823
SDCCAG8	CAD	1	0.071	0.028	0.012	1.073	0.061	-	No	1	0.071	0.028	0.012	-	0.061
SDHB	CAD	1	0.282	0.095	0.003	1.326	0.024	-	Yes	1	0.282	0.095	0.003	-	0.024
SERPINI1	CAD	6	0.015	0.014	0.307	1.015	0.591	0.995	No	6	0.015	0.014	0.307	0.932	0.591
SH2B3	CAD	1	0.443	0.074	0.000	1.557	0.000	-	Yes	1	0.443	0.074	0.000	-	0.000
SHMT1	CAD	4	0.005	0.013	0.717	1.005	0.894	0.163	No	4	0.005	0.013	0.717	0.040	0.894
SLC16A1	CAD	1	-0.245	0.096	0.011	0.783	0.059	-	No	1	-0.245	0.096	0.011	-	0.059
SLC9A3R2	CAD	1	0.143	0.058	0.014	1.154	0.068	-	No	1	0.143	0.058	0.014	-	0.068
SMTN	CAD	1	-0.052	0.147	0.723	0.949	0.894	-	No	1	-0.052	0.147	0.723	-	0.894

ST6: MR causal estimates for DBP-associated proteins on CAD.

Causal candidates prioritized for CAD were marked as "Yes" in column "Prioritized". All estimates are from inverse variance weighted method (nsnp >1) or Wald-ratio method (nsnp = 1). CAD, coronary artery disease.

										Steiger filtering					
**Exposure**	**Outocme**	**nsnp**	**Beta**	**SE**	**P-value**	**Odds ratio**	**FDR-corrected P-value**	**FDR-corrected Cochran's Q P-value**	**Prioritized**	**nsnp**	**Beta**	**SE**	**P-value**	**Cochran's Q P-value**	**FDR-corrected P-value**
SPINK8	CAD	1	0.067	0.012	0.000	1.070	0.000	-	Yes	1	0.067	0.012	0.000	-	0.000
STC1	CAD	1	-0.034	0.103	0.741	0.966	0.901	-	No	1	-0.034	0.103	0.741	-	0.901
STX4	CAD	1	0.060	0.142	0.672	1.062	0.867	-	No	1	0.060	0.142	0.672	-	0.867
TBC1D17	CAD	2	0.009	0.017	0.606	1.009	0.823	-	No	2	0.009	0.017	0.606	-	0.823
TIE1	CAD	1	-0.114	0.030	0.000	0.892	0.002	-	Yes	1	-0.114	0.030	0.000	-	0.002
TJAP1	CAD	1	-0.399	0.142	0.005	0.671	0.034	-	Yes	1	-0.399	0.142	0.005	-	0.034
TMEM106A	CAD	1	-0.159	0.058	0.006	0.853	0.038	-	Yes	1	-0.159	0.058	0.006	-	0.038
TNFRSF17	CAD	1	-0.070	0.043	0.106	0.932	0.322	-	No	1	-0.070	0.043	0.106	-	0.322
TNFSF12	CAD	2	-0.021	0.025	0.392	0.979	0.707	-	No	2	-0.021	0.025	0.392	-	0.707
TP53	CAD	1	0.085	0.101	0.399	1.089	0.714	-	No	1	0.085	0.101	0.399	-	0.714
UMOD	CAD	10	-0.002	0.007	0.806	0.998	0.921	0.819	No	10	-0.002	0.007	0.806	0.546	0.921
UXS1	CAD	2	0.034	0.051	0.500	1.035	0.782	-	No	2	0.034	0.051	0.500	-	0.782
VAT1	CAD	1	-0.112	0.041	0.006	0.894	0.036	-	Yes	1	-0.112	0.041	0.006	-	0.036
VSIG2	CAD	1	-0.023	0.032	0.476	0.978	0.782	-	No	1	-0.023	0.032	0.476	-	0.782
WNT9A	CAD	2	0.004	0.092	0.966	1.004	0.999	-	No	2	0.004	0.092	0.966	-	0.999
WWP2	CAD	1	-0.045	0.038	0.234	0.956	0.531	-	No	1	-0.045	0.038	0.234	-	0.531
YAP1	CAD	1	-0.115	0.089	0.198	0.891	0.486	-	No	1	-0.115	0.089	0.198	-	0.486
YOD1	CAD	1	0.023	0.053	0.661	1.023	0.858	-	No	1	0.023	0.053	0.661	-	0.858
ZBTB17	CAD	1	0.273	0.128	0.034	1.313	0.136	-	No	1	0.273	0.128	0.034	-	0.136

nsnp = number of single nucleotide polymorphisms; SE = standard error; Cochran's Q P-value = p-value from Cochran's Q test assessing heterogeneity; FDR=false discovery rate

ST7; MR causal estimates for SBP-associated proteins on all stroke.

Causal candidates prioritized for AS were marked as "Yes" in column "Prioritized". All estimates are from inverse variance weighted method (IVs >1) or Wald-ratio method (IV = 1). AS, all strokes.

										Steiger filtering					
**Exposure**	**Outocme**	**nsnp**	**Beta**	**SE**	**P-value**	**Odds ratio**	**FDR-corrected P-value**	**FDR-corrected Cochran's Q P-value**	**Prioritized**	**nsnp**	**Beta**	**SE**	**P-value**	**Cochran's Q P-value**	**FDR-corrected P-value**
ACOX1	AS	1	-0.006	0.096	0.946	0.994	0.976	-	No	1	-0.006	0.096	0.946	-	0.976
ACRBP	AS	1	0.028	0.047	0.561	1.028	0.902	-	No	1	0.028	0.047	0.561	-	0.902
ADAM23	AS	4	0.010	0.011	0.347	1.010	0.876	0.874	No	4	0.010	0.011	0.347	0.531	0.876
ADAMTS1	AS	1	0.049	0.164	0.762	1.051	0.923	-	No	1	0.049	0.164	0.762	-	0.923
ADAMTS4	AS	1	-0.021	0.055	0.705	0.979	0.902	-	No	1	-0.021	0.055	0.705	-	0.902
ADAMTS8	AS	5	0.000	0.014	0.992	1.000	0.996	0.751	No	5	0.000	0.014	0.992	0.424	0.996
ADAMTSL5	AS	2	-0.004	0.050	0.938	0.996	0.972	-	No	2	-0.004	0.050	0.938	-	0.972
ADM	AS	1	-0.079	0.181	0.662	0.924	0.902	-	No	1	-0.079	0.181	0.662	-	0.902
AMOTL2	AS	1	-0.021	0.135	0.877	0.979	0.969	-	No	1	-0.021	0.135	0.877	-	0.969
ANKMY2	AS	1	0.010	0.093	0.918	1.010	0.969	-	No	1	0.010	0.093	0.918	-	0.969
APOA1	AS	1	0.023	0.109	0.835	1.023	0.947	-	No	1	0.023	0.109	0.835	-	0.947
APOA2	AS	1	-0.094	0.106	0.377	0.910	0.876	-	No	1	-0.094	0.106	0.377	-	0.876
APOBR	AS	3	0.001	0.006	0.914	1.001	0.969	0.977	No	3	0.001	0.006	0.914	0.977	0.969
APOC1	AS	2	-0.070	0.081	0.391	0.933	0.876	-	No	2	-0.070	0.081	0.391	-	0.876
ASPN	AS	2	0.048	0.029	0.095	1.049	0.485	-	No	2	0.048	0.029	0.095	-	0.485
ATXN2L	AS	1	-0.088	0.179	0.622	0.916	0.902	-	No	1	-0.088	0.179	0.622	-	0.902
B4GAT1	AS	3	0.020	0.025	0.417	1.020	0.876	0.749	No	3	0.020	0.025	0.417	0.403	0.876
BAG4	AS	1	-0.407	0.181	0.024	0.665	0.205	-	No	1	-0.407	0.181	0.024	-	0.205
BCAM	AS	5	0.017	0.045	0.702	1.018	0.902	0.343	No	5	0.017	0.045	0.702	0.034	0.902
BMP6	AS	3	-0.014	0.043	0.742	0.986	0.919	0.913	No	3	-0.014	0.043	0.742	0.632	0.919
BNIP3L	AS	1	-0.023	0.135	0.862	0.977	0.958	-	No	1	-0.023	0.135	0.862	-	0.958
BRAP	AS	1	1.321	0.187	0.000	3.748	0.000	-	Yes	1	1.321	0.187	0.000	-	0.000
BRSK2	AS	1	-0.291	0.135	0.031	0.748	0.247	-	No	1	-0.291	0.135	0.031	-	0.247
CA12	AS	2	-0.004	0.023	0.860	0.996	0.958	-	No	2	-0.004	0.023	0.860	-	0.958
CA9	AS	2	0.022	0.069	0.751	1.022	0.919	-	No	2	0.022	0.069	0.751	-	0.919
CACNB3	AS	1	-0.043	0.068	0.526	0.958	0.902	-	No	1	-0.043	0.068	0.526	-	0.902
CALCA	AS	1	-0.065	0.064	0.310	0.937	0.866	-	No	1	-0.065	0.064	0.310	-	0.866
CALCOCO2	AS	1	0.276	0.118	0.020	1.318	0.195	-	No	1	0.276	0.118	0.020	-	0.195
CCN3	AS	3	-0.004	0.034	0.905	0.996	0.969	0.543	No	3	-0.004	0.034	0.905	0.197	0.969
CD14	AS	1	-0.050	0.023	0.034	0.952	0.256	-	No	1	-0.050	0.023	0.034	-	0.256
CD164L2	AS	1	0.022	0.015	0.128	1.023	0.611	-	No	1	0.022	0.015	0.128	-	0.611
CD46	AS	1	-0.066	0.084	0.431	0.936	0.876	-	No	1	-0.066	0.084	0.431	-	0.876
CD59	AS	2	-0.036	0.030	0.220	0.964	0.811	-	No	2	-0.036	0.030	0.220	-	0.811
CEP170	AS	1	-0.070	0.106	0.505	0.932	0.896	-	No	1	-0.070	0.106	0.505	-	0.896
CERT	AS	1	-0.107	0.144	0.456	0.898	0.884	-	No	1	-0.107	0.144	0.456	-	0.884
CETN3	AS	1	-0.074	0.052	0.159	0.929	0.662	-	No	1	-0.074	0.052	0.159	-	0.662
CFHR2	AS	5	-0.002	0.007	0.825	0.998	0.947	0.722	No	5	-0.002	0.007	0.825	0.370	0.947
CFHR4	AS	7	0.008	0.011	0.467	1.008	0.892	0.543	No	7	0.008	0.011	0.467	0.187	0.892
CLMP	AS	2	-0.013	0.024	0.572	0.987	0.902	-	No	2	-0.013	0.024	0.572	-	0.902
COL1A1	AS	1	-0.103	0.158	0.516	0.902	0.902	-	No	1	-0.103	0.158	0.516	-	0.902
COMP	AS	1	0.046	0.107	0.666	1.047	0.902	-	No	1	0.046	0.107	0.666	-	0.902
COMT	AS	2	-0.057	0.031	0.063	0.944	0.372	-	No	2	-0.057	0.031	0.063	-	0.372
CPXM1	AS	1	0.024	0.022	0.283	1.024	0.830	-	No	1	0.024	0.022	0.283	-	0.830
CTSO	AS	1	-0.077	0.037	0.036	0.926	0.256	-	No	1	-0.077	0.037	0.036	-	0.256
DAG1	AS	1	-0.066	0.109	0.543	0.936	0.902	-	No	1	-0.066	0.109	0.543	-	0.902
DDHD2	AS	1	-0.112	0.068	0.103	0.894	0.501	-	No	1	-0.112	0.068	0.103	-	0.501
DTX3	AS	1	0.046	0.074	0.531	1.047	0.902	-	No	1	0.046	0.074	0.531	-	0.902
DUSP13	AS	1	-0.019	0.025	0.440	0.981	0.876	-	No	1	-0.019	0.025	0.440	-	0.876
DUSP29	AS	1	-0.002	0.052	0.977	0.998	0.990	-	No	1	-0.002	0.052	0.977	-	0.990
EDN1	AS	1	0.024	0.037	0.523	1.024	0.902	-	No	1	0.024	0.037	0.523	-	0.902
EFEMP1	AS	2	-0.028	0.055	0.608	0.972	0.902	-	No	2	-0.028	0.055	0.608	-	0.902
EIF4G3	AS	1	0.097	0.088	0.269	1.102	0.830	-	No	1	0.097	0.088	0.269	-	0.830
ELOA	AS	1	0.162	0.087	0.063	1.175	0.372	-	No	1	0.162	0.087	0.063	-	0.372
ENPEP	AS	1	-0.558	0.195	0.004	0.573	0.058	-	No	1	-0.558	0.195	0.004	-	0.058
ERI1	AS	1	0.091	0.097	0.347	1.095	0.876	-	No	1	0.091	0.097	0.347	-	0.876
ERP29	AS	1	1.417	0.201	0.000	4.124	0.000	-	Yes	1	1.417	0.201	0.000	-	0.000

ST7; MR causal estimates for SBP-associated proteins on all stroke.

Causal candidates prioritized for AS were marked as "Yes" in column "Prioritized". All estimates are from inverse variance weighted method (IVs >1) or Wald-ratio method (IV = 1). AS, all strokes.

										Steiger filtering					
Exposure	Outocme	nsnp	Beta	SE	P-value	Odds ratio	FDR-corrected P-value	FDR-corrected Cochran's Q P-value	Prioritized	nsnp	Beta	SE	P-value	Cochran's Q P-value	FDR-corrected P-value
ESAM	AS	1	-0.052	0.054	0.330	0.949	0.876	-	No	1	-0.052	0.054	0.330	-	0.876
F13B	AS	1	-0.010	0.019	0.600	0.990	0.902	-	No	1	-0.010	0.019	0.600	-	0.902
FDX1	AS	1	-0.038	0.142	0.789	0.963	0.938	-	No	1	-0.038	0.142	0.789	-	0.938
FES	AS	1	-0.214	0.052	0.000	0.808	0.001	-	Yes	1	-0.214	0.052	0.000	-	0.001
FGF2	AS	3	0.021	0.016	0.175	1.022	0.716	0.543	No	3	0.021	0.016	0.175	0.203	0.716
FGF5	AS	4	0.036	0.010	0.001	1.037	0.013	0.931	Yes	4	0.036	0.010	0.001	0.698	0.013
FKBP7	AS	1	0.244	0.095	0.011	1.276	0.115	-	No	1	0.244	0.095	0.011	-	0.115
FN1	AS	2	-0.051	0.023	0.024	0.950	0.205	-	No	2	-0.051	0.023	0.024	-	0.205
FOXJ3	AS	1	0.114	0.100	0.256	1.120	0.830	-	No	1	0.114	0.100	0.256	-	0.830
FOXO3	AS	1	0.272	0.186	0.142	1.313	0.639	-	No	1	0.272	0.186	0.142	-	0.639
FUCA1	AS	1	0.024	0.018	0.194	1.024	0.764	-	No	1	0.024	0.018	0.194	-	0.764
FURIN	AS	1	0.179	0.037	0.000	1.196	0.000	-	Yes	1	0.179	0.037	0.000	-	0.000
GCHFR	AS	1	0.054	0.061	0.378	1.055	0.876	-	No	1	0.054	0.061	0.378	-	0.876
GFER	AS	1	-0.116	0.110	0.292	0.891	0.836	-	No	1	-0.116	0.110	0.292	-	0.836
GHR	AS	4	0.019	0.016	0.247	1.019	0.830	0.515	No	4	0.019	0.016	0.247	0.066	0.830
GIMAP7	AS	3	0.005	0.012	0.678	1.005	0.902	0.931	No	3	0.005	0.012	0.678	0.790	0.902
GIT1	AS	1	0.137	0.151	0.363	1.147	0.876	-	No	1	0.137	0.151	0.363	-	0.876
GORASP2	AS	1	-0.032	0.136	0.813	0.969	0.946	-	No	1	-0.032	0.136	0.813	-	0.946
GRP	AS	4	0.017	0.025	0.502	1.017	0.896	0.543	No	4	0.017	0.025	0.502	0.140	0.896
HADH	AS	1	0.103	0.132	0.438	1.108	0.876	-	No	1	0.103	0.132	0.438	-	0.876
HHEX	AS	1	-0.146	0.179	0.417	0.865	0.876	-	No	1	-0.146	0.179	0.417	-	0.876
HMOX2	AS	1	-0.031	0.091	0.731	0.969	0.919	-	No	1	-0.031	0.091	0.731	-	0.919
HYAL1	AS	2	-0.028	0.026	0.280	0.972	0.830	-	No	2	-0.028	0.026	0.280	-	0.830
ICAM2	AS	1	0.101	0.132	0.444	1.106	0.876	-	No	1	0.101	0.132	0.444	-	0.876
IDUA	AS	4	-0.002	0.019	0.918	0.998	0.969	0.543	No	4	-0.002	0.019	0.918	0.098	0.969
IFI30	AS	2	-0.028	0.016	0.091	0.973	0.473	-	No	2	-0.028	0.016	0.091	-	0.473
IFNGR2	AS	5	0.005	0.007	0.408	1.005	0.876	0.931	No	5	0.005	0.007	0.408	0.836	0.876
IGFBP3	AS	3	-0.045	0.019	0.021	0.956	0.200	0.931	No	3	-0.045	0.019	0.021	0.820	0.200
IMMT	AS	1	-0.032	0.057	0.569	0.968	0.902	-	No	1	-0.032	0.057	0.569	-	0.902
IMPA1	AS	1	0.066	0.034	0.055	1.068	0.342	-	No	1	0.066	0.034	0.055	-	0.342
ITGAL	AS	1	0.082	0.210	0.697	1.085	0.902	-	No	1	0.082	0.210	0.697	-	0.902
ITIH1	AS	1	0.042	0.049	0.388	1.043	0.876	-	No	1	0.042	0.049	0.388	-	0.876
KIFBP	AS	1	-0.119	0.149	0.427	0.888	0.876	-	No	1	-0.119	0.149	0.427	-	0.876
LMOD1	AS	1	-0.039	0.040	0.331	0.962	0.876	-	No	1	-0.039	0.040	0.331	-	0.876
LRIG1	AS	3	-0.018	0.009	0.041	0.982	0.286	0.681	No	3	-0.018	0.009	0.041	0.332	0.286
LYAR	AS	1	-0.066	0.155	0.668	0.936	0.902	-	No	1	-0.066	0.155	0.668	-	0.902
M6PR	AS	1	0.014	0.036	0.693	1.014	0.902	-	No	1	0.014	0.036	0.693	-	0.902
MANEAL	AS	1	-0.098	0.182	0.589	0.906	0.902	-	No	1	-0.098	0.182	0.589	-	0.902
MANSC4	AS	2	-0.006	0.010	0.539	0.994	0.902	-	No	2	-0.006	0.010	0.539	-	0.902
MAP4K5	AS	1	0.031	0.039	0.416	1.032	0.876	-	No	1	0.031	0.039	0.416	-	0.876
MDH1	AS	2	-0.007	0.084	0.937	0.993	0.972	-	No	2	-0.007	0.084	0.937	-	0.972
MEGF9	AS	1	-0.052	0.023	0.025	0.950	0.205	-	No	1	-0.052	0.023	0.025	-	0.205
MPHOSPH8	AS	1	0.003	0.157	0.983	1.003	0.992	-	No	1	0.003	0.157	0.983	-	0.992
MPI	AS	2	0.003	0.066	0.960	1.003	0.982	-	No	2	0.003	0.066	0.960	-	0.982
MSRA	AS	1	-0.014	0.129	0.912	0.986	0.969	-	No	1	-0.014	0.129	0.912	-	0.969
MST1	AS	2	0.003	0.013	0.803	1.003	0.946	-	No	2	0.003	0.013	0.803	-	0.946
MVK	AS	1	-0.055	0.080	0.489	0.946	0.896	-	No	1	-0.055	0.080	0.489	-	0.896
NADK	AS	1	-0.033	0.027	0.228	0.968	0.820	-	No	1	-0.033	0.027	0.228	-	0.820
NAGA	AS	2	-0.032	0.047	0.495	0.968	0.896	-	No	2	-0.032	0.047	0.495	-	0.896
NFE2	AS	1	-0.342	0.100	0.001	0.711	0.013	-	Yes	1	-0.342	0.100	0.001	-	0.013
NFU1	AS	1	0.006	0.067	0.934	1.006	0.972	-	No	1	0.006	0.067	0.934	-	0.972
NGF	AS	1	0.025	0.228	0.912	1.026	0.969	-	No	1	0.025	0.228	0.912	-	0.969
NOTCH3	AS	1	0.244	0.138	0.077	1.276	0.427	-	No	1	0.244	0.138	0.077	-	0.427
NPPB	AS	1	-0.023	0.045	0.604	0.977	0.902	-	No	1	-0.023	0.045	0.604	-	0.902
NTRK3	AS	2	-0.048	0.032	0.138	0.953	0.639	-	No	2	-0.048	0.032	0.138	-	0.639
NUCB2	AS	2	-0.029	0.049	0.555	0.972	0.902	-	No	2	-0.029	0.049	0.555	-	0.902

ST7; MR causal estimates for SBP-associated proteins on all stroke.

Causal candidates prioritized for AS were marked as "Yes" in column "Prioritized". All estimates are from inverse variance weighted method (IVs >1) or Wald-ratio method (IV = 1). AS, all strokes.

										Steiger filtering					
Exposure	Outocme	nsnp	Beta	SE	P-value	Odds ratio	FDR-corrected P-value	FDR-corrected Cochran's Q P-value	Prioritized	nsnp	Beta	SE	P-value	Cochran's Q P-value	FDR-corrected P-value
NUDT5	AS	1	-0.102	0.118	0.388	0.903	0.876	-	No	1	-0.102	0.118	0.388	-	0.876
NUMB	AS	1	0.045	0.076	0.559	1.046	0.902	-	No	1	0.045	0.076	0.559	-	0.902
OGA	AS	1	-0.006	0.065	0.931	0.994	0.972	-	No	1	-0.006	0.065	0.931	-	0.972
OPLAH	AS	1	-0.050	0.037	0.179	0.952	0.720	-	No	1	-0.050	0.037	0.179	-	0.720
PCBP2	AS	1	-0.467	0.136	0.001	0.627	0.013	-	Yes	1	-0.467	0.136	0.001	-	0.013
PCSK7	AS	2	0.020	0.021	0.354	1.020	0.876	-	No	2	0.020	0.021	0.354	-	0.876
PDE5A	AS	2	-0.075	0.042	0.073	0.928	0.420	-	No	2	-0.075	0.042	0.073	-	0.420
PDGFRA	AS	2	0.012	0.029	0.677	1.012	0.902	-	No	2	0.012	0.029	0.677	-	0.902
PECAM1	AS	1	-0.245	0.210	0.245	0.783	0.830	-	No	1	-0.245	0.210	0.245	-	0.830
PFKFB2	AS	1	0.022	0.036	0.550	1.022	0.902	-	No	1	0.022	0.036	0.550	-	0.902
PGF	AS	1	0.015	0.044	0.735	1.015	0.919	-	No	1	0.015	0.044	0.735	-	0.919
PHLDB1	AS	1	-0.052	0.153	0.735	0.949	0.919	-	No	1	-0.052	0.153	0.735	-	0.919
PKD1	AS	1	-0.214	0.152	0.159	0.807	0.662	-	No	1	-0.214	0.152	0.159	-	0.662
PLA2G1B	AS	1	-0.107	0.132	0.417	0.898	0.876	-	No	1	-0.107	0.132	0.417	-	0.876
PMS1	AS	1	0.028	0.144	0.843	1.029	0.947	-	No	1	0.028	0.144	0.843	-	0.947
PPP1R14D	AS	1	0.136	0.122	0.264	1.146	0.830	-	No	1	0.136	0.122	0.264	-	0.830
PRG2	AS	1	0.036	0.070	0.607	1.037	0.902	-	No	1	0.036	0.070	0.607	-	0.902
PRKAB1	AS	1	0.009	0.038	0.818	1.009	0.946	-	No	1	0.009	0.038	0.818	-	0.946
PSMD5	AS	1	0.433	0.150	0.004	1.543	0.055	-	No	1	0.433	0.150	0.004	-	0.055
PSRC1	AS	2	0.055	0.034	0.102	1.057	0.501	-	No	2	0.055	0.034	0.102	-	0.501
PTRHD1	AS	1	-0.160	0.178	0.368	0.852	0.876	-	No	1	-0.160	0.178	0.368	-	0.876
QPCT	AS	3	0.034	0.047	0.464	1.035	0.892	0.025	No	3	0.034	0.047	0.464	0.001	0.892
RABEPK	AS	1	0.119	0.045	0.008	1.127	0.100	-	No	1	0.119	0.045	0.008	-	0.100
RARRES1	AS	5	0.018	0.016	0.238	1.019	0.830	0.599	No	5	0.018	0.016	0.238	0.276	0.830
RARRES2	AS	1	0.013	0.034	0.705	1.013	0.902	-	No	1	0.013	0.034	0.705	-	0.902
RELT	AS	2	0.015	0.021	0.498	1.015	0.896	-	No	2	0.015	0.021	0.498	-	0.896
RSPO3	AS	1	0.005	0.042	0.901	1.005	0.969	-	No	1	0.005	0.042	0.901	-	0.969
SCARA5	AS	4	-0.069	0.023	0.003	0.934	0.042	0.592	Yes	4	-0.069	0.023	0.003	0.258	0.042
SDCCAG8	AS	1	0.016	0.033	0.633	1.016	0.902	-	No	1	0.016	0.033	0.633	-	0.902
SDHB	AS	1	0.043	0.113	0.702	1.044	0.902	-	No	1	0.043	0.113	0.702	-	0.902
SELENOP	AS	1	0.049	0.066	0.451	1.051	0.883	-	No	1	0.049	0.066	0.451	-	0.883
SEMA6C	AS	1	0.111	0.077	0.153	1.117	0.662	-	No	1	0.111	0.077	0.153	-	0.662
SERPINI1	AS	3	-0.020	0.017	0.246	0.980	0.830	0.931	No	3	-0.020	0.017	0.246	0.717	0.830
SH2B3	AS	1	0.454	0.095	0.000	1.574	0.000	-	Yes	1	0.454	0.095	0.000	-	0.000
SHMT1	AS	4	0.003	0.010	0.754	1.003	0.919	0.913	No	4	0.003	0.010	0.754	0.611	0.919
SIL1	AS	1	0.143	0.113	0.206	1.153	0.799	-	No	1	0.143	0.113	0.206	-	0.799
SLC16A1	AS	1	-0.123	0.110	0.261	0.884	0.830	-	No	1	-0.123	0.110	0.261	-	0.830
SLC39A14	AS	1	-0.065	0.081	0.420	0.937	0.876	-	No	1	-0.065	0.081	0.420	-	0.876
SLC9A3R2	AS	1	0.031	0.068	0.652	1.031	0.902	-	No	1	0.031	0.068	0.652	-	0.902
SMOC2	AS	4	-0.001	0.018	0.970	0.999	0.987	0.960	No	4	-0.001	0.018	0.970	0.935	0.987
SOST	AS	1	0.016	0.119	0.896	1.016	0.969	-	No	1	0.016	0.119	0.896	-	0.969
SPINK8	AS	1	0.019	0.016	0.229	1.019	0.820	-	No	1	0.019	0.016	0.229	-	0.820
SPRED2	AS	1	-0.041	0.104	0.694	0.960	0.902	-	No	1	-0.041	0.104	0.694	-	0.902
TARBP2	AS	1	-0.502	0.147	0.001	0.605	0.013	-	Yes	1	-0.502	0.147	0.001	-	0.013
TBC1D23	AS	1	-0.030	0.043	0.479	0.970	0.896	-	No	1	-0.030	0.043	0.479	-	0.896
TEK	AS	5	0.026	0.024	0.292	1.026	0.836	0.343	No	5	0.026	0.024	0.292	0.035	0.836
TGFB2	AS	1	0.113	0.048	0.018	1.119	0.190	-	No	1	0.113	0.048	0.018	-	0.190
TIE1	AS	1	0.030	0.036	0.407	1.030	0.876	-	No	1	0.030	0.036	0.407	-	0.876
TJAP1	AS	1	-0.527	0.163	0.001	0.590	0.023	-	Yes	1	-0.527	0.163	0.001	-	0.023
TNFRSF17	AS	1	0.000	0.050	1.000	1.000	1.000	-	No	1	0.000	0.050	1.000	-	1.000
TNFSF12	AS	2	0.018	0.019	0.339	1.018	0.876	-	No	2	0.018	0.019	0.339	-	0.876
TNFSF13B	AS	4	-0.030	0.060	0.617	0.970	0.902	0.945	No	4	-0.030	0.060	0.617	0.886	0.902
TWF2	AS	1	-0.025	0.105	0.811	0.975	0.946	-	No	1	-0.025	0.105	0.811	-	0.946
UBE2L6	AS	2	0.025	0.028	0.370	1.025	0.876	-	No	2	0.025	0.028	0.370	-	0.876
UMOD	AS	6	0.006	0.010	0.547	1.006	0.902	0.543	No	6	0.006	0.010	0.547	0.216	0.902
VSIG2	AS	1	0.035	0.036	0.330	1.036	0.876	-	No	1	0.035	0.036	0.330	-	0.876

ST7; MR causal estimates for SBP-associated proteins on all stroke.

Causal candidates prioritized for AS were marked as "Yes" in column "Prioritized". All estimates are from inverse variance weighted method (IVs >1) or Wald-ratio method (IV = 1). AS, all strokes.

										Steiger filtering					
**Exposure**	**Outocme**	**nsnp**	**Beta**	**SE**	**P-value**	**Odds ratio**	**FDR-corrected P-value**	**FDR-corrected Cochran's Q P-value**	**Prioritized**	**nsnp**	**Beta**	**SE**	**P-value**	**Cochran's Q P-value**	**FDR-corrected P-value**
WARS	AS	1	0.109	0.034	0.002	1.115	0.027	-	Yes	1	0.109	0.034	0.002	-	0.027
WASHC3	AS	1	0.149	0.085	0.080	1.161	0.427	-	No	1	0.149	0.085	0.080	-	0.427
WWP2	AS	1	0.039	0.043	0.362	1.040	0.876	-	No	1	0.039	0.043	0.362	-	0.876
YOD1	AS	1	0.067	0.061	0.275	1.069	0.830	-	No	1	0.067	0.061	0.275	-	0.830
ZBTB17	AS	1	-0.385	0.183	0.036	0.681	0.256	-	No	1	-0.385	0.183	0.036	-	0.256
ZFYVE19	AS	1	-0.020	0.018	0.276	0.980	0.830	-	No	1	-0.020	0.018	0.276	-	0.830

nsnp = number of single nucleotide polymorphisms; SE = standard error; Cochran's Q P-value = p-value from Cochran's Q test assessing heterogeneity; FDR=false discovery rate

ST8; MR causal estimates for DBP-associated proteins on all stroke.

Causal candidates prioritized for AS were marked as "Yes" in column "Prioritized". All estimates are from inverse variance weighted method (IVs >1) or Wald-ratio method (IV = 1). AS, all strokes.

										Steiger filtering					
**Exposure**	**Outocme**	**nsnp**	**Beta**	**SE**	**P-value**	**Odds ratio**	**FDR-corrected P-value**	**FDR-corrected Cochran's Q P-value**	**Prioritized**	**nsnp**	**Beta**	**SE**	**P-value**	**Cochran's Q P-value**	**FDR-corrected P-value**
AAMDC	AS	2	-0.008	0.009	0.411	0.992	0.876	-	No	2	-0.008	0.009	0.411	-	0.876
ABO	AS	7	0.032	0.008	0.000	1.033	0.001	0.543	Yes	7	0.032	0.008	0.000	0.138	0.001
ACOX1	AS	1	-0.006	0.096	0.946	0.994	0.976	-	No	1	-0.006	0.096	0.946	-	0.976
ADM	AS	1	-0.079	0.181	0.662	0.924	0.902	-	No	1	-0.079	0.181	0.662	-	0.902
AMFR	AS	1	0.236	0.161	0.142	1.266	0.639	-	No	1	0.236	0.161	0.142	-	0.639
AMOTL2	AS	1	-0.021	0.135	0.877	0.979	0.969	-	No	1	-0.021	0.135	0.877	-	0.969
ANKMY2	AS	1	0.010	0.093	0.918	1.010	0.969	-	No	1	0.010	0.093	0.918	-	0.969
AOC1	AS	3	-0.046	0.023	0.048	0.955	0.317	0.931	No	3	-0.046	0.023	0.048	0.767	0.317
AOC3	AS	2	-0.003	0.014	0.828	0.997	0.947	-	No	2	-0.003	0.014	0.828	-	0.947
APOA1	AS	1	0.023	0.109	0.835	1.023	0.947	-	No	1	0.023	0.109	0.835	-	0.947
APOBR	AS	3	0.001	0.006	0.914	1.001	0.969	0.977	No	3	0.001	0.006	0.914	0.977	0.969
APOC1	AS	2	-0.070	0.081	0.391	0.933	0.876	-	No	2	-0.070	0.081	0.391	-	0.876
ARSB	AS	3	-0.009	0.023	0.695	0.991	0.902	0.913	No	3	-0.009	0.023	0.695	0.608	0.902
ATXN2L	AS	1	-0.088	0.179	0.622	0.916	0.902	-	No	1	-0.088	0.179	0.622	-	0.902
AXL	AS	2	0.017	0.054	0.748	1.018	0.919	-	No	2	0.017	0.054	0.748	-	0.919
BAG4	AS	1	-0.407	0.181	0.024	0.665	0.205	-	No	1	-0.407	0.181	0.024	-	0.205
BNIP3L	AS	1	-0.023	0.135	0.862	0.977	0.958	-	No	1	-0.023	0.135	0.862	-	0.958
CA12	AS	2	-0.004	0.023	0.860	0.996	0.958	-	No	2	-0.004	0.023	0.860	-	0.958
CACNB3	AS	1	-0.043	0.068	0.526	0.958	0.902	-	No	1	-0.043	0.068	0.526	-	0.902
CALCA	AS	1	-0.065	0.064	0.310	0.937	0.866	-	No	1	-0.065	0.064	0.310	-	0.866
CEP170	AS	1	-0.070	0.106	0.505	0.932	0.896	-	No	1	-0.070	0.106	0.505	-	0.896
CFHR4	AS	7	0.008	0.011	0.467	1.008	0.892	0.543	No	7	0.008	0.011	0.467	0.187	0.892
CHMP1A	AS	1	0.069	0.075	0.354	1.072	0.876	-	No	1	0.069	0.075	0.354	-	0.876
CLIC5	AS	1	0.024	0.054	0.654	1.025	0.902	-	No	1	0.024	0.054	0.654	-	0.902
COL1A1	AS	1	-0.103	0.158	0.516	0.902	0.902	-	No	1	-0.103	0.158	0.516	-	0.902
CTF1	AS	1	-0.269	0.139	0.052	0.764	0.331	-	No	1	-0.269	0.139	0.052	-	0.331
DAG1	AS	1	-0.066	0.109	0.543	0.936	0.902	-	No	1	-0.066	0.109	0.543	-	0.902
DARS1	AS	1	-0.040	0.099	0.685	0.960	0.902	-	No	1	-0.040	0.099	0.685	-	0.902
DBN1	AS	1	0.059	0.132	0.654	1.061	0.902	-	No	1	0.059	0.132	0.654	-	0.902
DDHD2	AS	1	-0.112	0.068	0.103	0.894	0.501	-	No	1	-0.112	0.068	0.103	-	0.501
DENR	AS	1	-0.029	0.199	0.883	0.971	0.969	-	No	1	-0.029	0.199	0.883	-	0.969
DNER	AS	5	-0.016	0.020	0.439	0.985	0.876	0.945	No	5	-0.016	0.020	0.439	0.896	0.876
DOK2	AS	1	-0.123	0.120	0.303	0.884	0.856	-	No	1	-0.123	0.120	0.303	-	0.856
DPEP1	AS	4	-0.014	0.052	0.791	0.986	0.938	0.543	No	4	-0.014	0.052	0.791	0.223	0.938
DPP4	AS	2	-0.019	0.032	0.539	0.981	0.902	-	No	2	-0.019	0.032	0.539	-	0.902
DUSP13	AS	1	-0.019	0.025	0.440	0.981	0.876	-	No	1	-0.019	0.025	0.440	-	0.876
EFNA1	AS	1	-0.006	0.021	0.755	0.994	0.919	-	No	1	-0.006	0.021	0.755	-	0.919
EIF4G3	AS	1	0.097	0.088	0.269	1.102	0.830	-	No	1	0.097	0.088	0.269	-	0.830
ELOA	AS	1	0.162	0.087	0.063	1.175	0.372	-	No	1	0.162	0.087	0.063	-	0.372
ENPEP	AS	1	-0.558	0.195	0.004	0.573	0.058	-	No	1	-0.558	0.195	0.004	-	0.058
EPHA2	AS	2	-0.025	0.066	0.699	0.975	0.902	-	No	2	-0.025	0.066	0.699	-	0.902
EPO	AS	1	-0.131	0.092	0.157	0.878	0.662	-	No	1	-0.131	0.092	0.157	-	0.662
ERI1	AS	1	0.091	0.097	0.347	1.095	0.876	-	No	1	0.091	0.097	0.347	-	0.876
ESAM	AS	1	-0.052	0.054	0.330	0.949	0.876	-	No	1	-0.052	0.054	0.330	-	0.876
F12	AS	5	-0.013	0.010	0.213	0.987	0.811	0.543	No	5	-0.013	0.010	0.213	0.215	0.811
FADD	AS	1	-0.040	0.084	0.633	0.961	0.902	-	No	1	-0.040	0.084	0.633	-	0.902
FDX1	AS	1	-0.038	0.142	0.789	0.963	0.938	-	No	1	-0.038	0.142	0.789	-	0.938
FES	AS	1	-0.214	0.052	0.000	0.808	0.001	-	Yes	1	-0.214	0.052	0.000	-	0.001
FGF5	AS	4	0.036	0.010	0.001	1.037	0.013	0.931	Yes	4	0.036	0.010	0.001	0.698	0.013
FOXJ3	AS	1	0.114	0.100	0.256	1.120	0.830	-	No	1	0.114	0.100	0.256	-	0.830
FURIN	AS	1	0.179	0.037	0.000	1.196	0.000	-	Yes	1	0.179	0.037	0.000	-	0.000
GFER	AS	1	-0.116	0.110	0.292	0.891	0.836	-	No	1	-0.116	0.110	0.292	-	0.836
GIMAP7	AS	3	0.005	0.012	0.678	1.005	0.902	0.931	No	3	0.005	0.012	0.678	0.790	0.902
GLO1	AS	2	0.007	0.024	0.766	1.007	0.923	-	No	2	0.007	0.024	0.766	-	0.923
GORASP2	AS	1	-0.032	0.136	0.813	0.969	0.946	-	No	1	-0.032	0.136	0.813	-	0.946
GRP	AS	4	0.017	0.025	0.502	1.017	0.896	0.543	No	4	0.017	0.025	0.502	0.140	0.896

ST8; MR causal estimates for DBP-associated proteins on all stroke.

Causal candidates prioritized for AS were marked as "Yes" in column "Prioritized". All estimates are from inverse variance weighted method (IVs >1) or Wald-ratio method (IV = 1). AS, all strokes.

										Steiger filtering					
Exposure	Outocme	nsnp	Beta	SE	P-value	Odds ratio	FDR-corrected P-value	FDR-corrected Cochran's Q P-value	Prioritized	nsnp	Beta	SE	P-value	Cochran's Q P-value	FDR-corrected P-value
HADH	AS	1	0.103	0.132	0.438	1.108	0.876	-	No	1	0.103	0.132	0.438	-	0.876
HHEX	AS	1	-0.146	0.179	0.417	0.865	0.876	-	No	1	-0.146	0.179	0.417	-	0.876
HPGDS	AS	6	-0.009	0.017	0.620	0.991	0.902	0.543	No	6	-0.009	0.017	0.620	0.176	0.902
ICAM1	AS	4	-0.013	0.022	0.557	0.987	0.902	0.543	No	4	-0.013	0.022	0.557	0.149	0.902
ICAM4	AS	1	0.033	0.063	0.602	1.033	0.902	-	No	1	0.033	0.063	0.602	-	0.902
IFIT3	AS	1	0.033	0.064	0.603	1.034	0.902	-	No	1	0.033	0.064	0.603	-	0.902
IGFBP3	AS	3	-0.045	0.019	0.021	0.956	0.200	0.931	No	3	-0.045	0.019	0.021	0.820	0.200
IL1RL1	AS	6	-0.001	0.010	0.916	0.999	0.969	0.931	No	6	-0.001	0.010	0.916	0.710	0.969
IMMT	AS	1	-0.032	0.057	0.569	0.968	0.902	-	No	1	-0.032	0.057	0.569	-	0.902
ITGAL	AS	1	0.082	0.210	0.697	1.085	0.902	-	No	1	0.082	0.210	0.697	-	0.902
KIF22	AS	1	-0.052	0.187	0.781	0.949	0.937	-	No	1	-0.052	0.187	0.781	-	0.937
KIFBP	AS	1	-0.119	0.149	0.427	0.888	0.876	-	No	1	-0.119	0.149	0.427	-	0.876
LAYN	AS	1	-0.009	0.019	0.627	0.991	0.902	-	No	1	-0.009	0.019	0.627	-	0.902
LMOD1	AS	1	-0.039	0.040	0.331	0.962	0.876	-	No	1	-0.039	0.040	0.331	-	0.876
M6PR	AS	1	0.014	0.036	0.693	1.014	0.902	-	No	1	0.014	0.036	0.693	-	0.902
MAP4K5	AS	1	0.031	0.039	0.416	1.032	0.876	-	No	1	0.031	0.039	0.416	-	0.876
MFGE8	AS	2	0.005	0.020	0.818	1.005	0.946	-	No	2	0.005	0.020	0.818	-	0.946
MPHOSPH8	AS	1	0.003	0.157	0.983	1.003	0.992	-	No	1	0.003	0.157	0.983	-	0.992
MSRA	AS	1	-0.014	0.129	0.912	0.986	0.969	-	No	1	-0.014	0.129	0.912	-	0.969
MST1	AS	2	0.003	0.013	0.803	1.003	0.946	-	No	2	0.003	0.013	0.803	-	0.946
NADK	AS	1	-0.033	0.027	0.228	0.968	0.820	-	No	1	-0.033	0.027	0.228	-	0.820
NGF	AS	1	0.025	0.228	0.912	1.026	0.969	-	No	1	0.025	0.228	0.912	-	0.969
NOMO1	AS	1	-0.014	0.066	0.835	0.986	0.947	-	No	1	-0.014	0.066	0.835	-	0.947
NOS3	AS	1	-0.498	0.156	0.001	0.607	0.025	-	Yes	1	-0.498	0.156	0.001	-	0.025
NPPB	AS	1	-0.023	0.045	0.604	0.977	0.902	-	No	1	-0.023	0.045	0.604	-	0.902
NUCB2	AS	2	-0.029	0.049	0.555	0.972	0.902	-	No	2	-0.029	0.049	0.555	-	0.902
PAM	AS	4	-0.005	0.013	0.721	0.995	0.917	0.874	No	4	-0.005	0.013	0.721	0.538	0.917
PARP1	AS	1	0.051	0.053	0.335	1.053	0.876	-	No	1	0.051	0.053	0.335	-	0.876
PCOLCE	AS	1	-0.032	0.042	0.439	0.968	0.876	-	No	1	-0.032	0.042	0.439	-	0.876
PCSK7	AS	2	0.020	0.021	0.354	1.020	0.876	-	No	2	0.020	0.021	0.354	-	0.876
PDE5A	AS	2	-0.075	0.042	0.073	0.928	0.420	-	No	2	-0.075	0.042	0.073	-	0.420
PDIA3	AS	1	0.116	0.174	0.505	1.123	0.896	-	No	1	0.116	0.174	0.505	-	0.896
PECAM1	AS	1	-0.245	0.210	0.245	0.783	0.830	-	No	1	-0.245	0.210	0.245	-	0.830
PFKFB2	AS	1	0.022	0.036	0.550	1.022	0.902	-	No	1	0.022	0.036	0.550	-	0.902
PLA2G1B	AS	1	-0.107	0.132	0.417	0.898	0.876	-	No	1	-0.107	0.132	0.417	-	0.876
PLXDC2	AS	3	0.009	0.160	0.957	1.009	0.982	0.057	No	3	0.009	0.160	0.957	0.003	0.982
PMS1	AS	1	0.028	0.144	0.843	1.029	0.947	-	No	1	0.028	0.144	0.843	-	0.947
PRDX1	AS	1	-0.053	0.111	0.636	0.949	0.902	-	No	1	-0.053	0.111	0.636	-	0.902
PROCR	AS	1	-0.089	0.015	0.000	0.915	0.000	-	Yes	1	-0.089	0.015	0.000	-	0.000
PRSS53	AS	3	0.019	0.009	0.043	1.019	0.289	0.931	No	3	0.019	0.009	0.043	0.829	0.289
PRTFDC1	AS	2	0.022	0.032	0.493	1.022	0.896	-	No	2	0.022	0.032	0.493	-	0.896
PSRC1	AS	2	0.055	0.034	0.102	1.057	0.501	-	No	2	0.055	0.034	0.102	-	0.501
PTPRF	AS	1	-0.230	0.090	0.010	0.794	0.115	-	No	1	-0.230	0.090	0.010	-	0.115
PTRHD1	AS	1	-0.160	0.178	0.368	0.852	0.876	-	No	1	-0.160	0.178	0.368	-	0.876
PYDC1	AS	1	-0.028	0.023	0.219	0.972	0.811	-	No	1	-0.028	0.023	0.219	-	0.811
RELT	AS	2	0.015	0.021	0.498	1.015	0.896	-	No	2	0.015	0.021	0.498	-	0.896
SDC1	AS	2	0.053	0.046	0.253	1.054	0.830	-	No	2	0.053	0.046	0.253	-	0.830
SDCCAG8	AS	1	0.016	0.033	0.633	1.016	0.902	-	No	1	0.016	0.033	0.633	-	0.902
SDHB	AS	1	0.043	0.113	0.702	1.044	0.902	-	No	1	0.043	0.113	0.702	-	0.902
SERPINI1	AS	3	-0.020	0.017	0.246	0.980	0.830	0.931	No	3	-0.020	0.017	0.246	0.717	0.830
SH2B3	AS	1	0.454	0.095	0.000	1.574	0.000	-	Yes	1	0.454	0.095	0.000	-	0.000
SHMT1	AS	4	0.003	0.010	0.754	1.003	0.919	0.913	No	4	0.003	0.010	0.754	0.611	0.919
SLC16A1	AS	1	-0.123	0.110	0.261	0.884	0.830	-	No	1	-0.123	0.110	0.261	-	0.830
SLC9A3R2	AS	1	0.031	0.068	0.652	1.031	0.902	-	No	1	0.031	0.068	0.652	-	0.902
SMTN	AS	1	-0.129	0.181	0.476	0.879	0.896	-	No	1	-0.129	0.181	0.476	-	0.896
SPINK8	AS	1	0.019	0.016	0.229	1.019	0.820	-	No	1	0.019	0.016	0.229	-	0.820

ST8; MR causal estimates for DBP-associated proteins on all stroke.

Causal candidates prioritized for AS were marked as "Yes" in column "Prioritized". All estimates are from inverse variance weighted method (IVs >1) or Wald-ratio method (IV = 1). AS, all strokes.

										Steiger filtering					
**Exposure**	**Outocme**	**nsnp**	**Beta**	**SE**	**P-value**	**Odds ratio**	**FDR-corrected P-value**	**FDR-corrected Cochran's Q P-value**	**Prioritized**	**nsnp**	**Beta**	**SE**	**P-value**	**Cochran's Q P-value**	**FDR-corrected P-value**
STC1	AS	1	0.026	0.128	0.839	1.026	0.947	-	No	1	0.026	0.128	0.839	-	0.947
STX4	AS	1	0.360	0.159	0.024	1.433	0.205	-	No	1	0.360	0.159	0.024	-	0.205
TBC1D17	AS	2	-0.046	0.017	0.007	0.955	0.090	-	No	2	-0.046	0.017	0.007	-	0.090
TIE1	AS	1	0.030	0.036	0.407	1.030	0.876	-	No	1	0.030	0.036	0.407	-	0.876
TJAP1	AS	1	-0.527	0.163	0.001	0.590	0.023	-	Yes	1	-0.527	0.163	0.001	-	0.023
TMEM106A	AS	1	-0.037	0.066	0.576	0.964	0.902	-	No	1	-0.037	0.066	0.576	-	0.902
TNFRSF17	AS	1	0.000	0.050	1.000	1.000	1.000	-	No	1	0.000	0.050	1.000	-	1.000
TNFSF12	AS	2	0.018	0.019	0.339	1.018	0.876	-	No	2	0.018	0.019	0.339	-	0.876
TP53	AS	1	-0.202	0.115	0.080	0.817	0.427	-	No	1	-0.202	0.115	0.080	-	0.427
UMOD	AS	6	0.006	0.010	0.547	1.006	0.902	0.543	No	6	0.006	0.010	0.547	0.216	0.902
UXS1	AS	2	0.060	0.055	0.278	1.062	0.830	-	No	2	0.060	0.055	0.278	-	0.830
VAT1	AS	1	-0.046	0.047	0.324	0.955	0.876	-	No	1	-0.046	0.047	0.324	-	0.876
VSIG2	AS	1	0.035	0.036	0.330	1.036	0.876	-	No	1	0.035	0.036	0.330	-	0.876
WNT9A	AS	2	0.060	0.107	0.574	1.062	0.902	-	No	2	0.060	0.107	0.574	-	0.902
WWP2	AS	1	0.039	0.043	0.362	1.040	0.876	-	No	1	0.039	0.043	0.362	-	0.876
YAP1	AS	1	-0.045	0.103	0.659	0.956	0.902	-	No	1	-0.045	0.103	0.659	-	0.902
YOD1	AS	1	0.067	0.061	0.275	1.069	0.830	-	No	1	0.067	0.061	0.275	-	0.830
ZBTB17	AS	1	-0.385	0.183	0.036	0.681	0.256	-	No	1	-0.385	0.183	0.036	-	0.256

nsnp = number of single nucleotide polymorphisms; SE = standard error; Cochran's Q P-value = p-value from Cochran's Q test assessing heterogeneity; FDR=false discovery rate

ST9; MR causal estimates for SBP-associated proteins on ischemic stroke.

Causal candidates prioritized for AIS were marked as "Yes" in column "Prioritized". All estimates are from inverse variance weighted method (IVs >1) or Wald-ratio method (IV = 1). AIS, ischemic stroke.

										Steiger filtering					
**Exposure**	**Outcme**	**nsnp**	**Beta**	**SE**	**P-value**	**Odds ratio**	**FDR-corrected P-value**	**FDR-corrected Cochran's Q P-value**	**Prioritized**	**nsnp**	**Beta**	**SE**	**P-value**	**Cochran's Q P-value**	**FDR-corrected P-value**
ACOX1	AIS	1	-0.026	0.104	0.803	0.974	0.996	-	No	1	-0.026	0.104	0.803	-	0.996
ACRBP	AIS	1	0.031	0.052	0.553	1.031	0.957	-	No	1	0.031	0.052	0.553	-	0.957
ADAM23	AIS	4	0.014	0.012	0.252	1.014	0.770	0.941	No	4	0.014	0.012	0.252	0.821	0.770
ADAMTS1	AIS	1	0.112	0.177	0.526	1.119	0.957	-	No	1	0.112	0.177	0.526	-	0.957
ADAMTS4	AIS	1	-0.035	0.059	0.549	0.965	0.957	-	No	1	-0.035	0.059	0.549	-	0.957
ADAMTS8	AIS	5	0.007	0.015	0.662	1.007	0.970	0.900	No	5	0.007	0.015	0.662	0.665	0.970
ADAMTSL5	AIS	2	-0.005	0.055	0.921	0.995	0.996	-	No	2	-0.005	0.055	0.921	-	0.996
ADM	AIS	1	-0.125	0.198	0.530	0.883	0.957	-	No	1	-0.125	0.198	0.530	-	0.957
AMOTL2	AIS	1	-0.053	0.145	0.713	0.948	0.978	-	No	1	-0.053	0.145	0.713	-	0.978
ANKMY2	AIS	1	0.008	0.101	0.934	1.008	0.996	-	No	1	0.008	0.101	0.934	-	0.996
APOA1	AIS	1	-0.027	0.112	0.808	0.973	0.996	-	No	1	-0.027	0.112	0.808	-	0.996
APOA2	AIS	1	-0.028	0.114	0.808	0.973	0.996	-	No	1	-0.028	0.114	0.808	-	0.996
APOBR	AIS	3	-0.007	0.007	0.329	0.993	0.818	0.940	No	3	-0.007	0.007	0.329	0.796	0.818
APOC1	AIS	2	-0.145	0.088	0.100	0.865	0.610	-	No	2	-0.145	0.088	0.100	-	0.610
ASPN	AIS	2	0.048	0.031	0.119	1.050	0.622	-	No	2	0.048	0.031	0.119	-	0.622
ATXN2L	AIS	1	0.055	0.195	0.779	1.056	0.996	-	No	1	0.055	0.195	0.779	-	0.996
B4GAT1	AIS	3	0.006	0.032	0.857	1.006	0.996	0.783	No	3	0.006	0.032	0.857	0.297	0.996
BAG4	AIS	1	-0.464	0.198	0.019	0.628	0.205	-	No	1	-0.464	0.198	0.019	-	0.205
BCAM	AIS	5	0.053	0.044	0.226	1.054	0.729	0.524	No	5	0.053	0.044	0.226	0.094	0.729
BMP6	AIS	3	0.003	0.047	0.944	1.003	0.996	0.875	No	3	0.003	0.047	0.944	0.571	0.996
BNIP3L	AIS	1	-0.033	0.144	0.818	0.967	0.996	-	No	1	-0.033	0.144	0.818	-	0.996
BRAP	AIS	1	1.546	0.201	0.000	4.692	0.000	-	Yes	1	1.546	0.201	0.000	-	0.000
BRSK2	AIS	1	-0.328	0.151	0.030	0.721	0.258	-	No	1	-0.328	0.151	0.030	-	0.258
CA12	AIS	2	0.000	0.024	0.998	1.000	1.000	-	No	2	0.000	0.024	0.998	-	1.000
CA9	AIS	2	0.025	0.069	0.717	1.025	0.978	-	No	2	0.025	0.069	0.717	-	0.978
CACNB3	AIS	1	-0.008	0.073	0.910	0.992	0.996	-	No	1	-0.008	0.073	0.910	-	0.996
CALCA	AIS	1	-0.057	0.070	0.420	0.945	0.917	-	No	1	-0.057	0.070	0.420	-	0.917
CALCOCO2	AIS	1	0.317	0.129	0.014	1.373	0.180	-	No	1	0.317	0.129	0.014	-	0.180
CCN3	AIS	3	-0.016	0.029	0.586	0.984	0.967	0.875	No	3	-0.016	0.029	0.586	0.584	0.967
CD14	AIS	1	-0.036	0.025	0.153	0.965	0.713	-	No	1	-0.036	0.025	0.153	-	0.713
CD164L2	AIS	1	0.021	0.016	0.187	1.021	0.725	-	No	1	0.021	0.016	0.187	-	0.725
CD46	AIS	1	-0.104	0.095	0.274	0.901	0.794	-	No	1	-0.104	0.095	0.274	-	0.794
CD59	AIS	2	-0.035	0.032	0.273	0.965	0.794	-	No	2	-0.035	0.032	0.273	-	0.794
CEP170	AIS	1	-0.043	0.113	0.705	0.958	0.978	-	No	1	-0.043	0.113	0.705	-	0.978
CERT	AIS	1	-0.080	0.157	0.609	0.923	0.967	-	No	1	-0.080	0.157	0.609	-	0.967
CETN3	AIS	1	-0.023	0.059	0.701	0.978	0.978	-	No	1	-0.023	0.059	0.701	-	0.978
CFHR2	AIS	5	0.001	0.009	0.869	1.001	0.996	0.783	No	5	0.001	0.009	0.869	0.293	0.996
CFHR4	AIS	7	0.007	0.009	0.443	1.007	0.956	0.875	No	7	0.007	0.009	0.443	0.496	0.956
CLMP	AIS	2	-0.040	0.033	0.230	0.961	0.729	-	No	2	-0.040	0.033	0.230	-	0.729
COL1A1	AIS	1	-0.039	0.168	0.817	0.962	0.996	-	No	1	-0.039	0.168	0.817	-	0.996
COMP	AIS	1	0.034	0.116	0.767	1.035	0.996	-	No	1	0.034	0.116	0.767	-	0.996
COMT	AIS	2	-0.038	0.036	0.292	0.963	0.796	-	No	2	-0.038	0.036	0.292	-	0.796
CPXM1	AIS	1	0.015	0.024	0.539	1.015	0.957	-	No	1	0.015	0.024	0.539	-	0.957
CTSO	AIS	1	-0.086	0.040	0.033	0.918	0.258	-	No	1	-0.086	0.040	0.033	-	0.258
DAG1	AIS	1	-0.055	0.120	0.645	0.946	0.967	-	No	1	-0.055	0.120	0.645	-	0.967
DDHD2	AIS	1	-0.129	0.075	0.085	0.879	0.556	-	No	1	-0.129	0.075	0.085	-	0.556
DTX3	AIS	1	0.104	0.085	0.222	1.109	0.729	-	No	1	0.104	0.085	0.222	-	0.729
DUSP13	AIS	1	-0.024	0.027	0.375	0.976	0.904	-	No	1	-0.024	0.027	0.375	-	0.904
DUSP29	AIS	1	-0.026	0.056	0.650	0.975	0.967	-	No	1	-0.026	0.056	0.650	-	0.967
EDN1	AIS	1	0.022	0.041	0.598	1.022	0.967	-	No	1	0.022	0.041	0.598	-	0.967
EFEMP1	AIS	2	-0.028	0.053	0.600	0.973	0.967	-	No	2	-0.028	0.053	0.600	-	0.967
EIF4G3	AIS	1	0.093	0.092	0.310	1.098	0.796	-	No	1	0.093	0.092	0.310	-	0.796
ELOA	AIS	1	0.117	0.091	0.198	1.124	0.729	-	No	1	0.117	0.091	0.198	-	0.729
ENPEP	AIS	1	-0.545	0.213	0.010	0.580	0.140	-	No	1	-0.545	0.213	0.010	-	0.140
ERI1	AIS	1	0.048	0.107	0.650	1.050	0.967	-	No	1	0.048	0.107	0.650	-	0.967
ERP29	AIS	1	1.658	0.215	0.000	5.247	0.000	-	Yes	1	1.658	0.215	0.000	-	0.000

ST9; MR causal estimates for SBP-associated proteins on ischemic stroke.

Causal candidates prioritized for AIS were marked as "Yes" in column "Prioritized". All estimates are from inverse variance weighted method (IVs >1) or Wald-ratio method (IV = 1). AIS, ischemic stroke.

										Steiger filtering					
Exposure	Outcme	nsnp	Beta	SE	P-value	Odds ratio	FDR-corrected P-value	FDR-corrected Cochran's Q P-value	Prioritized	nsnp	Beta	SE	P-value	Cochran's Q P-value	FDR-corrected P-value
ESAM	AIS	1	-0.078	0.057	0.173	0.925	0.725	-	No	1	-0.078	0.057	0.173	-	0.725
F13B	AIS	1	0.007	0.021	0.726	1.007	0.978	-	No	1	0.007	0.021	0.726	-	0.978
FDX1	AIS	1	-0.010	0.152	0.948	0.990	0.996	-	No	1	-0.010	0.152	0.948	-	0.996
FES	AIS	1	-0.245	0.055	0.000	0.783	0.000	-	Yes	1	-0.245	0.055	0.000	-	0.000
FGF2	AIS	3	0.016	0.021	0.465	1.016	0.957	0.524	No	3	0.016	0.021	0.465	0.091	0.957
FGF5	AIS	4	0.041	0.011	0.000	1.041	0.009	0.875	Yes	4	0.041	0.011	0.000	0.551	0.009
FKBP7	AIS	1	0.217	0.105	0.039	1.243	0.285	-	No	1	0.217	0.105	0.039	-	0.285
FN1	AIS	2	-0.047	0.019	0.016	0.954	0.193	-	No	2	-0.047	0.019	0.016	-	0.193
FOXJ3	AIS	1	0.109	0.108	0.313	1.116	0.796	-	No	1	0.109	0.108	0.313	-	0.796
FOXO3	AIS	1	0.232	0.202	0.250	1.261	0.770	-	No	1	0.232	0.202	0.250	-	0.770
FUCA1	AIS	1	0.002	0.020	0.926	1.002	0.996	-	No	1	0.002	0.020	0.926	-	0.996
FURIN	AIS	1	0.217	0.040	0.000	1.242	0.000	-	Yes	1	0.217	0.040	0.000	-	0.000
GCHFR	AIS	1	0.079	0.066	0.228	1.082	0.729	-	No	1	0.079	0.066	0.228	-	0.729
GFER	AIS	1	-0.128	0.118	0.279	0.880	0.794	-	No	1	-0.128	0.118	0.279	-	0.794
GHR	AIS	4	0.010	0.014	0.476	1.010	0.957	0.752	No	4	0.010	0.014	0.476	0.231	0.957
GIMAP7	AIS	3	0.001	0.013	0.913	1.001	0.996	0.977	No	3	0.001	0.013	0.913	0.902	0.996
GIT1	AIS	1	0.085	0.166	0.608	1.089	0.967	-	No	1	0.085	0.166	0.608	-	0.967
GORASP2	AIS	1	0.006	0.147	0.969	1.006	0.996	-	No	1	0.006	0.147	0.969	-	0.996
GRP	AIS	4	0.020	0.025	0.409	1.021	0.913	0.752	No	4	0.020	0.025	0.409	0.215	0.913
HADH	AIS	1	0.103	0.143	0.472	1.108	0.957	-	No	1	0.103	0.143	0.472	-	0.957
HHEX	AIS	1	-0.190	0.193	0.324	0.827	0.816	-	No	1	-0.190	0.193	0.324	-	0.816
HMOX2	AIS	1	-0.030	0.100	0.768	0.971	0.996	-	No	1	-0.030	0.100	0.768	-	0.996
HYAL1	AIS	2	-0.024	0.028	0.392	0.976	0.904	-	No	2	-0.024	0.028	0.392	-	0.904
ICAM2	AIS	1	0.070	0.143	0.624	1.072	0.967	-	No	1	0.070	0.143	0.624	-	0.967
IDUA	AIS	4	-0.008	0.022	0.728	0.992	0.978	0.524	No	4	-0.008	0.022	0.728	0.073	0.978
IFI30	AIS	2	-0.026	0.016	0.114	0.974	0.610	-	No	2	-0.026	0.016	0.114	-	0.610
IFNGR2	AIS	5	0.004	0.007	0.527	1.004	0.957	0.923	No	5	0.004	0.007	0.527	0.757	0.957
IGFBP3	AIS	3	-0.045	0.021	0.033	0.956	0.258	0.875	No	3	-0.045	0.021	0.033	0.563	0.258
IMMT	AIS	1	-0.027	0.064	0.671	0.973	0.973	-	No	1	-0.027	0.064	0.671	-	0.973
IMPA1	AIS	1	0.075	0.037	0.043	1.078	0.306	-	No	1	0.075	0.037	0.043	-	0.306
ITGAL	AIS	1	-0.107	0.227	0.638	0.899	0.967	-	No	1	-0.107	0.227	0.638	-	0.967
ITIH1	AIS	1	0.063	0.053	0.232	1.065	0.729	-	No	1	0.063	0.053	0.232	-	0.729
KIFBP	AIS	1	-0.041	0.164	0.803	0.960	0.996	-	No	1	-0.041	0.164	0.803	-	0.996
LMOD1	AIS	1	-0.070	0.044	0.108	0.932	0.610	-	No	1	-0.070	0.044	0.108	-	0.610
LRIG1	AIS	3	-0.018	0.011	0.110	0.982	0.610	0.752	No	3	-0.018	0.011	0.110	0.219	0.610
LYAR	AIS	1	-0.080	0.165	0.626	0.923	0.967	-	No	1	-0.080	0.165	0.626	-	0.967
M6PR	AIS	1	0.027	0.039	0.484	1.028	0.957	-	No	1	0.027	0.039	0.484	-	0.957
MANEAL	AIS	1	-0.128	0.197	0.516	0.880	0.957	-	No	1	-0.128	0.197	0.516	-	0.957
MANSC4	AIS	2	-0.003	0.010	0.735	0.997	0.978	-	No	2	-0.003	0.010	0.735	-	0.978
MAP4K5	AIS	1	0.031	0.042	0.462	1.031	0.957	-	No	1	0.031	0.042	0.462	-	0.957
MDH1	AIS	2	0.010	0.096	0.917	1.010	0.996	-	No	2	0.010	0.096	0.917	-	0.996
MEGF9	AIS	1	-0.065	0.025	0.009	0.937	0.134	-	No	1	-0.065	0.025	0.009	-	0.134
MPHOSPH8	AIS	1	0.039	0.176	0.827	1.039	0.996	-	No	1	0.039	0.176	0.827	-	0.996
MPI	AIS	2	-0.009	0.067	0.891	0.991	0.996	-	No	2	-0.009	0.067	0.891	-	0.996
MSRA	AIS	1	0.034	0.140	0.808	1.035	0.996	-	No	1	0.034	0.140	0.808	-	0.996
MST1	AIS	2	0.002	0.011	0.875	1.002	0.996	-	No	2	0.002	0.011	0.875	-	0.996
MVK	AIS	1	-0.035	0.085	0.680	0.966	0.973	-	No	1	-0.035	0.085	0.680	-	0.973
NADK	AIS	1	-0.055	0.031	0.081	0.947	0.543	-	No	1	-0.055	0.031	0.081	-	0.543
NAGA	AIS	2	-0.043	0.053	0.411	0.958	0.913	-	No	2	-0.043	0.053	0.411	-	0.913
NFE2	AIS	1	-0.382	0.109	0.000	0.682	0.009	-	Yes	1	-0.382	0.109	0.000	-	0.009
NFU1	AIS	1	0.037	0.068	0.584	1.038	0.967	-	No	1	0.037	0.068	0.584	-	0.967
NGF	AIS	1	-0.025	0.251	0.919	0.975	0.996	-	No	1	-0.025	0.251	0.919	-	0.996
NOTCH3	AIS	1	0.190	0.152	0.210	1.210	0.729	-	No	1	0.190	0.152	0.210	-	0.729
NPPB	AIS	1	-0.022	0.050	0.665	0.979	0.970	-	No	1	-0.022	0.050	0.665	-	0.970
NTRK3	AIS	2	-0.037	0.035	0.294	0.964	0.796	-	No	2	-0.037	0.035	0.294	-	0.796
NUCB2	AIS	2	-0.026	0.056	0.640	0.974	0.967	-	No	2	-0.026	0.056	0.640	-	0.967

ST9; MR causal estimates for SBP-associated proteins on ischemic stroke.

Causal candidates prioritized for AIS were marked as "Yes" in column "Prioritized". All estimates are from inverse variance weighted method (IVs >1) or Wald-ratio method (IV = 1). AIS, ischemic stroke.

										Steiger filtering					
**Exposure**	**Outcme**	**nsnp**	**Beta**	**SE**	**P-value**	**Odds ratio**	**FDR-corrected P-value**	**FDR-corrected Cochran's Q P-value**	**Prioritized**	**nsnp**	**Beta**	**SE**	**P-value**	**Cochran's Q P-value**	**FDR-corrected P-value**
NUDT5	AIS	1	-0.093	0.131	0.476	0.911	0.957	-	No	1	-0.093	0.131	0.476	-	0.957
NUMB	AIS	1	0.032	0.082	0.700	1.032	0.978	-	No	1	0.032	0.082	0.700	-	0.978
OGA	AIS	1	-0.017	0.071	0.811	0.983	0.996	-	No	1	-0.017	0.071	0.811	-	0.996
OPLAH	AIS	1	-0.008	0.040	0.848	0.992	0.996	-	No	1	-0.008	0.040	0.848	-	0.996
PCBP2	AIS	1	-0.523	0.149	0.000	0.593	0.009	-	Yes	1	-0.523	0.149	0.000	-	0.009
PCSK7	AIS	2	0.019	0.030	0.538	1.019	0.957	-	No	2	0.019	0.030	0.538	-	0.957
PDE5A	AIS	2	-0.061	0.045	0.176	0.941	0.725	-	No	2	-0.061	0.045	0.176	-	0.725
PDGFRA	AIS	2	0.020	0.032	0.542	1.020	0.957	-	No	2	0.020	0.032	0.542	-	0.957
PECAM1	AIS	1	-0.270	0.219	0.217	0.763	0.729	-	No	1	-0.270	0.219	0.217	-	0.729
PFKFB2	AIS	1	0.013	0.040	0.739	1.014	0.978	-	No	1	0.013	0.040	0.739	-	0.978
PGF	AIS	1	-0.023	0.047	0.617	0.977	0.967	-	No	1	-0.023	0.047	0.617	-	0.967
PHLDB1	AIS	1	-0.104	0.165	0.529	0.901	0.957	-	No	1	-0.104	0.165	0.529	-	0.957
PKD1	AIS	1	-0.172	0.169	0.309	0.842	0.796	-	No	1	-0.172	0.169	0.309	-	0.796
PLA2G1B	AIS	1	-0.084	0.149	0.571	0.919	0.967	-	No	1	-0.084	0.149	0.571	-	0.967
PMS1	AIS	1	-0.011	0.158	0.946	0.989	0.996	-	No	1	-0.011	0.158	0.946	-	0.996
PPP1R14D	AIS	1	0.181	0.132	0.172	1.198	0.725	-	No	1	0.181	0.132	0.172	-	0.725
PRG2	AIS	1	0.007	0.077	0.931	1.007	0.996	-	No	1	0.007	0.077	0.931	-	0.996
PRKAB1	AIS	1	0.000	0.042	0.994	1.000	1.000	-	No	1	0.000	0.042	0.994	-	1.000
PSMD5	AIS	1	0.551	0.177	0.002	1.734	0.028	-	Yes	1	0.551	0.177	0.002	-	0.028
PSRC1	AIS	2	0.051	0.037	0.169	1.052	0.725	-	No	2	0.051	0.037	0.169	-	0.725
PTRHD1	AIS	1	-0.173	0.193	0.371	0.841	0.904	-	No	1	-0.173	0.193	0.371	-	0.904
QPCT	AIS	3	0.042	0.050	0.395	1.043	0.904	0.030	No	3	0.042	0.050	0.395	0.001	0.904
RABEPK	AIS	1	0.077	0.049	0.113	1.080	0.610	-	No	1	0.077	0.049	0.113	-	0.610
RARRES1	AIS	5	0.011	0.015	0.453	1.011	0.957	0.875	No	5	0.011	0.015	0.453	0.443	0.957
RARRES2	AIS	1	0.049	0.037	0.191	1.050	0.728	-	No	1	0.049	0.037	0.191	-	0.728
RELT	AIS	2	0.030	0.027	0.265	1.030	0.794	-	No	2	0.030	0.027	0.265	-	0.794
RSPO3	AIS	1	0.060	0.048	0.213	1.062	0.729	-	No	1	0.060	0.048	0.213	-	0.729
SCARA5	AIS	4	-0.060	0.027	0.026	0.942	0.256	0.752	No	4	-0.060	0.027	0.026	0.190	0.256
SDCCAG8	AIS	1	0.001	0.036	0.978	1.001	0.997	-	No	1	0.001	0.036	0.978	-	0.997
SDHB	AIS	1	0.018	0.124	0.883	1.018	0.996	-	No	1	0.018	0.124	0.883	-	0.996
SELENOP	AIS	1	-0.003	0.072	0.970	0.997	0.996	-	No	1	-0.003	0.072	0.970	-	0.996
SEMA6C	AIS	1	0.074	0.082	0.366	1.077	0.900	-	No	1	0.074	0.082	0.366	-	0.900
SERPINI1	AIS	3	-0.027	0.019	0.149	0.974	0.713	0.875	No	3	-0.027	0.019	0.149	0.606	0.713
SH2B3	AIS	1	0.551	0.104	0.000	1.735	0.000	-	Yes	1	0.551	0.104	0.000	-	0.000
SHMT1	AIS	4	0.007	0.011	0.503	1.007	0.957	0.900	No	4	0.007	0.011	0.503	0.678	0.957
SIL1	AIS	1	0.106	0.121	0.381	1.112	0.904	-	No	1	0.106	0.121	0.381	-	0.904
SLC16A1	AIS	1	-0.177	0.118	0.134	0.837	0.665	-	No	1	-0.177	0.118	0.134	-	0.665
SLC39A14	AIS	1	-0.097	0.089	0.277	0.907	0.794	-	No	1	-0.097	0.089	0.277	-	0.794
SLC9A3R2	AIS	1	-0.041	0.073	0.569	0.959	0.967	-	No	1	-0.041	0.073	0.569	-	0.967
SMOC2	AIS	4	0.000	0.019	0.980	1.000	0.997	0.978	No	4	0.000	0.019	0.980	0.978	0.997
SOST	AIS	1	0.057	0.129	0.660	1.059	0.970	-	No	1	0.057	0.129	0.660	-	0.970
SPINK8	AIS	1	0.021	0.018	0.231	1.021	0.729	-	No	1	0.021	0.018	0.231	-	0.729
SPRED2	AIS	1	-0.141	0.112	0.210	0.869	0.729	-	No	1	-0.141	0.112	0.210	-	0.729
TARBP2	AIS	1	-0.563	0.160	0.000	0.570	0.009	-	Yes	1	-0.563	0.160	0.000	-	0.009
TBC1D23	AIS	1	-0.028	0.045	0.543	0.973	0.957	-	No	1	-0.028	0.045	0.543	-	0.957
TEK	AIS	5	0.025	0.029	0.384	1.026	0.904	0.173	No	5	0.025	0.029	0.384	0.013	0.904
TGFB2	AIS	1	0.123	0.052	0.017	1.131	0.193	-	No	1	0.123	0.052	0.017	-	0.193
TIE1	AIS	1	0.000	0.041	1.000	1.000	1.000	-	No	1	0.000	0.041	1.000	-	1.000
TJAP1	AIS	1	-0.597	0.176	0.001	0.551	0.014	-	Yes	1	-0.597	0.176	0.001	-	0.014
TNFRSF17	AIS	1	0.025	0.054	0.641	1.026	0.967	-	No	1	0.025	0.054	0.641	-	0.967
TNFSF12	AIS	2	-0.008	0.020	0.709	0.992	0.978	-	No	2	-0.008	0.020	0.709	-	0.978
TNFSF13B	AIS	4	-0.023	0.065	0.722	0.977	0.978	0.977	No	4	-0.023	0.065	0.722	0.880	0.978
TWF2	AIS	1	-0.093	0.115	0.419	0.911	0.917	-	No	1	-0.093	0.115	0.419	-	0.917
UBE2L6	AIS	2	0.040	0.030	0.185	1.041	0.725	-	No	2	0.040	0.030	0.185	-	0.725
UMOD	AIS	6	0.005	0.009	0.582	1.005	0.967	0.875	No	6	0.005	0.009	0.582	0.560	0.967
VSIG2	AIS	1	0.053	0.039	0.173	1.054	0.725	-	No	1	0.053	0.039	0.173	-	0.725

ST9; MR causal estimates for SBP-associated proteins on ischemic stroke.

Causal candidates prioritized for AIS were marked as "Yes" in column "Prioritized". All estimates are from inverse variance weighted method (IVs >1) or Wald-ratio method (IV = 1). AIS, ischemic stroke.

										Steiger filtering					
**Exposure**	**Outocme**	**nsnp**	**Beta**	**SE**	**P-value**	**Odds ratio**	**FDR-corrected P-value**	**FDR-corrected Cochran's Q P-value**	**Prioritized**	**nsnp**	**Beta**	**SE**	**P-value**	**Cochran's Q P-value**	**FDR-corrected P-value**
WARS	AIS	1	0.119	0.037	0.001	1.127	0.026	-	Yes	1	0.119	0.037	0.001	-	0.026
WASHC3	AIS	1	0.094	0.093	0.312	1.099	0.796	-	No	1	0.094	0.093	0.312	-	0.796
WWP2	AIS	1	0.013	0.046	0.777	1.013	0.996	-	No	1	0.013	0.046	0.777	-	0.996
YOD1	AIS	1	0.049	0.067	0.462	1.050	0.957	-	No	1	0.049	0.067	0.462	-	0.957
ZBTB17	AIS	1	-0.451	0.204	0.027	0.637	0.256	-	No	1	-0.451	0.204	0.027	-	0.256
ZFYVE19	AIS	1	-0.025	0.019	0.201	0.975	0.729	-	No	1	-0.025	0.019	0.201	-	0.729

nsnp = number of single nucleotide polymorphisms; SE = standard error; Cochran's Q P-value = p-value from Cochran's Q test assessing heterogeneity; FDR=false discovery rate

ST10; MR causal estimates for DBP-associated proteins on ischemic stroke.

Causal candidates prioritized for AIS were marked as "Yes" in column "Prioritized". All estimates are from inverse variance weighted method (IVs >1) or Wald-ratio method (IV = 1). AIS, ischemic stroke.

										Steiger filtering					
**Exposure**	**Outcome**	**nsnp**	**Beta**	**SE**	**P-value**	**Odds ratio**	**FDR-corrected P-value**	**FDR-corrected Cochran's Q P-value**	**Prioritized**	**nsnp**	**Beta**	**SE**	**P-value**	**Cochran's Q P-value**	**FDR-corrected P-value**
AAMDC	AIS	2	-0.011	0.010	0.296	0.990	0.796	-	No	2	-0.011	0.010	0.296	-	0.796
ABO	AIS	7	0.041	0.006	0.000	1.042	0.000	0.875	Yes	7	0.041	0.006	0.000	0.487	0.000
ACOX1	AIS	1	-0.026	0.104	0.803	0.974	0.996	-	No	1	-0.026	0.104	0.803	-	0.996
ADM	AIS	1	-0.125	0.198	0.530	0.883	0.957	-	No	1	-0.125	0.198	0.530	-	0.957
AMFR	AIS	1	0.248	0.179	0.166	1.282	0.725	-	No	1	0.248	0.179	0.166	-	0.725
AMOTL2	AIS	1	-0.053	0.145	0.713	0.948	0.978	-	No	1	-0.053	0.145	0.713	-	0.978
ANKMY2	AIS	1	0.008	0.101	0.934	1.008	0.996	-	No	1	0.008	0.101	0.934	-	0.996
AOC1	AIS	3	-0.057	0.025	0.025	0.945	0.256	0.978	No	3	-0.057	0.025	0.025	0.932	0.256
AOC3	AIS	2	-0.012	0.014	0.389	0.988	0.904	-	No	2	-0.012	0.014	0.389	-	0.904
APOA1	AIS	1	-0.027	0.112	0.808	0.973	0.996	-	No	1	-0.027	0.112	0.808	-	0.996
APOBR	AIS	3	-0.007	0.007	0.329	0.993	0.818	0.940	No	3	-0.007	0.007	0.329	0.796	0.818
APOC1	AIS	2	-0.145	0.088	0.100	0.865	0.610	-	No	2	-0.145	0.088	0.100	-	0.610
ARSB	AIS	3	-0.003	0.026	0.893	0.997	0.996	0.900	No	3	-0.003	0.026	0.893	0.716	0.996
ATXN2L	AIS	1	0.055	0.195	0.779	1.056	0.996	-	No	1	0.055	0.195	0.779	-	0.996
AXL	AIS	2	-0.020	0.049	0.678	0.980	0.973	-	No	2	-0.020	0.049	0.678	-	0.973
BAG4	AIS	1	-0.464	0.198	0.019	0.628	0.205	-	No	1	-0.464	0.198	0.019	-	0.205
BNIP3L	AIS	1	-0.033	0.144	0.818	0.967	0.996	-	No	1	-0.033	0.144	0.818	-	0.996
CA12	AIS	2	0.000	0.024	0.998	1.000	1.000	-	No	2	0.000	0.024	0.998	-	1.000
CACNB3	AIS	1	-0.008	0.073	0.910	0.992	0.996	-	No	1	-0.008	0.073	0.910	-	0.996
CALCA	AIS	1	-0.057	0.070	0.420	0.945	0.917	-	No	1	-0.057	0.070	0.420	-	0.917
CEP170	AIS	1	-0.043	0.113	0.705	0.958	0.978	-	No	1	-0.043	0.113	0.705	-	0.978
CFHR4	AIS	7	0.007	0.009	0.443	1.007	0.956	0.875	No	7	0.007	0.009	0.443	0.496	0.956
CHMP1A	AIS	1	0.109	0.083	0.187	1.115	0.725	-	No	1	0.109	0.083	0.187	-	0.725
CLIC5	AIS	1	0.040	0.059	0.493	1.041	0.957	-	No	1	0.040	0.059	0.493	-	0.957
COL1A1	AIS	1	-0.039	0.168	0.817	0.962	0.996	-	No	1	-0.039	0.168	0.817	-	0.996
CTF1	AIS	1	-0.254	0.149	0.088	0.776	0.559	-	No	1	-0.254	0.149	0.088	-	0.559
DAG1	AIS	1	-0.055	0.120	0.645	0.946	0.967	-	No	1	-0.055	0.120	0.645	-	0.967
DARS1	AIS	1	-0.077	0.108	0.477	0.926	0.957	-	No	1	-0.077	0.108	0.477	-	0.957
DBN1	AIS	1	0.067	0.143	0.641	1.069	0.967	-	No	1	0.067	0.143	0.641	-	0.967
DDHD2	AIS	1	-0.129	0.075	0.085	0.879	0.556	-	No	1	-0.129	0.075	0.085	-	0.556
DENR	AIS	1	-0.031	0.214	0.883	0.969	0.996	-	No	1	-0.031	0.214	0.883	-	0.996
DNER	AIS	5	-0.014	0.022	0.517	0.986	0.957	0.875	No	5	-0.014	0.022	0.517	0.592	0.957
DOK2	AIS	1	-0.068	0.136	0.617	0.934	0.967	-	No	1	-0.068	0.136	0.617	-	0.967
DPEP1	AIS	4	0.002	0.047	0.966	1.002	0.996	0.875	No	4	0.002	0.047	0.966	0.391	0.996
DPP4	AIS	2	-0.044	0.033	0.180	0.957	0.725	-	No	2	-0.044	0.033	0.180	-	0.725
DUSP13	AIS	1	-0.024	0.027	0.375	0.976	0.904	-	No	1	-0.024	0.027	0.375	-	0.904
EFNA1	AIS	1	0.002	0.022	0.919	1.002	0.996	-	No	1	0.002	0.022	0.919	-	0.996
EIF4G3	AIS	1	0.093	0.092	0.310	1.098	0.796	-	No	1	0.093	0.092	0.310	-	0.796
ELOA	AIS	1	0.117	0.091	0.198	1.124	0.729	-	No	1	0.117	0.091	0.198	-	0.729
ENPEP	AIS	1	-0.545	0.213	0.010	0.580	0.140	-	No	1	-0.545	0.213	0.010	-	0.140
EPHA2	AIS	2	-0.026	0.057	0.647	0.974	0.967	-	No	2	-0.026	0.057	0.647	-	0.967
EPO	AIS	1	-0.158	0.100	0.114	0.854	0.610	-	No	1	-0.158	0.100	0.114	-	0.610
ERI1	AIS	1	0.048	0.107	0.650	1.050	0.967	-	No	1	0.048	0.107	0.650	-	0.967
ESAM	AIS	1	-0.078	0.057	0.173	0.925	0.725	-	No	1	-0.078	0.057	0.173	-	0.725
F12	AIS	5	-0.012	0.011	0.298	0.988	0.796	0.752	No	5	-0.012	0.011	0.298	0.213	0.796
FADD	AIS	1	-0.011	0.092	0.903	0.989	0.996	-	No	1	-0.011	0.092	0.903	-	0.996
FDX1	AIS	1	-0.010	0.152	0.948	0.990	0.996	-	No	1	-0.010	0.152	0.948	-	0.996
FES	AIS	1	-0.245	0.055	0.000	0.783	0.000	-	Yes	1	-0.245	0.055	0.000	-	0.000
FGF5	AIS	4	0.041	0.011	0.000	1.041	0.009	0.875	Yes	4	0.041	0.011	0.000	0.551	0.009
FOXJ3	AIS	1	0.109	0.108	0.313	1.116	0.796	-	No	1	0.109	0.108	0.313	-	0.796
FURIN	AIS	1	0.217	0.040	0.000	1.242	0.000	-	Yes	1	0.217	0.040	0.000	-	0.000
GFER	AIS	1	-0.128	0.118	0.279	0.880	0.794	-	No	1	-0.128	0.118	0.279	-	0.794
GIMAP7	AIS	3	0.001	0.013	0.913	1.001	0.996	0.977	No	3	0.001	0.013	0.913	0.902	0.996
GLO1	AIS	2	0.009	0.027	0.731	1.009	0.978	-	No	2	0.009	0.027	0.731	-	0.978
GORASP2	AIS	1	0.006	0.147	0.969	1.006	0.996	-	No	1	0.006	0.147	0.969	-	0.996
GRP	AIS	4	0.020	0.025	0.409	1.021	0.913	0.752	No	4	0.020	0.025	0.409	0.215	0.913

ST10; MR causal estimates for DBP-associated proteins on ischemic stroke.

Causal candidates prioritized for AIS were marked as "Yes" in column "Prioritized". All estimates are from inverse variance weighted method (IVs >1) or Wald-ratio method (IV = 1). AIS, ischemic stroke.

										Steiger filtering					
Exposure	Outcme	nsnp	Beta	SE	P-value	Odds ratio	FDR-corrected P-value	FDR-corrected Cochran's Q P-value	Prioritized	nsnp	Beta	SE	P-value	Cochran's Q P-value	FDR-corrected P-value
HADH	AIS	1	0.103	0.143	0.472	1.108	0.957	-	No	1	0.103	0.143	0.472	-	0.957
HHEX	AIS	1	-0.190	0.193	0.324	0.827	0.816	-	No	1	-0.190	0.193	0.324	-	0.816
HPGDS	AIS	6	-0.003	0.016	0.859	0.997	0.996	0.783	No	6	-0.003	0.016	0.859	0.301	0.996
ICAM1	AIS	4	-0.004	0.028	0.885	0.996	0.996	0.450	No	4	-0.004	0.028	0.885	0.046	0.996
ICAM4	AIS	1	-0.004	0.067	0.957	0.996	0.996	-	No	1	-0.004	0.067	0.957	-	0.996
IFIT3	AIS	1	0.012	0.069	0.863	1.012	0.996	-	No	1	0.012	0.069	0.863	-	0.996
IGFBP3	AIS	3	-0.045	0.021	0.033	0.956	0.258	0.875	No	3	-0.045	0.021	0.033	0.563	0.258
IL1RL1	AIS	6	0.000	0.010	0.992	1.000	1.000	0.978	No	6	0.000	0.010	0.992	0.954	1.000
IMMT	AIS	1	-0.027	0.064	0.671	0.973	0.973	-	No	1	-0.027	0.064	0.671	-	0.973
ITGAL	AIS	1	-0.107	0.227	0.638	0.899	0.967	-	No	1	-0.107	0.227	0.638	-	0.967
KIF22	AIS	1	-0.008	0.205	0.970	0.992	0.996	-	No	1	-0.008	0.205	0.970	-	0.996
KIFBP	AIS	1	-0.041	0.164	0.803	0.960	0.996	-	No	1	-0.041	0.164	0.803	-	0.996
LAYN	AIS	1	-0.018	0.021	0.400	0.982	0.906	-	No	1	-0.018	0.021	0.400	-	0.906
LMOD1	AIS	1	-0.070	0.044	0.108	0.932	0.610	-	No	1	-0.070	0.044	0.108	-	0.610
M6PR	AIS	1	0.027	0.039	0.484	1.028	0.957	-	No	1	0.027	0.039	0.484	-	0.957
MAP4K5	AIS	1	0.031	0.042	0.462	1.031	0.957	-	No	1	0.031	0.042	0.462	-	0.957
MFGE8	AIS	2	0.003	0.021	0.881	1.003	0.996	-	No	2	0.003	0.021	0.881	-	0.996
MPHOSPH8	AIS	1	0.039	0.176	0.827	1.039	0.996	-	No	1	0.039	0.176	0.827	-	0.996
MSRA	AIS	1	0.034	0.140	0.808	1.035	0.996	-	No	1	0.034	0.140	0.808	-	0.996
MST1	AIS	2	0.002	0.011	0.875	1.002	0.996	-	No	2	0.002	0.011	0.875	-	0.996
NADK	AIS	1	-0.055	0.031	0.081	0.947	0.543	-	No	1	-0.055	0.031	0.081	-	0.543
NGF	AIS	1	-0.025	0.251	0.919	0.975	0.996	-	No	1	-0.025	0.251	0.919	-	0.996
NOMO1	AIS	1	-0.014	0.071	0.841	0.986	0.996	-	No	1	-0.014	0.071	0.841	-	0.996
NOS3	AIS	1	-0.528	0.169	0.002	0.590	0.028	-	Yes	1	-0.528	0.169	0.002	-	0.028
NPPB	AIS	1	-0.022	0.050	0.665	0.979	0.970	-	No	1	-0.022	0.050	0.665	-	0.970
NUCB2	AIS	2	-0.026	0.056	0.640	0.974	0.967	-	No	2	-0.026	0.056	0.640	-	0.967
PAM	AIS	4	-0.003	0.016	0.852	0.997	0.996	0.787	No	4	-0.003	0.016	0.852	0.323	0.996
PARP1	AIS	1	0.034	0.058	0.556	1.035	0.957	-	No	1	0.034	0.058	0.556	-	0.957
PCOLCE	AIS	1	-0.047	0.045	0.305	0.954	0.796	-	No	1	-0.047	0.045	0.305	-	0.796
PCSK7	AIS	2	0.019	0.030	0.538	1.019	0.957	-	No	2	0.019	0.030	0.538	-	0.957
PDE5A	AIS	2	-0.061	0.045	0.176	0.941	0.725	-	No	2	-0.061	0.045	0.176	-	0.725
PDIA3	AIS	1	0.009	0.188	0.962	1.009	0.996	-	No	1	0.009	0.188	0.962	-	0.996
PECAM1	AIS	1	-0.270	0.219	0.217	0.763	0.729	-	No	1	-0.270	0.219	0.217	-	0.729
PFKFB2	AIS	1	0.013	0.040	0.739	1.014	0.978	-	No	1	0.013	0.040	0.739	-	0.978
PLA2G1B	AIS	1	-0.084	0.149	0.571	0.919	0.967	-	No	1	-0.084	0.149	0.571	-	0.967
PLXDC2	AIS	3	0.021	0.158	0.892	1.022	0.996	0.165	No	3	0.021	0.158	0.892	0.008	0.996
PMS1	AIS	1	-0.011	0.158	0.946	0.989	0.996	-	No	1	-0.011	0.158	0.946	-	0.996
PRDX1	AIS	1	-0.073	0.120	0.542	0.929	0.957	-	No	1	-0.073	0.120	0.542	-	0.957
PROCR	AIS	1	-0.100	0.016	0.000	0.905	0.000	-	Yes	1	-0.100	0.016	0.000	-	0.000
PRSS53	AIS	3	0.020	0.010	0.049	1.021	0.342	0.900	No	3	0.020	0.010	0.049	0.714	0.342
PRTFDC1	AIS	2	0.010	0.035	0.776	1.010	0.996	-	No	2	0.010	0.035	0.776	-	0.996
PSRC1	AIS	2	0.051	0.037	0.169	1.052	0.725	-	No	2	0.051	0.037	0.169	-	0.725
PTPRF	AIS	1	-0.200	0.096	0.038	0.819	0.285	-	No	1	-0.200	0.096	0.038	-	0.285
PTRHD1	AIS	1	-0.173	0.193	0.371	0.841	0.904	-	No	1	-0.173	0.193	0.371	-	0.904
PYDC1	AIS	1	-0.036	0.025	0.152	0.965	0.713	-	No	1	-0.036	0.025	0.152	-	0.713
RELT	AIS	2	0.030	0.027	0.265	1.030	0.794	-	No	2	0.030	0.027	0.265	-	0.794
SDC1	AIS	2	0.055	0.051	0.281	1.056	0.794	-	No	2	0.055	0.051	0.281	-	0.794
SDCCAG8	AIS	1	0.001	0.036	0.978	1.001	0.997	-	No	1	0.001	0.036	0.978	-	0.997
SDHB	AIS	1	0.018	0.124	0.883	1.018	0.996	-	No	1	0.018	0.124	0.883	-	0.996
SERPINI1	AIS	3	-0.027	0.019	0.149	0.974	0.713	0.875	No	3	-0.027	0.019	0.149	0.606	0.713
SH2B3	AIS	1	0.551	0.104	0.000	1.735	0.000	-	Yes	1	0.551	0.104	0.000	-	0.000
SHMT1	AIS	4	0.007	0.011	0.503	1.007	0.957	0.900	No	4	0.007	0.011	0.503	0.678	0.957
SLC16A1	AIS	1	-0.177	0.118	0.134	0.837	0.665	-	No	1	-0.177	0.118	0.134	-	0.665
SLC9A3R2	AIS	1	-0.041	0.073	0.569	0.959	0.967	-	No	1	-0.041	0.073	0.569	-	0.967
SMTN	AIS	1	-0.247	0.195	0.205	0.781	0.729	-	No	1	-0.247	0.195	0.205	-	0.729
SPINK8	AIS	1	0.021	0.018	0.231	1.021	0.729	-	No	1	0.021	0.018	0.231	-	0.729

ST10; MR causal estimates for DBP-associated proteins on ischemic stroke.

Causal candidates prioritized for AIS were marked as "Yes" in column "Prioritized". All estimates are from inverse variance weighted method (IVs >1) or Wald-ratio method (IV = 1). AIS, ischemic stroke.

										Steiger filtering					
**Exposure**	**Outcme**	**nsnp**	**Beta**	**SE**	**P-value**	**Odds ratio**	**FDR-corrected P-value**	**FDR-corrected Cochran's Q P-value**	**Prioritized**	**nsnp**	**Beta**	**SE**	**P-value**	**Cochran's Q P-value**	**FDR-corrected P-value**
STC1	AIS	1	0.089	0.140	0.525	1.093	0.957	-	No	1	0.089	0.140	0.525	-	0.957
STX4	AIS	1	0.374	0.171	0.029	1.454	0.258	-	No	1	0.374	0.171	0.029	-	0.258
TBC1D17	AIS	2	-0.041	0.019	0.032	0.960	0.258	-	No	2	-0.041	0.019	0.032	-	0.258
TIE1	AIS	1	0.000	0.041	1.000	1.000	1.000	-	No	1	0.000	0.041	1.000	-	1.000
TJAP1	AIS	1	-0.597	0.176	0.001	0.551	0.014	-	Yes	1	-0.597	0.176	0.001	-	0.014
TMEM106A	AIS	1	0.005	0.071	0.945	1.005	0.996	-	No	1	0.005	0.071	0.945	-	0.996
TNFRSF17	AIS	1	0.025	0.054	0.641	1.026	0.967	-	No	1	0.025	0.054	0.641	-	0.967
TNFSF12	AIS	2	-0.008	0.020	0.709	0.992	0.978	-	No	2	-0.008	0.020	0.709	-	0.978
TP53	AIS	1	-0.205	0.126	0.104	0.815	0.610	-	No	1	-0.205	0.126	0.104	-	0.610
UMOD	AIS	6	0.005	0.009	0.582	1.005	0.967	0.875	No	6	0.005	0.009	0.582	0.560	0.967
UXS1	AIS	2	0.090	0.060	0.129	1.095	0.659	-	No	2	0.090	0.060	0.129	-	0.659
VAT1	AIS	1	-0.019	0.050	0.701	0.981	0.978	-	No	1	-0.019	0.050	0.701	-	0.978
VSIG2	AIS	1	0.053	0.039	0.173	1.054	0.725	-	No	1	0.053	0.039	0.173	-	0.725
WNT9A	AIS	2	0.047	0.163	0.775	1.048	0.996	-	No	2	0.047	0.163	0.775	-	0.996
WWP2	AIS	1	0.013	0.046	0.777	1.013	0.996	-	No	1	0.013	0.046	0.777	-	0.996
YAP1	AIS	1	-0.068	0.112	0.543	0.934	0.957	-	No	1	-0.068	0.112	0.543	-	0.957
YOD1	AIS	1	0.049	0.067	0.462	1.050	0.957	-	No	1	0.049	0.067	0.462	-	0.957
ZBTB17	AIS	1	-0.451	0.204	0.027	0.637	0.256	-	No	1	-0.451	0.204	0.027	-	0.256

nsnp = number of single nucleotide polymorphisms; SE = standard error; Cochran's Q P-value = p-value from Cochran's Q test assessing heterogeneity; FDR=false discovery rate

ST11; MR causal estimates for SBP-associated proteins on small vessel stroke.

Causal candidates prioritized for SVS were marked as "Yes" in column "Prioritized". All estimates are from inverse variance weighted method (IVs >1) or Wald-ratio method (IV = 1). SVS, small vessel stroke.

										Steiger filtering					
**Exposure**	**Outocme**	**nsnp**	**Beta**	**SE**	**P-value**	**Odds ratio**	**FDR-corrected P-value**	**FDR-corrected Cochran's Q P-value**	**Prioritized**	**nsnp**	**Beta**	**SE**	**P-value**	**Cochran's Q P-value**	**FDR-corrected P-value**
ACOX1	SVS	1	-0.274	0.277	0.322	0.760	0.919	-	No	1	-0.274	0.277	0.322	-	0.919
ACRBP	SVS	1	-0.101	0.149	0.496	0.904	0.973	-	No	1	-0.101	0.149	0.496	-	0.973
ADAM23	SVS	4	-0.005	0.032	0.880	0.995	1.000	0.852	No	4	-0.005	0.032	0.880	0.639	1.000
ADAMTS1	SVS	1	0.820	0.495	0.097	2.271	0.640	-	No	1	0.820	0.495	0.097	-	0.640
ADAMTS4	SVS	1	-0.004	0.168	0.980	0.996	1.000	-	No	1	-0.004	0.168	0.980	-	1.000
ADAMTS8	SVS	4	-0.021	0.044	0.626	0.979	0.973	0.969	No	4	-0.021	0.044	0.626	0.969	0.973
ADAMTSL5	SVS	1	-0.017	0.062	0.789	0.983	1.000	-	No	1	-0.017	0.062	0.789	-	1.000
ADM	SVS	1	-0.572	0.561	0.308	0.564	0.902	-	No	1	-0.572	0.561	0.308	-	0.902
AMOTL2	SVS	1	0.204	0.402	0.612	1.226	0.973	-	No	1	0.204	0.402	0.612	-	0.973
ANKMY2	SVS	1	0.008	0.309	0.978	1.008	1.000	-	No	1	0.008	0.309	0.978	-	1.000
APOA1	SVS	1	0.341	0.312	0.275	1.406	0.856	-	No	1	0.341	0.312	0.275	-	0.856
APOA2	SVS	1	0.117	0.313	0.708	1.124	1.000	-	No	1	0.117	0.313	0.708	-	1.000
APOBR	SVS	2	-0.005	0.020	0.788	0.995	1.000	-	No	2	-0.005	0.020	0.788	-	1.000
APOC1	SVS	2	0.065	0.339	0.848	1.067	1.000	-	No	2	0.065	0.339	0.848	-	1.000
ASPN	SVS	2	-0.013	0.086	0.882	0.987	1.000	-	No	2	-0.013	0.086	0.882	-	1.000
ATXN2L	SVS	1	0.305	0.574	0.595	1.357	0.973	-	No	1	0.305	0.574	0.595	-	0.973
B4GAT1	SVS	3	0.006	0.081	0.939	1.006	1.000	0.817	No	3	0.006	0.081	0.939	0.583	1.000
BAG4	SVS	1	-2.046	0.550	0.000	0.129	0.033	-	Yes	1	-2.046	0.550	0.000	-	0.033
BCAM	SVS	3	0.153	0.109	0.163	1.165	0.767	0.557	No	3	0.153	0.109	0.163	0.272	0.767
BMP6	SVS	2	0.071	0.153	0.642	1.074	0.974	-	No	2	0.071	0.153	0.642	-	0.974
BNIP3L	SVS	1	-0.125	0.400	0.755	0.883	1.000	-	No	1	-0.125	0.400	0.755	-	1.000
BRAP	SVS	1	1.958	0.559	0.000	7.083	0.033	-	Yes	1	1.958	0.559	0.000	-	0.033
BRSK2	SVS	1	-0.876	0.424	0.039	0.416	0.460	-	No	1	-0.876	0.424	0.039	-	0.460
CA12	SVS	2	-0.107	0.069	0.123	0.899	0.701	-	No	2	-0.107	0.069	0.123	-	0.701
CA9	SVS	2	-0.121	0.157	0.443	0.886	0.973	-	No	2	-0.121	0.157	0.443	-	0.973
CACNB3	SVS	1	0.219	0.202	0.277	1.245	0.856	-	No	1	0.219	0.202	0.277	-	0.856
CALCA	SVS	1	-0.276	0.197	0.161	0.759	0.767	-	No	1	-0.276	0.197	0.161	-	0.767
CALCOCO2	SVS	1	-0.146	0.380	0.701	0.865	1.000	-	No	1	-0.146	0.380	0.701	-	1.000
CCN3	SVS	3	0.010	0.126	0.939	1.010	1.000	0.557	No	3	0.010	0.126	0.939	0.081	1.000
CD14	SVS	1	0.099	0.073	0.177	1.104	0.780	-	No	1	0.099	0.073	0.177	-	0.780
CD164L2	SVS	1	0.008	0.048	0.875	1.008	1.000	-	No	1	0.008	0.048	0.875	-	1.000
CD46	SVS	1	0.161	0.259	0.534	1.175	0.973	-	No	1	0.161	0.259	0.534	-	0.973
CD59	SVS	1	-0.136	0.093	0.144	0.873	0.742	-	No	1	-0.136	0.093	0.144	-	0.742
CEP170	SVS	1	-0.055	0.323	0.865	0.946	1.000	-	No	1	-0.055	0.323	0.865	-	1.000
CERT	SVS	1	0.165	0.433	0.704	1.179	1.000	-	No	1	0.165	0.433	0.704	-	1.000
CFHR2	SVS	3	0.046	0.032	0.151	1.047	0.743	0.557	No	3	0.046	0.032	0.151	0.136	0.743
CFHR4	SVS	6	0.056	0.028	0.042	1.058	0.460	0.925	No	6	0.056	0.028	0.042	0.859	0.460
CLMP	SVS	2	-0.072	0.066	0.273	0.930	0.856	-	No	2	-0.072	0.066	0.273	-	0.856
COL1A1	SVS	1	0.452	0.464	0.330	1.571	0.931	-	No	1	0.452	0.464	0.330	-	0.931
COMP	SVS	1	0.519	0.327	0.113	1.681	0.701	-	No	1	0.519	0.327	0.113	-	0.701
CPXM1	SVS	1	-0.004	0.067	0.948	0.996	1.000	-	No	1	-0.004	0.067	0.948	-	1.000
CTSO	SVS	1	-0.173	0.112	0.125	0.841	0.701	-	No	1	-0.173	0.112	0.125	-	0.701
DAG1	SVS	1	-0.544	0.357	0.127	0.580	0.701	-	No	1	-0.544	0.357	0.127	-	0.701
DDHD2	SVS	1	-0.610	0.211	0.004	0.543	0.212	-	No	1	-0.610	0.211	0.004	-	0.212
DTX3	SVS	1	-0.331	0.226	0.143	0.719	0.742	-	No	1	-0.331	0.226	0.143	-	0.742
DUSP13	SVS	1	-0.132	0.079	0.095	0.876	0.640	-	No	1	-0.132	0.079	0.095	-	0.640
DUSP29	SVS	1	0.049	0.159	0.758	1.050	1.000	-	No	1	0.049	0.159	0.758	-	1.000
EFEMP1	SVS	2	-0.024	0.172	0.891	0.977	1.000	-	No	2	-0.024	0.172	0.891	-	1.000
EIF4G3	SVS	1	-0.360	0.264	0.173	0.698	0.780	-	No	1	-0.360	0.264	0.173	-	0.780
ELOA	SVS	1	0.488	0.257	0.058	1.630	0.521	-	No	1	0.488	0.257	0.058	-	0.521
ENPEP	SVS	1	-0.744	0.586	0.204	0.475	0.812	-	No	1	-0.744	0.586	0.204	-	0.812
ERI1	SVS	1	-0.091	0.286	0.750	0.913	1.000	-	No	1	-0.091	0.286	0.750	-	1.000
ERP29	SVS	1	2.099	0.599	0.000	8.161	0.033	-	Yes	1	2.099	0.599	0.000	-	0.033
ESAM	SVS	1	-0.396	0.164	0.016	0.673	0.381	-	No	1	-0.396	0.164	0.016	-	0.381
FDX1	SVS	1	0.540	0.445	0.224	1.716	0.826	-	No	1	0.540	0.445	0.224	-	0.826
FES	SVS	1	-0.128	0.157	0.416	0.880	0.973	-	No	1	-0.128	0.157	0.416	-	0.973

ST11; MR causal estimates for SBP-associated proteins on small vessel stroke.

Causal candidates prioritized for SVS were marked as "Yes" in column "Prioritized". All estimates are from inverse variance weighted method (IVs >1) or Wald-ratio method (IV = 1). SVS, small vessel stroke.

										Steiger filtering					
**Exposure**	**Outcme**	**nsnp**	**Beta**	**SE**	**P-value**	**Odds ratio**	**FDR-corrected P-value**	**FDR-corrected Cochran's Q P-value**	**Prioritized**	**nsnp**	**Beta**	**SE**	**P-value**	**Cochran's Q P-value**	**FDR-corrected P-value**
FGF2	SVS	2	-0.680	0.508	0.180	0.506	0.780	-	No	2	-0.680	0.508	0.180	-	0.780
FGF5	SVS	3	0.065	0.032	0.042	1.067	0.460	0.667	No	3	0.065	0.032	0.042	0.405	0.460
FKBP7	SVS	1	0.048	0.294	0.869	1.050	1.000	-	No	1	0.048	0.294	0.869	-	1.000
FN1	SVS	2	-0.109	0.050	0.028	0.897	0.416	-	No	2	-0.109	0.050	0.028	-	0.416
FOXJ3	SVS	1	0.153	0.309	0.621	1.165	0.973	-	No	1	0.153	0.309	0.621	-	0.973
FOXO3	SVS	1	1.338	0.573	0.020	3.812	0.407	-	No	1	1.338	0.573	0.020	-	0.407
FUCA1	SVS	1	-0.012	0.059	0.844	0.988	1.000	-	No	1	-0.012	0.059	0.844	-	1.000
FURIN	SVS	1	0.027	0.114	0.813	1.027	1.000	-	No	1	0.027	0.114	0.813	-	1.000
GCHFR	SVS	1	0.010	0.185	0.957	1.010	1.000	-	No	1	0.010	0.185	0.957	-	1.000
GFER	SVS	1	-0.739	0.358	0.039	0.478	0.460	-	No	1	-0.739	0.358	0.039	-	0.460
GHR	SVS	2	-0.019	0.032	0.559	0.981	0.973	-	No	2	-0.019	0.032	0.559	-	0.973
GIMAP7	SVS	3	0.026	0.039	0.506	1.026	0.973	0.884	No	3	0.026	0.039	0.506	0.758	0.973
GIT1	SVS	1	-0.238	0.469	0.612	0.788	0.973	-	No	1	-0.238	0.469	0.612	-	0.973
GORASP2	SVS	1	-0.384	0.414	0.354	0.681	0.972	-	No	1	-0.384	0.414	0.354	-	0.972
GRP	SVS	4	-0.014	0.065	0.832	0.986	1.000	0.557	No	4	-0.014	0.065	0.832	0.298	1.000
HADH	SVS	1	0.652	0.371	0.079	1.920	0.584	-	No	1	0.652	0.371	0.079	-	0.584
HHEX	SVS	1	0.160	0.546	0.770	1.173	1.000	-	No	1	0.160	0.546	0.770	-	1.000
HYAL1	SVS	1	-0.049	0.082	0.552	0.952	0.973	-	No	1	-0.049	0.082	0.552	-	0.973
ICAM2	SVS	1	0.249	0.400	0.534	1.283	0.973	-	No	1	0.249	0.400	0.534	-	0.973
IDUA	SVS	1	-0.026	0.042	0.530	0.974	0.973	-	No	1	-0.026	0.042	0.530	-	0.973
IFI30	SVS	2	-0.015	0.070	0.824	0.985	1.000	-	No	2	-0.015	0.070	0.824	-	1.000
IFNGR2	SVS	4	0.044	0.023	0.053	1.045	0.501	0.557	No	4	0.044	0.023	0.053	0.291	0.501
IGFBP3	SVS	2	-0.063	0.173	0.715	0.939	1.000	-	No	2	-0.063	0.173	0.715	-	1.000
IMMT	SVS	1	-0.118	0.182	0.516	0.888	0.973	-	No	1	-0.118	0.182	0.516	-	0.973
IMPA1	SVS	1	0.216	0.106	0.042	1.241	0.460	-	No	1	0.216	0.106	0.042	-	0.460
ITGAL	SVS	1	0.223	0.666	0.737	1.250	1.000	-	No	1	0.223	0.666	0.737	-	1.000
ITIH1	SVS	1	-0.073	0.152	0.632	0.930	0.973	-	No	1	-0.073	0.152	0.632	-	0.973
KIFBP	SVS	1	0.127	0.470	0.787	1.135	1.000	-	No	1	0.127	0.470	0.787	-	1.000
LMOD1	SVS	1	-0.282	0.123	0.022	0.754	0.407	-	No	1	-0.282	0.123	0.022	-	0.407
LRIG1	SVS	3	-0.008	0.033	0.814	0.992	1.000	0.557	No	3	-0.008	0.033	0.814	0.198	1.000
LYAR	SVS	1	-0.086	0.462	0.852	0.917	1.000	-	No	1	-0.086	0.462	0.852	-	1.000
M6PR	SVS	1	0.001	0.114	0.993	1.001	1.000	-	No	1	0.001	0.114	0.993	-	1.000
MANEAL	SVS	1	-0.683	0.572	0.233	0.505	0.842	-	No	1	-0.683	0.572	0.233	-	0.842
MANSC4	SVS	2	0.020	0.025	0.429	1.020	0.973	-	No	2	0.020	0.025	0.429	-	0.973
MAP4K5	SVS	1	-0.105	0.117	0.371	0.900	0.973	-	No	1	-0.105	0.117	0.371	-	0.973
MDH1	SVS	2	-0.242	0.224	0.280	0.785	0.856	-	No	2	-0.242	0.224	0.280	-	0.856
MEGF9	SVS	1	-0.184	0.068	0.007	0.832	0.250	-	No	1	-0.184	0.068	0.007	-	0.250
MPI	SVS	1	-0.257	0.095	0.007	0.773	0.250	-	No	1	-0.257	0.095	0.007	-	0.250
MSRA	SVS	1	0.032	0.365	0.930	1.033	1.000	-	No	1	0.032	0.365	0.930	-	1.000
MST1	SVS	2	0.018	0.062	0.767	1.019	1.000	-	No	2	0.018	0.062	0.767	-	1.000
MVK	SVS	1	-0.207	0.241	0.389	0.813	0.973	-	No	1	-0.207	0.241	0.389	-	0.973
NADK	SVS	1	-0.037	0.085	0.660	0.963	0.988	-	No	1	-0.037	0.085	0.660	-	0.988
NAGA	SVS	2	-0.110	0.132	0.407	0.896	0.973	-	No	2	-0.110	0.132	0.407	-	0.973
NFE2	SVS	1	-0.531	0.304	0.081	0.588	0.584	-	No	1	-0.531	0.304	0.081	-	0.584
NTRK3	SVS	2	-0.028	0.098	0.780	0.973	1.000	-	No	2	-0.028	0.098	0.780	-	1.000
NUCB2	SVS	2	0.034	0.148	0.818	1.035	1.000	-	No	2	0.034	0.148	0.818	-	1.000
NUDT5	SVS	1	0.005	0.369	0.988	1.005	1.000	-	No	1	0.005	0.369	0.988	-	1.000
NUMB	SVS	1	0.021	0.233	0.928	1.021	1.000	-	No	1	0.021	0.233	0.928	-	1.000
OGA	SVS	1	-0.078	0.177	0.659	0.925	0.988	-	No	1	-0.078	0.177	0.659	-	0.988
OPLAH	SVS	1	-0.147	0.114	0.197	0.863	0.807	-	No	1	-0.147	0.114	0.197	-	0.807
PCBP2	SVS	1	-0.726	0.416	0.081	0.484	0.584	-	No	1	-0.726	0.416	0.081	-	0.584
PCSK7	SVS	2	0.001	0.062	0.991	1.001	1.000	-	No	2	0.001	0.062	0.991	-	1.000
PDE5A	SVS	2	0.013	0.226	0.954	1.013	1.000	-	No	2	0.013	0.226	0.954	-	1.000
PDGFRA	SVS	1	-0.054	0.460	0.907	0.948	1.000	-	No	1	-0.054	0.460	0.907	-	1.000
PECAM1	SVS	1	-0.674	0.651	0.301	0.510	0.894	-	No	1	-0.674	0.651	0.301	-	0.894
PFKFB2	SVS	1	-0.081	0.112	0.468	0.922	0.973	-	No	1	-0.081	0.112	0.468	-	0.973

ST11; MR causal estimates for SBP-associated proteins on small vessel stroke.

Causal candidates prioritized for SVS were marked as "Yes" in column "Prioritized". All estimates are from inverse variance weighted method (IVs >1) or Wald-ratio method (IV = 1). SVS, small vessel stroke.

										Steiger filtering					
Exposure	Outcocme	nsnp	Beta	SE	P-value	Odds ratio	FDR-corrected P-value	FDR-corrected Cochran's Q P-value	Prioritized	nsnp	Beta	SE	P-value	Cochran's Q P-value	FDR-corrected P-value
PGF	SVS	1	-0.080	0.134	0.552	0.923	0.973	-	No	1	-0.080	0.134	0.552	-	0.973
PHLDB1	SVS	1	0.325	0.451	0.472	1.384	0.973	-	No	1	0.325	0.451	0.472	-	0.973
PKD1	SVS	1	-0.096	0.466	0.836	0.908	1.000	-	No	1	-0.096	0.466	0.836	-	1.000
PLA2G1B	SVS	1	-0.532	0.403	0.187	0.588	0.780	-	No	1	-0.532	0.403	0.187	-	0.780
PMS1	SVS	1	0.278	0.440	0.527	1.321	0.973	-	No	1	0.278	0.440	0.527	-	0.973
PPP1R14D	SVS	1	0.436	0.372	0.241	1.547	0.854	-	No	1	0.436	0.372	0.241	-	0.854
PRG2	SVS	1	0.059	0.211	0.781	1.060	1.000	-	No	1	0.059	0.211	0.781	-	1.000
PRKAB1	SVS	1	0.084	0.124	0.501	1.087	0.973	-	No	1	0.084	0.124	0.501	-	0.973
PSMD5	SVS	1	0.966	0.423	0.023	2.626	0.407	-	No	1	0.966	0.423	0.023	-	0.407
PSRC1	SVS	1	0.240	0.189	0.206	1.271	0.812	-	No	1	0.240	0.189	0.206	-	0.812
PTRHD1	SVS	1	-0.277	0.533	0.604	0.758	0.973	-	No	1	-0.277	0.533	0.604	-	0.973
QPCT	SVS	3	-0.045	0.087	0.604	0.956	0.973	0.557	No	3	-0.045	0.087	0.604	0.088	0.973
RABEPK	SVS	1	-0.124	0.138	0.371	0.884	0.973	-	No	1	-0.124	0.138	0.371	-	0.973
RARRES1	SVS	5	-0.006	0.070	0.934	0.994	1.000	0.557	No	5	-0.006	0.070	0.934	0.030	1.000
RARRES2	SVS	1	-0.042	0.109	0.701	0.959	1.000	-	No	1	-0.042	0.109	0.701	-	1.000
RELT	SVS	2	0.046	0.086	0.591	1.047	0.973	-	No	2	0.046	0.086	0.591	-	0.973
RSPO3	SVS	1	-0.093	0.130	0.476	0.911	0.973	-	No	1	-0.093	0.130	0.476	-	0.973
SCARA5	SVS	4	-0.069	0.061	0.256	0.933	0.854	0.817	No	4	-0.069	0.061	0.256	0.561	0.854
SDCCAG8	SVS	1	-0.049	0.101	0.629	0.953	0.973	-	No	1	-0.049	0.101	0.629	-	0.973
SDHB	SVS	1	-0.078	0.347	0.822	0.925	1.000	-	No	1	-0.078	0.347	0.822	-	1.000
SELENOP	SVS	1	-0.038	0.202	0.852	0.963	1.000	-	No	1	-0.038	0.202	0.852	-	1.000
SEMA6C	SVS	1	-0.028	0.227	0.901	0.972	1.000	-	No	1	-0.028	0.227	0.901	-	1.000
SERPINI1	SVS	1	-0.003	0.054	0.951	0.997	1.000	-	No	1	-0.003	0.054	0.951	-	1.000
SH2B3	SVS	1	0.766	0.296	0.010	2.151	0.301	-	No	1	0.766	0.296	0.010	-	0.301
SHMT1	SVS	4	-0.007	0.030	0.813	0.993	1.000	0.918	No	4	-0.007	0.030	0.813	0.820	1.000
SIL1	SVS	1	0.521	0.343	0.129	1.683	0.701	-	No	1	0.521	0.343	0.129	-	0.701
SLC16A1	SVS	1	-0.483	0.336	0.151	0.617	0.743	-	No	1	-0.483	0.336	0.151	-	0.743
SLC39A14	SVS	1	-0.396	0.221	0.074	0.673	0.584	-	No	1	-0.396	0.221	0.074	-	0.584
SLC9A3R2	SVS	1	-0.156	0.212	0.462	0.856	0.973	-	No	1	-0.156	0.212	0.462	-	0.973
SMOC2	SVS	3	-0.014	0.064	0.829	0.986	1.000	0.557	No	3	-0.014	0.064	0.829	0.264	1.000
SOST	SVS	1	0.304	0.402	0.449	1.356	0.973	-	No	1	0.304	0.402	0.449	-	0.973
SPINK8	SVS	1	-0.006	0.051	0.909	0.994	1.000	-	No	1	-0.006	0.051	0.909	-	1.000
SPRED2	SVS	1	0.491	0.317	0.122	1.634	0.701	-	No	1	0.491	0.317	0.122	-	0.701
TARBP2	SVS	1	-0.780	0.447	0.081	0.458	0.584	-	No	1	-0.780	0.447	0.081	-	0.584
TBC1D23	SVS	1	0.160	0.130	0.218	1.174	0.826	-	No	1	0.160	0.130	0.218	-	0.826
TEK	SVS	3	0.088	0.071	0.217	1.092	0.826	0.557	No	3	0.088	0.071	0.217	0.153	0.826
TGFB2	SVS	1	0.121	0.148	0.412	1.129	0.973	-	No	1	0.121	0.148	0.412	-	0.973
TIE1	SVS	1	-0.003	0.107	0.981	0.997	1.000	-	No	1	-0.003	0.107	0.981	-	1.000
TJAP1	SVS	1	-0.771	0.502	0.124	0.463	0.701	-	No	1	-0.771	0.502	0.124	-	0.701
TNFRSF17	SVS	1	-0.017	0.155	0.911	0.983	1.000	-	No	1	-0.017	0.155	0.911	-	1.000
TNFSF12	SVS	2	0.046	0.058	0.429	1.047	0.973	-	No	2	0.046	0.058	0.429	-	0.973
TWF2	SVS	1	-0.050	0.325	0.877	0.951	1.000	-	No	1	-0.050	0.325	0.877	-	1.000
UBE2L6	SVS	2	0.069	0.084	0.413	1.071	0.973	-	No	2	0.069	0.084	0.413	-	0.973
UMOD	SVS	4	-0.058	0.034	0.087	0.943	0.608	0.557	No	4	-0.058	0.034	0.087	0.169	0.608
VSIG2	SVS	1	0.268	0.111	0.016	1.307	0.381	-	No	1	0.268	0.111	0.016	-	0.381
WARS	SVS	1	0.221	0.101	0.029	1.248	0.416	-	No	1	0.221	0.101	0.029	-	0.416
WASHC3	SVS	1	0.133	0.274	0.627	1.142	0.973	-	No	1	0.133	0.274	0.627	-	0.973
WWP2	SVS	1	-0.147	0.132	0.269	0.864	0.856	-	No	1	-0.147	0.132	0.269	-	0.856
YOD1	SVS	1	0.099	0.189	0.600	1.104	0.973	-	No	1	0.099	0.189	0.600	-	0.973
ZFYVE19	SVS	1	-0.041	0.055	0.457	0.960	0.973	-	No	1	-0.041	0.055	0.457	-	0.973

nsnp = number of single nucleotide polymorphisms; SE = standard error; Cochran's Q P-value = p-value from Cochran's Q test assessing heterogeneity; FDR=false discovery rate

ST12; MR causal estimates for DBP-associated proteins on small vessel stroke.

Causal candidates prioritized for SVS were marked as "Yes" in column "Prioritized". All estimates are from inverse variance weighted method (IVs >1) or Wald-ratio method (IV = 1). SVS, small vessel stroke.

										Steiger filtering					
Exposure	Outcme	nsnp	Beta	SE	P-value	Odds ratio	FDR-corrected P-value	FDR-corrected Cochran's Q P-value	Prioritized	nsnp	Beta	SE	P-value	Cochran's Q P-value	FDR-corrected P-value
AAMDC	SVS	1	0.018	0.031	0.549	1.019	0.973	-	No	1	0.018	0.031	0.549	-	0.973
ABO	SVS	6	-0.001	0.019	0.965	0.999	1.000	0.876	No	6	-0.001	0.019	0.965	0.719	1.000
ACOX1	SVS	1	-0.274	0.277	0.322	0.760	0.919	-	No	1	-0.274	0.277	0.322	-	0.919
ADM	SVS	1	-0.572	0.561	0.308	0.564	0.902	-	No	1	-0.572	0.561	0.308	-	0.902
AMFR	SVS	1	0.511	0.491	0.298	1.667	0.894	-	No	1	0.511	0.491	0.298	-	0.894
AMOTL2	SVS	1	0.204	0.402	0.612	1.226	0.973	-	No	1	0.204	0.402	0.612	-	0.973
ANKMY2	SVS	1	0.008	0.309	0.978	1.008	1.000	-	No	1	0.008	0.309	0.978	-	1.000
AOC1	SVS	3	-0.085	0.075	0.254	0.918	0.854	0.876	No	3	-0.085	0.075	0.254	0.700	0.854
AOC3	SVS	2	0.039	0.040	0.338	1.040	0.941	-	No	2	0.039	0.040	0.338	-	0.941
APOA1	SVS	1	0.341	0.312	0.275	1.406	0.856	-	No	1	0.341	0.312	0.275	-	0.856
APOBR	SVS	2	-0.005	0.020	0.788	0.995	1.000	-	No	2	-0.005	0.020	0.788	-	1.000
APOC1	SVS	2	0.065	0.339	0.848	1.067	1.000	-	No	2	0.065	0.339	0.848	-	1.000
ARSB	SVS	2	-0.081	0.139	0.560	0.922	0.973	-	No	2	-0.081	0.139	0.560	-	0.973
ATXN2L	SVS	1	0.305	0.574	0.595	1.357	0.973	-	No	1	0.305	0.574	0.595	-	0.973
AXL	SVS	2	-0.144	0.126	0.254	0.866	0.854	-	No	2	-0.144	0.126	0.254	-	0.854
BAG4	SVS	1	-2.046	0.550	0.000	0.129	0.033	-	Yes	1	-2.046	0.550	0.000	-	0.033
BNIP3L	SVS	1	-0.125	0.400	0.755	0.883	1.000	-	No	1	-0.125	0.400	0.755	-	1.000
CA12	SVS	2	-0.107	0.069	0.123	0.899	0.701	-	No	2	-0.107	0.069	0.123	-	0.701
CACNB3	SVS	1	0.219	0.202	0.277	1.245	0.856	-	No	1	0.219	0.202	0.277	-	0.856
CALCA	SVS	1	-0.276	0.197	0.161	0.759	0.767	-	No	1	-0.276	0.197	0.161	-	0.767
CEP170	SVS	1	-0.055	0.323	0.865	0.946	1.000	-	No	1	-0.055	0.323	0.865	-	1.000
CFHR4	SVS	6	0.056	0.028	0.042	1.058	0.460	0.925	No	6	0.056	0.028	0.042	0.859	0.460
CHMP1A	SVS	1	0.293	0.241	0.225	1.340	0.826	-	No	1	0.293	0.241	0.225	-	0.826
CLIC5	SVS	1	0.186	0.169	0.269	1.205	0.856	-	No	1	0.186	0.169	0.269	-	0.856
COL1A1	SVS	1	0.452	0.464	0.330	1.571	0.931	-	No	1	0.452	0.464	0.330	-	0.931
CTF1	SVS	1	0.329	0.408	0.420	1.389	0.973	-	No	1	0.329	0.408	0.420	-	0.973
DAG1	SVS	1	-0.544	0.357	0.127	0.580	0.701	-	No	1	-0.544	0.357	0.127	-	0.701
DARS1	SVS	1	0.141	0.294	0.632	1.151	0.973	-	No	1	0.141	0.294	0.632	-	0.973
DBN1	SVS	1	-0.538	0.407	0.186	0.584	0.780	-	No	1	-0.538	0.407	0.186	-	0.780
DDHD2	SVS	1	-0.610	0.211	0.004	0.543	0.212	-	No	1	-0.610	0.211	0.004	-	0.212
DENR	SVS	1	-0.639	0.635	0.314	0.528	0.909	-	No	1	-0.639	0.635	0.314	-	0.909
DNER	SVS	5	-0.052	0.091	0.566	0.949	0.973	0.557	No	5	-0.052	0.091	0.566	0.068	0.973
DOK2	SVS	1	-0.315	0.374	0.400	0.730	0.973	-	No	1	-0.315	0.374	0.400	-	0.973
DPEP1	SVS	4	-0.253	0.139	0.068	0.777	0.584	0.667	No	4	-0.253	0.139	0.068	0.387	0.584
DPP4	SVS	1	-0.040	0.097	0.677	0.961	1.000	-	No	1	-0.040	0.097	0.677	-	1.000
DUSP13	SVS	1	-0.132	0.079	0.095	0.876	0.640	-	No	1	-0.132	0.079	0.095	-	0.640
EFNA1	SVS	1	-0.123	0.064	0.053	0.884	0.501	-	No	1	-0.123	0.064	0.053	-	0.501
EIF4G3	SVS	1	-0.360	0.264	0.173	0.698	0.780	-	No	1	-0.360	0.264	0.173	-	0.780
ELOA	SVS	1	0.488	0.257	0.058	1.630	0.521	-	No	1	0.488	0.257	0.058	-	0.521
ENPEP	SVS	1	-0.744	0.586	0.204	0.475	0.812	-	No	1	-0.744	0.586	0.204	-	0.812
EPHA2	SVS	2	-0.026	0.160	0.868	0.974	1.000	-	No	2	-0.026	0.160	0.868	-	1.000
EPO	SVS	1	-0.182	0.277	0.511	0.834	0.973	-	No	1	-0.182	0.277	0.511	-	0.973
ERI1	SVS	1	-0.091	0.286	0.750	0.913	1.000	-	No	1	-0.091	0.286	0.750	-	1.000
ESAM	SVS	1	-0.396	0.164	0.016	0.673	0.381	-	No	1	-0.396	0.164	0.016	-	0.381
F12	SVS	3	0.018	0.258	0.945	1.018	1.000	0.946	No	3	0.018	0.258	0.945	0.913	1.000
FADD	SVS	1	0.001	0.264	0.996	1.001	1.000	-	No	1	0.001	0.264	0.996	-	1.000
FDX1	SVS	1	0.540	0.445	0.224	1.716	0.826	-	No	1	0.540	0.445	0.224	-	0.826
FES	SVS	1	-0.128	0.157	0.416	0.880	0.973	-	No	1	-0.128	0.157	0.416	-	0.973
FGF5	SVS	3	0.065	0.032	0.042	1.067	0.460	0.667	No	3	0.065	0.032	0.042	0.405	0.460
FOXJ3	SVS	1	0.153	0.309	0.621	1.165	0.973	-	No	1	0.153	0.309	0.621	-	0.973
FURIN	SVS	1	0.027	0.114	0.813	1.027	1.000	-	No	1	0.027	0.114	0.813	-	1.000
GFER	SVS	1	-0.739	0.358	0.039	0.478	0.460	-	No	1	-0.739	0.358	0.039	-	0.460
GIMAP7	SVS	3	0.026	0.039	0.506	1.026	0.973	0.884	No	3	0.026	0.039	0.506	0.758	0.973
GLO1	SVS	1	-0.146	0.370	0.692	0.864	1.000	-	No	1	-0.146	0.370	0.692	-	1.000
GORASP2	SVS	1	-0.384	0.414	0.354	0.681	0.972	-	No	1	-0.384	0.414	0.354	-	0.972

ST12; MR causal estimates for DBP-associated proteins on small vessel stroke.

Causal candidates prioritized for SVS were marked as "Yes" in column "Prioritized". All estimates are from inverse variance weighted method (IVs >1) or Wald-ratio method (IV = 1). SVS, small vessel stroke.

										Steiger filtering					
Exposure	Outocme	nsnp	Beta	SE	P-value	Odds ratio	FDR-corrected P-value	FDR-corrected Cochran's Q P-value	Prioritized	nsnp	Beta	SE	P-value	Cochran's Q P-value	FDR-corrected P-value
GRP	SVS	4	-0.014	0.065	0.832	0.986	1.000	0.557	No	4	-0.014	0.065	0.832	0.298	1.000
HADH	SVS	1	0.652	0.371	0.079	1.920	0.584	-	No	1	0.652	0.371	0.079	-	0.584
HHEX	SVS	1	0.160	0.546	0.770	1.173	1.000	-	No	1	0.160	0.546	0.770	-	1.000
HPGDS	SVS	6	0.002	0.042	0.962	1.002	1.000	0.691	No	6	0.002	0.042	0.962	0.444	1.000
ICAM1	SVS	4	0.037	0.060	0.537	1.038	0.973	0.557	No	4	0.037	0.060	0.537	0.210	0.973
ICAM4	SVS	1	-0.149	0.190	0.430	0.861	0.973	-	No	1	-0.149	0.190	0.430	-	0.973
IFIT3	SVS	1	0.146	0.195	0.453	1.157	0.973	-	No	1	0.146	0.195	0.453	-	0.973
IGFBP3	SVS	2	-0.063	0.173	0.715	0.939	1.000	-	No	2	-0.063	0.173	0.715	-	1.000
IL1RL1	SVS	6	0.001	0.035	0.966	1.001	1.000	0.557	No	6	0.001	0.035	0.966	0.198	1.000
IMMT	SVS	1	-0.118	0.182	0.516	0.888	0.973	-	No	1	-0.118	0.182	0.516	-	0.973
ITGAL	SVS	1	0.223	0.666	0.737	1.250	1.000	-	No	1	0.223	0.666	0.737	-	1.000
KIF22	SVS	1	0.389	0.592	0.511	1.476	0.973	-	No	1	0.389	0.592	0.511	-	0.973
KIFBP	SVS	1	0.127	0.470	0.787	1.135	1.000	-	No	1	0.127	0.470	0.787	-	1.000
LAYN	SVS	1	0.032	0.057	0.572	1.033	0.973	-	No	1	0.032	0.057	0.572	-	0.973
LMOD1	SVS	1	-0.282	0.123	0.022	0.754	0.407	-	No	1	-0.282	0.123	0.022	-	0.407
M6PR	SVS	1	0.001	0.114	0.993	1.001	1.000	-	No	1	0.001	0.114	0.993	-	1.000
MAP4K5	SVS	1	-0.105	0.117	0.371	0.900	0.973	-	No	1	-0.105	0.117	0.371	-	0.973
MFGE8	SVS	2	-0.031	0.061	0.607	0.969	0.973	-	No	2	-0.031	0.061	0.607	-	0.973
MSRA	SVS	1	0.032	0.365	0.930	1.033	1.000	-	No	1	0.032	0.365	0.930	-	1.000
MST1	SVS	2	0.018	0.062	0.767	1.019	1.000	-	No	2	0.018	0.062	0.767	-	1.000
NADK	SVS	1	-0.037	0.085	0.660	0.963	0.988	-	No	1	-0.037	0.085	0.660	-	0.988
NOMO1	SVS	1	0.169	0.209	0.418	1.185	0.973	-	No	1	0.169	0.209	0.418	-	0.973
NOS3	SVS	1	-1.032	0.469	0.028	0.356	0.416	-	No	1	-1.032	0.469	0.028	-	0.416
NUCB2	SVS	2	0.034	0.148	0.818	1.035	1.000	-	No	2	0.034	0.148	0.818	-	1.000
PAM	SVS	2	-0.046	0.054	0.397	0.955	0.973	-	No	2	-0.046	0.054	0.397	-	0.973
PARP1	SVS	1	-0.131	0.171	0.443	0.877	0.973	-	No	1	-0.131	0.171	0.443	-	0.973
PCOLCE	SVS	1	-0.076	0.126	0.545	0.927	0.973	-	No	1	-0.076	0.126	0.545	-	0.973
PCSK7	SVS	2	0.001	0.062	0.991	1.001	1.000	-	No	2	0.001	0.062	0.991	-	1.000
PDE5A	SVS	2	0.013	0.226	0.954	1.013	1.000	-	No	2	0.013	0.226	0.954	-	1.000
PDIA3	SVS	1	-0.031	0.476	0.948	0.970	1.000	-	No	1	-0.031	0.476	0.948	-	1.000
PECAM1	SVS	1	-0.674	0.651	0.301	0.510	0.894	-	No	1	-0.674	0.651	0.301	-	0.894
PFKFB2	SVS	1	-0.081	0.112	0.468	0.922	0.973	-	No	1	-0.081	0.112	0.468	-	0.973
PLA2G1B	SVS	1	-0.532	0.403	0.187	0.588	0.780	-	No	1	-0.532	0.403	0.187	-	0.780
PLXDC2	SVS	3	-0.085	0.231	0.713	0.918	1.000	0.557	No	3	-0.085	0.231	0.713	0.278	1.000
PMS1	SVS	1	0.278	0.440	0.527	1.321	0.973	-	No	1	0.278	0.440	0.527	-	0.973
PRDX1	SVS	1	0.021	0.328	0.950	1.021	1.000	-	No	1	0.021	0.328	0.950	-	1.000
PROCR	SVS	1	-0.043	0.047	0.361	0.958	0.973	-	No	1	-0.043	0.047	0.361	-	0.973
PRSS53	SVS	1	0.163	0.226	0.470	1.177	0.973	-	No	1	0.163	0.226	0.470	-	0.973
PRTFDC1	SVS	1	-0.360	0.421	0.393	0.698	0.973	-	No	1	-0.360	0.421	0.393	-	0.973
PSRC1	SVS	1	0.240	0.189	0.206	1.271	0.812	-	No	1	0.240	0.189	0.206	-	0.812
PTPRF	SVS	1	0.060	0.262	0.820	1.062	1.000	-	No	1	0.060	0.262	0.820	-	1.000
PTRHD1	SVS	1	-0.277	0.533	0.604	0.758	0.973	-	No	1	-0.277	0.533	0.604	-	0.973
PYDC1	SVS	1	-0.048	0.070	0.489	0.953	0.973	-	No	1	-0.048	0.070	0.489	-	0.973
RELT	SVS	2	0.046	0.086	0.591	1.047	0.973	-	No	2	0.046	0.086	0.591	-	0.973
SDC1	SVS	2	-0.163	0.142	0.251	0.849	0.854	-	No	2	-0.163	0.142	0.251	-	0.854
SDCCAG8	SVS	1	-0.049	0.101	0.629	0.953	0.973	-	No	1	-0.049	0.101	0.629	-	0.973
SDHB	SVS	1	-0.078	0.347	0.822	0.925	1.000	-	No	1	-0.078	0.347	0.822	-	1.000
SERPINI1	SVS	1	-0.003	0.054	0.951	0.997	1.000	-	No	1	-0.003	0.054	0.951	-	1.000
SH2B3	SVS	1	0.766	0.296	0.010	2.151	0.301	-	No	1	0.766	0.296	0.010	-	0.301
SHMT1	SVS	4	-0.007	0.030	0.813	0.993	1.000	0.918	No	4	-0.007	0.030	0.813	0.820	1.000
SLC16A1	SVS	1	-0.483	0.336	0.151	0.617	0.743	-	No	1	-0.483	0.336	0.151	-	0.743
SLC9A3R2	SVS	1	-0.156	0.212	0.462	0.856	0.973	-	No	1	-0.156	0.212	0.462	-	0.973
SMTN	SVS	1	-1.071	0.544	0.049	0.343	0.501	-	No	1	-1.071	0.544	0.049	-	0.501
SPINK8	SVS	1	-0.006	0.051	0.909	0.994	1.000	-	No	1	-0.006	0.051	0.909	-	1.000
STC1	SVS	1	0.245	0.392	0.533	1.277	0.973	-	No	1	0.245	0.392	0.533	-	0.973

ST12; MR causal estimates for DBP-associated proteins on small vessel stroke.

Causal candidates prioritized for SVS were marked as "Yes" in column "Prioritized". All estimates are from inverse variance weighted method (IVs >1) or Wald-ratio method (IV = 1). SVS, small vessel stroke.

										Steiger filtering					
Exposure	Outocme	nsnp	Beta	SE	P-value	Odds ratio	FDR-corrected P-value	FDR-corrected Cochran's Q P-value	Prioritized	nsnp	Beta	SE	P-value	Cochran's Q P-value	FDR-corrected P-value
STX4	SVS	1	-0.372	0.497	0.455	0.690	0.973	-	No	1	-0.372	0.497	0.455	-	0.973
TBC1D17	SVS	1	0.000	0.056	1.000	1.000	1.000	-	No	1	0.000	0.056	1.000	-	1.000
TIE1	SVS	1	-0.003	0.107	0.981	0.997	1.000	-	No	1	-0.003	0.107	0.981	-	1.000
TJAP1	SVS	1	-0.771	0.502	0.124	0.463	0.701	-	No	1	-0.771	0.502	0.124	-	0.701
TMEM106A	SVS	1	0.060	0.201	0.766	1.062	1.000	-	No	1	0.060	0.201	0.766	-	1.000
TNFRSF17	SVS	1	-0.017	0.155	0.911	0.983	1.000	-	No	1	-0.017	0.155	0.911	-	1.000
TNFSF12	SVS	2	0.046	0.058	0.429	1.047	0.973	-	No	2	0.046	0.058	0.429	-	0.973
TP53	SVS	1	-0.498	0.361	0.168	0.608	0.774	-	No	1	-0.498	0.361	0.168	-	0.774
UMOD	SVS	4	-0.058	0.034	0.087	0.943	0.608	0.557	No	4	-0.058	0.034	0.087	0.169	0.608
UXS1	SVS	2	0.008	0.246	0.976	1.008	1.000	-	No	2	0.008	0.246	0.976	-	1.000
VAT1	SVS	1	0.006	0.142	0.969	1.006	1.000	-	No	1	0.006	0.142	0.969	-	1.000
VSIG2	SVS	1	0.268	0.111	0.016	1.307	0.381	-	No	1	0.268	0.111	0.016	-	0.381
WNT9A	SVS	2	-0.072	0.153	0.637	0.930	0.974	-	No	2	-0.072	0.153	0.637	-	0.974
WWP2	SVS	1	-0.147	0.132	0.269	0.864	0.856	-	No	1	-0.147	0.132	0.269	-	0.856
YAP1	SVS	1	0.112	0.315	0.722	1.119	1.000	-	No	1	0.112	0.315	0.722	-	1.000
YOD1	SVS	1	0.099	0.189	0.600	1.104	0.973	-	No	1	0.099	0.189	0.600	-	0.973

nsnp = number of single nucleotide polymorphisms; SE = standard error; Cochran's Q P-value = p-value from Cochran's Q test assessing heterogeneity; FDR=false discovery rate

ST13; MR causal estimates for SBP-associated proteins on cardioembolic stroke.

Causal candidates prioritized for CES were marked as "Yes" in column "Prioritized". All estimates are from inverse variance weighted method (IVs >1) or Wald-ratio method (IV = 1). CES, cardioembolic stroke.

										Steiger filtering					
Exposure	Outocme	nsnp	Beta	SE	P-value	Odds ratio	FDR-corrected P-value	FDR-corrected Cochran's Q P-value	Prioritized	nsnp	Beta	SE	P-value	Cochran's Q P-value	FDR-corrected P-value
ACOX1	CES	1	0.200	0.226	0.376	1.221	0.839	-	No	1	0.200	0.226	0.376	-	0.839
ACRBP	CES	1	0.210	0.118	0.075	1.234	0.621	-	No	1	0.210	0.118	0.075	-	0.621
ADAM23	CES	4	0.039	0.026	0.131	1.040	0.699	0.955	No	4	0.039	0.026	0.131	0.803	0.699
ADAMTS1	CES	1	0.190	0.409	0.642	1.210	0.951	-	No	1	0.190	0.409	0.642	-	0.951
ADAMTS4	CES	1	0.171	0.138	0.214	1.187	0.787	-	No	1	0.171	0.138	0.214	-	0.787
ADAMTS8	CES	5	0.039	0.056	0.487	1.040	0.845	0.583	No	5	0.039	0.056	0.487	0.059	0.845
ADAMTSL5	CES	2	0.046	0.049	0.345	1.048	0.833	-	No	2	0.046	0.049	0.345	-	0.833
ADM	CES	1	-0.207	0.450	0.646	0.813	0.951	-	No	1	-0.207	0.450	0.646	-	0.951
AMOTL2	CES	1	-0.107	0.326	0.743	0.899	0.966	-	No	1	-0.107	0.326	0.743	-	0.966
ANKMY2	CES	1	-0.205	0.243	0.400	0.815	0.839	-	No	1	-0.205	0.243	0.400	-	0.839
APOA1	CES	1	-0.042	0.253	0.867	0.958	0.989	-	No	1	-0.042	0.253	0.867	-	0.989
APOA2	CES	1	-0.281	0.258	0.276	0.755	0.805	-	No	1	-0.281	0.258	0.276	-	0.805
APOBR	CES	3	-0.033	0.033	0.328	0.968	0.833	0.252	No	3	-0.033	0.033	0.328	0.009	0.833
APOC1	CES	2	-0.190	0.225	0.399	0.827	0.839	-	No	2	-0.190	0.225	0.399	-	0.839
ASPN	CES	2	0.103	0.149	0.489	1.108	0.845	-	No	2	0.103	0.149	0.489	-	0.845
ATXN2L	CES	1	0.563	0.474	0.236	1.755	0.805	-	No	1	0.563	0.474	0.236	-	0.805
B4GAT1	CES	3	-0.015	0.103	0.882	0.985	0.989	0.583	No	3	-0.015	0.103	0.882	0.067	0.989
BAG4	CES	1	-0.634	0.438	0.148	0.531	0.715	-	No	1	-0.634	0.438	0.148	-	0.715
BCAM	CES	3	0.021	0.071	0.768	1.021	0.966	0.955	No	3	0.021	0.071	0.768	0.771	0.966
BMP6	CES	2	-0.017	0.164	0.917	0.983	0.989	-	No	2	-0.017	0.164	0.917	-	0.989
BNIP3L	CES	1	-0.363	0.318	0.254	0.696	0.805	-	No	1	-0.363	0.318	0.254	-	0.805
BRAP	CES	1	1.193	0.457	0.009	3.296	0.208	-	No	1	1.193	0.457	0.009	-	0.208
BRSK2	CES	1	-0.605	0.347	0.082	0.546	0.621	-	No	1	-0.605	0.347	0.082	-	0.621
CA12	CES	2	-0.031	0.056	0.579	0.969	0.889	-	No	2	-0.031	0.056	0.579	-	0.889
CA9	CES	2	0.220	0.143	0.124	1.246	0.695	-	No	2	0.220	0.143	0.124	-	0.695
CACNB3	CES	1	-0.006	0.166	0.972	0.994	0.994	-	No	1	-0.006	0.166	0.972	-	0.994
CALCA	CES	1	0.069	0.160	0.665	1.072	0.951	-	No	1	0.069	0.160	0.665	-	0.951
CALCOCO2	CES	1	0.899	0.291	0.002	2.458	0.092	-	No	1	0.899	0.291	0.002	-	0.092
CCN3	CES	3	-0.038	0.065	0.554	0.962	0.869	0.906	No	3	-0.038	0.065	0.554	0.500	0.869
CD14	CES	1	0.001	0.058	0.987	1.001	0.994	-	No	1	0.001	0.058	0.987	-	0.994
CD164L2	CES	1	-0.035	0.039	0.368	0.966	0.839	-	No	1	-0.035	0.039	0.368	-	0.839
CD46	CES	1	-0.194	0.211	0.357	0.823	0.833	-	No	1	-0.194	0.211	0.357	-	0.833
CD59	CES	1	0.063	0.075	0.403	1.065	0.839	-	No	1	0.063	0.075	0.403	-	0.839
CEP170	CES	1	0.120	0.262	0.648	1.127	0.951	-	No	1	0.120	0.262	0.648	-	0.951
CERT	CES	1	0.486	0.355	0.170	1.626	0.747	-	No	1	0.486	0.355	0.170	-	0.747
CETN3	CES	1	0.333	0.159	0.036	1.395	0.484	-	No	1	0.333	0.159	0.036	-	0.484
CFHR2	CES	3	-0.013	0.018	0.490	0.987	0.845	0.955	No	3	-0.013	0.018	0.490	0.902	0.845
CFHR4	CES	6	-0.010	0.022	0.669	0.991	0.951	0.955	No	6	-0.010	0.022	0.669	0.890	0.951
CLMP	CES	2	0.014	0.053	0.795	1.014	0.979	-	No	2	0.014	0.053	0.795	-	0.979
COL1A1	CES	1	-0.082	0.376	0.827	0.921	0.989	-	No	1	-0.082	0.376	0.827	-	0.989
COMP	CES	1	-0.176	0.274	0.520	0.838	0.845	-	No	1	-0.176	0.274	0.520	-	0.845
COMT	CES	2	-0.091	0.135	0.500	0.913	0.845	-	No	2	-0.091	0.135	0.500	-	0.845
CPXM1	CES	1	-0.040	0.053	0.457	0.961	0.845	-	No	1	-0.040	0.053	0.457	-	0.845
CTSO	CES	1	-0.034	0.089	0.706	0.967	0.965	-	No	1	-0.034	0.089	0.706	-	0.965
DAG1	CES	1	-0.527	0.275	0.056	0.590	0.621	-	No	1	-0.527	0.275	0.056	-	0.621
DDHD2	CES	1	-0.198	0.168	0.239	0.820	0.805	-	No	1	-0.198	0.168	0.239	-	0.805
DTX3	CES	1	-0.381	0.182	0.036	0.683	0.484	-	No	1	-0.381	0.182	0.036	-	0.484
DUSP13	CES	1	0.011	0.062	0.854	1.012	0.989	-	No	1	0.011	0.062	0.854	-	0.989
DUSP29	CES	1	0.044	0.130	0.732	1.045	0.966	-	No	1	0.044	0.130	0.732	-	0.966
EDN1	CES	1	-0.044	0.113	0.699	0.957	0.965	-	No	1	-0.044	0.113	0.699	-	0.965
EFEMP1	CES	2	-0.056	0.089	0.535	0.946	0.853	-	No	2	-0.056	0.089	0.535	-	0.853
EIF4G3	CES	1	0.192	0.212	0.365	1.211	0.839	-	No	1	0.192	0.212	0.365	-	0.839
ELOA	CES	1	0.410	0.205	0.046	1.507	0.579	-	No	1	0.410	0.205	0.046	-	0.579
ENPEP	CES	1	-0.509	0.458	0.266	0.601	0.805	-	No	1	-0.509	0.458	0.266	-	0.805
ERI1	CES	1	-0.061	0.231	0.791	0.941	0.979	-	No	1	-0.061	0.231	0.791	-	0.979
ERP29	CES	1	1.279	0.490	0.009	3.594	0.208	-	No	1	1.279	0.490	0.009	-	0.208

ST13; MR causal estimates for SBP-associated proteins on cardioembolic stroke.

Causal candidates prioritized for CES were marked as "Yes" in column "Prioritized". All estimates are from inverse variance weighted method (IVs >1) or Wald-ratio method (IV = 1). CES, cardioembolic stroke.

										Steiger filtering					
**Exposure**	**Outcome**	**nsnp**	**Beta**	**SE**	**P-value**	**Odds ratio**	**FDR-corrected P-value**	**FDR-corrected Cochran's Q P-value**	**Prioritized**	**nsnp**	**Beta**	**SE**	**P-value**	**Cochran's Q P-value**	**FDR-corrected P-value**
ESAM	CES	1	-0.009	0.133	0.949	0.991	0.994	-	No	1	-0.009	0.133	0.949	-	0.994
F13B	CES	1	0.014	0.063	0.826	1.014	0.989	-	No	1	0.014	0.063	0.826	-	0.989
FDX1	CES	1	0.425	0.345	0.218	1.529	0.787	-	No	1	0.425	0.345	0.218	-	0.787
FES	CES	1	-0.335	0.126	0.008	0.715	0.208	-	No	1	-0.335	0.126	0.008	-	0.208
FGF2	CES	3	0.033	0.038	0.388	1.033	0.839	0.906	No	3	0.033	0.038	0.388	0.518	0.839
FGF5	CES	4	0.061	0.026	0.019	1.062	0.311	0.856	No	4	0.061	0.026	0.019	0.391	0.311
FKBP7	CES	1	0.330	0.234	0.159	1.391	0.745	-	No	1	0.330	0.234	0.159	-	0.745
FN1	CES	2	-0.087	0.109	0.422	0.916	0.845	-	No	2	-0.087	0.109	0.422	-	0.845
FOXJ3	CES	1	0.117	0.243	0.631	1.124	0.944	-	No	1	0.117	0.243	0.631	-	0.944
FOXO3	CES	1	0.307	0.460	0.505	1.359	0.845	-	No	1	0.307	0.460	0.505	-	0.845
FUCA1	CES	1	0.102	0.047	0.028	1.108	0.433	-	No	1	0.102	0.047	0.028	-	0.433
FURIN	CES	1	0.278	0.093	0.003	1.321	0.101	-	No	1	0.278	0.093	0.003	-	0.101
GCHFR	CES	1	0.260	0.150	0.084	1.297	0.621	-	No	1	0.260	0.150	0.084	-	0.621
GFER	CES	1	0.024	0.290	0.935	1.024	0.994	-	No	1	0.024	0.290	0.935	-	0.994
GHR	CES	3	0.032	0.026	0.223	1.032	0.787	0.944	No	3	0.032	0.026	0.223	0.593	0.787
GIMAP7	CES	3	0.021	0.031	0.503	1.021	0.845	0.955	No	3	0.021	0.031	0.503	0.906	0.845
GIT1	CES	1	0.164	0.375	0.662	1.178	0.951	-	No	1	0.164	0.375	0.662	-	0.951
GORASP2	CES	1	0.335	0.337	0.320	1.398	0.833	-	No	1	0.335	0.337	0.320	-	0.833
GRP	CES	4	0.000	0.048	0.994	1.000	0.994	0.955	No	4	0.000	0.048	0.994	0.949	0.994
HADH	CES	1	-0.245	0.308	0.426	0.782	0.845	-	No	1	-0.245	0.308	0.426	-	0.845
HHEX	CES	1	0.048	0.439	0.914	1.049	0.989	-	No	1	0.048	0.439	0.914	-	0.989
HMOX2	CES	1	-0.366	0.281	0.193	0.693	0.787	-	No	1	-0.366	0.281	0.193	-	0.787
HYAL1	CES	1	-0.013	0.068	0.846	0.987	0.989	-	No	1	-0.013	0.068	0.846	-	0.989
ICAM2	CES	1	0.329	0.328	0.315	1.389	0.833	-	No	1	0.329	0.328	0.315	-	0.833
IDUA	CES	2	0.028	0.032	0.390	1.028	0.839	-	No	2	0.028	0.032	0.390	-	0.839
IFI30	CES	2	-0.052	0.037	0.163	0.949	0.746	-	No	2	-0.052	0.037	0.163	-	0.746
IFNGR2	CES	5	-0.015	0.017	0.385	0.986	0.839	0.955	No	5	-0.015	0.017	0.385	0.717	0.839
IGFBP3	CES	3	-0.063	0.076	0.403	0.939	0.839	0.702	No	3	-0.063	0.076	0.403	0.175	0.839
IMMT	CES	1	0.203	0.141	0.150	1.225	0.715	-	No	1	0.203	0.141	0.150	-	0.715
IMPA1	CES	1	0.097	0.086	0.257	1.102	0.805	-	No	1	0.097	0.086	0.257	-	0.805
ITGAL	CES	1	-0.525	0.544	0.335	0.592	0.833	-	No	1	-0.525	0.544	0.335	-	0.833
ITIH1	CES	1	0.151	0.121	0.211	1.163	0.787	-	No	1	0.151	0.121	0.211	-	0.787
KIFBP	CES	1	0.100	0.374	0.789	1.105	0.979	-	No	1	0.100	0.374	0.789	-	0.979
LMOD1	CES	1	-0.036	0.100	0.717	0.964	0.966	-	No	1	-0.036	0.100	0.717	-	0.966
LRIG1	CES	3	-0.033	0.021	0.107	0.967	0.674	0.906	No	3	-0.033	0.021	0.107	0.543	0.674
LYAR	CES	1	0.253	0.368	0.491	1.288	0.845	-	No	1	0.253	0.368	0.491	-	0.845
M6PR	CES	1	-0.018	0.090	0.841	0.982	0.989	-	No	1	-0.018	0.090	0.841	-	0.989
MANEAL	CES	1	-0.455	0.464	0.328	0.635	0.833	-	No	1	-0.455	0.464	0.328	-	0.833
MANSC4	CES	2	-0.029	0.030	0.324	0.971	0.833	-	No	2	-0.029	0.030	0.324	-	0.833
MAP4K5	CES	1	-0.099	0.089	0.267	0.906	0.805	-	No	1	-0.099	0.089	0.267	-	0.805
MDH1	CES	2	0.445	0.179	0.013	1.560	0.264	-	No	2	0.445	0.179	0.013	-	0.264
MEGF9	CES	1	-0.103	0.055	0.063	0.902	0.621	-	No	1	-0.103	0.055	0.063	-	0.621
MPHOSPH8	CES	1	0.141	0.451	0.754	1.152	0.966	-	No	1	0.141	0.451	0.754	-	0.966
MPI	CES	2	-0.012	0.076	0.875	0.988	0.989	-	No	2	-0.012	0.076	0.875	-	0.989
MSRA	CES	1	0.165	0.293	0.575	1.179	0.889	-	No	1	0.165	0.293	0.575	-	0.889
MST1	CES	2	0.030	0.017	0.077	1.030	0.621	-	No	2	0.030	0.017	0.077	-	0.621
MVK	CES	1	0.012	0.194	0.950	1.012	0.994	-	No	1	0.012	0.194	0.950	-	0.994
NADK	CES	1	-0.114	0.071	0.109	0.892	0.674	-	No	1	-0.114	0.071	0.109	-	0.674
NAGA	CES	2	-0.126	0.171	0.462	0.882	0.845	-	No	2	-0.126	0.171	0.462	-	0.845
NFE2	CES	1	-0.455	0.259	0.078	0.634	0.621	-	No	1	-0.455	0.259	0.078	-	0.621
NFU1	CES	1	-0.247	0.158	0.118	0.781	0.677	-	No	1	-0.247	0.158	0.118	-	0.677
NGF	CES	1	0.220	0.723	0.762	1.246	0.966	-	No	1	0.220	0.723	0.762	-	0.966
NOTCH3	CES	1	0.063	0.371	0.865	1.065	0.989	-	No	1	0.063	0.371	0.865	-	0.989
NPPB	CES	1	-0.088	0.141	0.530	0.915	0.853	-	No	1	-0.088	0.141	0.530	-	0.853
NTRK3	CES	2	-0.123	0.079	0.118	0.884	0.677	-	No	2	-0.123	0.079	0.118	-	0.677
NUCB2	CES	2	-0.031	0.089	0.728	0.970	0.966	-	No	2	-0.031	0.089	0.728	-	0.966

ST13; MR causal estimates for SBP-associated proteins on cardioembolic stroke.

Causal candidates prioritized for CES were marked as "Yes" in column "Prioritized". All estimates are from inverse variance weighted method (IVs >1) or Wald-ratio method (IV = 1). CES, cardioembolic stroke.

										Steiger filtering					
**Exposure**	**Outocme**	**nsnp**	**Beta**	**SE**	**P-value**	**Odds ratio**	**FDR-corrected P-value**	**FDR-corrected Cochran's Q P-value**	**Prioritized**	**nsnp**	**Beta**	**SE**	**P-value**	**Cochran's Q P-value**	**FDR-corrected P-value**
NUDT5	CES	1	-0.467	0.295	0.114	0.627	0.677	-	No	1	-0.467	0.295	0.114	-	0.677
NUMB	CES	1	0.285	0.189	0.131	1.329	0.699	-	No	1	0.285	0.189	0.131	-	0.699
OGA	CES	1	-0.002	0.145	0.987	0.998	0.994	-	No	1	-0.002	0.145	0.987	-	0.994
OPLAH	CES	1	-0.061	0.093	0.513	0.941	0.845	-	No	1	-0.061	0.093	0.513	-	0.845
PCBP2	CES	1	-0.622	0.354	0.078	0.537	0.621	-	No	1	-0.622	0.354	0.078	-	0.621
PCSK7	CES	2	-0.034	0.080	0.676	0.967	0.951	-	No	2	-0.034	0.080	0.676	-	0.951
PDE5A	CES	2	0.010	0.099	0.920	1.010	0.989	-	No	2	0.010	0.099	0.920	-	0.989
PDGFRA	CES	2	-0.056	0.102	0.582	0.945	0.889	-	No	2	-0.056	0.102	0.582	-	0.889
PECAM1	CES	1	0.159	0.529	0.763	1.173	0.966	-	No	1	0.159	0.529	0.763	-	0.966
PFKFB2	CES	1	-0.117	0.091	0.199	0.890	0.787	-	No	1	-0.117	0.091	0.199	-	0.787
PGF	CES	1	0.130	0.106	0.220	1.139	0.787	-	No	1	0.130	0.106	0.220	-	0.787
PHLDB1	CES	1	-0.005	0.363	0.990	0.996	0.994	-	No	1	-0.005	0.363	0.990	-	0.994
PKD1	CES	1	0.387	0.356	0.278	1.472	0.805	-	No	1	0.387	0.356	0.278	-	0.805
PLA2G1B	CES	1	0.124	0.330	0.708	1.132	0.965	-	No	1	0.124	0.330	0.708	-	0.965
PMS1	CES	1	0.287	0.360	0.425	1.333	0.845	-	No	1	0.287	0.360	0.425	-	0.845
PPP1R14D	CES	1	0.397	0.302	0.189	1.487	0.787	-	No	1	0.397	0.302	0.189	-	0.787
PRG2	CES	1	-0.185	0.169	0.274	0.831	0.805	-	No	1	-0.185	0.169	0.274	-	0.805
PRKAB1	CES	1	0.164	0.097	0.090	1.179	0.627	-	No	1	0.164	0.097	0.090	-	0.627
PSMD5	CES	1	0.234	0.345	0.497	1.264	0.845	-	No	1	0.234	0.345	0.497	-	0.845
PSRC1	CES	1	-0.105	0.150	0.482	0.900	0.845	-	No	1	-0.105	0.150	0.482	-	0.845
PTRHD1	CES	1	0.340	0.447	0.446	1.405	0.845	-	No	1	0.340	0.447	0.446	-	0.845
QPCT	CES	3	0.032	0.072	0.656	1.033	0.951	0.637	No	3	0.032	0.072	0.656	0.091	0.951
RABEPK	CES	1	0.026	0.113	0.817	1.026	0.989	-	No	1	0.026	0.113	0.817	-	0.989
RARRES1	CES	5	-0.043	0.043	0.313	0.958	0.833	0.702	No	5	-0.043	0.043	0.313	0.181	0.833
RARRES2	CES	1	0.003	0.087	0.970	1.003	0.994	-	No	1	0.003	0.087	0.970	-	0.994
RELT	CES	2	0.008	0.055	0.890	1.008	0.989	-	No	2	0.008	0.055	0.890	-	0.989
RSPO3	CES	1	0.151	0.105	0.150	1.163	0.715	-	No	1	0.151	0.105	0.150	-	0.715
SCARA5	CES	4	-0.160	0.051	0.002	0.852	0.092	0.823	No	4	-0.160	0.051	0.002	0.353	0.092
SDCCAG8	CES	1	-0.058	0.081	0.478	0.944	0.845	-	No	1	-0.058	0.081	0.478	-	0.845
SDHB	CES	1	0.163	0.279	0.560	1.177	0.872	-	No	1	0.163	0.279	0.560	-	0.872
SELENOP	CES	1	0.124	0.164	0.451	1.132	0.845	-	No	1	0.124	0.164	0.451	-	0.845
SEMA6C	CES	1	-0.037	0.186	0.841	0.963	0.989	-	No	1	-0.037	0.186	0.841	-	0.989
SERPINI1	CES	2	-0.033	0.043	0.454	0.968	0.845	-	No	2	-0.033	0.043	0.454	-	0.845
SH2B3	CES	1	0.405	0.229	0.077	1.500	0.621	-	No	1	0.405	0.229	0.077	-	0.621
SHMT1	CES	4	0.018	0.024	0.445	1.018	0.845	0.955	No	4	0.018	0.024	0.445	0.823	0.845
SIL1	CES	1	0.169	0.279	0.544	1.184	0.859	-	No	1	0.169	0.279	0.544	-	0.859
SLC16A1	CES	1	-0.450	0.275	0.103	0.638	0.674	-	No	1	-0.450	0.275	0.103	-	0.674
SLC39A14	CES	1	-0.153	0.180	0.396	0.859	0.839	-	No	1	-0.153	0.180	0.396	-	0.839
SLC9A3R2	CES	1	0.302	0.171	0.077	1.353	0.621	-	No	1	0.302	0.171	0.077	-	0.621
SMOC2	CES	3	-0.005	0.093	0.960	0.995	0.994	0.252	No	3	-0.005	0.093	0.960	0.014	0.994
SOST	CES	1	-0.073	0.317	0.817	0.929	0.989	-	No	1	-0.073	0.317	0.817	-	0.989
SPINK8	CES	1	0.036	0.039	0.356	1.037	0.833	-	No	1	0.036	0.039	0.356	-	0.833
SPRED2	CES	1	-0.321	0.256	0.210	0.726	0.787	-	No	1	-0.321	0.256	0.210	-	0.787
TARBP2	CES	1	-0.669	0.380	0.078	0.512	0.621	-	No	1	-0.669	0.380	0.078	-	0.621
TBC1D23	CES	1	-0.073	0.104	0.478	0.929	0.845	-	No	1	-0.073	0.104	0.478	-	0.845
TEK	CES	3	0.039	0.040	0.329	1.040	0.833	0.955	No	3	0.039	0.040	0.329	0.829	0.833
TGFB2	CES	1	0.284	0.120	0.018	1.328	0.311	-	No	1	0.284	0.120	0.018	-	0.311
TIE1	CES	1	0.099	0.087	0.259	1.104	0.805	-	No	1	0.099	0.087	0.259	-	0.805
TJAP1	CES	1	-1.157	0.399	0.004	0.315	0.124	-	No	1	-1.157	0.399	0.004	-	0.124
TNFRSF17	CES	1	-0.013	0.126	0.918	0.987	0.989	-	No	1	-0.013	0.126	0.918	-	0.989
TNFSF12	CES	2	-0.116	0.047	0.014	0.890	0.264	-	No	2	-0.116	0.047	0.014	-	0.264
TNFSF13B	CES	2	-0.181	0.184	0.324	0.834	0.833	-	No	2	-0.181	0.184	0.324	-	0.833
TWF2	CES	1	-0.247	0.258	0.338	0.781	0.833	-	No	1	-0.247	0.258	0.338	-	0.833
UBE2L6	CES	2	0.043	0.103	0.677	1.044	0.951	-	No	2	0.043	0.103	0.677	-	0.951
UMOD	CES	6	-0.004	0.021	0.831	0.996	0.989	0.955	No	6	-0.004	0.021	0.831	0.955	0.989
VSIG2	CES	1	0.006	0.090	0.949	1.006	0.994	-	No	1	0.006	0.090	0.949	-	0.994

ST13; MR causal estimates for SBP-associated proteins on cardioembolic stroke.

Causal candidates prioritized for CES were marked as "Yes" in column "Prioritized". All estimates are from inverse variance weighted method (IVs >1) or Wald-ratio method (IV = 1). CES, cardioembolic stroke.

										Steiger filtering					
Exposure	Outocme	nsnp	Beta	SE	P-value	Odds ratio	FDR-corrected P-value	FDR-corrected Cochran's Q P-value	Prioritized	nsnp	Beta	SE	P-value	Cochran's Q P-value	FDR-corrected P-value
WARS	CES	1	0.090	0.084	0.284	1.094	0.809	-	No	1	0.090	0.084	0.284	-	0.809
WASHC3	CES	1	-0.199	0.216	0.356	0.819	0.833	-	No	1	-0.199	0.216	0.356	-	0.833
WWP2	CES	1	0.049	0.109	0.653	1.050	0.951	-	No	1	0.049	0.109	0.653	-	0.951
YOD1	CES	1	-0.221	0.152	0.147	0.802	0.715	-	No	1	-0.221	0.152	0.147	-	0.715
ZBTB17	CES	1	-0.712	0.584	0.223	0.491	0.787	-	No	1	-0.712	0.584	0.223	-	0.787
ZFYVE19	CES	1	-0.005	0.045	0.904	0.995	0.989	-	No	1	-0.005	0.045	0.904	-	0.989

nsnp = number of single nucleotide polymorphisms; SE = standard error; Cochran's Q P-value = p-value from Cochran's Q test assessing heterogeneity; FDR=false discovery rate

ST14; MR causal estimates for DBP-associated proteins on cardioembolic stroke.

Causal candidates prioritized for CES were marked as "Yes" in column "Prioritized". All estimates are from inverse variance weighted method (IVs >1) or Wald-ratio method (IV = 1). CES, cardioembolic stroke.

										Steiger filtering					
Exposure	Outocme	nsnp	Beta	SE	P-value	Odds ratio	FDR-corrected P-value	FDR-corrected Cochran's Q P-value	Prioritized	nsnp	Beta	SE	P-value	Cochran's Q P-value	FDR-corrected P-value
AAMDC	CES	2	0.019	0.026	0.461	1.019	0.845	-	No	2	0.019	0.026	0.461	-	0.845
ABO	CES	7	0.080	0.015	0.000	1.083	0.000	0.955	Yes	7	0.080	0.015	0.000	0.938	0.000
ACOX1	CES	1	0.200	0.226	0.376	1.221	0.839	-	No	1	0.200	0.226	0.376	-	0.839
ADM	CES	1	-0.207	0.450	0.646	0.813	0.951	-	No	1	-0.207	0.450	0.646	-	0.951
AMFR	CES	1	0.049	0.397	0.902	1.050	0.989	-	No	1	0.049	0.397	0.902	-	0.989
AMOTL2	CES	1	-0.107	0.326	0.743	0.899	0.966	-	No	1	-0.107	0.326	0.743	-	0.966
ANKMY2	CES	1	-0.205	0.243	0.400	0.815	0.839	-	No	1	-0.205	0.243	0.400	-	0.839
AOC1	CES	3	0.004	0.064	0.952	1.004	0.994	0.763	No	3	0.004	0.064	0.952	0.283	0.994
AOC3	CES	2	0.002	0.045	0.967	1.002	0.994	-	No	2	0.002	0.045	0.967	-	0.994
APOA1	CES	1	-0.042	0.253	0.867	0.958	0.989	-	No	1	-0.042	0.253	0.867	-	0.989
APOBR	CES	3	-0.033	0.033	0.328	0.968	0.833	0.252	No	3	-0.033	0.033	0.328	0.009	0.833
APOC1	CES	2	-0.190	0.225	0.399	0.827	0.839	-	No	2	-0.190	0.225	0.399	-	0.839
ARSB	CES	3	-0.002	0.070	0.973	0.998	0.994	0.823	No	3	-0.002	0.070	0.973	0.342	0.994
ATXN2L	CES	1	0.563	0.474	0.236	1.755	0.805	-	No	1	0.563	0.474	0.236	-	0.805
AXL	CES	2	-0.112	0.101	0.267	0.894	0.805	-	No	2	-0.112	0.101	0.267	-	0.805
BAG4	CES	1	-0.634	0.438	0.148	0.531	0.715	-	No	1	-0.634	0.438	0.148	-	0.715
BNIP3L	CES	1	-0.363	0.318	0.254	0.696	0.805	-	No	1	-0.363	0.318	0.254	-	0.805
CA12	CES	2	-0.031	0.056	0.579	0.969	0.889	-	No	2	-0.031	0.056	0.579	-	0.889
CACNB3	CES	1	-0.006	0.166	0.972	0.994	0.994	-	No	1	-0.006	0.166	0.972	-	0.994
CALCA	CES	1	0.069	0.160	0.665	1.072	0.951	-	No	1	0.069	0.160	0.665	-	0.951
CEP170	CES	1	0.120	0.262	0.648	1.127	0.951	-	No	1	0.120	0.262	0.648	-	0.951
CFHR4	CES	6	-0.010	0.022	0.669	0.991	0.951	0.955	No	6	-0.010	0.022	0.669	0.890	0.951
CHMP1A	CES	1	0.248	0.189	0.191	1.281	0.787	-	No	1	0.248	0.189	0.191	-	0.787
CLIC5	CES	1	0.116	0.136	0.391	1.123	0.839	-	No	1	0.116	0.136	0.391	-	0.839
COL1A1	CES	1	-0.082	0.376	0.827	0.921	0.989	-	No	1	-0.082	0.376	0.827	-	0.989
CTF1	CES	1	-0.048	0.329	0.885	0.954	0.989	-	No	1	-0.048	0.329	0.885	-	0.989
DAG1	CES	1	-0.527	0.275	0.056	0.590	0.621	-	No	1	-0.527	0.275	0.056	-	0.621
DARS1	CES	1	0.015	0.238	0.951	1.015	0.994	-	No	1	0.015	0.238	0.951	-	0.994
DBN1	CES	1	0.181	0.332	0.586	1.198	0.889	-	No	1	0.181	0.332	0.586	-	0.889
DDHD2	CES	1	-0.198	0.168	0.239	0.820	0.805	-	No	1	-0.198	0.168	0.239	-	0.805
DENR	CES	1	-0.725	0.497	0.144	0.484	0.715	-	No	1	-0.725	0.497	0.144	-	0.715
DNER	CES	5	-0.023	0.060	0.704	0.978	0.965	0.702	No	5	-0.023	0.060	0.704	0.221	0.965
DOK2	CES	1	0.057	0.296	0.847	1.059	0.989	-	No	1	0.057	0.296	0.847	-	0.989
DPEP1	CES	4	-0.041	0.138	0.765	0.960	0.966	0.702	No	4	-0.041	0.138	0.765	0.175	0.966
DPP4	CES	1	0.133	0.078	0.085	1.143	0.621	-	No	1	0.133	0.078	0.085	-	0.621
DUSP13	CES	1	0.011	0.062	0.854	1.012	0.989	-	No	1	0.011	0.062	0.854	-	0.989
EFNA1	CES	1	0.059	0.052	0.250	1.061	0.805	-	No	1	0.059	0.052	0.250	-	0.805
EIF4G3	CES	1	0.192	0.212	0.365	1.211	0.839	-	No	1	0.192	0.212	0.365	-	0.839
ELOA	CES	1	0.410	0.205	0.046	1.507	0.579	-	No	1	0.410	0.205	0.046	-	0.579
ENPEP	CES	1	-0.509	0.458	0.266	0.601	0.805	-	No	1	-0.509	0.458	0.266	-	0.805
EPHA2	CES	2	-0.062	0.185	0.739	0.940	0.966	-	No	2	-0.062	0.185	0.739	-	0.966
EPO	CES	1	-0.240	0.225	0.287	0.787	0.809	-	No	1	-0.240	0.225	0.287	-	0.809
ERI1	CES	1	-0.061	0.231	0.791	0.941	0.979	-	No	1	-0.061	0.231	0.791	-	0.979
ESAM	CES	1	-0.009	0.133	0.949	0.991	0.994	-	No	1	-0.009	0.133	0.949	-	0.994
F12	CES	5	-0.026	0.027	0.335	0.975	0.833	0.904	No	5	-0.026	0.027	0.335	0.451	0.833
FADD	CES	1	-0.136	0.211	0.519	0.873	0.845	-	No	1	-0.136	0.211	0.519	-	0.845
FDX1	CES	1	0.425	0.345	0.218	1.529	0.787	-	No	1	0.425	0.345	0.218	-	0.787
FES	CES	1	-0.335	0.126	0.008	0.715	0.208	-	No	1	-0.335	0.126	0.008	-	0.208
FGF5	CES	4	0.061	0.026	0.019	1.062	0.311	0.856	No	4	0.061	0.026	0.019	0.391	0.311
FOXJ3	CES	1	0.117	0.243	0.631	1.124	0.944	-	No	1	0.117	0.243	0.631	-	0.944
FURIN	CES	1	0.278	0.093	0.003	1.321	0.101	-	No	1	0.278	0.093	0.003	-	0.101
GFER	CES	1	0.024	0.290	0.935	1.024	0.994	-	No	1	0.024	0.290	0.935	-	0.994
GIMAP7	CES	3	0.021	0.031	0.503	1.021	0.845	0.955	No	3	0.021	0.031	0.503	0.906	0.845
GLO1	CES	2	-0.051	0.074	0.488	0.950	0.845	-	No	2	-0.051	0.074	0.488	-	0.845
GORASP2	CES	1	0.335	0.337	0.320	1.398	0.833	-	No	1	0.335	0.337	0.320	-	0.833
GRP	CES	4	0.000	0.048	0.994	1.000	0.994	0.955	No	4	0.000	0.048	0.994	0.949	0.994

ST14; MR causal estimates for DBP-associated proteins on cardioembolic stroke.

Causal candidates prioritized for CES were marked as "Yes" in column "Prioritized". All estimates are from inverse variance weighted method (IVs >1) or Wald-ratio method (IV = 1). CES, cardioembolic stroke.

										Steiger filtering					
**Exposure**	**Outcme**	**nsnp**	**Beta**	**SE**	**P-value**	**Odds ratio**	**FDR-corrected P-value**	**FDR-corrected Cochran's Q P-value**	**Prioritized**	**nsnp**	**Beta**	**SE**	**P-value**	**Cochran's Q P-value**	**FDR-corrected P-value**
HADH	CES	1	-0.245	0.308	0.426	0.782	0.845	-	No	1	-0.245	0.308	0.426	-	0.845
HHEX	CES	1	0.048	0.439	0.914	1.049	0.989	-	No	1	0.048	0.439	0.914	-	0.989
HPGDS	CES	6	0.013	0.045	0.775	1.013	0.970	0.674	No	6	0.013	0.045	0.775	0.116	0.970
ICAM1	CES	4	0.016	0.045	0.722	1.016	0.966	0.763	No	4	0.016	0.045	0.722	0.275	0.966
ICAM4	CES	1	-0.108	0.153	0.482	0.898	0.845	-	No	1	-0.108	0.153	0.482	-	0.845
IFIT3	CES	1	0.173	0.156	0.267	1.189	0.805	-	No	1	0.173	0.156	0.267	-	0.805
IGFBP3	CES	3	-0.063	0.076	0.403	0.939	0.839	0.702	No	3	-0.063	0.076	0.403	0.175	0.839
IL1RL1	CES	6	-0.037	0.023	0.108	0.964	0.674	0.955	No	6	-0.037	0.023	0.108	0.700	0.674
IMMT	CES	1	0.203	0.141	0.150	1.225	0.715	-	No	1	0.203	0.141	0.150	-	0.715
ITGAL	CES	1	-0.525	0.544	0.335	0.592	0.833	-	No	1	-0.525	0.544	0.335	-	0.833
KIF22	CES	1	0.304	0.472	0.520	1.355	0.845	-	No	1	0.304	0.472	0.520	-	0.845
KIFBP	CES	1	0.100	0.374	0.789	1.105	0.979	-	No	1	0.100	0.374	0.789	-	0.979
LAYN	CES	1	-0.053	0.046	0.256	0.949	0.805	-	No	1	-0.053	0.046	0.256	-	0.805
LMOD1	CES	1	-0.036	0.100	0.717	0.964	0.966	-	No	1	-0.036	0.100	0.717	-	0.966
M6PR	CES	1	-0.018	0.090	0.841	0.982	0.989	-	No	1	-0.018	0.090	0.841	-	0.989
MAP4K5	CES	1	-0.099	0.089	0.267	0.906	0.805	-	No	1	-0.099	0.089	0.267	-	0.805
MFGE8	CES	2	0.014	0.048	0.768	1.014	0.966	-	No	2	0.014	0.048	0.768	-	0.966
MPHOSPH8	CES	1	0.141	0.451	0.754	1.152	0.966	-	No	1	0.141	0.451	0.754	-	0.966
MSRA	CES	1	0.165	0.293	0.575	1.179	0.889	-	No	1	0.165	0.293	0.575	-	0.889
MST1	CES	2	0.030	0.017	0.077	1.030	0.621	-	No	2	0.030	0.017	0.077	-	0.621
NADK	CES	1	-0.114	0.071	0.109	0.892	0.674	-	No	1	-0.114	0.071	0.109	-	0.674
NGF	CES	1	0.220	0.723	0.762	1.246	0.966	-	No	1	0.220	0.723	0.762	-	0.966
NOMO1	CES	1	-0.131	0.167	0.434	0.877	0.845	-	No	1	-0.131	0.167	0.434	-	0.845
NOS3	CES	1	-1.464	0.371	0.000	0.231	0.009	-	Yes	1	-1.464	0.371	0.000	-	0.009
NPPB	CES	1	-0.088	0.141	0.530	0.915	0.853	-	No	1	-0.088	0.141	0.530	-	0.853
NUCB2	CES	2	-0.031	0.089	0.728	0.970	0.966	-	No	2	-0.031	0.089	0.728	-	0.966
PAM	CES	4	-0.006	0.044	0.883	0.994	0.989	0.702	No	4	-0.006	0.044	0.883	0.217	0.989
PARP1	CES	1	-0.015	0.136	0.910	0.985	0.989	-	No	1	-0.015	0.136	0.910	-	0.989
PCOLCE	CES	1	-0.073	0.102	0.474	0.929	0.845	-	No	1	-0.073	0.102	0.474	-	0.845
PCSK7	CES	2	-0.034	0.080	0.676	0.967	0.951	-	No	2	-0.034	0.080	0.676	-	0.951
PDE5A	CES	2	0.010	0.099	0.920	1.010	0.989	-	No	2	0.010	0.099	0.920	-	0.989
PDIA3	CES	1	0.480	0.393	0.222	1.616	0.787	-	No	1	0.480	0.393	0.222	-	0.787
PECAM1	CES	1	0.159	0.529	0.763	1.173	0.966	-	No	1	0.159	0.529	0.763	-	0.966
PFKFB2	CES	1	-0.117	0.091	0.199	0.890	0.787	-	No	1	-0.117	0.091	0.199	-	0.787
PLA2G1B	CES	1	0.124	0.330	0.708	1.132	0.965	-	No	1	0.124	0.330	0.708	-	0.965
PLXDC2	CES	3	-0.084	0.163	0.609	0.920	0.917	0.955	No	3	-0.084	0.163	0.609	0.655	0.917
PMS1	CES	1	0.287	0.360	0.425	1.333	0.845	-	No	1	0.287	0.360	0.425	-	0.845
PRDX1	CES	1	-0.158	0.256	0.536	0.854	0.853	-	No	1	-0.158	0.256	0.536	-	0.853
PROCR	CES	1	-0.139	0.037	0.000	0.870	0.014	-	Yes	1	-0.139	0.037	0.000	-	0.014
PRSS53	CES	3	0.010	0.029	0.736	1.010	0.966	0.904	No	3	0.010	0.029	0.736	0.465	0.966
PRTFDC1	CES	1	0.136	0.338	0.687	1.146	0.960	-	No	1	0.136	0.338	0.687	-	0.960
PSRC1	CES	1	-0.105	0.150	0.482	0.900	0.845	-	No	1	-0.105	0.150	0.482	-	0.845
PTPRF	CES	1	-0.392	0.212	0.065	0.676	0.621	-	No	1	-0.392	0.212	0.065	-	0.621
PTRHD1	CES	1	0.340	0.447	0.446	1.405	0.845	-	No	1	0.340	0.447	0.446	-	0.845
PYDC1	CES	1	-0.078	0.056	0.167	0.925	0.747	-	No	1	-0.078	0.056	0.167	-	0.747
RELT	CES	2	0.008	0.055	0.890	1.008	0.989	-	No	2	0.008	0.055	0.890	-	0.989
SDC1	CES	2	0.018	0.115	0.875	1.018	0.989	-	No	2	0.018	0.115	0.875	-	0.989
SDCCAG8	CES	1	-0.058	0.081	0.478	0.944	0.845	-	No	1	-0.058	0.081	0.478	-	0.845
SDHB	CES	1	0.163	0.279	0.560	1.177	0.872	-	No	1	0.163	0.279	0.560	-	0.872
SERPINI1	CES	2	-0.033	0.043	0.454	0.968	0.845	-	No	2	-0.033	0.043	0.454	-	0.845
SH2B3	CES	1	0.405	0.229	0.077	1.500	0.621	-	No	1	0.405	0.229	0.077	-	0.621
SHMT1	CES	4	0.018	0.024	0.445	1.018	0.845	0.955	No	4	0.018	0.024	0.445	0.823	0.845
SLC16A1	CES	1	-0.450	0.275	0.103	0.638	0.674	-	No	1	-0.450	0.275	0.103	-	0.674
SLC9A3R2	CES	1	0.302	0.171	0.077	1.353	0.621	-	No	1	0.302	0.171	0.077	-	0.621
SMTN	CES	1	0.149	0.430	0.728	1.161	0.966	-	No	1	0.149	0.430	0.728	-	0.966
SPINK8	CES	1	0.036	0.039	0.356	1.037	0.833	-	No	1	0.036	0.039	0.356	-	0.833

ST14; MR causal estimates for DBP-associated proteins on cardioembolic stroke.

Causal candidates prioritized for CES were marked as "Yes" in column "Prioritized". All estimates are from inverse variance weighted method (IVs >1) or Wald-ratio method (IV = 1). CES, cardioembolic stroke.

										Steiger filtering					
**Exposure**	**Outcme**	**nsnp**	**Beta**	**SE**	**P-value**	**Odds ratio**	**FDR-corrected P-value**	**FDR-corrected Cochran's Q P-value**	**Prioritized**	**nsnp**	**Beta**	**SE**	**P-value**	**Cochran's Q P-value**	**FDR-corrected P-value**
STC1	CES	1	0.205	0.318	0.520	1.227	0.845	-	No	1	0.205	0.318	0.520	-	0.845
STX4	CES	1	-0.005	0.396	0.990	0.995	0.994	-	No	1	-0.005	0.396	0.990	-	0.994
TBC1D17	CES	2	-0.041	0.043	0.346	0.960	0.833	-	No	2	-0.041	0.043	0.346	-	0.833
TIE1	CES	1	0.099	0.087	0.259	1.104	0.805	-	No	1	0.099	0.087	0.259	-	0.805
TJAP1	CES	1	-1.157	0.399	0.004	0.315	0.124	-	No	1	-1.157	0.399	0.004	-	0.124
TMEM106A	CES	1	-0.222	0.163	0.173	0.801	0.747	-	No	1	-0.222	0.163	0.173	-	0.747
TNFRSF17	CES	1	-0.013	0.126	0.918	0.987	0.989	-	No	1	-0.013	0.126	0.918	-	0.989
TNFSF12	CES	2	-0.116	0.047	0.014	0.890	0.264	-	No	2	-0.116	0.047	0.014	-	0.264
TP53	CES	1	-0.035	0.282	0.902	0.966	0.989	-	No	1	-0.035	0.282	0.902	-	0.989
UMOD	CES	6	-0.004	0.021	0.831	0.996	0.989	0.955	No	6	-0.004	0.021	0.831	0.955	0.989
UXS1	CES	2	-0.097	0.150	0.519	0.908	0.845	-	No	2	-0.097	0.150	0.519	-	0.845
VAT1	CES	1	-0.197	0.115	0.087	0.821	0.621	-	No	1	-0.197	0.115	0.087	-	0.621
VSIG2	CES	1	0.006	0.090	0.949	1.006	0.994	-	No	1	0.006	0.090	0.949	-	0.994
WNT9A	CES	2	0.148	0.140	0.290	1.159	0.809	-	No	2	0.148	0.140	0.290	-	0.809
WWP2	CES	1	0.049	0.109	0.653	1.050	0.951	-	No	1	0.049	0.109	0.653	-	0.951
YAP1	CES	1	-0.035	0.255	0.891	0.966	0.989	-	No	1	-0.035	0.255	0.891	-	0.989
YOD1	CES	1	-0.221	0.152	0.147	0.802	0.715	-	No	1	-0.221	0.152	0.147	-	0.715
ZBTB17	CES	1	-0.712	0.584	0.223	0.491	0.787	-	No	1	-0.712	0.584	0.223	-	0.787

nsnp = number of single nucleotide polymorphisms; SE = standard error; Cochran's Q P-value = p-value from Cochran's Q test assessing heterogeneity; FDR=false discovery rate

ST15; MR causal estimates for SBP-associated proteins on large artery stroke.

Causal candidates prioritized for LAS were marked as "Yes" in column "Prioritized". All estimates are from inverse variance weighted method (IVs >1) or Wald-ratio method (IV = 1). LAS, large artery stroke.

										Steiger filtering					
**Exposure**	**Outcome**	**nsnp**	**Beta**	**SE**	**P-value**	**Odds ratio**	**FDR-corrected P-value**	**FDR-corrected Cochran's Q P-value**	**Prioritized**	**nsnp**	**Beta**	**SE**	**P-value**	**Cochran's Q P-value**	**FDR-corrected P-value**
ACOX1	LAS	1	0.396	0.297	0.183	1.486	0.756	-	No	1	0.396	0.297	0.183	-	0.756
ACRBP	LAS	1	0.094	0.160	0.557	1.099	0.848	-	No	1	0.094	0.160	0.557	-	0.848
ADAM23	LAS	4	0.015	0.034	0.660	1.015	0.914	0.999	No	4	0.015	0.034	0.660	0.800	0.914
ADAMTS1	LAS	1	0.127	0.552	0.817	1.136	0.953	-	No	1	0.127	0.552	0.817	-	0.953
ADAMTS4	LAS	1	-0.044	0.179	0.807	0.957	0.953	-	No	1	-0.044	0.179	0.807	-	0.953
ADAMTS8	LAS	5	-0.067	0.046	0.149	0.935	0.729	0.999	No	5	-0.067	0.046	0.149	0.377	0.729
ADAMTSL5	LAS	1	0.046	0.073	0.526	1.047	0.837	-	No	1	0.046	0.073	0.526	-	0.837
ADM	LAS	1	-0.889	0.598	0.137	0.411	0.703	-	No	1	-0.889	0.598	0.137	-	0.703
AMOTL2	LAS	1	-0.287	0.426	0.500	0.750	0.837	-	No	1	-0.287	0.426	0.500	-	0.837
ANKMY2	LAS	1	0.050	0.358	0.888	1.052	0.971	-	No	1	0.050	0.358	0.888	-	0.971
APOA1	LAS	1	0.241	0.336	0.474	1.272	0.837	-	No	1	0.241	0.336	0.474	-	0.837
APOA2	LAS	1	-0.139	0.335	0.678	0.870	0.914	-	No	1	-0.139	0.335	0.678	-	0.914
APOBR	LAS	2	-0.003	0.021	0.895	0.997	0.971	-	No	2	-0.003	0.021	0.895	-	0.971
APOC1	LAS	2	-0.374	0.544	0.492	0.688	0.837	-	No	2	-0.374	0.544	0.492	-	0.837
ASPN	LAS	2	0.118	0.093	0.202	1.125	0.756	-	No	2	0.118	0.093	0.202	-	0.756
ATXN2L	LAS	1	-0.460	0.655	0.483	0.631	0.837	-	No	1	-0.460	0.655	0.483	-	0.837
B4GAT1	LAS	3	0.047	0.083	0.568	1.048	0.854	0.999	No	3	0.047	0.083	0.568	0.388	0.854
BAG4	LAS	1	-1.062	0.588	0.071	0.346	0.611	-	No	1	-1.062	0.588	0.071	-	0.611
BCAM	LAS	2	0.001	0.230	0.998	1.001	0.998	-	No	2	0.001	0.230	0.998	-	0.998
BMP6	LAS	2	0.139	0.146	0.341	1.150	0.837	-	No	2	0.139	0.146	0.341	-	0.837
BNIP3L	LAS	1	-0.332	0.423	0.433	0.718	0.837	-	No	1	-0.332	0.423	0.433	-	0.837
BRAP	LAS	1	1.928	0.604	0.001	6.877	0.069	-	No	1	1.928	0.604	0.001	-	0.069
BRSK2	LAS	1	-0.370	0.482	0.443	0.690	0.837	-	No	1	-0.370	0.482	0.443	-	0.837
CA12	LAS	2	0.132	0.078	0.092	1.141	0.611	-	No	2	0.132	0.078	0.092	-	0.611
CA9	LAS	1	-0.364	0.353	0.302	0.695	0.813	-	No	1	-0.364	0.353	0.302	-	0.813
CACNB3	LAS	1	-0.105	0.218	0.630	0.900	0.899	-	No	1	-0.105	0.218	0.630	-	0.899
CALCA	LAS	1	-0.162	0.212	0.444	0.851	0.837	-	No	1	-0.162	0.212	0.444	-	0.837
CALCOCO2	LAS	1	0.505	0.400	0.207	1.657	0.756	-	No	1	0.505	0.400	0.207	-	0.756
CCN3	LAS	3	-0.055	0.085	0.522	0.947	0.837	0.999	No	3	-0.055	0.085	0.522	0.440	0.837
CD14	LAS	1	-0.113	0.079	0.152	0.893	0.729	-	No	1	-0.113	0.079	0.152	-	0.729
CD164L2	LAS	1	0.068	0.053	0.199	1.070	0.756	-	No	1	0.068	0.053	0.199	-	0.756
CD46	LAS	1	0.048	0.275	0.860	1.049	0.960	-	No	1	0.048	0.275	0.860	-	0.960
CD59	LAS	1	-0.128	0.101	0.206	0.880	0.756	-	No	1	-0.128	0.101	0.206	-	0.756
CEP170	LAS	1	-0.218	0.346	0.530	0.804	0.837	-	No	1	-0.218	0.346	0.530	-	0.837
CERT	LAS	1	0.186	0.463	0.688	1.205	0.915	-	No	1	0.186	0.463	0.688	-	0.915
CFHR2	LAS	3	-0.012	0.026	0.654	0.988	0.914	0.999	No	3	-0.012	0.026	0.654	0.734	0.914
CFHR4	LAS	6	0.002	0.031	0.944	1.002	0.984	0.999	No	6	0.002	0.031	0.944	0.768	0.984
CLMP	LAS	2	-0.173	0.071	0.014	0.841	0.285	-	No	2	-0.173	0.071	0.014	-	0.285
COL1A1	LAS	1	-0.117	0.503	0.816	0.890	0.953	-	No	1	-0.117	0.503	0.816	-	0.953
COMP	LAS	1	-0.120	0.359	0.739	0.887	0.938	-	No	1	-0.120	0.359	0.739	-	0.938
CPXM1	LAS	1	0.025	0.071	0.720	1.026	0.938	-	No	1	0.025	0.071	0.720	-	0.938
CTSO	LAS	1	-0.086	0.124	0.487	0.918	0.837	-	No	1	-0.086	0.124	0.487	-	0.837
DAG1	LAS	1	0.201	0.371	0.588	1.223	0.854	-	No	1	0.201	0.371	0.588	-	0.854
DDHD2	LAS	1	-0.372	0.225	0.099	0.690	0.622	-	No	1	-0.372	0.225	0.099	-	0.622
DTX3	LAS	1	0.497	0.249	0.046	1.644	0.478	-	No	1	0.497	0.249	0.046	-	0.478
DUSP13	LAS	1	0.081	0.086	0.348	1.084	0.837	-	No	1	0.081	0.086	0.348	-	0.837
DUSP29	LAS	1	0.034	0.169	0.841	1.034	0.953	-	No	1	0.034	0.169	0.841	-	0.953
EDN1	LAS	1	0.194	0.185	0.294	1.214	0.813	-	No	1	0.194	0.185	0.294	-	0.813
EFEMP1	LAS	2	-0.194	0.178	0.276	0.824	0.813	-	No	2	-0.194	0.178	0.276	-	0.813
EIF4G3	LAS	1	-0.551	0.278	0.048	0.576	0.478	-	No	1	-0.551	0.278	0.048	-	0.478
ELOA	LAS	1	0.209	0.272	0.443	1.232	0.837	-	No	1	0.209	0.272	0.443	-	0.837
ENPEP	LAS	1	-1.424	0.588	0.015	0.241	0.285	-	No	1	-1.424	0.588	0.015	-	0.285
ERI1	LAS	1	0.400	0.306	0.191	1.492	0.756	-	No	1	0.400	0.306	0.191	-	0.756
ERP29	LAS	1	2.068	0.648	0.001	7.908	0.069	-	No	1	2.068	0.648	0.001	-	0.069
ESAM	LAS	1	-0.412	0.174	0.018	0.663	0.285	-	No	1	-0.412	0.174	0.018	-	0.285
FDX1	LAS	1	-0.251	0.457	0.582	0.778	0.854	-	No	1	-0.251	0.457	0.582	-	0.854

ST15; MR causal estimates for SBP-associated proteins on large artery stroke.

Causal candidates prioritized for LAS were marked as "Yes" in column "Prioritized". All estimates are from inverse variance weighted method (IVs >1) or Wald-ratio method (IV = 1). LAS, large artery stroke.

										Steiger filtering					
Exposure	Outcme	nsnp	Beta	SE	P-value	Odds ratio	FDR-corrected P-value	FDR-corrected Cochran's Q P-value	Prioritized	nsnp	Beta	SE	P-value	Cochran's Q P-value	FDR-corrected P-value
FES	LAS	1	-0.377	0.168	0.025	0.686	0.349	-	No	1	-0.377	0.168	0.025	-	0.349
FGF2	LAS	3	0.020	0.058	0.730	1.020	0.938	0.999	No	3	0.020	0.058	0.730	0.989	0.938
FGF5	LAS	3	0.034	0.035	0.337	1.034	0.837	0.999	No	3	0.034	0.035	0.337	0.999	0.837
FKBP7	LAS	1	-0.010	0.313	0.975	0.990	0.991	-	No	1	-0.010	0.313	0.975	-	0.991
FN1	LAS	2	-0.106	0.062	0.087	0.899	0.611	-	No	2	-0.106	0.062	0.087	-	0.611
FOXJ3	LAS	1	-0.129	0.329	0.695	0.879	0.919	-	No	1	-0.129	0.329	0.695	-	0.919
FOXO3	LAS	1	0.662	0.636	0.298	1.939	0.813	-	No	1	0.662	0.636	0.298	-	0.813
FUCA1	LAS	1	0.046	0.063	0.463	1.047	0.837	-	No	1	0.046	0.063	0.463	-	0.837
FURIN	LAS	1	0.381	0.129	0.003	1.463	0.113	-	No	1	0.381	0.129	0.003	-	0.113
GCHFR	LAS	1	-0.055	0.199	0.783	0.947	0.941	-	No	1	-0.055	0.199	0.783	-	0.941
GFER	LAS	1	-0.065	0.396	0.869	0.937	0.965	-	No	1	-0.065	0.396	0.869	-	0.965
GHR	LAS	1	0.032	0.035	0.362	1.032	0.837	-	No	1	0.032	0.035	0.362	-	0.837
GIMAP7	LAS	3	-0.002	0.041	0.956	0.998	0.991	0.999	No	3	-0.002	0.041	0.956	0.428	0.991
GORASP2	LAS	1	-0.401	0.439	0.361	0.670	0.837	-	No	1	-0.401	0.439	0.361	-	0.837
GRP	LAS	4	0.116	0.063	0.068	1.123	0.611	0.999	No	4	0.116	0.063	0.068	0.723	0.611
HADH	LAS	1	-0.433	0.418	0.300	0.648	0.813	-	No	1	-0.433	0.418	0.300	-	0.813
HHEX	LAS	1	-0.378	0.588	0.520	0.685	0.837	-	No	1	-0.378	0.588	0.520	-	0.837
HYAL1	LAS	1	-0.087	0.090	0.333	0.917	0.837	-	No	1	-0.087	0.090	0.333	-	0.837
ICAM2	LAS	1	0.487	0.424	0.251	1.628	0.813	-	No	1	0.487	0.424	0.251	-	0.813
IDUA	LAS	1	0.010	0.045	0.833	1.010	0.953	-	No	1	0.010	0.045	0.833	-	0.953
IFI30	LAS	2	-0.053	0.051	0.297	0.948	0.813	-	No	2	-0.053	0.051	0.297	-	0.813
IFNGR2	LAS	3	0.008	0.022	0.732	1.008	0.938	0.999	No	3	0.008	0.022	0.732	0.893	0.938
IGFBP3	LAS	2	0.048	0.421	0.908	1.050	0.971	-	No	2	0.048	0.421	0.908	-	0.971
IMMT	LAS	1	-0.202	0.184	0.271	0.817	0.813	-	No	1	-0.202	0.184	0.271	-	0.813
IMPA1	LAS	1	-0.039	0.114	0.736	0.962	0.938	-	No	1	-0.039	0.114	0.736	-	0.938
ITGAL	LAS	1	-0.513	0.789	0.516	0.599	0.837	-	No	1	-0.513	0.789	0.516	-	0.837
ITIH1	LAS	1	0.046	0.164	0.777	1.047	0.941	-	No	1	0.046	0.164	0.777	-	0.941
KIFBP	LAS	1	0.020	0.491	0.967	1.021	0.991	-	No	1	0.020	0.491	0.967	-	0.991
LMOD1	LAS	1	-0.134	0.133	0.313	0.874	0.824	-	No	1	-0.134	0.133	0.313	-	0.824
LRIG1	LAS	3	0.019	0.027	0.498	1.019	0.837	0.999	No	3	0.019	0.027	0.498	0.924	0.837
LYAR	LAS	1	0.153	0.494	0.757	1.165	0.941	-	No	1	0.153	0.494	0.757	-	0.941
M6PR	LAS	1	-0.104	0.121	0.390	0.901	0.837	-	No	1	-0.104	0.121	0.390	-	0.837
MANEAL	LAS	1	-0.501	0.619	0.418	0.606	0.837	-	No	1	-0.501	0.619	0.418	-	0.837
MANSC4	LAS	2	-0.049	0.042	0.242	0.952	0.813	-	No	2	-0.049	0.042	0.242	-	0.813
MAP4K5	LAS	1	0.036	0.124	0.769	1.037	0.941	-	No	1	0.036	0.124	0.769	-	0.941
MDH1	LAS	1	-0.053	0.253	0.833	0.948	0.953	-	No	1	-0.053	0.253	0.833	-	0.953
MEGF9	LAS	1	-0.250	0.072	0.001	0.779	0.062	-	No	1	-0.250	0.072	0.001	-	0.062
MPHOSPH8	LAS	1	-1.067	0.637	0.094	0.344	0.611	-	No	1	-1.067	0.637	0.094	-	0.611
MPI	LAS	1	0.028	0.104	0.783	1.029	0.941	-	No	1	0.028	0.104	0.783	-	0.941
MSRA	LAS	1	0.281	0.392	0.473	1.324	0.837	-	No	1	0.281	0.392	0.473	-	0.837
MST1	LAS	2	-0.022	0.043	0.611	0.979	0.877	-	No	2	-0.022	0.043	0.611	-	0.877
MVK	LAS	1	0.076	0.258	0.768	1.079	0.941	-	No	1	0.076	0.258	0.768	-	0.941
NADK	LAS	1	-0.122	0.097	0.209	0.885	0.756	-	No	1	-0.122	0.097	0.209	-	0.756
NAGA	LAS	2	0.090	0.142	0.523	1.095	0.837	-	No	2	0.090	0.142	0.523	-	0.837
NFE2	LAS	1	-0.544	0.325	0.094	0.580	0.611	-	No	1	-0.544	0.325	0.094	-	0.611
NGF	LAS	1	0.887	1.081	0.412	2.427	0.837	-	No	1	0.887	1.081	0.412	-	0.837
NPPB	LAS	1	-0.190	0.218	0.384	0.827	0.837	-	No	1	-0.190	0.218	0.384	-	0.837
NTRK3	LAS	2	-0.172	0.103	0.093	0.842	0.611	-	No	2	-0.172	0.103	0.093	-	0.611
NUCB2	LAS	2	-0.129	0.054	0.016	0.879	0.285	-	No	2	-0.129	0.054	0.016	-	0.285
NUDT5	LAS	1	-0.462	0.395	0.243	0.630	0.813	-	No	1	-0.462	0.395	0.243	-	0.813
NUMB	LAS	1	0.175	0.250	0.484	1.191	0.837	-	No	1	0.175	0.250	0.484	-	0.837
OGA	LAS	1	0.081	0.191	0.673	1.084	0.914	-	No	1	0.081	0.191	0.673	-	0.914
OPLAH	LAS	1	0.115	0.124	0.353	1.122	0.837	-	No	1	0.115	0.124	0.353	-	0.837
PCBP2	LAS	1	-0.744	0.445	0.094	0.475	0.611	-	No	1	-0.744	0.445	0.094	-	0.611
PCSK7	LAS	2	0.092	0.067	0.170	1.096	0.756	-	No	2	0.092	0.067	0.170	-	0.756
PDE5A	LAS	2	-0.090	0.134	0.503	0.914	0.837	-	No	2	-0.090	0.134	0.503	-	0.837

ST15; MR causal estimates for SBP-associated proteins on large artery stroke.

Causal candidates prioritized for LAS were marked as "Yes" in column "Prioritized". All estimates are from inverse variance weighted method (IVs >1) or Wald-ratio method (IV = 1). LAS, large artery stroke.

										Steiger filtering					
Exposure	Outcome	nsnp	Beta	SE	P-value	Odds ratio	FDR-corrected P-value	FDR-corrected Cochran's Q P-value	Prioritized	nsnp	Beta	SE	P-value	Cochran's Q P-value	FDR-corrected P-value
PDGFRA	LAS	1	0.834	0.492	0.090	2.304	0.611	-	No	1	0.834	0.492	0.090	-	0.611
PECAM1	LAS	1	-0.958	0.734	0.191	0.384	0.756	-	No	1	-0.958	0.734	0.191	-	0.756
PFKFB2	LAS	1	0.083	0.119	0.488	1.086	0.837	-	No	1	0.083	0.119	0.488	-	0.837
PGF	LAS	1	0.011	0.143	0.937	1.011	0.984	-	No	1	0.011	0.143	0.937	-	0.984
PHLDB1	LAS	1	0.437	0.483	0.365	1.549	0.837	-	No	1	0.437	0.483	0.365	-	0.837
PKD1	LAS	1	-1.385	0.480	0.004	0.250	0.124	-	No	1	-1.385	0.480	0.004	-	0.124
PLA2G1B	LAS	1	-0.041	0.439	0.925	0.960	0.983	-	No	1	-0.041	0.439	0.925	-	0.983
PMS1	LAS	1	-0.287	0.477	0.547	0.750	0.848	-	No	1	-0.287	0.477	0.547	-	0.848
PPP1R14D	LAS	1	-0.055	0.429	0.897	0.946	0.971	-	No	1	-0.055	0.429	0.897	-	0.971
PRG2	LAS	1	-0.083	0.224	0.713	0.921	0.938	-	No	1	-0.083	0.224	0.713	-	0.938
PRKAB1	LAS	1	0.143	0.132	0.278	1.154	0.813	-	No	1	0.143	0.132	0.278	-	0.813
PSMD5	LAS	1	1.430	0.452	0.002	4.178	0.069	-	No	1	1.430	0.452	0.002	-	0.069
PSRC1	LAS	1	-0.341	0.201	0.090	0.711	0.611	-	No	1	-0.341	0.201	0.090	-	0.611
PTRHD1	LAS	1	0.089	0.597	0.882	1.093	0.969	-	No	1	0.089	0.597	0.882	-	0.969
QPCT	LAS	2	0.067	0.106	0.531	1.069	0.837	-	No	2	0.067	0.106	0.531	-	0.837
RABEPK	LAS	1	0.274	0.144	0.058	1.315	0.556	-	No	1	0.274	0.144	0.058	-	0.556
RARRES1	LAS	5	0.002	0.046	0.971	1.002	0.991	0.999	No	5	0.002	0.046	0.971	0.929	0.991
RARRES2	LAS	1	0.158	0.117	0.177	1.171	0.756	-	No	1	0.158	0.117	0.177	-	0.756
RELT	LAS	2	-0.102	0.078	0.189	0.903	0.756	-	No	2	-0.102	0.078	0.189	-	0.756
RSPO3	LAS	1	0.122	0.141	0.387	1.129	0.837	-	No	1	0.122	0.141	0.387	-	0.837
SCARA5	LAS	4	-0.041	0.065	0.527	0.959	0.837	0.999	No	4	-0.041	0.065	0.527	0.989	0.837
SDCCAG8	LAS	1	0.073	0.107	0.498	1.075	0.837	-	No	1	0.073	0.107	0.498	-	0.837
SDHB	LAS	1	0.329	0.370	0.375	1.389	0.837	-	No	1	0.329	0.370	0.375	-	0.837
SELENOP	LAS	1	0.121	0.217	0.577	1.129	0.854	-	No	1	0.121	0.217	0.577	-	0.854
SEMA6C	LAS	1	-0.492	0.245	0.045	0.612	0.478	-	No	1	-0.492	0.245	0.045	-	0.478
SERPINI1	LAS	1	0.018	0.058	0.759	1.018	0.941	-	No	1	0.018	0.058	0.759	-	0.941
SH2B3	LAS	1	0.643	0.314	0.041	1.902	0.478	-	No	1	0.643	0.314	0.041	-	0.478
SHMT1	LAS	4	-0.027	0.032	0.410	0.974	0.837	0.999	No	4	-0.027	0.032	0.410	0.949	0.837
SIL1	LAS	1	-0.075	0.372	0.841	0.928	0.953	-	No	1	-0.075	0.372	0.841	-	0.953
SLC16A1	LAS	1	-0.056	0.360	0.877	0.946	0.969	-	No	1	-0.056	0.360	0.877	-	0.969
SLC39A14	LAS	1	-0.280	0.241	0.246	0.756	0.813	-	No	1	-0.280	0.241	0.246	-	0.813
SLC9A3R2	LAS	1	0.243	0.226	0.283	1.275	0.813	-	No	1	0.243	0.226	0.283	-	0.813
SMOC2	LAS	3	-0.045	0.066	0.498	0.956	0.837	0.999	No	3	-0.045	0.066	0.498	0.298	0.837
SOST	LAS	1	-0.262	0.445	0.556	0.770	0.848	-	No	1	-0.262	0.445	0.556	-	0.848
SPINK8	LAS	1	0.115	0.053	0.031	1.122	0.397	-	No	1	0.115	0.053	0.031	-	0.397
SPRED2	LAS	1	-0.831	0.335	0.013	0.436	0.285	-	No	1	-0.831	0.335	0.013	-	0.285
TARBP2	LAS	1	-0.800	0.478	0.094	0.449	0.611	-	No	1	-0.800	0.478	0.094	-	0.611
TBC1D23	LAS	1	0.077	0.139	0.581	1.080	0.854	-	No	1	0.077	0.139	0.581	-	0.854
TEK	LAS	2	-0.293	0.415	0.481	0.746	0.837	-	No	2	-0.293	0.415	0.481	-	0.837
TGFB2	LAS	1	0.036	0.156	0.818	1.037	0.953	-	No	1	0.036	0.156	0.818	-	0.953
TIE1	LAS	1	0.102	0.114	0.372	1.108	0.837	-	No	1	0.102	0.114	0.372	-	0.837
TJAP1	LAS	1	0.005	0.539	0.993	1.005	0.998	-	No	1	0.005	0.539	0.993	-	0.998
TNFRSF17	LAS	1	-0.001	0.167	0.993	0.999	0.998	-	No	1	-0.001	0.167	0.993	-	0.998
TNFSF12	LAS	2	0.004	0.137	0.978	1.004	0.991	-	No	2	0.004	0.137	0.978	-	0.991
TWF2	LAS	1	-0.149	0.346	0.666	0.861	0.914	-	No	1	-0.149	0.346	0.666	-	0.914
UBE2L6	LAS	2	0.061	0.091	0.502	1.063	0.837	-	No	2	0.061	0.091	0.502	-	0.837
UMOD	LAS	3	0.035	0.029	0.229	1.035	0.809	0.999	No	3	0.035	0.029	0.229	0.873	0.809
VSIG2	LAS	1	0.278	0.118	0.018	1.321	0.285	-	No	1	0.278	0.118	0.018	-	0.285
WARS	LAS	1	0.130	0.109	0.231	1.139	0.809	-	No	1	0.130	0.109	0.231	-	0.809
WASHC3	LAS	1	0.161	0.295	0.585	1.175	0.854	-	No	1	0.161	0.295	0.585	-	0.854
WWP2	LAS	1	-0.148	0.141	0.295	0.862	0.813	-	No	1	-0.148	0.141	0.295	-	0.813
YOD1	LAS	1	0.306	0.200	0.127	1.358	0.682	-	No	1	0.306	0.200	0.127	-	0.682
ZBTB17	LAS	1	-0.183	0.893	0.837	0.833	0.953	-	No	1	-0.183	0.893	0.837	-	0.953
ZFYVE19	LAS	1	0.007	0.059	0.902	1.007	0.971	-	No	1	0.007	0.059	0.902	-	0.971

nsnp = number of single nucleotide polymorphisms; SE = standard error; Cochran's Q P-value = p-value from Cochran's Q test assessing heterogeneity; FDR=false discovery rate

ST16; MR causal estimates for DBP-associated proteins on large artery stroke.

Causal candidates prioritized for LAS were marked as "Yes" in column "Prioritized". All estimates are from inverse variance weighted method (IVs >1) or Wald-ratio method (IV = 1). LAS, large artery stroke.

										Steiger filtering					
**Exposure**	**Outcome**	**nsnp**	**Beta**	**SE**	**P-value**	**Odds ratio**	**FDR-corrected P-value**	**FDR-corrected Cochran's Q P-value**	**Prioritized**	**nsnp**	**Beta**	**SE**	**P-value**	**Cochran's Q P-value**	**FDR-corrected P-value**
AAMDC	LAS	1	-0.033	0.033	0.312	0.967	0.824	-	No	1	-0.033	0.033	0.312	-	0.824
ABO	LAS	6	0.108	0.020	0.000	1.114	0.000	0.999	Yes	6	0.108	0.020	0.000	0.669	0.000
ACOX1	LAS	1	0.396	0.297	0.183	1.486	0.756	-	No	1	0.396	0.297	0.183	-	0.756
ADM	LAS	1	-0.889	0.598	0.137	0.411	0.703	-	No	1	-0.889	0.598	0.137	-	0.703
AMFR	LAS	1	-0.428	0.525	0.416	0.652	0.837	-	No	1	-0.428	0.525	0.416	-	0.837
AMOTL2	LAS	1	-0.287	0.426	0.500	0.750	0.837	-	No	1	-0.287	0.426	0.500	-	0.837
ANKMY2	LAS	1	0.050	0.358	0.888	1.052	0.971	-	No	1	0.050	0.358	0.888	-	0.971
AOC1	LAS	3	0.055	0.080	0.490	1.057	0.837	0.999	No	3	0.055	0.080	0.490	0.668	0.837
AOC3	LAS	2	0.005	0.052	0.931	1.005	0.984	-	No	2	0.005	0.052	0.931	-	0.984
APOA1	LAS	1	0.241	0.336	0.474	1.272	0.837	-	No	1	0.241	0.336	0.474	-	0.837
APOBR	LAS	2	-0.003	0.021	0.895	0.997	0.971	-	No	2	-0.003	0.021	0.895	-	0.971
APOC1	LAS	2	-0.374	0.544	0.492	0.688	0.837	-	No	2	-0.374	0.544	0.492	-	0.837
ARSB	LAS	3	0.050	0.156	0.747	1.052	0.938	0.777	No	3	0.050	0.156	0.747	0.073	0.938
ATXN2L	LAS	1	-0.460	0.655	0.483	0.631	0.837	-	No	1	-0.460	0.655	0.483	-	0.837
AXL	LAS	2	0.321	0.206	0.120	1.378	0.663	-	No	2	0.321	0.206	0.120	-	0.663
BAG4	LAS	1	-1.062	0.588	0.071	0.346	0.611	-	No	1	-1.062	0.588	0.071	-	0.611
BNIP3L	LAS	1	-0.332	0.423	0.433	0.718	0.837	-	No	1	-0.332	0.423	0.433	-	0.837
CA12	LAS	2	0.132	0.078	0.092	1.141	0.611	-	No	2	0.132	0.078	0.092	-	0.611
CACNB3	LAS	1	-0.105	0.218	0.630	0.900	0.899	-	No	1	-0.105	0.218	0.630	-	0.899
CALCA	LAS	1	-0.162	0.212	0.444	0.851	0.837	-	No	1	-0.162	0.212	0.444	-	0.837
CEP170	LAS	1	-0.218	0.346	0.530	0.804	0.837	-	No	1	-0.218	0.346	0.530	-	0.837
CFHR4	LAS	6	0.002	0.031	0.944	1.002	0.984	0.999	No	6	0.002	0.031	0.944	0.768	0.984
CHMP1A	LAS	1	-0.276	0.261	0.290	0.758	0.813	-	No	1	-0.276	0.261	0.290	-	0.813
CLIC5	LAS	1	-0.129	0.191	0.500	0.879	0.837	-	No	1	-0.129	0.191	0.500	-	0.837
COL1A1	LAS	1	-0.117	0.503	0.816	0.890	0.953	-	No	1	-0.117	0.503	0.816	-	0.953
CTF1	LAS	1	-0.145	0.438	0.741	0.865	0.938	-	No	1	-0.145	0.438	0.741	-	0.938
DAG1	LAS	1	0.201	0.371	0.588	1.223	0.854	-	No	1	0.201	0.371	0.588	-	0.854
DARS1	LAS	1	0.288	0.297	0.333	1.334	0.837	-	No	1	0.288	0.297	0.333	-	0.837
DBN1	LAS	1	-0.613	0.438	0.162	0.542	0.745	-	No	1	-0.613	0.438	0.162	-	0.745
DDHD2	LAS	1	-0.372	0.225	0.099	0.690	0.622	-	No	1	-0.372	0.225	0.099	-	0.622
DENR	LAS	1	-1.107	0.686	0.106	0.331	0.644	-	No	1	-1.107	0.686	0.106	-	0.644
DNER	LAS	5	0.039	0.091	0.667	1.040	0.914	0.777	No	5	0.039	0.091	0.667	0.102	0.914
DOK2	LAS	1	-0.129	0.397	0.746	0.879	0.938	-	No	1	-0.129	0.397	0.746	-	0.938
DPEP1	LAS	4	0.014	0.271	0.960	1.014	0.991	0.501	No	4	0.014	0.271	0.960	0.019	0.991
DPP4	LAS	1	-0.020	0.103	0.848	0.980	0.956	-	No	1	-0.020	0.103	0.848	-	0.956
DUSP13	LAS	1	0.081	0.086	0.348	1.084	0.837	-	No	1	0.081	0.086	0.348	-	0.837
EFNA1	LAS	1	0.012	0.068	0.856	1.012	0.960	-	No	1	0.012	0.068	0.856	-	0.960
EIF4G3	LAS	1	-0.551	0.278	0.048	0.576	0.478	-	No	1	-0.551	0.278	0.048	-	0.478
ELOA	LAS	1	0.209	0.272	0.443	1.232	0.837	-	No	1	0.209	0.272	0.443	-	0.837
ENPEP	LAS	1	-1.424	0.588	0.015	0.241	0.285	-	No	1	-1.424	0.588	0.015	-	0.285
EPHA2	LAS	2	-0.030	0.268	0.910	0.970	0.971	-	No	2	-0.030	0.268	0.910	-	0.971
EPO	LAS	1	0.331	0.305	0.277	1.392	0.813	-	No	1	0.331	0.305	0.277	-	0.813
ERI1	LAS	1	0.400	0.306	0.191	1.492	0.756	-	No	1	0.400	0.306	0.191	-	0.756
ESAM	LAS	1	-0.412	0.174	0.018	0.663	0.285	-	No	1	-0.412	0.174	0.018	-	0.285
F12	LAS	3	-0.111	0.273	0.683	0.895	0.915	0.999	No	3	-0.111	0.273	0.683	0.675	0.915
FADD	LAS	1	-0.242	0.281	0.389	0.785	0.837	-	No	1	-0.242	0.281	0.389	-	0.837
FDX1	LAS	1	-0.251	0.457	0.582	0.778	0.854	-	No	1	-0.251	0.457	0.582	-	0.854
FES	LAS	1	-0.377	0.168	0.025	0.686	0.349	-	No	1	-0.377	0.168	0.025	-	0.349
FGF5	LAS	3	0.034	0.035	0.337	1.034	0.837	0.999	No	3	0.034	0.035	0.337	0.999	0.837
FOXJ3	LAS	1	-0.129	0.329	0.695	0.879	0.919	-	No	1	-0.129	0.329	0.695	-	0.919
FURIN	LAS	1	0.381	0.129	0.003	1.463	0.113	-	No	1	0.381	0.129	0.003	-	0.113
GFER	LAS	1	-0.065	0.396	0.869	0.937	0.965	-	No	1	-0.065	0.396	0.869	-	0.965
GIMAP7	LAS	3	-0.002	0.041	0.956	0.998	0.991	0.999	No	3	-0.002	0.041	0.956	0.428	0.991
GLO1	LAS	2	-0.071	0.116	0.544	0.932	0.848	-	No	2	-0.071	0.116	0.544	-	0.848
GORASP2	LAS	1	-0.401	0.439	0.361	0.670	0.837	-	No	1	-0.401	0.439	0.361	-	0.837
GRP	LAS	4	0.116	0.063	0.068	1.123	0.611	0.999	No	4	0.116	0.063	0.068	0.723	0.611

ST16; MR causal estimates for DBP-associated proteins on large artery stroke.

Causal candidates prioritized for LAS were marked as "Yes" in column "Prioritized". All estimates are from inverse variance weighted method (IVs >1) or Wald-ratio method (IV = 1). LAS, large artery stroke.

										Steiger filtering					
Exposure	Outcme	nsnp	Beta	SE	P-value	Odds ratio	FDR-corrected P-value	FDR-corrected Cochran's Q P-value	Prioritized	nsnp	Beta	SE	P-value	Cochran's Q P-value	FDR-corrected P-value
HADH	LAS	1	-0.433	0.418	0.300	0.648	0.813	-	No	1	-0.433	0.418	0.300	-	0.813
HHEX	LAS	1	-0.378	0.588	0.520	0.685	0.837	-	No	1	-0.378	0.588	0.520	-	0.837
HPGDS	LAS	6	-0.024	0.045	0.601	0.977	0.868	0.999	No	6	-0.024	0.045	0.601	0.910	0.868
ICAM1	LAS	4	-0.039	0.059	0.507	0.961	0.837	0.999	No	4	-0.039	0.059	0.507	0.281	0.837
ICAM4	LAS	1	0.142	0.203	0.484	1.153	0.837	-	No	1	0.142	0.203	0.484	-	0.837
IFIT3	LAS	1	0.096	0.208	0.645	1.100	0.914	-	No	1	0.096	0.208	0.645	-	0.914
IGFBP3	LAS	2	0.048	0.421	0.908	1.050	0.971	-	No	2	0.048	0.421	0.908	-	0.971
IL1RL1	LAS	5	-0.018	0.042	0.660	0.982	0.914	0.777	No	5	-0.018	0.042	0.660	0.115	0.914
IMMT	LAS	1	-0.202	0.184	0.271	0.817	0.813	-	No	1	-0.202	0.184	0.271	-	0.813
ITGAL	LAS	1	-0.513	0.789	0.516	0.599	0.837	-	No	1	-0.513	0.789	0.516	-	0.837
KIF22	LAS	1	0.465	0.646	0.472	1.591	0.837	-	No	1	0.465	0.646	0.472	-	0.837
KIFBP	LAS	1	0.020	0.491	0.967	1.021	0.991	-	No	1	0.020	0.491	0.967	-	0.991
LAYN	LAS	1	-0.082	0.062	0.183	0.921	0.756	-	No	1	-0.082	0.062	0.183	-	0.756
LMOD1	LAS	1	-0.134	0.133	0.313	0.874	0.824	-	No	1	-0.134	0.133	0.313	-	0.824
M6PR	LAS	1	-0.104	0.121	0.390	0.901	0.837	-	No	1	-0.104	0.121	0.390	-	0.837
MAP4K5	LAS	1	0.036	0.124	0.769	1.037	0.941	-	No	1	0.036	0.124	0.769	-	0.941
MFGE8	LAS	2	0.035	0.064	0.587	1.035	0.854	-	No	2	0.035	0.064	0.587	-	0.854
MPHOSPH8	LAS	1	-1.067	0.637	0.094	0.344	0.611	-	No	1	-1.067	0.637	0.094	-	0.611
MSRA	LAS	1	0.281	0.392	0.473	1.324	0.837	-	No	1	0.281	0.392	0.473	-	0.837
MST1	LAS	2	-0.022	0.043	0.611	0.979	0.877	-	No	2	-0.022	0.043	0.611	-	0.877
NADK	LAS	1	-0.122	0.097	0.209	0.885	0.756	-	No	1	-0.122	0.097	0.209	-	0.756
NGF	LAS	1	0.887	1.081	0.412	2.427	0.837	-	No	1	0.887	1.081	0.412	-	0.837
NOMO1	LAS	1	0.359	0.238	0.132	1.432	0.694	-	No	1	0.359	0.238	0.132	-	0.694
NOS3	LAS	1	-0.327	0.486	0.500	0.721	0.837	-	No	1	-0.327	0.486	0.500	-	0.837
NPPB	LAS	1	-0.190	0.218	0.384	0.827	0.837	-	No	1	-0.190	0.218	0.384	-	0.837
NUCB2	LAS	2	-0.129	0.054	0.016	0.879	0.285	-	No	2	-0.129	0.054	0.016	-	0.285
PAM	LAS	2	-0.053	0.050	0.292	0.949	0.813	-	No	2	-0.053	0.050	0.292	-	0.813
PARP1	LAS	1	-0.045	0.185	0.806	0.956	0.953	-	No	1	-0.045	0.185	0.806	-	0.953
PCOLCE	LAS	1	0.188	0.138	0.173	1.207	0.756	-	No	1	0.188	0.138	0.173	-	0.756
PCSK7	LAS	2	0.092	0.067	0.170	1.096	0.756	-	No	2	0.092	0.067	0.170	-	0.756
PDE5A	LAS	2	-0.090	0.134	0.503	0.914	0.837	-	No	2	-0.090	0.134	0.503	-	0.837
PDIA3	LAS	1	0.145	0.527	0.783	1.156	0.941	-	No	1	0.145	0.527	0.783	-	0.941
PECAM1	LAS	1	-0.958	0.734	0.191	0.384	0.756	-	No	1	-0.958	0.734	0.191	-	0.756
PFKFB2	LAS	1	0.083	0.119	0.488	1.086	0.837	-	No	1	0.083	0.119	0.488	-	0.837
PLA2G1B	LAS	1	-0.041	0.439	0.925	0.960	0.983	-	No	1	-0.041	0.439	0.925	-	0.983
PLXDC2	LAS	3	-0.199	0.215	0.354	0.820	0.837	0.999	No	3	-0.199	0.215	0.354	0.380	0.837
PMS1	LAS	1	-0.287	0.477	0.547	0.750	0.848	-	No	1	-0.287	0.477	0.547	-	0.848
PRDX1	LAS	1	0.494	0.349	0.156	1.639	0.735	-	No	1	0.494	0.349	0.156	-	0.735
PROCR	LAS	1	-0.035	0.050	0.483	0.966	0.837	-	No	1	-0.035	0.050	0.483	-	0.837
PRSS53	LAS	2	0.051	0.046	0.274	1.052	0.813	-	No	2	0.051	0.046	0.274	-	0.813
PRTFDC1	LAS	1	0.185	0.444	0.677	1.203	0.914	-	No	1	0.185	0.444	0.677	-	0.914
PSRC1	LAS	1	-0.341	0.201	0.090	0.711	0.611	-	No	1	-0.341	0.201	0.090	-	0.611
PTPRF	LAS	1	-0.429	0.274	0.118	0.651	0.663	-	No	1	-0.429	0.274	0.118	-	0.663
PTRHD1	LAS	1	0.089	0.597	0.882	1.093	0.969	-	No	1	0.089	0.597	0.882	-	0.969
PYDC1	LAS	1	-0.177	0.074	0.018	0.838	0.285	-	No	1	-0.177	0.074	0.018	-	0.285
RELT	LAS	2	-0.102	0.078	0.189	0.903	0.756	-	No	2	-0.102	0.078	0.189	-	0.756
SDC1	LAS	2	-0.171	0.160	0.284	0.843	0.813	-	No	2	-0.171	0.160	0.284	-	0.813
SDCCAG8	LAS	1	0.073	0.107	0.498	1.075	0.837	-	No	1	0.073	0.107	0.498	-	0.837
SDHB	LAS	1	0.329	0.370	0.375	1.389	0.837	-	No	1	0.329	0.370	0.375	-	0.837
SERPINI1	LAS	1	0.018	0.058	0.759	1.018	0.941	-	No	1	0.018	0.058	0.759	-	0.941
SH2B3	LAS	1	0.643	0.314	0.041	1.902	0.478	-	No	1	0.643	0.314	0.041	-	0.478
SHMT1	LAS	4	-0.027	0.032	0.410	0.974	0.837	0.999	No	4	-0.027	0.032	0.410	0.949	0.837
SLC16A1	LAS	1	-0.056	0.360	0.877	0.946	0.969	-	No	1	-0.056	0.360	0.877	-	0.969
SLC9A3R2	LAS	1	0.243	0.226	0.283	1.275	0.813	-	No	1	0.243	0.226	0.283	-	0.813
SMTN	LAS	1	-0.947	0.589	0.108	0.388	0.644	-	No	1	-0.947	0.589	0.108	-	0.644
SPINK8	LAS	1	0.115	0.053	0.031	1.122	0.397	-	No	1	0.115	0.053	0.031	-	0.397

ST16; MR causal estimates for DBP-associated proteins on large artery stroke.

Causal candidates prioritized for LAS were marked as "Yes" in column "Prioritized". All estimates are from inverse variance weighted method (IVs >1) or Wald-ratio method (IV = 1). LAS, large artery stroke.

										Steiger filtering					
**Exposure**	**Outocme**	**nsnp**	**Beta**	**SE**	**P-value**	**Odds ratio**	**FDR-corrected P-value**	**FDR-corrected Cochran's Q P-value**	**Prioritized**	**nsnp**	**Beta**	**SE**	**P-value**	**Cochran's Q P-value**	**FDR-corrected P-value**
STC1	LAS	1	0.609	0.415	0.143	1.839	0.716	-	No	1	0.609	0.415	0.143	-	0.716
STX4	LAS	1	0.847	0.536	0.114	2.333	0.662	-	No	1	0.847	0.536	0.114	-	0.662
TBC1D17	LAS	1	0.012	0.059	0.839	1.012	0.953	-	No	1	0.012	0.059	0.839	-	0.953
TIE1	LAS	1	0.102	0.114	0.372	1.108	0.837	-	No	1	0.102	0.114	0.372	-	0.837
TJAP1	LAS	1	0.005	0.539	0.993	1.005	0.998	-	No	1	0.005	0.539	0.993	-	0.998
TMEM106A	LAS	1	-0.438	0.217	0.044	0.646	0.478	-	No	1	-0.438	0.217	0.044	-	0.478
TNFRSF17	LAS	1	-0.001	0.167	0.993	0.999	0.998	-	No	1	-0.001	0.167	0.993	-	0.998
TNFSF12	LAS	2	0.004	0.137	0.978	1.004	0.991	-	No	2	0.004	0.137	0.978	-	0.991
TP53	LAS	1	0.028	0.387	0.942	1.029	0.984	-	No	1	0.028	0.387	0.942	-	0.984
UMOD	LAS	3	0.035	0.029	0.229	1.035	0.809	0.999	No	3	0.035	0.029	0.229	0.873	0.809
UXS1	LAS	2	0.189	0.219	0.389	1.208	0.837	-	No	2	0.189	0.219	0.389	-	0.837
VAT1	LAS	1	-0.356	0.153	0.020	0.700	0.296	-	No	1	-0.356	0.153	0.020	-	0.296
VSIG2	LAS	1	0.278	0.118	0.018	1.321	0.285	-	No	1	0.278	0.118	0.018	-	0.285
WNT9A	LAS	2	0.142	0.237	0.550	1.152	0.848	-	No	2	0.142	0.237	0.550	-	0.848
WWP2	LAS	1	-0.148	0.141	0.295	0.862	0.813	-	No	1	-0.148	0.141	0.295	-	0.813
YAP1	LAS	1	-0.318	0.338	0.346	0.727	0.837	-	No	1	-0.318	0.338	0.346	-	0.837
YOD1	LAS	1	0.306	0.200	0.127	1.358	0.682	-	No	1	0.306	0.200	0.127	-	0.682
ZBTB17	LAS	1	-0.183	0.893	0.837	0.833	0.953	-	No	1	-0.183	0.893	0.837	-	0.953

nsnp = number of single nucleotide polymorphisms; SE = standard error; Cochran's Q P-value = p-value from Cochran's Q test assessing heterogeneity; FDR=false discovery rate

ST17; MR causal estimates for CAD and stroke on BP-associated proteins.

All estimates are from inverse variance weighted method (IVs >1) or Wald-ratio method (IV = 1). CAD, coronary artery disease; AS, all strokes; AIS, ischemic stroke; SVS, small vessel stroke; CES, cardioembolic stroke; LAS, large artery stroke

Exposure	Outocme	nsnp	Beta	SE	P-value	Odds ratio	FDR-corrected P-value	FDR-corrected Cochran's Q P-value
CAD	AAMDC	163	0.018	0.013	0.165	1.018	0.431	0.000
CAD	ABO	163	0.053	0.073	0.468	1.054	0.693	0.000
CAD	ACADM	163	0.003	0.012	0.765	1.003	0.864	0.059
CAD	ACOX1	163	0.005	0.011	0.662	1.005	0.818	0.153
CAD	ACRBP	163	0.000	0.014	0.976	1.000	0.983	0.000
CAD	ADAM23	163	-0.037	0.019	0.054	0.964	0.273	0.000
CAD	ADAMTS1	163	-0.015	0.012	0.229	0.985	0.513	0.002
CAD	ADAMTS13	163	-0.011	0.016	0.493	0.989	0.703	0.000
CAD	ADAMTS15	163	0.015	0.016	0.377	1.015	0.632	0.000
CAD	ADAMTS16	163	-0.026	0.012	0.033	0.975	0.251	0.000
CAD	ADAMTS4	163	-0.009	0.011	0.419	0.991	0.667	0.122
CAD	ADAMTS8	163	-0.032	0.012	0.009	0.968	0.127	0.000
CAD	ADAMTSL5	163	-0.026	0.012	0.036	0.974	0.253	0.000
CAD	ADCYAP1R1	163	-0.008	0.011	0.487	0.992	0.703	0.123
CAD	ADM	163	0.025	0.013	0.052	1.025	0.273	0.000
CAD	AGRP	163	0.076	0.025	0.003	1.079	0.062	0.000
CAD	AMFR	163	0.009	0.012	0.451	1.009	0.686	0.023
CAD	AMOTL2	163	0.007	0.013	0.578	1.007	0.761	0.000
CAD	ANKMY2	163	0.006	0.013	0.641	1.006	0.804	0.000
CAD	AOC1	163	-0.012	0.011	0.276	0.988	0.550	0.092
CAD	AOC3	163	-0.032	0.015	0.034	0.968	0.251	0.000
CAD	APOA1	163	-0.074	0.024	0.002	0.929	0.059	0.000
CAD	APOA2	163	-0.021	0.019	0.274	0.980	0.550	0.000
CAD	APOBR	163	-0.030	0.011	0.007	0.971	0.097	0.000
CAD	APOC1	163	-0.104	0.029	0.000	0.901	0.009	0.000
CAD	ARHGEF12	163	0.028	0.014	0.043	1.028	0.264	0.000
CAD	ARSB	163	0.002	0.017	0.912	1.002	0.951	0.000
CAD	ASPN	163	-0.057	0.015	0.000	0.944	0.004	0.000
CAD	ATXN2L	163	0.015	0.013	0.270	1.015	0.546	0.000
CAD	AXL	163	0.002	0.024	0.943	1.002	0.971	0.000
CAD	B4GAT1	163	-0.027	0.017	0.116	0.974	0.396	0.000
CAD	BAG4	163	0.004	0.015	0.767	1.004	0.864	0.000
CAD	BCAM	163	-0.018	0.022	0.404	0.982	0.655	0.000
CAD	BMP6	163	-0.006	0.017	0.721	0.994	0.850	0.000
CAD	BNIP3L	163	0.021	0.014	0.137	1.021	0.399	0.000
CAD	BRAP	163	0.016	0.013	0.236	1.016	0.520	0.000
CAD	BRSK2	163	0.004	0.011	0.748	1.004	0.855	0.005
CAD	CA12	163	-0.032	0.015	0.030	0.968	0.251	0.000
CAD	CA9	163	-0.027	0.013	0.041	0.973	0.264	0.000
CAD	CACNB3	163	0.025	0.014	0.075	1.025	0.339	0.000
CAD	CALB2	163	-0.010	0.012	0.418	0.990	0.667	0.002
CAD	CALCA	163	0.007	0.013	0.582	1.007	0.763	0.000
CAD	CALCOCO2	163	0.027	0.015	0.071	1.027	0.327	0.000
CAD	CCN3	163	-0.046	0.013	0.000	0.955	0.011	0.000
CAD	CCND2	163	-0.012	0.011	0.300	0.988	0.572	0.123
CAD	CD14	163	0.016	0.018	0.363	1.016	0.626	0.000
CAD	CD164L2	163	-0.010	0.011	0.367	0.990	0.630	0.132
CAD	CD46	163	-0.008	0.016	0.593	0.992	0.772	0.000
CAD	CD59	163	0.007	0.013	0.613	1.007	0.777	0.000
CAD	CEP170	163	0.021	0.013	0.124	1.021	0.396	0.000
CAD	CERT	163	0.014	0.013	0.261	1.014	0.535	0.000
CAD	CES2	163	-0.011	0.016	0.500	0.989	0.709	0.000
CAD	CETN3	163	0.010	0.013	0.466	1.010	0.693	0.000
CAD	CFHR2	163	0.012	0.008	0.143	1.012	0.399	0.000
CAD	CFHR4	163	0.018	0.012	0.122	1.018	0.396	0.000
CAD	CHMP1A	163	0.013	0.013	0.312	1.013	0.587	0.000
CAD	CLIC5	163	0.013	0.013	0.323	1.013	0.592	0.000

ST17; MR causal estimates for CAD and stroke on BP-associated proteins.

All estimates are from inverse variance weighted method (IVs >1) or Wald-ratio method (IV = 1). CAD, coronary artery disease; AS, all strokes; AIS, ischemic stroke; SVS, small vessel stroke; CES, cardioembolic stroke; LAS, large artery stroke

Exposure	Outocme	nsnp	Beta	SE	P-value	Odds ratio	FDR-corrected P-value	FDR-corrected Cochran's Q P-value
CAD	CLMP	163	-0.012	0.014	0.393	0.988	0.645	0.000
CAD	COL1A1	163	-0.024	0.016	0.143	0.977	0.399	0.000
CAD	COMP	163	-0.057	0.016	0.000	0.944	0.010	0.000
CAD	COMT	163	0.009	0.013	0.492	1.009	0.703	0.000
CAD	CPTP	163	-0.008	0.013	0.532	0.992	0.725	0.000
CAD	CPXM1	163	0.014	0.018	0.426	1.015	0.667	0.000
CAD	CTF1	163	-0.004	0.013	0.771	0.996	0.864	0.000
CAD	CTSO	163	0.029	0.019	0.134	1.030	0.399	0.000
CAD	DAG1	163	0.007	0.017	0.673	1.007	0.821	0.000
CAD	DARS1	163	0.009	0.013	0.489	1.009	0.703	0.000
CAD	DBN1	163	0.001	0.013	0.946	1.001	0.971	0.000
CAD	DDHD2	163	0.010	0.013	0.436	1.011	0.671	0.000
CAD	DENR	163	-0.004	0.013	0.734	0.996	0.853	0.000
CAD	DNAJC9	163	0.004	0.013	0.725	1.004	0.851	0.000
CAD	DNER	163	-0.001	0.018	0.942	0.999	0.971	0.000
CAD	DOK2	163	0.017	0.015	0.252	1.017	0.525	0.000
CAD	DPEP1	163	-0.003	0.012	0.770	0.997	0.864	0.000
CAD	DPP4	163	-0.009	0.015	0.532	0.991	0.725	0.000
CAD	DTX3	163	-0.015	0.013	0.246	0.985	0.525	0.000
CAD	DUSP13	163	0.018	0.015	0.232	1.018	0.516	0.000
CAD	DUSP29	163	0.002	0.012	0.853	1.002	0.906	0.000
CAD	EDN1	163	-0.026	0.013	0.043	0.974	0.264	0.000
CAD	EFEMP1	163	-0.038	0.014	0.006	0.962	0.094	0.000
CAD	EFNA1	163	-0.010	0.015	0.480	0.990	0.702	0.000
CAD	EIF4G3	163	0.023	0.013	0.066	1.024	0.319	0.000
CAD	ELOA	163	0.026	0.015	0.093	1.026	0.381	0.000
CAD	ENPEP	163	-0.005	0.011	0.667	0.995	0.818	0.398
CAD	EPHA2	163	-0.033	0.016	0.046	0.968	0.264	0.000
CAD	EPO	163	0.025	0.015	0.106	1.025	0.396	0.000
CAD	ERI1	163	0.003	0.012	0.780	1.003	0.866	0.001
CAD	ERP29	163	0.020	0.014	0.136	1.020	0.399	0.000
CAD	ESAM	163	-0.027	0.019	0.155	0.973	0.425	0.000
CAD	F12	163	0.018	0.010	0.077	1.018	0.341	0.000
CAD	F13B	163	0.002	0.015	0.898	1.002	0.943	0.000
CAD	FADD	163	0.005	0.014	0.740	1.005	0.854	0.000
CAD	FDX1	163	0.003	0.012	0.817	1.003	0.886	0.013
CAD	FES	163	-0.012	0.021	0.558	0.988	0.751	0.000
CAD	FGF12	163	-0.017	0.011	0.110	0.983	0.396	0.684
CAD	FGF2	163	0.032	0.014	0.026	1.032	0.227	0.000
CAD	FGF20	163	-0.015	0.011	0.175	0.985	0.443	0.453
CAD	FGF21	163	0.023	0.014	0.107	1.024	0.396	0.000
CAD	FGF23	163	0.017	0.016	0.282	1.018	0.554	0.000
CAD	FGF5	163	0.037	0.072	0.604	1.038	0.776	0.000
CAD	FKBP7	163	-0.005	0.013	0.718	0.995	0.850	0.001
CAD	FN1	163	-0.060	0.052	0.243	0.942	0.525	0.000
CAD	FOXJ3	163	-0.001	0.013	0.944	0.999	0.971	0.000
CAD	FOXO3	163	-0.001	0.012	0.949	0.999	0.971	0.006
CAD	FUCA1	163	0.000	0.009	0.987	1.000	0.987	0.000
CAD	FURIN	163	0.046	0.023	0.048	1.047	0.273	0.000
CAD	GCHFR	163	0.019	0.013	0.149	1.019	0.413	0.000
CAD	GFER	163	0.013	0.013	0.327	1.013	0.594	0.000
CAD	GHR	163	0.006	0.017	0.730	1.006	0.853	0.000
CAD	GHRHR	163	0.005	0.011	0.644	1.005	0.804	0.859
CAD	GHRL	163	0.021	0.015	0.183	1.021	0.458	0.000
CAD	GIMAP7	163	-0.028	0.015	0.068	0.973	0.323	0.000
CAD	GIT1	163	0.029	0.015	0.046	1.029	0.264	0.000
CAD	GLO1	163	0.020	0.013	0.132	1.020	0.399	0.000

ST17; MR causal estimates for CAD and stroke on BP-associated proteins.

All estimates are from inverse variance weighted method (IVs >1) or Wald-ratio method (IV = 1). CAD, coronary artery disease; AS, all strokes; AIS, ischemic stroke; SVS, small vessel stroke; CES, cardioembolic stroke; LAS, large artery stroke

Exposure	Outocme	nsnp	Beta	SE	P-value	Odds ratio	FDR-corrected P-value	FDR-corrected Cochran's Q P-value
CAD	GORASP2	163	0.016	0.014	0.258	1.016	0.534	0.000
CAD	GRP	163	0.006	0.019	0.740	1.006	0.854	0.000
CAD	GRPEL1	163	0.037	0.014	0.006	1.038	0.096	0.000
CAD	GSTM4	163	0.004	0.012	0.721	1.004	0.850	0.019
CAD	HADH	163	-0.013	0.011	0.210	0.987	0.490	0.638
CAD	HEXIM1	163	0.035	0.015	0.023	1.035	0.212	0.000
CAD	HHEX	163	0.043	0.017	0.012	1.044	0.135	0.000
CAD	HMOX2	163	0.033	0.013	0.012	1.033	0.135	0.000
CAD	HPGDS	163	0.005	0.012	0.705	1.005	0.844	0.000
CAD	HYAL1	163	0.048	0.022	0.033	1.049	0.251	0.000
CAD	ICAM1	163	0.008	0.033	0.820	1.008	0.886	0.000
CAD	ICAM2	163	-0.067	0.077	0.387	0.935	0.639	0.000
CAD	ICAM4	163	-0.046	0.037	0.217	0.956	0.492	0.000
CAD	IDUA	163	0.017	0.017	0.321	1.018	0.591	0.000
CAD	IFI30	163	0.055	0.019	0.003	1.057	0.063	0.000
CAD	IFIT3	163	0.015	0.013	0.251	1.015	0.525	0.000
CAD	IFNGR2	163	-0.009	0.018	0.617	0.991	0.777	0.000
CAD	IGFBP3	163	0.006	0.013	0.615	1.006	0.777	0.000
CAD	IL1RL1	163	-0.003	0.015	0.868	0.997	0.918	0.000
CAD	IMMT	163	0.028	0.014	0.038	1.029	0.253	0.000
CAD	IMPA1	163	0.001	0.013	0.962	1.001	0.973	0.000
CAD	ING1	163	0.000	0.013	0.987	1.000	0.987	0.001
CAD	ITGAL	163	0.029	0.019	0.124	1.029	0.396	0.000
CAD	ITIH1	163	-0.003	0.012	0.833	0.997	0.895	0.010
CAD	KIF22	163	0.028	0.015	0.053	1.029	0.273	0.000
CAD	KIFBP	163	0.018	0.013	0.164	1.018	0.431	0.000
CAD	LACRT	163	0.010	0.013	0.450	1.010	0.686	0.000
CAD	LAYN	163	-0.010	0.013	0.455	0.990	0.687	0.000
CAD	LMOD1	163	-0.062	0.026	0.017	0.940	0.173	0.000
CAD	LMOD1	163	-0.062	0.024	0.010	0.939	0.132	0.000
CAD	LMOD1	163	-0.065	0.026	0.013	0.937	0.136	0.000
CAD	LMOD1	163	-0.052	0.024	0.033	0.949	0.251	0.000
CAD	LRIG1	163	-0.004	0.018	0.839	0.996	0.897	0.000
CAD	LYAR	163	-0.007	0.012	0.579	0.993	0.761	0.005
CAD	M6PR	163	0.016	0.012	0.201	1.016	0.477	0.002
CAD	MANEAL	163	-0.003	0.013	0.796	0.997	0.874	0.000
CAD	MANSC4	163	-0.014	0.016	0.378	0.986	0.632	0.000
CAD	MAP4K5	163	0.010	0.014	0.464	1.010	0.693	0.000
CAD	MDH1	163	0.007	0.014	0.599	1.007	0.776	0.000
CAD	MEGF9	163	0.013	0.020	0.521	1.013	0.723	0.000
CAD	MFGE8	163	0.044	0.024	0.062	1.045	0.306	0.000
CAD	MPHOSPH8	163	0.017	0.014	0.197	1.018	0.477	0.000
CAD	MPI	163	0.016	0.013	0.216	1.016	0.492	0.000
CAD	MPIG6B	163	0.022	0.015	0.139	1.023	0.399	0.000
CAD	MSRA	163	0.009	0.014	0.504	1.009	0.709	0.000
CAD	MST1	163	0.043	0.033	0.187	1.044	0.464	0.000
CAD	MVK	163	0.060	0.015	0.000	1.061	0.004	0.000
CAD	MXRA8	163	-0.088	0.022	0.000	0.916	0.004	0.000
CAD	NADK	163	-0.004	0.014	0.779	0.996	0.866	0.000
CAD	NAGA	163	-0.021	0.017	0.199	0.979	0.477	0.000
CAD	NBN	163	0.012	0.014	0.408	1.012	0.658	0.000
CAD	NFE2	163	0.032	0.014	0.026	1.032	0.227	0.000
CAD	NFU1	163	0.022	0.014	0.113	1.022	0.396	0.000
CAD	NGF	163	0.012	0.012	0.316	1.012	0.587	0.016
CAD	NGFR	163	-0.003	0.011	0.790	0.997	0.873	0.649
CAD	NOMO1	163	-0.017	0.018	0.339	0.983	0.606	0.000
CAD	NOS3	163	0.045	0.019	0.017	1.046	0.173	0.000

ST17; MR causal estimates for CAD and stroke on BP-associated proteins.

All estimates are from inverse variance weighted method (IVs >1) or Wald-ratio method (IV = 1). CAD, coronary artery disease; AS, all strokes; AIS, ischemic stroke; SVS, small vessel stroke; CES, cardioembolic stroke; LAS, large artery stroke

Exposure	Outocme	nsnp	Beta	SE	P-value	Odds ratio	FDR-corrected P-value	FDR-corrected Cochran's Q P-value
CAD	NOTCH3	163	-0.082	0.017	0.000	0.921	0.000	0.000
CAD	NPPB	163	0.033	0.012	0.006	1.033	0.094	0.003
CAD	NTRK3	163	-0.076	0.018	0.000	0.927	0.002	0.000
CAD	NUCB2	163	0.029	0.017	0.081	1.029	0.355	0.000
CAD	NUDT5	163	0.020	0.016	0.203	1.021	0.479	0.000
CAD	NUMB	163	0.026	0.016	0.091	1.027	0.381	0.000
CAD	OGA	163	0.027	0.014	0.052	1.028	0.273	0.000
CAD	OPLAH	163	0.026	0.015	0.070	1.027	0.327	0.000
CAD	OTUD6B	163	0.013	0.013	0.340	1.013	0.606	0.000
CAD	PAM	163	-0.030	0.014	0.036	0.970	0.253	0.000
CAD	PAMR1	163	0.073	0.019	0.000	1.076	0.006	0.000
CAD	PARP1	163	0.019	0.016	0.217	1.020	0.492	0.000
CAD	PCBP2	163	0.014	0.015	0.374	1.014	0.632	0.000
CAD	PCOLCE	163	0.096	0.020	0.000	1.101	0.000	0.000
CAD	PCSK7	163	0.012	0.016	0.421	1.013	0.667	0.000
CAD	PDE5A	163	0.012	0.014	0.381	1.013	0.634	0.000
CAD	PDGFRA	163	-0.017	0.014	0.243	0.984	0.525	0.000
CAD	PDIA3	163	-0.018	0.013	0.161	0.982	0.431	0.000
CAD	PECAM1	163	-0.054	0.059	0.362	0.948	0.626	0.000
CAD	PFKFB2	163	0.015	0.015	0.315	1.015	0.587	0.000
CAD	PGF	163	0.021	0.023	0.347	1.022	0.614	0.000
CAD	PHLDB1	163	0.005	0.012	0.664	1.005	0.818	0.000
CAD	PKD1	163	-0.038	0.016	0.018	0.962	0.174	0.000
CAD	PLA2G1B	163	-0.007	0.018	0.691	0.993	0.832	0.000
CAD	PLXDC2	163	-0.050	0.023	0.034	0.952	0.251	0.000
CAD	PMS1	163	0.001	0.012	0.908	1.001	0.950	0.042
CAD	PMVK	163	0.019	0.013	0.126	1.019	0.397	0.001
CAD	PPP1R14D	163	0.012	0.013	0.357	1.012	0.624	0.000
CAD	PRDX1	163	-0.023	0.016	0.142	0.977	0.399	0.000
CAD	PRG2	163	0.025	0.016	0.121	1.025	0.396	0.000
CAD	PRKAB1	163	0.009	0.012	0.475	1.009	0.700	0.001
CAD	PROCR	163	-0.066	0.053	0.218	0.936	0.492	0.000
CAD	PRSS53	163	-0.018	0.014	0.192	0.982	0.473	0.000
CAD	PRTFDC1	163	0.011	0.013	0.403	1.011	0.655	0.000
CAD	PSMD5	163	-0.006	0.011	0.560	0.994	0.751	0.557
CAD	PSRC1	163	0.022	0.016	0.174	1.022	0.443	0.000
CAD	PTPRF	163	-0.036	0.021	0.094	0.965	0.381	0.000
CAD	PTRHD1	163	0.009	0.014	0.487	1.010	0.703	0.000
CAD	PYDC1	163	0.001	0.011	0.959	1.001	0.973	0.000
CAD	QPCT	163	-0.006	0.016	0.707	0.994	0.844	0.000
CAD	RABEPK	163	0.009	0.011	0.428	1.009	0.667	0.138
CAD	RANBP1	163	0.007	0.012	0.574	1.007	0.761	0.000
CAD	RARRES1	163	0.004	0.014	0.796	1.004	0.874	0.000
CAD	RARRES2	163	0.044	0.016	0.005	1.045	0.094	0.000
CAD	RELT	163	0.017	0.015	0.250	1.017	0.525	0.000
CAD	RSPO3	163	-0.014	0.014	0.336	0.986	0.606	0.000
CAD	SCARA5	163	-0.009	0.018	0.602	0.991	0.776	0.000
CAD	SDC1	163	0.003	0.019	0.870	1.003	0.918	0.000
CAD	SDCCAG8	163	0.028	0.013	0.038	1.028	0.253	0.000
CAD	SDHB	163	0.021	0.013	0.110	1.021	0.396	0.000
CAD	SELENOP	163	-0.017	0.012	0.163	0.984	0.431	0.007
CAD	SEMA6C	163	0.003	0.011	0.803	1.003	0.877	0.910
CAD	SERPING1	163	0.033	0.024	0.171	1.033	0.441	0.000
CAD	SERPINI1	163	-0.021	0.013	0.109	0.979	0.396	0.000
CAD	SH2B3	163	0.020	0.013	0.132	1.020	0.399	0.000
CAD	SHMT1	163	0.020	0.013	0.110	1.021	0.396	0.000
CAD	SIL1	163	-0.016	0.021	0.456	0.985	0.687	0.000

ST17; MR causal estimates for CAD and stroke on BP-associated proteins.

All estimates are from inverse variance weighted method (IVs >1) or Wald-ratio method (IV = 1). CAD, coronary artery disease; AS, all strokes; AIS, ischemic stroke; SVS, small vessel stroke; CES, cardioembolic stroke; LAS, large artery stroke

Exposure	Outocme	nsnp	Beta	SE	P-value	Odds ratio	FDR-corrected P-value	FDR-corrected Cochran's Q P-value
CAD	SLC16A1	163	0.023	0.014	0.099	1.023	0.387	0.000
CAD	SLC39A14	163	-0.012	0.019	0.530	0.988	0.725	0.000
CAD	SLC9A3R2	163	0.001	0.016	0.958	1.001	0.973	0.000
CAD	SMOC2	163	-0.013	0.016	0.426	0.987	0.667	0.000
CAD	SMTN	163	0.018	0.012	0.134	1.018	0.399	0.002
CAD	SOST	163	0.011	0.016	0.505	1.011	0.709	0.000
CAD	SPINK8	163	0.028	0.043	0.523	1.028	0.723	0.000
CAD	SPRED2	163	-0.009	0.012	0.435	0.991	0.671	0.026
CAD	SPRING1	163	-0.004	0.012	0.748	0.996	0.855	0.065
CAD	STC1	163	0.010	0.017	0.565	1.010	0.754	0.000
CAD	STX4	163	0.017	0.014	0.246	1.017	0.525	0.000
CAD	SYAP1	163	0.029	0.015	0.052	1.029	0.273	0.000
CAD	TARBP2	163	0.021	0.013	0.123	1.021	0.396	0.000
CAD	TBC1D17	163	0.007	0.011	0.546	1.007	0.739	0.026
CAD	TBC1D23	163	0.021	0.014	0.121	1.021	0.396	0.000
CAD	TEK	163	-0.026	0.017	0.124	0.974	0.396	0.000
CAD	TGFB2	163	-0.019	0.011	0.094	0.981	0.381	0.120
CAD	TIE1	163	0.004	0.022	0.840	1.004	0.897	0.000
CAD	TJAP1	163	0.027	0.014	0.052	1.028	0.273	0.000
CAD	TMEM106A	163	0.009	0.015	0.518	1.009	0.723	0.000
CAD	TNFRSF12A	163	0.028	0.017	0.086	1.029	0.370	0.000
CAD	TNFRSF13B	163	0.032	0.016	0.045	1.033	0.264	0.000
CAD	TNFRSF17	163	-0.006	0.015	0.667	0.994	0.818	0.000
CAD	TNFSF12	163	-0.009	0.021	0.684	0.991	0.831	0.000
CAD	TNFSF13B	163	0.026	0.016	0.096	1.027	0.384	0.000
CAD	TP53	163	0.004	0.013	0.769	1.004	0.864	0.001
CAD	TP53BP1	163	-0.014	0.013	0.296	0.986	0.570	0.000
CAD	TP53I3	163	0.007	0.014	0.607	1.007	0.776	0.000
CAD	TP53INP1	163	-0.011	0.011	0.281	0.989	0.554	0.979
CAD	TWF2	163	0.013	0.014	0.353	1.013	0.621	0.000
CAD	UBE2L6	163	0.024	0.015	0.119	1.024	0.396	0.000
CAD	UMOD	163	-0.014	0.013	0.288	0.986	0.558	0.000
CAD	UXS1	163	-0.021	0.016	0.196	0.979	0.477	0.000
CAD	VAT1	163	-0.020	0.020	0.313	0.980	0.587	0.000
CAD	VSIG2	163	0.004	0.017	0.806	1.004	0.877	0.000
CAD	WARS	163	0.020	0.018	0.268	1.020	0.546	0.000
CAD	WASHC3	163	0.021	0.014	0.128	1.021	0.399	0.000
CAD	WNT9A	163	-0.039	0.013	0.003	0.962	0.066	0.000
CAD	WWP2	163	0.015	0.014	0.287	1.015	0.558	0.000
CAD	YAP1	163	-0.031	0.012	0.011	0.969	0.135	0.000
CAD	YOD1	163	0.012	0.013	0.372	1.012	0.632	0.000
CAD	ZBTB17	163	0.005	0.013	0.690	1.005	0.832	0.000
CAD	ZFYVE19	163	0.018	0.013	0.163	1.019	0.431	0.000
CES	AAMDC	8	0.042	0.015	0.006	1.043	0.775	0.872
CES	ABO	8	0.238	0.489	0.626	1.269	0.895	0.000
CES	ACADM	8	-0.010	0.018	0.575	0.990	0.878	0.761
CES	ACOX1	8	-0.009	0.018	0.612	0.991	0.893	0.977
CES	ACRBP	8	0.016	0.027	0.537	1.017	0.853	0.106
CES	ADAM23	8	-0.003	0.018	0.857	0.997	0.948	0.343
CES	ADAMTS1	8	0.026	0.020	0.191	1.026	0.775	0.442
CES	ADAMTS13	8	0.065	0.093	0.481	1.068	0.822	0.000
CES	ADAMTS15	8	0.019	0.043	0.658	1.019	0.917	0.000
CES	ADAMTS16	8	-0.014	0.021	0.517	0.986	0.853	0.246
CES	ADAMTS4	8	-0.045	0.018	0.014	0.956	0.775	0.503
CES	ADAMTS8	8	-0.001	0.027	0.962	0.999	0.974	0.041
CES	ADAMTSL5	8	-0.004	0.018	0.838	0.996	0.941	0.487
CES	ADCYAP1R1	8	-0.015	0.018	0.386	0.985	0.808	0.943

ST17; MR causal estimates for CAD and stroke on BP-associated proteins.

All estimates are from inverse variance weighted method (IVs >1) or Wald-ratio method (IV = 1). CAD, coronary artery disease; AS, all strokes; AIS, ischemic stroke; SVS, small vessel stroke; CES, cardioembolic stroke; LAS, large artery stroke

Exposure	Outocme	nsnp	Beta	SE	P-value	Odds ratio	FDR-corrected P-value	FDR-corrected Cochran's Q P-value
CES	ADM	8	0.033	0.016	0.044	1.034	0.775	0.518
CES	AGRP	8	0.002	0.025	0.939	1.002	0.964	0.108
CES	AMFR	8	0.020	0.018	0.261	1.020	0.785	0.951
CES	AMOTL2	8	0.019	0.017	0.276	1.019	0.788	0.701
CES	ANKMY2	8	0.018	0.025	0.469	1.018	0.820	0.181
CES	AOC1	8	0.007	0.020	0.726	1.007	0.917	0.413
CES	AOC3	8	0.009	0.022	0.698	1.009	0.917	0.151
CES	APOA1	8	0.024	0.016	0.147	1.024	0.775	0.607
CES	APOA2	8	0.040	0.019	0.034	1.040	0.775	0.443
CES	APOBR	8	-0.028	0.023	0.230	0.973	0.775	0.004
CES	APOC1	8	0.023	0.016	0.152	1.023	0.775	0.943
CES	ARHGEF12	8	0.002	0.021	0.915	1.002	0.964	0.321
CES	ARSB	8	0.024	0.017	0.163	1.024	0.775	0.546
CES	ASPN	8	0.008	0.022	0.706	1.008	0.917	0.259
CES	ATXN2L	8	0.011	0.017	0.526	1.011	0.853	0.613
CES	AXL	8	0.031	0.042	0.453	1.032	0.820	0.000
CES	B4GAT1	8	0.016	0.021	0.446	1.016	0.820	0.266
CES	BAG4	8	0.013	0.017	0.439	1.014	0.820	0.673
CES	BCAM	8	0.151	0.175	0.390	1.163	0.808	0.000
CES	BMP6	8	-0.030	0.060	0.620	0.971	0.895	0.000
CES	BNIP3L	8	0.017	0.017	0.315	1.018	0.792	0.813
CES	BRAP	8	0.028	0.024	0.258	1.028	0.785	0.175
CES	BRSK2	8	0.030	0.017	0.079	1.030	0.775	0.525
CES	CA12	8	0.011	0.026	0.674	1.011	0.917	0.108
CES	CA9	8	0.025	0.028	0.373	1.026	0.808	0.042
CES	CACNB3	8	0.024	0.019	0.219	1.024	0.775	0.449
CES	CALB2	8	0.032	0.018	0.082	1.032	0.775	0.518
CES	CALCA	8	0.018	0.016	0.250	1.018	0.785	0.449
CES	CALCOCO2	8	0.007	0.029	0.798	1.007	0.932	0.039
CES	CCN3	8	0.016	0.015	0.314	1.016	0.792	0.943
CES	CCND2	8	0.005	0.019	0.778	1.005	0.930	0.508
CES	CD14	8	-0.025	0.040	0.531	0.975	0.853	0.000
CES	CD164L2	8	0.019	0.017	0.268	1.019	0.788	0.830
CES	CD46	8	0.028	0.021	0.171	1.029	0.775	0.348
CES	CD59	8	0.031	0.025	0.205	1.032	0.775	0.099
CES	CEP170	8	0.017	0.022	0.446	1.017	0.820	0.307
CES	CERT	8	0.024	0.024	0.327	1.024	0.797	0.193
CES	CES2	8	-0.005	0.030	0.863	0.995	0.948	0.028
CES	CETN3	8	0.032	0.023	0.163	1.032	0.775	0.246
CES	CFHR2	8	0.020	0.011	0.056	1.020	0.775	0.597
CES	CFHR4	8	0.024	0.018	0.179	1.024	0.775	0.086
CES	CHMP1A	8	0.033	0.026	0.192	1.034	0.775	0.151
CES	CLIC5	8	0.037	0.018	0.034	1.038	0.775	0.857
CES	CLMP	8	0.031	0.016	0.053	1.031	0.775	0.518
CES	COL1A1	8	-0.001	0.033	0.985	0.999	0.990	0.005
CES	COMP	8	0.008	0.016	0.612	1.008	0.893	0.627
CES	COMT	8	0.029	0.024	0.218	1.030	0.775	0.168
CES	CPTP	8	0.023	0.019	0.229	1.023	0.775	0.509
CES	CPXM1	8	-0.007	0.022	0.763	0.993	0.925	0.246
CES	CTF1	8	0.035	0.023	0.121	1.036	0.775	0.275
CES	CTSO	8	0.031	0.017	0.062	1.032	0.775	0.972
CES	DAG1	8	0.027	0.026	0.311	1.027	0.792	0.108
CES	DARS1	8	0.027	0.025	0.278	1.027	0.788	0.179
CES	DBN1	8	0.020	0.024	0.386	1.021	0.808	0.228
CES	DDHD2	8	0.026	0.023	0.272	1.026	0.788	0.205
CES	DENR	8	0.042	0.020	0.039	1.043	0.775	0.409
CES	DNAJC9	8	0.024	0.017	0.150	1.025	0.775	0.804

ST17; MR causal estimates for CAD and stroke on BP-associated proteins.

All estimates are from inverse variance weighted method (IVs >1) or Wald-ratio method (IV = 1). CAD, coronary artery disease; AS, all strokes; AIS, ischemic stroke; SVS, small vessel stroke; CES, cardioembolic stroke; LAS, large artery stroke

Exposure	Outocme	nsnp	Beta	SE	P-value	Odds ratio	FDR-corrected P-value	FDR-corrected Cochran's Q P-value
CES	DNER	8	-0.012	0.016	0.466	0.988	0.820	0.617
CES	DOK2	8	0.036	0.023	0.113	1.037	0.775	0.246
CES	DPEP1	8	0.035	0.038	0.357	1.036	0.808	0.000
CES	DPP4	8	-0.012	0.016	0.468	0.988	0.820	0.727
CES	DTX3	8	0.032	0.022	0.152	1.032	0.775	0.181
CES	DUSP13	8	-0.006	0.019	0.737	0.994	0.917	0.443
CES	DUSP29	8	-0.007	0.017	0.679	0.993	0.917	0.752
CES	EDN1	8	-0.002	0.020	0.906	0.998	0.961	0.348
CES	EFEMP1	8	0.014	0.016	0.355	1.014	0.808	0.607
CES	EFNA1	8	0.033	0.017	0.048	1.033	0.775	0.997
CES	EIF4G3	8	0.006	0.019	0.735	1.006	0.917	0.451
CES	ELOA	8	0.014	0.017	0.392	1.014	0.808	0.752
CES	ENPEP	8	0.004	0.019	0.842	1.004	0.941	0.518
CES	EPHA2	8	0.016	0.017	0.323	1.016	0.795	0.702
CES	EPO	8	0.005	0.023	0.824	1.005	0.939	0.246
CES	ERI1	8	0.013	0.017	0.443	1.013	0.820	0.627
CES	ERP29	8	0.024	0.021	0.256	1.024	0.785	0.348
CES	ESAM	8	0.034	0.017	0.051	1.035	0.775	0.518
CES	F12	8	0.015	0.020	0.458	1.015	0.820	0.043
CES	F13B	8	-0.026	0.016	0.104	0.974	0.775	0.997
CES	FADD	8	0.027	0.021	0.202	1.027	0.775	0.348
CES	FDX1	8	0.044	0.018	0.013	1.045	0.775	0.577
CES	FES	8	0.003	0.018	0.881	1.003	0.952	0.609
CES	FGF12	8	0.008	0.021	0.717	1.008	0.917	0.379
CES	FGF2	8	0.002	0.026	0.950	1.002	0.967	0.085
CES	FGF20	8	0.020	0.023	0.389	1.020	0.808	0.294
CES	FGF21	8	0.026	0.019	0.172	1.027	0.775	0.426
CES	FGF23	8	0.064	0.051	0.207	1.067	0.775	0.000
CES	FGF5	8	-0.008	0.020	0.680	0.992	0.917	0.266
CES	FKBP7	8	0.026	0.018	0.139	1.027	0.775	0.761
CES	FN1	8	-0.034	0.024	0.158	0.967	0.775	0.167
CES	FOXJ3	8	0.013	0.019	0.506	1.013	0.851	0.450
CES	FOXO3	8	0.036	0.018	0.042	1.036	0.775	0.782
CES	FUCA1	8	-0.001	0.009	0.932	0.999	0.964	0.614
CES	FURIN	8	0.013	0.017	0.447	1.013	0.820	0.617
CES	GCHFR	8	0.018	0.017	0.294	1.018	0.792	0.661
CES	GFER	8	0.013	0.026	0.604	1.014	0.893	0.128
CES	GHR	8	0.005	0.028	0.872	1.005	0.951	0.016
CES	GHRHR	8	0.010	0.018	0.587	1.010	0.890	0.857
CES	GHRL	8	-0.035	0.034	0.307	0.965	0.792	0.001
CES	GIMAP7	8	0.033	0.017	0.046	1.034	0.775	0.525
CES	GIT1	8	0.016	0.021	0.446	1.016	0.820	0.348
CES	GLO1	8	0.010	0.017	0.545	1.010	0.859	0.645
CES	GORASP2	8	0.031	0.021	0.144	1.032	0.775	0.321
CES	GRP	8	0.012	0.024	0.626	1.012	0.895	0.167
CES	GRPEL1	8	0.015	0.021	0.460	1.015	0.820	0.352
CES	GSTM4	8	0.007	0.024	0.780	1.007	0.930	0.214
CES	HADH	8	-0.006	0.018	0.749	0.994	0.918	0.645
CES	HEXIM1	8	0.007	0.021	0.726	1.007	0.917	0.307
CES	HHEX	8	0.028	0.021	0.178	1.028	0.775	0.350
CES	HMOX2	8	0.014	0.023	0.529	1.015	0.853	0.246
CES	HPGDS	8	0.040	0.026	0.126	1.041	0.775	0.100
CES	HYAL1	8	-0.033	0.022	0.141	0.968	0.775	0.259
CES	ICAM1	8	-0.083	0.096	0.385	0.920	0.808	0.000
CES	ICAM2	8	-0.142	0.246	0.564	0.867	0.866	0.000
CES	ICAM4	8	-0.081	0.086	0.346	0.922	0.808	0.000
CES	IDUA	8	0.010	0.016	0.525	1.010	0.853	0.887

ST17; MR causal estimates for CAD and stroke on BP-associated proteins.

All estimates are from inverse variance weighted method (IVs >1) or Wald-ratio method (IV = 1). CAD, coronary artery disease; AS, all strokes; AIS, ischemic stroke; SVS, small vessel stroke; CES, cardioembolic stroke; LAS, large artery stroke

Exposure	Outocme	nsnp	Beta	SE	P-value	Odds ratio	FDR-corrected P-value	FDR-corrected Cochran's Q P-value
CES	IFI30	8	0.012	0.029	0.689	1.012	0.917	0.016
CES	IFIT3	8	0.028	0.021	0.185	1.029	0.775	0.337
CES	IFNGR2	8	0.003	0.022	0.879	1.003	0.952	0.017
CES	IGFBP3	8	-0.004	0.018	0.812	0.996	0.939	0.421
CES	IL1RL1	8	-0.023	0.030	0.453	0.978	0.820	0.000
CES	IMMT	8	-0.010	0.017	0.564	0.990	0.866	0.963
CES	IMPA1	8	0.035	0.017	0.044	1.036	0.775	0.577
CES	ING1	8	0.005	0.019	0.806	1.005	0.937	0.503
CES	ITGAL	8	0.003	0.017	0.866	1.003	0.948	0.813
CES	ITIH1	8	-0.002	0.017	0.917	0.998	0.964	0.702
CES	KIF22	8	0.026	0.019	0.177	1.026	0.775	0.477
CES	KIFBP	8	0.039	0.022	0.073	1.040	0.775	0.307
CES	LACRT	8	0.014	0.017	0.402	1.014	0.808	0.951
CES	LAYN	8	0.059	0.049	0.225	1.061	0.775	0.000
CES	LMOD1	8	0.014	0.016	0.359	1.014	0.808	0.627
CES	LMOD1	8	0.006	0.022	0.798	1.006	0.932	0.193
CES	LMOD1	8	0.010	0.015	0.536	1.010	0.853	0.728
CES	LMOD1	8	0.003	0.016	0.832	1.003	0.939	0.701
CES	LRIG1	8	0.004	0.021	0.856	1.004	0.948	0.134
CES	LYAR	8	-0.003	0.019	0.894	0.997	0.952	0.489
CES	M6PR	8	0.018	0.020	0.369	1.018	0.808	0.449
CES	MANEAL	8	0.007	0.018	0.695	1.007	0.917	0.828
CES	MANSC4	8	0.017	0.019	0.391	1.017	0.808	0.245
CES	MAP4K5	8	0.033	0.029	0.248	1.034	0.785	0.046
CES	MDH1	8	0.016	0.017	0.347	1.016	0.808	0.799
CES	MEGF9	8	0.000	0.030	0.991	1.000	0.991	0.018
CES	MFGE8	8	0.008	0.019	0.686	1.008	0.917	0.426
CES	MPHOSPH8	8	0.025	0.018	0.153	1.025	0.775	0.701
CES	MPI	8	0.032	0.022	0.148	1.033	0.775	0.252
CES	MPIG6B	8	0.027	0.022	0.223	1.027	0.775	0.294
CES	MSRA	8	0.033	0.021	0.110	1.034	0.775	0.348
CES	MST1	8	0.007	0.010	0.479	1.007	0.822	0.988
CES	MVK	8	0.014	0.024	0.552	1.014	0.859	0.193
CES	MXRA8	8	0.023	0.016	0.151	1.023	0.775	0.583
CES	NADK	8	-0.005	0.016	0.779	0.995	0.930	0.907
CES	NAGA	8	0.035	0.017	0.044	1.035	0.775	0.842
CES	NBN	8	-0.011	0.025	0.665	0.989	0.917	0.151
CES	NFE2	8	0.028	0.017	0.107	1.028	0.775	0.653
CES	NFU1	8	0.021	0.025	0.397	1.022	0.808	0.160
CES	NGF	8	-0.007	0.018	0.687	0.993	0.917	0.727
CES	NGFR	8	0.020	0.018	0.246	1.021	0.785	0.701
CES	NOMO1	8	-0.003	0.019	0.893	0.997	0.952	0.348
CES	NOS3	8	0.035	0.047	0.455	1.036	0.820	0.000
CES	NOTCH3	8	0.003	0.029	0.922	1.003	0.964	0.018
CES	NPPB	8	0.007	0.019	0.732	1.007	0.917	0.413
CES	NTRK3	8	0.004	0.017	0.827	1.004	0.939	0.754
CES	NUCB2	8	0.038	0.022	0.078	1.039	0.775	0.246
CES	NUDT5	8	0.024	0.017	0.169	1.024	0.775	0.577
CES	NUMB	8	0.022	0.024	0.367	1.022	0.808	0.205
CES	OGA	8	0.017	0.021	0.410	1.017	0.816	0.348
CES	OPLAH	8	0.030	0.025	0.222	1.030	0.775	0.175
CES	OTUD6B	8	0.033	0.025	0.186	1.033	0.775	0.168
CES	PAM	8	0.035	0.021	0.105	1.035	0.775	0.193
CES	PAMR1	8	0.003	0.021	0.886	1.003	0.952	0.224
CES	PARP1	8	0.009	0.017	0.598	1.009	0.890	0.701
CES	PCBP2	8	0.031	0.019	0.110	1.031	0.775	0.450
CES	PCOLCE	8	0.003	0.031	0.935	1.003	0.964	0.413

ST17; MR causal estimates for CAD and stroke on BP-associated proteins.

All estimates are from inverse variance weighted method (IVs >1) or Wald-ratio method (IV = 1). CAD, coronary artery disease; AS, all strokes; AIS, ischemic stroke; SVS, small vessel stroke; CES, cardioembolic stroke; LAS, large artery stroke

Exposure	Outocme	nsnp	Beta	SE	P-value	Odds ratio	FDR-corrected P-value	FDR-corrected Cochran's Q P-value
CES	PCSK7	8	-0.002	0.019	0.931	0.998	0.964	0.443
CES	PDE5A	8	0.026	0.025	0.305	1.026	0.792	0.151
CES	PDGFRA	8	-0.006	0.017	0.713	0.994	0.917	0.764
CES	PDIA3	8	0.005	0.018	0.792	1.005	0.932	0.813
CES	PECAM1	8	-0.068	0.143	0.633	0.934	0.895	0.000
CES	PFKFB2	8	0.024	0.024	0.321	1.025	0.795	0.167
CES	PGF	8	0.028	0.023	0.221	1.028	0.775	0.133
CES	PHLDB1	8	0.024	0.020	0.227	1.025	0.775	0.364
CES	PKD1	8	0.011	0.021	0.595	1.011	0.890	0.246
CES	PLA2G1B	8	0.005	0.021	0.827	1.005	0.939	0.307
CES	PLXDC2	8	-0.027	0.041	0.511	0.973	0.853	0.000
CES	PMS1	8	-0.013	0.021	0.550	0.987	0.859	0.348
CES	PMVK	8	0.022	0.027	0.400	1.023	0.808	0.110
CES	PPP1R14D	8	-0.004	0.018	0.832	0.996	0.939	0.521
CES	PRDX1	8	0.005	0.021	0.794	1.005	0.932	0.348
CES	PRG2	8	0.029	0.019	0.131	1.030	0.775	0.358
CES	PRKAB1	8	0.001	0.018	0.945	1.001	0.966	0.508
CES	PROCR	8	0.007	0.022	0.747	1.007	0.918	0.266
CES	PRSS53	8	-0.001	0.016	0.965	0.999	0.974	0.348
CES	PRTFDC1	8	0.029	0.027	0.283	1.029	0.790	0.100
CES	PSMD5	8	0.016	0.020	0.423	1.016	0.820	0.428
CES	PSRC1	8	0.009	0.023	0.688	1.009	0.917	0.241
CES	PTPRF	8	-0.010	0.024	0.667	0.990	0.917	0.151
CES	PTRHD1	8	0.025	0.022	0.240	1.026	0.785	0.318
CES	PYDC1	8	0.023	0.020	0.250	1.023	0.785	0.308
CES	QPCT	8	0.011	0.022	0.631	1.011	0.895	0.245
CES	RABEPK	8	0.020	0.023	0.390	1.020	0.808	0.255
CES	RANBP1	8	0.032	0.017	0.064	1.032	0.775	0.767
CES	RARRES1	8	-0.017	0.026	0.503	0.983	0.851	0.100
CES	RARRES2	8	0.031	0.017	0.063	1.032	0.775	0.597
CES	RELT	8	0.023	0.015	0.133	1.023	0.775	0.857
CES	RSPO3	8	-0.007	0.021	0.725	0.993	0.917	0.246
CES	SCARA5	8	0.075	0.074	0.315	1.077	0.792	0.000
CES	SDC1	8	-0.041	0.039	0.287	0.959	0.791	0.000
CES	SDCCAG8	8	0.016	0.020	0.429	1.016	0.820	0.348
CES	SDHB	8	0.007	0.022	0.747	1.007	0.918	0.266
CES	SELENOP	8	0.016	0.017	0.354	1.016	0.808	0.951
CES	SEMA6C	8	0.022	0.021	0.309	1.022	0.792	0.348
CES	SERPING1	8	0.019	0.017	0.279	1.019	0.788	0.525
CES	SERPINI1	8	0.031	0.043	0.481	1.031	0.822	0.000
CES	SH2B3	8	0.010	0.028	0.716	1.010	0.917	0.086
CES	SHMT1	8	0.011	0.020	0.593	1.011	0.890	0.217
CES	SIL1	8	0.006	0.019	0.762	1.006	0.925	0.477
CES	SLC16A1	8	0.031	0.018	0.078	1.031	0.775	0.701
AIS	AAMDC	26	0.072	0.034	0.033	1.075	0.347	0.002
AIS	ABO	26	0.312	0.458	0.496	1.366	0.663	0.000
AIS	ACADM	26	-0.023	0.032	0.474	0.978	0.663	0.089
AIS	ACOX1	26	0.067	0.026	0.011	1.069	0.347	0.817
AIS	ACRBP	26	0.052	0.045	0.247	1.053	0.539	0.000
AIS	ADAM23	26	-0.111	0.092	0.230	0.895	0.526	0.000
AIS	ADAMTS1	26	0.024	0.035	0.491	1.024	0.663	0.019
AIS	ADAMTS13	26	0.026	0.040	0.510	1.026	0.675	0.000
AIS	ADAMTS15	26	0.014	0.042	0.739	1.014	0.829	0.000
AIS	ADAMTS16	26	-0.037	0.036	0.296	0.963	0.575	0.001
AIS	ADAMTS4	26	0.054	0.039	0.159	1.056	0.430	0.001
AIS	ADAMTS8	26	-0.035	0.039	0.379	0.966	0.611	0.000
AIS	ADAMTSL5	26	-0.034	0.037	0.360	0.967	0.599	0.001

ST17; MR causal estimates for CAD and stroke on BP-associated proteins.

All estimates are from inverse variance weighted method (IVs >1) or Wald-ratio method (IV = 1). CAD, coronary artery disease; AS, all strokes; AIS, ischemic stroke; SVS, small vessel stroke; CES, cardioembolic stroke; LAS, large artery stroke

Exposure	Outocme	nsnp	Beta	SE	P-value	Odds ratio	FDR-corrected P-value	FDR-corrected Cochran's Q P-value
AIS	ADCYAP1R1	26	0.016	0.027	0.563	1.016	0.714	0.528
AIS	ADM	26	0.065	0.039	0.093	1.068	0.355	0.000
AIS	AGRP	26	0.087	0.044	0.049	1.091	0.347	0.000
AIS	AMFR	26	0.012	0.030	0.698	1.012	0.812	0.167
AIS	AMOTL2	26	0.054	0.030	0.070	1.056	0.355	0.155
AIS	ANKMY2	26	0.072	0.033	0.031	1.075	0.347	0.032
AIS	AOC1	26	-0.032	0.049	0.516	0.969	0.677	0.000
AIS	AOC3	26	-0.020	0.034	0.562	0.980	0.714	0.001
AIS	APOA1	26	0.010	0.033	0.764	1.010	0.839	0.010
AIS	APOA2	26	0.062	0.025	0.014	1.064	0.347	0.894
AIS	APOBR	26	-0.027	0.038	0.473	0.973	0.663	0.000
AIS	APOC1	26	-0.004	0.044	0.924	0.996	0.935	0.000
AIS	ARHGEF12	26	0.056	0.029	0.050	1.058	0.347	0.206
AIS	ARSB	26	0.066	0.045	0.147	1.068	0.423	0.000
AIS	ASPN	26	0.004	0.032	0.897	1.004	0.917	0.026
AIS	ATXN2L	26	0.040	0.040	0.309	1.041	0.579	0.000
AIS	AXL	26	0.054	0.058	0.351	1.055	0.592	0.000
AIS	B4GAT1	26	-0.045	0.038	0.247	0.956	0.539	0.000
AIS	BAG4	26	0.067	0.036	0.065	1.069	0.355	0.005
AIS	BCAM	26	0.082	0.086	0.340	1.085	0.592	0.000
AIS	BMP6	26	-0.073	0.083	0.380	0.930	0.611	0.000
AIS	BNIP3L	26	0.060	0.035	0.090	1.062	0.355	0.010
AIS	BRAP	26	0.088	0.034	0.010	1.092	0.347	0.017
AIS	BRSK2	26	0.015	0.024	0.549	1.015	0.713	0.603
AIS	CA12	26	-0.056	0.041	0.169	0.946	0.440	0.000
AIS	CA9	26	0.023	0.030	0.457	1.023	0.663	0.090
AIS	CACNB3	26	0.074	0.031	0.016	1.077	0.347	0.107
AIS	CALB2	26	-0.009	0.032	0.779	0.991	0.849	0.068
AIS	CALCA	26	0.047	0.045	0.297	1.048	0.575	0.000
AIS	CALCOCO2	26	0.049	0.050	0.330	1.050	0.592	0.000
AIS	CCN3	26	-0.066	0.037	0.069	0.936	0.355	0.000
AIS	CCND2	26	0.007	0.036	0.834	1.008	0.887	0.011
AIS	CD14	26	-0.098	0.050	0.049	0.906	0.347	0.000
AIS	CD164L2	26	-0.012	0.027	0.654	0.988	0.782	0.364
AIS	CD46	26	-0.021	0.055	0.709	0.980	0.812	0.000
AIS	CD59	26	0.024	0.026	0.343	1.025	0.592	0.283
AIS	CEP170	26	0.056	0.035	0.108	1.057	0.377	0.015
AIS	CERT	26	0.081	0.036	0.022	1.085	0.347	0.012
AIS	CES2	26	-0.044	0.041	0.287	0.957	0.575	0.000
AIS	CETN3	26	0.063	0.033	0.059	1.065	0.355	0.025
AIS	CFHR2	26	-0.020	0.024	0.421	0.981	0.624	0.000
AIS	CFHR4	26	0.023	0.034	0.495	1.024	0.663	0.000
AIS	CHMP1A	26	0.069	0.037	0.061	1.072	0.355	0.004
AIS	CLIC5	26	0.017	0.033	0.614	1.017	0.747	0.053
AIS	CLMP	26	-0.006	0.034	0.867	0.994	0.901	0.001
AIS	COL1A1	26	-0.095	0.053	0.071	0.909	0.355	0.000
AIS	COMP	26	-0.090	0.045	0.045	0.914	0.347	0.000
AIS	COMT	26	0.066	0.038	0.078	1.069	0.355	0.001
AIS	CPTP	26	-0.029	0.027	0.276	0.971	0.574	0.690
AIS	CPXM1	26	0.065	0.053	0.226	1.067	0.526	0.000
AIS	CTF1	26	0.022	0.030	0.475	1.022	0.663	0.167
AIS	CTSO	26	0.028	0.039	0.477	1.028	0.663	0.000
AIS	DAG1	26	0.067	0.058	0.246	1.070	0.539	0.000
AIS	DARS1	26	0.064	0.037	0.083	1.066	0.355	0.003
AIS	DBN1	26	0.047	0.030	0.124	1.048	0.389	0.128
AIS	DDHD2	26	0.072	0.032	0.025	1.075	0.347	0.034
AIS	DENR	26	0.062	0.042	0.138	1.064	0.406	0.000

ST17; MR causal estimates for CAD and stroke on BP-associated proteins.

All estimates are from inverse variance weighted method (IVs >1) or Wald-ratio method (IV = 1). CAD, coronary artery disease; AS, all strokes; AIS, ischemic stroke; SVS, small vessel stroke; CES, cardioembolic stroke; LAS, large artery stroke

Exposure	Outocme	nsnp	Beta	SE	P-value	Odds ratio	FDR-corrected P-value	FDR-corrected Cochran's Q P-value
AIS	DNAJC9	26	0.057	0.032	0.074	1.058	0.355	0.049
AIS	DNER	26	0.008	0.035	0.825	1.008	0.884	0.002
AIS	DOK2	26	0.069	0.041	0.088	1.072	0.355	0.000
AIS	DPEP1	26	0.037	0.044	0.408	1.037	0.621	0.000
AIS	DPP4	26	-0.067	0.031	0.029	0.935	0.347	0.054
AIS	DTX3	26	-0.010	0.031	0.748	0.990	0.829	0.016
AIS	DUSP13	26	0.015	0.026	0.574	1.015	0.714	0.454
AIS	DUSP29	26	-0.005	0.028	0.849	0.995	0.888	0.297
AIS	EDN1	26	-0.036	0.043	0.393	0.964	0.617	0.000
AIS	EFEMP1	26	-0.037	0.038	0.324	0.963	0.591	0.000
AIS	EFNA1	26	-0.003	0.038	0.928	0.997	0.935	0.000
AIS	EIF4G3	26	0.044	0.027	0.109	1.045	0.377	0.330
AIS	ELOA	26	0.048	0.047	0.305	1.050	0.579	0.000
AIS	ENPEP	26	-0.004	0.027	0.880	0.996	0.907	0.685
AIS	EPHA2	26	-0.052	0.053	0.324	0.949	0.591	0.000
AIS	EPO	26	-0.055	0.045	0.218	0.946	0.517	0.000
AIS	ERI1	26	0.014	0.038	0.709	1.014	0.812	0.001
AIS	ERP29	26	0.063	0.036	0.084	1.065	0.355	0.006
AIS	ESAM	26	-0.023	0.066	0.728	0.977	0.825	0.000
AIS	F12	26	0.004	0.021	0.846	1.004	0.888	0.164
AIS	F13B	26	-0.026	0.032	0.410	0.974	0.621	0.021
AIS	FADD	26	0.053	0.039	0.169	1.055	0.440	0.001
AIS	FDX1	26	0.045	0.031	0.150	1.046	0.423	0.128
AIS	FES	26	-0.056	0.100	0.577	0.945	0.714	0.000
AIS	FGF12	26	0.023	0.027	0.382	1.024	0.611	0.718
AIS	FGF2	26	0.067	0.042	0.116	1.069	0.383	0.000
AIS	FGF20	26	0.036	0.032	0.261	1.036	0.551	0.103
AIS	FGF21	26	-0.002	0.031	0.960	0.998	0.960	0.075
AIS	FGF23	26	0.065	0.062	0.298	1.067	0.575	0.000
AIS	FGF5	26	-0.061	0.041	0.137	0.941	0.406	0.000
AIS	FKBP7	26	0.035	0.032	0.282	1.035	0.575	0.076
AIS	FN1	26	-0.043	0.025	0.085	0.958	0.355	0.564
AIS	FOXJ3	26	0.053	0.036	0.134	1.055	0.406	0.010
AIS	FOXO3	26	0.044	0.035	0.211	1.045	0.508	0.012
AIS	FUCA1	26	-0.029	0.025	0.256	0.972	0.548	0.000
AIS	FURIN	26	0.138	0.147	0.348	1.148	0.592	0.000
AIS	GCHFR	26	0.057	0.036	0.116	1.059	0.383	0.002
AIS	GFER	26	0.073	0.036	0.046	1.075	0.347	0.004
AIS	GHR	26	0.030	0.032	0.349	1.031	0.592	0.007
AIS	GHRHR	26	-0.048	0.029	0.095	0.953	0.355	0.302
AIS	GHRL	26	0.010	0.027	0.705	1.010	0.812	0.252
AIS	GIMAP7	26	0.084	0.042	0.043	1.088	0.347	0.000
AIS	GIT1	26	0.067	0.029	0.020	1.069	0.347	0.200
AIS	GLO1	26	0.027	0.039	0.488	1.027	0.663	0.000
AIS	GORASP2	26	0.051	0.039	0.187	1.053	0.467	0.001
AIS	GRP	26	0.029	0.033	0.384	1.029	0.611	0.021
AIS	GRPEL1	26	0.083	0.043	0.051	1.087	0.347	0.000
AIS	GSTM4	26	0.010	0.031	0.749	1.010	0.829	0.089
AIS	HADH	26	-0.017	0.035	0.624	0.983	0.752	0.017
AIS	HEXIM1	26	0.075	0.038	0.049	1.078	0.347	0.001
AIS	HHEX	26	0.061	0.039	0.120	1.063	0.385	0.000
AIS	HMOX2	26	0.072	0.037	0.051	1.075	0.347	0.004
AIS	HPGDS	26	-0.062	0.044	0.159	0.940	0.430	0.000
AIS	HYAL1	26	-0.081	0.036	0.023	0.922	0.347	0.006
AIS	ICAM1	26	-0.100	0.191	0.603	0.905	0.740	0.000
AIS	ICAM2	26	-0.364	0.476	0.444	0.695	0.652	0.000
AIS	ICAM4	26	-0.178	0.215	0.409	0.837	0.621	0.000

ST17; MR causal estimates for CAD and stroke on BP-associated proteins.

All estimates are from inverse variance weighted method (IVs >1) or Wald-ratio method (IV = 1). CAD, coronary artery disease; AS, all strokes; AIS, ischemic stroke; SVS, small vessel stroke; CES, cardioembolic stroke; LAS, large artery stroke

Exposure	Outocme	nsnp	Beta	SE	P-value	Odds ratio	FDR-corrected P-value	FDR-corrected Cochran's Q P-value
AIS	IDUA	26	-0.018	0.047	0.702	0.982	0.812	0.000
AIS	IFI30	26	0.046	0.080	0.571	1.047	0.714	0.000
AIS	IFIT3	26	0.060	0.045	0.176	1.062	0.449	0.000
AIS	IFNGR2	26	-0.014	0.054	0.797	0.986	0.860	0.000
AIS	IGFBP3	26	-0.048	0.030	0.106	0.953	0.377	0.038
AIS	IL1RL1	26	-0.053	0.065	0.414	0.948	0.621	0.000
AIS	IMMT	26	0.046	0.036	0.210	1.047	0.508	0.002
AIS	IMPA1	26	0.058	0.032	0.064	1.060	0.355	0.077
LAS	AAMDC	3	0.013	0.027	0.635	1.013	0.978	0.813
LAS	ABO	3	1.529	2.396	0.523	4.614	0.962	0.000
LAS	ACADM	3	0.023	0.030	0.438	1.024	0.962	0.659
LAS	ACOX1	3	0.033	0.030	0.265	1.034	0.962	0.661
LAS	ACRBP	3	-0.060	0.048	0.206	0.941	0.962	0.176
LAS	ADAM23	3	-0.104	0.086	0.223	0.901	0.962	0.000
LAS	ADAMTS1	3	-0.019	0.030	0.537	0.982	0.962	0.566
LAS	ADAMTS13	3	0.082	0.088	0.352	1.085	0.962	0.000
LAS	ADAMTS15	3	0.066	0.050	0.184	1.069	0.962	0.144
LAS	ADAMTS16	3	-0.022	0.069	0.743	0.978	0.990	0.009
LAS	ADAMTS4	3	0.008	0.029	0.781	1.008	0.990	0.838
LAS	ADAMTS8	3	-0.024	0.034	0.492	0.977	0.962	0.390
LAS	ADAMTSL5	3	-0.014	0.029	0.624	0.986	0.978	0.782
LAS	ADCYAP1R1	3	0.015	0.030	0.614	1.015	0.974	0.727
LAS	ADM	3	0.103	0.056	0.064	1.109	0.962	0.051
LAS	AGRP	3	-0.068	0.060	0.257	0.934	0.962	0.045
LAS	AMFR	3	0.010	0.030	0.743	1.010	0.990	0.950
LAS	AMOTL2	3	-0.048	0.030	0.106	0.953	0.962	0.826
LAS	ANKMY2	3	0.026	0.037	0.485	1.026	0.962	0.394
LAS	AOC1	3	-0.062	0.064	0.330	0.940	0.962	0.044
LAS	AOC3	3	0.022	0.026	0.387	1.023	0.962	0.770
LAS	APOA1	3	-0.013	0.074	0.866	0.988	0.990	0.004
LAS	APOA2	3	0.019	0.029	0.502	1.019	0.962	0.679
LAS	APOBR	3	-0.110	0.092	0.234	0.896	0.962	0.000
LAS	APOC1	3	-0.028	0.094	0.767	0.973	0.990	0.000
LAS	ARHGEF12	3	-0.005	0.042	0.901	0.995	0.992	0.259
LAS	ARSB	3	-0.009	0.050	0.849	0.991	0.990	0.132
LAS	ASPN	3	0.064	0.055	0.240	1.066	0.962	0.086
LAS	ATXN2L	3	-0.012	0.030	0.675	0.988	0.990	0.770
LAS	AXL	3	0.184	0.124	0.136	1.203	0.962	0.000
LAS	B4GAT1	3	-0.069	0.031	0.029	0.934	0.962	0.467
LAS	BAG4	3	0.022	0.044	0.614	1.023	0.974	0.232
LAS	BCAM	3	0.644	0.709	0.363	1.904	0.962	0.000
LAS	BMP6	3	-0.073	0.104	0.483	0.930	0.962	0.000
LAS	BNIP3L	3	-0.012	0.054	0.825	0.988	0.990	0.110
LAS	BRAP	3	0.008	0.029	0.792	1.008	0.990	0.693
LAS	BRSK2	3	-0.008	0.028	0.763	0.992	0.990	0.810
LAS	CA12	3	0.037	0.037	0.314	1.038	0.962	0.346
LAS	CA9	3	0.039	0.120	0.749	1.039	0.990	0.000
LAS	CACNB3	3	0.007	0.029	0.811	1.007	0.990	0.565
LAS	CALB2	3	0.052	0.053	0.330	1.053	0.962	0.117
LAS	CALCA	3	0.029	0.024	0.231	1.029	0.962	0.557
LAS	CALCOCO2	3	-0.048	0.104	0.648	0.953	0.990	0.000
LAS	CCN3	3	-0.018	0.057	0.760	0.983	0.990	0.038
LAS	CCND2	3	0.011	0.030	0.710	1.011	0.990	0.926
LAS	CD14	3	-0.170	0.153	0.269	0.844	0.962	0.000
LAS	CD164L2	3	-0.012	0.044	0.786	0.988	0.990	0.225
LAS	CD46	3	0.005	0.055	0.925	1.005	0.992	0.094
LAS	CD59	3	0.070	0.060	0.238	1.073	0.962	0.037

ST17; MR causal estimates for CAD and stroke on BP-associated proteins.

All estimates are from inverse variance weighted method (IVs >1) or Wald-ratio method (IV = 1). CAD, coronary artery disease; AS, all strokes; AIS, ischemic stroke; SVS, small vessel stroke; CES, cardioembolic stroke; LAS, large artery stroke

Exposure	Outocme	nsnp	Beta	SE	P-value	Odds ratio	FDR-corrected P-value	FDR-corrected Cochran's Q P-value
LAS	CEP170	3	0.012	0.034	0.734	1.012	0.990	0.449
LAS	CERT	3	0.027	0.030	0.364	1.028	0.962	0.566
LAS	CES2	3	-0.110	0.085	0.198	0.896	0.962	0.001
LAS	CETN3	3	0.000	0.029	1.000	1.000	1.000	0.633
LAS	CFHR2	3	0.031	0.045	0.492	1.031	0.962	0.008
LAS	CFHR4	3	0.054	0.064	0.395	1.056	0.962	0.000
LAS	CHMP1A	3	-0.030	0.041	0.455	0.970	0.962	0.302
LAS	CLIC5	3	0.045	0.058	0.440	1.046	0.962	0.079
LAS	CLMP	3	0.059	0.026	0.025	1.060	0.962	0.669
LAS	COL1A1	3	-0.165	0.111	0.138	0.848	0.962	0.000
LAS	COMP	3	-0.018	0.029	0.523	0.982	0.962	0.517
LAS	COMT	3	-0.005	0.028	0.863	0.995	0.990	0.566
LAS	CPTP	3	0.036	0.030	0.238	1.036	0.962	0.565
LAS	CPXM1	3	-0.042	0.062	0.493	0.959	0.962	0.037
LAS	CTF1	3	-0.016	0.046	0.728	0.984	0.990	0.224
LAS	CTSO	3	0.029	0.047	0.541	1.029	0.962	0.165
LAS	DAG1	3	-0.036	0.052	0.489	0.964	0.962	0.120
LAS	DARS1	3	-0.016	0.029	0.586	0.984	0.974	0.826
LAS	DBN1	3	0.003	0.030	0.933	1.003	0.992	0.612
LAS	DDHD2	3	0.014	0.029	0.634	1.014	0.978	0.538
LAS	DENR	3	-0.007	0.038	0.846	0.993	0.990	0.369
LAS	DNAJC9	3	-0.010	0.053	0.853	0.990	0.990	0.110
LAS	DNER	3	-0.042	0.032	0.181	0.959	0.962	0.449
LAS	DOK2	3	0.005	0.031	0.880	1.005	0.990	0.512
LAS	DPEP1	3	0.122	0.106	0.248	1.130	0.962	0.000
LAS	DPP4	3	-0.002	0.028	0.934	0.998	0.992	0.643
LAS	DTX3	3	0.000	0.027	0.994	1.000	0.998	0.783
LAS	DUSP13	3	0.032	0.029	0.272	1.032	0.962	0.608
LAS	DUSP29	3	-0.017	0.030	0.559	0.983	0.962	0.566
LAS	EDN1	3	-0.044	0.078	0.571	0.957	0.970	0.004
LAS	EFEMP1	3	0.027	0.036	0.455	1.027	0.962	0.302
LAS	EFNA1	3	-0.019	0.039	0.633	0.982	0.978	0.293
LAS	EIF4G3	3	0.004	0.029	0.879	1.004	0.990	0.727
LAS	ELOA	3	0.010	0.050	0.850	1.010	0.990	0.129
LAS	ENPEP	3	-0.007	0.037	0.850	0.993	0.990	0.421
LAS	EPHA2	3	0.008	0.050	0.881	1.008	0.990	0.120
LAS	EPO	3	-0.069	0.045	0.132	0.934	0.962	0.219
LAS	ERI1	3	-0.020	0.030	0.501	0.980	0.962	0.767
LAS	ERP29	3	-0.004	0.030	0.889	0.996	0.992	0.565
LAS	ESAM	3	0.029	0.038	0.443	1.030	0.962	0.318
LAS	F12	3	-0.012	0.020	0.558	0.988	0.962	0.826
LAS	F13B	3	0.026	0.032	0.423	1.026	0.962	0.442
LAS	FADD	3	-0.002	0.029	0.953	0.998	0.992	0.643
LAS	FDX1	3	0.062	0.033	0.061	1.064	0.962	0.487
LAS	FES	3	0.001	0.045	0.977	1.001	0.992	0.232
LAS	FGF12	3	-0.011	0.050	0.822	0.989	0.990	0.165
LAS	FGF2	3	-0.011	0.026	0.676	0.989	0.990	0.679
LAS	FGF20	3	-0.020	0.030	0.514	0.980	0.962	0.745
LAS	FGF21	3	0.066	0.034	0.050	1.069	0.962	0.443
LAS	FGF23	3	0.180	0.200	0.370	1.197	0.962	0.000
LAS	FGF5	3	-0.051	0.064	0.429	0.950	0.962	0.005
LAS	FKBP7	3	0.051	0.030	0.092	1.052	0.962	0.742
LAS	FN1	3	-0.035	0.059	0.550	0.965	0.962	0.051
LAS	FOXJ3	3	0.039	0.042	0.351	1.039	0.962	0.288
LAS	FOXO3	3	-0.013	0.030	0.672	0.987	0.990	0.554
LAS	FUCA1	3	-0.035	0.016	0.025	0.966	0.962	0.708
LAS	FURIN	3	0.016	0.043	0.719	1.016	0.990	0.232

ST17; MR causal estimates for CAD and stroke on BP-associated proteins.

All estimates are from inverse variance weighted method (IVs >1) or Wald-ratio method (IV = 1). CAD, coronary artery disease; AS, all strokes; AIS, ischemic stroke; SVS, small vessel stroke; CES, cardioembolic stroke; LAS, large artery stroke

Exposure	Outocme	nsnp	Beta	SE	P-value	Odds ratio	FDR-corrected P-value	FDR-corrected Cochran's Q P-value
LAS	GCHFR	3	0.007	0.044	0.868	1.007	0.990	0.223
LAS	GFER	3	0.017	0.033	0.611	1.017	0.974	0.467
LAS	GHR	3	0.084	0.097	0.385	1.088	0.962	0.000
LAS	GHRHR	3	-0.023	0.044	0.599	0.977	0.974	0.260
LAS	GHRL	3	-0.153	0.115	0.181	0.858	0.962	0.000
LAS	GIMAP7	3	0.050	0.061	0.408	1.051	0.962	0.041
LAS	GIT1	3	0.032	0.045	0.482	1.032	0.962	0.221
LAS	GLO1	3	0.001	0.038	0.983	1.001	0.994	0.338
LAS	GORASP2	3	-0.041	0.030	0.163	0.959	0.962	0.538
LAS	GRP	3	-0.047	0.088	0.595	0.954	0.974	0.001
LAS	GRPEL1	3	0.086	0.030	0.004	1.090	0.955	0.560
LAS	GSTM4	3	-0.005	0.031	0.866	0.995	0.990	0.520
LAS	HADH	3	0.007	0.030	0.811	1.007	0.990	0.858
LAS	HEXIM1	3	0.004	0.034	0.898	1.004	0.992	0.424
LAS	HHEX	3	-0.002	0.034	0.959	0.998	0.992	0.448
LAS	HMOX2	3	0.089	0.043	0.039	1.093	0.962	0.258
LAS	HPGDS	3	0.082	0.107	0.446	1.085	0.962	0.000
LAS	HYAL1	3	-0.025	0.076	0.746	0.976	0.990	0.005
LAS	ICAM1	3	-0.349	0.432	0.420	0.706	0.962	0.000
LAS	ICAM2	3	-0.935	1.112	0.400	0.392	0.962	0.000
LAS	ICAM4	3	-0.414	0.399	0.300	0.661	0.962	0.000
LAS	IDUA	3	-0.023	0.026	0.370	0.977	0.962	0.566
LAS	IFI30	3	-0.051	0.085	0.548	0.950	0.962	0.000
LAS	IFIT3	3	-0.007	0.049	0.880	0.993	0.990	0.165
LAS	IFNGR2	3	-0.046	0.051	0.373	0.955	0.962	0.011
LAS	IGFBP3	3	-0.054	0.027	0.046	0.948	0.962	0.826
LAS	IL1RL1	3	-0.124	0.098	0.204	0.883	0.962	0.000
LAS	IMMT	3	0.025	0.029	0.381	1.026	0.962	0.756
LAS	IMPA1	3	0.003	0.030	0.915	1.003	0.992	0.679
LAS	ING1	3	-0.018	0.035	0.613	0.983	0.974	0.449
LAS	ITGAL	3	0.001	0.030	0.976	1.001	0.992	0.783
LAS	ITIH1	3	-0.010	0.034	0.756	0.990	0.990	0.467
LAS	KIF22	3	0.061	0.046	0.185	1.063	0.962	0.219
LAS	KIFBP	3	0.007	0.035	0.841	1.007	0.990	0.424
LAS	LACRT	3	-0.020	0.035	0.573	0.980	0.970	0.424
LAS	LAYN	3	0.216	0.159	0.173	1.242	0.962	0.000
LAS	LMOD1	3	-0.012	0.027	0.659	0.988	0.990	0.590
LAS	LMOD1	3	-0.027	0.027	0.323	0.974	0.962	0.826
LAS	LMOD1	3	-0.033	0.026	0.202	0.967	0.962	0.668
LAS	LMOD1	3	-0.029	0.027	0.267	0.971	0.962	0.985
LAS	LRIG1	3	-0.055	0.077	0.478	0.947	0.962	0.000
LAS	LYAR	3	-0.010	0.054	0.859	0.990	0.990	0.117
LAS	M6PR	3	-0.045	0.049	0.358	0.956	0.962	0.176
LAS	MANEAL	3	-0.043	0.030	0.159	0.958	0.962	0.863
LAS	MANSC4	3	0.085	0.059	0.153	1.089	0.962	0.014
LAS	MAP4K5	3	-0.019	0.037	0.605	0.981	0.974	0.378
LAS	MDH1	3	0.003	0.034	0.932	1.003	0.992	0.442
LAS	MEGF9	3	0.079	0.129	0.544	1.082	0.962	0.000
LAS	MFGE8	3	0.064	0.058	0.275	1.066	0.962	0.051
LAS	MPHOSPH8	3	0.033	0.030	0.266	1.034	0.962	0.991
LAS	MPI	3	0.021	0.029	0.470	1.021	0.962	0.679
LAS	MPIG6B	3	-0.011	0.056	0.848	0.989	0.990	0.086
LAS	MSRA	3	-0.002	0.029	0.958	0.998	0.992	0.566
LAS	MST1	3	-0.004	0.035	0.906	0.996	0.992	0.068
LAS	MVK	3	0.002	0.053	0.970	1.002	0.992	0.119
LAS	MXRA8	3	-0.042	0.028	0.126	0.959	0.962	0.669
LAS	NADK	3	0.048	0.031	0.116	1.049	0.962	0.487

ST17; MR causal estimates for CAD and stroke on BP-associated proteins.

All estimates are from inverse variance weighted method (IVs >1) or Wald-ratio method (IV = 1). CAD, coronary artery disease; AS, all strokes; AIS, ischemic stroke; SVS, small vessel stroke; CES, cardioembolic stroke; LAS, large artery stroke

Exposure	Outocme	nsnp	Beta	SE	P-value	Odds ratio	FDR-corrected P-value	FDR-corrected Cochran's Q P-value
LAS	NAGA	3	-0.027	0.036	0.458	0.973	0.962	0.394
LAS	NBN	3	-0.033	0.034	0.334	0.968	0.962	0.445
LAS	NFE2	3	-0.001	0.029	0.971	0.999	0.992	0.601
LAS	NFU1	3	0.018	0.030	0.543	1.018	0.962	0.804
LAS	NGF	3	-0.011	0.042	0.788	0.989	0.990	0.285
LAS	NGFR	3	-0.004	0.043	0.927	0.996	0.992	0.266
LAS	NOMO1	3	0.046	0.058	0.422	1.047	0.962	0.047
LAS	NOS3	3	0.175	0.160	0.275	1.191	0.962	0.000
LAS	NOTCH3	3	-0.118	0.076	0.123	0.889	0.962	0.003
LAS	NPPB	3	-0.024	0.029	0.398	0.976	0.962	0.832
LAS	NTRK3	3	-0.038	0.028	0.184	0.963	0.962	0.900
LAS	NUCB2	3	-0.008	0.048	0.862	0.992	0.990	0.143
LAS	NUDT5	3	0.025	0.045	0.580	1.025	0.971	0.222
LAS	NUMB	3	-0.003	0.051	0.957	0.997	0.992	0.132
LAS	OGA	3	0.001	0.029	0.977	1.001	0.992	0.669
LAS	OPLAH	3	0.005	0.029	0.868	1.005	0.990	0.838
LAS	OTUD6B	3	-0.023	0.041	0.576	0.977	0.970	0.285
LAS	PAM	3	0.028	0.040	0.478	1.029	0.962	0.224
LAS	PAMR1	3	0.071	0.039	0.072	1.073	0.962	0.253
LAS	PARP1	3	-0.001	0.040	0.972	0.999	0.992	0.301
LAS	PCBP2	3	-0.007	0.029	0.803	0.993	0.990	0.594
LAS	PCOLCE	3	-0.050	0.127	0.693	0.951	0.990	0.003
LAS	PCSK7	3	-0.014	0.059	0.814	0.986	0.990	0.066
LAS	PDE5A	3	-0.002	0.032	0.959	0.998	0.992	0.481
LAS	PDGFRA	3	-0.015	0.035	0.666	0.985	0.990	0.394
LAS	PDIA3	3	0.015	0.041	0.708	1.015	0.990	0.304
LAS	PECAM1	3	-0.562	0.599	0.348	0.570	0.962	0.000
LAS	PFKFB2	3	-0.027	0.029	0.345	0.973	0.962	0.704
LAS	PGF	3	0.068	0.054	0.209	1.071	0.962	0.045
LAS	PHLDB1	3	0.005	0.032	0.870	1.005	0.990	0.487
LAS	PKD1	3	0.016	0.064	0.803	1.016	0.990	0.015
LAS	PLA2G1B	3	-0.038	0.041	0.356	0.963	0.962	0.272
LAS	PLXDC2	3	-0.218	0.216	0.313	0.804	0.962	0.000
LAS	PMS1	3	-0.016	0.032	0.608	0.984	0.974	0.515
LAS	PMVK	3	-0.027	0.034	0.416	0.973	0.962	0.467
LAS	PPP1R14D	3	-0.017	0.029	0.561	0.983	0.962	0.668
LAS	PRDX1	3	0.029	0.030	0.329	1.029	0.962	0.566
LAS	PRG2	3	0.102	0.041	0.012	1.108	0.962	0.249
LAS	PRKAB1	3	0.011	0.030	0.709	1.011	0.990	0.659
LAS	PROCR	3	-0.062	0.029	0.034	0.940	0.962	0.701
LAS	PRSS53	3	-0.048	0.058	0.406	0.953	0.962	0.004
LAS	PRTFDC1	3	-0.019	0.030	0.518	0.981	0.962	0.557
LAS	PSMD5	3	-0.002	0.030	0.941	0.998	0.992	0.557
LAS	PSRC1	3	-0.042	0.059	0.475	0.959	0.962	0.069
LAS	PTPRF	3	-0.068	0.066	0.299	0.934	0.962	0.018
LAS	PTRHD1	3	-0.021	0.031	0.492	0.979	0.962	0.518
LAS	PYDC1	3	0.022	0.025	0.394	1.022	0.962	0.826
LAS	QPCT	3	-0.055	0.053	0.304	0.947	0.962	0.087
LAS	RABEPK	3	-0.011	0.031	0.725	0.989	0.990	0.517
LAS	RANBP1	3	-0.017	0.056	0.763	0.983	0.990	0.083
LAS	RARRES1	3	-0.073	0.062	0.243	0.930	0.962	0.036
LAS	RARRES2	3	0.062	0.038	0.106	1.064	0.962	0.318
LAS	RELT	3	0.030	0.026	0.240	1.031	0.962	0.668
LAS	RSPO3	3	-0.034	0.046	0.460	0.966	0.962	0.135
LAS	SCARA5	3	0.279	0.273	0.307	1.321	0.962	0.000
LAS	SDC1	3	-0.127	0.193	0.510	0.881	0.962	0.000
LAS	SDCCAG8	3	0.011	0.029	0.702	1.011	0.990	0.697

ST17; MR causal estimates for CAD and stroke on BP-associated proteins.

All estimates are from inverse variance weighted method (IVs >1) or Wald-ratio method (IV = 1). CAD, coronary artery disease; AS, all strokes; AIS, ischemic stroke; SVS, small vessel stroke; CES, cardioembolic stroke; LAS, large artery stroke

Exposure	Outocme	nsnp	Beta	SE	P-value	Odds ratio	FDR-corrected P-value	FDR-corrected Cochran's Q P-value
LAS	SDHB	3	0.039	0.051	0.437	1.040	0.962	0.140
LAS	SELENOP	3	0.049	0.082	0.547	1.051	0.962	0.003
LAS	SEMA6C	3	0.058	0.064	0.368	1.059	0.962	0.045
LAS	SERPING1	3	0.057	0.056	0.313	1.058	0.962	0.068
LAS	SERPINI1	3	0.070	0.087	0.419	1.073	0.962	0.000
LAS	SH2B3	3	0.004	0.043	0.922	1.004	0.992	0.266
LAS	SHMT1	3	0.005	0.028	0.856	1.005	0.990	0.468
LAS	SIL1	3	-0.020	0.059	0.739	0.981	0.990	0.075
LAS	SLC16A1	3	-0.021	0.030	0.484	0.979	0.962	0.598
LAS	SLC39A14	3	-0.010	0.053	0.854	0.990	0.990	0.119
LAS	SLC9A3R2	3	0.137	0.197	0.487	1.147	0.962	0.000
LAS	SMOC2	3	0.032	0.047	0.500	1.032	0.962	0.110
LAS	SMTN	3	0.026	0.029	0.372	1.026	0.962	0.612
LAS	SOST	3	-0.068	0.082	0.407	0.934	0.962	0.001
LAS	SPINK8	3	0.000	0.030	0.991	1.000	0.998	0.517
LAS	SPRED2	3	0.029	0.030	0.337	1.029	0.962	0.950
LAS	SPRING1	3	0.053	0.060	0.383	1.054	0.962	0.068
LAS	STC1	3	-0.055	0.083	0.503	0.946	0.962	0.001
LAS	STX4	3	0.044	0.029	0.131	1.045	0.962	0.742
LAS	SYAP1	3	-0.002	0.030	0.946	0.998	0.992	0.679
LAS	TARBP2	3	0.027	0.029	0.354	1.028	0.962	0.629
LAS	TBC1D17	3	-0.006	0.028	0.820	0.994	0.990	0.538
LAS	TBC1D23	3	0.005	0.029	0.874	1.005	0.990	0.600
LAS	TEK	3	0.315	0.350	0.369	1.370	0.962	0.000
LAS	TGFB2	3	0.007	0.047	0.877	1.007	0.990	0.209
LAS	TIE1	3	0.900	0.988	0.362	2.460	0.962	0.000
LAS	TJAP1	3	0.009	0.029	0.763	1.009	0.990	0.566
LAS	TMEM106A	3	-0.052	0.029	0.074	0.949	0.962	0.566
LAS	TNFRSF12A	3	-0.040	0.062	0.524	0.961	0.962	0.024
LAS	TNFRSF13B	3	-0.036	0.052	0.484	0.964	0.962	0.110
LAS	TNFRSF17	3	-0.092	0.078	0.238	0.912	0.962	0.004
LAS	TNFSF12	3	-0.218	0.166	0.190	0.804	0.962	0.000
LAS	TNFSF13B	3	0.079	0.107	0.462	1.082	0.962	0.000
LAS	TP53	3	0.032	0.030	0.297	1.032	0.962	0.915
LAS	TP53BP1	3	-0.029	0.059	0.627	0.972	0.978	0.068
LAS	TP53I3	3	0.003	0.028	0.904	1.003	0.992	0.566
LAS	TP53INP1	3	0.018	0.030	0.550	1.018	0.962	0.764
LAS	TWF2	3	-0.006	0.029	0.845	0.994	0.990	0.655
LAS	UBE2L6	3	-0.004	0.038	0.922	0.996	0.992	0.341
LAS	UMOD	3	0.038	0.053	0.477	1.039	0.962	0.020
LAS	UXS1	3	-0.121	0.046	0.009	0.886	0.962	0.214
LAS	VAT1	3	0.078	0.050	0.122	1.081	0.962	0.142
LAS	VSIG2	3	-0.067	0.030	0.027	0.935	0.962	0.487
LAS	WARS	3	0.075	0.095	0.431	1.077	0.962	0.000
LAS	WASHC3	3	0.009	0.029	0.758	1.009	0.990	0.566
LAS	WNT9A	3	-0.015	0.046	0.744	0.985	0.990	0.132
LAS	WWP2	3	0.025	0.031	0.433	1.025	0.962	0.487
LAS	YAP1	3	0.035	0.026	0.185	1.036	0.962	0.668
LAS	YOD1	3	-0.022	0.049	0.656	0.978	0.990	0.142
LAS	ZBTB17	3	0.067	0.029	0.022	1.070	0.962	0.756
LAS	ZFYVE19	3	-0.025	0.029	0.391	0.976	0.962	0.580
AS	AAMDC	24	0.069	0.038	0.069	1.071	0.367	0.002
AS	ADAMTSL5	24	-0.041	0.037	0.269	0.960	0.548	0.015
AS	APOBR	24	-0.004	0.044	0.921	0.996	0.973	0.000
AS	ASPN	24	-0.013	0.037	0.722	0.987	0.811	0.014
AS	CALCOCO2	24	0.069	0.060	0.251	1.072	0.532	0.000
AS	CEP170	24	0.071	0.041	0.081	1.074	0.391	0.005

ST17; MR causal estimates for CAD and stroke on BP-associated proteins.

All estimates are from inverse variance weighted method (IVs >1) or Wald-ratio method (IV = 1). CAD, coronary artery disease; AS, all strokes; AIS, ischemic stroke; SVS, small vessel stroke; CES, cardioembolic stroke; LAS, large artery stroke

Exposure	Outocme	nsnp	Beta	SE	P-value	Odds ratio	FDR-corrected P-value	FDR-corrected Cochran's Q P-value
AS	CPTP	24	-0.018	0.030	0.547	0.982	0.699	0.543
AS	EDN1	24	-0.026	0.050	0.599	0.974	0.732	0.000
AS	ENPEP	24	0.040	0.033	0.215	1.041	0.489	0.249
AS	FDX1	24	0.028	0.040	0.476	1.029	0.688	0.014
AS	GRP	24	0.031	0.039	0.433	1.031	0.675	0.005
AS	GSTM4	24	-0.018	0.031	0.553	0.982	0.699	0.340
AS	HADH	24	-0.023	0.040	0.559	0.977	0.699	0.017
AS	KIF22	24	0.049	0.033	0.135	1.050	0.409	0.186
AS	LMOD1	24	-0.037	0.058	0.526	0.964	0.699	0.000
AS	M6PR	24	0.033	0.046	0.477	1.033	0.688	0.000
AS	MANSC4	24	-0.026	0.033	0.427	0.974	0.675	0.012
AS	NFE2	24	0.112	0.048	0.020	1.118	0.346	0.000
AS	NGFR	24	0.035	0.029	0.236	1.035	0.511	0.562
AS	OPLAH	24	0.092	0.054	0.089	1.096	0.391	0.000
AS	PKD1	24	-0.067	0.053	0.200	0.935	0.476	0.000
AS	PRG2	24	0.115	0.061	0.060	1.122	0.359	0.000
AS	PROCR	24	-0.310	0.318	0.329	0.733	0.610	0.000
AS	PYDC1	24	0.049	0.025	0.051	1.050	0.346	0.824
AS	RANBP1	24	0.039	0.048	0.418	1.040	0.671	0.000
AS	SIL1	24	0.000	0.031	0.991	1.000	0.991	0.340
AS	SPINK8	24	-0.029	0.029	0.317	0.971	0.596	0.444
AS	TWF2	24	0.072	0.042	0.089	1.074	0.391	0.002
AS	UBE2L6	24	0.123	0.063	0.050	1.131	0.346	0.000
AS	YOD1	24	0.025	0.042	0.546	1.026	0.699	0.001
AS	ABO	24	0.365	0.524	0.487	1.440	0.691	0.000
AS	ADAMTS1	24	-0.004	0.038	0.906	0.996	0.968	0.027
AS	ADAMTS4	24	0.065	0.044	0.145	1.067	0.409	0.000
AS	ANKMY2	24	0.068	0.043	0.110	1.070	0.409	0.002
AS	APOA1	24	0.022	0.036	0.551	1.022	0.699	0.013
AS	APOA2	24	0.068	0.028	0.015	1.070	0.346	0.743
AS	APOC1	24	-0.020	0.046	0.666	0.980	0.789	0.000
AS	BAG4	24	0.083	0.043	0.051	1.087	0.346	0.001
AS	BNIP3L	24	0.083	0.042	0.052	1.086	0.346	0.002
AS	CFHR2	24	0.015	0.025	0.543	1.016	0.699	0.006
AS	CFHR4	24	0.042	0.039	0.285	1.043	0.567	0.000
AS	DBN1	24	0.036	0.036	0.310	1.037	0.587	0.059
AS	DENR	24	0.041	0.047	0.383	1.042	0.641	0.000
AS	ERP29	24	0.067	0.043	0.114	1.070	0.409	0.002
AS	F12	24	0.005	0.020	0.790	1.005	0.871	0.521
AS	F13B	24	-0.003	0.037	0.932	0.997	0.974	0.007
AS	FGF12	24	0.003	0.030	0.922	1.003	0.973	0.849
AS	FGF20	24	-0.012	0.032	0.704	0.988	0.805	0.296
AS	FN1	24	-0.025	0.034	0.467	0.976	0.688	0.072
AS	FOXJ3	24	0.061	0.042	0.141	1.063	0.409	0.004
AS	GCHFR	24	0.077	0.039	0.051	1.080	0.346	0.005
AS	GHR	24	0.046	0.037	0.215	1.047	0.489	0.004
AS	GIMAP7	24	0.104	0.048	0.029	1.110	0.346	0.000
AS	GIT1	24	0.072	0.037	0.055	1.075	0.346	0.022
AS	ITGAL	24	0.088	0.094	0.351	1.092	0.634	0.000
AS	ITIH1	24	0.034	0.032	0.284	1.035	0.567	0.249
AS	LMOD1	24	-0.033	0.047	0.475	0.967	0.688	0.000
AS	MDH1	24	0.063	0.043	0.147	1.065	0.409	0.001
AS	MST1	24	-0.007	0.018	0.716	0.993	0.810	0.264
AS	MXRA8	24	-0.081	0.039	0.037	0.922	0.346	0.002
AS	NAGA	24	-0.005	0.044	0.914	0.995	0.971	0.001
AS	NUMB	24	0.066	0.042	0.116	1.069	0.409	0.002
AS	PDE5A	24	0.082	0.042	0.052	1.085	0.346	0.002

ST17; MR causal estimates for CAD and stroke on BP-associated proteins.

All estimates are from inverse variance weighted method (IVs >1) or Wald-ratio method (IV = 1). CAD, coronary artery disease; AS, all strokes; AIS, ischemic stroke; SVS, small vessel stroke; CES, cardioembolic stroke; LAS, large artery stroke

Exposure	Outocme	nsnp	Beta	SE	P-value	Odds ratio	FDR-corrected P-value	FDR-corrected Cochran's Q P-value
AS	PDIA3	24	-0.003	0.036	0.931	0.997	0.974	0.070
AS	PTRHD1	24	0.061	0.042	0.147	1.063	0.409	0.002
AS	SELENOP	24	0.018	0.044	0.685	1.018	0.790	0.000
AS	SEMA6C	24	-0.031	0.035	0.384	0.970	0.641	0.092
AS	SERPING1	24	0.052	0.058	0.373	1.053	0.641	0.000
AS	SERPINI1	24	-0.065	0.035	0.062	0.937	0.365	0.027
AS	SPRED2	24	0.025	0.032	0.430	1.026	0.675	0.249
AS	SYAP1	24	0.095	0.041	0.021	1.100	0.346	0.004
AS	TNFRSF17	24	-0.004	0.047	0.937	0.996	0.974	0.000
AS	TP53BP1	24	0.010	0.037	0.786	1.010	0.869	0.048
AS	TP53I3	24	0.067	0.042	0.107	1.070	0.409	0.001
AS	ATXN2L	24	0.035	0.048	0.459	1.036	0.688	0.000
AS	BRSK2	24	0.018	0.027	0.507	1.018	0.697	0.673
AS	CACNB3	24	0.077	0.035	0.027	1.080	0.346	0.081
AS	CD164L2	24	-0.019	0.029	0.516	0.981	0.698	0.463
AS	CLIC5	24	0.027	0.037	0.477	1.027	0.688	0.036
AS	DDHD2	24	0.089	0.041	0.031	1.093	0.346	0.002
AS	DUSP29	24	0.023	0.032	0.472	1.023	0.688	0.258
AS	EIF4G3	24	0.066	0.033	0.049	1.068	0.346	0.134
AS	HHEX	24	0.069	0.043	0.108	1.071	0.409	0.001
AS	LMOD1	24	-0.039	0.054	0.475	0.962	0.688	0.000
AS	PCBP2	24	0.075	0.049	0.123	1.078	0.409	0.000
AS	PLXDC2	24	-0.165	0.140	0.239	0.848	0.515	0.000
AS	PMS1	24	0.059	0.040	0.144	1.061	0.409	0.011
AS	PPP1R14D	24	0.024	0.036	0.496	1.025	0.696	0.055
AS	PRSS53	24	-0.039	0.035	0.267	0.962	0.548	0.000
AS	PSMD5	24	-0.041	0.029	0.163	0.960	0.413	0.714
AS	SMTN	24	0.091	0.032	0.005	1.095	0.346	0.170
AS	WASHC3	24	0.094	0.042	0.024	1.099	0.346	0.002
AS	YAP1	24	0.019	0.043	0.667	1.019	0.789	0.000
AS	ACADM	24	-0.054	0.038	0.151	0.947	0.409	0.032
AS	ACRBP	24	0.076	0.053	0.154	1.079	0.409	0.000
AS	AMOTL2	24	0.052	0.038	0.173	1.053	0.422	0.022
AS	BRAP	24	0.101	0.045	0.025	1.106	0.346	0.000
AS	CCND2	24	-0.009	0.038	0.804	0.991	0.883	0.028
AS	CETN3	24	0.084	0.040	0.036	1.087	0.346	0.005
AS	DNAJC9	24	0.068	0.037	0.066	1.070	0.367	0.021
AS	DUSP13	24	0.019	0.028	0.505	1.019	0.697	0.560
AS	ERI1	24	-0.015	0.041	0.718	0.985	0.810	0.005
AS	GORASP2	24	0.043	0.048	0.369	1.044	0.641	0.000
AS	IFI30	24	0.040	0.088	0.653	1.040	0.780	0.000
AS	IFIT3	24	0.036	0.046	0.433	1.037	0.675	0.000
AS	IMMT	24	0.063	0.043	0.141	1.065	0.409	0.001
AS	LACRT	24	0.002	0.029	0.955	1.002	0.984	0.477
AS	LMOD1	24	-0.050	0.046	0.279	0.951	0.564	0.000
AS	MANEAL	24	0.018	0.030	0.554	1.018	0.699	0.825
AS	NFU1	24	0.079	0.050	0.116	1.082	0.409	0.000
AS	OGA	24	0.067	0.048	0.165	1.069	0.413	0.000
AS	OTUD6B	24	0.062	0.049	0.205	1.064	0.479	0.000
AS	PCSK7	24	-0.041	0.040	0.304	0.960	0.581	0.007
AS	PHLDB1	24	0.061	0.036	0.088	1.063	0.391	0.045
AS	SDCCAG8	24	0.087	0.039	0.025	1.091	0.346	0.007
AS	SDHB	24	0.102	0.048	0.032	1.107	0.346	0.000
AS	SLC9A3R2	24	0.024	0.059	0.683	1.025	0.790	0.000
AS	SPRING1	24	0.007	0.040	0.861	1.007	0.933	0.015
AS	TGFB2	24	-0.011	0.031	0.716	0.989	0.810	0.360
AS	TMEM106A	24	0.060	0.044	0.166	1.062	0.413	0.001

ST17; MR causal estimates for CAD and stroke on BP-associated proteins.

All estimates are from inverse variance weighted method (IVs >1) or Wald-ratio method (IV = 1). CAD, coronary artery disease; AS, all strokes; AIS, ischemic stroke; SVS, small vessel stroke; CES, cardioembolic stroke; LAS, large artery stroke

Exposure	Outocme	nsnp	Beta	SE	P-value	Odds ratio	FDR-corrected P-value	FDR-corrected Cochran's Q P-value
AS	VSIG2	24	-0.073	0.094	0.441	0.930	0.679	0.000
AS	ZFYVE19	24	0.059	0.044	0.182	1.061	0.440	0.000
AS	ACOX1	24	0.067	0.034	0.049	1.069	0.346	0.142
AS	ADAMTS13	24	-0.024	0.040	0.547	0.976	0.699	0.001
AS	ADAMTS16	24	-0.061	0.038	0.105	0.941	0.409	0.005
AS	AOC3	24	-0.047	0.037	0.211	0.954	0.489	0.001
AS	AXL	24	0.073	0.066	0.268	1.075	0.548	0.000
AS	BMP6	24	-0.050	0.081	0.539	0.952	0.699	0.000
AS	CCN3	24	-0.059	0.042	0.160	0.943	0.413	0.000
AS	CD14	24	-0.074	0.058	0.205	0.929	0.479	0.000
AS	CD46	24	-0.005	0.062	0.938	0.995	0.974	0.000
AS	CD59	24	0.039	0.026	0.140	1.040	0.409	0.479
AS	COL1A1	24	-0.087	0.061	0.153	0.916	0.409	0.000
AS	COMP	24	-0.118	0.046	0.010	0.888	0.346	0.000
AS	COMT	24	0.066	0.044	0.133	1.069	0.409	0.000
AS	CTF1	24	0.021	0.032	0.498	1.022	0.696	0.276
AS	DOK2	24	0.078	0.046	0.090	1.081	0.391	0.000
AS	DPP4	24	-0.090	0.030	0.003	0.914	0.346	0.228
AS	EFEMP1	24	-0.018	0.041	0.671	0.983	0.790	0.000
AS	ESAM	24	-0.022	0.074	0.765	0.978	0.857	0.000
AS	FADD	24	0.061	0.044	0.166	1.063	0.413	0.000
AS	FUCA1	24	-0.043	0.028	0.124	0.958	0.409	0.000
AS	GHRL	24	-0.002	0.036	0.952	0.998	0.984	0.015
AS	GLO1	24	0.031	0.045	0.484	1.032	0.690	0.000
AS	HYAL1	24	-0.064	0.041	0.120	0.938	0.409	0.003
AS	ICAM1	24	-0.134	0.219	0.540	0.874	0.699	1.39025e-319
AS	ICAM2	24	-0.398	0.547	0.467	0.671	0.688	0.000
AS	IGFBP3	24	-0.060	0.032	0.064	0.942	0.366	0.054
AS	IL1RL1	24	-0.055	0.077	0.470	0.946	0.688	0.000
AS	MEGF9	24	0.034	0.045	0.440	1.035	0.679	0.000
AS	MPHOSPH8	24	0.064	0.043	0.140	1.066	0.409	0.001
AS	NADK	24	-0.001	0.038	0.981	0.999	0.988	0.008
AS	NOTCH3	24	-0.128	0.061	0.037	0.880	0.346	0.000
AS	NPPB	24	0.064	0.039	0.096	1.066	0.401	0.010
AS	PAM	24	-0.049	0.034	0.145	0.952	0.409	0.020
AS	PCOLCE	24	0.089	0.060	0.139	1.093	0.409	0.013
AS	PDGFRA	24	-0.041	0.044	0.346	0.959	0.630	0.000
AS	PLA2G1B	24	-0.036	0.049	0.467	0.965	0.688	0.000
AS	PTPRF	24	-0.039	0.064	0.544	0.962	0.699	0.000
AS	QPCT	24	0.002	0.047	0.958	1.002	0.984	0.000
AS	RARRES2	24	0.014	0.035	0.688	1.014	0.790	0.044
AS	SDC1	24	-0.084	0.079	0.288	0.919	0.570	0.000
AS	SOST	24	-0.035	0.041	0.390	0.965	0.641	0.000
AS	TIE1	24	0.086	0.134	0.521	1.090	0.698	0.000
AS	TNFSF13B	24	0.026	0.049	0.595	1.026	0.729	0.000
AS	TP53INP1	24	-0.047	0.029	0.110	0.954	0.409	0.722
AS	UMOD	24	-0.019	0.033	0.568	0.981	0.706	0.001
AS	ZBTB17	24	0.052	0.047	0.269	1.053	0.548	0.000
AS	ADAM23	24	-0.098	0.107	0.357	0.906	0.636	0.000
AS	AGRP	24	0.075	0.052	0.145	1.078	0.409	0.000
AS	AOC1	24	-0.066	0.053	0.217	0.936	0.489	0.000
AS	ARHGEF12	24	0.063	0.039	0.103	1.065	0.409	0.011
AS	CTSO	24	0.041	0.045	0.355	1.042	0.636	0.000
AS	DAG1	24	0.081	0.068	0.230	1.085	0.509	0.000
AS	DNER	24	-0.024	0.027	0.379	0.977	0.641	0.496
AS	EPO	24	-0.077	0.046	0.091	0.925	0.392	0.000
AS	FGF2	24	0.068	0.049	0.166	1.070	0.413	0.000

ST17; MR causal estimates for CAD and stroke on BP-associated proteins.
All estimates are from inverse variance weighted method (IVs >1) or Wald-ratio method (IV = 1). CAD, coronary artery disease; AS, all strokes; AIS, ischemic stroke; SVS, small vessel stroke; CES, cardioembolic stroke; LAS, large artery stroke

Exposure	Outocme	nsnp	Beta	SE	P-value	Odds ratio	FDR-corrected P-value	FDR-corrected Cochran's Q P-value
AS	FGF5	24	-0.069	0.046	0.140	0.934	0.409	0.000
AS	HEXIM1	24	0.087	0.046	0.058	1.090	0.355	0.000
AS	ICAM4	24	-0.210	0.250	0.401	0.811	0.654	0.000
AS	MPIG6B	24	0.056	0.047	0.232	1.058	0.509	0.000
AS	MVK	24	0.099	0.051	0.053	1.105	0.346	0.000
AS	NBN	24	0.026	0.047	0.579	1.026	0.714	0.000
AS	PARP1	24	0.074	0.048	0.125	1.077	0.409	0.000
AS	PGF	24	0.088	0.044	0.043	1.092	0.346	0.000
AS	PRKAB1	24	0.057	0.040	0.152	1.059	0.409	0.008
AS	SHMT1	24	0.065	0.036	0.075	1.067	0.390	0.002
AS	SMOC2	24	-0.011	0.041	0.784	0.989	0.869	0.000
AS	TNFRSF13B	24	0.077	0.067	0.251	1.080	0.532	0.000
AS	TNFSF12	24	-0.014	0.110	0.897	0.986	0.965	0.000
AS	WNT9A	24	0.013	0.032	0.680	1.013	0.790	0.053
AS	AMFR	24	0.022	0.034	0.520	1.022	0.698	0.147
AS	B4GAT1	24	-0.071	0.038	0.065	0.932	0.366	0.005
AS	BCAM	24	0.091	0.099	0.359	1.095	0.636	0.000
AS	CALB2	24	-0.018	0.036	0.618	0.982	0.741	0.050
AS	CALCA	24	0.034	0.033	0.298	1.035	0.573	0.006
AS	CERT	24	0.089	0.042	0.033	1.093	0.346	0.004
AS	CHMP1A	24	0.066	0.045	0.148	1.068	0.409	0.000
AS	DARS1	24	0.065	0.043	0.128	1.068	0.409	0.001
AS	DPEP1	24	0.045	0.050	0.367	1.046	0.641	0.000
AS	EFNA1	24	0.037	0.053	0.490	1.037	0.691	0.000
AS	FKBP7	24	0.039	0.038	0.298	1.040	0.573	0.038
AS	GHRHR	24	-0.058	0.033	0.080	0.944	0.391	0.220
AS	HMOX2	24	0.115	0.045	0.011	1.122	0.346	0.000
AS	IFNGR2	24	-0.038	0.061	0.537	0.963	0.699	0.000
AS	IMPA1	24	0.068	0.038	0.069	1.071	0.367	0.025
AS	ING1	24	-0.001	0.046	0.984	0.999	0.988	0.000
AS	LAYN	24	0.043	0.050	0.387	1.044	0.641	0.000
AS	MAP4K5	24	0.075	0.045	0.095	1.078	0.401	0.000
AS	MFGE8	24	0.019	0.035	0.580	1.020	0.714	0.032
AS	NGF	24	0.040	0.029	0.170	1.041	0.418	0.994
AS	NOMO1	24	-0.008	0.034	0.816	0.992	0.888	0.047
AS	NOS3	24	0.150	0.077	0.052	1.162	0.346	0.000
AS	NTRK3	24	-0.124	0.072	0.084	0.884	0.391	0.000
AS	NUDT5	24	0.068	0.045	0.135	1.070	0.409	0.000
AS	PAMR1	24	0.047	0.039	0.230	1.048	0.509	0.001
AS	PECAM1	24	-0.316	0.415	0.446	0.729	0.683	0.000
AS	PMVK	24	0.051	0.038	0.183	1.052	0.440	0.021
AS	PRDX1	24	0.022	0.038	0.556	1.023	0.699	0.020
AS	PRTFDC1	24	0.060	0.042	0.153	1.062	0.409	0.003
AS	RELT	24	0.059	0.056	0.294	1.061	0.573	0.000
AS	SCARA5	24	0.064	0.091	0.482	1.066	0.690	0.000
AS	SLC16A1	24	0.023	0.035	0.516	1.023	0.698	0.083
AS	SLC39A14	24	0.055	0.031	0.078	1.057	0.391	0.288
AS	STC1	24	-0.046	0.069	0.506	0.955	0.697	0.000
AS	TARBP2	24	0.091	0.044	0.038	1.095	0.346	0.001
AS	TBC1D17	24	0.040	0.042	0.337	1.041	0.620	0.001
AS	WARS	24	0.111	0.078	0.153	1.117	0.409	0.000
AS	WWP2	24	0.089	0.038	0.021	1.093	0.346	0.012
AS	ADAMTS15	24	0.061	0.032	0.054	1.063	0.346	0.227
AS	ADAMTS8	24	-0.068	0.035	0.051	0.934	0.346	0.021
AS	ADCYAP1R1	24	0.001	0.030	0.968	1.001	0.986	0.597
AS	ADM	24	0.100	0.041	0.014	1.105	0.346	0.000
AS	ARSB	24	0.044	0.054	0.421	1.045	0.671	0.000

ST17; MR causal estimates for CAD and stroke on BP-associated proteins.

All estimates are from inverse variance weighted method (IVs >1) or Wald-ratio method (IV = 1). CAD, coronary artery disease; AS, all strokes; AIS, ischemic stroke; SVS, small vessel stroke; CES, cardioembolic stroke; LAS, large artery stroke

Exposure	Outocme	nsnp	Beta	SE	P-value	Odds ratio	FDR-corrected P-value	FDR-corrected Cochran's Q P-value
AS	CA12	24	-0.025	0.044	0.560	0.975	0.699	0.000
AS	CA9	24	-0.010	0.036	0.775	0.990	0.864	0.043
AS	CES2	24	-0.042	0.049	0.387	0.959	0.641	0.000
AS	CLMP	24	0.026	0.032	0.418	1.026	0.671	0.044
AS	CPXM1	24	0.052	0.060	0.386	1.053	0.641	0.000
AS	DTX3	24	-0.023	0.027	0.390	0.977	0.641	0.425
AS	ELOA	24	0.056	0.053	0.297	1.057	0.573	0.000
AS	EPHA2	24	-0.039	0.060	0.516	0.962	0.698	0.000
AS	FES	24	0.019	0.037	0.605	1.019	0.736	0.051
AS	FGF21	24	-0.001	0.034	0.974	0.999	0.987	0.098
AS	FGF23	24	0.061	0.070	0.388	1.063	0.641	0.000
AS	FOXO3	24	0.036	0.045	0.420	1.037	0.671	0.000
AS	FURIN	24	0.009	0.040	0.815	1.009	0.888	0.005
AS	GFER	24	0.092	0.044	0.036	1.096	0.346	0.001
AS	GRPEL1	24	0.132	0.053	0.014	1.141	0.346	0.000
AS	HPGDS	24	-0.057	0.051	0.259	0.944	0.543	0.000
AS	IDUA	24	-0.027	0.053	0.613	0.974	0.741	0.000
AS	KIFBP	24	0.089	0.039	0.021	1.093	0.346	0.015
AS	LRIG1	24	0.048	0.040	0.233	1.049	0.509	0.000
AS	LYAR	24	-0.007	0.039	0.866	0.994	0.935	0.021
AS	MPI	24	0.087	0.041	0.037	1.090	0.346	0.002
AS	MSRA	24	0.075	0.043	0.084	1.077	0.391	0.001
AS	NUCB2	24	0.114	0.059	0.052	1.121	0.346	0.000
AS	PFKFB2	24	0.054	0.038	0.159	1.055	0.413	0.013
AS	PSRC1	24	0.039	0.041	0.346	1.039	0.630	0.005
AS	RABEPK	24	0.017	0.034	0.618	1.017	0.741	0.162
AS	RARRES1	24	0.001	0.042	0.976	1.001	0.987	0.001
AS	RSPO3	24	0.002	0.039	0.964	1.002	0.985	0.001
AS	SH2B3	24	0.071	0.041	0.082	1.074	0.391	0.005
AS	STX4	24	0.075	0.038	0.047	1.078	0.346	0.017
AS	TBC1D23	24	0.087	0.036	0.016	1.091	0.346	0.044
AS	TEK	24	-0.037	0.090	0.684	0.964	0.790	0.000
AS	TJAP1	24	0.082	0.039	0.036	1.086	0.346	0.007
AS	TNFRSF12A	24	-0.016	0.038	0.674	0.984	0.790	0.004
AS	TP53	24	-0.004	0.037	0.907	0.996	0.968	0.041
AS	UXS1	24	-0.094	0.061	0.120	0.910	0.409	0.000
AS	VAT1	24	0.045	0.045	0.320	1.046	0.597	0.000
SVS	AAMDC	31	0.003	0.010	0.760	1.003	1.000	0.987
SVS	ABO	31	0.000	0.007	0.995	1.000	1.000	0.884
SVS	ACADM	31	-0.010	0.011	0.358	0.990	1.000	0.973
SVS	ACOX1	31	-0.008	0.011	0.503	0.993	1.000	0.982
SVS	ACRBP	31	0.000	0.012	0.980	1.000	1.000	0.781
SVS	ADAM23	31	0.017	0.012	0.138	1.018	1.000	0.190
SVS	ADAMTS1	31	0.008	0.011	0.498	1.008	1.000	0.987
SVS	ADAMTS13	31	0.002	0.014	0.885	1.002	1.000	0.056
SVS	ADAMTS15	31	0.016	0.013	0.240	1.016	1.000	0.296
SVS	ADAMTS16	31	0.000	0.010	0.986	1.000	1.000	0.865
SVS	ADAMTS4	31	0.001	0.012	0.934	1.001	1.000	0.713
SVS	ADAMTS8	31	-0.002	0.011	0.845	0.998	1.000	0.665
SVS	ADAMTSL5	31	-0.006	0.011	0.569	0.994	1.000	0.865
SVS	ADCYAP1R1	31	0.018	0.011	0.125	1.018	1.000	0.987
SVS	ADM	31	-0.006	0.011	0.577	0.994	1.000	0.581
SVS	AGRP	31	0.001	0.015	0.959	1.001	1.000	0.025
SVS	AMFR	31	0.008	0.011	0.460	1.008	1.000	0.987
SVS	AMOTL2	31	-0.004	0.012	0.748	0.996	1.000	0.581
SVS	ANKMY2	31	-0.001	0.011	0.945	0.999	1.000	0.987
SVS	AOC1	31	0.008	0.011	0.475	1.008	1.000	0.945

ST17; MR causal estimates for CAD and stroke on BP-associated proteins.

All estimates are from inverse variance weighted method (IVs >1) or Wald-ratio method (IV = 1). CAD, coronary artery disease; AS, all strokes; AIS, ischemic stroke; SVS, small vessel stroke; CES, cardioembolic stroke; LAS, large artery stroke

Exposure	Outocme	nsnp	Beta	SE	P-value	Odds ratio	FDR-corrected P-value	FDR-corrected Cochran's Q P-value
SVS	AOC3	31	-0.009	0.010	0.363	0.991	1.000	0.884
SVS	APOA1	31	0.010	0.010	0.332	1.010	1.000	0.987
SVS	APOA2	31	0.007	0.011	0.515	1.007	1.000	0.987
SVS	APOBR	31	0.008	0.008	0.325	1.008	1.000	0.824
SVS	APOC1	31	0.001	0.011	0.943	1.001	1.000	0.764
SVS	ARHGEF12	31	0.001	0.011	0.908	1.001	1.000	0.865
SVS	ARSB	31	0.000	0.011	0.983	1.000	1.000	0.987
SVS	ASPN	31	-0.013	0.012	0.242	0.987	1.000	0.665
SVS	ATXN2L	31	0.001	0.011	0.932	1.001	1.000	0.959
SVS	AXL	31	-0.016	0.013	0.237	0.984	1.000	0.237
SVS	B4GAT1	31	-0.006	0.010	0.548	0.994	1.000	0.987
SVS	BAG4	31	0.009	0.011	0.441	1.009	1.000	0.989
SVS	BCAM	31	0.011	0.010	0.295	1.011	1.000	0.987
SVS	BMP6	31	0.007	0.013	0.582	1.007	1.000	0.531
SVS	BNIP3L	31	0.004	0.011	0.708	1.004	1.000	0.987
SVS	BRAP	31	-0.003	0.012	0.804	0.997	1.000	0.581
SVS	BRSK2	31	0.001	0.010	0.907	1.001	1.000	0.884
SVS	CA12	31	0.003	0.012	0.829	1.003	1.000	0.660
SVS	CA9	31	-0.015	0.013	0.243	0.985	1.000	0.447
SVS	CACNB3	31	0.002	0.011	0.825	1.002	1.000	0.865
SVS	CALB2	31	0.014	0.012	0.259	1.014	1.000	0.581
SVS	CALCA	31	0.007	0.014	0.601	1.007	1.000	0.003
SVS	CALCOCO2	31	0.005	0.011	0.668	1.005	1.000	0.816
SVS	CCN3	31	0.011	0.011	0.310	1.011	1.000	0.665
SVS	CCND2	31	0.002	0.011	0.852	1.002	1.000	0.987
SVS	CD14	31	-0.020	0.014	0.157	0.980	1.000	0.083
SVS	CD164L2	31	0.010	0.011	0.384	1.010	1.000	0.987
SVS	CD46	31	-0.012	0.011	0.283	0.988	1.000	0.987
SVS	CD59	31	0.001	0.012	0.912	1.001	1.000	0.404
SVS	CEP170	31	0.014	0.011	0.210	1.014	1.000	0.987
SVS	CERT	31	-0.003	0.012	0.781	0.997	1.000	0.744
SVS	CES2	31	-0.009	0.012	0.492	0.992	1.000	0.531
SVS	CETN3	31	0.000	0.011	0.977	1.000	1.000	0.987
SVS	CFHR2	31	0.008	0.008	0.324	1.008	1.000	0.383
SVS	CFHR4	31	0.008	0.008	0.339	1.008	1.000	0.581
SVS	CHMP1A	31	0.004	0.011	0.750	1.004	1.000	0.987
SVS	CLIC5	31	0.000	0.012	0.991	1.000	1.000	0.790
SVS	CLMP	31	0.011	0.012	0.350	1.011	1.000	0.278
SVS	COL1A1	31	-0.007	0.014	0.605	0.993	1.000	0.080
SVS	COMP	31	-0.006	0.012	0.617	0.994	1.000	0.404
SVS	COMT	31	-0.015	0.011	0.164	0.985	1.000	0.987
SVS	CPTP	31	0.007	0.012	0.537	1.007	1.000	0.783
SVS	CPXM1	31	-0.009	0.011	0.393	0.991	1.000	0.987
SVS	CTF1	31	-0.009	0.011	0.425	0.991	1.000	0.987
SVS	CTSO	31	0.005	0.013	0.694	1.005	1.000	0.346
SVS	DAG1	31	-0.004	0.011	0.705	0.996	1.000	0.987
SVS	DARS1	31	0.001	0.011	0.934	1.001	1.000	0.884
SVS	DBN1	31	0.004	0.011	0.692	1.004	1.000	0.987
SVS	DDHD2	31	0.001	0.011	0.896	1.001	1.000	0.865
SVS	DENR	31	0.005	0.011	0.668	1.005	1.000	0.987
SVS	DNAJC9	31	0.013	0.011	0.222	1.013	1.000	0.988
SVS	DNER	31	0.001	0.013	0.935	1.001	1.000	0.111
SVS	DOK2	31	-0.005	0.011	0.663	0.995	1.000	0.982
SVS	DPEP1	31	0.002	0.008	0.836	1.002	1.000	0.980
SVS	DPP4	31	-0.007	0.011	0.530	0.993	1.000	0.897
SVS	DTX3	31	-0.004	0.011	0.721	0.996	1.000	0.532
SVS	DUSP13	31	0.014	0.012	0.269	1.014	1.000	0.531

ST17; MR causal estimates for CAD and stroke on BP-associated proteins.

All estimates are from inverse variance weighted method (IVs >1) or Wald-ratio method (IV = 1). CAD, coronary artery disease; AS, all strokes; AIS, ischemic stroke; SVS, small vessel stroke; CES, cardioembolic stroke; LAS, large artery stroke

Exposure	Outocme	nsnp	Beta	SE	P-value	Odds ratio	FDR-corrected P-value	FDR-corrected Cochran's Q P-value
SVS	DUSP29	31	0.019	0.015	0.199	1.019	1.000	0.083
SVS	EDN1	31	-0.001	0.015	0.925	0.999	1.000	0.040
SVS	EFEMP1	31	-0.007	0.013	0.588	0.993	1.000	0.122
SVS	EFNA1	31	0.013	0.014	0.344	1.013	1.000	0.080
SVS	EIF4G3	31	0.022	0.011	0.042	1.023	1.000	0.987
SVS	ELOA	31	0.016	0.011	0.127	1.017	1.000	0.973
SVS	ENPEP	31	-0.003	0.012	0.775	0.997	1.000	0.764
SVS	EPHA2	31	0.004	0.012	0.734	1.004	1.000	0.447
SVS	EPO	31	0.001	0.013	0.947	1.001	1.000	0.381
SVS	ERI1	31	-0.002	0.014	0.913	0.998	1.000	0.260
SVS	ERP29	31	-0.003	0.011	0.815	0.997	1.000	0.989
SVS	ESAM	31	-0.005	0.011	0.620	0.995	1.000	0.987
SVS	F12	31	0.000	0.009	0.978	1.000	1.000	0.462
SVS	F13B	31	0.015	0.017	0.382	1.015	1.000	0.001
SVS	FADD	31	-0.004	0.011	0.722	0.996	1.000	0.987
SVS	FDX1	31	-0.024	0.012	0.044	0.976	1.000	0.783
SVS	FES	31	-0.006	0.011	0.619	0.994	1.000	0.987
SVS	FGF12	31	-0.006	0.012	0.608	0.994	1.000	0.824
SVS	FGF2	31	-0.006	0.010	0.559	0.994	1.000	0.987
SVS	FGF20	31	0.022	0.012	0.073	1.023	1.000	0.618
SVS	FGF21	31	-0.026	0.013	0.039	0.974	1.000	0.498
SVS	FGF23	31	0.024	0.011	0.031	1.024	1.000	0.816
SVS	FGF5	31	-0.005	0.012	0.676	0.995	1.000	0.208
SVS	FKBP7	31	0.001	0.011	0.960	1.001	1.000	0.987
SVS	FN1	31	-0.007	0.013	0.578	0.993	1.000	0.278
SVS	FOXJ3	31	0.010	0.011	0.364	1.010	1.000	0.989
SVS	FOXO3	31	-0.002	0.011	0.840	0.998	1.000	0.987
SVS	FUCA1	31	-0.006	0.007	0.410	0.994	1.000	0.278
SVS	FURIN	31	-0.013	0.012	0.277	0.987	1.000	0.624
SVS	GCHFR	31	0.002	0.011	0.862	1.002	1.000	0.790
SVS	GFER	31	0.008	0.011	0.450	1.008	1.000	0.989
SVS	GHR	31	0.006	0.010	0.548	1.006	1.000	0.865
SVS	GHRHR	31	0.006	0.011	0.629	1.006	1.000	0.987
SVS	GHRL	31	-0.008	0.011	0.439	0.992	1.000	0.801
SVS	GIMAP7	31	0.023	0.011	0.037	1.023	1.000	0.759
SVS	GIT1	31	0.007	0.011	0.541	1.007	1.000	0.960
SVS	GLO1	31	0.000	0.011	0.996	1.000	1.000	0.987
SVS	GORASP2	31	-0.001	0.011	0.901	0.999	1.000	0.865
SVS	GRP	31	-0.001	0.014	0.938	0.999	1.000	0.083
SVS	GRPEL1	31	-0.004	0.011	0.693	0.996	1.000	0.824
SVS	GSTM4	31	-0.006	0.012	0.640	0.994	1.000	0.749
SVS	HADH	31	-0.008	0.013	0.538	0.992	1.000	0.618
SVS	HEXIM1	31	0.013	0.011	0.223	1.013	1.000	0.864
SVS	HHEX	31	0.000	0.011	0.991	1.000	1.000	0.987
SVS	HMOX2	31	0.004	0.013	0.763	1.004	1.000	0.531
SVS	HPGDS	31	0.013	0.014	0.343	1.014	1.000	0.056
SVS	HYAL1	31	-0.001	0.012	0.907	0.999	1.000	0.531
SVS	ICAM1	31	-0.010	0.011	0.390	0.990	1.000	0.584
SVS	ICAM2	31	-0.006	0.009	0.530	0.994	1.000	0.660
SVS	ICAM4	31	0.011	0.010	0.264	1.011	1.000	0.848
SVS	IDUA	31	-0.014	0.011	0.185	0.986	1.000	0.618
SVS	IFI30	31	-0.009	0.010	0.374	0.991	1.000	0.865
SVS	IFIT3	31	0.016	0.011	0.151	1.016	1.000	0.979
SVS	IFNGR2	31	-0.007	0.008	0.397	0.993	1.000	0.865
SVS	IGFBP3	31	0.010	0.010	0.305	1.010	1.000	0.884
SVS	IL1RL1	31	-0.019	0.009	0.041	0.981	1.000	0.531
SVS	IMMT	31	-0.005	0.011	0.630	0.995	1.000	0.987

ST17; MR causal estimates for CAD and stroke on BP-associated proteins.
All estimates are from inverse variance weighted method (IVs >1) or Wald-ratio method (IV = 1). CAD, coronary artery disease; AS, all strokes; AIS, ischemic stroke; SVS, small vessel stroke; CES, cardioembolic stroke; LAS, large artery stroke

Exposure	Outocme	nsnp	Beta	SE	P-value	Odds ratio	FDR-corrected P-value	FDR-corrected Cochran's Q P-value
SVS	IMPA1	31	0.002	0.011	0.869	1.002	1.000	0.987
SVS	ING1	31	-0.003	0.011	0.771	0.997	1.000	0.884
SVS	ITGAL	31	-0.012	0.011	0.286	0.988	1.000	0.865
SVS	ITIH1	31	0.011	0.011	0.329	1.011	1.000	0.865
SVS	KIF22	31	-0.007	0.011	0.534	0.993	1.000	0.989
SVS	KIFBP	31	0.004	0.011	0.697	1.004	1.000	0.987
SVS	LACRT	31	-0.011	0.011	0.323	0.989	1.000	0.987
SVS	LAYN	31	0.009	0.011	0.399	1.009	1.000	0.618
SVS	LMOD1	31	-0.005	0.012	0.668	0.995	1.000	0.447
SVS	LMOD1	31	-0.011	0.011	0.335	0.989	1.000	0.531
SVS	LMOD1	31	-0.005	0.010	0.641	0.995	1.000	0.960
SVS	LMOD1	31	0.005	0.010	0.588	1.005	1.000	0.884
SVS	LRIG1	31	0.015	0.012	0.223	1.015	1.000	0.056
SVS	LYAR	31	0.002	0.011	0.872	1.002	1.000	0.987
SVS	M6PR	31	0.007	0.011	0.549	1.007	1.000	0.987
SVS	MANEAL	31	0.030	0.011	0.008	1.031	1.000	0.987
SVS	MANSC4	31	0.013	0.011	0.237	1.014	1.000	0.278
SVS	MAP4K5	31	-0.002	0.011	0.886	0.998	1.000	0.987
SVS	MDH1	31	-0.001	0.011	0.942	0.999	1.000	0.996
SVS	MEGF9	31	0.009	0.014	0.520	1.009	1.000	0.125
SVS	MFGE8	31	0.002	0.012	0.843	1.002	1.000	0.397
SVS	MPHOSPH8	31	-0.011	0.013	0.412	0.989	1.000	0.379
SVS	MPI	31	-0.002	0.011	0.885	0.998	1.000	0.987
SVS	MPIG6B	31	-0.008	0.012	0.475	0.992	1.000	0.790
SVS	MSRA	31	0.000	0.011	1.000	1.000	1.000	0.979
SVS	MST1	31	0.004	0.007	0.585	1.004	1.000	0.531
SVS	MVK	31	-0.007	0.011	0.537	0.993	1.000	0.987
SVS	MXRA8	31	-0.019	0.012	0.133	0.982	1.000	0.379
SVS	NADK	31	0.003	0.012	0.834	1.003	1.000	0.531
SVS	NAGA	31	-0.018	0.011	0.101	0.982	1.000	0.884
SVS	NBN	31	0.019	0.011	0.078	1.019	1.000	0.897
SVS	NFE2	31	0.010	0.011	0.367	1.010	1.000	0.897
SVS	NFU1	31	-0.011	0.011	0.333	0.989	1.000	0.987
SVS	NGF	31	0.011	0.011	0.313	1.011	1.000	0.897
SVS	NGFR	31	0.001	0.012	0.929	1.001	1.000	0.594
SVS	NOMO1	31	-0.008	0.012	0.495	0.992	1.000	0.531
SVS	NOS3	31	0.003	0.016	0.852	1.003	1.000	0.019
SVS	NOTCH3	31	-0.016	0.013	0.211	0.984	1.000	0.174
SVS	NPPB	31	-0.012	0.011	0.282	0.988	1.000	0.987
SVS	NTRK3	31	0.004	0.013	0.741	1.004	1.000	0.278
SVS	NUCB2	31	0.005	0.011	0.661	1.005	1.000	0.987
SVS	NUDT5	31	-0.001	0.011	0.909	0.999	1.000	0.987
SVS	NUMB	31	0.004	0.011	0.705	1.004	1.000	0.987
SVS	OGA	31	-0.001	0.011	0.939	0.999	1.000	0.987
SVS	OPLAH	31	0.003	0.012	0.827	1.003	1.000	0.532
SVS	OTUD6B	31	0.003	0.011	0.802	1.003	1.000	0.897
SVS	PAM	31	0.007	0.011	0.531	1.007	1.000	0.531
SVS	PAMR1	31	0.008	0.014	0.548	1.008	1.000	0.051
SVS	PARP1	31	0.006	0.011	0.589	1.006	1.000	0.987
SVS	PCBP2	31	-0.005	0.011	0.678	0.995	1.000	0.987
SVS	PCOLCE	31	0.024	0.019	0.204	1.024	1.000	0.618
SVS	PCSK7	31	0.005	0.012	0.699	1.005	1.000	0.665
SVS	PDE5A	31	0.002	0.011	0.876	1.002	1.000	0.987
SVS	PDGFRA	31	-0.009	0.012	0.461	0.991	1.000	0.462
SVS	PDIA3	31	-0.003	0.011	0.763	0.997	1.000	0.996
SVS	PECAM1	31	0.004	0.010	0.717	1.004	1.000	0.884
SVS	PFKFB2	31	0.006	0.011	0.577	1.006	1.000	0.972

ST17; MR causal estimates for CAD and stroke on BP-associated proteins.

All estimates are from inverse variance weighted method (IVs >1) or Wald-ratio method (IV = 1). CAD, coronary artery disease; AS, all strokes; AIS, ischemic stroke; SVS, small vessel stroke; CES, cardioembolic stroke; LAS, large artery stroke

Exposure	Outocme	nsnp	Beta	SE	P-value	Odds ratio	FDR-corrected P-value	FDR-corrected Cochran's Q P-value
SVS	PGF	31	0.004	0.013	0.762	1.004	1.000	0.083
SVS	PHLDB1	31	0.005	0.011	0.673	1.005	1.000	0.987
SVS	PKD1	31	-0.008	0.010	0.452	0.993	1.000	0.984
SVS	PLA2G1B	31	0.006	0.012	0.586	1.006	1.000	0.624
SVS	PLXDC2	31	0.000	0.011	0.989	1.000	1.000	0.818
SVS	PMS1	31	0.026	0.011	0.023	1.026	1.000	0.984
SVS	PMVK	31	0.004	0.011	0.701	1.004	1.000	0.973
SVS	PPP1R14D	31	-0.003	0.015	0.831	0.997	1.000	0.058
SVS	PRDX1	31	-0.004	0.012	0.753	0.996	1.000	0.790
SVS	PRG2	31	-0.009	0.010	0.407	0.991	1.000	0.897
SVS	PRKAB1	31	-0.008	0.011	0.472	0.992	1.000	0.989
SVS	PROCR	31	-0.006	0.012	0.647	0.994	1.000	0.532
SVS	PRSS53	31	0.004	0.008	0.622	1.004	1.000	0.987
SVS	PRTFDC1	31	0.000	0.011	0.966	1.000	1.000	0.973
SVS	PSMD5	31	0.017	0.013	0.177	1.018	1.000	0.531
SVS	PSRC1	31	0.012	0.011	0.267	1.012	1.000	0.987
SVS	PTPRF	31	0.018	0.011	0.113	1.018	1.000	0.666
SVS	PTRHD1	31	-0.002	0.011	0.880	0.998	1.000	0.996
SVS	PYDC1	31	0.004	0.010	0.704	1.004	1.000	0.987
SVS	QPCT	31	0.002	0.015	0.886	1.002	1.000	0.040
SVS	RABEPK	31	0.012	0.011	0.286	1.012	1.000	0.989
SVS	RANBP1	31	0.007	0.011	0.503	1.007	1.000	0.989
SVS	RARRES1	31	0.006	0.011	0.559	1.006	1.000	0.987
SVS	RARRES2	31	-0.007	0.013	0.591	0.993	1.000	0.450
SVS	RELT	31	0.003	0.012	0.803	1.003	1.000	0.133
SVS	RSPO3	31	-0.002	0.012	0.884	0.998	1.000	0.495
SVS	SCARA5	31	-0.007	0.011	0.555	0.993	1.000	0.531
SVS	SDC1	31	-0.008	0.011	0.452	0.992	1.000	0.791
SVS	SDCCAG8	31	0.012	0.011	0.278	1.012	1.000	0.884
SVS	SDHB	31	-0.014	0.011	0.213	0.986	1.000	0.891
SVS	SELENOP	31	0.016	0.011	0.159	1.016	1.000	0.790
SVS	SEMA6C	31	0.009	0.012	0.461	1.009	1.000	0.618
SVS	SERPING1	31	0.011	0.017	0.521	1.011	1.000	0.001
SVS	SERPINI1	31	0.001	0.010	0.920	1.001	1.000	0.987
SVS	SH2B3	31	-0.003	0.011	0.809	0.997	1.000	0.884
SVS	SHMT1	31	-0.008	0.009	0.414	0.992	1.000	0.987
SVS	SIL1	31	-0.008	0.012	0.487	0.992	1.000	0.744
SVS	SLC16A1	31	-0.002	0.011	0.888	0.998	1.000	0.979
SVS	SLC39A14	31	0.008	0.011	0.450	1.009	1.000	0.960
SVS	SLC9A3R2	31	0.001	0.011	0.939	1.001	1.000	0.824
SVS	SMOC2	31	0.012	0.014	0.404	1.012	1.000	0.019
SVS	SMTN	31	0.009	0.012	0.422	1.009	1.000	0.716
SVS	SOST	31	0.020	0.011	0.069	1.021	1.000	0.581
SVS	SPINK8	31	-0.017	0.011	0.125	0.983	1.000	0.819
SVS	SPRED2	31	0.002	0.011	0.884	1.002	1.000	0.987
SVS	SPRING1	31	0.004	0.012	0.755	1.004	1.000	0.812
SVS	STC1	31	0.011	0.012	0.363	1.011	1.000	0.581
SVS	STX4	31	0.016	0.011	0.138	1.016	1.000	0.884
SVS	SYAP1	31	-0.004	0.011	0.745	0.996	1.000	0.987
SVS	TARBP2	31	0.008	0.011	0.488	1.008	1.000	0.987
SVS	TBC1D17	31	0.012	0.012	0.306	1.012	1.000	0.581
SVS	TBC1D23	31	0.004	0.011	0.707	1.004	1.000	0.979
SVS	TEK	31	-0.006	0.010	0.578	0.994	1.000	0.725
SVS	TGFB2	31	0.009	0.013	0.469	1.010	1.000	0.450
SVS	TIE1	31	-0.008	0.010	0.454	0.992	1.000	0.865
SVS	TJAP1	31	0.005	0.011	0.648	1.005	1.000	0.759
SVS	TMEM106A	31	-0.015	0.011	0.178	0.985	1.000	0.987

ST17; MR causal estimates for CAD and stroke on BP-associated proteins.

All estimates are from inverse variance weighted method (IVs >1) or Wald-ratio method (IV = 1). CAD, coronary artery disease; AS, all strokes; AIS, ischemic stroke; SVS, small vessel stroke; CES, cardioembolic stroke; LAS, large artery stroke

Exposure	Outocme	nsnp	Beta	SE	P-value	Odds ratio	FDR-corrected P-value	FDR-corrected Cochran's Q P-value
SVS	TNFRSF12A	31	-0.024	0.010	0.020	0.976	1.000	0.982
SVS	TNFRSF13B	31	0.020	0.013	0.104	1.021	1.000	0.427
SVS	TNFRSF17	31	-0.005	0.014	0.740	0.995	1.000	0.099
SVS	TNFSF12	31	0.008	0.014	0.578	1.008	1.000	0.089
SVS	TNFSF13B	31	-0.002	0.013	0.898	0.998	1.000	0.314
SVS	TP53	31	-0.014	0.011	0.223	0.986	1.000	0.884
SVS	TP53BP1	31	-0.006	0.011	0.585	0.994	1.000	0.987
SVS	TP53I3	31	-0.001	0.011	0.950	0.999	1.000	0.989
SVS	TP53INP1	31	0.002	0.013	0.876	1.002	1.000	0.560
SVS	TWF2	31	-0.004	0.011	0.703	0.996	1.000	0.987
SVS	UBE2L6	31	0.004	0.011	0.708	1.004	1.000	0.996
SVS	UMOD	31	-0.006	0.012	0.610	0.994	1.000	0.025
SVS	UXS1	31	0.005	0.013	0.672	1.006	1.000	0.447
SVS	VAT1	31	0.008	0.016	0.609	1.008	1.000	0.032
SVS	VSIG2	31	-0.008	0.011	0.439	0.992	1.000	0.824
SVS	WARS	31	-0.003	0.011	0.798	0.997	1.000	0.989
SVS	WASHC3	31	0.007	0.011	0.512	1.007	1.000	0.973
SVS	WNT9A	31	-0.020	0.010	0.043	0.980	1.000	0.989
SVS	WWP2	31	0.009	0.011	0.403	1.009	1.000	0.987
SVS	YAP1	31	0.000	0.015	0.977	1.000	1.000	0.011
SVS	YOD1	31	0.017	0.011	0.120	1.017	1.000	0.987
SVS	ZBTB17	31	0.004	0.013	0.757	1.004	1.000	0.285
SVS	ZFYVE19	31	-0.002	0.011	0.867	0.998	1.000	0.987

nsnp = number of single nucleotide polymorphisms; SE = standard error; Cochran's Q P-value = p-value from Cochran's Q test assessing heterogeneity; FDR=false discovery rate

ST18; Protein-Protein interactions and respective scores derived from the STRING v12.0 database.

Source	Target	Experimentally determined interaction	Database annotated	Automated textmining	Combined score
ADAMTS4	FN1	0.000	0.000	0.644	0.644
ADM	CALCA	0.000	0.900	0.000	0.900
AMOTL2	YAP1	0.631	0.500	0.144	0.828
APOA1	SDC1	0.000	0.500	0.000	0.499
APOA1	APOBR	0.000	0.540	0.000	0.540
APOA1	FN1	0.077	0.000	0.713	0.723
APOA1	APOC1	0.248	0.720	0.952	0.989
APOA1	APOA2	0.457	0.800	0.982	0.997
APOA2	SDC1	0.000	0.500	0.000	0.499
APOA2	APOBR	0.000	0.540	0.000	0.540
APOA2	APOC1	0.000	0.720	0.820	0.947
APOBR	APOC1	0.000	0.540	0.000	0.540
ASPN	TGFB2	0.000	0.500	0.000	0.499
ATXN2L	HEXIM1	0.457	0.000	0.000	0.457
ATXN2L	SLC9A3R2	0.451	0.000	0.000	0.451
AXL	MFGE8	0.000	0.000	0.454	0.454
BMP6	SOST	0.000	0.000	0.444	0.444
CA12	CA9	0.000	0.500	0.050	0.504
CD14	FADD	0.000	0.500	0.000	0.499
CFHR2	CFHR4	0.000	0.500	0.000	0.499
CLIC5	TWF2	0.000	0.500	0.000	0.499
COL1A1	ESAM	0.000	0.000	0.426	0.426
COL1A1	EFEMP1	0.000	0.000	0.432	0.432
COL1A1	IGFBP3	0.292	0.000	0.250	0.446
COL1A1	FN1	0.483	0.000	0.464	0.711
COMP	FN1	0.000	0.000	0.916	0.916
DAG1	FN1	0.000	0.000	0.410	0.410
DAG1	ITGAL	0.000	0.000	0.414	0.414
DOK2	KIFBP	0.587	0.000	0.000	0.587
DOK2	TEK	0.472	0.500	0.155	0.757
DPP4	FN1	0.000	0.000	0.969	0.969
EFNA1	EPHA2	0.960	0.500	0.983	0.999
FADD	TP53	0.000	0.000	0.539	0.539

ST18; Protein-Protein interactions and respective scores derived from the STRING v12.0 database.

Source	Target	Experimentally determined interaction	Database annotated	Automated textmining	Combined score
FGF2	PDGFRA	0.064	0.000	0.832	0.836
FGF2	FGF5	0.000	0.400	0.000	0.400
FGF2	TEK	0.064	0.000	0.424	0.438
FGF2	FN1	0.000	0.000	0.837	0.837
FGF2	SDC1	0.457	0.500	0.977	0.993
FGF5	FN1	0.000	0.000	0.415	0.414
FGF5	SDC1	0.000	0.000	0.632	0.632
FN1	PKD1	0.292	0.000	0.252	0.447
FN1	ICAM1	0.000	0.000	0.672	0.672
FN1	PGF	0.000	0.000	0.894	0.894
FN1	IGFBP3	0.457	0.000	0.866	0.924
FN1	SDC1	0.000	0.000	0.982	0.982
FOXO3	NOTCH3	0.000	0.000	0.419	0.418
FOXO3	TP53	0.542	0.000	0.947	0.974
GFER	RARRES2	0.000	0.000	0.468	0.468
GSTM4	HPGDS	0.000	0.500	0.000	0.499
ICAM1	MFGE8	0.000	0.000	0.701	0.701
ICAM1	ICAM2	0.000	0.900	0.887	0.988
ICAM1	ITGAL	0.960	0.900	0.982	0.999
ICAM2	TP53	0.000	0.000	0.432	0.432
ICAM2	ITGAL	0.000	0.900	0.982	0.998
ICAM4	ITGAL	0.000	0.500	0.605	0.794
ING1	TP53	0.457	0.000	0.200	0.547
LACRT	SDC1	0.457	0.000	0.601	0.774
M6PR	RABEPK	0.000	0.500	0.000	0.499
NBN	PARP1	0.292	0.500	0.000	0.630
NGF	NTRK3	0.000	0.000	0.982	0.982
NGF	TIE1	0.000	0.000	0.663	0.663
NOTCH3	WWP2	0.292	0.700	0.411	0.863
NUMB	TP53	0.457	0.000	0.323	0.616
OGA	TP53	0.594	0.000	0.000	0.594
PARP1	TP53	0.457	0.000	0.000	0.457
PARP1	WWP2	0.457	0.000	0.000	0.457

ST18; Protein-Protein interactions and respective scores derived from the STRING v12.0 database.

Source	Target	Experimentally determined interaction	Database annotated	Automated textmining	Combined score
PKD1	WNT9A	0.000	0.000	0.401	0.401
SHMT1	TNFSF12	0.000	0.000	0.604	0.604
SLC9A3R2	WWP2	0.502	0.000	0.000	0.502
SLC9A3R2	YAP1	0.457	0.000	0.000	0.457
TEK	TIE1	0.292	0.000	0.538	0.658
TNFRSF17	TNFSF13B	0.960	0.900	0.982	0.999
TNFSF12	TNFSF13B	0.292	0.500	0.410	0.772
TP53	ZBTB17	0.457	0.000	0.000	0.457
TP53	WWP2	0.164	0.000	0.409	0.484
YAP1	ZFYVE19	0.553	0.000	0.050	0.557

ST19; Bayesian colocalization analysis with BP for shared causal proteins between CVDs and BP.

Only protein-trait pairs prioritized in the MR analysis were included. PP, posterior probability; SBP, systolic blood pressure; DBP, diastolic blood pressure.

Outcome	Exposure	nsnps	PP.H0	PP.H1	PP.H2	PP.H3	PP.H4
SBP	ACOX1	6629	0.00%	1.88%	0.00%	18.77%	79.35%
SBP	ACRBP	6329	0.00%	38.76%	0.00%	47.32%	13.92%
SBP	APOA1	6917	0.00%	0.02%	0.00%	99.79%	0.20%
SBP	BAG4	4166	0.00%	0.00%	0.00%	11.75%	88.25%
SBP	BMP6	8012	0.00%	8.92%	0.00%	38.27%	52.81%
SBP	BRAP	3043	0.00%	0.00%	0.00%	0.89%	99.11%
SBP	CD164L2	941	0.00%	1.69%	0.00%	96.63%	1.68%
SBP	DAG1	2828	0.00%	0.00%	0.00%	98.16%	1.84%
SBP	DUSP13	3797	0.00%	0.00%	0.00%	100.00%	0.00%
SBP	DUSP29	3829	0.00%	0.00%	0.00%	100.00%	0.00%
SBP	EFEMP1	6971	0.00%	0.00%	0.00%	97.44%	2.56%
SBP	ERP29	3065	0.00%	0.00%	0.08%	1.59%	98.33%
SBP	FDX1	5568	0.00%	41.50%	0.00%	52.04%	6.46%
SBP	FES	6450	0.00%	0.00%	0.00%	99.98%	0.02%
SBP	FGF5	5484	0.00%	0.00%	0.00%	0.47%	99.53%
SBP	FURIN	6446	0.00%	0.00%	0.00%	2.14%	97.86%
SBP	HHEX	5712	0.00%	0.00%	0.07%	25.09%	74.83%
SBP	HMOX2	7339	0.00%	0.00%	0.00%	100.00%	0.00%
SBP	ITIH1	4213	0.00%	0.00%	0.00%	100.00%	0.00%
SBP	LMOD1	5863	0.00%	48.52%	0.00%	7.09%	44.39%
SBP	MANEAL	5525	0.00%	0.02%	0.35%	97.14%	2.49%
SBP	MST1	2815	0.00%	0.00%	0.00%	98.79%	1.21%
SBP	NADK	6601	0.00%	0.00%	0.00%	8.07%	91.93%
SBP	NFE2	5482	0.00%	0.00%	0.00%	100.00%	0.00%
SBP	NTRK3	7585	0.00%	26.87%	0.00%	71.65%	1.47%
SBP	PCBP2	5765	0.00%	0.00%	0.00%	100.00%	0.00%
SBP	PDE5A	7413	0.00%	4.09%	0.00%	83.85%	12.06%
SBP	PGF	4971	0.00%	0.00%	0.00%	99.91%	0.09%
SBP	PSMD5	4530	0.00%	1.39%	0.00%	1.22%	97.39%
SBP	RSPO3	3973	0.00%	0.00%	0.00%	100.00%	0.00%
SBP	SCARA5	6786	0.00%	75.84%	0.00%	21.77%	2.39%
SBP	SDHB	6042	0.00%	17.72%	0.00%	52.96%	29.33%
SBP	SH2B3	3070	0.00%	0.00%	0.00%	100.00%	0.00%
SBP	SPINK8	2511	0.00%	0.00%	0.00%	29.59%	70.41%

ST19; Bayesian colocalization analysis with BP for shared causal proteins between CVDs and BP.

Only protein-trait pairs prioritized in the MR analysis were included. PP, posterior probability; SBP, systolic blood pressure; DBP, diastolic blood pressure.

Outcome	Exposure	nsnps	PP.H0	PP.H1	PP.H2	PP.H3	PP.H4
SBP	TARBP2	5562	0.00%	0.00%	0.00%	100.00%	0.00%
SBP	TIE1	5034	0.00%	0.00%	0.00%	16.20%	83.80%
SBP	TJAP1	5404	0.00%	0.00%	0.00%	1.51%	98.49%
SBP	WARS	6140	0.00%	0.04%	0.00%	99.68%	0.28%
DBP	AAMDC	5598	0.00%	0.00%	0.00%	16.36%	83.64%
DBP	ABO	7729	0.00%	0.00%	0.00%	100.00%	0.00%
DBP	ACOX1	6629	0.00%	1.29%	0.00%	74.92%	23.80%
DBP	APOA1	6917	0.00%	17.09%	0.00%	59.85%	23.06%
DBP	BAG4	4166	0.00%	10.79%	0.00%	10.81%	78.39%
DBP	DAG1	2828	0.00%	0.00%	0.00%	100.00%	0.00%
DBP	DOK2	6706	0.00%	40.20%	0.00%	54.10%	5.70%
DBP	DUSP13	3797	0.00%	17.75%	0.00%	27.36%	54.89%
DBP	EPHA2	5942	0.00%	5.37%	0.00%	92.41%	2.21%
DBP	FDX1	5568	0.00%	47.20%	0.00%	26.72%	26.08%
DBP	FES	6450	0.00%	0.00%	0.00%	99.98%	0.02%
DBP	FGF5	5484	0.00%	0.00%	0.00%	0.32%	99.68%
DBP	FURIN	6446	0.00%	0.00%	0.00%	2.86%	97.14%
DBP	HHEX	5712	0.00%	0.39%	0.08%	26.36%	73.17%
DBP	LAYN	5059	0.00%	0.00%	0.00%	99.50%	0.50%
DBP	LMOD1	5863	0.00%	0.00%	0.00%	58.68%	41.32%
DBP	MST1	2815	0.00%	0.00%	0.00%	100.00%	0.00%
DBP	NADK	6601	0.00%	0.04%	0.00%	24.27%	75.69%
DBP	NOS3	7194	0.00%	0.00%	0.00%	86.97%	13.03%
DBP	PARP1	6197	0.00%	0.00%	0.00%	100.00%	0.00%
DBP	PDE5A	7413	0.00%	0.00%	0.00%	99.93%	0.07%
DBP	PROCR	3888	0.00%	59.12%	0.00%	18.44%	22.44%
DBP	SDHB	6042	0.00%	18.79%	0.00%	71.75%	9.46%
DBP	SH2B3	3070	0.00%	0.00%	0.00%	100.00%	0.00%
DBP	SPINK8	2511	0.00%	0.00%	0.00%	38.19%	61.81%
DBP	TIE1	5034	0.00%	0.00%	0.00%	95.18%	4.82%
DBP	TJAP1	5404	0.00%	0.00%	0.01%	99.92%	0.08%
DBP	TMEM106A	3898	0.00%	4.87%	0.00%	94.54%	0.59%
DBP	VAT1	3874	0.00%	4.86%	0.00%	94.27%	0.87%

ST20; Bayesian colocalization analysis with CVDs for 13 proteins shared between BP and CVDs.

Only protein-trait pairs prioritized in the MR analysis were included. PP, posterior probability; CAD, coronary artery disease; AS, all strokes; AIS, ischemic stroke; SVS, small vessel stroke.

Outcome	Exposure	nsnps	PP.H0	PP.H1	PP.H2	PP.H3	PP.H4
CAD	AAMDC	4842	0.00%	51.22%	0.00%	30.40%	18.38%
CAD	ACOX1	5509	0.00%	4.46%	0.00%	11.91%	83.63%
CAD	BRAP	2593	0.00%	0.00%	0.00%	0.26%	99.74%
CAD	ERP29	2698	0.00%	0.00%	0.01%	0.20%	99.79%
CAD	FGF5	4808	0.00%	0.00%	0.00%	0.23%	99.77%
CAD	FURIN	5573	0.00%	0.00%	0.00%	77.01%	22.99%
CAD	HHEX	5204	0.06%	21.55%	0.21%	72.73%	5.44%
CAD	NADK	3531	0.00%	0.50%	0.00%	99.28%	0.22%
CAD	SPINK8	2146	0.00%	0.01%	0.00%	53.51%	46.48%
CAD	TIE1	4575	0.00%	21.25%	0.00%	19.86%	58.89%
CAD	TJAP1	4831	0.00%	0.00%	0.01%	99.99%	0.00%
AS	BRAP	2211	0.00%	0.00%	0.00%	0.86%	99.14%
AS	ERP29	2386	0.00%	0.00%	0.07%	1.29%	98.64%
AS	FGF5	4284	0.00%	31.99%	0.00%	2.68%	65.33%
AS	FURIN	5174	0.00%	0.18%	0.00%	2.16%	97.66%
AS	TJAP1	4344	0.00%	9.70%	0.00%	29.98%	60.31%
AIS	BRAP	2210	0.00%	0.00%	0.00%	0.78%	99.21%
AIS	ERP29	2372	0.00%	0.00%	0.06%	1.20%	98.74%
AIS	FGF5	4270	0.00%	19.26%	0.00%	2.74%	78.00%
AIS	FURIN	5174	0.00%	0.02%	0.00%	2.92%	97.06%
AIS	PSMD5	3593	0.00%	76.10%	0.00%	12.52%	11.38%
AIS	TJAP1	4336	0.00%	31.48%	0.00%	24.07%	44.45%
SVS	BAG4	2949	0.00%	10.36%	0.01%	25.35%	64.27%
SVS	BRAP	1828	0.03%	12.28%	0.05%	19.23%	68.41%
SVS	ERP29	2028	0.66%	13.12%	1.03%	20.55%	64.64%

ST21; PairWise Conditional and Colocalisation (PWCoCo) for 22 BP-associated proteins with overlapped genomic windows.

The configuration with the largest PP.H4 was shown for each outcome-exposure pair. * indicates that the SNP was the only conditionally independent SNP found and was conditioned upon.

Outcome	Exposure	SNP from outcome	SNP from exposure	nsnps	PP.H0	PP.H1	PP.H2	PP.H3	PP.H4
SBP	FURIN	rs8027450	rs4932372	6003	0.00%	0.00%	0.00%	0.48%	99.52%
DBP	FURIN	rs2521501	rs4932372	6003	0.00%	0.00%	0.00%	0.68%	99.32%
DBP	BRAP	unconditioned	unconditioned	2682	0.00%	0.00%	0.00%	1.57%	98.43%
SBP	BRAP	rs3184504	unconditioned	2592	0.00%	0.00%	0.00%	1.64%	98.36%
DBP	ERP29	unconditioned	unconditioned	2854	0.00%	0.00%	0.07%	2.35%	97.58%
SBP	NADK	unconditioned	rs17162854	5835	0.00%	0.00%	0.00%	2.44%	97.56%
SBP	ERP29	rs3184504	unconditioned	2760	0.00%	0.08%	0.00%	2.45%	97.48%
SBP	PSMD5	unconditioned	unconditioned	4277	0.00%	1.81%	0.00%	1.75%	96.44%
DBP	NADK	unconditioned	rs17162854	5835	0.00%	0.02%	0.00%	10.13%	89.85%
SBP	BAG4	unconditioned	unconditioned	3805	0.00%	0.00%	0.00%	13.22%	86.78%
SBP	TIE1	unconditioned	unconditioned	4849	0.00%	0.00%	0.00%	16.59%	83.41%
DBP	BAG4	unconditioned	unconditioned	3805	0.00%	10.64%	0.00%	12.21%	77.14%
SBP	MST1	unconditioned	rs34484573	1458	0.00%	0.00%	0.00%	23.61%	76.39%
CAD		unconditioned	rs34484573	1225	0.00%	0.01%	0.00%	3.47%	96.52%
DBP	NOS3	rs3918226	rs1800783*	6810	0.00%	0.00%	23.99%	9.00%	67.01%
DBP	MST1	rs547649546*	rs67286839	1727	0.00%	2.26%	0.00%	42.89%	54.85%
DBP	DUSP13	unconditioned	unconditioned	3429	0.00%	17.23%	0.00%	28.91%	53.87%
CAD	FURIN	unconditioned	rs4932372	5109	0.00%	0.00%	0.00%	48.24%	51.76%
DBP	APOA1	unconditioned	rs2727784	6552	0.00%	13.80%	0.00%	48.70%	37.50%
SBP	FES	rs78861158	rs12903530	6091	0.00%	0.01%	0.07%	79.57%	20.35%
SBP	DUSP29	rs537008421*	rs11001272	3329	0.00%	58.85%	0.00%	31.69%	9.46%
SBP	APOA1	rs57767677	rs2727784	6470	0.00%	0.39%	0.00%	90.91%	8.70%
CAD	NADK	rs36096196*	rs17162854	3306	0.00%	28.13%	0.00%	65.48%	6.39%
DBP	DOK2	unconditioned	unconditioned	6290	0.00%	39.55%	0.00%	54.72%	5.72%
DBP	TIE1	unconditioned	unconditioned	4849	0.00%	0.00%	0.00%	95.76%	4.24%
SBP	DUSP13	rs140590393*	unconditioned	3309	0.00%	0.00%	84.06%	13.45%	2.49%
DBP	EPHA2	unconditioned	rs924204	5738	0.00%	5.17%	0.00%	92.63%	2.20%
SBP	NFE2	rs7134677*	rs60822569	5019	0.00%	82.96%	0.00%	14.89%	2.14%
DBP	VAT1	unconditioned	rs9911630	3615	0.00%	4.79%	0.00%	93.85%	1.37%
DBP	DAG1	rs5848856	rs6446277*	2281	1.44%	0.06%	93.53%	3.78%	1.19%
DBP	SH2B3	unconditioned	rs10849949*	2448	0.00%	0.00%	91.01%	7.85%	1.13%
SBP	SH2B3	rs3184504	rs10849949*	2442	0.00%	0.00%	91.02%	7.85%	1.13%
SBP	DAG1	rs547649546*	rs6446277*	2281	2.54%	0.10%	92.51%	3.74%	1.10%
SBP	PCBP2	rs7315980	rs10876550*	5312	0.00%	0.00%	85.22%	13.76%	1.01%
DBP	TMEM106A	unconditioned	rs6503726	3630	0.00%	4.80%	0.00%	94.22%	0.98%
SBP	TARBP2	rs7315980	rs10876550*	5123	0.00%	0.00%	80.50%	18.92%	0.59%
DBP	FES	rs78861158	rs60994696	6095	0.00%	0.03%	1.27%	98.27%	0.42%

ST22; Lookup of cis-pQTLs for the prioritised protein in publicly available eQTL databases.

pQTL: protein quantitative trait locus; SNP: single nucleotide polymorphism.

Portein	Gene Symbol	chr_position (build37)	SNP	P-value (eQTL)	Tissue	Trait
ACOX1	*ACOX1*	17:73951864	rs10852766	3.00E-12	Cells - Cultured fibroblasts	CAD
ACOX1	*ACOX1*	17:73951865	rs10852766	7.50E-11	Thyroid	CAD
ACOX1	*ACOX1*	17:73951866	rs10852766	3.10E-08	Esophagus - Gastroesophageal Junction	CAD
ACOX1	*ACOX1*	17:73951867	rs10852766	9.20E-08	Artery - Tibial	CAD
ACOX1	*ACOX1*	17:73951868	rs10852766	5.10E-07	Esophagus - Muscularis	CAD
ACOX1	*ACOX1*	17:73951869	rs10852766	0.0000011	Whole Blood	CAD
ACOX1	*ACOX1*	17:73951870	rs10852766	0.0000016	Artery - Aorta	CAD
ACOX1	*ACOX1*	17:73951871	rs10852766	0.000057	Nerve - Tibial	CAD
ACOX1	*ACOX1*	17:73951872	rs10852766	0.000086	Cells - EBV-transformed lymphocytes	CAD
MST1	*MST1*	3:49334768	rs3774800	0.000034	Liver	CAD
MST1	*MST1*	3:49705512	rs9823546	1.80E-30	Thyroid	CAD
MST1	*MST1*	3:49705512	rs9823546	2.50E-25	Skin - Not Sun Exposed (Suprapubic)	CAD
MST1	*MST1*	3:49705512	rs9823546	4.10E-25	Skin - Sun Exposed (Lower leg)	CAD
MST1	*MST1*	3:49705512	rs9823546	1.10E-21	Nerve - Tibial	CAD
MST1	*MST1*	3:49705512	rs9823546	1.30E-16	Esophagus - Mucosa	CAD
MST1	*MST1*	3:49705512	rs9823546	1.50E-12	Artery - Tibial	CAD
MST1	*MST1*	3:49705512	rs9823546	6.20E-10	Adipose - Subcutaneous	CAD
MST1	*MST1*	3:49705512	rs9823546	1.00E-09	Prostate	CAD
MST1	*MST1*	3:49705512	rs9823546	1.10E-09	Stomach	CAD
MST1	*MST1*	3:49705512	rs9823546	3.90E-09	Artery - Aorta	CAD
MST1	*MST1*	3:49705512	rs9823546	6.20E-09	Brain - Caudate (basal ganglia)	CAD
MST1	*MST1*	3:49705512	rs9823546	9.40E-09	Adipose - Visceral (Omentum)	CAD
MST1	*MST1*	3:49705512	rs9823546	9.70E-09	Brain - Hypothalamus	CAD
MST1	*MST1*	3:49705512	rs9823546	2.20E-08	Cells - Cultured fibroblasts	CAD
MST1	*MST1*	3:49705512	rs9823546	4.00E-08	Brain - Nucleus accumbens (basal ganglia)	CAD
MST1	*MST1*	3:49705512	rs9823546	5.70E-08	Brain - Hippocampus	CAD
MST1	*MST1*	3:49705512	rs9823546	3.10E-07	Brain - Cerebellum	CAD
MST1	*MST1*	3:49705512	rs9823546	4.50E-07	Brain - Putamen (basal ganglia)	CAD
MST1	*MST1*	3:49705512	rs9823546	7.50E-07	Heart - Atrial Appendage	CAD
MST1	*MST1*	3:49705512	rs9823546	0.0000012	Breast - Mammary Tissue	CAD
MST1	*MST1*	3:49705512	rs9823546	0.000002	Colon - Sigmoid	CAD
MST1	*MST1*	3:49705512	rs9823546	0.000002	Muscle - Skeletal	CAD
MST1	*MST1*	3:49705512	rs9823546	0.000004	Lung	CAD

ST22; Lookup of cis-pQTLs for the prioritised protein in publicly available eQTL databases.

pQTL: protein quantitative trait locus; SNP: single nucleotide polymorphism.

Portein	Gene Symbol	chr_position (build37)	SNP	P-value (eQTL)	Tissue	Trait
MST1	*MST1*	3:49705512	rs9823546	0.0000043	Brain - Cerebellar Hemisphere	CAD
MST1	*MST1*	3:49705512	rs9823546	0.0000071	Artery - Coronary	CAD
MST1	*MST1*	3:49705512	rs9823546	0.0000072	Esophagus - Muscularis	CAD
MST1	*MST1*	3:49705512	rs9823546	0.000021	Brain - Cortex	CAD
MST1	*MST1*	3:49705512	rs9823546	0.000035	Colon - Transverse	CAD
MST1	*MST1*	3:49705512	rs9823546	0.000041	Brain - Anterior qcingulate cortex (BA24)	CAD
MST1	*MST1*	3:49705512	rs9823546	0.000082	Heart - Left Ventricle	CAD
MST1	*MST1*	3:49705512	rs9823546	0.00018	Brain - Frontal Cortex (BA9)	CAD
FGF5	*FGF5*	4:81182554	rs12509595	4.40E-17	Kidney - Cortex	Stroke
FGF5	*FGF5*	4:81182554	rs12509595	0.000065	Brain - Cerebellar Hemisphere	Stroke
FURIN	*FURIN*	15:91407275	rs4932372	9.90E-27	Esophagus - Mucosa	Stroke
FURIN	*FURIN*	15:91407275	rs4932372	1.30E-07	Esophagus - Muscularis	Stroke
FURIN	*FURIN*	15:91407275	rs4932372	0.000002	Esophagus - Gastroesophageal Junction	Stroke
FURIN	*FURIN*	15:91407275	rs4932372	0.000025	Artery - Aorta	Stroke

ST23; Multi-trait colocalization analysis for shared causal proteins between CVDs and BP.

PP, posterior probability; CAD, coronary artery disease; AS, all strokes; AIS, ischemic stroke; SVS, small vessel stroke.

TraitB	TraitA	TraitC	nSNPs	Configuration	PP
SBP	ACOX1	CAD	5499	abc	72.03%
SBP	FGF5	CAD	4769	abc	95.24%
SBP	FGF5	AIS	4270	abc	21.66%
SBP	FGF5	AS	4284	abc	13.41%
SBP	FURIN	AS	5174	abc	79.22%
SBP	FURIN	AIS	5174	abc	49.19%
DBP	FGF5	CAD	4769	abc	96.65%
DBP	FGF5	AIS	4270	abc	21.88%
DBP	FGF5	AS	4284	abc	13.47%
DBP	FURIN	AS	5174	abc	81.47%
DBP	FURIN	AIS	5174	abc	51.00%

ST24; Genetic variants used in Mendelian randomization to explore the causal relation of the prioritized plasma proteins (ACOX1, FGF5, FURIN and MST1) with BP and CVDs.

SBP, systolic blood pressure; DBP, diastolic blood pressure; CAD, coronary artery disease; AS, all strokes; AIS, ischemic stroke.

								SNP's effect on exposure				SNP's effect on outcome				MR filters
Exposure	Outcome	SNP	Chromosome	Position	Effect allele	Other allele	Effect allele frequency	Beta	SE	P-value	Sample size	Beta	SE	P-value	Sample size	F-statistics
ACOX1	CAD	rs10852766	17	73951864	C	T	0.644	0.077	0.007	7.52E-32	54,219	0.023	0.006	0.0001371	296,525	136.56
ACOX1	DBP	rs10852766	17	73951864	C	T	0.644	0.077	0.007	7.52E-32	54,219	0.103	0.024	3.30E-05	410,170	136.56
ACOX1	SBP	rs10852766	17	73951864	C	T	0.644	0.077	0.007	7.52E-32	54,219	0.187	0.042	1.00E-05	410,170	136.56
FGF5	CAD	rs12509595	4	81182554	C	T	0.285	0.664	0.006	1.00E-300	54,219	0.047	0.006	3.55E-14	296,525	10660.20
FGF5	CAD	rs189447480	4	81238633	C	T	0.011	0.155	0.028	1.50E-08	54,219	-0.007	0.029	0.8159	296,525	30.71
FGF5	CAD	rs35087646	4	81126567	G	A	0.080	0.131	0.011	3.54E-34	54,219	-0.002	0.010	0.8393	296,525	147.20
FGF5	CAD	rs6814407	4	81058566	G	T	0.205	0.056	0.008	2.77E-13	54,219	0.008	0.008	0.2808	296,525	52.00
FGF5	CAD	rs76133235	4	82133746	A	G	0.037	-0.101	0.015	3.49E-11	54,219	0.005	0.017	0.7928	296,525	42.53
FGF5	DBP	rs12509595	4	81182554	C	T	0.285	0.664	0.006	1.00E-300	54,219	0.529	0.025	1.40E-99	410,170	10660.20
FGF5	DBP	rs189447480	4	81238633	C	T	0.011	0.155	0.028	1.50E-08	54,219	0.157	0.110	0.21	410,170	30.71
FGF5	DBP	rs2867769	4	81307677	A	G	0.089	0.085	0.011	9.86E-15	54,219	0.091	0.047	0.041	410,170	58.56
FGF5	DBP	rs35087646	4	81126567	G	A	0.080	0.131	0.011	3.54E-34	54,219	0.005	0.044	0.79	410,170	147.20
FGF5	DBP	rs538681538	4	81330231	A	T	0.012	0.168	0.030	1.54E-08	54,219	-0.070	0.117	0.72	410,170	30.66
FGF5	DBP	rs6814407	4	81058566	G	T	0.205	0.056	0.008	2.77E-13	54,219	0.022	0.031	0.44	410,170	52.00
FGF5	DBP	rs76133235	4	82133746	A	G	0.037	-0.101	0.015	3.49E-11	54,219	-0.019	0.059	0.74	410,170	42.53
FGF5	DBP	rs79966133	4	82019094	A	T	0.012	-0.288	0.027	1.34E-26	54,219	-0.228	0.107	0.027	410,170	112.57
FGF5	AIS	rs12509595	4	81182554	C	T	0.285	0.664	0.006	1.00E-300	54,219	0.028	0.008	0.0001808	1,296,908	10660.20
FGF5	AIS	rs35087646	4	81126567	G	A	0.080	0.131	0.011	3.54E-34	54,219	-0.006	0.012	0.6097	1,296,908	147.20
FGF5	AIS	rs6814407	4	81058566	G	T	0.205	0.056	0.008	2.77E-13	54,219	-0.003	0.010	0.801	1,296,908	52.00
FGF5	AIS	rs76133235	4	82133746	A	G	0.037	-0.101	0.015	3.49E-11	54,219	0.020	0.024	0.421	1,296,908	42.53
FGF5	SBP	rs12509595	4	81182554	C	T	0.285	0.664	0.006	1.00E-300	54,219	0.931	0.043	4.70E-103	410,170	10660.20
FGF5	SBP	rs189447480	4	81238633	C	T	0.011	0.155	0.028	1.50E-08	54,219	0.321	0.190	0.11	410,170	30.71
FGF5	SBP	rs2867769	4	81307677	A	G	0.089	0.085	0.011	9.86E-15	54,219	0.111	0.081	0.14	410,170	58.56
FGF5	SBP	rs35087646	4	81126567	G	A	0.080	0.131	0.011	3.54E-34	54,219	-0.043	0.076	0.57	410,170	147.20
FGF5	SBP	rs538681538	4	81330231	A	T	0.012	0.168	0.030	1.54E-08	54,219	-0.143	0.203	0.58	410,170	30.66
FGF5	SBP	rs6814407	4	81058566	G	T	0.205	0.056	0.008	2.77E-13	54,219	-0.033	0.053	0.61	410,170	52.00
FGF5	SBP	rs76133235	4	82133746	A	G	0.037	-0.101	0.015	3.49E-11	54,219	0.017	0.102	0.83	410,170	42.53
FGF5	SBP	rs79966133	4	82019094	A	T	0.012	-0.288	0.027	1.34E-26	54,219	-0.378	0.185	0.033	410,170	112.57
FURIN	AS	rs4932372	15	91407275	C	A	0.323	0.197	0.006	2.78E-204	54,219	0.035	0.007	9.67E-07	1,308,460	928.73
FURIN	DBP	rs4932372	15	91407275	C	A	0.323	0.197	0.006	2.78E-204	54,219	0.343	0.024	1.80E-46	410,170	928.73
FURIN	AIS	rs4932372	15	91407275	C	A	0.323	0.197	0.006	2.78E-204	54,219	0.043	0.008	6.48E-08	1,296,908	928.73
FURIN	SBP	rs4932372	15	91407275	C	A	0.323	0.197	0.006	2.78E-204	54,219	0.635	0.042	7.80E-53	410,170	928.73
MST1	CAD	rs3774800	3	49334768	A	G	0.646	-0.111	0.006	1.48E-65	54,219	-0.005	0.006	0.4405	296,525	291.04
MST1	CAD	rs9823546	3	49705512	A	T	0.289	-1.045	0.007	1.00E-300	54,219	-0.030	0.006	1.75E-06	296,525	23129.33
MST1	DBP	rs3774800	3	49334768	A	G	0.646	-0.111	0.006	1.48E-65	54,219	-0.020	0.024	0.43	410,170	291.04
MST1	DBP	rs9823546	3	49705512	A	T	0.289	-1.045	0.007	1.00E-300	54,219	-0.161	0.025	1.50E-10	410,170	23129.33
MST1	SBP	rs3774800	3	49334768	A	G	0.646	-0.111	0.006	1.48E-65	54,219	-0.116	0.041	0.0073	410,170	291.04
MST1	SBP	rs9823546	3	49705512	A	T	0.289	-1.045	0.007	1.00E-300	54,219	-0.258	0.043	9.40E-10	410,170	23129.33

ST25; MR causal estimates for blood pressure measures on CAD and stroke.

Outlier-corrected estimates refering to inverse variance weighted estimates calculated after removing outliers from MR-PRESSO. CAD, coronary artery disease; AS, all strokes; AIS, ischemic stroke; SVS, small vessel stroke; CES, cardioembolic stroke; LAS, large artery stroke

Outcome	Exposure	Method	nsnp	Beta	SE	P-value	Egger intercept P-value	Cochran's Q P-value	FDR-corrected P-value
CAD	SBP	MR Egger	396	0.028	0.006	0.000	0.106	0.000	0.000
AS	SBP	MR Egger	383	0.030	0.004	0.000	0.058	0.000	0.000
AIS	SBP	MR Egger	381	0.032	0.005	0.000	0.043	0.000	0.000
CES	SBP	MR Egger	375	0.022	0.009	0.020	0.883	0.000	0.020
LAS	SBP	MR Egger	363	0.049	0.012	0.000	0.781	0.000	0.000
SVS	SBP	MR Egger	359	0.043	0.012	0.000	0.135	0.000	0.000
CAD	SBP	Inverse variance weighted	396	0.037	0.002	0.000	0.106	0.000	0.000
AS	SBP	Inverse variance weighted	383	0.022	0.002	0.000	0.058	0.000	0.000
AIS	SBP	Inverse variance weighted	381	0.023	0.002	0.000	0.043	0.000	0.000
CES	SBP	Inverse variance weighted	375	0.021	0.003	0.000	0.883	0.000	0.000
LAS	SBP	Inverse variance weighted	363	0.046	0.004	0.000	0.781	0.000	0.000
SVS	SBP	Inverse variance weighted	359	0.026	0.004	0.000	0.135	0.000	0.000
CAD	SBP	Weighted median	396	0.033	0.002	0.000	0.106	-	0.000
AS	SBP	Weighted median	383	0.024	0.002	0.000	0.058	-	0.000
AIS	SBP	Weighted median	381	0.025	0.002	0.000	0.043	-	0.000
CES	SBP	Weighted median	375	0.022	0.005	0.000	0.883	-	0.000
LAS	SBP	Weighted median	363	0.040	0.006	0.000	0.781	-	0.000
SVS	SBP	Weighted median	359	0.025	0.006	0.000	0.135	-	0.000
CAD	DBP	MR Egger	388	0.073	0.010	0.000	0.126	0.000	0.000
AS	DBP	MR Egger	381	0.039	0.008	0.000	0.453	0.000	0.000
AIS	DBP	MR Egger	381	0.046	0.008	0.000	0.176	0.000	0.000
CES	DBP	MR Egger	375	0.009	0.016	0.566	0.228	0.000	0.566
LAS	DBP	MR Egger	364	0.053	0.023	0.019	0.466	0.000	0.027
SVS	DBP	MR Egger	363	0.050	0.022	0.023	0.926	0.000	0.027
CAD	DBP	Inverse variance weighted	388	0.059	0.004	0.000	0.126	0.000	0.000
AS	DBP	Inverse variance weighted	381	0.033	0.003	0.000	0.453	0.000	0.000
AIS	DBP	Inverse variance weighted	381	0.035	0.003	0.000	0.176	0.000	0.000
CES	DBP	Inverse variance weighted	375	0.028	0.006	0.000	0.228	0.000	0.000
LAS	DBP	Inverse variance weighted	364	0.038	0.008	0.000	0.466	0.000	0.000
SVS	DBP	Inverse variance weighted	363	0.048	0.007	0.000	0.926	0.000	0.000
CAD	DBP	Weighted median	388	0.057	0.003	0.000	0.126	-	0.000
AS	DBP	Weighted median	381	0.035	0.003	0.000	0.453	-	0.000
AIS	DBP	Weighted median	381	0.036	0.004	0.000	0.176	-	0.000
CES	DBP	Weighted median	375	0.033	0.008	0.000	0.228	-	0.000
LAS	DBP	Weighted median	364	0.037	0.010	0.000	0.466	-	0.000
SVS	DBP	Weighted median	363	0.046	0.010	0.000	0.926	-	0.000
CAD	DBP	Outlier-corrected	357	0.059	0.003	0.000	-	-	-
AS	DBP	Outlier-corrected	375	0.033	0.003	0.000	-	-	-
AIS	DBP	Outlier-corrected	376	0.035	0.003	0.000	-	-	-
CES	DBP	Outlier-corrected	374	-	-	-	-	-	-
LAS	DBP	Outlier-corrected	363	-	-	-	-	-	-
SVS	DBP	Outlier-corrected	362	0.047	0.007	0.000	-	-	-
CAD	SBP	Outlier-corrected	368	0.038	0.002	0.000	-	-	-
AS	SBP	Outlier-corrected	380	0.022	0.002	0.000	-	-	-
AIS	SBP	Outlier-corrected	377	0.022	0.002	0.000	-	-	-
CES	SBP	Outlier-corrected	374	0.022	0.003	0.000	-	-	-
LAS	SBP	Outlier-corrected	362	0.045	0.004	0.000	-	-	-
SVS	SBP	Outlier-corrected	358	-	-	-	-	-	-

ST26; Table showing components of mediation analysis and proportion mediated.

CAD, coronary artery disease; AS, all strokes; AIS, all ischemic stroke; SE, standard error; CI, confidence interval

Exposure	Outcome	Mediator	*β1*	*β2*	*βTE*	Indirect effect	SE	95% CI	Proportion Mediated (%)	95% CI of Proportion Mediated
ACOX1	***CAD***	SBP	2.43	0.04	0.30	0.090	0.021	0.049-0.131	30.5	9.5-51.4
FGF5			1.36	0.04	0.07	0.051	0.005	0.041-0.06	72.6	49.2-96.1
MST1			0.26	0.04	0.03	0.010	0.003	0.003-0.016	33.2	6.9-59.5
FURIN	***AS***		3.23	0.02	0.18	0.072	0.007	0.058-0.086	40	22.2-57.8
FGF5	***AIS***		1.36	0.02	0.04	0.031	0.003	0.025-0.038	77.2	31.9-100
FURIN			3.23	0.02	0.22	0.074	0.008	0.06-0.089	34.3	20.1-48.5
FGF5	***CAD***	DBP	0.78	0.06	0.07	0.046	0.004	0.039-0.054	66.5	46.1-86.8
FURIN	***AS***		1.75	0.03	0.18	0.058	0.006	0.046-0.071	32.6	17.8-47.5
FGF5	***AIS***		0.78	0.04	0.04	0.028	0.003	0.022-0.033	68.1	28.5-100
FURIN			1.75	0.04	0.22	0.062	0.007	0.048-0.075	28.5	16.5-40.6

ST27; Comparative analysis of protein levels in hypertensive individuals within UK Biobank separated by BP medication usage.

BP1_cases (taking BP medication); BP0_cases (not taking BP medication).

All participants

Protein	BP1_cases	BP0_cases	BP1_Mean	BP0_Mean	BP1_std	BP0_std	P-value	log2-fc	FDR-corrected P-value
ACOX1	6,222	12,313	0.823	0.860	1.181	1.208	0.043	-0.065	0.067
ACRBP	5,306	10,629	0.782	0.844	1.163	1.203	0.002	-0.110	0.004
APOA1	5,315	10,711	0.676	0.732	1.090	1.136	0.003	-0.115	0.006
CD164L2	5,190	10,377	0.803	0.796	1.175	1.175	0.719	0.013	0.788
DAG1	6,223	12,369	0.826	0.884	1.167	1.220	0.002	-0.098	0.004
DOK2	6,222	12,313	0.795	0.856	1.166	1.208	0.001	-0.106	0.003
DUSP13	5,306	10,629	0.751	0.778	1.178	1.149	0.174	-0.050	0.230
DUSP29	5,190	10,377	0.893	0.830	1.206	1.185	0.002	0.106	0.004
EFEMP1	6,219	12,376	1.084	0.923	1.441	1.273	0.000	0.232	0.000
EPHA2	5,990	11,860	1.052	0.919	1.471	1.290	0.000	0.194	0.000
FES	6,080	12,019	0.789	0.829	1.160	1.188	0.031	-0.071	0.050
FGF5	6,086	12,053	0.882	0.785	1.254	1.173	0.000	0.169	0.000
FURIN	6,211	12,370	0.891	1.060	1.180	1.315	0.000	-0.250	0.000
HMOX2	6,079	12,000	0.773	0.880	1.132	1.190	0.000	-0.186	0.000
ITIH1	5,315	10,711	0.792	0.821	1.146	1.186	0.136	-0.052	0.194
LAYN	6,139	12,176	1.020	0.855	1.470	1.266	0.000	0.255	0.000
LMOD1	5,307	10,651	0.883	0.882	1.278	1.222	0.997	0.000	0.997
MANEAL	5,148	10,315	0.794	0.810	1.174	1.182	0.418	-0.029	0.516
MST1	5,248	10,518	0.908	0.900	1.278	1.244	0.706	0.013	0.788
NADK	6,222	12,313	0.922	0.895	1.220	1.208	0.146	0.044	0.200
NFE2	5,166	10,400	0.800	0.857	1.154	1.196	0.005	-0.099	0.009
NOS3	6,079	12,000	0.960	0.962	1.231	1.255	0.908	-0.003	0.933
NTRK3	6,260	12,398	0.584	0.731	1.038	1.149	0.000	-0.324	0.000
PCBP2	5,307	10,651	0.791	0.856	1.160	1.202	0.001	-0.113	0.003
PDE5A	5,185	10,444	0.807	0.852	1.176	1.202	0.024	-0.079	0.041
PGF	6,276	12,441	1.155	0.975	1.461	1.293	0.000	0.244	0.000
PROCR	5,127	10,316	0.790	0.776	1.125	1.148	0.483	0.025	0.576
SCARA5	6,260	12,398	1.029	0.932	1.420	1.277	0.000	0.143	0.000
SDHB	5,312	10,641	0.715	0.846	1.070	1.197	0.000	-0.243	0.000
SH2B3	6,080	12,019	0.792	0.841	1.164	1.200	0.009	-0.086	0.016
SPINK8	5,127	10,316	0.795	0.802	1.146	1.176	0.724	-0.013	0.788
TARBP2	6,079	12,000	0.800	0.862	1.168	1.201	0.001	-0.106	0.003
TIE1	6,297	12,468	0.838	0.860	1.216	1.220	0.233	-0.038	0.298
TJAP1	6,211	12,370	0.785	0.854	1.161	1.199	0.000	-0.121	0.001
WARS	6,206	12,330	0.913	0.918	1.249	1.236	0.791	-0.008	0.837

Cholesterol & Diabetes medication removed

Protein	BP1_cases	BP0_cases	BP1_Mean	BP0_Mean	BP1_std	BP0_std	P-value	log2-fc	FDR-corrected P-value
ACOX1	2,375	11,409	0.851	0.864	1.198	1.209	0.619	-0.023	0.778
ACRBP	2,037	9,836	0.823	0.851	1.172	1.209	0.338	-0.048	0.544
APOA1	2,048	9,921	0.775	0.736	1.169	1.139	0.159	0.075	0.392
CD164L2	1,998	9,596	0.762	0.799	1.146	1.178	0.192	-0.070	0.418
DAG1	2,373	11,459	0.854	0.894	1.173	1.228	0.150	-0.065	0.392
DOK2	2,375	11,409	0.816	0.862	1.167	1.212	0.089	-0.080	0.281
DUSP13	2,037	9,836	0.762	0.776	1.184	1.142	0.623	-0.026	0.778
DUSP29	1,998	9,596	0.892	0.832	1.225	1.190	0.042	0.100	0.172
EFEMP1	2,375	11,464	1.057	0.928	1.382	1.276	0.000	0.189	0.000
EPHA2	2,286	10,973	1.000	0.925	1.399	1.292	0.013	0.113	0.067
FES	2,318	11,123	0.805	0.834	1.181	1.189	0.280	-0.052	0.511
FGF5	2,323	11,159	0.784	0.783	1.163	1.169	0.980	0.001	0.980
FURIN	2,366	11,447	0.919	1.072	1.209	1.323	0.000	-0.222	0.000
HMOX2	2,326	11,101	0.835	0.892	1.174	1.192	0.036	-0.095	0.166
ITIH1	2,048	9,921	0.772	0.816	1.134	1.182	0.127	-0.079	0.362
LAYN	2,343	11,272	0.907	0.858	1.363	1.270	0.091	0.081	0.281
LMOD1	2,036	9,856	0.892	0.890	1.235	1.227	0.945	0.003	0.972
MANEAL	1,976	9,550	0.800	0.814	1.185	1.185	0.631	-0.025	0.778
MST1	2,022	9,736	0.771	0.890	1.168	1.236	0.000	-0.207	0.001
NADK	2,375	11,409	0.919	0.901	1.228	1.210	0.517	0.028	0.709
NFE2	1,990	9,624	0.839	0.861	1.191	1.196	0.470	-0.036	0.668
NOS3	2,326	11,101	0.855	0.962	1.153	1.256	0.000	-0.169	0.001
NTRK3	2,391	11,477	0.735	0.746	1.135	1.161	0.674	-0.021	0.805
PCBP2	2,036	9,856	0.858	0.863	1.208	1.206	0.872	-0.008	0.949
PDE5A	1,999	9,660	0.825	0.857	1.178	1.208	0.276	-0.055	0.511
PGF	2,395	11,516	1.115	0.984	1.415	1.297	0.000	0.180	0.000
PROCR	1,975	9,548	0.799	0.773	1.140	1.150	0.372	0.047	0.574
SCARA5	2,391	11,477	0.931	0.937	1.303	1.281	0.840	-0.009	0.942
SDHB	2,042	9,851	0.767	0.858	1.108	1.206	0.002	-0.161	0.011
SH2B3	2,318	11,123	0.826	0.847	1.183	1.204	0.463	-0.035	0.668
SPINK8	1,975	9,548	0.768	0.798	1.121	1.176	0.290	-0.056	0.511
TARBP2	2,326	11,101	0.841	0.869	1.199	1.205	0.307	-0.047	0.516
TIE1	2,402	11,545	0.870	0.862	1.230	1.224	0.770	0.013	0.890
TJAP1	2,366	11,447	0.814	0.860	1.200	1.203	0.090	-0.079	0.281
WARS	2,362	11,408	0.887	0.924	1.218	1.240	0.185	-0.059	0.418

ST28; Effects of plasma proteins on BP in observational analysis using linear regression models.

"Number of observations" - individuals with protein measurements, covariates and not prevalent cases; "Number of cases" - incident cases of individuals with protein measurements; CAD, coronary artery disease; AS, all strokes; AIS, all ischemic stroke. Model 1 was adjusted for age and genetic sex; model 2 further adjusted for Townsend deprivation index, body mass index, and smoking status; model 3 further adjusted for LDL and HDL cholesterol, diabetes and medication for lipid or diabetes status.

Protein	Outcome	*Model 1*				*Model 2*				*Model 3*				
		Coefficient	lower CI	Upper CI	P-value	Coefficient	lower CI	Upper CI	P-value	Coefficient	lower CI	Upper CI	P-value	Number of observations
ACOX1	SBP	0.71	0.54	0.87	7.17E-17	0.51	0.35	0.68	7.74E-10	0.52	0.35	0.69	**4.66E-09**	41,626
FGF5	SBP	-0.18	-0.35	-0.01	3.45E-02	0.36	0.19	0.52	2.91E-05	0.30	0.12	0.48	**1.04E-03**	40,846
FURIN	SBP	2.39	2.23	2.56	2.50E-178	1.25	1.07	1.43	7.05E-42	1.31	1.11	1.51	**9.23E-39**	41,726
MST1	SBP	1.31	1.14	1.49	4.61E-47	1.09	0.92	1.27	1.03E-33	1.02	0.84	1.21	**1.70E-26**	35,616
ACOX1	DBP	0.45	0.35	0.54	3.94E-20	0.27	0.18	0.36	7.30E-09	0.25	0.15	0.35	**4.95E-07**	41,626
FGF5	DBP	-0.43	-0.53	-0.34	1.03E-18	0.04	-0.06	0.13	4.52E-01	0.05	-0.05	0.15	2.94E-01	40,846
FURIN	DBP	1.94	1.85	2.04	0.00E+00	0.90	0.80	1.01	2.54E-68	0.86	0.75	0.97	**4.82E-53**	41,726
MST1	DBP	0.89	0.78	0.99	3.66E-64	0.69	0.59	0.79	1.13E-41	0.60	0.49	0.70	**9.71E-29**	35,616

CI=confidence interval

ST29; Effects of plasma proteins on CAD and stroke in observational analysis using Cox proportional hazard models.

"Number of observations" - individuals with protein measurements, covariates and not prevalent cases; "Number of cases" - incident cases of individuals with protein measurements; CAD, coronary artery disease; AS, all strokes; AIS, all ischemic stroke. Model 1 was adjusted for age and genetic sex; model 2 further adjusted for Townsend deprivation index, body mass index, and smoking status; model 3 further adjusted for LDL and HDL cholesterol, diabetes and medication for lipid or diabetes status.

Protein	Outcome	***Model 1***				***Model 2***				***Model 3***					
		**Hazard ratio**	**lower CI**	**Upper CI**	**P-value**	**Hazard ratio**	**lower CI**	**Upper CI**	**P-value**	**Hazard ratio**	**lower CI**	**Upper CI**	**P-value**	**Number of observations**	**Number of cases**
ACOX1	**CAD**	1.04	1.01	1.07	4.76E-03	1.03	1.00	1.06	9.14E-02	1.02	0.99	1.05	2.47E-01	42,441	3,959
FGF5	**CAD**	1.06	1.03	1.09	2.93E-04	1.10	1.07	1.13	3.45E-10	1.10	1.06	1.13	**7.81E-09**	41,658	3,905
MST1	**CAD**	1.10	1.07	1.14	1.96E-09	1.07	1.04	1.10	2.11E-05	1.07	1.03	1.11	**9.20E-05**	36,414	3,353
FURIN	**AS**	1.20	1.13	1.27	5.19E-09	1.12	1.05	1.20	7.50E-04	1.09	1.02	1.17	**1.53E-02**	44,174	1,013
FGF5	**AIS**	1.10	1.03	1.17	2.32E-03	1.12	1.05	1.19	2.72E-04	1.10	1.03	1.17	**6.30E-03**	43,401	914
FURIN	**AIS**	1.27	1.19	1.35	1.33E-13	1.20	1.12	1.28	3.75E-07	1.19	1.10	1.28	**5.85E-06**	44,351	930

CI=confidence interval

ST30; Effects of plasma proteins on composite CVD (CAD and stroke) in observational analysis using Cox proportional hazard models.

"Number of observations" - individuals with protein measurements, covariates and not prevalent cases; "Number of cases" - incident cases of individuals with protein measurements; CAD, coronary artery disease; AS, all strokes; AIS, all ischemic stroke. Model 1 was adjusted for age and genetic sex; model 2 further adjusted for Townsend deprivation index, body mass index, and smoking status; model 3 further adjusted for LDL and HDL cholesterol, diabetes and medication for lipid or diabetes status.

Protein	Outcome	***Model 1***				***Model 2***				***Model 3***					
		**Hazard ratio**	**lower CI**	**Upper CI**	**P-value**	**Hazard ratio**	**lower CI**	**Upper CI**	**P-value**	**Hazard ratio**	**lower CI**	**Upper CI**	**P-value**	**Number of observations**	**Number of cases**
ACOX1	**CAD + Stroke**	1.05	1.02	1.08	7.76E-04	1.03	1.00	1.06	3.23E-02	1.02	0.99	1.05	1.83E-01	42,160	4,615
FGF5		1.05	1.02	1.08	3.08E-04	1.09	1.06	1.12	5.16E-10	1.09	1.06	1.12	**1.93E-08**	41,378	4,531
FURIN		1.29	1.26	1.33	1.36E-71	1.17	1.14	1.21	1.83E-24	1.14	1.11	1.18	**4.57E-15**	42,285	4,638
MST1		1.10	1.07	1.13	2.75E-10	1.07	1.04	1.10	6.57E-06	1.07	1.04	1.10	**2.51E-05**	36,160	3,921

CI=confidence interval

ST31; PheWAS results for the lead cis-pQTLs of the 4 proteins prioritized (ACOX1, FGF5, FURIN, and MST1).

SNP = Single Nucleotide Polymorphism; SE = Standard error HWE_p = P-value for Hardy-Weinberg equilibrium test

Phenotype	Description	Group	SNP	Beta	SE	Odds tatio	P-value	Type	n_total	n_cases	n_controls	HWE_p	allele_freq
401.1	Essential hypertension	circulatory system	rs4932372	0.061	0.006	1.062	2.144E-25	logistic	389876	99213	290663	0.994	0.331
401	Hypertension	circulatory system	rs4932372	0.060	0.006	1.062	3.295E-25	logistic	390097	99434	290663	0.994	0.331
411	Ischemic Heart Disease	circulatory system	rs4932372	0.057	0.008	1.059	1.468E-12	logistic	410606	40056	370550	0.999	0.331
411.8	Other chronic ischemic heart disease, unspecified	circulatory system	rs4932372	0.071	0.011	1.073	7.220E-10	logistic	389207	18657	370550	0.998	0.331
411.2	Myocardial infarction	circulatory system	rs4932372	0.075	0.013	1.078	4.457E-09	logistic	385085	14535	370550	0.997	0.331
411.4	Coronary atherosclerosis	circulatory system	rs4932372	0.060	0.010	1.061	5.735E-09	logistic	394337	23787	370550	0.999	0.331
411.3	Angina pectoris	circulatory system	rs4932372	0.063	0.012	1.065	1.658E-07	logistic	387093	16543	370550	0.996	0.330
272.1	Hyperlipidemia	endocrine/metabolic	rs4932372	0.035	0.008	1.035	2.364E-05	logistic	394381	38616	355765	0.996	0.331
272	Disorders of lipoid metabolism	endocrine/metabolic	rs4932372	0.034	0.008	1.035	3.173E-05	logistic	394625	38860	355765	0.996	0.331
272.11	Hypercholesterolemia	endocrine/metabolic	rs4932372	0.034	0.009	1.034	8.616E-05	logistic	390444	34679	355765	0.996	0.331
426.24	Atrioventricular block, complete	circulatory system	rs4932372	0.168	0.047	1.183	3.154E-04	logistic	372833	985	371848	0.999	0.331
378	Strabismus and other disorders of binocular eye movements	sense organs	rs4932372	-0.170	0.050	0.844	7.190E-04	logistic	412884	956	411928	0.994	0.331
740	Osteoarthrosis	musculoskeletal	rs4932372	-0.025	0.008	0.975	7.771E-04	logistic	392374	46516	345858	0.999	0.331
293	Symptoms involving head and neck	mental disorders	rs4932372	-0.231	0.073	0.794	1.512E-03	logistic	420175	464	419711	0.997	0.331
740.1	Osteoarthritis; localized	musculoskeletal	rs4932372	-0.029	0.009	0.971	2.087E-03	logistic	374099	28241	345858	0.996	0.331
454	Varicose veins	circulatory system	rs4932372	-0.066	0.022	0.936	2.677E-03	logistic	385827	4861	380966	0.989	0.331
454.1	Varicose veins of lower extremity	circulatory system	rs4932372	-0.068	0.023	0.935	2.936E-03	logistic	385486	4520	380966	0.989	0.331
476	Allergic rhinitis	respiratory	rs4932372	0.133	0.045	1.142	3.207E-03	logistic	397580	1063	396517	0.994	0.331
618.2	Uterine/Uterovaginal prolapse	genitourinary	rs4932372	-0.154	0.052	0.858	3.276E-03	logistic	214329	874	213455	0.967	0.331
411.1	Unstable angina (intermediate coronary syndrome)	circulatory system	rs4932372	0.072	0.025	1.075	3.286E-03	logistic	374269	3719	370550	0.996	0.330
198.2	Secondary malignancy of respiratory organs	neoplasms	rs4932372	-0.063	0.022	0.939	4.380E-03	logistic	390707	4701	386006	0.997	0.331
198	Secondary malignant neoplasm	neoplasms	rs4932372	-0.033	0.012	0.968	5.037E-03	logistic	403287	17281	386006	0.999	0.331
720.1	Spinal stenosis of lumbar region	musculoskeletal	rs4932372	0.081	0.029	1.085	5.237E-03	logistic	411268	2598	408670	0.997	0.331
496.1	Emphysema	respiratory	rs4932372	-0.078	0.028	0.925	5.356E-03	logistic	401774	2965	398809	0.996	0.331
286.12	Congenital deficiency of other clotting factors (including factor VII	hematopoietic	rs4932372	0.261	0.094	1.298	5.704E-03	logistic	417788	235	417553	0.999	0.331
366	Cataract	sense organs	rs4932372	-0.024	0.009	0.977	6.448E-03	logistic	406937	35259	371678	0.993	0.331
496	Chronic airway obstruction	respiratory	rs4932372	-0.032	0.012	0.969	6.540E-03	logistic	416701	17892	398809	0.997	0.331
733	Other disorders of bone and cartilage	musculoskeletal	rs4932372	-0.138	0.051	0.871	6.664E-03	logistic	399585	913	398672	0.990	0.331
165	Cancer within the respiratory system	neoplasms	rs4932372	-0.056	0.021	0.946	7.567E-03	logistic	422554	5325	417229	0.998	0.331
428	Congestive heart failure; nonhypertensive	circulatory system	rs4932372	0.044	0.017	1.045	8.395E-03	logistic	417859	8156	409703	0.998	0.331
916	Contusion	injuries & poisonings	rs4932372	-0.104	0.041	0.901	1.113E-02	logistic	421588	1393	420195	0.998	0.331
613	Other nonmalignant breast conditions	genitourinary	rs4932372	-0.196	0.078	0.822	1.229E-02	logistic	421594	397	421197	0.997	0.331
433.2	Occlusion of cerebral arteries	circulatory system	rs4932372	0.059	0.024	1.061	1.401E-02	logistic	406563	3878	402685	0.998	0.331
618	Genital prolapse	genitourinary	rs4932372	-0.047	0.019	0.954	1.536E-02	logistic	219680	6225	213455	0.952	0.331
378.1	Strabismus (not specified as paralytic)	sense organs	rs4932372	-0.232	0.096	0.793	1.538E-02	logistic	412196	268	411928	0.996	0.331
426	Cardiac conduction disorders	circulatory system	rs4932372	0.036	0.015	1.037	1.554E-02	logistic	382540	10692	371848	0.998	0.331
261	Vitamin deficiency	endocrine/metabolic	rs4932372	-0.059	0.024	0.943	1.582E-02	logistic	416032	3935	412097	0.996	0.331
244.1	Secondary hypothyroidism	endocrine/metabolic	rs4932372	-0.106	0.044	0.900	1.615E-02	logistic	393443	1213	392230	0.999	0.331
202	Cancer of other lymphoid, histiocytic tissue	neoplasms	rs4932372	-0.071	0.030	0.931	1.623E-02	logistic	418277	2664	415613	0.997	0.331
202.2	Non-Hodgkins lymphoma	neoplasms	rs4932372	-0.071	0.030	0.931	1.623E-02	logistic	418277	2664	415613	0.997	0.331
217	Vascular hamartomas and non-neoplastic nevi	neoplasms	rs4932372	-0.217	0.090	0.805	1.625E-02	logistic	414259	301	413958	0.999	0.331
430.2	Intracerebral hemorrhage	circulatory system	rs4932372	-0.110	0.046	0.896	1.829E-02	logistic	403773	1088	402685	0.999	0.331
433.21	Cerebral artery occlusion, with cerebral infarction	circulatory system	rs4932372	0.057	0.024	1.059	1.831E-02	logistic	406495	3810	402685	0.998	0.331
428.2	Heart failure NOS	circulatory system	rs4932372	0.050	0.021	1.052	1.871E-02	logistic	414622	4919	409703	0.997	0.331
369.5	Conjunctivitis, infectious	sense organs	rs4932372	-0.258	0.110	0.772	1.875E-02	logistic	410999	206	410793	0.996	0.331
364	Corneal opacity and other disorders of cornea	sense organs	rs4932372	-0.085	0.036	0.918	1.939E-02	logistic	399513	1750	397763	0.998	0.331
214.1	Lipoma of skin and subcutaneous tissue	neoplasms	rs4932372	-0.133	0.057	0.876	1.973E-02	logistic	414958	731	414227	0.997	0.331
217.1	Nevus, non-neoplastic	neoplasms	rs4932372	-0.219	0.094	0.803	2.018E-02	logistic	414233	275	413958	0.999	0.331
586.2	Cyst of kidney, acquired	genitourinary	rs4932372	0.080	0.034	1.083	2.026E-02	logistic	382916	1854	381062	1.000	0.331
599.4	Urinary incontinence	genitourinary	rs4932372	0.065	0.029	1.067	2.297E-02	logistic	392806	2700	390106	0.996	0.331
698	Pruritus and related conditions	dermatologic	rs4932372	-0.211	0.094	0.810	2.418E-02	logistic	422717	278	422439	0.998	0.331
555	Inflammatory bowel disease and other gastroenteritis and coliti	digestive	rs4932372	0.049	0.022	1.050	2.451E-02	logistic	339234	4749	334485	0.999	0.332
165.1	Cancer of bronchus; lung	neoplasms	rs4932372	-0.052	0.023	0.949	2.626E-02	logistic	421452	4223	417229	0.998	0.331
286.1	Congenital coagulation defects	hematopoietic	rs4932372	0.158	0.071	1.171	2.666E-02	logistic	417976	423	417553	0.999	0.331
244	Hypothyroidism	endocrine/metabolic	rs4932372	-0.025	0.011	0.975	2.697E-02	logistic	411296	19066	392230	0.999	0.331
596.5	Functional disorders of bladder	genitourinary	rs4932372	-0.134	0.061	0.875	2.765E-02	logistic	402194	640	401554	0.995	0.331
819	Skull and face fracture and other intercranial injury	injuries & poisonings	rs4932372	-0.151	0.069	0.860	2.848E-02	logistic	422666	501	422165	0.998	0.331
783	Fever of unknown origin	symptoms	rs4932372	-0.068	0.031	0.934	2.909E-02	logistic	418176	2397	415779	0.999	0.331
634	Miscarriage; stillbirth	pregnancy complications	rs4932372	-0.128	0.059	0.880	2.917E-02	logistic	223299	707	222592	0.987	0.331
252.2	Hypoparathyroidism	endocrine/metabolic	rs4932372	0.209	0.097	1.232	3.114E-02	logistic	415471	225	415246	0.998	0.331
426.2	Atrioventricular [AV] block	circulatory system	rs4932372	0.052	0.025	1.054	3.301E-02	logistic	375580	3732	371848	0.999	0.331
763	Thoracic or lumbosacral neuritis or radiculitis, unspecified	symptoms	rs4932372	0.184	0.087	1.202	3.381E-02	logistic	418269	283	417986	0.998	0.331
170	Cancer of bone and connective tissue	neoplasms	rs4932372	0.164	0.077	1.178	3.418E-02	logistic	423460	359	423101	0.998	0.331
70.4	Chronic hepatitis	infectious diseases	rs4932372	-0.194	0.092	0.824	3.481E-02	logistic	415491	287	415204	0.999	0.331
722	Intervertebral disc disorders	musculoskeletal	rs4932372	-0.125	0.060	0.882	3.577E-02	logistic	409333	663	408670	0.996	0.331
270.33	Amyloidosis	endocrine/metabolic	rs4932372	0.169	0.080	1.184	3.603E-02	logistic	422032	330	421702	0.999	0.331
426.32	Left bundle branch block	circulatory system	rs4932372	0.057	0.027	1.058	3.608E-02	logistic	374914	3066	371848	0.999	0.331
480	Pneumonia	respiratory	rs4932372	-0.026	0.012	0.975	3.773E-02	logistic	412462	15662	396800	0.998	0.331
594.1	Calculus of kidney	genitourinary	rs4932372	-0.056	0.027	0.946	3.798E-02	logistic	415555	3179	412376	0.997	0.331
447.1	Stricture of artery	circulatory system	rs4932372	0.122	0.059	1.130	4.037E-02	logistic	406083	614	405469	0.992	0.331
281.11	Pernicious anemia	hematopoietic	rs4932372	-0.115	0.056	0.891	4.168E-02	logistic	383186	737	382449	0.997	0.331

ST31; PheWAS results for the lead cis-pQTLs of the 4 proteins prioritized (ACOX1, FGF5, FURIN, and MST1).

SNP = Single Nucleotide Polymorphism; SE = Standard error HWE_p = P-value for Hardy-Weinberg equilibrium test

Phenotype	Description	Group	SNP	Beta	SE	Odds tatio	P-value	Type	n_total	n_cases	n_controls	HWE_p	allele_freq
728.71	Contracture of palmar fascia [Dupuytren's disease]	musculoskeletal	rs4932372	-0.075	0.037	0.928	4.212E-02	logistic	390879	1705	389174	0.985	0.331
444.1	Arterial embolism and thrombosis of lower extremity artery	circulatory system	rs4932372	0.141	0.070	1.152	4.257E-02	logistic	405915	446	405469	0.991	0.331
242.1	Graves' disease	endocrine/metabolic	rs4932372	0.154	0.076	1.167	4.293E-02	logistic	392598	368	392230	0.999	0.332
608	Other disorders of male genital organs	genitourinary	rs4932372	0.108	0.054	1.115	4.388E-02	logistic	162533	755	161778	0.754	0.332
153	Colorectal cancer	neoplasms	rs4932372	-0.037	0.018	0.964	4.452E-02	logistic	377768	7048	370720	0.998	0.332
458.1	Orthostatic hypotension	circulatory system	rs4932372	0.054	0.027	1.055	4.481E-02	logistic	375723	3151	372572	0.996	0.331
695.42	Systemic lupus erythematosus	dermatologic	rs4932372	-0.154	0.077	0.857	4.511E-02	logistic	403885	403	403482	0.995	0.331
587	Kidney replaced by transpant	genitourinary	rs4932372	0.132	0.066	1.141	4.567E-02	logistic	381556	494	381062	0.999	0.331
369	Infection of the eye	sense organs	rs4932372	-0.150	0.076	0.861	4.724E-02	logistic	411210	417	410793	0.996	0.331
287.31	Primary thrombocytopenia	hematopoietic	rs4932372	-0.133	0.067	0.875	4.840E-02	logistic	418072	519	417553	0.999	0.331
740.9	Osteoarthrosis NOS	musculoskeletal	rs4932372	-0.024	0.012	0.976	4.886E-02	logistic	361594	15736	345858	1.000	0.332
296	Mood disorders	mental disorders	rs4932372	-0.018	0.009	0.982	4.902E-02	logistic	409186	27564	381622	0.993	0.331
443.9	Peripheral vascular disease, unspecified	circulatory system	rs4932372	0.049	0.025	1.051	4.909E-02	logistic	409046	3577	405469	0.995	0.331
153.2	Colon cancer	neoplasms	rs4932372	-0.044	0.023	0.957	4.914E-02	logistic	375260	4540	370720	0.998	0.332
296.1	Bipolar	mental disorders	rs4932372	-0.088	0.045	0.916	5.026E-02	logistic	382786	1164	381622	0.981	0.331
79.9	Viremia, NOS	infectious diseases	rs4932372	-0.123	0.063	0.884	5.070E-02	logistic	415798	594	415204	0.999	0.331
745	Pain in joint	musculoskeletal	rs4932372	0.049	0.025	1.051	5.076E-02	logistic	414363	3501	410862	0.998	0.331
386.9	Dizziness and giddiness (Light-headedness and vertigo)	sense organs	rs4932372	0.053	0.027	1.054	5.156E-02	logistic	412711	3046	409665	0.997	0.331
586	Other disorders of the kidney and ureters	genitourinary	rs4932372	0.047	0.024	1.048	5.196E-02	logistic	384904	3842	381062	1.000	0.331
317	Alcohol-related disorders	mental disorders	rs4932372	-0.042	0.021	0.959	5.211E-02	logistic	408346	5053	403293	0.995	0.331
622.1	Polyp of corpus uteri	genitourinary	rs4932372	0.072	0.037	1.074	5.353E-02	logistic	215181	1609	213572	0.967	0.331
555.2	Ulcerative colitis	digestive	rs4932372	0.051	0.026	1.052	5.502E-02	logistic	337679	3194	334485	0.999	0.332
288.1	Decreased white blood cell count	hematopoietic	rs4932372	-0.051	0.027	0.950	5.590E-02	logistic	411502	3193	408309	0.997	0.331
288.11	Neutropenia	hematopoietic	rs4932372	-0.051	0.027	0.950	5.590E-02	logistic	411502	3193	408309	0.997	0.331
695.4	Lupus (localized and systemic)	dermatologic	rs4932372	-0.132	0.069	0.876	5.596E-02	logistic	403975	493	403482	0.995	0.331
447	Other disorders of arteries and arterioles	circulatory system	rs4932372	0.086	0.045	1.090	5.613E-02	logistic	406558	1089	405469	0.991	0.331
261.2	Vitamin B-complex deficiencies	endocrine/metabolic	rs4932372	-0.070	0.037	0.933	5.774E-02	logistic	413809	1712	412097	0.994	0.331
480.11	Pneumococcal pneumonia	respiratory	rs4932372	-0.030	0.016	0.971	5.807E-02	logistic	406463	9663	396800	0.996	0.331
444	Arterial embolism and thrombosis	circulatory system	rs4932372	0.102	0.054	1.108	5.870E-02	logistic	406215	746	405469	0.990	0.331
559	Ileostomy status	digestive	rs4932372	0.065	0.035	1.067	6.368E-02	logistic	336295	1810	334485	0.999	0.331
720	Spinal stenosis	musculoskeletal	rs4932372	0.043	0.023	1.044	6.531E-02	logistic	412805	4135	408670	0.998	0.331
296.2	Depression	mental disorders	rs4932372	-0.017	0.009	0.983	6.542E-02	logistic	408593	26971	381622	0.993	0.331
198.3	Secondary malignant neoplasm of digestive systems	neoplasms	rs4932372	-0.053	0.029	0.948	6.633E-02	logistic	388741	2735	386006	0.996	0.331
334.2	Anterior horn cell disease	neurological	rs4932372	0.127	0.069	1.135	6.691E-02	logistic	399497	450	399047	0.998	0.331
184.2	Cancer of other female genital organs (excluding uterus and ovary	neoplasms	rs4932372	-0.181	0.099	0.834	6.894E-02	logistic	205080	243	204837	0.993	0.331
289.3	Personal history of diseases of blood and blood-forming organs	hematopoietic	rs4932372	-0.163	0.090	0.850	7.047E-02	logistic	408604	295	408309	0.999	0.331
564.1	Irritable Bowel Syndrome	digestive	rs4932372	0.039	0.022	1.040	7.491E-02	logistic	339145	4660	334485	0.999	0.332
573	Other disorders of liver	digestive	rs4932372	-0.060	0.034	0.942	7.560E-02	logistic	402294	2020	400274	0.998	0.331
426.3	Bundle branch block	circulatory system	rs4932372	0.045	0.025	1.046	7.636E-02	logistic	375377	3529	371848	0.999	0.331
443	Peripheral vascular disease	circulatory system	rs4932372	0.037	0.021	1.038	7.669E-02	logistic	410595	5126	405469	0.996	0.331
722.9	Other and unspecified disc disorder	musculoskeletal	rs4932372	-0.118	0.067	0.888	7.715E-02	logistic	409194	524	408670	0.996	0.331
317.1	Alcoholism	mental disorders	rs4932372	-0.044	0.025	0.957	7.803E-02	logistic	407082	3789	403293	0.995	0.331
374.1	Ectropion or entropion	sense organs	rs4932372	-0.102	0.058	0.903	7.820E-02	logistic	411493	700	410793	0.996	0.331
368.4	Visual field defects	sense organs	rs4932372	-0.128	0.073	0.880	7.898E-02	logistic	415988	443	415545	0.999	0.331
727.1	Synovitis and tenosynovitis	musculoskeletal	rs4932372	-0.153	0.087	0.858	8.056E-02	logistic	389486	312	389174	0.983	0.331
433.12	Cerebral atherosclerosis	circulatory system	rs4932372	0.138	0.079	1.148	8.080E-02	logistic	403029	344	402685	0.998	0.331
614.3	Pelvic inflammatory disease (PID)	genitourinary	rs4932372	-0.176	0.101	0.838	8.121E-02	logistic	217675	237	217438	0.990	0.331
286.7	Other and unspecified coagulation defects	hematopoietic	rs4932372	-0.165	0.095	0.847	8.141E-02	logistic	417818	265	417553	0.999	0.331
634.1	Missed abortion/Hydatidiform mole	pregnancy complications	rs4932372	-0.186	0.107	0.830	8.198E-02	logistic	222807	215	222592	0.988	0.331
78	Viral warts & HPV	infectious diseases	rs4932372	0.170	0.098	1.186	8.286E-02	logistic	415426	222	415204	0.999	0.331
480.1	Bacterial pneumonia	respiratory	rs4932372	-0.026	0.015	0.974	8.385E-02	logistic	407095	10295	396800	0.996	0.331
262	Mineral deficiency NEC	endocrine/metabolic	rs4932372	-0.143	0.083	0.866	8.454E-02	logistic	412440	343	412097	0.992	0.331
426.9	Cardiac pacemaker/device in situ	circulatory system	rs4932372	0.037	0.021	1.037	8.456E-02	logistic	376950	5102	371848	0.999	0.331
170.2	Cancer of connective tissue	neoplasms	rs4932372	0.160	0.093	1.174	8.495E-02	logistic	423349	248	423101	0.998	0.331
428.1	Congestive heart failure (CHF) NOS	circulatory system	rs4932372	0.041	0.024	1.042	8.674E-02	logistic	413649	3946	409703	0.997	0.331
244.4	Hypothyroidism NOS	endocrine/metabolic	rs4932372	-0.020	0.012	0.980	8.762E-02	logistic	410296	18066	392230	0.999	0.331
440	Atherosclerosis	circulatory system	rs4932372	0.077	0.045	1.080	8.791E-02	logistic	406545	1076	405469	0.991	0.331
433.31	Transient cerebral ischemia	circulatory system	rs4932372	-0.074	0.044	0.928	8.877E-02	logistic	403901	1216	402685	0.998	0.331
378.5	Paralytic strabismus	sense organs	rs4932372	-0.159	0.094	0.853	8.878E-02	logistic	412201	273	411928	0.995	0.331
197	Chemotherapy	neoplasms	rs4932372	-0.024	0.014	0.976	8.907E-02	logistic	397532	11526	386006	0.995	0.331
202.21	Nodular lymphoma	neoplasms	rs4932372	-0.120	0.071	0.887	9.137E-02	logistic	416077	464	415613	0.998	0.331
200	Myeloproliferative disease	neoplasms	rs4932372	0.065	0.039	1.067	9.172E-02	logistic	417103	1490	415613	0.998	0.331
565	Anal and rectal conditions	digestive	rs4932372	-0.029	0.018	0.971	9.343E-02	logistic	391353	7514	383839	0.994	0.331
198.1	Secondary malignancy of lymph nodes	neoplasms	rs4932372	-0.028	0.017	0.972	9.395E-02	logistic	394015	8009	386006	0.998	0.331
297.2	Suicide or self-inflicted injury	mental disorders	rs4932372	-0.142	0.086	0.868	9.667E-02	logistic	381948	326	381622	0.979	0.331
496.2	Chronic bronchitis	respiratory	rs4932372	-0.034	0.021	0.967	9.756E-02	logistic	404328	5519	398809	0.996	0.331
79	Viral infection	infectious diseases	rs4932372	-0.079	0.048	0.924	9.865E-02	logistic	416221	1017	415204	0.999	0.331
427.5	Arrhythmia (cardiac) NOS	circulatory system	rs4932372	-0.113	0.069	0.893	9.912E-02	logistic	372348	500	371848	0.999	0.331
721.1	Spondylosis without myelopathy	musculoskeletal	rs4932372	0.063	0.038	1.065	1.000E-01	logistic	410174	1504	408670	0.997	0.331
8.5	Bacterial enteritis	infectious diseases	rs4932372	0.069	0.042	1.071	1.018E-01	logistic	418567	1251	417316	0.997	0.331
618.1	Prolapse of vaginal walls	genitourinary	rs4932372	-0.040	0.024	0.961	1.025E-01	logistic	217426	3971	213455	0.954	0.331

ST31; PheWAS results for the lead cis-pQTLs of the 4 proteins prioritized (ACOX1, FGF5, FURIN, and MST1).

SNP = Single Nucleotide Polymorphism; SE = Standard error HWE_p = P-value for Hardy-Weinberg equilibrium test

Phenotype	Description	Group	SNP	Beta	SE	Odds tatio	P-value	Type	n_total	n_cases	n_controls	HWE_p	allele_freq
367.2	Astigmatism	sense organs	rs4932372	-0.042	0.026	0.959	1.065E-01	logistic	414418	3412	411006	0.997	0.331
521	Diseases of hard tissues of teeth	digestive	rs4932372	0.084	0.052	1.088	1.069E-01	logistic	410337	801	409536	0.997	0.331
316	Substance addiction and disorders	mental disorders	rs4932372	-0.132	0.082	0.877	1.073E-01	logistic	403645	352	403293	0.992	0.332
521.1	Dental caries	digestive	rs4932372	0.087	0.054	1.091	1.077E-01	logistic	410280	744	409536	0.997	0.331
241	Nontoxic nodular goiter	endocrine/metabolic	rs4932372	-0.088	0.055	0.915	1.081E-01	logistic	392996	766	392230	0.999	0.331
578.8	Hemorrhage of rectum and anus	digestive	rs4932372	0.049	0.031	1.051	1.107E-01	logistic	385630	2327	383303	0.994	0.331
281	Other deficiency anemia	hematopoietic	rs4932372	-0.067	0.042	0.936	1.117E-01	logistic	383766	1317	382449	0.997	0.331
496.21	Obstructive chronic bronchitis	respiratory	rs4932372	-0.033	0.021	0.968	1.134E-01	logistic	404163	5354	398809	0.996	0.331
529	Diseases and other conditions of the tongue	digestive	rs4932372	0.139	0.088	1.149	1.136E-01	logistic	417990	280	417710	0.998	0.331
801.1	Fracture of foot	injuries & poisonings	rs4932372	-0.165	0.105	0.848	1.151E-01	logistic	396597	219	396378	0.998	0.331
151	Cancer of stomach	neoplasms	rs4932372	-0.077	0.049	0.926	1.158E-01	logistic	394829	967	393862	0.999	0.331
378.2	Nystagmus and other irregular eye movements	sense organs	rs4932372	-0.168	0.107	0.845	1.166E-01	logistic	412137	209	411928	0.995	0.331
175	Acquired absence of breast	neoplasms	rs4932372	-0.038	0.024	0.963	1.175E-01	logistic	206502	3992	202510	0.986	0.331
871	Open wounds of extremities	injuries & poisonings	rs4932372	0.120	0.077	1.127	1.177E-01	logistic	420520	370	420150	0.998	0.331
280.1	Iron deficiency anemias, unspecified or not due to blood loss	hematopoietic	rs4932372	0.025	0.016	1.026	1.181E-01	logistic	391238	8789	382449	0.998	0.331
571	Chronic liver disease and cirrhosis	digestive	rs4932372	-0.031	0.020	0.970	1.187E-01	logistic	406135	5861	400274	0.998	0.331
253.2	Pituitary hypofunction	endocrine/metabolic	rs4932372	-0.141	0.091	0.868	1.196E-01	logistic	415534	288	415246	0.998	0.331
280	Iron deficiency anemias	hematopoietic	rs4932372	0.025	0.016	1.025	1.203E-01	logistic	391534	9085	382449	0.998	0.331
278.1	Obesity	endocrine/metabolic	rs4932372	0.019	0.012	1.019	1.213E-01	logistic	405667	15114	390553	0.995	0.331
38	Septicemia	infectious diseases	rs4932372	-0.028	0.018	0.973	1.223E-01	logistic	403876	7229	396647	0.998	0.331
385.3	Cholesteatoma	sense organs	rs4932372	-0.159	0.104	0.853	1.265E-01	logistic	418937	221	418716	0.996	0.331
507	Pleurisy; pleural effusion	respiratory	rs4932372	-0.024	0.015	0.977	1.274E-01	logistic	400937	9880	391057	0.998	0.331
473.3	Paralysis/spasm of vocal cords or larynx	respiratory	rs4932372	0.149	0.098	1.161	1.277E-01	logistic	396742	225	396517	0.992	0.331
379.2	Disorders of vitreous body	sense organs	rs4932372	-0.116	0.076	0.891	1.286E-01	logistic	412333	405	411928	0.996	0.331
252.1	Hyperparathyroidism	endocrine/metabolic	rs4932372	-0.072	0.047	0.931	1.294E-01	logistic	416271	1025	415246	0.998	0.331
214	Lipoma	neoplasms	rs4932372	-0.069	0.046	0.933	1.295E-01	logistic	415339	1112	414227	0.998	0.331
512.8	Cough	respiratory	rs4932372	-0.058	0.038	0.944	1.312E-01	logistic	406184	1585	404599	0.998	0.331
202.24	Large cell lymphoma	neoplasms	rs4932372	-0.069	0.046	0.934	1.327E-01	logistic	416717	1104	415613	0.997	0.331
531.3	Duodenal ulcer	digestive	rs4932372	0.066	0.044	1.069	1.328E-01	logistic	412469	1130	411339	0.997	0.331
364.4	Corneal degenerations	sense organs	rs4932372	-0.067	0.045	0.935	1.333E-01	logistic	398917	1154	397763	0.998	0.331
530.12	Ulcer of esophagus	digestive	rs4932372	-0.044	0.030	0.957	1.334E-01	logistic	359422	2634	356788	0.993	0.331
415.2	Chronic pulmonary heart disease	circulatory system	rs4932372	0.055	0.037	1.057	1.335E-01	logistic	414491	1653	412838	0.998	0.331
721	Spondylosis and allied disorders	musculoskeletal	rs4932372	0.051	0.034	1.053	1.336E-01	logistic	410559	1889	408670	0.997	0.331
204.2	Myeloid leukemia	neoplasms	rs4932372	0.081	0.054	1.084	1.344E-01	logistic	416360	747	415613	0.998	0.331
287.3	Thrombocytopenia	hematopoietic	rs4932372	-0.048	0.032	0.953	1.347E-01	logistic	419777	2224	417553	0.998	0.331
216	Benign neoplasm of skin	neoplasms	rs4932372	0.066	0.044	1.068	1.373E-01	logistic	415074	1116	413958	0.998	0.331
697	Sarcoidosis	dermatologic	rs4932372	-0.092	0.063	0.912	1.425E-01	logistic	405795	589	405206	0.992	0.331
585.3	Chronic renal failure [CKD]	genitourinary	rs4932372	0.020	0.014	1.020	1.429E-01	logistic	393739	12677	381062	1.000	0.331
297	Suicidal ideation or attempt	mental disorders	rs4932372	-0.118	0.081	0.888	1.441E-01	logistic	381982	360	381622	0.979	0.331
278	Overweight, obesity and other hyperalimentation	endocrine/metabolic	rs4932372	0.018	0.012	1.018	1.468E-01	logistic	405726	15173	390553	0.995	0.331
367.8	Hypermetropia	sense organs	rs4932372	0.127	0.088	1.136	1.481E-01	logistic	411285	279	411006	0.998	0.331
994	Sepsis and SIRS	injuries & poisonings	rs4932372	-0.027	0.019	0.974	1.511E-01	logistic	418535	6755	411780	0.998	0.331
994.2	Sepsis	injuries & poisonings	rs4932372	-0.027	0.019	0.974	1.511E-01	logistic	418535	6755	411780	0.998	0.331
427.9	Palpitations	circulatory system	rs4932372	0.051	0.036	1.053	1.515E-01	logistic	373589	1741	371848	0.999	0.331
281.1	Megaloblastic anemia	hematopoietic	rs4932372	-0.061	0.043	0.941	1.533E-01	logistic	383709	1260	382449	0.997	0.331
642	Hypertension complicating pregnancy, childbirth, and the puerperium	pregnancy complications	rs4932372	0.100	0.070	1.105	1.534E-01	logistic	228250	456	227794	0.986	0.331
149.4	Cancer of larynx	neoplasms	rs4932372	-0.132	0.093	0.877	1.549E-01	logistic	420618	276	420342	0.998	0.331
182	Malignant neoplasm of uterus	neoplasms	rs4932372	-0.061	0.043	0.941	1.557E-01	logistic	197217	1245	195972	0.983	0.331
427.6	Premature beats	circulatory system	rs4932372	0.085	0.060	1.089	1.583E-01	logistic	372449	601	371848	0.999	0.331
250	Diabetes mellitus	endocrine/metabolic	rs4932372	0.013	0.009	1.013	1.590E-01	logistic	414239	27602	386637	0.995	0.331
415	Pulmonary heart disease	circulatory system	rs4932372	0.051	0.036	1.052	1.603E-01	logistic	414538	1700	412838	0.998	0.331
747.1	Cardiac congenital anomalies	congenital anomalies	rs4932372	0.067	0.048	1.070	1.634E-01	logistic	422536	941	421595	0.997	0.331
747.11	Cardiac shunt/ heart septal defect	congenital anomalies	rs4932372	0.097	0.070	1.102	1.649E-01	logistic	422041	446	421595	0.998	0.331
287	Purpura and other hemorrhagic conditions	hematopoietic	rs4932372	-0.043	0.031	0.957	1.652E-01	logistic	419896	2343	417553	0.998	0.331
530.14	Reflux esophagitis	digestive	rs4932372	-0.037	0.027	0.964	1.679E-01	logistic	360032	3244	356788	0.995	0.331
427.41	Ventricular fibrillation and flutter	circulatory system	rs4932372	0.124	0.090	1.132	1.688E-01	logistic	372113	265	371848	0.999	0.331
564.9	Personal history of diseases of digestive system	digestive	rs4932372	-0.018	0.013	0.982	1.690E-01	logistic	348229	13744	334485	0.999	0.331
752	Nervous system congenital anomalies	congenital anomalies	rs4932372	0.095	0.069	1.100	1.692E-01	logistic	422992	453	422539	0.998	0.331
496.3	Bronchiectasis	respiratory	rs4932372	-0.034	0.025	0.967	1.714E-01	logistic	402578	3769	398809	0.996	0.332
261.4	Vitamin D deficiency	endocrine/metabolic	rs4932372	-0.044	0.032	0.957	1.715E-01	logistic	414357	2260	412097	0.995	0.331
110	Dermatophytosis / Dermatomycosis	infectious diseases	rs4932372	-0.136	0.100	0.872	1.718E-01	logistic	418617	237	418380	0.998	0.331
961	Poisoning by other anti-infectives	injuries & poisonings	rs4932372	-0.123	0.090	0.884	1.723E-01	logistic	367461	288	367173	0.999	0.331
364.5	Corneal dystrophy	sense organs	rs4932372	-0.105	0.077	0.900	1.737E-01	logistic	398152	389	397763	0.998	0.331
250.2	Type 2 diabetes	endocrine/metabolic	rs4932372	0.013	0.010	1.013	1.742E-01	logistic	413206	26569	386637	0.995	0.331
599	Other symptoms/disorders or the urinary system	genitourinary	rs4932372	0.023	0.017	1.023	1.760E-01	logistic	398281	8175	390106	0.996	0.331
430.1	Subarachnoid hemorrhage	circulatory system	rs4932372	0.074	0.055	1.077	1.786E-01	logistic	403416	731	402685	0.999	0.331
198.4	Secondary malignant neoplasm of liver	neoplasms	rs4932372	-0.028	0.021	0.973	1.795E-01	logistic	391482	5476	386006	0.994	0.331
514	Abnormal findings examination of lungs	respiratory	rs4932372	-0.044	0.033	0.957	1.805E-01	logistic	419804	2147	417657	0.998	0.331
687	Symptoms affecting skin	dermatologic	rs4932372	-0.106	0.080	0.899	1.824E-01	logistic	413083	367	412716	0.994	0.331
157	Pancreatic cancer	neoplasms	rs4932372	-0.054	0.041	0.947	1.827E-01	logistic	395256	1394	393862	0.999	0.331
585.32	End stage renal disease	genitourinary	rs4932372	0.064	0.049	1.066	1.870E-01	logistic	381994	932	381062	1.000	0.331

ST31; PheWAS results for the lead cis-pQTLs of the 4 proteins prioritized (ACOX1, FGF5, FURIN, and MST1).

SNP = Single Nucleotide Polymorphism; SE = Standard error HWE_p = P-value for Hardy-Weinberg equilibrium test

Phenotype	Description	Group	SNP	Beta	SE	Odds tatio	P-value	Type	n_total	n_cases	n_controls	HWE_p	allele_freq
738	Other acquired musculoskeletal deformity	musculoskeletal	rs4932372	0.054	0.041	1.055	1.871E-01	logistic	403982	1328	402654	0.993	0.331
800.2	Fracture of unspecified part of femur	injuries & poisonings	rs4932372	-0.137	0.105	0.872	1.914E-01	logistic	396592	214	396378	0.998	0.331
274.2	Crystal arthropathies	endocrine/metabolic	rs4932372	-0.128	0.099	0.880	1.931E-01	logistic	416033	243	415790	0.997	0.331
550.5	Ventral hernia	digestive	rs4932372	0.048	0.037	1.049	1.934E-01	logistic	367031	1637	365394	0.986	0.331
8	Intestinal infection	infectious diseases	rs4932372	0.044	0.034	1.045	1.935E-01	logistic	419231	1915	417316	0.998	0.331
594	Urinary calculus	genitourinary	rs4932372	-0.026	0.020	0.974	1.936E-01	logistic	418195	5819	412376	0.997	0.331
715.2	Ankylosing spondylitis	musculoskeletal	rs4932372	0.090	0.070	1.095	1.953E-01	logistic	408956	449	408507	0.995	0.331
540.1	Appendicitis	digestive	rs4932372	0.105	0.081	1.111	1.954E-01	logistic	421984	328	421656	0.997	0.331
728.7	Fasciitis	musculoskeletal	rs4932372	-0.046	0.035	0.955	1.957E-01	logistic	391018	1844	389174	0.985	0.331
560.2	Impaction of intestine	digestive	rs4932372	0.125	0.097	1.133	1.979E-01	logistic	334714	229	334485	0.999	0.331
362.31	Separation of retinal layers	sense organs	rs4932372	-0.087	0.067	0.917	1.983E-01	logistic	398271	514	397757	0.998	0.331
385	Other disorders of middle ear and mastoid	sense organs	rs4932372	-0.112	0.087	0.894	2.000E-01	logistic	419023	307	418716	0.996	0.331
614.5	Inflammatory disease of cervix, vagina, and vulva	genitourinary	rs4932372	0.082	0.064	1.086	2.000E-01	logistic	217969	531	217438	0.990	0.331
189.4	Malignant neoplasm of other urinary organs	neoplasms	rs4932372	-0.137	0.107	0.872	2.007E-01	logistic	417393	206	417187	0.998	0.331
315	Develomental delays and disorders	mental disorders	rs4932372	0.073	0.057	1.075	2.020E-01	logistic	422717	680	422037	0.998	0.331
433	Cerebrovascular disease	circulatory system	rs4932372	0.018	0.014	1.018	2.023E-01	logistic	414008	11323	402685	0.997	0.331
41.1	Staphylococcus infections	infectious diseases	rs4932372	-0.040	0.031	0.961	2.027E-01	logistic	398981	2334	396647	0.999	0.331
577	Diseases of pancreas	digestive	rs4932372	0.033	0.026	1.034	2.029E-01	logistic	421247	3239	418008	0.998	0.331
320	Meningitis	neurological	rs4932372	-0.112	0.088	0.894	2.042E-01	logistic	422374	303	422071	0.998	0.331
426.91	Cardiac pacemaker in situ	circulatory system	rs4932372	0.027	0.021	1.028	2.042E-01	logistic	376826	4978	371848	0.999	0.331
977	Personal history of allergy to medicinal agents	injuries & poisonings	rs4932372	0.021	0.017	1.021	2.044E-01	logistic	375378	8205	367173	0.999	0.331
571.5	Other chronic nonalcoholic liver disease	digestive	rs4932372	-0.029	0.023	0.971	2.060E-01	logistic	404607	4333	400274	0.998	0.331
506	Empyema and pneumothorax	respiratory	rs4932372	-0.056	0.044	0.946	2.077E-01	logistic	392231	1174	391057	0.998	0.331
184	Cancer of other female genital organs	neoplasms	rs4932372	-0.035	0.028	0.966	2.098E-01	logistic	207829	2992	204837	0.995	0.331
738.4	Acquired spondylolisthesis	musculoskeletal	rs4932372	0.054	0.043	1.056	2.100E-01	logistic	403846	1192	402654	0.993	0.331
575.7	Other disorders of gallbladder	digestive	rs4932372	0.075	0.060	1.078	2.112E-01	logistic	395657	610	395047	0.997	0.331
374	Other disorders of eyelids	sense organs	rs4932372	-0.040	0.032	0.961	2.118E-01	logistic	413055	2262	410793	0.997	0.331
276.41	Acidosis	endocrine/metabolic	rs4932372	-0.044	0.036	0.956	2.125E-01	logistic	397376	1804	395572	0.994	0.331
427.2	Atrial fibrillation and flutter	circulatory system	rs4932372	0.015	0.012	1.015	2.126E-01	logistic	388316	16468	371848	0.998	0.331
574.12	Cholelithiasis with other cholecystitis	digestive	rs4932372	-0.047	0.038	0.954	2.135E-01	logistic	396636	1589	395047	0.998	0.331
38.1	Gram negative septicemia	infectious diseases	rs4932372	0.103	0.083	1.109	2.141E-01	logistic	396961	314	396647	0.999	0.331
519	Other diseases of respiratory system, not elsewhere classified	respiratory	rs4932372	-0.020	0.016	0.980	2.145E-01	logistic	411962	8832	403130	0.999	0.331
604	Disorders of penis	genitourinary	rs4932372	-0.085	0.069	0.918	2.172E-01	logistic	162266	488	161778	0.793	0.332
709	Diffuse diseases of connective tissue	dermatologic	rs4932372	-0.056	0.045	0.946	2.187E-01	logistic	405028	1129	403899	0.992	0.331
636.3	Hemorrhage in early pregnancy	pregnancy complications	rs4932372	0.123	0.100	1.131	2.189E-01	logistic	222810	218	222592	0.986	0.331
565.1	Anal and rectal polyp	digestive	rs4932372	-0.036	0.029	0.965	2.191E-01	logistic	386554	2715	383839	0.991	0.331
569.1	Toxic gastroenteritis and colitis	digestive	rs4932372	0.101	0.083	1.106	2.222E-01	logistic	384157	318	383839	0.993	0.331
585.33	Chronic Kidney Disease, Stage III	genitourinary	rs4932372	0.021	0.018	1.022	2.251E-01	logistic	388537	7475	381062	1.000	0.331
550.2	Diaphragmatic hernia	digestive	rs4932372	0.012	0.010	1.012	2.282E-01	logistic	388258	22864	365394	0.993	0.331
530.2	Esophageal bleeding (varices/hemorrhage)	digestive	rs4932372	0.087	0.072	1.091	2.298E-01	logistic	357206	418	356788	0.993	0.331
803.1	Fracture of humerus	injuries & poisonings	rs4932372	0.054	0.045	1.056	2.301E-01	logistic	397468	1090	396378	0.998	0.331
274.1	Gout	endocrine/metabolic	rs4932372	0.028	0.023	1.028	2.305E-01	logistic	420024	4234	415790	0.997	0.331
519.8	Other diseases of respiratory system, NEC	respiratory	rs4932372	-0.020	0.016	0.981	2.312E-01	logistic	411807	8677	403130	0.999	0.331
201	Hodgkin's disease	neoplasms	rs4932372	0.104	0.088	1.109	2.372E-01	logistic	415895	282	415613	0.998	0.331
530.3	Stricture and stenosis of esophagus	digestive	rs4932372	-0.052	0.044	0.949	2.379E-01	logistic	357976	1188	356788	0.991	0.331
362.3	Other nondiabetic retinopathy	sense organs	rs4932372	-0.076	0.065	0.927	2.384E-01	logistic	398314	557	397757	0.998	0.331
386.3	Labyrinthitis	sense organs	rs4932372	0.094	0.079	1.098	2.388E-01	logistic	410012	347	409665	0.997	0.331
624.9	stress incontinence, female	genitourinary	rs4932372	-0.041	0.035	0.960	2.407E-01	logistic	220155	1871	218284	0.988	0.330
361	Retinal detachments and defects	sense organs	rs4932372	-0.079	0.067	0.924	2.408E-01	logistic	398276	513	397763	0.998	0.331
752.1	Neural tube defects	congenital anomalies	rs4932372	0.087	0.075	1.091	2.425E-01	logistic	422933	394	422539	0.998	0.331
251.1	Hypoglycemia	endocrine/metabolic	rs4932372	0.045	0.038	1.046	2.432E-01	logistic	383028	1531	381497	0.988	0.331
277	Other disorders of metabolism	endocrine/metabolic	rs4932372	-0.053	0.046	0.948	2.455E-01	logistic	421029	1088	419941	0.998	0.331
253	Disorders of the pituitary gland and its hypothalamic contro	endocrine/metabolic	rs4932372	-0.059	0.051	0.943	2.478E-01	logistic	416136	890	415246	0.997	0.331
295	Schizophrenia and other psychotic disorders	mental disorders	rs4932372	-0.061	0.052	0.941	2.479E-01	logistic	382460	838	381622	0.982	0.331
241.2	Nontoxic multinodular goiter	endocrine/metabolic	rs4932372	-0.115	0.099	0.892	2.481E-01	logistic	392467	237	392230	0.999	0.332
695	Erythematous conditions	dermatologic	rs4932372	-0.041	0.036	0.960	2.499E-01	logistic	407007	1801	405206	0.993	0.331
363	Chorioretinal inflammations, scars, and other disorders of choroid	sense organs	rs4932372	-0.116	0.101	0.891	2.513E-01	logistic	397993	230	397763	0.998	0.331
440.2	Atherosclerosis of the extremities	circulatory system	rs4932372	0.058	0.050	1.060	2.517E-01	logistic	406343	874	405469	0.992	0.331
599.5	Frequency of urination and polyuria	genitourinary	rs4932372	-0.059	0.052	0.942	2.523E-01	logistic	390965	859	390106	0.996	0.331
604.3	Peyronie's disease	genitourinary	rs4932372	-0.117	0.102	0.889	2.525E-01	logistic	162002	224	161778	0.766	0.332
427.22	Atrial flutter	circulatory system	rs4932372	-0.068	0.060	0.935	2.556E-01	logistic	372499	651	371848	0.999	0.331
359	Muscular dystrophies and other myopathies	neurological	rs4932372	-0.104	0.092	0.901	2.590E-01	logistic	419442	274	419168	0.998	0.331
586.11	Small kidney	genitourinary	rs4932372	0.112	0.099	1.119	2.598E-01	logistic	381281	219	381062	0.999	0.331
357	Inflammatory and toxic neuropathy	neurological	rs4932372	-0.038	0.034	0.963	2.600E-01	logistic	421163	1995	419168	0.997	0.331
571.8	Liver abscess and sequelae of chronic liver disease	digestive	rs4932372	-0.044	0.039	0.957	2.621E-01	logistic	401788	1514	400274	0.998	0.331
960.2	Allergy/adverse effect of penicillin	injuries & poisonings	rs4932372	0.013	0.011	1.013	2.623E-01	logistic	385833	18660	367173	0.997	0.331
395.2	Nonrheumatic aortic valve disorders	circulatory system	rs4932372	0.024	0.021	1.024	2.627E-01	logistic	406373	4959	401414	0.999	0.331
276.14	Hypopotassemia	endocrine/metabolic	rs4932372	0.028	0.025	1.029	2.666E-01	logistic	399074	3502	395572	0.993	0.331
741.3	Difficulty in walking	musculoskeletal	rs4932372	0.076	0.069	1.079	2.692E-01	logistic	415474	464	415010	0.999	0.331
600	Hyperplasia of prostate	genitourinary	rs4932372	-0.016	0.014	0.985	2.787E-01	logistic	169018	12557	156461	0.870	0.332
555.1	Regional enteritis	digestive	rs4932372	0.038	0.035	1.038	2.810E-01	logistic	336302	1817	334485	1.000	0.331

ST31; PheWAS results for the lead cis-pQTLs of the 4 proteins prioritized (ACOX1, FGF5, FURIN, and MST1).

SNP = Single Nucleotide Polymorphism; SE = Standard error HWE_p = P-value for Hardy-Weinberg equilibrium test

Phenotype	Description	Group	SNP	Beta	SE	Odds tatio	P-value	Type	n_total	n_cases	n_controls	HWE_p	allele_freq
386	Vertiginous syndromes and other disorders of vestibular systen	sense organs	rs4932372	0.039	0.036	1.040	2.816E-01	logistic	411346	1681	409665	0.998	0.331
255.1	Adrenal hyperfunction	endocrine/metabolic	rs4932372	-0.116	0.108	0.890	2.819E-01	logistic	415447	201	415246	0.997	0.331
276.6	Fluid overload	endocrine/metabolic	rs4932372	0.039	0.037	1.040	2.819E-01	logistic	397239	1667	395572	0.995	0.331
334	Degenerative disease of the spinal corc	neurological	rs4932372	0.038	0.036	1.039	2.820E-01	logistic	400789	1742	399047	0.998	0.331
416	Cardiomegaly	circulatory system	rs4932372	0.024	0.023	1.025	2.833E-01	logistic	417255	4417	412838	0.999	0.331
592.1	Cystitis	genitourinary	rs4932372	-0.046	0.044	0.955	2.872E-01	logistic	378131	1204	376927	0.990	0.331
530.9	Heartburn	digestive	rs4932372	-0.103	0.097	0.902	2.877E-01	logistic	357038	250	356788	0.993	0.331
442.8	Aneurysm of other specified artery	circulatory system	rs4932372	0.097	0.091	1.102	2.891E-01	logistic	405731	262	405469	0.990	0.331
300.12	Agorophobia, social phobia, and panic disordeı	mental disorders	rs4932372	-0.062	0.059	0.940	2.917E-01	logistic	382286	664	381622	0.980	0.331
427.21	Atrial fibrillation	circulatory system	rs4932372	0.024	0.023	1.025	2.919E-01	logistic	376121	4273	371848	0.999	0.331
208	Benign neoplasm of colon	neoplasms	rs4932372	-0.012	0.011	0.989	2.928E-01	logistic	395855	20019	375836	0.998	0.332
289	Other diseases of blood and blood-forming organs	hematopoietic	rs4932372	-0.056	0.053	0.946	2.941E-01	logistic	409120	811	408309	0.998	0.331
946	Anaphylactic shock NOS	injuries & poisonings	rs4932372	-0.107	0.102	0.898	2.948E-01	logistic	415210	223	414987	0.998	0.331
367	Disorders of refraction and accommodation; blindness and low visior	sense organs	rs4932372	-0.020	0.019	0.980	2.951E-01	logistic	417170	6164	411006	0.997	0.331
537	Other disorders of stomach and duodenum	digestive	rs4932372	-0.037	0.035	0.964	2.982E-01	logistic	364031	1817	362214	0.997	0.331
352.2	Facial nerve disorders [CN7]	neurological	rs4932372	0.059	0.057	1.061	2.996E-01	logistic	403092	677	402415	0.998	0.331
789	Nausea and vomiting	symptoms	rs4932372	0.019	0.018	1.019	3.014E-01	logistic	407825	6923	400902	0.998	0.331
81	Infection/inflammation of internal prosthetic device; implant; and graf	infectious diseases	rs4932372	-0.035	0.034	0.966	3.088E-01	logistic	414783	1939	412844	0.996	0.331
381	Otitis media and Eustachian tube disorders	sense organs	rs4932372	-0.053	0.052	0.949	3.096E-01	logistic	419568	852	418716	0.997	0.331
204.21	Myeloid leukemia, acute	neoplasms	rs4932372	0.062	0.062	1.064	3.129E-01	logistic	416195	582	415613	0.998	0.331
225	Benign neoplasm of brain and other parts of nervous systen	neoplasms	rs4932372	-0.053	0.052	0.949	3.139E-01	logistic	421233	836	420397	0.998	0.331
276.4	Acid-base balance disorder	endocrine/metabolic	rs4932372	-0.033	0.033	0.967	3.141E-01	logistic	397692	2120	395572	0.994	0.331
250.42	Other abnormal glucose	endocrine/metabolic	rs4932372	-0.088	0.088	0.916	3.155E-01	logistic	386940	303	386637	0.987	0.331
454.11	Varicose veins of lower extremity, symptomtic	circulatory system	rs4932372	-0.063	0.062	0.939	3.156E-01	logistic	381560	594	380966	0.984	0.332
337	Disorders of the autonomic nervous systen	neurological	rs4932372	0.079	0.079	1.083	3.164E-01	logistic	399396	349	399047	0.997	0.331
577.1	Acute pancreatitis	digestive	rs4932372	0.035	0.035	1.035	3.181E-01	logistic	419858	1850	418008	0.998	0.331
572	Ascites (non malignant)	digestive	rs4932372	-0.033	0.033	0.968	3.204E-01	logistic	402409	2135	400274	0.997	0.331
801	Fracture of ankle and foot	injuries & poisonings	rs4932372	-0.058	0.059	0.943	3.209E-01	logistic	397040	662	396378	0.998	0.331
193	Thyroid cancer	neoplasms	rs4932372	-0.074	0.075	0.929	3.218E-01	logistic	421300	413	420887	0.998	0.331
38.2	Gram positive septicemia	infectious diseases	rs4932372	-0.060	0.061	0.942	3.227E-01	logistic	397268	621	396647	0.999	0.331
857	Mechanical complication of unspecified genitourinary device, implant, and graf	injuries & poisonings	rs4932372	-0.038	0.038	0.963	3.232E-01	logistic	418243	1591	416652	0.998	0.331
218.1	Uterine leiomyoma	neoplasms	rs4932372	-0.028	0.029	0.972	3.280E-01	logistic	209756	2761	206995	0.984	0.331
276.13	Hyperpotassemia	endocrine/metabolic	rs4932372	0.035	0.036	1.036	3.280E-01	logistic	397299	1727	395572	0.996	0.331
367.9	Blindness and low vision	sense organs	rs4932372	-0.050	0.051	0.951	3.281E-01	logistic	411881	875	411006	0.997	0.331
628	Ovarian cyst	genitourinary	rs4932372	0.042	0.043	1.043	3.286E-01	logistic	193944	1197	192747	0.972	0.331
761	Cervicalgia	symptoms	rs4932372	-0.054	0.056	0.947	3.300E-01	logistic	422454	744	421710	0.998	0.331
276.11	Hyperosmolality and/or hypernatremia	endocrine/metabolic	rs4932372	-0.055	0.057	0.946	3.301E-01	logistic	396286	714	395572	0.994	0.331
386.1	Meniere's disease	sense organs	rs4932372	0.057	0.059	1.059	3.309E-01	logistic	410308	643	409665	0.997	0.331
750.1	Upper gastrointestinal congenital anomalies	congenital anomalies	rs4932372	0.098	0.102	1.103	3.334E-01	logistic	420464	211	420253	0.998	0.331
752.11	Spina bifida	congenital anomalies	rs4932372	0.100	0.103	1.105	3.344E-01	logistic	422743	204	422539	0.998	0.331
531.1	Hemorrhage from gastrointestinal ulcer	digestive	rs4932372	-0.061	0.063	0.941	3.352E-01	logistic	411913	574	411339	0.998	0.331
592.12	Chronic cystitis	genitourinary	rs4932372	-0.093	0.097	0.911	3.358E-01	logistic	377176	249	376927	0.991	0.331
586.1	Anatomical abnormatilies of kidney and ureters	genitourinary	rs4932372	0.092	0.096	1.096	3.364E-01	logistic	381301	239	381062	0.999	0.331
290	Delirium dementia and amnestic and other cognitive disorders	mental disorders	rs4932372	0.017	0.018	1.017	3.365E-01	logistic	412158	7185	404973	0.998	0.331
592	Cystitis and urethritis	genitourinary	rs4932372	-0.041	0.043	0.960	3.372E-01	logistic	378176	1249	376927	0.990	0.331
200.1	Polycythemia vera	neoplasms	rs4932372	0.062	0.065	1.064	3.387E-01	logistic	408705	523	408182	0.998	0.331
558	Noninfectious gastroenteritis	digestive	rs4932372	-0.022	0.023	0.979	3.404E-01	logistic	338928	4443	334485	0.999	0.331
619.3	Noninflammatory disorders of cervix	genitourinary	rs4932372	-0.078	0.082	0.925	3.411E-01	logistic	217308	342	216966	0.988	0.331
695.2	Bullous dermatoses	dermatologic	rs4932372	-0.102	0.107	0.903	3.411E-01	logistic	405408	202	405206	0.993	0.331
427.3	Other specified cardiac dysrhythmias	circulatory system	rs4932372	-0.028	0.030	0.972	3.420E-01	logistic	374456	2608	371848	0.999	0.331
394.2	Mitral valve disease	circulatory system	rs4932372	0.080	0.084	1.083	3.422E-01	logistic	401726	312	401414	0.999	0.331
375	Disorders of lacrimal system	sense organs	rs4932372	0.037	0.039	1.038	3.438E-01	logistic	413379	1451	411928	0.995	0.331
803.2	Fracture of radius and ulna	injuries & poisonings	rs4932372	0.030	0.032	1.030	3.446E-01	logistic	398618	2240	396378	0.998	0.331
427	Cardiac dysrhythmias	circulatory system	rs4932372	0.009	0.010	1.009	3.460E-01	logistic	398369	26521	371848	0.999	0.331
446.5	Giant cell arteritis	circulatory system	rs4932372	0.057	0.061	1.059	3.470E-01	logistic	406071	602	405469	0.990	0.331
569.2	Gastrointestinal complications	digestive	rs4932372	0.083	0.089	1.086	3.514E-01	logistic	384117	278	383839	0.993	0.331
960	Poisoning by antibiotics	injuries & poisonings	rs4932372	0.010	0.010	1.010	3.524E-01	logistic	389532	22359	367173	0.998	0.331
516	Abnormal sputum	respiratory	rs4932372	0.044	0.048	1.045	3.527E-01	logistic	421370	981	420389	0.998	0.331
947	Urticaria	dermatologic	rs4932372	0.095	0.102	1.100	3.528E-01	logistic	415196	209	414987	0.997	0.331
174	Breast cancer	neoplasms	rs4932372	-0.014	0.015	0.986	3.558E-01	logistic	401305	10248	391057	0.998	0.331
365.2	Primary angle-closure glaucoma	sense organs	rs4932372	-0.054	0.058	0.948	3.569E-01	logistic	398441	678	397763	0.998	0.331
225.1	Benign neoplasm of brain, cranial nerves, meninges	neoplasms	rs4932372	-0.048	0.053	0.953	3.645E-01	logistic	421204	807	420397	0.998	0.331
615	Endometriosis	genitourinary	rs4932372	0.039	0.043	1.040	3.651E-01	logistic	218669	1231	217438	0.988	0.331
540.11	Acute appendicitis	digestive	rs4932372	0.090	0.100	1.094	3.691E-01	logistic	421875	219	421656	0.997	0.331
301	Personality disorders	mental disorders	rs4932372	-0.073	0.082	0.929	3.711E-01	logistic	381968	346	381622	0.979	0.332
585.31	Renal dialysis	genitourinary	rs4932372	0.050	0.056	1.051	3.714E-01	logistic	381775	713	381062	1.000	0.331
707	Chronic ulcer of skin	dermatologic	rs4932372	0.019	0.022	1.020	3.729E-01	logistic	420904	4774	416130	0.997	0.331
253.7	Other disorders of neurohypophysis	endocrine/metabolic	rs4932372	-0.073	0.081	0.930	3.729E-01	logistic	415594	348	415246	0.998	0.331
333.4	Torsion dystonia	neurological	rs4932372	0.076	0.085	1.079	3.751E-01	logistic	399349	302	399047	0.998	0.331
497	Bronchitis	respiratory	rs4932372	-0.078	0.088	0.925	3.773E-01	logistic	399105	296	398809	0.997	0.332
451	Phlebitis and thrombophlebitis	circulatory system	rs4932372	-0.050	0.057	0.951	3.775E-01	logistic	381675	709	380966	0.982	0.332

ST31; PheWAS results for the lead cis-pQTLs of the 4 proteins prioritized (ACOX1, FGF5, FURIN, and MST1).

SNP = Single Nucleotide Polymorphism; SE = Standard error HWE_p = P-value for Hardy-Weinberg equilibrium test

Phenotype	Description	Group	SNP	Beta	SE	Odds tatio	P-value	Type	n_total	n_cases	n_controls	HWE_p	allele_freq
575.2	Obstruction of bile duct	digestive	rs4932372	-0.042	0.047	0.959	3.784E-01	logistic	396076	1029	395047	0.998	0.331
415.21	Primary pulmonary hypertensior	circulatory system	rs4932372	0.070	0.079	1.072	3.798E-01	logistic	413189	351	412838	0.998	0.331
276.42	Alkalosis	endocrine/metabolic	rs4932372	0.074	0.085	1.077	3.819E-01	logistic	395878	306	395572	0.994	0.331
174.1	Breast cancer [female]	neoplasms	rs4932372	-0.013	0.015	0.987	3.821E-01	logistic	213896	10137	203759	0.992	0.331
285.2	Anemia of chronic disease	hematopoietic	rs4932372	0.046	0.053	1.047	3.828E-01	logistic	383237	788	382449	0.997	0.331
710.1	Osteomyelitis	musculoskeletal	rs4932372	0.085	0.098	1.088	3.874E-01	logistic	412277	229	412048	0.997	0.331
803	Fracture of upper limb	injuries & poisonings	rs4932372	0.020	0.023	1.020	3.890E-01	logistic	400593	4215	396378	0.998	0.331
803.3	Fracture of clavicle or scapula	injuries & poisonings	rs4932372	-0.049	0.057	0.952	3.895E-01	logistic	397087	709	396378	0.998	0.331
740.11	Osteoarthrosis, localized, primary	musculoskeletal	rs4932372	-0.018	0.021	0.982	3.913E-01	logistic	351108	5250	345858	0.999	0.332
446.4	Wegener's granulomatosis	circulatory system	rs4932372	-0.085	0.100	0.918	3.920E-01	logistic	405703	234	405469	0.991	0.331
790	Nonspecific findings on examination of blood	symptoms	rs4932372	-0.081	0.095	0.922	3.949E-01	logistic	422095	255	421840	0.997	0.331
702.2	Seborrheic keratosis	dermatologic	rs4932372	-0.055	0.065	0.946	3.959E-01	logistic	413611	539	413072	0.993	0.331
727.6	Rupture of tendon, nontraumatic	musculoskeletal	rs4932372	0.035	0.041	1.035	3.959E-01	logistic	390517	1343	389174	0.979	0.331
522.5	Periapical abscess	digestive	rs4932372	-0.088	0.106	0.915	4.024E-01	logistic	409744	208	409536	0.998	0.331
53	Herpes zoster	infectious diseases	rs4932372	-0.067	0.080	0.935	4.026E-01	logistic	415563	359	415204	0.999	0.331
530.5	Disorders of esophageal motility	digestive	rs4932372	0.060	0.072	1.062	4.027E-01	logistic	357212	424	356788	0.993	0.331
295.1	Schizophrenia	mental disorders	rs4932372	-0.049	0.059	0.952	4.039E-01	logistic	382292	670	381622	0.982	0.331
742	Derangement of joint, non-traumatic	musculoskeletal	rs4932372	-0.061	0.073	0.941	4.054E-01	logistic	415444	434	415010	0.998	0.331
701.2	Scar conditions and fibrosis of skin	dermatologic	rs4932372	-0.064	0.077	0.938	4.060E-01	logistic	415576	390	415186	0.998	0.331
550.4	Umbilical hernia	digestive	rs4932372	-0.033	0.040	0.967	4.075E-01	logistic	366811	1417	365394	0.988	0.331
241.1	Nontoxic uninodular goiter	endocrine/metabolic	rs4932372	-0.084	0.101	0.920	4.092E-01	logistic	392455	225	392230	0.999	0.332
149.1	Cancer of oropharynx	neoplasms	rs4932372	0.062	0.076	1.064	4.141E-01	logistic	420725	383	420342	0.998	0.331
695.9	Unspecified erythematous conditior	dermatologic	rs4932372	0.068	0.083	1.070	4.141E-01	logistic	405524	318	405206	0.993	0.331
550	Abdominal hernia	digestive	rs4932372	0.008	0.009	1.008	4.150E-01	logistic	392811	27417	365394	0.992	0.331
747	Cardiac and circulatory congenital anomalies	congenital anomalies	rs4932372	0.037	0.045	1.037	4.165E-01	logistic	422691	1096	421595	0.997	0.331
283	Acquired hemolytic anemias	hematopoietic	rs4932372	-0.087	0.108	0.916	4.171E-01	logistic	382649	200	382449	0.997	0.331
184.1	Malignant neoplasm of ovary and other uterine adnexa	neoplasms	rs4932372	-0.023	0.029	0.977	4.173E-01	logistic	207626	2789	204837	0.994	0.331
292	Neurological disorders	mental disorders	rs4932372	-0.017	0.021	0.983	4.194E-01	logistic	410372	5399	404973	0.994	0.331
350.1	Abnormal involuntary movements	neurological	rs4932372	0.043	0.053	1.043	4.195E-01	logistic	421010	800	420210	0.999	0.331
350	Abnormal movement	neurological	rs4932372	0.035	0.043	1.035	4.206E-01	logistic	421410	1200	420210	0.998	0.331
580.2	Nephrotic syndrome without mention of glomerulonephritis	genitourinary	rs4932372	-0.061	0.076	0.941	4.221E-01	logistic	381457	395	381062	0.999	0.331
800.1	Fracture of neck of femur	injuries & poisonings	rs4932372	-0.027	0.033	0.974	4.222E-01	logistic	398432	2054	396378	0.998	0.331
726.3	Bursitis	musculoskeletal	rs4932372	-0.085	0.106	0.918	4.225E-01	logistic	389380	206	389174	0.982	0.331
536.8	Dyspepsia and other specified disorders of function of stomach	digestive	rs4932372	-0.030	0.037	0.971	4.227E-01	logistic	363851	1637	362214	0.997	0.331
707.2	Chronic ulcer of leg or foot	dermatologic	rs4932372	0.026	0.033	1.027	4.235E-01	logistic	418232	2102	416130	0.997	0.331
276.12	Hyposmolality and/or hyponatremia	endocrine/metabolic	rs4932372	-0.017	0.022	0.983	4.246E-01	logistic	400551	4979	395572	0.993	0.331
184.11	Malignant neoplasm of ovary	neoplasms	rs4932372	-0.023	0.029	0.977	4.270E-01	logistic	207552	2715	204837	0.994	0.331
717	Polymyalgia Rheumatica	musculoskeletal	rs4932372	-0.024	0.031	0.976	4.291E-01	logistic	422397	2442	419955	0.998	0.331
401.22	Hypertensive chronic kidney disease	circulatory system	rs4932372	0.038	0.048	1.039	4.298E-01	logistic	291632	969	290663	0.940	0.328
270	Disorders of protein plasma/amino-acid transport and metabolism	endocrine/metabolic	rs4932372	0.042	0.053	1.042	4.335E-01	logistic	422490	788	421702	0.998	0.331
556.1	Ulceration of intestine	digestive	rs4932372	0.081	0.105	1.085	4.378E-01	logistic	334685	200	334485	0.999	0.331
218	Benign neoplasm of uterus	neoplasms	rs4932372	-0.022	0.028	0.978	4.382E-01	logistic	209861	2866	206995	0.987	0.331
199	Neoplasm of uncertain behavior	neoplasms	rs4932372	-0.047	0.061	0.954	4.386E-01	logistic	386620	614	386006	0.995	0.332
788	Syncope and collapse	symptoms	rs4932372	-0.015	0.019	0.985	4.399E-01	logistic	412052	6241	405811	0.997	0.331
574	Cholelithiasis and cholecystitis	digestive	rs4932372	-0.011	0.014	0.989	4.416E-01	logistic	406113	11066	395047	0.998	0.331
227.1	Benign neoplasm of adrenal gland	neoplasms	rs4932372	-0.072	0.095	0.930	4.456E-01	logistic	421144	257	420887	0.999	0.331
715	Other inflammatory spondylopathies	musculoskeletal	rs4932372	0.032	0.041	1.032	4.458E-01	logistic	409805	1298	408507	0.996	0.331
627.1	Postmenopausal bleeding	genitourinary	rs4932372	0.027	0.035	1.027	4.494E-01	logistic	194538	1791	192747	0.982	0.331
635	Hemorrhage during pregnancy; childbirth and postpartum	pregnancy complications	rs4932372	0.056	0.074	1.057	4.523E-01	logistic	223005	413	222592	0.986	0.331
599.2	Retention of urine	genitourinary	rs4932372	-0.014	0.018	0.986	4.532E-01	logistic	396988	6882	390106	0.997	0.331
741	Symptoms and disorders of the joints	musculoskeletal	rs4932372	-0.028	0.038	0.972	4.575E-01	logistic	416612	1602	415010	0.999	0.331
724	Other and unspecified disorders of back	musculoskeletal	rs4932372	0.047	0.064	1.049	4.583E-01	logistic	409212	542	408670	0.996	0.331
540	Appendiceal conditions	digestive	rs4932372	0.051	0.069	1.052	4.585E-01	logistic	422123	467	421656	0.998	0.331
835	Internal derangement of knee	injuries & poisonings	rs4932372	-0.016	0.022	0.984	4.641E-01	logistic	409924	4709	405215	0.997	0.331
145	Cancer of mouth	neoplasms	rs4932372	0.042	0.057	1.043	4.646E-01	logistic	421016	674	420342	0.998	0.331
296.22	Major depressive disorder	mental disorders	rs4932372	-0.009	0.012	0.991	4.659E-01	logistic	396820	15198	381622	0.987	0.331
401.2	Hypertensive heart and/or renal disease	circulatory system	rs4932372	0.032	0.044	1.033	4.660E-01	logistic	291807	1144	290663	0.948	0.328
450	Noninfectious disorders of lymphatic channels	circulatory system	rs4932372	-0.032	0.044	0.968	4.674E-01	logistic	422720	1167	421553	0.998	0.331
958	Certain early complications of trauma or procedure	injuries & poisonings	rs4932372	0.064	0.089	1.066	4.679E-01	logistic	423583	281	423302	0.998	0.331
345.1	Epilepsy	neurological	rs4932372	0.046	0.064	1.047	4.680E-01	logistic	399597	550	399047	0.997	0.331
535.6	Duodenitis	digestive	rs4932372	0.026	0.036	1.026	4.689E-01	logistic	363993	1779	362214	0.997	0.331
41.4	E. coli	infectious diseases	rs4932372	0.019	0.026	1.019	4.701E-01	logistic	399991	3344	396647	0.999	0.331
366.2	Senile cataract	sense organs	rs4932372	-0.012	0.016	0.988	4.707E-01	logistic	380814	9136	371678	0.984	0.332
427.4	Cardiac arrest and ventricular fibrillation	circulatory system	rs4932372	0.036	0.050	1.036	4.710E-01	logistic	372755	907	371848	0.999	0.331
425	Cardiomyopathy	circulatory system	rs4932372	-0.025	0.035	0.976	4.728E-01	logistic	418683	1913	416770	0.999	0.331
619.2	Disorders of uterus, NEC	genitourinary	rs4932372	0.052	0.073	1.054	4.732E-01	logistic	217379	413	216966	0.983	0.331
590	Pyelonephritis	genitourinary	rs4932372	-0.029	0.041	0.971	4.735E-01	logistic	378294	1367	376927	0.991	0.331
536	Disorders of function of stomach	digestive	rs4932372	-0.027	0.037	0.974	4.736E-01	logistic	363858	1644	362214	0.997	0.331
735.3	Hallux valgus (Bunion)	musculoskeletal	rs4932372	-0.022	0.030	0.978	4.747E-01	logistic	405116	2462	402654	0.995	0.331
701	Other hypertrophic and atrophic conditions of skin	dermatologic	rs4932372	0.031	0.044	1.032	4.749E-01	logistic	416347	1161	415186	0.997	0.331
560.3	Peritoneal or intestinal adhesions	digestive	rs4932372	-0.037	0.052	0.963	4.754E-01	logistic	335317	832	334485	0.999	0.331

ST31; PheWAS results for the lead cis-pQTLs of the 4 proteins prioritized (ACOX1, FGF5, FURIN, and MST1).

SNP = Single Nucleotide Polymorphism; SE = Standard error HWE_p = P-value for Hardy-Weinberg equilibrium test

Phenotype	Description	Group	SNP	Beta	SE	Odds tatio	P-value	Type	n_total	n_cases	n_controls	HWE_p	allele_freq
575.9	Nonspecific abnormal findings on radiological and other examination of biliary trac	digestive	rs4932372	-0.072	0.101	0.931	4.768E-01	logistic	395275	228	395047	0.998	0.331
512	Other symptoms of respiratory systen	respiratory	rs4932372	-0.013	0.019	0.987	4.791E-01	logistic	411090	6491	404599	0.998	0.331
579.2	Splenomegaly	digestive	rs4932372	0.042	0.060	1.043	4.828E-01	logistic	383919	616	383303	0.993	0.331
689	Disorder of skin and subcutaneous tissue NOS	dermatologic	rs4932372	0.023	0.033	1.023	4.839E-01	logistic	413188	2098	411090	0.997	0.331
187	Cancer of other male genital organs	neoplasms	rs4932372	-0.012	0.018	0.988	4.840E-01	logistic	186921	7527	179394	0.921	0.332
703.1	Ingrowing nail	dermatologic	rs4932372	0.072	0.104	1.075	4.871E-01	logistic	421519	205	421314	0.999	0.331
389.3	Degenerative and vascular disorders of ear	sense organs	rs4932372	0.055	0.081	1.057	4.913E-01	logistic	408638	341	408297	0.998	0.331
800.3	Fracture of tibia and fibula	injuries & poisonings	rs4932372	0.043	0.062	1.043	4.929E-01	logistic	396955	577	396378	0.998	0.331
741.4	Joint effusions	musculoskeletal	rs4932372	-0.040	0.058	0.961	4.929E-01	logistic	415693	683	415010	0.998	0.331
743	Osteoporosis, osteopenia and pathological fracture	musculoskeletal	rs4932372	-0.030	0.044	0.970	4.955E-01	logistic	422387	1153	421234	0.998	0.331
681	Superficial cellulitis and abscess	dermatologic	rs4932372	-0.059	0.087	0.942	4.961E-01	logistic	418799	302	418497	0.997	0.331
481	Influenza	respiratory	rs4932372	-0.048	0.070	0.953	4.966E-01	logistic	397264	464	396800	0.998	0.331
352.1	Trigeminal nerve disorders [CN5]	neurological	rs4932372	-0.046	0.068	0.955	4.978E-01	logistic	402912	497	402415	0.998	0.331
292.1	Aphasia/speech disturbance	mental disorders	rs4932372	-0.031	0.047	0.969	5.015E-01	logistic	406020	1047	404973	0.993	0.331
394.7	Disease of tricuspid valve	circulatory system	rs4932372	-0.040	0.059	0.961	5.016E-01	logistic	402072	658	401414	0.999	0.331
70	Viral hepatitis	infectious diseases	rs4932372	-0.043	0.064	0.958	5.016E-01	logistic	415755	551	415204	0.999	0.331
172.1	Melanomas of skin, dx or hx	neoplasms	rs4932372	-0.024	0.035	0.977	5.035E-01	logistic	409038	1830	407208	0.999	0.331
172.11	Melanomas of skin	neoplasms	rs4932372	-0.024	0.035	0.977	5.035E-01	logistic	409038	1830	407208	0.999	0.331
368.2	Diplopia and disorders of binocular vision	sense organs	rs4932372	-0.044	0.066	0.957	5.051E-01	logistic	416069	524	415545	0.998	0.331
365	Glaucoma	sense organs	rs4932372	-0.012	0.018	0.988	5.057E-01	logistic	405209	7446	397763	0.997	0.331
368	Visual disturbances	sense organs	rs4932372	-0.021	0.031	0.979	5.070E-01	logistic	417843	2298	415545	0.998	0.331
574.11	Cholelithiasis with acute cholecystitis	digestive	rs4932372	-0.031	0.047	0.969	5.079E-01	logistic	396059	1012	395047	0.998	0.331
358	Myoneural disorders	neurological	rs4932372	-0.056	0.085	0.945	5.090E-01	logistic	419485	317	419168	0.998	0.331
159	Malignant neoplasm of other and ill-defined sites within the digestive organs and peritoneum	neoplasms	rs4932372	-0.034	0.051	0.967	5.099E-01	logistic	394736	874	393862	0.999	0.331
610	Benign mammary dysplasias	genitourinary	rs4932372	0.044	0.066	1.045	5.099E-01	logistic	220777	505	220272	0.972	0.331
564	Functional digestive disorders	digestive	rs4932372	-0.007	0.011	0.993	5.116E-01	logistic	354387	19902	334485	0.998	0.331
531.2	Gastric ulcer	digestive	rs4932372	-0.024	0.037	0.976	5.172E-01	logistic	413012	1673	411339	0.998	0.331
710	Osteomyelitis, periostitis, and other infections involving bone	musculoskeletal	rs4932372	0.058	0.089	1.059	5.172E-01	logistic	412326	278	412048	0.997	0.331
465	Acute upper respiratory infections of multiple or unspecified sites	respiratory	rs4932372	-0.037	0.057	0.964	5.195E-01	logistic	420386	694	419692	0.998	0.331
459.9	Circulatory disease NEC	circulatory system	rs4932372	0.007	0.010	1.007	5.206E-01	logistic	395548	22976	372572	0.998	0.331
381.11	Suppurative and unspecified otitis media	sense organs	rs4932372	0.060	0.093	1.062	5.218E-01	logistic	418969	253	418716	0.997	0.331
580.14	Chronic glomerulonephritis, NOS	genitourinary	rs4932372	0.053	0.084	1.055	5.242E-01	logistic	381376	314	381062	1.000	0.331
345.11	Generalized convulsive epilepsy	neurological	rs4932372	0.048	0.075	1.049	5.248E-01	logistic	399441	394	399047	0.997	0.331
351	Other peripheral nerve disorders	neurological	rs4932372	-0.012	0.018	0.988	5.262E-01	logistic	409126	6711	402415	0.999	0.331
375.2	Epiphora	sense organs	rs4932372	0.052	0.082	1.053	5.262E-01	logistic	412257	329	411928	0.996	0.331
276.5	Hypovolemia	endocrine/metabolic	rs4932372	0.013	0.021	1.013	5.294E-01	logistic	400853	5281	395572	0.995	0.331
274	Gout and other crystal arthropathies	endocrine/metabolic	rs4932372	0.014	0.023	1.014	5.302E-01	logistic	420276	4486	415790	0.997	0.331
471	Nasal polyps	respiratory	rs4932372	0.029	0.047	1.030	5.309E-01	logistic	397549	1032	396517	0.993	0.331
430	Intracranial hemorrhage	circulatory system	rs4932372	-0.022	0.035	0.979	5.316E-01	logistic	404595	1910	402685	0.999	0.331
709.3	Systemic sclerosis	dermatologic	rs4932372	-0.064	0.102	0.938	5.350E-01	logistic	404118	219	403899	0.991	0.331
174.11	Malignant neoplasm of female breast	neoplasms	rs4932372	-0.010	0.016	0.990	5.364E-01	logistic	212959	9200	203759	0.992	0.331
939	Atopic/contact dermatitis due to other or unspecified	dermatologic	rs4932372	0.021	0.034	1.021	5.365E-01	logistic	416950	1963	414987	0.998	0.331
858	Complication of internal orthopedic device	injuries & poisonings	rs4932372	0.026	0.043	1.027	5.369E-01	logistic	417889	1237	416652	0.998	0.331
601.12	Chronic prostatitis	genitourinary	rs4932372	0.060	0.097	1.062	5.371E-01	logistic	156694	233	156461	0.836	0.333
586.4	Stricture/obstruction of ureter	genitourinary	rs4932372	0.032	0.052	1.032	5.377E-01	logistic	381896	834	381062	1.000	0.331
474	Acute and chronic tonsillitis	respiratory	rs4932372	-0.048	0.078	0.953	5.396E-01	logistic	396895	378	396517	0.992	0.331
800	Fracture of lower limb	injuries & poisonings	rs4932372	-0.017	0.027	0.984	5.404E-01	logistic	399482	3104	396378	0.999	0.331
414	Other forms of chronic heart disease	circulatory system	rs4932372	0.014	0.024	1.014	5.445E-01	logistic	374670	4120	370550	0.994	0.330
335	Multiple sclerosis	neurological	rs4932372	-0.024	0.039	0.976	5.460E-01	logistic	400514	1467	399047	0.996	0.331
433.5	Cerebral aneurysm	circulatory system	rs4932372	-0.043	0.072	0.958	5.461E-01	logistic	403130	445	402685	0.998	0.331
611	Abnormal findings on mammogram or breast exam	genitourinary	rs4932372	-0.056	0.093	0.946	5.488E-01	logistic	415016	264	414752	0.995	0.331
611.3	Lump or mass in breast	genitourinary	rs4932372	-0.056	0.093	0.946	5.488E-01	logistic	415016	264	414752	0.995	0.331
333.8	Other degenerative diseases of the basal ganglia	neurological	rs4932372	0.059	0.099	1.061	5.514E-01	logistic	399271	224	399047	0.998	0.331
339	Other headache syndromes	neurological	rs4932372	0.014	0.023	1.014	5.523E-01	logistic	409356	4169	405187	0.995	0.331
204.12	Lymphoid leukemia, chronic	neoplasms	rs4932372	-0.031	0.053	0.969	5.536E-01	logistic	416428	815	415613	0.998	0.331
276	Disorders of fluid, electrolyte, and acid-base balance	endocrine/metabolic	rs4932372	-0.007	0.012	0.993	5.568E-01	logistic	411277	15705	395572	0.996	0.331
395.1	Nonrheumatic mitral valve disorders	circulatory system	rs4932372	-0.015	0.025	0.985	5.570E-01	logistic	405090	3676	401414	0.998	0.331
155	Cancer of liver and intrahepatic bile duct	neoplasms	rs4932372	-0.032	0.055	0.968	5.593E-01	logistic	394604	742	393862	0.999	0.331
317.11	Alcoholic liver damage	mental disorders	rs4932372	-0.025	0.044	0.975	5.606E-01	logistic	404493	1200	403293	0.992	0.332
418.1	Precordial pain	circulatory system	rs4932372	0.020	0.035	1.020	5.643E-01	logistic	417539	1859	415680	0.996	0.331
530.1	Esophagitis, GERD and related diseases	digestive	rs4932372	-0.005	0.009	0.995	5.646E-01	logistic	385890	29102	356788	0.998	0.331
285	Other anemias	hematopoietic	rs4932372	-0.008	0.014	0.992	5.660E-01	logistic	393921	11472	382449	0.999	0.331
290.1	Dementias	mental disorders	rs4932372	0.013	0.023	1.013	5.671E-01	logistic	409256	4283	404973	0.997	0.331
288	Diseases of white blood cells	hematopoietic	rs4932372	-0.042	0.074	0.958	5.672E-01	logistic	408723	414	408309	0.999	0.331
300	Anxiety disorders	mental disorders	rs4932372	-0.009	0.015	0.991	5.674E-01	logistic	391438	9816	381622	0.989	0.331
327.3	Sleep apnea	neurological	rs4932372	-0.012	0.021	0.988	5.697E-01	logistic	418773	5389	413384	0.998	0.331
619.4	Noninflammatory disorders of vagina	genitourinary	rs4932372	-0.057	0.100	0.945	5.697E-01	logistic	217194	228	216966	0.986	0.331
573.3	Hepatomegaly	digestive	rs4932372	0.050	0.089	1.051	5.741E-01	logistic	400551	277	400274	0.998	0.331
735.21	Hammer toe (acquired)	musculoskeletal	rs4932372	0.039	0.070	1.040	5.756E-01	logistic	403105	451	402654	0.994	0.331
580.32	Nephritis and nephropathy with pathological lesion	genitourinary	rs4932372	-0.051	0.091	0.950	5.768E-01	logistic	381336	274	381062	1.000	0.331
379	Other disorders of eye	sense organs	rs4932372	-0.026	0.047	0.974	5.807E-01	logistic	412963	1035	411928	0.996	0.331

ST31; PheWAS results for the lead cis-pQTLs of the 4 proteins prioritized (ACOX1, FGF5, FURIN, and MST1).

SNP = Single Nucleotide Polymorphism; SE = Standard error HWE_p = P-value for Hardy-Weinberg equilibrium test

Phenotype	Description	Group	SNP	Beta	SE	Odds tatio	P-value	Type	n_total	n_cases	n_controls	HWE_p	allele_freq
280.2	Iron deficiency anemia secondary to blood loss (chronic	hematopoietic	rs4932372	0.047	0.086	1.048	5.821E-01	logistic	382749	300	382449	0.997	0.331
425.1	Primary/intrinsic cardiomyopathies	circulatory system	rs4932372	-0.019	0.035	0.981	5.834E-01	logistic	418636	1866	416770	0.999	0.331
596.1	Bladder neck obstruction	genitourinary	rs4932372	-0.035	0.064	0.966	5.839E-01	logistic	402115	561	401554	0.995	0.331
740.2	Osteoarthrosis, generalized	musculoskeletal	rs4932372	-0.012	0.022	0.988	5.844E-01	logistic	350458	4600	345858	0.999	0.332
324	Other CNS infection and poliomyelitis	neurological	rs4932372	-0.044	0.081	0.957	5.847E-01	logistic	422418	347	422071	0.998	0.331
172.2	Other non-epithelial cancer of skin	neoplasms	rs4932372	0.014	0.025	1.014	5.849E-01	logistic	410817	3609	407208	0.999	0.331
578.1	Hematemesis	digestive	rs4932372	-0.022	0.041	0.978	5.853E-01	logistic	384647	1344	383303	0.994	0.331
473.4	Voice disturbance	respiratory	rs4932372	-0.049	0.090	0.952	5.867E-01	logistic	396801	284	396517	0.992	0.331
626.1	Irregular menstrual cycle/bleeding	genitourinary	rs4932372	0.013	0.024	1.013	5.879E-01	logistic	196900	4153	192747	0.961	0.331
477	Epistaxis or throat hemorrhage	respiratory	rs4932372	0.023	0.042	1.023	5.885E-01	logistic	397807	1290	396517	0.991	0.331
426.21	First degree AV block	circulatory system	rs4932372	0.019	0.035	1.019	5.887E-01	logistic	373661	1813	371848	0.999	0.331
443.1	Raynaud's syndrome	circulatory system	rs4932372	0.020	0.038	1.021	5.889E-01	logistic	407046	1577	405469	0.992	0.331
250.11	Type 1 diabetes with ketoacidosis	endocrine/metabolic	rs4932372	-0.052	0.096	0.949	5.897E-01	logistic	386885	248	386637	0.990	0.331
585	Renal failure	genitourinary	rs4932372	0.006	0.010	1.006	5.906E-01	logistic	403986	22924	381062	0.999	0.331
289.8	Polycythemia, secondary	hematopoietic	rs4932372	0.037	0.069	1.038	5.919E-01	logistic	407605	466	407139	0.998	0.331
550.6	Incisional hernia	digestive	rs4932372	0.037	0.070	1.038	5.931E-01	logistic	365851	457	365394	0.988	0.331
626.12	Excessive or frequent menstruation	genitourinary	rs4932372	-0.017	0.033	0.983	5.941E-01	logistic	194922	2175	192747	0.965	0.331
158	Neoplasm of unspecified nature of digestive system	neoplasms	rs4932372	0.041	0.077	1.042	5.964E-01	logistic	394238	376	393862	0.999	0.331
117	Mycoses	infectious diseases	rs4932372	-0.049	0.092	0.953	5.969E-01	logistic	418652	272	418380	0.997	0.331
427.8	Sinoatrial node dysfunction (Bradycardia)	circulatory system	rs4932372	-0.033	0.063	0.968	5.986E-01	logistic	372427	579	371848	0.999	0.331
292.6	Hallucinations	mental disorders	rs4932372	0.042	0.080	1.043	5.996E-01	logistic	405317	344	404973	0.995	0.331
290.11	Alzheimer's disease	mental disorders	rs4932372	0.016	0.031	1.016	6.001E-01	logistic	407367	2394	404973	0.996	0.331
591	Urinary tract infection	genitourinary	rs4932372	-0.007	0.014	0.993	6.010E-01	logistic	388723	11796	376927	0.997	0.331
348	Other conditions of brain	neurological	rs4932372	-0.030	0.058	0.970	6.016E-01	logistic	399717	670	399047	0.998	0.331
516.1	Hemoptysis	respiratory	rs4932372	0.025	0.049	1.026	6.038E-01	logistic	421320	931	420389	0.998	0.331
204.1	Lymphoid leukemia	neoplasms	rs4932372	-0.026	0.050	0.974	6.043E-01	logistic	416514	901	415613	0.998	0.331
204.4	Multiple myeloma	neoplasms	rs4932372	-0.023	0.045	0.977	6.051E-01	logistic	416757	1144	415613	0.998	0.331
441	Vascular insufficiency of intestine	circulatory system	rs4932372	-0.030	0.058	0.971	6.054E-01	logistic	406151	682	405469	0.992	0.331
456	Chronic venous insufficiency [CVI]	circulatory system	rs4932372	0.045	0.088	1.046	6.071E-01	logistic	381250	284	380966	0.985	0.332
535.8	Other specified gastritis	digestive	rs4932372	0.015	0.028	1.015	6.073E-01	logistic	365017	2803	362214	0.996	0.331
411.9	Other acute and subacute forms of ischemic heart disease	circulatory system	rs4932372	0.020	0.039	1.020	6.074E-01	logistic	372042	1492	370550	0.995	0.330
694	Dyschromia and Vitiligo	dermatologic	rs4932372	-0.045	0.087	0.956	6.084E-01	logistic	405505	299	405206	0.994	0.331
870	Open wounds of head; neck; and trunk	injuries & poisonings	rs4932372	-0.027	0.052	0.973	6.088E-01	logistic	420976	826	420150	0.998	0.331
579	Other symptoms involving abdomen and pelvis	digestive	rs4932372	0.024	0.046	1.024	6.090E-01	logistic	384340	1037	383303	0.994	0.331
743.2	Pathologic fracture	musculoskeletal	rs4932372	-0.025	0.049	0.975	6.093E-01	logistic	422164	930	421234	0.998	0.331
293.1	Swelling, mass, or lump in head and neck [Space-occupying lesion, intracranial NOS]	mental disorders	rs4932372	0.043	0.084	1.044	6.093E-01	logistic	420026	315	419711	0.997	0.331
159.3	Malignant neoplasm of gallbladder and extrahepatic bile ducts	neoplasms	rs4932372	-0.042	0.083	0.959	6.096E-01	logistic	394194	332	393862	0.999	0.331
426.23	Second degree AV block	circulatory system	rs4932372	-0.030	0.060	0.970	6.101E-01	logistic	372488	640	371848	0.999	0.331
395.3	Nonrheumatic tricuspid valve disorders	circulatory system	rs4932372	0.049	0.096	1.050	6.106E-01	logistic	401653	239	401414	0.999	0.331
272.9	Unspecified disorder of lipoid metabolism	endocrine/metabolic	rs4932372	-0.043	0.085	0.958	6.108E-01	logistic	356085	320	355765	0.997	0.330
331	Other cerebral degenerations	neurological	rs4932372	0.019	0.037	1.019	6.109E-01	logistic	400699	1652	399047	0.998	0.331
276.1	Electrolyte imbalance	endocrine/metabolic	rs4932372	-0.008	0.015	0.992	6.113E-01	logistic	406083	10511	395572	0.995	0.331
601.1	Prostatitis	genitourinary	rs4932372	-0.029	0.057	0.971	6.114E-01	logistic	157164	703	156461	0.859	0.333
458.9	Hypotension NOS	circulatory system	rs4932372	-0.012	0.023	0.988	6.114E-01	logistic	376953	4381	372572	0.994	0.331
172.3	Carcinoma in situ of skin	neoplasms	rs4932372	0.037	0.074	1.038	6.121E-01	logistic	407616	408	407208	0.999	0.331
292.4	Altered mental status	mental disorders	rs4932372	-0.013	0.026	0.987	6.121E-01	logistic	408434	3461	404973	0.992	0.331
159.4	Malignant neoplasm of retroperitoneum and peritoneum	neoplasms	rs4932372	-0.047	0.093	0.954	6.124E-01	logistic	394127	265	393862	0.999	0.331
510	Other diseases of lung	respiratory	rs4932372	0.020	0.040	1.020	6.133E-01	logistic	421711	1427	420284	0.998	0.331
780	Hypothermia/Chills	symptoms	rs4932372	-0.051	0.102	0.950	6.142E-01	logistic	423421	221	423200	0.998	0.331
751.22	Other specified congenital anomalies of kidney	congenital anomalies	rs4932372	0.040	0.080	1.041	6.158E-01	logistic	420601	348	420253	0.997	0.331
500.2	Pneumoconiosis	respiratory	rs4932372	0.039	0.077	1.039	6.166E-01	logistic	391432	375	391057	0.998	0.331
389.4	Tinnitus	sense organs	rs4932372	0.034	0.069	1.035	6.189E-01	logistic	408770	473	408297	0.998	0.331
750	Digestive congenital anomalies	congenital anomalies	rs4932372	0.036	0.073	1.037	6.201E-01	logistic	420674	421	420253	0.998	0.331
574.1	Cholelithiasis	digestive	rs4932372	-0.008	0.016	0.992	6.202E-01	logistic	404312	9265	395047	0.998	0.331
255	Disorders of adrenal glands	endocrine/metabolic	rs4932372	-0.027	0.054	0.974	6.211E-01	logistic	416034	788	415246	0.997	0.331
70.3	Viral hepatitis C	infectious diseases	rs4932372	-0.046	0.093	0.955	6.213E-01	logistic	415468	264	415204	0.999	0.331
285.22	Anemia in neoplastic disease	hematopoietic	rs4932372	0.040	0.082	1.041	6.232E-01	logistic	382780	331	382449	0.997	0.331
41.11	Methicillin sensitive Staphylococcus aureus	infectious diseases	rs4932372	-0.018	0.037	0.982	6.233E-01	logistic	398282	1635	396647	0.999	0.331
690	Erythematosquamous dermatosis	dermatologic	rs4932372	-0.029	0.060	0.971	6.252E-01	logistic	405831	625	405206	0.995	0.331
509.8	Dependence on respirator [Ventilator] or supplemental oxygen	respiratory	rs4932372	-0.028	0.057	0.973	6.268E-01	logistic	391755	698	391057	0.998	0.331
377	Disorders of optic nerve and visual pathways	sense organs	rs4932372	0.038	0.078	1.039	6.279E-01	logistic	412293	365	411928	0.995	0.331
602	Other disorders of prostate	genitourinary	rs4932372	0.039	0.081	1.040	6.280E-01	logistic	156802	341	156461	0.812	0.333
189.21	Malignant neoplasm of bladder	neoplasms	rs4932372	0.013	0.027	1.013	6.290E-01	logistic	420210	3023	417187	0.997	0.331
729	Other disorders of soft tissues	musculoskeletal	rs4932372	-0.046	0.096	0.955	6.296E-01	logistic	389423	249	389174	0.983	0.331
275.5	Disorders of calcium/phosphorus metabolism	endocrine/metabolic	rs4932372	-0.015	0.031	0.985	6.297E-01	logistic	418502	2317	416185	0.997	0.331
260	Protein-calorie malnutrition	endocrine/metabolic	rs4932372	0.037	0.077	1.038	6.303E-01	logistic	412473	376	412097	0.993	0.331
574.2	Calculus of bile duct	digestive	rs4932372	-0.047	0.098	0.954	6.312E-01	logistic	395287	240	395047	0.998	0.331
577.2	Chronic pancreatitis	digestive	rs4932372	0.029	0.061	1.029	6.354E-01	logistic	418611	603	418008	0.998	0.331
8.52	Intestinal infection due to C. difficile	infectious diseases	rs4932372	0.027	0.058	1.028	6.376E-01	logistic	417984	668	417316	0.998	0.331
368.91	Psychophysical visual disturbances	sense organs	rs4932372	-0.044	0.093	0.957	6.394E-01	logistic	415808	263	415545	0.998	0.331
686	Other local infections of skin and subcutaneous tissue	dermatologic	rs4932372	-0.022	0.046	0.979	6.396E-01	logistic	419554	1057	418497	0.997	0.331

ST31; PheWAS results for the lead cis-pQTLs of the 4 proteins prioritized (ACOX1, FGF5, FURIN, and MST1).

SNP = Single Nucleotide Polymorphism; SE = Standard error HWE_p = P-value for Hardy-Weinberg equilibrium test

Phenotype	Description	Group	SNP	Beta	SE	Odds tatio	P-value	Type	n_total	n_cases	n_controls	HWE_p	allele_freq
751.2	Congenital anomalies of urinary system	congenital anomalies	rs4932372	0.025	0.055	1.026	6.411E-01	logistic	421003	750	420253	0.997	0.331
420.3	Endocarditis	circulatory system	rs4932372	-0.028	0.061	0.973	6.472E-01	logistic	417383	613	416770	0.998	0.331
574.3	Cholecystitis without cholelithiasis	digestive	rs4932372	0.020	0.043	1.020	6.477E-01	logistic	396244	1197	395047	0.998	0.331
286	Coagulation defects	hematopoietic	rs4932372	0.022	0.049	1.023	6.483E-01	logistic	418481	928	417553	0.999	0.331
459	Other disorders of circulatory system	circulatory system	rs4932372	0.005	0.010	1.005	6.515E-01	logistic	395720	23148	372572	0.998	0.331
594.8	Renal colic	genitourinary	rs4932372	0.029	0.064	1.029	6.525E-01	logistic	412927	551	412376	0.997	0.331
252	Disorders of parathyroid gland	endocrine/metabolic	rs4932372	-0.019	0.043	0.981	6.552E-01	logistic	416485	1239	415246	0.998	0.331
371.1	Uveitis, noninfectious or NOS	sense organs	rs4932372	0.042	0.093	1.042	6.564E-01	logistic	411047	254	410793	0.995	0.331
394	Rheumatic disease of the heart valves	circulatory system	rs4932372	0.010	0.022	1.010	6.566E-01	logistic	406218	4804	401414	0.999	0.331
333	Extrapyramidal disease and abnormal movement disorders	neurological	rs4932372	0.019	0.043	1.019	6.568E-01	logistic	400270	1223	399047	0.997	0.331
751.21	Cystic kidney disease	congenital anomalies	rs4932372	0.034	0.076	1.034	6.578E-01	logistic	420636	383	420253	0.997	0.331
80	Postoperative infection	infectious diseases	rs4932372	0.015	0.034	1.015	6.585E-01	logistic	414741	1897	412844	0.996	0.331
735	Acquired foot deformities	musculoskeletal	rs4932372	0.009	0.019	1.009	6.602E-01	logistic	408734	6080	402654	0.994	0.331
504	Other alveolar and parietoalveolar pneumonopathy	respiratory	rs4932372	-0.026	0.059	0.975	6.602E-01	logistic	391718	661	391057	0.998	0.331
573.9	Abnormal serum enzyme levels	digestive	rs4932372	-0.045	0.103	0.956	6.639E-01	logistic	400489	215	400274	0.998	0.331
802	Fracture of pelvis	injuries & poisonings	rs4932372	-0.021	0.049	0.979	6.661E-01	logistic	397331	953	396378	0.998	0.331
277.4	Disorders of bilirubin excretion	endocrine/metabolic	rs4932372	0.030	0.069	1.030	6.681E-01	logistic	420403	462	419941	0.997	0.331
695.3	Rosacea	dermatologic	rs4932372	0.040	0.094	1.041	6.684E-01	logistic	405456	250	405206	0.992	0.331
756	Other congenital musculoskeletal anomalies	congenital anomalies	rs4932372	-0.021	0.048	0.980	6.689E-01	logistic	422256	974	421282	0.998	0.331
580	Nephritis; nephrosis; renal sclerosis	genitourinary	rs4932372	-0.020	0.046	0.981	6.697E-01	logistic	382137	1075	381062	1.000	0.331
595	Hydronephrosis	genitourinary	rs4932372	0.020	0.048	1.020	6.707E-01	logistic	413362	986	412376	0.997	0.331
371	Inflammation of the eye	sense organs	rs4932372	0.020	0.047	1.020	6.735E-01	logistic	411823	1030	410793	0.996	0.331
512.7	Shortness of breath	respiratory	rs4932372	-0.010	0.024	0.990	6.749E-01	logistic	408527	3928	404599	0.998	0.331
275.3	Disorders of magnesium metabolism	endocrine/metabolic	rs4932372	-0.016	0.038	0.984	6.752E-01	logistic	417729	1544	416185	0.997	0.331
185	Cancer of prostate	neoplasms	rs4932372	-0.006	0.015	0.994	6.757E-01	logistic	167540	11079	156461	0.860	0.332
300.9	Posttraumatic stress disorder	mental disorders	rs4932372	-0.039	0.094	0.962	6.776E-01	logistic	381881	259	381622	0.982	0.332
619	Noninflammatory female genital disorders	genitourinary	rs4932372	-0.013	0.032	0.987	6.783E-01	logistic	219175	2209	216966	0.983	0.331
750.2	Lower gastrointestinal congenital anomalies	congenital anomalies	rs4932372	-0.044	0.107	0.957	6.811E-01	logistic	420453	200	420253	0.998	0.331
342	Hemiplegia	neurological	rs4932372	0.013	0.031	1.013	6.813E-01	logistic	401449	2402	399047	0.998	0.331
523	Gingival and periodontal diseases	digestive	rs4932372	0.031	0.075	1.031	6.828E-01	logistic	409931	395	409536	0.997	0.331
195	Cancer, suspected or other	neoplasms	rs4932372	-0.007	0.017	0.993	6.853E-01	logistic	393669	7663	386006	0.999	0.332
560.4	Other intestinal obstruction	digestive	rs4932372	0.013	0.032	1.013	6.854E-01	logistic	336617	2132	334485	0.999	0.331
389.1	Sensorineural hearing loss	sense organs	rs4932372	-0.022	0.054	0.978	6.868E-01	logistic	409063	766	408297	0.998	0.331
571.6	Primary biliary cirrhosis	digestive	rs4932372	0.034	0.084	1.034	6.884E-01	logistic	400586	312	400274	0.998	0.331
340	Migraine	neurological	rs4932372	0.012	0.030	1.012	6.887E-01	logistic	407732	2545	405187	0.993	0.331
751	Genitourinary congenital anomalies	congenital anomalies	rs4932372	0.020	0.051	1.020	6.896E-01	logistic	421129	876	420253	0.997	0.331
395.6	Heart valve replaced	circulatory system	rs4932372	-0.012	0.030	0.988	6.900E-01	logistic	403924	2510	401414	0.999	0.331
465.2	Acute pharyngitis	respiratory	rs4932372	0.038	0.095	1.038	6.901E-01	logistic	419940	248	419692	0.998	0.331
585.1	Acute renal failure	genitourinary	rs4932372	-0.005	0.013	0.995	6.921E-01	logistic	394375	13313	381062	0.999	0.331
331.9	Cerebral degeneration, unspecified	neurological	rs4932372	0.019	0.048	1.019	6.944E-01	logistic	400009	962	399047	0.997	0.331
180.3	Cervical intraepithelial neoplasia [CIN] [Cervical dysplasia]	neoplasms	rs4932372	0.027	0.068	1.027	6.957E-01	logistic	196385	481	195904	0.983	0.331
242	Thyrotoxicosis with or without goiter	endocrine/metabolic	rs4932372	0.015	0.038	1.015	6.964E-01	logistic	393812	1582	392230	0.999	0.332
442	Other aneurysm	circulatory system	rs4932372	0.011	0.027	1.011	6.975E-01	logistic	408493	3024	405469	0.989	0.331
555.21	Ulcerative colitis (chronic)	digestive	rs4932372	0.030	0.076	1.030	6.980E-01	logistic	334866	381	334485	0.999	0.331
851	Complications of transplants and reattached limbs	injuries & poisonings	rs4932372	0.037	0.096	1.038	6.988E-01	logistic	416895	243	416652	0.998	0.331
535	Gastritis and duodenitis	digestive	rs4932372	-0.005	0.012	0.995	7.033E-01	logistic	378649	16435	362214	0.998	0.331
702.1	Actinic keratosis	dermatologic	rs4932372	0.019	0.049	1.019	7.052E-01	logistic	413997	925	413072	0.992	0.331
728	Disorders of muscle, ligament, and fascia	musculoskeletal	rs4932372	-0.035	0.094	0.965	7.064E-01	logistic	389432	258	389174	0.984	0.331
575.1	Cholangitis	digestive	rs4932372	-0.019	0.051	0.981	7.077E-01	logistic	395932	885	395047	0.997	0.331
475	Chronic sinusitis	respiratory	rs4932372	-0.018	0.049	0.982	7.080E-01	logistic	397468	951	396517	0.992	0.331
530	Diseases of esophagus	digestive	rs4932372	-0.003	0.009	0.997	7.087E-01	logistic	388733	31945	356788	0.997	0.331
368.1	Amblyopia	sense organs	rs4932372	0.025	0.067	1.025	7.110E-01	logistic	416041	496	415545	0.998	0.331
635.2	Antepartum hemorrhage, abruptio placentae, and placenta previa	pregnancy complications	rs4932372	0.039	0.105	1.039	7.136E-01	logistic	222797	205	222592	0.986	0.331
384	Other disorders of tympanic membrane	sense organs	rs4932372	-0.029	0.079	0.972	7.148E-01	logistic	419080	364	418716	0.996	0.331
580.1	Glomerulonephritis	genitourinary	rs4932372	-0.024	0.067	0.976	7.160E-01	logistic	381574	512	381062	1.000	0.331
41.2	Streptococcus infection	infectious diseases	rs4932372	0.014	0.039	1.014	7.207E-01	logistic	398155	1508	396647	0.999	0.331
531	Peptic ulcer (excl. esophageal)	digestive	rs4932372	0.009	0.024	1.009	7.209E-01	logistic	415329	3990	411339	0.998	0.331
626.8	Infertility, female	genitourinary	rs4932372	0.032	0.090	1.032	7.225E-01	logistic	193029	282	192747	0.971	0.331
764	Sciatica	symptoms	rs4932372	0.014	0.041	1.015	7.264E-01	logistic	419302	1316	417986	0.998	0.331
368.9	Subjective visual disturbances	sense organs	rs4932372	-0.023	0.067	0.977	7.274E-01	logistic	416057	512	415545	0.998	0.331
427.11	Paroxysmal supraventricular tachycardia	circulatory system	rs4932372	-0.011	0.033	0.989	7.294E-01	logistic	373959	2111	371848	0.999	0.331
636	Early or threatened labor; hemorrhage in early pregnancy	pregnancy complications	rs4932372	0.023	0.066	1.023	7.296E-01	logistic	223122	530	222592	0.988	0.331
327	Sleep disorders	neurological	rs4932372	-0.022	0.063	0.978	7.302E-01	logistic	413949	565	413384	0.997	0.331
627	Menopausal and postmenopausal disorders	genitourinary	rs4932372	0.010	0.029	1.010	7.315E-01	logistic	195450	2703	192747	0.978	0.331
480.5	Bronchopneumonia and lung abscess	respiratory	rs4932372	-0.024	0.073	0.976	7.378E-01	logistic	397229	429	396800	0.999	0.331
568	Other disorders of peritoneum	digestive	rs4932372	-0.013	0.039	0.987	7.401E-01	logistic	385305	1466	383839	0.995	0.331
585.34	Chronic Kidney Disease, Stage IV	genitourinary	rs4932372	0.015	0.044	1.015	7.409E-01	logistic	382213	1151	381062	1.000	0.331
509	Respiratory failure, insufficiency, arrest	respiratory	rs4932372	-0.006	0.020	0.994	7.411E-01	logistic	397038	5981	391057	0.998	0.331
556	Ulceration of the lower GI tract	digestive	rs4932372	-0.025	0.076	0.975	7.417E-01	logistic	334879	394	334485	0.999	0.331
323	Encephalitis	neurological	rs4932372	-0.026	0.081	0.974	7.431E-01	logistic	422420	349	422071	0.998	0.331
300.13	Phobia	mental disorders	rs4932372	0.020	0.061	1.020	7.459E-01	logistic	382219	597	381622	0.983	0.332

ST31; PheWAS results for the lead cis-pQTLs of the 4 proteins prioritized (ACOX1, FGF5, FURIN, and MST1).

SNP = Single Nucleotide Polymorphism; SE = Standard error HWE_p = P-value for Hardy-Weinberg equilibrium test

Phenotype	Description	Group	SNP	Beta	SE	Odds tatio	P-value	Type	n_total	n_cases	n_controls	HWE_p	allele_freq
747.12	Valvular heart disease/ heart chambers	congenital anomalies	rs4932372	0.029	0.089	1.029	7.462E-01	logistic	421875	280	421595	0.997	0.331
622.2	Mucous polyp of cervix	genitourinary	rs4932372	-0.032	0.098	0.969	7.473E-01	logistic	213810	238	213572	0.969	0.331
455	Hemorrhoids	circulatory system	rs4932372	-0.007	0.021	0.993	7.483E-01	logistic	386244	5278	380966	0.985	0.332
279.11	Deficiency of humoral immunity	endocrine/metabolic	rs4932372	0.030	0.096	1.031	7.509E-01	logistic	423118	242	422876	0.998	0.331
578	Gastrointestinal hemorrhage	digestive	rs4932372	-0.005	0.016	0.995	7.510E-01	logistic	392729	9426	383303	0.997	0.331
195.1	Malignant neoplasm, other	neoplasms	rs4932372	-0.006	0.017	0.995	7.526E-01	logistic	393640	7634	386006	0.998	0.332
180	Cervical cancer and dysplasia	neoplasms	rs4932372	0.009	0.030	1.009	7.531E-01	logistic	198422	2518	195904	0.988	0.331
696.42	Psoriatic arthropathy	dermatologic	rs4932372	-0.016	0.050	0.984	7.555E-01	logistic	400183	893	399290	0.994	0.331
145.2	Cancer of tongue	neoplasms	rs4932372	0.023	0.076	1.024	7.567E-01	logistic	420733	391	420342	0.998	0.331
228	Hemangioma and lymphangioma, any site	neoplasms	rs4932372	-0.020	0.065	0.980	7.602E-01	logistic	421655	529	421126	0.998	0.331
580.3	Nephritis and nephropathy without mention of glomerulonephritis	genitourinary	rs4932372	-0.027	0.088	0.974	7.619E-01	logistic	381356	294	381062	1.000	0.331
575	Other biliary tract disease	digestive	rs4932372	-0.007	0.025	0.993	7.631E-01	logistic	398734	3687	395047	0.997	0.331
318	Tobacco use disorder	mental disorders	rs4932372	-0.006	0.021	0.994	7.642E-01	logistic	408298	5005	403293	0.995	0.332
187.2	Malignant neoplasm of testis	neoplasms	rs4932372	0.007	0.022	1.007	7.653E-01	logistic	184014	4620	179394	0.947	0.332
522	Diseases of pulp and periapical tissues	digestive	rs4932372	-0.026	0.088	0.974	7.654E-01	logistic	409832	296	409536	0.998	0.331
352	Disorders of other cranial nerves	neurological	rs4932372	0.013	0.043	1.013	7.658E-01	logistic	403637	1222	402415	0.998	0.331
597	Other disorders of urethra and urinary tract	genitourinary	rs4932372	0.010	0.033	1.010	7.683E-01	logistic	403600	2046	401554	0.996	0.331
790.6	Other abnormal blood chemistry	symptoms	rs4932372	-0.029	0.098	0.972	7.705E-01	logistic	422077	237	421840	0.997	0.331
578.9	Hemorrhage of gastrointestinal tract	digestive	rs4932372	-0.008	0.028	0.992	7.718E-01	logistic	386245	2942	383303	0.994	0.331
429	Ill-defined descriptions and complications of heart disease	circulatory system	rs4932372	-0.024	0.082	0.976	7.725E-01	logistic	410037	334	409703	0.999	0.331
358.1	Myasthenia gravis	neurological	rs4932372	-0.025	0.088	0.975	7.746E-01	logistic	419463	295	419168	0.998	0.331
386.2	Peripheral or central vertigo	sense organs	rs4932372	-0.017	0.061	0.983	7.746E-01	logistic	410281	616	409665	0.997	0.331
961.1	Poisoning/allergy of sulfonamides	injuries & poisonings	rs4932372	0.017	0.059	1.017	7.748E-01	logistic	367807	634	367173	0.999	0.331
525	Other diseases of the teeth and supporting structures	digestive	rs4932372	-0.019	0.068	0.981	7.792E-01	logistic	410027	491	409536	0.998	0.331
427.12	Paroxysmal ventricular tachycardia	circulatory system	rs4932372	0.013	0.048	1.013	7.848E-01	logistic	372834	986	371848	0.999	0.331
270.3	Disorders of plasma protein metabolism	endocrine/metabolic	rs4932372	0.015	0.055	1.015	7.849E-01	logistic	422449	747	421702	0.998	0.331
501	Pneumonitis due to inhalation of food or vomitus	respiratory	rs4932372	-0.010	0.036	0.990	7.874E-01	logistic	392843	1786	391057	0.998	0.331
187.1	Malignant neoplasm of unspecified male genital organ	neoplasms	rs4932372	0.006	0.023	1.006	7.881E-01	logistic	183955	4561	179394	0.944	0.332
117.4	Aspergillosis	infectious diseases	rs4932372	-0.028	0.104	0.972	7.884E-01	logistic	418590	210	418380	0.997	0.331
270.32	Paraproteinemia	endocrine/metabolic	rs4932372	0.016	0.060	1.016	7.937E-01	logistic	422319	617	421702	0.998	0.331
189.2	Cancer of bladder	neoplasms	rs4932372	0.007	0.026	1.007	8.010E-01	logistic	420532	3345	417187	0.997	0.331
727.4	Ganglion and cyst of synovium, tendon, and bursa	musculoskeletal	rs4932372	0.017	0.069	1.017	8.021E-01	logistic	389641	467	389174	0.982	0.331
967	Adverse effects of sedatives or other central nervous system depressants and anesthetics	injuries & poisonings	rs4932372	0.024	0.100	1.024	8.089E-01	logistic	367398	225	367173	0.999	0.331
333.1	Essential tremor	neurological	rs4932372	0.016	0.065	1.016	8.089E-01	logistic	399579	532	399047	0.998	0.331
433.3	Cerebral ischemia	circulatory system	rs4932372	-0.005	0.023	0.995	8.118E-01	logistic	407003	4318	402685	0.998	0.331
332	Parkinson's disease	neurological	rs4932372	-0.007	0.028	0.993	8.128E-01	logistic	401966	2919	399047	0.998	0.331
429.2	Abnormal function study of cardiovascular system	circulatory system	rs4932372	-0.019	0.082	0.981	8.135E-01	logistic	410036	333	409703	0.999	0.331
596	Other disorders of bladder	genitourinary	rs4932372	-0.005	0.019	0.996	8.144E-01	logistic	407805	6251	401554	0.996	0.331
585.4	Chronic kidney disease, Stage I or II	genitourinary	rs4932372	-0.012	0.053	0.988	8.150E-01	logistic	381861	799	381062	0.999	0.331
714	Rheumatoid arthritis and other inflammatory polyarthropathies	musculoskeletal	rs4932372	-0.005	0.024	0.995	8.171E-01	logistic	412574	4067	408507	0.996	0.331
191.11	Cancer of brain	neoplasms	rs4932372	-0.012	0.054	0.988	8.197E-01	logistic	421187	790	420397	0.998	0.331
578.2	Blood in stool	digestive	rs4932372	-0.008	0.035	0.992	8.213E-01	logistic	385103	1800	383303	0.996	0.331
149	Cancer of larynx, pharynx, nasal cavities	neoplasms	rs4932372	-0.012	0.052	0.989	8.233E-01	logistic	421191	849	420342	0.998	0.331
721.8	Other allied disorders of spine	musculoskeletal	rs4932372	0.023	0.105	1.024	8.239E-01	logistic	408871	201	408670	0.996	0.331
442.11	Abdominal aortic aneurysm	circulatory system	rs4932372	-0.008	0.037	0.992	8.240E-01	logistic	407127	1658	405469	0.991	0.331
690.1	Seborrheic dermatitis	dermatologic	rs4932372	-0.013	0.061	0.987	8.295E-01	logistic	405818	612	405206	0.995	0.331
395.4	Nonrheumatic pulmonary valve disorders	circulatory system	rs4932372	0.018	0.083	1.018	8.300E-01	logistic	401735	321	401414	0.999	0.331
941	Adverse reaction to serum or vaccine	injuries & poisonings	rs4932372	0.021	0.100	1.021	8.310E-01	logistic	415212	225	414987	0.997	0.331
523.3	Periodontitis (acute or chronic)	digestive	rs4932372	0.021	0.098	1.021	8.315E-01	logistic	409770	234	409536	0.997	0.331
625	Pain and other symptoms associated with female genital organs	genitourinary	rs4932372	-0.019	0.088	0.981	8.318E-01	logistic	218575	291	218284	0.992	0.330
289.4	Lymphadenitis	hematopoietic	rs4932372	-0.008	0.036	0.992	8.319E-01	logistic	410013	1704	408309	0.998	0.331
433.1	Occlusion and stenosis of precerebral arteries	circulatory system	rs4932372	0.008	0.038	1.008	8.335E-01	logistic	404257	1572	402685	0.998	0.331
260.6	Anorexia	endocrine/metabolic	rs4932372	0.011	0.051	1.011	8.350E-01	logistic	412968	871	412097	0.994	0.331
696.41	Psoriasis vulgaris	dermatologic	rs4932372	0.007	0.033	1.007	8.361E-01	logistic	401395	2105	399290	0.994	0.331
250.1	Type 1 diabetes	endocrine/metabolic	rs4932372	-0.006	0.031	0.994	8.377E-01	logistic	389041	2404	386637	0.990	0.331
687.4	Disturbance of skin sensation	dermatologic	rs4932372	-0.008	0.040	0.992	8.388E-01	logistic	414113	1397	412716	0.995	0.331
528.1	Stomatitis and mucositis	digestive	rs4932372	0.020	0.097	1.020	8.404E-01	logistic	417947	237	417710	0.997	0.331
362.29	Macular degeneration (senile) of retina NOS	sense organs	rs4932372	-0.005	0.024	0.995	8.405E-01	logistic	401665	3908	397757	0.997	0.331
441.1	Acute vascular insufficiency of intestine	circulatory system	rs4932372	0.017	0.086	1.017	8.434E-01	logistic	405771	302	405469	0.991	0.331
159.2	Malignant neoplasm of small intestine, including duodenum	neoplasms	rs4932372	-0.018	0.094	0.982	8.442E-01	logistic	394117	255	393862	0.999	0.331
191	Manlignant and unknown neoplasms of brain and nervous system	neoplasms	rs4932372	0.009	0.048	1.010	8.443E-01	logistic	421363	966	420397	0.998	0.331
291	Other specified nonpsychotic and/or transient mental disorders	mental disorders	rs4932372	0.015	0.075	1.015	8.447E-01	logistic	405371	398	404973	0.994	0.331
557.1	Celiac disease	digestive	rs4932372	-0.007	0.035	0.993	8.455E-01	logistic	336383	1898	334485	0.999	0.331
255.2	Adrenal hypofunction	endocrine/metabolic	rs4932372	0.016	0.080	1.016	8.456E-01	logistic	415595	349	415246	0.998	0.331
255.21	Glucocorticoid deficiency	endocrine/metabolic	rs4932372	0.016	0.080	1.016	8.456E-01	logistic	415595	349	415246	0.998	0.331
714.1	Rheumatoid arthritis	musculoskeletal	rs4932372	-0.005	0.024	0.995	8.464E-01	logistic	412357	3850	408507	0.995	0.331
458	Hypotension	circulatory system	rs4932372	0.003	0.017	1.003	8.464E-01	logistic	380778	8206	372572	0.995	0.331
153.3	Malignant neoplasm of rectum, rectosigmoid junction, and anus	neoplasms	rs4932372	-0.005	0.028	0.995	8.475E-01	logistic	373609	2889	370720	0.997	0.332
737	Curvature of spine	musculoskeletal	rs4932372	-0.010	0.050	0.990	8.479E-01	logistic	403550	896	402654	0.994	0.331
500	Lung disease due to external agents	respiratory	rs4932372	0.012	0.060	1.012	8.486E-01	logistic	391675	618	391057	0.998	0.331
282	Hereditary hemolytic anemias	hematopoietic	rs4932372	-0.019	0.102	0.981	8.491E-01	logistic	382667	218	382449	0.997	0.331

ST31; PheWAS results for the lead cis-pQTLs of the 4 proteins prioritized (ACOX1, FGF5, FURIN, and MST1).

SNP = Single Nucleotide Polymorphism; SE = Standard error HWE_p = P-value for Hardy-Weinberg equilibrium test

Phenotype	Description	Group	SNP	Beta	SE	Odds tatio	P-value	Type	n_total	n_cases	n_controls	HWE_p	allele_freq
614	Inflammatory diseases of female pelvic organs	genitourinary	rs4932372	-0.007	0.038	0.993	8.497E-01	logistic	218979	1541	217438	0.990	0.331
112	Candidiasis	infectious diseases	rs4932372	0.007	0.038	1.007	8.514E-01	logistic	419935	1555	418380	0.998	0.331
687.1	Rash and other nonspecific skin eruption	dermatologic	rs4932372	-0.009	0.046	0.991	8.515E-01	logistic	413803	1087	412716	0.995	0.331
599.3	Dysuria	genitourinary	rs4932372	-0.015	0.082	0.985	8.591E-01	logistic	390438	332	390106	0.996	0.331
479	Other upper respiratory disease	respiratory	rs4932372	-0.013	0.073	0.987	8.599E-01	logistic	396937	420	396517	0.993	0.331
180.1	Cervical cancer	neoplasms	rs4932372	0.006	0.034	1.006	8.606E-01	logistic	197916	2012	195904	0.989	0.331
502	Postinflammatory pulmonary fibrosis	respiratory	rs4932372	-0.006	0.034	0.994	8.621E-01	logistic	393073	2016	391057	0.998	0.331
785	Abdominal pain	symptoms	rs4932372	-0.003	0.018	0.997	8.627E-01	logistic	401183	7225	393958	0.997	0.331
603	Other disorders of testis	genitourinary	rs4932372	-0.012	0.070	0.988	8.638E-01	logistic	162246	468	161778	0.762	0.332
726.1	Enthesopathy	musculoskeletal	rs4932372	0.004	0.026	1.004	8.643E-01	logistic	392491	3317	389174	0.981	0.331
965	Poisoning by analgesics, antipyretics, and antirheumatics	injuries & poisonings	rs4932372	0.004	0.022	1.004	8.649E-01	logistic	371918	4745	367173	0.999	0.331
289.5	Diseases of spleen	hematopoietic	rs4932372	-0.009	0.054	0.991	8.658E-01	logistic	409070	761	408309	0.999	0.331
396	Abnormal heart sounds	circulatory system	rs4932372	-0.008	0.049	0.992	8.679E-01	logistic	402357	943	401414	0.998	0.331
472	Chronic pharyngitis and nasopharyngitis	respiratory	rs4932372	-0.015	0.093	0.985	8.684E-01	logistic	396779	262	396517	0.992	0.331
473	Diseases of the larynx and vocal cords	respiratory	rs4932372	0.007	0.042	1.007	8.694E-01	logistic	397819	1302	396517	0.994	0.331
291.8	Alteration of consciousness	mental disorders	rs4932372	0.013	0.078	1.013	8.700E-01	logistic	405343	370	404973	0.994	0.331
189.11	Malignant neoplasm of kidney, except pelvis	neoplasms	rs4932372	0.007	0.044	1.007	8.715E-01	logistic	418324	1137	417187	0.998	0.331
530.13	Barrett's esophagus	digestive	rs4932372	-0.004	0.023	0.996	8.725E-01	logistic	361195	4407	356788	0.993	0.331
345	Epilepsy, recurrent seizures, convulsions	neurological	rs4932372	-0.004	0.024	0.996	8.750E-01	logistic	403151	4104	399047	0.997	0.331
344	Other paralytic syndromes	neurological	rs4932372	-0.009	0.058	0.991	8.763E-01	logistic	399714	667	399047	0.998	0.331
530.11	GERD	digestive	rs4932372	-0.002	0.013	0.998	8.776E-01	logistic	371656	14868	356788	0.997	0.331
189.1	Cancer of kidney and renal pelvis	neoplasms	rs4932372	0.007	0.043	1.007	8.778E-01	logistic	418392	1205	417187	0.998	0.331
527	Diseases of the salivary glands	digestive	rs4932372	0.015	0.100	1.015	8.787E-01	logistic	417933	223	417710	0.998	0.331
250.4	Abnormal glucose	endocrine/metabolic	rs4932372	-0.007	0.047	0.993	8.795E-01	logistic	387669	1032	386637	0.987	0.331
709.2	Sicca syndrome	dermatologic	rs4932372	-0.008	0.055	0.992	8.795E-01	logistic	404638	739	403899	0.993	0.331
279.1	Immunity deficiency	endocrine/metabolic	rs4932372	0.013	0.083	1.013	8.799E-01	logistic	423199	323	422876	0.998	0.331
509.2	Respiratory insufficiency	respiratory	rs4932372	-0.006	0.041	0.994	8.811E-01	logistic	392402	1345	391057	0.998	0.331
371.3	Inflammation of eyelids	sense organs	rs4932372	0.009	0.058	1.009	8.828E-01	logistic	411465	672	410793	0.996	0.331
446	Polyarteritis nodosa and allied conditions	circulatory system	rs4932372	0.006	0.044	1.006	8.850E-01	logistic	406656	1187	405469	0.990	0.331
284	Aplastic anemia	hematopoietic	rs4932372	0.008	0.055	1.008	8.887E-01	logistic	383179	730	382449	0.997	0.331
573.7	Abnormal results of function study of liver	digestive	rs4932372	0.003	0.024	1.003	8.901E-01	logistic	404090	3816	400274	0.997	0.331
965.1	Opiates and related narcotics causing adverse effects in therapeutic use	injuries & poisonings	rs4932372	0.004	0.032	1.004	8.902E-01	logistic	369447	2274	367173	0.999	0.331
577.3	Cyst and pseudocyst of pancreas	digestive	rs4932372	0.009	0.066	1.009	8.936E-01	logistic	418522	514	418008	0.998	0.331
626.14	Irregular menstrual bleeding	genitourinary	rs4932372	-0.010	0.076	0.990	8.939E-01	logistic	193146	399	192747	0.966	0.331
571.81	Portal hypertension	digestive	rs4932372	-0.007	0.051	0.993	8.947E-01	logistic	401135	861	400274	0.998	0.331
770	Myalgia and myositis unspecified	symptoms	rs4932372	-0.005	0.035	0.995	8.970E-01	logistic	422525	1839	420686	0.998	0.331
601	Inflammatory diseases of prostate	genitourinary	rs4932372	-0.006	0.050	0.994	9.008E-01	logistic	157382	921	156461	0.846	0.333
381.1	Otitis media	sense organs	rs4932372	0.007	0.059	1.007	9.009E-01	logistic	419355	639	418716	0.997	0.331
695.7	Prurigo and Lichen	dermatologic	rs4932372	-0.011	0.086	0.989	9.010E-01	logistic	405510	304	405206	0.994	0.331
198.6	Secondary malignancy of bone	neoplasms	rs4932372	0.003	0.022	1.003	9.039E-01	logistic	390762	4756	386006	0.998	0.332
362	Other retinal disorders	sense organs	rs4932372	0.002	0.015	1.002	9.043E-01	logistic	407698	9941	397757	0.997	0.331
724.2	Disorders of coccyx	musculoskeletal	rs4932372	-0.012	0.103	0.988	9.063E-01	logistic	408885	215	408670	0.996	0.331
561	Symptoms involving digestive system	digestive	rs4932372	-0.002	0.021	0.998	9.069E-01	logistic	339539	5054	334485	0.999	0.331
350.3	Lack of coordination	neurological	rs4932372	-0.010	0.088	0.990	9.070E-01	logistic	420499	289	420210	0.998	0.331
726	Peripheral enthesopathies and allied syndromes	musculoskeletal	rs4932372	0.002	0.015	1.002	9.077E-01	logistic	399760	10586	389174	0.986	0.331
250.23	Type 2 diabetes with ophthalmic manifestations	endocrine/metabolic	rs4932372	-0.004	0.034	0.996	9.079E-01	logistic	388590	1953	386637	0.988	0.331
451.2	Phlebitis and thrombophlebitis of lower extremities	circulatory system	rs4932372	-0.008	0.068	0.992	9.088E-01	logistic	381453	487	380966	0.983	0.332
573.5	Jaundice (not of newborn)	digestive	rs4932372	-0.007	0.057	0.993	9.088E-01	logistic	400970	696	400274	0.998	0.331
150	Cancer of esophagus	neoplasms	rs4932372	-0.005	0.040	0.995	9.092E-01	logistic	395291	1429	393862	0.999	0.331
702	Degenerative skin conditions and other dermatoses	dermatologic	rs4932372	0.004	0.035	1.004	9.098E-01	logistic	407215	1867	405348	0.995	0.331
781	Symptoms involving nervous and musculoskeletal system	symptoms	rs4932372	-0.002	0.017	0.998	9.106E-01	logistic	419375	7893	411482	0.998	0.331
706.2	Sebaceous cyst	dermatologic	rs4932372	0.004	0.040	1.004	9.129E-01	logistic	413393	1394	411999	0.996	0.331
246	Other disorders of thyroid	endocrine/metabolic	rs4932372	0.009	0.086	1.009	9.154E-01	logistic	392532	302	392230	0.999	0.332
292.3	Memory loss	mental disorders	rs4932372	0.007	0.067	1.007	9.169E-01	logistic	405478	505	404973	0.994	0.331
196	Radiotherapy	neoplasms	rs4932372	0.004	0.038	1.004	9.174E-01	logistic	387589	1583	386006	0.996	0.332
724.1	Disorders of sacrum	musculoskeletal	rs4932372	-0.010	0.099	0.990	9.177E-01	logistic	408900	230	408670	0.996	0.331
740.12	Osteoarthrosis, localized, secondary	musculoskeletal	rs4932372	-0.007	0.073	0.993	9.180E-01	logistic	346286	428	345858	0.998	0.332
735.23	Hallux rigidus	musculoskeletal	rs4932372	-0.009	0.084	0.992	9.194E-01	logistic	402973	319	402654	0.993	0.331
760	Back pain	symptoms	rs4932372	0.002	0.021	1.002	9.198E-01	logistic	414925	5193	409732	0.999	0.331
568.1	Peritoneal adhesions (postoperative) (postinfection)	digestive	rs4932372	0.004	0.042	1.004	9.210E-01	logistic	385136	1297	383839	0.995	0.331
155.1	Malignant neoplasm of liver, primary	neoplasms	rs4932372	-0.008	0.083	0.992	9.225E-01	logistic	394187	325	393862	0.999	0.331
470	Septal Deviations/Turbinate Hypertrophy	respiratory	rs4932372	0.004	0.037	1.004	9.228E-01	logistic	398163	1646	396517	0.994	0.331
594.2	Calculus of lower urinary tract	genitourinary	rs4932372	-0.006	0.063	0.994	9.247E-01	logistic	412949	573	412376	0.997	0.331
41	Bacterial infection NOS	infectious diseases	rs4932372	0.001	0.016	1.001	9.264E-01	logistic	406056	9409	396647	0.999	0.331
736	Other acquired deformities of limbs	musculoskeletal	rs4932372	-0.004	0.049	0.996	9.268E-01	logistic	403605	951	402654	0.993	0.331
362.2	Degeneration of macula and posterior pole of retina	sense organs	rs4932372	0.001	0.016	1.001	9.273E-01	logistic	406927	9170	397757	0.997	0.331
594.3	Calculus of ureter	genitourinary	rs4932372	0.003	0.037	1.003	9.287E-01	logistic	414010	1634	412376	0.997	0.331
394.1	Mitral valve stenosis and aortic valve stenosis	circulatory system	rs4932372	0.004	0.045	1.004	9.288E-01	logistic	402530	1116	401414	0.999	0.331
743.21	Pathologic fracture of vertebrae	musculoskeletal	rs4932372	-0.005	0.060	0.995	9.307E-01	logistic	421861	627	421234	0.998	0.331
696.4	Psoriasis	dermatologic	rs4932372	-0.002	0.028	0.998	9.348E-01	logistic	402177	2887	399290	0.994	0.331
528	Diseases of the oral soft tissues, excluding lesions specific for gingiva and tongue	digestive	rs4932372	0.006	0.070	1.006	9.358E-01	logistic	418169	459	417710	0.998	0.331

ST31; PheWAS results for the lead cis-pQTLs of the 4 proteins prioritized (ACOX1, FGF5, FURIN, and MST1).

SNP = Single Nucleotide Polymorphism; SE = Standard error HWE_p = P-value for Hardy-Weinberg equilibrium test

Phenotype	Description	Group	SNP	Beta	SE	Odds tatio	P-value	Type	n_total	n_cases	n_controls	HWE_p	allele_freq
766	Neuralgia, neuritis, and radiculitis NOS	symptoms	rs4932372	-0.007	0.090	0.993	9.372E-01	logistic	418267	281	417986	0.998	0.331
290.13	Senile dementia	mental disorders	rs4932372	-0.002	0.032	0.998	9.388E-01	logistic	407253	2280	404973	0.996	0.331
353	Nerve root and plexus disorders	neurological	rs4932372	0.007	0.086	1.007	9.390E-01	logistic	402720	305	402415	0.998	0.331
442.1	Aortic aneurysm	circulatory system	rs4932372	-0.002	0.030	0.998	9.394E-01	logistic	407951	2482	405469	0.989	0.331
279	Disorders involving the immune mechanisr	endocrine/metabolic	rs4932372	0.006	0.078	1.006	9.400E-01	logistic	423247	371	422876	0.998	0.331
275	Disorders of mineral metabolism	endocrine/metabolic	rs4932372	-0.002	0.022	0.998	9.406E-01	logistic	420958	4773	416185	0.997	0.331
597.1	Urethral stricture (not specified as infectious)	genitourinary	rs4932372	0.003	0.035	1.003	9.418E-01	logistic	403370	1816	401554	0.995	0.331
211	Benign neoplasm of other parts of digestive systen	neoplasms	rs4932372	-0.002	0.026	0.998	9.421E-01	logistic	400517	3467	397050	0.999	0.331
427.7	Tachycardia NOS	circulatory system	rs4932372	0.003	0.035	1.003	9.421E-01	logistic	373741	1893	371848	0.999	0.331
701.3	Circumscribed scleroderma	dermatologic	rs4932372	-0.007	0.100	0.993	9.421E-01	logistic	415410	224	415186	0.997	0.331
735.2	Acquired toe deformities	musculoskeletal	rs4932372	0.002	0.034	1.002	9.432E-01	logistic	404574	1920	402654	0.994	0.331
395	Heart valve disorders	circulatory system	rs4932372	-0.001	0.016	0.999	9.442E-01	logistic	411117	9703	401414	0.999	0.331
626	Disorders of menstruation and other abnormal bleeding from female genital trac	genitourinary	rs4932372	-0.001	0.020	0.999	9.447E-01	logistic	198823	6076	192747	0.974	0.331
198.7	Secondary malignant neoplasm of skir	neoplasms	rs4932372	-0.006	0.093	0.994	9.453E-01	logistic	386268	262	386006	0.996	0.332
560	Intestinal obstruction without mention of hernia	digestive	rs4932372	-0.002	0.025	0.998	9.462E-01	logistic	338110	3625	334485	0.999	0.331
614.1	Pelvic peritoneal adhesions, female (postoperative) (postinfection	genitourinary	rs4932372	-0.005	0.076	0.995	9.477E-01	logistic	217833	395	217438	0.990	0.331
681.1	Cellulitis and abscess of fingers/toes	dermatologic	rs4932372	0.007	0.099	1.007	9.478E-01	logistic	418724	227	418497	0.997	0.331
384.4	Perforation of tympanic membrane	sense organs	rs4932372	0.006	0.095	1.006	9.487E-01	logistic	418963	247	418716	0.996	0.331
420	Carditis	circulatory system	rs4932372	-0.002	0.032	0.998	9.508E-01	logistic	418942	2172	416770	0.997	0.331
560.1	Paralytic ileus	digestive	rs4932372	-0.005	0.075	0.995	9.508E-01	logistic	334880	395	334485	0.999	0.331
737.3	Kyphoscoliosis and scoliosis	musculoskeletal	rs4932372	-0.003	0.053	0.997	9.509E-01	logistic	403443	789	402654	0.993	0.331
281.13	Folate-deficiency anemia	hematopoietic	rs4932372	0.005	0.077	1.005	9.530E-01	logistic	382829	380	382449	0.997	0.331
782.3	Edema	symptoms	rs4932372	-0.002	0.030	0.998	9.530E-01	logistic	419509	2451	417058	0.998	0.331
622	Polyp of female genital organs	genitourinary	rs4932372	-0.002	0.030	0.998	9.534E-01	logistic	216102	2530	213572	0.972	0.331
531.4	Peptic ulcer, site unspecified	digestive	rs4932372	0.006	0.100	1.006	9.546E-01	logistic	411565	226	411339	0.998	0.331
374.3	Ptosis of eyelid	sense organs	rs4932372	-0.002	0.043	0.998	9.557E-01	logistic	411997	1204	410793	0.996	0.331
727	Other disorders of synovium, tendon, and bursa	musculoskeletal	rs4932372	0.001	0.029	1.001	9.596E-01	logistic	391842	2668	389174	0.971	0.331
446.9	Arteritis NOS	circulatory system	rs4932372	-0.004	0.079	0.996	9.601E-01	logistic	405830	361	405469	0.990	0.331
798	Malaise and fatigue	symptoms	rs4932372	0.001	0.023	1.001	9.603E-01	logistic	418584	4264	414320	0.998	0.331
420.2	Pericarditis	circulatory system	rs4932372	0.002	0.039	1.002	9.605E-01	logistic	418236	1466	416770	0.998	0.331
528.11	Stomatitis and mucositis (ulcerative)	digestive	rs4932372	-0.005	0.104	0.995	9.641E-01	logistic	417919	209	417710	0.997	0.331
191.1	Cancer of brain and nervous system	neoplasms	rs4932372	-0.002	0.052	0.998	9.661E-01	logistic	421229	832	420397	0.998	0.331
567	Peritonitis and retroperitoneal infections	digestive	rs4932372	-0.002	0.056	0.998	9.664E-01	logistic	384568	729	383839	0.993	0.331
990	Effects radiation NOS	injuries & poisonings	rs4932372	-0.001	0.018	0.999	9.671E-01	logistic	408877	6919	401958	0.999	0.331
579.8	Nonspecific abnormal findings in stool contents	digestive	rs4932372	0.002	0.060	1.002	9.694E-01	logistic	383921	618	383303	0.994	0.331
575.8	Other disorders of biliary tract	digestive	rs4932372	0.002	0.046	1.002	9.700E-01	logistic	396103	1056	395047	0.998	0.331
227	Benign neoplasm of other endocrine glands and related structures	neoplasms	rs4932372	0.002	0.056	1.002	9.706E-01	logistic	421596	709	420887	0.998	0.331
452	Other venous embolism and thrombosis	circulatory system	rs4932372	0.003	0.083	1.003	9.728E-01	logistic	381290	324	380966	0.986	0.332
603.1	Hydrocele	genitourinary	rs4932372	-0.002	0.071	0.998	9.731E-01	logistic	162225	447	161778	0.761	0.332
696	Psoriasis and related disorders	dermatologic	rs4932372	-0.001	0.028	0.999	9.741E-01	logistic	402206	2916	399290	0.995	0.331
365.1	Open-angle glaucoma	sense organs	rs4932372	-0.001	0.038	0.999	9.785E-01	logistic	399325	1562	397763	0.998	0.331
367.1	Myopia	sense organs	rs4932372	0.001	0.041	1.001	9.787E-01	logistic	412331	1325	411006	0.997	0.331
427.1	Paroxysmal tachycardia, unspecified	circulatory system	rs4932372	0.001	0.027	1.001	9.788E-01	logistic	374981	3133	371848	0.999	0.331
300.1	Anxiety disorder	mental disorders	rs4932372	0.000	0.016	1.000	9.795E-01	logistic	390805	9183	381622	0.989	0.332
8.6	Viral Enteritis	infectious diseases	rs4932372	-0.002	0.067	0.998	9.811E-01	logistic	417821	505	417316	0.998	0.331
569	Other disorders of intestine	digestive	rs4932372	0.001	0.032	1.001	9.818E-01	logistic	385990	2151	383839	0.993	0.331
204	Leukemia	neoplasms	rs4932372	0.001	0.027	1.001	9.822E-01	logistic	418621	3008	415613	0.998	0.331
189	Cancer of urinary organs (incl. kidney and bladder)	neoplasms	rs4932372	0.000	0.022	1.000	9.840E-01	logistic	421760	4573	417187	0.997	0.331
513	Respiratory abnormalities	respiratory	rs4932372	-0.001	0.072	0.999	9.877E-01	logistic	423122	431	422691	0.998	0.331
331.1	Hydrocephalus	neurological	rs4932372	0.001	0.060	1.001	9.881E-01	logistic	399668	621	399047	0.998	0.331
593	Hematuria	genitourinary	rs4932372	0.000	0.021	1.000	9.884E-01	logistic	381996	5069	376927	0.993	0.331
389	Hearing loss	sense organs	rs4932372	0.000	0.018	1.000	9.886E-01	logistic	415421	7124	408297	0.999	0.331
798.1	Chronic fatigue syndrome	symptoms	rs4932372	-0.001	0.060	0.999	9.920E-01	logistic	414937	617	414320	0.998	0.331
275.1	Disorders of iron metabolism	hematopoietic	rs4932372	0.000	0.046	1.000	9.926E-01	logistic	417274	1089	416185	0.997	0.331
172	Skin cancer	neoplasms	rs4932372	0.000	0.019	1.000	9.933E-01	logistic	413263	6055	407208	0.999	0.331
585.2	Renal failure NOS	genitourinary	rs4932372	0.000	0.055	1.000	9.934E-01	logistic	381806	744	381062	1.000	0.331
707.1	Decubitus ulcer	dermatologic	rs4932372	0.000	0.028	1.000	9.936E-01	logistic	419126	2996	416130	0.997	0.331
804	Fracture of hand or wrist	injuries & poisonings	rs4932372	0.000	0.062	1.000	9.940E-01	logistic	396955	577	396378	0.998	0.331
227.3	Benign neoplasm of pituitary gland and craniopharyngeal duct (pouch	neoplasms	rs4932372	0.001	0.079	1.001	9.941E-01	logistic	421248	361	420887	0.998	0.331
508	Pulmonary collapse; interstitial and compensatory emphysema	respiratory	rs4932372	0.000	0.024	1.000	9.943E-01	logistic	394890	3833	391057	0.998	0.331
348.2	Cerebral edema and compression of brain	neurological	rs4932372	0.000	0.077	1.000	9.970E-01	logistic	399425	378	399047	0.998	0.331
706	Diseases of sebaceous glands	dermatologic	rs4932372	0.000	0.040	1.000	9.972E-01	logistic	413434	1435	411999	0.996	0.331
509.1	Respiratory failure	respiratory	rs4932372	0.000	0.023	1.000	9.973E-01	logistic	395437	4380	391057	0.998	0.331
425.12	Other hypertrophic cardiomyopathy	circulatory system	rs4932372	0.000	0.091	1.000	9.981E-01	logistic	417044	274	416770	0.998	0.331
38.1	Gram negative septicemia	infectious diseases	rs6814407	0.319	0.100	1.376	1.398E-03	logistic	396961	314	396647	1.000	0.150
433.2	Occlusion of cerebral arteries	circulatory system	rs6814407	0.095	0.031	1.100	2.005E-03	logistic	406563	3878	402685	1.000	0.150
695.3	Rosacea	dermatologic	rs6814407	0.334	0.112	1.396	2.775E-03	logistic	405456	250	405206	1.000	0.150
433.21	Cerebral artery occlusion, with cerebral infarctior	circulatory system	rs6814407	0.093	0.031	1.097	2.952E-03	logistic	406495	3810	402685	1.000	0.150
790	Nonspecific findings on examination of blood	symptoms	rs6814407	0.328	0.110	1.388	3.018E-03	logistic	422095	255	421840	1.000	0.150
381.1	Otitis media	sense organs	rs6814407	0.204	0.073	1.226	5.056E-03	logistic	419355	639	418716	1.000	0.150
381	Otitis media and Eustachian tube disorders	sense organs	rs6814407	0.173	0.064	1.189	6.673E-03	logistic	419568	852	418716	1.000	0.150

ST31; PheWAS results for the lead cis-pQTLs of the 4 proteins prioritized (ACOX1, FGF5, FURIN, and MST1).
SNP = Single Nucleotide Polymorphism; SE = Standard error HWE_p = P-value for Hardy-Weinberg equilibrium test

Phenotype	Description	Group	SNP	Beta	SE	Odds tatio	P-value	Type	n_total	n_cases	n_controls	HWE_p	allele_freq
585.2	Renal failure NOS	genitourinary	rs6814407	-0.207	0.078	0.813	7.834E-03	logistic	381806	744	381062	1.000	0.150
785	Abdominal pain	symptoms	rs6814407	0.060	0.023	1.062	9.196E-03	logistic	401183	7225	393958	1.000	0.150
426.2	Atrioventricular [AV] block	circulatory system	rs6814407	0.081	0.032	1.085	1.031E-02	logistic	375580	3732	371848	1.000	0.150
426.24	Atrioventricular block, complete	circulatory system	rs6814407	0.151	0.060	1.163	1.148E-02	logistic	372833	985	371848	1.000	0.150
965.1	Opiates and related narcotics causing adverse effects in therapeutic use	injuries & poisonings	rs6814407	0.101	0.040	1.106	1.184E-02	logistic	369447	2274	367173	1.000	0.150
411.2	Myocardial infarction	circulatory system	rs6814407	0.042	0.017	1.043	1.266E-02	logistic	385085	14535	370550	1.000	0.150
579.8	Nonspecific abnormal findings in stool contents	digestive	rs6814407	0.185	0.074	1.204	1.274E-02	logistic	383921	618	383303	1.000	0.150
41	Bacterial infection NOS	infectious diseases	rs6814407	0.050	0.020	1.052	1.306E-02	logistic	406056	9409	396647	1.000	0.150
395.6	Heart valve replaced	circulatory system	rs6814407	0.093	0.038	1.098	1.444E-02	logistic	403924	2510	401414	1.000	0.150
454.11	Varicose veins of lower extremity, symptomtic	circulatory system	rs6814407	-0.210	0.087	0.811	1.634E-02	logistic	381560	594	380966	1.000	0.150
202.21	Nodular lymphoma	neoplasms	rs6814407	0.205	0.085	1.227	1.641E-02	logistic	416077	464	415613	1.000	0.150
204.21	Myeloid leukemia, acute	neoplasms	rs6814407	-0.208	0.088	0.813	1.857E-02	logistic	416195	582	415613	1.000	0.150
300.12	Agorophobia, social phobia, and panic disorder	mental disorders	rs6814407	0.167	0.072	1.182	2.059E-02	logistic	382286	664	381622	1.000	0.150
331.9	Cerebral degeneration, unspecified	neurological	rs6814407	-0.155	0.067	0.856	2.126E-02	logistic	400009	962	399047	1.000	0.150
253.2	Pituitary hypofunction	endocrine/metabolic	rs6814407	0.246	0.107	1.279	2.148E-02	logistic	415534	288	415246	1.000	0.150
145	Cancer of mouth	neoplasms	rs6814407	-0.187	0.081	0.830	2.180E-02	logistic	421016	674	420342	1.000	0.150
420	Carditis	circulatory system	rs6814407	0.094	0.041	1.098	2.234E-02	logistic	418942	2172	416770	1.000	0.150
709.2	Sicca syndrome	dermatologic	rs6814407	0.156	0.069	1.169	2.341E-02	logistic	404638	739	403899	1.000	0.150
695.2	Bullous dermatoses	dermatologic	rs6814407	-0.350	0.158	0.704	2.624E-02	logistic	405408	202	405206	1.000	0.150
204.2	Myeloid leukemia	neoplasms	rs6814407	-0.170	0.077	0.844	2.673E-02	logistic	416360	747	415613	1.000	0.150
225	Benign neoplasm of brain and other parts of nervous system	neoplasms	rs6814407	0.143	0.065	1.154	2.754E-02	logistic	421233	836	420397	1.000	0.150
364.4	Corneal degenerations	sense organs	rs6814407	0.122	0.056	1.130	2.825E-02	logistic	398917	1154	397763	1.000	0.150
446.9	Arteritis NOS	circulatory system	rs6814407	0.208	0.096	1.232	3.061E-02	logistic	405830	361	405469	1.000	0.150
458.9	Hypotension NOS	circulatory system	rs6814407	0.063	0.029	1.065	3.139E-02	logistic	376953	4381	372572	1.000	0.150
225.1	Benign neoplasm of brain, cranial nerves, meninges	neoplasms	rs6814407	0.142	0.066	1.152	3.159E-02	logistic	421204	807	420397	1.000	0.150
564.1	Irritable Bowel Syndrome	digestive	rs6814407	0.061	0.029	1.063	3.187E-02	logistic	339145	4660	334485	1.000	0.150
939	Atopic/contact dermatitis due to other or unspecified	dermatologic	rs6814407	-0.098	0.046	0.906	3.284E-02	logistic	416950	1963	414987	1.000	0.150
916	Contusion	injuries & poisonings	rs6814407	-0.117	0.055	0.889	3.339E-02	logistic	421588	1393	420195	1.000	0.150
200.1	Polycythemia vera	neoplasms	rs6814407	-0.194	0.092	0.824	3.620E-02	logistic	408705	523	408182	1.000	0.150
70.4	Chronic hepatitis	infectious diseases	rs6814407	0.225	0.108	1.252	3.728E-02	logistic	415491	287	415204	1.000	0.150
364	Corneal opacity and other disorders of cornea	sense organs	rs6814407	0.094	0.046	1.099	3.899E-02	logistic	399513	1750	397763	1.000	0.150
459.9	Circulatory disease NEC	circulatory system	rs6814407	0.028	0.013	1.028	3.978E-02	logistic	395548	22976	372572	1.000	0.150
193	Thyroid cancer	neoplasms	rs6814407	-0.216	0.105	0.806	3.987E-02	logistic	421300	413	420887	1.000	0.150
426.21	First degree AV block	circulatory system	rs6814407	0.092	0.045	1.096	4.118E-02	logistic	373661	1813	371848	1.000	0.150
450	Noninfectious disorders of lymphatic channels	circulatory system	rs6814407	-0.123	0.060	0.885	4.218E-02	logistic	422720	1167	421553	1.000	0.150
295.1	Schizophrenia	mental disorders	rs6814407	-0.164	0.081	0.849	4.248E-02	logistic	382292	670	381622	1.000	0.150
201	Hodgkin's disease	neoplasms	rs6814407	0.220	0.109	1.246	4.308E-02	logistic	415895	282	415613	1.000	0.150
514	Abnormal findings examination of lungs	respiratory	rs6814407	-0.089	0.044	0.915	4.338E-02	logistic	419804	2147	417657	1.000	0.150
295	Schizophrenia and other psychotic disorders	mental disorders	rs6814407	-0.144	0.072	0.866	4.486E-02	logistic	382460	838	381622	1.000	0.150
569.1	Toxic gastroenteritis and colitis	digestive	rs6814407	-0.239	0.121	0.787	4.753E-02	logistic	384157	318	383839	1.000	0.150
189.2	Cancer of bladder	neoplasms	rs6814407	-0.069	0.035	0.933	4.849E-02	logistic	420532	3345	417187	1.000	0.150
362	Other retinal disorders	sense organs	rs6814407	0.039	0.020	1.040	4.856E-02	logistic	407698	9941	397757	1.000	0.150
459	Other disorders of circulatory system	circulatory system	rs6814407	0.026	0.013	1.027	4.865E-02	logistic	395720	23148	372572	1.000	0.150
374.3	Ptosis of eyelid	sense organs	rs6814407	0.108	0.055	1.114	4.922E-02	logistic	411997	1204	410793	1.000	0.150
433	Cerebrovascular disease	circulatory system	rs6814407	0.036	0.019	1.037	5.209E-02	logistic	414008	11323	402685	1.000	0.150
189.21	Malignant neoplasm of bladder	neoplasms	rs6814407	-0.072	0.037	0.931	5.219E-02	logistic	420210	3023	417187	1.000	0.150
446	Polyarteritis nodosa and allied conditions	circulatory system	rs6814407	0.107	0.055	1.113	5.245E-02	logistic	406656	1187	405469	1.000	0.150
317.11	Alcoholic liver damage	mental disorders	rs6814407	-0.115	0.059	0.891	5.264E-02	logistic	404493	1200	403293	1.000	0.150
244.1	Secondary hypothyroidism	endocrine/metabolic	rs6814407	-0.114	0.059	0.892	5.291E-02	logistic	393443	1213	392230	1.000	0.150
202.24	Large cell lymphoma	neoplasms	rs6814407	0.110	0.057	1.117	5.355E-02	logistic	416717	1104	415613	1.000	0.150
428	Congestive heart failure; nonhypertensive	circulatory system	rs6814407	0.042	0.022	1.043	5.581E-02	logistic	417859	8156	409703	1.000	0.150
367.1	Myopia	sense organs	rs6814407	0.100	0.052	1.105	5.636E-02	logistic	412331	1325	411006	1.000	0.150
960	Poisoning by antibiotics	injuries & poisonings	rs6814407	-0.026	0.014	0.974	5.672E-02	logistic	389532	22359	367173	1.000	0.150
158	Neoplasm of unspecified nature of digestive system	neoplasms	rs6814407	0.181	0.095	1.199	5.752E-02	logistic	394238	376	393862	1.000	0.150
70.3	Viral hepatitis C	infectious diseases	rs6814407	-0.249	0.132	0.780	5.972E-02	logistic	415468	264	415204	1.000	0.150
724	Other and unspecified disorders of back	musculoskeletal	rs6814407	-0.169	0.090	0.844	6.049E-02	logistic	409212	542	408670	1.000	0.150
593	Hematuria	genitourinary	rs6814407	0.052	0.027	1.053	6.065E-02	logistic	381996	5069	376927	1.000	0.150
261.2	Vitamin B-complex deficiencies	endocrine/metabolic	rs6814407	0.086	0.046	1.090	6.207E-02	logistic	413809	1712	412097	1.000	0.150
451.2	Phlebitis and thrombophlebitis of lower extremities	circulatory system	rs6814407	0.158	0.085	1.171	6.213E-02	logistic	381453	487	380966	1.000	0.150
512.7	Shortness of breath	respiratory	rs6814407	0.057	0.031	1.059	6.402E-02	logistic	408527	3928	404599	1.000	0.150
721.1	Spondylosis without myelopathy	musculoskeletal	rs6814407	0.091	0.049	1.095	6.469E-02	logistic	410174	1504	408670	1.000	0.150
427.9	Palpitations	circulatory system	rs6814407	0.085	0.046	1.088	6.499E-02	logistic	373589	1741	371848	1.000	0.150
569	Other disorders of intestine	digestive	rs6814407	-0.080	0.044	0.923	6.780E-02	logistic	385990	2151	383839	1.000	0.150
698	Pruritus and related conditions	dermatologic	rs6814407	-0.235	0.129	0.791	6.854E-02	logistic	422717	278	422439	1.000	0.150
189	Cancer of urinary organs (incl. kidney and bladder)	neoplasms	rs6814407	-0.054	0.030	0.947	6.890E-02	logistic	421760	4573	417187	1.000	0.150
960.2	Allergy/adverse effect of penicillin	injuries & poisonings	rs6814407	-0.027	0.015	0.973	6.914E-02	logistic	385833	18660	367173	1.000	0.150
191	Manlignant and unknown neoplasms of brain and nervous system	neoplasms	rs6814407	0.111	0.061	1.117	6.939E-02	logistic	421363	966	420397	1.000	0.150
451	Phlebitis and thrombophlebitis	circulatory system	rs6814407	0.128	0.071	1.137	7.012E-02	logistic	381675	709	380966	1.000	0.150
362.29	Macular degeneration (senile) of retina NOS	sense organs	rs6814407	0.056	0.031	1.058	7.069E-02	logistic	401665	3908	397757	1.000	0.150
738	Other acquired musculoskeletal deformity	musculoskeletal	rs6814407	-0.101	0.056	0.904	7.083E-02	logistic	403982	1328	402654	1.000	0.150
202	Cancer of other lymphoid, histiocytic tissue	neoplasms	rs6814407	0.067	0.037	1.070	7.142E-02	logistic	418277	2664	415613	1.000	0.150

ST31; PheWAS results for the lead cis-pQTLs of the 4 proteins prioritized (ACOX1, FGF5, FURIN, and MST1).

SNP = Single Nucleotide Polymorphism; SE = Standard error HWE_p = P-value for Hardy-Weinberg equilibrium test

Phenotype	Description	Group	SNP	Beta	SE	Odds tatio	P-value	Type	n_total	n_cases	n_controls	HWE_p	allele_freq
202.2	Non-Hodgkins lymphoma	neoplasms	rs6814407	0.067	0.037	1.070	7.142E-02	logistic	418277	2664	415613	1.000	0.150
317	Alcohol-related disorders	mental disorders	rs6814407	-0.051	0.028	0.950	7.216E-02	logistic	408346	5053	403293	1.000	0.150
425	Cardiomyopathy	circulatory system	rs6814407	0.079	0.044	1.082	7.252E-02	logistic	418683	1913	416770	1.000	0.150
574.12	Cholelithiasis with other cholecystitis	digestive	rs6814407	0.086	0.048	1.090	7.285E-02	logistic	396636	1589	395047	1.000	0.150
379.2	Disorders of vitreous body	sense organs	rs6814407	0.165	0.092	1.179	7.518E-02	logistic	412333	405	411928	1.000	0.150
440.2	Atherosclerosis of the extremities	circulatory system	rs6814407	-0.123	0.070	0.884	7.697E-02	logistic	406343	874	405469	1.000	0.150
420.2	Pericarditis	circulatory system	rs6814407	0.088	0.050	1.092	7.949E-02	logistic	418236	1466	416770	1.000	0.150
728.71	Contracture of palmar fascia [Dupuytren's disease]	musculoskeletal	rs6814407	0.081	0.047	1.085	8.072E-02	logistic	390879	1705	389174	1.000	0.150
362.3	Other nondiabetic retinopathy	sense organs	rs6814407	0.139	0.080	1.149	8.077E-02	logistic	398314	557	397757	1.000	0.150
535	Gastritis and duodenitis	digestive	rs6814407	-0.028	0.016	0.973	8.127E-02	logistic	378649	16435	362214	1.000	0.150
738.4	Acquired spondylolisthesis	musculoskeletal	rs6814407	-0.103	0.059	0.902	8.178E-02	logistic	403846	1192	402654	1.000	0.150
389.1	Sensorineural hearing loss	sense organs	rs6814407	0.119	0.068	1.126	8.178E-02	logistic	409063	766	408297	1.000	0.150
172.3	Carcinoma in situ of skin	neoplasms	rs6814407	-0.181	0.104	0.834	8.208E-02	logistic	407616	408	407208	1.000	0.150
216	Benign neoplasm of skin	neoplasms	rs6814407	-0.106	0.061	0.899	8.347E-02	logistic	415074	1116	413958	1.000	0.150
594.2	Calculus of lower urinary tract	genitourinary	rs6814407	0.136	0.078	1.145	8.357E-02	logistic	412949	573	412376	1.000	0.150
253	Disorders of the pituitary gland and its hypothalamic control	endocrine/metabolic	rs6814407	0.110	0.064	1.116	8.384E-02	logistic	416136	890	415246	1.000	0.150
317.1	Alcoholism	mental disorders	rs6814407	-0.057	0.033	0.945	8.387E-02	logistic	407082	3789	403293	1.000	0.150
159	Malignant neoplasm of other and ill-defined sites within the digestive organs and peritoneum	neoplasms	rs6814407	-0.120	0.070	0.887	8.475E-02	logistic	394736	874	393862	1.000	0.150
519.8	Other diseases of respiratory system, NEC	respiratory	rs6814407	-0.037	0.022	0.963	8.547E-02	logistic	411807	8677	403130	1.000	0.150
519	Other diseases of respiratory system, not elsewhere classified	respiratory	rs6814407	-0.037	0.022	0.964	8.554E-02	logistic	411962	8832	403130	1.000	0.150
458	Hypotension	circulatory system	rs6814407	0.037	0.022	1.038	8.624E-02	logistic	380778	8206	372572	1.000	0.150
250.4	Abnormal glucose	endocrine/metabolic	rs6814407	0.101	0.059	1.106	8.912E-02	logistic	387669	1032	386637	1.000	0.150
345	Epilepsy, recurrent seizures, convulsions	neurological	rs6814407	-0.053	0.032	0.949	9.407E-02	logistic	403151	4104	399047	1.000	0.150
741.3	Difficulty in walking	musculoskeletal	rs6814407	-0.162	0.097	0.851	9.437E-02	logistic	415474	464	415010	1.000	0.150
411.8	Other chronic ischemic heart disease, unspecified	circulatory system	rs6814407	0.025	0.015	1.025	9.473E-02	logistic	389207	18657	370550	1.000	0.150
513	Respiratory abnormalities	respiratory	rs6814407	-0.168	0.101	0.846	9.638E-02	logistic	423122	431	422691	1.000	0.150
585.4	Chronic kidney disease, Stage I or II	genitourinary	rs6814407	0.111	0.067	1.118	9.723E-02	logistic	381861	799	381062	1.000	0.150
385	Other disorders of middle ear and mastoid	sense organs	rs6814407	-0.199	0.121	0.820	1.010E-01	logistic	419023	307	418716	1.000	0.150
574.2	Calculus of bile duct	digestive	rs6814407	0.193	0.119	1.213	1.044E-01	logistic	395287	240	395047	1.000	0.150
184.2	Cancer of other female genital organs (excluding uterus and ovary	neoplasms	rs6814407	-0.222	0.137	0.801	1.056E-01	logistic	205080	243	204837	1.000	0.150
560.3	Peritoneal or intestinal adhesions	digestive	rs6814407	-0.115	0.071	0.891	1.064E-01	logistic	335317	832	334485	1.000	0.150
242.1	Graves' disease	endocrine/metabolic	rs6814407	0.157	0.097	1.169	1.078E-01	logistic	392598	368	392230	1.000	0.150
378.1	Strabismus (not specified as paralytic)	sense organs	rs6814407	0.180	0.113	1.198	1.102E-01	logistic	412196	268	411928	1.000	0.150
425.1	Primary/intrinsic cardiomyopathies	circulatory system	rs6814407	0.071	0.045	1.074	1.108E-01	logistic	418636	1866	416770	1.000	0.150
715.2	Ankylosing spondylitis	musculoskeletal	rs6814407	0.141	0.089	1.151	1.111E-01	logistic	408956	449	408507	1.000	0.150
573	Other disorders of liver	digestive	rs6814407	-0.072	0.045	0.931	1.116E-01	logistic	402294	2020	400274	1.000	0.150
804	Fracture of hand or wrist	injuries & poisonings	rs6814407	0.125	0.078	1.133	1.120E-01	logistic	396955	577	396378	1.000	0.150
858	Complication of internal orthopedic device	injuries & poisonings	rs6814407	0.086	0.054	1.090	1.153E-01	logistic	417889	1237	416652	1.000	0.150
835	Internal derangement of knee	injuries & poisonings	rs6814407	-0.046	0.029	0.955	1.176E-01	logistic	409924	4709	405215	1.000	0.150
287.31	Primary thrombocytopenia	hematopoietic	rs6814407	0.129	0.083	1.138	1.177E-01	logistic	418072	519	417553	1.000	0.150
760	Back pain	symptoms	rs6814407	0.042	0.027	1.043	1.186E-01	logistic	414925	5193	409732	1.000	0.150
747.11	Cardiac shunt/ heart septal defect	congenital anomalies	rs6814407	0.139	0.089	1.149	1.195E-01	logistic	422041	446	421595	1.000	0.150
596	Other disorders of bladder	genitourinary	rs6814407	0.039	0.025	1.039	1.197E-01	logistic	407805	6251	401554	1.000	0.150
377	Disorders of optic nerve and visual pathways	sense organs	rs6814407	-0.171	0.110	0.843	1.210E-01	logistic	412293	365	411928	1.000	0.150
381.11	Suppurative and unspecified otitis media	sense organs	rs6814407	0.180	0.117	1.197	1.236E-01	logistic	418969	253	418716	1.000	0.150
411	Ischemic Heart Disease	circulatory system	rs6814407	0.016	0.011	1.017	1.237E-01	logistic	410606	40056	370550	1.000	0.150
695.42	Systemic lupus erythematosus	dermatologic	rs6814407	0.143	0.093	1.154	1.246E-01	logistic	403885	403	403482	1.000	0.150
261	Vitamin deficiency	endocrine/metabolic	rs6814407	0.048	0.031	1.049	1.250E-01	logistic	416032	3935	412097	1.000	0.150
369.5	Conjunctivitis, infectious	sense organs	rs6814407	-0.228	0.149	0.796	1.261E-01	logistic	410999	206	410793	1.000	0.150
728.7	Fasciitis	musculoskeletal	rs6814407	0.068	0.045	1.071	1.275E-01	logistic	391018	1844	389174	1.000	0.150
529	Diseases and other conditions of the tongue	digestive	rs6814407	-0.192	0.127	0.825	1.290E-01	logistic	417990	280	417710	1.000	0.150
426.23	Second degree AV block	circulatory system	rs6814407	0.113	0.075	1.120	1.306E-01	logistic	372488	640	371848	1.000	0.150
362.2	Degeneration of macula and posterior pole of retina	sense organs	rs6814407	0.031	0.021	1.032	1.311E-01	logistic	406927	9170	397757	1.000	0.150
509.1	Respiratory failure	respiratory	rs6814407	-0.046	0.030	0.955	1.312E-01	logistic	395437	4380	391057	1.000	0.150
227.1	Benign neoplasm of adrenal gland	neoplasms	rs6814407	-0.199	0.132	0.819	1.313E-01	logistic	421144	257	420887	1.000	0.150
727.4	Ganglion and cyst of synovium, tendon, and bursa	musculoskeletal	rs6814407	0.131	0.087	1.140	1.315E-01	logistic	389641	467	389174	1.000	0.150
715	Other inflammatory spondylopathies	musculoskeletal	rs6814407	0.080	0.053	1.083	1.322E-01	logistic	409805	1298	408507	1.000	0.150
635	Hemorrhage during pregnancy; childbirth and postpartum	pregnancy complications	rs6814407	-0.154	0.103	0.857	1.333E-01	logistic	223005	413	222592	1.000	0.150
276.41	Acidosis	endocrine/metabolic	rs6814407	0.068	0.045	1.070	1.360E-01	logistic	397376	1804	395572	1.000	0.150
159.3	Malignant neoplasm of gallbladder and extrahepatic bile ducts	neoplasms	rs6814407	-0.171	0.115	0.843	1.374E-01	logistic	394194	332	393862	1.000	0.150
353	Nerve root and plexus disorders	neurological	rs6814407	-0.179	0.120	0.837	1.375E-01	logistic	402720	305	402415	1.000	0.150
198.6	Secondary malignancy of bone	neoplasms	rs6814407	-0.043	0.029	0.958	1.379E-01	logistic	390762	4756	386006	1.000	0.150
709.3	Systemic sclerosis	dermatologic	rs6814407	-0.213	0.144	0.808	1.390E-01	logistic	404118	219	403899	1.000	0.150
175	Acquired absence of breast	neoplasms	rs6814407	-0.047	0.032	0.954	1.393E-01	logistic	206502	3992	202510	1.000	0.150
342	Hemiplegia	neurological	rs6814407	0.058	0.040	1.060	1.405E-01	logistic	401449	2402	399047	1.000	0.150
274	Gout and other crystal arthropathies	endocrine/metabolic	rs6814407	-0.044	0.030	0.957	1.415E-01	logistic	420276	4486	415790	1.000	0.150
473.3	Paralysis/spasm of vocal cords or larynx	respiratory	rs6814407	-0.208	0.141	0.812	1.417E-01	logistic	396742	225	396517	1.000	0.150
394.2	Mitral valve disease	circulatory system	rs6814407	0.154	0.106	1.167	1.448E-01	logistic	401726	312	401414	1.000	0.150
603.1	Hydrocele	genitourinary	rs6814407	-0.143	0.098	0.867	1.457E-01	logistic	162225	447	161778	1.000	0.150
396	Abnormal heart sounds	circulatory system	rs6814407	-0.097	0.066	0.908	1.463E-01	logistic	402357	943	401414	1.000	0.150
479	Other upper respiratory disease	respiratory	rs6814407	-0.147	0.101	0.863	1.470E-01	logistic	396937	420	396517	1.000	0.150

ST31; PheWAS results for the lead cis-pQTLs of the 4 proteins prioritized (ACOX1, FGF5, FURIN, and MST1).

SNP = Single Nucleotide Polymorphism; SE = Standard error HWE_p = P-value for Hardy-Weinberg equilibrium test

Phenotype	Description	Group	SNP	Beta	SE	Odds tatio	P-value	Type	n_total	n_cases	n_controls	HWE_p	allele_freq
721	Spondylosis and allied disorders	musculoskeletal	rs6814407	0.064	0.044	1.066	1.475E-01	logistic	410559	1889	408670	1.000	0.150
394	Rheumatic disease of the heart valves	circulatory system	rs6814407	0.041	0.028	1.042	1.486E-01	logistic	406218	4804	401414	1.000	0.150
315	Develomental delays and disorders	mental disorders	rs6814407	-0.113	0.079	0.893	1.500E-01	logistic	422717	680	422037	1.000	0.150
316	Substance addiction and disorders	mental disorders	rs6814407	0.143	0.100	1.154	1.507E-01	logistic	403645	352	403293	1.000	0.150
747.1	Cardiac congenital anomalies	congenital anomalies	rs6814407	0.089	0.062	1.093	1.530E-01	logistic	422536	941	421595	1.000	0.150
335	Multiple sclerosis	neurological	rs6814407	0.072	0.050	1.074	1.546E-01	logistic	400514	1467	399047	1.000	0.150
351	Other peripheral nerve disorders	neurological	rs6814407	-0.035	0.025	0.966	1.549E-01	logistic	409126	6711	402415	1.000	0.150
369	Infection of the eye	sense organs	rs6814407	-0.144	0.102	0.866	1.568E-01	logistic	411210	417	410793	1.000	0.150
695.4	Lupus (localized and systemic)	dermatologic	rs6814407	0.120	0.085	1.128	1.586E-01	logistic	403975	493	403482	1.000	0.150
557.1	Celiac disease	digestive	rs6814407	0.063	0.044	1.065	1.598E-01	logistic	336383	1898	334485	1.000	0.150
80	Postoperative infection	infectious diseases	rs6814407	0.062	0.044	1.064	1.613E-01	logistic	414741	1897	412844	1.000	0.150
851	Complications of transplants and reattached limbs	injuries & poisonings	rs6814407	0.166	0.119	1.181	1.630E-01	logistic	416895	243	416652	1.000	0.150
961	Poisoning by other anti-infectives	injuries & poisonings	rs6814407	-0.172	0.124	0.842	1.634E-01	logistic	367461	288	367173	1.000	0.150
798.1	Chronic fatigue syndrome	symptoms	rs6814407	0.107	0.077	1.113	1.636E-01	logistic	414937	617	414320	1.000	0.150
8.6	Viral Enteritis	infectious diseases	rs6814407	-0.128	0.092	0.880	1.648E-01	logistic	417821	505	417316	1.000	0.150
578.9	Hemorrhage of gastrointestinal tract	digestive	rs6814407	-0.052	0.037	0.950	1.650E-01	logistic	386245	2942	383303	1.000	0.150
452	Other venous embolism and thrombosis	circulatory system	rs6814407	0.144	0.104	1.155	1.668E-01	logistic	381290	324	380966	1.000	0.150
871	Open wounds of extremities	injuries & poisonings	rs6814407	0.135	0.098	1.145	1.668E-01	logistic	420520	370	420150	1.000	0.150
191.1	Cancer of brain and nervous system	neoplasms	rs6814407	0.091	0.066	1.096	1.670E-01	logistic	421229	832	420397	1.000	0.150
274.1	Gout	endocrine/metabolic	rs6814407	-0.043	0.031	0.958	1.683E-01	logistic	420024	4234	415790	1.000	0.150
555.2	Ulcerative colitis	digestive	rs6814407	-0.049	0.036	0.952	1.686E-01	logistic	337679	3194	334485	1.000	0.150
602	Other disorders of prostate	genitourinary	rs6814407	0.140	0.102	1.150	1.690E-01	logistic	156802	341	156461	1.000	0.150
395.3	Nonrheumatic tricuspid valve disorders	circulatory system	rs6814407	-0.186	0.137	0.830	1.720E-01	logistic	401653	239	401414	1.000	0.150
285	Other anemias	hematopoietic	rs6814407	-0.026	0.019	0.975	1.741E-01	logistic	393921	11472	382449	1.000	0.150
426	Cardiac conduction disorders	circulatory system	rs6814407	0.026	0.019	1.027	1.749E-01	logistic	382540	10692	371848	1.000	0.150
636	Early or threatened labor; hemorrhage in early pregnancy	pregnancy complications	rs6814407	-0.121	0.090	0.886	1.767E-01	logistic	223122	530	222592	1.000	0.150
241	Nontoxic nodular goiter	endocrine/metabolic	rs6814407	0.093	0.069	1.097	1.772E-01	logistic	392996	766	392230	1.000	0.150
695.9	Unspecified erythematous condition	dermatologic	rs6814407	-0.158	0.117	0.854	1.774E-01	logistic	405524	318	405206	1.000	0.150
990	Effects radiation NOS	injuries & poisonings	rs6814407	0.032	0.024	1.032	1.774E-01	logistic	408877	6919	401958	1.000	0.150
427.41	Ventricular fibrillation and flutter	circulatory system	rs6814407	-0.174	0.129	0.840	1.779E-01	logistic	372113	265	371848	1.000	0.150
368.4	Visual field defects	sense organs	rs6814407	0.121	0.090	1.128	1.783E-01	logistic	415988	443	415545	1.000	0.150
414	Other forms of chronic heart disease	circulatory system	rs6814407	0.041	0.031	1.042	1.800E-01	logistic	374670	4120	370550	1.000	0.150
474	Acute and chronic tonsillitis	respiratory	rs6814407	-0.143	0.107	0.867	1.808E-01	logistic	396895	378	396517	1.000	0.150
428.1	Congestive heart failure (CHF) NOS	circulatory system	rs6814407	0.041	0.031	1.042	1.832E-01	logistic	413649	3946	409703	1.000	0.150
456	Chronic venous insufficiency [CVI]	circulatory system	rs6814407	-0.165	0.124	0.848	1.841E-01	logistic	381250	284	380966	1.000	0.150
359	Muscular dystrophies and other myopathies	neurological	rs6814407	0.149	0.113	1.161	1.876E-01	logistic	419442	274	419168	1.000	0.150
174.11	Malignant neoplasm of female breast	neoplasms	rs6814407	-0.028	0.021	0.973	1.903E-01	logistic	212959	9200	203759	1.000	0.150
530.3	Stricture and stenosis of esophagus	digestive	rs6814407	0.073	0.056	1.076	1.906E-01	logistic	357976	1188	356788	1.000	0.150
159.2	Malignant neoplasm of small intestine, including duodenum	neoplasms	rs6814407	-0.172	0.131	0.842	1.916E-01	logistic	394117	255	393862	1.000	0.150
79	Viral infection	infectious diseases	rs6814407	-0.083	0.064	0.920	1.918E-01	logistic	416221	1017	415204	1.000	0.150
145.2	Cancer of tongue	neoplasms	rs6814407	-0.136	0.105	0.872	1.930E-01	logistic	420733	391	420342	1.000	0.150
743.21	Pathologic fracture of vertebrae	musculoskeletal	rs6814407	-0.106	0.082	0.899	1.931E-01	logistic	421861	627	421234	1.000	0.150
374	Other disorders of eyelids	sense organs	rs6814407	0.053	0.041	1.054	1.932E-01	logistic	413055	2262	410793	1.000	0.150
365.2	Primary angle-closure glaucoma	sense organs	rs6814407	0.095	0.073	1.100	1.937E-01	logistic	398441	678	397763	1.000	0.150
242	Thyrotoxicosis with or without goiter	endocrine/metabolic	rs6814407	0.063	0.049	1.065	1.941E-01	logistic	393812	1582	392230	1.000	0.150
78	Viral warts & HPV	infectious diseases	rs6814407	0.162	0.125	1.176	1.956E-01	logistic	415426	222	415204	1.000	0.150
701.3	Circumscribed scleroderma	dermatologic	rs6814407	-0.182	0.140	0.834	1.958E-01	logistic	415410	224	415186	1.000	0.150
507	Pleurisy; pleural effusion	respiratory	rs6814407	-0.026	0.020	0.974	1.965E-01	logistic	400937	9880	391057	1.000	0.150
429.2	Abnormal function study of cardiovascular system	circulatory system	rs6814407	0.133	0.103	1.142	1.966E-01	logistic	410036	333	409703	1.000	0.150
276.12	Hyposmolality and/or hyponatremia	endocrine/metabolic	rs6814407	-0.037	0.029	0.964	1.975E-01	logistic	400551	4979	395572	1.000	0.150
420.3	Endocarditis	circulatory system	rs6814407	0.099	0.077	1.104	1.982E-01	logistic	417383	613	416770	1.000	0.150
870	Open wounds of head; neck; and trunk	injuries & poisonings	rs6814407	-0.091	0.071	0.913	1.986E-01	logistic	420976	826	420150	1.000	0.150
509	Respiratory failure, insufficiency, arrest	respiratory	rs6814407	-0.033	0.026	0.967	1.990E-01	logistic	397038	5981	391057	1.000	0.150
428.2	Heart failure NOS	circulatory system	rs6814407	0.036	0.028	1.037	1.997E-01	logistic	414622	4919	409703	1.000	0.150
747	Cardiac and circulatory congenital anomalies	congenital anomalies	rs6814407	0.074	0.058	1.077	2.000E-01	logistic	422691	1096	421595	1.000	0.150
262	Mineral deficiency NEC	endocrine/metabolic	rs6814407	0.130	0.102	1.139	2.013E-01	logistic	412440	343	412097	1.000	0.150
724.1	Disorders of sacrum	musculoskeletal	rs6814407	-0.177	0.139	0.838	2.017E-01	logistic	408900	230	408670	1.000	0.150
591	Urinary tract infection	genitourinary	rs6814407	0.023	0.018	1.024	2.023E-01	logistic	388723	11796	376927	1.000	0.150
564	Functional digestive disorders	digestive	rs6814407	0.018	0.014	1.018	2.031E-01	logistic	354387	19902	334485	1.000	0.150
41.11	Methicillin sensitive Staphylococcus aureus	infectious diseases	rs6814407	0.061	0.048	1.063	2.039E-01	logistic	398282	1635	396647	1.000	0.150
362.31	Separation of retinal layers	sense organs	rs6814407	0.106	0.084	1.112	2.060E-01	logistic	398271	514	397757	1.000	0.150
724.2	Disorders of coccyx	musculoskeletal	rs6814407	-0.181	0.144	0.834	2.080E-01	logistic	408885	215	408670	1.000	0.150
429	Ill-defined descriptions and complications of heart disease	circulatory system	rs6814407	0.130	0.103	1.138	2.085E-01	logistic	410037	334	409703	1.000	0.150
565	Anal and rectal conditions	digestive	rs6814407	0.028	0.023	1.029	2.109E-01	logistic	391353	7514	383839	1.000	0.150
285.22	Anemia in neoplastic disease	hematopoietic	rs6814407	-0.142	0.114	0.867	2.121E-01	logistic	382780	331	382449	1.000	0.150
8.52	Intestinal infection due to C. difficile	infectious diseases	rs6814407	-0.098	0.079	0.907	2.142E-01	logistic	417984	668	417316	1.000	0.150
735.23	Hallux rigidus	musculoskeletal	rs6814407	0.131	0.105	1.140	2.151E-01	logistic	402973	319	402654	1.000	0.150
289.8	Polycythemia, secondary	hematopoietic	rs6814407	-0.117	0.095	0.889	2.168E-01	logistic	407605	466	407139	1.000	0.150
750.2	Lower gastrointestinal congenital anomalies	congenital anomalies	rs6814407	0.162	0.132	1.176	2.178E-01	logistic	420453	200	420253	1.000	0.150
587	Kidney replaced by transpant	genitourinary	rs6814407	0.105	0.085	1.110	2.212E-01	logistic	381556	494	381062	1.000	0.150
204.1	Lymphoid leukemia	neoplasms	rs6814407	0.078	0.064	1.081	2.216E-01	logistic	416514	901	415613	1.000	0.150

ST31; PheWAS results for the lead cis-pQTLs of the 4 proteins prioritized (ACOX1, FGF5, FURIN, and MST1).
SNP = Single Nucleotide Polymorphism; SE = Standard error HWE_p = P-value for Hardy-Weinberg equilibrium test

Phenotype	Description	Group	SNP	Beta	SE	Odds tatio	P-value	Type	n_total	n_cases	n_controls	HWE_p	allele_freq
580.1	Glomerulonephritis	genitourinary	rs6814407	0.102	0.084	1.107	2.255E-01	logistic	381574	512	381062	1.000	0.150
241.2	Nontoxic multinodular goiter	endocrine/metabolic	rs6814407	0.147	0.122	1.158	2.265E-01	logistic	392467	237	392230	1.000	0.150
709	Diffuse diseases of connective tissue	dermatologic	rs6814407	0.069	0.057	1.072	2.268E-01	logistic	405028	1129	403899	1.000	0.150
327.3	Sleep apnea	neurological	rs6814407	0.032	0.027	1.033	2.274E-01	logistic	418773	5389	413384	1.000	0.150
395	Heart valve disorders	circulatory system	rs6814407	0.024	0.020	1.025	2.274E-01	logistic	411117	9703	401414	1.000	0.150
430.1	Subarachnoid hemorrhage	circulatory system	rs6814407	0.085	0.071	1.089	2.294E-01	logistic	403416	731	402685	1.000	0.150
568	Other disorders of peritoneum	digestive	rs6814407	-0.063	0.053	0.939	2.315E-01	logistic	385305	1466	383839	1.000	0.150
550.5	Ventral hernia	digestive	rs6814407	-0.059	0.050	0.942	2.345E-01	logistic	367031	1637	365394	1.000	0.150
290.11	Alzheimer's disease	mental disorders	rs6814407	0.047	0.040	1.048	2.362E-01	logistic	407367	2394	404973	1.000	0.150
191.11	Cancer of brain	neoplasms	rs6814407	0.081	0.068	1.084	2.364E-01	logistic	421187	790	420397	1.000	0.150
385.3	Cholesteatoma	sense organs	rs6814407	-0.167	0.141	0.846	2.369E-01	logistic	418937	221	418716	1.000	0.150
368	Visual disturbances	sense organs	rs6814407	0.048	0.041	1.049	2.371E-01	logistic	417843	2298	415545	1.000	0.150
333.1	Essential tremor	neurological	rs6814407	0.096	0.083	1.101	2.436E-01	logistic	399579	532	399047	1.000	0.150
149.4	Cancer of larynx	neoplasms	rs6814407	-0.146	0.125	0.864	2.439E-01	logistic	420618	276	420342	1.000	0.150
211	Benign neoplasm of other parts of digestive system	neoplasms	rs6814407	0.038	0.033	1.039	2.475E-01	logistic	400517	3467	397050	1.000	0.150
81	Infection/inflammation of internal prosthetic device; implant; and graf	infectious diseases	rs6814407	-0.053	0.046	0.949	2.477E-01	logistic	414783	1939	412844	1.000	0.150
241.1	Nontoxic uninodular goiter	endocrine/metabolic	rs6814407	-0.161	0.139	0.851	2.481E-01	logistic	392455	225	392230	1.000	0.150
447	Other disorders of arteries and arterioles	circulatory system	rs6814407	0.067	0.058	1.069	2.506E-01	logistic	406558	1089	405469	1.000	0.150
608	Other disorders of male genital organs	genitourinary	rs6814407	-0.084	0.074	0.919	2.561E-01	logistic	162533	755	161778	1.000	0.150
276.4	Acid-base balance disorder	endocrine/metabolic	rs6814407	0.048	0.042	1.049	2.574E-01	logistic	397692	2120	395572	1.000	0.150
366	Cataract	sense organs	rs6814407	-0.013	0.011	0.987	2.576E-01	logistic	406937	35259	371678	1.000	0.150
550	Abdominal hernia	digestive	rs6814407	0.014	0.012	1.014	2.583E-01	logistic	392811	27417	365394	1.000	0.150
395.1	Nonrheumatic mitral valve disorders	circulatory system	rs6814407	0.036	0.032	1.037	2.593E-01	logistic	405090	3676	401414	1.000	0.150
717	Polymyalgia Rheumatica	musculoskeletal	rs6814407	-0.046	0.041	0.955	2.594E-01	logistic	422397	2442	419955	1.000	0.150
244.4	Hypothyroidism NOS	endocrine/metabolic	rs6814407	0.017	0.015	1.017	2.626E-01	logistic	410296	18066	392230	1.000	0.150
600	Hyperplasia of prostate	genitourinary	rs6814407	0.021	0.019	1.021	2.652E-01	logistic	169018	12557	156461	1.000	0.150
780	Hypothermia/Chills	symptoms	rs6814407	-0.156	0.140	0.855	2.653E-01	logistic	423421	221	423200	1.000	0.150
189.4	Malignant neoplasm of other urinary organs	neoplasms	rs6814407	-0.162	0.145	0.851	2.664E-01	logistic	417393	206	417187	1.000	0.150
756	Other congenital musculoskeletal anomalies	congenital anomalies	rs6814407	-0.072	0.065	0.931	2.678E-01	logistic	422256	974	421282	1.000	0.150
184	Cancer of other female genital organs	neoplasms	rs6814407	-0.041	0.037	0.960	2.679E-01	logistic	207829	2992	204837	1.000	0.150
446.4	Wegener's granulomatosis	circulatory system	rs6814407	0.136	0.123	1.145	2.685E-01	logistic	405703	234	405469	1.000	0.150
803.2	Fracture of radius and ulna	injuries & poisonings	rs6814407	-0.047	0.042	0.954	2.717E-01	logistic	398618	2240	396378	1.000	0.150
624.9	stress incontinence, female	genitourinary	rs6814407	0.049	0.045	1.051	2.736E-01	logistic	220155	1871	218284	1.000	0.150
332	Parkinson's disease	neurological	rs6814407	0.040	0.036	1.040	2.736E-01	logistic	401966	2919	399047	1.000	0.150
198.2	Secondary malignancy of respiratory organs	neoplasms	rs6814407	-0.032	0.029	0.969	2.739E-01	logistic	390707	4701	386006	1.000	0.150
735.2	Acquired toe deformities	musculoskeletal	rs6814407	0.048	0.044	1.050	2.752E-01	logistic	404574	1920	402654	1.000	0.150
714.1	Rheumatoid arthritis	musculoskeletal	rs6814407	0.034	0.032	1.035	2.758E-01	logistic	412357	3850	408507	1.000	0.150
198.1	Secondary malignancy of lymph nodes	neoplasms	rs6814407	-0.024	0.022	0.976	2.778E-01	logistic	394015	8009	386006	1.000	0.150
331	Other cerebral degenerations	neurological	rs6814407	-0.054	0.050	0.948	2.793E-01	logistic	400699	1652	399047	1.000	0.150
599.3	Dysuria	genitourinary	rs6814407	0.112	0.104	1.119	2.794E-01	logistic	390438	332	390106	1.000	0.150
292.1	Aphasia/speech disturbance	mental disorders	rs6814407	-0.067	0.062	0.935	2.806E-01	logistic	406020	1047	404973	1.000	0.150
596.5	Functional disorders of bladder	genitourinary	rs6814407	0.082	0.076	1.085	2.824E-01	logistic	402194	640	401554	1.000	0.150
575.1	Cholangitis	digestive	rs6814407	-0.073	0.068	0.930	2.836E-01	logistic	395932	885	395047	1.000	0.150
941	Adverse reaction to serum or vaccine	injuries & poisonings	rs6814407	-0.148	0.139	0.862	2.842E-01	logistic	415212	225	414987	1.000	0.150
522.5	Periapical abscess	digestive	rs6814407	0.139	0.130	1.150	2.843E-01	logistic	409744	208	409536	1.000	0.150
348.2	Cerebral edema and compression of brain	neurological	rs6814407	-0.113	0.106	0.893	2.851E-01	logistic	399425	378	399047	1.000	0.150
153.3	Malignant neoplasm of rectum, rectosigmoid junction, and anus	neoplasms	rs6814407	0.039	0.036	1.040	2.853E-01	logistic	373609	2889	370720	1.000	0.150
569.2	Gastrointestinal complications	digestive	rs6814407	-0.132	0.124	0.876	2.859E-01	logistic	384117	278	383839	1.000	0.150
255	Disorders of adrenal glands	endocrine/metabolic	rs6814407	-0.077	0.072	0.926	2.864E-01	logistic	416034	788	415246	1.000	0.150
41.1	Staphylococcus infections	infectious diseases	rs6814407	0.043	0.040	1.044	2.876E-01	logistic	398981	2334	396647	1.000	0.150
465	Acute upper respiratory infections of multiple or unspecified sites	respiratory	rs6814407	0.077	0.073	1.080	2.880E-01	logistic	420386	694	419692	1.000	0.150
348	Other conditions of brain	neurological	rs6814407	-0.083	0.078	0.920	2.885E-01	logistic	399717	670	399047	1.000	0.150
395.2	Nonrheumatic aortic valve disorders	circulatory system	rs6814407	0.030	0.028	1.030	2.888E-01	logistic	406373	4959	401414	1.000	0.150
425.12	Other hypertrophic cardiomyopathy	circulatory system	rs6814407	-0.132	0.124	0.876	2.890E-01	logistic	417044	274	416770	1.000	0.150
79.9	Viremia, NOS	infectious diseases	rs6814407	-0.088	0.084	0.916	2.915E-01	logistic	415798	594	415204	1.000	0.150
112	Candidiasis	infectious diseases	rs6814407	-0.054	0.051	0.948	2.921E-01	logistic	419935	1555	418380	1.000	0.150
447.1	Stricture of artery	circulatory system	rs6814407	0.081	0.077	1.085	2.922E-01	logistic	406083	614	405469	1.000	0.150
525	Other diseases of the teeth and supporting structures	digestive	rs6814407	-0.096	0.092	0.908	2.951E-01	logistic	410027	491	409536	1.000	0.150
440	Atherosclerosis	circulatory system	rs6814407	-0.064	0.062	0.938	2.994E-01	logistic	406545	1076	405469	1.000	0.150
521	Diseases of hard tissues of teeth	digestive	rs6814407	0.070	0.068	1.073	3.000E-01	logistic	410337	801	409536	1.000	0.150
443.1	Raynaud's syndrome	circulatory system	rs6814407	-0.052	0.051	0.949	3.005E-01	logistic	407046	1577	405469	1.000	0.150
781	Symptoms involving nervous and musculoskeletal system	symptoms	rs6814407	0.023	0.022	1.023	3.028E-01	logistic	419375	7893	411482	1.000	0.150
790.6	Other abnormal blood chemistry	symptoms	rs6814407	0.125	0.123	1.133	3.085E-01	logistic	422077	237	421840	1.000	0.150
530.12	Ulcer of esophagus	digestive	rs6814407	0.039	0.038	1.040	3.089E-01	logistic	359422	2634	356788	1.000	0.150
184.11	Malignant neoplasm of ovary	neoplasms	rs6814407	-0.039	0.039	0.962	3.113E-01	logistic	207552	2715	204837	1.000	0.150
568.1	Peritoneal adhesions (postoperative) (postinfection	digestive	rs6814407	-0.057	0.056	0.945	3.125E-01	logistic	385136	1297	383839	1.000	0.150
290	Delirium dementia and amnestic and other cognitive disorders	mental disorders	rs6814407	0.024	0.023	1.024	3.128E-01	logistic	412158	7185	404973	1.000	0.150
180	Cervical cancer and dysplasia	neoplasms	rs6814407	-0.040	0.040	0.961	3.154E-01	logistic	198422	2518	195904	1.000	0.150
180.1	Cervical cancer	neoplasms	rs6814407	-0.045	0.045	0.956	3.154E-01	logistic	197916	2012	195904	1.000	0.150
770	Myalgia and myositis unspecified	symptoms	rs6814407	0.045	0.045	1.047	3.162E-01	logistic	422525	1839	420686	1.000	0.150
750	Digestive congenital anomalies	congenital anomalies	rs6814407	0.093	0.093	1.098	3.165E-01	logistic	420674	421	420253	1.000	0.150

ST31; PheWAS results for the lead cis-pQTLs of the 4 proteins prioritized (ACOX1, FGF5, FURIN, and MST1).

SNP = Single Nucleotide Polymorphism; SE = Standard error HWE_p = P-value for Hardy-Weinberg equilibrium test

Phenotype	Description	Group	SNP	Beta	SE	Odds tatio	P-value	Type	n_total	n_cases	n_controls	HWE_p	allele_freq
284	Aplastic anemia	hematopoietic	rs6814407	-0.075	0.075	0.928	3.178E-01	logistic	383179	730	382449	1.000	0.150
559	Ileostomy status	digestive	rs6814407	-0.047	0.047	0.954	3.218E-01	logistic	336295	1810	334485	1.000	0.150
751.22	Other specified congenital anomalies of kidney	congenital anomalies	rs6814407	-0.109	0.110	0.897	3.222E-01	logistic	420601	348	420253	1.000	0.150
550.2	Diaphragmatic hernia	digestive	rs6814407	0.013	0.013	1.013	3.231E-01	logistic	388258	22864	365394	1.000	0.150
288	Diseases of white blood cells	hematopoietic	rs6814407	0.092	0.094	1.097	3.246E-01	logistic	408723	414	408309	1.000	0.150
293	Symptoms involving head and neck	mental disorders	rs6814407	-0.092	0.094	0.912	3.284E-01	logistic	420175	464	419711	1.000	0.150
155	Cancer of liver and intrahepatic bile duct	neoplasms	rs6814407	-0.072	0.074	0.930	3.293E-01	logistic	394604	742	393862	1.000	0.150
368.2	Diplopia and disorders of binocular vision	sense organs	rs6814407	0.081	0.084	1.085	3.298E-01	logistic	416069	524	415545	1.000	0.150
751.21	Cystic kidney disease	congenital anomalies	rs6814407	0.095	0.097	1.099	3.299E-01	logistic	420636	383	420253	1.000	0.150
764	Sciatica	symptoms	rs6814407	0.052	0.053	1.053	3.307E-01	logistic	419302	1316	417986	1.000	0.150
496.3	Bronchiectasis	respiratory	rs6814407	-0.032	0.033	0.969	3.311E-01	logistic	402578	3769	398809	1.000	0.150
350.3	Lack of coordination	neurological	rs6814407	-0.117	0.121	0.889	3.315E-01	logistic	420499	289	420210	1.000	0.150
378	Strabismus and other disorders of binocular eye movements	sense organs	rs6814407	0.060	0.062	1.062	3.337E-01	logistic	412884	956	411928	1.000	0.150
567	Peritonitis and retroperitoneal infections	digestive	rs6814407	-0.072	0.075	0.931	3.384E-01	logistic	384568	729	383839	1.000	0.150
227	Benign neoplasm of other endocrine glands and related structures	neoplasms	rs6814407	-0.072	0.076	0.930	3.398E-01	logistic	421596	709	420887	1.000	0.150
531.3	Duodenal ulcer	digestive	rs6814407	-0.057	0.060	0.945	3.412E-01	logistic	412469	1130	411339	1.000	0.150
687.1	Rash and other nonspecific skin eruption	dermatologic	rs6814407	-0.058	0.061	0.944	3.417E-01	logistic	413803	1087	412716	1.000	0.150
287.3	Thrombocytopenia	hematopoietic	rs6814407	0.039	0.041	1.040	3.434E-01	logistic	419777	2224	417553	1.000	0.150
318	Tobacco use disorder	mental disorders	rs6814407	-0.027	0.028	0.974	3.448E-01	logistic	408298	5005	403293	1.000	0.150
696.41	Psoriasis vulgaris	dermatologic	rs6814407	0.040	0.042	1.041	3.452E-01	logistic	401395	2105	399290	1.000	0.150
174	Breast cancer	neoplasms	rs6814407	-0.019	0.020	0.981	3.453E-01	logistic	401305	10248	391057	1.000	0.150
195.1	Malignant neoplasm, other	neoplasms	rs6814407	-0.022	0.023	0.979	3.466E-01	logistic	393640	7634	386006	1.000	0.150
473.4	Voice disturbance	respiratory	rs6814407	-0.114	0.122	0.893	3.504E-01	logistic	396801	284	396517	1.000	0.150
195	Cancer, suspected or other	neoplasms	rs6814407	-0.021	0.023	0.979	3.507E-01	logistic	393669	7663	386006	1.000	0.150
289	Other diseases of blood and blood-forming organs	hematopoietic	rs6814407	0.063	0.068	1.065	3.508E-01	logistic	409120	811	408309	1.000	0.150
184.1	Malignant neoplasm of ovary and other uterine adnexa	neoplasms	rs6814407	-0.035	0.038	0.965	3.520E-01	logistic	207626	2789	204837	1.000	0.150
288.1	Decreased white blood cell count	hematopoietic	rs6814407	-0.033	0.035	0.968	3.541E-01	logistic	411502	3193	408309	1.000	0.150
288.11	Neutropenia	hematopoietic	rs6814407	-0.033	0.035	0.968	3.541E-01	logistic	411502	3193	408309	1.000	0.150
603	Other disorders of testis	genitourinary	rs6814407	-0.087	0.094	0.917	3.547E-01	logistic	162246	468	161778	1.000	0.150
540	Appendiceal conditions	digestive	rs6814407	0.082	0.089	1.085	3.556E-01	logistic	422123	467	421656	1.000	0.150
626.1	Irregular menstrual cycle/bleeding	genitourinary	rs6814407	-0.029	0.031	0.972	3.606E-01	logistic	196900	4153	192747	1.000	0.150
389.4	Tinnitus	sense organs	rs6814407	0.080	0.088	1.084	3.626E-01	logistic	408770	473	408297	1.000	0.150
502	Postinflammatory pulmonary fibrosis	respiratory	rs6814407	-0.040	0.045	0.961	3.669E-01	logistic	393073	2016	391057	1.000	0.150
714	Rheumatoid arthritis and other inflammatory polyarthropathies	musculoskeletal	rs6814407	0.028	0.031	1.028	3.673E-01	logistic	412574	4067	408507	1.000	0.150
444	Arterial embolism and thrombosis	circulatory system	rs6814407	-0.067	0.074	0.936	3.676E-01	logistic	406215	746	405469	1.000	0.150
604.3	Peyronie's disease	genitourinary	rs6814407	0.114	0.127	1.121	3.681E-01	logistic	162002	224	161778	1.000	0.150
726.1	Enthesopathy	musculoskeletal	rs6814407	0.030	0.034	1.031	3.703E-01	logistic	392491	3317	389174	1.000	0.150
344	Other paralytic syndromes	neurological	rs6814407	-0.070	0.078	0.932	3.704E-01	logistic	399714	667	399047	1.000	0.150
331.1	Hydrocephalus	neurological	rs6814407	0.069	0.077	1.072	3.705E-01	logistic	399668	621	399047	1.000	0.150
418.1	Precordial pain	circulatory system	rs6814407	0.040	0.045	1.041	3.710E-01	logistic	417539	1859	415680	1.000	0.150
8	Intestinal infection	infectious diseases	rs6814407	-0.041	0.046	0.960	3.715E-01	logistic	419231	1915	417316	1.000	0.150
250.42	Other abnormal glucose	endocrine/metabolic	rs6814407	0.097	0.109	1.102	3.717E-01	logistic	386940	303	386637	1.000	0.150
255.2	Adrenal hypofunction	endocrine/metabolic	rs6814407	-0.098	0.109	0.907	3.724E-01	logistic	415595	349	415246	1.000	0.150
255.21	Glucocorticoid deficiency	endocrine/metabolic	rs6814407	-0.098	0.109	0.907	3.724E-01	logistic	415595	349	415246	1.000	0.150
555	Inflammatory bowel disease and other gastroenteritis and colitis	digestive	rs6814407	-0.026	0.029	0.974	3.741E-01	logistic	339234	4749	334485	1.000	0.150
626.14	Irregular menstrual bleeding	genitourinary	rs6814407	-0.091	0.102	0.913	3.744E-01	logistic	193146	399	192747	1.000	0.150
571.5	Other chronic nonalcoholic liver disease	digestive	rs6814407	-0.027	0.030	0.974	3.767E-01	logistic	404607	4333	400274	1.000	0.150
604	Disorders of penis	genitourinary	rs6814407	0.077	0.087	1.080	3.783E-01	logistic	162266	488	161778	1.000	0.150
735.3	Hallux valgus (Bunion)	musculoskeletal	rs6814407	-0.036	0.040	0.965	3.784E-01	logistic	405116	2462	402654	1.000	0.150
276.42	Alkalosis	endocrine/metabolic	rs6814407	-0.103	0.117	0.902	3.792E-01	logistic	395878	306	395572	1.000	0.150
174.1	Breast cancer [female]	neoplasms	rs6814407	-0.018	0.020	0.982	3.803E-01	logistic	213896	10137	203759	1.000	0.150
696.4	Psoriasis	dermatologic	rs6814407	0.032	0.036	1.032	3.828E-01	logistic	402177	2887	399290	1.000	0.150
441.1	Acute vascular insufficiency of intestine	circulatory system	rs6814407	0.096	0.110	1.100	3.843E-01	logistic	405771	302	405469	1.000	0.150
204.12	Lymphoid leukemia, chronic	neoplasms	rs6814407	0.059	0.068	1.060	3.855E-01	logistic	416428	815	415613	1.000	0.150
504	Other alveolar and parietoalveolar pneumonopathy	respiratory	rs6814407	-0.067	0.078	0.935	3.901E-01	logistic	391718	661	391057	1.000	0.150
540.11	Acute appendicitis	digestive	rs6814407	0.109	0.128	1.116	3.932E-01	logistic	421875	219	421656	1.000	0.150
368.91	Psychophysical visual disturbances	sense organs	rs6814407	-0.107	0.126	0.898	3.966E-01	logistic	415808	263	415545	1.000	0.150
560	Intestinal obstruction without mention of hernia	digestive	rs6814407	-0.028	0.033	0.972	3.988E-01	logistic	338110	3625	334485	1.000	0.150
285.2	Anemia of chronic disease	hematopoietic	rs6814407	-0.060	0.072	0.941	3.994E-01	logistic	383237	788	382449	1.000	0.150
208	Benign neoplasm of colon	neoplasms	rs6814407	0.012	0.014	1.012	3.997E-01	logistic	395855	20019	375836	1.000	0.150
740.9	Osteoarthrosis NOS	musculoskeletal	rs6814407	0.014	0.016	1.014	4.003E-01	logistic	361594	15736	345858	1.000	0.150
187	Cancer of other male genital organs	neoplasms	rs6814407	-0.020	0.024	0.981	4.036E-01	logistic	186921	7527	179394	1.000	0.150
530.9	Heartburn	digestive	rs6814407	0.100	0.120	1.105	4.053E-01	logistic	357038	250	356788	1.000	0.150
228	Hemangioma and lymphangioma, any site	neoplasms	rs6814407	0.069	0.084	1.071	4.120E-01	logistic	421655	529	421126	1.000	0.150
182	Malignant neoplasm of uterus	neoplasms	rs6814407	-0.047	0.057	0.954	4.123E-01	logistic	197217	1245	195972	1.000	0.150
535.6	Duodenitis	digestive	rs6814407	0.038	0.046	1.038	4.138E-01	logistic	363993	1779	362214	1.000	0.150
282	Hereditary hemolytic anemias	hematopoietic	rs6814407	-0.110	0.136	0.896	4.182E-01	logistic	382667	218	382449	1.000	0.150
301	Personality disorders	mental disorders	rs6814407	-0.089	0.109	0.915	4.182E-01	logistic	381968	346	381622	1.000	0.150
580.14	Chronic glomerulonephritis, NOS	genitourinary	rs6814407	0.087	0.108	1.091	4.184E-01	logistic	381376	314	381062	1.000	0.150
578.2	Blood in stool	digestive	rs6814407	-0.038	0.047	0.963	4.198E-01	logistic	385103	1800	383303	1.000	0.150
594.3	Calculus of ureter	genitourinary	rs6814407	-0.040	0.050	0.961	4.202E-01	logistic	414010	1634	412376	1.000	0.150

ST31; PheWAS results for the lead cis-pQTLs of the 4 proteins prioritized (ACOX1, FGF5, FURIN, and MST1).

SNP = Single Nucleotide Polymorphism; SE = Standard error HWE_p = P-value for Hardy-Weinberg equilibrium test

Phenotype	Description	Group	SNP	Beta	SE	Odds tatio	P-value	Type	n_total	n_cases	n_controls	HWE_p	allele_freq
601.1	Prostatitis	genitourinary	rs6814407	-0.061	0.076	0.941	4.216E-01	logistic	157164	703	156461	1.000	0.150
783	Fever of unknown origin	symptoms	rs6814407	0.032	0.040	1.032	4.237E-01	logistic	418176	2397	415779	1.000	0.150
444.1	Arterial embolism and thrombosis of lower extremity arter	circulatory system	rs6814407	-0.076	0.096	0.927	4.260E-01	logistic	405915	446	405469	1.000	0.150
594	Urinary calculus	genitourinary	rs6814407	0.021	0.026	1.021	4.276E-01	logistic	418195	5819	412376	1.000	0.150
586.1	Anatomical abnormatilies of kidney and ureters	genitourinary	rs6814407	0.098	0.123	1.103	4.282E-01	logistic	381301	239	381062	1.000	0.150
363	Chorioretinal inflammations, scars, and other disorders of choroic	sense organs	rs6814407	-0.107	0.135	0.899	4.294E-01	logistic	397993	230	397763	1.000	0.150
471	Nasal polyps	respiratory	rs6814407	-0.049	0.062	0.952	4.303E-01	logistic	397549	1032	396517	1.000	0.150
246	Other disorders of thyroid	endocrine/metabolic	rs6814407	0.086	0.110	1.090	4.313E-01	logistic	392532	302	392230	1.000	0.150
537	Other disorders of stomach and duodenum	digestive	rs6814407	-0.037	0.047	0.964	4.316E-01	logistic	364031	1817	362214	1.000	0.150
521.1	Dental caries	digestive	rs6814407	0.056	0.071	1.057	4.323E-01	logistic	410280	744	409536	1.000	0.150
561	Symptoms involving digestive systen	digestive	rs6814407	0.022	0.028	1.022	4.327E-01	logistic	339539	5054	334485	1.000	0.150
172.1	Melanomas of skin, dx or hx	neoplasms	rs6814407	0.036	0.046	1.036	4.349E-01	logistic	409038	1830	407208	1.000	0.150
172.11	Melanomas of skin	neoplasms	rs6814407	0.036	0.046	1.036	4.349E-01	logistic	409038	1830	407208	1.000	0.150
367	Disorders of refraction and accommodation; blindness and low visior	sense organs	rs6814407	0.020	0.025	1.020	4.378E-01	logistic	417170	6164	411006	1.000	0.150
636.3	Hemorrhage in early pregnancy	pregnancy complications	rs6814407	-0.107	0.139	0.899	4.415E-01	logistic	222810	218	222592	1.000	0.150
626.12	Excessive or frequent menstruation	genitourinary	rs6814407	-0.033	0.043	0.967	4.421E-01	logistic	194922	2175	192747	1.000	0.150
626	Disorders of menstruation and other abnormal bleeding from female genital trac	genitourinary	rs6814407	-0.020	0.026	0.980	4.433E-01	logistic	198823	6076	192747	1.000	0.150
743.2	Pathologic fracture	musculoskeletal	rs6814407	-0.050	0.066	0.951	4.455E-01	logistic	422164	930	421234	1.000	0.150
594.1	Calculus of kidney	genitourinary	rs6814407	0.026	0.035	1.027	4.457E-01	logistic	415555	3179	412376	1.000	0.150
531.2	Gastric ulcer	digestive	rs6814407	0.036	0.048	1.037	4.466E-01	logistic	413012	1673	411339	1.000	0.150
292.4	Altered mental status	mental disorders	rs6814407	0.025	0.033	1.026	4.472E-01	logistic	408434	3461	404973	1.000	0.150
578.8	Hemorrhage of rectum and anus	digestive	rs6814407	0.031	0.040	1.031	4.478E-01	logistic	385630	2327	383303	1.000	0.150
516	Abnormal sputum	respiratory	rs6814407	-0.048	0.064	0.953	4.526E-01	logistic	421370	981	420389	1.000	0.150
626.8	Infertility, female	genitourinary	rs6814407	0.085	0.114	1.089	4.563E-01	logistic	193029	282	192747	1.000	0.150
389.3	Degenerative and vascular disorders of ear	sense organs	rs6814407	0.077	0.104	1.080	4.565E-01	logistic	408638	341	408297	1.000	0.150
595	Hydronephrosis	genitourinary	rs6814407	0.046	0.062	1.047	4.568E-01	logistic	413362	986	412376	1.000	0.150
292	Neurological disorders	mental disorders	rs6814407	0.020	0.027	1.020	4.574E-01	logistic	410372	5399	404973	1.000	0.150
575	Other biliary tract disease	digestive	rs6814407	-0.024	0.033	0.976	4.591E-01	logistic	398734	3687	395047	1.000	0.150
571	Chronic liver disease and cirrhosis	digestive	rs6814407	-0.019	0.026	0.981	4.591E-01	logistic	406135	5861	400274	1.000	0.150
276.5	Hypovolemia	endocrine/metabolic	rs6814407	-0.020	0.028	0.980	4.618E-01	logistic	400853	5281	395572	1.000	0.150
696	Psoriasis and related disorders	dermatologic	rs6814407	0.027	0.036	1.027	4.632E-01	logistic	402206	2916	399290	1.000	0.150
430.2	Intracerebral hemorrhage	circulatory system	rs6814407	-0.045	0.061	0.956	4.632E-01	logistic	403773	1088	402685	1.000	0.150
198	Secondary malignant neoplasm	neoplasms	rs6814407	-0.011	0.015	0.989	4.642E-01	logistic	403287	17281	386006	1.000	0.150
721.8	Other allied disorders of spine	musculoskeletal	rs6814407	-0.106	0.144	0.900	4.651E-01	logistic	408871	201	408670	1.000	0.150
389	Hearing loss	sense organs	rs6814407	0.017	0.023	1.017	4.662E-01	logistic	415421	7124	408297	1.000	0.150
395.4	Nonrheumatic pulmonary valve disorders	circulatory system	rs6814407	-0.082	0.113	0.921	4.678E-01	logistic	401735	321	401414	1.000	0.150
540.1	Appendicitis	digestive	rs6814407	0.077	0.106	1.080	4.680E-01	logistic	421984	328	421656	1.000	0.150
287	Purpura and other hemorrhagic conditions	hematopoietic	rs6814407	0.029	0.040	1.030	4.688E-01	logistic	419896	2343	417553	1.000	0.150
803.3	Fracture of clavicle or scapula	injuries & poisonings	rs6814407	-0.055	0.075	0.947	4.703E-01	logistic	397087	709	396378	1.000	0.150
560.2	Impaction of intestine	digestive	rs6814407	-0.098	0.135	0.907	4.704E-01	logistic	334714	229	334485	1.000	0.150
292.6	Hallucinations	mental disorders	rs6814407	0.075	0.104	1.078	4.705E-01	logistic	405317	344	404973	1.000	0.150
339	Other headache syndromes	neurological	rs6814407	-0.022	0.031	0.978	4.705E-01	logistic	409356	4169	405187	1.000	0.150
610	Benign mammary dysplasias	genitourinary	rs6814407	-0.065	0.090	0.937	4.724E-01	logistic	220777	505	220272	1.000	0.150
375.2	Epiphora	sense organs	rs6814407	-0.079	0.111	0.924	4.789E-01	logistic	412257	329	411928	1.000	0.150
701.2	Scar conditions and fibrosis of skin	dermatologic	rs6814407	0.069	0.097	1.071	4.795E-01	logistic	415576	390	415186	1.000	0.150
427.6	Premature beats	circulatory system	rs6814407	0.056	0.079	1.057	4.805E-01	logistic	372449	601	371848	1.000	0.150
634.1	Missed abortion/Hydatidiform mole	pregnancy complications	rs6814407	-0.098	0.139	0.907	4.810E-01	logistic	222807	215	222592	1.000	0.150
586.4	Stricture/obstruction of ureter	genitourinary	rs6814407	0.047	0.067	1.048	4.818E-01	logistic	381896	834	381062	1.000	0.150
695.7	Prurigo and Lichen	dermatologic	rs6814407	-0.082	0.116	0.921	4.820E-01	logistic	405510	304	405206	1.000	0.150
386.2	Peripheral or central vertigo	sense organs	rs6814407	-0.057	0.081	0.945	4.821E-01	logistic	410281	616	409665	1.000	0.150
752.11	Spina bifida	congenital anomalies	rs6814407	0.094	0.134	1.098	4.833E-01	logistic	422743	204	422539	1.000	0.150
204.4	Multiple myeloma	neoplasms	rs6814407	-0.041	0.059	0.959	4.838E-01	logistic	416757	1144	415613	1.000	0.150
702	Degenerative skin conditions and other dermatoses	dermatologic	rs6814407	-0.032	0.046	0.968	4.839E-01	logistic	407215	1867	405348	1.000	0.150
550.4	Umbilical hernia	digestive	rs6814407	0.036	0.052	1.037	4.840E-01	logistic	366811	1417	365394	1.000	0.150
564.9	Personal history of diseases of digestive systen	digestive	rs6814407	0.012	0.017	1.012	4.860E-01	logistic	348229	13744	334485	1.000	0.150
352.1	Trigeminal nerve disorders [CN5]	neurological	rs6814407	-0.063	0.090	0.939	4.871E-01	logistic	402912	497	402415	1.000	0.150
710	Osteomyelitis, periostitis, and other infections involving bone	musculoskeletal	rs6814407	-0.084	0.122	0.919	4.879E-01	logistic	412326	278	412048	1.000	0.150
681.1	Cellulitis and abscess of fingers/toes	dermatologic	rs6814407	-0.094	0.135	0.911	4.882E-01	logistic	418724	227	418497	1.000	0.150
427.2	Atrial fibrillation and flutter	circulatory system	rs6814407	-0.011	0.016	0.989	4.885E-01	logistic	388316	16468	371848	1.000	0.150
577	Diseases of pancreas	digestive	rs6814407	-0.024	0.035	0.976	4.910E-01	logistic	421247	3239	418008	1.000	0.150
687	Symptoms affecting skin	dermatologic	rs6814407	0.069	0.100	1.072	4.911E-01	logistic	413083	367	412716	1.000	0.150
270.33	Amyloidosis	endocrine/metabolic	rs6814407	-0.076	0.111	0.926	4.924E-01	logistic	422032	330	421702	1.000	0.150
958	Certain early complications of trauma or procedure	injuries & poisonings	rs6814407	-0.083	0.121	0.921	4.942E-01	logistic	423583	281	423302	1.000	0.150
427.11	Paroxysmal supraventricular tachycardia	circulatory system	rs6814407	-0.030	0.043	0.971	4.960E-01	logistic	373959	2111	371848	1.000	0.150
153	Colorectal cancer	neoplasms	rs6814407	0.016	0.024	1.016	4.970E-01	logistic	377768	7048	370720	1.000	0.150
642	Hypertension complicating pregnancy, childbirth, and the puerperium	pregnancy complications	rs6814407	-0.064	0.095	0.938	4.973E-01	logistic	228250	456	227794	1.000	0.150
729	Other disorders of soft tissues	musculoskeletal	rs6814407	0.081	0.121	1.085	5.032E-01	logistic	389423	249	389174	1.000	0.150
800.1	Fracture of neck of femur	injuries & poisonings	rs6814407	-0.029	0.044	0.971	5.046E-01	logistic	398432	2054	396378	1.000	0.150
334	Degenerative disease of the spinal cord	neurological	rs6814407	-0.032	0.048	0.969	5.069E-01	logistic	400789	1742	399047	1.000	0.150
374.1	Ectropion or entropion	sense organs	rs6814407	-0.050	0.076	0.951	5.073E-01	logistic	411493	700	410793	1.000	0.150
592.12	Chronic cystitis	genitourinary	rs6814407	-0.085	0.129	0.918	5.074E-01	logistic	377176	249	376927	1.000	0.150

ST31; PheWAS results for the lead cis-pQTLs of the 4 proteins prioritized (ACOX1, FGF5, FURIN, and MST1).

SNP = Single Nucleotide Polymorphism; SE = Standard error HWE_p = P-value for Hardy-Weinberg equilibrium test

Phenotype	Description	Group	SNP	Beta	SE	Odds tatio	P-value	Type	n_total	n_cases	n_controls	HWE_p	allele_freq
172.2	Other non-epithelial cancer of skin	neoplasms	rs6814407	-0.022	0.033	0.978	5.075E-01	logistic	410817	3609	407208	1.000	0.150
394.1	Mitral valve stenosis and aortic valve stenosis	circulatory system	rs6814407	-0.040	0.060	0.961	5.075E-01	logistic	402530	1116	401414	1.000	0.150
596.1	Bladder neck obstruction	genitourinary	rs6814407	0.054	0.082	1.056	5.083E-01	logistic	402115	561	401554	1.000	0.150
577.3	Cyst and pseudocyst of pancreas	digestive	rs6814407	-0.058	0.089	0.943	5.100E-01	logistic	418522	514	418008	1.000	0.150
454.1	Varicose veins of lower extremity	circulatory system	rs6814407	0.019	0.029	1.019	5.134E-01	logistic	385486	4520	380966	1.000	0.150
250.23	Type 2 diabetes with ophthalmic manifestations	endocrine/metabolic	rs6814407	0.029	0.044	1.029	5.142E-01	logistic	388590	1953	386637	1.000	0.150
41.4	E. coli	infectious diseases	rs6814407	0.022	0.034	1.022	5.161E-01	logistic	399991	3344	396647	1.000	0.150
516.1	Hemoptysis	respiratory	rs6814407	-0.043	0.066	0.958	5.170E-01	logistic	421320	931	420389	1.000	0.150
857	Mechanical complication of unspecified genitourinary device, implant, and graf	injuries & poisonings	rs6814407	0.032	0.049	1.032	5.174E-01	logistic	418243	1591	416652	1.000	0.150
599.5	Frequency of urination and polyuria	genitourinary	rs6814407	0.043	0.066	1.044	5.197E-01	logistic	390965	859	390106	1.000	0.150
272	Disorders of lipoid metabolism	endocrine/metabolic	rs6814407	0.007	0.011	1.007	5.234E-01	logistic	394625	38860	355765	1.000	0.150
352	Disorders of other cranial nerves	neurological	rs6814407	-0.036	0.057	0.964	5.249E-01	logistic	403637	1222	402415	1.000	0.150
198.4	Secondary malignant neoplasm of liver	neoplasms	rs6814407	-0.017	0.027	0.983	5.251E-01	logistic	391482	5476	386006	1.000	0.150
272.1	Hyperlipidemia	endocrine/metabolic	rs6814407	0.007	0.011	1.007	5.251E-01	logistic	394381	38616	355765	1.000	0.150
802	Fracture of pelvis	injuries & poisonings	rs6814407	0.040	0.063	1.041	5.264E-01	logistic	397331	953	396378	1.000	0.150
741	Symptoms and disorders of the joints	musculoskeletal	rs6814407	-0.032	0.050	0.969	5.270E-01	logistic	416612	1602	415010	1.000	0.150
375	Disorders of lacrimal system	sense organs	rs6814407	-0.033	0.052	0.968	5.297E-01	logistic	413379	1451	411928	1.000	0.150
38	Septicemia	infectious diseases	rs6814407	0.015	0.023	1.015	5.301E-01	logistic	403876	7229	396647	1.000	0.150
386.9	Dizziness and giddiness (Light-headedness and vertigo)	sense organs	rs6814407	0.022	0.036	1.023	5.311E-01	logistic	412711	3046	409665	1.000	0.150
522	Diseases of pulp and periapical tissues	digestive	rs6814407	0.070	0.112	1.072	5.339E-01	logistic	409832	296	409536	1.000	0.150
165.1	Cancer of bronchus; lung	neoplasms	rs6814407	0.019	0.030	1.019	5.340E-01	logistic	421452	4223	417229	1.000	0.150
260	Protein-calorie malnutrition	endocrine/metabolic	rs6814407	0.062	0.100	1.064	5.344E-01	logistic	412473	376	412097	1.000	0.150
735.21	Hammer toe (acquired)	musculoskeletal	rs6814407	-0.059	0.095	0.943	5.365E-01	logistic	403105	451	402654	1.000	0.150
726	Peripheral enthesopathies and allied syndromes	musculoskeletal	rs6814407	-0.012	0.019	0.988	5.369E-01	logistic	399760	10586	389174	1.000	0.150
384	Other disorders of tympanic membrane	sense organs	rs6814407	0.062	0.101	1.064	5.370E-01	logistic	419080	364	418716	1.000	0.150
180.3	Cervical intraepithelial neoplasia [CIN] [Cervical dysplasia	neoplasms	rs6814407	-0.057	0.092	0.945	5.377E-01	logistic	196385	481	195904	1.000	0.150
366.2	Senile cataract	sense organs	rs6814407	0.013	0.021	1.013	5.399E-01	logistic	380814	9136	371678	1.000	0.150
801.1	Fracture of foot	injuries & poisonings	rs6814407	0.079	0.130	1.083	5.407E-01	logistic	396597	219	396378	1.000	0.150
722.9	Other and unspecified disc disorder	musculoskeletal	rs6814407	-0.053	0.088	0.948	5.444E-01	logistic	409194	524	408670	1.000	0.150
394.7	Disease of tricuspid valve	circulatory system	rs6814407	0.046	0.076	1.047	5.459E-01	logistic	402072	658	401414	1.000	0.150
742	Derangement of joint, non-traumatic	musculoskeletal	rs6814407	0.055	0.093	1.057	5.501E-01	logistic	415444	434	415010	1.000	0.150
411.4	Coronary atherosclerosis	circulatory system	rs6814407	0.008	0.014	1.008	5.501E-01	logistic	394337	23787	370550	1.000	0.150
622.1	Polyp of corpus uteri	genitourinary	rs6814407	-0.029	0.050	0.971	5.545E-01	logistic	215181	1609	213572	1.000	0.150
149.1	Cancer of oropharynx	neoplasms	rs6814407	0.058	0.099	1.060	5.551E-01	logistic	420725	383	420342	1.000	0.150
251.1	Hypoglycemia	endocrine/metabolic	rs6814407	-0.030	0.051	0.970	5.555E-01	logistic	383028	1531	381497	1.000	0.150
300.13	Phobia	mental disorders	rs6814407	-0.048	0.082	0.953	5.600E-01	logistic	382219	597	381622	1.000	0.150
38.2	Gram positive septicemia	infectious diseases	rs6814407	-0.046	0.080	0.955	5.639E-01	logistic	397268	621	396647	1.000	0.150
357	Inflammatory and toxic neuropathy	neurological	rs6814407	0.025	0.044	1.026	5.641E-01	logistic	421163	1995	419168	1.000	0.150
386.1	Meniere's disease	sense organs	rs6814407	0.044	0.077	1.045	5.651E-01	logistic	410308	643	409665	1.000	0.150
277.4	Disorders of bilirubin excretion	endocrine/metabolic	rs6814407	-0.054	0.093	0.948	5.652E-01	logistic	420403	462	419941	1.000	0.150
276.13	Hyperpotassemia	endocrine/metabolic	rs6814407	0.027	0.047	1.027	5.666E-01	logistic	397299	1727	395572	1.000	0.150
586.2	Cyst of kidney, acquired	genitourinary	rs6814407	0.026	0.045	1.026	5.668E-01	logistic	382916	1854	381062	1.000	0.150
446.5	Giant cell arteritis	circulatory system	rs6814407	0.045	0.079	1.046	5.677E-01	logistic	406071	602	405469	1.000	0.150
427.21	Atrial fibrillation	circulatory system	rs6814407	0.017	0.030	1.017	5.699E-01	logistic	376121	4273	371848	1.000	0.150
358.1	Myasthenia gravis	neurological	rs6814407	-0.066	0.117	0.936	5.710E-01	logistic	419463	295	419168	1.000	0.150
501	Pneumonitis due to inhalation of food or vomitus	respiratory	rs6814407	-0.027	0.047	0.974	5.714E-01	logistic	392843	1786	391057	1.000	0.150
277	Other disorders of metabolism	endocrine/metabolic	rs6814407	0.033	0.059	1.034	5.734E-01	logistic	421029	1088	419941	1.000	0.150
350	Abnormal movement	neurological	rs6814407	-0.032	0.058	0.968	5.765E-01	logistic	421410	1200	420210	1.000	0.150
946	Anaphylactic shock NOS	injuries & poisonings	rs6814407	0.072	0.129	1.074	5.781E-01	logistic	415210	223	414987	1.000	0.150
586.11	Small kidney	genitourinary	rs6814407	0.072	0.130	1.074	5.800E-01	logistic	381281	219	381062	1.000	0.150
509.8	Dependence on respirator [Ventilator] or supplemental oxygen	respiratory	rs6814407	-0.042	0.076	0.959	5.819E-01	logistic	391755	698	391057	1.000	0.150
613	Other nonmalignant breast conditions	genitourinary	rs6814407	-0.055	0.101	0.946	5.850E-01	logistic	421594	397	421197	1.000	0.150
575.9	Nonspecific abnormal findings on radiological and other examination of biliary trac	digestive	rs6814407	0.069	0.127	1.072	5.856E-01	logistic	395275	228	395047	1.000	0.150
427.22	Atrial flutter	circulatory system	rs6814407	-0.043	0.078	0.958	5.864E-01	logistic	372499	651	371848	1.000	0.150
614.1	Pelvic peritoneal adhesions, female (postoperative) (postinfection	genitourinary	rs6814407	0.053	0.098	1.054	5.879E-01	logistic	217833	395	217438	1.000	0.150
367.9	Blindness and low vision	sense organs	rs6814407	-0.036	0.068	0.964	5.889E-01	logistic	411881	875	411006	1.000	0.150
512	Other symptoms of respiratory system	respiratory	rs6814407	0.013	0.025	1.013	5.894E-01	logistic	411090	6491	404599	1.000	0.150
481	Influenza	respiratory	rs6814407	-0.050	0.093	0.951	5.917E-01	logistic	397264	464	396800	1.000	0.150
475	Chronic sinusitis	respiratory	rs6814407	0.034	0.063	1.034	5.919E-01	logistic	397468	951	396517	1.000	0.150
578.1	Hematemesis	digestive	rs6814407	-0.029	0.055	0.971	5.925E-01	logistic	384647	1344	383303	1.000	0.150
117	Mycoses	infectious diseases	rs6814407	0.062	0.117	1.064	5.930E-01	logistic	418652	272	418380	1.000	0.150
217	Vascular hamartomas and non-neoplastic nevi	neoplasms	rs6814407	-0.062	0.116	0.940	5.934E-01	logistic	414259	301	413958	1.000	0.150
477	Epistaxis or throat hemorrhage	respiratory	rs6814407	-0.030	0.055	0.971	5.934E-01	logistic	397807	1290	396517	1.000	0.150
496.1	Emphysema	respiratory	rs6814407	0.019	0.036	1.019	5.975E-01	logistic	401774	2965	398809	1.000	0.150
619.2	Disorders of uterus, NEC	genitourinary	rs6814407	-0.052	0.099	0.950	6.014E-01	logistic	217379	413	216966	1.000	0.150
585.33	Chronic Kidney Disease, Stage III	genitourinary	rs6814407	0.012	0.023	1.012	6.017E-01	logistic	388537	7475	381062	1.000	0.150
530.13	Barrett's esophagus	digestive	rs6814407	0.015	0.030	1.016	6.030E-01	logistic	361195	4407	356788	1.000	0.150
149	Cancer of larynx, pharynx, nasal cavities	neoplasms	rs6814407	0.035	0.067	1.035	6.031E-01	logistic	421191	849	420342	1.000	0.150
70	Viral hepatitis	infectious diseases	rs6814407	-0.044	0.085	0.957	6.041E-01	logistic	415755	551	415204	1.000	0.150
800	Fracture of lower limb	injuries & poisonings	rs6814407	-0.019	0.036	0.982	6.049E-01	logistic	399482	3104	396378	1.000	0.150
270	Disorders of protein plasma/amino-acid transport and metabolism	endocrine/metabolic	rs6814407	-0.037	0.071	0.964	6.058E-01	logistic	422490	788	421702	1.000	0.150

ST31; PheWAS results for the lead cis-pQTLs of the 4 proteins prioritized (ACOX1, FGF5, FURIN, and MST1).

SNP = Single Nucleotide Polymorphism; SE = Standard error HWE_p = P-value for Hardy-Weinberg equilibrium test

Phenotype	Description	Group	SNP	Beta	SE	Odds tatio	P-value	Type	n_total	n_cases	n_controls	HWE_p	allele_freq
443	Peripheral vascular disease	circulatory system	rs6814407	-0.014	0.028	0.986	6.059E-01	logistic	410595	5126	405469	1.000	0.150
401.1	Essential hypertension	circulatory system	rs6814407	0.004	0.008	1.004	6.068E-01	logistic	389876	99213	290663	1.000	0.150
361	Retinal detachments and defects	sense organs	rs6814407	0.044	0.086	1.045	6.069E-01	logistic	398276	513	397763	1.000	0.150
442	Other aneurysm	circulatory system	rs6814407	-0.019	0.036	0.982	6.079E-01	logistic	408493	3024	405469	1.000	0.150
585.34	Chronic Kidney Disease, Stage IV	genitourinary	rs6814407	0.029	0.058	1.030	6.101E-01	logistic	382213	1151	381062	1.000	0.150
272.9	Unspecified disorder of lipoid metabolism	endocrine/metabolic	rs6814407	0.055	0.108	1.057	6.103E-01	logistic	356085	320	355765	1.000	0.150
276	Disorders of fluid, electrolyte, and acid-base balance	endocrine/metabolic	rs6814407	-0.008	0.016	0.992	6.108E-01	logistic	411277	15705	395572	1.000	0.150
597.1	Urethral stricture (not specified as infectious)	genitourinary	rs6814407	0.023	0.046	1.024	6.118E-01	logistic	403370	1816	401554	1.000	0.150
530.11	GERD	digestive	rs6814407	0.008	0.016	1.008	6.131E-01	logistic	371656	14868	356788	1.000	0.150
585	Renal failure	genitourinary	rs6814407	0.007	0.014	1.007	6.151E-01	logistic	403986	22924	381062	1.000	0.150
686	Other local infections of skin and subcutaneous tissue	dermatologic	rs6814407	-0.031	0.061	0.970	6.151E-01	logistic	419554	1057	418497	1.000	0.150
371.1	Uveitis, noninfectious or NOS	sense organs	rs6814407	-0.063	0.127	0.939	6.162E-01	logistic	411047	254	410793	1.000	0.150
689	Disorder of skin and subcutaneous tissue NOS	dermatologic	rs6814407	-0.021	0.043	0.979	6.224E-01	logistic	413188	2098	411090	1.000	0.150
722	Intervertebral disc disorders	musculoskeletal	rs6814407	-0.038	0.078	0.962	6.226E-01	logistic	409333	663	408670	1.000	0.150
250.2	Type 2 diabetes	endocrine/metabolic	rs6814407	0.006	0.013	1.006	6.229E-01	logistic	413206	26569	386637	1.000	0.150
579.2	Splenomegaly	digestive	rs6814407	0.038	0.078	1.039	6.264E-01	logistic	383919	616	383303	1.000	0.150
297	Suicidal ideation or attempt	mental disorders	rs6814407	0.050	0.102	1.051	6.269E-01	logistic	381982	360	381622	1.000	0.150
255.1	Adrenal hyperfunction	endocrine/metabolic	rs6814407	-0.069	0.142	0.933	6.278E-01	logistic	415447	201	415246	1.000	0.150
752.1	Neural tube defects	congenital anomalies	rs6814407	0.047	0.098	1.048	6.280E-01	logistic	422933	394	422539	1.000	0.150
578	Gastrointestinal hemorrhage	digestive	rs6814407	-0.010	0.021	0.990	6.285E-01	logistic	392729	9426	383303	1.000	0.150
345.11	Generalized convulsive epilepsy	neurological	rs6814407	0.047	0.098	1.048	6.293E-01	logistic	399441	394	399047	1.000	0.150
252.2	Hypoparathyroidism	endocrine/metabolic	rs6814407	-0.065	0.134	0.937	6.304E-01	logistic	415471	225	415246	1.000	0.150
218.1	Uterine leiomyoma	neoplasms	rs6814407	-0.018	0.038	0.982	6.305E-01	logistic	209756	2761	206995	1.000	0.150
599.2	Retention of urine	genitourinary	rs6814407	0.012	0.024	1.012	6.307E-01	logistic	396988	6882	390106	1.000	0.150
536	Disorders of function of stomach	digestive	rs6814407	0.023	0.048	1.023	6.311E-01	logistic	363858	1644	362214	1.000	0.150
165	Cancer within the respiratory system	neoplasms	rs6814407	0.013	0.027	1.013	6.315E-01	logistic	422554	5325	417229	1.000	0.150
586	Other disorders of the kidney and ureters	genitourinary	rs6814407	0.015	0.032	1.015	6.324E-01	logistic	384904	3842	381062	1.000	0.150
577.1	Acute pancreatitis	digestive	rs6814407	-0.022	0.046	0.978	6.336E-01	logistic	419858	1850	418008	1.000	0.150
250.1	Type 1 diabetes	endocrine/metabolic	rs6814407	0.019	0.040	1.019	6.340E-01	logistic	389041	2404	386637	1.000	0.150
497	Bronchitis	respiratory	rs6814407	0.053	0.112	1.055	6.352E-01	logistic	399105	296	398809	1.000	0.150
340	Migraine	neurological	rs6814407	0.018	0.039	1.019	6.370E-01	logistic	407732	2545	405187	1.000	0.150
250	Diabetes mellitus	endocrine/metabolic	rs6814407	0.006	0.012	1.006	6.402E-01	logistic	414239	27602	386637	1.000	0.150
585.32	End stage renal disease	genitourinary	rs6814407	0.030	0.064	1.030	6.406E-01	logistic	381994	932	381062	1.000	0.150
580.3	Nephritis and nephropathy without mention of glomerulonephritis	genitourinary	rs6814407	-0.055	0.117	0.947	6.417E-01	logistic	381356	294	381062	1.000	0.150
967	Adverse effects of sedatives or other central nervous system depressants and anesthetics	injuries & poisonings	rs6814407	-0.062	0.134	0.940	6.442E-01	logistic	367398	225	367173	1.000	0.150
556.1	Ulceration of intestine	digestive	rs6814407	-0.066	0.143	0.936	6.446E-01	logistic	334685	200	334485	1.000	0.150
427.12	Paroxysmal ventricular tachycardia	circulatory system	rs6814407	-0.029	0.063	0.971	6.465E-01	logistic	372834	986	371848	1.000	0.150
500.2	Pneumoconiosis	respiratory	rs6814407	0.046	0.100	1.047	6.471E-01	logistic	391432	375	391057	1.000	0.150
601	Inflammatory diseases of prostate	genitourinary	rs6814407	-0.030	0.066	0.970	6.480E-01	logistic	157382	921	156461	1.000	0.150
433.1	Occlusion and stenosis of precerebral arteries	circulatory system	rs6814407	-0.023	0.050	0.977	6.481E-01	logistic	404257	1572	402685	1.000	0.150
401	Hypertension	circulatory system	rs6814407	0.003	0.008	1.003	6.499E-01	logistic	390097	99434	290663	1.000	0.150
323	Encephalitis	neurological	rs6814407	-0.048	0.108	0.953	6.524E-01	logistic	422420	349	422071	1.000	0.150
41.2	Streptococcus infection	infectious diseases	rs6814407	0.023	0.050	1.023	6.525E-01	logistic	398155	1508	396647	1.000	0.150
53	Herpes zoster	infectious diseases	rs6814407	0.046	0.102	1.047	6.528E-01	logistic	415563	359	415204	1.000	0.150
702.1	Actinic keratosis	dermatologic	rs6814407	0.029	0.064	1.029	6.546E-01	logistic	413997	925	413072	1.000	0.150
577.2	Chronic pancreatitis	digestive	rs6814407	0.035	0.079	1.036	6.573E-01	logistic	418611	603	418008	1.000	0.150
197	Chemotherapy	neoplasms	rs6814407	-0.008	0.019	0.992	6.575E-01	logistic	397532	11526	386006	1.000	0.150
159.4	Malignant neoplasm of retroperitoneum and peritoneum	neoplasms	rs6814407	-0.055	0.123	0.947	6.580E-01	logistic	394127	265	393862	1.000	0.150
627	Menopausal and postmenopausal disorders	genitourinary	rs6814407	-0.017	0.038	0.983	6.584E-01	logistic	195450	2703	192747	1.000	0.150
536.8	Dyspepsia and other specified disorders of function of stomach	digestive	rs6814407	0.021	0.048	1.022	6.589E-01	logistic	363851	1637	362214	1.000	0.150
798	Malaise and fatigue	symptoms	rs6814407	-0.013	0.030	0.987	6.589E-01	logistic	418584	4264	414320	1.000	0.150
727.1	Synovitis and tenosynovitis	musculoskeletal	rs6814407	-0.050	0.113	0.951	6.593E-01	logistic	389486	312	389174	1.000	0.150
580.2	Nephrotic syndrome without mention of glomerulonephritis	genitourinary	rs6814407	-0.044	0.101	0.957	6.599E-01	logistic	381457	395	381062	1.000	0.150
454	Varicose veins	circulatory system	rs6814407	0.012	0.028	1.012	6.617E-01	logistic	385827	4861	380966	1.000	0.150
619	Noninflammatory female genital disorders	genitourinary	rs6814407	0.018	0.042	1.018	6.626E-01	logistic	219175	2209	216966	1.000	0.150
800.2	Fracture of unspecified part of femur	injuries & poisonings	rs6814407	-0.060	0.138	0.942	6.637E-01	logistic	396592	214	396378	1.000	0.150
585.1	Acute renal failure	genitourinary	rs6814407	0.008	0.018	1.008	6.639E-01	logistic	394375	13313	381062	1.000	0.150
614	Inflammatory diseases of female pelvic organs	genitourinary	rs6814407	0.022	0.050	1.022	6.666E-01	logistic	218979	1541	217438	1.000	0.150
707.1	Decubitus ulcer	dermatologic	rs6814407	-0.016	0.036	0.984	6.667E-01	logistic	419126	2996	416130	1.000	0.150
530.5	Disorders of esophageal motility	digestive	rs6814407	0.040	0.094	1.041	6.681E-01	logistic	357212	424	356788	1.000	0.150
601.12	Chronic prostatitis	genitourinary	rs6814407	-0.056	0.132	0.945	6.692E-01	logistic	156694	233	156461	1.000	0.150
455	Hemorrhoids	circulatory system	rs6814407	0.012	0.027	1.012	6.697E-01	logistic	386244	5278	380966	1.000	0.150
509.2	Respiratory insufficiency	respiratory	rs6814407	-0.023	0.054	0.977	6.698E-01	logistic	392402	1345	391057	1.000	0.150
427.4	Cardiac arrest and ventricular fibrillation	circulatory system	rs6814407	0.028	0.065	1.028	6.713E-01	logistic	372755	907	371848	1.000	0.150
571.81	Portal hypertension	digestive	rs6814407	0.028	0.067	1.029	6.724E-01	logistic	401135	861	400274	1.000	0.150
367.8	Hypermetropia	sense organs	rs6814407	0.049	0.116	1.050	6.727E-01	logistic	411285	279	411006	1.000	0.150
327	Sleep disorders	neurological	rs6814407	-0.035	0.084	0.965	6.748E-01	logistic	413949	565	413384	1.000	0.150
411.1	Unstable angina (intermediate coronary syndrome)	circulatory system	rs6814407	-0.014	0.033	0.986	6.763E-01	logistic	374269	3719	370550	1.000	0.150
433.31	Transient cerebral ischemia	circulatory system	rs6814407	0.023	0.056	1.024	6.769E-01	logistic	403901	1216	402685	1.000	0.150
244	Hypothyroidism	endocrine/metabolic	rs6814407	0.006	0.015	1.006	6.771E-01	logistic	411296	19066	392230	1.000	0.150
276.14	Hypopotassemia	endocrine/metabolic	rs6814407	-0.014	0.034	0.986	6.781E-01	logistic	399074	3502	395572	1.000	0.150

ST31; PheWAS results for the lead cis-pQTLs of the 4 proteins prioritized (ACOX1, FGF5, FURIN, and MST1).

SNP = Single Nucleotide Polymorphism; SE = Standard error HWE_p = P-value for Hardy-Weinberg equilibrium test

Phenotype	Description	Group	SNP	Beta	SE	Odds tatio	P-value	Type	n_total	n_cases	n_controls	HWE_p	allele_freq
117.4	Aspergillosis	infectious diseases	rs6814407	0.055	0.133	1.057	6.783E-01	logistic	418590	210	418380	1.000	0.150
694	Dyschromia and Vitiligo	dermatologic	rs6814407	0.046	0.112	1.047	6.803E-01	logistic	405505	299	405206	1.000	0.150
289.5	Diseases of spleen	hematopoietic	rs6814407	0.029	0.071	1.029	6.816E-01	logistic	409070	761	408309	1.000	0.150
681	Superficial cellulitis and abscess	dermatologic	rs6814407	-0.047	0.115	0.954	6.853E-01	logistic	418799	302	418497	1.000	0.150
170	Cancer of bone and connective tissue	neoplasms	rs6814407	0.041	0.102	1.042	6.860E-01	logistic	423460	359	423101	1.000	0.150
575.8	Other disorders of biliary tract	digestive	rs6814407	-0.025	0.061	0.976	6.863E-01	logistic	396103	1056	395047	1.000	0.150
707.2	Chronic ulcer of leg or foot	dermatologic	rs6814407	0.017	0.043	1.017	6.884E-01	logistic	418232	2102	416130	1.000	0.150
278.1	Obesity	endocrine/metabolic	rs6814407	0.007	0.016	1.007	6.887E-01	logistic	405667	15114	390553	1.000	0.150
275.3	Disorders of magnesium metabolism	endocrine/metabolic	rs6814407	0.020	0.050	1.020	6.894E-01	logistic	417729	1544	416185	1.000	0.150
766	Neuralgia, neuritis, and radiculitis NOS	symptoms	rs6814407	0.046	0.116	1.047	6.900E-01	logistic	418267	281	417986	1.000	0.150
710.1	Osteomyelitis	musculoskeletal	rs6814407	-0.053	0.133	0.949	6.913E-01	logistic	412277	229	412048	1.000	0.150
627.1	Postmenopausal bleeding	genitourinary	rs6814407	0.018	0.046	1.019	6.914E-01	logistic	194538	1791	192747	1.000	0.150
155.1	Malignant neoplasm of liver, primary	neoplasms	rs6814407	0.042	0.107	1.043	6.938E-01	logistic	394187	325	393862	1.000	0.150
278	Overweight, obesity and other hyperalimentation	endocrine/metabolic	rs6814407	0.006	0.016	1.006	6.955E-01	logistic	405726	15173	390553	1.000	0.150
560.4	Other intestinal obstruction	digestive	rs6814407	-0.017	0.043	0.983	6.960E-01	logistic	336617	2132	334485	1.000	0.150
324	Other CNS infection and poliomyelitis	neurological	rs6814407	0.041	0.104	1.042	6.962E-01	logistic	422418	347	422071	1.000	0.150
334.2	Anterior horn cell disease	neurological	rs6814407	-0.037	0.094	0.964	6.975E-01	logistic	399497	450	399047	1.000	0.150
496.2	Chronic bronchitis	respiratory	rs6814407	-0.010	0.027	0.990	7.010E-01	logistic	404328	5519	398809	1.000	0.150
172	Skin cancer	neoplasms	rs6814407	-0.010	0.026	0.990	7.011E-01	logistic	413263	6055	407208	1.000	0.150
300	Anxiety disorders	mental disorders	rs6814407	-0.008	0.020	0.992	7.027E-01	logistic	391438	9816	381622	1.000	0.150
697	Sarcoidosis	dermatologic	rs6814407	-0.031	0.082	0.969	7.034E-01	logistic	405795	589	405206	1.000	0.150
618.2	Uterine/Uterovaginal prolapse	genitourinary	rs6814407	-0.026	0.067	0.975	7.040E-01	logistic	214329	874	213455	1.000	0.150
279	Disorders involving the immune mechanism	endocrine/metabolic	rs6814407	0.038	0.101	1.039	7.058E-01	logistic	423247	371	422876	1.000	0.150
599.4	Urinary incontinence	genitourinary	rs6814407	-0.014	0.038	0.986	7.060E-01	logistic	392806	2700	390106	1.000	0.150
496.21	Obstructive chronic bronchitis	respiratory	rs6814407	-0.010	0.027	0.990	7.080E-01	logistic	404163	5354	398809	1.000	0.150
695	Erythematous conditions	dermatologic	rs6814407	0.017	0.046	1.017	7.084E-01	logistic	407007	1801	405206	1.000	0.150
573.7	Abnormal results of function study of liver	digestive	rs6814407	0.012	0.032	1.012	7.098E-01	logistic	404090	3816	400274	1.000	0.150
204	Leukemia	neoplasms	rs6814407	-0.013	0.036	0.987	7.100E-01	logistic	418621	3008	415613	1.000	0.150
283	Acquired hemolytic anemias	hematopoietic	rs6814407	0.051	0.136	1.052	7.103E-01	logistic	382649	200	382449	1.000	0.150
261.4	Vitamin D deficiency	endocrine/metabolic	rs6814407	-0.015	0.042	0.985	7.107E-01	logistic	414357	2260	412097	1.000	0.150
333.8	Other degenerative diseases of the basal ganglia	neurological	rs6814407	-0.049	0.134	0.952	7.136E-01	logistic	399271	224	399047	1.000	0.150
580.32	Nephritis and nephropathy with pathological lesion	genitourinary	rs6814407	-0.044	0.121	0.957	7.136E-01	logistic	381336	274	381062	1.000	0.150
365.1	Open-angle glaucoma	sense organs	rs6814407	0.018	0.050	1.018	7.144E-01	logistic	399325	1562	397763	1.000	0.150
496	Chronic airway obstruction	respiratory	rs6814407	0.006	0.015	1.006	7.152E-01	logistic	416701	17892	398809	1.000	0.150
333	Extrapyramidal disease and abnormal movement disorders	neurological	rs6814407	0.020	0.056	1.021	7.159E-01	logistic	400270	1223	399047	1.000	0.150
687.4	Disturbance of skin sensation	dermatologic	rs6814407	0.019	0.052	1.019	7.163E-01	logistic	414113	1397	412716	1.000	0.150
217.1	Nevus, non-neoplastic	neoplasms	rs6814407	-0.044	0.121	0.957	7.170E-01	logistic	414233	275	413958	1.000	0.150
747.12	Valvular heart disease/ heart chambers	congenital anomalies	rs6814407	-0.043	0.120	0.958	7.185E-01	logistic	421875	280	421595	1.000	0.150
293.1	Swelling, mass, or lump in head and neck [Space-occupying lesion, intracranial NOS]	mental disorders	rs6814407	0.039	0.109	1.040	7.195E-01	logistic	420026	315	419711	1.000	0.150
619.4	Noninflammatory disorders of vagina	genitourinary	rs6814407	0.046	0.129	1.047	7.197E-01	logistic	217194	228	216966	1.000	0.150
480.5	Bronchopneumonia and lung abscess	respiratory	rs6814407	-0.035	0.096	0.966	7.197E-01	logistic	397229	429	396800	1.000	0.150
530.14	Reflux esophagitis	digestive	rs6814407	-0.013	0.035	0.988	7.199E-01	logistic	360032	3244	356788	1.000	0.150
458.1	Orthostatic hypotension	circulatory system	rs6814407	-0.013	0.035	0.987	7.216E-01	logistic	375723	3151	372572	1.000	0.150
580	Nephritis; nephrosis; renal sclerosis	genitourinary	rs6814407	-0.021	0.061	0.979	7.233E-01	logistic	382137	1075	381062	1.000	0.150
199	Neoplasm of uncertain behavior	neoplasms	rs6814407	0.028	0.079	1.028	7.239E-01	logistic	386620	614	386006	1.000	0.150
585.31	Renal dialysis	genitourinary	rs6814407	0.026	0.073	1.026	7.242E-01	logistic	381775	713	381062	1.000	0.150
427.3	Other specified cardiac dysrhythmias	circulatory system	rs6814407	0.014	0.039	1.014	7.248E-01	logistic	374456	2608	371848	1.000	0.150
275.5	Disorders of calcium/phosphorus metabolism	endocrine/metabolic	rs6814407	-0.014	0.041	0.986	7.258E-01	logistic	418502	2317	416185	1.000	0.150
470	Septal Deviations/Turbinate Hypertrophy	respiratory	rs6814407	0.017	0.048	1.017	7.262E-01	logistic	398163	1646	396517	1.000	0.150
574.11	Cholelithiasis with acute cholecystitis	digestive	rs6814407	-0.022	0.062	0.978	7.263E-01	logistic	396059	1012	395047	1.000	0.150
333.4	Torsion dystonia	neurological	rs6814407	0.039	0.112	1.040	7.274E-01	logistic	399349	302	399047	1.000	0.150
994	Sepsis and SIRS	injuries & poisonings	rs6814407	0.008	0.024	1.008	7.290E-01	logistic	418535	6755	411780	1.000	0.150
994.2	Sepsis	injuries & poisonings	rs6814407	0.008	0.024	1.008	7.290E-01	logistic	418535	6755	411780	1.000	0.150
801	Fracture of ankle and foot	injuries & poisonings	rs6814407	-0.027	0.077	0.974	7.298E-01	logistic	397040	662	396378	1.000	0.150
416	Cardiomegaly	circulatory system	rs6814407	-0.010	0.030	0.990	7.301E-01	logistic	417255	4417	412838	1.000	0.150
300.1	Anxiety disorder	mental disorders	rs6814407	-0.007	0.021	0.993	7.302E-01	logistic	390805	9183	381622	1.000	0.150
214	Lipoma	neoplasms	rs6814407	0.020	0.059	1.020	7.308E-01	logistic	415339	1112	414227	1.000	0.150
590	Pyelonephritis	genitourinary	rs6814407	0.018	0.053	1.018	7.324E-01	logistic	378294	1367	376927	1.000	0.150
472	Chronic pharyngitis and nasopharyngitis	respiratory	rs6814407	0.041	0.120	1.042	7.325E-01	logistic	396779	262	396517	1.000	0.150
157	Pancreatic cancer	neoplasms	rs6814407	0.018	0.052	1.018	7.327E-01	logistic	395256	1394	393862	1.000	0.150
508	Pulmonary collapse; interstitial and compensatory emphysema	respiratory	rs6814407	-0.011	0.032	0.989	7.328E-01	logistic	394890	3833	391057	1.000	0.150
290.1	Dementias	mental disorders	rs6814407	0.010	0.030	1.010	7.338E-01	logistic	409256	4283	404973	1.000	0.150
579	Other symptoms involving abdomen and pelvis	digestive	rs6814407	0.021	0.061	1.021	7.342E-01	logistic	384340	1037	383303	1.000	0.150
286.12	Congenital deficiency of other clotting factors (including factor VII	hematopoietic	rs6814407	-0.044	0.130	0.957	7.369E-01	logistic	417788	235	417553	1.000	0.150
386	Vertiginous syndromes and other disorders of vestibular system	sense organs	rs6814407	0.016	0.048	1.016	7.377E-01	logistic	411346	1681	409665	1.000	0.150
573.5	Jaundice (not of newborn)	digestive	rs6814407	0.025	0.074	1.025	7.381E-01	logistic	400970	696	400274	1.000	0.150
350.1	Abnormal involuntary movements	neurological	rs6814407	0.023	0.069	1.023	7.382E-01	logistic	421010	800	420210	1.000	0.150
585.3	Chronic renal failure [CKD]	genitourinary	rs6814407	0.006	0.018	1.006	7.383E-01	logistic	393739	12677	381062	1.000	0.150
535.8	Other specified gastritis	digestive	rs6814407	0.012	0.037	1.012	7.385E-01	logistic	365017	2803	362214	1.000	0.150
378.2	Nystagmus and other irregular eye movements	sense organs	rs6814407	-0.046	0.138	0.955	7.390E-01	logistic	412137	209	411928	1.000	0.150
961.1	Poisoning/allergy of sulfonamides	injuries & poisonings	rs6814407	-0.026	0.079	0.974	7.390E-01	logistic	367807	634	367173	1.000	0.150

ST31; PheWAS results for the lead cis-pQTLs of the 4 proteins prioritized (ACOX1, FGF5, FURIN, and MST1).

SNP = Single Nucleotide Polymorphism; SE = Standard error HWE_p = P-value for Hardy-Weinberg equilibrium test

Phenotype	Description	Group	SNP	Beta	SE	Odds tatio	P-value	Type	n_total	n_cases	n_controls	HWE_p	allele_freq
531.4	Peptic ulcer, site unspecified	digestive	rs6814407	-0.044	0.133	0.957	7.399E-01	logistic	411565	226	411339	1.000	0.150
702.2	Seborrheic keratosis	dermatologic	rs6814407	0.028	0.084	1.028	7.404E-01	logistic	413611	539	413072	1.000	0.150
290.13	Senile dementia	mental disorders	rs6814407	-0.014	0.042	0.986	7.408E-01	logistic	407253	2280	404973	1.000	0.150
592.1	Cystitis	genitourinary	rs6814407	-0.019	0.057	0.982	7.458E-01	logistic	378131	1204	376927	1.000	0.150
510	Other diseases of lung	respiratory	rs6814407	0.017	0.052	1.017	7.466E-01	logistic	421711	1427	420284	1.000	0.150
635.2	Antepartum hemorrhage, abruptio placentae, and placenta previa	pregnancy complications	rs6814407	-0.045	0.140	0.956	7.469E-01	logistic	222797	205	222592	1.000	0.150
415.21	Primary pulmonary hypertension	circulatory system	rs6814407	0.033	0.104	1.034	7.486E-01	logistic	413189	351	412838	1.000	0.150
741.4	Joint effusions	musculoskeletal	rs6814407	-0.024	0.076	0.976	7.508E-01	logistic	415693	683	415010	1.000	0.150
297.2	Suicide or self-inflicted injury	mental disorders	rs6814407	0.034	0.108	1.034	7.540E-01	logistic	381948	326	381622	1.000	0.150
365	Glaucoma	sense organs	rs6814407	-0.007	0.023	0.993	7.566E-01	logistic	405209	7446	397763	1.000	0.150
280	Iron deficiency anemias	hematopoietic	rs6814407	0.006	0.021	1.006	7.567E-01	logistic	391534	9085	382449	1.000	0.150
427	Cardiac dysrhythmias	circulatory system	rs6814407	-0.004	0.013	0.996	7.579E-01	logistic	398369	26521	371848	1.000	0.150
740	Osteoarthrosis	musculoskeletal	rs6814407	0.003	0.010	1.003	7.588E-01	logistic	392374	46516	345858	1.000	0.150
696.42	Psoriatic arthropathy	dermatologic	rs6814407	-0.020	0.066	0.980	7.593E-01	logistic	400183	893	399290	1.000	0.150
260.6	Anorexia	endocrine/metabolic	rs6814407	0.020	0.066	1.020	7.620E-01	logistic	412968	871	412097	1.000	0.150
320	Meningitis	neurological	rs6814407	-0.035	0.115	0.966	7.630E-01	logistic	422374	303	422071	1.000	0.150
401.22	Hypertensive chronic kidney disease	circulatory system	rs6814407	0.019	0.063	1.019	7.634E-01	logistic	291632	969	290663	1.000	0.150
571.8	Liver abscess and sequelae of chronic liver disease	digestive	rs6814407	0.015	0.050	1.015	7.652E-01	logistic	401788	1514	400274	1.000	0.150
573.9	Abnormal serum enzyme levels	digestive	rs6814407	0.039	0.132	1.040	7.681E-01	logistic	400489	215	400274	1.000	0.150
200	Myeloproliferative disease	neoplasms	rs6814407	-0.015	0.051	0.985	7.697E-01	logistic	417103	1490	415613	1.000	0.150
788	Syncope and collapse	symptoms	rs6814407	0.007	0.025	1.007	7.713E-01	logistic	412052	6241	405811	1.000	0.150
281.11	Pernicious anemia	hematopoietic	rs6814407	-0.021	0.073	0.979	7.717E-01	logistic	383186	737	382449	1.000	0.150
170.2	Cancer of connective tissue	neoplasms	rs6814407	0.036	0.123	1.036	7.726E-01	logistic	423349	248	423101	1.000	0.150
367.2	Astigmatism	sense organs	rs6814407	0.010	0.034	1.010	7.752E-01	logistic	414418	3412	411006	1.000	0.150
253.7	Other disorders of neurohypophysis	endocrine/metabolic	rs6814407	0.030	0.105	1.030	7.760E-01	logistic	415594	348	415246	1.000	0.150
198.3	Secondary malignant neoplasm of digestive systems	neoplasms	rs6814407	0.011	0.038	1.011	7.766E-01	logistic	388741	2735	386006	1.000	0.150
427.1	Paroxysmal tachycardia, unspecified	circulatory system	rs6814407	-0.010	0.035	0.990	7.776E-01	logistic	374981	3133	371848	1.000	0.150
276.1	Electrolyte imbalance	endocrine/metabolic	rs6814407	-0.005	0.020	0.995	7.790E-01	logistic	406083	10511	395572	1.000	0.150
599	Other symptoms/disorders or the urinary system	genitourinary	rs6814407	0.006	0.022	1.006	7.792E-01	logistic	398281	8175	390106	1.000	0.150
720.1	Spinal stenosis of lumbar region	musculoskeletal	rs6814407	0.011	0.039	1.011	7.792E-01	logistic	411268	2598	408670	1.000	0.150
803	Fracture of upper limb	injuries & poisonings	rs6814407	-0.009	0.031	0.991	7.796E-01	logistic	400593	4215	396378	1.000	0.150
415	Pulmonary heart disease	circulatory system	rs6814407	-0.013	0.048	0.987	7.810E-01	logistic	414538	1700	412838	1.000	0.150
358	Myoneural disorders	neurological	rs6814407	-0.031	0.112	0.969	7.814E-01	logistic	419485	317	419168	1.000	0.150
500	Lung disease due to external agents	respiratory	rs6814407	-0.022	0.080	0.978	7.817E-01	logistic	391675	618	391057	1.000	0.150
550.6	Incisional hernia	digestive	rs6814407	0.025	0.091	1.026	7.824E-01	logistic	365851	457	365394	1.000	0.150
368.1	Amblyopia	sense organs	rs6814407	0.024	0.088	1.024	7.872E-01	logistic	416041	496	415545	1.000	0.150
433.3	Cerebral ischemia	circulatory system	rs6814407	-0.008	0.030	0.992	7.931E-01	logistic	407003	4318	402685	1.000	0.150
441	Vascular insufficiency of intestine	circulatory system	rs6814407	0.019	0.075	1.020	7.959E-01	logistic	406151	682	405469	1.000	0.150
272.11	Hypercholesterolemia	endocrine/metabolic	rs6814407	0.003	0.011	1.003	7.963E-01	logistic	390444	34679	355765	1.000	0.150
740.12	Osteoarthrosis, localized, secondary	musculoskeletal	rs6814407	0.024	0.094	1.024	7.981E-01	logistic	346286	428	345858	1.000	0.150
153.2	Colon cancer	neoplasms	rs6814407	0.007	0.029	1.007	8.002E-01	logistic	375260	4540	370720	1.000	0.150
480.11	Pneumococcal pneumonia	respiratory	rs6814407	0.005	0.020	1.005	8.023E-01	logistic	406463	9663	396800	1.000	0.150
384.4	Perforation of tympanic membrane	sense organs	rs6814407	0.031	0.124	1.031	8.031E-01	logistic	418963	247	418716	1.000	0.150
291	Other specified nonpsychotic and/or transient mental disorders	mental disorders	rs6814407	0.024	0.098	1.025	8.033E-01	logistic	405371	398	404973	1.000	0.150
150	Cancer of esophagus	neoplasms	rs6814407	-0.013	0.052	0.987	8.042E-01	logistic	395291	1429	393862	1.000	0.150
427.5	Arrhythmia (cardiac) NOS	circulatory system	rs6814407	-0.022	0.089	0.978	8.047E-01	logistic	372348	500	371848	1.000	0.150
819	Skull and face fracture and other intercranial injury	injuries & poisonings	rs6814407	0.021	0.087	1.022	8.058E-01	logistic	422666	501	422165	1.000	0.150
531	Peptic ulcer (excl. esophageal)	digestive	rs6814407	-0.008	0.031	0.992	8.059E-01	logistic	415329	3990	411339	1.000	0.150
289.4	Lymphadenitis	hematopoietic	rs6814407	-0.012	0.048	0.988	8.059E-01	logistic	410013	1704	408309	1.000	0.150
618.1	Prolapse of vaginal walls	genitourinary	rs6814407	0.008	0.032	1.008	8.082E-01	logistic	217426	3971	213455	1.000	0.150
411.3	Angina pectoris	circulatory system	rs6814407	0.004	0.016	1.004	8.090E-01	logistic	387093	16543	370550	1.000	0.150
296	Mood disorders	mental disorders	rs6814407	-0.003	0.012	0.997	8.098E-01	logistic	409186	27564	381622	1.000	0.150
690.1	Seborrheic dermatitis	dermatologic	rs6814407	0.019	0.079	1.019	8.099E-01	logistic	405818	612	405206	1.000	0.150
281.1	Megaloblastic anemia	hematopoietic	rs6814407	0.013	0.055	1.013	8.106E-01	logistic	383709	1260	382449	1.000	0.150
556	Ulceration of the lower GI tract	digestive	rs6814407	-0.024	0.100	0.976	8.112E-01	logistic	334879	394	334485	1.000	0.150
614.3	Pelvic inflammatory disease (PID)	genitourinary	rs6814407	-0.030	0.130	0.970	8.156E-01	logistic	217675	237	217438	1.000	0.150
531.1	Hemorrhage from gastrointestinal ulcer	digestive	rs6814407	-0.019	0.083	0.981	8.163E-01	logistic	411913	574	411339	1.000	0.150
737	Curvature of spine	musculoskeletal	rs6814407	-0.015	0.066	0.985	8.169E-01	logistic	403550	896	402654	1.000	0.150
415.2	Chronic pulmonary heart disease	circulatory system	rs6814407	-0.011	0.049	0.989	8.206E-01	logistic	414491	1653	412838	1.000	0.150
337	Disorders of the autonomic nervous system	neurological	rs6814407	0.024	0.105	1.024	8.214E-01	logistic	399396	349	399047	1.000	0.150
574.3	Cholecystitis without cholelithiasis	digestive	rs6814407	0.013	0.057	1.013	8.237E-01	logistic	396244	1197	395047	1.000	0.150
296.22	Major depressive disorder	mental disorders	rs6814407	-0.004	0.016	0.996	8.243E-01	logistic	396820	15198	381622	1.000	0.150
597	Other disorders of urethra and urinary tract	genitourinary	rs6814407	-0.010	0.044	0.990	8.269E-01	logistic	403600	2046	401554	1.000	0.150
379	Other disorders of eye	sense organs	rs6814407	0.013	0.061	1.013	8.271E-01	logistic	412963	1035	411928	1.000	0.150
270.3	Disorders of plasma protein metabolism	endocrine/metabolic	rs6814407	-0.016	0.072	0.984	8.275E-01	logistic	422449	747	421702	1.000	0.150
614.5	Inflammatory disease of cervix, vagina, and vulva	genitourinary	rs6814407	-0.019	0.086	0.981	8.276E-01	logistic	217969	531	217438	1.000	0.150
364.5	Corneal dystrophy	sense organs	rs6814407	0.022	0.099	1.022	8.278E-01	logistic	398152	389	397763	1.000	0.150
442.11	Abdominal aortic aneurysm	circulatory system	rs6814407	0.010	0.048	1.011	8.289E-01	logistic	407127	1658	405469	1.000	0.150
634	Miscarriage; stillbirth	pregnancy complications	rs6814407	-0.016	0.075	0.984	8.292E-01	logistic	223299	707	222592	1.000	0.150
706	Diseases of sebaceous glands	dermatologic	rs6814407	-0.011	0.052	0.989	8.293E-01	logistic	413434	1435	411999	1.000	0.150
750.1	Upper gastrointestinal congenital anomalies	congenital anomalies	rs6814407	0.029	0.134	1.029	8.300E-01	logistic	420464	211	420253	1.000	0.150

ST31; PheWAS results for the lead cis-pQTLs of the 4 proteins prioritized (ACOX1, FGF5, FURIN, and MST1).

SNP = Single Nucleotide Polymorphism; SE = Standard error HWE_p = P-value for Hardy-Weinberg equilibrium test

Phenotype	Description	Group	SNP	Beta	SE	Odds tatio	P-value	Type	n_total	n_cases	n_controls	HWE_p	allele_freq
703.1	Ingrowing nail	dermatologic	rs6814407	-0.029	0.139	0.971	8.349E-01	logistic	421519	205	421314	1.000	0.150
411.9	Other acute and subacute forms of ischemic heart diseasc	circulatory system	rs6814407	-0.011	0.051	0.989	8.352E-01	logistic	372042	1492	370550	1.000	0.150
555.1	Regional enteritis	digestive	rs6814407	0.010	0.046	1.010	8.356E-01	logistic	336302	1817	334485	1.000	0.150
480.1	Bacterial pneumonia	respiratory	rs6814407	0.004	0.020	1.004	8.371E-01	logistic	407095	10295	396800	1.000	0.150
523.3	Periodontitis (acute or chronic)	digestive	rs6814407	-0.027	0.130	0.974	8.372E-01	logistic	409770	234	409536	1.000	0.150
574	Cholelithiasis and cholecystitis	digestive	rs6814407	0.004	0.019	1.004	8.374E-01	logistic	406113	11066	395047	1.000	0.150
625	Pain and other symptoms associated with female genital organs	genitourinary	rs6814407	-0.024	0.117	0.976	8.375E-01	logistic	218575	291	218284	1.000	0.150
733	Other disorders of bone and cartilage	musculoskeletal	rs6814407	0.013	0.065	1.013	8.379E-01	logistic	399585	913	398672	1.000	0.150
528	Diseases of the oral soft tissues, excluding lesions specific for gingiva and tongu	digestive	rs6814407	0.019	0.091	1.019	8.392E-01	logistic	418169	459	417710	1.000	0.150
628	Ovarian cyst	genitourinary	rs6814407	0.011	0.057	1.012	8.406E-01	logistic	193944	1197	192747	1.000	0.150
433.12	Cerebral atherosclerosis	circulatory system	rs6814407	0.021	0.105	1.021	8.421E-01	logistic	403029	344	402685	1.000	0.150
782.3	Edema	symptoms	rs6814407	0.008	0.040	1.008	8.423E-01	logistic	419509	2451	417058	1.000	0.150
555.21	Ulcerative colitis (chronic)	digestive	rs6814407	-0.020	0.101	0.980	8.431E-01	logistic	334866	381	334485	1.000	0.150
727	Other disorders of synovium, tendon, and bursa	musculoskeletal	rs6814407	0.008	0.038	1.008	8.434E-01	logistic	391842	2668	389174	1.000	0.150
198.7	Secondary malignant neoplasm of skin	neoplasms	rs6814407	-0.024	0.123	0.976	8.437E-01	logistic	386268	262	386006	1.000	0.150
218	Benign neoplasm of uterus	neoplasms	rs6814407	-0.007	0.037	0.993	8.459E-01	logistic	209861	2866	206995	1.000	0.150
185	Cancer of prostate	neoplasms	rs6814407	-0.004	0.020	0.996	8.467E-01	logistic	167540	11079	156461	1.000	0.150
701	Other hypertrophic and atrophic conditions of skin	dermatologic	rs6814407	-0.011	0.058	0.989	8.470E-01	logistic	416347	1161	415186	1.000	0.150
573.3	Hepatomegaly	digestive	rs6814407	0.022	0.117	1.023	8.490E-01	logistic	400551	277	400274	1.000	0.150
110	Dermatophytosis / Dermatomycosis	infectious diseases	rs6814407	-0.025	0.129	0.976	8.493E-01	logistic	418617	237	418380	1.000	0.150
270.32	Paraproteinemia	endocrine/metabolic	rs6814407	0.015	0.079	1.015	8.500E-01	logistic	422319	617	421702	1.000	0.150
523	Gingival and periodontal diseases	digestive	rs6814407	-0.019	0.100	0.981	8.509E-01	logistic	409931	395	409536	1.000	0.150
371.3	Inflammation of eyelids	sense organs	rs6814407	-0.014	0.076	0.986	8.516E-01	logistic	411465	672	410793	1.000	0.150
296.2	Depression	mental disorders	rs6814407	-0.002	0.012	0.998	8.536E-01	logistic	408593	26971	381622	1.000	0.150
745	Pain in joint	musculoskeletal	rs6814407	0.006	0.033	1.006	8.538E-01	logistic	414363	3501	410862	1.000	0.150
214.1	Lipoma of skin and subcutaneous tissue	neoplasms	rs6814407	0.013	0.072	1.013	8.542E-01	logistic	414958	731	414227	1.000	0.150
371	Inflammation of the eye	sense organs	rs6814407	-0.011	0.062	0.989	8.546E-01	logistic	411823	1030	410793	1.000	0.150
401.2	Hypertensive heart and/or renal diseasc	circulatory system	rs6814407	0.010	0.058	1.010	8.589E-01	logistic	291807	1144	290663	1.000	0.150
480	Pneumonia	respiratory	rs6814407	-0.003	0.016	0.997	8.590E-01	logistic	412462	15662	396800	1.000	0.150
300.9	Posttraumatic stress disorder	mental disorders	rs6814407	-0.022	0.123	0.978	8.594E-01	logistic	381881	259	381622	1.000	0.150
592	Cystitis and urethritis	genitourinary	rs6814407	-0.010	0.056	0.990	8.603E-01	logistic	378176	1249	376927	1.000	0.150
743	Osteoporosis, osteopenia and pathological fracture	musculoskeletal	rs6814407	-0.010	0.058	0.990	8.627E-01	logistic	422387	1153	421234	1.000	0.150
296.1	Bipolar	mental disorders	rs6814407	-0.010	0.058	0.990	8.638E-01	logistic	382786	1164	381622	1.000	0.150
430	Intracranial hemorrhage	circulatory system	rs6814407	0.008	0.045	1.008	8.652E-01	logistic	404595	1910	402685	1.000	0.150
274.2	Crystal arthropathies	endocrine/metabolic	rs6814407	0.021	0.125	1.021	8.672E-01	logistic	416033	243	415790	1.000	0.150
619.3	Noninflammatory disorders of cervix	genitourinary	rs6814407	0.018	0.106	1.018	8.688E-01	logistic	217308	342	216966	1.000	0.150
706.2	Sebaceous cyst	dermatologic	rs6814407	-0.009	0.053	0.991	8.696E-01	logistic	413393	1394	411999	1.000	0.150
279.11	Deficiency of humoral immunity	endocrine/metabolic	rs6814407	-0.021	0.127	0.980	8.713E-01	logistic	423118	242	422876	1.000	0.150
433.5	Cerebral aneurysm	circulatory system	rs6814407	0.015	0.093	1.015	8.717E-01	logistic	403130	445	402685	1.000	0.150
281.13	Folate-deficiency anemia	hematopoietic	rs6814407	-0.016	0.102	0.984	8.741E-01	logistic	382829	380	382449	1.000	0.150
611	Abnormal findings on mammogram or breast exam	genitourinary	rs6814407	-0.019	0.122	0.981	8.753E-01	logistic	415016	264	414752	1.000	0.150
611.3	Lump or mass in breast	genitourinary	rs6814407	-0.019	0.122	0.981	8.753E-01	logistic	415016	264	414752	1.000	0.150
800.3	Fracture of tibia and fibula	injuries & poisonings	rs6814407	-0.013	0.082	0.987	8.784E-01	logistic	396955	577	396378	1.000	0.150
227.3	Benign neoplasm of pituitary gland and craniopharyngeal duct (pouch	neoplasms	rs6814407	-0.016	0.104	0.984	8.786E-01	logistic	421248	361	420887	1.000	0.150
789	Nausea and vomiting	symptoms	rs6814407	0.004	0.024	1.004	8.803E-01	logistic	407825	6923	400902	1.000	0.150
720	Spinal stenosis	musculoskeletal	rs6814407	-0.005	0.031	0.995	8.817E-01	logistic	412805	4135	408670	1.000	0.150
558	Noninfectious gastroenteritis	digestive	rs6814407	0.004	0.030	1.004	8.861E-01	logistic	338928	4443	334485	1.000	0.150
530.2	Esophageal bleeding (varices/hemorrhage)	digestive	rs6814407	-0.014	0.097	0.986	8.870E-01	logistic	357206	418	356788	1.000	0.150
512.8	Cough	respiratory	rs6814407	0.007	0.049	1.007	8.888E-01	logistic	406184	1585	404599	1.000	0.150
442.1	Aortic aneurysm	circulatory system	rs6814407	-0.006	0.040	0.994	8.892E-01	logistic	407951	2482	405469	1.000	0.150
528.1	Stomatitis and mucositis	digestive	rs6814407	-0.018	0.129	0.982	8.910E-01	logistic	417947	237	417710	1.000	0.150
8.5	Bacterial enteritis	infectious diseases	rs6814407	-0.007	0.056	0.993	8.937E-01	logistic	418567	1251	417316	1.000	0.150
426.91	Cardiac pacemaker in situ	circulatory system	rs6814407	-0.004	0.028	0.996	8.963E-01	logistic	376826	4978	371848	1.000	0.150
275.1	Disorders of iron metabolism	hematopoietic	rs6814407	0.008	0.060	1.008	9.000E-01	logistic	417274	1089	416185	1.000	0.150
252.1	Hyperparathyroidism	endocrine/metabolic	rs6814407	0.008	0.061	1.008	9.001E-01	logistic	416271	1025	415246	1.000	0.150
187.2	Malignant neoplasm of testis	neoplasms	rs6814407	-0.004	0.030	0.996	9.007E-01	logistic	184014	4620	179394	1.000	0.150
276.11	Hyperosmolality and/or hypernatremia	endocrine/metabolic	rs6814407	0.009	0.073	1.009	9.009E-01	logistic	396286	714	395572	1.000	0.150
736	Other acquired deformities of limbs	musculoskeletal	rs6814407	-0.008	0.064	0.992	9.012E-01	logistic	403605	951	402654	1.000	0.150
286.7	Other and unspecified coagulation defects	hematopoietic	rs6814407	-0.015	0.121	0.985	9.028E-01	logistic	417818	265	417553	1.000	0.150
386.3	Labyrinthitis	sense organs	rs6814407	0.012	0.105	1.013	9.056E-01	logistic	410012	347	409665	1.000	0.150
427.8	Sinoatrial node dysfunction (Bradycardia)	circulatory system	rs6814407	-0.010	0.082	0.990	9.057E-01	logistic	372427	579	371848	1.000	0.150
151	Cancer of stomach	neoplasms	rs6814407	-0.007	0.064	0.993	9.090E-01	logistic	394829	967	393862	1.000	0.150
280.2	Iron deficiency anemia secondary to blood loss (chronic	hematopoietic	rs6814407	0.013	0.113	1.013	9.093E-01	logistic	382749	300	382449	1.000	0.150
615	Endometriosis	genitourinary	rs6814407	-0.006	0.057	0.994	9.112E-01	logistic	218669	1231	217438	1.000	0.150
378.5	Paralytic strabismus	sense organs	rs6814407	0.013	0.119	1.013	9.114E-01	logistic	412201	273	411928	1.000	0.150
368.9	Subjective visual disturbances	sense organs	rs6814407	-0.010	0.088	0.990	9.117E-01	logistic	416057	512	415545	1.000	0.150
426.3	Bundle branch block	circulatory system	rs6814407	0.004	0.033	1.004	9.121E-01	logistic	375377	3529	371848	1.000	0.150
281	Other deficiency anemia	hematopoietic	rs6814407	-0.006	0.054	0.994	9.124E-01	logistic	383766	1317	382449	1.000	0.150
275	Disorders of mineral metabolism	endocrine/metabolic	rs6814407	-0.003	0.029	0.997	9.143E-01	logistic	420958	4773	416185	1.000	0.150
571.6	Primary biliary cirrhosis	digestive	rs6814407	0.012	0.111	1.012	9.150E-01	logistic	400586	312	400274	1.000	0.150
728	Disorders of muscle, ligament, and fascia	musculoskeletal	rs6814407	-0.013	0.123	0.987	9.159E-01	logistic	389432	258	389174	1.000	0.150

ST31; PheWAS results for the lead cis-pQTLs of the 4 proteins prioritized (ACOX1, FGF5, FURIN, and MST1).

SNP = Single Nucleotide Polymorphism; SE = Standard error HWE_p = P-value for Hardy-Weinberg equilibrium test

Phenotype	Description	Group	SNP	Beta	SE	Odds tatio	P-value	Type	n_total	n_cases	n_controls	HWE_p	allele_freq
735	Acquired foot deformities	musculoskeletal	rs6814407	0.003	0.026	1.003	9.161E-01	logistic	408734	6080	402654	1.000	0.150
196	Radiotherapy	neoplasms	rs6814407	0.005	0.050	1.005	9.173E-01	logistic	387589	1583	386006	1.000	0.150
427.7	Tachycardia NOS	circulatory system	rs6814407	0.005	0.045	1.005	9.178E-01	logistic	373741	1893	371848	1.000	0.150
622.2	Mucous polyp of cervix	genitourinary	rs6814407	0.013	0.127	1.013	9.182E-01	logistic	213810	238	213572	1.000	0.150
622	Polyp of female genital organs	genitourinary	rs6814407	-0.004	0.039	0.996	9.194E-01	logistic	216102	2530	213572	1.000	0.150
528.11	Stomatitis and mucositis (ulcerative)	digestive	rs6814407	-0.014	0.137	0.986	9.197E-01	logistic	417919	209	417710	1.000	0.150
280.1	Iron deficiency anemias, unspecified or not due to blood loss	hematopoietic	rs6814407	0.002	0.021	1.002	9.204E-01	logistic	391238	8789	382449	1.000	0.150
740.2	Osteoarthrosis, generalized	musculoskeletal	rs6814407	-0.003	0.029	0.997	9.209E-01	logistic	350458	4600	345858	1.000	0.150
763	Thoracic or lumbosacral neuritis or radiculitis, unspecified	symptoms	rs6814407	-0.011	0.118	0.989	9.248E-01	logistic	418269	283	417986	1.000	0.150
618	Genital prolapse	genitourinary	rs6814407	-0.002	0.025	0.998	9.253E-01	logistic	219680	6225	213455	1.000	0.150
803.1	Fracture of humerus	injuries & poisonings	rs6814407	0.006	0.060	1.006	9.263E-01	logistic	397468	1090	396378	1.000	0.150
707	Chronic ulcer of skin	dermatologic	rs6814407	-0.003	0.029	0.997	9.265E-01	logistic	420904	4774	416130	1.000	0.150
752	Nervous system congenital anomalies	congenital anomalies	rs6814407	-0.008	0.093	0.993	9.355E-01	logistic	422992	453	422539	1.000	0.150
352.2	Facial nerve disorders [CN7]	neurological	rs6814407	-0.006	0.076	0.994	9.361E-01	logistic	403092	677	402415	1.000	0.150
187.1	Malignant neoplasm of unspecified male genital organ	neoplasms	rs6814407	0.002	0.030	1.002	9.368E-01	logistic	183955	4561	179394	1.000	0.150
252	Disorders of parathyroid gland	endocrine/metabolic	rs6814407	-0.004	0.056	0.996	9.371E-01	logistic	416485	1239	415246	1.000	0.150
189.1	Cancer of kidney and renal pelvis	neoplasms	rs6814407	0.004	0.057	1.004	9.371E-01	logistic	418392	1205	417187	1.000	0.150
276.6	Fluid overload	endocrine/metabolic	rs6814407	-0.004	0.048	0.996	9.392E-01	logistic	397239	1667	395572	1.000	0.150
727.6	Rupture of tendon, nontraumatic	musculoskeletal	rs6814407	-0.004	0.054	0.996	9.410E-01	logistic	390517	1343	389174	1.000	0.150
575.2	Obstruction of bile duct	digestive	rs6814407	-0.004	0.062	0.996	9.418E-01	logistic	396076	1029	395047	1.000	0.150
286.1	Congenital coagulation defects	hematopoietic	rs6814407	0.007	0.095	1.007	9.452E-01	logistic	417976	423	417553	1.000	0.150
726.3	Bursitis	musculoskeletal	rs6814407	0.009	0.137	1.009	9.462E-01	logistic	389380	206	389174	1.000	0.150
289.3	Personal history of diseases of blood and blood-forming organs	hematopoietic	rs6814407	-0.008	0.115	0.992	9.475E-01	logistic	408604	295	408309	1.000	0.150
565.1	Anal and rectal polyp	digestive	rs6814407	0.002	0.038	1.002	9.544E-01	logistic	386554	2715	383839	1.000	0.150
751.2	Congenital anomalies of urinary system	congenital anomalies	rs6814407	0.004	0.072	1.004	9.545E-01	logistic	421003	750	420253	1.000	0.150
947	Urticaria	dermatologic	rs6814407	0.008	0.136	1.008	9.553E-01	logistic	415196	209	414987	1.000	0.150
426.9	Cardiac pacemaker/device in situ	circulatory system	rs6814407	-0.002	0.028	0.998	9.553E-01	logistic	376950	5102	371848	1.000	0.150
761	Cervicalgia	symptoms	rs6814407	-0.004	0.072	0.996	9.555E-01	logistic	422454	744	421710	1.000	0.150
426.32	Left bundle branch block	circulatory system	rs6814407	-0.002	0.036	0.998	9.562E-01	logistic	374914	3066	371848	1.000	0.150
473	Diseases of the larynx and vocal cords	respiratory	rs6814407	0.003	0.055	1.003	9.607E-01	logistic	397819	1302	396517	1.000	0.150
506	Empyema and pneumothorax	respiratory	rs6814407	-0.003	0.058	0.997	9.643E-01	logistic	392231	1174	391057	1.000	0.150
737.3	Kyphoscoliosis and scoliosis	musculoskeletal	rs6814407	0.003	0.070	1.003	9.655E-01	logistic	403443	789	402654	1.000	0.150
572	Ascites (non malignant)	digestive	rs6814407	0.002	0.043	1.002	9.657E-01	logistic	402409	2135	400274	1.000	0.150
560.1	Paralytic ileus	digestive	rs6814407	-0.004	0.099	0.996	9.667E-01	logistic	334880	395	334485	1.000	0.150
292.3	Memory loss	mental disorders	rs6814407	-0.004	0.088	0.996	9.676E-01	logistic	405478	505	404973	1.000	0.150
476	Allergic rhinitis	respiratory	rs6814407	0.002	0.060	1.002	9.677E-01	logistic	397580	1063	396517	1.000	0.150
465.2	Acute pharyngitis	respiratory	rs6814407	0.005	0.125	1.005	9.678E-01	logistic	419940	248	419692	1.000	0.150
286	Coagulation defects	hematopoietic	rs6814407	-0.002	0.065	0.998	9.709E-01	logistic	418481	928	417553	1.000	0.150
575.7	Other disorders of gallbladder	digestive	rs6814407	-0.002	0.080	0.998	9.788E-01	logistic	395657	610	395047	1.000	0.150
740.1	Osteoarthritis; localized	musculoskeletal	rs6814407	0.000	0.012	1.000	9.802E-01	logistic	374099	28241	345858	1.000	0.150
530	Diseases of esophagus	digestive	rs6814407	0.000	0.012	1.000	9.808E-01	logistic	388733	31945	356788	1.000	0.150
443.9	Peripheral vascular disease, unspecified	circulatory system	rs6814407	0.001	0.033	1.001	9.818E-01	logistic	409046	3577	405469	1.000	0.150
345.1	Epilepsy	neurological	rs6814407	-0.002	0.084	0.998	9.827E-01	logistic	399597	550	399047	1.000	0.150
442.8	Aneurysm of other specified artery	circulatory system	rs6814407	0.002	0.122	1.002	9.853E-01	logistic	405731	262	405469	1.000	0.150
977	Personal history of allergy to medicinal agents	injuries & poisonings	rs6814407	0.000	0.022	1.000	9.874E-01	logistic	375378	8205	367173	1.000	0.150
291.8	Alteration of consciousness	mental disorders	rs6814407	-0.002	0.102	0.998	9.879E-01	logistic	405343	370	404973	1.000	0.150
740.11	Osteoarthrosis, localized, primary	musculoskeletal	rs6814407	0.000	0.027	1.000	9.889E-01	logistic	351108	5250	345858	1.000	0.150
690	Erythematosquamous dermatosis	dermatologic	rs6814407	0.001	0.079	1.001	9.898E-01	logistic	405831	625	405206	1.000	0.150
574.1	Cholelithiasis	digestive	rs6814407	0.000	0.021	1.000	9.924E-01	logistic	404312	9265	395047	1.000	0.150
527	Diseases of the salivary glands	digestive	rs6814407	-0.001	0.132	0.999	9.934E-01	logistic	417933	223	417710	1.000	0.150
530.1	Esophagitis, GERD and related diseases	digestive	rs6814407	0.000	0.012	1.000	9.944E-01	logistic	385890	29102	356788	1.000	0.150
594.8	Renal colic	genitourinary	rs6814407	0.001	0.084	1.001	9.946E-01	logistic	412927	551	412376	1.000	0.150
189.11	Malignant neoplasm of kidney, except pelvis	neoplasms	rs6814407	0.000	0.058	1.000	9.957E-01	logistic	418324	1137	417187	1.000	0.150
751	Genitourinary congenital anomalies	congenital anomalies	rs6814407	0.000	0.067	1.000	9.958E-01	logistic	421129	876	420253	1.000	0.150
965	Poisoning by analgesics, antipyretics, and antirheumatics	injuries & poisonings	rs6814407	0.000	0.029	1.000	9.978E-01	logistic	371918	4745	367173	1.000	0.150
250.11	Type 1 diabetes with ketoacidosis	endocrine/metabolic	rs6814407	0.000	0.125	1.000	9.979E-01	logistic	386885	248	386637	1.000	0.150
279.1	Immunity deficiency	endocrine/metabolic	rs6814407	0.000	0.109	1.000	9.996E-01	logistic	423199	323	422876	1.000	0.150
149	Cancer of larynx, pharynx, nasal cavities	neoplasms	rs10852766	-0.185	0.053	0.831	5.110E-04	logistic	421191	849	420342	1.000	0.337
198.1	Secondary malignancy of lymph nodes	neoplasms	rs10852766	-0.054	0.017	0.948	1.602E-03	logistic	394015	8009	386006	0.998	0.337
965.1	Opiates and related narcotics causing adverse effects in therapeutic use	injuries & poisonings	rs10852766	0.095	0.031	1.099	2.276E-03	logistic	369447	2274	367173	0.998	0.336
278	Overweight, obesity and other hyperalimentation	endocrine/metabolic	rs10852766	0.036	0.012	1.037	3.008E-03	logistic	405726	15173	390553	1.000	0.337
278.1	Obesity	endocrine/metabolic	rs10852766	0.036	0.012	1.037	3.502E-03	logistic	405667	15114	390553	1.000	0.337
578.8	Hemorrhage of rectum and anus	digestive	rs10852766	0.089	0.031	1.093	3.750E-03	logistic	385630	2327	383303	0.999	0.336
198	Secondary malignant neoplasm	neoplasms	rs10852766	-0.033	0.012	0.968	5.213E-03	logistic	403287	17281	386006	0.999	0.337
350	Abnormal movement	neurological	rs10852766	0.115	0.042	1.122	6.807E-03	logistic	421410	1200	420210	1.000	0.337
502	Postinflammatory pulmonary fibrosis	respiratory	rs10852766	0.088	0.033	1.092	7.673E-03	logistic	393073	2016	391057	1.000	0.337
81	Infection/inflammation of internal prosthetic device; implant; and graft	infectious diseases	rs10852766	0.089	0.034	1.093	8.308E-03	logistic	414783	1939	412844	1.000	0.337
149.4	Cancer of larynx	neoplasms	rs10852766	-0.248	0.094	0.780	8.579E-03	logistic	420618	276	420342	1.000	0.337
696.4	Psoriasis	dermatologic	rs10852766	0.070	0.028	1.073	1.083E-02	logistic	402177	2887	399290	1.000	0.337
295.1	Schizophrenia	mental disorders	rs10852766	-0.151	0.059	0.860	1.100E-02	logistic	382292	670	381622	0.999	0.337
473	Diseases of the larynx and vocal cords	respiratory	rs10852766	-0.107	0.042	0.898	1.102E-02	logistic	397819	1302	396517	1.000	0.336

ST31; PheWAS results for the lead cis-pQTLs of the 4 proteins prioritized (ACOX1, FGF5, FURIN, and MST1).

SNP = Single Nucleotide Polymorphism; SE = Standard error HWE_p = P-value for Hardy-Weinberg equilibrium test

Phenotype	Description	Group	SNP	Beta	SE	Odds tatio	P-value	Type	n_total	n_cases	n_controls	HWE_p	allele_freq
788	Syncope and collapse	symptoms	rs10852766	0.047	0.019	1.048	1.334E-02	logistic	412052	6241	405811	1.000	0.337
618.2	Uterine/Uterovaginal prolapse	genitourinary	rs10852766	0.118	0.050	1.126	1.720E-02	logistic	214329	874	213455	0.998	0.336
696	Psoriasis and related disorders	dermatologic	rs10852766	0.065	0.027	1.068	1.720E-02	logistic	402206	2916	399290	1.000	0.337
696.41	Psoriasis vulgaris	dermatologic	rs10852766	0.075	0.032	1.078	1.943E-02	logistic	401395	2105	399290	1.000	0.337
301	Personality disorders	mental disorders	rs10852766	0.183	0.078	1.200	1.970E-02	logistic	381968	346	381622	0.999	0.337
295	Schizophrenia and other psychotic disorders	mental disorders	rs10852766	-0.122	0.053	0.885	2.042E-02	logistic	382460	838	381622	0.999	0.337
216	Benign neoplasm of skin	neoplasms	rs10852766	0.101	0.044	1.107	2.138E-02	logistic	415074	1116	413958	0.999	0.337
559	Ileostomy status	digestive	rs10852766	-0.082	0.036	0.921	2.143E-02	logistic	336295	1810	334485	0.997	0.336
738	Other acquired musculoskeletal deformity	musculoskeletal	rs10852766	0.093	0.040	1.097	2.196E-02	logistic	403982	1328	402654	0.999	0.337
204.12	Lymphoid leukemia, chronic	neoplasms	rs10852766	0.117	0.051	1.124	2.295E-02	logistic	416428	815	415613	1.000	0.337
783	Fever of unknown origin	symptoms	rs10852766	0.069	0.030	1.071	2.332E-02	logistic	418176	2397	415779	1.000	0.337
276.41	Acidosis	endocrine/metabolic	rs10852766	0.079	0.035	1.082	2.391E-02	logistic	397376	1804	395572	0.999	0.337
276.4	Acid-base balance disorder	endocrine/metabolic	rs10852766	0.073	0.032	1.075	2.403E-02	logistic	397692	2120	395572	0.999	0.337
331.1	Hydrocephalus	neurological	rs10852766	0.132	0.059	1.141	2.456E-02	logistic	399668	621	399047	1.000	0.337
198.4	Secondary malignant neoplasm of liver	neoplasms	rs10852766	-0.046	0.021	0.955	2.512E-02	logistic	391482	5476	386006	0.999	0.337
389.4	Tinnitus	sense organs	rs10852766	0.150	0.067	1.162	2.518E-02	logistic	408770	473	408297	1.000	0.337
565	Anal and rectal conditions	digestive	rs10852766	0.038	0.017	1.039	2.631E-02	logistic	391353	7514	383839	0.998	0.336
454.11	Varicose veins of lower extremity, symptomtic	circulatory system	rs10852766	0.130	0.060	1.139	3.074E-02	logistic	381560	594	380966	0.999	0.336
149.1	Cancer of oropharynx	neoplasms	rs10852766	-0.170	0.079	0.844	3.153E-02	logistic	420725	383	420342	1.000	0.337
327	Sleep disorders	neurological	rs10852766	0.132	0.062	1.141	3.227E-02	logistic	413949	565	413384	1.000	0.337
217.1	Nevus, non-neoplastic	neoplasms	rs10852766	0.187	0.088	1.206	3.291E-02	logistic	414233	275	413958	0.999	0.337
151	Cancer of stomach	neoplasms	rs10852766	0.101	0.047	1.106	3.298E-02	logistic	394829	967	393862	0.999	0.337
272.1	Hyperlipidemia	endocrine/metabolic	rs10852766	-0.017	0.008	0.983	3.371E-02	logistic	394381	38616	355765	1.000	0.337
560.3	Peritoneal or intestinal adhesions	digestive	rs10852766	0.108	0.051	1.114	3.375E-02	logistic	335317	832	334485	0.997	0.337
411.1	Unstable angina (intermediate coronary syndrome	circulatory system	rs10852766	-0.053	0.025	0.949	3.483E-02	logistic	374269	3719	370550	0.999	0.336
519.8	Other diseases of respiratory system, NEC	respiratory	rs10852766	0.034	0.016	1.035	3.544E-02	logistic	411807	8677	403130	0.999	0.337
571.81	Portal hypertension	digestive	rs10852766	0.105	0.050	1.111	3.557E-02	logistic	401135	861	400274	0.999	0.337
594	Urinary calculus	genitourinary	rs10852766	0.041	0.020	1.042	3.602E-02	logistic	418195	5819	412376	0.999	0.337
262	Mineral deficiency NEC	endocrine/metabolic	rs10852766	0.165	0.079	1.179	3.629E-02	logistic	412440	343	412097	1.000	0.336
433.12	Cerebral atherosclerosis	circulatory system	rs10852766	-0.174	0.083	0.840	3.646E-02	logistic	403029	344	402685	0.999	0.337
738.4	Acquired spondylolisthesis	musculoskeletal	rs10852766	0.089	0.043	1.093	3.660E-02	logistic	403846	1192	402654	0.999	0.337
272	Disorders of lipoid metabolism	endocrine/metabolic	rs10852766	-0.017	0.008	0.983	3.895E-02	logistic	394625	38860	355765	1.000	0.337
965	Poisoning by analgesics, antipyretics, and antirheumatics	injuries & poisonings	rs10852766	0.044	0.022	1.045	4.059E-02	logistic	371918	4745	367173	0.998	0.336
960	Poisoning by antibiotics	injuries & poisonings	rs10852766	0.021	0.010	1.021	4.161E-02	logistic	389532	22359	367173	0.999	0.337
977	Personal history of allergy to medicinal agents	injuries & poisonings	rs10852766	0.034	0.017	1.034	4.206E-02	logistic	375378	8205	367173	0.998	0.336
386.3	Labyrinthitis	sense organs	rs10852766	-0.168	0.083	0.846	4.264E-02	logistic	410012	347	409665	1.000	0.337
184.1	Malignant neoplasm of ovary and other uterine adnexa	neoplasms	rs10852766	-0.058	0.029	0.943	4.315E-02	logistic	207626	2789	204837	0.996	0.336
395.6	Heart valve replaced	circulatory system	rs10852766	0.060	0.030	1.062	4.353E-02	logistic	403924	2510	401414	1.000	0.337
193	Thyroid cancer	neoplasms	rs10852766	-0.152	0.076	0.859	4.363E-02	logistic	421300	413	420887	1.000	0.337
198.3	Secondary malignant neoplasm of digestive systems	neoplasms	rs10852766	-0.058	0.029	0.944	4.502E-02	logistic	388741	2735	386006	0.999	0.337
184.11	Malignant neoplasm of ovary	neoplasms	rs10852766	-0.058	0.029	0.943	4.546E-02	logistic	207552	2715	204837	0.996	0.336
250.23	Type 2 diabetes with ophthalmic manifestations	endocrine/metabolic	rs10852766	0.067	0.034	1.069	4.555E-02	logistic	388590	1953	386637	0.999	0.337
427.11	Paroxysmal supraventricular tachycardia	circulatory system	rs10852766	-0.066	0.033	0.936	4.582E-02	logistic	373959	2111	371848	1.000	0.337
427.1	Paroxysmal tachycardia, unspecified	circulatory system	rs10852766	-0.054	0.027	0.947	4.613E-02	logistic	374981	3133	371848	1.000	0.337
361	Retinal detachments and defects	sense organs	rs10852766	0.129	0.065	1.138	4.637E-02	logistic	398276	513	397763	1.000	0.337
350.3	Lack of coordination	neurological	rs10852766	0.171	0.086	1.186	4.653E-02	logistic	420499	289	420210	1.000	0.337
578	Gastrointestinal hemorrhage	digestive	rs10852766	0.031	0.016	1.031	4.718E-02	logistic	392729	9426	383303	0.999	0.336
622	Polyp of female genital organs	genitourinary	rs10852766	-0.060	0.030	0.942	4.773E-02	logistic	216102	2530	213572	0.997	0.336
465.2	Acute pharyngitis	respiratory	rs10852766	-0.194	0.098	0.824	4.835E-02	logistic	419940	248	419692	1.000	0.337
79.9	Viremia, NOS	infectious diseases	rs10852766	-0.124	0.063	0.884	4.874E-02	logistic	415798	594	415204	1.000	0.337
433.21	Cerebral artery occlusion, with cerebral infarction	circulatory system	rs10852766	-0.048	0.025	0.953	5.013E-02	logistic	406495	3810	402685	0.999	0.336
790	Nonspecific findings on examination of blood	symptoms	rs10852766	0.178	0.091	1.195	5.022E-02	logistic	422095	255	421840	1.000	0.337
366	Cataract	sense organs	rs10852766	0.017	0.009	1.017	5.062E-02	logistic	406937	35259	371678	0.999	0.337
260.6	Anorexia	endocrine/metabolic	rs10852766	0.097	0.050	1.101	5.307E-02	logistic	412968	871	412097	1.000	0.336
79	Viral infection	infectious diseases	rs10852766	-0.092	0.048	0.912	5.418E-02	logistic	416221	1017	415204	1.000	0.337
519	Other diseases of respiratory system, not elsewhere classified	respiratory	rs10852766	0.031	0.016	1.031	5.438E-02	logistic	411962	8832	403130	0.999	0.337
802	Fracture of pelvis	injuries & poisonings	rs10852766	-0.095	0.049	0.910	5.491E-02	logistic	397331	953	396378	1.000	0.336
395.2	Nonrheumatic aortic valve disorders	circulatory system	rs10852766	0.041	0.021	1.042	5.546E-02	logistic	406373	4959	401414	0.999	0.337
740.12	Osteoarthrosis, localized, secondary	musculoskeletal	rs10852766	0.135	0.071	1.145	5.574E-02	logistic	346286	428	345858	1.000	0.337
204.1	Lymphoid leukemia	neoplasms	rs10852766	0.094	0.049	1.098	5.593E-02	logistic	416514	901	415613	1.000	0.337
38.1	Gram negative septicemia	infectious diseases	rs10852766	0.157	0.082	1.170	5.607E-02	logistic	396961	314	396647	0.999	0.336
395	Heart valve disorders	circulatory system	rs10852766	0.029	0.015	1.030	5.607E-02	logistic	411117	9703	401414	1.000	0.337
411.9	Other acute and subacute forms of ischemic heart disease	circulatory system	rs10852766	-0.075	0.039	0.928	5.628E-02	logistic	372042	1492	370550	0.998	0.337
569	Other disorders of intestine	digestive	rs10852766	0.061	0.032	1.063	5.643E-02	logistic	385990	2151	383839	0.995	0.336
433.3	Cerebral ischemia	circulatory system	rs10852766	0.043	0.023	1.044	5.664E-02	logistic	407003	4318	402685	0.998	0.337
594.2	Calculus of lower urinary tract	genitourinary	rs10852766	0.117	0.061	1.124	5.705E-02	logistic	412949	573	412376	0.999	0.337
348	Other conditions of brain	neurological	rs10852766	0.107	0.057	1.113	6.018E-02	logistic	399717	670	399047	1.000	0.337
241	Nontoxic nodular goiter	endocrine/metabolic	rs10852766	-0.102	0.055	0.903	6.267E-02	logistic	392996	766	392230	0.999	0.336
694	Dyschromia and Vitiligo	dermatologic	rs10852766	0.157	0.084	1.170	6.312E-02	logistic	405505	299	405206	1.000	0.336
535.8	Other specified gastritis	digestive	rs10852766	-0.053	0.029	0.948	6.328E-02	logistic	365017	2803	362214	0.999	0.336
596	Other disorders of bladder	genitourinary	rs10852766	0.035	0.019	1.036	6.480E-02	logistic	407805	6251	401554	1.000	0.337

ST31; PheWAS results for the lead cis-pQTLs of the 4 proteins prioritized (ACOX1, FGF5, FURIN, and MST1).
SNP = Single Nucleotide Polymorphism; SE = Standard error HWE_p = P-value for Hardy-Weinberg equilibrium test

Phenotype	Description	Group	SNP	Beta	SE	Odds tatio	P-value	Type	n_total	n_cases	n_controls	HWE_p	allele_freq
565.1	Anal and rectal polyp	digestive	rs10852766	0.053	0.029	1.054	6.550E-02	logistic	386554	2715	383839	0.996	0.336
201	Hodgkin's disease	neoplasms	rs10852766	0.160	0.087	1.173	6.569E-02	logistic	415895	282	415613	1.000	0.337
496.3	Bronchiectasis	respiratory	rs10852766	0.044	0.024	1.046	6.723E-02	logistic	402578	3769	398809	1.000	0.337
272.11	Hypercholesterolemia	endocrine/metabolic	rs10852766	-0.016	0.009	0.985	6.987E-02	logistic	390444	34679	355765	1.000	0.337
516	Abnormal sputum	respiratory	rs10852766	-0.088	0.048	0.916	7.023E-02	logistic	421370	981	420389	1.000	0.337
619.3	Noninflammatory disorders of cervix	genitourinary	rs10852766	0.143	0.079	1.154	7.032E-02	logistic	217308	342	216966	0.998	0.336
261.4	Vitamin D deficiency	endocrine/metabolic	rs10852766	0.056	0.031	1.058	7.081E-02	logistic	414357	2260	412097	1.000	0.336
578.1	Hematemesis	digestive	rs10852766	-0.074	0.041	0.928	7.171E-02	logistic	384647	1344	383303	0.999	0.336
750.2	Lower gastrointestinal congenital anomalies	congenital anomalies	rs10852766	0.185	0.103	1.203	7.209E-02	logistic	420453	200	420253	1.000	0.337
735.23	Hallux rigidus	musculoskeletal	rs10852766	-0.155	0.086	0.857	7.225E-02	logistic	402973	319	402654	0.999	0.337
426.23	Second degree AV block	circulatory system	rs10852766	-0.106	0.060	0.899	7.684E-02	logistic	372488	640	371848	1.000	0.337
803.3	Fracture of clavicle or scapula	injuries & poisonings	rs10852766	0.097	0.055	1.102	7.864E-02	logistic	397087	709	396378	1.000	0.337
601.1	Prostatitis	genitourinary	rs10852766	-0.101	0.057	0.904	7.957E-02	logistic	157164	703	156461	0.892	0.337
255.2	Adrenal hypofunction	endocrine/metabolic	rs10852766	0.137	0.078	1.147	7.997E-02	logistic	415595	349	415246	1.000	0.337
255.21	Glucocorticoid deficiency	endocrine/metabolic	rs10852766	0.137	0.078	1.147	7.997E-02	logistic	415595	349	415246	1.000	0.337
686	Other local infections of skin and subcutaneous tissue	dermatologic	rs10852766	0.079	0.045	1.083	8.040E-02	logistic	419554	1057	418497	1.000	0.337
333.1	Essential tremor	neurological	rs10852766	0.111	0.064	1.118	8.048E-02	logistic	399579	532	399047	1.000	0.337
433.2	Occlusion of cerebral arteries	circulatory system	rs10852766	-0.042	0.024	0.958	8.114E-02	logistic	406563	3878	402685	0.999	0.336
701	Other hypertrophic and atrophic conditions of skin	dermatologic	rs10852766	0.075	0.043	1.078	8.213E-02	logistic	416347	1161	415186	1.000	0.337
244.4	Hypothyroidism NOS	endocrine/metabolic	rs10852766	0.020	0.011	1.020	8.430E-02	logistic	410296	18066	392230	1.000	0.337
497	Bronchitis	respiratory	rs10852766	-0.154	0.089	0.857	8.437E-02	logistic	399105	296	398809	1.000	0.337
250.4	Abnormal glucose	endocrine/metabolic	rs10852766	-0.080	0.047	0.923	8.856E-02	logistic	387669	1032	386637	0.999	0.337
601	Inflammatory diseases of prostate	genitourinary	rs10852766	-0.085	0.050	0.919	9.130E-02	logistic	157382	921	156461	0.875	0.337
766	Neuralgia, neuritis, and radiculitis NOS	symptoms	rs10852766	0.147	0.087	1.158	9.247E-02	logistic	418267	281	417986	1.000	0.337
292.3	Memory loss	mental disorders	rs10852766	0.110	0.065	1.116	9.284E-02	logistic	405478	505	404973	1.000	0.337
317.11	Alcoholic liver damage	mental disorders	rs10852766	0.072	0.043	1.074	9.306E-02	logistic	404493	1200	403293	1.000	0.337
782.3	Edema	symptoms	rs10852766	0.050	0.030	1.052	9.388E-02	logistic	419509	2451	417058	1.000	0.337
564.1	Irritable Bowel Syndrome	digestive	rs10852766	0.037	0.022	1.037	9.397E-02	logistic	339145	4660	334485	0.996	0.337
344	Other paralytic syndromes	neurological	rs10852766	-0.098	0.059	0.906	9.516E-02	logistic	399714	667	399047	1.000	0.337
569.1	Toxic gastroenteritis and colitis	digestive	rs10852766	0.136	0.082	1.146	9.660E-02	logistic	384157	318	383839	0.995	0.336
277.4	Disorders of bilirubin excretion	endocrine/metabolic	rs10852766	0.113	0.068	1.119	9.837E-02	logistic	420403	462	419941	1.000	0.337
272.9	Unspecified disorder of lipoid metabolism	endocrine/metabolic	rs10852766	0.134	0.082	1.144	1.003E-01	logistic	356085	320	355765	1.000	0.337
41.1	Staphylococcus infections	infectious diseases	rs10852766	0.051	0.031	1.052	1.005E-01	logistic	398981	2334	396647	1.000	0.336
573.5	Jaundice (not of newborn)	digestive	rs10852766	0.092	0.056	1.096	1.009E-01	logistic	400970	696	400274	0.998	0.336
300	Anxiety disorders	mental disorders	rs10852766	0.025	0.015	1.025	1.010E-01	logistic	391438	9816	381622	0.999	0.337
443.1	Raynaud's syndrome	circulatory system	rs10852766	-0.062	0.038	0.940	1.013E-01	logistic	407046	1577	405469	1.000	0.337
315	Develomental delays and disorders	mental disorders	rs10852766	0.092	0.057	1.097	1.024E-01	logistic	422717	680	422037	1.000	0.337
384.4	Perforation of tympanic membrane	sense organs	rs10852766	-0.159	0.098	0.853	1.052E-01	logistic	418963	247	418716	1.000	0.337
290.13	Senile dementia	mental disorders	rs10852766	0.051	0.031	1.052	1.057E-01	logistic	407253	2280	404973	1.000	0.337
557.1	Celiac disease	digestive	rs10852766	0.055	0.034	1.057	1.065E-01	logistic	336383	1898	334485	0.998	0.337
591	Urinary tract infection	genitourinary	rs10852766	0.023	0.014	1.023	1.073E-01	logistic	388723	11796	376927	0.999	0.336
444	Arterial embolism and thrombosis	circulatory system	rs10852766	0.086	0.054	1.090	1.095E-01	logistic	406215	746	405469	1.000	0.337
721	Spondylosis and allied disorders	musculoskeletal	rs10852766	0.054	0.034	1.056	1.106E-01	logistic	410559	1889	408670	1.000	0.337
764	Sciatica	symptoms	rs10852766	0.065	0.041	1.067	1.107E-01	logistic	419302	1316	417986	1.000	0.337
200.1	Polycythemia vera	neoplasms	rs10852766	0.103	0.064	1.108	1.107E-01	logistic	408705	523	408182	0.999	0.337
244	Hypothyroidism	endocrine/metabolic	rs10852766	0.018	0.011	1.018	1.122E-01	logistic	411296	19066	392230	1.000	0.337
516.1	Hemoptysis	respiratory	rs10852766	-0.079	0.050	0.924	1.124E-01	logistic	421320	931	420389	1.000	0.337
800.3	Fracture of tibia and fibula	injuries & poisonings	rs10852766	-0.100	0.063	0.905	1.133E-01	logistic	396955	577	396378	1.000	0.336
737	Curvature of spine	musculoskeletal	rs10852766	-0.080	0.051	0.923	1.134E-01	logistic	403550	896	402654	0.999	0.337
172.2	Other non-epithelial cancer of skin	neoplasms	rs10852766	-0.040	0.025	0.961	1.140E-01	logistic	410817	3609	407208	1.000	0.337
381.1	Otitis media	sense organs	rs10852766	0.092	0.058	1.096	1.149E-01	logistic	419355	639	418716	1.000	0.337
941	Adverse reaction to serum or vaccine	injuries & poisonings	rs10852766	-0.161	0.102	0.851	1.162E-01	logistic	415212	225	414987	1.000	0.337
509.1	Respiratory failure	respiratory	rs10852766	0.035	0.023	1.036	1.172E-01	logistic	395437	4380	391057	0.999	0.337
155.1	Malignant neoplasm of liver, primary	neoplasms	rs10852766	0.127	0.081	1.136	1.179E-01	logistic	394187	325	393862	0.999	0.337
184	Cancer of other female genital organs	neoplasms	rs10852766	-0.043	0.028	0.958	1.192E-01	logistic	207829	2992	204837	0.996	0.336
540.11	Acute appendicitis	digestive	rs10852766	-0.162	0.104	0.850	1.192E-01	logistic	421875	219	421656	1.000	0.337
241.1	Nontoxic uninodular goiter	endocrine/metabolic	rs10852766	-0.160	0.102	0.852	1.193E-01	logistic	392455	225	392230	0.999	0.336
990	Effects radiation NOS	injuries & poisonings	rs10852766	-0.028	0.018	0.972	1.198E-01	logistic	408877	6919	401958	1.000	0.337
604.3	Peyronie's disease	genitourinary	rs10852766	-0.160	0.103	0.852	1.200E-01	logistic	162002	224	161778	0.927	0.337
960.2	Allergy/adverse effect of penicillin	injuries & poisonings	rs10852766	0.017	0.011	1.018	1.200E-01	logistic	385833	18660	367173	0.999	0.336
368.1	Amblyopia	sense organs	rs10852766	0.103	0.066	1.108	1.201E-01	logistic	416041	496	415545	0.999	0.337
751.21	Cystic kidney disease	congenital anomalies	rs10852766	0.116	0.075	1.124	1.204E-01	logistic	420636	383	420253	1.000	0.337
751.2	Congenital anomalies of urinary system	congenital anomalies	rs10852766	0.083	0.054	1.087	1.220E-01	logistic	421003	750	420253	1.000	0.337
496	Chronic airway obstruction	respiratory	rs10852766	0.018	0.011	1.018	1.229E-01	logistic	416701	17892	398809	1.000	0.337
428.2	Heart failure NOS	circulatory system	rs10852766	0.033	0.021	1.034	1.231E-01	logistic	414622	4919	409703	0.999	0.337
300.1	Anxiety disorder	mental disorders	rs10852766	0.024	0.016	1.025	1.233E-01	logistic	390805	9183	381622	0.999	0.337
798	Malaise and fatigue	symptoms	rs10852766	-0.036	0.023	0.965	1.240E-01	logistic	418584	4264	414320	1.000	0.337
594.1	Calculus of kidney	genitourinary	rs10852766	0.041	0.026	1.041	1.252E-01	logistic	415555	3179	412376	0.999	0.337
611	Abnormal findings on mammogram or breast exam	genitourinary	rs10852766	-0.143	0.094	0.866	1.283E-01	logistic	415016	264	414752	1.000	0.337
611.3	Lump or mass in breast	genitourinary	rs10852766	-0.143	0.094	0.866	1.283E-01	logistic	415016	264	414752	1.000	0.337
289	Other diseases of blood and blood-forming organs	hematopoietic	rs10852766	-0.081	0.053	0.922	1.286E-01	logistic	409120	811	408309	1.000	0.337

ST31; PheWAS results for the lead cis-pQTLs of the 4 proteins prioritized (ACOX1, FGF5, FURIN, and MST1).

SNP = Single Nucleotide Polymorphism; SE = Standard error HWE_p = P-value for Hardy-Weinberg equilibrium test

Phenotype	Description	Group	SNP	Beta	SE	Odds tatio	P-value	Type	n_total	n_cases	n_controls	HWE_p	allele_freq
180.1	Cervical cancer	neoplasms	rs10852766	-0.051	0.034	0.950	1.305E-01	logistic	197916	2012	195904	0.995	0.336
512	Other symptoms of respiratory systen	respiratory	rs10852766	0.028	0.019	1.028	1.323E-01	logistic	411090	6491	404599	1.000	0.337
597	Other disorders of urethra and urinary tract	genitourinary	rs10852766	0.049	0.033	1.051	1.332E-01	logistic	403600	2046	401554	1.000	0.337
540	Appendiceal conditions	digestive	rs10852766	-0.105	0.070	0.901	1.371E-01	logistic	422123	467	421656	1.000	0.337
281	Other deficiency anemia	hematopoietic	rs10852766	0.061	0.041	1.062	1.379E-01	logistic	383766	1317	382449	1.000	0.337
619.4	Noninflammatory disorders of vagina	genitourinary	rs10852766	-0.150	0.102	0.860	1.392E-01	logistic	217194	228	216966	0.998	0.336
389	Hearing loss	sense organs	rs10852766	0.026	0.018	1.027	1.402E-01	logistic	415421	7124	408297	1.000	0.337
366.2	Senile cataract	sense organs	rs10852766	0.023	0.016	1.024	1.421E-01	logistic	380814	9136	371678	0.999	0.337
358	Myoneural disorders	neurological	rs10852766	0.121	0.082	1.128	1.434E-01	logistic	419485	317	419168	1.000	0.337
496.2	Chronic bronchitis	respiratory	rs10852766	0.030	0.020	1.030	1.437E-01	logistic	404328	5519	398809	1.000	0.337
592.1	Cystitis	genitourinary	rs10852766	0.062	0.043	1.064	1.441E-01	logistic	378131	1204	376927	0.998	0.336
316	Substance addiction and disorders	mental disorders	rs10852766	-0.118	0.081	0.888	1.455E-01	logistic	403645	352	403293	1.000	0.337
530.13	Barrett's esophagus	digestive	rs10852766	-0.033	0.023	0.968	1.490E-01	logistic	361195	4407	356788	0.999	0.336
622.1	Polyp of corpus uteri	genitourinary	rs10852766	-0.054	0.038	0.947	1.491E-01	logistic	215181	1609	213572	0.996	0.336
276.1	Electrolyte imbalance	endocrine/metabolic	rs10852766	0.021	0.015	1.022	1.500E-01	logistic	406083	10511	395572	0.999	0.337
745	Pain in joint	musculoskeletal	rs10852766	0.036	0.025	1.037	1.505E-01	logistic	414363	3501	410862	1.000	0.337
727	Other disorders of synovium, tendon, and bursa	musculoskeletal	rs10852766	0.041	0.029	1.042	1.524E-01	logistic	391842	2668	389174	0.999	0.337
602	Other disorders of prostate	genitourinary	rs10852766	-0.118	0.083	0.888	1.527E-01	logistic	156802	341	156461	0.904	0.337
689	Disorder of skin and subcutaneous tissue NOS	dermatologic	rs10852766	0.046	0.032	1.047	1.543E-01	logistic	413188	2098	411090	1.000	0.337
599.5	Frequency of urination and polyuria	genitourinary	rs10852766	0.072	0.050	1.074	1.556E-01	logistic	390965	859	390106	0.999	0.336
571.8	Liver abscess and sequelae of chronic liver disease	digestive	rs10852766	0.054	0.038	1.056	1.559E-01	logistic	401788	1514	400274	0.999	0.337
368.2	Diplopia and disorders of binocular vision	sense organs	rs10852766	-0.094	0.066	0.910	1.561E-01	logistic	416069	524	415545	0.999	0.337
217	Vascular hamartomas and non-neoplastic nevi	neoplasms	rs10852766	0.120	0.085	1.127	1.567E-01	logistic	414259	301	413958	0.999	0.337
415.2	Chronic pulmonary heart disease	circulatory system	rs10852766	0.052	0.037	1.053	1.575E-01	logistic	414491	1653	412838	1.000	0.337
427.21	Atrial fibrillation	circulatory system	rs10852766	0.032	0.023	1.033	1.599E-01	logistic	376121	4273	371848	1.000	0.337
381	Otitis media and Eustachian tube disorders	sense organs	rs10852766	0.071	0.051	1.074	1.604E-01	logistic	419568	852	418716	1.000	0.337
636	Early or threatened labor; hemorrhage in early pregnancy	pregnancy complications	rs10852766	0.091	0.065	1.095	1.605E-01	logistic	223122	530	222592	0.999	0.336
198.2	Secondary malignancy of respiratory organs	neoplasms	rs10852766	-0.031	0.022	0.970	1.623E-01	logistic	390707	4701	386006	0.999	0.337
575.7	Other disorders of gallbladder	digestive	rs10852766	0.083	0.060	1.087	1.642E-01	logistic	395657	610	395047	0.999	0.337
362.3	Other nondiabetic retinopathy	sense organs	rs10852766	0.087	0.062	1.091	1.648E-01	logistic	398314	557	397757	0.999	0.337
592	Cystitis and urethritis	genitourinary	rs10852766	0.058	0.042	1.060	1.674E-01	logistic	378176	1249	376927	0.998	0.336
281.1	Megaloblastic anemia	hematopoietic	rs10852766	0.057	0.042	1.059	1.701E-01	logistic	383709	1260	382449	1.000	0.337
180	Cervical cancer and dysplasia	neoplasms	rs10852766	-0.041	0.030	0.960	1.714E-01	logistic	198422	2518	195904	0.996	0.336
261	Vitamin deficiency	endocrine/metabolic	rs10852766	0.033	0.024	1.033	1.720E-01	logistic	416032	3935	412097	1.000	0.336
701.2	Scar conditions and fibrosis of skin	dermatologic	rs10852766	0.101	0.074	1.107	1.733E-01	logistic	415576	390	415186	1.000	0.337
287	Purpura and other hemorrhagic conditions	hematopoietic	rs10852766	0.042	0.031	1.043	1.738E-01	logistic	419896	2343	417553	1.000	0.337
740.9	Osteoarthrosis NOS	musculoskeletal	rs10852766	0.017	0.012	1.017	1.744E-01	logistic	361594	15736	345858	1.000	0.337
208	Benign neoplasm of colon	neoplasms	rs10852766	0.015	0.011	1.015	1.753E-01	logistic	395855	20019	375836	0.999	0.337
480.5	Bronchopneumonia and lung abscess	respiratory	rs10852766	-0.099	0.073	0.905	1.755E-01	logistic	397229	429	396800	1.000	0.336
531.1	Hemorrhage from gastrointestinal ulcer	digestive	rs10852766	-0.086	0.063	0.918	1.755E-01	logistic	411913	574	411339	1.000	0.337
276.11	Hyperosmolality and/or hypernatremia	endocrine/metabolic	rs10852766	0.074	0.055	1.077	1.793E-01	logistic	396286	714	395572	0.999	0.337
592.12	Chronic cystitis	genitourinary	rs10852766	0.124	0.093	1.132	1.811E-01	logistic	377176	249	376927	0.998	0.336
80	Postoperative infection	infectious diseases	rs10852766	-0.046	0.035	0.955	1.813E-01	logistic	414741	1897	412844	1.000	0.337
577.3	Cyst and pseudocyst of pancreas	digestive	rs10852766	-0.089	0.067	0.915	1.815E-01	logistic	418522	514	418008	1.000	0.337
594.3	Calculus of ureter	genitourinary	rs10852766	0.049	0.037	1.050	1.825E-01	logistic	414010	1634	412376	0.999	0.337
426.91	Cardiac pacemaker in situ	circulatory system	rs10852766	0.028	0.021	1.029	1.831E-01	logistic	376826	4978	371848	1.000	0.337
626.12	Excessive or frequent menstruation	genitourinary	rs10852766	-0.043	0.033	0.958	1.835E-01	logistic	194922	2175	192747	0.992	0.337
687	Symptoms affecting skin	dermatologic	rs10852766	0.102	0.077	1.107	1.851E-01	logistic	413083	367	412716	1.000	0.337
458	Hypotension	circulatory system	rs10852766	0.022	0.017	1.022	1.865E-01	logistic	380778	8206	372572	0.995	0.336
574.2	Calculus of bile duct	digestive	rs10852766	-0.130	0.099	0.878	1.867E-01	logistic	395287	240	395047	0.999	0.337
426.9	Cardiac pacemaker/device in situ	circulatory system	rs10852766	0.028	0.021	1.028	1.868E-01	logistic	376950	5102	371848	1.000	0.337
378.1	Strabismus (not specified as paralytic)	sense organs	rs10852766	-0.123	0.093	0.884	1.871E-01	logistic	412196	268	411928	0.999	0.337
958	Certain early complications of trauma or procedure	injuries & poisonings	rs10852766	0.115	0.088	1.121	1.905E-01	logistic	423583	281	423302	1.000	0.337
384	Other disorders of tympanic membrane	sense organs	rs10852766	-0.104	0.080	0.901	1.917E-01	logistic	419080	364	418716	1.000	0.337
560.2	Impaction of intestine	digestive	rs10852766	-0.131	0.101	0.877	1.946E-01	logistic	334714	229	334485	0.997	0.336
561	Symptoms involving digestive systen	digestive	rs10852766	-0.027	0.021	0.973	1.975E-01	logistic	339539	5054	334485	0.998	0.336
270	Disorders of protein plasma/amino-acid transport and metabolism	endocrine/metabolic	rs10852766	0.068	0.053	1.070	1.978E-01	logistic	422490	788	421702	1.000	0.337
733	Other disorders of bone and cartilage	musculoskeletal	rs10852766	0.063	0.049	1.065	1.981E-01	logistic	399585	913	398672	1.000	0.337
172	Skin cancer	neoplasms	rs10852766	-0.025	0.019	0.975	1.986E-01	logistic	413263	6055	407208	1.000	0.337
496.21	Obstructive chronic bronchitis	respiratory	rs10852766	0.026	0.021	1.027	1.996E-01	logistic	404163	5354	398809	1.000	0.337
339	Other headache syndromes	neurological	rs10852766	-0.030	0.023	0.971	2.019E-01	logistic	409356	4169	405187	1.000	0.337
362.31	Separation of retinal layers	sense organs	rs10852766	0.083	0.065	1.086	2.024E-01	logistic	398271	514	397757	0.999	0.337
252	Disorders of parathyroid gland	endocrine/metabolic	rs10852766	0.053	0.042	1.055	2.053E-01	logistic	416485	1239	415246	1.000	0.337
276.13	Hyperpotassemia	endocrine/metabolic	rs10852766	0.045	0.036	1.046	2.059E-01	logistic	397299	1727	395572	0.999	0.337
696.42	Psoriatic arthropathy	dermatologic	rs10852766	0.062	0.050	1.064	2.096E-01	logistic	400183	893	399290	1.000	0.337
350.1	Abnormal involuntary movements	neurological	rs10852766	0.066	0.052	1.068	2.102E-01	logistic	421010	800	420210	1.000	0.337
710.1	Osteomyelitis	musculoskeletal	rs10852766	0.121	0.097	1.129	2.116E-01	logistic	412277	229	412048	1.000	0.337
153.2	Colon cancer	neoplasms	rs10852766	0.028	0.022	1.028	2.144E-01	logistic	375260	4540	370720	0.996	0.337
459.9	Circulatory disease NEC	circulatory system	rs10852766	0.013	0.010	1.013	2.149E-01	logistic	395548	22976	372572	0.999	0.336
358.1	Myasthenia gravis	neurological	rs10852766	0.106	0.086	1.112	2.158E-01	logistic	419463	295	419168	0.999	0.337
281.13	Folate-deficiency anemia	hematopoietic	rs10852766	0.093	0.076	1.098	2.164E-01	logistic	382829	380	382449	0.999	0.337

ST31; PheWAS results for the lead cis-pQTLs of the 4 proteins prioritized (ACOX1, FGF5, FURIN, and MST1).
SNP = Single Nucleotide Polymorphism; SE = Standard error HWE_p = P-value for Hardy-Weinberg equilibrium test

Phenotype	Description	Group	SNP	Beta	SE	Odds tatio	P-value	Type	n_total	n_cases	n_controls	HWE_p	allele_freq
367	Disorders of refraction and accommodation; blindness and low visior	sense organs	rs10852766	-0.024	0.019	0.977	2.194E-01	logistic	417170	6164	411006	1.000	0.337
803.1	Fracture of humerus	injuries & poisonings	rs10852766	-0.056	0.046	0.946	2.218E-01	logistic	397468	1090	396378	1.000	0.336
427.3	Other specified cardiac dysrhythmias	circulatory system	rs10852766	-0.036	0.030	0.965	2.234E-01	logistic	374456	2608	371848	1.000	0.337
367.2	Astigmatism	sense organs	rs10852766	-0.031	0.026	0.969	2.239E-01	logistic	414418	3412	411006	0.999	0.337
293	Symptoms involving head and neck	mental disorders	rs10852766	0.083	0.068	1.087	2.249E-01	logistic	420175	464	419711	1.000	0.337
260	Protein-calorie malnutrition	endocrine/metabolic	rs10852766	0.092	0.076	1.097	2.253E-01	logistic	412473	376	412097	1.000	0.336
364	Corneal opacity and other disorders of cornea	sense organs	rs10852766	-0.044	0.036	0.957	2.255E-01	logistic	399513	1750	397763	0.999	0.337
252.2	Hypoparathyroidism	endocrine/metabolic	rs10852766	0.118	0.098	1.126	2.256E-01	logistic	415471	225	415246	1.000	0.337
441.1	Acute vascular insufficiency of intestine	circulatory system	rs10852766	0.102	0.085	1.108	2.274E-01	logistic	405771	302	405469	1.000	0.337
198.6	Secondary malignancy of bone	neoplasms	rs10852766	-0.026	0.022	0.974	2.278E-01	logistic	390762	4756	386006	0.998	0.337
597.1	Urethral stricture (not specified as infectious)	genitourinary	rs10852766	0.042	0.035	1.043	2.307E-01	logistic	403370	1816	401554	1.000	0.337
695.42	Systemic lupus erythematosus	dermatologic	rs10852766	-0.090	0.076	0.914	2.318E-01	logistic	403885	403	403482	1.000	0.336
252.1	Hyperparathyroidism	endocrine/metabolic	rs10852766	0.055	0.046	1.057	2.329E-01	logistic	416271	1025	415246	1.000	0.337
695	Erythematous conditions	dermatologic	rs10852766	0.042	0.035	1.043	2.330E-01	logistic	407007	1801	405206	1.000	0.337
246	Other disorders of thyroid	endocrine/metabolic	rs10852766	-0.104	0.088	0.901	2.342E-01	logistic	392532	302	392230	0.999	0.336
871	Open wounds of extremities	injuries & poisonings	rs10852766	-0.094	0.079	0.911	2.349E-01	logistic	420520	370	420150	1.000	0.337
480	Pneumonia	respiratory	rs10852766	0.015	0.012	1.015	2.351E-01	logistic	412462	15662	396800	1.000	0.337
386.2	Peripheral or central vertigo	sense organs	rs10852766	0.071	0.060	1.073	2.358E-01	logistic	410281	616	409665	1.000	0.337
386.9	Dizziness and giddiness (Light-headedness and vertigo)	sense organs	rs10852766	-0.032	0.027	0.968	2.362E-01	logistic	412711	3046	409665	1.000	0.337
603.1	Hydrocele	genitourinary	rs10852766	0.083	0.070	1.086	2.371E-01	logistic	162225	447	161778	0.933	0.337
187.1	Malignant neoplasm of unspecified male genital organ	neoplasms	rs10852766	0.026	0.022	1.027	2.410E-01	logistic	183955	4561	179394	0.913	0.337
214.1	Lipoma of skin and subcutaneous tissue	neoplasms	rs10852766	0.064	0.055	1.066	2.441E-01	logistic	414958	731	414227	1.000	0.337
540.1	Appendicitis	digestive	rs10852766	-0.098	0.084	0.907	2.444E-01	logistic	421984	328	421656	1.000	0.337
374	Other disorders of eyelids	sense organs	rs10852766	0.036	0.031	1.037	2.451E-01	logistic	413055	2262	410793	1.000	0.337
357	Inflammatory and toxic neuropathy	neurological	rs10852766	0.039	0.033	1.039	2.470E-01	logistic	421163	1995	419168	1.000	0.337
364.4	Corneal degenerations	sense organs	rs10852766	-0.051	0.044	0.950	2.479E-01	logistic	398917	1154	397763	1.000	0.337
415	Pulmonary heart disease	circulatory system	rs10852766	0.042	0.036	1.042	2.493E-01	logistic	414538	1700	412838	1.000	0.337
426.21	First degree AV block	circulatory system	rs10852766	0.040	0.035	1.041	2.523E-01	logistic	373661	1813	371848	1.000	0.337
385.3	Cholesteatoma	sense organs	rs10852766	-0.117	0.103	0.890	2.547E-01	logistic	418937	221	418716	1.000	0.337
182	Malignant neoplasm of uterus	neoplasms	rs10852766	-0.049	0.043	0.952	2.551E-01	logistic	197217	1245	195972	0.994	0.336
857	Mechanical complication of unspecified genitourinary device, implant, and graft	injuries & poisonings	rs10852766	0.042	0.037	1.043	2.561E-01	logistic	418243	1591	416652	1.000	0.337
459	Other disorders of circulatory system	circulatory system	rs10852766	0.012	0.010	1.012	2.564E-01	logistic	395720	23148	372572	0.999	0.336
743.2	Pathologic fracture	musculoskeletal	rs10852766	0.055	0.049	1.057	2.575E-01	logistic	422164	930	421234	1.000	0.337
480.1	Bacterial pneumonia	respiratory	rs10852766	0.017	0.015	1.017	2.593E-01	logistic	407095	10295	396800	1.000	0.337
530.5	Disorders of esophageal motility	digestive	rs10852766	-0.083	0.074	0.920	2.601E-01	logistic	357212	424	356788	0.999	0.336
255.1	Adrenal hyperfunction	endocrine/metabolic	rs10852766	-0.121	0.108	0.886	2.612E-01	logistic	415447	201	415246	1.000	0.337
371.1	Uveitis, noninfectious or NOS	sense organs	rs10852766	0.104	0.092	1.109	2.615E-01	logistic	411047	254	410793	1.000	0.337
200	Myeloproliferative disease	neoplasms	rs10852766	0.043	0.039	1.044	2.617E-01	logistic	417103	1490	415613	1.000	0.337
634	Miscarriage; stillbirth	pregnancy complications	rs10852766	0.063	0.056	1.065	2.618E-01	logistic	223299	707	222592	0.999	0.336
710	Osteomyelitis, periostitis, and other infections involving bone	musculoskeletal	rs10852766	0.099	0.088	1.104	2.618E-01	logistic	412326	278	412048	1.000	0.337
332	Parkinson's disease	neurological	rs10852766	-0.031	0.028	0.969	2.622E-01	logistic	401966	2919	399047	1.000	0.337
740.1	Osteoarthritis; localized	musculoskeletal	rs10852766	0.010	0.009	1.010	2.633E-01	logistic	374099	28241	345858	1.000	0.337
333.8	Other degenerative diseases of the basal ganglia	neurological	rs10852766	-0.114	0.102	0.892	2.633E-01	logistic	399271	224	399047	1.000	0.337
737.3	Kyphoscoliosis and scoliosis	musculoskeletal	rs10852766	-0.060	0.054	0.942	2.672E-01	logistic	403443	789	402654	0.999	0.337
596.1	Bladder neck obstruction	genitourinary	rs10852766	0.069	0.062	1.072	2.679E-01	logistic	402115	561	401554	1.000	0.336
512.8	Cough	respiratory	rs10852766	0.041	0.037	1.042	2.689E-01	logistic	406184	1585	404599	1.000	0.336
276.42	Alkalosis	endocrine/metabolic	rs10852766	0.093	0.084	1.097	2.693E-01	logistic	395878	306	395572	0.999	0.337
187.2	Malignant neoplasm of testis	neoplasms	rs10852766	0.025	0.022	1.025	2.711E-01	logistic	184014	4620	179394	0.912	0.337
251.1	Hypoglycemia	endocrine/metabolic	rs10852766	0.042	0.038	1.043	2.726E-01	logistic	383028	1531	381497	0.999	0.337
536.8	Dyspepsia and other specified disorders of function of stomach	digestive	rs10852766	-0.041	0.037	0.960	2.737E-01	logistic	363851	1637	362214	0.999	0.336
426	Cardiac conduction disorders	circulatory system	rs10852766	0.016	0.015	1.016	2.742E-01	logistic	382540	10692	371848	1.000	0.337
530.12	Ulcer of esophagus	digestive	rs10852766	-0.032	0.029	0.968	2.743E-01	logistic	359422	2634	356788	0.999	0.336
270.3	Disorders of plasma protein metabolism	endocrine/metabolic	rs10852766	0.059	0.054	1.061	2.745E-01	logistic	422449	747	421702	1.000	0.337
204.21	Myeloid leukemia, acute	neoplasms	rs10852766	0.067	0.061	1.069	2.758E-01	logistic	416195	582	415613	0.999	0.337
287.3	Thrombocytopenia	hematopoietic	rs10852766	0.034	0.032	1.035	2.762E-01	logistic	419777	2224	417553	1.000	0.337
550.4	Umbilical hernia	digestive	rs10852766	-0.044	0.040	0.957	2.763E-01	logistic	366811	1417	365394	0.995	0.336
870	Open wounds of head; neck; and trunk	injuries & poisonings	rs10852766	-0.057	0.053	0.945	2.779E-01	logistic	420976	826	420150	1.000	0.337
270.32	Paraproteinemia	endocrine/metabolic	rs10852766	0.064	0.060	1.066	2.816E-01	logistic	422319	617	421702	1.000	0.337
531.4	Peptic ulcer, site unspecified	digestive	rs10852766	-0.109	0.101	0.897	2.828E-01	logistic	411565	226	411339	1.000	0.337
331.9	Cerebral degeneration, unspecified	neurological	rs10852766	-0.052	0.049	0.949	2.866E-01	logistic	400009	962	399047	1.000	0.337
558	Noninfectious gastroenteritis	digestive	rs10852766	0.024	0.022	1.024	2.874E-01	logistic	338928	4443	334485	0.995	0.337
283	Acquired hemolytic anemias	hematopoietic	rs10852766	-0.114	0.108	0.892	2.878E-01	logistic	382649	200	382449	0.999	0.336
334	Degenerative disease of the spinal cord	neurological	rs10852766	-0.038	0.036	0.962	2.892E-01	logistic	400789	1742	399047	1.000	0.337
296.1	Bipolar	mental disorders	rs10852766	-0.047	0.044	0.954	2.915E-01	logistic	382786	1164	381622	0.999	0.337
585.34	Chronic Kidney Disease, Stage IV	genitourinary	rs10852766	-0.047	0.044	0.954	2.916E-01	logistic	382213	1151	381062	0.998	0.337
198.7	Secondary malignant neoplasm of skin	neoplasms	rs10852766	-0.099	0.094	0.906	2.927E-01	logistic	386268	262	386006	0.999	0.337
204.4	Multiple myeloma	neoplasms	rs10852766	-0.047	0.045	0.954	2.929E-01	logistic	416757	1144	415613	0.999	0.337
536	Disorders of function of stomach	digestive	rs10852766	-0.039	0.037	0.962	2.938E-01	logistic	363858	1644	362214	0.999	0.336
41	Bacterial infection NOS	infectious diseases	rs10852766	0.016	0.016	1.016	2.987E-01	logistic	406056	9409	396647	0.999	0.336
575.1	Cholangitis	digestive	rs10852766	0.052	0.050	1.053	3.005E-01	logistic	395932	885	395047	0.999	0.337
530.14	Reflux esophagitis	digestive	rs10852766	0.027	0.026	1.027	3.047E-01	logistic	360032	3244	356788	1.000	0.337

ST31; PheWAS results for the lead cis-pQTLs of the 4 proteins prioritized (ACOX1, FGF5, FURIN, and MST1).

SNP = Single Nucleotide Polymorphism; SE = Standard error HWE_p = P-value for Hardy-Weinberg equilibrium test

Phenotype	Description	Group	SNP	Beta	SE	Odds tatio	P-value	Type	n_total	n_cases	n_controls	HWE_p	allele_freq
433.5	Cerebral aneurysm	circulatory system	rs10852766	0.072	0.070	1.074	3.065E-01	logistic	403130	445	402685	0.999	0.337
715.2	Ankylosing spondylitis	musculoskeletal	rs10852766	-0.073	0.071	0.930	3.068E-01	logistic	408956	449	408507	1.000	0.337
535	Gastritis and duodenitis	digestive	rs10852766	-0.012	0.012	0.988	3.085E-01	logistic	378649	16435	362214	1.000	0.336
750.1	Upper gastrointestinal congenital anomalies	congenital anomalies	rs10852766	-0.107	0.105	0.899	3.089E-01	logistic	420464	211	420253	1.000	0.337
41.11	Methicillin sensitive Staphylococcus aureus	infectious diseases	rs10852766	0.037	0.037	1.038	3.090E-01	logistic	398282	1635	396647	1.000	0.336
427.12	Paroxysmal ventricular tachycardia	circulatory system	rs10852766	-0.049	0.048	0.952	3.103E-01	logistic	372834	986	371848	1.000	0.337
706	Diseases of sebaceous glands	dermatologic	rs10852766	-0.040	0.040	0.961	3.129E-01	logistic	413434	1435	411999	1.000	0.337
742	Derangement of joint, non-traumatic	musculoskeletal	rs10852766	0.071	0.071	1.074	3.136E-01	logistic	415444	434	415010	1.000	0.337
374.1	Ectropion or entropion	sense organs	rs10852766	0.056	0.056	1.058	3.140E-01	logistic	411493	700	410793	1.000	0.337
465	Acute upper respiratory infections of multiple or unspecified sites	respiratory	rs10852766	-0.057	0.057	0.944	3.170E-01	logistic	420386	694	419692	1.000	0.337
287.31	Primary thrombocytopenia	hematopoietic	rs10852766	0.065	0.065	1.067	3.172E-01	logistic	418072	519	417553	1.000	0.337
740	Osteoarthrosis	musculoskeletal	rs10852766	0.008	0.008	1.008	3.172E-01	logistic	392374	46516	345858	1.000	0.337
280.1	Iron deficiency anemias, unspecified or not due to blood loss	hematopoietic	rs10852766	0.016	0.016	1.016	3.187E-01	logistic	391238	8789	382449	0.999	0.337
721.1	Spondylosis without myelopathy	musculoskeletal	rs10852766	0.038	0.038	1.039	3.226E-01	logistic	410174	1504	408670	1.000	0.337
585.4	Chronic kidney disease, Stage I or II	genitourinary	rs10852766	-0.053	0.053	0.949	3.232E-01	logistic	381861	799	381062	0.999	0.337
509.8	Dependence on respirator [Ventilator] or supplemental oxygen	respiratory	rs10852766	-0.056	0.057	0.945	3.234E-01	logistic	391755	698	391057	1.000	0.337
472	Chronic pharyngitis and nasopharyngitis	respiratory	rs10852766	-0.093	0.094	0.912	3.235E-01	logistic	396779	262	396517	1.000	0.336
275.3	Disorders of magnesium metabolism	endocrine/metabolic	rs10852766	0.037	0.038	1.038	3.238E-01	logistic	417729	1544	416185	1.000	0.337
473.3	Paralysis/spasm of vocal cords or larynx	respiratory	rs10852766	-0.100	0.101	0.905	3.239E-01	logistic	396742	225	396517	1.000	0.336
579.8	Nonspecific abnormal findings in stool contents	digestive	rs10852766	0.059	0.060	1.060	3.241E-01	logistic	383921	618	383303	1.000	0.336
199	Neoplasm of uncertain behavior	neoplasms	rs10852766	0.059	0.060	1.061	3.249E-01	logistic	386620	614	386006	0.999	0.337
751	Genitourinary congenital anomalies	congenital anomalies	rs10852766	0.049	0.050	1.050	3.261E-01	logistic	421129	876	420253	1.000	0.337
375	Disorders of lacrimal system	sense organs	rs10852766	0.038	0.039	1.039	3.274E-01	logistic	413379	1451	411928	0.999	0.337
573.3	Hepatomegaly	digestive	rs10852766	-0.089	0.091	0.915	3.286E-01	logistic	400551	277	400274	0.998	0.336
444.1	Arterial embolism and thrombosis of lower extremity artery	circulatory system	rs10852766	0.068	0.070	1.071	3.293E-01	logistic	405915	446	405469	1.000	0.337
174.11	Malignant neoplasm of female breast	neoplasms	rs10852766	-0.016	0.016	0.985	3.301E-01	logistic	212959	9200	203759	0.993	0.336
747.12	Valvular heart disease/ heart chambers	congenital anomalies	rs10852766	0.086	0.088	1.089	3.308E-01	logistic	421875	280	421595	1.000	0.336
446	Polyarteritis nodosa and allied conditions	circulatory system	rs10852766	0.042	0.043	1.043	3.342E-01	logistic	406656	1187	405469	1.000	0.337
476	Allergic rhinitis	respiratory	rs10852766	0.044	0.045	1.045	3.348E-01	logistic	397580	1063	396517	1.000	0.336
573.9	Abnormal serum enzyme levels	digestive	rs10852766	0.096	0.100	1.101	3.383E-01	logistic	400489	215	400274	0.999	0.336
626.1	Irregular menstrual cycle/bleeding	genitourinary	rs10852766	-0.023	0.024	0.978	3.387E-01	logistic	196900	4153	192747	0.990	0.336
394	Rheumatic disease of the heart valves	circulatory system	rs10852766	0.021	0.022	1.021	3.392E-01	logistic	406218	4804	401414	0.999	0.337
496.1	Emphysema	respiratory	rs10852766	0.026	0.027	1.027	3.401E-01	logistic	401774	2965	398809	1.000	0.337
481	Influenza	respiratory	rs10852766	0.065	0.069	1.067	3.418E-01	logistic	397264	464	396800	1.000	0.336
296.2	Depression	mental disorders	rs10852766	0.009	0.009	1.009	3.419E-01	logistic	408593	26971	381622	1.000	0.337
189.1	Cancer of kidney and renal pelvis	neoplasms	rs10852766	0.041	0.043	1.042	3.420E-01	logistic	418392	1205	417187	1.000	0.337
446.5	Giant cell arteritis	circulatory system	rs10852766	0.057	0.060	1.059	3.437E-01	logistic	406071	602	405469	1.000	0.337
577	Diseases of pancreas	digestive	rs10852766	-0.025	0.026	0.975	3.461E-01	logistic	421247	3239	418008	1.000	0.337
596.5	Functional disorders of bladder	genitourinary	rs10852766	0.055	0.059	1.057	3.471E-01	logistic	402194	640	401554	1.000	0.336
961.1	Poisoning/allergy of sulfonamides	injuries & poisonings	rs10852766	-0.056	0.060	0.946	3.507E-01	logistic	367807	634	367173	0.998	0.336
610	Benign mammary dysplasias	genitourinary	rs10852766	-0.063	0.067	0.939	3.513E-01	logistic	220777	505	220272	0.999	0.336
530.11	GERD	digestive	rs10852766	0.012	0.013	1.012	3.515E-01	logistic	371656	14868	356788	1.000	0.337
117	Mycoses	infectious diseases	rs10852766	0.083	0.089	1.087	3.529E-01	logistic	418652	272	418380	1.000	0.337
567	Peritonitis and retroperitoneal infections	digestive	rs10852766	-0.052	0.056	0.949	3.540E-01	logistic	384568	729	383839	0.995	0.336
500.2	Pneumoconiosis	respiratory	rs10852766	0.071	0.076	1.073	3.552E-01	logistic	391432	375	391057	0.999	0.337
335	Multiple sclerosis	neurological	rs10852766	0.036	0.039	1.036	3.563E-01	logistic	400514	1467	399047	1.000	0.337
153	Colorectal cancer	neoplasms	rs10852766	0.017	0.018	1.017	3.583E-01	logistic	377768	7048	370720	0.995	0.337
803	Fracture of upper limb	injuries & poisonings	rs10852766	0.021	0.023	1.021	3.594E-01	logistic	400593	4215	396378	1.000	0.337
728.7	Fasciitis	musculoskeletal	rs10852766	-0.032	0.035	0.968	3.602E-01	logistic	391018	1844	389174	0.999	0.337
642	Hypertension complicating pregnancy, childbirth, and the puerperium	pregnancy complications	rs10852766	0.064	0.070	1.066	3.606E-01	logistic	228250	456	227794	0.999	0.336
761	Cervicalgia	symptoms	rs10852766	0.050	0.054	1.051	3.620E-01	logistic	422454	744	421710	1.000	0.337
150	Cancer of esophagus	neoplasms	rs10852766	0.036	0.039	1.037	3.623E-01	logistic	395291	1429	393862	0.999	0.337
790.6	Other abnormal blood chemistry	symptoms	rs10852766	-0.089	0.099	0.914	3.642E-01	logistic	422077	237	421840	1.000	0.337
395.3	Nonrheumatic tricuspid valve disorders	circulatory system	rs10852766	-0.089	0.098	0.915	3.666E-01	logistic	401653	239	401414	0.999	0.336
743	Osteoporosis, osteopenia and pathological fracture	musculoskeletal	rs10852766	0.040	0.044	1.040	3.667E-01	logistic	422387	1153	421234	1.000	0.337
159.2	Malignant neoplasm of small intestine, including duodenum	neoplasms	rs10852766	-0.085	0.095	0.918	3.687E-01	logistic	394117	255	393862	0.999	0.337
458.9	Hypotension NOS	circulatory system	rs10852766	0.020	0.023	1.021	3.688E-01	logistic	376953	4381	372572	0.996	0.336
165	Cancer within the respiratory system	neoplasms	rs10852766	-0.019	0.021	0.982	3.711E-01	logistic	422554	5325	417229	1.000	0.337
426.24	Atrioventricular block, complete	circulatory system	rs10852766	0.042	0.047	1.043	3.724E-01	logistic	372833	985	371848	1.000	0.337
635	Hemorrhage during pregnancy; childbirth and postpartum	pregnancy complications	rs10852766	-0.067	0.075	0.935	3.748E-01	logistic	223005	413	222592	0.998	0.336
626.8	Infertility, female	genitourinary	rs10852766	0.078	0.088	1.081	3.759E-01	logistic	193029	282	192747	0.993	0.337
394.7	Disease of tricuspid valve	circulatory system	rs10852766	0.051	0.058	1.052	3.769E-01	logistic	402072	658	401414	0.999	0.336
401	Hypertension	circulatory system	rs10852766	-0.005	0.006	0.995	3.787E-01	logistic	390097	99434	290663	0.999	0.337
428	Congestive heart failure; nonhypertensive	circulatory system	rs10852766	0.015	0.017	1.015	3.789E-01	logistic	417859	8156	409703	0.999	0.337
427.7	Tachycardia NOS	circulatory system	rs10852766	0.030	0.034	1.031	3.794E-01	logistic	373741	1893	371848	1.000	0.337
396	Abnormal heart sounds	circulatory system	rs10852766	-0.043	0.049	0.958	3.804E-01	logistic	402357	943	401414	0.999	0.336
420.3	Endocarditis	circulatory system	rs10852766	0.052	0.060	1.054	3.815E-01	logistic	417383	613	416770	1.000	0.337
961	Poisoning by other anti-infectives	injuries & poisonings	rs10852766	0.076	0.087	1.079	3.838E-01	logistic	367461	288	367173	0.998	0.336
441	Vascular insufficiency of intestine	circulatory system	rs10852766	0.049	0.057	1.051	3.839E-01	logistic	406151	682	405469	1.000	0.337
578.2	Blood in stool	digestive	rs10852766	-0.031	0.035	0.970	3.848E-01	logistic	385103	1800	383303	0.999	0.336
555.2	Ulcerative colitis	digestive	rs10852766	0.023	0.026	1.023	3.850E-01	logistic	337679	3194	334485	0.997	0.337

ST31; PheWAS results for the lead cis-pQTLs of the 4 proteins prioritized (ACOX1, FGF5, FURIN, and MST1).

SNP = Single Nucleotide Polymorphism; SE = Standard error HWE_p = P-value for Hardy-Weinberg equilibrium test

Phenotype	Description	Group	SNP	Beta	SE	Odds tatio	P-value	Type	n_total	n_cases	n_controls	HWE_p	allele_freq
286	Coagulation defects	hematopoietic	rs10852766	-0.043	0.049	0.958	3.866E-01	logistic	418481	928	417553	1.000	0.337
577.2	Chronic pancreatitis	digestive	rs10852766	-0.053	0.061	0.948	3.869E-01	logistic	418611	603	418008	1.000	0.337
574	Cholelithiasis and cholecystitis	digestive	rs10852766	0.012	0.014	1.012	3.878E-01	logistic	406113	11066	395047	0.999	0.337
564.9	Personal history of diseases of digestive systen	digestive	rs10852766	-0.011	0.013	0.989	3.883E-01	logistic	348229	13744	334485	0.998	0.336
385	Other disorders of middle ear and mastoid	sense organs	rs10852766	-0.074	0.086	0.928	3.893E-01	logistic	419023	307	418716	1.000	0.337
835	Internal derangement of knee	injuries & poisonings	rs10852766	-0.019	0.022	0.981	3.931E-01	logistic	409924	4709	405215	1.000	0.337
780	Hypothermia/Chills	symptoms	rs10852766	0.084	0.099	1.088	3.947E-01	logistic	423421	221	423200	1.000	0.337
172.3	Carcinoma in situ of skin	neoplasms	rs10852766	-0.063	0.075	0.938	3.956E-01	logistic	407616	408	407208	0.999	0.337
204.2	Myeloid leukemia	neoplasms	rs10852766	0.046	0.054	1.047	3.959E-01	logistic	416360	747	415613	1.000	0.337
994	Sepsis and SIRS	injuries & poisonings	rs10852766	-0.016	0.018	0.984	3.959E-01	logistic	418535	6755	411780	1.000	0.337
994.2	Sepsis	injuries & poisonings	rs10852766	-0.016	0.018	0.984	3.959E-01	logistic	418535	6755	411780	1.000	0.337
334.2	Anterior horn cell disease	neurological	rs10852766	-0.060	0.071	0.942	3.989E-01	logistic	399497	450	399047	1.000	0.337
603	Other disorders of testis	genitourinary	rs10852766	0.058	0.068	1.059	4.009E-01	logistic	162246	468	161778	0.933	0.337
323	Encephalitis	neurological	rs10852766	0.066	0.079	1.069	4.018E-01	logistic	422420	349	422071	1.000	0.337
286.12	Congenital deficiency of other clotting factors (including factor VII	hematopoietic	rs10852766	0.080	0.096	1.084	4.028E-01	logistic	417788	235	417553	1.000	0.337
174.1	Breast cancer [female]	neoplasms	rs10852766	-0.013	0.015	0.987	4.032E-01	logistic	213896	10137	203759	0.993	0.336
703.1	Ingrowing nail	dermatologic	rs10852766	0.086	0.103	1.090	4.045E-01	logistic	421519	205	421314	1.000	0.337
430.2	Intracerebral hemorrhage	circulatory system	rs10852766	0.038	0.045	1.038	4.045E-01	logistic	403773	1088	402685	0.999	0.337
401.1	Essential hypertension	circulatory system	rs10852766	-0.005	0.006	0.995	4.048E-01	logistic	389876	99213	290663	0.999	0.337
939	Atopic/contact dermatitis due to other or unspecified	dermatologic	rs10852766	0.028	0.034	1.028	4.064E-01	logistic	416950	1963	414987	1.000	0.337
586.2	Cyst of kidney, acquired	genitourinary	rs10852766	-0.029	0.035	0.971	4.077E-01	logistic	382916	1854	381062	0.999	0.337
614	Inflammatory diseases of female pelvic organs	genitourinary	rs10852766	0.031	0.038	1.032	4.082E-01	logistic	218979	1541	217438	0.996	0.336
586	Other disorders of the kidney and ureters	genitourinary	rs10852766	-0.020	0.024	0.980	4.088E-01	logistic	384904	3842	381062	0.999	0.337
281.11	Pernicious anemia	hematopoietic	rs10852766	0.045	0.055	1.046	4.131E-01	logistic	383186	737	382449	1.000	0.337
276.12	Hyposmolality and/or hyponatremia	endocrine/metabolic	rs10852766	0.017	0.021	1.018	4.136E-01	logistic	400551	4979	395572	0.999	0.337
573.7	Abnormal results of function study of liver	digestive	rs10852766	0.020	0.024	1.020	4.169E-01	logistic	404090	3816	400274	0.999	0.336
687.1	Rash and other nonspecific skin eruption	dermatologic	rs10852766	-0.037	0.046	0.964	4.179E-01	logistic	413803	1087	412716	1.000	0.337
112	Candidiasis	infectious diseases	rs10852766	0.030	0.038	1.031	4.208E-01	logistic	419935	1555	418380	1.000	0.337
297	Suicidal ideation or attempt	mental disorders	rs10852766	-0.064	0.080	0.938	4.210E-01	logistic	381982	360	381622	0.999	0.337
433.1	Occlusion and stenosis of precerebral arteries	circulatory system	rs10852766	-0.031	0.038	0.970	4.215E-01	logistic	404257	1572	402685	0.999	0.337
695.7	Prurigo and Lichen	dermatologic	rs10852766	0.068	0.085	1.070	4.228E-01	logistic	405510	304	405206	1.000	0.336
276.5	Hypovolemia	endocrine/metabolic	rs10852766	0.017	0.021	1.017	4.250E-01	logistic	400853	5281	395572	0.999	0.337
157	Pancreatic cancer	neoplasms	rs10852766	-0.032	0.040	0.968	4.257E-01	logistic	395256	1394	393862	0.999	0.337
709.3	Systemic sclerosis	dermatologic	rs10852766	-0.081	0.102	0.922	4.265E-01	logistic	404118	219	403899	1.000	0.336
555.1	Regional enteritis	digestive	rs10852766	-0.028	0.035	0.972	4.266E-01	logistic	336302	1817	334485	0.998	0.336
580.14	Chronic glomerulonephritis, NOS	genitourinary	rs10852766	0.066	0.083	1.068	4.272E-01	logistic	381376	314	381062	0.999	0.337
475	Chronic sinusitis	respiratory	rs10852766	0.038	0.048	1.039	4.276E-01	logistic	397468	951	396517	1.000	0.336
378	Strabismus and other disorders of binocular eye movements	sense organs	rs10852766	-0.039	0.049	0.962	4.278E-01	logistic	412884	956	411928	0.999	0.337
288.1	Decreased white blood cell count	hematopoietic	rs10852766	0.021	0.026	1.021	4.332E-01	logistic	411502	3193	408309	0.999	0.337
288.11	Neutropenia	hematopoietic	rs10852766	0.021	0.026	1.021	4.332E-01	logistic	411502	3193	408309	0.999	0.337
729	Other disorders of soft tissues	musculoskeletal	rs10852766	-0.075	0.096	0.928	4.333E-01	logistic	389423	249	389174	0.999	0.337
277	Other disorders of metabolism	endocrine/metabolic	rs10852766	-0.036	0.046	0.965	4.338E-01	logistic	421029	1088	419941	1.000	0.337
500	Lung disease due to external agents	respiratory	rs10852766	0.047	0.060	1.048	4.339E-01	logistic	391675	618	391057	0.999	0.337
473.4	Voice disturbance	respiratory	rs10852766	-0.070	0.090	0.933	4.366E-01	logistic	396801	284	396517	1.000	0.336
706.2	Sebaceous cyst	dermatologic	rs10852766	-0.031	0.040	0.969	4.390E-01	logistic	413393	1394	411999	1.000	0.337
375.2	Epiphora	sense organs	rs10852766	0.063	0.081	1.065	4.418E-01	logistic	412257	329	411928	0.999	0.337
395.1	Nonrheumatic mitral valve disorders	circulatory system	rs10852766	0.019	0.025	1.019	4.473E-01	logistic	405090	3676	401414	1.000	0.337
627.1	Postmenopausal bleeding	genitourinary	rs10852766	0.027	0.035	1.027	4.517E-01	logistic	194538	1791	192747	0.992	0.337
364.5	Corneal dystrophy	sense organs	rs10852766	0.056	0.075	1.058	4.528E-01	logistic	398152	389	397763	0.999	0.337
530	Diseases of esophagus	digestive	rs10852766	-0.007	0.009	0.993	4.534E-01	logistic	388733	31945	356788	1.000	0.336
189.11	Malignant neoplasm of kidney, except pelvis	neoplasms	rs10852766	0.033	0.044	1.034	4.544E-01	logistic	418324	1137	417187	1.000	0.337
41.4	E. coli	infectious diseases	rs10852766	0.019	0.026	1.020	4.547E-01	logistic	399991	3344	396647	0.999	0.336
250.42	Other abnormal glucose	endocrine/metabolic	rs10852766	0.063	0.085	1.065	4.552E-01	logistic	386940	303	386637	0.999	0.337
427.41	Ventricular fibrillation and flutter	circulatory system	rs10852766	-0.069	0.093	0.933	4.567E-01	logistic	372113	265	371848	1.000	0.337
280.2	Iron deficiency anemia secondary to blood loss (chronic	hematopoietic	rs10852766	-0.065	0.087	0.937	4.580E-01	logistic	382749	300	382449	0.999	0.336
743.21	Pathologic fracture of vertebrae	musculoskeletal	rs10852766	0.044	0.059	1.045	4.583E-01	logistic	421861	627	421234	1.000	0.337
369	Infection of the eye	sense organs	rs10852766	-0.055	0.074	0.947	4.590E-01	logistic	411210	417	410793	1.000	0.337
275.5	Disorders of calcium/phosphorus metabolism	endocrine/metabolic	rs10852766	-0.023	0.031	0.977	4.622E-01	logistic	418502	2317	416185	1.000	0.337
770	Myalgia and myositis unspecified	symptoms	rs10852766	-0.026	0.035	0.975	4.624E-01	logistic	422525	1839	420686	1.000	0.337
720.1	Spinal stenosis of lumbar region	musculoskeletal	rs10852766	-0.022	0.029	0.979	4.659E-01	logistic	411268	2598	408670	1.000	0.337
585.3	Chronic renal failure [CKD]	genitourinary	rs10852766	-0.010	0.014	0.990	4.673E-01	logistic	393739	12677	381062	0.999	0.337
714.1	Rheumatoid arthritis	musculoskeletal	rs10852766	-0.018	0.024	0.983	4.676E-01	logistic	412357	3850	408507	1.000	0.337
587	Kidney replaced by transpant	genitourinary	rs10852766	0.048	0.067	1.049	4.725E-01	logistic	381556	494	381062	0.998	0.337
480.11	Pneumococcal pneumonia	respiratory	rs10852766	0.011	0.015	1.011	4.739E-01	logistic	406463	9663	396800	1.000	0.337
333.4	Torsion dystonia	neurological	rs10852766	-0.062	0.087	0.940	4.740E-01	logistic	399349	302	399047	1.000	0.337
159.4	Malignant neoplasm of retroperitoneum and peritoneum	neoplasms	rs10852766	0.065	0.091	1.067	4.757E-01	logistic	394127	265	393862	0.999	0.337
695.4	Lupus (localized and systemic)	dermatologic	rs10852766	-0.048	0.068	0.953	4.772E-01	logistic	403975	493	403482	1.000	0.336
411.8	Other chronic ischemic heart disease, unspecified	circulatory system	rs10852766	-0.008	0.012	0.992	4.785E-01	logistic	389207	18657	370550	0.999	0.336
70.4	Chronic hepatitis	infectious diseases	rs10852766	0.062	0.087	1.064	4.789E-01	logistic	415491	287	415204	1.000	0.337
174	Breast cancer	neoplasms	rs10852766	-0.011	0.015	0.989	4.789E-01	logistic	401305	10248	391057	0.998	0.337
386.1	Meniere's disease	sense organs	rs10852766	0.041	0.059	1.042	4.813E-01	logistic	410308	643	409665	1.000	0.337

ST31; PheWAS results for the lead cis-pQTLs of the 4 proteins prioritized (ACOX1, FGF5, FURIN, and MST1).
SNP = Single Nucleotide Polymorphism; SE = Standard error HWE_p = P-value for Hardy-Weinberg equilibrium test

Phenotype	Description	Group	SNP	Beta	SE	Odds tatio	P-value	Type	n_total	n_cases	n_controls	HWE_p	allele_freq
285	Other anemias	hematopoietic	rs10852766	0.010	0.014	1.010	4.814E-01	logistic	393921	11472	382449	1.000	0.337
537	Other disorders of stomach and duodenum	digestive	rs10852766	0.024	0.035	1.025	4.843E-01	logistic	364031	1817	362214	0.998	0.336
443.9	Peripheral vascular disease, unspecifiec	circulatory system	rs10852766	0.018	0.025	1.018	4.852E-01	logistic	409046	3577	405469	1.000	0.337
803.2	Fracture of radius and ulna	injuries & poisonings	rs10852766	0.022	0.032	1.022	4.863E-01	logistic	398618	2240	396378	1.000	0.337
600	Hyperplasia of prostate	genitourinary	rs10852766	0.010	0.014	1.010	4.864E-01	logistic	169018	12557	156461	0.753	0.337
292	Neurological disorders	mental disorders	rs10852766	0.014	0.020	1.014	4.873E-01	logistic	410372	5399	404973	1.000	0.337
513	Respiratory abnormalities	respiratory	rs10852766	-0.050	0.073	0.951	4.884E-01	logistic	423122	431	422691	1.000	0.337
701.3	Circumscribed scleroderma	dermatologic	rs10852766	0.068	0.099	1.070	4.910E-01	logistic	415410	224	415186	1.000	0.337
727.1	Synovitis and tenosynovitis	musculoskeletal	rs10852766	0.057	0.084	1.059	4.932E-01	logistic	389486	312	389174	0.999	0.337
290.1	Dementias	mental disorders	rs10852766	0.016	0.023	1.016	4.938E-01	logistic	409256	4283	404973	1.000	0.337
415.21	Primary pulmonary hypertensior	circulatory system	rs10852766	0.054	0.079	1.056	4.944E-01	logistic	413189	351	412838	0.999	0.337
289.3	Personal history of diseases of blood and blood-forming organs	hematopoietic	rs10852766	-0.060	0.088	0.942	4.945E-01	logistic	408604	295	408309	0.999	0.337
280	Iron deficiency anemias	hematopoietic	rs10852766	0.011	0.016	1.011	4.947E-01	logistic	391534	9085	382449	0.999	0.337
187	Cancer of other male genital organs	neoplasms	rs10852766	0.012	0.018	1.012	4.982E-01	logistic	186921	7527	179394	0.941	0.337
420.2	Pericarditis	circulatory system	rs10852766	0.026	0.039	1.027	4.985E-01	logistic	418236	1466	416770	1.000	0.337
727.6	Rupture of tendon, nontraumatic	musculoskeletal	rs10852766	0.027	0.041	1.028	5.000E-01	logistic	390517	1343	389174	0.999	0.337
379	Other disorders of eye	sense organs	rs10852766	-0.031	0.047	0.969	5.039E-01	logistic	412963	1035	411928	0.999	0.337
512.7	Shortness of breath	respiratory	rs10852766	0.016	0.024	1.016	5.054E-01	logistic	408527	3928	404599	1.000	0.336
296	Mood disorders	mental disorders	rs10852766	0.006	0.009	1.006	5.081E-01	logistic	409186	27564	381622	1.000	0.337
695.3	Rosacea	dermatologic	rs10852766	0.061	0.094	1.063	5.117E-01	logistic	405456	250	405206	1.000	0.336
720	Spinal stenosis	musculoskeletal	rs10852766	-0.015	0.023	0.985	5.127E-01	logistic	412805	4135	408670	1.000	0.337
290	Delirium dementia and amnestic and other cognitive disorders	mental disorders	rs10852766	0.012	0.018	1.012	5.133E-01	logistic	412158	7185	404973	1.000	0.337
585.1	Acute renal failure	genitourinary	rs10852766	0.009	0.013	1.009	5.134E-01	logistic	394375	13313	381062	0.999	0.337
195.1	Malignant neoplasm, other	neoplasms	rs10852766	-0.011	0.017	0.989	5.140E-01	logistic	393640	7634	386006	0.999	0.337
470	Septal Deviations/Turbinate Hypertrophy	respiratory	rs10852766	0.024	0.037	1.024	5.146E-01	logistic	398163	1646	396517	1.000	0.336
250.1	Type 1 diabetes	endocrine/metabolic	rs10852766	0.020	0.030	1.020	5.147E-01	logistic	389041	2404	386637	0.999	0.337
411.3	Angina pectoris	circulatory system	rs10852766	0.008	0.012	1.008	5.152E-01	logistic	387093	16543	370550	0.999	0.337
253.2	Pituitary hypofunction	endocrine/metabolic	rs10852766	-0.058	0.089	0.944	5.158E-01	logistic	415534	288	415246	1.000	0.337
345.11	Generalized convulsive epilepsy	neurological	rs10852766	0.048	0.075	1.050	5.166E-01	logistic	399441	394	399047	1.000	0.337
531	Peptic ulcer (excl. esophageal)	digestive	rs10852766	0.015	0.024	1.015	5.174E-01	logistic	415329	3990	411339	1.000	0.337
578.9	Hemorrhage of gastrointestinal tract	digestive	rs10852766	0.018	0.028	1.018	5.175E-01	logistic	386245	2942	383303	1.000	0.336
614.1	Pelvic peritoneal adhesions, female (postoperative) (postinfection	genitourinary	rs10852766	0.048	0.075	1.049	5.211E-01	logistic	217833	395	217438	0.997	0.336
300.13	Phobia	mental disorders	rs10852766	0.039	0.061	1.039	5.238E-01	logistic	382219	597	381622	0.999	0.337
369.5	Conjunctivitis, infectious	sense organs	rs10852766	-0.067	0.105	0.935	5.239E-01	logistic	410999	206	410793	1.000	0.337
698	Pruritus and related conditions	dermatologic	rs10852766	-0.057	0.090	0.944	5.272E-01	logistic	422717	278	422439	1.000	0.337
195	Cancer, suspected or other	neoplasms	rs10852766	-0.011	0.017	0.989	5.280E-01	logistic	393669	7663	386006	0.999	0.337
707.2	Chronic ulcer of leg or foot	dermatologic	rs10852766	0.020	0.033	1.021	5.308E-01	logistic	418232	2102	416130	1.000	0.337
721.8	Other allied disorders of spine	musculoskeletal	rs10852766	0.065	0.104	1.068	5.308E-01	logistic	408871	201	408670	1.000	0.337
117.4	Aspergillosis	infectious diseases	rs10852766	0.064	0.102	1.066	5.322E-01	logistic	418590	210	418380	1.000	0.337
427.6	Premature beats	circulatory system	rs10852766	-0.038	0.061	0.963	5.354E-01	logistic	372449	601	371848	1.000	0.337
728.71	Contracture of palmar fascia [Dupuytren's disease]	musculoskeletal	rs10852766	-0.023	0.036	0.978	5.354E-01	logistic	390879	1705	389174	0.999	0.337
276	Disorders of fluid, electrolyte, and acid-base balance	endocrine/metabolic	rs10852766	0.008	0.012	1.008	5.388E-01	logistic	411277	15705	395572	0.999	0.337
427.5	Arrhythmia (cardiac) NOS	circulatory system	rs10852766	0.041	0.066	1.042	5.395E-01	logistic	372348	500	371848	1.000	0.337
571	Chronic liver disease and cirrhosis	digestive	rs10852766	0.012	0.020	1.012	5.411E-01	logistic	406135	5861	400274	0.999	0.336
290.11	Alzheimer's disease	mental disorders	rs10852766	0.019	0.031	1.019	5.431E-01	logistic	407367	2394	404973	1.000	0.337
352.1	Trigeminal nerve disorders [CN5]	neurological	rs10852766	-0.041	0.067	0.960	5.437E-01	logistic	402912	497	402415	1.000	0.337
440	Atherosclerosis	circulatory system	rs10852766	-0.028	0.046	0.973	5.442E-01	logistic	406545	1076	405469	1.000	0.337
531.3	Duodenal ulcer	digestive	rs10852766	0.027	0.044	1.027	5.463E-01	logistic	412469	1130	411339	1.000	0.337
446.9	Arteritis NOS	circulatory system	rs10852766	0.047	0.078	1.048	5.464E-01	logistic	405830	361	405469	1.000	0.337
368.4	Visual field defects	sense organs	rs10852766	0.042	0.070	1.043	5.506E-01	logistic	415988	443	415545	0.999	0.337
585.33	Chronic Kidney Disease, Stage III	genitourinary	rs10852766	-0.011	0.018	0.990	5.507E-01	logistic	388537	7475	381062	0.999	0.337
242.1	Graves' disease	endocrine/metabolic	rs10852766	-0.047	0.079	0.954	5.517E-01	logistic	392598	368	392230	0.999	0.336
427.2	Atrial fibrillation and flutter	circulatory system	rs10852766	0.007	0.012	1.007	5.526E-01	logistic	388316	16468	371848	1.000	0.337
285.2	Anemia of chronic disease	hematopoietic	rs10852766	0.031	0.053	1.032	5.575E-01	logistic	383237	788	382449	0.999	0.337
506	Empyema and pneumothorax	respiratory	rs10852766	0.025	0.043	1.026	5.580E-01	logistic	392231	1174	391057	0.999	0.337
352.2	Facial nerve disorders [CN7]	neurological	rs10852766	0.033	0.057	1.034	5.582E-01	logistic	403092	677	402415	1.000	0.337
727.4	Ganglion and cyst of synovium, tendon, and bursa	musculoskeletal	rs10852766	0.040	0.069	1.041	5.593E-01	logistic	389641	467	389174	0.999	0.337
447.1	Stricture of artery	circulatory system	rs10852766	-0.035	0.061	0.965	5.597E-01	logistic	406083	614	405469	1.000	0.337
580.2	Nephrotic syndrome without mention of glomerulonephritis	genitourinary	rs10852766	0.043	0.075	1.044	5.605E-01	logistic	381457	395	381062	0.998	0.337
420	Carditis	circulatory system	rs10852766	0.019	0.032	1.019	5.619E-01	logistic	418942	2172	416770	1.000	0.337
709	Diffuse diseases of connective tissue	dermatologic	rs10852766	-0.026	0.045	0.975	5.633E-01	logistic	405028	1129	403899	1.000	0.336
368.9	Subjective visual disturbances	sense organs	rs10852766	0.038	0.066	1.039	5.635E-01	logistic	416057	512	415545	0.999	0.337
282	Hereditary hemolytic anemias	hematopoietic	rs10852766	-0.059	0.102	0.943	5.644E-01	logistic	382667	218	382449	0.999	0.337
599.3	Dysuria	genitourinary	rs10852766	0.047	0.081	1.048	5.649E-01	logistic	390438	332	390106	0.999	0.336
504	Other alveolar and parietoalveolar pneumonopathy	respiratory	rs10852766	-0.034	0.058	0.967	5.658E-01	logistic	391718	661	391057	1.000	0.337
8.5	Bacterial enteritis	infectious diseases	rs10852766	-0.024	0.042	0.976	5.661E-01	logistic	418567	1251	417316	1.000	0.337
426.32	Left bundle branch block	circulatory system	rs10852766	-0.016	0.027	0.985	5.663E-01	logistic	374914	3066	371848	1.000	0.337
368.91	Psychophysical visual disturbances	sense organs	rs10852766	0.052	0.091	1.054	5.664E-01	logistic	415808	263	415545	0.999	0.337
458.1	Orthostatic hypotension	circulatory system	rs10852766	0.015	0.027	1.015	5.668E-01	logistic	375723	3151	372572	0.995	0.336
367.8	Hypermetropia	sense organs	rs10852766	0.051	0.089	1.052	5.689E-01	logistic	411285	279	411006	0.999	0.337
172.1	Melanomas of skin, dx or hx	neoplasms	rs10852766	-0.020	0.035	0.980	5.706E-01	logistic	409038	1830	407208	1.000	0.337

ST31; PheWAS results for the lead cis-pQTLs of the 4 proteins prioritized (ACOX1, FGF5, FURIN, and MST1).
SNP = Single Nucleotide Polymorphism; SE = Standard error HWE_p = P-value for Hardy-Weinberg equilibrium test

Phenotype	Description	Group	SNP	Beta	SE	Odds tatio	P-value	Type	n_total	n_cases	n_controls	HWE_p	allele_freq
172.11	Melanomas of skin	neoplasms	rs10852766	-0.020	0.035	0.980	5.706E-01	logistic	409038	1830	407208	1.000	0.337
702	Degenerative skin conditions and other dermatoses	dermatologic	rs10852766	-0.020	0.035	0.981	5.735E-01	logistic	407215	1867	405348	1.000	0.336
572	Ascites (non malignant)	digestive	rs10852766	0.018	0.032	1.018	5.748E-01	logistic	402409	2135	400274	0.998	0.336
38	Septicemia	infectious diseases	rs10852766	-0.010	0.018	0.990	5.828E-01	logistic	403876	7229	396647	0.999	0.336
365.2	Primary angle-closure glaucoma	sense organs	rs10852766	-0.031	0.058	0.969	5.871E-01	logistic	398441	678	397763	0.999	0.337
296.22	Major depressive disorder	mental disorders	rs10852766	0.007	0.012	1.007	5.874E-01	logistic	396820	15198	381622	0.999	0.337
747.1	Cardiac congenital anomalies	congenital anomalies	rs10852766	0.026	0.049	1.027	5.876E-01	logistic	422536	941	421595	1.000	0.337
636.3	Hemorrhage in early pregnancy	pregnancy complications	rs10852766	0.054	0.101	1.056	5.911E-01	logistic	222810	218	222592	0.999	0.336
741.3	Difficulty in walking	musculoskeletal	rs10852766	-0.037	0.070	0.963	5.916E-01	logistic	415474	464	415010	1.000	0.337
586.11	Small kidney	genitourinary	rs10852766	0.054	0.100	1.055	5.919E-01	logistic	381281	219	381062	0.999	0.337
379.2	Disorders of vitreous body	sense organs	rs10852766	-0.040	0.075	0.961	5.954E-01	logistic	412333	405	411928	0.999	0.337
255	Disorders of adrenal glands	endocrine/metabolic	rs10852766	0.028	0.053	1.028	5.971E-01	logistic	416034	788	415246	1.000	0.337
750	Digestive congenital anomalies	congenital anomalies	rs10852766	0.038	0.072	1.039	5.976E-01	logistic	420674	421	420253	1.000	0.337
625	Pain and other symptoms associated with female genital organs	genitourinary	rs10852766	-0.047	0.088	0.954	5.981E-01	logistic	218575	291	218284	0.999	0.336
359	Muscular dystrophies and other myopathies	neurological	rs10852766	0.047	0.090	1.048	6.013E-01	logistic	419442	274	419168	0.999	0.337
687.4	Disturbance of skin sensation	dermatologic	rs10852766	-0.021	0.040	0.979	6.016E-01	logistic	414113	1397	412716	1.000	0.337
292.6	Hallucinations	mental disorders	rs10852766	-0.042	0.081	0.959	6.021E-01	logistic	405317	344	404973	1.000	0.337
78	Viral warts & HPV	infectious diseases	rs10852766	0.052	0.100	1.053	6.044E-01	logistic	415426	222	415204	1.000	0.337
293.1	Swelling, mass, or lump in head and neck [Space-occupying lesion, intracranial NOS	mental disorders	rs10852766	-0.044	0.085	0.957	6.049E-01	logistic	420026	315	419711	1.000	0.337
342	Hemiplegia	neurological	rs10852766	-0.016	0.031	0.984	6.053E-01	logistic	401449	2402	399047	1.000	0.337
501	Pneumonitis due to inhalation of food or vomitus	respiratory	rs10852766	0.018	0.035	1.018	6.060E-01	logistic	392843	1786	391057	1.000	0.337
800	Fracture of lower limb	injuries & poisonings	rs10852766	-0.014	0.027	0.986	6.079E-01	logistic	399482	3104	396378	0.999	0.336
724	Other and unspecified disorders of back	musculoskeletal	rs10852766	0.033	0.064	1.033	6.087E-01	logistic	409212	542	408670	1.000	0.337
575	Other biliary tract disease	digestive	rs10852766	0.013	0.025	1.013	6.103E-01	logistic	398734	3687	395047	0.999	0.337
508	Pulmonary collapse; interstitial and compensatory emphysema	respiratory	rs10852766	0.012	0.024	1.012	6.107E-01	logistic	394890	3833	391057	0.999	0.337
622.2	Mucous polyp of cervix	genitourinary	rs10852766	-0.050	0.098	0.952	6.110E-01	logistic	213810	238	213572	0.997	0.336
697	Sarcoidosis	dermatologic	rs10852766	-0.031	0.062	0.969	6.111E-01	logistic	405795	589	405206	1.000	0.336
389.1	Sensorineural hearing loss	sense organs	rs10852766	-0.027	0.054	0.973	6.124E-01	logistic	409063	766	408297	1.000	0.336
735	Acquired foot deformities	musculoskeletal	rs10852766	0.010	0.019	1.010	6.183E-01	logistic	408734	6080	402654	0.999	0.337
292.1	Aphasia/speech disturbance	mental disorders	rs10852766	0.023	0.046	1.023	6.219E-01	logistic	406020	1047	404973	1.000	0.337
580	Nephritis; nephrosis; renal sclerosis	genitourinary	rs10852766	0.022	0.045	1.023	6.241E-01	logistic	382137	1075	381062	0.998	0.337
214	Lipoma	neoplasms	rs10852766	0.022	0.045	1.022	6.242E-01	logistic	415339	1112	414227	1.000	0.337
442.1	Aortic aneurysm	circulatory system	rs10852766	-0.015	0.030	0.985	6.265E-01	logistic	407951	2482	405469	1.000	0.337
741.4	Joint effusions	musculoskeletal	rs10852766	0.028	0.057	1.028	6.265E-01	logistic	415693	683	415010	1.000	0.337
362.29	Macular degeneration (senile) of retina NOS	sense organs	rs10852766	0.012	0.024	1.012	6.290E-01	logistic	401665	3908	397757	1.000	0.337
471	Nasal polyps	respiratory	rs10852766	0.022	0.046	1.023	6.309E-01	logistic	397549	1032	396517	1.000	0.336
580.1	Glomerulonephritis	genitourinary	rs10852766	0.032	0.066	1.032	6.313E-01	logistic	381574	512	381062	0.998	0.337
110	Dermatophytosis / Dermatomycosis	infectious diseases	rs10852766	-0.047	0.098	0.954	6.317E-01	logistic	418617	237	418380	1.000	0.337
276.6	Fluid overload	endocrine/metabolic	rs10852766	0.018	0.037	1.018	6.324E-01	logistic	397239	1667	395572	0.999	0.337
586.1	Anatomical abnormatilies of kidney and ureters	genitourinary	rs10852766	0.046	0.096	1.047	6.325E-01	logistic	381301	239	381062	0.999	0.337
726.3	Bursitis	musculoskeletal	rs10852766	-0.050	0.105	0.951	6.333E-01	logistic	389380	206	389174	0.999	0.337
577.1	Acute pancreatitis	digestive	rs10852766	0.016	0.035	1.017	6.359E-01	logistic	419858	1850	418008	1.000	0.337
756	Other congenital musculoskeletal anomalies	congenital anomalies	rs10852766	-0.023	0.048	0.978	6.371E-01	logistic	422256	974	421282	1.000	0.337
155	Cancer of liver and intrahepatic bile duct	neoplasms	rs10852766	0.026	0.055	1.026	6.381E-01	logistic	394604	742	393862	0.999	0.337
274.2	Crystal arthropathies	endocrine/metabolic	rs10852766	0.045	0.095	1.046	6.382E-01	logistic	416033	243	415790	1.000	0.337
574.12	Cholelithiasis with other cholecystitis	digestive	rs10852766	-0.018	0.038	0.983	6.405E-01	logistic	396636	1589	395047	0.999	0.337
510	Other diseases of lung	respiratory	rs10852766	0.018	0.039	1.019	6.411E-01	logistic	421711	1427	420284	1.000	0.337
690.1	Seborrheic dermatitis	dermatologic	rs10852766	-0.028	0.061	0.972	6.427E-01	logistic	405818	612	405206	1.000	0.336
585.31	Renal dialysis	genitourinary	rs10852766	-0.026	0.056	0.974	6.433E-01	logistic	381775	713	381062	0.998	0.337
401.22	Hypertensive chronic kidney disease	circulatory system	rs10852766	-0.022	0.048	0.978	6.437E-01	logistic	291632	969	290663	0.976	0.337
763	Thoracic or lumbosacral neuritis or radiculitis, unspecified	symptoms	rs10852766	-0.041	0.089	0.960	6.440E-01	logistic	418269	283	417986	1.000	0.337
300.9	Posttraumatic stress disorder	mental disorders	rs10852766	-0.043	0.094	0.958	6.468E-01	logistic	381881	259	381622	0.999	0.337
170	Cancer of bone and connective tissue	neoplasms	rs10852766	0.036	0.078	1.037	6.470E-01	logistic	423460	359	423101	1.000	0.337
427.22	Atrial flutter	circulatory system	rs10852766	0.027	0.058	1.027	6.477E-01	logistic	372499	651	371848	1.000	0.337
695.9	Unspecified erythematous condition	dermatologic	rs10852766	0.038	0.083	1.039	6.490E-01	logistic	405524	318	405206	1.000	0.336
801	Fracture of ankle and foot	injuries & poisonings	rs10852766	0.026	0.058	1.027	6.492E-01	logistic	397040	662	396378	1.000	0.337
550.5	Ventral hernia	digestive	rs10852766	-0.017	0.037	0.983	6.494E-01	logistic	367031	1637	365394	0.995	0.336
414	Other forms of chronic heart disease	circulatory system	rs10852766	0.011	0.023	1.011	6.519E-01	logistic	374670	4120	370550	0.998	0.337
348.2	Cerebral edema and compression of brain	neurological	rs10852766	0.034	0.076	1.035	6.558E-01	logistic	399425	378	399047	1.000	0.337
509	Respiratory failure, insufficiency, arrest	respiratory	rs10852766	0.009	0.019	1.009	6.558E-01	logistic	397038	5981	391057	0.999	0.337
327.3	Sleep apnea	neurological	rs10852766	-0.009	0.021	0.991	6.564E-01	logistic	418773	5389	413384	1.000	0.337
371.3	Inflammation of eyelids	sense organs	rs10852766	-0.026	0.058	0.975	6.572E-01	logistic	411465	672	410793	1.000	0.337
819	Skull and face fracture and other intercranial injury	injuries & poisonings	rs10852766	-0.030	0.067	0.971	6.579E-01	logistic	422666	501	422165	1.000	0.337
599.2	Retention of urine	genitourinary	rs10852766	-0.008	0.018	0.992	6.615E-01	logistic	396988	6882	390106	0.999	0.336
728	Disorders of muscle, ligament, and fascia	musculoskeletal	rs10852766	-0.041	0.094	0.960	6.650E-01	logistic	389432	258	389174	0.999	0.337
574.1	Cholelithiasis	digestive	rs10852766	0.007	0.016	1.007	6.674E-01	logistic	404312	9265	395047	0.999	0.337
530.1	Esophagitis, GERD and related diseases	digestive	rs10852766	-0.004	0.009	0.996	6.683E-01	logistic	385890	29102	356788	1.000	0.336
53	Herpes zoster	infectious diseases	rs10852766	0.034	0.078	1.034	6.688E-01	logistic	415563	359	415204	1.000	0.337
634.1	Missed abortion/Hydatidiform mole	pregnancy complications	rs10852766	0.043	0.102	1.044	6.723E-01	logistic	222807	215	222592	0.999	0.336
702.1	Actinic keratosis	dermatologic	rs10852766	-0.021	0.049	0.979	6.726E-01	logistic	413997	925	413072	1.000	0.337
760	Back pain	symptoms	rs10852766	0.009	0.021	1.009	6.772E-01	logistic	414925	5193	409732	1.000	0.337

ST31; PheWAS results for the lead cis-pQTLs of the 4 proteins prioritized (ACOX1, FGF5, FURIN, and MST1).

SNP = Single Nucleotide Polymorphism; SE = Standard error HWE_p = P-value for Hardy-Weinberg equilibrium test

Phenotype	Description	Group	SNP	Beta	SE	Odds tatio	P-value	Type	n_total	n_cases	n_controls	HWE_p	allele_freq
367.9	Blindness and low vision	sense organs	rs10852766	-0.021	0.051	0.979	6.779E-01	logistic	411881	875	411006	0.999	0.337
618	Genital prolapse	genitourinary	rs10852766	0.008	0.019	1.008	6.796E-01	logistic	219680	6225	213455	0.999	0.336
626.14	Irregular menstrual bleeding	genitourinary	rs10852766	0.031	0.075	1.031	6.808E-01	logistic	193146	399	192747	0.993	0.337
442.11	Abdominal aortic aneurysm	circulatory system	rs10852766	-0.015	0.037	0.985	6.837E-01	logistic	407127	1658	405469	1.000	0.337
289.5	Diseases of spleen	hematopoietic	rs10852766	-0.022	0.054	0.978	6.844E-01	logistic	409070	761	408309	1.000	0.337
442.8	Aneurysm of other specified artery	circulatory system	rs10852766	0.037	0.092	1.038	6.847E-01	logistic	405731	262	405469	1.000	0.337
196	Radiotherapy	neoplasms	rs10852766	-0.015	0.038	0.985	6.878E-01	logistic	387589	1583	386006	0.999	0.337
185	Cancer of prostate	neoplasms	rs10852766	0.006	0.015	1.006	6.890E-01	logistic	167540	11079	156461	0.917	0.337
411	Ischemic Heart Disease	circulatory system	rs10852766	0.003	0.008	1.003	6.904E-01	logistic	410606	40056	370550	1.000	0.337
604	Disorders of penis	genitourinary	rs10852766	-0.027	0.068	0.973	6.905E-01	logistic	162266	488	161778	0.930	0.337
286.7	Other and unspecified coagulation defects	hematopoietic	rs10852766	0.036	0.091	1.037	6.917E-01	logistic	417818	265	417553	1.000	0.337
197	Chemotherapy	neoplasms	rs10852766	-0.006	0.014	0.994	6.934E-01	logistic	397532	11526	386006	0.999	0.337
291.8	Alteration of consciousness	mental disorders	rs10852766	-0.031	0.078	0.970	6.950E-01	logistic	405343	370	404973	1.000	0.337
189	Cancer of urinary organs (incl. kidney and bladder)	neoplasms	rs10852766	0.009	0.022	1.009	6.962E-01	logistic	421760	4573	417187	1.000	0.337
41.2	Streptococcus infection	infectious diseases	rs10852766	-0.015	0.039	0.985	6.968E-01	logistic	398155	1508	396647	1.000	0.336
722.9	Other and unspecified disc disorder	musculoskeletal	rs10852766	0.025	0.065	1.025	6.988E-01	logistic	409194	524	408670	1.000	0.337
740.2	Osteoarthrosis, generalized	musculoskeletal	rs10852766	0.009	0.022	1.009	6.992E-01	logistic	350458	4600	345858	1.000	0.337
690	Erythematosquamous dermatosis	dermatologic	rs10852766	-0.023	0.060	0.977	7.020E-01	logistic	405831	625	405206	1.000	0.336
337	Disorders of the autonomic nervous system	neurological	rs10852766	0.030	0.080	1.031	7.026E-01	logistic	399396	349	399047	1.000	0.337
365.1	Open-angle glaucoma	sense organs	rs10852766	0.014	0.038	1.014	7.035E-01	logistic	399325	1562	397763	0.999	0.337
211	Benign neoplasm of other parts of digestive system	neoplasms	rs10852766	-0.010	0.026	0.990	7.054E-01	logistic	400517	3467	397050	0.999	0.337
586.4	Stricture/obstruction of ureter	genitourinary	rs10852766	-0.020	0.052	0.981	7.058E-01	logistic	381896	834	381062	0.999	0.337
635.2	Antepartum hemorrhage, abruptio placentae, and placenta previa	pregnancy complications	rs10852766	-0.040	0.106	0.961	7.064E-01	logistic	222797	205	222592	0.999	0.336
736	Other acquired deformities of limbs	musculoskeletal	rs10852766	-0.018	0.049	0.982	7.068E-01	logistic	403605	951	402654	0.999	0.337
345.1	Epilepsy	neurological	rs10852766	0.023	0.063	1.024	7.119E-01	logistic	399597	550	399047	1.000	0.337
575.9	Nonspecific abnormal findings on radiological and other examination of biliary trac	digestive	rs10852766	-0.037	0.099	0.964	7.124E-01	logistic	395275	228	395047	0.999	0.337
411.2	Myocardial infarction	circulatory system	rs10852766	-0.005	0.013	0.995	7.126E-01	logistic	385085	14535	370550	0.999	0.337
751.22	Other specified congenital anomalies of kidney	congenital anomalies	rs10852766	0.029	0.080	1.030	7.132E-01	logistic	420601	348	420253	1.000	0.337
530.3	Stricture and stenosis of esophagus	digestive	rs10852766	-0.016	0.044	0.984	7.167E-01	logistic	357976	1188	356788	1.000	0.337
626	Disorders of menstruation and other abnormal bleeding from female genital trac	genitourinary	rs10852766	-0.007	0.020	0.993	7.172E-01	logistic	198823	6076	192747	0.990	0.337
858	Complication of internal orthopedic device	injuries & poisonings	rs10852766	0.015	0.042	1.015	7.216E-01	logistic	417889	1237	416652	1.000	0.337
531.2	Gastric ulcer	digestive	rs10852766	0.013	0.036	1.013	7.222E-01	logistic	413012	1673	411339	1.000	0.337
619	Noninflammatory female genital disorders	genitourinary	rs10852766	0.011	0.032	1.011	7.225E-01	logistic	219175	2209	216966	0.998	0.336
175	Acquired absence of breast	neoplasms	rs10852766	0.008	0.024	1.008	7.240E-01	logistic	206502	3992	202510	0.994	0.337
351	Other peripheral nerve disorders	neurological	rs10852766	-0.006	0.018	0.994	7.243E-01	logistic	409126	6711	402415	1.000	0.337
747.11	Cardiac shunt/ heart septal defect	congenital anomalies	rs10852766	-0.025	0.071	0.975	7.261E-01	logistic	422041	446	421595	1.000	0.337
8.6	Viral Enteritis	infectious diseases	rs10852766	-0.023	0.067	0.977	7.276E-01	logistic	417821	505	417316	0.999	0.337
416	Cardiomegaly	circulatory system	rs10852766	0.008	0.023	1.008	7.288E-01	logistic	417255	4417	412838	1.000	0.337
394.1	Mitral valve stenosis and aortic valve stenosis	circulatory system	rs10852766	-0.015	0.045	0.985	7.309E-01	logistic	402530	1116	401414	0.999	0.336
253.7	Other disorders of neurohypophysis	endocrine/metabolic	rs10852766	0.027	0.080	1.028	7.310E-01	logistic	415594	348	415246	1.000	0.337
378.2	Nystagmus and other irregular eye movements	sense organs	rs10852766	-0.036	0.104	0.965	7.316E-01	logistic	412137	209	411928	0.999	0.337
947	Urticaria	dermatologic	rs10852766	-0.036	0.104	0.965	7.324E-01	logistic	415196	209	414987	1.000	0.337
608	Other disorders of male genital organs	genitourinary	rs10852766	0.018	0.054	1.018	7.370E-01	logistic	162533	755	161778	0.950	0.337
218	Benign neoplasm of uterus	neoplasms	rs10852766	-0.009	0.028	0.991	7.374E-01	logistic	209861	2866	206995	0.998	0.336
747	Cardiac and circulatory congenital anomalies	congenital anomalies	rs10852766	0.015	0.045	1.015	7.375E-01	logistic	422691	1096	421595	1.000	0.337
225	Benign neoplasm of brain and other parts of nervous system	neoplasms	rs10852766	-0.017	0.052	0.983	7.388E-01	logistic	421233	836	420397	1.000	0.337
726.1	Enthesopathy	musculoskeletal	rs10852766	0.009	0.026	1.009	7.403E-01	logistic	392491	3317	389174	0.999	0.337
202.21	Nodular lymphoma	neoplasms	rs10852766	-0.023	0.070	0.977	7.416E-01	logistic	416077	464	415613	1.000	0.337
509.2	Respiratory insufficiency	respiratory	rs10852766	0.013	0.041	1.013	7.424E-01	logistic	392402	1345	391057	1.000	0.337
270.33	Amyloidosis	endocrine/metabolic	rs10852766	-0.027	0.083	0.973	7.435E-01	logistic	422032	330	421702	1.000	0.337
447	Other disorders of arteries and arterioles	circulatory system	rs10852766	0.015	0.045	1.015	7.444E-01	logistic	406558	1089	405469	1.000	0.337
365	Glaucoma	sense organs	rs10852766	0.006	0.018	1.006	7.450E-01	logistic	405209	7446	397763	0.999	0.337
292.4	Altered mental status	mental disorders	rs10852766	0.008	0.026	1.008	7.459E-01	logistic	408434	3461	404973	1.000	0.337
613	Other nonmalignant breast conditions	genitourinary	rs10852766	0.024	0.075	1.024	7.476E-01	logistic	421594	397	421197	1.000	0.337
599	Other symptoms/disorders or the urinary system	genitourinary	rs10852766	0.005	0.017	1.005	7.483E-01	logistic	398281	8175	390106	0.999	0.336
430.1	Subarachnoid hemorrhage	circulatory system	rs10852766	-0.018	0.055	0.983	7.505E-01	logistic	403416	731	402685	0.998	0.337
8.52	Intestinal infection due to C. difficile	infectious diseases	rs10852766	-0.018	0.058	0.982	7.521E-01	logistic	417984	668	417316	0.999	0.337
556	Ulceration of the lower GI tract	digestive	rs10852766	0.024	0.075	1.024	7.528E-01	logistic	334879	394	334485	0.998	0.337
535.6	Duodenitis	digestive	rs10852766	0.011	0.035	1.011	7.538E-01	logistic	363993	1779	362214	0.999	0.336
374.3	Ptosis of eyelid	sense organs	rs10852766	0.013	0.043	1.014	7.551E-01	logistic	411997	1204	410793	1.000	0.337
523	Gingival and periodontal diseases	digestive	rs10852766	0.023	0.075	1.023	7.573E-01	logistic	409931	395	409536	1.000	0.337
317.1	Alcoholism	mental disorders	rs10852766	0.008	0.024	1.008	7.578E-01	logistic	407082	3789	403293	1.000	0.337
425.1	Primary/intrinsic cardiomyopathies	circulatory system	rs10852766	-0.011	0.035	0.989	7.588E-01	logistic	418636	1866	416770	1.000	0.337
528	Diseases of the oral soft tissues, excluding lesions specific for gingiva and tongue	digestive	rs10852766	0.021	0.069	1.021	7.609E-01	logistic	418169	459	417710	1.000	0.337
353	Nerve root and plexus disorders	neurological	rs10852766	0.026	0.085	1.026	7.609E-01	logistic	402720	305	402415	1.000	0.337
527	Diseases of the salivary glands	digestive	rs10852766	-0.031	0.101	0.970	7.610E-01	logistic	417933	223	417710	1.000	0.337
70.3	Viral hepatitis C	infectious diseases	rs10852766	0.028	0.091	1.028	7.618E-01	logistic	415468	264	415204	1.000	0.337
426.3	Bundle branch block	circulatory system	rs10852766	-0.008	0.025	0.992	7.622E-01	logistic	375377	3529	371848	1.000	0.337
550.2	Diaphragmatic hernia	digestive	rs10852766	0.003	0.010	1.003	7.651E-01	logistic	388258	22864	365394	0.994	0.336
401.2	Hypertensive heart and/or renal disease	circulatory system	rs10852766	-0.013	0.044	0.987	7.676E-01	logistic	291807	1144	290663	0.976	0.337
595	Hydronephrosis	genitourinary	rs10852766	-0.014	0.048	0.986	7.678E-01	logistic	413362	986	412376	0.999	0.337

ST31; PheWAS results for the lead cis-pQTLs of the 4 proteins prioritized (ACOX1, FGF5, FURIN, and MST1).

SNP = Single Nucleotide Polymorphism; SE = Standard error HWE_p = P-value for Hardy-Weinberg equilibrium test

Phenotype	Description	Group	SNP	Beta	SE	Odds tatio	P-value	Type	n_total	n_cases	n_controls	HWE_p	allele_freq
615	Endometriosis	genitourinary	rs10852766	0.013	0.043	1.013	7.679E-01	logistic	218669	1231	217438	0.997	0.336
571.5	Other chronic nonalcoholic liver disease	digestive	rs10852766	0.007	0.023	1.007	7.693E-01	logistic	404607	4333	400274	0.999	0.336
426.2	Atrioventricular [AV] block	circulatory system	rs10852766	0.007	0.025	1.007	7.718E-01	logistic	375580	3732	371848	1.000	0.337
456	Chronic venous insufficiency [CVI]	circulatory system	rs10852766	0.026	0.088	1.026	7.726E-01	logistic	381250	284	380966	0.998	0.336
801.1	Fracture of foot	injuries & poisonings	rs10852766	-0.029	0.102	0.971	7.730E-01	logistic	396597	219	396378	1.000	0.337
574.3	Cholecystitis without cholelithiasis	digestive	rs10852766	0.012	0.043	1.013	7.732E-01	logistic	396244	1197	395047	0.999	0.337
804	Fracture of hand or wrist	injuries & poisonings	rs10852766	0.018	0.062	1.018	7.734E-01	logistic	396955	577	396378	1.000	0.337
395.4	Nonrheumatic pulmonary valve disorders	circulatory system	rs10852766	0.024	0.083	1.024	7.743E-01	logistic	401735	321	401414	0.999	0.336
145.2	Cancer of tongue	neoplasms	rs10852766	0.022	0.075	1.022	7.746E-01	logistic	420733	391	420342	0.999	0.337
300.12	Agorophobia, social phobia, and panic disorder	mental disorders	rs10852766	0.017	0.058	1.017	7.752E-01	logistic	382286	664	381622	0.999	0.337
514	Abnormal findings examination of lungs	respiratory	rs10852766	0.009	0.032	1.009	7.754E-01	logistic	419804	2147	417657	1.000	0.337
521	Diseases of hard tissues of teeth	digestive	rs10852766	0.015	0.053	1.015	7.761E-01	logistic	410337	801	409536	1.000	0.337
250.2	Type 2 diabetes	endocrine/metabolic	rs10852766	0.003	0.010	1.003	7.779E-01	logistic	413206	26569	386637	1.000	0.337
451.2	Phlebitis and thrombophlebitis of lower extremities	circulatory system	rs10852766	-0.019	0.068	0.981	7.788E-01	logistic	381453	487	380966	0.999	0.336
443	Peripheral vascular disease	circulatory system	rs10852766	-0.006	0.021	0.994	7.790E-01	logistic	410595	5126	405469	1.000	0.337
726	Peripheral enthesopathies and allied syndromes	musculoskeletal	rs10852766	-0.004	0.015	0.996	7.790E-01	logistic	399760	10586	389174	1.000	0.337
227	Benign neoplasm of other endocrine glands and related structures	neoplasms	rs10852766	0.016	0.056	1.016	7.791E-01	logistic	421596	709	420887	1.000	0.337
601.12	Chronic prostatitis	genitourinary	rs10852766	0.027	0.097	1.027	7.814E-01	logistic	156694	233	156461	0.895	0.337
681.1	Cellulitis and abscess of fingers/toes	dermatologic	rs10852766	-0.028	0.100	0.973	7.817E-01	logistic	418724	227	418497	1.000	0.337
285.22	Anemia in neoplastic disease	hematopoietic	rs10852766	0.023	0.082	1.023	7.823E-01	logistic	382780	331	382449	0.999	0.337
714	Rheumatoid arthritis and other inflammatory polyarthropathies	musculoskeletal	rs10852766	-0.006	0.024	0.994	7.844E-01	logistic	412574	4067	408507	1.000	0.337
250	Diabetes mellitus	endocrine/metabolic	rs10852766	0.003	0.009	1.003	7.847E-01	logistic	414239	27602	386637	1.000	0.337
585.2	Renal failure NOS	genitourinary	rs10852766	-0.015	0.055	0.985	7.857E-01	logistic	381806	744	381062	0.999	0.337
159	Malignant neoplasm of other and ill-defined sites within the digestive organs and peritoneum	neoplasms	rs10852766	-0.014	0.051	0.986	7.878E-01	logistic	394736	874	393862	0.999	0.337
331	Other cerebral degenerations	neurological	rs10852766	0.010	0.037	1.010	7.897E-01	logistic	400699	1652	399047	1.000	0.337
317	Alcohol-related disorders	mental disorders	rs10852766	0.006	0.021	1.006	7.906E-01	logistic	408346	5053	403293	1.000	0.337
371	Inflammation of the eye	sense organs	rs10852766	0.012	0.046	1.012	7.913E-01	logistic	411823	1030	410793	1.000	0.337
189.21	Malignant neoplasm of bladder	neoplasms	rs10852766	0.007	0.027	1.007	7.920E-01	logistic	420210	3023	417187	1.000	0.337
528.1	Stomatitis and mucositis	digestive	rs10852766	0.025	0.097	1.026	7.922E-01	logistic	417947	237	417710	1.000	0.337
785	Abdominal pain	symptoms	rs10852766	-0.005	0.018	0.995	7.924E-01	logistic	401183	7225	393958	0.999	0.336
916	Contusion	injuries & poisonings	rs10852766	-0.010	0.040	0.990	7.950E-01	logistic	421588	1393	420195	1.000	0.337
284	Aplastic anemia	hematopoietic	rs10852766	0.014	0.055	1.014	7.958E-01	logistic	383179	730	382449	1.000	0.337
279	Disorders involving the immune mechanism	endocrine/metabolic	rs10852766	0.019	0.077	1.020	8.009E-01	logistic	423247	371	422876	1.000	0.337
528.11	Stomatitis and mucositis (ulcerative)	digestive	rs10852766	0.026	0.103	1.026	8.016E-01	logistic	417919	209	417710	1.000	0.337
579	Other symptoms involving abdomen and pelvis	digestive	rs10852766	-0.012	0.047	0.988	8.030E-01	logistic	384340	1037	383303	0.999	0.336
590	Pyelonephritis	genitourinary	rs10852766	0.010	0.040	1.010	8.035E-01	logistic	378294	1367	376927	0.999	0.336
345	Epilepsy, recurrent seizures, convulsions	neurological	rs10852766	0.006	0.023	1.006	8.036E-01	logistic	403151	4104	399047	1.000	0.337
454.1	Varicose veins of lower extremity	circulatory system	rs10852766	-0.006	0.022	0.994	8.045E-01	logistic	385486	4520	380966	0.999	0.336
189.4	Malignant neoplasm of other urinary organs	neoplasms	rs10852766	0.026	0.104	1.026	8.055E-01	logistic	417393	206	417187	1.000	0.337
427.9	Palpitations	circulatory system	rs10852766	-0.009	0.036	0.991	8.057E-01	logistic	373589	1741	371848	1.000	0.337
530.9	Heartburn	digestive	rs10852766	0.023	0.094	1.023	8.066E-01	logistic	357038	250	356788	0.999	0.337
585.32	End stage renal disease	genitourinary	rs10852766	0.012	0.049	1.012	8.076E-01	logistic	381994	932	381062	0.997	0.337
191	Manlignant and unknown neoplasms of brain and nervous system	neoplasms	rs10852766	0.012	0.048	1.012	8.098E-01	logistic	421363	966	420397	1.000	0.337
289.8	Polycythemia, secondary	hematopoietic	rs10852766	-0.017	0.069	0.984	8.111E-01	logistic	407605	466	407139	0.999	0.337
261.2	Vitamin B-complex deficiencies	endocrine/metabolic	rs10852766	0.008	0.036	1.008	8.156E-01	logistic	413809	1712	412097	1.000	0.336
740.11	Osteoarthrosis, localized, primary	musculoskeletal	rs10852766	0.005	0.021	1.005	8.156E-01	logistic	351108	5250	345858	1.000	0.337
735.2	Acquired toe deformities	musculoskeletal	rs10852766	0.008	0.034	1.008	8.159E-01	logistic	404574	1920	402654	0.999	0.337
477	Epistaxis or throat hemorrhage	respiratory	rs10852766	0.010	0.042	1.010	8.176E-01	logistic	397807	1290	396517	1.000	0.336
145	Cancer of mouth	neoplasms	rs10852766	0.013	0.057	1.013	8.193E-01	logistic	421016	674	420342	0.999	0.337
180.3	Cervical intraepithelial neoplasia [CIN] [Cervical dysplasia	neoplasms	rs10852766	0.015	0.068	1.016	8.202E-01	logistic	196385	481	195904	0.993	0.337
184.2	Cancer of other female genital organs (excluding uterus and ovary	neoplasms	rs10852766	-0.021	0.096	0.979	8.255E-01	logistic	205080	243	204837	0.991	0.336
333	Extrapyramidal disease and abnormal movement disorders	neurological	rs10852766	0.009	0.043	1.009	8.271E-01	logistic	400270	1223	399047	1.000	0.337
594.8	Renal colic	genitourinary	rs10852766	0.014	0.064	1.014	8.297E-01	logistic	412927	551	412376	0.999	0.337
38.2	Gram positive septicemia	infectious diseases	rs10852766	-0.013	0.060	0.987	8.299E-01	logistic	397268	621	396647	1.000	0.336
225.1	Benign neoplasm of brain, cranial nerves, meninges	neoplasms	rs10852766	0.011	0.052	1.011	8.302E-01	logistic	421204	807	420397	1.000	0.337
324	Other CNS infection and poliomyelitis	neurological	rs10852766	-0.017	0.080	0.983	8.313E-01	logistic	422418	347	422071	1.000	0.337
619.2	Disorders of uterus, NEC	genitourinary	rs10852766	0.015	0.073	1.016	8.338E-01	logistic	217379	413	216966	0.998	0.336
479	Other upper respiratory disease	respiratory	rs10852766	-0.015	0.073	0.985	8.361E-01	logistic	396937	420	396517	1.000	0.336
279.1	Immunity deficiency	endocrine/metabolic	rs10852766	-0.017	0.083	0.983	8.367E-01	logistic	423199	323	422876	1.000	0.337
800.2	Fracture of unspecified part of femur	injuries & poisonings	rs10852766	-0.021	0.102	0.979	8.373E-01	logistic	396592	214	396378	1.000	0.337
288	Diseases of white blood cells	hematopoietic	rs10852766	-0.015	0.074	0.985	8.373E-01	logistic	408723	414	408309	1.000	0.337
709.2	Sicca syndrome	dermatologic	rs10852766	0.011	0.055	1.011	8.374E-01	logistic	404638	739	403899	1.000	0.336
253	Disorders of the pituitary gland and its hypothalamic contro	endocrine/metabolic	rs10852766	-0.010	0.050	0.990	8.379E-01	logistic	416136	890	415246	1.000	0.337
276.14	Hypopotassemia	endocrine/metabolic	rs10852766	0.005	0.025	1.005	8.389E-01	logistic	399074	3502	395572	1.000	0.337
153.3	Malignant neoplasm of rectum, rectosigmoid junction, and anus	neoplasms	rs10852766	0.006	0.028	1.006	8.427E-01	logistic	373609	2889	370720	0.995	0.337
522.5	Periapical abscess	digestive	rs10852766	0.020	0.103	1.021	8.435E-01	logistic	409744	208	409536	1.000	0.337
8	Intestinal infection	infectious diseases	rs10852766	-0.007	0.034	0.993	8.445E-01	logistic	419231	1915	417316	0.999	0.337
555.21	Ulcerative colitis (chronic)	digestive	rs10852766	0.014	0.076	1.015	8.501E-01	logistic	334866	381	334485	0.998	0.337
571.6	Primary biliary cirrhosis	digestive	rs10852766	0.016	0.084	1.016	8.535E-01	logistic	400586	312	400274	0.999	0.336
411.4	Coronary atherosclerosis	circulatory system	rs10852766	-0.002	0.010	0.998	8.543E-01	logistic	394337	23787	370550	0.999	0.337
752	Nervous system congenital anomalies	congenital anomalies	rs10852766	0.013	0.070	1.013	8.551E-01	logistic	422992	453	422539	1.000	0.337

ST31; PheWAS results for the lead cis-pQTLs of the 4 proteins prioritized (ACOX1, FGF5, FURIN, and MST1).

SNP = Single Nucleotide Polymorphism; SE = Standard error HWE_p = P-value for Hardy-Weinberg equilibrium test

Phenotype	Description	Group	SNP	Beta	SE	Odds tatio	P-value	Type	n_total	n_cases	n_controls	HWE_p	allele_freq
227.1	Benign neoplasm of adrenal gland	neoplasms	rs10852766	0.017	0.093	1.017	8.551E-01	logistic	421144	257	420887	1.000	0.337
529	Diseases and other conditions of the tongue	digestive	rs10852766	-0.016	0.090	0.984	8.558E-01	logistic	417990	280	417710	1.000	0.337
389.3	Degenerative and vascular disorders of ear	sense organs	rs10852766	-0.015	0.081	0.986	8.573E-01	logistic	408638	341	408297	1.000	0.337
320	Meningitis	neurological	rs10852766	-0.015	0.086	0.985	8.638E-01	logistic	422374	303	422071	1.000	0.337
575.2	Obstruction of bile duct	digestive	rs10852766	0.008	0.047	1.008	8.658E-01	logistic	396076	1029	395047	0.999	0.337
628	Ovarian cyst	genitourinary	rs10852766	-0.007	0.043	0.993	8.659E-01	logistic	193944	1197	192747	0.995	0.337
291	Other specified nonpsychotic and/or transient mental disorders	mental disorders	rs10852766	0.013	0.075	1.013	8.663E-01	logistic	405371	398	404973	1.000	0.337
724.1	Disorders of sacrum	musculoskeletal	rs10852766	0.017	0.098	1.017	8.666E-01	logistic	408900	230	408670	1.000	0.337
378.5	Paralytic strabismus	sense organs	rs10852766	-0.015	0.091	0.985	8.669E-01	logistic	412201	273	411928	0.999	0.337
741	Symptoms and disorders of the joints	musculoskeletal	rs10852766	0.006	0.037	1.006	8.695E-01	logistic	416612	1602	415010	1.000	0.337
362.2	Degeneration of macula and posterior pole of retina	sense organs	rs10852766	-0.003	0.016	0.997	8.697E-01	logistic	406927	9170	397757	1.000	0.337
202.24	Large cell lymphoma	neoplasms	rs10852766	-0.007	0.045	0.993	8.707E-01	logistic	416717	1104	415613	0.999	0.337
530.2	Esophageal bleeding (varices/hemorrhage)	digestive	rs10852766	0.012	0.073	1.012	8.717E-01	logistic	357206	418	356788	0.999	0.337
717	Polymyalgia Rheumatica	musculoskeletal	rs10852766	-0.005	0.030	0.995	8.736E-01	logistic	422397	2442	419955	1.000	0.337
569.2	Gastrointestinal complications	digestive	rs10852766	-0.014	0.090	0.986	8.745E-01	logistic	384117	278	383839	0.995	0.336
274.1	Gout	endocrine/metabolic	rs10852766	0.004	0.023	1.004	8.747E-01	logistic	420024	4234	415790	1.000	0.337
455	Hemorrhoids	circulatory system	rs10852766	0.003	0.021	1.003	8.760E-01	logistic	386244	5278	380966	0.998	0.336
425	Cardiomyopathy	circulatory system	rs10852766	-0.005	0.034	0.995	8.794E-01	logistic	418683	1913	416770	1.000	0.337
318	Tobacco use disorder	mental disorders	rs10852766	-0.003	0.021	0.997	8.831E-01	logistic	408298	5005	403293	1.000	0.337
507	Pleurisy; pleural effusion	respiratory	rs10852766	-0.002	0.015	0.998	8.836E-01	logistic	400937	9880	391057	1.000	0.337
722	Intervertebral disc disorders	musculoskeletal	rs10852766	-0.008	0.058	0.992	8.900E-01	logistic	409333	663	408670	1.000	0.337
593	Hematuria	genitourinary	rs10852766	0.003	0.021	1.003	8.926E-01	logistic	381996	5069	376927	0.999	0.336
367.1	Myopia	sense organs	rs10852766	-0.006	0.041	0.994	8.931E-01	logistic	412331	1325	411006	0.999	0.337
580.32	Nephritis and nephropathy with pathological lesion	genitourinary	rs10852766	0.012	0.090	1.012	8.939E-01	logistic	381336	274	381062	0.998	0.337
204	Leukemia	neoplasms	rs10852766	0.004	0.027	1.004	8.959E-01	logistic	418621	3008	415613	0.999	0.337
707.1	Decubitus ulcer	dermatologic	rs10852766	0.004	0.027	1.004	8.969E-01	logistic	419126	2996	416130	1.000	0.337
851	Complications of transplants and reattached limbs	injuries & poisonings	rs10852766	0.012	0.096	1.012	8.980E-01	logistic	416895	243	416652	1.000	0.337
521.1	Dental caries	digestive	rs10852766	-0.007	0.055	0.993	8.993E-01	logistic	410280	744	409536	1.000	0.337
967	Adverse effects of sedatives or other central nervous system depressants and anesthetics	injuries & poisonings	rs10852766	0.013	0.099	1.013	8.995E-01	logistic	367398	225	367173	0.998	0.336
279.11	Deficiency of humoral immunity	endocrine/metabolic	rs10852766	0.012	0.096	1.012	9.003E-01	logistic	423118	242	422876	1.000	0.337
250.11	Type 1 diabetes with ketoacidosis	endocrine/metabolic	rs10852766	-0.012	0.095	0.988	9.013E-01	logistic	386885	248	386637	0.999	0.337
446.4	Wegener's granulomatosis	circulatory system	rs10852766	0.012	0.097	1.012	9.016E-01	logistic	405703	234	405469	1.000	0.337
550.6	Incisional hernia	digestive	rs10852766	0.009	0.070	1.009	9.016E-01	logistic	365851	457	365394	0.996	0.336
556.1	Ulceration of intestine	digestive	rs10852766	-0.013	0.106	0.987	9.019E-01	logistic	334685	200	334485	0.997	0.336
452	Other venous embolism and thrombosis	circulatory system	rs10852766	-0.010	0.083	0.990	9.020E-01	logistic	381290	324	380966	0.998	0.336
386	Vertiginous syndromes and other disorders of vestibular system	sense organs	rs10852766	-0.004	0.037	0.996	9.049E-01	logistic	411346	1681	409665	1.000	0.337
191.1	Cancer of brain and nervous system	neoplasms	rs10852766	-0.006	0.052	0.994	9.059E-01	logistic	421229	832	420397	1.000	0.337
946	Anaphylactic shock NOS	injuries & poisonings	rs10852766	0.011	0.100	1.011	9.092E-01	logistic	415210	223	414987	1.000	0.337
377	Disorders of optic nerve and visual pathways	sense organs	rs10852766	0.009	0.078	1.009	9.120E-01	logistic	412293	365	411928	0.999	0.337
702.2	Seborrheic keratosis	dermatologic	rs10852766	-0.007	0.064	0.993	9.129E-01	logistic	413611	539	413072	1.000	0.337
624.9	stress incontinence, female	genitourinary	rs10852766	0.004	0.035	1.004	9.141E-01	logistic	220155	1871	218284	0.999	0.336
523.3	Periodontitis (acute or chronic)	digestive	rs10852766	-0.011	0.098	0.990	9.144E-01	logistic	409770	234	409536	1.000	0.337
451	Phlebitis and thrombophlebitis	circulatory system	rs10852766	-0.006	0.056	0.994	9.145E-01	logistic	381675	709	380966	0.998	0.336
752.11	Spina bifida	congenital anomalies	rs10852766	-0.011	0.105	0.989	9.171E-01	logistic	422743	204	422539	1.000	0.337
362	Other retinal disorders	sense organs	rs10852766	0.002	0.015	1.002	9.204E-01	logistic	407698	9941	397757	1.000	0.337
614.3	Pelvic inflammatory disease (PID)	genitourinary	rs10852766	-0.010	0.097	0.990	9.207E-01	logistic	217675	237	217438	0.997	0.336
715	Other inflammatory spondylopathies	musculoskeletal	rs10852766	-0.004	0.042	0.996	9.217E-01	logistic	409805	1298	408507	1.000	0.337
450	Noninfectious disorders of lymphatic channels	circulatory system	rs10852766	0.004	0.044	1.004	9.224E-01	logistic	422720	1167	421553	1.000	0.337
427.8	Sinoatrial node dysfunction (Bradycardia)	circulatory system	rs10852766	0.006	0.062	1.006	9.231E-01	logistic	372427	579	371848	1.000	0.337
165.1	Cancer of bronchus; lung	neoplasms	rs10852766	-0.002	0.023	0.998	9.243E-01	logistic	421452	4223	417229	1.000	0.337
352	Disorders of other cranial nerves	neurological	rs10852766	-0.004	0.043	0.996	9.244E-01	logistic	403637	1222	402415	1.000	0.337
286.1	Congenital coagulation defects	hematopoietic	rs10852766	-0.007	0.073	0.993	9.254E-01	logistic	417976	423	417553	1.000	0.337
474	Acute and chronic tonsillitis	respiratory	rs10852766	0.007	0.077	1.007	9.259E-01	logistic	396895	378	396517	1.000	0.336
241.2	Nontoxic multinodular goiter	endocrine/metabolic	rs10852766	0.009	0.097	1.009	9.263E-01	logistic	392467	237	392230	0.999	0.336
568	Other disorders of peritoneum	digestive	rs10852766	-0.004	0.039	0.996	9.268E-01	logistic	385305	1466	383839	0.992	0.336
202	Cancer of other lymphoid, histiocytic tissue	neoplasms	rs10852766	0.003	0.029	1.003	9.283E-01	logistic	418277	2664	415613	1.000	0.337
202.2	Non-Hodgkins lymphoma	neoplasms	rs10852766	0.003	0.029	1.003	9.283E-01	logistic	418277	2664	415613	1.000	0.337
580.3	Nephritis and nephropathy without mention of glomerulonephritis	genitourinary	rs10852766	0.008	0.087	1.008	9.291E-01	logistic	381356	294	381062	0.998	0.337
681	Superficial cellulitis and abscess	dermatologic	rs10852766	0.008	0.086	1.008	9.293E-01	logistic	418799	302	418497	1.000	0.337
340	Migraine	neurological	rs10852766	-0.003	0.030	0.997	9.302E-01	logistic	407732	2545	405187	1.000	0.337
560.4	Other intestinal obstruction	digestive	rs10852766	0.003	0.032	1.003	9.306E-01	logistic	336617	2132	334485	0.998	0.337
800.1	Fracture of neck of femur	injuries & poisonings	rs10852766	0.003	0.033	1.003	9.319E-01	logistic	398432	2054	396378	0.999	0.337
573	Other disorders of liver	digestive	rs10852766	-0.003	0.033	0.997	9.322E-01	logistic	402294	2020	400274	0.998	0.336
550	Abdominal hernia	digestive	rs10852766	-0.001	0.009	0.999	9.325E-01	logistic	392811	27417	365394	0.996	0.336
368	Visual disturbances	sense organs	rs10852766	-0.002	0.031	0.998	9.374E-01	logistic	417843	2298	415545	1.000	0.337
275	Disorders of mineral metabolism	endocrine/metabolic	rs10852766	-0.002	0.022	0.998	9.416E-01	logistic	420958	4773	416185	1.000	0.337
442	Other aneurysm	circulatory system	rs10852766	-0.002	0.027	0.998	9.418E-01	logistic	408493	3024	405469	1.000	0.337
724.2	Disorders of coccyx	musculoskeletal	rs10852766	0.007	0.102	1.007	9.421E-01	logistic	408885	215	408670	1.000	0.337
735.3	Hallux valgus (Bunion)	musculoskeletal	rs10852766	0.002	0.030	1.002	9.470E-01	logistic	405116	2462	402654	0.999	0.337
575.8	Other disorders of biliary tract	digestive	rs10852766	0.003	0.046	1.003	9.477E-01	logistic	396103	1056	395047	0.999	0.337
440.2	Atherosclerosis of the extremities	circulatory system	rs10852766	0.003	0.051	1.003	9.486E-01	logistic	406343	874	405469	1.000	0.337

ST31; PheWAS results for the lead cis-pQTLs of the 4 proteins prioritized (ACOX1, FGF5, FURIN, and MST1).

SNP = Single Nucleotide Polymorphism; SE = Standard error HWE_p = P-value for Hardy-Weinberg equilibrium test

Phenotype	Description	Group	SNP	Beta	SE	Odds tatio	P-value	Type	n_total	n_cases	n_controls	HWE_p	allele_freq
242	Thyrotoxicosis with or without goiter	endocrine/metabolic	rs10852766	0.002	0.038	1.002	9.487E-01	logistic	393812	1582	392230	0.999	0.336
429.2	Abnormal function study of cardiovascular system	circulatory system	rs10852766	0.005	0.082	1.005	9.512E-01	logistic	410036	333	409703	0.999	0.337
627	Menopausal and postmenopausal disorders	genitourinary	rs10852766	0.002	0.029	1.002	9.521E-01	logistic	195450	2703	192747	0.992	0.337
189.2	Cancer of bladder	neoplasms	rs10852766	-0.002	0.026	0.998	9.528E-01	logistic	420532	3345	417187	1.000	0.337
560	Intestinal obstruction without mention of hernia	digestive	rs10852766	0.001	0.025	1.001	9.530E-01	logistic	338110	3625	334485	0.998	0.337
599.4	Urinary incontinence	genitourinary	rs10852766	-0.002	0.029	0.998	9.538E-01	logistic	392806	2700	390106	0.999	0.336
70	Viral hepatitis	infectious diseases	rs10852766	-0.004	0.064	0.996	9.543E-01	logistic	415755	551	415204	1.000	0.337
752.1	Neural tube defects	congenital anomalies	rs10852766	-0.004	0.075	0.996	9.570E-01	logistic	422933	394	422539	1.000	0.337
433	Cerebrovascular disease	circulatory system	rs10852766	-0.001	0.014	0.999	9.595E-01	logistic	414008	11323	402685	0.999	0.337
159.3	Malignant neoplasm of gallbladder and extrahepatic bile ducts	neoplasms	rs10852766	0.004	0.082	1.004	9.610E-01	logistic	394194	332	393862	0.999	0.337
297.2	Suicide or self-inflicted injury	mental disorders	rs10852766	0.004	0.083	1.004	9.633E-01	logistic	381948	326	381622	0.999	0.337
394.2	Mitral valve disease	circulatory system	rs10852766	-0.004	0.085	0.996	9.646E-01	logistic	401726	312	401414	0.999	0.336
695.2	Bullous dermatoses	dermatologic	rs10852766	0.004	0.105	1.004	9.671E-01	logistic	405408	202	405206	1.000	0.336
430	Intracranial hemorrhage	circulatory system	rs10852766	-0.001	0.034	0.999	9.681E-01	logistic	404595	1910	402685	0.999	0.337
522	Diseases of pulp and periapical tissues	digestive	rs10852766	0.003	0.087	1.003	9.689E-01	logistic	409832	296	409536	1.000	0.337
781	Symptoms involving nervous and musculoskeletal system	symptoms	rs10852766	0.001	0.017	1.001	9.691E-01	logistic	419375	7893	411482	1.000	0.337
579.2	Splenomegaly	digestive	rs10852766	0.002	0.060	1.002	9.691E-01	logistic	383919	616	383303	0.999	0.336
228	Hemangioma and lymphangioma, any site	neoplasms	rs10852766	0.002	0.065	1.002	9.712E-01	logistic	421655	529	421126	1.000	0.337
555	Inflammatory bowel disease and other gastroenteritis and coliti	digestive	rs10852766	-0.001	0.022	0.999	9.722E-01	logistic	339234	4749	334485	0.998	0.336
425.12	Other hypertrophic cardiomyopathy	circulatory system	rs10852766	-0.003	0.090	0.997	9.732E-01	logistic	417044	274	416770	0.999	0.337
585	Renal failure	genitourinary	rs10852766	0.000	0.010	1.000	9.757E-01	logistic	403986	22924	381062	0.999	0.337
454	Varicose veins	circulatory system	rs10852766	-0.001	0.022	0.999	9.763E-01	logistic	385827	4861	380966	0.999	0.336
170.2	Cancer of connective tissue	neoplasms	rs10852766	0.003	0.095	1.003	9.768E-01	logistic	423349	248	423101	1.000	0.337
274	Gout and other crystal arthropathies	endocrine/metabolic	rs10852766	0.001	0.023	1.001	9.769E-01	logistic	420276	4486	415790	1.000	0.337
428.1	Congestive heart failure (CHF) NOS	circulatory system	rs10852766	-0.001	0.024	0.999	9.783E-01	logistic	413649	3946	409703	0.999	0.337
574.11	Cholelithiasis with acute cholecystitis	digestive	rs10852766	-0.001	0.047	0.999	9.789E-01	logistic	396059	1012	395047	0.999	0.337
427	Cardiac dysrhythmias	circulatory system	rs10852766	0.000	0.010	1.000	9.810E-01	logistic	398369	26521	371848	1.000	0.337
789	Nausea and vomiting	symptoms	rs10852766	0.000	0.018	1.000	9.813E-01	logistic	407825	6923	400902	1.000	0.337
618.1	Prolapse of vaginal walls	genitourinary	rs10852766	-0.001	0.024	0.999	9.816E-01	logistic	217426	3971	213455	0.998	0.336
568.1	Peritoneal adhesions (postoperative) (postinfection	digestive	rs10852766	-0.001	0.042	0.999	9.817E-01	logistic	385136	1297	383839	0.993	0.336
427.4	Cardiac arrest and ventricular fibrillation	circulatory system	rs10852766	0.001	0.050	1.001	9.825E-01	logistic	372755	907	371848	1.000	0.337
560.1	Paralytic ileus	digestive	rs10852766	-0.002	0.075	0.998	9.840E-01	logistic	334880	395	334485	0.997	0.336
525	Other diseases of the teeth and supporting structures	digestive	rs10852766	0.001	0.067	1.001	9.844E-01	logistic	410027	491	409536	1.000	0.337
735.21	Hammer toe (acquired)	musculoskeletal	rs10852766	0.001	0.070	1.001	9.844E-01	logistic	403105	451	402654	0.999	0.337
381.11	Suppurative and unspecified otitis media	sense organs	rs10852766	0.002	0.094	1.002	9.849E-01	logistic	418969	253	418716	1.000	0.337
289.4	Lymphadenitis	hematopoietic	rs10852766	-0.001	0.036	0.999	9.852E-01	logistic	410013	1704	408309	1.000	0.337
227.3	Benign neoplasm of pituitary gland and craniopharyngeal duct (pouch	neoplasms	rs10852766	0.001	0.079	1.001	9.868E-01	logistic	421248	361	420887	1.000	0.337
275.1	Disorders of iron metabolism	hematopoietic	rs10852766	0.001	0.045	1.001	9.886E-01	logistic	417274	1089	416185	1.000	0.337
363	Chorioretinal inflammations, scars, and other disorders of choroid	sense organs	rs10852766	-0.001	0.098	0.999	9.895E-01	logistic	397993	230	397763	0.999	0.337
191.11	Cancer of brain	neoplasms	rs10852766	-0.001	0.053	0.999	9.895E-01	logistic	421187	790	420397	1.000	0.337
564	Functional digestive disorders	digestive	rs10852766	0.000	0.011	1.000	9.907E-01	logistic	354387	19902	334485	0.998	0.337
244.1	Secondary hypothyroidism	endocrine/metabolic	rs10852766	0.000	0.043	1.000	9.908E-01	logistic	393443	1213	392230	0.999	0.336
707	Chronic ulcer of skin	dermatologic	rs10852766	0.000	0.022	1.000	9.913E-01	logistic	420904	4774	416130	1.000	0.337
418.1	Precordial pain	circulatory system	rs10852766	0.000	0.035	1.000	9.914E-01	logistic	417539	1859	415680	1.000	0.337
218.1	Uterine leiomyoma	neoplasms	rs10852766	0.000	0.029	1.000	9.919E-01	logistic	209756	2761	206995	0.998	0.336
798.1	Chronic fatigue syndrome	symptoms	rs10852766	0.001	0.060	1.001	9.928E-01	logistic	414937	617	414320	1.000	0.337
614.5	Inflammatory disease of cervix, vagina, and vulva	genitourinary	rs10852766	0.001	0.065	1.001	9.931E-01	logistic	217969	531	217438	0.995	0.336
158	Neoplasm of unspecified nature of digestive system	neoplasms	rs10852766	-0.001	0.077	0.999	9.948E-01	logistic	394238	376	393862	0.999	0.337
429	Ill-defined descriptions and complications of heart disease	circulatory system	rs10852766	0.001	0.082	1.001	9.950E-01	logistic	410037	334	409703	0.999	0.337
433.31	Transient cerebral ischemia	circulatory system	rs10852766	0.000	0.043	1.000	9.961E-01	logistic	403901	1216	402685	0.999	0.337
401	Hypertension	circulatory system	rs12509595	0.095	0.006	1.099	1.235E-55	logistic	390097	99434	290663	0.986	0.289
401.1	Essential hypertension	circulatory system	rs12509595	0.094	0.006	1.099	3.298E-55	logistic	389876	99213	290663	0.986	0.289
411	Ischemic Heart Disease	circulatory system	rs12509595	0.050	0.008	1.051	3.709E-09	logistic	410606	40056	370550	0.991	0.289
411.4	Coronary atherosclerosis	circulatory system	rs12509595	0.062	0.011	1.064	4.205E-09	logistic	394337	23787	370550	0.993	0.289
613	Other nonmalignant breast conditions	genitourinary	rs12509595	-0.456	0.088	0.634	2.028E-07	logistic	421594	397	421197	0.989	0.290
426.3	Bundle branch block	circulatory system	rs12509595	0.128	0.026	1.136	6.799E-07	logistic	375377	3529	371848	0.996	0.289
411.8	Other chronic ischemic heart disease, unspecified	circulatory system	rs12509595	0.056	0.012	1.057	2.651E-06	logistic	389207	18657	370550	0.987	0.289
426.32	Left bundle branch block	circulatory system	rs12509595	0.127	0.028	1.135	4.414E-06	logistic	374914	3066	371848	0.996	0.289
411.3	Angina pectoris	circulatory system	rs12509595	0.051	0.012	1.053	3.875E-05	logistic	387093	16543	370550	0.982	0.289
426	Cardiac conduction disorders	circulatory system	rs12509595	0.062	0.015	1.063	5.660E-05	logistic	382540	10692	371848	0.996	0.289
411.2	Myocardial infarction	circulatory system	rs12509595	0.052	0.013	1.053	8.944E-05	logistic	385085	14535	370550	0.988	0.289
585.3	Chronic renal failure [CKD]	genitourinary	rs12509595	-0.056	0.014	0.946	1.034E-04	logistic	393739	12677	381062	0.989	0.290
626.8	Infertility, female	genitourinary	rs12509595	-0.353	0.101	0.703	4.909E-04	logistic	193029	282	192747	0.994	0.290
585.34	Chronic Kidney Disease, Stage IV	genitourinary	rs12509595	-0.151	0.047	0.860	1.496E-03	logistic	382213	1151	381062	0.989	0.290
599.4	Urinary incontinence	genitourinary	rs12509595	0.092	0.030	1.096	1.861E-03	logistic	392806	2700	390106	0.968	0.289
427.2	Atrial fibrillation and flutter	circulatory system	rs12509595	0.038	0.013	1.039	2.252E-03	logistic	388316	16468	371848	0.972	0.289
272	Disorders of lipoid metabolism	endocrine/metabolic	rs12509595	0.026	0.008	1.026	2.328E-03	logistic	394625	38860	355765	0.999	0.289
272.1	Hyperlipidemia	endocrine/metabolic	rs12509595	0.026	0.009	1.026	2.755E-03	logistic	394381	38616	355765	0.999	0.289
276.14	Hypopotassemia	endocrine/metabolic	rs12509595	0.078	0.026	1.081	2.791E-03	logistic	399074	3502	395572	0.992	0.290
337	Disorders of the autonomic nervous system	neurological	rs12509595	0.231	0.080	1.259	3.849E-03	logistic	399396	349	399047	0.990	0.290
242	Thyrotoxicosis with or without goiter	endocrine/metabolic	rs12509595	0.111	0.038	1.117	3.933E-03	logistic	393812	1582	392230	0.974	0.290

ST31; PheWAS results for the lead cis-pQTLs of the 4 proteins prioritized (ACOX1, FGF5, FURIN, and MST1).
SNP = Single Nucleotide Polymorphism; SE = Standard error HWE_p = P-value for Hardy-Weinberg equilibrium test

Phenotype	Description	Group	SNP	Beta	SE	Odds tatio	P-value	Type	n_total	n_cases	n_controls	HWE_p	allele_freq
411.1	Unstable angina (intermediate coronary syndrome	circulatory system	rs12509595	0.070	0.025	1.073	5.561E-03	logistic	374269	3719	370550	0.989	0.288
427	Cardiac dysrhythmias	circulatory system	rs12509595	0.028	0.010	1.028	5.582E-03	logistic	398369	26521	371848	0.982	0.289
585.1	Acute renal failure	genitourinary	rs12509595	-0.038	0.014	0.962	6.097E-03	logistic	394375	13313	381062	0.994	0.290
585	Renal failure	genitourinary	rs12509595	-0.029	0.011	0.971	6.585E-03	logistic	403986	22924	381062	0.991	0.290
427.22	Atrial flutter	circulatory system	rs12509595	0.161	0.059	1.174	6.676E-03	logistic	372499	651	371848	0.993	0.289
599	Other symptoms/disorders or the urinary system	genitourinary	rs12509595	0.047	0.017	1.048	7.091E-03	logistic	398281	8175	390106	0.982	0.289
707.1	Decubitus ulcer	dermatologic	rs12509595	-0.078	0.029	0.925	7.123E-03	logistic	419126	2996	416130	0.992	0.289
272.11	Hypercholesterolemia	endocrine/metabolic	rs12509595	0.024	0.009	1.024	7.785E-03	logistic	390444	34679	355765	0.999	0.289
334	Degenerative disease of the spinal cord	neurological	rs12509595	0.095	0.037	1.100	9.531E-03	logistic	400789	1742	399047	0.990	0.290
740.1	Osteoarthritis; localized	musculoskeletal	rs12509595	-0.025	0.010	0.975	1.099E-02	logistic	374099	28241	345858	0.982	0.290
189.2	Cancer of bladder	neoplasms	rs12509595	0.067	0.027	1.069	1.224E-02	logistic	420532	3345	417187	0.990	0.290
365	Glaucoma	sense organs	rs12509595	0.044	0.018	1.045	1.429E-02	logistic	405209	7446	397763	0.992	0.290
735.21	Hammer toe (acquired)	musculoskeletal	rs12509595	-0.186	0.077	0.830	1.497E-02	logistic	403105	451	402654	0.982	0.289
189.21	Malignant neoplasm of bladder	neoplasms	rs12509595	0.068	0.028	1.071	1.504E-02	logistic	420210	3023	417187	0.990	0.290
636.3	Hemorrhage in early pregnancy	pregnancy complications	rs12509595	-0.269	0.113	0.764	1.713E-02	logistic	222810	218	222592	0.982	0.290
285.2	Anemia of chronic disease	hematopoietic	rs12509595	-0.135	0.057	0.874	1.809E-02	logistic	383237	788	382449	0.983	0.290
172	Skin cancer	neoplasms	rs12509595	-0.048	0.020	0.953	1.822E-02	logistic	413263	6055	407208	0.994	0.290
365.2	Primary angle-closure glaucoma	sense organs	rs12509595	-0.145	0.062	0.865	1.896E-02	logistic	398441	678	397763	0.990	0.289
425.1	Primary/intrinsic cardiomyopathies	circulatory system	rs12509595	0.083	0.036	1.087	1.927E-02	logistic	418636	1866	416770	0.990	0.289
459	Other disorders of circulatory system	circulatory system	rs12509595	0.025	0.011	1.025	1.928E-02	logistic	395720	23148	372572	0.991	0.289
459.9	Circulatory disease NEC	circulatory system	rs12509595	0.025	0.011	1.025	2.032E-02	logistic	395548	22976	372572	0.991	0.289
324	Other CNS infection and poliomyelitis	neurological	rs12509595	0.186	0.081	1.205	2.069E-02	logistic	422418	347	422071	0.992	0.290
521	Diseases of hard tissues of teeth	digestive	rs12509595	-0.130	0.057	0.878	2.209E-02	logistic	410337	801	409536	0.993	0.289
585.33	Chronic Kidney Disease, Stage III	genitourinary	rs12509595	-0.042	0.018	0.959	2.251E-02	logistic	388537	7475	381062	0.987	0.290
750.2	Lower gastrointestinal congenital anomalies	congenital anomalies	rs12509595	-0.266	0.117	0.767	2.346E-02	logistic	420453	200	420253	0.991	0.290
189	Cancer of urinary organs (incl. kidney and bladder)	neoplasms	rs12509595	0.052	0.023	1.053	2.378E-02	logistic	421760	4573	417187	0.990	0.290
740.12	Osteoarthrosis, localized, secondary	musculoskeletal	rs12509595	-0.177	0.078	0.838	2.384E-02	logistic	346286	428	345858	0.989	0.290
395	Heart valve disorders	circulatory system	rs12509595	0.036	0.016	1.037	2.391E-02	logistic	411117	9703	401414	0.996	0.289
433	Cerebrovascular disease	circulatory system	rs12509595	0.033	0.015	1.034	2.582E-02	logistic	414008	11323	402685	0.992	0.290
272.9	Unspecified disorder of lipoid metabolism	endocrine/metabolic	rs12509595	0.187	0.084	1.205	2.594E-02	logistic	356085	320	355765	0.999	0.289
530.5	Disorders of esophageal motility	digestive	rs12509595	0.161	0.073	1.175	2.801E-02	logistic	357212	424	356788	0.989	0.290
595	Hydronephrosis	genitourinary	rs12509595	0.106	0.049	1.112	2.927E-02	logistic	413362	986	412376	0.992	0.290
728	Disorders of muscle, ligament, and fascia	musculoskeletal	rs12509595	0.203	0.093	1.225	2.955E-02	logistic	389432	258	389174	0.994	0.289
572	Ascites (non malignant)	digestive	rs12509595	0.072	0.033	1.074	3.091E-02	logistic	402409	2135	400274	0.987	0.290
425	Cardiomyopathy	circulatory system	rs12509595	0.076	0.035	1.079	3.095E-02	logistic	418683	1913	416770	0.990	0.289
585.31	Renal dialysis	genitourinary	rs12509595	-0.129	0.060	0.879	3.158E-02	logistic	381775	713	381062	0.992	0.290
426.91	Cardiac pacemaker in situ	circulatory system	rs12509595	0.048	0.022	1.049	3.170E-02	logistic	376826	4978	371848	0.991	0.289
379	Other disorders of eye	sense organs	rs12509595	0.102	0.047	1.107	3.230E-02	logistic	412963	1035	411928	0.991	0.289
172.2	Other non-epithelial cancer of skin	neoplasms	rs12509595	-0.056	0.026	0.945	3.267E-02	logistic	410817	3609	407208	0.994	0.290
702	Degenerative skin conditions and other dermatoses	dermatologic	rs12509595	-0.078	0.037	0.925	3.329E-02	logistic	407215	1867	405348	0.994	0.290
416	Cardiomegaly	circulatory system	rs12509595	0.050	0.023	1.051	3.395E-02	logistic	417255	4417	412838	0.997	0.290
642	Hypertension complicating pregnancy, childbirth, and the puerperium	pregnancy complications	rs12509595	0.150	0.072	1.162	3.650E-02	logistic	228250	456	227794	0.985	0.289
281.1	Megaloblastic anemia	hematopoietic	rs12509595	-0.093	0.045	0.911	3.759E-02	logistic	383709	1260	382449	0.983	0.290
426.9	Cardiac pacemaker/device in situ	circulatory system	rs12509595	0.045	0.022	1.046	3.818E-02	logistic	376950	5102	371848	0.991	0.289
770	Myalgia and myositis unspecified	symptoms	rs12509595	0.074	0.036	1.076	4.004E-02	logistic	422525	1839	420686	0.993	0.290
396	Abnormal heart sounds	circulatory system	rs12509595	0.102	0.050	1.107	4.107E-02	logistic	402357	943	401414	0.994	0.289
504	Other alveolar and parietoalveolar pneumonopathy	respiratory	rs12509595	-0.127	0.062	0.881	4.200E-02	logistic	391718	661	391057	0.994	0.289
628	Ovarian cyst	genitourinary	rs12509595	0.090	0.044	1.094	4.210E-02	logistic	193944	1197	192747	0.994	0.290
281	Other deficiency anemia	hematopoietic	rs12509595	-0.089	0.044	0.915	4.220E-02	logistic	383766	1317	382449	0.984	0.290
521.1	Dental caries	digestive	rs12509595	-0.119	0.059	0.888	4.268E-02	logistic	410280	744	409536	0.993	0.289
627	Menopausal and postmenopausal disorders	genitourinary	rs12509595	-0.062	0.031	0.940	4.309E-02	logistic	195450	2703	192747	0.992	0.290
427.11	Paroxysmal supraventricular tachycardia	circulatory system	rs12509595	-0.069	0.034	0.933	4.396E-02	logistic	373959	2111	371848	0.992	0.289
721.8	Other allied disorders of spine	musculoskeletal	rs12509595	0.210	0.105	1.234	4.604E-02	logistic	408871	201	408670	0.991	0.290
571.81	Portal hypertension	digestive	rs12509595	-0.108	0.054	0.898	4.691E-02	logistic	401135	861	400274	0.988	0.290
592.1	Cystitis	genitourinary	rs12509595	-0.091	0.046	0.913	4.719E-02	logistic	378131	1204	376927	0.982	0.290
442	Other aneurysm	circulatory system	rs12509595	0.056	0.028	1.057	4.734E-02	logistic	408493	3024	405469	0.991	0.289
78	Viral warts & HPV	infectious diseases	rs12509595	-0.216	0.110	0.805	4.884E-02	logistic	415426	222	415204	0.988	0.290
172.1	Melanomas of skin, dx or hx	neoplasms	rs12509595	-0.073	0.037	0.930	4.956E-02	logistic	409038	1830	407208	0.993	0.290
172.11	Melanomas of skin	neoplasms	rs12509595	-0.073	0.037	0.930	4.956E-02	logistic	409038	1830	407208	0.993	0.290
750	Digestive congenital anomalies	congenital anomalies	rs12509595	-0.154	0.079	0.857	4.996E-02	logistic	420674	421	420253	0.991	0.290
636	Early or threatened labor; hemorrhage in early pregnancy	pregnancy complications	rs12509595	-0.138	0.071	0.871	5.005E-02	logistic	223122	530	222592	0.980	0.290
634.1	Missed abortion/Hydatidiform mole	pregnancy complications	rs12509595	0.201	0.103	1.222	5.061E-02	logistic	222807	215	222592	0.981	0.290
592	Cystitis and urethritis	genitourinary	rs12509595	-0.088	0.045	0.916	5.117E-02	logistic	378176	1249	376927	0.982	0.290
327	Sleep disorders	neurological	rs12509595	0.124	0.064	1.132	5.299E-02	logistic	413949	565	413384	0.992	0.289
804	Fracture of hand or wrist	injuries & poisonings	rs12509595	0.122	0.063	1.130	5.389E-02	logistic	396955	577	396378	0.983	0.289
785	Abdominal pain	symptoms	rs12509595	-0.036	0.019	0.965	5.518E-02	logistic	401183	7225	393958	0.986	0.289
585.32	End stage renal disease	genitourinary	rs12509595	-0.099	0.052	0.905	5.656E-02	logistic	381994	932	381062	0.991	0.290
446.9	Arteritis NOS	circulatory system	rs12509595	0.151	0.080	1.163	5.723E-02	logistic	405830	361	405469	0.992	0.289
740	Osteoarthrosis	musculoskeletal	rs12509595	-0.015	0.008	0.985	6.044E-02	logistic	392374	46516	345858	0.982	0.290
395.6	Heart valve replaced	circulatory system	rs12509595	0.058	0.031	1.060	6.050E-02	logistic	403924	2510	401414	0.995	0.289
153.2	Colon cancer	neoplasms	rs12509595	-0.044	0.023	0.957	6.143E-02	logistic	375260	4540	370720	0.995	0.289

ST31; PheWAS results for the lead cis-pQTLs of the 4 proteins prioritized (ACOX1, FGF5, FURIN, and MST1).

SNP = Single Nucleotide Polymorphism; SE = Standard error HWE_p = P-value for Hardy-Weinberg equilibrium test

Phenotype	Description	Group	SNP	Beta	SE	Odds tatio	P-value	Type	n_total	n_cases	n_controls	HWE_p	allele_freq
433.1	Occlusion and stenosis of precerebral arteries	circulatory system	rs12509595	0.072	0.039	1.075	6.199E-02	logistic	404257	1572	402685	0.996	0.289
386.2	Peripheral or central vertigo	sense organs	rs12509595	-0.119	0.064	0.888	6.475E-02	logistic	410281	616	409665	0.996	0.289
442.1	Aortic aneurysm	circulatory system	rs12509595	0.057	0.031	1.059	6.525E-02	logistic	407951	2482	405469	0.991	0.289
428.2	Heart failure NOS	circulatory system	rs12509595	0.041	0.022	1.042	6.581E-02	logistic	414622	4919	409703	0.989	0.289
513	Respiratory abnormalities	respiratory	rs12509595	0.134	0.073	1.143	6.695E-02	logistic	423122	431	422691	0.992	0.290
214	Lipoma	neoplasms	rs12509595	-0.087	0.048	0.917	6.788E-02	logistic	415339	1112	414227	0.988	0.290
433.2	Occlusion of cerebral arteries	circulatory system	rs12509595	0.045	0.025	1.047	6.801E-02	logistic	406563	3878	402685	0.997	0.289
741.3	Difficulty in walking	musculoskeletal	rs12509595	0.128	0.071	1.137	6.899E-02	logistic	415474	464	415010	0.990	0.289
395.2	Nonrheumatic aortic valve disorders	circulatory system	rs12509595	0.040	0.022	1.041	6.986E-02	logistic	406373	4959	401414	0.994	0.289
433.3	Cerebral ischemia	circulatory system	rs12509595	0.043	0.024	1.044	7.053E-02	logistic	407003	4318	402685	0.994	0.289
362.3	Other nondiabetic retinopathy	sense organs	rs12509595	0.117	0.064	1.124	7.073E-02	logistic	398314	557	397757	0.991	0.289
433.21	Cerebral artery occlusion, with cerebral infarction	circulatory system	rs12509595	0.045	0.025	1.046	7.144E-02	logistic	406495	3810	402685	0.997	0.289
687.1	Rash and other nonspecific skin eruption	dermatologic	rs12509595	0.083	0.046	1.087	7.285E-02	logistic	413803	1087	412716	0.989	0.289
726.1	Enthesopathy	musculoskeletal	rs12509595	0.048	0.027	1.049	7.289E-02	logistic	392491	3317	389174	0.993	0.290
707	Chronic ulcer of skin	dermatologic	rs12509595	-0.041	0.023	0.960	7.331E-02	logistic	420904	4774	416130	0.994	0.290
798.1	Chronic fatigue syndrome	symptoms	rs12509595	0.110	0.061	1.116	7.402E-02	logistic	414937	617	414320	0.996	0.290
721.1	Spondylosis without myelopathy	musculoskeletal	rs12509595	0.070	0.040	1.073	7.527E-02	logistic	410174	1504	408670	0.992	0.290
151	Cancer of stomach	neoplasms	rs12509595	0.087	0.049	1.091	7.581E-02	logistic	394829	967	393862	0.999	0.290
253.7	Other disorders of neurohypophysis	endocrine/metabolic	rs12509595	-0.153	0.086	0.858	7.599E-02	logistic	415594	348	415246	0.991	0.290
426.24	Atrioventricular block, complete	circulatory system	rs12509595	0.087	0.049	1.090	7.649E-02	logistic	372833	985	371848	0.993	0.289
334.2	Anterior horn cell disease	neurological	rs12509595	0.125	0.072	1.133	8.099E-02	logistic	399497	450	399047	0.989	0.290
276.42	Alkalosis	endocrine/metabolic	rs12509595	-0.160	0.092	0.852	8.217E-02	logistic	395878	306	395572	0.993	0.290
446	Polyarteritis nodosa and allied conditions	circulatory system	rs12509595	0.077	0.044	1.080	8.307E-02	logistic	406656	1187	405469	0.994	0.289
550.4	Umbilical hernia	digestive	rs12509595	-0.073	0.042	0.930	8.362E-02	logistic	366811	1417	365394	0.983	0.290
780	Hypothermia/Chills	symptoms	rs12509595	0.175	0.101	1.191	8.418E-02	logistic	423421	221	423200	0.993	0.290
561	Symptoms involving digestive system	digestive	rs12509595	0.038	0.022	1.039	8.429E-02	logistic	339539	5054	334485	0.997	0.290
946	Anaphylactic shock NOS	injuries & poisonings	rs12509595	0.173	0.101	1.189	8.516E-02	logistic	415210	223	414987	0.993	0.290
727	Other disorders of synovium, tendon, and bursa	musculoskeletal	rs12509595	0.051	0.030	1.053	8.555E-02	logistic	391842	2668	389174	0.992	0.289
172.3	Carcinoma in situ of skin	neoplasms	rs12509595	-0.136	0.079	0.873	8.578E-02	logistic	407616	408	407208	0.994	0.290
721	Spondylosis and allied disorders	musculoskeletal	rs12509595	0.061	0.035	1.063	8.669E-02	logistic	410559	1889	408670	0.991	0.290
428	Congestive heart failure; nonhypertensive	circulatory system	rs12509595	0.030	0.017	1.030	8.731E-02	logistic	417859	8156	409703	0.990	0.289
8.6	Viral Enteritis	infectious diseases	rs12509595	0.116	0.068	1.123	8.738E-02	logistic	417821	505	417316	0.995	0.290
293.1	Swelling, mass, or lump in head and neck [Space-occupying lesion, intracranial NOS]	mental disorders	rs12509595	-0.154	0.091	0.857	8.941E-02	logistic	420026	315	419711	0.994	0.290
411.9	Other acute and subacute forms of ischemic heart disease	circulatory system	rs12509595	0.067	0.040	1.069	9.277E-02	logistic	372042	1492	370550	0.985	0.288
214.1	Lipoma of skin and subcutaneous tissue	neoplasms	rs12509595	-0.098	0.059	0.907	9.639E-02	logistic	414958	731	414227	0.987	0.290
512.7	Shortness of breath	respiratory	rs12509595	-0.042	0.025	0.959	9.696E-02	logistic	408527	3928	404599	0.995	0.289
857	Mechanical complication of unspecified genitourinary device, implant, and graft	injuries & poisonings	rs12509595	0.064	0.039	1.066	9.830E-02	logistic	418243	1591	416652	0.992	0.290
789	Nausea and vomiting	symptoms	rs12509595	-0.031	0.019	0.969	9.854E-02	logistic	407825	6923	400902	0.993	0.290
960	Poisoning by antibiotics	injuries & poisonings	rs12509595	0.018	0.011	1.018	1.013E-01	logistic	389532	22359	367173	0.994	0.289
253.2	Pituitary hypofunction	endocrine/metabolic	rs12509595	0.145	0.089	1.156	1.034E-01	logistic	415534	288	415246	0.992	0.290
500	Lung disease due to external agents	respiratory	rs12509595	-0.104	0.064	0.902	1.057E-01	logistic	391675	618	391057	0.993	0.289
500.2	Pneumoconiosis	respiratory	rs12509595	-0.133	0.083	0.876	1.094E-01	logistic	391432	375	391057	0.993	0.290
740.11	Osteoarthrosis, localized, primary	musculoskeletal	rs12509595	-0.035	0.022	0.966	1.097E-01	logistic	351108	5250	345858	0.990	0.290
735.2	Acquired toe deformities	musculoskeletal	rs12509595	-0.057	0.036	0.944	1.116E-01	logistic	404574	1920	402654	0.985	0.289
295.1	Schizophrenia	mental disorders	rs12509595	-0.098	0.062	0.907	1.124E-01	logistic	382292	670	381622	0.988	0.289
362.31	Separation of retinal layers	sense organs	rs12509595	0.107	0.067	1.112	1.128E-01	logistic	398271	514	397757	0.990	0.289
702.1	Actinic keratosis	dermatologic	rs12509595	-0.083	0.052	0.921	1.131E-01	logistic	413997	925	413072	0.990	0.289
198.3	Secondary malignant neoplasm of digestive systems	neoplasms	rs12509595	0.047	0.030	1.048	1.147E-01	logistic	388741	2735	386006	0.983	0.290
590	Pyelonephritis	genitourinary	rs12509595	-0.067	0.043	0.935	1.153E-01	logistic	378294	1367	376927	0.987	0.290
697	Sarcoidosis	dermatologic	rs12509595	0.099	0.063	1.104	1.161E-01	logistic	405795	589	405206	0.996	0.289
571.8	Liver abscess and sequelae of chronic liver disease	digestive	rs12509595	-0.063	0.041	0.939	1.187E-01	logistic	401788	1514	400274	0.987	0.290
594.1	Calculus of kidney	genitourinary	rs12509595	0.043	0.027	1.044	1.197E-01	logistic	415555	3179	412376	0.991	0.290
250.11	Type 1 diabetes with ketoacidosis	endocrine/metabolic	rs12509595	0.149	0.096	1.161	1.212E-01	logistic	386885	248	386637	0.988	0.289
715.2	Ankylosing spondylitis	musculoskeletal	rs12509595	0.111	0.072	1.118	1.216E-01	logistic	408956	449	408507	0.996	0.289
803.3	Fracture of clavicle or scapula	injuries & poisonings	rs12509595	0.088	0.057	1.092	1.244E-01	logistic	397087	709	396378	0.983	0.289
800.3	Fracture of tibia and fibula	injuries & poisonings	rs12509595	-0.101	0.066	0.904	1.269E-01	logistic	396955	577	396378	0.985	0.289
729	Other disorders of soft tissues	musculoskeletal	rs12509595	0.146	0.096	1.157	1.274E-01	logistic	389423	249	389174	0.994	0.289
198.2	Secondary malignancy of respiratory organs	neoplasms	rs12509595	-0.035	0.023	0.966	1.277E-01	logistic	390707	4701	386006	0.994	0.290
496.21	Obstructive chronic bronchitis	respiratory	rs12509595	0.032	0.021	1.033	1.280E-01	logistic	404163	5354	398809	0.992	0.289
496.2	Chronic bronchitis	respiratory	rs12509595	0.032	0.021	1.032	1.336E-01	logistic	404328	5519	398809	0.991	0.289
579.8	Nonspecific abnormal findings in stool contents	digestive	rs12509595	0.091	0.061	1.095	1.384E-01	logistic	383921	618	383303	0.972	0.289
252	Disorders of parathyroid gland	endocrine/metabolic	rs12509595	-0.066	0.045	0.936	1.393E-01	logistic	416485	1239	415246	0.993	0.289
687	Symptoms affecting skin	dermatologic	rs12509595	0.117	0.079	1.124	1.395E-01	logistic	413083	367	412716	0.985	0.289
378.2	Nystagmus and other irregular eye movements	sense organs	rs12509595	-0.165	0.112	0.848	1.402E-01	logistic	412137	209	411928	0.988	0.289
345.1	Epilepsy	neurological	rs12509595	0.095	0.065	1.100	1.428E-01	logistic	399597	550	399047	0.988	0.290
727.4	Ganglion and cyst of synovium, tendon, and bursa	musculoskeletal	rs12509595	-0.108	0.074	0.898	1.430E-01	logistic	389641	467	389174	0.993	0.289
509.2	Respiratory insufficiency	respiratory	rs12509595	-0.063	0.043	0.939	1.435E-01	logistic	392402	1345	391057	0.993	0.289
274.1	Gout	endocrine/metabolic	rs12509595	-0.036	0.024	0.965	1.439E-01	logistic	420024	4234	415790	0.986	0.290
270.3	Disorders of plasma protein metabolism	endocrine/metabolic	rs12509595	0.082	0.056	1.085	1.454E-01	logistic	422449	747	421702	0.993	0.290
512	Other symptoms of respiratory system	respiratory	rs12509595	-0.028	0.020	0.972	1.514E-01	logistic	411090	6491	404599	0.995	0.289
395.1	Nonrheumatic mitral valve disorders	circulatory system	rs12509595	0.037	0.026	1.037	1.521E-01	logistic	405090	3676	401414	0.996	0.289

ST31; PheWAS results for the lead cis-pQTLs of the 4 proteins prioritized (ACOX1, FGF5, FURIN, and MST1).

SNP = Single Nucleotide Polymorphism; SE = Standard error HWE_p = P-value for Hardy-Weinberg equilibrium test

Phenotype	Description	Group	SNP	Beta	SE	Odds tatio	P-value	Type	n_total	n_cases	n_controls	HWE_p	allele_freq
150	Cancer of esophagus	neoplasms	rs12509595	-0.060	0.042	0.942	1.526E-01	logistic	395291	1429	393862	0.998	0.289
577	Diseases of pancreas	digestive	rs12509595	0.039	0.027	1.040	1.533E-01	logistic	421247	3239	418008	0.992	0.290
509	Respiratory failure, insufficiency, arrest	respiratory	rs12509595	-0.029	0.020	0.971	1.536E-01	logistic	397038	5981	391057	0.996	0.289
281.11	Pernicious anemia	hematopoietic	rs12509595	-0.083	0.058	0.920	1.548E-01	logistic	383186	737	382449	0.983	0.290
371.3	Inflammation of eyelids	sense organs	rs12509595	0.084	0.059	1.087	1.562E-01	logistic	411465	672	410793	0.988	0.289
270.32	Paraproteinemia	endocrine/metabolic	rs12509595	0.087	0.062	1.091	1.564E-01	logistic	422319	617	421702	0.993	0.290
281.13	Folate-deficiency anemia	hematopoietic	rs12509595	-0.116	0.082	0.891	1.572E-01	logistic	382829	380	382449	0.978	0.290
441.1	Acute vascular insufficiency of intestine	circulatory system	rs12509595	0.123	0.087	1.131	1.584E-01	logistic	405771	302	405469	0.991	0.289
965	Poisoning by analgesics, antipyretics, and antirheumatics	injuries & poisonings	rs12509595	-0.032	0.023	0.968	1.587E-01	logistic	371918	4745	367173	0.994	0.289
696	Psoriasis and related disorders	dermatologic	rs12509595	0.040	0.029	1.041	1.591E-01	logistic	402206	2916	399290	0.995	0.289
418.1	Precordial pain	circulatory system	rs12509595	0.050	0.036	1.051	1.610E-01	logistic	417539	1859	415680	0.991	0.289
285	Other anemias	hematopoietic	rs12509595	-0.021	0.015	0.979	1.631E-01	logistic	393921	11472	382449	0.990	0.290
428.1	Congestive heart failure (CHF) NOS	circulatory system	rs12509595	0.035	0.025	1.035	1.639E-01	logistic	413649	3946	409703	0.989	0.289
580.2	Nephrotic syndrome without mention of glomerulonephritis	genitourinary	rs12509595	0.106	0.077	1.112	1.652E-01	logistic	381457	395	381062	0.989	0.290
627.1	Postmenopausal bleeding	genitourinary	rs12509595	-0.052	0.037	0.950	1.658E-01	logistic	194538	1791	192747	0.993	0.290
442.11	Abdominal aortic aneurysm	circulatory system	rs12509595	0.052	0.038	1.054	1.690E-01	logistic	407127	1658	405469	0.992	0.289
550.5	Ventral hernia	digestive	rs12509595	-0.054	0.039	0.948	1.690E-01	logistic	367031	1637	365394	0.985	0.290
557.1	Celiac disease	digestive	rs12509595	-0.050	0.036	0.951	1.691E-01	logistic	336383	1898	334485	0.994	0.289
709	Diffuse diseases of connective tissue	dermatologic	rs12509595	0.063	0.046	1.065	1.708E-01	logistic	405028	1129	403899	0.996	0.289
276.4	Acid-base balance disorder	endocrine/metabolic	rs12509595	-0.047	0.034	0.954	1.714E-01	logistic	397692	2120	395572	0.993	0.290
420	Carditis	circulatory system	rs12509595	0.045	0.033	1.046	1.731E-01	logistic	418942	2172	416770	0.992	0.289
611	Abnormal findings on mammogram or breast exam	genitourinary	rs12509595	-0.134	0.099	0.875	1.756E-01	logistic	415016	264	414752	0.989	0.290
611.3	Lump or mass in breast	genitourinary	rs12509595	-0.134	0.099	0.875	1.756E-01	logistic	415016	264	414752	0.989	0.290
696.4	Psoriasis	dermatologic	rs12509595	0.039	0.029	1.040	1.764E-01	logistic	402177	2887	399290	0.995	0.289
801.1	Fracture of foot	injuries & poisonings	rs12509595	0.138	0.102	1.148	1.773E-01	logistic	396597	219	396378	0.981	0.289
149.4	Cancer of larynx	neoplasms	rs12509595	-0.130	0.096	0.878	1.790E-01	logistic	420618	276	420342	0.991	0.290
327.3	Sleep apnea	neurological	rs12509595	0.029	0.021	1.029	1.801E-01	logistic	418773	5389	413384	0.993	0.290
371.1	Uveitis, noninfectious or NOS	sense organs	rs12509595	0.127	0.095	1.135	1.825E-01	logistic	411047	254	410793	0.990	0.289
371	Inflammation of the eye	sense organs	rs12509595	0.064	0.048	1.066	1.832E-01	logistic	411823	1030	410793	0.989	0.289
709.2	Sicca syndrome	dermatologic	rs12509595	0.075	0.056	1.078	1.840E-01	logistic	404638	739	403899	0.996	0.289
427.9	Palpitations	circulatory system	rs12509595	0.049	0.037	1.050	1.841E-01	logistic	373589	1741	371848	0.995	0.289
375	Disorders of lacrimal system	sense organs	rs12509595	0.054	0.040	1.055	1.843E-01	logistic	413379	1451	411928	0.985	0.289
394	Rheumatic disease of the heart valves	circulatory system	rs12509595	0.030	0.023	1.030	1.859E-01	logistic	406218	4804	401414	0.991	0.289
740.2	Osteoarthrosis, generalized	musculoskeletal	rs12509595	0.030	0.023	1.031	1.875E-01	logistic	350458	4600	345858	0.992	0.290
496	Chronic airway obstruction	respiratory	rs12509595	0.016	0.012	1.016	1.905E-01	logistic	416701	17892	398809	0.991	0.289
300.1	Anxiety disorder	mental disorders	rs12509595	-0.021	0.017	0.979	1.937E-01	logistic	390805	9183	381622	0.994	0.289
559	Ileostomy status	digestive	rs12509595	-0.048	0.037	0.953	1.955E-01	logistic	336295	1810	334485	0.996	0.289
290.13	Senile dementia	mental disorders	rs12509595	0.042	0.032	1.043	1.957E-01	logistic	407253	2280	404973	0.992	0.290
555.1	Regional enteritis	digestive	rs12509595	-0.048	0.037	0.953	1.967E-01	logistic	336302	1817	334485	0.997	0.289
592.12	Chronic cystitis	genitourinary	rs12509595	-0.131	0.102	0.877	1.970E-01	logistic	377176	249	376927	0.983	0.290
200.1	Polycythemia vera	neoplasms	rs12509595	-0.090	0.069	0.914	1.970E-01	logistic	408705	523	408182	0.995	0.289
274	Gout and other crystal arthropathies	endocrine/metabolic	rs12509595	-0.030	0.024	0.970	1.990E-01	logistic	420276	4486	415790	0.987	0.290
443.9	Peripheral vascular disease, unspecified	circulatory system	rs12509595	0.033	0.026	1.034	2.011E-01	logistic	409046	3577	405469	0.991	0.289
159	Malignant neoplasm of other and ill-defined sites within the digestive organs and peritoneum	neoplasms	rs12509595	0.066	0.052	1.069	2.015E-01	logistic	394736	874	393862	0.999	0.290
270	Disorders of protein plasma/amino-acid transport and metabolism	endocrine/metabolic	rs12509595	0.070	0.055	1.072	2.027E-01	logistic	422490	788	421702	0.993	0.290
703.1	Ingrowing nail	dermatologic	rs12509595	-0.143	0.112	0.867	2.034E-01	logistic	421519	205	421314	0.993	0.289
474	Acute and chronic tonsillitis	respiratory	rs12509595	-0.104	0.082	0.901	2.034E-01	logistic	396895	378	396517	0.990	0.289
610	Benign mammary dysplasias	genitourinary	rs12509595	-0.090	0.071	0.914	2.036E-01	logistic	220777	505	220272	0.979	0.290
250.2	Type 2 diabetes	endocrine/metabolic	rs12509595	0.013	0.010	1.013	2.039E-01	logistic	413206	26569	386637	0.989	0.290
696.41	Psoriasis vulgaris	dermatologic	rs12509595	0.043	0.034	1.044	2.042E-01	logistic	401395	2105	399290	0.996	0.289
960.2	Allergy/adverse effect of penicillin	injuries & poisonings	rs12509595	0.015	0.012	1.015	2.043E-01	logistic	385833	18660	367173	0.994	0.289
586	Other disorders of the kidney and ureters	genitourinary	rs12509595	-0.032	0.025	0.968	2.064E-01	logistic	384904	3842	381062	0.988	0.290
368.2	Diplopia and disorders of binocular vision	sense organs	rs12509595	0.084	0.067	1.088	2.074E-01	logistic	416069	524	415545	0.989	0.289
477	Epistaxis or throat hemorrhage	respiratory	rs12509595	0.054	0.043	1.056	2.081E-01	logistic	397807	1290	396517	0.988	0.289
591	Urinary tract infection	genitourinary	rs12509595	-0.018	0.015	0.982	2.122E-01	logistic	388723	11796	376927	0.980	0.290
555	Inflammatory bowel disease and other gastroenteritis and colitis	digestive	rs12509595	-0.029	0.023	0.972	2.125E-01	logistic	339234	4749	334485	0.998	0.289
427.12	Paroxysmal ventricular tachycardia	circulatory system	rs12509595	0.061	0.049	1.063	2.145E-01	logistic	372834	986	371848	0.993	0.289
550	Abdominal hernia	digestive	rs12509595	-0.012	0.010	0.988	2.156E-01	logistic	392811	27417	365394	0.969	0.289
333.8	Other degenerative diseases of the basal ganglia	neurological	rs12509595	-0.133	0.107	0.876	2.159E-01	logistic	399271	224	399047	0.990	0.290
594.2	Calculus of lower urinary tract	genitourinary	rs12509595	-0.082	0.066	0.921	2.161E-01	logistic	412949	573	412376	0.993	0.289
153.3	Malignant neoplasm of rectum, rectosigmoid junction, and anus	neoplasms	rs12509595	0.036	0.029	1.036	2.166E-01	logistic	373609	2889	370720	0.995	0.290
497	Bronchitis	respiratory	rs12509595	-0.114	0.093	0.892	2.178E-01	logistic	399105	296	398809	0.991	0.289
187.2	Malignant neoplasm of testis	neoplasms	rs12509595	-0.029	0.024	0.971	2.180E-01	logistic	184014	4620	179394	0.630	0.289
204.1	Lymphoid leukemia	neoplasms	rs12509595	0.062	0.051	1.064	2.265E-01	logistic	416514	901	415613	0.991	0.290
339	Other headache syndromes	neurological	rs12509595	-0.029	0.024	0.971	2.266E-01	logistic	409356	4169	405187	0.994	0.289
602	Other disorders of prostate	genitourinary	rs12509595	-0.105	0.086	0.901	2.266E-01	logistic	156802	341	156461	0.579	0.289
367.2	Astigmatism	sense organs	rs12509595	-0.032	0.027	0.968	2.307E-01	logistic	414418	3412	411006	0.988	0.290
353	Nerve root and plexus disorders	neurological	rs12509595	-0.109	0.091	0.897	2.321E-01	logistic	402720	305	402415	0.991	0.289
289.8	Polycythemia, secondary	hematopoietic	rs12509595	-0.088	0.074	0.916	2.338E-01	logistic	407605	466	407139	0.988	0.289
315	Develomental delays and disorders	mental disorders	rs12509595	0.069	0.059	1.071	2.446E-01	logistic	422717	680	422037	0.992	0.290
352.1	Trigeminal nerve disorders [CN5]	neurological	rs12509595	-0.083	0.071	0.921	2.458E-01	logistic	402912	497	402415	0.990	0.289

ST31; PheWAS results for the lead cis-pQTLs of the 4 proteins prioritized (ACOX1, FGF5, FURIN, and MST1).

SNP = Single Nucleotide Polymorphism; SE = Standard error HWE_p = P-value for Hardy-Weinberg equilibrium test

Phenotype	Description	Group	SNP	Beta	SE	Odds tatio	P-value	Type	n_total	n_cases	n_controls	HWE_p	allele_freq
798	Malaise and fatigue	symptoms	rs12509595	0.028	0.024	1.028	2.464E-01	logistic	418584	4264	414320	0.995	0.290
501	Pneumonitis due to inhalation of food or vomitus	respiratory	rs12509595	0.042	0.037	1.043	2.465E-01	logistic	392843	1786	391057	0.993	0.290
333.4	Torsion dystonia	neurological	rs12509595	0.102	0.088	1.107	2.469E-01	logistic	399349	302	399047	0.990	0.290
276.13	Hyperpotassemia	endocrine/metabolic	rs12509595	-0.044	0.038	0.957	2.488E-01	logistic	397299	1727	395572	0.994	0.290
286.7	Other and unspecified coagulation defects	hematopoietic	rs12509595	-0.113	0.098	0.893	2.509E-01	logistic	417818	265	417553	0.993	0.289
586.2	Cyst of kidney, acquired	genitourinary	rs12509595	-0.042	0.037	0.959	2.513E-01	logistic	382916	1854	381062	0.987	0.290
694	Dyschromia and Vitiligo	dermatologic	rs12509595	-0.105	0.092	0.900	2.546E-01	logistic	405505	299	405206	0.997	0.289
426.2	Atrioventricular [AV] block	circulatory system	rs12509595	0.029	0.026	1.030	2.549E-01	logistic	375580	3732	371848	0.994	0.289
741	Symptoms and disorders of the joints	musculoskeletal	rs12509595	0.044	0.039	1.045	2.575E-01	logistic	416612	1602	415010	0.989	0.289
246	Other disorders of thyroid	endocrine/metabolic	rs12509595	-0.103	0.092	0.902	2.610E-01	logistic	392532	302	392230	0.976	0.289
531.2	Gastric ulcer	digestive	rs12509595	0.042	0.038	1.043	2.630E-01	logistic	413012	1673	411339	0.985	0.290
41.2	Streptococcus infection	infectious diseases	rs12509595	0.044	0.040	1.045	2.637E-01	logistic	398155	1508	396647	0.995	0.290
575.7	Other disorders of gallbladder	digestive	rs12509595	-0.071	0.064	0.931	2.642E-01	logistic	395657	610	395047	0.971	0.290
320	Meningitis	neurological	rs12509595	-0.102	0.092	0.903	2.645E-01	logistic	422374	303	422071	0.992	0.289
378.5	Paralytic strabismus	sense organs	rs12509595	0.103	0.092	1.108	2.647E-01	logistic	412201	273	411928	0.989	0.289
250	Diabetes mellitus	endocrine/metabolic	rs12509595	0.011	0.010	1.011	2.654E-01	logistic	414239	27602	386637	0.988	0.290
367	Disorders of refraction and accommodation; blindness and low vision	sense organs	rs12509595	-0.022	0.020	0.978	2.662E-01	logistic	417170	6164	411006	0.990	0.290
456	Chronic venous insufficiency [CVI]	circulatory system	rs12509595	-0.105	0.094	0.901	2.685E-01	logistic	381250	284	380966	0.956	0.290
292.6	Hallucinations	mental disorders	rs12509595	0.091	0.082	1.095	2.692E-01	logistic	405317	344	404973	0.990	0.289
352	Disorders of other cranial nerves	neurological	rs12509595	-0.050	0.045	0.952	2.704E-01	logistic	403637	1222	402415	0.990	0.289
335	Multiple sclerosis	neurological	rs12509595	-0.045	0.041	0.956	2.704E-01	logistic	400514	1467	399047	0.990	0.289
386.3	Labyrinthitis	sense organs	rs12509595	0.090	0.082	1.094	2.713E-01	logistic	410012	347	409665	0.996	0.289
394.1	Mitral valve stenosis and aortic valve stenosis	circulatory system	rs12509595	0.051	0.046	1.052	2.723E-01	logistic	402530	1116	401414	0.994	0.289
342	Hemiplegia	neurological	rs12509595	0.035	0.032	1.035	2.738E-01	logistic	401449	2402	399047	0.988	0.290
357	Inflammatory and toxic neuropathy	neurological	rs12509595	0.038	0.035	1.039	2.753E-01	logistic	421163	1995	419168	0.993	0.290
622	Polyp of female genital organs	genitourinary	rs12509595	-0.034	0.031	0.966	2.754E-01	logistic	216102	2530	213572	0.981	0.290
385	Other disorders of middle ear and mastoid	sense organs	rs12509595	-0.099	0.091	0.906	2.757E-01	logistic	419023	307	418716	0.993	0.290
586.1	Anatomical abnormatilies of kidney and ureters	genitourinary	rs12509595	-0.112	0.103	0.894	2.777E-01	logistic	381301	239	381062	0.990	0.290
458	Hypotension	circulatory system	rs12509595	-0.019	0.018	0.981	2.782E-01	logistic	380778	8206	372572	0.995	0.289
145.2	Cancer of tongue	neoplasms	rs12509595	-0.086	0.080	0.917	2.815E-01	logistic	420733	391	420342	0.991	0.289
345.11	Generalized convulsive epilepsy	neurological	rs12509595	0.083	0.077	1.086	2.838E-01	logistic	399441	394	399047	0.988	0.290
525	Other diseases of the teeth and supporting structures	digestive	rs12509595	-0.076	0.071	0.926	2.846E-01	logistic	410027	491	409536	0.993	0.290
53	Herpes zoster	infectious diseases	rs12509595	0.086	0.081	1.090	2.854E-01	logistic	415563	359	415204	0.989	0.290
444.1	Arterial embolism and thrombosis of lower extremity artery	circulatory system	rs12509595	0.077	0.073	1.080	2.866E-01	logistic	405915	446	405469	0.990	0.289
965.1	Opiates and related narcotics causing adverse effects in therapeutic use	injuries & poisonings	rs12509595	-0.035	0.033	0.966	2.873E-01	logistic	369447	2274	367173	0.994	0.289
727.6	Rupture of tendon, nontraumatic	musculoskeletal	rs12509595	0.045	0.042	1.046	2.877E-01	logistic	390517	1343	389174	0.992	0.289
367.9	Blindness and low vision	sense organs	rs12509595	-0.057	0.053	0.945	2.890E-01	logistic	411881	875	411006	0.991	0.290
803	Fracture of upper limb	injuries & poisonings	rs12509595	0.025	0.024	1.026	2.924E-01	logistic	400593	4215	396378	0.983	0.290
427.3	Other specified cardiac dysrhythmias	circulatory system	rs12509595	0.032	0.030	1.032	2.936E-01	logistic	374456	2608	371848	0.992	0.289
378.1	Strabismus (not specified as paralytic)	sense organs	rs12509595	-0.102	0.097	0.903	2.939E-01	logistic	412196	268	411928	0.989	0.289
369.5	Conjunctivitis, infectious	sense organs	rs12509595	0.111	0.106	1.117	2.968E-01	logistic	410999	206	410793	0.990	0.289
556.1	Ulceration of intestine	digestive	rs12509595	-0.118	0.113	0.889	2.983E-01	logistic	334685	200	334485	0.997	0.289
575.2	Obstruction of bile duct	digestive	rs12509595	0.050	0.048	1.051	2.999E-01	logistic	396076	1029	395047	0.972	0.290
573.3	Hepatomegaly	digestive	rs12509595	-0.099	0.096	0.906	3.009E-01	logistic	400551	277	400274	0.989	0.290
599.3	Dysuria	genitourinary	rs12509595	0.087	0.084	1.091	3.025E-01	logistic	390438	332	390106	0.971	0.289
276.41	Acidosis	endocrine/metabolic	rs12509595	-0.038	0.037	0.963	3.046E-01	logistic	397376	1804	395572	0.994	0.290
575.9	Nonspecific abnormal findings on radiological and other examination of biliary tract	digestive	rs12509595	-0.108	0.106	0.897	3.049E-01	logistic	395275	228	395047	0.971	0.290
601.1	Prostatitis	genitourinary	rs12509595	-0.061	0.060	0.941	3.059E-01	logistic	157164	703	156461	0.486	0.289
252.1	Hyperparathyroidism	endocrine/metabolic	rs12509595	-0.050	0.049	0.951	3.065E-01	logistic	416271	1025	415246	0.992	0.290
600	Hyperplasia of prostate	genitourinary	rs12509595	0.015	0.015	1.015	3.067E-01	logistic	169018	12557	156461	0.250	0.289
528	Diseases of the oral soft tissues, excluding lesions specific for gingiva and tongue	digestive	rs12509595	0.072	0.072	1.075	3.119E-01	logistic	418169	459	417710	0.991	0.290
295	Schizophrenia and other psychotic disorders	mental disorders	rs12509595	-0.055	0.055	0.947	3.132E-01	logistic	382460	838	381622	0.987	0.289
599.2	Retention of urine	genitourinary	rs12509595	0.019	0.019	1.019	3.139E-01	logistic	396988	6882	390106	0.971	0.289
389.3	Degenerative and vascular disorders of ear	sense organs	rs12509595	-0.086	0.086	0.917	3.148E-01	logistic	408638	341	408297	0.984	0.290
535.6	Duodenitis	digestive	rs12509595	-0.037	0.037	0.963	3.149E-01	logistic	363993	1779	362214	0.944	0.289
275.3	Disorders of magnesium metabolism	endocrine/metabolic	rs12509595	-0.040	0.040	0.961	3.189E-01	logistic	417729	1544	416185	0.993	0.290
454.1	Varicose veins of lower extremity	circulatory system	rs12509595	0.023	0.023	1.023	3.192E-01	logistic	385486	4520	380966	0.966	0.290
601.12	Chronic prostatitis	genitourinary	rs12509595	-0.104	0.105	0.901	3.194E-01	logistic	156694	233	156461	0.495	0.289
184	Cancer of other female genital organs	neoplasms	rs12509595	-0.029	0.029	0.972	3.196E-01	logistic	207829	2992	204837	0.982	0.290
198.1	Secondary malignancy of lymph nodes	neoplasms	rs12509595	-0.017	0.018	0.983	3.221E-01	logistic	394015	8009	386006	0.990	0.290
727.1	Synovitis and tenosynovitis	musculoskeletal	rs12509595	0.085	0.087	1.089	3.242E-01	logistic	389486	312	389174	0.994	0.289
280.1	Iron deficiency anemias, unspecified or not due to blood loss	hematopoietic	rs12509595	-0.017	0.017	0.984	3.245E-01	logistic	391238	8789	382449	0.983	0.290
165.1	Cancer of bronchus; lung	neoplasms	rs12509595	0.024	0.024	1.024	3.251E-01	logistic	421452	4223	417229	0.994	0.290
290.1	Dementias	mental disorders	rs12509595	0.024	0.024	1.024	3.253E-01	logistic	409256	4283	404973	0.994	0.290
430	Intracranial hemorrhage	circulatory system	rs12509595	0.035	0.035	1.035	3.264E-01	logistic	404595	1910	402685	0.997	0.289
603	Other disorders of testis	genitourinary	rs12509595	0.070	0.071	1.072	3.280E-01	logistic	162246	468	161778	0.820	0.289
634	Miscarriage; stillbirth	pregnancy complications	rs12509595	0.057	0.059	1.059	3.295E-01	logistic	223299	707	222592	0.984	0.290
300.9	Posttraumatic stress disorder	mental disorders	rs12509595	0.092	0.095	1.097	3.319E-01	logistic	381881	259	381622	0.990	0.290
530.3	Stricture and stenosis of esophagus	digestive	rs12509595	-0.044	0.046	0.957	3.326E-01	logistic	357976	1188	356788	0.989	0.290
695	Erythematous conditions	dermatologic	rs12509595	-0.036	0.037	0.965	3.341E-01	logistic	407007	1801	405206	0.996	0.289
159.3	Malignant neoplasm of gallbladder and extrahepatic bile ducts	neoplasms	rs12509595	0.081	0.084	1.084	3.355E-01	logistic	394194	332	393862	0.999	0.290

ST31; PheWAS results for the lead cis-pQTLs of the 4 proteins prioritized (ACOX1, FGF5, FURIN, and MST1).

SNP = Single Nucleotide Polymorphism; SE = Standard error HWE_p = P-value for Hardy-Weinberg equilibrium test

Phenotype	Description	Group	SNP	Beta	SE	Odds tatio	P-value	Type	n_total	n_cases	n_controls	HWE_p	allele_freq
690	Erythematosquamous dermatosis	dermatologic	rs12509595	-0.061	0.063	0.941	3.363E-01	logistic	405831	625	405206	0.996	0.289
427.1	Paroxysmal tachycardia, unspecified	circulatory system	rs12509595	-0.027	0.028	0.973	3.367E-01	logistic	374981	3133	371848	0.993	0.289
279	Disorders involving the immune mechanism	endocrine/metabolic	rs12509595	0.076	0.080	1.079	3.402E-01	logistic	423247	371	422876	0.992	0.290
366.2	Senile cataract	sense organs	rs12509595	0.016	0.017	1.016	3.414E-01	logistic	380814	9136	371678	0.959	0.289
350.1	Abnormal involuntary movements	neurological	rs12509595	-0.053	0.056	0.948	3.421E-01	logistic	421010	800	420210	0.990	0.290
577.2	Chronic pancreatitis	digestive	rs12509595	0.059	0.063	1.061	3.437E-01	logistic	418611	603	418008	0.990	0.289
348.2	Cerebral edema and compression of brain	neurological	rs12509595	-0.077	0.081	0.926	3.440E-01	logistic	399425	378	399047	0.991	0.290
420.2	Pericarditis	circulatory system	rs12509595	0.038	0.040	1.039	3.446E-01	logistic	418236	1466	416770	0.991	0.290
348	Other conditions of brain	neurological	rs12509595	-0.058	0.061	0.944	3.449E-01	logistic	399717	670	399047	0.991	0.290
433.12	Cerebral atherosclerosis	circulatory system	rs12509595	0.078	0.083	1.081	3.450E-01	logistic	403029	344	402685	0.997	0.289
618.2	Uterine/Uterovaginal prolapse	genitourinary	rs12509595	-0.050	0.053	0.951	3.454E-01	logistic	214329	874	213455	0.982	0.290
530.14	Reflux esophagitis	digestive	rs12509595	0.026	0.027	1.026	3.457E-01	logistic	360032	3244	356788	0.987	0.290
159.4	Malignant neoplasm of retroperitoneum and peritoneum	neoplasms	rs12509595	0.088	0.094	1.092	3.464E-01	logistic	394127	265	393862	0.998	0.290
690.1	Seborrheic dermatitis	dermatologic	rs12509595	-0.060	0.064	0.942	3.488E-01	logistic	405818	612	405206	0.996	0.289
279.11	Deficiency of humoral immunity	endocrine/metabolic	rs12509595	0.092	0.098	1.096	3.493E-01	logistic	423118	242	422876	0.992	0.290
386.1	Meniere's disease	sense organs	rs12509595	0.057	0.061	1.058	3.507E-01	logistic	410308	643	409665	0.995	0.289
614.5	Inflammatory disease of cervix, vagina, and vulva	genitourinary	rs12509595	-0.064	0.069	0.938	3.510E-01	logistic	217969	531	217438	0.990	0.290
195	Cancer, suspected or other	neoplasms	rs12509595	-0.017	0.018	0.983	3.520E-01	logistic	393669	7663	386006	0.990	0.290
297.2	Suicide or self-inflicted injury	mental disorders	rs12509595	0.079	0.085	1.082	3.528E-01	logistic	381948	326	381622	0.989	0.290
290	Delirium dementia and amnestic and other cognitive disorders	mental disorders	rs12509595	0.017	0.019	1.017	3.537E-01	logistic	412158	7185	404973	0.996	0.290
722.9	Other and unspecified disc disorder	musculoskeletal	rs12509595	0.062	0.067	1.064	3.537E-01	logistic	409194	524	408670	0.991	0.290
523.3	Periodontitis (acute or chronic)	digestive	rs12509595	0.093	0.100	1.097	3.538E-01	logistic	409770	234	409536	0.992	0.290
374	Other disorders of eyelids	sense organs	rs12509595	0.030	0.033	1.031	3.543E-01	logistic	413055	2262	410793	0.989	0.289
362	Other retinal disorders	sense organs	rs12509595	0.015	0.016	1.015	3.552E-01	logistic	407698	9941	397757	0.993	0.289
250.4	Abnormal glucose	endocrine/metabolic	rs12509595	-0.045	0.049	0.956	3.565E-01	logistic	387669	1032	386637	0.988	0.289
244.4	Hypothyroidism NOS	endocrine/metabolic	rs12509595	-0.011	0.012	0.989	3.568E-01	logistic	410296	18066	392230	0.996	0.289
574.12	Cholelithiasis with other cholecystitis	digestive	rs12509595	-0.036	0.039	0.964	3.580E-01	logistic	396636	1589	395047	0.962	0.290
585.2	Renal failure NOS	genitourinary	rs12509595	-0.053	0.058	0.949	3.623E-01	logistic	381806	744	381062	0.989	0.290
283	Acquired hemolytic anemias	hematopoietic	rs12509595	0.098	0.108	1.103	3.631E-01	logistic	382649	200	382449	0.979	0.290
275	Disorders of mineral metabolism	endocrine/metabolic	rs12509595	-0.021	0.023	0.980	3.648E-01	logistic	420958	4773	416185	0.995	0.290
297	Suicidal ideation or attempt	mental disorders	rs12509595	0.073	0.081	1.076	3.657E-01	logistic	381982	360	381622	0.989	0.290
317.11	Alcoholic liver damage	mental disorders	rs12509595	-0.041	0.045	0.960	3.661E-01	logistic	404493	1200	403293	0.995	0.289
242.1	Graves' disease	endocrine/metabolic	rs12509595	0.072	0.080	1.075	3.683E-01	logistic	392598	368	392230	0.975	0.289
728.71	Contracture of palmar fascia [Dupuytren's disease]	musculoskeletal	rs12509595	0.034	0.038	1.034	3.690E-01	logistic	390879	1705	389174	0.991	0.289
276.6	Fluid overload	endocrine/metabolic	rs12509595	-0.035	0.038	0.966	3.691E-01	logistic	397239	1667	395572	0.995	0.290
710.1	Osteomyelitis	musculoskeletal	rs12509595	-0.094	0.105	0.910	3.709E-01	logistic	412277	229	412048	0.991	0.290
182	Malignant neoplasm of uterus	neoplasms	rs12509595	-0.040	0.045	0.961	3.716E-01	logistic	197217	1245	195972	0.988	0.290
756	Other congenital musculoskeletal anomalies	congenital anomalies	rs12509595	-0.045	0.050	0.956	3.743E-01	logistic	422256	974	421282	0.993	0.290
509.1	Respiratory failure	respiratory	rs12509595	-0.021	0.024	0.979	3.752E-01	logistic	395437	4380	391057	0.996	0.289
819	Skull and face fracture and other intercranial injury	injuries & poisonings	rs12509595	-0.062	0.070	0.939	3.753E-01	logistic	422666	501	422165	0.991	0.289
296.22	Major depressive disorder	mental disorders	rs12509595	0.011	0.013	1.011	3.753E-01	logistic	396820	15198	381622	0.994	0.290
427.6	Premature beats	circulatory system	rs12509595	0.056	0.063	1.057	3.765E-01	logistic	372449	601	371848	0.994	0.289
801	Fracture of ankle and foot	injuries & poisonings	rs12509595	-0.054	0.061	0.947	3.776E-01	logistic	397040	662	396378	0.984	0.289
681.1	Cellulitis and abscess of fingers/toes	dermatologic	rs12509595	0.089	0.101	1.094	3.779E-01	logistic	418724	227	418497	0.995	0.290
578.8	Hemorrhage of rectum and anus	digestive	rs12509595	-0.029	0.033	0.972	3.785E-01	logistic	385630	2327	383303	0.970	0.289
452	Other venous embolism and thrombosis	circulatory system	rs12509595	-0.077	0.088	0.926	3.814E-01	logistic	381290	324	380966	0.953	0.290
112	Candidiasis	infectious diseases	rs12509595	-0.035	0.040	0.966	3.831E-01	logistic	419935	1555	418380	0.993	0.290
202	Cancer of other lymphoid, histiocytic tissue	neoplasms	rs12509595	-0.027	0.030	0.974	3.832E-01	logistic	418277	2664	415613	0.994	0.290
202.2	Non-Hodgkins lymphoma	neoplasms	rs12509595	-0.027	0.030	0.974	3.832E-01	logistic	418277	2664	415613	0.994	0.290
800.1	Fracture of neck of femur	injuries & poisonings	rs12509595	0.030	0.034	1.030	3.836E-01	logistic	398432	2054	396378	0.976	0.289
574	Cholelithiasis and cholecystitis	digestive	rs12509595	-0.013	0.015	0.987	3.892E-01	logistic	406113	11066	395047	0.979	0.290
159.2	Malignant neoplasm of small intestine, including duodenum	neoplasms	rs12509595	0.082	0.096	1.086	3.894E-01	logistic	394117	255	393862	0.998	0.290
415.21	Primary pulmonary hypertension	circulatory system	rs12509595	-0.072	0.084	0.931	3.944E-01	logistic	413189	351	412838	0.998	0.289
550.6	Incisional hernia	digestive	rs12509595	0.061	0.072	1.063	3.956E-01	logistic	365851	457	365394	0.985	0.290
195.1	Malignant neoplasm, other	neoplasms	rs12509595	-0.015	0.018	0.985	3.959E-01	logistic	393640	7634	386006	0.990	0.290
350	Abnormal movement	neurological	rs12509595	-0.038	0.045	0.962	3.964E-01	logistic	421410	1200	420210	0.991	0.290
473.4	Voice disturbance	respiratory	rs12509595	0.077	0.091	1.080	3.973E-01	logistic	396801	284	396517	0.989	0.289
427.41	Ventricular fibrillation and flutter	circulatory system	rs12509595	0.080	0.094	1.083	3.974E-01	logistic	372113	265	371848	0.993	0.289
626.12	Excessive or frequent menstruation	genitourinary	rs12509595	-0.028	0.034	0.972	4.019E-01	logistic	194922	2175	192747	0.991	0.290
201	Hodgkin's disease	neoplasms	rs12509595	-0.079	0.094	0.924	4.026E-01	logistic	415895	282	415613	0.992	0.290
577.3	Cyst and pseudocyst of pancreas	digestive	rs12509595	0.057	0.068	1.058	4.042E-01	logistic	418522	514	418008	0.991	0.289
430.2	Intracerebral hemorrhage	circulatory system	rs12509595	0.039	0.047	1.040	4.052E-01	logistic	403773	1088	402685	0.996	0.289
764	Sciatica	symptoms	rs12509595	-0.036	0.043	0.965	4.052E-01	logistic	419302	1316	417986	0.995	0.290
573.5	Jaundice (not of newborn)	digestive	rs12509595	0.049	0.058	1.050	4.056E-01	logistic	400970	696	400274	0.986	0.290
851	Complications of transplants and reattached limbs	injuries & poisonings	rs12509595	0.082	0.098	1.085	4.057E-01	logistic	416895	243	416652	0.993	0.290
187.1	Malignant neoplasm of unspecified male genital organ	neoplasms	rs12509595	-0.020	0.024	0.981	4.072E-01	logistic	183955	4561	179394	0.634	0.290
947	Urticaria	dermatologic	rs12509595	0.088	0.106	1.091	4.076E-01	logistic	415196	209	414987	0.994	0.290
750.1	Upper gastrointestinal congenital anomalies	congenital anomalies	rs12509595	-0.088	0.109	0.915	4.182E-01	logistic	420464	211	420253	0.991	0.290
244	Hypothyroidism	endocrine/metabolic	rs12509595	-0.009	0.012	0.991	4.196E-01	logistic	411296	19066	392230	0.997	0.289
351	Other peripheral nerve disorders	neurological	rs12509595	0.015	0.019	1.016	4.199E-01	logistic	409126	6711	402415	0.991	0.289
735.23	Hallux rigidus	musculoskeletal	rs12509595	0.069	0.086	1.072	4.209E-01	logistic	402973	319	402654	0.981	0.289

ST31; PheWAS results for the lead cis-pQTLs of the 4 proteins prioritized (ACOX1, FGF5, FURIN, and MST1).

SNP = Single Nucleotide Polymorphism; SE = Standard error HWE_p = P-value for Hardy-Weinberg equilibrium test

Phenotype	Description	Group	SNP	Beta	SE	Odds tatio	P-value	Type	n_total	n_cases	n_controls	HWE_p	allele_freq
225.1	Benign neoplasm of brain, cranial nerves, meninges	neoplasms	rs12509595	0.044	0.054	1.045	4.215E-01	logistic	421204	807	420397	0.990	0.290
260.6	Anorexia	endocrine/metabolic	rs12509595	0.042	0.052	1.043	4.217E-01	logistic	412968	871	412097	0.993	0.290
527	Diseases of the salivary glands	digestive	rs12509595	0.082	0.102	1.086	4.220E-01	logistic	417933	223	417710	0.993	0.290
522.5	Periapical abscess	digestive	rs12509595	-0.088	0.110	0.915	4.223E-01	logistic	409744	208	409536	0.992	0.290
225	Benign neoplasm of brain and other parts of nervous system	neoplasms	rs12509595	0.043	0.053	1.044	4.231E-01	logistic	421233	836	420397	0.990	0.290
728.7	Fasciitis	musculoskeletal	rs12509595	0.029	0.036	1.029	4.251E-01	logistic	391018	1844	389174	0.991	0.289
450	Noninfectious disorders of lymphatic channels	circulatory system	rs12509595	0.036	0.045	1.037	4.266E-01	logistic	422720	1167	421553	0.993	0.290
580	Nephritis; nephrosis; renal sclerosis	genitourinary	rs12509595	0.037	0.047	1.038	4.280E-01	logistic	382137	1075	381062	0.990	0.290
280	Iron deficiency anemias	hematopoietic	rs12509595	-0.013	0.017	0.987	4.295E-01	logistic	391534	9085	382449	0.982	0.290
300	Anxiety disorders	mental disorders	rs12509595	-0.013	0.016	0.987	4.300E-01	logistic	391438	9816	381622	0.995	0.289
184.11	Malignant neoplasm of ovary	neoplasms	rs12509595	-0.024	0.030	0.977	4.323E-01	logistic	207552	2715	204837	0.983	0.290
523	Gingival and periodontal diseases	digestive	rs12509595	0.061	0.077	1.063	4.328E-01	logistic	409931	395	409536	0.992	0.290
560.3	Peritoneal or intestinal adhesions	digestive	rs12509595	-0.043	0.054	0.958	4.330E-01	logistic	335317	832	334485	0.996	0.289
41.1	Staphylococcus infections	infectious diseases	rs12509595	0.025	0.032	1.025	4.344E-01	logistic	398981	2334	396647	0.993	0.290
530.11	GERD	digestive	rs12509595	-0.010	0.013	0.990	4.354E-01	logistic	371656	14868	356788	0.991	0.289
577.1	Acute pancreatitis	digestive	rs12509595	0.028	0.036	1.028	4.370E-01	logistic	419858	1850	418008	0.991	0.289
38.2	Gram positive septicemia	infectious diseases	rs12509595	-0.049	0.063	0.952	4.372E-01	logistic	397268	621	396647	0.995	0.290
574.3	Cholecystitis without cholelithiasis	digestive	rs12509595	-0.035	0.045	0.965	4.373E-01	logistic	396244	1197	395047	0.972	0.290
686	Other local infections of skin and subcutaneous tissue	dermatologic	rs12509595	0.037	0.048	1.038	4.384E-01	logistic	419554	1057	418497	0.995	0.290
596.1	Bladder neck obstruction	genitourinary	rs12509595	-0.051	0.067	0.950	4.397E-01	logistic	402115	561	401554	0.973	0.289
747.11	Cardiac shunt/ heart septal defect	congenital anomalies	rs12509595	0.056	0.073	1.058	4.397E-01	logistic	422041	446	421595	0.993	0.290
184.1	Malignant neoplasm of ovary and other uterine adnexa	neoplasms	rs12509595	-0.023	0.030	0.977	4.397E-01	logistic	207626	2789	204837	0.982	0.290
70	Viral hepatitis	infectious diseases	rs12509595	0.051	0.066	1.052	4.419E-01	logistic	415755	551	415204	0.988	0.290
446.5	Giant cell arteritis	circulatory system	rs12509595	0.048	0.063	1.049	4.451E-01	logistic	406071	602	405469	0.993	0.289
198.7	Secondary malignant neoplasm of skin	neoplasms	rs12509595	0.072	0.095	1.074	4.498E-01	logistic	386268	262	386006	0.988	0.290
331.1	Hydrocephalus	neurological	rs12509595	-0.048	0.063	0.953	4.500E-01	logistic	399668	621	399047	0.991	0.290
276.12	Hyposmolality and/or hyponatremia	endocrine/metabolic	rs12509595	-0.017	0.022	0.983	4.504E-01	logistic	400551	4979	395572	0.994	0.290
276.11	Hyperosmolality and/or hypernatremia	endocrine/metabolic	rs12509595	0.044	0.058	1.044	4.510E-01	logistic	396286	714	395572	0.994	0.290
695.9	Unspecified erythematous condition	dermatologic	rs12509595	-0.066	0.088	0.936	4.533E-01	logistic	405524	318	405206	0.997	0.289
374.1	Ectropion or entropion	sense organs	rs12509595	0.044	0.058	1.045	4.544E-01	logistic	411493	700	410793	0.990	0.289
379.2	Disorders of vitreous body	sense organs	rs12509595	0.057	0.076	1.059	4.553E-01	logistic	412333	405	411928	0.989	0.289
255	Disorders of adrenal glands	endocrine/metabolic	rs12509595	-0.042	0.056	0.959	4.572E-01	logistic	416034	788	415246	0.993	0.290
579.2	Splenomegaly	digestive	rs12509595	-0.047	0.063	0.954	4.590E-01	logistic	383919	616	383303	0.973	0.289
447.1	Stricture of artery	circulatory system	rs12509595	0.046	0.062	1.047	4.600E-01	logistic	406083	614	405469	0.990	0.289
386.9	Dizziness and giddiness (Light-headedness and vertigo)	sense organs	rs12509595	0.021	0.028	1.021	4.605E-01	logistic	412711	3046	409665	0.995	0.289
528.11	Stomatitis and mucositis (ulcerative)	digestive	rs12509595	0.078	0.106	1.081	4.616E-01	logistic	417919	209	417710	0.991	0.290
352.2	Facial nerve disorders [CN7]	neurological	rs12509595	-0.044	0.060	0.957	4.619E-01	logistic	403092	677	402415	0.990	0.289
702.2	Seborrheic keratosis	dermatologic	rs12509595	-0.050	0.068	0.951	4.628E-01	logistic	413611	539	413072	0.986	0.290
681	Superficial cellulitis and abscess	dermatologic	rs12509595	-0.067	0.091	0.936	4.635E-01	logistic	418799	302	418497	0.995	0.290
286.1	Congenital coagulation defects	hematopoietic	rs12509595	0.055	0.075	1.056	4.652E-01	logistic	417976	423	417553	0.994	0.289
961.1	Poisoning/allergy of sulfonamides	injuries & poisonings	rs12509595	-0.045	0.062	0.956	4.681E-01	logistic	367807	634	367173	0.995	0.289
790	Nonspecific findings on examination of blood	symptoms	rs12509595	0.069	0.096	1.071	4.728E-01	logistic	422095	255	421840	0.993	0.290
364	Corneal opacity and other disorders of cornea	sense organs	rs12509595	0.027	0.037	1.027	4.740E-01	logistic	399513	1750	397763	0.983	0.289
211	Benign neoplasm of other parts of digestive system	neoplasms	rs12509595	0.019	0.026	1.019	4.761E-01	logistic	400517	3467	397050	0.998	0.290
939	Atopic/contact dermatitis due to other or unspecified	dermatologic	rs12509595	0.025	0.035	1.025	4.779E-01	logistic	416950	1963	414987	0.996	0.290
204.12	Lymphoid leukemia, chronic	neoplasms	rs12509595	0.038	0.054	1.039	4.789E-01	logistic	416428	815	415613	0.991	0.290
287	Purpura and other hemorrhagic conditions	hematopoietic	rs12509595	-0.023	0.032	0.977	4.798E-01	logistic	419896	2343	417553	0.993	0.289
292.1	Aphasia/speech disturbance	mental disorders	rs12509595	0.034	0.048	1.034	4.832E-01	logistic	406020	1047	404973	0.986	0.289
514	Abnormal findings examination of lungs	respiratory	rs12509595	0.023	0.033	1.024	4.841E-01	logistic	419804	2147	417657	0.994	0.289
200	Myeloproliferative disease	neoplasms	rs12509595	0.028	0.040	1.028	4.854E-01	logistic	417103	1490	415613	0.993	0.290
389.4	Tinnitus	sense organs	rs12509595	0.049	0.071	1.050	4.877E-01	logistic	408770	473	408297	0.983	0.290
458.9	Hypotension NOS	circulatory system	rs12509595	-0.016	0.024	0.984	4.887E-01	logistic	376953	4381	372572	0.995	0.289
362.29	Macular degeneration (senile) of retina NOS	sense organs	rs12509595	0.017	0.025	1.017	4.894E-01	logistic	401665	3908	397757	0.994	0.289
604	Disorders of penis	genitourinary	rs12509595	0.048	0.070	1.049	4.898E-01	logistic	162266	488	161778	0.842	0.289
564	Functional digestive disorders	digestive	rs12509595	0.008	0.011	1.008	4.918E-01	logistic	354387	19902	334485	0.997	0.290
555.21	Ulcerative colitis (chronic)	digestive	rs12509595	-0.055	0.081	0.946	4.941E-01	logistic	334866	381	334485	0.997	0.289
596	Other disorders of bladder	genitourinary	rs12509595	-0.014	0.020	0.986	4.954E-01	logistic	407805	6251	401554	0.982	0.289
174.11	Malignant neoplasm of female breast	neoplasms	rs12509595	-0.011	0.017	0.989	4.956E-01	logistic	212959	9200	203759	0.959	0.290
175	Acquired absence of breast	neoplasms	rs12509595	0.017	0.025	1.017	4.963E-01	logistic	206502	3992	202510	0.964	0.290
446.4	Wegener's granulomatosis	circulatory system	rs12509595	0.068	0.100	1.070	5.004E-01	logistic	405703	234	405469	0.992	0.289
535.8	Other specified gastritis	digestive	rs12509595	0.020	0.029	1.020	5.019E-01	logistic	365017	2803	362214	0.932	0.289
344	Other paralytic syndromes	neurological	rs12509595	-0.041	0.061	0.960	5.036E-01	logistic	399714	667	399047	0.989	0.290
385.3	Cholesteatoma	sense organs	rs12509595	-0.071	0.106	0.931	5.039E-01	logistic	418937	221	418716	0.993	0.290
359	Muscular dystrophies and other myopathies	neurological	rs12509595	0.062	0.093	1.064	5.044E-01	logistic	419442	274	419168	0.995	0.289
579	Other symptoms involving abdomen and pelvis	digestive	rs12509595	-0.032	0.049	0.968	5.075E-01	logistic	384340	1037	383303	0.975	0.289
174.1	Breast cancer [female]	neoplasms	rs12509595	-0.011	0.016	0.990	5.076E-01	logistic	213896	10137	203759	0.959	0.290
8	Intestinal infection	infectious diseases	rs12509595	0.023	0.035	1.024	5.089E-01	logistic	419231	1915	417316	0.995	0.290
528.1	Stomatitis and mucositis	digestive	rs12509595	0.066	0.100	1.068	5.092E-01	logistic	417947	237	417710	0.992	0.290
990	Effects radiation NOS	injuries & poisonings	rs12509595	0.012	0.019	1.012	5.101E-01	logistic	408877	6919	401958	0.987	0.290
276.5	Hypovolemia	endocrine/metabolic	rs12509595	-0.014	0.022	0.986	5.109E-01	logistic	400853	5281	395572	0.994	0.290
187	Cancer of other male genital organs	neoplasms	rs12509595	0.012	0.018	1.012	5.112E-01	logistic	186921	7527	179394	0.705	0.289

ST31; PheWAS results for the lead cis-pQTLs of the 4 proteins prioritized (ACOX1, FGF5, FURIN, and MST1).

SNP = Single Nucleotide Polymorphism; SE = Standard error HWE_p = P-value for Hardy-Weinberg equilibrium test

Phenotype	Description	Group	SNP	Beta	SE	Odds tatio	P-value	Type	n_total	n_cases	n_controls	HWE_p	allele_freq
760	Back pain	symptoms	rs12509595	0.014	0.022	1.014	5.127E-01	logistic	414925	5193	409732	0.992	0.290
471	Nasal polyps	respiratory	rs12509595	-0.032	0.049	0.969	5.138E-01	logistic	397549	1032	396517	0.986	0.289
618	Genital prolapse	genitourinary	rs12509595	-0.013	0.020	0.987	5.157E-01	logistic	219680	6225	213455	0.976	0.290
427.7	Tachycardia NOS	circulatory system	rs12509595	0.023	0.036	1.023	5.169E-01	logistic	373741	1893	371848	0.992	0.289
198	Secondary malignant neoplasm	neoplasms	rs12509595	-0.008	0.012	0.992	5.190E-01	logistic	403287	17281	386006	0.987	0.290
550.2	Diaphragmatic hernia	digestive	rs12509595	-0.007	0.011	0.993	5.223E-01	logistic	388258	22864	365394	0.976	0.290
145	Cancer of mouth	neoplasms	rs12509595	-0.039	0.060	0.962	5.232E-01	logistic	421016	674	420342	0.991	0.290
401.22	Hypertensive chronic kidney disease	circulatory system	rs12509595	0.032	0.050	1.032	5.233E-01	logistic	291632	969	290663	0.999	0.284
555.2	Ulcerative colitis	digestive	rs12509595	-0.018	0.028	0.982	5.244E-01	logistic	337679	3194	334485	0.998	0.289
701.2	Scar conditions and fibrosis of skin	dermatologic	rs12509595	-0.051	0.080	0.951	5.247E-01	logistic	415576	390	415186	0.996	0.290
429	Ill-defined descriptions and complications of heart disease	circulatory system	rs12509595	-0.055	0.086	0.947	5.251E-01	logistic	410037	334	409703	0.986	0.289
531.1	Hemorrhage from gastrointestinal ulcer	digestive	rs12509595	-0.042	0.066	0.959	5.254E-01	logistic	411913	574	411339	0.985	0.290
174	Breast cancer	neoplasms	rs12509595	-0.010	0.016	0.990	5.256E-01	logistic	401305	10248	391057	0.976	0.290
502	Postinflammatory pulmonary fibrosis	respiratory	rs12509595	-0.022	0.035	0.978	5.281E-01	logistic	393073	2016	391057	0.993	0.290
994	Sepsis and SIRS	injuries & poisonings	rs12509595	0.012	0.019	1.012	5.305E-01	logistic	418535	6755	411780	0.991	0.290
994.2	Sepsis	injuries & poisonings	rs12509595	0.012	0.019	1.012	5.305E-01	logistic	418535	6755	411780	0.991	0.290
531	Peptic ulcer (excl. esophageal)	digestive	rs12509595	0.015	0.025	1.016	5.332E-01	logistic	415329	3990	411339	0.987	0.290
285.22	Anemia in neoplastic disease	hematopoietic	rs12509595	-0.054	0.086	0.948	5.351E-01	logistic	382780	331	382449	0.981	0.290
292.4	Altered mental status	mental disorders	rs12509595	0.016	0.027	1.016	5.386E-01	logistic	408434	3461	404973	0.983	0.290
41	Bacterial infection NOS	infectious diseases	rs12509595	0.010	0.016	1.010	5.402E-01	logistic	406056	9409	396647	0.988	0.290
800	Fracture of lower limb	injuries & poisonings	rs12509595	-0.017	0.028	0.983	5.402E-01	logistic	399482	3104	396378	0.979	0.289
560.2	Impaction of intestine	digestive	rs12509595	0.062	0.101	1.064	5.404E-01	logistic	334714	229	334485	0.996	0.289
571	Chronic liver disease and cirrhosis	digestive	rs12509595	-0.013	0.021	0.988	5.408E-01	logistic	406135	5861	400274	0.989	0.290
573	Other disorders of liver	digestive	rs12509595	-0.021	0.035	0.979	5.413E-01	logistic	402294	2020	400274	0.991	0.290
296.2	Depression	mental disorders	rs12509595	0.006	0.010	1.006	5.427E-01	logistic	408593	26971	381622	0.992	0.290
443	Peripheral vascular disease	circulatory system	rs12509595	0.013	0.022	1.013	5.438E-01	logistic	410595	5126	405469	0.991	0.289
741.4	Joint effusions	musculoskeletal	rs12509595	-0.036	0.060	0.964	5.443E-01	logistic	415693	683	415010	0.990	0.289
255.2	Adrenal hypofunction	endocrine/metabolic	rs12509595	-0.051	0.084	0.951	5.471E-01	logistic	415595	349	415246	0.992	0.290
255.21	Glucocorticoid deficiency	endocrine/metabolic	rs12509595	-0.051	0.084	0.951	5.471E-01	logistic	415595	349	415246	0.992	0.290
292.3	Memory loss	mental disorders	rs12509595	0.041	0.069	1.042	5.511E-01	logistic	405478	505	404973	0.989	0.289
41.11	Methicillin sensitive Staphylococcus aureus	infectious diseases	rs12509595	0.023	0.038	1.023	5.521E-01	logistic	398282	1635	396647	0.994	0.290
358	Myoneural disorders	neurological	rs12509595	-0.052	0.088	0.949	5.526E-01	logistic	419485	317	419168	0.994	0.289
227.1	Benign neoplasm of adrenal gland	neoplasms	rs12509595	0.057	0.096	1.058	5.542E-01	logistic	421144	257	420887	0.991	0.290
300.12	Agorophobia, social phobia, and panic disorder	mental disorders	rs12509595	-0.036	0.061	0.965	5.549E-01	logistic	382286	664	381622	0.992	0.289
429.2	Abnormal function study of cardiovascular system	circulatory system	rs12509595	-0.051	0.086	0.951	5.573E-01	logistic	410036	333	409703	0.986	0.289
241	Nontoxic nodular goiter	endocrine/metabolic	rs12509595	-0.033	0.057	0.968	5.614E-01	logistic	392996	766	392230	0.978	0.289
752.1	Neural tube defects	congenital anomalies	rs12509595	-0.046	0.079	0.955	5.630E-01	logistic	422933	394	422539	0.991	0.290
569.2	Gastrointestinal complications	digestive	rs12509595	-0.055	0.095	0.947	5.631E-01	logistic	384117	278	383839	0.998	0.289
743.21	Pathologic fracture of vertebrae	musculoskeletal	rs12509595	-0.036	0.063	0.965	5.680E-01	logistic	421861	627	421234	0.992	0.289
580.14	Chronic glomerulonephritis, NOS	genitourinary	rs12509595	-0.051	0.089	0.951	5.685E-01	logistic	381376	314	381062	0.990	0.290
687.4	Disturbance of skin sensation	dermatologic	rs12509595	0.024	0.042	1.024	5.692E-01	logistic	414113	1397	412716	0.985	0.289
596.5	Functional disorders of bladder	genitourinary	rs12509595	-0.035	0.062	0.966	5.717E-01	logistic	402194	640	401554	0.974	0.290
227	Benign neoplasm of other endocrine glands and related structures	neoplasms	rs12509595	0.033	0.058	1.033	5.757E-01	logistic	421596	709	420887	0.991	0.290
720.1	Spinal stenosis of lumbar region	musculoskeletal	rs12509595	0.017	0.031	1.017	5.770E-01	logistic	411268	2598	408670	0.989	0.290
835	Internal derangement of knee	injuries & poisonings	rs12509595	-0.013	0.023	0.987	5.778E-01	logistic	409924	4709	405215	0.989	0.290
433.5	Cerebral aneurysm	circulatory system	rs12509595	-0.041	0.074	0.960	5.793E-01	logistic	403130	445	402685	0.996	0.289
368.4	Visual field defects	sense organs	rs12509595	0.041	0.073	1.041	5.794E-01	logistic	415988	443	415545	0.989	0.289
157	Pancreatic cancer	neoplasms	rs12509595	0.023	0.042	1.023	5.795E-01	logistic	395256	1394	393862	0.999	0.290
296	Mood disorders	mental disorders	rs12509595	0.005	0.010	1.005	5.815E-01	logistic	409186	27564	381622	0.989	0.290
443.1	Raynaud's syndrome	circulatory system	rs12509595	-0.022	0.039	0.979	5.817E-01	logistic	407046	1577	405469	0.991	0.289
204.4	Multiple myeloma	neoplasms	rs12509595	-0.025	0.046	0.975	5.835E-01	logistic	416757	1144	415613	0.991	0.290
427.8	Sinoatrial node dysfunction (Bradycardia)	circulatory system	rs12509595	-0.036	0.065	0.965	5.852E-01	logistic	372427	579	371848	0.994	0.289
698	Pruritus and related conditions	dermatologic	rs12509595	0.050	0.092	1.052	5.859E-01	logistic	422717	278	422439	0.992	0.290
766	Neuralgia, neuritis, and radiculitis NOS	symptoms	rs12509595	-0.051	0.094	0.950	5.864E-01	logistic	418267	281	417986	0.994	0.290
530.1	Esophagitis, GERD and related diseases	digestive	rs12509595	-0.005	0.010	0.995	5.865E-01	logistic	385890	29102	356788	0.991	0.290
358.1	Myasthenia gravis	neurological	rs12509595	-0.050	0.092	0.951	5.867E-01	logistic	419463	295	419168	0.994	0.289
802	Fracture of pelvis	injuries & poisonings	rs12509595	0.027	0.050	1.028	5.880E-01	logistic	397331	953	396378	0.982	0.289
362.2	Degeneration of macula and posterior pole of retina	sense organs	rs12509595	0.009	0.017	1.009	5.884E-01	logistic	406927	9170	397757	0.992	0.289
496.1	Emphysema	respiratory	rs12509595	0.015	0.029	1.016	5.900E-01	logistic	401774	2965	398809	0.990	0.289
586.11	Small kidney	genitourinary	rs12509595	-0.057	0.106	0.944	5.908E-01	logistic	381281	219	381062	0.990	0.290
425.12	Other hypertrophic cardiomyopathy	circulatory system	rs12509595	-0.051	0.095	0.950	5.919E-01	logistic	417044	274	416770	0.989	0.289
255.1	Adrenal hyperfunction	endocrine/metabolic	rs12509595	0.058	0.109	1.060	5.929E-01	logistic	415447	201	415246	0.991	0.290
444	Arterial embolism and thrombosis	circulatory system	rs12509595	0.030	0.057	1.031	5.940E-01	logistic	406215	746	405469	0.989	0.289
465.2	Acute pharyngitis	respiratory	rs12509595	0.052	0.098	1.053	5.971E-01	logistic	419940	248	419692	0.992	0.290
516.1	Hemoptysis	respiratory	rs12509595	-0.027	0.051	0.973	5.997E-01	logistic	421320	931	420389	0.994	0.290
296.1	Bipolar	mental disorders	rs12509595	0.024	0.045	1.024	5.999E-01	logistic	382786	1164	381622	0.985	0.290
415.2	Chronic pulmonary heart disease	circulatory system	rs12509595	0.020	0.038	1.020	6.014E-01	logistic	414491	1653	412838	0.997	0.289
800.2	Fracture of unspecified part of femur	injuries & poisonings	rs12509595	-0.056	0.108	0.945	6.026E-01	logistic	396592	214	396378	0.980	0.289
529	Diseases and other conditions of the tongue	digestive	rs12509595	-0.048	0.094	0.953	6.061E-01	logistic	417990	280	417710	0.992	0.290
288	Diseases of white blood cells	hematopoietic	rs12509595	0.039	0.076	1.040	6.061E-01	logistic	408723	414	408309	0.994	0.290
384	Other disorders of tympanic membrane	sense organs	rs12509595	-0.042	0.082	0.959	6.069E-01	logistic	419080	364	418716	0.993	0.290

ST31; PheWAS results for the lead cis-pQTLs of the 4 proteins prioritized (ACOX1, FGF5, FURIN, and MST1).

SNP = Single Nucleotide Polymorphism; SE = Standard error HWE_p = P-value for Hardy-Weinberg equilibrium test

Phenotype	Description	Group	SNP	Beta	SE	Odds tatio	P-value	Type	n_total	n_cases	n_controls	HWE_p	allele_freq
594	Urinary calculus	genitourinary	rs12509595	0.011	0.021	1.011	6.070E-01	logistic	418195	5819	412376	0.991	0.289
364.5	Corneal dystrophy	sense organs	rs12509595	0.040	0.078	1.041	6.088E-01	logistic	398152	389	397763	0.988	0.289
535	Gastritis and duodenitis	digestive	rs12509595	0.006	0.012	1.006	6.089E-01	logistic	378649	16435	362214	0.954	0.290
717	Polymyalgia Rheumatica	musculoskeletal	rs12509595	0.016	0.031	1.016	6.121E-01	logistic	422397	2442	419955	0.993	0.290
280.2	Iron deficiency anemia secondary to blood loss (chronic	hematopoietic	rs12509595	0.045	0.089	1.046	6.135E-01	logistic	382749	300	382449	0.978	0.290
170	Cancer of bone and connective tissue	neoplasms	rs12509595	0.041	0.082	1.042	6.160E-01	logistic	423460	359	423101	0.993	0.290
695.2	Bullous dermatoses	dermatologic	rs12509595	-0.055	0.111	0.946	6.172E-01	logistic	405408	202	405206	0.997	0.289
38	Septicemia	infectious diseases	rs12509595	0.009	0.018	1.009	6.180E-01	logistic	403876	7229	396647	0.994	0.290
707.2	Chronic ulcer of leg or foot	dermatologic	rs12509595	0.017	0.034	1.017	6.185E-01	logistic	418232	2102	416130	0.993	0.290
279.1	Immunity deficiency	endocrine/metabolic	rs12509595	0.043	0.086	1.044	6.191E-01	logistic	423199	323	422876	0.992	0.290
695.3	Rosacea	dermatologic	rs12509595	-0.049	0.099	0.952	6.194E-01	logistic	405456	250	405206	0.997	0.289
251.1	Hypoglycemia	endocrine/metabolic	rs12509595	0.020	0.040	1.020	6.203E-01	logistic	383028	1531	381497	0.987	0.289
519.8	Other diseases of respiratory system, NEC	respiratory	rs12509595	-0.008	0.017	0.992	6.218E-01	logistic	411807	8677	403130	0.995	0.290
430.1	Subarachnoid hemorrhage	circulatory system	rs12509595	-0.028	0.058	0.972	6.240E-01	logistic	403416	731	402685	0.997	0.289
480.5	Bronchopneumonia and lung abscess	respiratory	rs12509595	0.036	0.074	1.037	6.241E-01	logistic	397229	429	396800	0.992	0.290
276.1	Electrolyte imbalance	endocrine/metabolic	rs12509595	0.008	0.015	1.008	6.244E-01	logistic	406083	10511	395572	0.991	0.290
289.5	Diseases of spleen	hematopoietic	rs12509595	0.027	0.056	1.028	6.266E-01	logistic	409070	761	408309	0.991	0.290
568	Other disorders of peritoneum	digestive	rs12509595	-0.020	0.041	0.980	6.278E-01	logistic	385305	1466	383839	0.996	0.289
155	Cancer of liver and intrahepatic bile duct	neoplasms	rs12509595	0.027	0.057	1.028	6.286E-01	logistic	394604	742	393862	0.998	0.290
701.3	Circumscribed scleroderma	dermatologic	rs12509595	-0.051	0.105	0.951	6.296E-01	logistic	415410	224	415186	0.996	0.290
70.4	Chronic hepatitis	infectious diseases	rs12509595	0.044	0.091	1.045	6.298E-01	logistic	415491	287	415204	0.987	0.290
580.32	Nephritis and nephropathy with pathological lesion	genitourinary	rs12509595	-0.045	0.095	0.956	6.318E-01	logistic	381336	274	381062	0.990	0.290
440.2	Atherosclerosis of the extremities	circulatory system	rs12509595	0.025	0.052	1.025	6.328E-01	logistic	406343	874	405469	0.990	0.289
603.1	Hydrocele	genitourinary	rs12509595	0.035	0.073	1.035	6.360E-01	logistic	162225	447	161778	0.819	0.289
540	Appendiceal conditions	digestive	rs12509595	-0.034	0.073	0.967	6.400E-01	logistic	422123	467	421656	0.987	0.290
451	Phlebitis and thrombophlebitis	circulatory system	rs12509595	0.027	0.058	1.028	6.403E-01	logistic	381675	709	380966	0.946	0.290
516	Abnormal sputum	respiratory	rs12509595	-0.023	0.050	0.977	6.409E-01	logistic	421370	981	420389	0.993	0.290
427.5	Arrhythmia (cardiac) NOS	circulatory system	rs12509595	-0.033	0.070	0.968	6.409E-01	logistic	372348	500	371848	0.993	0.289
710	Osteomyelitis, periostitis, and other infections involving bone	musculoskeletal	rs12509595	-0.044	0.094	0.957	6.418E-01	logistic	412326	278	412048	0.991	0.290
365.1	Open-angle glaucoma	sense organs	rs12509595	0.018	0.039	1.018	6.422E-01	logistic	399325	1562	397763	0.990	0.289
366	Cataract	sense organs	rs12509595	0.004	0.009	1.004	6.426E-01	logistic	406937	35259	371678	0.969	0.290
781	Symptoms involving nervous and musculoskeletal system	symptoms	rs12509595	0.008	0.018	1.008	6.439E-01	logistic	419375	7893	411482	0.995	0.289
720	Spinal stenosis	musculoskeletal	rs12509595	-0.011	0.024	0.989	6.448E-01	logistic	412805	4135	408670	0.988	0.290
519	Other diseases of respiratory system, not elsewhere classified	respiratory	rs12509595	-0.008	0.017	0.992	6.490E-01	logistic	411962	8832	403130	0.996	0.290
262	Mineral deficiency NEC	endocrine/metabolic	rs12509595	0.038	0.083	1.038	6.509E-01	logistic	412440	343	412097	0.992	0.290
198.6	Secondary malignancy of bone	neoplasms	rs12509595	-0.010	0.023	0.990	6.523E-01	logistic	390762	4756	386006	0.988	0.290
531.4	Peptic ulcer, site unspecified	digestive	rs12509595	0.046	0.103	1.047	6.550E-01	logistic	411565	226	411339	0.983	0.290
401.2	Hypertensive heart and/or renal disease	circulatory system	rs12509595	0.021	0.046	1.021	6.563E-01	logistic	291807	1144	290663	0.999	0.284
508	Pulmonary collapse; interstitial and compensatory emphysema	respiratory	rs12509595	-0.011	0.025	0.989	6.570E-01	logistic	394890	3833	391057	0.996	0.290
745	Pain in joint	musculoskeletal	rs12509595	-0.012	0.026	0.988	6.572E-01	logistic	414363	3501	410862	0.993	0.290
260	Protein-calorie malnutrition	endocrine/metabolic	rs12509595	0.035	0.080	1.036	6.574E-01	logistic	412473	376	412097	0.992	0.290
289.4	Lymphadenitis	hematopoietic	rs12509595	-0.017	0.038	0.984	6.615E-01	logistic	410013	1704	408309	0.992	0.290
763	Thoracic or lumbosacral neuritis or radiculitis, unspecified	symptoms	rs12509595	0.040	0.092	1.041	6.630E-01	logistic	418269	283	417986	0.995	0.290
701	Other hypertrophic and atrophic conditions of skin	dermatologic	rs12509595	-0.020	0.046	0.980	6.640E-01	logistic	416347	1161	415186	0.996	0.290
180.1	Cervical cancer	neoplasms	rs12509595	-0.015	0.035	0.985	6.649E-01	logistic	197916	2012	195904	0.989	0.290
870	Open wounds of head; neck; and trunk	injuries & poisonings	rs12509595	-0.024	0.054	0.977	6.654E-01	logistic	420976	826	420150	0.992	0.290
117.4	Aspergillosis	infectious diseases	rs12509595	0.046	0.106	1.047	6.658E-01	logistic	418590	210	418380	0.993	0.290
442.8	Aneurysm of other specified artery	circulatory system	rs12509595	-0.042	0.097	0.959	6.661E-01	logistic	405731	262	405469	0.991	0.289
287.3	Thrombocytopenia	hematopoietic	rs12509595	-0.014	0.033	0.986	6.662E-01	logistic	419777	2224	417553	0.994	0.289
626.1	Irregular menstrual cycle/bleeding	genitourinary	rs12509595	0.011	0.024	1.011	6.673E-01	logistic	196900	4153	192747	0.984	0.290
252.2	Hypoparathyroidism	endocrine/metabolic	rs12509595	-0.045	0.105	0.956	6.701E-01	logistic	415471	225	415246	0.992	0.290
585.4	Chronic kidney disease, Stage I or II	genitourinary	rs12509595	0.023	0.055	1.023	6.723E-01	logistic	381861	799	381062	0.990	0.290
414	Other forms of chronic heart disease	circulatory system	rs12509595	0.010	0.024	1.010	6.724E-01	logistic	374670	4120	370550	0.979	0.288
738	Other acquired musculoskeletal deformity	musculoskeletal	rs12509595	-0.018	0.043	0.982	6.726E-01	logistic	403982	1328	402654	0.983	0.289
803.1	Fracture of humerus	injuries & poisonings	rs12509595	0.020	0.047	1.020	6.726E-01	logistic	397468	1090	396378	0.982	0.289
275.5	Disorders of calcium/phosphorus metabolism	endocrine/metabolic	rs12509595	-0.014	0.032	0.986	6.741E-01	logistic	418502	2317	416185	0.995	0.290
695.42	Systemic lupus erythematosus	dermatologic	rs12509595	-0.033	0.078	0.968	6.753E-01	logistic	403885	403	403482	0.996	0.289
197	Chemotherapy	neoplasms	rs12509595	-0.006	0.015	0.994	6.765E-01	logistic	397532	11526	386006	0.984	0.290
761	Cervicalgia	symptoms	rs12509595	0.024	0.057	1.024	6.788E-01	logistic	422454	744	421710	0.991	0.290
722	Intervertebral disc disorders	musculoskeletal	rs12509595	0.025	0.060	1.025	6.796E-01	logistic	409333	663	408670	0.991	0.290
625	Pain and other symptoms associated with female genital organs	genitourinary	rs12509595	-0.038	0.092	0.963	6.799E-01	logistic	218575	291	218284	0.988	0.290
480	Pneumonia	respiratory	rs12509595	0.005	0.013	1.005	6.806E-01	logistic	412462	15662	396800	0.993	0.290
217	Vascular hamartomas and non-neoplastic nevi	neoplasms	rs12509595	-0.037	0.090	0.964	6.827E-01	logistic	414259	301	413958	0.986	0.289
217.1	Nevus, non-neoplastic	neoplasms	rs12509595	-0.039	0.095	0.962	6.832E-01	logistic	414233	275	413958	0.986	0.289
537	Other disorders of stomach and duodenum	digestive	rs12509595	-0.015	0.037	0.985	6.837E-01	logistic	364031	1817	362214	0.926	0.289
473	Diseases of the larynx and vocal cords	respiratory	rs12509595	0.017	0.043	1.017	6.869E-01	logistic	397819	1302	396517	0.989	0.289
331	Other cerebral degenerations	neurological	rs12509595	-0.015	0.038	0.985	6.910E-01	logistic	400699	1652	399047	0.989	0.290
454	Varicose veins	circulatory system	rs12509595	0.009	0.022	1.009	6.922E-01	logistic	385827	4861	380966	0.969	0.290
363	Chorioretinal inflammations, scars, and other disorders of choroid	sense organs	rs12509595	0.040	0.102	1.041	6.934E-01	logistic	397993	230	397763	0.991	0.289
368	Visual disturbances	sense organs	rs12509595	0.013	0.032	1.013	6.936E-01	logistic	417843	2298	415545	0.991	0.289
961	Poisoning by other anti-infectives	injuries & poisonings	rs12509595	0.035	0.091	1.036	6.995E-01	logistic	367461	288	367173	0.995	0.289

ST31; PheWAS results for the lead cis-pQTLs of the 4 proteins prioritized (ACOX1, FGF5, FURIN, and MST1).

SNP = Single Nucleotide Polymorphism; SE = Standard error HWE_p = P-value for Hardy-Weinberg equilibrium test

Phenotype	Description	Group	SNP	Beta	SE	Odds tatio	P-value	Type	n_total	n_cases	n_controls	HWE_p	allele_freq
332	Parkinson's disease	neurological	rs12509595	0.011	0.029	1.011	6.999E-01	logistic	401966	2919	399047	0.980	0.290
575.8	Other disorders of biliary tract	digestive	rs12509595	0.018	0.048	1.018	7.031E-01	logistic	396103	1056	395047	0.965	0.290
622.2	Mucous polyp of cervix	genitourinary	rs12509595	-0.039	0.102	0.962	7.041E-01	logistic	213810	238	213572	0.985	0.290
261	Vitamin deficiency	endocrine/metabolic	rs12509595	0.009	0.025	1.009	7.051E-01	logistic	416032	3935	412097	0.991	0.290
751	Genitourinary congenital anomalies	congenital anomalies	rs12509595	0.020	0.052	1.020	7.054E-01	logistic	421129	876	420253	0.991	0.290
740.9	Osteoarthrosis NOS	musculoskeletal	rs12509595	-0.005	0.013	0.995	7.071E-01	logistic	361594	15736	345858	0.990	0.290
530.2	Esophageal bleeding (varices/hemorrhage	digestive	rs12509595	0.028	0.076	1.029	7.085E-01	logistic	357206	418	356788	0.990	0.290
743	Osteoporosis, osteopenia and pathological fracture	musculoskeletal	rs12509595	0.017	0.046	1.017	7.086E-01	logistic	422387	1153	421234	0.992	0.289
560.1	Paralytic ileus	digestive	rs12509595	-0.029	0.079	0.971	7.116E-01	logistic	334880	395	334485	0.996	0.289
395.4	Nonrheumatic pulmonary valve disorders	circulatory system	rs12509595	0.032	0.086	1.032	7.150E-01	logistic	401735	321	401414	0.994	0.289
597	Other disorders of urethra and urinary tract	genitourinary	rs12509595	-0.013	0.035	0.987	7.153E-01	logistic	403600	2046	401554	0.983	0.289
569	Other disorders of intestine	digestive	rs12509595	-0.012	0.034	0.988	7.162E-01	logistic	385990	2151	383839	0.998	0.289
227.3	Benign neoplasm of pituitary gland and craniopharyngeal duct (pouch	neoplasms	rs12509595	0.029	0.082	1.030	7.175E-01	logistic	421248	361	420887	0.991	0.290
475	Chronic sinusitis	respiratory	rs12509595	0.018	0.050	1.018	7.179E-01	logistic	397468	951	396517	0.990	0.289
276	Disorders of fluid, electrolyte, and acid-base balance	endocrine/metabolic	rs12509595	-0.005	0.013	0.995	7.182E-01	logistic	411277	15705	395572	0.994	0.290
614	Inflammatory diseases of female pelvic organs	genitourinary	rs12509595	-0.014	0.040	0.986	7.191E-01	logistic	218979	1541	217438	0.990	0.290
427.4	Cardiac arrest and ventricular fibrillation	circulatory system	rs12509595	-0.019	0.052	0.982	7.210E-01	logistic	372755	907	371848	0.992	0.289
481	Influenza	respiratory	rs12509595	-0.026	0.073	0.974	7.214E-01	logistic	397264	464	396800	0.993	0.290
389	Hearing loss	sense organs	rs12509595	0.007	0.019	1.007	7.217E-01	logistic	415421	7124	408297	0.985	0.290
573.9	Abnormal serum enzyme levels	digestive	rs12509595	0.037	0.105	1.038	7.259E-01	logistic	400489	215	400274	0.989	0.290
709.3	Systemic sclerosis	dermatologic	rs12509595	-0.037	0.106	0.964	7.290E-01	logistic	404118	219	403899	0.996	0.289
594.3	Calculus of ureter	genitourinary	rs12509595	-0.013	0.039	0.987	7.291E-01	logistic	414010	1634	412376	0.992	0.289
506	Empyema and pneumothorax	respiratory	rs12509595	-0.016	0.046	0.984	7.295E-01	logistic	392231	1174	391057	0.993	0.290
565	Anal and rectal conditions	digestive	rs12509595	-0.006	0.018	0.994	7.338E-01	logistic	391353	7514	383839	0.997	0.289
277	Other disorders of metabolism	endocrine/metabolic	rs12509595	0.016	0.047	1.016	7.344E-01	logistic	421029	1088	419941	0.994	0.290
384.4	Perforation of tympanic membrane	sense organs	rs12509595	-0.034	0.100	0.967	7.345E-01	logistic	418963	247	418716	0.994	0.290
170.2	Cancer of connective tissue	neoplasms	rs12509595	-0.034	0.100	0.967	7.347E-01	logistic	423349	248	423101	0.993	0.290
597.1	Urethral stricture (not specified as infectious)	genitourinary	rs12509595	-0.012	0.037	0.988	7.354E-01	logistic	403370	1816	401554	0.984	0.290
569.1	Toxic gastroenteritis and colitis	digestive	rs12509595	0.029	0.087	1.029	7.383E-01	logistic	384157	318	383839	0.998	0.289
858	Complication of internal orthopedic device	injuries & poisonings	rs12509595	-0.015	0.044	0.985	7.386E-01	logistic	417889	1237	416652	0.991	0.290
608	Other disorders of male genital organs	genitourinary	rs12509595	-0.019	0.057	0.981	7.397E-01	logistic	162533	755	161778	0.812	0.289
318	Tobacco use disorder	mental disorders	rs12509595	0.007	0.022	1.007	7.402E-01	logistic	408298	5005	403293	0.993	0.289
158	Neoplasm of unspecified nature of digestive system	neoplasms	rs12509595	-0.027	0.081	0.974	7.413E-01	logistic	394238	376	393862	0.998	0.290
80	Postoperative infection	infectious diseases	rs12509595	-0.012	0.036	0.989	7.484E-01	logistic	414741	1897	412844	0.996	0.290
189.11	Malignant neoplasm of kidney, except pelvis	neoplasms	rs12509595	0.015	0.046	1.015	7.486E-01	logistic	418324	1137	417187	0.990	0.289
441	Vascular insufficiency of intestine	circulatory system	rs12509595	0.019	0.059	1.019	7.487E-01	logistic	406151	682	405469	0.991	0.289
601	Inflammatory diseases of prostate	genitourinary	rs12509595	-0.017	0.052	0.984	7.492E-01	logistic	157382	921	156461	0.549	0.289
696.42	Psoriatic arthropathy	dermatologic	rs12509595	0.017	0.052	1.017	7.500E-01	logistic	400183	893	399290	0.996	0.289
724	Other and unspecified disorders of back	musculoskeletal	rs12509595	0.021	0.067	1.021	7.510E-01	logistic	409212	542	408670	0.990	0.290
286	Coagulation defects	hematopoietic	rs12509595	-0.016	0.051	0.984	7.517E-01	logistic	418481	928	417553	0.993	0.289
614.3	Pelvic inflammatory disease (PID)	genitourinary	rs12509595	-0.032	0.102	0.968	7.527E-01	logistic	217675	237	217438	0.991	0.290
241.2	Nontoxic multinodular goiter	endocrine/metabolic	rs12509595	-0.032	0.102	0.969	7.534E-01	logistic	392467	237	392230	0.978	0.289
624.9	stress incontinence, female	genitourinary	rs12509595	-0.011	0.036	0.989	7.539E-01	logistic	220155	1871	218284	0.993	0.290
470	Septal Deviations/Turbinate Hypertrophy	respiratory	rs12509595	-0.012	0.039	0.988	7.544E-01	logistic	398163	1646	396517	0.988	0.289
208	Benign neoplasm of colon	neoplasms	rs12509595	0.004	0.011	1.004	7.544E-01	logistic	395855	20019	375836	0.999	0.290
292	Neurological disorders	mental disorders	rs12509595	0.007	0.021	1.007	7.570E-01	logistic	410372	5399	404973	0.980	0.289
288.1	Decreased white blood cell count	hematopoietic	rs12509595	-0.009	0.028	0.992	7.589E-01	logistic	411502	3193	408309	0.990	0.290
288.11	Neutropenia	hematopoietic	rs12509595	-0.009	0.028	0.992	7.589E-01	logistic	411502	3193	408309	0.990	0.290
277.4	Disorders of bilirubin excretion	endocrine/metabolic	rs12509595	-0.022	0.073	0.978	7.619E-01	logistic	420403	462	419941	0.993	0.290
752.11	Spina bifida	congenital anomalies	rs12509595	0.033	0.108	1.033	7.633E-01	logistic	422743	204	422539	0.991	0.290
81	Infection/inflammation of internal prosthetic device; implant; and graf	infectious diseases	rs12509595	-0.011	0.036	0.989	7.636E-01	logistic	414783	1939	412844	0.997	0.290
622.1	Polyp of corpus uteri	genitourinary	rs12509595	-0.012	0.039	0.988	7.647E-01	logistic	215181	1609	213572	0.982	0.290
278	Overweight, obesity and other hyperalimentation	endocrine/metabolic	rs12509595	0.004	0.013	1.004	7.650E-01	logistic	405726	15173	390553	0.988	0.290
593	Hematuria	genitourinary	rs12509595	0.007	0.022	1.007	7.671E-01	logistic	381996	5069	376927	0.988	0.290
594.8	Renal colic	genitourinary	rs12509595	0.020	0.066	1.020	7.681E-01	logistic	412927	551	412376	0.993	0.289
261.2	Vitamin B-complex deficiencies	endocrine/metabolic	rs12509595	0.011	0.038	1.011	7.701E-01	logistic	413809	1712	412097	0.992	0.290
117	Mycoses	infectious diseases	rs12509595	0.027	0.094	1.028	7.707E-01	logistic	418652	272	418380	0.993	0.290
364.4	Corneal degenerations	sense organs	rs12509595	-0.013	0.046	0.987	7.736E-01	logistic	398917	1154	397763	0.986	0.289
574.2	Calculus of bile duct	digestive	rs12509595	0.029	0.100	1.029	7.737E-01	logistic	395287	240	395047	0.968	0.290
110	Dermatophytosis / Dermatomycosis	infectious diseases	rs12509595	0.029	0.101	1.029	7.749E-01	logistic	418617	237	418380	0.993	0.290
317.1	Alcoholism	mental disorders	rs12509595	0.007	0.025	1.007	7.763E-01	logistic	407082	3789	403293	0.994	0.289
578	Gastrointestinal hemorrhage	digestive	rs12509595	0.005	0.016	1.005	7.772E-01	logistic	392729	9426	383303	0.977	0.289
155.1	Malignant neoplasm of liver, primary	neoplasms	rs12509595	-0.025	0.087	0.976	7.773E-01	logistic	394187	325	393862	0.998	0.290
742	Derangement of joint, non-traumatic	musculoskeletal	rs12509595	0.021	0.074	1.021	7.796E-01	logistic	415444	434	415010	0.990	0.289
278.1	Obesity	endocrine/metabolic	rs12509595	0.004	0.013	1.004	7.801E-01	logistic	405667	15114	390553	0.988	0.290
726	Peripheral enthesopathies and allied syndromes	musculoskeletal	rs12509595	0.004	0.015	1.004	7.810E-01	logistic	399760	10586	389174	0.992	0.289
977	Personal history of allergy to medicinal agents	injuries & poisonings	rs12509595	-0.005	0.017	0.995	7.848E-01	logistic	375378	8205	367173	0.998	0.289
575	Other biliary tract disease	digestive	rs12509595	0.007	0.026	1.007	7.856E-01	logistic	398734	3687	395047	0.963	0.290
724.1	Disorders of sacrum	musculoskeletal	rs12509595	0.028	0.102	1.028	7.871E-01	logistic	408900	230	408670	0.990	0.290
530	Diseases of esophagus	digestive	rs12509595	-0.002	0.009	0.998	7.895E-01	logistic	388733	31945	356788	0.992	0.290
496.3	Bronchiectasis	respiratory	rs12509595	-0.007	0.026	0.993	7.899E-01	logistic	402578	3769	398809	0.990	0.289

ST31; PheWAS results for the lead cis-pQTLs of the 4 proteins prioritized (ACOX1, FGF5, FURIN, and MST1).

SNP = Single Nucleotide Polymorphism; SE = Standard error HWE_p = P-value for Hardy-Weinberg equilibrium test

Phenotype	Description	Group	SNP	Beta	SE	Odds tatio	P-value	Type	n_total	n_cases	n_controls	HWE_p	allele_freq
747.1	Cardiac congenital anomalies	congenital anomalies	rs12509595	0.014	0.051	1.014	7.899E-01	logistic	422536	941	421595	0.991	0.290
790.6	Other abnormal blood chemistry	symptoms	rs12509595	-0.027	0.102	0.973	7.914E-01	logistic	422077	237	421840	0.993	0.290
530.13	Barrett's esophagus	digestive	rs12509595	-0.006	0.024	0.994	7.925E-01	logistic	361195	4407	356788	0.988	0.290
714.1	Rheumatoid arthritis	musculoskeletal	rs12509595	0.007	0.025	1.007	7.957E-01	logistic	412357	3850	408507	0.996	0.289
695.4	Lupus (localized and systemic)	dermatologic	rs12509595	-0.018	0.070	0.982	7.974E-01	logistic	403975	493	403482	0.996	0.289
724.2	Disorders of coccyx	musculoskeletal	rs12509595	0.027	0.106	1.027	7.989E-01	logistic	408885	215	408670	0.991	0.290
626	Disorders of menstruation and other abnormal bleeding from female genital trac	genitourinary	rs12509595	-0.005	0.020	0.995	7.999E-01	logistic	198823	6076	192747	0.983	0.290
38.1	Gram negative septicemia	infectious diseases	rs12509595	0.022	0.087	1.022	8.022E-01	logistic	396961	314	396647	0.995	0.290
386	Vertiginous syndromes and other disorders of vestibular systen	sense organs	rs12509595	0.009	0.038	1.009	8.052E-01	logistic	411346	1681	409665	0.995	0.289
556	Ulceration of the lower GI tract	digestive	rs12509595	0.019	0.078	1.019	8.062E-01	logistic	334879	394	334485	0.998	0.289
567	Peritonitis and retroperitoneal infections	digestive	rs12509595	0.014	0.057	1.014	8.108E-01	logistic	384568	729	383839	0.997	0.289
619.3	Noninflammatory disorders of cervix	genitourinary	rs12509595	-0.020	0.085	0.980	8.121E-01	logistic	217308	342	216966	0.973	0.289
571.5	Other chronic nonalcoholic liver disease	digestive	rs12509595	0.006	0.024	1.006	8.121E-01	logistic	404607	4333	400274	0.990	0.290
575.1	Cholangitis	digestive	rs12509595	0.012	0.052	1.012	8.145E-01	logistic	395932	885	395047	0.966	0.290
510	Other diseases of lung	respiratory	rs12509595	0.009	0.041	1.009	8.206E-01	logistic	421711	1427	420284	0.993	0.290
368.1	Amblyopia	sense organs	rs12509595	-0.016	0.070	0.984	8.227E-01	logistic	416041	496	415545	0.991	0.289
282	Hereditary hemolytic anemias	hematopoietic	rs12509595	-0.024	0.107	0.976	8.248E-01	logistic	382667	218	382449	0.980	0.290
451.2	Phlebitis and thrombophlebitis of lower extremities	circulatory system	rs12509595	0.016	0.070	1.016	8.248E-01	logistic	381453	487	380966	0.944	0.290
465	Acute upper respiratory infections of multiple or unspecified sites	respiratory	rs12509595	-0.013	0.059	0.987	8.251E-01	logistic	420386	694	419692	0.991	0.290
574.11	Cholelithiasis with acute cholecystitis	digestive	rs12509595	-0.011	0.049	0.989	8.258E-01	logistic	396059	1012	395047	0.968	0.290
568.1	Peritoneal adhesions (postoperative) (postinfection)	digestive	rs12509595	-0.010	0.043	0.991	8.260E-01	logistic	385136	1297	383839	0.996	0.289
79.9	Viremia, NOS	infectious diseases	rs12509595	0.014	0.064	1.014	8.262E-01	logistic	415798	594	415204	0.989	0.290
180	Cervical cancer and dysplasia	neoplasms	rs12509595	-0.007	0.031	0.993	8.268E-01	logistic	198422	2518	195904	0.990	0.290
202.21	Nodular lymphoma	neoplasms	rs12509595	-0.016	0.072	0.984	8.269E-01	logistic	416077	464	415613	0.993	0.290
743.2	Pathologic fracture	musculoskeletal	rs12509595	0.011	0.051	1.011	8.292E-01	logistic	422164	930	421234	0.993	0.289
204.21	Myeloid leukemia, acute	neoplasms	rs12509595	-0.014	0.065	0.986	8.319E-01	logistic	416195	582	415613	0.992	0.290
193	Thyroid cancer	neoplasms	rs12509595	-0.016	0.077	0.984	8.319E-01	logistic	421300	413	420887	0.990	0.290
368.91	Psychophysical visual disturbances	sense organs	rs12509595	0.020	0.095	1.020	8.322E-01	logistic	415808	263	415545	0.990	0.289
871	Open wounds of extremities	injuries & poisonings	rs12509595	0.017	0.081	1.017	8.336E-01	logistic	420520	370	420150	0.992	0.290
619.2	Disorders of uterus, NEC	genitourinary	rs12509595	-0.016	0.077	0.984	8.340E-01	logistic	217379	413	216966	0.971	0.289
377	Disorders of optic nerve and visual pathways	sense organs	rs12509595	0.017	0.081	1.017	8.377E-01	logistic	412293	365	411928	0.988	0.289
476	Allergic rhinitis	respiratory	rs12509595	0.010	0.048	1.010	8.384E-01	logistic	397580	1063	396517	0.990	0.289
316	Substance addiction and disorders	mental disorders	rs12509595	-0.017	0.083	0.983	8.395E-01	logistic	403645	352	403293	0.995	0.289
635	Hemorrhage during pregnancy; childbirth and postpartum	pregnancy complications	rs12509595	0.016	0.077	1.016	8.408E-01	logistic	223005	413	222592	0.982	0.290
185	Cancer of prostate	neoplasms	rs12509595	0.003	0.016	1.003	8.425E-01	logistic	167540	11079	156461	0.769	0.289
381.11	Suppurative and unspecified otitis media	sense organs	rs12509595	-0.019	0.098	0.981	8.427E-01	logistic	418969	253	418716	0.993	0.290
509.8	Dependence on respirator [Ventilator] or supplemental oxygen	respiratory	rs12509595	-0.012	0.059	0.988	8.427E-01	logistic	391755	698	391057	0.994	0.290
270.33	Amyloidosis	endocrine/metabolic	rs12509595	0.017	0.085	1.017	8.429E-01	logistic	422032	330	421702	0.994	0.289
530.12	Ulcer of esophagus	digestive	rs12509595	0.006	0.030	1.006	8.430E-01	logistic	359422	2634	356788	0.985	0.290
323	Encephalitis	neurological	rs12509595	0.016	0.083	1.016	8.437E-01	logistic	422420	349	422071	0.992	0.289
250.23	Type 2 diabetes with ophthalmic manifestations	endocrine/metabolic	rs12509595	-0.007	0.035	0.993	8.444E-01	logistic	388590	1953	386637	0.990	0.289
560.4	Other intestinal obstruction	digestive	rs12509595	0.007	0.034	1.007	8.450E-01	logistic	336617	2132	334485	0.996	0.289
803.2	Fracture of radius and ulna	injuries & poisonings	rs12509595	0.006	0.033	1.006	8.455E-01	logistic	398618	2240	396378	0.980	0.289
375.2	Epiphora	sense organs	rs12509595	0.016	0.086	1.017	8.474E-01	logistic	412257	329	411928	0.986	0.289
426.23	Second degree AV block	circulatory system	rs12509595	0.012	0.061	1.012	8.486E-01	logistic	372488	640	371848	0.993	0.289
599.5	Frequency of urination and polyuria	genitourinary	rs12509595	-0.010	0.053	0.990	8.489E-01	logistic	390965	859	390106	0.968	0.289
361	Retinal detachments and defects	sense organs	rs12509595	0.013	0.069	1.013	8.518E-01	logistic	398276	513	397763	0.990	0.289
783	Fever of unknown origin	symptoms	rs12509595	-0.006	0.032	0.994	8.524E-01	logistic	418176	2397	415779	0.993	0.290
340	Migraine	neurological	rs12509595	0.006	0.031	1.006	8.526E-01	logistic	407732	2545	405187	0.995	0.289
635.2	Antepartum hemorrhage, abruptio placentae, and placenta previa	pregnancy complications	rs12509595	0.020	0.109	1.020	8.533E-01	logistic	222797	205	222592	0.982	0.290
286.12	Congenital deficiency of other clotting factors (including factor VII	hematopoietic	rs12509595	-0.019	0.102	0.981	8.538E-01	logistic	417788	235	417553	0.995	0.289
394.7	Disease of tricuspid valve	circulatory system	rs12509595	0.011	0.061	1.011	8.565E-01	logistic	402072	658	401414	0.993	0.289
512.8	Cough	respiratory	rs12509595	-0.007	0.039	0.993	8.578E-01	logistic	406184	1585	404599	0.990	0.289
615	Endometriosis	genitourinary	rs12509595	-0.008	0.045	0.992	8.586E-01	logistic	218669	1231	217438	0.992	0.290
958	Certain early complications of trauma or procedure	injuries & poisonings	rs12509595	-0.016	0.093	0.984	8.636E-01	logistic	423583	281	423302	0.992	0.290
204.2	Myeloid leukemia	neoplasms	rs12509595	-0.010	0.057	0.990	8.637E-01	logistic	416360	747	415613	0.992	0.290
350.3	Lack of coordination	neurological	rs12509595	0.016	0.091	1.016	8.641E-01	logistic	420499	289	420210	0.993	0.290
695.7	Prurigo and Lichen	dermatologic	rs12509595	0.015	0.089	1.015	8.652E-01	logistic	405510	304	405206	0.996	0.289
941	Adverse reaction to serum or vaccine	injuries & poisonings	rs12509595	0.017	0.103	1.018	8.659E-01	logistic	415212	225	414987	0.993	0.289
367.1	Myopia	sense organs	rs12509595	0.007	0.043	1.007	8.666E-01	logistic	412331	1325	411006	0.992	0.290
415	Pulmonary heart disease	circulatory system	rs12509595	0.006	0.038	1.006	8.666E-01	logistic	414538	1700	412838	0.997	0.289
614.1	Pelvic peritoneal adhesions, female (postoperative) (postinfection	genitourinary	rs12509595	0.013	0.078	1.013	8.674E-01	logistic	217833	395	217438	0.990	0.290
751.21	Cystic kidney disease	congenital anomalies	rs12509595	-0.013	0.080	0.987	8.680E-01	logistic	420636	383	420253	0.991	0.290
480.1	Bacterial pneumonia	respiratory	rs12509595	0.003	0.016	1.003	8.684E-01	logistic	407095	10295	396800	0.990	0.290
300.13	Phobia	mental disorders	rs12509595	-0.011	0.064	0.990	8.690E-01	logistic	382219	597	381622	0.990	0.290
250.1	Type 1 diabetes	endocrine/metabolic	rs12509595	-0.005	0.032	0.995	8.706E-01	logistic	389041	2404	386637	0.988	0.289
578.9	Hemorrhage of gastrointestinal tract	digestive	rs12509595	0.005	0.029	1.005	8.742E-01	logistic	386245	2942	383303	0.971	0.289
317	Alcohol-related disorders	mental disorders	rs12509595	-0.003	0.022	0.997	8.743E-01	logistic	408346	5053	403293	0.994	0.289
149	Cancer of larynx, pharynx, nasal cavities	neoplasms	rs12509595	-0.008	0.054	0.992	8.744E-01	logistic	421191	849	420342	0.990	0.290
472	Chronic pharyngitis and nasopharyngitis	respiratory	rs12509595	0.015	0.096	1.015	8.763E-01	logistic	396779	262	396517	0.989	0.289
967	Adverse effects of sedatives or other central nervous system depressants and anesthetic	injuries & poisonings	rs12509595	0.016	0.103	1.016	8.767E-01	logistic	367398	225	367173	0.995	0.289

ST31; PheWAS results for the lead cis-pQTLs of the 4 proteins prioritized (ACOX1, FGF5, FURIN, and MST1).

SNP = Single Nucleotide Polymorphism; SE = Standard error HWE_p = P-value for Hardy-Weinberg equilibrium test

Phenotype	Description	Group	SNP	Beta	SE	Odds tatio	P-value	Type	n_total	n_cases	n_controls	HWE_p	allele_freq
586.4	Stricture/obstruction of ureter	genitourinary	rs12509595	0.008	0.054	1.008	8.770E-01	logistic	381896	834	381062	0.992	0.290
333.1	Essential tremor	neurological	rs12509595	0.010	0.067	1.010	8.774E-01	logistic	399579	532	399047	0.990	0.290
8.52	Intestinal infection due to C. difficile	infectious diseases	rs12509595	-0.009	0.060	0.991	8.790E-01	logistic	417984	668	417316	0.996	0.290
715	Other inflammatory spondylopathies	musculoskeletal	rs12509595	0.007	0.043	1.007	8.795E-01	logistic	409805	1298	408507	0.995	0.289
345	Epilepsy, recurrent seizures, convulsions	neurological	rs12509595	-0.004	0.024	0.996	8.796E-01	logistic	403151	4104	399047	0.986	0.290
287.31	Primary thrombocytopenia	hematopoietic	rs12509595	0.010	0.068	1.010	8.808E-01	logistic	418072	519	417553	0.994	0.289
275.1	Disorders of iron metabolism	hematopoietic	rs12509595	0.007	0.047	1.007	8.832E-01	logistic	417274	1089	416185	0.994	0.290
333	Extrapyramidal disease and abnormal movement disorders	neurological	rs12509595	-0.007	0.045	0.993	8.835E-01	logistic	400270	1223	399047	0.990	0.290
578.1	Hematemesis	digestive	rs12509595	-0.006	0.043	0.994	8.849E-01	logistic	384647	1344	383303	0.975	0.289
619	Noninflammatory female genital disorders	genitourinary	rs12509595	0.005	0.033	1.005	8.855E-01	logistic	219175	2209	216966	0.982	0.289
480.11	Pneumococcal pneumonia	respiratory	rs12509595	0.002	0.016	1.002	8.861E-01	logistic	406463	9663	396800	0.991	0.290
738.4	Acquired spondylolisthesis	musculoskeletal	rs12509595	-0.006	0.045	0.994	8.864E-01	logistic	403846	1192	402654	0.984	0.289
782.3	Edema	symptoms	rs12509595	-0.004	0.032	0.996	8.880E-01	logistic	419509	2451	417058	0.990	0.290
564.9	Personal history of diseases of digestive system	digestive	rs12509595	0.002	0.014	1.002	8.886E-01	logistic	348229	13744	334485	0.993	0.289
191	Manlignant and unknown neoplasms of brain and nervous system	neoplasms	rs12509595	-0.007	0.050	0.993	8.903E-01	logistic	421363	966	420397	0.989	0.290
531.3	Duodenal ulcer	digestive	rs12509595	0.006	0.046	1.006	8.906E-01	logistic	412469	1130	411339	0.985	0.290
381.1	Otitis media	sense organs	rs12509595	-0.008	0.062	0.992	8.908E-01	logistic	419355	639	418716	0.993	0.290
426.21	First degree AV block	circulatory system	rs12509595	0.005	0.037	1.005	8.923E-01	logistic	373661	1813	371848	0.995	0.289
199	Neoplasm of uncertain behavior	neoplasms	rs12509595	-0.009	0.063	0.992	8.923E-01	logistic	386620	614	386006	0.987	0.290
250.42	Other abnormal glucose	endocrine/metabolic	rs12509595	-0.012	0.090	0.988	8.927E-01	logistic	386940	303	386637	0.989	0.289
289	Other diseases of blood and blood-forming organs	hematopoietic	rs12509595	0.007	0.055	1.007	8.936E-01	logistic	409120	811	408309	0.992	0.290
284	Aplastic anemia	hematopoietic	rs12509595	0.008	0.058	1.008	8.938E-01	logistic	383179	730	382449	0.980	0.290
564.1	Irritable Bowel Syndrome	digestive	rs12509595	0.003	0.023	1.003	8.949E-01	logistic	339145	4660	334485	0.998	0.289
241.1	Nontoxic uninodular goiter	endocrine/metabolic	rs12509595	-0.014	0.104	0.986	8.952E-01	logistic	392455	225	392230	0.977	0.289
261.4	Vitamin D deficiency	endocrine/metabolic	rs12509595	-0.004	0.033	0.996	8.961E-01	logistic	414357	2260	412097	0.992	0.290
736	Other acquired deformities of limbs	musculoskeletal	rs12509595	-0.007	0.051	0.993	8.962E-01	logistic	403605	951	402654	0.984	0.289
574.1	Cholelithiasis	digestive	rs12509595	0.002	0.016	1.002	8.972E-01	logistic	404312	9265	395047	0.978	0.290
737	Curvature of spine	musculoskeletal	rs12509595	0.006	0.052	1.006	9.057E-01	logistic	403550	896	402654	0.981	0.289
604.3	Peyronie's disease	genitourinary	rs12509595	0.012	0.104	1.012	9.067E-01	logistic	162002	224	161778	0.835	0.289
204	Leukemia	neoplasms	rs12509595	-0.003	0.029	0.997	9.095E-01	logistic	418621	3008	415613	0.991	0.290
751.22	Other specified congenital anomalies of kidney	congenital anomalies	rs12509595	0.009	0.083	1.010	9.095E-01	logistic	420601	348	420253	0.991	0.290
369	Infection of the eye	sense organs	rs12509595	0.009	0.076	1.009	9.103E-01	logistic	411210	417	410793	0.991	0.289
189.1	Cancer of kidney and renal pelvis	neoplasms	rs12509595	0.005	0.045	1.005	9.103E-01	logistic	418392	1205	417187	0.989	0.289
427.21	Atrial fibrillation	circulatory system	rs12509595	0.003	0.024	1.003	9.106E-01	logistic	376121	4273	371848	0.985	0.289
580.1	Glomerulonephritis	genitourinary	rs12509595	-0.008	0.069	0.992	9.119E-01	logistic	381574	512	381062	0.990	0.290
558	Noninfectious gastroenteritis	digestive	rs12509595	0.003	0.023	1.003	9.139E-01	logistic	338928	4443	334485	0.997	0.289
540.1	Appendicitis	digestive	rs12509595	0.009	0.086	1.009	9.192E-01	logistic	421984	328	421656	0.988	0.290
228	Hemangioma and lymphangioma, any site	neoplasms	rs12509595	-0.007	0.068	0.993	9.205E-01	logistic	421655	529	421126	0.995	0.290
706	Diseases of sebaceous glands	dermatologic	rs12509595	-0.004	0.041	0.996	9.234E-01	logistic	413434	1435	411999	0.991	0.290
274.2	Crystal arthropathies	endocrine/metabolic	rs12509595	-0.009	0.100	0.991	9.263E-01	logistic	416033	243	415790	0.986	0.290
291.8	Alteration of consciousness	mental disorders	rs12509595	0.007	0.081	1.007	9.267E-01	logistic	405343	370	404973	0.990	0.289
433.31	Transient cerebral ischemia	circulatory system	rs12509595	0.004	0.045	1.004	9.267E-01	logistic	403901	1216	402685	0.995	0.289
196	Radiotherapy	neoplasms	rs12509595	0.004	0.039	1.004	9.270E-01	logistic	387589	1583	386006	0.989	0.290
253	Disorders of the pituitary gland and its hypothalamic control	endocrine/metabolic	rs12509595	-0.005	0.052	0.995	9.274E-01	logistic	416136	890	415246	0.991	0.290
184.2	Cancer of other female genital organs (excluding uterus and ovary)	neoplasms	rs12509595	0.009	0.100	1.009	9.276E-01	logistic	205080	243	204837	0.978	0.290
507	Pleurisy; pleural effusion	respiratory	rs12509595	0.001	0.016	1.001	9.277E-01	logistic	400937	9880	391057	0.994	0.290
565.1	Anal and rectal polyp	digestive	rs12509595	-0.003	0.030	0.997	9.291E-01	logistic	386554	2715	383839	0.997	0.289
216	Benign neoplasm of skin	neoplasms	rs12509595	0.004	0.047	1.004	9.301E-01	logistic	415074	1116	413958	0.987	0.289
706.2	Sebaceous cyst	dermatologic	rs12509595	0.004	0.042	1.004	9.304E-01	logistic	413393	1394	411999	0.991	0.290
571.6	Primary biliary cirrhosis	digestive	rs12509595	-0.008	0.088	0.992	9.317E-01	logistic	400586	312	400274	0.989	0.290
619.4	Noninflammatory disorders of vagina	genitourinary	rs12509595	0.008	0.103	1.009	9.342E-01	logistic	217194	228	216966	0.974	0.289
289.3	Personal history of diseases of blood and blood-forming organs	hematopoietic	rs12509595	-0.007	0.091	0.993	9.348E-01	logistic	408604	295	408309	0.992	0.290
191.1	Cancer of brain and nervous system	neoplasms	rs12509595	0.004	0.054	1.004	9.357E-01	logistic	421229	832	420397	0.990	0.290
530.9	Heartburn	digestive	rs12509595	-0.008	0.099	0.992	9.390E-01	logistic	357038	250	356788	0.990	0.290
747.12	Valvular heart disease/ heart chambers	congenital anomalies	rs12509595	-0.007	0.093	0.993	9.396E-01	logistic	421875	280	421595	0.992	0.290
454.11	Varicose veins of lower extremity, symptomtic	circulatory system	rs12509595	-0.005	0.064	0.995	9.401E-01	logistic	381560	594	380966	0.949	0.290
395.3	Nonrheumatic tricuspid valve disorders	circulatory system	rs12509595	-0.007	0.101	0.993	9.410E-01	logistic	401653	239	401414	0.993	0.289
41.4	E. coli	infectious diseases	rs12509595	0.002	0.027	1.002	9.416E-01	logistic	399991	3344	396647	0.994	0.290
293	Symptoms involving head and neck	mental disorders	rs12509595	0.005	0.072	1.005	9.426E-01	logistic	420175	464	419711	0.994	0.290
618.1	Prolapse of vaginal walls	genitourinary	rs12509595	-0.002	0.025	0.998	9.457E-01	logistic	217426	3971	213455	0.977	0.290
291	Other specified nonpsychotic and/or transient mental disorders	mental disorders	rs12509595	0.005	0.078	1.005	9.462E-01	logistic	405371	398	404973	0.990	0.289
735.3	Hallux valgus (Bunion)	musculoskeletal	rs12509595	-0.002	0.032	0.998	9.465E-01	logistic	405116	2462	402654	0.982	0.289
689	Disorder of skin and subcutaneous tissue NOS	dermatologic	rs12509595	-0.002	0.034	0.998	9.475E-01	logistic	413188	2098	411090	0.991	0.290
440	Atherosclerosis	circulatory system	rs12509595	-0.003	0.048	0.997	9.489E-01	logistic	406545	1076	405469	0.990	0.289
447	Other disorders of arteries and arterioles	circulatory system	rs12509595	-0.003	0.047	0.997	9.515E-01	logistic	406558	1089	405469	0.989	0.289
751.2	Congenital anomalies of urinary system	congenital anomalies	rs12509595	0.003	0.057	1.003	9.520E-01	logistic	421003	750	420253	0.991	0.290
389.1	Sensorineural hearing loss	sense organs	rs12509595	0.003	0.056	1.003	9.528E-01	logistic	409063	766	408297	0.986	0.290
368.9	Subjective visual disturbances	sense organs	rs12509595	0.004	0.069	1.004	9.548E-01	logistic	416057	512	415545	0.990	0.289
189.4	Malignant neoplasm of other urinary organs	neoplasms	rs12509595	-0.006	0.109	0.994	9.558E-01	logistic	417393	206	417187	0.989	0.289
726.3	Bursitis	musculoskeletal	rs12509595	-0.006	0.109	0.994	9.561E-01	logistic	389380	206	389174	0.994	0.289
714	Rheumatoid arthritis and other inflammatory polyarthropathies	musculoskeletal	rs12509595	-0.001	0.025	0.999	9.566E-01	logistic	412574	4067	408507	0.995	0.289

ST31; PheWAS results for the lead cis-pQTLs of the 4 proteins prioritized (ACOX1, FGF5, FURIN, and MST1).

SNP = Single Nucleotide Polymorphism; SE = Standard error HWE_p = P-value for Hardy-Weinberg equilibrium test

Phenotype	Description	Group	SNP	Beta	SE	Odds tatio	P-value	Type	n_total	n_cases	n_controls	HWE_p	allele_freq
536	Disorders of function of stomach	digestive	rs12509595	0.002	0.038	1.002	9.568E-01	logistic	363858	1644	362214	0.935	0.289
733	Other disorders of bone and cartilage	musculoskeletal	rs12509595	0.003	0.052	1.003	9.574E-01	logistic	399585	913	398672	0.970	0.289
8.5	Bacterial enteritis	infectious diseases	rs12509595	0.002	0.044	1.002	9.580E-01	logistic	418567	1251	417316	0.995	0.290
290.11	Alzheimer's disease	mental disorders	rs12509595	0.002	0.032	1.002	9.588E-01	logistic	407367	2394	404973	0.993	0.289
202.24	Large cell lymphoma	neoplasms	rs12509595	-0.002	0.047	0.998	9.593E-01	logistic	416717	1104	415613	0.992	0.290
153	Colorectal cancer	neoplasms	rs12509595	-0.001	0.019	0.999	9.648E-01	logistic	377768	7048	370720	0.993	0.290
752	Nervous system congenital anomalies	congenital anomalies	rs12509595	-0.003	0.073	0.997	9.650E-01	logistic	422992	453	422539	0.991	0.290
149.1	Cancer of oropharynx	neoplasms	rs12509595	-0.003	0.080	0.997	9.656E-01	logistic	420725	383	420342	0.991	0.290
626.14	Irregular menstrual bleeding	genitourinary	rs12509595	0.003	0.078	1.003	9.656E-01	logistic	193146	399	192747	0.993	0.290
165	Cancer within the respiratory system	neoplasms	rs12509595	-0.001	0.022	0.999	9.661E-01	logistic	422554	5325	417229	0.993	0.290
367.8	Hypermetropia	sense organs	rs12509595	0.004	0.093	1.004	9.670E-01	logistic	411285	279	411006	0.991	0.290
573.7	Abnormal results of function study of liver	digestive	rs12509595	0.001	0.025	1.001	9.726E-01	logistic	404090	3816	400274	0.984	0.290
578.2	Blood in stool	digestive	rs12509595	-0.001	0.037	0.999	9.758E-01	logistic	385103	1800	383303	0.967	0.289
420.3	Endocarditis	circulatory system	rs12509595	0.002	0.063	1.002	9.783E-01	logistic	417383	613	416770	0.991	0.289
244.1	Secondary hypothyroidism	endocrine/metabolic	rs12509595	0.001	0.045	1.001	9.790E-01	logistic	393443	1213	392230	0.982	0.289
536.8	Dyspepsia and other specified disorders of function of stomach	digestive	rs12509595	-0.001	0.039	0.999	9.802E-01	logistic	363851	1637	362214	0.936	0.289
737.3	Kyphoscoliosis and scoliosis	musculoskeletal	rs12509595	0.001	0.055	1.001	9.812E-01	logistic	403443	789	402654	0.982	0.289
560	Intestinal obstruction without mention of hernia	digestive	rs12509595	-0.001	0.026	0.999	9.813E-01	logistic	338110	3625	334485	0.993	0.289
580.3	Nephritis and nephropathy without mention of glomerulonephritis	genitourinary	rs12509595	0.002	0.091	1.002	9.815E-01	logistic	381356	294	381062	0.990	0.290
79	Viral infection	infectious diseases	rs12509595	-0.001	0.049	0.999	9.824E-01	logistic	416221	1017	415204	0.989	0.290
218.1	Uterine leiomyoma	neoplasms	rs12509595	0.001	0.030	1.001	9.835E-01	logistic	209756	2761	206995	0.970	0.290
540.11	Acute appendicitis	digestive	rs12509595	0.002	0.105	1.002	9.839E-01	logistic	421875	219	421656	0.989	0.290
479	Other upper respiratory disease	respiratory	rs12509595	0.002	0.076	1.002	9.842E-01	logistic	396937	420	396517	0.987	0.289
788	Syncope and collapse	symptoms	rs12509595	0.000	0.020	1.000	9.846E-01	logistic	412052	6241	405811	0.995	0.289
735	Acquired foot deformities	musculoskeletal	rs12509595	0.000	0.020	1.000	9.850E-01	logistic	408734	6080	402654	0.984	0.289
458.1	Orthostatic hypotension	circulatory system	rs12509595	-0.001	0.028	0.999	9.853E-01	logistic	375723	3151	372572	0.994	0.289
522	Diseases of pulp and periapical tissues	digestive	rs12509595	-0.002	0.091	0.998	9.866E-01	logistic	409832	296	409536	0.991	0.290
916	Contusion	injuries & poisonings	rs12509595	-0.001	0.042	0.999	9.873E-01	logistic	421588	1393	420195	0.992	0.290
747	Cardiac and circulatory congenital anomalies	congenital anomalies	rs12509595	-0.001	0.047	0.999	9.880E-01	logistic	422691	1096	421595	0.992	0.290
374.3	Ptosis of eyelid	sense organs	rs12509595	0.001	0.045	1.001	9.903E-01	logistic	411997	1204	410793	0.990	0.289
394.2	Mitral valve disease	circulatory system	rs12509595	-0.001	0.088	0.999	9.915E-01	logistic	401726	312	401414	0.994	0.289
381	Otitis media and Eustachian tube disorders	sense organs	rs12509595	0.001	0.053	1.001	9.917E-01	logistic	419568	852	418716	0.993	0.290
180.3	Cervical intraepithelial neoplasia [CIN] [Cervical dysplasia	neoplasms	rs12509595	-0.001	0.071	0.999	9.918E-01	logistic	196385	481	195904	0.986	0.290
331.9	Cerebral degeneration, unspecified	neurological	rs12509595	0.000	0.050	1.000	9.933E-01	logistic	400009	962	399047	0.986	0.290
70.3	Viral hepatitis C	infectious diseases	rs12509595	0.001	0.096	1.001	9.958E-01	logistic	415468	264	415204	0.988	0.290
473.3	Paralysis/spasm of vocal cords or larynx	respiratory	rs12509595	-0.001	0.104	0.999	9.961E-01	logistic	396742	225	396517	0.989	0.289
378	Strabismus and other disorders of binocular eye movements	sense organs	rs12509595	0.000	0.050	1.000	9.965E-01	logistic	412884	956	411928	0.989	0.289
198.4	Secondary malignant neoplasm of liver	neoplasms	rs12509595	0.000	0.021	1.000	9.968E-01	logistic	391482	5476	386006	0.981	0.290
455	Hemorrhoids	circulatory system	rs12509595	0.000	0.022	1.000	9.968E-01	logistic	386244	5278	380966	0.967	0.290
587	Kidney replaced by transpant	genitourinary	rs12509595	0.000	0.070	1.000	9.981E-01	logistic	381556	494	381062	0.991	0.290
191.11	Cancer of brain	neoplasms	rs12509595	0.000	0.055	1.000	9.983E-01	logistic	421187	790	420397	0.990	0.290
301	Personality disorders	mental disorders	rs12509595	0.000	0.084	1.000	9.991E-01	logistic	381968	346	381622	0.990	0.290
218	Benign neoplasm of uterus	neoplasms	rs12509595	0.000	0.029	1.000	9.996E-01	logistic	209861	2866	206995	0.967	0.290
427.1	Paroxysmal tachycardia, unspecified	circulatory system	rs35087646	0.166	0.046	1.181	3.047E-04	logistic	374981	3133	371848	0.999	0.072
316	Substance addiction and disorders	mental disorders	rs35087646	0.406	0.123	1.500	9.428E-04	logistic	403645	352	403293	0.999	0.072
427.12	Paroxysmal ventricular tachycardia	circulatory system	rs35087646	0.254	0.079	1.289	1.247E-03	logistic	372834	986	371848	0.998	0.072
414	Other forms of chronic heart disease	circulatory system	rs35087646	-0.140	0.046	0.869	2.213E-03	logistic	374670	4120	370550	0.999	0.072
352.2	Facial nerve disorders [CN7]	neurological	rs35087646	-0.380	0.124	0.684	2.283E-03	logistic	403092	677	402415	0.998	0.072
286	Coagulation defects	hematopoietic	rs35087646	-0.292	0.102	0.747	4.185E-03	logistic	418481	928	417553	0.997	0.072
530.11	GERD	digestive	rs35087646	-0.066	0.024	0.936	5.467E-03	logistic	371656	14868	356788	0.986	0.072
394	Rheumatic disease of the heart valves	circulatory system	rs35087646	-0.114	0.042	0.892	6.452E-03	logistic	406218	4804	401414	0.998	0.072
747.11	Cardiac shunt/ heart septal defect	congenital anomalies	rs35087646	-0.416	0.156	0.659	7.682E-03	logistic	422041	446	421595	0.998	0.072
529	Diseases and other conditions of the tongue	digestive	rs35087646	-0.539	0.209	0.583	9.736E-03	logistic	417990	280	417710	0.997	0.072
352	Disorders of other cranial nerves	neurological	rs35087646	-0.216	0.086	0.806	1.223E-02	logistic	403637	1222	402415	0.998	0.072
525	Other diseases of the teeth and supporting structures	digestive	rs35087646	0.274	0.110	1.316	1.267E-02	logistic	410027	491	409536	0.995	0.072
286.12	Congenital deficiency of other clotting factors (including factor VII	hematopoietic	rs35087646	-0.562	0.228	0.570	1.398E-02	logistic	417788	235	417553	0.997	0.072
433.31	Transient cerebral ischemia	circulatory system	rs35087646	0.179	0.073	1.196	1.467E-02	logistic	403901	1216	402685	0.997	0.072
153.2	Colon cancer	neoplasms	rs35087646	0.093	0.039	1.097	1.862E-02	logistic	375260	4540	370720	0.994	0.072
78	Viral warts & HPV	infectious diseases	rs35087646	-0.541	0.234	0.582	2.111E-02	logistic	415426	222	415204	0.997	0.072
369	Infection of the eye	sense organs	rs35087646	0.274	0.119	1.316	2.170E-02	logistic	411210	417	410793	0.998	0.072
740.1	Osteoarthritis; localized	musculoskeletal	rs35087646	-0.040	0.017	0.961	2.332E-02	logistic	374099	28241	345858	0.999	0.072
38.1	Gram negative septicemia	infectious diseases	rs35087646	0.305	0.136	1.357	2.548E-02	logistic	396961	314	396647	0.997	0.072
599.3	Dysuria	genitourinary	rs35087646	0.289	0.133	1.335	3.008E-02	logistic	390438	332	390106	0.999	0.072
276.11	Hyperosmolality and/or hypernatremia	endocrine/metabolic	rs35087646	-0.245	0.114	0.783	3.236E-02	logistic	396286	714	395572	0.999	0.072
702.1	Actinic keratosis	dermatologic	rs35087646	-0.208	0.099	0.812	3.593E-02	logistic	413997	925	413072	0.997	0.072
585.32	End stage renal disease	genitourinary	rs35087646	-0.205	0.098	0.814	3.667E-02	logistic	381994	932	381062	0.999	0.072
502	Postinflammatory pulmonary fibrosis	respiratory	rs35087646	-0.135	0.065	0.874	3.801E-02	logistic	393073	2016	391057	0.999	0.072
452	Other venous embolism and thrombosis	circulatory system	rs35087646	-0.367	0.179	0.693	4.020E-02	logistic	381290	324	380966	0.991	0.072
427.11	Paroxysmal supraventricular tachycardia	circulatory system	rs35087646	0.116	0.057	1.123	4.149E-02	logistic	373959	2111	371848	0.998	0.072
446.5	Giant cell arteritis	circulatory system	rs35087646	0.209	0.103	1.232	4.215E-02	logistic	406071	602	405469	0.999	0.072
575.1	Cholangitis	digestive	rs35087646	0.174	0.086	1.190	4.299E-02	logistic	395932	885	395047	0.999	0.072

ST31; PheWAS results for the lead cis-pQTLs of the 4 proteins prioritized (ACOX1, FGF5, FURIN, and MST1).
SNP = Single Nucleotide Polymorphism; SE = Standard error HWE_p = P-value for Hardy-Weinberg equilibrium test

Phenotype	Description	Group	SNP	Beta	SE	Odds tatio	P-value	Type	n_total	n_cases	n_controls	HWE_p	allele_freq
145	Cancer of mouth	neoplasms	rs35087646	-0.237	0.117	0.789	4.337E-02	logistic	421016	674	420342	0.996	0.072
396	Abnormal heart sounds	circulatory systen	rs35087646	0.165	0.083	1.180	4.671E-02	logistic	402357	943	401414	0.998	0.072
535.6	Duodenitis	digestive	rs35087646	-0.135	0.069	0.874	5.083E-02	logistic	363993	1779	362214	0.994	0.072
471	Nasal polyps	respiratory	rs35087646	-0.181	0.093	0.835	5.090E-02	logistic	397549	1032	396517	0.994	0.072
740	Osteoarthrosis	musculoskeletal	rs35087646	-0.027	0.014	0.973	5.155E-02	logistic	392374	46516	345858	0.999	0.072
599	Other symptoms/disorders or the urinary systen	genitourinary	rs35087646	0.058	0.030	1.060	5.279E-02	logistic	398281	8175	390106	0.999	0.072
270.33	Amyloidosis	endocrine/metabolic	rs35087646	-0.338	0.176	0.713	5.458E-02	logistic	422032	330	421702	0.997	0.072
149.4	Cancer of larynx	neoplasms	rs35087646	0.281	0.147	1.324	5.555E-02	logistic	420618	276	420342	0.996	0.072
479	Other upper respiratory diseasc	respiratory	rs35087646	-0.289	0.152	0.749	5.695E-02	logistic	396937	420	396517	0.993	0.072
211	Benign neoplasm of other parts of digestive systen	neoplasms	rs35087646	-0.093	0.049	0.912	5.737E-02	logistic	400517	3467	397050	0.996	0.072
687.1	Rash and other nonspecific skin eruption	dermatologic	rs35087646	0.148	0.078	1.160	5.799E-02	logistic	413803	1087	412716	0.998	0.072
728.7	Fasciitis	musculoskeletal	rs35087646	-0.129	0.068	0.879	5.858E-02	logistic	391018	1844	389174	0.993	0.072
433.3	Cerebral ischemia	circulatory systen	rs35087646	0.077	0.041	1.080	5.925E-02	logistic	407003	4318	402685	0.997	0.072
703.1	Ingrowing nail	dermatologic	rs35087646	-0.433	0.230	0.649	6.002E-02	logistic	421519	205	421314	0.998	0.072
217	Vascular hamartomas and non-neoplastic nevi	neoplasms	rs35087646	0.261	0.141	1.299	6.477E-02	logistic	414259	301	413958	0.993	0.072
112	Candidiasis	infectious diseases	rs35087646	-0.135	0.074	0.873	6.749E-02	logistic	419935	1555	418380	0.997	0.072
729	Other disorders of soft tissues	musculoskeletal	rs35087646	-0.372	0.205	0.689	6.965E-02	logistic	389423	249	389174	0.994	0.072
290.1	Dementias	mental disorders	rs35087646	0.075	0.041	1.078	6.979E-02	logistic	409256	4283	404973	0.998	0.072
241	Nontoxic nodular goiter	endocrine/metabolic	rs35087646	-0.195	0.108	0.823	7.053E-02	logistic	392996	766	392230	0.997	0.072
433	Cerebrovascular diseasc	circulatory systen	rs35087646	0.047	0.026	1.048	7.078E-02	logistic	414008	11323	402685	0.998	0.072
366	Cataract	sense organs	rs35087646	-0.029	0.016	0.972	7.177E-02	logistic	406937	35259	371678	0.997	0.072
427.41	Ventricular fibrillation and fluttei	circulatory systen	rs35087646	-0.346	0.197	0.707	7.901E-02	logistic	372113	265	371848	0.998	0.072
184	Cancer of other female genital organs	neoplasms	rs35087646	0.086	0.049	1.090	7.956E-02	logistic	207829	2992	204837	0.117	0.072
333	Extrapyramidal disease and abnormal movement disorders	neurological	rs35087646	-0.147	0.084	0.864	7.997E-02	logistic	400270	1223	399047	0.997	0.072
571.5	Other chronic nonalcoholic liver diseasc	digestive	rs35087646	-0.076	0.043	0.927	8.016E-02	logistic	404607	4333	400274	0.999	0.072
446	Polyarteritis nodosa and allied conditions	circulatory systen	rs35087646	0.132	0.075	1.141	8.057E-02	logistic	406656	1187	405469	0.999	0.072
286.7	Other and unspecified coagulation defects	hematopoietic	rs35087646	-0.339	0.194	0.713	8.140E-02	logistic	417818	265	417553	0.997	0.072
530.1	Esophagitis, GERD and related diseases	digestive	rs35087646	-0.030	0.017	0.971	8.178E-02	logistic	385890	29102	356788	0.990	0.072
286.1	Congenital coagulation defects	hematopoietic	rs35087646	-0.257	0.149	0.773	8.295E-02	logistic	417976	423	417553	0.997	0.072
602	Other disorders of prostate	genitourinary	rs35087646	-0.292	0.169	0.747	8.358E-02	logistic	156802	341	156461	1.000	0.071
702	Degenerative skin conditions and other dermatoses	dermatologic	rs35087646	-0.116	0.067	0.891	8.426E-02	logistic	407215	1867	405348	0.996	0.072
362.2	Degeneration of macula and posterior pole of retina	sense organs	rs35087646	-0.051	0.030	0.950	8.526E-02	logistic	406927	9170	397757	0.997	0.072
217.1	Nevus, non-neoplastic	neoplasms	rs35087646	0.254	0.148	1.290	8.629E-02	logistic	414233	275	413958	0.993	0.072
184.1	Malignant neoplasm of ovary and other uterine adnexa	neoplasms	rs35087646	0.087	0.051	1.091	8.641E-02	logistic	207626	2789	204837	0.096	0.072
594.3	Calculus of ureter	genitourinary	rs35087646	0.110	0.065	1.116	8.997E-02	logistic	414010	1634	412376	0.998	0.072
365.1	Open-angle glaucoma	sense organs	rs35087646	-0.123	0.073	0.884	9.209E-02	logistic	399325	1562	397763	0.997	0.072
701.2	Scar conditions and fibrosis of skin	dermatologic	rs35087646	-0.261	0.155	0.770	9.273E-02	logistic	415576	390	415186	0.997	0.072
276.5	Hypovolemia	endocrine/metabolic	rs35087646	-0.066	0.039	0.936	9.361E-02	logistic	400853	5281	395572	0.999	0.072
745	Pain in joint	musculoskeletal	rs35087646	-0.081	0.048	0.922	9.376E-02	logistic	414363	3501	410862	0.997	0.072
506	Empyema and pneumothorax	respiratory	rs35087646	-0.142	0.085	0.868	9.554E-02	logistic	392231	1174	391057	0.999	0.072
362	Other retinal disorders	sense organs	rs35087646	-0.048	0.029	0.954	9.696E-02	logistic	407698	9941	397757	0.998	0.072
458.1	Orthostatic hypotension	circulatory systen	rs35087646	-0.083	0.051	0.920	1.023E-01	logistic	375723	3151	372572	0.998	0.072
530.3	Stricture and stenosis of esophagus	digestive	rs35087646	-0.138	0.085	0.871	1.034E-01	logistic	357976	1188	356788	0.979	0.072
184.11	Malignant neoplasm of ovary	neoplasms	rs35087646	0.083	0.051	1.086	1.076E-01	logistic	207552	2715	204837	0.084	0.072
342	Hemiplegia	neurological	rs35087646	-0.094	0.058	0.910	1.078E-01	logistic	401449	2402	399047	0.997	0.072
279.11	Deficiency of humoral immunity	endocrine/metabolic	rs35087646	0.253	0.158	1.288	1.091E-01	logistic	423118	242	422876	0.998	0.072
701	Other hypertrophic and atrophic conditions of skir	dermatologic	rs35087646	-0.135	0.085	0.873	1.127E-01	logistic	416347	1161	415186	0.997	0.072
292.3	Memory loss	mental disorders	rs35087646	0.179	0.113	1.196	1.127E-01	logistic	405478	505	404973	0.999	0.072
735	Acquired foot deformities	musculoskeletal	rs35087646	-0.058	0.036	0.944	1.141E-01	logistic	408734	6080	402654	0.999	0.072
728.71	Contracture of palmar fascia [Dupuytren's disease]	musculoskeletal	rs35087646	-0.111	0.070	0.895	1.142E-01	logistic	390879	1705	389174	0.993	0.072
747	Cardiac and circulatory congenital anomalies	congenital anomalies	rs35087646	-0.139	0.088	0.870	1.145E-01	logistic	422691	1096	421595	0.998	0.072
172.3	Carcinoma in situ of skin	neoplasms	rs35087646	-0.239	0.152	0.787	1.146E-01	logistic	407616	408	407208	0.998	0.072
250.42	Other abnormal glucose	endocrine/metabolic	rs35087646	0.222	0.143	1.249	1.192E-01	logistic	386940	303	386637	0.991	0.072
585.34	Chronic Kidney Disease, Stage IV	genitourinary	rs35087646	0.120	0.077	1.127	1.197E-01	logistic	382213	1151	381062	0.999	0.072
204	Leukemia	neoplasms	rs35087646	-0.081	0.052	0.923	1.208E-01	logistic	418621	3008	415613	0.997	0.072
425	Cardiomyopathy	circulatory systen	rs35087646	0.094	0.060	1.098	1.215E-01	logistic	418683	1913	416770	0.997	0.072
327	Sleep disorders	neurological	rs35087646	0.165	0.107	1.180	1.221E-01	logistic	413949	565	413384	0.996	0.072
255	Disorders of adrenal glands	endocrine/metabolic	rs35087646	0.141	0.092	1.151	1.251E-01	logistic	416034	788	415246	0.998	0.072
727.1	Synovitis and tenosynovitis	musculoskeletal	rs35087646	-0.267	0.174	0.766	1.254E-01	logistic	389486	312	389174	0.994	0.072
530	Diseases of esophagus	digestive	rs35087646	-0.025	0.016	0.976	1.281E-01	logistic	388733	31945	356788	0.995	0.072
454.1	Varicose veins of lower extremity	circulatory systen	rs35087646	0.061	0.040	1.062	1.304E-01	logistic	385486	4520	380966	0.993	0.072
274.1	Gout	endocrine/metabolic	rs35087646	-0.066	0.044	0.936	1.306E-01	logistic	420024	4234	415790	0.998	0.072
362.29	Macular degeneration (senile) of retina NOS	sense organs	rs35087646	-0.069	0.046	0.933	1.313E-01	logistic	401665	3908	397757	0.998	0.072
287	Purpura and other hemorrhagic conditions	hematopoietic	rs35087646	-0.089	0.059	0.915	1.319E-01	logistic	419896	2343	417553	0.998	0.072
564.1	Irritable Bowel Syndrome	digestive	rs35087646	-0.063	0.042	0.939	1.325E-01	logistic	339145	4660	334485	0.988	0.072
509.2	Respiratory insufficiency	respiratory	rs35087646	0.107	0.072	1.113	1.335E-01	logistic	392402	1345	391057	0.999	0.072
747.1	Cardiac congenital anomalies	congenital anomalies	rs35087646	-0.143	0.095	0.867	1.337E-01	logistic	422536	941	421595	0.998	0.072
761	Cervicalgia	symptoms	rs35087646	-0.160	0.108	0.852	1.364E-01	logistic	422454	744	421710	0.997	0.072
425.1	Primary/intrinsic cardiomyopathies	circulatory systen	rs35087646	0.091	0.061	1.095	1.381E-01	logistic	418636	1866	416770	0.997	0.072
241.1	Nontoxic uninodular goiter	endocrine/metabolic	rs35087646	-0.310	0.210	0.733	1.392E-01	logistic	392455	225	392230	0.997	0.072
418.1	Precordial pain	circulatory systen	rs35087646	-0.097	0.066	0.907	1.428E-01	logistic	417539	1859	415680	0.998	0.072

ST31; PheWAS results for the lead cis-pQTLs of the 4 proteins prioritized (ACOX1, FGF5, FURIN, and MST1).

SNP = Single Nucleotide Polymorphism; SE = Standard error HWE_p = P-value for Hardy-Weinberg equilibrium test

Phenotype	Description	Group	SNP	Beta	SE	Odds tatio	P-value	Type	n_total	n_cases	n_controls	HWE_p	allele_freq
557.1	Celiac disease	digestive	rs35087646	0.089	0.061	1.093	1.435E-01	logistic	336383	1898	334485	0.990	0.072
155.1	Malignant neoplasm of liver, primary	neoplasms	rs35087646	-0.247	0.169	0.781	1.441E-01	logistic	394187	325	393862	0.996	0.072
281	Other deficiency anemia	hematopoietic	rs35087646	-0.116	0.080	0.890	1.443E-01	logistic	383766	1317	382449	0.999	0.072
395.1	Nonrheumatic mitral valve disorders	circulatory system	rs35087646	-0.068	0.047	0.934	1.463E-01	logistic	405090	3676	401414	0.999	0.072
365.2	Primary angle-closure glaucoma	sense organs	rs35087646	0.144	0.099	1.155	1.467E-01	logistic	398441	678	397763	0.996	0.072
593	Hematuria	genitourinary	rs35087646	0.055	0.038	1.057	1.472E-01	logistic	381996	5069	376927	0.998	0.072
473	Diseases of the larynx and vocal cords	respiratory	rs35087646	0.105	0.073	1.111	1.491E-01	logistic	397819	1302	396517	0.996	0.072
320	Meningitis	neurological	rs35087646	-0.254	0.176	0.775	1.492E-01	logistic	422374	303	422071	0.998	0.072
297.2	Suicide or self-inflicted injury	mental disorders	rs35087646	-0.240	0.168	0.787	1.528E-01	logistic	381948	326	381622	0.994	0.072
540	Appendiceal conditions	digestive	rs35087646	0.169	0.118	1.184	1.530E-01	logistic	422123	467	421656	0.998	0.072
276.1	Electrolyte imbalance	endocrine/metabolic	rs35087646	-0.039	0.028	0.962	1.565E-01	logistic	406083	10511	395572	0.999	0.072
324	Other CNS infection and poliomyelitis	neurological	rs35087646	0.192	0.136	1.212	1.578E-01	logistic	422418	347	422071	0.998	0.072
327.3	Sleep apnea	neurological	rs35087646	-0.054	0.038	0.947	1.595E-01	logistic	418773	5389	413384	0.996	0.072
364.4	Corneal degenerations	sense organs	rs35087646	0.108	0.077	1.114	1.615E-01	logistic	398917	1154	397763	0.997	0.072
780	Hypothermia/Chills	symptoms	rs35087646	0.234	0.167	1.264	1.618E-01	logistic	423421	221	423200	0.998	0.072
389.1	Sensorineural hearing loss	sense organs	rs35087646	-0.147	0.105	0.863	1.629E-01	logistic	409063	766	408297	0.996	0.072
281.1	Megaloblastic anemia	hematopoietic	rs35087646	-0.113	0.081	0.893	1.634E-01	logistic	383709	1260	382449	0.999	0.072
510	Other diseases of lung	respiratory	rs35087646	0.097	0.070	1.102	1.635E-01	logistic	421711	1427	420284	0.996	0.072
110	Dermatophytosis / Dermatomycosis	infectious diseases	rs35087646	-0.280	0.201	0.756	1.651E-01	logistic	418617	237	418380	0.998	0.072
497	Bronchitis	respiratory	rs35087646	0.203	0.146	1.225	1.657E-01	logistic	399105	296	398809	0.999	0.072
521.1	Dental caries	digestive	rs35087646	0.131	0.095	1.140	1.659E-01	logistic	410280	744	409536	0.996	0.072
738	Other acquired musculoskeletal deformity	musculoskeletal	rs35087646	-0.109	0.079	0.896	1.669E-01	logistic	403982	1328	402654	0.998	0.072
695	Erythematous conditions	dermatologic	rs35087646	-0.092	0.067	0.912	1.700E-01	logistic	407007	1801	405206	0.997	0.072
426.3	Bundle branch block	circulatory system	rs35087646	-0.066	0.048	0.936	1.708E-01	logistic	375377	3529	371848	0.997	0.071
465.2	Acute pharyngitis	respiratory	rs35087646	-0.267	0.195	0.766	1.710E-01	logistic	419940	248	419692	0.997	0.072
274	Gout and other crystal arthropathies	endocrine/metabolic	rs35087646	-0.058	0.042	0.944	1.726E-01	logistic	420276	4486	415790	0.998	0.072
261.2	Vitamin B-complex deficiencies	endocrine/metabolic	rs35087646	0.087	0.064	1.091	1.729E-01	logistic	413809	1712	412097	0.997	0.072
726.3	Bursitis	musculoskeletal	rs35087646	-0.298	0.219	0.742	1.732E-01	logistic	389380	206	389174	0.994	0.072
592.12	Chronic cystitis	genitourinary	rs35087646	-0.264	0.194	0.768	1.744E-01	logistic	377176	249	376927	0.999	0.072
764	Sciatica	symptoms	rs35087646	-0.107	0.079	0.898	1.752E-01	logistic	419302	1316	417986	0.998	0.072
276	Disorders of fluid, electrolyte, and acid-base balance	endocrine/metabolic	rs35087646	-0.031	0.023	0.970	1.761E-01	logistic	411277	15705	395572	0.999	0.072
501	Pneumonitis due to inhalation of food or vomitus	respiratory	rs35087646	-0.091	0.068	0.913	1.785E-01	logistic	392843	1786	391057	0.999	0.072
619.4	Noninflammatory disorders of vagina	genitourinary	rs35087646	0.222	0.166	1.249	1.807E-01	logistic	217194	228	216966	0.143	0.072
198.3	Secondary malignant neoplasm of digestive systems	neoplasms	rs35087646	0.069	0.051	1.071	1.807E-01	logistic	388741	2735	386006	0.998	0.072
440.2	Atherosclerosis of the extremities	circulatory system	rs35087646	-0.131	0.098	0.877	1.814E-01	logistic	406343	874	405469	0.999	0.072
521	Diseases of hard tissues of teeth	digestive	rs35087646	0.123	0.092	1.130	1.816E-01	logistic	410337	801	409536	0.996	0.072
575	Other biliary tract disease	digestive	rs35087646	0.059	0.044	1.061	1.820E-01	logistic	398734	3687	395047	0.999	0.072
560.2	Impaction of intestine	digestive	rs35087646	-0.273	0.205	0.761	1.844E-01	logistic	334714	229	334485	0.986	0.072
262	Mineral deficiency NEC	endocrine/metabolic	rs35087646	0.181	0.137	1.198	1.877E-01	logistic	412440	343	412097	0.997	0.072
537	Other disorders of stomach and duodenum	digestive	rs35087646	-0.088	0.067	0.916	1.878E-01	logistic	364031	1817	362214	0.991	0.072
752	Nervous system congenital anomalies	congenital anomalies	rs35087646	-0.183	0.139	0.833	1.895E-01	logistic	422992	453	422539	0.997	0.072
287.3	Thrombocytopenia	hematopoietic	rs35087646	-0.079	0.060	0.924	1.900E-01	logistic	419777	2224	417553	0.998	0.072
947	Urticaria	dermatologic	rs35087646	-0.280	0.214	0.755	1.909E-01	logistic	415196	209	414987	0.997	0.072
345.11	Generalized convulsive epilepsy	neurological	rs35087646	0.168	0.128	1.182	1.920E-01	logistic	399441	394	399047	0.997	0.072
740.9	Osteoarthrosis NOS	musculoskeletal	rs35087646	-0.030	0.023	0.971	1.924E-01	logistic	361594	15736	345858	0.999	0.072
695.3	Rosacea	dermatologic	rs35087646	-0.253	0.194	0.776	1.926E-01	logistic	405456	250	405206	0.998	0.072
721	Spondylosis and allied disorders	musculoskeletal	rs35087646	-0.085	0.066	0.918	1.939E-01	logistic	410559	1889	408670	0.996	0.072
770	Myalgia and myositis unspecified	symptoms	rs35087646	-0.086	0.067	0.917	1.947E-01	logistic	422525	1839	420686	0.997	0.072
724.2	Disorders of coccyx	musculoskeletal	rs35087646	-0.273	0.210	0.761	1.947E-01	logistic	408885	215	408670	0.997	0.072
394.1	Mitral valve stenosis and aortic valve stenosis	circulatory system	rs35087646	-0.111	0.086	0.895	1.960E-01	logistic	402530	1116	401414	0.998	0.072
395.4	Nonrheumatic pulmonary valve disorders	circulatory system	rs35087646	-0.218	0.169	0.804	1.977E-01	logistic	401735	321	401414	0.998	0.072
601.1	Prostatitis	genitourinary	rs35087646	-0.141	0.110	0.868	1.985E-01	logistic	157164	703	156461	1.000	0.071
625	Pain and other symptoms associated with female genital organs	genitourinary	rs35087646	-0.227	0.177	0.797	2.001E-01	logistic	218575	291	218284	0.595	0.072
721.8	Other allied disorders of spine	musculoskeletal	rs35087646	-0.280	0.219	0.756	2.013E-01	logistic	408871	201	408670	0.997	0.072
586	Other disorders of the kidney and ureters	genitourinary	rs35087646	0.055	0.043	1.057	2.015E-01	logistic	384904	3842	381062	0.999	0.072
315	Develomental delays and disorders	mental disorders	rs35087646	-0.143	0.112	0.867	2.016E-01	logistic	422717	680	422037	0.998	0.072
368.2	Diplopia and disorders of binocular vision	sense organs	rs35087646	0.143	0.113	1.154	2.032E-01	logistic	416069	524	415545	0.993	0.072
350.1	Abnormal involuntary movements	neurological	rs35087646	0.117	0.092	1.124	2.046E-01	logistic	421010	800	420210	0.997	0.072
530.14	Reflux esophagitis	digestive	rs35087646	0.060	0.047	1.061	2.055E-01	logistic	360032	3244	356788	0.972	0.072
281.13	Folate-deficiency anemia	hematopoietic	rs35087646	-0.193	0.153	0.824	2.073E-01	logistic	382829	380	382449	0.999	0.072
595	Hydronephrosis	genitourinary	rs35087646	0.105	0.083	1.110	2.092E-01	logistic	413362	986	412376	0.997	0.072
159.4	Malignant neoplasm of retroperitoneum and peritoneum	neoplasms	rs35087646	0.194	0.156	1.215	2.129E-01	logistic	394127	265	393862	0.996	0.072
433.1	Occlusion and stenosis of precerebral arteries	circulatory system	rs35087646	0.083	0.067	1.087	2.152E-01	logistic	404257	1572	402685	0.997	0.072
290.13	Senile dementia	mental disorders	rs35087646	0.070	0.056	1.072	2.158E-01	logistic	407253	2280	404973	0.998	0.072
798.1	Chronic fatigue syndrome	symptoms	rs35087646	-0.146	0.118	0.864	2.159E-01	logistic	414937	617	414320	0.996	0.072
182	Malignant neoplasm of uterus	neoplasms	rs35087646	-0.100	0.082	0.904	2.184E-01	logistic	197217	1245	195972	0.054	0.072
717	Polymyalgia Rheumatica	musculoskeletal	rs35087646	-0.071	0.058	0.931	2.191E-01	logistic	422397	2442	419955	0.998	0.072
290.11	Alzheimer's disease	mental disorders	rs35087646	0.068	0.055	1.070	2.193E-01	logistic	407367	2394	404973	0.999	0.072
751.22	Other specified congenital anomalies of kidney	congenital anomalies	rs35087646	0.168	0.137	1.183	2.193E-01	logistic	420601	348	420253	0.998	0.072
540.1	Appendicitis	digestive	rs35087646	0.172	0.141	1.188	2.208E-01	logistic	421984	328	421656	0.997	0.072
508	Pulmonary collapse; interstitial and compensatory emphysema	respiratory	rs35087646	-0.056	0.046	0.946	2.226E-01	logistic	394890	3833	391057	0.999	0.072

ST31; PheWAS results for the lead cis-pQTLs of the 4 proteins prioritized (ACOX1, FGF5, FURIN, and MST1).

SNP = Single Nucleotide Polymorphism; SE = Standard error HWE_p = P-value for Hardy-Weinberg equilibrium test

Phenotype	Description	Group	SNP	Beta	SE	Odds tatio	P-value	Type	n_total	n_cases	n_controls	HWE_p	allele_freq
636	Early or threatened labor; hemorrhage in early pregnanc	pregnancy complications	rs35087646	-0.156	0.129	0.856	2.266E-01	logistic	223122	530	222592	0.603	0.072
150	Cancer of esophagus	neoplasms	rs35087646	-0.092	0.076	0.912	2.272E-01	logistic	395291	1429	393862	0.995	0.072
496	Chronic airway obstruction	respiratory	rs35087646	0.025	0.021	1.026	2.283E-01	logistic	416701	17892	398809	0.996	0.072
428	Congestive heart failure; nonhypertensive	circulatory system	rs35087646	-0.038	0.031	0.963	2.291E-01	logistic	417859	8156	409703	0.997	0.072
851	Complications of transplants and reattached limbs	injuries & poisonings	rs35087646	-0.234	0.194	0.791	2.291E-01	logistic	416895	243	416652	0.997	0.072
782.3	Edema	symptoms	rs35087646	-0.069	0.057	0.933	2.292E-01	logistic	419509	2451	417058	0.998	0.072
977	Personal history of allergy to medicinal agents	injuries & poisonings	rs35087646	-0.037	0.031	0.963	2.308E-01	logistic	375378	8205	367173	0.997	0.072
218	Benign neoplasm of uterus	neoplasms	rs35087646	-0.063	0.053	0.939	2.314E-01	logistic	209861	2866	206995	0.209	0.072
512	Other symptoms of respiratory system	respiratory	rs35087646	-0.042	0.035	0.959	2.325E-01	logistic	411090	6491	404599	0.994	0.072
727	Other disorders of synovium, tendon, and bursa	musculoskeletal	rs35087646	-0.065	0.055	0.937	2.327E-01	logistic	391842	2668	389174	0.995	0.072
961.1	Poisoning/allergy of sulfonamides	injuries & poisonings	rs35087646	-0.138	0.116	0.871	2.327E-01	logistic	367807	634	367173	0.998	0.072
530.9	Heartburn	digestive	rs35087646	0.189	0.160	1.208	2.372E-01	logistic	357038	250	356788	0.977	0.072
153.3	Malignant neoplasm of rectum, rectosigmoid junction, and anus	neoplasms	rs35087646	-0.062	0.053	0.940	2.379E-01	logistic	373609	2889	370720	0.989	0.072
244.4	Hypothyroidism NOS	endocrine/metabolic	rs35087646	0.025	0.021	1.025	2.385E-01	logistic	410296	18066	392230	0.997	0.072
574.1	Cholelithiasis	digestive	rs35087646	-0.034	0.029	0.966	2.397E-01	logistic	404312	9265	395047	0.999	0.072
149.1	Cancer of oropharynx	neoplasms	rs35087646	-0.178	0.152	0.837	2.399E-01	logistic	420725	383	420342	0.995	0.072
599.4	Urinary incontinence	genitourinary	rs35087646	0.061	0.052	1.062	2.422E-01	logistic	392806	2700	390106	0.999	0.072
473.3	Paralysis/spasm of vocal cords or larynx	respiratory	rs35087646	-0.236	0.202	0.790	2.429E-01	logistic	396742	225	396517	0.994	0.072
365	Glaucoma	sense organs	rs35087646	-0.038	0.033	0.963	2.441E-01	logistic	405209	7446	397763	0.996	0.072
579	Other symptoms involving abdomen and pelvis	digestive	rs35087646	0.095	0.082	1.100	2.448E-01	logistic	384340	1037	383303	0.997	0.072
738.4	Acquired spondylolisthesis	musculoskeletal	rs35087646	-0.097	0.083	0.908	2.449E-01	logistic	403846	1192	402654	0.998	0.072
204.4	Multiple myeloma	neoplasms	rs35087646	-0.098	0.085	0.907	2.470E-01	logistic	416757	1144	415613	0.995	0.072
394.2	Mitral valve disease	circulatory system	rs35087646	0.167	0.145	1.182	2.478E-01	logistic	401726	312	401414	0.999	0.072
300.13	Phobia	mental disorders	rs35087646	-0.137	0.119	0.872	2.502E-01	logistic	382219	597	381622	0.994	0.072
586.2	Cyst of kidney, acquired	genitourinary	rs35087646	0.071	0.062	1.074	2.505E-01	logistic	382916	1854	381062	0.999	0.072
513	Respiratory abnormalities	respiratory	rs35087646	-0.163	0.142	0.850	2.512E-01	logistic	423122	431	422691	0.998	0.072
454	Varicose veins	circulatory system	rs35087646	0.045	0.039	1.046	2.514E-01	logistic	385827	4861	380966	0.993	0.072
634.1	Missed abortion/Hydatidiform mole	pregnancy complications	rs35087646	-0.237	0.207	0.789	2.520E-01	logistic	222807	215	222592	0.590	0.072
285.22	Anemia in neoplastic disease	hematopoietic	rs35087646	0.161	0.141	1.175	2.524E-01	logistic	382780	331	382449	0.999	0.072
377	Disorders of optic nerve and visual pathways	sense organs	rs35087646	-0.177	0.155	0.837	2.534E-01	logistic	412293	365	411928	0.997	0.072
530.13	Barrett's esophagus	digestive	rs35087646	-0.048	0.043	0.953	2.544E-01	logistic	361195	4407	356788	0.986	0.072
803.3	Fracture of clavicle or scapula	injuries & poisonings	rs35087646	0.112	0.098	1.118	2.563E-01	logistic	397087	709	396378	0.993	0.072
737	Curvature of spine	musculoskeletal	rs35087646	0.100	0.088	1.105	2.573E-01	logistic	403550	896	402654	0.997	0.072
241.2	Nontoxic multinodular goiter	endocrine/metabolic	rs35087646	-0.219	0.195	0.803	2.616E-01	logistic	392467	237	392230	0.997	0.072
555.21	Ulcerative colitis (chronic)	digestive	rs35087646	-0.168	0.151	0.845	2.639E-01	logistic	334866	381	334485	0.986	0.072
473.4	Voice disturbance	respiratory	rs35087646	0.168	0.151	1.183	2.646E-01	logistic	396801	284	396517	0.994	0.072
218.1	Uterine leiomyoma	neoplasms	rs35087646	-0.060	0.054	0.942	2.651E-01	logistic	209756	2761	206995	0.219	0.072
411.3	Angina pectoris	circulatory system	rs35087646	-0.025	0.022	0.975	2.665E-01	logistic	387093	16543	370550	0.999	0.072
608	Other disorders of male genital organs	genitourinary	rs35087646	-0.117	0.105	0.890	2.669E-01	logistic	162533	755	161778	1.000	0.071
8	Intestinal infection	infectious diseases	rs35087646	-0.072	0.065	0.931	2.673E-01	logistic	419231	1915	417316	0.997	0.072
433.5	Cerebral aneurysm	circulatory system	rs35087646	0.135	0.123	1.145	2.717E-01	logistic	403130	445	402685	0.997	0.072
724.1	Disorders of sacrum	musculoskeletal	rs35087646	-0.218	0.199	0.804	2.721E-01	logistic	408900	230	408670	0.997	0.072
79	Viral infection	infectious diseases	rs35087646	-0.098	0.090	0.906	2.723E-01	logistic	416221	1017	415204	0.996	0.072
158	Neoplasm of unspecified nature of digestive system	neoplasms	rs35087646	-0.167	0.152	0.846	2.727E-01	logistic	394238	376	393862	0.995	0.072
627.1	Postmenopausal bleeding	genitourinary	rs35087646	0.069	0.063	1.072	2.734E-01	logistic	194538	1791	192747	0.308	0.072
628	Ovarian cyst	genitourinary	rs35087646	0.084	0.077	1.087	2.739E-01	logistic	193944	1197	192747	0.234	0.072
474	Acute and chronic tonsillitis	respiratory	rs35087646	0.145	0.132	1.156	2.742E-01	logistic	396895	378	396517	0.995	0.072
585.2	Renal failure NOS	genitourinary	rs35087646	-0.116	0.106	0.891	2.744E-01	logistic	381806	744	381062	0.999	0.072
426.32	Left bundle branch block	circulatory system	rs35087646	-0.056	0.051	0.946	2.756E-01	logistic	374914	3066	371848	0.998	0.071
600	Hyperplasia of prostate	genitourinary	rs35087646	-0.029	0.026	0.972	2.759E-01	logistic	169018	12557	156461	1.000	0.071
300.9	Posttraumatic stress disorder	mental disorders	rs35087646	-0.201	0.185	0.818	2.777E-01	logistic	381881	259	381622	0.994	0.072
275.5	Disorders of calcium/phosphorus metabolism	endocrine/metabolic	rs35087646	-0.064	0.059	0.938	2.787E-01	logistic	418502	2317	416185	0.998	0.072
614.1	Pelvic peritoneal adhesions, female (postoperative) (postinfection)	genitourinary	rs35087646	-0.160	0.148	0.852	2.798E-01	logistic	217833	395	217438	0.336	0.072
180	Cervical cancer and dysplasia	neoplasms	rs35087646	0.058	0.054	1.059	2.823E-01	logistic	198422	2518	195904	0.162	0.072
789	Nausea and vomiting	symptoms	rs35087646	0.035	0.033	1.036	2.825E-01	logistic	407825	6923	400902	1.000	0.072
180.3	Cervical intraepithelial neoplasia [CIN] [Cervical dysplasia]	neoplasms	rs35087646	0.127	0.118	1.136	2.828E-01	logistic	196385	481	195904	0.101	0.072
747.12	Valvular heart disease/ heart chambers	congenital anomalies	rs35087646	0.163	0.152	1.177	2.840E-01	logistic	421875	280	421595	0.998	0.072
560	Intestinal obstruction without mention of hernia	digestive	rs35087646	-0.050	0.047	0.951	2.848E-01	logistic	338110	3625	334485	0.985	0.072
284	Aplastic anemia	hematopoietic	rs35087646	0.103	0.097	1.109	2.863E-01	logistic	383179	730	382449	0.998	0.072
800.2	Fracture of unspecified part of femur	injuries & poisonings	rs35087646	-0.220	0.206	0.803	2.864E-01	logistic	396592	214	396378	0.992	0.072
752.1	Neural tube defects	congenital anomalies	rs35087646	-0.157	0.148	0.855	2.887E-01	logistic	422933	394	422539	0.997	0.072
368.1	Amblyopia	sense organs	rs35087646	-0.139	0.131	0.870	2.889E-01	logistic	416041	496	415545	0.993	0.072
585.31	Renal dialysis	genitourinary	rs35087646	-0.114	0.108	0.892	2.898E-01	logistic	381775	713	381062	0.999	0.072
430.1	Subarachnoid hemorrhage	circulatory system	rs35087646	-0.112	0.106	0.894	2.907E-01	logistic	403416	731	402685	0.997	0.072
198.1	Secondary malignancy of lymph nodes	neoplasms	rs35087646	-0.033	0.031	0.967	2.915E-01	logistic	394015	8009	386006	0.998	0.072
279	Disorders involving the immune mechanism	endocrine/metabolic	rs35087646	0.141	0.134	1.151	2.920E-01	logistic	423247	371	422876	0.997	0.072
244.1	Secondary hypothyroidism	endocrine/metabolic	rs35087646	-0.086	0.082	0.918	2.927E-01	logistic	393443	1213	392230	0.997	0.072
381.11	Suppurative and unspecified otitis media	sense organs	rs35087646	-0.197	0.188	0.821	2.952E-01	logistic	418969	253	418716	0.996	0.072
415	Pulmonary heart disease	circulatory system	rs35087646	-0.072	0.069	0.931	2.964E-01	logistic	414538	1700	412838	0.998	0.072
293.1	Swelling, mass, or lump in head and neck [Space-occupying lesion, intracranial NOS]	mental disorders	rs35087646	0.151	0.145	1.163	2.967E-01	logistic	420026	315	419711	0.997	0.072
427.4	Cardiac arrest and ventricular fibrillation	circulatory system	rs35087646	-0.099	0.095	0.905	2.968E-01	logistic	372755	907	371848	0.997	0.072

ST31; PheWAS results for the lead cis-pQTLs of the 4 proteins prioritized (ACOX1, FGF5, FURIN, and MST1).

SNP = Single Nucleotide Polymorphism; SE = Standard error HWE_p = P-value for Hardy-Weinberg equilibrium test

Phenotype	Description	Group	SNP	Beta	SE	Odds tatio	P-value	Type	n_total	n_cases	n_controls	HWE_p	allele_freq
564	Functional digestive disorders	digestive	rs35087646	-0.021	0.020	0.979	2.981E-01	logistic	354387	19902	334485	0.970	0.071
763	Thoracic or lumbosacral neuritis or radiculitis, unspecifiec	symptoms	rs35087646	-0.183	0.177	0.833	2.999E-01	logistic	418269	283	417986	0.998	0.072
687.4	Disturbance of skin sensation	dermatologic	rs35087646	0.074	0.071	1.076	3.007E-01	logistic	414113	1397	412716	0.997	0.072
227.1	Benign neoplasm of adrenal glanc	neoplasms	rs35087646	0.164	0.159	1.178	3.041E-01	logistic	421144	257	420887	0.997	0.072
601	Inflammatory diseases of prostate	genitourinary	rs35087646	-0.096	0.094	0.908	3.065E-01	logistic	157382	921	156461	1.000	0.071
185	Cancer of prostate	neoplasms	rs35087646	0.028	0.027	1.028	3.067E-01	logistic	167540	11079	156461	1.000	0.072
574.3	Cholecystitis without cholelithiasis	digestive	rs35087646	0.078	0.077	1.081	3.074E-01	logistic	396244	1197	395047	0.999	0.072
416	Cardiomegaly	circulatory system	rs35087646	-0.043	0.042	0.958	3.075E-01	logistic	417255	4417	412838	0.999	0.072
470	Septal Deviations/Turbinate Hypertrophy	respiratory	rs35087646	0.067	0.066	1.069	3.078E-01	logistic	398163	1646	396517	0.994	0.072
216	Benign neoplasm of skin	neoplasms	rs35087646	-0.087	0.085	0.917	3.081E-01	logistic	415074	1116	413958	0.992	0.072
571.8	Liver abscess and sequelae of chronic liver disease	digestive	rs35087646	0.070	0.068	1.072	3.085E-01	logistic	401788	1514	400274	0.998	0.072
333.1	Essential tremor	neurological	rs35087646	-0.128	0.126	0.880	3.119E-01	logistic	399579	532	399047	0.997	0.072
514	Abnormal findings examination of lungs	respiratory	rs35087646	0.059	0.058	1.060	3.121E-01	logistic	419804	2147	417657	0.996	0.072
375	Disorders of lacrimal system	sense organs	rs35087646	-0.075	0.074	0.928	3.128E-01	logistic	413379	1451	411928	0.997	0.072
573.9	Abnormal serum enzyme levels	digestive	rs35087646	0.175	0.174	1.191	3.139E-01	logistic	400489	215	400274	0.998	0.072
440	Atherosclerosis	circulatory system	rs35087646	-0.087	0.087	0.916	3.141E-01	logistic	406545	1076	405469	0.999	0.072
425.12	Other hypertrophic cardiomyopathy	circulatory system	rs35087646	0.155	0.154	1.168	3.142E-01	logistic	417044	274	416770	0.996	0.072
359	Muscular dystrophies and other myopathies	neurological	rs35087646	-0.179	0.179	0.836	3.169E-01	logistic	419442	274	419168	0.995	0.072
184.2	Cancer of other female genital organs (excluding uterus and ovary	neoplasms	rs35087646	-0.192	0.192	0.825	3.172E-01	logistic	205080	243	204837	0.168	0.072
250.11	Type 1 diabetes with ketoacidosis	endocrine/metabolic	rs35087646	-0.188	0.189	0.828	3.182E-01	logistic	386885	248	386637	0.993	0.072
715.2	Ankylosing spondylitis	musculoskeletal	rs35087646	0.122	0.123	1.130	3.185E-01	logistic	408956	449	408507	0.997	0.072
353	Nerve root and plexus disorders	neurological	rs35087646	-0.167	0.168	0.846	3.198E-01	logistic	402720	305	402415	0.998	0.072
707	Chronic ulcer of skin	dermatologic	rs35087646	0.039	0.039	1.040	3.203E-01	logistic	420904	4774	416130	0.997	0.072
427.7	Tachycardia NOS	circulatory system	rs35087646	-0.064	0.065	0.938	3.223E-01	logistic	373741	1893	371848	0.999	0.071
344	Other paralytic syndromes	neurological	rs35087646	0.100	0.102	1.106	3.234E-01	logistic	399714	667	399047	0.997	0.072
596	Other disorders of bladder	genitourinary	rs35087646	-0.035	0.036	0.966	3.252E-01	logistic	407805	6251	401554	0.998	0.072
571	Chronic liver disease and cirrhosis	digestive	rs35087646	-0.036	0.037	0.965	3.277E-01	logistic	406135	5861	400274	0.999	0.072
348	Other conditions of brain	neurological	rs35087646	0.099	0.102	1.104	3.309E-01	logistic	399717	670	399047	0.997	0.072
441	Vascular insufficiency of intestine	circulatory system	rs35087646	-0.107	0.110	0.898	3.311E-01	logistic	406151	682	405469	0.999	0.072
578.9	Hemorrhage of gastrointestinal tract	digestive	rs35087646	0.048	0.050	1.049	3.313E-01	logistic	386245	2942	383303	0.997	0.072
426.21	First degree AV block	circulatory system	rs35087646	0.061	0.063	1.063	3.315E-01	logistic	373661	1813	371848	0.997	0.072
594	Urinary calculus	genitourinary	rs35087646	0.035	0.036	1.035	3.317E-01	logistic	418195	5819	412376	0.999	0.072
442.8	Aneurysm of other specified artery	circulatory system	rs35087646	-0.177	0.182	0.838	3.326E-01	logistic	405731	262	405469	0.999	0.072
800.1	Fracture of neck of femur	injuries & poisonings	rs35087646	0.057	0.060	1.059	3.366E-01	logistic	398432	2054	396378	0.994	0.072
578	Gastrointestinal hemorrhage	digestive	rs35087646	0.027	0.028	1.027	3.378E-01	logistic	392729	9426	383303	0.997	0.072
740.12	Osteoarthrosis, localized, secondary	musculoskeletal	rs35087646	-0.134	0.139	0.875	3.383E-01	logistic	346286	428	345858	1.000	0.072
427	Cardiac dysrhythmias	circulatory system	rs35087646	0.017	0.018	1.017	3.391E-01	logistic	398369	26521	371848	0.997	0.072
500	Lung disease due to external agents	respiratory	rs35087646	-0.111	0.116	0.895	3.393E-01	logistic	391675	618	391057	0.999	0.072
281.11	Pernicious anemia	hematopoietic	rs35087646	-0.101	0.106	0.904	3.410E-01	logistic	383186	737	382449	0.999	0.072
242	Thyrotoxicosis with or without goiter	endocrine/metabolic	rs35087646	0.064	0.067	1.066	3.432E-01	logistic	393812	1582	392230	0.996	0.072
531.3	Duodenal ulcer	digestive	rs35087646	-0.080	0.085	0.923	3.441E-01	logistic	412469	1130	411339	0.998	0.072
214.1	Lipoma of skin and subcutaneous tissue	neoplasms	rs35087646	-0.100	0.106	0.905	3.453E-01	logistic	414958	731	414227	0.995	0.072
523	Gingival and periodontal diseases	digestive	rs35087646	0.123	0.130	1.131	3.460E-01	logistic	409931	395	409536	0.994	0.072
426.23	Second degree AV block	circulatory system	rs35087646	0.098	0.104	1.103	3.465E-01	logistic	372488	640	371848	0.998	0.072
443.1	Raynaud's syndrome	circulatory system	rs35087646	0.063	0.067	1.065	3.476E-01	logistic	407046	1577	405469	0.999	0.072
345.1	Epilepsy	neurological	rs35087646	0.105	0.112	1.110	3.485E-01	logistic	399597	550	399047	0.997	0.072
369.5	Conjunctivitis, infectious	sense organs	rs35087646	0.167	0.179	1.182	3.496E-01	logistic	410999	206	410793	0.998	0.072
428.2	Heart failure NOS	circulatory system	rs35087646	-0.038	0.040	0.963	3.499E-01	logistic	414622	4919	409703	0.997	0.072
279.1	Immunity deficiency	endocrine/metabolic	rs35087646	0.133	0.143	1.143	3.526E-01	logistic	423199	323	422876	0.997	0.072
8.6	Viral Enteritis	infectious diseases	rs35087646	-0.119	0.128	0.888	3.540E-01	logistic	417821	505	417316	0.997	0.072
472	Chronic pharyngitis and nasopharyngitis	respiratory	rs35087646	0.147	0.159	1.158	3.555E-01	logistic	396779	262	396517	0.993	0.072
368.9	Subjective visual disturbances	sense organs	rs35087646	-0.118	0.128	0.888	3.558E-01	logistic	416057	512	415545	0.994	0.072
530.2	Esophageal bleeding (varices/hemorrhage	digestive	rs35087646	0.118	0.127	1.125	3.559E-01	logistic	357206	418	356788	0.980	0.072
244	Hypothyroidism	endocrine/metabolic	rs35087646	0.019	0.020	1.019	3.588E-01	logistic	411296	19066	392230	0.997	0.072
301	Personality disorders	mental disorders	rs35087646	0.128	0.139	1.136	3.600E-01	logistic	381968	346	381622	0.994	0.072
527	Diseases of the salivary glands	digestive	rs35087646	-0.180	0.198	0.835	3.646E-01	logistic	417933	223	417710	0.997	0.072
351	Other peripheral nerve disorders	neurological	rs35087646	-0.031	0.034	0.970	3.649E-01	logistic	409126	6711	402415	0.999	0.072
79.9	Viremia, NOS	infectious diseases	rs35087646	-0.106	0.118	0.900	3.689E-01	logistic	415798	594	415204	0.996	0.072
707.2	Chronic ulcer of leg or foot	dermatologic	rs35087646	0.053	0.059	1.054	3.697E-01	logistic	418232	2102	416130	0.998	0.072
622	Polyp of female genital organs	genitourinary	rs35087646	-0.050	0.056	0.951	3.699E-01	logistic	216102	2530	213572	0.460	0.072
619.2	Disorders of uterus, NEC	genitourinary	rs35087646	-0.129	0.143	0.879	3.699E-01	logistic	217379	413	216966	0.241	0.072
276.13	Hyperpotassemia	endocrine/metabolic	rs35087646	-0.061	0.068	0.941	3.709E-01	logistic	397299	1727	395572	0.999	0.072
378.1	Strabismus (not specified as paralytic)	sense organs	rs35087646	0.141	0.157	1.151	3.714E-01	logistic	412196	268	411928	0.996	0.072
722	Intervertebral disc disorders	musculoskeletal	rs35087646	0.092	0.102	1.096	3.716E-01	logistic	409333	663	408670	0.997	0.072
297	Suicidal ideation or attempt	mental disorders	rs35087646	-0.136	0.153	0.873	3.731E-01	logistic	381982	360	381622	0.994	0.072
750.2	Lower gastrointestinal congenital anomalies	congenital anomalies	rs35087646	-0.187	0.210	0.830	3.742E-01	logistic	420453	200	420253	0.998	0.072
189.1	Cancer of kidney and renal pelvis	neoplasms	rs35087646	0.068	0.077	1.070	3.753E-01	logistic	418392	1205	417187	0.998	0.072
246	Other disorders of thyroid	endocrine/metabolic	rs35087646	-0.148	0.168	0.862	3.764E-01	logistic	392532	302	392230	0.997	0.072
702.2	Seborrheic keratosis	dermatologic	rs35087646	-0.110	0.124	0.896	3.766E-01	logistic	413611	539	413072	0.996	0.072
260.6	Anorexia	endocrine/metabolic	rs35087646	0.079	0.090	1.083	3.773E-01	logistic	412968	871	412097	0.998	0.072
965.1	Opiates and related narcotics causing adverse effects in therapeutic use	injuries & poisonings	rs35087646	-0.052	0.059	0.949	3.773E-01	logistic	369447	2274	367173	0.998	0.072

ST31; PheWAS results for the lead cis-pQTLs of the 4 proteins prioritized (ACOX1, FGF5, FURIN, and MST1).
SNP = Single Nucleotide Polymorphism; SE = Standard error HWE_p = P-value for Hardy-Weinberg equilibrium test

Phenotype	Description	Group	SNP	Beta	SE	Odds tatio	P-value	Type	n_total	n_cases	n_controls	HWE_p	allele_freq
454.11	Varicose veins of lower extremity, symptomtic	circulatory system	rs35087646	0.095	0.108	1.100	3.782E-01	logistic	381560	594	380966	0.990	0.072
348.2	Cerebral edema and compression of brain	neurological	rs35087646	0.118	0.134	1.125	3.789E-01	logistic	399425	378	399047	0.997	0.072
415.2	Chronic pulmonary heart disease	circulatory system	rs35087646	-0.061	0.069	0.941	3.797E-01	logistic	414491	1653	412838	0.998	0.072
157	Pancreatic cancer	neoplasms	rs35087646	0.063	0.072	1.065	3.808E-01	logistic	395256	1394	393862	0.995	0.072
202.21	Nodular lymphoma	neoplasms	rs35087646	0.107	0.122	1.112	3.816E-01	logistic	416077	464	415613	0.995	0.072
395	Heart valve disorders	circulatory system	rs35087646	-0.025	0.029	0.975	3.829E-01	logistic	411117	9703	401414	0.999	0.072
252.2	Hypoparathyroidism	endocrine/metabolic	rs35087646	0.149	0.171	1.161	3.846E-01	logistic	415471	225	415246	0.997	0.072
174.11	Malignant neoplasm of female breast	neoplasms	rs35087646	-0.026	0.030	0.975	3.860E-01	logistic	212959	9200	203759	0.228	0.072
401.1	Essential hypertension	circulatory system	rs35087646	-0.009	0.011	0.991	3.862E-01	logistic	389876	99213	290663	0.998	0.072
695.7	Prurigo and Lichen	dermatologic	rs35087646	-0.144	0.167	0.866	3.898E-01	logistic	405510	304	405206	0.997	0.072
386.2	Peripheral or central vertigo	sense organs	rs35087646	-0.099	0.116	0.906	3.910E-01	logistic	410281	616	409665	0.995	0.072
496.3	Bronchiectasis	respiratory	rs35087646	0.038	0.044	1.039	3.911E-01	logistic	402578	3769	398809	0.998	0.072
80	Postoperative infection	infectious diseases	rs35087646	0.053	0.062	1.054	3.934E-01	logistic	414741	1897	412844	0.998	0.072
586.4	Stricture/obstruction of ureter	genitourinary	rs35087646	-0.084	0.099	0.919	3.937E-01	logistic	381896	834	381062	0.999	0.072
568	Other disorders of peritoneum	digestive	rs35087646	-0.063	0.074	0.939	3.944E-01	logistic	385305	1466	383839	0.996	0.072
411.8	Other chronic ischemic heart disease, unspecified	circulatory system	rs35087646	-0.018	0.021	0.982	3.947E-01	logistic	389207	18657	370550	0.999	0.072
189	Cancer of urinary organs (incl. kidney and bladder)	neoplasms	rs35087646	0.034	0.040	1.035	3.952E-01	logistic	421760	4573	417187	0.998	0.072
459	Other disorders of circulatory system	circulatory system	rs35087646	-0.016	0.019	0.984	3.954E-01	logistic	395720	23148	372572	0.998	0.072
290	Delirium dementia and amnestic and other cognitive disorders	mental disorders	rs35087646	0.028	0.033	1.028	3.964E-01	logistic	412158	7185	404973	0.998	0.072
603.1	Hydrocele	genitourinary	rs35087646	-0.115	0.136	0.891	3.969E-01	logistic	162225	447	161778	1.000	0.071
477	Epistaxis or throat hemorrhage	respiratory	rs35087646	-0.066	0.079	0.936	3.977E-01	logistic	397807	1290	396517	0.993	0.072
379.2	Disorders of vitreous body	sense organs	rs35087646	0.109	0.130	1.116	4.004E-01	logistic	412333	405	411928	0.997	0.072
251.1	Hypoglycemia	endocrine/metabolic	rs35087646	0.058	0.069	1.059	4.013E-01	logistic	383028	1531	381497	0.989	0.072
335	Multiple sclerosis	neurological	rs35087646	0.059	0.070	1.060	4.021E-01	logistic	400514	1467	399047	0.997	0.072
41.2	Streptococcus infection	infectious diseases	rs35087646	0.058	0.069	1.059	4.040E-01	logistic	398155	1508	396647	0.998	0.072
871	Open wounds of extremities	injuries & poisonings	rs35087646	0.113	0.136	1.120	4.043E-01	logistic	420520	370	420150	0.996	0.072
530.12	Ulcer of esophagus	digestive	rs35087646	-0.046	0.055	0.955	4.058E-01	logistic	359422	2634	356788	0.985	0.072
318	Tobacco use disorder	mental disorders	rs35087646	0.032	0.038	1.032	4.065E-01	logistic	408298	5005	403293	0.999	0.072
599.2	Retention of urine	genitourinary	rs35087646	0.028	0.033	1.028	4.077E-01	logistic	396988	6882	390106	0.999	0.072
172	Skin cancer	neoplasms	rs35087646	-0.030	0.036	0.971	4.087E-01	logistic	413263	6055	407208	0.999	0.072
635	Hemorrhage during pregnancy; childbirth and postpartum	pregnancy complications	rs35087646	0.108	0.131	1.114	4.104E-01	logistic	223005	413	222592	0.574	0.072
401	Hypertension	circulatory system	rs35087646	-0.009	0.011	0.991	4.114E-01	logistic	390097	99434	290663	0.998	0.072
285.2	Anemia of chronic disease	hematopoietic	rs35087646	0.078	0.095	1.081	4.121E-01	logistic	383237	788	382449	0.999	0.072
367.9	Blindness and low vision	sense organs	rs35087646	-0.078	0.096	0.925	4.140E-01	logistic	411881	875	411006	0.997	0.072
574.11	Cholelithiasis with acute cholecystitis	digestive	rs35087646	0.068	0.084	1.071	4.152E-01	logistic	396059	1012	395047	0.999	0.072
604	Disorders of penis	genitourinary	rs35087646	-0.106	0.130	0.900	4.155E-01	logistic	162266	488	161778	1.000	0.071
371.3	Inflammation of eyelids	sense organs	rs35087646	-0.090	0.110	0.914	4.155E-01	logistic	411465	672	410793	0.998	0.072
394.7	Disease of tricuspid valve	circulatory system	rs35087646	0.084	0.103	1.087	4.167E-01	logistic	402072	658	401414	0.998	0.072
295.1	Schizophrenia	mental disorders	rs35087646	0.083	0.102	1.086	4.169E-01	logistic	382292	670	381622	0.995	0.072
427.2	Atrial fibrillation and flutter	circulatory system	rs35087646	0.018	0.022	1.018	4.180E-01	logistic	388316	16468	371848	0.997	0.072
743	Osteoporosis, osteopenia and pathological fracture	musculoskeletal	rs35087646	0.064	0.079	1.066	4.185E-01	logistic	422387	1153	421234	0.997	0.072
323	Encephalitis	neurological	rs35087646	0.113	0.140	1.120	4.193E-01	logistic	422420	349	422071	0.998	0.072
785	Abdominal pain	symptoms	rs35087646	-0.027	0.033	0.974	4.198E-01	logistic	401183	7225	393958	0.997	0.072
509.8	Dependence on respirator [Ventilator] or supplemental oxygen	respiratory	rs35087646	-0.086	0.107	0.917	4.209E-01	logistic	391755	698	391057	0.999	0.072
819	Skull and face fracture and other intercranial injury	injuries & poisonings	rs35087646	0.094	0.117	1.099	4.227E-01	logistic	422666	501	422165	0.998	0.072
180.1	Cervical cancer	neoplasms	rs35087646	0.048	0.060	1.049	4.230E-01	logistic	197916	2012	195904	0.132	0.072
289.3	Personal history of diseases of blood and blood-forming organs	hematopoietic	rs35087646	-0.135	0.170	0.873	4.244E-01	logistic	408604	295	408309	0.998	0.072
395.2	Nonrheumatic aortic valve disorders	circulatory system	rs35087646	-0.032	0.040	0.969	4.266E-01	logistic	406373	4959	401414	0.998	0.072
451.2	Phlebitis and thrombophlebitis of lower extremities	circulatory system	rs35087646	-0.103	0.130	0.902	4.285E-01	logistic	381453	487	380966	0.990	0.072
626.1	Irregular menstrual cycle/bleeding	genitourinary	rs35087646	0.034	0.043	1.034	4.287E-01	logistic	196900	4153	192747	0.240	0.072
289	Other diseases of blood and blood-forming organs	hematopoietic	rs35087646	-0.079	0.100	0.924	4.289E-01	logistic	409120	811	408309	0.997	0.072
282	Hereditary hemolytic anemias	hematopoietic	rs35087646	-0.147	0.188	0.863	4.326E-01	logistic	382667	218	382449	0.999	0.072
198.6	Secondary malignancy of bone	neoplasms	rs35087646	0.031	0.040	1.032	4.331E-01	logistic	390762	4756	386006	0.997	0.072
701.3	Circumscribed scleroderma	dermatologic	rs35087646	0.135	0.172	1.144	4.340E-01	logistic	415410	224	415186	0.997	0.072
721.1	Spondylosis without myelopathy	musculoskeletal	rs35087646	-0.057	0.073	0.945	4.344E-01	logistic	410174	1504	408670	0.996	0.072
367.8	Hypermetropia	sense organs	rs35087646	-0.136	0.175	0.873	4.357E-01	logistic	411285	279	411006	0.997	0.072
574.2	Calculus of bile duct	digestive	rs35087646	-0.146	0.188	0.864	4.381E-01	logistic	395287	240	395047	0.999	0.072
597.1	Urethral stricture (not specified as infectious)	genitourinary	rs35087646	0.049	0.063	1.050	4.385E-01	logistic	403370	1816	401554	0.997	0.072
38	Septicemia	infectious diseases	rs35087646	0.025	0.032	1.025	4.387E-01	logistic	403876	7229	396647	0.999	0.072
687	Symptoms affecting skin	dermatologic	rs35087646	-0.116	0.151	0.890	4.406E-01	logistic	413083	367	412716	0.998	0.072
41.4	E. coli	infectious diseases	rs35087646	0.036	0.047	1.037	4.406E-01	logistic	399991	3344	396647	0.997	0.072
573.5	Jaundice (not of newborn)	digestive	rs35087646	0.077	0.101	1.080	4.466E-01	logistic	400970	696	400274	0.998	0.072
288.1	Decreased white blood cell count	hematopoietic	rs35087646	-0.037	0.049	0.964	4.534E-01	logistic	411502	3193	408309	0.998	0.072
288.11	Neutropenia	hematopoietic	rs35087646	-0.037	0.049	0.964	4.534E-01	logistic	411502	3193	408309	0.998	0.072
737.3	Kyphoscoliosis and scoliosis	musculoskeletal	rs35087646	0.071	0.095	1.074	4.544E-01	logistic	403443	789	402654	0.997	0.072
272.11	Hypercholesterolemia	endocrine/metabolic	rs35087646	-0.012	0.016	0.988	4.550E-01	logistic	390444	34679	355765	0.999	0.071
227.3	Benign neoplasm of pituitary gland and craniopharyngeal duct (pouch	neoplasms	rs35087646	-0.113	0.151	0.893	4.553E-01	logistic	421248	361	420887	0.997	0.072
272.9	Unspecified disorder of lipoid metabolism	endocrine/metabolic	rs35087646	-0.120	0.161	0.887	4.575E-01	logistic	356085	320	355765	0.996	0.072
569.2	Gastrointestinal complications	digestive	rs35087646	0.115	0.155	1.122	4.588E-01	logistic	384117	278	383839	0.997	0.072
200.1	Polycythemia vera	neoplasms	rs35087646	-0.092	0.124	0.912	4.604E-01	logistic	408705	523	408182	0.996	0.072
367.2	Astigmatism	sense organs	rs35087646	0.034	0.047	1.035	4.632E-01	logistic	414418	3412	411006	0.997	0.072

ST31; PheWAS results for the lead cis-pQTLs of the 4 proteins prioritized (ACOX1, FGF5, FURIN, and MST1).

SNP = Single Nucleotide Polymorphism; SE = Standard error HWE_p = P-value for Hardy-Weinberg equilibrium test

Phenotype	Description	Group	SNP	Beta	SE	Odds tatio	P-value	Type	n_total	n_cases	n_controls	HWE_p	allele_freq
590	Pyelonephritis	genitourinary	rs35087646	-0.055	0.076	0.946	4.650E-01	logistic	378294	1367	376927	0.999	0.072
189.2	Cancer of bladder	neoplasms	rs35087646	0.034	0.047	1.035	4.653E-01	logistic	420532	3345	417187	0.998	0.072
635.2	Antepartum hemorrhage, abruptio placentae, and placenta previa	pregnancy complications	rs35087646	0.133	0.183	1.142	4.660E-01	logistic	222797	205	222592	0.596	0.072
456	Chronic venous insufficiency [CVI]	circulatory system	rs35087646	-0.125	0.172	0.882	4.671E-01	logistic	381250	284	380966	0.991	0.072
361	Retinal detachments and defects	sense organs	rs35087646	-0.091	0.126	0.913	4.672E-01	logistic	398276	513	397763	0.997	0.072
603	Other disorders of testis	genitourinary	rs35087646	-0.095	0.131	0.909	4.676E-01	logistic	162246	468	161778	1.000	0.071
627	Menopausal and postmenopausal disorders	genitourinary	rs35087646	0.038	0.052	1.039	4.678E-01	logistic	195450	2703	192747	0.269	0.072
459.9	Circulatory disease NEC	circulatory system	rs35087646	-0.014	0.019	0.986	4.687E-01	logistic	395548	22976	372572	0.998	0.072
41	Bacterial infection NOS	infectious diseases	rs35087646	0.021	0.028	1.021	4.690E-01	logistic	406056	9409	396647	0.998	0.072
580.2	Nephrotic syndrome without mention of glomerulonephritis	genitourinary	rs35087646	-0.104	0.144	0.901	4.711E-01	logistic	381457	395	381062	0.999	0.072
561	Symptoms involving digestive system	digestive	rs35087646	0.028	0.038	1.028	4.729E-01	logistic	339539	5054	334485	0.986	0.072
367.1	Myopia	sense organs	rs35087646	-0.055	0.077	0.946	4.737E-01	logistic	412331	1325	411006	0.997	0.072
250.2	Type 2 diabetes	endocrine/metabolic	rs35087646	0.012	0.017	1.013	4.751E-01	logistic	413206	26569	386637	0.994	0.072
153	Colorectal cancer	neoplasms	rs35087646	0.023	0.033	1.024	4.766E-01	logistic	377768	7048	370720	0.994	0.072
334	Degenerative disease of the spinal cord	neurological	rs35087646	0.046	0.065	1.047	4.767E-01	logistic	400789	1742	399047	0.997	0.072
536.8	Dyspepsia and other specified disorders of function of stomach	digestive	rs35087646	-0.049	0.069	0.952	4.775E-01	logistic	363851	1637	362214	0.994	0.072
357	Inflammatory and toxic neuropathy	neurological	rs35087646	-0.045	0.063	0.956	4.775E-01	logistic	421163	1995	419168	0.996	0.072
261.4	Vitamin D deficiency	endocrine/metabolic	rs35087646	-0.042	0.059	0.959	4.778E-01	logistic	414357	2260	412097	0.998	0.072
274.2	Crystal arthropathies	endocrine/metabolic	rs35087646	0.118	0.167	1.125	4.789E-01	logistic	416033	243	415790	0.998	0.072
337	Disorders of the autonomic nervous system	neurological	rs35087646	-0.109	0.154	0.897	4.791E-01	logistic	399396	349	399047	0.997	0.072
475	Chronic sinusitis	respiratory	rs35087646	0.061	0.087	1.063	4.796E-01	logistic	397468	951	396517	0.993	0.072
333.8	Other degenerative diseases of the basal ganglia	neurological	rs35087646	-0.137	0.195	0.872	4.826E-01	logistic	399271	224	399047	0.997	0.072
427.3	Other specified cardiac dysrhythmias	circulatory system	rs35087646	0.037	0.053	1.038	4.838E-01	logistic	374456	2608	371848	0.998	0.072
577.2	Chronic pancreatitis	digestive	rs35087646	0.075	0.108	1.078	4.843E-01	logistic	418611	603	418008	0.997	0.072
709.3	Systemic sclerosis	dermatologic	rs35087646	-0.137	0.196	0.872	4.849E-01	logistic	404118	219	403899	0.997	0.072
276.14	Hypopotassemia	endocrine/metabolic	rs35087646	-0.033	0.047	0.968	4.852E-01	logistic	399074	3502	395572	0.999	0.072
270	Disorders of protein plasma/amino-acid transport and metabolism	endocrine/metabolic	rs35087646	-0.070	0.101	0.932	4.856E-01	logistic	422490	788	421702	0.997	0.072
743.2	Pathologic fracture	musculoskeletal	rs35087646	0.061	0.088	1.063	4.879E-01	logistic	422164	930	421234	0.997	0.072
350	Abnormal movement	neurological	rs35087646	0.054	0.077	1.055	4.882E-01	logistic	421410	1200	420210	0.998	0.072
990	Effects radiation NOS	injuries & poisonings	rs35087646	-0.023	0.034	0.977	4.892E-01	logistic	408877	6919	401958	0.998	0.072
204.2	Myeloid leukemia	neoplasms	rs35087646	-0.071	0.103	0.931	4.897E-01	logistic	416360	747	415613	0.996	0.072
191.1	Cancer of brain and nervous system	neoplasms	rs35087646	-0.067	0.098	0.935	4.909E-01	logistic	421229	832	420397	0.997	0.072
550.4	Umbilical hernia	digestive	rs35087646	0.049	0.071	1.050	4.929E-01	logistic	366811	1417	365394	0.998	0.072
735.3	Hallux valgus (Bunion)	musculoskeletal	rs35087646	-0.039	0.056	0.962	4.933E-01	logistic	405116	2462	402654	0.998	0.072
577	Diseases of pancreas	digestive	rs35087646	0.032	0.048	1.033	4.954E-01	logistic	421247	3239	418008	0.998	0.072
735.2	Acquired toe deformities	musculoskeletal	rs35087646	-0.044	0.064	0.957	4.959E-01	logistic	404574	1920	402654	0.998	0.072
366.2	Senile cataract	sense organs	rs35087646	0.020	0.029	1.020	5.012E-01	logistic	380814	9136	371678	0.997	0.072
252	Disorders of parathyroid gland	endocrine/metabolic	rs35087646	0.051	0.076	1.052	5.025E-01	logistic	416485	1239	415246	0.997	0.072
504	Other alveolar and parietoalveolar pneumonopathy	respiratory	rs35087646	-0.074	0.110	0.929	5.027E-01	logistic	391718	661	391057	0.999	0.072
592	Cystitis and urethritis	genitourinary	rs35087646	-0.053	0.079	0.948	5.027E-01	logistic	378176	1249	376927	0.999	0.072
145.2	Cancer of tongue	neoplasms	rs35087646	-0.096	0.145	0.909	5.079E-01	logistic	420733	391	420342	0.996	0.072
426.2	Atrioventricular [AV] block	circulatory system	rs35087646	0.030	0.045	1.030	5.095E-01	logistic	375580	3732	371848	0.996	0.072
585.33	Chronic Kidney Disease, Stage III	genitourinary	rs35087646	0.021	0.032	1.021	5.096E-01	logistic	388537	7475	381062	0.999	0.072
340	Migraine	neurological	rs35087646	-0.036	0.055	0.964	5.108E-01	logistic	407732	2545	405187	0.999	0.072
378.5	Paralytic strabismus	sense organs	rs35087646	-0.115	0.175	0.892	5.111E-01	logistic	412201	273	411928	0.997	0.072
967	Adverse effects of sedatives or other central nervous system depressants and anesthetics	injuries & poisonings	rs35087646	-0.127	0.193	0.881	5.113E-01	logistic	367398	225	367173	0.998	0.072
411	Ischemic Heart Disease	circulatory system	rs35087646	-0.010	0.015	0.990	5.116E-01	logistic	410606	40056	370550	0.998	0.072
255.2	Adrenal hypofunction	endocrine/metabolic	rs35087646	0.093	0.141	1.097	5.119E-01	logistic	415595	349	415246	0.998	0.072
255.21	Glucocorticoid deficiency	endocrine/metabolic	rs35087646	0.093	0.141	1.097	5.119E-01	logistic	415595	349	415246	0.998	0.072
601.12	Chronic prostatitis	genitourinary	rs35087646	0.112	0.171	1.118	5.125E-01	logistic	156694	233	156461	1.000	0.072
801.1	Fracture of foot	injuries & poisonings	rs35087646	0.115	0.176	1.122	5.130E-01	logistic	396597	219	396378	0.993	0.072
742	Derangement of joint, non-traumatic	musculoskeletal	rs35087646	-0.089	0.137	0.915	5.147E-01	logistic	415444	434	415010	0.998	0.072
339	Other headache syndromes	neurological	rs35087646	0.027	0.042	1.028	5.150E-01	logistic	409356	4169	405187	0.996	0.072
592.1	Cystitis	genitourinary	rs35087646	-0.052	0.081	0.949	5.166E-01	logistic	378131	1204	376927	0.999	0.072
386.1	Meniere's disease	sense organs	rs35087646	0.068	0.106	1.071	5.169E-01	logistic	410308	643	409665	0.994	0.072
378	Strabismus and other disorders of binocular eye movements	sense organs	rs35087646	-0.059	0.091	0.943	5.177E-01	logistic	412884	956	411928	0.996	0.072
189.11	Malignant neoplasm of kidney, except pelvis	neoplasms	rs35087646	0.051	0.080	1.053	5.181E-01	logistic	418324	1137	417187	0.998	0.072
420.2	Pericarditis	circulatory system	rs35087646	-0.047	0.073	0.954	5.190E-01	logistic	418236	1466	416770	0.996	0.072
800	Fracture of lower limb	injuries & poisonings	rs35087646	0.032	0.049	1.032	5.198E-01	logistic	399482	3104	396378	0.992	0.072
426	Cardiac conduction disorders	circulatory system	rs35087646	0.017	0.027	1.018	5.205E-01	logistic	382540	10692	371848	0.997	0.072
614	Inflammatory diseases of female pelvic organs	genitourinary	rs35087646	-0.046	0.071	0.955	5.215E-01	logistic	218979	1541	217438	0.362	0.072
458	Hypotension	circulatory system	rs35087646	-0.020	0.031	0.980	5.216E-01	logistic	380778	8206	372572	0.997	0.072
296	Mood disorders	mental disorders	rs35087646	0.011	0.017	1.011	5.217E-01	logistic	409186	27564	381622	0.996	0.072
580.32	Nephritis and nephropathy with pathological lesion	genitourinary	rs35087646	0.102	0.159	1.107	5.218E-01	logistic	381336	274	381062	0.999	0.072
727.6	Rupture of tendon, nontraumatic	musculoskeletal	rs35087646	-0.049	0.076	0.952	5.234E-01	logistic	390517	1343	389174	0.995	0.072
480.1	Bacterial pneumonia	respiratory	rs35087646	0.017	0.027	1.018	5.243E-01	logistic	407095	10295	396800	0.998	0.072
580.14	Chronic glomerulonephritis, NOS	genitourinary	rs35087646	-0.102	0.161	0.903	5.264E-01	logistic	381376	314	381062	0.999	0.072
752.11	Spina bifida	congenital anomalies	rs35087646	-0.128	0.203	0.880	5.264E-01	logistic	422743	204	422539	0.997	0.072
690	Erythematosquamous dermatosis	dermatologic	rs35087646	-0.072	0.114	0.931	5.275E-01	logistic	405831	625	405206	0.997	0.072
204.1	Lymphoid leukemia	neoplasms	rs35087646	-0.059	0.094	0.943	5.296E-01	logistic	416514	901	415613	0.997	0.072
577.1	Acute pancreatitis	digestive	rs35087646	-0.041	0.065	0.960	5.312E-01	logistic	419858	1850	418008	0.998	0.072

ST31; PheWAS results for the lead cis-pQTLs of the 4 proteins prioritized (ACOX1, FGF5, FURIN, and MST1).

SNP = Single Nucleotide Polymorphism; SE = Standard error HWE_p = P-value for Hardy-Weinberg equilibrium test

Phenotype	Description	Group	SNP	Beta	SE	Odds tatio	P-value	Type	n_total	n_cases	n_controls	HWE_p	allele_freq
591	Urinary tract infection	genitourinary	rs35087646	-0.016	0.026	0.984	5.315E-01	logistic	388723	11796	376927	0.997	0.072
426.9	Cardiac pacemaker/device in situ	circulatory system	rs35087646	0.024	0.039	1.024	5.322E-01	logistic	376950	5102	371848	0.998	0.072
451	Phlebitis and thrombophlebitis	circulatory system	rs35087646	-0.066	0.106	0.936	5.325E-01	logistic	381675	709	380966	0.989	0.072
411.4	Coronary atherosclerosis	circulatory system	rs35087646	-0.012	0.019	0.988	5.346E-01	logistic	394337	23787	370550	0.999	0.072
597	Other disorders of urethra and urinary tract	genitourinary	rs35087646	0.037	0.060	1.038	5.352E-01	logistic	403600	2046	401554	0.997	0.072
555.1	Regional enteritis	digestive	rs35087646	0.039	0.063	1.040	5.369E-01	logistic	336302	1817	334485	0.987	0.072
574	Cholelithiasis and cholecystitis	digestive	rs35087646	-0.016	0.027	0.984	5.419E-01	logistic	406113	11066	395047	0.999	0.072
227	Benign neoplasm of other endocrine glands and related structures	neoplasms	rs35087646	0.061	0.100	1.063	5.421E-01	logistic	421596	709	420887	0.998	0.072
270.32	Paraproteinemia	endocrine/metabolic	rs35087646	-0.069	0.114	0.933	5.422E-01	logistic	422319	617	421702	0.997	0.072
276.6	Fluid overload	endocrine/metabolic	rs35087646	-0.042	0.069	0.959	5.436E-01	logistic	397239	1667	395572	0.999	0.072
427.21	Atrial fibrillation	circulatory system	rs35087646	-0.026	0.043	0.974	5.436E-01	logistic	376121	4273	371848	0.998	0.071
250.1	Type 1 diabetes	endocrine/metabolic	rs35087646	-0.034	0.057	0.966	5.444E-01	logistic	389041	2404	386637	0.993	0.072
696.41	Psoriasis vulgaris	dermatologic	rs35087646	-0.037	0.061	0.964	5.447E-01	logistic	401395	2105	399290	0.997	0.072
573	Other disorders of liver	digestive	rs35087646	-0.038	0.062	0.963	5.448E-01	logistic	402294	2020	400274	0.999	0.072
790	Nonspecific findings on examination of blood	symptoms	rs35087646	0.099	0.164	1.104	5.460E-01	logistic	422095	255	421840	0.998	0.072
565	Anal and rectal conditions	digestive	rs35087646	-0.019	0.032	0.981	5.476E-01	logistic	391353	7514	383839	0.998	0.072
444	Arterial embolism and thrombosis	circulatory system	rs35087646	0.059	0.098	1.061	5.477E-01	logistic	406215	746	405469	0.999	0.072
385	Other disorders of middle ear and mastoid	sense organs	rs35087646	-0.098	0.163	0.906	5.480E-01	logistic	419023	307	418716	0.996	0.072
568.1	Peritoneal adhesions (postoperative) (postinfection)	digestive	rs35087646	-0.046	0.078	0.955	5.513E-01	logistic	385136	1297	383839	0.997	0.072
191.11	Cancer of brain	neoplasms	rs35087646	-0.059	0.100	0.943	5.549E-01	logistic	421187	790	420397	0.997	0.072
681	Superficial cellulitis and abscess	dermatologic	rs35087646	0.090	0.152	1.094	5.553E-01	logistic	418799	302	418497	0.996	0.072
280.2	Iron deficiency anemia secondary to blood loss (chronic)	hematopoietic	rs35087646	-0.097	0.165	0.908	5.581E-01	logistic	382749	300	382449	0.999	0.072
443.9	Peripheral vascular disease, unspecified	circulatory system	rs35087646	-0.027	0.047	0.973	5.592E-01	logistic	409046	3577	405469	0.999	0.072
766	Neuralgia, neuritis, and radiculitis NOS	symptoms	rs35087646	0.092	0.157	1.096	5.597E-01	logistic	418267	281	417986	0.998	0.072
572	Ascites (non malignant)	digestive	rs35087646	-0.035	0.060	0.966	5.626E-01	logistic	402409	2135	400274	0.998	0.072
204.21	Myeloid leukemia, acute	neoplasms	rs35087646	-0.068	0.117	0.935	5.634E-01	logistic	416195	582	415613	0.996	0.072
378.2	Nystagmus and other irregular eye movements	sense organs	rs35087646	-0.115	0.199	0.892	5.640E-01	logistic	412137	209	411928	0.997	0.072
53	Herpes zoster	infectious diseases	rs35087646	-0.087	0.151	0.917	5.659E-01	logistic	415563	359	415204	0.997	0.072
802	Fracture of pelvis	injuries & poisonings	rs35087646	0.050	0.087	1.051	5.660E-01	logistic	397331	953	396378	0.991	0.072
374.1	Ectropion or entropion	sense organs	rs35087646	0.058	0.101	1.059	5.691E-01	logistic	411493	700	410793	0.999	0.072
707.1	Decubitus ulcer	dermatologic	rs35087646	0.028	0.050	1.029	5.719E-01	logistic	419126	2996	416130	0.997	0.072
317.1	Alcoholism	mental disorders	rs35087646	-0.025	0.045	0.975	5.734E-01	logistic	407082	3789	403293	0.999	0.072
740.2	Osteoarthrosis, generalized	musculoskeletal	rs35087646	-0.023	0.041	0.977	5.741E-01	logistic	350458	4600	345858	1.000	0.072
610	Benign mammary dysplasias	genitourinary	rs35087646	0.067	0.119	1.069	5.750E-01	logistic	220777	505	220272	0.388	0.072
622.2	Mucous polyp of cervix	genitourinary	rs35087646	-0.104	0.186	0.901	5.754E-01	logistic	213810	238	213572	0.415	0.072
198.7	Secondary malignant neoplasm of skin	neoplasms	rs35087646	-0.099	0.177	0.906	5.754E-01	logistic	386268	262	386006	0.997	0.072
686	Other local infections of skin and subcutaneous tissue	dermatologic	rs35087646	-0.048	0.086	0.953	5.757E-01	logistic	419554	1057	418497	0.996	0.072
519	Other diseases of respiratory system, not elsewhere classified	respiratory	rs35087646	-0.017	0.030	0.984	5.776E-01	logistic	411962	8832	403130	0.999	0.072
331.9	Cerebral degeneration, unspecified	neurological	rs35087646	0.048	0.087	1.050	5.780E-01	logistic	400009	962	399047	0.997	0.072
333.4	Torsion dystonia	neurological	rs35087646	-0.091	0.164	0.913	5.794E-01	logistic	399349	302	399047	0.997	0.072
296.2	Depression	mental disorders	rs35087646	0.010	0.017	1.010	5.808E-01	logistic	408593	26971	381622	0.995	0.072
159	Malignant neoplasm of other and ill-defined sites within the digestive organs and peritoneum	neoplasms	rs35087646	0.050	0.091	1.052	5.810E-01	logistic	394736	874	393862	0.995	0.072
689	Disorder of skin and subcutaneous tissue NOS	dermatologic	rs35087646	-0.034	0.061	0.967	5.821E-01	logistic	413188	2098	411090	0.996	0.072
751.2	Congenital anomalies of urinary system	congenital anomalies	rs35087646	0.054	0.098	1.055	5.824E-01	logistic	421003	750	420253	0.998	0.072
569.1	Toxic gastroenteritis and colitis	digestive	rs35087646	-0.088	0.160	0.916	5.831E-01	logistic	384157	318	383839	0.997	0.072
558	Noninfectious gastroenteritis	digestive	rs35087646	0.022	0.041	1.023	5.841E-01	logistic	338928	4443	334485	0.987	0.072
690.1	Seborrheic dermatitis	dermatologic	rs35087646	-0.062	0.114	0.940	5.854E-01	logistic	405818	612	405206	0.997	0.072
636.3	Hemorrhage in early pregnancy	pregnancy complications	rs35087646	-0.107	0.196	0.899	5.861E-01	logistic	222810	218	222592	0.604	0.072
567	Peritonitis and retroperitoneal infections	digestive	rs35087646	0.054	0.099	1.056	5.869E-01	logistic	384568	729	383839	0.998	0.072
81	Infection/inflammation of internal prosthetic device; implant; and graft	infectious diseases	rs35087646	0.033	0.062	1.034	5.878E-01	logistic	414783	1939	412844	0.998	0.072
743.21	Pathologic fracture of vertebrae	musculoskeletal	rs35087646	0.058	0.107	1.060	5.881E-01	logistic	421861	627	421234	0.997	0.072
296.22	Major depressive disorder	mental disorders	rs35087646	0.012	0.023	1.012	5.919E-01	logistic	396820	15198	381622	0.997	0.072
516	Abnormal sputum	respiratory	rs35087646	-0.048	0.089	0.953	5.924E-01	logistic	421370	981	420389	0.998	0.072
428.1	Congestive heart failure (CHF) NOS	circulatory system	rs35087646	-0.024	0.045	0.976	5.930E-01	logistic	413649	3946	409703	0.997	0.072
512.7	Shortness of breath	respiratory	rs35087646	-0.024	0.044	0.977	5.936E-01	logistic	408527	3928	404599	0.995	0.072
70.4	Chronic hepatitis	infectious diseases	rs35087646	0.083	0.157	1.087	5.949E-01	logistic	415491	287	415204	0.996	0.072
528	Diseases of the oral soft tissues, excluding lesions specific for gingiva and tongue	digestive	rs35087646	0.066	0.124	1.068	5.949E-01	logistic	418169	459	417710	0.998	0.072
292.4	Altered mental status	mental disorders	rs35087646	-0.025	0.047	0.975	5.982E-01	logistic	408434	3461	404973	0.999	0.072
270.3	Disorders of plasma protein metabolism	endocrine/metabolic	rs35087646	-0.054	0.103	0.947	5.982E-01	logistic	422449	747	421702	0.997	0.072
196	Radiotherapy	neoplasms	rs35087646	-0.037	0.070	0.964	5.987E-01	logistic	387589	1583	386006	0.998	0.072
352.1	Trigeminal nerve disorders [CN5]	neurological	rs35087646	-0.066	0.126	0.936	6.002E-01	logistic	402912	497	402415	0.998	0.072
580.1	Glomerulonephritis	genitourinary	rs35087646	-0.065	0.125	0.937	6.016E-01	logistic	381574	512	381062	0.999	0.072
720.1	Spinal stenosis of lumbar region	musculoskeletal	rs35087646	-0.028	0.055	0.972	6.039E-01	logistic	411268	2598	408670	0.998	0.072
444.1	Arterial embolism and thrombosis of lower extremity artery	circulatory system	rs35087646	0.065	0.126	1.068	6.040E-01	logistic	405915	446	405469	0.999	0.072
480.11	Pneumococcal pneumonia	respiratory	rs35087646	0.015	0.028	1.015	6.061E-01	logistic	406463	9663	396800	0.998	0.072
189.4	Malignant neoplasm of other urinary organs	neoplasms	rs35087646	0.095	0.184	1.099	6.062E-01	logistic	417393	206	417187	0.997	0.072
960.2	Allergy/adverse effect of penicillin	injuries & poisonings	rs35087646	-0.011	0.021	0.989	6.063E-01	logistic	385833	18660	367173	0.995	0.072
371.1	Uveitis, noninfectious or NOS	sense organs	rs35087646	0.085	0.166	1.089	6.077E-01	logistic	411047	254	410793	0.999	0.072
587	Kidney replaced by transpant	genitourinary	rs35087646	0.061	0.120	1.063	6.085E-01	logistic	381556	494	381062	0.999	0.072
536	Disorders of function of stomach	digestive	rs35087646	-0.035	0.069	0.966	6.099E-01	logistic	363858	1644	362214	0.994	0.072
395.6	Heart valve replaced	circulatory system	rs35087646	-0.028	0.056	0.972	6.117E-01	logistic	403924	2510	401414	0.999	0.072

ST31; PheWAS results for the lead cis-pQTLs of the 4 proteins prioritized (ACOX1, FGF5, FURIN, and MST1).

SNP = Single Nucleotide Polymorphism; SE = Standard error HWE_p = P-value for Hardy-Weinberg equilibrium test

Phenotype	Description	Group	SNP	Beta	SE	Odds tatio	P-value	Type	n_total	n_cases	n_controls	HWE_p	allele_freq
965	Poisoning by analgesics, antipyretics, and antirheumatics	injuries & poisonings	rs35087646	-0.021	0.040	0.980	6.119E-01	logistic	371918	4745	367173	0.997	0.072
295	Schizophrenia and other psychotic disorders	mental disorders	rs35087646	0.047	0.092	1.048	6.121E-01	logistic	382460	838	381622	0.996	0.072
740.11	Osteoarthrosis, localized, primary	musculoskeletal	rs35087646	-0.019	0.039	0.981	6.134E-01	logistic	351108	5250	345858	0.999	0.072
800.3	Fracture of tibia and fibula	injuries & poisonings	rs35087646	0.056	0.112	1.058	6.153E-01	logistic	396955	577	396378	0.991	0.072
172.2	Other non-epithelial cancer of skin	neoplasms	rs35087646	-0.023	0.047	0.977	6.154E-01	logistic	410817	3609	407208	0.998	0.072
276.4	Acid-base balance disorder	endocrine/metabolic	rs35087646	0.029	0.059	1.030	6.180E-01	logistic	397692	2120	395572	0.999	0.072
559	Ileostomy status	digestive	rs35087646	-0.033	0.066	0.968	6.192E-01	logistic	336295	1810	334485	0.988	0.072
728	Disorders of muscle, ligament, and fascia	musculoskeletal	rs35087646	0.082	0.165	1.085	6.206E-01	logistic	389432	258	389174	0.995	0.072
364.5	Corneal dystrophy	sense organs	rs35087646	-0.070	0.143	0.932	6.241E-01	logistic	398152	389	397763	0.997	0.072
585	Renal failure	genitourinary	rs35087646	0.009	0.019	1.009	6.248E-01	logistic	403986	22924	381062	0.999	0.072
386	Vertiginous syndromes and other disorders of vestibular system	sense organs	rs35087646	-0.033	0.068	0.967	6.248E-01	logistic	411346	1681	409665	0.995	0.072
857	Mechanical complication of unspecified genitourinary device, implant, and graft	injuries & poisonings	rs35087646	-0.034	0.070	0.967	6.262E-01	logistic	418243	1591	416652	0.997	0.072
450	Noninfectious disorders of lymphatic channels	circulatory system	rs35087646	-0.040	0.082	0.961	6.271E-01	logistic	422720	1167	421553	0.998	0.072
389	Hearing loss	sense organs	rs35087646	-0.016	0.033	0.984	6.290E-01	logistic	415421	7124	408297	0.996	0.072
835	Internal derangement of knee	injuries & poisonings	rs35087646	-0.019	0.040	0.981	6.338E-01	logistic	409924	4709	405215	0.994	0.072
317	Alcohol-related disorders	mental disorders	rs35087646	0.018	0.039	1.019	6.343E-01	logistic	408346	5053	403293	0.999	0.072
626.12	Excessive or frequent menstruation	genitourinary	rs35087646	0.028	0.059	1.028	6.357E-01	logistic	194922	2175	192747	0.292	0.072
585.3	Chronic renal failure [CKD]	genitourinary	rs35087646	0.012	0.025	1.012	6.359E-01	logistic	393739	12677	381062	0.999	0.072
252.1	Hyperparathyroidism	endocrine/metabolic	rs35087646	0.040	0.084	1.041	6.362E-01	logistic	416271	1025	415246	0.997	0.072
165.1	Cancer of bronchus; lung	neoplasms	rs35087646	-0.020	0.043	0.980	6.370E-01	logistic	421452	4223	417229	0.996	0.072
741.3	Difficulty in walking	musculoskeletal	rs35087646	-0.061	0.130	0.941	6.373E-01	logistic	415474	464	415010	0.997	0.072
994	Sepsis and SIRS	injuries & poisonings	rs35087646	0.016	0.034	1.016	6.394E-01	logistic	418535	6755	411780	0.998	0.072
994.2	Sepsis	injuries & poisonings	rs35087646	0.016	0.034	1.016	6.394E-01	logistic	418535	6755	411780	0.998	0.072
276.41	Acidosis	endocrine/metabolic	rs35087646	0.030	0.064	1.030	6.413E-01	logistic	397376	1804	395572	0.999	0.072
560.1	Paralytic ileus	digestive	rs35087646	-0.066	0.142	0.936	6.438E-01	logistic	334880	395	334485	0.986	0.072
573.7	Abnormal results of function study of liver	digestive	rs35087646	0.020	0.044	1.021	6.441E-01	logistic	404090	3816	400274	0.998	0.072
698	Pruritus and related conditions	dermatologic	rs35087646	-0.078	0.170	0.925	6.465E-01	logistic	422717	278	422439	0.996	0.072
199	Neoplasm of uncertain behavior	neoplasms	rs35087646	0.050	0.108	1.051	6.465E-01	logistic	386620	614	386006	0.996	0.072
516.1	Hemoptysis	respiratory	rs35087646	-0.042	0.092	0.959	6.467E-01	logistic	421320	931	420389	0.998	0.072
556	Ulceration of the lower GI tract	digestive	rs35087646	-0.065	0.142	0.937	6.470E-01	logistic	334879	394	334485	0.987	0.072
288	Diseases of white blood cells	hematopoietic	rs35087646	-0.063	0.138	0.939	6.499E-01	logistic	408723	414	408309	0.998	0.072
187	Cancer of other male genital organs	neoplasms	rs35087646	-0.015	0.033	0.985	6.517E-01	logistic	186921	7527	179394	1.000	0.071
345	Epilepsy, recurrent seizures, convulsions	neurological	rs35087646	0.019	0.043	1.019	6.523E-01	logistic	403151	4104	399047	0.997	0.072
571.81	Portal hypertension	digestive	rs35087646	0.041	0.092	1.042	6.532E-01	logistic	401135	861	400274	0.998	0.072
594.2	Calculus of lower urinary tract	genitourinary	rs35087646	-0.052	0.117	0.949	6.543E-01	logistic	412949	573	412376	0.997	0.072
253	Disorders of the pituitary gland and its hypothalamic control	endocrine/metabolic	rs35087646	-0.042	0.094	0.959	6.578E-01	logistic	416136	890	415246	0.997	0.072
386.3	Labyrinthitis	sense organs	rs35087646	-0.066	0.151	0.936	6.590E-01	logistic	410012	347	409665	0.995	0.072
575.7	Other disorders of gallbladder	digestive	rs35087646	-0.050	0.114	0.951	6.593E-01	logistic	395657	610	395047	0.999	0.072
803.1	Fracture of humerus	injuries & poisonings	rs35087646	-0.037	0.084	0.964	6.599E-01	logistic	397468	1090	396378	0.992	0.072
296.1	Bipolar	mental disorders	rs35087646	-0.036	0.082	0.965	6.602E-01	logistic	382786	1164	381622	0.995	0.072
250	Diabetes mellitus	endocrine/metabolic	rs35087646	0.008	0.017	1.008	6.618E-01	logistic	414239	27602	386637	0.994	0.072
575.2	Obstruction of bile duct	digestive	rs35087646	0.037	0.084	1.037	6.625E-01	logistic	396076	1029	395047	0.999	0.072
496.1	Emphysema	respiratory	rs35087646	0.021	0.050	1.022	6.706E-01	logistic	401774	2965	398809	0.998	0.072
8.52	Intestinal infection due to C. difficile	infectious diseases	rs35087646	-0.046	0.108	0.955	6.711E-01	logistic	417984	668	417316	0.997	0.072
614.3	Pelvic inflammatory disease (PID)	genitourinary	rs35087646	-0.078	0.184	0.925	6.714E-01	logistic	217675	237	217438	0.323	0.072
695.9	Unspecified erythematous condition	dermatologic	rs35087646	0.063	0.150	1.065	6.743E-01	logistic	405524	318	405206	0.997	0.072
214	Lipoma	neoplasms	rs35087646	-0.035	0.083	0.966	6.749E-01	logistic	415339	1112	414227	0.994	0.072
946	Anaphylactic shock NOS	injuries & poisonings	rs35087646	0.074	0.178	1.077	6.772E-01	logistic	415210	223	414987	0.997	0.072
381.1	Otitis media	sense organs	rs35087646	-0.046	0.111	0.955	6.774E-01	logistic	419355	639	418716	0.996	0.072
535	Gastritis and duodenitis	digestive	rs35087646	-0.009	0.022	0.991	6.774E-01	logistic	378649	16435	362214	0.993	0.072
250.4	Abnormal glucose	endocrine/metabolic	rs35087646	0.035	0.084	1.036	6.777E-01	logistic	387669	1032	386637	0.993	0.072
696	Psoriasis and related disorders	dermatologic	rs35087646	-0.021	0.051	0.979	6.788E-01	logistic	402206	2916	399290	0.997	0.072
442.11	Abdominal aortic aneurysm	circulatory system	rs35087646	-0.028	0.068	0.972	6.799E-01	logistic	407127	1658	405469	0.999	0.072
455	Hemorrhoids	circulatory system	rs35087646	0.015	0.038	1.015	6.830E-01	logistic	386244	5278	380966	0.992	0.072
735.23	Hallux rigidus	musculoskeletal	rs35087646	-0.064	0.158	0.938	6.833E-01	logistic	402973	319	402654	0.998	0.072
447.1	Stricture of artery	circulatory system	rs35087646	-0.046	0.113	0.955	6.844E-01	logistic	406083	614	405469	0.999	0.072
531	Peptic ulcer (excl. esophageal)	digestive	rs35087646	-0.018	0.044	0.982	6.846E-01	logistic	415329	3990	411339	0.997	0.072
750	Digestive congenital anomalies	congenital anomalies	rs35087646	-0.056	0.137	0.946	6.847E-01	logistic	420674	421	420253	0.998	0.072
519.8	Other diseases of respiratory system, NEC	respiratory	rs35087646	-0.012	0.030	0.988	6.848E-01	logistic	411807	8677	403130	0.999	0.072
191	Manlignant and unknown neoplasms of brain and nervous system	neoplasms	rs35087646	-0.036	0.090	0.965	6.866E-01	logistic	421363	966	420397	0.997	0.072
619.3	Noninflammatory disorders of cervix	genitourinary	rs35087646	0.058	0.145	1.060	6.892E-01	logistic	217308	342	216966	0.200	0.072
427.5	Arrhythmia (cardiac) NOS	circulatory system	rs35087646	0.048	0.121	1.049	6.899E-01	logistic	372348	500	371848	0.997	0.072
528.11	Stomatitis and mucositis (ulcerative)	digestive	rs35087646	0.073	0.184	1.076	6.906E-01	logistic	417919	209	417710	0.998	0.072
367	Disorders of refraction and accommodation; blindness and low vision	sense organs	rs35087646	-0.014	0.036	0.986	6.907E-01	logistic	417170	6164	411006	0.998	0.072
289.5	Diseases of spleen	hematopoietic	rs35087646	-0.040	0.101	0.961	6.932E-01	logistic	409070	761	408309	0.998	0.072
571.6	Primary biliary cirrhosis	digestive	rs35087646	-0.063	0.160	0.939	6.956E-01	logistic	400586	312	400274	0.998	0.072
523.3	Periodontitis (acute or chronic)	digestive	rs35087646	-0.072	0.183	0.931	6.962E-01	logistic	409770	234	409536	0.995	0.072
426.91	Cardiac pacemaker in situ	circulatory system	rs35087646	0.015	0.039	1.015	6.971E-01	logistic	376826	4978	371848	0.998	0.072
613	Other nonmalignant breast conditions	genitourinary	rs35087646	-0.055	0.141	0.947	6.980E-01	logistic	421594	397	421197	0.997	0.072
433.12	Cerebral atherosclerosis	circulatory system	rs35087646	0.055	0.144	1.057	7.002E-01	logistic	403029	344	402685	0.998	0.072
695.2	Bullous dermatoses	dermatologic	rs35087646	0.072	0.187	1.074	7.003E-01	logistic	405408	202	405206	0.997	0.072

ST31; PheWAS results for the lead cis-pQTLs of the 4 proteins prioritized (ACOX1, FGF5, FURIN, and MST1).

SNP = Single Nucleotide Polymorphism; SE = Standard error HWE_p = P-value for Hardy-Weinberg equilibrium test

Phenotype	Description	Group	SNP	Beta	SE	Odds tatio	P-value	Type	n_total	n_cases	n_controls	HWE_p	allele_freq
331.1	Hydrocephalus	neurological	rs35087646	-0.043	0.112	0.958	7.003E-01	logistic	399668	621	399047	0.997	0.072
575.9	Nonspecific abnormal findings on radiological and other examination of biliary trac	digestive	rs35087646	0.068	0.176	1.070	7.008E-01	logistic	395275	228	395047	0.999	0.072
395.3	Nonrheumatic tricuspid valve disorders	circulatory system	rs35087646	-0.070	0.183	0.932	7.011E-01	logistic	401653	239	401414	0.998	0.072
618	Genital prolapse	genitourinary	rs35087646	0.014	0.035	1.014	7.011E-01	logistic	219680	6225	213455	0.466	0.072
411.9	Other acute and subacute forms of ischemic heart disease	circulatory system	rs35087646	0.027	0.070	1.027	7.011E-01	logistic	372042	1492	370550	1.000	0.072
720	Spinal stenosis	musculoskeletal	rs35087646	-0.017	0.043	0.984	7.017E-01	logistic	412805	4135	408670	0.997	0.072
741	Symptoms and disorders of the joints	musculoskeletal	rs35087646	-0.026	0.069	0.974	7.031E-01	logistic	416612	1602	415010	0.997	0.072
476	Allergic rhinitis	respiratory	rs35087646	-0.032	0.085	0.968	7.037E-01	logistic	397580	1063	396517	0.996	0.072
528.1	Stomatitis and mucositis	digestive	rs35087646	0.066	0.173	1.068	7.045E-01	logistic	417947	237	417710	0.998	0.072
363	Chorioretinal inflammations, scars, and other disorders of choroid	sense organs	rs35087646	0.066	0.176	1.069	7.057E-01	logistic	397993	230	397763	0.997	0.072
350.3	Lack of coordination	neurological	rs35087646	-0.062	0.166	0.940	7.074E-01	logistic	420499	289	420210	0.997	0.072
586.1	Anatomical abnormatilies of kidney and ureters	genitourinary	rs35087646	-0.068	0.183	0.934	7.083E-01	logistic	381301	239	381062	0.999	0.072
564.9	Personal history of diseases of digestive system	digestive	rs35087646	-0.009	0.024	0.991	7.119E-01	logistic	348229	13744	334485	0.978	0.072
375.2	Epiphora	sense organs	rs35087646	-0.056	0.154	0.945	7.143E-01	logistic	412257	329	411928	0.997	0.072
198.4	Secondary malignant neoplasm of liver	neoplasms	rs35087646	0.014	0.037	1.014	7.144E-01	logistic	391482	5476	386006	0.997	0.072
293	Symptoms involving head and neck	mental disorders	rs35087646	0.045	0.124	1.046	7.159E-01	logistic	420175	464	419711	0.997	0.072
696.4	Psoriasis	dermatologic	rs35087646	-0.019	0.051	0.982	7.183E-01	logistic	402177	2887	399290	0.997	0.072
427.8	Sinoatrial node dysfunction (Bradycardia)	circulatory system	rs35087646	-0.042	0.116	0.959	7.192E-01	logistic	372427	579	371848	0.998	0.072
756	Other congenital musculoskeletal anomalies	congenital anomalies	rs35087646	-0.032	0.089	0.968	7.196E-01	logistic	422256	974	421282	0.997	0.072
415.21	Primary pulmonary hypertension	circulatory system	rs35087646	0.051	0.143	1.052	7.229E-01	logistic	413189	351	412838	0.998	0.072
159.2	Malignant neoplasm of small intestine, including duodenum	neoplasms	rs35087646	-0.063	0.177	0.939	7.229E-01	logistic	394117	255	393862	0.995	0.072
604.3	Peyronie's disease	genitourinary	rs35087646	0.063	0.178	1.065	7.243E-01	logistic	162002	224	161778	1.000	0.071
714.1	Rheumatoid arthritis	musculoskeletal	rs35087646	0.016	0.044	1.016	7.245E-01	logistic	412357	3850	408507	0.997	0.072
165	Cancer within the respiratory system	neoplasms	rs35087646	-0.013	0.038	0.987	7.248E-01	logistic	422554	5325	417229	0.997	0.072
531.2	Gastric ulcer	digestive	rs35087646	-0.024	0.068	0.977	7.270E-01	logistic	413012	1673	411339	0.996	0.072
197	Chemotherapy	neoplasms	rs35087646	0.009	0.026	1.009	7.272E-01	logistic	397532	11526	386006	0.996	0.072
710.1	Osteomyelitis	musculoskeletal	rs35087646	0.061	0.176	1.063	7.282E-01	logistic	412277	229	412048	0.996	0.072
715	Other inflammatory spondylopathies	musculoskeletal	rs35087646	0.026	0.075	1.026	7.287E-01	logistic	409805	1298	408507	0.997	0.072
578.1	Hematemesis	digestive	rs35087646	-0.026	0.076	0.974	7.287E-01	logistic	384647	1344	383303	0.998	0.071
599.5	Frequency of urination and polyuria	genitourinary	rs35087646	0.032	0.092	1.032	7.296E-01	logistic	390965	859	390106	0.999	0.072
722.9	Other and unspecified disc disorder	musculoskeletal	rs35087646	0.041	0.118	1.041	7.300E-01	logistic	409194	524	408670	0.997	0.072
187.2	Malignant neoplasm of testis	neoplasms	rs35087646	0.014	0.041	1.014	7.311E-01	logistic	184014	4620	179394	1.000	0.071
916	Contusion	injuries & poisonings	rs35087646	-0.025	0.075	0.975	7.332E-01	logistic	421588	1393	420195	0.996	0.072
287.31	Primary thrombocytopenia	hematopoietic	rs35087646	-0.042	0.122	0.959	7.334E-01	logistic	418072	519	417553	0.997	0.072
611	Abnormal findings on mammogram or breast exam	genitourinary	rs35087646	0.056	0.165	1.058	7.340E-01	logistic	415016	264	414752	0.997	0.072
611.3	Lump or mass in breast	genitourinary	rs35087646	0.056	0.165	1.058	7.340E-01	logistic	415016	264	414752	0.997	0.072
289.4	Lymphadenitis	hematopoietic	rs35087646	0.022	0.066	1.023	7.348E-01	logistic	410013	1704	408309	0.998	0.072
208	Benign neoplasm of colon	neoplasms	rs35087646	0.007	0.020	1.007	7.353E-01	logistic	395855	20019	375836	0.995	0.072
368	Visual disturbances	sense organs	rs35087646	0.019	0.057	1.019	7.358E-01	logistic	417843	2298	415545	0.994	0.072
580.3	Nephritis and nephropathy without mention of glomerulonephritis	genitourinary	rs35087646	0.053	0.156	1.054	7.369E-01	logistic	381356	294	381062	0.999	0.072
594.8	Renal colic	genitourinary	rs35087646	-0.040	0.119	0.961	7.376E-01	logistic	412927	551	412376	0.997	0.072
278	Overweight, obesity and other hyperalimentation	endocrine/metabolic	rs35087646	-0.008	0.023	0.993	7.412E-01	logistic	405726	15173	390553	0.998	0.072
204.12	Lymphoid leukemia, chronic	neoplasms	rs35087646	-0.032	0.097	0.969	7.436E-01	logistic	416428	815	415613	0.997	0.072
272	Disorders of lipoid metabolism	endocrine/metabolic	rs35087646	-0.005	0.015	0.995	7.441E-01	logistic	394625	38860	355765	0.999	0.072
724	Other and unspecified disorders of back	musculoskeletal	rs35087646	-0.039	0.120	0.962	7.456E-01	logistic	409212	542	408670	0.997	0.072
751.21	Cystic kidney disease	congenital anomalies	rs35087646	-0.046	0.142	0.955	7.488E-01	logistic	420636	383	420253	0.998	0.072
371	Inflammation of the eye	sense organs	rs35087646	-0.027	0.087	0.973	7.512E-01	logistic	411823	1030	410793	0.998	0.072
596.1	Bladder neck obstruction	genitourinary	rs35087646	-0.037	0.118	0.964	7.525E-01	logistic	402115	561	401554	0.997	0.072
735.21	Hammer toe (acquired)	musculoskeletal	rs35087646	-0.041	0.132	0.959	7.529E-01	logistic	403105	451	402654	0.998	0.072
202	Cancer of other lymphoid, histiocytic tissue	neoplasms	rs35087646	-0.017	0.054	0.983	7.536E-01	logistic	418277	2664	415613	0.996	0.072
202.2	Non-Hodgkins lymphoma	neoplasms	rs35087646	-0.017	0.054	0.983	7.536E-01	logistic	418277	2664	415613	0.996	0.072
634	Miscarriage; stillbirth	pregnancy complications	rs35087646	-0.033	0.105	0.968	7.542E-01	logistic	223299	707	222592	0.593	0.072
292.6	Hallucinations	mental disorders	rs35087646	0.045	0.145	1.046	7.546E-01	logistic	405317	344	404973	0.999	0.072
726.1	Enthesopathy	musculoskeletal	rs35087646	0.015	0.047	1.015	7.554E-01	logistic	392491	3317	389174	0.994	0.072
292.1	Aphasia/speech disturbance	mental disorders	rs35087646	0.026	0.084	1.026	7.563E-01	logistic	406020	1047	404973	0.999	0.072
174	Breast cancer	neoplasms	rs35087646	-0.009	0.028	0.991	7.579E-01	logistic	401305	10248	391057	0.998	0.072
622.1	Polyp of corpus uteri	genitourinary	rs35087646	-0.021	0.069	0.979	7.584E-01	logistic	215181	1609	213572	0.458	0.072
300.12	Agorophobia, social phobia, and panic disorder	mental disorders	rs35087646	0.032	0.105	1.032	7.620E-01	logistic	382286	664	381622	0.995	0.072
695.4	Lupus (localized and systemic)	dermatologic	rs35087646	-0.038	0.125	0.963	7.620E-01	logistic	403975	493	403482	0.997	0.072
278.1	Obesity	endocrine/metabolic	rs35087646	-0.007	0.023	0.993	7.635E-01	logistic	405667	15114	390553	0.998	0.072
496.21	Obstructive chronic bronchitis	respiratory	rs35087646	-0.011	0.038	0.989	7.641E-01	logistic	404163	5354	398809	0.998	0.072
401.2	Hypertensive heart and/or renal disease	circulatory system	rs35087646	-0.024	0.082	0.976	7.692E-01	logistic	291807	1144	290663	0.999	0.072
790.6	Other abnormal blood chemistry	symptoms	rs35087646	-0.053	0.183	0.948	7.700E-01	logistic	422077	237	421840	0.998	0.072
300	Anxiety disorders	mental disorders	rs35087646	-0.008	0.028	0.992	7.702E-01	logistic	391438	9816	381622	0.994	0.072
368.91	Psychophysical visual disturbances	sense organs	rs35087646	0.048	0.166	1.050	7.705E-01	logistic	415808	263	415545	0.993	0.072
149	Cancer of larynx, pharynx, nasal cavities	neoplasms	rs35087646	0.027	0.093	1.027	7.715E-01	logistic	421191	849	420342	0.995	0.072
172.1	Melanomas of skin, dx or hx	neoplasms	rs35087646	0.018	0.064	1.018	7.746E-01	logistic	409038	1830	407208	0.999	0.072
172.11	Melanomas of skin	neoplasms	rs35087646	0.018	0.064	1.018	7.746E-01	logistic	409038	1830	407208	0.999	0.072
626.14	Irregular menstrual bleeding	genitourinary	rs35087646	0.038	0.136	1.039	7.771E-01	logistic	193146	399	192747	0.184	0.072
550.2	Diaphragmatic hernia	digestive	rs35087646	-0.005	0.019	0.995	7.772E-01	logistic	388258	22864	365394	0.998	0.072
242.1	Graves' disease	endocrine/metabolic	rs35087646	0.040	0.140	1.040	7.776E-01	logistic	392598	368	392230	0.997	0.072

ST31; PheWAS results for the lead cis-pQTLs of the 4 proteins prioritized (ACOX1, FGF5, FURIN, and MST1).

SNP = Single Nucleotide Polymorphism; SE = Standard error HWE_p = P-value for Hardy-Weinberg equilibrium test

Phenotype	Description	Group	SNP	Beta	SE	Odds tatio	P-value	Type	n_total	n_cases	n_controls	HWE_p	allele_freq
411.2	Myocardial infarction	circulatory system	rs35087646	-0.007	0.024	0.993	7.777E-01	logistic	385085	14535	370550	0.999	0.072
783	Fever of unknown origin	symptoms	rs35087646	0.016	0.056	1.016	7.789E-01	logistic	418176	2397	415779	0.998	0.072
509.1	Respiratory failure	respiratory	rs35087646	-0.012	0.042	0.988	7.803E-01	logistic	395437	4380	391057	0.999	0.072
550	Abdominal hernia	digestive	rs35087646	-0.005	0.017	0.995	7.853E-01	logistic	392811	27417	365394	0.998	0.072
385.3	Cholesteatoma	sense organs	rs35087646	-0.051	0.189	0.950	7.859E-01	logistic	418937	221	418716	0.996	0.072
8.5	Bacterial enteritis	infectious diseases	rs35087646	-0.021	0.078	0.979	7.863E-01	logistic	418567	1251	417316	0.997	0.072
560.3	Peritoneal or intestinal adhesions	digestive	rs35087646	0.025	0.094	1.026	7.873E-01	logistic	335317	832	334485	0.986	0.072
578.8	Hemorrhage of rectum and anus	digestive	rs35087646	0.015	0.057	1.015	7.875E-01	logistic	385630	2327	383303	0.996	0.072
427.6	Premature beats	circulatory system	rs35087646	0.030	0.111	1.030	7.884E-01	logistic	372449	601	371848	0.998	0.072
596.5	Functional disorders of bladder	genitourinary	rs35087646	-0.029	0.110	0.971	7.884E-01	logistic	402194	640	401554	0.997	0.072
858	Complication of internal orthopedic device	injuries & poisonings	rs35087646	0.021	0.077	1.021	7.891E-01	logistic	417889	1237	416652	0.998	0.072
870	Open wounds of head; neck; and trunk	injuries & poisonings	rs35087646	-0.026	0.097	0.975	7.895E-01	logistic	420976	826	420150	0.997	0.072
727.4	Ganglion and cyst of synovium, tendon, and bursa	musculoskeletal	rs35087646	-0.034	0.129	0.967	7.925E-01	logistic	389641	467	389174	0.994	0.072
225	Benign neoplasm of brain and other parts of nervous system	neoplasms	rs35087646	-0.025	0.096	0.975	7.934E-01	logistic	421233	836	420397	0.997	0.072
575.8	Other disorders of biliary tract	digestive	rs35087646	0.022	0.084	1.022	7.959E-01	logistic	396103	1056	395047	0.999	0.072
250.23	Type 2 diabetes with ophthalmic manifestations	endocrine/metabolic	rs35087646	-0.016	0.063	0.984	7.968E-01	logistic	388590	1953	386637	0.990	0.072
272.1	Hyperlipidemia	endocrine/metabolic	rs35087646	-0.004	0.015	0.996	7.999E-01	logistic	394381	38616	355765	0.999	0.072
225.1	Benign neoplasm of brain, cranial nerves, meninges	neoplasms	rs35087646	-0.024	0.097	0.976	8.032E-01	logistic	421204	807	420397	0.997	0.072
697	Sarcoidosis	dermatologic	rs35087646	0.028	0.111	1.028	8.035E-01	logistic	405795	589	405206	0.997	0.072
332	Parkinson's disease	neurological	rs35087646	0.013	0.051	1.013	8.037E-01	logistic	401966	2919	399047	0.997	0.072
300.1	Anxiety disorder	mental disorders	rs35087646	-0.007	0.029	0.993	8.046E-01	logistic	390805	9183	381622	0.994	0.072
803	Fracture of upper limb	injuries & poisonings	rs35087646	0.010	0.042	1.010	8.056E-01	logistic	400593	4215	396378	0.989	0.072
569	Other disorders of intestine	digestive	rs35087646	0.014	0.059	1.015	8.059E-01	logistic	385990	2151	383839	0.997	0.072
480.5	Bronchopneumonia and lung abscess	respiratory	rs35087646	-0.033	0.135	0.968	8.070E-01	logistic	397229	429	396800	0.998	0.072
201	Hodgkin's disease	neoplasms	rs35087646	-0.040	0.166	0.961	8.096E-01	logistic	415895	282	415613	0.996	0.072
586.11	Small kidney	genitourinary	rs35087646	-0.045	0.189	0.956	8.101E-01	logistic	381281	219	381062	0.999	0.072
277.4	Disorders of bilirubin excretion	endocrine/metabolic	rs35087646	-0.031	0.129	0.970	8.109E-01	logistic	420403	462	419941	0.998	0.072
198.2	Secondary malignancy of respiratory organs	neoplasms	rs35087646	-0.010	0.040	0.990	8.119E-01	logistic	390707	4701	386006	0.998	0.072
38.2	Gram positive septicemia	infectious diseases	rs35087646	-0.026	0.112	0.974	8.141E-01	logistic	397268	621	396647	0.998	0.072
939	Atopic/contact dermatitis due to other or unspecified	dermatologic	rs35087646	0.014	0.062	1.014	8.159E-01	logistic	416950	1963	414987	0.997	0.072
374	Other disorders of eyelids	sense organs	rs35087646	0.013	0.058	1.013	8.163E-01	logistic	413055	2262	410793	0.999	0.072
531.4	Peptic ulcer, site unspecified	digestive	rs35087646	-0.043	0.186	0.958	8.178E-01	logistic	411565	226	411339	0.997	0.072
801	Fracture of ankle and foot	injuries & poisonings	rs35087646	-0.025	0.108	0.976	8.189E-01	logistic	397040	662	396378	0.993	0.072
535.8	Other specified gastritis	digestive	rs35087646	0.012	0.052	1.012	8.191E-01	logistic	365017	2803	362214	0.995	0.072
578.2	Blood in stool	digestive	rs35087646	-0.014	0.065	0.986	8.256E-01	logistic	385103	1800	383303	0.997	0.071
781	Symptoms involving nervous and musculoskeletal system	symptoms	rs35087646	0.007	0.031	1.007	8.257E-01	logistic	419375	7893	411482	0.997	0.072
530.5	Disorders of esophageal motility	digestive	rs35087646	-0.030	0.135	0.971	8.265E-01	logistic	357212	424	356788	0.979	0.072
512.8	Cough	respiratory	rs35087646	-0.015	0.069	0.985	8.273E-01	logistic	406184	1585	404599	0.995	0.072
626	Disorders of menstruation and other abnormal bleeding from female genital trac	genitourinary	rs35087646	0.008	0.036	1.008	8.302E-01	logistic	198823	6076	192747	0.362	0.072
624.9	stress incontinence, female	genitourinary	rs35087646	-0.014	0.064	0.986	8.311E-01	logistic	220155	1871	218284	0.585	0.072
429.2	Abnormal function study of cardiovascular system	circulatory system	rs35087646	0.032	0.148	1.032	8.314E-01	logistic	410036	333	409703	0.998	0.072
430.2	Intracerebral hemorrhage	circulatory system	rs35087646	-0.018	0.084	0.982	8.315E-01	logistic	403773	1088	402685	0.997	0.072
580	Nephritis; nephrosis; renal sclerosis	genitourinary	rs35087646	-0.018	0.084	0.982	8.325E-01	logistic	382137	1075	381062	0.999	0.072
480	Pneumonia	respiratory	rs35087646	-0.005	0.023	0.995	8.336E-01	logistic	412462	15662	396800	0.998	0.072
446.9	Arteritis NOS	circulatory system	rs35087646	0.030	0.142	1.030	8.338E-01	logistic	405830	361	405469	0.999	0.072
364	Corneal opacity and other disorders of cornea	sense organs	rs35087646	0.014	0.065	1.014	8.343E-01	logistic	399513	1750	397763	0.997	0.072
706.2	Sebaceous cyst	dermatologic	rs35087646	-0.015	0.074	0.985	8.354E-01	logistic	413393	1394	411999	0.998	0.072
496.2	Chronic bronchitis	respiratory	rs35087646	-0.008	0.037	0.992	8.371E-01	logistic	404328	5519	398809	0.998	0.072
174.1	Breast cancer [female]	neoplasms	rs35087646	-0.006	0.028	0.994	8.372E-01	logistic	213896	10137	203759	0.338	0.072
155	Cancer of liver and intrahepatic bile duct	neoplasms	rs35087646	0.021	0.100	1.021	8.372E-01	logistic	394604	742	393862	0.996	0.072
585.1	Acute renal failure	genitourinary	rs35087646	0.005	0.024	1.005	8.381E-01	logistic	394375	13313	381062	1.000	0.072
709	Diffuse diseases of connective tissue	dermatologic	rs35087646	-0.017	0.082	0.983	8.384E-01	logistic	405028	1129	403899	0.997	0.072
550.6	Incisional hernia	digestive	rs35087646	0.026	0.127	1.026	8.385E-01	logistic	365851	457	365394	0.998	0.072
750.1	Upper gastrointestinal congenital anomalies	congenital anomalies	rs35087646	0.038	0.186	1.038	8.400E-01	logistic	420464	211	420253	0.998	0.072
726	Peripheral enthesopathies and allied syndromes	musculoskeletal	rs35087646	0.005	0.027	1.005	8.423E-01	logistic	399760	10586	389174	0.998	0.072
175	Acquired absence of breast	neoplasms	rs35087646	0.009	0.044	1.009	8.427E-01	logistic	206502	3992	202510	0.295	0.072
331	Other cerebral degenerations	neurological	rs35087646	0.013	0.067	1.013	8.428E-01	logistic	400699	1652	399047	0.997	0.072
961	Poisoning by other anti-infectives	injuries & poisonings	rs35087646	-0.032	0.164	0.968	8.431E-01	logistic	367461	288	367173	0.998	0.072
550.5	Ventral hernia	digestive	rs35087646	-0.013	0.068	0.987	8.458E-01	logistic	367031	1637	365394	0.998	0.072
70.3	Viral hepatitis C	infectious diseases	rs35087646	-0.032	0.168	0.968	8.477E-01	logistic	415468	264	415204	0.997	0.072
522	Diseases of pulp and periapical tissues	digestive	rs35087646	-0.031	0.162	0.969	8.481E-01	logistic	409832	296	409536	0.995	0.072
429	Ill-defined descriptions and complications of heart disease	circulatory system	rs35087646	0.028	0.148	1.029	8.481E-01	logistic	410037	334	409703	0.998	0.072
255.1	Adrenal hyperfunction	endocrine/metabolic	rs35087646	-0.037	0.196	0.964	8.502E-01	logistic	415447	201	415246	0.997	0.072
368.4	Visual field defects	sense organs	rs35087646	-0.024	0.132	0.976	8.534E-01	logistic	415988	443	415545	0.992	0.072
170	Cancer of bone and connective tissue	neoplasms	rs35087646	0.026	0.143	1.027	8.538E-01	logistic	423460	359	423101	0.997	0.072
285	Other anemias	hematopoietic	rs35087646	0.005	0.026	1.005	8.563E-01	logistic	393921	11472	382449	0.999	0.072
619	Noninflammatory female genital disorders	genitourinary	rs35087646	0.011	0.058	1.011	8.568E-01	logistic	219175	2209	216966	0.062	0.072
560.4	Other intestinal obstruction	digestive	rs35087646	0.011	0.059	1.011	8.584E-01	logistic	336617	2132	334485	0.986	0.072
714	Rheumatoid arthritis and other inflammatory polyarthropathies	musculoskeletal	rs35087646	0.008	0.043	1.008	8.589E-01	logistic	412574	4067	408507	0.997	0.072
555.2	Ulcerative colitis	digestive	rs35087646	-0.009	0.049	0.991	8.607E-01	logistic	337679	3194	334485	0.989	0.072
798	Malaise and fatigue	symptoms	rs35087646	0.007	0.042	1.007	8.632E-01	logistic	418584	4264	414320	0.997	0.072

ST31; PheWAS results for the lead cis-pQTLs of the 4 proteins prioritized (ACOX1, FGF5, FURIN, and MST1).

SNP = Single Nucleotide Polymorphism; SE = Standard error HWE_p = P-value for Hardy-Weinberg equilibrium test

Phenotype	Description	Group	SNP	Beta	SE	Odds tatio	P-value	Type	n_total	n_cases	n_controls	HWE_p	allele_freq
433.21	Cerebral artery occlusion, with cerebral infarctior	circulatory system	rs35087646	0.008	0.045	1.008	8.633E-01	logistic	406495	3810	402685	0.998	0.072
614.5	Inflammatory disease of cervix, vagina, and vulva	genitourinary	rs35087646	-0.020	0.120	0.980	8.651E-01	logistic	217969	531	217438	0.423	0.072
577.3	Cyst and pseudocyst of pancreas	digestive	rs35087646	0.020	0.120	1.021	8.654E-01	logistic	418522	514	418008	0.997	0.072
253.2	Pituitary hypofunction	endocrine/metabolic	rs35087646	-0.028	0.164	0.973	8.660E-01	logistic	415534	288	415246	0.997	0.072
374.3	Ptosis of eyelid	sense organs	rs35087646	-0.013	0.080	0.987	8.674E-01	logistic	411997	1204	410793	0.999	0.072
427.22	Atrial flutter	circulatory system	rs35087646	-0.018	0.109	0.982	8.677E-01	logistic	372499	651	371848	0.998	0.072
420.3	Endocarditis	circulatory system	rs35087646	0.018	0.110	1.018	8.695E-01	logistic	417383	613	416770	0.997	0.072
411.1	Unstable angina (intermediate coronary syndrome)	circulatory system	rs35087646	0.007	0.045	1.007	8.713E-01	logistic	374269	3719	370550	1.000	0.072
427.9	Palpitations	circulatory system	rs35087646	-0.011	0.066	0.989	8.720E-01	logistic	373589	1741	371848	0.997	0.072
941	Adverse reaction to serum or vaccine	injuries & poisonings	rs35087646	0.029	0.181	1.029	8.735E-01	logistic	415212	225	414987	0.997	0.072
574.12	Cholelithiasis with other cholecystitis	digestive	rs35087646	0.011	0.069	1.011	8.761E-01	logistic	396636	1589	395047	0.999	0.072
555	Inflammatory bowel disease and other gastroenteritis and coliti	digestive	rs35087646	0.006	0.040	1.006	8.766E-01	logistic	339234	4749	334485	0.990	0.072
198	Secondary malignant neoplasm	neoplasms	rs35087646	0.003	0.021	1.003	8.784E-01	logistic	403287	17281	386006	0.998	0.072
458.9	Hypotension NOS	circulatory system	rs35087646	0.006	0.042	1.006	8.787E-01	logistic	376953	4381	372572	0.997	0.072
334.2	Anterior horn cell disease	neurological	rs35087646	-0.019	0.130	0.981	8.832E-01	logistic	399497	450	399047	0.997	0.072
389.3	Degenerative and vascular disorders of ear	sense organs	rs35087646	0.022	0.147	1.022	8.837E-01	logistic	408638	341	408297	0.996	0.072
117.4	Aspergillosis	infectious diseases	rs35087646	0.027	0.187	1.027	8.844E-01	logistic	418590	210	418380	0.998	0.072
594.1	Calculus of kidney	genitourinary	rs35087646	0.007	0.049	1.007	8.845E-01	logistic	415555	3179	412376	0.998	0.072
710	Osteomyelitis, periostitis, and other infections involving bone	musculoskeletal	rs35087646	0.023	0.162	1.024	8.857E-01	logistic	412326	278	412048	0.996	0.072
733	Other disorders of bone and cartilage	musculoskeletal	rs35087646	-0.013	0.091	0.987	8.882E-01	logistic	399585	913	398672	0.997	0.072
531.1	Hemorrhage from gastrointestinal ulcer	digestive	rs35087646	-0.016	0.115	0.984	8.899E-01	logistic	411913	574	411339	0.997	0.072
358.1	Myasthenia gravis	neurological	rs35087646	0.022	0.158	1.022	8.900E-01	logistic	419463	295	419168	0.996	0.072
384	Other disorders of tympanic membrane	sense organs	rs35087646	-0.020	0.145	0.981	8.923E-01	logistic	419080	364	418716	0.997	0.072
446.4	Wegener's granulomatosis	circulatory system	rs35087646	-0.024	0.180	0.976	8.940E-01	logistic	405703	234	405469	0.999	0.072
442.1	Aortic aneurysm	circulatory system	rs35087646	0.007	0.055	1.007	8.967E-01	logistic	407951	2482	405469	0.999	0.072
751	Genitourinary congenital anomalies	congenital anomalies	rs35087646	0.012	0.092	1.012	8.976E-01	logistic	421129	876	420253	0.998	0.072
195	Cancer, suspected or other	neoplasms	rs35087646	-0.004	0.032	0.996	8.990E-01	logistic	393669	7663	386006	0.995	0.072
70	Viral hepatitis	infectious diseases	rs35087646	0.014	0.115	1.015	8.995E-01	logistic	415755	551	415204	0.996	0.072
430	Intracranial hemorrhage	circulatory system	rs35087646	-0.008	0.063	0.992	9.004E-01	logistic	404595	1910	402685	0.997	0.072
579.8	Nonspecific abnormal findings in stool contents	digestive	rs35087646	0.014	0.110	1.014	9.009E-01	logistic	383921	618	383303	0.998	0.071
289.8	Polycythemia, secondary	hematopoietic	rs35087646	-0.016	0.128	0.984	9.016E-01	logistic	407605	466	407139	0.997	0.072
195.1	Malignant neoplasm, other	neoplasms	rs35087646	-0.004	0.032	0.996	9.019E-01	logistic	393640	7634	386006	0.995	0.072
280	Iron deficiency anemias	hematopoietic	rs35087646	-0.003	0.029	0.997	9.050E-01	logistic	391534	9085	382449	0.998	0.072
362.3	Other nondiabetic retinopathy	sense organs	rs35087646	-0.014	0.117	0.986	9.061E-01	logistic	398314	557	397757	0.997	0.072
573.3	Hepatomegaly	digestive	rs35087646	-0.019	0.166	0.981	9.067E-01	logistic	400551	277	400274	0.998	0.072
540.11	Acute appendicitis	digestive	rs35087646	0.021	0.183	1.022	9.069E-01	logistic	421875	219	421656	0.997	0.072
696.42	Psoriatic arthropathy	dermatologic	rs35087646	-0.011	0.092	0.989	9.076E-01	logistic	400183	893	399290	0.998	0.072
379	Other disorders of eye	sense organs	rs35087646	-0.009	0.086	0.991	9.120E-01	logistic	412963	1035	411928	0.997	0.072
960	Poisoning by antibiotics	injuries & poisonings	rs35087646	-0.002	0.019	0.998	9.148E-01	logistic	389532	22359	367173	0.996	0.072
159.3	Malignant neoplasm of gallbladder and extrahepatic bile ducts	neoplasms	rs35087646	0.016	0.150	1.016	9.151E-01	logistic	394194	332	393862	0.995	0.072
317.11	Alcoholic liver damage	mental disorders	rs35087646	-0.008	0.079	0.992	9.162E-01	logistic	404493	1200	403293	0.999	0.072
442	Other aneurysm	circulatory system	rs35087646	-0.005	0.050	0.995	9.186E-01	logistic	408493	3024	405469	0.999	0.072
291	Other specified nonpsychotic and/or transient mental disorders	mental disorders	rs35087646	-0.014	0.138	0.986	9.206E-01	logistic	405371	398	404973	0.998	0.072
291.8	Alteration of consciousness	mental disorders	rs35087646	-0.014	0.144	0.986	9.215E-01	logistic	405343	370	404973	0.999	0.072
509	Respiratory failure, insufficiency, arrest	respiratory	rs35087646	0.003	0.036	1.003	9.224E-01	logistic	397038	5981	391057	0.998	0.072
695.42	Systemic lupus erythematosus	dermatologic	rs35087646	-0.013	0.136	0.987	9.224E-01	logistic	403885	403	403482	0.997	0.072
202.24	Large cell lymphoma	neoplasms	rs35087646	-0.008	0.083	0.992	9.227E-01	logistic	416717	1104	415613	0.996	0.072
579.2	Splenomegaly	digestive	rs35087646	0.010	0.110	1.011	9.241E-01	logistic	383919	616	383303	0.997	0.071
170.2	Cancer of connective tissue	neoplasms	rs35087646	-0.016	0.176	0.984	9.256E-01	logistic	423349	248	423101	0.997	0.072
958	Certain early complications of trauma or procedure	injuries & poisonings	rs35087646	0.015	0.162	1.015	9.257E-01	logistic	423583	281	423302	0.998	0.072
41.11	Methicillin sensitive Staphylococcus aureus	infectious diseases	rs35087646	0.006	0.068	1.006	9.262E-01	logistic	398282	1635	396647	0.998	0.072
522.5	Periapical abscess	digestive	rs35087646	0.017	0.189	1.017	9.271E-01	logistic	409744	208	409536	0.995	0.072
481	Influenza	respiratory	rs35087646	-0.011	0.128	0.989	9.286E-01	logistic	397264	464	396800	0.998	0.072
760	Back pain	symptoms	rs35087646	0.003	0.038	1.003	9.373E-01	logistic	414925	5193	409732	0.997	0.072
276.42	Alkalosis	endocrine/metabolic	rs35087646	0.012	0.156	1.012	9.379E-01	logistic	395878	306	395572	0.999	0.072
788	Syncope and collapse	symptoms	rs35087646	0.003	0.035	1.003	9.390E-01	logistic	412052	6241	405811	0.997	0.072
433.2	Occlusion of cerebral arteries	circulatory system	rs35087646	-0.003	0.045	0.997	9.411E-01	logistic	406563	3878	402685	0.998	0.072
277	Other disorders of metabolism	endocrine/metabolic	rs35087646	0.006	0.083	1.006	9.414E-01	logistic	421029	1088	419941	0.997	0.072
275	Disorders of mineral metabolism	endocrine/metabolic	rs35087646	-0.003	0.040	0.997	9.419E-01	logistic	420958	4773	416185	0.997	0.072
189.21	Malignant neoplasm of bladder	neoplasms	rs35087646	-0.004	0.050	0.996	9.420E-01	logistic	420210	3023	417187	0.998	0.072
803.2	Fracture of radius and ulna	injuries & poisonings	rs35087646	-0.004	0.058	0.996	9.440E-01	logistic	398618	2240	396378	0.988	0.072
193	Thyroid cancer	neoplasms	rs35087646	0.009	0.134	1.009	9.452E-01	logistic	421300	413	420887	0.996	0.072
292	Neurological disorders	mental disorders	rs35087646	-0.003	0.038	0.997	9.454E-01	logistic	410372	5399	404973	0.999	0.072
41.1	Staphylococcus infections	infectious diseases	rs35087646	0.004	0.057	1.004	9.462E-01	logistic	398981	2334	396647	0.998	0.072
447	Other disorders of arteries and arterioles	circulatory system	rs35087646	-0.005	0.083	0.995	9.481E-01	logistic	406558	1089	405469	0.999	0.072
426.24	Atrioventricular block, complete	circulatory system	rs35087646	0.006	0.088	1.006	9.482E-01	logistic	372833	985	371848	0.998	0.072
401.22	Hypertensive chronic kidney disease	circulatory system	rs35087646	-0.006	0.088	0.994	9.484E-01	logistic	291632	969	290663	0.999	0.072
556.1	Ulceration of intestine	digestive	rs35087646	0.012	0.193	1.013	9.484E-01	logistic	334685	200	334485	0.986	0.072
280.1	Iron deficiency anemias, unspecified or not due to blood loss	hematopoietic	rs35087646	0.002	0.030	1.002	9.494E-01	logistic	391238	8789	382449	0.998	0.072
283	Acquired hemolytic anemias	hematopoietic	rs35087646	0.012	0.192	1.012	9.495E-01	logistic	382649	200	382449	0.999	0.072
741.4	Joint effusions	musculoskeletal	rs35087646	0.006	0.105	1.006	9.512E-01	logistic	415693	683	415010	0.997	0.072

ST31; PheWAS results for the lead cis-pQTLs of the 4 proteins prioritized (ACOX1, FGF5, FURIN, and MST1).

SNP = Single Nucleotide Polymorphism; SE = Standard error HWE_p = P-value for Hardy-Weinberg equilibrium test

Phenotype	Description	Group	SNP	Beta	SE	Odds tatio	P-value	Type	n_total	n_cases	n_controls	HWE_p	allele_freq
585.4	Chronic kidney disease, Stage I or II	genitourinary	rs35087646	-0.006	0.097	0.994	9.532E-01	logistic	381861	799	381062	0.999	0.072
642	Hypertension complicating pregnancy, childbirth, and the puerperiun	pregnancy complications	rs35087646	0.008	0.130	1.008	9.533E-01	logistic	228250	456	227794	0.555	0.072
228	Hemangioma and lymphangioma, any site	neoplasms	rs35087646	-0.006	0.119	0.994	9.585E-01	logistic	421655	529	421126	0.997	0.072
565.1	Anal and rectal polyp	digestive	rs35087646	0.003	0.053	1.003	9.587E-01	logistic	386554	2715	383839	0.997	0.072
706	Diseases of sebaceous glands	dermatologic	rs35087646	-0.004	0.073	0.996	9.588E-01	logistic	413434	1435	411999	0.998	0.072
618.2	Uterine/Uterovaginal prolapse	genitourinary	rs35087646	-0.005	0.093	0.995	9.588E-01	logistic	214329	874	213455	0.531	0.072
253.7	Other disorders of neurohypophysis	endocrine/metabolic	rs35087646	-0.008	0.148	0.993	9.595E-01	logistic	415594	348	415246	0.997	0.072
151	Cancer of stomach	neoplasms	rs35087646	0.004	0.088	1.004	9.599E-01	logistic	394829	967	393862	0.997	0.072
681.1	Cellulitis and abscess of fingers/toes	dermatologic	rs35087646	0.009	0.181	1.009	9.609E-01	logistic	418724	227	418497	0.996	0.072
187.1	Malignant neoplasm of unspecified male genital orgar	neoplasms	rs35087646	-0.002	0.041	0.998	9.627E-01	logistic	183955	4561	179394	1.000	0.071
389.4	Tinnitus	sense organs	rs35087646	-0.006	0.127	0.994	9.638E-01	logistic	408770	473	408297	0.997	0.072
507	Pleurisy; pleural effusion	respiratory	rs35087646	-0.001	0.028	0.999	9.658E-01	logistic	400937	9880	391057	0.999	0.072
736	Other acquired deformities of limbs	musculoskeletal	rs35087646	-0.004	0.089	0.996	9.663E-01	logistic	403605	951	402654	0.997	0.072
260	Protein-calorie malnutrition	endocrine/metabolic	rs35087646	0.006	0.141	1.006	9.686E-01	logistic	412473	376	412097	0.997	0.072
275.1	Disorders of iron metabolism	hematopoietic	rs35087646	-0.003	0.083	0.997	9.690E-01	logistic	417274	1089	416185	0.997	0.072
500.2	Pneumoconiosis	respiratory	rs35087646	-0.005	0.142	0.995	9.735E-01	logistic	391432	375	391057	0.999	0.072
694	Dyschromia and Vitiligo	dermatologic	rs35087646	0.005	0.157	1.005	9.749E-01	logistic	405505	299	405206	0.997	0.072
358	Myoneural disorders	neurological	rs35087646	-0.005	0.154	0.995	9.757E-01	logistic	419485	317	419168	0.996	0.072
386.9	Dizziness and giddiness (Light-headedness and vertigo)	sense organs	rs35087646	-0.002	0.050	0.998	9.760E-01	logistic	412711	3046	409665	0.995	0.072
362.31	Separation of retinal layers	sense organs	rs35087646	0.003	0.121	1.003	9.773E-01	logistic	398271	514	397757	0.997	0.072
381	Otitis media and Eustachian tube disorders	sense organs	rs35087646	-0.003	0.094	0.997	9.775E-01	logistic	419568	852	418716	0.996	0.072
384.4	Perforation of tympanic membrane	sense organs	rs35087646	-0.005	0.175	0.995	9.781E-01	logistic	418963	247	418716	0.996	0.072
443	Peripheral vascular disease	circulatory system	rs35087646	0.001	0.039	1.001	9.789E-01	logistic	410595	5126	405469	0.999	0.072
276.12	Hyposmolality and/or hyponatremia	endocrine/metabolic	rs35087646	0.001	0.039	1.001	9.832E-01	logistic	400551	4979	395572	0.999	0.072
261	Vitamin deficiency	endocrine/metabolic	rs35087646	0.001	0.044	1.001	9.845E-01	logistic	416032	3935	412097	0.997	0.072
441.1	Acute vascular insufficiency of intestine	circulatory system	rs35087646	0.002	0.158	1.002	9.890E-01	logistic	405771	302	405469	0.999	0.072
626.8	Infertility, female	genitourinary	rs35087646	0.002	0.163	1.002	9.892E-01	logistic	193029	282	192747	0.202	0.072
618.1	Prolapse of vaginal walls	genitourinary	rs35087646	-0.001	0.044	0.999	9.908E-01	logistic	217426	3971	213455	0.451	0.072
117	Mycoses	infectious diseases	rs35087646	0.002	0.166	1.002	9.910E-01	logistic	418652	272	418380	0.998	0.072
615	Endometriosis	genitourinary	rs35087646	-0.001	0.078	0.999	9.912E-01	logistic	218669	1231	217438	0.426	0.072
804	Fracture of hand or wrist	injuries & poisonings	rs35087646	0.001	0.114	1.001	9.912E-01	logistic	396955	577	396378	0.994	0.072
465	Acute upper respiratory infections of multiple or unspecified sites	respiratory	rs35087646	0.001	0.104	1.001	9.919E-01	logistic	420386	694	419692	0.997	0.072
275.3	Disorders of magnesium metabolism	endocrine/metabolic	rs35087646	0.001	0.070	1.001	9.938E-01	logistic	417729	1544	416185	0.997	0.072
709.2	Sicca syndrome	dermatologic	rs35087646	0.000	0.101	1.000	9.966E-01	logistic	404638	739	403899	0.997	0.072
420	Carditis	circulatory system	rs35087646	0.000	0.059	1.000	9.974E-01	logistic	418942	2172	416770	0.997	0.072
200	Myeloproliferative disease	neoplasms	rs35087646	0.000	0.071	1.000	9.984E-01	logistic	417103	1490	415613	0.996	0.072
564.1	Irritable Bowel Syndrome	digestive	rs76133235	-0.180	0.059	0.835	2.220E-03	logistic	339145	4660	334485	0.065	0.039
535.8	Other specified gastritis	digestive	rs76133235	-0.233	0.078	0.792	2.705E-03	logistic	365017	2803	362214	0.038	0.038
279.1	Immunity deficiency	endocrine/metabolic	rs76133235	-0.837	0.304	0.433	5.929E-03	logistic	423199	323	422876	0.464	0.038
569.2	Gastrointestinal complications	digestive	rs76133235	0.482	0.177	1.620	6.404E-03	logistic	384117	278	383839	0.427	0.039
710.1	Osteomyelitis	musculoskeletal	rs76133235	0.520	0.191	1.681	6.659E-03	logistic	412277	229	412048	0.635	0.038
714.1	Rheumatoid arthritis	musculoskeletal	rs76133235	-0.175	0.065	0.839	6.733E-03	logistic	412357	3850	408507	0.593	0.038
601	Inflammatory diseases of prostate	genitourinary	rs76133235	0.281	0.106	1.325	8.031E-03	logistic	157382	921	156461	0.804	0.039
279.11	Deficiency of humoral immunity	endocrine/metabolic	rs76133235	-1.004	0.381	0.366	8.325E-03	logistic	423118	242	422876	0.465	0.038
41.2	Streptococcus infection	infectious diseases	rs76133235	-0.276	0.108	0.759	1.051E-02	logistic	398155	1508	396647	0.005	0.038
365.2	Primary angle-closure glaucoma	sense organs	rs76133235	-0.440	0.174	0.644	1.132E-02	logistic	398441	678	397763	0.483	0.038
531.1	Hemorrhage from gastrointestinal ulcer	digestive	rs76133235	0.332	0.131	1.394	1.157E-02	logistic	411913	574	411339	0.447	0.038
70.3	Viral hepatitis C	infectious diseases	rs76133235	-0.837	0.336	0.433	1.272E-02	logistic	415468	264	415204	0.400	0.038
496.1	Emphysema	respiratory	rs76133235	0.156	0.063	1.169	1.333E-02	logistic	401774	2965	398809	0.232	0.038
798	Malaise and fatigue	symptoms	rs76133235	-0.150	0.061	0.861	1.343E-02	logistic	418584	4264	414320	0.387	0.038
695.7	Prurigo and Lichen	dermatologic	rs76133235	0.430	0.174	1.538	1.348E-02	logistic	405510	304	405206	0.488	0.038
743.2	Pathologic fracture	musculoskeletal	rs76133235	-0.347	0.142	0.706	1.445E-02	logistic	422164	930	421234	0.265	0.038
558	Noninfectious gastroenteritis	digestive	rs76133235	-0.144	0.059	0.866	1.466E-02	logistic	338928	4443	334485	0.203	0.039
610	Benign mammary dysplasias	genitourinary	rs76133235	0.338	0.142	1.403	1.684E-02	logistic	220777	505	220272	0.622	0.038
550.2	Diaphragmatic hernia	digestive	rs76133235	-0.061	0.026	0.941	1.768E-02	logistic	388258	22864	365394	0.033	0.038
500.2	Pneumoconiosis	respiratory	rs76133235	0.375	0.159	1.455	1.815E-02	logistic	391432	375	391057	0.237	0.038
279	Disorders involving the immune mechanism	endocrine/metabolic	rs76133235	-0.596	0.253	0.551	1.827E-02	logistic	423247	371	422876	0.461	0.038
414	Other forms of chronic heart disease	circulatory system	rs76133235	-0.144	0.061	0.866	1.900E-02	logistic	374670	4120	370550	0.435	0.038
595	Hydronephrosis	genitourinary	rs76133235	-0.318	0.136	0.728	1.912E-02	logistic	413362	986	412376	0.628	0.038
714	Rheumatoid arthritis and other inflammatory polyarthropathies	musculoskeletal	rs76133235	-0.145	0.062	0.865	1.986E-02	logistic	412574	4067	408507	0.580	0.038
743	Osteoporosis, osteopenia and pathological fracture	musculoskeletal	rs76133235	-0.287	0.124	0.750	2.053E-02	logistic	422387	1153	421234	0.256	0.038
710	Osteomyelitis, periostitis, and other infections involving bone	musculoskeletal	rs76133235	0.419	0.182	1.520	2.126E-02	logistic	412326	278	412048	0.634	0.038
290.13	Senile dementia	mental disorders	rs76133235	0.165	0.072	1.180	2.131E-02	logistic	407253	2280	404973	0.426	0.039
585.4	Chronic kidney disease, Stage I or II	genitourinary	rs76133235	-0.352	0.153	0.703	2.149E-02	logistic	381861	799	381062	0.191	0.038
496.21	Obstructive chronic bronchitis	respiratory	rs76133235	0.110	0.048	1.116	2.311E-02	logistic	404163	5354	398809	0.393	0.038
440.2	Atherosclerosis of the extremities	circulatory system	rs76133235	0.249	0.111	1.283	2.426E-02	logistic	406343	874	405469	0.449	0.038
550	Abdominal hernia	digestive	rs76133235	-0.053	0.024	0.948	2.530E-02	logistic	392811	27417	365394	0.046	0.038
187	Cancer of other male genital organs	neoplasms	rs76133235	-0.101	0.045	0.904	2.531E-02	logistic	186921	7527	179394	0.871	0.039
601.1	Prostatitis	genitourinary	rs76133235	0.271	0.122	1.311	2.645E-02	logistic	157164	703	156461	0.817	0.039
496.2	Chronic bronchitis	respiratory	rs76133235	0.105	0.048	1.111	2.754E-02	logistic	404328	5519	398809	0.416	0.038
195	Cancer, suspected or other	neoplasms	rs76133235	-0.098	0.044	0.907	2.765E-02	logistic	393669	7663	386006	0.208	0.038

ST31; PheWAS results for the lead cis-pQTLs of the 4 proteins prioritized (ACOX1, FGF5, FURIN, and MST1).

SNP = Single Nucleotide Polymorphism; SE = Standard error HWE_p = P-value for Hardy-Weinberg equilibrium test

Phenotype	Description	Group	SNP	Beta	SE	Odds tatio	P-value	Type	n_total	n_cases	n_controls	HWE_p	allele_freq
365.1	Open-angle glaucoma	sense organs	rs76133235	0.185	0.085	1.203	3.032E-02	logistic	399325	1562	397763	0.512	0.039
337	Disorders of the autonomic nervous system	neurological	rs76133235	0.361	0.167	1.435	3.051E-02	logistic	399396	349	399047	0.308	0.038
195.1	Malignant neoplasm, other	neoplasms	rs76133235	-0.096	0.044	0.909	3.110E-02	logistic	393640	7634	386006	0.208	0.038
496	Chronic airway obstruction	respiratory	rs76133235	0.060	0.028	1.061	3.140E-02	logistic	416701	17892	398809	0.363	0.038
394.1	Mitral valve stenosis and aortic valve stenosis	circulatory system	rs76133235	0.213	0.100	1.237	3.280E-02	logistic	402530	1116	401414	0.470	0.038
433.21	Cerebral artery occlusion, with cerebral infarction	circulatory system	rs76133235	-0.136	0.064	0.873	3.307E-02	logistic	406495	3810	402685	0.263	0.038
720.1	Spinal stenosis of lumbar region	musculoskeletal	rs76133235	-0.163	0.078	0.849	3.626E-02	logistic	411268	2598	408670	0.422	0.038
350	Abnormal movement	neurological	rs76133235	0.203	0.097	1.224	3.657E-02	logistic	421410	1200	420210	0.402	0.038
70	Viral hepatitis	infectious diseases	rs76133235	-0.392	0.188	0.676	3.730E-02	logistic	415755	551	415204	0.419	0.038
242.1	Graves' disease	endocrine/metabolic	rs76133235	0.342	0.165	1.407	3.811E-02	logistic	392598	368	392230	0.419	0.038
586.2	Cyst of kidney, acquired	genitourinary	rs76133235	-0.186	0.093	0.830	4.525E-02	logistic	382916	1854	381062	0.170	0.038
241.1	Nontoxic uninodular goiter	endocrine/metabolic	rs76133235	-0.669	0.337	0.512	4.671E-02	logistic	392455	225	392230	0.357	0.038
603	Other disorders of testis	genitourinary	rs76133235	0.293	0.148	1.340	4.847E-02	logistic	162246	468	161778	0.643	0.039
369	Infection of the eye	sense organs	rs76133235	-0.435	0.221	0.647	4.872E-02	logistic	411210	417	410793	0.489	0.038
564	Functional digestive disorders	digestive	rs76133235	-0.054	0.027	0.948	4.994E-02	logistic	354387	19902	334485	0.370	0.039
603.1	Hydrocele	genitourinary	rs76133235	0.296	0.151	1.345	5.053E-02	logistic	162225	447	161778	0.644	0.039
433.2	Occlusion of cerebral arteries	circulatory system	rs76133235	-0.122	0.063	0.885	5.108E-02	logistic	406563	3878	402685	0.256	0.038
180.3	Cervical intraepithelial neoplasia [CIN] [Cervical dysplasia	neoplasms	rs76133235	0.290	0.149	1.336	5.109E-02	logistic	196385	481	195904	0.798	0.038
525	Other diseases of the teeth and supporting structures	digestive	rs76133235	-0.386	0.199	0.680	5.226E-02	logistic	410027	491	409536	0.438	0.038
276.6	Fluid overload	endocrine/metabolic	rs76133235	-0.190	0.098	0.827	5.345E-02	logistic	397239	1667	395572	0.303	0.038
362.29	Macular degeneration (senile) of retina NOS	sense organs	rs76133235	-0.120	0.063	0.887	5.481E-02	logistic	401665	3908	397757	0.504	0.038
159.3	Malignant neoplasm of gallbladder and extrahepatic bile ducts	neoplasms	rs76133235	-0.483	0.253	0.617	5.611E-02	logistic	394194	332	393862	0.360	0.038
427.21	Atrial fibrillation	circulatory system	rs76133235	-0.113	0.060	0.893	5.675E-02	logistic	376121	4273	371848	0.477	0.038
858	Complication of internal orthopedic device	injuries & poisonings	rs76133235	0.182	0.096	1.200	5.890E-02	logistic	417889	1237	416652	0.440	0.038
350.1	Abnormal involuntary movements	neurological	rs76133235	0.221	0.118	1.247	6.020E-02	logistic	421010	800	420210	0.396	0.038
763	Thoracic or lumbosacral neuritis or radiculitis, unspecified	symptoms	rs76133235	-0.527	0.281	0.590	6.025E-02	logistic	418269	283	417986	0.289	0.038
604.3	Peyronie's disease	genitourinary	rs76133235	0.381	0.206	1.464	6.365E-02	logistic	162002	224	161778	0.694	0.039
289.3	Personal history of diseases of blood and blood-forming organs	hematopoietic	rs76133235	-0.501	0.270	0.606	6.402E-02	logistic	408604	295	408309	0.091	0.038
300.9	Posttraumatic stress disorder	mental disorders	rs76133235	0.352	0.194	1.422	7.020E-02	logistic	381881	259	381622	0.201	0.039
613	Other nonmalignant breast conditions	genitourinary	rs76133235	0.293	0.163	1.340	7.187E-02	logistic	421594	397	421197	0.490	0.038
578.1	Hematemesis	digestive	rs76133235	0.167	0.093	1.181	7.221E-02	logistic	384647	1344	383303	0.483	0.039
374.1	Ectropion or entropion	sense organs	rs76133235	0.225	0.125	1.252	7.238E-02	logistic	411493	700	410793	0.479	0.038
241	Nontoxic nodular goiter	endocrine/metabolic	rs76133235	-0.272	0.151	0.762	7.280E-02	logistic	392996	766	392230	0.370	0.038
427.12	Paroxysmal ventricular tachycardia	circulatory system	rs76133235	-0.233	0.130	0.792	7.298E-02	logistic	372834	986	371848	0.539	0.038
502	Postinflammatory pulmonary fibrosis	respiratory	rs76133235	-0.157	0.088	0.855	7.518E-02	logistic	393073	2016	391057	0.200	0.038
334.2	Anterior horn cell disease	neurological	rs76133235	0.272	0.153	1.312	7.543E-02	logistic	399497	450	399047	0.335	0.038
741.3	Difficulty in walking	musculoskeletal	rs76133235	0.268	0.151	1.308	7.601E-02	logistic	415474	464	415010	0.408	0.038
367	Disorders of refraction and accommodation; blindness and low vision	sense organs	rs76133235	0.081	0.046	1.084	7.709E-02	logistic	417170	6164	411006	0.494	0.038
550.5	Ventral hernia	digestive	rs76133235	-0.174	0.098	0.841	7.765E-02	logistic	367031	1637	365394	0.020	0.039
331	Other cerebral degenerations	neurological	rs76133235	0.149	0.085	1.160	7.862E-02	logistic	400699	1652	399047	0.330	0.038
187.2	Malignant neoplasm of testis	neoplasms	rs76133235	-0.100	0.057	0.905	7.877E-02	logistic	184014	4620	179394	0.790	0.039
428.1	Congestive heart failure (CHF) NOS	circulatory system	rs76133235	-0.108	0.062	0.898	8.043E-02	logistic	413649	3946	409703	0.423	0.038
159.4	Malignant neoplasm of retroperitoneum and peritoneum	neoplasms	rs76133235	0.338	0.194	1.403	8.164E-02	logistic	394127	265	393862	0.373	0.038
375	Disorders of lacrimal system	sense organs	rs76133235	-0.184	0.106	0.832	8.202E-02	logistic	413379	1451	411928	0.607	0.038
695	Erythematous conditions	dermatologic	rs76133235	0.142	0.082	1.152	8.251E-02	logistic	407007	1801	405206	0.500	0.038
369.5	Conjunctivitis, infectious	sense organs	rs76133235	-0.583	0.337	0.558	8.338E-02	logistic	410999	206	410793	0.493	0.038
153	Colorectal cancer	neoplasms	rs76133235	-0.079	0.046	0.924	8.350E-02	logistic	377768	7048	370720	0.048	0.038
695.9	Unspecified erythematous condition	dermatologic	rs76133235	-0.437	0.253	0.646	8.417E-02	logistic	405524	318	405206	0.481	0.038
642	Hypertension complicating pregnancy, childbirth, and the puerperium	pregnancy complications	rs76133235	0.267	0.155	1.306	8.525E-02	logistic	228250	456	227794	0.450	0.038
371.1	Uveitis, noninfectious or NOS	sense organs	rs76133235	-0.503	0.292	0.605	8.529E-02	logistic	411047	254	410793	0.465	0.038
153.3	Malignant neoplasm of rectum, rectosigmoid junction, and anus	neoplasms	rs76133235	-0.125	0.073	0.883	8.586E-02	logistic	373609	2889	370720	0.035	0.038
565	Anal and rectal conditions	digestive	rs76133235	-0.076	0.044	0.927	8.745E-02	logistic	391353	7514	383839	0.310	0.038
720	Spinal stenosis	musculoskeletal	rs76133235	-0.102	0.060	0.903	9.139E-02	logistic	412805	4135	408670	0.442	0.038
783	Fever of unknown origin	symptoms	rs76133235	-0.135	0.080	0.874	9.203E-02	logistic	418176	2397	415779	0.346	0.038
591	Urinary tract infection	genitourinary	rs76133235	0.057	0.034	1.059	9.247E-02	logistic	388723	11796	376927	0.249	0.038
172	Skin cancer	neoplasms	rs76133235	0.077	0.046	1.080	9.551E-02	logistic	413263	6055	407208	0.486	0.038
614.3	Pelvic inflammatory disease (PID)	genitourinary	rs76133235	-0.507	0.305	0.603	9.707E-02	logistic	217675	237	217438	0.452	0.038
426.32	Left bundle branch block	circulatory system	rs76133235	-0.116	0.070	0.890	9.723E-02	logistic	374914	3066	371848	0.621	0.038
727	Other disorders of synovium, tendon, and bursa	musculoskeletal	rs76133235	-0.125	0.076	0.882	9.747E-02	logistic	391842	2668	389174	0.783	0.038
250.23	Type 2 diabetes with ophthalmic manifestations	endocrine/metabolic	rs76133235	-0.147	0.089	0.864	9.990E-02	logistic	388590	1953	386637	0.369	0.038
729	Other disorders of soft tissues	musculoskeletal	rs76133235	-0.480	0.292	0.619	1.004E-01	logistic	389423	249	389174	0.799	0.039
366.2	Senile cataract	sense organs	rs76133235	0.063	0.038	1.065	1.018E-01	logistic	380814	9136	371678	0.691	0.038
277	Other disorders of metabolism	endocrine/metabolic	rs76133235	-0.200	0.122	0.819	1.027E-01	logistic	421029	1088	419941	0.344	0.038
743.21	Pathologic fracture of vertebrae	musculoskeletal	rs76133235	-0.272	0.167	0.762	1.033E-01	logistic	421861	627	421234	0.268	0.038
289	Other diseases of blood and blood-forming organs	hematopoietic	rs76133235	-0.233	0.144	0.793	1.057E-01	logistic	409120	811	408309	0.104	0.038
331.9	Cerebral degeneration, unspecified	neurological	rs76133235	0.176	0.109	1.193	1.070E-01	logistic	400009	962	399047	0.338	0.038
454.1	Varicose veins of lower extremity	circulatory system	rs76133235	-0.092	0.057	0.912	1.074E-01	logistic	385486	4520	380966	0.127	0.038
281.13	Folate-deficiency anemia	hematopoietic	rs76133235	0.267	0.166	1.306	1.080E-01	logistic	382829	380	382449	0.445	0.039
800.1	Fracture of neck of femur	injuries & poisonings	rs76133235	-0.139	0.087	0.871	1.108E-01	logistic	398432	2054	396378	0.464	0.038
253.7	Other disorders of neurohypophysis	endocrine/metabolic	rs76133235	0.275	0.173	1.317	1.122E-01	logistic	415594	348	415246	0.312	0.038
367.9	Blindness and low vision	sense organs	rs76133235	0.182	0.114	1.199	1.126E-01	logistic	411881	875	411006	0.489	0.038

ST31; PheWAS results for the lead cis-pQTLs of the 4 proteins prioritized (ACOX1, FGF5, FURIN, and MST1).

SNP = Single Nucleotide Polymorphism; SE = Standard error HWE_p = P-value for Hardy-Weinberg equilibrium test

Phenotype	Description	Group	SNP	Beta	SE	Odds tatio	P-value	Type	n_total	n_cases	n_controls	HWE_p	allele_freq
187.1	Malignant neoplasm of unspecified male genital orgar	neoplasms	rs76133235	-0.090	0.057	0.914	1.144E-01	logistic	183955	4561	179394	0.789	0.039
411.2	Myocardial infarction	circulatory system	rs76133235	-0.050	0.032	0.951	1.166E-01	logistic	385085	14535	370550	0.587	0.038
745	Pain in joint	musculoskeletal	rs76133235	0.094	0.060	1.098	1.168E-01	logistic	414363	3501	410862	0.387	0.038
155.1	Malignant neoplasm of liver, primary	neoplasms	rs76133235	0.280	0.178	1.323	1.170E-01	logistic	394187	325	393862	0.430	0.038
561	Symptoms involving digestive systen	digestive	rs76133235	-0.085	0.054	0.919	1.172E-01	logistic	339539	5054	334485	0.292	0.039
427.6	Premature beats	circulatory system	rs76133235	-0.262	0.169	0.769	1.210E-01	logistic	372449	601	371848	0.495	0.038
427.1	Paroxysmal tachycardia, unspecified	circulatory system	rs76133235	-0.107	0.069	0.898	1.210E-01	logistic	374981	3133	371848	0.542	0.038
284	Aplastic anemia	hematopoietic	rs76133235	-0.235	0.151	0.791	1.212E-01	logistic	383179	730	382449	0.393	0.039
323	Encephalitis	neurological	rs76133235	-0.360	0.233	0.698	1.219E-01	logistic	422420	349	422071	0.465	0.038
426.3	Bundle branch block	circulatory system	rs76133235	-0.100	0.065	0.904	1.227E-01	logistic	375377	3529	371848	0.604	0.038
172.2	Other non-epithelial cancer of skin	neoplasms	rs76133235	0.091	0.059	1.095	1.237E-01	logistic	410817	3609	407208	0.460	0.038
389	Hearing loss	sense organs	rs76133235	-0.070	0.045	0.933	1.241E-01	logistic	415421	7124	408297	0.368	0.038
568	Other disorders of peritoneum	digestive	rs76133235	-0.158	0.103	0.853	1.254E-01	logistic	385305	1466	383839	0.407	0.038
574.11	Cholelithiasis with acute cholecystitis	digestive	rs76133235	0.164	0.107	1.179	1.257E-01	logistic	396059	1012	395047	0.342	0.038
454	Varicose veins	circulatory system	rs76133235	-0.084	0.055	0.919	1.276E-01	logistic	385827	4861	380966	0.143	0.038
440	Atherosclerosis	circulatory system	rs76133235	0.158	0.104	1.171	1.287E-01	logistic	406545	1076	405469	0.473	0.038
368.91	Psychophysical visual disturbances	sense organs	rs76133235	0.299	0.197	1.348	1.300E-01	logistic	415808	263	415545	0.400	0.038
8.52	Intestinal infection due to C. difficile	infectious diseases	rs76133235	0.196	0.130	1.217	1.311E-01	logistic	417984	668	417316	0.373	0.038
250.1	Type 1 diabetes	endocrine/metabolic	rs76133235	-0.120	0.079	0.887	1.314E-01	logistic	389041	2404	386637	0.349	0.038
415.2	Chronic pulmonary heart disease	circulatory system	rs76133235	0.129	0.085	1.137	1.316E-01	logistic	414491	1653	412838	0.330	0.038
285	Other anemias	hematopoietic	rs76133235	-0.054	0.036	0.948	1.335E-01	logistic	393921	11472	382449	0.590	0.038
740.11	Osteoarthrosis, localized, primary	musculoskeletal	rs76133235	-0.079	0.053	0.924	1.359E-01	logistic	351108	5250	345858	0.803	0.038
428	Congestive heart failure; nonhypertensive	circulatory system	rs76133235	-0.063	0.042	0.939	1.382E-01	logistic	417859	8156	409703	0.448	0.038
443.1	Raynaud's syndrome	circulatory system	rs76133235	-0.147	0.099	0.863	1.396E-01	logistic	407046	1577	405469	0.370	0.038
332	Parkinson's disease	neurological	rs76133235	-0.105	0.072	0.900	1.424E-01	logistic	401966	2919	399047	0.441	0.038
575.2	Obstruction of bile duct	digestive	rs76133235	0.155	0.107	1.168	1.456E-01	logistic	396076	1029	395047	0.373	0.038
276	Disorders of fluid, electrolyte, and acid-base balance	endocrine/metabolic	rs76133235	0.043	0.030	1.044	1.464E-01	logistic	411277	15705	395572	0.402	0.038
804	Fracture of hand or wrist	injuries & poisonings	rs76133235	-0.249	0.172	0.780	1.476E-01	logistic	396955	577	396378	0.458	0.038
690	Erythematosquamous dermatosis	dermatologic	rs76133235	0.194	0.134	1.214	1.490E-01	logistic	405831	625	405206	0.419	0.038
965	Poisoning by analgesics, antipyretics, and antirheumatics	injuries & poisonings	rs76133235	-0.080	0.056	0.923	1.499E-01	logistic	371918	4745	367173	0.228	0.038
415	Pulmonary heart disease	circulatory system	rs76133235	0.121	0.085	1.129	1.516E-01	logistic	414538	1700	412838	0.329	0.038
455	Hemorrhoids	circulatory system	rs76133235	-0.075	0.053	0.928	1.561E-01	logistic	386244	5278	380966	0.086	0.038
579.8	Nonspecific abnormal findings in stool contents	digestive	rs76133235	-0.234	0.165	0.792	1.562E-01	logistic	383921	618	383303	0.401	0.038
198	Secondary malignant neoplasm	neoplasms	rs76133235	-0.041	0.029	0.960	1.574E-01	logistic	403287	17281	386006	0.266	0.038
276.1	Electrolyte imbalance	endocrine/metabolic	rs76133235	0.050	0.036	1.052	1.591E-01	logistic	406083	10511	395572	0.286	0.038
594.1	Calculus of kidney	genitourinary	rs76133235	-0.096	0.068	0.909	1.608E-01	logistic	415555	3179	412376	0.575	0.038
371	Inflammation of the eye	sense organs	rs76133235	-0.174	0.124	0.840	1.615E-01	logistic	411823	1030	410793	0.465	0.038
340	Migraine	neurological	rs76133235	-0.108	0.077	0.898	1.622E-01	logistic	407732	2545	405187	0.436	0.038
785	Abdominal pain	symptoms	rs76133235	-0.063	0.045	0.939	1.626E-01	logistic	401183	7225	393958	0.330	0.038
368.2	Diplopia and disorders of binocular vision	sense organs	rs76133235	0.204	0.146	1.227	1.626E-01	logistic	416069	524	415545	0.356	0.038
282	Hereditary hemolytic anemias	hematopoietic	rs76133235	-0.426	0.306	0.653	1.629E-01	logistic	382667	218	382449	0.410	0.039
198.7	Secondary malignant neoplasm of skin	neoplasms	rs76133235	-0.378	0.271	0.685	1.632E-01	logistic	386268	262	386006	0.154	0.038
501	Pneumonitis due to inhalation of food or vomitus	respiratory	rs76133235	-0.128	0.092	0.880	1.654E-01	logistic	392843	1786	391057	0.269	0.038
458.9	Hypotension NOS	circulatory system	rs76133235	-0.080	0.058	0.923	1.671E-01	logistic	376953	4381	372572	0.202	0.038
427.2	Atrial fibrillation and flutter	circulatory system	rs76133235	-0.042	0.030	0.959	1.679E-01	logistic	388316	16468	371848	0.446	0.038
740.12	Osteoarthrosis, localized, secondary	musculoskeletal	rs76133235	-0.279	0.203	0.756	1.684E-01	logistic	346286	428	345858	0.759	0.038
363	Chorioretinal inflammations, scars, and other disorders of choroid	sense organs	rs76133235	-0.403	0.292	0.669	1.684E-01	logistic	397993	230	397763	0.488	0.038
286	Coagulation defects	hematopoietic	rs76133235	-0.180	0.131	0.835	1.695E-01	logistic	418481	928	417553	0.398	0.038
415.21	Primary pulmonary hypertension	circulatory system	rs76133235	0.241	0.176	1.273	1.699E-01	logistic	413189	351	412838	0.340	0.038
597	Other disorders of urethra and urinary tract	genitourinary	rs76133235	-0.118	0.086	0.889	1.704E-01	logistic	403600	2046	401554	0.586	0.038
590	Pyelonephritis	genitourinary	rs76133235	0.128	0.094	1.136	1.750E-01	logistic	378294	1367	376927	0.036	0.038
727.6	Rupture of tendon, nontraumatic	musculoskeletal	rs76133235	-0.145	0.107	0.865	1.768E-01	logistic	390517	1343	389174	0.793	0.038
569.1	Toxic gastroenteritis and colitis	digestive	rs76133235	0.246	0.184	1.279	1.812E-01	logistic	384157	318	383839	0.433	0.039
800	Fracture of lower limb	injuries & poisonings	rs76133235	-0.093	0.069	0.912	1.813E-01	logistic	399482	3104	396378	0.420	0.038
681.1	Cellulitis and abscess of fingers/toes	dermatologic	rs76133235	0.285	0.214	1.329	1.831E-01	logistic	418724	227	418497	0.448	0.038
344	Other paralytic syndromes	neurological	rs76133235	-0.208	0.157	0.812	1.842E-01	logistic	399714	667	399047	0.380	0.038
702.2	Seborrheic keratosis	dermatologic	rs76133235	0.192	0.145	1.212	1.847E-01	logistic	413611	539	413072	0.466	0.038
568.1	Peritoneal adhesions (postoperative) (postinfection	digestive	rs76133235	-0.144	0.109	0.866	1.865E-01	logistic	385136	1297	383839	0.411	0.038
458.1	Orthostatic hypotension	circulatory system	rs76133235	0.084	0.063	1.087	1.871E-01	logistic	375723	3151	372572	0.272	0.038
500	Lung disease due to external agents	respiratory	rs76133235	0.178	0.136	1.195	1.890E-01	logistic	391675	618	391057	0.229	0.038
690.1	Seborrheic dermatitis	dermatologic	rs76133235	0.179	0.137	1.196	1.901E-01	logistic	405818	612	405206	0.421	0.038
618.2	Uterine/Uterovaginal prolapsc	genitourinary	rs76133235	0.152	0.117	1.165	1.921E-01	logistic	214329	874	213455	0.198	0.038
202.24	Large cell lymphoma	neoplasms	rs76133235	0.135	0.104	1.145	1.923E-01	logistic	416717	1104	415613	0.371	0.038
696.42	Psoriatic arthropathy	dermatologic	rs76133235	-0.173	0.134	0.841	1.943E-01	logistic	400183	893	399290	0.462	0.038
735.3	Hallux valgus (Bunion)	musculoskeletal	rs76133235	0.093	0.072	1.097	1.948E-01	logistic	405116	2462	402654	0.702	0.038
281.11	Pernicious anemia	hematopoietic	rs76133235	-0.192	0.148	0.825	1.954E-01	logistic	383186	737	382449	0.421	0.039
348.2	Cerebral edema and compression of brain	neurological	rs76133235	0.221	0.171	1.247	1.959E-01	logistic	399425	378	399047	0.343	0.038
594	Urinary calculus	genitourinary	rs76133235	-0.064	0.050	0.938	1.971E-01	logistic	418195	5819	412376	0.555	0.038
287.31	Primary thrombocytopenia	hematopoietic	rs76133235	0.190	0.148	1.210	1.978E-01	logistic	418072	519	417553	0.505	0.038
990	Effects radiation NOS	injuries & poisonings	rs76133235	-0.059	0.046	0.943	1.989E-01	logistic	408877	6919	401958	0.397	0.038
741	Symptoms and disorders of the joints	musculoskeletal	rs76133235	0.112	0.087	1.119	2.003E-01	logistic	416612	1602	415010	0.354	0.038

ST31; PheWAS results for the lead cis-pQTLs of the 4 proteins prioritized (ACOX1, FGF5, FURIN, and MST1).

SNP = Single Nucleotide Polymorphism; SE = Standard error HWE_p = P-value for Hardy-Weinberg equilibrium test

Phenotype	Description	Group	SNP	Beta	SE	Odds tatio	P-value	Type	n_total	n_cases	n_controls	HWE_p	allele_freq
528.11	Stomatitis and mucositis (ulcerative)	digestive	rs76133235	-0.390	0.305	0.677	2.011E-01	logistic	417919	209	417710	0.419	0.038
253	Disorders of the pituitary gland and its hypothalamic contro	endocrine/metabolic	rs76133235	0.146	0.115	1.158	2.035E-01	logistic	416136	890	415246	0.346	0.038
389.4	Tinnitus	sense organs	rs76133235	0.196	0.154	1.216	2.051E-01	logistic	408770	473	408297	0.347	0.038
615	Endometriosis	genitourinary	rs76133235	-0.143	0.114	0.867	2.085E-01	logistic	218669	1231	217438	0.420	0.038
368	Visual disturbances	sense organs	rs76133235	0.093	0.074	1.097	2.086E-01	logistic	417843	2298	415545	0.445	0.038
367.2	Astigmatism	sense organs	rs76133235	0.077	0.061	1.080	2.095E-01	logistic	414418	3412	411006	0.515	0.038
386.1	Meniere's disease	sense organs	rs76133235	0.168	0.134	1.183	2.123E-01	logistic	410308	643	409665	0.318	0.038
579	Other symptoms involving abdomen and pelvis	digestive	rs76133235	-0.152	0.123	0.859	2.133E-01	logistic	384340	1037	383303	0.413	0.038
296.22	Major depressive disorder	mental disorders	rs76133235	-0.038	0.031	0.962	2.141E-01	logistic	396820	15198	381622	0.416	0.038
961.1	Poisoning/allergy of sulfonamides	injuries & poisonings	rs76133235	0.168	0.136	1.183	2.155E-01	logistic	367807	634	367173	0.319	0.038
528.1	Stomatitis and mucositis	digestive	rs76133235	-0.348	0.281	0.706	2.160E-01	logistic	417947	237	417710	0.418	0.038
727.1	Synovitis and tenosynovitis	musculoskeletal	rs76133235	-0.296	0.239	0.744	2.161E-01	logistic	389486	312	389174	0.812	0.039
530.14	Reflux esophagitis	digestive	rs76133235	0.077	0.062	1.080	2.189E-01	logistic	360032	3244	356788	0.304	0.039
275	Disorders of mineral metabolism	endocrine/metabolic	rs76133235	0.064	0.052	1.066	2.207E-01	logistic	420958	4773	416185	0.371	0.038
702.1	Actinic keratosis	dermatologic	rs76133235	0.138	0.113	1.148	2.218E-01	logistic	413997	925	413072	0.448	0.038
275.5	Disorders of calcium/phosphorus metabolism	endocrine/metabolic	rs76133235	0.090	0.074	1.094	2.226E-01	logistic	418502	2317	416185	0.427	0.038
751.21	Cystic kidney disease	congenital anomalies	rs76133235	-0.258	0.212	0.773	2.230E-01	logistic	420636	383	420253	0.431	0.038
153.2	Colon cancer	neoplasms	rs76133235	-0.068	0.057	0.934	2.271E-01	logistic	375260	4540	370720	0.020	0.038
497	Bronchitis	respiratory	rs76133235	-0.297	0.246	0.743	2.280E-01	logistic	399105	296	398809	0.285	0.038
681	Superficial cellulitis and abscess	dermatologic	rs76133235	0.229	0.190	1.257	2.294E-01	logistic	418799	302	418497	0.444	0.038
530.5	Disorders of esophageal motility	digestive	rs76133235	-0.239	0.199	0.787	2.296E-01	logistic	357212	424	356788	0.409	0.039
381.1	Otitis media	sense organs	rs76133235	-0.190	0.159	0.827	2.323E-01	logistic	419355	639	418716	0.487	0.038
782.3	Edema	symptoms	rs76133235	0.085	0.072	1.089	2.347E-01	logistic	419509	2451	417058	0.293	0.038
587	Kidney replaced by transpant	genitourinary	rs76133235	-0.216	0.183	0.806	2.378E-01	logistic	381556	494	381062	0.190	0.038
726	Peripheral enthesopathies and allied syndromes	musculoskeletal	rs76133235	-0.044	0.037	0.957	2.379E-01	logistic	399760	10586	389174	0.720	0.038
165.1	Cancer of bronchus; lung	neoplasms	rs76133235	0.065	0.055	1.067	2.384E-01	logistic	421452	4223	417229	0.406	0.038
722.9	Other and unspecified disc disorder	musculoskeletal	rs76133235	-0.208	0.177	0.812	2.385E-01	logistic	409194	524	408670	0.430	0.038
228	Hemangioma and lymphangioma, any site	neoplasms	rs76133235	0.174	0.148	1.190	2.391E-01	logistic	421655	529	421126	0.346	0.038
250.11	Type 1 diabetes with ketoacidosis	endocrine/metabolic	rs76133235	-0.318	0.271	0.727	2.404E-01	logistic	386885	248	386637	0.338	0.038
170.2	Cancer of connective tissue	neoplasms	rs76133235	-0.318	0.271	0.728	2.408E-01	logistic	423349	248	423101	0.464	0.038
916	Contusion	injuries & poisonings	rs76133235	0.110	0.094	1.116	2.411E-01	logistic	421588	1393	420195	0.384	0.038
770	Myalgia and myositis unspecified	symptoms	rs76133235	-0.106	0.091	0.899	2.417E-01	logistic	422525	1839	420686	0.469	0.038
278.1	Obesity	endocrine/metabolic	rs76133235	-0.036	0.031	0.964	2.419E-01	logistic	405667	15114	390553	0.352	0.038
695.42	Systemic lupus erythematosus	dermatologic	rs76133235	0.197	0.168	1.218	2.419E-01	logistic	403885	403	403482	0.495	0.038
508	Pulmonary collapse; interstitial and compensatory emphysema	respiratory	rs76133235	-0.072	0.062	0.931	2.433E-01	logistic	394890	3833	391057	0.304	0.038
384.4	Perforation of tympanic membrane	sense organs	rs76133235	-0.315	0.271	0.729	2.444E-01	logistic	418963	247	418716	0.472	0.038
286.1	Congenital coagulation defects	hematopoietic	rs76133235	-0.231	0.199	0.793	2.454E-01	logistic	417976	423	417553	0.415	0.038
185	Cancer of prostate	neoplasms	rs76133235	-0.043	0.037	0.958	2.466E-01	logistic	167540	11079	156461	0.864	0.039
781	Symptoms involving nervous and musculoskeletal system	symptoms	rs76133235	0.048	0.041	1.049	2.474E-01	logistic	419375	7893	411482	0.439	0.038
150	Cancer of esophagus	neoplasms	rs76133235	0.106	0.092	1.112	2.494E-01	logistic	395291	1429	393862	0.434	0.038
601.12	Chronic prostatitis	genitourinary	rs76133235	0.246	0.214	1.279	2.495E-01	logistic	156694	233	156461	0.822	0.039
352	Disorders of other cranial nerves	neurological	rs76133235	-0.128	0.112	0.880	2.511E-01	logistic	403637	1222	402415	0.525	0.038
531.2	Gastric ulcer	digestive	rs76133235	-0.107	0.095	0.898	2.574E-01	logistic	413012	1673	411339	0.461	0.038
261	Vitamin deficiency	endocrine/metabolic	rs76133235	0.065	0.057	1.067	2.588E-01	logistic	416032	3935	412097	0.330	0.038
579.2	Splenomegaly	digestive	rs76133235	-0.181	0.161	0.834	2.600E-01	logistic	383919	616	383303	0.398	0.038
145	Cancer of mouth	neoplasms	rs76133235	0.148	0.132	1.160	2.608E-01	logistic	421016	674	420342	0.512	0.038
790.6	Other abnormal blood chemistry	symptoms	rs76133235	0.240	0.214	1.271	2.621E-01	logistic	422077	237	421840	0.347	0.038
385	Other disorders of middle ear and mastoid	sense organs	rs76133235	0.213	0.190	1.237	2.627E-01	logistic	419023	307	418716	0.459	0.038
635.2	Antepartum hemorrhage, abruptio placentae, and placenta previa	pregnancy complications	rs76133235	-0.340	0.306	0.712	2.656E-01	logistic	222797	205	222592	0.478	0.038
507	Pleurisy; pleural effusion	respiratory	rs76133235	0.041	0.037	1.042	2.666E-01	logistic	400937	9880	391057	0.269	0.038
301	Personality disorders	mental disorders	rs76133235	-0.246	0.222	0.782	2.671E-01	logistic	381968	346	381622	0.248	0.038
70.4	Chronic hepatitis	infectious diseases	rs76133235	0.217	0.197	1.242	2.709E-01	logistic	415491	287	415204	0.380	0.038
426.24	Atrioventricular block, complete	circulatory system	rs76133235	-0.137	0.124	0.872	2.712E-01	logistic	372833	985	371848	0.505	0.038
451.2	Phlebitis and thrombophlebitis of lower extremities	circulatory system	rs76133235	-0.201	0.182	0.818	2.712E-01	logistic	381453	487	380966	0.061	0.038
703.1	Ingrowing nail	dermatologic	rs76133235	0.252	0.229	1.287	2.714E-01	logistic	421519	205	421314	0.508	0.038
411.9	Other acute and subacute forms of ischemic heart disease	circulatory system	rs76133235	0.100	0.091	1.105	2.716E-01	logistic	372042	1492	370550	0.573	0.039
426.23	Second degree AV block	circulatory system	rs76133235	-0.172	0.157	0.842	2.716E-01	logistic	372488	640	371848	0.518	0.038
300.1	Anxiety disorder	mental disorders	rs76133235	-0.044	0.040	0.957	2.720E-01	logistic	390805	9183	381622	0.307	0.038
709.2	Sicca syndrome	dermatologic	rs76133235	-0.161	0.147	0.851	2.722E-01	logistic	404638	739	403899	0.492	0.038
149	Cancer of larynx, pharynx, nasal cavities	neoplasms	rs76133235	-0.148	0.135	0.863	2.729E-01	logistic	421191	849	420342	0.522	0.038
278	Overweight, obesity and other hyperalimentation	endocrine/metabolic	rs76133235	-0.034	0.031	0.967	2.735E-01	logistic	405726	15173	390553	0.377	0.038
530.12	Ulcer of esophagus	digestive	rs76133235	0.076	0.069	1.078	2.751E-01	logistic	359422	2634	356788	0.402	0.039
218.1	Uterine leiomyoma	neoplasms	rs76133235	-0.080	0.074	0.923	2.755E-01	logistic	209756	2761	206995	0.826	0.038
427.9	Palpitations	circulatory system	rs76133235	0.092	0.085	1.097	2.767E-01	logistic	373589	1741	371848	0.449	0.038
280.2	Iron deficiency anemia secondary to blood loss (chronic	hematopoietic	rs76133235	-0.260	0.239	0.771	2.771E-01	logistic	382749	300	382449	0.405	0.039
559	Ileostomy status	digestive	rs76133235	-0.098	0.090	0.907	2.778E-01	logistic	336295	1810	334485	0.192	0.039
599	Other symptoms/disorders or the urinary system	genitourinary	rs76133235	-0.045	0.042	0.956	2.811E-01	logistic	398281	8175	390106	0.485	0.038
170	Cancer of bone and connective tissue	neoplasms	rs76133235	-0.233	0.216	0.792	2.812E-01	logistic	423460	359	423101	0.460	0.038
977	Personal history of allergy to medicinal agents	injuries & poisonings	rs76133235	-0.045	0.042	0.956	2.815E-01	logistic	375378	8205	367173	0.353	0.038
614.5	Inflammatory disease of cervix, vagina, and vulva	genitourinary	rs76133235	0.161	0.150	1.174	2.822E-01	logistic	217969	531	217438	0.449	0.038
182	Malignant neoplasm of uterus	neoplasms	rs76133235	-0.120	0.112	0.887	2.826E-01	logistic	197217	1245	195972	0.762	0.038

ST31; PheWAS results for the lead cis-pQTLs of the 4 proteins prioritized (ACOX1, FGF5, FURIN, and MST1).
SNP = Single Nucleotide Polymorphism; SE = Standard error HWE_p = P-value for Hardy-Weinberg equilibrium test

Phenotype	Description	Group	SNP	Beta	SE	Odds tatio	P-value	Type	n_total	n_cases	n_controls	HWE_p	allele_freq
175	Acquired absence of breast	neoplasms	rs76133235	-0.066	0.061	0.937	2.832E-01	logistic	206502	3992	202510	0.832	0.038
218	Benign neoplasm of uterus	neoplasms	rs76133235	-0.077	0.072	0.926	2.843E-01	logistic	209861	2866	206995	0.810	0.038
368.4	Visual field defects	sense organs	rs76133235	-0.205	0.192	0.815	2.862E-01	logistic	415988	443	415545	0.377	0.038
433	Cerebrovascular disease	circulatory system	rs76133235	-0.038	0.036	0.963	2.894E-01	logistic	414008	11323	402685	0.342	0.038
870	Open wounds of head; neck; and trunk	injuries & poisonings	rs76133235	0.128	0.120	1.136	2.895E-01	logistic	420976	826	420150	0.512	0.038
597.1	Urethral stricture (not specified as infectious)	genitourinary	rs76133235	-0.095	0.090	0.909	2.914E-01	logistic	403370	1816	401554	0.590	0.038
300	Anxiety disorders	mental disorders	rs76133235	-0.040	0.038	0.960	2.924E-01	logistic	391438	9816	381622	0.308	0.038
624.9	stress incontinence, female	genitourinary	rs76133235	0.087	0.082	1.091	2.934E-01	logistic	220155	1871	218284	0.433	0.038
217	Vascular hamartomas and non-neoplastic nevi	neoplasms	rs76133235	0.202	0.193	1.224	2.966E-01	logistic	414259	301	413958	0.422	0.038
871	Open wounds of extremities	injuries & poisonings	rs76133235	-0.221	0.212	0.802	2.975E-01	logistic	420520	370	420150	0.465	0.038
401	Hypertension	circulatory system	rs76133235	-0.015	0.014	0.985	2.985E-01	logistic	390097	99434	290663	0.095	0.038
200.1	Polycythemia vera	neoplasms	rs76133235	-0.180	0.174	0.835	3.006E-01	logistic	408705	523	408182	0.193	0.038
292	Neurological disorders	mental disorders	rs76133235	-0.053	0.052	0.948	3.030E-01	logistic	410372	5399	404973	0.406	0.038
540	Appendiceal conditions	digestive	rs76133235	0.163	0.158	1.177	3.031E-01	logistic	422123	467	421656	0.499	0.038
634.1	Missed abortion/Hydatidiform mole	pregnancy complications	rs76133235	-0.301	0.293	0.740	3.043E-01	logistic	222807	215	222592	0.440	0.038
599.4	Urinary incontinence	genitourinary	rs76133235	-0.075	0.074	0.927	3.051E-01	logistic	392806	2700	390106	0.450	0.038
292.4	Altered mental status	mental disorders	rs76133235	-0.066	0.065	0.936	3.054E-01	logistic	408434	3461	404973	0.399	0.038
374	Other disorders of eyelids	sense organs	rs76133235	0.077	0.075	1.080	3.056E-01	logistic	413055	2262	410793	0.470	0.038
334	Degenerative disease of the spinal cord	neurological	rs76133235	0.087	0.085	1.091	3.062E-01	logistic	400789	1742	399047	0.336	0.038
276.13	Hyperpotassemia	endocrine/metabolic	rs76133235	0.087	0.085	1.091	3.069E-01	logistic	397299	1727	395572	0.354	0.038
556	Ulceration of the lower GI tract	digestive	rs76133235	-0.208	0.203	0.813	3.070E-01	logistic	334879	394	334485	0.187	0.039
604	Disorders of penis	genitourinary	rs76133235	0.157	0.154	1.170	3.082E-01	logistic	162266	488	161778	0.714	0.039
586	Other disorders of the kidney and ureters	genitourinary	rs76133235	-0.062	0.061	0.940	3.089E-01	logistic	384904	3842	381062	0.039	0.038
276.4	Acid-base balance disorder	endocrine/metabolic	rs76133235	0.078	0.077	1.081	3.102E-01	logistic	397692	2120	395572	0.239	0.038
722	Intervertebral disc disorders	musculoskeletal	rs76133235	-0.155	0.153	0.856	3.110E-01	logistic	409333	663	408670	0.423	0.038
8.5	Bacterial enteritis	infectious diseases	rs76133235	0.100	0.099	1.105	3.129E-01	logistic	418567	1251	417316	0.378	0.038
352.1	Trigeminal nerve disorders [CN5]	neurological	rs76133235	-0.181	0.180	0.834	3.133E-01	logistic	402912	497	402415	0.548	0.039
764	Sciatica	symptoms	rs76133235	0.098	0.097	1.103	3.143E-01	logistic	419302	1316	417986	0.312	0.038
470	Septal Deviations/Turbinate Hypertrophy	respiratory	rs76133235	0.087	0.087	1.091	3.158E-01	logistic	398163	1646	396517	0.438	0.038
411.4	Coronary atherosclerosis	circulatory system	rs76133235	-0.025	0.025	0.975	3.160E-01	logistic	394337	23787	370550	0.492	0.038
727.4	Ganglion and cyst of synovium, tendon, and bursa	musculoskeletal	rs76133235	-0.186	0.186	0.831	3.172E-01	logistic	389641	467	389174	0.794	0.039
286.7	Other and unspecified coagulation defects	hematopoietic	rs76133235	-0.253	0.254	0.776	3.182E-01	logistic	417818	265	417553	0.419	0.038
738	Other acquired musculoskeletal deformity	musculoskeletal	rs76133235	-0.106	0.106	0.900	3.205E-01	logistic	403982	1328	402654	0.633	0.038
523	Gingival and periodontal diseases	digestive	rs76133235	-0.201	0.203	0.818	3.230E-01	logistic	409931	395	409536	0.463	0.038
471	Nasal polyps	respiratory	rs76133235	-0.119	0.121	0.888	3.236E-01	logistic	397549	1032	396517	0.370	0.038
198.2	Secondary malignancy of respiratory organs	neoplasms	rs76133235	-0.055	0.055	0.947	3.238E-01	logistic	390707	4701	386006	0.115	0.038
529	Diseases and other conditions of the tongue	digestive	rs76133235	0.198	0.201	1.219	3.249E-01	logistic	417990	280	417710	0.405	0.038
275.3	Disorders of magnesium metabolism	endocrine/metabolic	rs76133235	0.089	0.090	1.093	3.259E-01	logistic	417729	1544	416185	0.350	0.038
530.9	Heartburn	digestive	rs76133235	-0.257	0.262	0.773	3.269E-01	logistic	357038	250	356788	0.351	0.039
619	Noninflammatory female genital disorders	genitourinary	rs76133235	0.074	0.076	1.077	3.300E-01	logistic	219175	2209	216966	0.604	0.038
227.1	Benign neoplasm of adrenal gland	neoplasms	rs76133235	0.203	0.209	1.225	3.305E-01	logistic	421144	257	420887	0.370	0.038
594.2	Calculus of lower urinary tract	genitourinary	rs76133235	0.139	0.143	1.149	3.308E-01	logistic	412949	573	412376	0.641	0.038
433.31	Transient cerebral ischemia	circulatory system	rs76133235	0.098	0.101	1.103	3.313E-01	logistic	403901	1216	402685	0.299	0.038
198.1	Secondary malignancy of lymph nodes	neoplasms	rs76133235	-0.041	0.042	0.960	3.328E-01	logistic	394015	8009	386006	0.159	0.038
255.2	Adrenal hypofunction	endocrine/metabolic	rs76133235	-0.208	0.217	0.812	3.371E-01	logistic	415595	349	415246	0.358	0.038
255.21	Glucocorticoid deficiency	endocrine/metabolic	rs76133235	-0.208	0.217	0.812	3.371E-01	logistic	415595	349	415246	0.358	0.038
427.8	Sinoatrial node dysfunction (Bradycardia)	circulatory system	rs76133235	0.136	0.143	1.146	3.409E-01	logistic	372427	579	371848	0.506	0.038
801.1	Fracture of foot	injuries & poisonings	rs76133235	-0.267	0.281	0.765	3.421E-01	logistic	396597	219	396378	0.467	0.038
377	Disorders of optic nerve and visual pathways	sense organs	rs76133235	0.169	0.178	1.184	3.428E-01	logistic	412293	365	411928	0.576	0.038
367.1	Myopia	sense organs	rs76133235	0.092	0.097	1.096	3.429E-01	logistic	412331	1325	411006	0.553	0.038
158	Neoplasm of unspecified nature of digestive system	neoplasms	rs76133235	-0.196	0.207	0.822	3.449E-01	logistic	394238	376	393862	0.352	0.038
401.1	Essential hypertension	circulatory system	rs76133235	-0.014	0.014	0.987	3.458E-01	logistic	389876	99213	290663	0.094	0.038
201	Hodgkin's disease	neoplasms	rs76133235	0.188	0.201	1.207	3.491E-01	logistic	415895	282	415613	0.444	0.038
750.2	Lower gastrointestinal congenital anomalies	congenital anomalies	rs76133235	0.219	0.235	1.245	3.514E-01	logistic	420453	200	420253	0.456	0.038
353	Nerve root and plexus disorders	neurological	rs76133235	-0.217	0.233	0.805	3.516E-01	logistic	402720	305	402415	0.528	0.039
728	Disorders of muscle, ligament, and fascia	musculoskeletal	rs76133235	0.194	0.209	1.214	3.524E-01	logistic	389432	258	389174	0.792	0.039
427.22	Atrial flutter	circulatory system	rs76133235	-0.141	0.153	0.868	3.563E-01	logistic	372499	651	371848	0.516	0.038
335	Multiple sclerosis	neurological	rs76133235	0.085	0.093	1.089	3.569E-01	logistic	400514	1467	399047	0.321	0.038
752	Nervous system congenital anomalies	congenital anomalies	rs76133235	0.149	0.162	1.160	3.586E-01	logistic	422992	453	422539	0.465	0.038
364.5	Corneal dystrophy	sense organs	rs76133235	-0.186	0.203	0.830	3.594E-01	logistic	398152	389	397763	0.481	0.038
290.1	Dementias	mental disorders	rs76133235	0.051	0.055	1.052	3.598E-01	logistic	409256	4283	404973	0.496	0.038
560.2	Impaction of intestine	digestive	rs76133235	-0.248	0.271	0.780	3.598E-01	logistic	334714	229	334485	0.194	0.039
571.8	Liver abscess and sequelae of chronic liver disease	digestive	rs76133235	-0.090	0.099	0.914	3.599E-01	logistic	401788	1514	400274	0.041	0.038
611	Abnormal findings on mammogram or breast exam	genitourinary	rs76133235	-0.232	0.254	0.793	3.611E-01	logistic	415016	264	414752	0.255	0.038
611.3	Lump or mass in breast	genitourinary	rs76133235	-0.232	0.254	0.793	3.611E-01	logistic	415016	264	414752	0.255	0.038
320	Meningitis	neurological	rs76133235	-0.212	0.233	0.809	3.625E-01	logistic	422374	303	422071	0.464	0.038
288.1	Decreased white blood cell count	hematopoietic	rs76133235	-0.061	0.067	0.941	3.640E-01	logistic	411502	3193	408309	0.154	0.038
288.11	Neutropenia	hematopoietic	rs76133235	-0.061	0.067	0.941	3.640E-01	logistic	411502	3193	408309	0.154	0.038
270.33	Amyloidosis	endocrine/metabolic	rs76133235	0.169	0.187	1.185	3.644E-01	logistic	422032	330	421702	0.453	0.038
117	Mycoses	infectious diseases	rs76133235	0.185	0.205	1.203	3.662E-01	logistic	418652	272	418380	0.390	0.038
8	Intestinal infection	infectious diseases	rs76133235	0.074	0.081	1.076	3.665E-01	logistic	419231	1915	417316	0.347	0.038

ST31; PheWAS results for the lead cis-pQTLs of the 4 proteins prioritized (ACOX1, FGF5, FURIN, and MST1).
SNP = Single Nucleotide Polymorphism; SE = Standard error HWE_p = P-value for Hardy-Weinberg equilibrium test

Phenotype	Description	Group	SNP	Beta	SE	Odds tatio	P-value	Type	n_total	n_cases	n_controls	HWE_p	allele_freq
433.12	Cerebral atherosclerosis	circulatory system	rs76133235	-0.195	0.217	0.823	3.682E-01	logistic	403029	344	402685	0.325	0.038
289.5	Diseases of spleen	hematopoietic	rs76133235	-0.127	0.141	0.881	3.682E-01	logistic	409070	761	408309	0.165	0.038
557.1	Celiac disease	digestive	rs76133235	-0.079	0.088	0.924	3.685E-01	logistic	336383	1898	334485	0.221	0.039
738.4	Acquired spondylolisthesis	musculoskeletal	rs76133235	-0.100	0.112	0.905	3.704E-01	logistic	403846	1192	402654	0.636	0.038
285.2	Anemia of chronic disease	hematopoietic	rs76133235	-0.124	0.138	0.883	3.709E-01	logistic	383237	788	382449	0.445	0.039
270.3	Disorders of plasma protein metabolism	endocrine/metabolic	rs76133235	-0.127	0.142	0.880	3.710E-01	logistic	422449	747	421702	0.448	0.038
411	Ischemic Heart Disease	circulatory system	rs76133235	-0.018	0.020	0.982	3.723E-01	logistic	410606	40056	370550	0.587	0.038
204.2	Myeloid leukemia	neoplasms	rs76133235	-0.126	0.142	0.882	3.771E-01	logistic	416360	747	415613	0.436	0.038
476	Allergic rhinitis	respiratory	rs76133235	-0.104	0.118	0.901	3.777E-01	logistic	397580	1063	396517	0.337	0.038
596	Other disorders of bladder	genitourinary	rs76133235	0.041	0.046	1.041	3.783E-01	logistic	407805	6251	401554	0.637	0.038
695.2	Bullous dermatoses	dermatologic	rs76133235	0.206	0.235	1.229	3.792E-01	logistic	405408	202	405206	0.445	0.038
575.7	Other disorders of gallbladder	digestive	rs76133235	-0.140	0.159	0.870	3.794E-01	logistic	395657	610	395047	0.322	0.038
560	Intestinal obstruction without mention of hernia	digestive	rs76133235	0.052	0.060	1.054	3.800E-01	logistic	338110	3625	334485	0.163	0.039
694	Dyschromia and Vitiligo	dermatologic	rs76133235	0.172	0.197	1.188	3.822E-01	logistic	405505	299	405206	0.440	0.038
851	Complications of transplants and reattached limbs	injuries & poisonings	rs76133235	-0.229	0.262	0.795	3.826E-01	logistic	416895	243	416652	0.449	0.038
695.3	Rosacea	dermatologic	rs76133235	0.185	0.213	1.203	3.854E-01	logistic	405456	250	405206	0.498	0.038
472	Chronic pharyngitis and nasopharyngitis	respiratory	rs76133235	0.181	0.209	1.199	3.857E-01	logistic	396779	262	396517	0.360	0.038
530.11	GERD	digestive	rs76133235	-0.027	0.031	0.973	3.864E-01	logistic	371656	14868	356788	0.444	0.039
429.2	Abnormal function study of cardiovascular system	circulatory system	rs76133235	0.161	0.187	1.175	3.877E-01	logistic	410036	333	409703	0.399	0.039
496.3	Bronchiectasis	respiratory	rs76133235	-0.053	0.062	0.948	3.879E-01	logistic	402578	3769	398809	0.354	0.038
352.2	Facial nerve disorders [CN7]	neurological	rs76133235	-0.129	0.150	0.879	3.912E-01	logistic	403092	677	402415	0.515	0.039
246	Other disorders of thyroid	endocrine/metabolic	rs76133235	-0.200	0.233	0.819	3.915E-01	logistic	392532	302	392230	0.380	0.038
359	Muscular dystrophies and other myopathies	neurological	rs76133235	0.175	0.205	1.191	3.926E-01	logistic	419442	274	419168	0.458	0.038
578.8	Hemorrhage of rectum and anus	digestive	rs76133235	-0.067	0.079	0.935	3.955E-01	logistic	385630	2327	383303	0.416	0.038
429	Ill-defined descriptions and complications of heart disease	circulatory system	rs76133235	0.158	0.187	1.171	3.969E-01	logistic	410037	334	409703	0.399	0.039
395.3	Nonrheumatic tricuspid valve disorders	circulatory system	rs76133235	0.185	0.218	1.203	3.970E-01	logistic	401653	239	401414	0.468	0.038
585.32	End stage renal disease	genitourinary	rs76133235	-0.107	0.126	0.898	3.970E-01	logistic	381994	932	381062	0.110	0.038
317.1	Alcoholism	mental disorders	rs76133235	0.050	0.059	1.051	3.972E-01	logistic	407082	3789	403293	0.344	0.038
272	Disorders of lipoid metabolism	endocrine/metabolic	rs76133235	-0.017	0.020	0.983	3.996E-01	logistic	394625	38860	355765	0.462	0.039
364	Corneal opacity and other disorders of cornea	sense organs	rs76133235	-0.077	0.091	0.926	4.002E-01	logistic	399513	1750	397763	0.485	0.038
530.3	Stricture and stenosis of esophagus	digestive	rs76133235	0.085	0.102	1.089	4.035E-01	logistic	357976	1188	356788	0.354	0.039
276.11	Hyperosmolality and/or hypernatremia	endocrine/metabolic	rs76133235	-0.121	0.145	0.886	4.040E-01	logistic	396286	714	395572	0.265	0.038
803.2	Fracture of radius and ulna	injuries & poisonings	rs76133235	0.063	0.076	1.065	4.056E-01	logistic	398618	2240	396378	0.423	0.038
366	Cataract	sense organs	rs76133235	0.018	0.021	1.018	4.073E-01	logistic	406937	35259	371678	0.473	0.038
585	Renal failure	genitourinary	rs76133235	-0.021	0.026	0.979	4.079E-01	logistic	403986	22924	381062	0.065	0.038
751.22	Other specified congenital anomalies of kidney	congenital anomalies	rs76133235	0.151	0.184	1.163	4.122E-01	logistic	420601	348	420253	0.449	0.038
687	Symptoms affecting skin	dermatologic	rs76133235	-0.170	0.207	0.844	4.131E-01	logistic	413083	367	412716	0.214	0.039
79.9	Viremia, NOS	infectious diseases	rs76133235	0.117	0.143	1.124	4.133E-01	logistic	415798	594	415204	0.366	0.038
274.1	Gout	endocrine/metabolic	rs76133235	-0.047	0.058	0.954	4.154E-01	logistic	420024	4234	415790	0.450	0.038
272.11	Hypercholesterolemia	endocrine/metabolic	rs76133235	-0.017	0.021	0.983	4.178E-01	logistic	390444	34679	355765	0.410	0.039
371.3	Inflammation of eyelids	sense organs	rs76133235	-0.122	0.150	0.886	4.180E-01	logistic	411465	672	410793	0.473	0.038
578.9	Hemorrhage of gastrointestinal tract	digestive	rs76133235	-0.056	0.070	0.945	4.186E-01	logistic	386245	2942	383303	0.418	0.038
202.21	Nodular lymphoma	neoplasms	rs76133235	-0.146	0.183	0.864	4.233E-01	logistic	416077	464	415613	0.418	0.038
573.3	Hepatomegaly	digestive	rs76133235	0.164	0.205	1.178	4.235E-01	logistic	400551	277	400274	0.052	0.038
172.1	Melanomas of skin, dx or hx	neoplasms	rs76133235	0.066	0.084	1.069	4.260E-01	logistic	409038	1830	407208	0.523	0.038
172.11	Melanomas of skin	neoplasms	rs76133235	0.066	0.084	1.069	4.260E-01	logistic	409038	1830	407208	0.523	0.038
736	Other acquired deformities of limbs	musculoskeletal	rs76133235	0.091	0.114	1.095	4.273E-01	logistic	403605	951	402654	0.695	0.038
81	Infection/inflammation of internal prosthetic device; implant; and graft	infectious diseases	rs76133235	0.064	0.081	1.066	4.297E-01	logistic	414783	1939	412844	0.391	0.038
735	Acquired foot deformities	musculoskeletal	rs76133235	0.037	0.047	1.038	4.308E-01	logistic	408734	6080	402654	0.631	0.038
619.3	Noninflammatory disorders of cervix	genitourinary	rs76133235	0.147	0.187	1.159	4.309E-01	logistic	217308	342	216966	0.571	0.038
276.41	Acidosis	endocrine/metabolic	rs76133235	0.066	0.084	1.068	4.335E-01	logistic	397376	1804	395572	0.226	0.038
510	Other diseases of lung	respiratory	rs76133235	0.074	0.094	1.076	4.342E-01	logistic	421711	1427	420284	0.454	0.038
362.2	Degeneration of macula and posterior pole of retina	sense organs	rs76133235	-0.031	0.040	0.969	4.348E-01	logistic	406927	9170	397757	0.380	0.038
395.2	Nonrheumatic aortic valve disorders	circulatory system	rs76133235	-0.042	0.054	0.959	4.355E-01	logistic	406373	4959	401414	0.511	0.038
626	Disorders of menstruation and other abnormal bleeding from female genital tract	genitourinary	rs76133235	0.037	0.048	1.038	4.356E-01	logistic	198823	6076	192747	0.576	0.038
571	Chronic liver disease and cirrhosis	digestive	rs76133235	-0.038	0.049	0.962	4.369E-01	logistic	406135	5861	400274	0.137	0.038
741.4	Joint effusions	musculoskeletal	rs76133235	-0.115	0.148	0.891	4.385E-01	logistic	415693	683	415010	0.416	0.038
198.4	Secondary malignant neoplasm of liver	neoplasms	rs76133235	-0.039	0.051	0.962	4.426E-01	logistic	391482	5476	386006	0.273	0.038
270	Disorders of protein plasma/amino-acid transport and metabolism	endocrine/metabolic	rs76133235	-0.105	0.137	0.901	4.461E-01	logistic	422490	788	421702	0.445	0.038
689	Disorder of skin and subcutaneous tissue NOS	dermatologic	rs76133235	0.059	0.078	1.061	4.473E-01	logistic	413188	2098	411090	0.305	0.038
628	Ovarian cyst	genitourinary	rs76133235	-0.085	0.112	0.918	4.483E-01	logistic	193944	1197	192747	0.657	0.038
416	Cardiomegaly	circulatory system	rs76133235	0.041	0.055	1.042	4.490E-01	logistic	417255	4417	412838	0.359	0.038
272.1	Hyperlipidemia	endocrine/metabolic	rs76133235	-0.015	0.020	0.985	4.494E-01	logistic	394381	38616	355765	0.467	0.039
41.4	E. coli	infectious diseases	rs76133235	0.047	0.063	1.048	4.513E-01	logistic	399991	3344	396647	0.067	0.038
159.2	Malignant neoplasm of small intestine, including duodenum	neoplasms	rs76133235	0.160	0.213	1.174	4.519E-01	logistic	394117	255	393862	0.348	0.038
695.4	Lupus (localized and systemic)	dermatologic	rs76133235	0.119	0.158	1.126	4.527E-01	logistic	403975	493	403482	0.493	0.038
512	Other symptoms of respiratory system	respiratory	rs76133235	-0.035	0.047	0.966	4.530E-01	logistic	411090	6491	404599	0.310	0.038
155	Cancer of liver and intrahepatic bile duct	neoplasms	rs76133235	0.096	0.129	1.101	4.551E-01	logistic	394604	742	393862	0.472	0.038
214	Lipoma	neoplasms	rs76133235	-0.085	0.115	0.918	4.562E-01	logistic	415339	1112	414227	0.540	0.038
225.1	Benign neoplasm of brain, cranial nerves, meninges	neoplasms	rs76133235	-0.101	0.136	0.904	4.569E-01	logistic	421204	807	420397	0.417	0.038
378.2	Nystagmus and other irregular eye movements	sense organs	rs76133235	0.174	0.235	1.191	4.572E-01	logistic	412137	209	411928	0.559	0.038

ST31; PheWAS results for the lead cis-pQTLs of the 4 proteins prioritized (ACOX1, FGF5, FURIN, and MST1).

SNP = Single Nucleotide Polymorphism; SE = Standard error HWE_p = P-value for Hardy-Weinberg equilibrium test

Phenotype	Description	Group	SNP	Beta	SE	Odds tatio	P-value	Type	n_total	n_cases	n_controls	HWE_p	allele_freq
608	Other disorders of male genital organs	genitourinary	rs76133235	0.095	0.128	1.099	4.597E-01	logistic	162533	755	161778	0.702	0.039
958	Certain early complications of trauma or procedure	injuries & poisonings	rs76133235	0.150	0.204	1.162	4.633E-01	logistic	423583	281	423302	0.444	0.038
465	Acute upper respiratory infections of multiple or unspecified sites	respiratory	rs76133235	-0.108	0.147	0.898	4.643E-01	logistic	420386	694	419692	0.348	0.038
780	Hypothermia/Chills	symptoms	rs76133235	0.166	0.229	1.181	4.669E-01	logistic	423421	221	423200	0.465	0.038
174.11	Malignant neoplasm of female breast	neoplasms	rs76133235	0.028	0.039	1.029	4.675E-01	logistic	212959	9200	203759	0.863	0.038
286.12	Congenital deficiency of other clotting factors (including factor VII	hematopoietic	rs76133235	-0.190	0.262	0.827	4.690E-01	logistic	417788	235	417553	0.419	0.038
227.3	Benign neoplasm of pituitary gland and craniopharyngeal duct (pouch	neoplasms	rs76133235	-0.150	0.208	0.861	4.695E-01	logistic	421248	361	420887	0.404	0.038
276.42	Alkalosis	endocrine/metabolic	rs76133235	0.142	0.197	1.153	4.698E-01	logistic	395878	306	395572	0.273	0.038
803	Fracture of upper limb	injuries & poisonings	rs76133235	0.040	0.056	1.041	4.703E-01	logistic	400593	4215	396378	0.451	0.038
803.3	Fracture of clavicle or scapula	injuries & poisonings	rs76133235	0.095	0.132	1.100	4.708E-01	logistic	397087	709	396378	0.465	0.038
446.4	Wegener's granulomatosis	circulatory system	rs76133235	-0.189	0.262	0.828	4.716E-01	logistic	405703	234	405469	0.385	0.038
358	Myoneural disorders	neurological	rs76133235	-0.158	0.222	0.854	4.769E-01	logistic	419485	317	419168	0.464	0.038
726.1	Enthesopathy	musculoskeletal	rs76133235	-0.046	0.065	0.955	4.781E-01	logistic	392491	3317	389174	0.776	0.039
250	Diabetes mellitus	endocrine/metabolic	rs76133235	-0.016	0.023	0.984	4.796E-01	logistic	414239	27602	386637	0.454	0.038
560.3	Peritoneal or intestinal adhesions	digestive	rs76133235	0.086	0.122	1.090	4.809E-01	logistic	335317	832	334485	0.148	0.039
274	Gout and other crystal arthropathies	endocrine/metabolic	rs76133235	-0.040	0.056	0.961	4.815E-01	logistic	420276	4486	415790	0.465	0.038
586.4	Stricture/obstruction of ureter	genitourinary	rs76133235	0.086	0.122	1.090	4.822E-01	logistic	381896	834	381062	0.156	0.039
735.23	Hallux rigidus	musculoskeletal	rs76133235	-0.156	0.222	0.856	4.830E-01	logistic	402973	319	402654	0.661	0.038
289.8	Polycythemia, secondary	hematopoietic	rs76133235	-0.126	0.180	0.882	4.846E-01	logistic	407605	466	407139	0.029	0.038
577.1	Acute pancreatitis	digestive	rs76133235	-0.061	0.088	0.941	4.867E-01	logistic	419858	1850	418008	0.343	0.038
327.3	Sleep apnea	neurological	rs76133235	-0.036	0.051	0.965	4.869E-01	logistic	418773	5389	413384	0.367	0.038
216	Benign neoplasm of skin	neoplasms	rs76133235	0.074	0.107	1.077	4.883E-01	logistic	415074	1116	413958	0.415	0.038
516.1	Hemoptysis	respiratory	rs76133235	-0.087	0.125	0.917	4.885E-01	logistic	421320	931	420389	0.489	0.038
967	Adverse effects of sedatives or other central nervous system depressants and anesthetic	injuries & poisonings	rs76133235	0.158	0.229	1.172	4.886E-01	logistic	367398	225	367173	0.345	0.038
426	Cardiac conduction disorders	circulatory system	rs76133235	-0.025	0.037	0.975	4.891E-01	logistic	382540	10692	371848	0.669	0.038
261.4	Vitamin D deficiency	endocrine/metabolic	rs76133235	0.053	0.076	1.054	4.891E-01	logistic	414357	2260	412097	0.296	0.038
636	Early or threatened labor; hemorrhage in early pregnancy	pregnancy complications	rs76133235	0.107	0.155	1.112	4.919E-01	logistic	223122	530	222592	0.506	0.038
149.1	Cancer of oropharynx	neoplasms	rs76133235	-0.137	0.199	0.872	4.933E-01	logistic	420725	383	420342	0.509	0.038
634	Miscarriage; stillbirth	pregnancy complications	rs76133235	-0.101	0.148	0.904	4.937E-01	logistic	223299	707	222592	0.427	0.038
573.7	Abnormal results of function study of liver	digestive	rs76133235	0.040	0.059	1.041	4.957E-01	logistic	404090	3816	400274	0.068	0.038
580.14	Chronic glomerulonephritis, NOS	genitourinary	rs76133235	-0.151	0.222	0.860	4.960E-01	logistic	381376	314	381062	0.186	0.039
180.1	Cervical cancer	neoplasms	rs76133235	-0.058	0.085	0.944	4.964E-01	logistic	197916	2012	195904	0.761	0.038
625	Pain and other symptoms associated with female genital organs	genitourinary	rs76133235	-0.158	0.234	0.853	4.976E-01	logistic	218575	291	218284	0.439	0.038
577.2	Chronic pancreatitis	digestive	rs76133235	0.097	0.143	1.101	4.993E-01	logistic	418611	603	418008	0.354	0.038
592.12	Chronic cystitis	genitourinary	rs76133235	0.147	0.218	1.158	5.012E-01	logistic	377176	249	376927	0.033	0.038
292.3	Memory loss	mental disorders	rs76133235	0.105	0.156	1.110	5.019E-01	logistic	405478	505	404973	0.358	0.038
255.1	Adrenal hyperfunction	endocrine/metabolic	rs76133235	0.161	0.241	1.175	5.037E-01	logistic	415447	201	415246	0.326	0.038
261.2	Vitamin B-complex deficiencies	endocrine/metabolic	rs76133235	0.058	0.087	1.059	5.059E-01	logistic	413809	1712	412097	0.383	0.038
456	Chronic venous insufficiency [CVI]	circulatory system	rs76133235	0.135	0.204	1.144	5.104E-01	logistic	381250	284	380966	0.023	0.039
427	Cardiac dysrhythmias	circulatory system	rs76133235	-0.016	0.024	0.985	5.155E-01	logistic	398369	26521	371848	0.332	0.038
217.1	Nevus, non-neoplastic	neoplasms	rs76133235	0.136	0.209	1.145	5.156E-01	logistic	414233	275	413958	0.426	0.038
687.4	Disturbance of skin sensation	dermatologic	rs76133235	-0.065	0.101	0.937	5.188E-01	logistic	414113	1397	412716	0.232	0.039
798.1	Chronic fatigue syndrome	symptoms	rs76133235	-0.100	0.155	0.905	5.193E-01	logistic	414937	617	414320	0.354	0.038
702	Degenerative skin conditions and other dermatoses	dermatologic	rs76133235	0.053	0.083	1.054	5.236E-01	logistic	407215	1867	405348	0.541	0.038
573.9	Abnormal serum enzyme levels	digestive	rs76133235	-0.172	0.272	0.842	5.267E-01	logistic	400489	215	400274	0.005	0.038
531.4	Peptic ulcer, site unspecified	digestive	rs76133235	0.144	0.228	1.155	5.280E-01	logistic	411565	226	411339	0.449	0.038
260.6	Anorexia	endocrine/metabolic	rs76133235	0.076	0.120	1.079	5.281E-01	logistic	412968	871	412097	0.363	0.038
189.1	Cancer of kidney and renal pelvis	neoplasms	rs76133235	-0.069	0.109	0.934	5.293E-01	logistic	418392	1205	417187	0.381	0.038
580.32	Nephritis and nephropathy with pathological lesion	genitourinary	rs76133235	0.131	0.209	1.140	5.303E-01	logistic	381336	274	381062	0.158	0.039
227	Benign neoplasm of other endocrine glands and related structures	neoplasms	rs76133235	0.083	0.133	1.087	5.325E-01	logistic	421596	709	420887	0.409	0.038
519.8	Other diseases of respiratory system, NEC	respiratory	rs76133235	-0.025	0.040	0.975	5.345E-01	logistic	411807	8677	403130	0.524	0.038
717	Polymyalgia Rheumatica	musculoskeletal	rs76133235	-0.047	0.076	0.954	5.350E-01	logistic	422397	2442	419955	0.468	0.038
575.9	Nonspecific abnormal findings on radiological and other examination of biliary trac	digestive	rs76133235	-0.162	0.262	0.850	5.367E-01	logistic	395275	228	395047	0.335	0.038
790	Nonspecific findings on examination of blood	symptoms	rs76133235	-0.151	0.247	0.860	5.404E-01	logistic	422095	255	421840	0.325	0.038
686	Other local infections of skin and subcutaneous tissue	dermatologic	rs76133235	0.067	0.110	1.069	5.409E-01	logistic	419554	1057	418497	0.412	0.038
316	Substance addiction and disorders	mental disorders	rs76133235	-0.127	0.208	0.881	5.412E-01	logistic	403645	352	403293	0.340	0.038
198.3	Secondary malignant neoplasm of digestive systems	neoplasms	rs76133235	0.042	0.069	1.043	5.426E-01	logistic	388741	2735	386006	0.180	0.039
317	Alcohol-related disorders	mental disorders	rs76133235	0.031	0.051	1.032	5.427E-01	logistic	408346	5053	403293	0.350	0.038
752.11	Spina bifida	congenital anomalies	rs76133235	0.147	0.241	1.158	5.427E-01	logistic	422743	204	422539	0.478	0.038
444.1	Arterial embolism and thrombosis of lower extremity artery	circulatory system	rs76133235	-0.111	0.183	0.895	5.439E-01	logistic	405915	446	405469	0.435	0.038
374.3	Ptosis of eyelid	sense organs	rs76133235	0.062	0.103	1.064	5.453E-01	logistic	411997	1204	410793	0.467	0.038
586.1	Anatomical abnormatilies of kidney and ureters	genitourinary	rs76133235	0.134	0.223	1.144	5.471E-01	logistic	381301	239	381062	0.190	0.039
618	Genital prolapse	genitourinary	rs76133235	0.028	0.047	1.029	5.489E-01	logistic	219680	6225	213455	0.444	0.038
350.3	Lack of coordination	neurological	rs76133235	0.122	0.204	1.130	5.489E-01	logistic	420499	289	420210	0.344	0.038
571.5	Other chronic nonalcoholic liver disease	digestive	rs76133235	-0.034	0.057	0.966	5.505E-01	logistic	404607	4333	400274	0.004	0.038
481	Influenza	respiratory	rs76133235	0.097	0.164	1.102	5.525E-01	logistic	397264	464	396800	0.309	0.038
701.2	Scar conditions and fibrosis of skin	dermatologic	rs76133235	0.105	0.178	1.111	5.534E-01	logistic	415576	390	415186	0.572	0.038
443	Peripheral vascular disease	circulatory system	rs76133235	-0.031	0.052	0.969	5.540E-01	logistic	410595	5126	405469	0.354	0.038
585.33	Chronic Kidney Disease, Stage III	genitourinary	rs76133235	-0.026	0.044	0.974	5.547E-01	logistic	388537	7475	381062	0.293	0.038
715	Other inflammatory spondylopathies	musculoskeletal	rs76133235	-0.062	0.105	0.940	5.557E-01	logistic	409805	1298	408507	0.598	0.038
420.2	Pericarditis	circulatory system	rs76133235	-0.058	0.099	0.944	5.572E-01	logistic	418236	1466	416770	0.428	0.038

ST31; PheWAS results for the lead cis-pQTLs of the 4 proteins prioritized (ACOX1, FGF5, FURIN, and MST1).
SNP = Single Nucleotide Polymorphism; SE = Standard error HWE_p = P-value for Hardy-Weinberg equilibrium test

Phenotype	Description	Group	SNP	Beta	SE	Odds tatio	P-value	Type	n_total	n_cases	n_controls	HWE_p	allele_freq
560.1	Paralytic ileus	digestive	rs76133235	0.103	0.175	1.108	5.572E-01	logistic	334880	395	334485	0.211	0.039
575.1	Cholangitis	digestive	rs76133235	0.070	0.120	1.073	5.577E-01	logistic	395932	885	395047	0.388	0.038
750.1	Upper gastrointestinal congenital anomalies	congenital anomalies	rs76133235	-0.159	0.272	0.853	5.578E-01	logistic	420464	211	420253	0.434	0.038
151	Cancer of stomach	neoplasms	rs76133235	0.066	0.114	1.069	5.605E-01	logistic	394829	967	393862	0.404	0.038
574.2	Calculus of bile duct	digestive	rs76133235	-0.147	0.254	0.863	5.634E-01	logistic	395287	240	395047	0.335	0.038
519	Other diseases of respiratory system, not elsewhere classified	respiratory	rs76133235	-0.023	0.040	0.977	5.649E-01	logistic	411962	8832	403130	0.517	0.038
585.34	Chronic Kidney Disease, Stage IV	genitourinary	rs76133235	-0.064	0.111	0.938	5.655E-01	logistic	382213	1151	381062	0.089	0.039
184.2	Cancer of other female genital organs (excluding uterus and ovary	neoplasms	rs76133235	-0.146	0.255	0.864	5.656E-01	logistic	205080	243	204837	0.716	0.038
550.4	Umbilical hernia	digestive	rs76133235	-0.057	0.100	0.944	5.661E-01	logistic	366811	1417	365394	0.149	0.039
747.11	Cardiac shunt/ heart septal defect	congenital anomalies	rs76133235	-0.105	0.183	0.900	5.661E-01	logistic	422041	446	421595	0.327	0.038
585.31	Renal dialysis	genitourinary	rs76133235	-0.081	0.143	0.922	5.709E-01	logistic	381775	713	381062	0.102	0.039
252.2	Hypoparathyroidism	endocrine/metabolic	rs76133235	-0.149	0.263	0.862	5.710E-01	logistic	415471	225	415246	0.361	0.038
260	Protein-calorie malnutrition	endocrine/metabolic	rs76133235	-0.112	0.199	0.894	5.746E-01	logistic	412473	376	412097	0.364	0.038
540.1	Appendicitis	digestive	rs76133235	0.108	0.193	1.114	5.766E-01	logistic	421984	328	421656	0.508	0.038
577.3	Cyst and pseudocyst of pancreas	digestive	rs76133235	0.087	0.156	1.091	5.776E-01	logistic	418522	514	418008	0.446	0.038
196	Radiotherapy	neoplasms	rs76133235	-0.053	0.095	0.949	5.786E-01	logistic	387589	1583	386006	0.052	0.038
149.4	Cancer of larynx	neoplasms	rs76133235	-0.128	0.233	0.880	5.820E-01	logistic	420618	276	420342	0.538	0.038
365	Glaucoma	sense organs	rs76133235	0.023	0.043	1.024	5.821E-01	logistic	405209	7446	397763	0.526	0.039
707.2	Chronic ulcer of leg or foot	dermatologic	rs76133235	0.043	0.079	1.044	5.835E-01	logistic	418232	2102	416130	0.173	0.038
293	Symptoms involving head and neck	mental disorders	rs76133235	0.089	0.164	1.093	5.848E-01	logistic	420175	464	419711	0.380	0.038
555.2	Ulcerative colitis	digestive	rs76133235	0.035	0.064	1.035	5.865E-01	logistic	337679	3194	334485	0.196	0.039
721.1	Spondylosis without myelopathy	musculoskeletal	rs76133235	-0.053	0.097	0.948	5.866E-01	logistic	410174	1504	408670	0.498	0.038
110	Dermatophytosis / Dermatomycosis	infectious diseases	rs76133235	-0.138	0.254	0.871	5.869E-01	logistic	418617	237	418380	0.427	0.038
512.7	Shortness of breath	respiratory	rs76133235	-0.032	0.060	0.968	5.878E-01	logistic	408527	3928	404599	0.265	0.038
687.1	Rash and other nonspecific skin eruption	dermatologic	rs76133235	-0.062	0.115	0.940	5.888E-01	logistic	413803	1087	412716	0.244	0.039
8.6	Viral Enteritis	infectious diseases	rs76133235	0.084	0.158	1.088	5.925E-01	logistic	417821	505	417316	0.359	0.038
599.2	Retention of urine	genitourinary	rs76133235	-0.024	0.045	0.976	5.932E-01	logistic	396988	6882	390106	0.491	0.038
540.11	Acute appendicitis	digestive	rs76133235	0.125	0.234	1.133	5.932E-01	logistic	421875	219	421656	0.513	0.038
752.1	Neural tube defects	congenital anomalies	rs76133235	0.094	0.178	1.098	5.980E-01	logistic	422933	394	422539	0.470	0.038
41	Bacterial infection NOS	infectious diseases	rs76133235	-0.020	0.039	0.980	6.003E-01	logistic	406056	9409	396647	0.297	0.038
145.2	Cancer of tongue	neoplasms	rs76133235	0.093	0.178	1.097	6.027E-01	logistic	420733	391	420342	0.503	0.038
747	Cardiac and circulatory congenital anomalies	congenital anomalies	rs76133235	0.056	0.108	1.058	6.056E-01	logistic	422691	1096	421595	0.349	0.038
250.2	Type 2 diabetes	endocrine/metabolic	rs76133235	-0.012	0.024	0.988	6.056E-01	logistic	413206	26569	386637	0.486	0.038
250.4	Abnormal glucose	endocrine/metabolic	rs76133235	-0.061	0.118	0.941	6.057E-01	logistic	387669	1032	386637	0.363	0.038
364.4	Corneal degenerations	sense organs	rs76133235	-0.057	0.111	0.945	6.114E-01	logistic	398917	1154	397763	0.504	0.038
800.3	Fracture of tibia and fibula	injuries & poisonings	rs76133235	-0.081	0.159	0.923	6.121E-01	logistic	396955	577	396378	0.452	0.038
318	Tobacco use disorder	mental disorders	rs76133235	0.026	0.052	1.026	6.127E-01	logistic	408298	5005	403293	0.263	0.038
225	Benign neoplasm of brain and other parts of nervous system	neoplasms	rs76133235	-0.066	0.132	0.936	6.140E-01	logistic	421233	836	420397	0.414	0.038
788	Syncope and collapse	symptoms	rs76133235	0.023	0.046	1.024	6.150E-01	logistic	412052	6241	405811	0.448	0.038
418.1	Precordial pain	circulatory system	rs76133235	0.042	0.084	1.043	6.154E-01	logistic	417539	1859	415680	0.299	0.038
506	Empyema and pneumothorax	respiratory	rs76133235	-0.055	0.110	0.946	6.170E-01	logistic	392231	1174	391057	0.285	0.038
947	Urticaria	dermatologic	rs76133235	0.120	0.241	1.128	6.178E-01	logistic	415196	209	414987	0.515	0.038
594.3	Calculus of ureter	genitourinary	rs76133235	-0.046	0.093	0.955	6.183E-01	logistic	414010	1634	412376	0.615	0.038
585.2	Renal failure NOS	genitourinary	rs76133235	-0.069	0.139	0.933	6.193E-01	logistic	381806	744	381062	0.173	0.039
285.22	Anemia in neoplastic disease	hematopoietic	rs76133235	0.096	0.193	1.100	6.200E-01	logistic	382780	331	382449	0.395	0.039
250.42	Other abnormal glucose	endocrine/metabolic	rs76133235	-0.110	0.222	0.896	6.210E-01	logistic	386940	303	386637	0.363	0.038
600	Hyperplasia of prostate	genitourinary	rs76133235	0.017	0.034	1.017	6.220E-01	logistic	169018	12557	156461	0.857	0.039
701.3	Circumscribed scleroderma	dermatologic	rs76133235	-0.129	0.263	0.879	6.230E-01	logistic	415410	224	415186	0.583	0.038
296	Mood disorders	mental disorders	rs76133235	-0.011	0.023	0.989	6.235E-01	logistic	409186	27564	381622	0.494	0.038
450	Noninfectious disorders of lymphatic channels	circulatory system	rs76133235	-0.054	0.111	0.947	6.243E-01	logistic	422720	1167	421553	0.385	0.038
78	Viral warts & HPV	infectious diseases	rs76133235	0.115	0.235	1.122	6.243E-01	logistic	415426	222	415204	0.387	0.038
174	Breast cancer	neoplasms	rs76133235	0.018	0.037	1.018	6.252E-01	logistic	401305	10248	391057	0.117	0.038
580.3	Nephritis and nephropathy without mention of glomerulonephritis	genitourinary	rs76133235	0.100	0.204	1.105	6.261E-01	logistic	381356	294	381062	0.158	0.039
479	Other upper respiratory disease	respiratory	rs76133235	0.084	0.173	1.087	6.272E-01	logistic	396937	420	396517	0.384	0.038
696	Psoriasis and related disorders	dermatologic	rs76133235	-0.034	0.069	0.967	6.273E-01	logistic	402206	2916	399290	0.428	0.038
385.3	Cholesteatoma	sense organs	rs76133235	0.114	0.234	1.120	6.279E-01	logistic	418937	221	418716	0.466	0.038
441	Vascular insufficiency of intestine	circulatory system	rs76133235	-0.070	0.145	0.932	6.282E-01	logistic	406151	682	405469	0.395	0.038
707.1	Decubitus ulcer	dermatologic	rs76133235	0.032	0.066	1.033	6.286E-01	logistic	419126	2996	416130	0.351	0.038
626.1	Irregular menstrual cycle/bleeding	genitourinary	rs76133235	0.028	0.058	1.028	6.286E-01	logistic	196900	4153	192747	0.541	0.038
599.3	Dysuria	genitourinary	rs76133235	0.093	0.193	1.098	6.297E-01	logistic	390438	332	390106	0.417	0.038
411.1	Unstable angina (intermediate coronary syndrome	circulatory system	rs76133235	-0.029	0.061	0.971	6.306E-01	logistic	374269	3719	370550	0.582	0.038
401.2	Hypertensive heart and/or renal disease	circulatory system	rs76133235	-0.053	0.111	0.948	6.312E-01	logistic	291807	1144	290663	0.301	0.039
348	Other conditions of brain	neurological	rs76133235	0.066	0.138	1.068	6.312E-01	logistic	399717	670	399047	0.334	0.038
395.6	Heart valve replaced	circulatory system	rs76133235	-0.036	0.075	0.965	6.321E-01	logistic	403924	2510	401414	0.494	0.038
535.6	Duodenitis	digestive	rs76133235	0.041	0.086	1.042	6.331E-01	logistic	363993	1779	362214	0.088	0.038
555	Inflammatory bowel disease and other gastroenteritis and coliti	digestive	rs76133235	0.025	0.053	1.026	6.335E-01	logistic	339234	4749	334485	0.146	0.039
333.1	Essential tremor	neurological	rs76133235	0.073	0.154	1.076	6.355E-01	logistic	399579	532	399047	0.309	0.038
565.1	Anal and rectal polyp	digestive	rs76133235	-0.034	0.072	0.967	6.365E-01	logistic	386554	2715	383839	0.369	0.038
573.5	Jaundice (not of newborn)	digestive	rs76133235	0.064	0.135	1.066	6.373E-01	logistic	400970	696	400274	0.031	0.038
696.4	Psoriasis	dermatologic	rs76133235	-0.033	0.070	0.968	6.374E-01	logistic	402177	2887	399290	0.429	0.038
255	Disorders of adrenal glands	endocrine/metabolic	rs76133235	-0.064	0.135	0.938	6.376E-01	logistic	416034	788	415246	0.337	0.038

ST31; PheWAS results for the lead cis-pQTLs of the 4 proteins prioritized (ACOX1, FGF5, FURIN, and MST1).

SNP = Single Nucleotide Polymorphism; SE = Standard error HWE_p = P-value for Hardy-Weinberg equilibrium test

Phenotype	Description	Group	SNP	Beta	SE	Odds tatio	P-value	Type	n_total	n_cases	n_controls	HWE_p	allele_freq
174.1	Breast cancer [female]	neoplasms	rs76133235	0.018	0.038	1.018	6.377E-01	logistic	213896	10137	203759	0.865	0.038
291.8	Alteration of consciousness	mental disorders	rs76133235	-0.094	0.200	0.910	6.379E-01	logistic	405343	370	404973	0.341	0.038
735.2	Acquired toe deformities	musculoskeletal	rs76133235	-0.040	0.086	0.961	6.399E-01	logistic	404574	1920	402654	0.654	0.038
117.4	Aspergillosis	infectious diseases	rs76133235	0.113	0.241	1.119	6.400E-01	logistic	418590	210	418380	0.395	0.038
715.2	Ankylosing spondylitis	musculoskeletal	rs76133235	-0.084	0.180	0.919	6.407E-01	logistic	408956	449	408507	0.625	0.038
721	Spondylosis and allied disorders	musculoskeletal	rs76133235	-0.040	0.086	0.961	6.422E-01	logistic	410559	1889	408670	0.509	0.038
574.12	Cholelithiasis with other cholecystitis	digestive	rs76133235	-0.044	0.094	0.957	6.425E-01	logistic	396636	1589	395047	0.306	0.038
522	Diseases of pulp and periapical tissues	digestive	rs76133235	0.095	0.204	1.099	6.437E-01	logistic	409832	296	409536	0.431	0.039
742	Derangement of joint, non-traumatic	musculoskeletal	rs76133235	0.079	0.170	1.082	6.444E-01	logistic	415444	434	415010	0.418	0.038
454.11	Varicose veins of lower extremity, symptomtic	circulatory system	rs76133235	0.067	0.146	1.069	6.468E-01	logistic	381560	594	380966	0.007	0.039
401.22	Hypertensive chronic kidney disease	circulatory system	rs76133235	-0.055	0.121	0.946	6.475E-01	logistic	291632	969	290663	0.310	0.039
381	Otitis media and Eustachian tube disorders	sense organs	rs76133235	-0.059	0.129	0.943	6.479E-01	logistic	419568	852	418716	0.474	0.038
527	Diseases of the salivary glands	digestive	rs76133235	0.107	0.234	1.113	6.480E-01	logistic	417933	223	417710	0.439	0.038
375.2	Epiphora	sense organs	rs76133235	-0.097	0.212	0.908	6.482E-01	logistic	412257	329	411928	0.560	0.038
701	Other hypertrophic and atrophic conditions of skin	dermatologic	rs76133235	0.048	0.106	1.049	6.498E-01	logistic	416347	1161	415186	0.568	0.038
750	Digestive congenital anomalies	congenital anomalies	rs76133235	0.078	0.173	1.081	6.516E-01	logistic	420674	421	420253	0.448	0.038
427.11	Paroxysmal supraventricular tachycardia	circulatory system	rs76133235	-0.037	0.082	0.964	6.532E-01	logistic	373959	2111	371848	0.510	0.038
276.14	Hypopotassemia	endocrine/metabolic	rs76133235	0.028	0.062	1.028	6.532E-01	logistic	399074	3502	395572	0.212	0.038
395	Heart valve disorders	circulatory system	rs76133235	-0.017	0.038	0.983	6.536E-01	logistic	411117	9703	401414	0.555	0.038
477	Epistaxis or throat hemorrhage	respiratory	rs76133235	-0.047	0.105	0.954	6.553E-01	logistic	397807	1290	396517	0.353	0.038
580.2	Nephrotic syndrome without mention of glomerulonephritis	genitourinary	rs76133235	0.079	0.178	1.082	6.560E-01	logistic	381457	395	381062	0.197	0.039
378.5	Paralytic strabismus	sense organs	rs76133235	-0.103	0.233	0.902	6.580E-01	logistic	412201	273	411928	0.562	0.038
994	Sepsis and SIRS	injuries & poisonings	rs76133235	0.020	0.045	1.020	6.600E-01	logistic	418535	6755	411780	0.424	0.038
994.2	Sepsis	injuries & poisonings	rs76133235	0.020	0.045	1.020	6.600E-01	logistic	418535	6755	411780	0.424	0.038
446.9	Arteritis NOS	circulatory system	rs76133235	0.081	0.186	1.085	6.627E-01	logistic	405830	361	405469	0.373	0.038
580	Nephritis; nephrosis; renal sclerosis	genitourinary	rs76133235	0.047	0.110	1.049	6.651E-01	logistic	382137	1075	381062	0.200	0.039
211	Benign neoplasm of other parts of digestive system	neoplasms	rs76133235	-0.027	0.064	0.973	6.689E-01	logistic	400517	3467	397050	0.224	0.038
362.31	Separation of retinal layers	sense organs	rs76133235	-0.072	0.167	0.931	6.692E-01	logistic	398271	514	397757	0.473	0.038
157	Pancreatic cancer	neoplasms	rs76133235	0.041	0.097	1.042	6.692E-01	logistic	395256	1394	393862	0.416	0.038
379	Other disorders of eye	sense organs	rs76133235	-0.050	0.117	0.951	6.704E-01	logistic	412963	1035	411928	0.534	0.038
516	Abnormal sputum	respiratory	rs76133235	-0.051	0.120	0.950	6.705E-01	logistic	421370	981	420389	0.484	0.038
536.8	Dyspepsia and other specified disorders of function of stomach	digestive	rs76133235	-0.039	0.093	0.961	6.717E-01	logistic	363851	1637	362214	0.101	0.038
252	Disorders of parathyroid gland	endocrine/metabolic	rs76133235	-0.045	0.107	0.956	6.728E-01	logistic	416485	1239	415246	0.317	0.038
442.11	Abdominal aortic aneurysm	circulatory system	rs76133235	0.037	0.089	1.038	6.729E-01	logistic	407127	1658	405469	0.429	0.038
289.4	Lymphadenitis	hematopoietic	rs76133235	-0.038	0.091	0.963	6.752E-01	logistic	410013	1704	408309	0.082	0.038
580.1	Glomerulonephritis	genitourinary	rs76133235	-0.070	0.168	0.933	6.777E-01	logistic	381574	512	381062	0.197	0.039
961	Poisoning by other anti-infectives	injuries & poisonings	rs76133235	0.087	0.209	1.090	6.782E-01	logistic	367461	288	367173	0.375	0.038
165	Cancer within the respiratory system	neoplasms	rs76133235	-0.021	0.051	0.979	6.790E-01	logistic	422554	5325	417229	0.419	0.038
578	Gastrointestinal hemorrhage	digestive	rs76133235	-0.016	0.039	0.984	6.807E-01	logistic	392729	9426	383303	0.536	0.038
537	Other disorders of stomach and duodenum	digestive	rs76133235	0.035	0.085	1.036	6.811E-01	logistic	364031	1817	362214	0.089	0.038
627	Menopausal and postmenopausal disorders	genitourinary	rs76133235	0.029	0.071	1.030	6.819E-01	logistic	195450	2703	192747	0.675	0.038
339	Other headache syndromes	neurological	rs76133235	-0.024	0.058	0.977	6.834E-01	logistic	409356	4169	405187	0.439	0.038
379.2	Disorders of vitreous body	sense organs	rs76133235	-0.077	0.189	0.926	6.841E-01	logistic	412333	405	411928	0.557	0.038
594.8	Renal colic	genitourinary	rs76133235	-0.065	0.161	0.937	6.860E-01	logistic	412927	551	412376	0.649	0.038
189.11	Malignant neoplasm of kidney, except pelvis	neoplasms	rs76133235	-0.045	0.111	0.956	6.870E-01	logistic	418324	1137	417187	0.382	0.038
530.1	Esophagitis, GERD and related diseases	digestive	rs76133235	-0.009	0.023	0.991	6.878E-01	logistic	385890	29102	356788	0.386	0.039
425.1	Primary/intrinsic cardiomyopathies	circulatory system	rs76133235	0.034	0.084	1.034	6.882E-01	logistic	418636	1866	416770	0.308	0.038
789	Nausea and vomiting	symptoms	rs76133235	-0.018	0.045	0.982	6.885E-01	logistic	407825	6923	400902	0.341	0.038
38	Septicemia	infectious diseases	rs76133235	0.017	0.043	1.018	6.889E-01	logistic	403876	7229	396647	0.009	0.038
389.1	Sensorineural hearing loss	sense organs	rs76133235	-0.054	0.136	0.948	6.931E-01	logistic	409063	766	408297	0.347	0.038
509.8	Dependence on respirator [Ventilator] or supplemental oxygen	respiratory	rs76133235	-0.056	0.143	0.946	6.947E-01	logistic	391755	698	391057	0.208	0.038
724.2	Disorders of coccyx	musculoskeletal	rs76133235	0.094	0.241	1.099	6.952E-01	logistic	408885	215	408670	0.461	0.038
721.8	Other allied disorders of spine	musculoskeletal	rs76133235	-0.106	0.272	0.899	6.955E-01	logistic	408871	201	408670	0.437	0.038
550.6	Incisional hernia	digestive	rs76133235	-0.069	0.177	0.933	6.977E-01	logistic	365851	457	365394	0.016	0.039
556.1	Ulceration of intestine	digestive	rs76133235	-0.106	0.272	0.900	6.979E-01	logistic	334685	200	334485	0.192	0.039
473.4	Voice disturbance	respiratory	rs76133235	-0.088	0.227	0.916	6.983E-01	logistic	396801	284	396517	0.365	0.038
459	Other disorders of circulatory system	circulatory system	rs76133235	-0.010	0.025	0.990	7.015E-01	logistic	395720	23148	372572	0.156	0.038
200	Myeloproliferative disease	neoplasms	rs76133235	-0.037	0.097	0.964	7.026E-01	logistic	417103	1490	415613	0.430	0.038
536	Disorders of function of stomach	digestive	rs76133235	-0.035	0.092	0.965	7.030E-01	logistic	363858	1644	362214	0.102	0.038
707	Chronic ulcer of skin	dermatologic	rs76133235	0.020	0.053	1.020	7.055E-01	logistic	420904	4774	416130	0.331	0.038
459.9	Circulatory disease NEC	circulatory system	rs76133235	-0.010	0.025	0.990	7.057E-01	logistic	395548	22976	372572	0.164	0.038
509	Respiratory failure, insufficiency, arrest	respiratory	rs76133235	-0.018	0.048	0.982	7.060E-01	logistic	397038	5981	391057	0.118	0.038
530	Diseases of esophagus	digestive	rs76133235	-0.008	0.022	0.992	7.060E-01	logistic	388733	31945	356788	0.466	0.039
740.2	Osteoarthrosis, generalized	musculoskeletal	rs76133235	-0.021	0.055	0.979	7.072E-01	logistic	350458	4600	345858	0.714	0.038
571.6	Primary biliary cirrhosis	digestive	rs76133235	-0.081	0.217	0.922	7.081E-01	logistic	400586	312	400274	0.058	0.038
751.2	Congenital anomalies of urinary system	congenital anomalies	rs76133235	-0.051	0.137	0.950	7.082E-01	logistic	421003	750	420253	0.440	0.038
358.1	Myasthenia gravis	neurological	rs76133235	-0.083	0.222	0.920	7.082E-01	logistic	419463	295	419168	0.463	0.038
433.5	Cerebral aneurysm	circulatory system	rs76133235	-0.067	0.180	0.935	7.092E-01	logistic	403130	445	402685	0.318	0.038
362	Other retinal disorders	sense organs	rs76133235	-0.014	0.038	0.986	7.094E-01	logistic	407698	9941	397757	0.363	0.038
585.1	Acute renal failure	genitourinary	rs76133235	-0.012	0.033	0.988	7.099E-01	logistic	394375	13313	381062	0.151	0.039
283	Acquired hemolytic anemias	hematopoietic	rs76133235	-0.101	0.272	0.904	7.099E-01	logistic	382649	200	382449	0.406	0.039

ST31; PheWAS results for the lead cis-pQTLs of the 4 proteins prioritized (ACOX1, FGF5, FURIN, and MST1).

SNP = Single Nucleotide Polymorphism; SE = Standard error HWE_p = P-value for Hardy-Weinberg equilibrium test

Phenotype	Description	Group	SNP	Beta	SE	Odds tatio	P-value	Type	n_total	n_cases	n_controls	HWE_p	allele_freq
395.4	Nonrheumatic pulmonary valve disorders	circulatory system	rs76133235	-0.079	0.212	0.924	7.113E-01	logistic	401735	321	401414	0.471	0.038
327	Sleep disorders	neurological	rs76133235	-0.059	0.159	0.943	7.117E-01	logistic	413949	565	413384	0.361	0.038
386	Vertiginous syndromes and other disorders of vestibular system	sense organs	rs76133235	0.033	0.089	1.033	7.128E-01	logistic	411346	1681	409665	0.306	0.038
420.3	Endocarditis	circulatory system	rs76133235	0.053	0.144	1.055	7.129E-01	logistic	417383	613	416770	0.368	0.038
835	Internal derangement of knee	injuries & poisonings	rs76133235	-0.020	0.054	0.980	7.161E-01	logistic	409924	4709	405215	0.376	0.038
315	Develomental delays and disorders	mental disorders	rs76133235	0.050	0.138	1.051	7.176E-01	logistic	422717	680	422037	0.412	0.038
756	Other congenital musculoskeletal anomalies	congenital anomalies	rs76133235	0.042	0.116	1.043	7.181E-01	logistic	422256	974	421282	0.474	0.038
531.3	Duodenal ulcer	digestive	rs76133235	-0.040	0.111	0.961	7.182E-01	logistic	412469	1130	411339	0.445	0.038
698	Pruritus and related conditions	dermatologic	rs76133235	0.077	0.213	1.080	7.190E-01	logistic	422717	278	422439	0.440	0.038
198.6	Secondary malignancy of bone	neoplasms	rs76133235	-0.019	0.054	0.981	7.191E-01	logistic	390762	4756	386006	0.164	0.039
575.8	Other disorders of biliary tract	digestive	rs76133235	0.040	0.111	1.041	7.204E-01	logistic	396103	1056	395047	0.383	0.038
474	Acute and chronic tonsillitis	respiratory	rs76133235	0.066	0.183	1.068	7.204E-01	logistic	396895	378	396517	0.417	0.038
345.1	Epilepsy	neurological	rs76133235	-0.058	0.161	0.944	7.206E-01	logistic	399597	550	399047	0.346	0.038
244.1	Secondary hypothyroidism	endocrine/metabolic	rs76133235	0.037	0.104	1.038	7.229E-01	logistic	393443	1213	392230	0.391	0.038
444	Arterial embolism and thrombosis	circulatory system	rs76133235	-0.049	0.137	0.953	7.237E-01	logistic	406215	746	405469	0.421	0.038
296.2	Depression	mental disorders	rs76133235	-0.008	0.023	0.992	7.244E-01	logistic	408593	26971	381622	0.456	0.038
622	Polyp of female genital organs	genitourinary	rs76133235	-0.026	0.075	0.974	7.264E-01	logistic	216102	2530	213572	0.520	0.038
426.21	First degree AV block	circulatory system	rs76133235	0.030	0.085	1.030	7.273E-01	logistic	373661	1813	371848	0.538	0.038
475	Chronic sinusitis	respiratory	rs76133235	0.041	0.117	1.042	7.284E-01	logistic	397468	951	396517	0.329	0.038
447	Other disorders of arteries and arterioles	circulatory system	rs76133235	-0.039	0.113	0.962	7.296E-01	logistic	406558	1089	405469	0.544	0.038
381.11	Suppurative and unspecified otitis media	sense organs	rs76133235	-0.083	0.240	0.920	7.297E-01	logistic	418969	253	418716	0.468	0.038
514	Abnormal findings examination of lungs	respiratory	rs76133235	-0.028	0.080	0.973	7.312E-01	logistic	419804	2147	417657	0.492	0.038
724.1	Disorders of sacrum	musculoskeletal	rs76133235	0.080	0.234	1.084	7.322E-01	logistic	408900	230	408670	0.461	0.038
159	Malignant neoplasm of other and ill-defined sites within the digestive organs and peritoneum	neoplasms	rs76133235	0.041	0.122	1.042	7.343E-01	logistic	394736	874	393862	0.351	0.038
189	Cancer of urinary organs (incl. kidney and bladder)	neoplasms	rs76133235	-0.019	0.055	0.981	7.348E-01	logistic	421760	4573	417187	0.378	0.038
728.7	Fasciitis	musculoskeletal	rs76133235	0.028	0.084	1.029	7.377E-01	logistic	391018	1844	389174	0.816	0.039
427.4	Cardiac arrest and ventricular fibrillation	circulatory system	rs76133235	0.040	0.119	1.041	7.383E-01	logistic	372755	907	371848	0.495	0.038
696.41	Psoriasis vulgaris	dermatologic	rs76133235	0.027	0.079	1.027	7.383E-01	logistic	401395	2105	399290	0.451	0.038
766	Neuralgia, neuritis, and radiculitis NOS	symptoms	rs76133235	-0.076	0.228	0.927	7.397E-01	logistic	418267	281	417986	0.281	0.038
262	Mineral deficiency NEC	endocrine/metabolic	rs76133235	0.064	0.193	1.066	7.411E-01	logistic	412440	343	412097	0.299	0.038
530.13	Barrett's esophagus	digestive	rs76133235	0.018	0.055	1.018	7.431E-01	logistic	361195	4407	356788	0.261	0.039
740.9	Osteoarthrosis NOS	musculoskeletal	rs76133235	-0.010	0.030	0.990	7.437E-01	logistic	361594	15736	345858	0.659	0.038
446	Polyarteritis nodosa and allied conditions	circulatory system	rs76133235	-0.035	0.109	0.965	7.449E-01	logistic	406656	1187	405469	0.373	0.038
728.71	Contracture of palmar fascia [Dupuytren's disease]	musculoskeletal	rs76133235	0.028	0.088	1.029	7.472E-01	logistic	390879	1705	389174	0.804	0.039
622.1	Polyp of corpus uteri	genitourinary	rs76133235	-0.030	0.094	0.970	7.474E-01	logistic	215181	1609	213572	0.546	0.038
513	Respiratory abnormalities	respiratory	rs76133235	0.055	0.172	1.057	7.486E-01	logistic	423122	431	422691	0.443	0.038
509.2	Respiratory insufficiency	respiratory	rs76133235	0.031	0.099	1.032	7.500E-01	logistic	392402	1345	391057	0.134	0.038
709	Diffuse diseases of connective tissue	dermatologic	rs76133235	-0.036	0.112	0.965	7.503E-01	logistic	405028	1129	403899	0.471	0.038
368.1	Amblyopia	sense organs	rs76133235	0.051	0.161	1.052	7.522E-01	logistic	416041	496	415545	0.366	0.038
577	Diseases of pancreas	digestive	rs76133235	0.020	0.064	1.020	7.523E-01	logistic	421247	3239	418008	0.385	0.038
751	Genitourinary congenital anomalies	congenital anomalies	rs76133235	-0.040	0.126	0.961	7.537E-01	logistic	421129	876	420253	0.434	0.038
274.2	Crystal arthropathies	endocrine/metabolic	rs76133235	0.072	0.228	1.074	7.539E-01	logistic	416033	243	415790	0.463	0.038
345.11	Generalized convulsive epilepsy	neurological	rs76133235	0.056	0.180	1.058	7.555E-01	logistic	399441	394	399047	0.348	0.038
596.5	Functional disorders of bladder	genitourinary	rs76133235	-0.046	0.149	0.955	7.584E-01	logistic	402194	640	401554	0.646	0.038
442	Other aneurysm	circulatory system	rs76133235	-0.021	0.068	0.980	7.604E-01	logistic	408493	3024	405469	0.466	0.038
362.3	Other nondiabetic retinopathy	sense organs	rs76133235	-0.048	0.159	0.953	7.637E-01	logistic	398314	557	397757	0.470	0.038
426.91	Cardiac pacemaker in situ	circulatory system	rs76133235	-0.016	0.053	0.984	7.640E-01	logistic	376826	4978	371848	0.499	0.038
193	Thyroid cancer	neoplasms	rs76133235	-0.055	0.186	0.946	7.657E-01	logistic	421300	413	420887	0.370	0.038
427.3	Other specified cardiac dysrhythmias	circulatory system	rs76133235	0.021	0.071	1.021	7.703E-01	logistic	374456	2608	371848	0.477	0.038
292.6	Hallucinations	mental disorders	rs76133235	0.056	0.193	1.057	7.725E-01	logistic	405317	344	404973	0.398	0.038
204	Leukemia	neoplasms	rs76133235	-0.019	0.068	0.981	7.737E-01	logistic	418621	3008	415613	0.450	0.038
317.11	Alcoholic liver damage	mental disorders	rs76133235	-0.030	0.108	0.970	7.767E-01	logistic	404493	1200	403293	0.361	0.038
280	Iron deficiency anemias	hematopoietic	rs76133235	-0.011	0.039	0.989	7.772E-01	logistic	391534	9085	382449	0.544	0.039
560.4	Other intestinal obstruction	digestive	rs76133235	0.022	0.079	1.022	7.793E-01	logistic	336617	2132	334485	0.224	0.039
706	Diseases of sebaceous glands	dermatologic	rs76133235	0.026	0.096	1.027	7.830E-01	logistic	413434	1435	411999	0.311	0.038
441.1	Acute vascular insufficiency of intestine	circulatory system	rs76133235	-0.058	0.217	0.944	7.898E-01	logistic	405771	302	405469	0.409	0.038
619.2	Disorders of uterus, NEC	genitourinary	rs76133235	0.047	0.178	1.049	7.901E-01	logistic	217379	413	216966	0.605	0.038
586.11	Small kidney	genitourinary	rs76133235	0.064	0.241	1.066	7.903E-01	logistic	381281	219	381062	0.187	0.039
760	Back pain	symptoms	rs76133235	-0.014	0.052	0.986	7.923E-01	logistic	414925	5193	409732	0.498	0.038
726.3	Bursitis	musculoskeletal	rs76133235	0.064	0.247	1.066	7.947E-01	logistic	389380	206	389174	0.795	0.039
442.8	Aneurysm of other specified artery	circulatory system	rs76133235	-0.061	0.234	0.941	7.953E-01	logistic	405731	262	405469	0.440	0.038
733	Other disorders of bone and cartilage	musculoskeletal	rs76133235	0.031	0.120	1.032	7.958E-01	logistic	399585	913	398672	0.741	0.038
425	Cardiomyopathy	circulatory system	rs76133235	0.022	0.083	1.022	7.958E-01	logistic	418683	1913	416770	0.308	0.038
290	Delirium dementia and amnestic and other cognitive disorders	mental disorders	rs76133235	-0.011	0.044	0.989	7.969E-01	logistic	412158	7185	404973	0.510	0.038
960	Poisoning by antibiotics	injuries & poisonings	rs76133235	0.007	0.025	1.007	7.972E-01	logistic	389532	22359	367173	0.489	0.038
480	Pneumonia	respiratory	rs76133235	-0.008	0.030	0.992	7.988E-01	logistic	412462	15662	396800	0.304	0.038
857	Mechanical complication of unspecified genitourinary device, implant, and graft	injuries & poisonings	rs76133235	0.023	0.091	1.023	8.021E-01	logistic	418243	1591	416652	0.522	0.038
324	Other CNS infection and poliomyelitis	neurological	rs76133235	0.048	0.193	1.049	8.026E-01	logistic	422418	347	422071	0.483	0.038
38.1	Gram negative septicemia	infectious diseases	rs76133235	-0.053	0.212	0.948	8.029E-01	logistic	396961	314	396647	0.072	0.038
287	Purpura and other hemorrhagic conditions	hematopoietic	rs76133235	0.018	0.075	1.019	8.076E-01	logistic	419896	2343	417553	0.490	0.038
180	Cervical cancer and dysplasia	neoplasms	rs76133235	0.018	0.074	1.018	8.099E-01	logistic	198422	2518	195904	0.739	0.038

ST31; PheWAS results for the lead cis-pQTLs of the 4 proteins prioritized (ACOX1, FGF5, FURIN, and MST1).

SNP = Single Nucleotide Polymorphism; SE = Standard error HWE_p = P-value for Hardy-Weinberg equilibrium test

Phenotype	Description	Group	SNP	Beta	SE	Odds tatio	P-value	Type	n_total	n_cases	n_controls	HWE_p	allele_freq
426.2	Atrioventricular [AV] block	circulatory system	rs76133235	-0.015	0.061	0.986	8.109E-01	logistic	375580	3732	371848	0.599	0.038
451	Phlebitis and thrombophlebitis	circulatory system	rs76133235	-0.033	0.140	0.967	8.119E-01	logistic	381675	709	380966	0.112	0.039
270.32	Paraproteinemia	endocrine/metabolic	rs76133235	-0.036	0.150	0.965	8.122E-01	logistic	422319	617	421702	0.448	0.038
626.8	Infertility, female	genitourinary	rs76133235	0.052	0.218	1.053	8.126E-01	logistic	193029	282	192747	0.625	0.038
351	Other peripheral nerve disorders	neurological	rs76133235	-0.011	0.046	0.989	8.128E-01	logistic	409126	6711	402415	0.402	0.039
297.2	Suicide or self-inflicted injury	mental disorders	rs76133235	-0.049	0.208	0.953	8.157E-01	logistic	381948	326	381622	0.210	0.038
426.9	Cardiac pacemaker/device in situ	circulatory system	rs76133235	-0.012	0.052	0.988	8.188E-01	logistic	376950	5102	371848	0.520	0.038
465.2	Acute pharyngitis	respiratory	rs76133235	-0.055	0.240	0.946	8.188E-01	logistic	419940	248	419692	0.363	0.038
574.1	Cholelithiasis	digestive	rs76133235	0.009	0.039	1.009	8.196E-01	logistic	404312	9265	395047	0.343	0.038
567	Peritonitis and retroperitoneal infections	digestive	rs76133235	0.031	0.134	1.031	8.198E-01	logistic	384568	729	383839	0.389	0.039
530.2	Esophageal bleeding (varices/hemorrhage)	digestive	rs76133235	-0.042	0.183	0.959	8.199E-01	logistic	357206	418	356788	0.339	0.039
208	Benign neoplasm of colon	neoplasms	rs76133235	0.006	0.027	1.006	8.212E-01	logistic	395855	20019	375836	0.244	0.039
523.3	Periodontitis (acute or chronic)	digestive	rs76133235	-0.056	0.247	0.946	8.215E-01	logistic	409770	234	409536	0.437	0.038
427.5	Arrhythmia (cardiac) NOS	circulatory system	rs76133235	0.036	0.161	1.037	8.237E-01	logistic	372348	500	371848	0.486	0.038
528	Diseases of the oral soft tissues, excluding lesions specific for gingiva and tongue	digestive	rs76133235	-0.038	0.175	0.963	8.275E-01	logistic	418169	459	417710	0.432	0.038
189.21	Malignant neoplasm of bladder	neoplasms	rs76133235	0.014	0.067	1.014	8.291E-01	logistic	420210	3023	417187	0.377	0.038
564.9	Personal history of diseases of digestive system	digestive	rs76133235	-0.007	0.032	0.993	8.298E-01	logistic	348229	13744	334485	0.448	0.039
345	Epilepsy, recurrent seizures, convulsions	neurological	rs76133235	-0.012	0.058	0.988	8.323E-01	logistic	403151	4104	399047	0.485	0.038
535	Gastritis and duodenitis	digestive	rs76133235	-0.006	0.029	0.994	8.338E-01	logistic	378649	16435	362214	0.083	0.038
386.9	Dizziness and giddiness (Light-headedness and vertigo)	sense organs	rs76133235	-0.014	0.067	0.986	8.349E-01	logistic	412711	3046	409665	0.283	0.038
452	Other venous embolism and thrombosis	circulatory system	rs76133235	-0.043	0.208	0.958	8.364E-01	logistic	381290	324	380966	0.096	0.039
394.2	Mitral valve disease	circulatory system	rs76133235	-0.043	0.212	0.957	8.379E-01	logistic	401726	312	401414	0.470	0.038
300.13	Phobia	mental disorders	rs76133235	0.030	0.149	1.031	8.382E-01	logistic	382219	597	381622	0.193	0.038
184	Cancer of other female genital organs	neoplasms	rs76133235	-0.014	0.069	0.986	8.389E-01	logistic	207829	2992	204837	0.683	0.038
251.1	Hypoglycemia	endocrine/metabolic	rs76133235	-0.019	0.095	0.981	8.415E-01	logistic	383028	1531	381497	0.281	0.038
333	Extrapyramidal disease and abnormal movement disorders	neurological	rs76133235	0.021	0.104	1.021	8.424E-01	logistic	400270	1223	399047	0.310	0.038
574.3	Cholecystitis without cholelithiasis	digestive	rs76133235	0.021	0.105	1.021	8.432E-01	logistic	396244	1197	395047	0.284	0.038
276.12	Hyposmolality and/or hyponatremia	endocrine/metabolic	rs76133235	0.010	0.052	1.010	8.476E-01	logistic	400551	4979	395572	0.210	0.038
292.1	Aphasia/speech disturbance	mental disorders	rs76133235	-0.022	0.115	0.978	8.492E-01	logistic	406020	1047	404973	0.370	0.038
214.1	Lipoma of skin and subcutaneous tissue	neoplasms	rs76133235	-0.026	0.137	0.974	8.503E-01	logistic	414958	731	414227	0.549	0.038
333.4	Torsion dystonia	neurological	rs76133235	0.039	0.208	1.040	8.506E-01	logistic	399349	302	399047	0.323	0.038
378	Strabismus and other disorders of binocular eye movements	sense organs	rs76133235	0.022	0.118	1.022	8.515E-01	logistic	412884	956	411928	0.532	0.038
295	Schizophrenia and other psychotic disorders	mental disorders	rs76133235	-0.024	0.129	0.976	8.519E-01	logistic	382460	838	381622	0.218	0.038
512.8	Cough	respiratory	rs76133235	-0.017	0.093	0.983	8.527E-01	logistic	406184	1585	404599	0.239	0.038
593	Hematuria	genitourinary	rs76133235	0.010	0.052	1.010	8.528E-01	logistic	381996	5069	376927	0.109	0.038
737	Curvature of spine	musculoskeletal	rs76133235	-0.023	0.125	0.977	8.531E-01	logistic	403550	896	402654	0.640	0.038
287.3	Thrombocytopenia	hematopoietic	rs76133235	0.014	0.077	1.014	8.534E-01	logistic	419777	2224	417553	0.496	0.038
386.2	Peripheral or central vertigo	sense organs	rs76133235	-0.027	0.150	0.973	8.555E-01	logistic	410281	616	409665	0.361	0.038
242	Thyrotoxicosis with or without goiter	endocrine/metabolic	rs76133235	0.017	0.092	1.017	8.561E-01	logistic	393812	1582	392230	0.464	0.038
204.4	Multiple myeloma	neoplasms	rs76133235	0.019	0.108	1.020	8.576E-01	logistic	416757	1144	415613	0.438	0.038
480.5	Bronchopneumonia and lung abscess	respiratory	rs76133235	0.031	0.175	1.032	8.583E-01	logistic	397229	429	396800	0.345	0.038
443.9	Peripheral vascular disease, unspecified	circulatory system	rs76133235	0.011	0.061	1.011	8.584E-01	logistic	409046	3577	405469	0.376	0.038
288	Diseases of white blood cells	hematopoietic	rs76133235	-0.033	0.183	0.968	8.585E-01	logistic	408723	414	408309	0.109	0.038
276.5	Hypovolemia	endocrine/metabolic	rs76133235	-0.009	0.051	0.991	8.595E-01	logistic	400853	5281	395572	0.300	0.038
614	Inflammatory diseases of female pelvic organs	genitourinary	rs76133235	-0.017	0.096	0.983	8.611E-01	logistic	218979	1541	217438	0.491	0.038
571.81	Portal hypertension	digestive	rs76133235	-0.022	0.127	0.978	8.626E-01	logistic	401135	861	400274	0.005	0.038
331.1	Hydrocephalus	neurological	rs76133235	0.025	0.146	1.026	8.628E-01	logistic	399668	621	399047	0.338	0.038
396	Abnormal heart sounds	circulatory system	rs76133235	-0.021	0.121	0.979	8.634E-01	logistic	402357	943	401414	0.444	0.038
38.2	Gram positive septicemia	infectious diseases	rs76133235	0.025	0.146	1.025	8.640E-01	logistic	397268	621	396647	0.056	0.038
80	Postoperative infection	infectious diseases	rs76133235	-0.014	0.085	0.986	8.662E-01	logistic	414741	1897	412844	0.435	0.038
296.1	Bipolar	mental disorders	rs76133235	0.018	0.107	1.018	8.683E-01	logistic	382786	1164	381622	0.232	0.038
281	Other deficiency anemia	hematopoietic	rs76133235	0.017	0.101	1.017	8.691E-01	logistic	383766	1317	382449	0.471	0.039
411.8	Other chronic ischemic heart disease, unspecified	circulatory system	rs76133235	-0.005	0.028	0.995	8.694E-01	logistic	389207	18657	370550	0.555	0.039
433.1	Occlusion and stenosis of precerebral arteries	circulatory system	rs76133235	-0.015	0.093	0.985	8.719E-01	logistic	404257	1572	402685	0.357	0.038
252.1	Hyperparathyroidism	endocrine/metabolic	rs76133235	0.018	0.114	1.018	8.730E-01	logistic	416271	1025	415246	0.321	0.038
521	Diseases of hard tissues of teeth	digestive	rs76133235	0.021	0.129	1.021	8.734E-01	logistic	410337	801	409536	0.439	0.039
801	Fracture of ankle and foot	injuries & poisonings	rs76133235	0.022	0.142	1.023	8.741E-01	logistic	397040	662	396378	0.443	0.038
204.1	Lymphoid leukemia	neoplasms	rs76133235	0.019	0.121	1.019	8.753E-01	logistic	416514	901	415613	0.362	0.038
293.1	Swelling, mass, or lump in head and neck [Space-occupying lesion, intracranial NOS]	mental disorders	rs76133235	0.032	0.204	1.032	8.756E-01	logistic	420026	315	419711	0.330	0.038
458	Hypotension	circulatory system	rs76133235	-0.006	0.041	0.994	8.765E-01	logistic	380778	8206	372572	0.250	0.038
602	Other disorders of prostate	genitourinary	rs76133235	-0.031	0.200	0.970	8.771E-01	logistic	156802	341	156461	0.800	0.039
191.11	Cancer of brain	neoplasms	rs76133235	0.020	0.130	1.020	8.790E-01	logistic	421187	790	420397	0.409	0.038
619.4	Noninflammatory disorders of vagina	genitourinary	rs76133235	0.036	0.241	1.037	8.797E-01	logistic	217194	228	216966	0.628	0.038
367.8	Hypermetropia	sense organs	rs76133235	0.033	0.217	1.033	8.807E-01	logistic	411285	279	411006	0.525	0.038
635	Hemorrhage during pregnancy; childbirth and postpartum	pregnancy complications	rs76133235	-0.028	0.187	0.972	8.808E-01	logistic	223005	413	222592	0.493	0.038
802	Fracture of pelvis	injuries & poisonings	rs76133235	-0.018	0.120	0.983	8.836E-01	logistic	397331	953	396378	0.517	0.038
189.2	Cancer of bladder	neoplasms	rs76133235	0.009	0.063	1.009	8.845E-01	logistic	420532	3345	417187	0.393	0.038
480.11	Pneumococcal pneumonia	respiratory	rs76133235	0.005	0.038	1.005	8.879E-01	logistic	406463	9663	396800	0.287	0.038
204.21	Myeloid leukemia, acute	neoplasms	rs76133235	-0.021	0.154	0.979	8.905E-01	logistic	416195	582	415613	0.437	0.038
244	Hypothyroidism	endocrine/metabolic	rs76133235	-0.004	0.028	0.996	8.922E-01	logistic	411296	19066	392230	0.103	0.038
112	Candidiasis	infectious diseases	rs76133235	0.012	0.093	1.012	8.947E-01	logistic	419935	1555	418380	0.452	0.038

ST31; PheWAS results for the lead cis-pQTLs of the 4 proteins prioritized (ACOX1, FGF5, FURIN, and MST1).

SNP = Single Nucleotide Polymorphism; SE = Standard error HWE_p = P-value for Hardy-Weinberg equilibrium test

Phenotype	Description	Group	SNP	Beta	SE	Odds tatio	P-value	Type	n_total	n_cases	n_controls	HWE_p	allele_freq
592.1	Cystitis	genitourinary	rs76133235	-0.014	0.107	0.986	8.949E-01	logistic	378131	1204	376927	0.042	0.038
277.4	Disorders of bilirubin excretion	endocrine/metabolic	rs76133235	-0.023	0.172	0.978	8.959E-01	logistic	420403	462	419941	0.325	0.038
41.1	Staphylococcus infections	infectious diseases	rs76133235	-0.010	0.077	0.990	8.961E-01	logistic	398981	2334	396647	0.100	0.038
427.41	Ventricular fibrillation and flutter	circulatory system	rs76133235	-0.029	0.228	0.972	9.003E-01	logistic	372113	265	371848	0.498	0.038
761	Cervicalgia	symptoms	rs76133235	0.017	0.134	1.017	9.008E-01	logistic	422454	744	421710	0.412	0.038
378.1	Strabismus (not specified as paralytic)	sense organs	rs76133235	-0.028	0.228	0.972	9.023E-01	logistic	412196	268	411928	0.560	0.038
627.1	Postmenopausal bleeding	genitourinary	rs76133235	-0.010	0.089	0.990	9.059E-01	logistic	194538	1791	192747	0.658	0.038
204.12	Lymphoid leukemia, chronic	neoplasms	rs76133235	0.015	0.128	1.015	9.081E-01	logistic	416428	815	415613	0.366	0.038
291	Other specified nonpsychotic and/or transient mental disorders	mental disorders	rs76133235	-0.021	0.186	0.979	9.084E-01	logistic	405371	398	404973	0.337	0.038
574	Cholelithiasis and cholecystitis	digestive	rs76133235	-0.004	0.036	0.996	9.088E-01	logistic	406113	11066	395047	0.333	0.038
433.3	Cerebral ischemia	circulatory system	rs76133235	0.006	0.056	1.006	9.091E-01	logistic	407003	4318	402685	0.319	0.038
275.1	Disorders of iron metabolism	hematopoietic	rs76133235	-0.012	0.112	0.988	9.116E-01	logistic	417274	1089	416185	0.383	0.038
636.3	Hemorrhage in early pregnancy	pregnancy complications	rs76133235	-0.028	0.255	0.973	9.132E-01	logistic	222810	218	222592	0.484	0.038
800.2	Fracture of unspecified part of femur	injuries & poisonings	rs76133235	-0.028	0.255	0.973	9.133E-01	logistic	396592	214	396378	0.464	0.038
430.2	Intracerebral hemorrhage	circulatory system	rs76133235	-0.012	0.112	0.988	9.138E-01	logistic	403773	1088	402685	0.379	0.038
290.11	Alzheimer's disease	mental disorders	rs76133235	-0.008	0.076	0.992	9.147E-01	logistic	407367	2394	404973	0.428	0.038
697	Sarcoidosis	dermatologic	rs76133235	0.016	0.150	1.016	9.155E-01	logistic	405795	589	405206	0.431	0.038
965.1	Opiates and related narcotics causing adverse effects in therapeutic use	injuries & poisonings	rs76133235	-0.008	0.078	0.992	9.159E-01	logistic	369447	2274	367173	0.284	0.038
473	Diseases of the larynx and vocal cords	respiratory	rs76133235	-0.011	0.103	0.989	9.160E-01	logistic	397819	1302	396517	0.409	0.038
281.1	Megaloblastic anemia	hematopoietic	rs76133235	0.011	0.103	1.011	9.170E-01	logistic	383709	1260	382449	0.474	0.039
368.9	Subjective visual disturbances	sense organs	rs76133235	0.016	0.161	1.017	9.188E-01	logistic	416057	512	415545	0.396	0.038
280.1	Iron deficiency anemias, unspecified or not due to blood loss	hematopoietic	rs76133235	-0.004	0.040	0.996	9.198E-01	logistic	391238	8789	382449	0.551	0.039
819	Skull and face fracture and other intercranial injury	injuries & poisonings	rs76133235	-0.017	0.165	0.984	9.204E-01	logistic	422666	501	422165	0.372	0.038
172.3	Carcinoma in situ of skin	neoplasms	rs76133235	0.018	0.180	1.018	9.210E-01	logistic	407616	408	407208	0.535	0.038
41.11	Methicillin sensitive Staphylococcus aureus	infectious diseases	rs76133235	0.009	0.091	1.009	9.219E-01	logistic	398282	1635	396647	0.005	0.038
740.1	Osteoarthritis; localized	musculoskeletal	rs76133235	-0.002	0.023	0.998	9.231E-01	logistic	374099	28241	345858	0.581	0.038
578.2	Blood in stool	digestive	rs76133235	-0.008	0.087	0.992	9.233E-01	logistic	385103	1800	383303	0.427	0.039
747.12	Valvular heart disease/ heart chambers	congenital anomalies	rs76133235	0.021	0.217	1.021	9.238E-01	logistic	421875	280	421595	0.330	0.038
573	Other disorders of liver	digestive	rs76133235	-0.008	0.082	0.992	9.242E-01	logistic	402294	2020	400274	0.063	0.038
386.3	Labyrinthitis	sense organs	rs76133235	0.018	0.196	1.019	9.254E-01	logistic	410012	347	409665	0.341	0.038
394	Rheumatic disease of the heart valves	circulatory system	rs76133235	0.005	0.053	1.005	9.274E-01	logistic	406218	4804	401414	0.445	0.038
357	Inflammatory and toxic neuropathy	neurological	rs76133235	0.007	0.082	1.007	9.281E-01	logistic	421163	1995	419168	0.499	0.038
199	Neoplasm of uncertain behavior	neoplasms	rs76133235	0.013	0.147	1.013	9.287E-01	logistic	386620	614	386006	0.127	0.039
960.2	Allergy/adverse effect of penicillin	injuries & poisonings	rs76133235	0.002	0.028	1.002	9.292E-01	logistic	385833	18660	367173	0.482	0.038
442.1	Aortic aneurysm	circulatory system	rs76133235	0.007	0.074	1.007	9.295E-01	logistic	407951	2482	405469	0.426	0.038
531	Peptic ulcer (excl. esophageal)	digestive	rs76133235	0.005	0.058	1.005	9.319E-01	logistic	415329	3990	411339	0.460	0.038
521.1	Dental caries	digestive	rs76133235	0.011	0.134	1.011	9.325E-01	logistic	410280	744	409536	0.442	0.038
555.21	Ulcerative colitis (chronic)	digestive	rs76133235	0.016	0.186	1.016	9.330E-01	logistic	334866	381	334485	0.216	0.039
295.1	Schizophrenia	mental disorders	rs76133235	-0.012	0.143	0.988	9.336E-01	logistic	382292	670	381622	0.226	0.038
53	Herpes zoster	infectious diseases	rs76133235	0.015	0.193	1.015	9.364E-01	logistic	415563	359	415204	0.383	0.038
569	Other disorders of intestine	digestive	rs76133235	0.006	0.079	1.006	9.367E-01	logistic	385990	2151	383839	0.475	0.039
724	Other and unspecified disorders of back	musculoskeletal	rs76133235	-0.013	0.159	0.987	9.368E-01	logistic	409212	542	408670	0.450	0.038
709.3	Systemic sclerosis	dermatologic	rs76133235	0.019	0.247	1.019	9.384E-01	logistic	404118	219	403899	0.504	0.038
430	Intracranial hemorrhage	circulatory system	rs76133235	-0.006	0.085	0.994	9.397E-01	logistic	404595	1910	402685	0.459	0.038
939	Atopic/contact dermatitis due to other or unspecified	dermatologic	rs76133235	0.006	0.083	1.006	9.399E-01	logistic	416950	1963	414987	0.551	0.038
427.7	Tachycardia NOS	circulatory system	rs76133235	-0.006	0.085	0.994	9.408E-01	logistic	373741	1893	371848	0.486	0.038
361	Retinal detachments and defects	sense organs	rs76133235	0.012	0.161	1.012	9.409E-01	logistic	398276	513	397763	0.470	0.038
592	Cystitis and urethritis	genitourinary	rs76133235	-0.008	0.105	0.992	9.410E-01	logistic	378176	1249	376927	0.045	0.038
618.1	Prolapse of vaginal walls	genitourinary	rs76133235	-0.004	0.059	0.996	9.421E-01	logistic	217426	3971	213455	0.331	0.038
333.8	Other degenerative diseases of the basal ganglia	neurological	rs76133235	-0.018	0.247	0.982	9.425E-01	logistic	399271	224	399047	0.359	0.038
572	Ascites (non malignant)	digestive	rs76133235	0.006	0.080	1.006	9.426E-01	logistic	402409	2135	400274	0.030	0.038
447.1	Stricture of artery	circulatory system	rs76133235	0.010	0.147	1.010	9.459E-01	logistic	406083	614	405469	0.479	0.038
735.21	Hammer toe (acquired)	musculoskeletal	rs76133235	-0.012	0.175	0.989	9.473E-01	logistic	403105	451	402654	0.696	0.038
342	Hemiplegia	neurological	rs76133235	0.005	0.075	1.005	9.478E-01	logistic	401449	2402	399047	0.349	0.038
596.1	Bladder neck obstruction	genitourinary	rs76133235	0.010	0.154	1.010	9.484E-01	logistic	402115	561	401554	0.599	0.038
941	Adverse reaction to serum or vaccine	injuries & poisonings	rs76133235	-0.015	0.247	0.985	9.509E-01	logistic	415212	225	414987	0.516	0.038
473.3	Paralysis/spasm of vocal cords or larynx	respiratory	rs76133235	-0.015	0.247	0.985	9.510E-01	logistic	396742	225	396517	0.396	0.038
244.4	Hypothyroidism NOS	endocrine/metabolic	rs76133235	0.002	0.028	1.002	9.523E-01	logistic	410296	18066	392230	0.076	0.038
555.1	Regional enteritis	digestive	rs76133235	-0.005	0.086	0.995	9.537E-01	logistic	336302	1817	334485	0.103	0.039
585.3	Chronic renal failure [CKD]	genitourinary	rs76133235	0.002	0.034	1.002	9.547E-01	logistic	393739	12677	381062	0.240	0.039
253.2	Pituitary hypofunction	endocrine/metabolic	rs76133235	-0.012	0.217	0.988	9.572E-01	logistic	415534	288	415246	0.356	0.038
575	Other biliary tract disease	digestive	rs76133235	0.003	0.061	1.003	9.592E-01	logistic	398734	3687	395047	0.475	0.038
747.1	Cardiac congenital anomalies	congenital anomalies	rs76133235	-0.006	0.120	0.994	9.597E-01	logistic	422536	941	421595	0.331	0.038
411.3	Angina pectoris	circulatory system	rs76133235	-0.001	0.030	0.999	9.610E-01	logistic	387093	16543	370550	0.634	0.039
803.1	Fracture of humerus	injuries & poisonings	rs76133235	0.005	0.111	1.005	9.624E-01	logistic	397468	1090	396378	0.482	0.038
425.12	Other hypertrophic cardiomyopathy	circulatory system	rs76133235	-0.010	0.222	0.990	9.630E-01	logistic	417044	274	416770	0.356	0.038
389.3	Degenerative and vascular disorders of ear	sense organs	rs76133235	-0.009	0.200	0.991	9.642E-01	logistic	408638	341	408297	0.363	0.038
737.3	Kyphoscoliosis and scoliosis	musculoskeletal	rs76133235	-0.006	0.132	0.994	9.659E-01	logistic	403443	789	402654	0.643	0.038
191	Manlignant and unknown neoplasms of brain and nervous system	neoplasms	rs76133235	-0.005	0.119	0.995	9.663E-01	logistic	421363	966	420397	0.404	0.038
297	Suicidal ideation or attempt	mental disorders	rs76133235	0.008	0.193	1.008	9.686E-01	logistic	381982	360	381622	0.206	0.038
272.9	Unspecified disorder of lipoid metabolism	endocrine/metabolic	rs76133235	0.008	0.204	1.008	9.693E-01	logistic	356085	320	355765	0.392	0.039

ST31; PheWAS results for the lead cis-pQTLs of the 4 proteins prioritized (ACOX1, FGF5, FURIN, and MST1).

SNP = Single Nucleotide Polymorphism; SE = Standard error HWE_p = P-value for Hardy-Weinberg equilibrium test

Phenotype	Description	Group	SNP	Beta	SE	Odds tatio	P-value	Type	n_total	n_cases	n_controls	HWE_p	allele_freq
946	Anaphylactic shock NOS	injuries & poisonings	rs76133235	-0.009	0.247	0.991	9.702E-01	logistic	415210	223	414987	0.516	0.038
197	Chemotherapy	neoplasms	rs76133235	0.001	0.035	1.001	9.704E-01	logistic	397532	11526	386006	0.187	0.039
394.7	Disease of tricuspid valve	circulatory system	rs76133235	0.005	0.143	1.005	9.719E-01	logistic	402072	658	401414	0.482	0.038
622.2	Mucous polyp of cervix	genitourinary	rs76133235	-0.008	0.241	0.992	9.725E-01	logistic	213810	238	213572	0.516	0.038
202	Cancer of other lymphoid, histiocytic tissue	neoplasms	rs76133235	-0.002	0.071	0.998	9.767E-01	logistic	418277	2664	415613	0.375	0.038
202.2	Non-Hodgkins lymphoma	neoplasms	rs76133235	-0.002	0.071	0.998	9.767E-01	logistic	418277	2664	415613	0.375	0.038
626.12	Excessive or frequent menstruation	genitourinary	rs76133235	-0.002	0.081	0.998	9.775E-01	logistic	194922	2175	192747	0.610	0.038
480.1	Bacterial pneumonia	respiratory	rs76133235	-0.001	0.037	0.999	9.782E-01	logistic	407095	10295	396800	0.263	0.038
184.1	Malignant neoplasm of ovary and other uterine adnexa	neoplasms	rs76133235	0.002	0.071	1.002	9.789E-01	logistic	207626	2789	204837	0.678	0.038
509.1	Respiratory failure	respiratory	rs76133235	0.001	0.056	1.001	9.812E-01	logistic	395437	4380	391057	0.086	0.038
420	Carditis	circulatory system	rs76133235	0.002	0.079	1.002	9.836E-01	logistic	418942	2172	416770	0.447	0.038
599.5	Frequency of urination and polyuria	genitourinary	rs76133235	-0.002	0.126	0.998	9.888E-01	logistic	390965	859	390106	0.484	0.038
522.5	Periapical abscess	digestive	rs76133235	-0.004	0.255	0.996	9.888E-01	logistic	409744	208	409536	0.437	0.038
241.2	Nontoxic multinodular goiter	endocrine/metabolic	rs76133235	-0.003	0.240	0.997	9.895E-01	logistic	392467	237	392230	0.378	0.038
626.14	Irregular menstrual bleeding	genitourinary	rs76133235	-0.002	0.187	0.998	9.896E-01	logistic	193146	399	192747	0.629	0.038
614.1	Pelvic peritoneal adhesions, female (postoperative) (postinfection	genitourinary	rs76133235	0.002	0.186	1.002	9.933E-01	logistic	217833	395	217438	0.470	0.038
740	Osteoarthrosis	musculoskeletal	rs76133235	0.000	0.019	1.000	9.941E-01	logistic	392374	46516	345858	0.518	0.038
79	Viral infection	infectious diseases	rs76133235	-0.001	0.116	0.999	9.943E-01	logistic	416221	1017	415204	0.384	0.038
189.4	Malignant neoplasm of other urinary organs	neoplasms	rs76133235	0.001	0.255	1.001	9.955E-01	logistic	417393	206	417187	0.418	0.038
300.12	Agorophobia, social phobia, and panic disorder	mental disorders	rs76133235	0.001	0.143	1.001	9.958E-01	logistic	382286	664	381622	0.192	0.038
395.1	Nonrheumatic mitral valve disorders	circulatory system	rs76133235	0.000	0.061	1.000	9.960E-01	logistic	405090	3676	401414	0.494	0.038
446.5	Giant cell arteritis	circulatory system	rs76133235	-0.001	0.150	0.999	9.960E-01	logistic	406071	602	405469	0.365	0.038
191.1	Cancer of brain and nervous system	neoplasms	rs76133235	-0.001	0.127	0.999	9.962E-01	logistic	421229	832	420397	0.409	0.038
428.2	Heart failure NOS	circulatory system	rs76133235	0.000	0.053	1.000	9.972E-01	logistic	414622	4919	409703	0.433	0.039
504	Other alveolar and parietoalveolar pneumonopathy	respiratory	rs76133235	0.000	0.143	1.000	9.979E-01	logistic	391718	661	391057	0.172	0.038
706.2	Sebaceous cyst	dermatologic	rs76133235	0.000	0.099	1.000	9.984E-01	logistic	413393	1394	411999	0.317	0.038
430.1	Subarachnoid hemorrhage	circulatory system	rs76133235	0.000	0.136	1.000	9.985E-01	logistic	403416	731	402685	0.394	0.038
184.11	Malignant neoplasm of ovary	neoplasms	rs76133235	0.000	0.072	1.000	9.988E-01	logistic	207552	2715	204837	0.674	0.038
384	Other disorders of tympanic membrane	sense organs	rs76133235	0.000	0.193	1.000	9.996E-01	logistic	419080	364	418716	0.462	0.038
519.8	Other diseases of respiratory system, NEC	respiratory	rs189447480	0.218	0.069	1.244	1.519E-03	logistic	411807	8677	403130	0.246	0.010
441	Vascular insufficiency of intestine	circulatory system	rs189447480	0.623	0.198	1.864	1.679E-03	logistic	406151	682	405469	0.040	0.010
519	Other diseases of respiratory system, not elsewhere classified	respiratory	rs189447480	0.214	0.068	1.239	1.749E-03	logistic	411962	8832	403130	0.130	0.010
216	Benign neoplasm of skin	neoplasms	rs189447480	0.506	0.164	1.659	2.035E-03	logistic	415074	1116	413958	0.061	0.010
441.1	Acute vascular insufficiency of intestine	circulatory system	rs189447480	0.822	0.270	2.274	2.363E-03	logistic	405771	302	405469	0.030	0.010
353	Nerve root and plexus disorders	neurological	rs189447480	0.817	0.271	2.264	2.525E-03	logistic	402720	305	402415	0.019	0.010
766	Neuralgia, neuritis, and radiculitis NOS	symptoms	rs189447480	0.819	0.281	2.268	3.525E-03	logistic	418267	281	417986	0.089	0.010
365.2	Primary angle-closure glaucoma	sense organs	rs189447480	0.541	0.206	1.718	8.633E-03	logistic	398441	678	397763	0.106	0.010
189.11	Malignant neoplasm of kidney, except pelvis	neoplasms	rs189447480	0.436	0.168	1.547	9.585E-03	logistic	418324	1137	417187	0.087	0.010
327.3	Sleep apnea	neurological	rs189447480	0.217	0.087	1.242	1.249E-02	logistic	418773	5389	413384	0.078	0.010
214.1	Lipoma of skin and subcutaneous tissue	neoplasms	rs189447480	-1.112	0.447	0.329	1.292E-02	logistic	414958	731	414227	0.085	0.010
642	Hypertension complicating pregnancy, childbirth, and the puerperium	pregnancy complications	rs189447480	0.608	0.248	1.837	1.431E-02	logistic	228250	456	227794	0.392	0.010
531	Peptic ulcer (excl. esophageal)	digestive	rs189447480	-0.304	0.129	0.738	1.865E-02	logistic	415329	3990	411339	0.035	0.010
270.32	Paraproteinemia	endocrine/metabolic	rs189447480	0.508	0.220	1.662	2.102E-02	logistic	422319	617	421702	0.125	0.010
369.5	Conjunctivitis, infectious	sense organs	rs189447480	0.762	0.337	2.142	2.367E-02	logistic	410999	206	410793	0.237	0.010
288.1	Decreased white blood cell count	hematopoietic	rs189447480	0.249	0.110	1.283	2.390E-02	logistic	411502	3193	408309	0.227	0.010
288.11	Neutropenia	hematopoietic	rs189447480	0.249	0.110	1.283	2.390E-02	logistic	411502	3193	408309	0.227	0.010
189.1	Cancer of kidney and renal pelvis	neoplasms	rs189447480	0.377	0.168	1.458	2.505E-02	logistic	418392	1205	417187	0.086	0.010
270.3	Disorders of plasma protein metabolism	endocrine/metabolic	rs189447480	0.450	0.206	1.568	2.898E-02	logistic	422449	747	421702	0.127	0.010
747	Cardiac and circulatory congenital anomalies	congenital anomalies	rs189447480	0.384	0.176	1.468	2.905E-02	logistic	422691	1096	421595	0.002	0.010
270	Disorders of protein plasma/amino-acid transport and metabolism	endocrine/metabolic	rs189447480	0.437	0.202	1.548	3.040E-02	logistic	422490	788	421702	0.128	0.010
870	Open wounds of head; neck; and trunk	injuries & poisonings	rs189447480	-0.763	0.354	0.466	3.123E-02	logistic	420976	826	420150	0.000	0.010
420.2	Pericarditis	circulatory system	rs189447480	0.335	0.156	1.397	3.174E-02	logistic	418236	1466	416770	0.295	0.010
275.3	Disorders of magnesium metabolism	endocrine/metabolic	rs189447480	0.325	0.152	1.384	3.282E-02	logistic	417729	1544	416185	0.195	0.010
580.14	Chronic glomerulonephritis, NOS	genitourinary	rs189447480	0.619	0.291	1.857	3.366E-02	logistic	381376	314	381062	0.269	0.010
735.3	Hallux valgus (Bunion)	musculoskeletal	rs189447480	0.265	0.125	1.303	3.400E-02	logistic	405116	2462	402654	0.284	0.010
465.2	Acute pharyngitis	respiratory	rs189447480	0.678	0.320	1.969	3.403E-02	logistic	419940	248	419692	0.006	0.010
198.2	Secondary malignancy of respiratory organs	neoplasms	rs189447480	0.198	0.093	1.219	3.427E-02	logistic	390707	4701	386006	0.468	0.010
428.2	Heart failure NOS	circulatory system	rs189447480	0.195	0.092	1.215	3.475E-02	logistic	414622	4919	409703	0.026	0.010
447.1	Stricture of artery	circulatory system	rs189447480	0.471	0.226	1.602	3.691E-02	logistic	406083	614	405469	0.155	0.010
81	Infection/inflammation of internal prosthetic device; implant; and graft	infectious diseases	rs189447480	-0.397	0.193	0.673	4.037E-02	logistic	414783	1939	412844	0.268	0.010
427.22	Atrial flutter	circulatory system	rs189447480	0.451	0.220	1.569	4.085E-02	logistic	372499	651	371848	0.237	0.010
977	Personal history of allergy to medicinal agents	injuries & poisonings	rs189447480	0.149	0.073	1.161	4.172E-02	logistic	375378	8205	367173	0.109	0.010
509.2	Respiratory insufficiency	respiratory	rs189447480	-0.493	0.244	0.611	4.305E-02	logistic	392402	1345	391057	0.453	0.010
735	Acquired foot deformities	musculoskeletal	rs189447480	0.166	0.084	1.181	4.742E-02	logistic	408734	6080	402654	0.220	0.010
735.21	Hammer toe (acquired)	musculoskeletal	rs189447480	-1.137	0.578	0.321	4.921E-02	logistic	403105	451	402654	0.234	0.010
586.4	Stricture/obstruction of ureter	genitourinary	rs189447480	-0.656	0.334	0.519	4.965E-02	logistic	381896	834	381062	0.259	0.010
8.5	Bacterial enteritis	infectious diseases	rs189447480	-0.483	0.251	0.617	5.444E-02	logistic	418567	1251	417316	0.047	0.010
375.2	Epiphora	sense organs	rs189447480	-1.922	1.000	0.146	5.468E-02	logistic	412257	329	411928	0.122	0.010
443	Peripheral vascular disease	circulatory system	rs189447480	0.175	0.091	1.191	5.492E-02	logistic	410595	5126	405469	0.225	0.010
455	Hemorrhoids	circulatory system	rs189447480	-0.203	0.107	0.816	5.730E-02	logistic	386244	5278	380966	0.125	0.010
681	Superficial cellulitis and abscess	dermatologic	rs189447480	0.576	0.304	1.780	5.826E-02	logistic	418799	302	418497	0.198	0.010

ST31; PheWAS results for the lead cis-pQTLs of the 4 proteins prioritized (ACOX1, FGF5, FURIN, and MST1).

SNP = Single Nucleotide Polymorphism; SE = Standard error HWE_p = P-value for Hardy-Weinberg equilibrium test

Phenotype	Description	Group	SNP	Beta	SE	Odds tatio	P-value	Type	n_total	n_cases	n_controls	HWE_p	allele_freq
537	Other disorders of stomach and duodenum	digestive	rs189447480	-0.368	0.197	0.692	6.208E-02	logistic	364031	1817	362214	0.217	0.010
334	Degenerative disease of the spinal corc	neurological	rs189447480	0.274	0.147	1.316	6.241E-02	logistic	400789	1742	399047	0.023	0.010
475	Chronic sinusitis	respiratory	rs189447480	0.355	0.191	1.426	6.265E-02	logistic	397468	951	396517	0.156	0.010
447	Other disorders of arteries and arterioles	circulatory system	rs189447480	0.332	0.181	1.394	6.692E-02	logistic	406558	1089	405469	0.163	0.010
375	Disorders of lacrimal system	sense organs	rs189447480	-0.408	0.225	0.665	6.938E-02	logistic	413379	1451	411928	0.131	0.010
703.1	Ingrowing nail	dermatologic	rs189447480	0.643	0.357	1.901	7.212E-02	logistic	421519	205	421314	0.008	0.010
394.2	Mitral valve disease	circulatory system	rs189447480	0.542	0.304	1.720	7.448E-02	logistic	401726	312	401414	0.088	0.010
323	Encephalitis	neurological	rs189447480	0.517	0.291	1.677	7.571E-02	logistic	422420	349	422071	0.008	0.010
571	Chronic liver disease and cirrhosis	digestive	rs189447480	-0.177	0.100	0.838	7.670E-02	logistic	406135	5861	400274	0.185	0.010
788	Syncope and collapse	symptoms	rs189447480	-0.169	0.097	0.844	8.067E-02	logistic	412052	6241	405811	0.001	0.010
289.8	Polycythemia, secondary	hematopoietic	rs189447480	0.454	0.260	1.575	8.118E-02	logistic	407605	466	407139	0.160	0.010
197	Chemotherapy	neoplasms	rs189447480	-0.122	0.070	0.885	8.191E-02	logistic	397532	11526	386006	0.273	0.010
958	Certain early complications of trauma or procedure	injuries & poisonings	rs189447480	0.554	0.319	1.741	8.224E-02	logistic	423583	281	423302	0.035	0.010
443.9	Peripheral vascular disease, unspecified	circulatory system	rs189447480	0.188	0.108	1.207	8.263E-02	logistic	409046	3577	405469	0.200	0.010
79	Viral infection	infectious diseases	rs189447480	0.325	0.187	1.383	8.325E-02	logistic	416221	1017	415204	0.078	0.010
364.5	Corneal dystrophy	sense organs	rs189447480	-0.993	0.578	0.370	8.591E-02	logistic	398152	389	397763	0.083	0.010
706	Diseases of sebaceous glands	dermatologic	rs189447480	0.277	0.162	1.320	8.610E-02	logistic	413434	1435	411999	0.086	0.010
80	Postoperative infection	infectious diseases	rs189447480	0.245	0.143	1.278	8.628E-02	logistic	414741	1897	412844	0.295	0.010
300.9	Posttraumatic stress disorder	mental disorders	rs189447480	-1.701	1.001	0.183	8.931E-02	logistic	381881	259	381622	0.460	0.010
364	Corneal opacity and other disorders of cornea	sense organs	rs189447480	-0.334	0.197	0.716	9.074E-02	logistic	399513	1750	397763	0.029	0.010
740.11	Osteoarthrosis, localized, primary	musculoskeletal	rs189447480	-0.179	0.106	0.836	9.211E-02	logistic	351108	5250	345858	0.198	0.010
368.9	Subjective visual disturbances	sense organs	rs189447480	-0.755	0.448	0.470	9.212E-02	logistic	416057	512	415545	0.169	0.010
426.24	Atrioventricular block, complete	circulatory system	rs189447480	0.318	0.191	1.375	9.493E-02	logistic	372833	985	371848	0.010	0.010
290.11	Alzheimer's disease	mental disorders	rs189447480	0.216	0.130	1.242	9.620E-02	logistic	407367	2394	404973	0.396	0.010
159.2	Malignant neoplasm of small intestine, including duodenum	neoplasms	rs189447480	-1.664	1.001	0.189	9.635E-02	logistic	394117	255	393862	0.121	0.010
590	Pyelonephritis	genitourinary	rs189447480	0.272	0.166	1.313	1.010E-01	logistic	378294	1367	376927	0.080	0.010
202.24	Large cell lymphoma	neoplasms	rs189447480	-0.422	0.259	0.656	1.037E-01	logistic	416717	1104	415613	0.051	0.010
300.13	Phobia	mental disorders	rs189447480	0.385	0.238	1.470	1.057E-01	logistic	382219	597	381622	0.366	0.010
367.2	Astigmatism	sense organs	rs189447480	-0.216	0.134	0.806	1.059E-01	logistic	414418	3412	411006	0.273	0.010
599.2	Retention of urine	genitourinary	rs189447480	0.130	0.081	1.139	1.065E-01	logistic	396988	6882	390106	0.128	0.010
41.1	Staphylococcus infections	infectious diseases	rs189447480	-0.266	0.165	0.767	1.082E-01	logistic	398981	2334	396647	0.189	0.010
377	Disorders of optic nerve and visual pathways	sense organs	rs189447480	-0.929	0.579	0.395	1.082E-01	logistic	412293	365	411928	0.124	0.010
586.1	Anatomical abnormatilies of kidney and ureters	genitourinary	rs189447480	-1.607	1.001	0.200	1.083E-01	logistic	381301	239	381062	0.256	0.010
433.31	Transient cerebral ischemia	circulatory system	rs189447480	0.281	0.176	1.324	1.099E-01	logistic	403901	1216	402685	0.010	0.010
395.3	Nonrheumatic tricuspid valve disorders	circulatory system	rs189447480	-1.598	1.001	0.202	1.103E-01	logistic	401653	239	401414	0.077	0.010
525	Other diseases of the teeth and supporting structures	digestive	rs189447480	-0.709	0.448	0.492	1.134E-01	logistic	410027	491	409536	0.408	0.010
747.11	Cardiac shunt/ heart septal defect	congenital anomalies	rs189447480	0.424	0.270	1.528	1.158E-01	logistic	422041	446	421595	0.014	0.010
564.1	Irritable Bowel Syndrome	digestive	rs189447480	-0.174	0.112	0.840	1.211E-01	logistic	339145	4660	334485	0.366	0.010
430.2	Intracerebral hemorrhage	circulatory system	rs189447480	-0.401	0.259	0.670	1.217E-01	logistic	403773	1088	402685	0.011	0.010
198.7	Secondary malignant neoplasm of skin	neoplasms	rs189447480	0.515	0.337	1.674	1.257E-01	logistic	386268	262	386006	0.600	0.010
301	Personality disorders	mental disorders	rs189447480	-0.885	0.579	0.413	1.265E-01	logistic	381968	346	381622	0.352	0.010
456	Chronic venous insufficiency [CVI]	circulatory system	rs189447480	-1.078	0.707	0.340	1.274E-01	logistic	381250	284	380966	0.046	0.010
681.1	Cellulitis and abscess of fingers/toes	dermatologic	rs189447480	0.543	0.357	1.722	1.277E-01	logistic	418724	227	418497	0.195	0.010
706.2	Sebaceous cyst	dermatologic	rs189447480	0.252	0.166	1.287	1.284E-01	logistic	413393	1394	411999	0.088	0.010
586.11	Small kidney	genitourinary	rs189447480	-1.520	1.001	0.219	1.290E-01	logistic	381281	219	381062	0.257	0.010
858	Complication of internal orthopedic device	injuries & poisonings	rs189447480	-0.358	0.237	0.699	1.307E-01	logistic	417889	1237	416652	0.029	0.010
172.3	Carcinoma in situ of skin	neoplasms	rs189447480	-0.751	0.501	0.472	1.340E-01	logistic	407616	408	407208	0.081	0.010
747.1	Cardiac congenital anomalies	congenital anomalies	rs189447480	0.296	0.198	1.344	1.349E-01	logistic	422536	941	421595	0.005	0.010
611	Abnormal findings on mammogram or breast exam	genitourinary	rs189447480	0.501	0.337	1.651	1.363E-01	logistic	415016	264	414752	0.067	0.010
611.3	Lump or mass in breast	genitourinary	rs189447480	0.501	0.337	1.651	1.363E-01	logistic	415016	264	414752	0.067	0.010
369	Infection of the eye	sense organs	rs189447480	0.416	0.280	1.516	1.365E-01	logistic	411210	417	410793	0.239	0.010
465	Acute upper respiratory infections of multiple or unspecified sites	respiratory	rs189447480	0.336	0.225	1.399	1.366E-01	logistic	420386	694	419692	0.000	0.010
695.4	Lupus (localized and systemic)	dermatologic	rs189447480	0.387	0.261	1.473	1.374E-01	logistic	403975	493	403482	0.052	0.010
331.1	Hydrocephalus	neurological	rs189447480	0.347	0.238	1.415	1.436E-01	logistic	399668	621	399047	0.078	0.010
444	Arterial embolism and thrombosis	circulatory system	rs189447480	0.321	0.220	1.379	1.442E-01	logistic	406215	746	405469	0.155	0.010
389.1	Sensorineural hearing loss	sense organs	rs189447480	-0.461	0.317	0.630	1.459E-01	logistic	409063	766	408297	0.038	0.010
214	Lipoma	neoplasms	rs189447480	-0.365	0.251	0.694	1.465E-01	logistic	415339	1112	414227	0.076	0.010
290.1	Dementias	mental disorders	rs189447480	0.147	0.101	1.158	1.466E-01	logistic	409256	4283	404973	0.419	0.010
614.1	Pelvic peritoneal adhesions, female (postoperative) (postinfection	genitourinary	rs189447480	-0.727	0.502	0.483	1.473E-01	logistic	217833	395	217438	0.633	0.010
599.3	Dysuria	genitourinary	rs189447480	-0.836	0.579	0.434	1.487E-01	logistic	390438	332	390106	0.001	0.010
204.21	Myeloid leukemia, acute	neoplasms	rs189447480	0.352	0.244	1.421	1.503E-01	logistic	416195	582	415613	0.060	0.010
394	Rheumatic disease of the heart valves	circulatory system	rs189447480	0.137	0.096	1.147	1.503E-01	logistic	406218	4804	401414	0.087	0.010
596	Other disorders of bladder	genitourinary	rs189447480	-0.137	0.095	0.872	1.521E-01	logistic	407805	6251	401554	0.060	0.010
471	Nasal polyps	respiratory	rs189447480	0.272	0.191	1.313	1.532E-01	logistic	397549	1032	396517	0.157	0.010
721.1	Spondylosis without myelopathy	musculoskeletal	rs189447480	0.230	0.162	1.259	1.538E-01	logistic	410174	1504	408670	0.119	0.010
204.2	Myeloid leukemia	neoplasms	rs189447480	0.313	0.220	1.368	1.540E-01	logistic	416360	747	415613	0.063	0.010
751	Genitourinary congenital anomalies	congenital anomalies	rs189447480	-0.413	0.290	0.662	1.543E-01	logistic	421129	876	420253	0.030	0.010
345.11	Generalized convulsive epilepsy	neurological	rs189447480	-0.714	0.501	0.490	1.547E-01	logistic	399441	394	399047	0.064	0.010
368.91	Psychophysical visual disturbances	sense organs	rs189447480	-1.006	0.708	0.366	1.556E-01	logistic	415808	263	415545	0.169	0.010
574	Cholelithiasis and cholecystitis	digestive	rs189447480	-0.100	0.071	0.905	1.574E-01	logistic	406113	11066	395047	0.172	0.010
283	Acquired hemolytic anemias	hematopoietic	rs189447480	0.535	0.381	1.708	1.600E-01	logistic	382649	200	382449	0.149	0.010

ST31; PheWAS results for the lead cis-pQTLs of the 4 proteins prioritized (ACOX1, FGF5, FURIN, and MST1).
SNP = Single Nucleotide Polymorphism; SE = Standard error HWE_p = P-value for Hardy-Weinberg equilibrium test

Phenotype	Description	Group	SNP	Beta	SE	Odds tatio	P-value	Type	n_total	n_cases	n_controls	HWE_p	allele_freq
624.9	stress incontinence, female	genitourinary	rs189447480	-0.258	0.184	0.772	1.600E-01	logistic	220155	1871	218284	0.390	0.010
965.1	Opiates and related narcotics causing adverse effects in therapeutic use	injuries & poisonings	rs189447480	0.190	0.135	1.209	1.606E-01	logistic	369447	2274	367173	0.008	0.010
189.21	Malignant neoplasm of bladder	neoplasms	rs189447480	-0.197	0.141	0.821	1.628E-01	logistic	420210	3023	417187	0.200	0.010
751.2	Congenital anomalies of urinary system	congenital anomalies	rs189447480	-0.440	0.317	0.644	1.658E-01	logistic	421003	750	420253	0.031	0.010
574.12	Cholelithiasis with other cholecystitis	digestive	rs189447480	-0.278	0.201	0.757	1.664E-01	logistic	396636	1589	395047	0.188	0.010
572	Ascites (non malignant)	digestive	rs189447480	-0.235	0.170	0.790	1.667E-01	logistic	402409	2135	400274	0.029	0.010
571.81	Portal hypertension	digestive	rs189447480	-0.401	0.290	0.670	1.668E-01	logistic	401135	861	400274	0.138	0.010
741	Symptoms and disorders of the joints	musculoskeletal	rs189447480	0.218	0.158	1.243	1.670E-01	logistic	416612	1602	415010	0.057	0.010
428	Congestive heart failure; nonhypertensive	circulatory system	rs189447480	0.104	0.075	1.110	1.671E-01	logistic	417859	8156	409703	0.058	0.010
374.3	Ptosis of eyelid	sense organs	rs189447480	-0.326	0.237	0.722	1.690E-01	logistic	411997	1204	410793	0.235	0.010
622.2	Mucous polyp of cervix	genitourinary	rs189447480	0.490	0.357	1.632	1.705E-01	logistic	213810	238	213572	0.596	0.010
211	Benign neoplasm of other parts of digestive system	neoplasms	rs189447480	0.152	0.111	1.164	1.707E-01	logistic	400517	3467	397050	0.024	0.010
8	Intestinal infection	infectious diseases	rs189447480	-0.246	0.181	0.782	1.726E-01	logistic	419231	1915	417316	0.037	0.010
585.1	Acute renal failure	genitourinary	rs189447480	-0.088	0.065	0.916	1.731E-01	logistic	394375	13313	381062	0.158	0.010
428.1	Congestive heart failure (CHF) NOS	circulatory system	rs189447480	0.143	0.105	1.154	1.737E-01	logistic	413649	3946	409703	0.002	0.010
474	Acute and chronic tonsillitis	respiratory	rs189447480	-0.682	0.502	0.506	1.740E-01	logistic	396895	378	396517	0.179	0.010
521	Diseases of hard tissues of teeth	digestive	rs189447480	-0.410	0.303	0.664	1.759E-01	logistic	410337	801	409536	0.411	0.010
389.4	Tinnitus	sense organs	rs189447480	0.364	0.269	1.439	1.766E-01	logistic	408770	473	408297	0.029	0.010
204.4	Multiple myeloma	neoplasms	rs189447480	-0.329	0.244	0.720	1.769E-01	logistic	416757	1144	415613	0.053	0.010
473	Diseases of the larynx and vocal cords	respiratory	rs189447480	-0.302	0.225	0.739	1.785E-01	logistic	397819	1302	396517	0.171	0.010
580.1	Glomerulonephritis	genitourinary	rs189447480	0.350	0.260	1.419	1.786E-01	logistic	381574	512	381062	0.270	0.010
592.12	Chronic cystitis	genitourinary	rs189447480	-0.949	0.708	0.387	1.806E-01	logistic	377176	249	376927	0.049	0.010
571.6	Primary biliary cirrhosis	digestive	rs189447480	-0.772	0.579	0.462	1.819E-01	logistic	400586	312	400274	0.134	0.010
198.6	Secondary malignancy of bone	neoplasms	rs189447480	-0.146	0.110	0.864	1.825E-01	logistic	390762	4756	386006	0.535	0.010
454	Varicose veins	circulatory system	rs189447480	0.126	0.095	1.135	1.829E-01	logistic	385827	4861	380966	0.087	0.010
272.9	Unspecified disorder of lipoid metabolism	endocrine/metabolic	rs189447480	0.423	0.319	1.526	1.845E-01	logistic	356085	320	355765	0.020	0.010
315	Develomental delays and disorders	mental disorders	rs189447480	0.306	0.231	1.358	1.853E-01	logistic	422717	680	422037	0.020	0.010
427.11	Paroxysmal supraventricular tachycardia	circulatory system	rs189447480	-0.225	0.170	0.799	1.870E-01	logistic	373959	2111	371848	0.236	0.010
180.1	Cervical cancer	neoplasms	rs189447480	0.188	0.143	1.207	1.892E-01	logistic	197916	2012	195904	0.222	0.010
574.11	Cholelithiasis with acute cholecystitis	digestive	rs189447480	0.255	0.194	1.290	1.895E-01	logistic	396059	1012	395047	0.198	0.010
332	Parkinson's disease	neurological	rs189447480	0.159	0.121	1.172	1.902E-01	logistic	401966	2919	399047	0.009	0.010
327	Sleep disorders	neurological	rs189447480	0.328	0.252	1.389	1.924E-01	logistic	413949	565	413384	0.011	0.010
274.2	Crystal arthropathies	endocrine/metabolic	rs189447480	-0.921	0.708	0.398	1.936E-01	logistic	416033	243	415790	0.078	0.010
276.13	Hyperpotassemia	endocrine/metabolic	rs189447480	-0.245	0.190	0.783	1.985E-01	logistic	397299	1727	395572	0.297	0.010
454.1	Varicose veins of lower extremity	circulatory system	rs189447480	0.126	0.098	1.134	2.000E-01	logistic	385486	4520	380966	0.093	0.010
721	Spondylosis and allied disorders	musculoskeletal	rs189447480	0.188	0.147	1.207	2.005E-01	logistic	410559	1889	408670	0.124	0.010
275.5	Disorders of calcium/phosphorus metabolism	endocrine/metabolic	rs189447480	-0.206	0.161	0.814	2.023E-01	logistic	418502	2317	416185	0.180	0.010
155.1	Malignant neoplasm of liver, primary	neoplasms	rs189447480	0.405	0.318	1.499	2.035E-01	logistic	394187	325	393862	0.232	0.010
339	Other headache syndromes	neurological	rs189447480	0.130	0.102	1.139	2.039E-01	logistic	409356	4169	405187	0.146	0.010
284	Aplastic anemia	hematopoietic	rs189447480	0.285	0.225	1.330	2.051E-01	logistic	383179	730	382449	0.139	0.010
384	Other disorders of tympanic membrane	sense organs	rs189447480	0.383	0.304	1.466	2.082E-01	logistic	419080	364	418716	0.018	0.010
571.5	Other chronic nonalcoholic liver disease	digestive	rs189447480	-0.143	0.114	0.867	2.107E-01	logistic	404607	4333	400274	0.175	0.010
427.6	Premature beats	circulatory system	rs189447480	-0.443	0.355	0.642	2.117E-01	logistic	372449	601	371848	0.222	0.010
420	Carditis	circulatory system	rs189447480	0.173	0.139	1.189	2.125E-01	logistic	418942	2172	416770	0.184	0.010
425.1	Primary/intrinsic cardiomyopathies	circulatory system	rs189447480	0.185	0.149	1.203	2.141E-01	logistic	418636	1866	416770	0.179	0.010
585.31	Renal dialysis	genitourinary	rs189447480	-0.394	0.318	0.674	2.148E-01	logistic	381775	713	381062	0.261	0.010
180	Cervical cancer and dysplasia	neoplasms	rs189447480	0.161	0.130	1.174	2.161E-01	logistic	198422	2518	195904	0.231	0.010
433	Cerebrovascular disease	circulatory system	rs189447480	0.080	0.065	1.083	2.174E-01	logistic	414008	11323	402685	0.089	0.010
386.9	Dizziness and giddiness (Light-headedness and vertigo)	sense organs	rs189447480	-0.171	0.138	0.843	2.181E-01	logistic	412711	3046	409665	0.339	0.010
363	Chorioretinal inflammations, scars, and other disorders of choroid	sense organs	rs189447480	-0.872	0.708	0.418	2.183E-01	logistic	397993	230	397763	0.084	0.010
334.2	Anterior horn cell disease	neurological	rs189447480	0.343	0.279	1.410	2.189E-01	logistic	399497	450	399047	0.074	0.010
585	Renal failure	genitourinary	rs189447480	-0.061	0.050	0.941	2.209E-01	logistic	403986	22924	381062	0.155	0.010
619.4	Noninflammatory disorders of vagina	genitourinary	rs189447480	-0.867	0.709	0.420	2.212E-01	logistic	217194	228	216966	0.365	0.010
244.1	Secondary hypothyroidism	endocrine/metabolic	rs189447480	0.221	0.181	1.247	2.233E-01	logistic	393443	1213	392230	0.453	0.010
626.8	Infertility, female	genitourinary	rs189447480	0.412	0.339	1.510	2.243E-01	logistic	193029	282	192747	0.913	0.010
477	Epistaxis or throat hemorrhage	respiratory	rs189447480	0.212	0.175	1.237	2.259E-01	logistic	397807	1290	396517	0.154	0.010
426.21	First degree AV block	circulatory system	rs189447480	0.183	0.151	1.200	2.259E-01	logistic	373661	1813	371848	0.128	0.010
585.33	Chronic Kidney Disease, Stage III	genitourinary	rs189447480	0.095	0.079	1.100	2.260E-01	logistic	388537	7475	381062	0.251	0.010
573.5	Jaundice (not of newborn)	digestive	rs189447480	0.279	0.231	1.322	2.275E-01	logistic	400970	696	400274	0.027	0.010
364.4	Corneal degenerations	sense organs	rs189447480	-0.285	0.237	0.752	2.290E-01	logistic	398917	1154	397763	0.092	0.010
790	Nonspecific findings on examination of blood	symptoms	rs189447480	0.424	0.356	1.529	2.333E-01	logistic	422095	255	421840	0.008	0.010
289.3	Personal history of diseases of blood and blood-forming organs	hematopoietic	rs189447480	0.399	0.336	1.491	2.346E-01	logistic	408604	295	408309	0.173	0.010
946	Anaphylactic shock NOS	injuries & poisonings	rs189447480	-0.841	0.709	0.431	2.356E-01	logistic	415210	223	414987	0.039	0.010
352.1	Trigeminal nerve disorders [CN5]	neurological	rs189447480	0.318	0.269	1.375	2.374E-01	logistic	402912	497	402415	0.021	0.010
752	Nervous system congenital anomalies	congenital anomalies	rs189447480	0.329	0.280	1.390	2.391E-01	logistic	422992	453	422539	0.031	0.010
550.6	Incisional hernia	digestive	rs189447480	0.327	0.279	1.387	2.409E-01	logistic	365851	457	365394	0.387	0.010
780	Hypothermia/Chills	symptoms	rs189447480	-0.831	0.709	0.436	2.412E-01	logistic	423421	221	423200	0.029	0.010
751.22	Other specified congenital anomalies of kidney	congenital anomalies	rs189447480	-0.588	0.501	0.556	2.413E-01	logistic	420601	348	420253	0.034	0.010
507	Pleurisy; pleural effusion	respiratory	rs189447480	0.081	0.069	1.084	2.423E-01	logistic	400937	9880	391057	0.006	0.010
227.1	Benign neoplasm of adrenal gland	neoplasms	rs189447480	0.414	0.356	1.513	2.453E-01	logistic	421144	257	420887	0.236	0.010
317.1	Alcoholism	mental disorders	rs189447480	0.125	0.107	1.133	2.461E-01	logistic	407082	3789	403293	0.027	0.010

ST31; PheWAS results for the lead cis-pQTLs of the 4 proteins prioritized (ACOX1, FGF5, FURIN, and MST1).

SNP = Single Nucleotide Polymorphism; SE = Standard error HWE_p = P-value for Hardy-Weinberg equilibrium test

Phenotype	Description	Group	SNP	Beta	SE	Odds tatio	P-value	Type	n_total	n_cases	n_controls	HWE_p	allele_freq
798	Malaise and fatigue	symptoms	rs189447480	0.118	0.102	1.125	2.464E-01	logistic	418584	4264	414320	0.020	0.010
165	Cancer within the respiratory system	neoplasms	rs189447480	-0.118	0.102	0.888	2.469E-01	logistic	422554	5325	417229	0.021	0.010
318	Tobacco use disorder	mental disorders	rs189447480	-0.122	0.105	0.885	2.474E-01	logistic	408298	5005	403293	0.203	0.010
619.3	Noninflammatory disorders of cervix	genitourinary	rs189447480	-0.578	0.502	0.561	2.494E-01	logistic	217308	342	216966	0.366	0.010
401.1	Essential hypertension	circulatory system	rs189447480	0.031	0.027	1.032	2.503E-01	logistic	389876	99213	290663	0.136	0.010
571.8	Liver abscess and sequelae of chronic liver disease	digestive	rs189447480	-0.230	0.201	0.795	2.532E-01	logistic	401788	1514	400274	0.146	0.010
389.3	Degenerative and vascular disorders of ear	sense organs	rs189447480	-0.572	0.501	0.564	2.536E-01	logistic	408638	341	408297	0.040	0.010
724.2	Disorders of coccyx	musculoskeletal	rs189447480	-0.809	0.709	0.445	2.542E-01	logistic	408885	215	408670	0.210	0.010
275.1	Disorders of iron metabolism	hematopoietic	rs189447480	0.216	0.191	1.242	2.562E-01	logistic	417274	1089	416185	0.058	0.010
281.13	Folate-deficiency anemia	hematopoietic	rs189447480	0.343	0.303	1.410	2.576E-01	logistic	382829	380	382449	0.146	0.010
800.2	Fracture of unspecified part of femur	injuries & poisonings	rs189447480	-0.800	0.708	0.449	2.581E-01	logistic	396592	214	396378	0.060	0.010
710	Osteomyelitis, periostitis, and other infections involving bone	musculoskeletal	rs189447480	-0.651	0.579	0.521	2.603E-01	logistic	412326	278	412048	0.005	0.010
961.1	Poisoning/allergy of sulfonamides	injuries & poisonings	rs189447480	0.274	0.244	1.315	2.627E-01	logistic	367807	634	367173	0.021	0.010
401	Hypertension	circulatory system	rs189447480	0.030	0.027	1.031	2.629E-01	logistic	390097	99434	290663	0.135	0.010
287	Purpura and other hemorrhagic conditions	hematopoietic	rs189447480	0.150	0.135	1.162	2.649E-01	logistic	419896	2343	417553	0.026	0.010
608	Other disorders of male genital organs	genitourinary	rs189447480	-0.338	0.303	0.713	2.653E-01	logistic	162533	755	161778	0.200	0.010
293	Symptoms involving head and neck	mental disorders	rs189447480	0.310	0.280	1.364	2.666E-01	logistic	420175	464	419711	0.014	0.010
165.1	Cancer of bronchus; lung	neoplasms	rs189447480	-0.128	0.115	0.880	2.668E-01	logistic	421452	4223	417229	0.009	0.010
350.1	Abnormal involuntary movements	neurological	rs189447480	0.244	0.220	1.276	2.669E-01	logistic	421010	800	420210	0.010	0.010
521.1	Dental caries	digestive	rs189447480	-0.335	0.303	0.715	2.682E-01	logistic	410280	744	409536	0.411	0.010
433.12	Cerebral atherosclerosis	circulatory system	rs189447480	0.352	0.318	1.422	2.691E-01	logistic	403029	344	402685	0.008	0.010
743.2	Pathologic fracture	musculoskeletal	rs189447480	0.226	0.206	1.254	2.718E-01	logistic	422164	930	421234	0.003	0.010
292.6	Hallucinations	mental disorders	rs189447480	0.350	0.319	1.419	2.723E-01	logistic	405317	344	404973	0.364	0.010
745	Pain in joint	musculoskeletal	rs189447480	-0.139	0.127	0.870	2.732E-01	logistic	414363	3501	410862	0.362	0.010
426.2	Atrioventricular [AV] block	circulatory system	rs189447480	0.119	0.109	1.126	2.755E-01	logistic	375580	3732	371848	0.097	0.010
782.3	Edema	symptoms	rs189447480	-0.167	0.154	0.846	2.758E-01	logistic	419509	2451	417058	0.437	0.010
411.8	Other chronic ischemic heart disease, unspecified	circulatory system	rs189447480	0.057	0.052	1.059	2.784E-01	logistic	389207	18657	370550	0.139	0.010
578	Gastrointestinal hemorrhage	digestive	rs189447480	0.076	0.070	1.079	2.787E-01	logistic	392729	9426	383303	0.008	0.010
695	Erythematous conditions	dermatologic	rs189447480	0.164	0.152	1.179	2.794E-01	logistic	407007	1801	405206	0.135	0.010
365	Glaucoma	sense organs	rs189447480	0.085	0.079	1.089	2.795E-01	logistic	405209	7446	397763	0.076	0.010
586	Other disorders of the kidney and ureters	genitourinary	rs189447480	-0.131	0.121	0.878	2.795E-01	logistic	384904	3842	381062	0.174	0.010
261.2	Vitamin B-complex deficiencies	endocrine/metabolic	rs189447480	-0.202	0.187	0.817	2.801E-01	logistic	413809	1712	412097	0.141	0.010
916	Contusion	injuries & poisonings	rs189447480	-0.226	0.210	0.798	2.807E-01	logistic	421588	1393	420195	0.007	0.010
701.2	Scar conditions and fibrosis of skin	dermatologic	rs189447480	-0.484	0.449	0.616	2.810E-01	logistic	415576	390	415186	0.028	0.010
614	Inflammatory diseases of female pelvic organs	genitourinary	rs189447480	-0.212	0.198	0.809	2.831E-01	logistic	218979	1541	217438	0.644	0.010
425	Cardiomyopathy	circulatory system	rs189447480	0.160	0.149	1.173	2.831E-01	logistic	418683	1913	416770	0.179	0.010
496.3	Bronchiectasis	respiratory	rs189447480	0.116	0.109	1.123	2.839E-01	logistic	402578	3769	398809	0.212	0.010
743	Osteoporosis, osteopenia and pathological fracture	musculoskeletal	rs189447480	0.200	0.187	1.222	2.847E-01	logistic	422387	1153	421234	0.007	0.010
367.1	Myopia	sense organs	rs189447480	0.187	0.176	1.206	2.862E-01	logistic	412331	1325	411006	0.381	0.010
276.42	Alkalosis	endocrine/metabolic	rs189447480	0.358	0.336	1.430	2.869E-01	logistic	395878	306	395572	0.403	0.010
726.3	Bursitis	musculoskeletal	rs189447480	-0.753	0.709	0.471	2.879E-01	logistic	389380	206	389174	0.319	0.010
470	Septal Deviations/Turbinate Hypertrophy	respiratory	rs189447480	-0.202	0.190	0.817	2.886E-01	logistic	398163	1646	396517	0.165	0.010
565.1	Anal and rectal polyp	digestive	rs189447480	-0.154	0.145	0.857	2.887E-01	logistic	386554	2715	383839	0.019	0.010
289	Other diseases of blood and blood-forming organs	hematopoietic	rs189447480	0.233	0.220	1.263	2.889E-01	logistic	409120	811	408309	0.181	0.010
573	Other disorders of liver	digestive	rs189447480	-0.180	0.170	0.836	2.908E-01	logistic	402294	2020	400274	0.030	0.010
564.9	Personal history of diseases of digestive system	digestive	rs189447480	0.062	0.059	1.064	2.942E-01	logistic	348229	13744	334485	0.396	0.010
427.5	Arrhythmia (cardiac) NOS	circulatory system	rs189447480	-0.395	0.379	0.673	2.974E-01	logistic	372348	500	371848	0.222	0.010
567	Peritonitis and retroperitoneal infections	digestive	rs189447480	-0.315	0.303	0.730	2.977E-01	logistic	384568	729	383839	0.002	0.010
295	Schizophrenia and other psychotic disorders	mental disorders	rs189447480	-0.290	0.279	0.748	2.979E-01	logistic	382460	838	381622	0.466	0.010
565	Anal and rectal conditions	digestive	rs189447480	0.082	0.078	1.085	2.981E-01	logistic	391353	7514	383839	0.015	0.010
368.4	Visual field defects	sense organs	rs189447480	-0.426	0.410	0.653	2.981E-01	logistic	415988	443	415545	0.171	0.010
280	Iron deficiency anemias	hematopoietic	rs189447480	-0.080	0.077	0.923	2.992E-01	logistic	391534	9085	382449	0.295	0.010
290.13	Senile dementia	mental disorders	rs189447480	0.142	0.138	1.153	3.023E-01	logistic	407253	2280	404973	0.390	0.010
575.7	Other disorders of gallbladder	digestive	rs189447480	-0.342	0.335	0.711	3.075E-01	logistic	395657	610	395047	0.299	0.010
556	Ulceration of the lower GI tract	digestive	rs189447480	0.310	0.304	1.364	3.078E-01	logistic	334879	394	334485	0.453	0.010
587	Kidney replaced by transpant	genitourinary	rs189447480	-0.385	0.379	0.681	3.103E-01	logistic	381556	494	381062	0.260	0.010
379	Other disorders of eye	sense organs	rs189447480	0.200	0.198	1.221	3.116E-01	logistic	412963	1035	411928	0.144	0.010
496	Chronic airway obstruction	respiratory	rs189447480	0.053	0.053	1.055	3.117E-01	logistic	416701	17892	398809	0.041	0.010
781	Symptoms involving nervous and musculoskeletal system	symptoms	rs189447480	0.078	0.077	1.081	3.122E-01	logistic	419375	7893	411482	0.112	0.010
411.3	Angina pectoris	circulatory system	rs189447480	-0.058	0.058	0.943	3.152E-01	logistic	387093	16543	370550	0.182	0.010
580	Nephritis; nephrosis; renal sclerosis	genitourinary	rs189447480	0.194	0.194	1.214	3.171E-01	logistic	382137	1075	381062	0.277	0.010
378.5	Paralytic strabismus	sense organs	rs189447480	0.355	0.356	1.426	3.189E-01	logistic	412201	273	411928	0.132	0.010
728	Disorders of muscle, ligament, and fascia	musculoskeletal	rs189447480	-0.576	0.579	0.562	3.200E-01	logistic	389432	258	389174	0.320	0.010
395.2	Nonrheumatic aortic valve disorders	circulatory system	rs189447480	-0.105	0.106	0.900	3.205E-01	logistic	406373	4959	401414	0.007	0.010
800.1	Fracture of neck of femur	injuries & poisonings	rs189447480	0.143	0.144	1.153	3.225E-01	logistic	398432	2054	396378	0.025	0.010
446.5	Giant cell arteritis	circulatory system	rs189447480	-0.330	0.335	0.719	3.246E-01	logistic	406071	602	405469	0.142	0.010
577.2	Chronic pancreatitis	digestive	rs189447480	-0.329	0.335	0.720	3.257E-01	logistic	418611	603	418008	0.016	0.010
158	Neoplasm of unspecified nature of digestive system	neoplasms	rs189447480	-0.440	0.449	0.644	3.263E-01	logistic	394238	376	393862	0.118	0.010
293.1	Swelling, mass, or lump in head and neck [Space-occupying lesion, intracranial NOS]	mental disorders	rs189447480	-0.491	0.502	0.612	3.276E-01	logistic	420026	315	419711	0.024	0.010
217.1	Nevus, non-neoplastic	neoplasms	rs189447480	0.347	0.356	1.415	3.295E-01	logistic	414233	275	413958	0.088	0.010
286.12	Congenital deficiency of other clotting factors (including factor VII)	hematopoietic	rs189447480	0.370	0.381	1.448	3.314E-01	logistic	417788	235	417553	0.060	0.010

ST31; PheWAS results for the lead cis-pQTLs of the 4 proteins prioritized (ACOX1, FGF5, FURIN, and MST1).

SNP = Single Nucleotide Polymorphism; SE = Standard error HWE_p = P-value for Hardy-Weinberg equilibrium test

Phenotype	Description	Group	SNP	Beta	SE	Odds tatio	P-value	Type	n_total	n_cases	n_controls	HWE_p	allele_freq
38.1	Gram negative septicemia	infectious diseases	rs189447480	-0.485	0.502	0.616	3.337E-01	logistic	396961	314	396647	0.174	0.010
509.1	Respiratory failure	respiratory	rs189447480	-0.109	0.112	0.897	3.339E-01	logistic	395437	4380	391057	0.485	0.010
733	Other disorders of bone and cartilage	musculoskeletal	rs189447480	0.203	0.210	1.225	3.343E-01	logistic	399585	913	398672	0.318	0.010
296.22	Major depressive disorder	mental disorders	rs189447480	0.054	0.056	1.056	3.358E-01	logistic	396820	15198	381622	0.208	0.010
444.1	Arterial embolism and thrombosis of lower extremity artery	circulatory system	rs189447480	0.278	0.291	1.320	3.393E-01	logistic	405915	446	405469	0.147	0.010
531.2	Gastric ulcer	digestive	rs189447480	-0.178	0.187	0.837	3.397E-01	logistic	413012	1673	411339	0.025	0.010
357	Inflammatory and toxic neuropathy	neurological	rs189447480	-0.162	0.170	0.850	3.398E-01	logistic	421163	1995	419168	0.017	0.010
411.1	Unstable angina (intermediate coronary syndrome)	circulatory system	rs189447480	0.104	0.110	1.110	3.431E-01	logistic	374269	3719	370550	0.174	0.010
446.9	Arteritis NOS	circulatory system	rs189447480	0.302	0.319	1.352	3.437E-01	logistic	405830	361	405469	0.146	0.010
702.1	Actinic keratosis	dermatologic	rs189447480	-0.245	0.259	0.783	3.452E-01	logistic	413997	925	413072	0.033	0.010
401.22	Hypertensive chronic kidney disease	circulatory system	rs189447480	0.194	0.206	1.215	3.452E-01	logistic	291632	969	290663	0.410	0.010
159	Malignant neoplasm of other and ill-defined sites within the digestive organs and peritoneum	neoplasms	rs189447480	-0.252	0.268	0.777	3.474E-01	logistic	394736	874	393862	0.113	0.010
433.5	Cerebral aneurysm	circulatory system	rs189447480	0.272	0.291	1.312	3.502E-01	logistic	403130	445	402685	0.006	0.010
614.3	Pelvic inflammatory disease (PID)	genitourinary	rs189447480	0.356	0.382	1.428	3.516E-01	logistic	217675	237	217438	0.638	0.010
578.9	Hemorrhage of gastrointestinal tract	digestive	rs189447480	0.114	0.123	1.120	3.535E-01	logistic	386245	2942	383303	0.161	0.010
530.12	Ulcer of esophagus	digestive	rs189447480	0.120	0.130	1.128	3.541E-01	logistic	359422	2634	356788	0.058	0.010
367.9	Blindness and low vision	sense organs	rs189447480	0.199	0.215	1.220	3.547E-01	logistic	411881	875	411006	0.262	0.010
367.8	Hypermetropia	sense organs	rs189447480	0.329	0.356	1.390	3.554E-01	logistic	411285	279	411006	0.365	0.010
41.2	Streptococcus infection	infectious diseases	rs189447480	-0.182	0.197	0.834	3.563E-01	logistic	398155	1508	396647	0.186	0.010
189.2	Cancer of bladder	neoplasms	rs189447480	-0.119	0.129	0.888	3.573E-01	logistic	420532	3345	417187	0.208	0.010
504	Other alveolar and parietoalveolar pneumonopathy	respiratory	rs189447480	0.224	0.244	1.251	3.592E-01	logistic	391718	661	391057	0.460	0.010
287.3	Thrombocytopenia	hematopoietic	rs189447480	0.128	0.140	1.137	3.593E-01	logistic	419777	2224	417553	0.029	0.010
386.2	Peripheral or central vertigo	sense organs	rs189447480	0.231	0.252	1.259	3.598E-01	logistic	410281	616	409665	0.326	0.010
512.8	Cough	respiratory	rs189447480	0.150	0.164	1.161	3.605E-01	logistic	406184	1585	404599	0.519	0.010
601.1	Prostatitis	genitourinary	rs189447480	-0.277	0.303	0.758	3.613E-01	logistic	157164	703	156461	0.125	0.010
695.42	Systemic lupus erythematosus	dermatologic	rs189447480	0.277	0.304	1.320	3.616E-01	logistic	403885	403	403482	0.056	0.010
599.5	Frequency of urination and polyuria	genitourinary	rs189447480	-0.244	0.269	0.783	3.630E-01	logistic	390965	859	390106	0.008	0.010
960.2	Allergy/adverse effect of penicillin	injuries & poisonings	rs189447480	0.047	0.052	1.048	3.637E-01	logistic	385833	18660	367173	0.264	0.010
601	Inflammatory diseases of prostate	genitourinary	rs189447480	-0.235	0.260	0.790	3.650E-01	logistic	157382	921	156461	0.129	0.010
252	Disorders of parathyroid gland	endocrine/metabolic	rs189447480	-0.199	0.219	0.820	3.650E-01	logistic	416485	1239	415246	0.035	0.010
280.1	Iron deficiency anemias, unspecified or not due to blood loss	hematopoietic	rs189447480	-0.070	0.078	0.932	3.677E-01	logistic	391238	8789	382449	0.296	0.010
726.1	Enthesopathy	musculoskeletal	rs189447480	0.103	0.116	1.109	3.736E-01	logistic	392491	3317	389174	0.366	0.010
961	Poisoning by other anti-infectives	injuries & poisonings	rs189447480	0.315	0.356	1.370	3.763E-01	logistic	367461	288	367173	0.028	0.010
695.2	Bullous dermatoses	dermatologic	rs189447480	0.363	0.411	1.438	3.766E-01	logistic	405408	202	405206	0.161	0.010
702	Degenerative skin conditions and other dermatoses	dermatologic	rs189447480	-0.155	0.175	0.857	3.772E-01	logistic	407215	1867	405348	0.050	0.010
395.1	Nonrheumatic mitral valve disorders	circulatory system	rs189447480	0.098	0.111	1.103	3.780E-01	logistic	405090	3676	401414	0.135	0.010
340	Migraine	neurological	rs189447480	0.116	0.132	1.123	3.781E-01	logistic	407732	2545	405187	0.174	0.010
800.3	Fracture of tibia and fibula	injuries & poisonings	rs189447480	0.229	0.260	1.257	3.791E-01	logistic	396955	577	396378	0.049	0.010
574.2	Calculus of bile duct	digestive	rs189447480	-0.509	0.579	0.601	3.792E-01	logistic	395287	240	395047	0.296	0.010
276.12	Hyposmolality and/or hyponatremia	endocrine/metabolic	rs189447480	0.084	0.096	1.088	3.793E-01	logistic	400551	4979	395572	0.239	0.010
720.1	Spinal stenosis of lumbar region	musculoskeletal	rs189447480	0.115	0.130	1.121	3.796E-01	logistic	411268	2598	408670	0.131	0.010
687	Symptoms affecting skin	dermatologic	rs189447480	0.280	0.319	1.323	3.803E-01	logistic	413083	367	412716	0.379	0.010
246	Other disorders of thyroid	endocrine/metabolic	rs189447480	-0.440	0.502	0.644	3.807E-01	logistic	392532	302	392230	0.435	0.010
250.2	Type 2 diabetes	endocrine/metabolic	rs189447480	0.039	0.044	1.039	3.823E-01	logistic	413206	26569	386637	0.138	0.010
727.4	Ganglion and cyst of synovium, tendon, and bursa	musculoskeletal	rs189447480	-0.326	0.380	0.722	3.899E-01	logistic	389641	467	389174	0.322	0.010
740.1	Osteoarthritis; localized	musculoskeletal	rs189447480	-0.038	0.045	0.962	3.901E-01	logistic	374099	28241	345858	0.118	0.010
288	Diseases of white blood cells	hematopoietic	rs189447480	-0.351	0.410	0.704	3.910E-01	logistic	408723	414	408309	0.168	0.010
316	Substance addiction and disorders	mental disorders	rs189447480	-0.385	0.449	0.680	3.911E-01	logistic	403645	352	403293	0.155	0.010
619	Noninflammatory female genital disorders	genitourinary	rs189447480	-0.136	0.160	0.873	3.950E-01	logistic	219175	2209	216966	0.391	0.010
857	Mechanical complication of unspecified genitourinary device, implant, and graft	injuries & poisonings	rs189447480	-0.162	0.190	0.851	3.952E-01	logistic	418243	1591	416652	0.021	0.010
871	Open wounds of extremities	injuries & poisonings	rs189447480	0.271	0.318	1.311	3.955E-01	logistic	420520	370	420150	0.008	0.010
790.6	Other abnormal blood chemistry	symptoms	rs189447480	-0.492	0.579	0.612	3.956E-01	logistic	422077	237	421840	0.002	0.010
70.4	Chronic hepatitis	infectious diseases	rs189447480	0.301	0.356	1.352	3.975E-01	logistic	415491	287	415204	0.062	0.010
427.9	Palpitations	circulatory system	rs189447480	-0.153	0.181	0.858	3.978E-01	logistic	373589	1741	371848	0.235	0.010
411.2	Myocardial infarction	circulatory system	rs189447480	0.050	0.059	1.051	3.996E-01	logistic	385085	14535	370550	0.198	0.010
241.2	Nontoxic multinodular goiter	endocrine/metabolic	rs189447480	-0.487	0.579	0.614	4.006E-01	logistic	392467	237	392230	0.434	0.010
427.1	Paroxysmal tachycardia, unspecified	circulatory system	rs189447480	-0.111	0.133	0.895	4.013E-01	logistic	374981	3133	371848	0.251	0.010
358.1	Myasthenia gravis	neurological	rs189447480	-0.421	0.502	0.656	4.014E-01	logistic	419463	295	419168	0.086	0.010
569	Other disorders of intestine	digestive	rs189447480	0.120	0.143	1.127	4.018E-01	logistic	385990	2151	383839	0.096	0.010
575.8	Other disorders of biliary tract	digestive	rs189447480	-0.198	0.237	0.821	4.042E-01	logistic	396103	1056	395047	0.304	0.010
415.21	Primary pulmonary hypertension	circulatory system	rs189447480	-0.372	0.449	0.689	4.070E-01	logistic	413189	351	412838	0.352	0.010
528	Diseases of the oral soft tissues, excluding lesions specific for gingiva and tongue	digestive	rs189447480	0.240	0.291	1.271	4.098E-01	logistic	418169	459	417710	0.063	0.010
225.1	Benign neoplasm of brain, cranial nerves, meninges	neoplasms	rs189447480	0.185	0.225	1.203	4.114E-01	logistic	421204	807	420397	0.010	0.010
277.4	Disorders of bilirubin excretion	endocrine/metabolic	rs189447480	-0.311	0.379	0.733	4.128E-01	logistic	420403	462	419941	0.116	0.010
601.12	Chronic prostatitis	genitourinary	rs189447480	-0.473	0.579	0.623	4.139E-01	logistic	156694	233	156461	0.119	0.010
292.4	Altered mental status	mental disorders	rs189447480	0.094	0.115	1.098	4.141E-01	logistic	408434	3461	404973	0.055	0.010
261	Vitamin deficiency	endocrine/metabolic	rs189447480	-0.096	0.117	0.909	4.153E-01	logistic	416032	3935	412097	0.047	0.010
300.1	Anxiety disorder	mental disorders	rs189447480	0.058	0.072	1.060	4.160E-01	logistic	390805	9183	381622	0.001	0.010
696.42	Psoriatic arthropathy	dermatologic	rs189447480	0.175	0.215	1.191	4.161E-01	logistic	400183	893	399290	0.008	0.010
379.2	Disorders of vitreous body	sense organs	rs189447480	-0.333	0.410	0.717	4.164E-01	logistic	412333	405	411928	0.127	0.010
724.1	Disorders of sacrum	musculoskeletal	rs189447480	-0.469	0.580	0.626	4.186E-01	logistic	408900	230	408670	0.211	0.010

ST31; PheWAS results for the lead cis-pQTLs of the 4 proteins prioritized (ACOX1, FGF5, FURIN, and MST1).

SNP = Single Nucleotide Polymorphism; SE = Standard error HWE_p = P-value for Hardy-Weinberg equilibrium test

Phenotype	Description	Group	SNP	Beta	SE	Odds tatio	P-value	Type	n_total	n_cases	n_controls	HWE_p	allele_freq
157	Pancreatic cancer	neoplasms	rs189447480	0.141	0.175	1.152	4.204E-01	logistic	395256	1394	393862	0.095	0.010
202	Cancer of other lymphoid, histiocytic tissue	neoplasms	rs189447480	-0.116	0.144	0.891	4.208E-01	logistic	418277	2664	415613	0.047	0.010
202.2	Non-Hodgkins lymphoma	neoplasms	rs189447480	-0.116	0.144	0.891	4.208E-01	logistic	418277	2664	415613	0.047	0.010
750.1	Upper gastrointestinal congenital anomalies	congenital anomalies	rs189447480	0.328	0.411	1.389	4.239E-01	logistic	420464	211	420253	0.029	0.010
204.1	Lymphoid leukemia	neoplasms	rs189447480	0.171	0.215	1.187	4.251E-01	logistic	416514	901	415613	0.062	0.010
350.3	Lack of coordination	neurological	rs189447480	-0.399	0.502	0.671	4.265E-01	logistic	420499	289	420210	0.025	0.010
741.3	Difficulty in walking	musculoskeletal	rs189447480	0.231	0.291	1.260	4.266E-01	logistic	415474	464	415010	0.042	0.010
454.11	Varicose veins of lower extremity, symptomtic	circulatory system	rs189447480	0.206	0.260	1.229	4.281E-01	logistic	381560	594	380966	0.159	0.010
618	Genital prolapse	genitourinary	rs189447480	-0.073	0.093	0.929	4.293E-01	logistic	219680	6225	213455	0.283	0.010
710.1	Osteomyelitis	musculoskeletal	rs189447480	-0.457	0.579	0.633	4.306E-01	logistic	412277	229	412048	0.006	0.010
175	Acquired absence of breast	neoplasms	rs189447480	0.084	0.107	1.088	4.328E-01	logistic	206502	3992	202510	0.389	0.010
560.2	Impaction of intestine	digestive	rs189447480	-0.453	0.579	0.635	4.339E-01	logistic	334714	229	334485	0.446	0.010
281.1	Megaloblastic anemia	hematopoietic	rs189447480	0.144	0.184	1.154	4.350E-01	logistic	383709	1260	382449	0.133	0.010
352	Disorders of other cranial nerves	neurological	rs189447480	0.145	0.187	1.156	4.389E-01	logistic	403637	1222	402415	0.012	0.010
579.8	Nonspecific abnormal findings in stool contents	digestive	rs189447480	-0.246	0.318	0.782	4.392E-01	logistic	383921	618	383303	0.241	0.010
592.1	Cystitis	genitourinary	rs189447480	-0.170	0.219	0.844	4.392E-01	logistic	378131	1204	376927	0.066	0.010
295.1	Schizophrenia	mental disorders	rs189447480	-0.234	0.303	0.791	4.393E-01	logistic	382292	670	381622	0.466	0.010
366	Cataract	sense organs	rs189447480	0.031	0.040	1.031	4.410E-01	logistic	406937	35259	371678	0.070	0.010
362.2	Degeneration of macula and posterior pole of retina	sense organs	rs189447480	-0.059	0.077	0.943	4.410E-01	logistic	406927	9170	397757	0.188	0.010
555.21	Ulcerative colitis (chronic)	digestive	rs189447480	0.245	0.319	1.278	4.416E-01	logistic	334866	381	334485	0.334	0.010
286.1	Congenital coagulation defects	hematopoietic	rs189447480	0.233	0.304	1.262	4.427E-01	logistic	417976	423	417553	0.057	0.010
200	Myeloproliferative disease	neoplasms	rs189447480	0.131	0.170	1.140	4.428E-01	logistic	417103	1490	415613	0.071	0.010
763	Thoracic or lumbosacral neuritis or radiculitis, unspecified	symptoms	rs189447480	-0.385	0.502	0.680	4.429E-01	logistic	418269	283	417986	0.078	0.010
512.7	Shortness of breath	respiratory	rs189447480	0.083	0.108	1.086	4.434E-01	logistic	408527	3928	404599	0.106	0.010
530.5	Disorders of esophageal motility	digestive	rs189447480	0.232	0.304	1.261	4.447E-01	logistic	357212	424	356788	0.095	0.010
473.3	Paralysis/spasm of vocal cords or larynx	respiratory	rs189447480	-0.442	0.579	0.643	4.456E-01	logistic	396742	225	396517	0.178	0.010
430.1	Subarachnoid hemorrhage	circulatory system	rs189447480	0.181	0.237	1.198	4.463E-01	logistic	403416	731	402685	0.002	0.010
250	Diabetes mellitus	endocrine/metabolic	rs189447480	0.033	0.044	1.034	4.466E-01	logistic	414239	27602	386637	0.148	0.010
550.5	Ventral hernia	digestive	rs189447480	0.124	0.163	1.132	4.495E-01	logistic	367031	1637	365394	0.481	0.010
740.2	Osteoarthrosis, generalized	musculoskeletal	rs189447480	-0.082	0.108	0.922	4.505E-01	logistic	350458	4600	345858	0.203	0.010
367	Disorders of refraction and accommodation; blindness and low vision	sense organs	rs189447480	-0.070	0.093	0.932	4.513E-01	logistic	417170	6164	411006	0.196	0.010
361	Retinal detachments and defects	sense organs	rs189447480	0.209	0.279	1.233	4.531E-01	logistic	398276	513	397763	0.094	0.010
835	Internal derangement of knee	injuries & poisonings	rs189447480	-0.080	0.107	0.923	4.550E-01	logistic	409924	4709	405215	0.092	0.010
592	Cystitis and urethritis	genitourinary	rs189447480	-0.160	0.214	0.852	4.561E-01	logistic	378176	1249	376927	0.065	0.010
564	Functional digestive disorders	digestive	rs189447480	0.037	0.050	1.038	4.570E-01	logistic	354387	19902	334485	0.227	0.010
274.1	Gout	endocrine/metabolic	rs189447480	0.078	0.105	1.081	4.582E-01	logistic	420024	4234	415790	0.015	0.010
480.11	Pneumococcal pneumonia	respiratory	rs189447480	0.052	0.071	1.054	4.591E-01	logistic	406463	9663	396800	0.297	0.010
149.1	Cancer of oropharynx	neoplasms	rs189447480	0.236	0.319	1.266	4.594E-01	logistic	420725	383	420342	0.001	0.010
362	Other retinal disorders	sense organs	rs189447480	-0.054	0.073	0.948	4.625E-01	logistic	407698	9941	397757	0.198	0.010
574.1	Cholelithiasis	digestive	rs189447480	-0.055	0.075	0.946	4.630E-01	logistic	404312	9265	395047	0.163	0.010
496.1	Emphysema	respiratory	rs189447480	-0.100	0.136	0.905	4.632E-01	logistic	401774	2965	398809	0.412	0.010
281	Other deficiency anemia	hematopoietic	rs189447480	0.132	0.181	1.141	4.648E-01	logistic	383766	1317	382449	0.132	0.010
698	Pruritus and related conditions	dermatologic	rs189447480	-0.367	0.502	0.693	4.649E-01	logistic	422717	278	422439	0.024	0.010
509	Respiratory failure, insufficiency, arrest	respiratory	rs189447480	-0.069	0.095	0.933	4.652E-01	logistic	397038	5981	391057	0.502	0.010
480.5	Bronchopneumonia and lung abscess	respiratory	rs189447480	0.222	0.304	1.248	4.658E-01	logistic	397229	429	396800	0.293	0.010
555.1	Regional enteritis	digestive	rs189447480	-0.128	0.175	0.880	4.668E-01	logistic	336302	1817	334485	0.085	0.010
800	Fracture of lower limb	injuries & poisonings	rs189447480	0.088	0.121	1.092	4.678E-01	logistic	399482	3104	396378	0.012	0.010
573.3	Hepatomegaly	digestive	rs189447480	-0.364	0.502	0.695	4.679E-01	logistic	400551	277	400274	0.136	0.010
540.11	Acute appendicitis	digestive	rs189447480	-0.417	0.579	0.659	4.715E-01	logistic	421875	219	421656	0.010	0.010
217	Vascular hamartomas and non-neoplastic nevi	neoplasms	rs189447480	0.255	0.356	1.290	4.741E-01	logistic	414259	301	413958	0.088	0.010
960	Poisoning by antibiotics	injuries & poisonings	rs189447480	0.034	0.048	1.035	4.759E-01	logistic	389532	22359	367173	0.219	0.010
689	Disorder of skin and subcutaneous tissue NOS	dermatologic	rs189447480	0.104	0.146	1.109	4.764E-01	logistic	413188	2098	411090	0.088	0.010
578.1	Hematemesis	digestive	rs189447480	-0.146	0.205	0.864	4.770E-01	logistic	384647	1344	383303	0.001	0.010
634.1	Missed abortion/Hydatidiform mole	pregnancy complications	rs189447480	-0.413	0.581	0.662	4.771E-01	logistic	222807	215	222592	0.293	0.010
429	Ill-defined descriptions and complications of heart disease	circulatory system	rs189447480	-0.319	0.449	0.727	4.777E-01	logistic	410037	334	409703	0.181	0.010
513	Respiratory abnormalities	respiratory	rs189447480	0.215	0.304	1.240	4.789E-01	logistic	423122	431	422691	0.027	0.010
591	Urinary tract infection	genitourinary	rs189447480	-0.048	0.067	0.954	4.789E-01	logistic	388723	11796	376927	0.068	0.010
276.41	Acidosis	endocrine/metabolic	rs189447480	-0.124	0.175	0.883	4.794E-01	logistic	397376	1804	395572	0.301	0.010
429.2	Abnormal function study of cardiovascular system	circulatory system	rs189447480	-0.316	0.449	0.729	4.820E-01	logistic	410036	333	409703	0.181	0.010
458.1	Orthostatic hypotension	circulatory system	rs189447480	0.085	0.120	1.088	4.821E-01	logistic	375723	3151	372572	0.633	0.010
297.2	Suicide or self-inflicted injury	mental disorders	rs189447480	-0.316	0.449	0.729	4.824E-01	logistic	381948	326	381622	0.463	0.010
594.8	Renal colic	genitourinary	rs189447480	-0.235	0.335	0.790	4.824E-01	logistic	412927	551	412376	0.100	0.010
285.22	Anemia in neoplastic disease	hematopoietic	rs189447480	-0.315	0.449	0.730	4.826E-01	logistic	382780	331	382449	0.153	0.010
274	Gout and other crystal arthropathies	endocrine/metabolic	rs189447480	0.071	0.102	1.074	4.842E-01	logistic	420276	4486	415790	0.012	0.010
785	Abdominal pain	symptoms	rs189447480	-0.059	0.085	0.943	4.893E-01	logistic	401183	7225	393958	0.160	0.010
451	Phlebitis and thrombophlebitis	circulatory system	rs189447480	-0.200	0.290	0.819	4.906E-01	logistic	381675	709	380966	0.039	0.010
244	Hypothyroidism	endocrine/metabolic	rs189447480	0.036	0.052	1.036	4.908E-01	logistic	411296	19066	392230	0.441	0.010
255	Disorders of adrenal glands	endocrine/metabolic	rs189447480	0.159	0.231	1.172	4.915E-01	logistic	416034	788	415246	0.039	0.010
289.4	Lymphadenitis	hematopoietic	rs189447480	-0.124	0.181	0.883	4.926E-01	logistic	410013	1704	408309	0.182	0.010
200.1	Polycythemia vera	neoplasms	rs189447480	0.191	0.279	1.211	4.935E-01	logistic	408705	523	408182	0.190	0.010
472	Chronic pharyngitis and nasopharyngitis	respiratory	rs189447480	0.260	0.380	1.297	4.937E-01	logistic	396779	262	396517	0.174	0.010

ST31; PheWAS results for the lead cis-pQTLs of the 4 proteins prioritized (ACOX1, FGF5, FURIN, and MST1).

SNP = Single Nucleotide Polymorphism; SE = Standard error HWE_p = P-value for Hardy-Weinberg equilibrium test

Phenotype	Description	Group	SNP	Beta	SE	Odds tatio	P-value	Type	n_total	n_cases	n_controls	HWE_p	allele_freq
751.21	Cystic kidney disease	congenital anomalies	rs189447480	-0.279	0.410	0.757	4.964E-01	logistic	420636	383	420253	0.032	0.010
761	Cervicalgia	symptoms	rs189447480	0.161	0.237	1.174	4.983E-01	logistic	422454	744	421710	0.132	0.010
789	Nausea and vomiting	symptoms	rs189447480	0.056	0.083	1.058	4.985E-01	logistic	407825	6923	400902	0.070	0.010
801.1	Fracture of foot	injuries & poisonings	rs189447480	0.278	0.411	1.320	4.991E-01	logistic	396597	219	396378	0.055	0.010
427	Cardiac dysrhythmias	circulatory system	rs189447480	0.030	0.045	1.030	5.006E-01	logistic	398369	26521	371848	0.030	0.010
558	Noninfectious gastroenteritis	digestive	rs189447480	-0.074	0.110	0.929	5.025E-01	logistic	338928	4443	334485	0.374	0.010
292	Neurological disorders	mental disorders	rs189447480	0.062	0.093	1.064	5.042E-01	logistic	410372	5399	404973	0.042	0.010
560.1	Paralytic ileus	digestive	rs189447480	0.213	0.319	1.237	5.044E-01	logistic	334880	395	334485	0.452	0.010
225	Benign neoplasm of brain and other parts of nervous system	neoplasms	rs189447480	0.149	0.225	1.161	5.080E-01	logistic	421233	836	420397	0.009	0.010
385.3	Cholesteatoma	sense organs	rs189447480	0.272	0.411	1.313	5.081E-01	logistic	418937	221	418716	0.022	0.010
626.12	Excessive or frequent menstruation	genitourinary	rs189447480	0.095	0.144	1.100	5.082E-01	logistic	194922	2175	192747	0.920	0.010
394.1	Mitral valve stenosis and aortic valve stenosis	circulatory system	rs189447480	0.130	0.198	1.139	5.100E-01	logistic	402530	1116	401414	0.026	0.010
182	Malignant neoplasm of uterus	neoplasms	rs189447480	0.123	0.188	1.131	5.107E-01	logistic	197217	1245	195972	0.200	0.010
433.1	Occlusion and stenosis of precerebral arteries	circulatory system	rs189447480	0.110	0.168	1.117	5.111E-01	logistic	404257	1572	402685	0.009	0.010
378.1	Strabismus (not specified as paralytic)	sense organs	rs189447480	-0.328	0.502	0.720	5.131E-01	logistic	412196	268	411928	0.127	0.010
151	Cancer of stomach	neoplasms	rs189447480	-0.159	0.244	0.853	5.131E-01	logistic	394829	967	393862	0.110	0.010
286	Coagulation defects	hematopoietic	rs189447480	0.140	0.215	1.150	5.147E-01	logistic	418481	928	417553	0.050	0.010
196	Radiotherapy	neoplasms	rs189447480	-0.121	0.187	0.886	5.165E-01	logistic	387589	1583	386006	0.513	0.010
622	Polyp of female genital organs	genitourinary	rs189447480	0.087	0.134	1.091	5.168E-01	logistic	216102	2530	213572	0.624	0.010
622.1	Polyp of corpus uteri	genitourinary	rs189447480	0.108	0.166	1.114	5.175E-01	logistic	215181	1609	213572	0.612	0.010
241.1	Nontoxic uninodular goiter	endocrine/metabolic	rs189447480	0.266	0.411	1.305	5.180E-01	logistic	392455	225	392230	0.438	0.010
752.1	Neural tube defects	congenital anomalies	rs189447480	0.204	0.319	1.226	5.220E-01	logistic	422933	394	422539	0.028	0.010
941	Adverse reaction to serum or vaccine	injuries & poisonings	rs189447480	0.262	0.411	1.300	5.232E-01	logistic	415212	225	414987	0.044	0.010
476	Allergic rhinitis	respiratory	rs189447480	0.129	0.201	1.137	5.233E-01	logistic	397580	1063	396517	0.161	0.010
783	Fever of unknown origin	symptoms	rs189447480	-0.095	0.150	0.909	5.252E-01	logistic	418176	2397	415779	0.074	0.010
333.1	Essential tremor	neurological	rs189447480	0.177	0.279	1.194	5.254E-01	logistic	399579	532	399047	0.073	0.010
296.1	Bipolar	mental disorders	rs189447480	-0.139	0.220	0.870	5.267E-01	logistic	382786	1164	381622	0.471	0.010
150	Cancer of esophagus	neoplasms	rs189447480	-0.125	0.197	0.883	5.268E-01	logistic	395291	1429	393862	0.105	0.010
286.7	Other and unspecified coagulation defects	hematopoietic	rs189447480	-0.317	0.502	0.729	5.280E-01	logistic	417818	265	417553	0.064	0.010
697	Sarcoidosis	dermatologic	rs189447480	-0.200	0.318	0.818	5.282E-01	logistic	405795	589	405206	0.041	0.010
480	Pneumonia	respiratory	rs189447480	0.036	0.056	1.036	5.294E-01	logistic	412462	15662	396800	0.138	0.010
530	Diseases of esophagus	digestive	rs189447480	-0.026	0.042	0.974	5.297E-01	logistic	388733	31945	356788	0.055	0.010
172.1	Melanomas of skin, dx or hx	neoplasms	rs189447480	-0.108	0.173	0.898	5.315E-01	logistic	409038	1830	407208	0.100	0.010
172.11	Melanomas of skin	neoplasms	rs189447480	-0.108	0.173	0.898	5.315E-01	logistic	409038	1830	407208	0.100	0.010
378.2	Nystagmus and other irregular eye movements	sense organs	rs189447480	-0.362	0.579	0.696	5.317E-01	logistic	412137	209	411928	0.126	0.010
727.6	Rupture of tendon, nontraumatic	musculoskeletal	rs189447480	0.113	0.181	1.119	5.339E-01	logistic	390517	1343	389174	0.339	0.010
522.5	Periapical abscess	digestive	rs189447480	-0.359	0.580	0.698	5.354E-01	logistic	409744	208	409536	0.409	0.010
331.9	Cerebral degeneration, unspecified	neurological	rs189447480	-0.150	0.244	0.860	5.373E-01	logistic	400009	962	399047	0.073	0.010
426.23	Second degree AV block	circulatory system	rs189447480	-0.187	0.303	0.830	5.375E-01	logistic	372488	640	371848	0.225	0.010
701	Other hypertrophic and atrophic conditions of skin	dermatologic	rs189447480	-0.135	0.219	0.873	5.376E-01	logistic	416347	1161	415186	0.038	0.010
577	Diseases of pancreas	digestive	rs189447480	-0.079	0.128	0.924	5.377E-01	logistic	421247	3239	418008	0.016	0.010
798.1	Chronic fatigue syndrome	symptoms	rs189447480	0.160	0.260	1.174	5.380E-01	logistic	414937	617	414320	0.163	0.010
550	Abdominal hernia	digestive	rs189447480	0.027	0.044	1.027	5.398E-01	logistic	392811	27417	365394	0.377	0.010
613	Other nonmalignant breast conditions	genitourinary	rs189447480	0.195	0.319	1.215	5.406E-01	logistic	421594	397	421197	0.002	0.010
242	Thyrotoxicosis with or without goiter	endocrine/metabolic	rs189447480	0.103	0.168	1.108	5.417E-01	logistic	393812	1582	392230	0.346	0.010
585.2	Renal failure NOS	genitourinary	rs189447480	-0.170	0.279	0.844	5.419E-01	logistic	381806	744	381062	0.265	0.010
443.1	Raynaud's syndrome	circulatory system	rs189447480	0.102	0.168	1.108	5.419E-01	logistic	407046	1577	405469	0.163	0.010
395.4	Nonrheumatic pulmonary valve disorders	circulatory system	rs189447480	-0.273	0.449	0.761	5.423E-01	logistic	401735	321	401414	0.081	0.010
174.11	Malignant neoplasm of female breast	neoplasms	rs189447480	0.045	0.073	1.046	5.428E-01	logistic	212959	9200	203759	0.338	0.010
170	Cancer of bone and connective tissue	neoplasms	rs189447480	0.204	0.335	1.226	5.437E-01	logistic	423460	359	423101	0.027	0.010
184.1	Malignant neoplasm of ovary and other uterine adnexa	neoplasms	rs189447480	0.078	0.129	1.081	5.470E-01	logistic	207626	2789	204837	0.366	0.010
442.8	Aneurysm of other specified artery	circulatory system	rs189447480	-0.302	0.502	0.739	5.472E-01	logistic	405731	262	405469	0.140	0.010
427.12	Paroxysmal ventricular tachycardia	circulatory system	rs189447480	0.126	0.210	1.135	5.478E-01	logistic	372834	986	371848	0.235	0.010
276.6	Fluid overload	endocrine/metabolic	rs189447480	0.098	0.164	1.103	5.491E-01	logistic	397239	1667	395572	0.192	0.010
358	Myoneural disorders	neurological	rs189447480	-0.269	0.449	0.765	5.497E-01	logistic	419485	317	419168	0.087	0.010
53	Herpes zoster	infectious diseases	rs189447480	0.198	0.335	1.219	5.542E-01	logistic	415563	359	415204	0.062	0.010
709.2	Sicca syndrome	dermatologic	rs189447480	-0.164	0.279	0.849	5.559E-01	logistic	404638	739	403899	0.061	0.010
450	Noninfectious disorders of lymphatic channels	circulatory system	rs189447480	0.114	0.194	1.121	5.571E-01	logistic	422720	1167	421553	0.025	0.010
575.2	Obstruction of bile duct	digestive	rs189447480	0.121	0.206	1.128	5.571E-01	logistic	396076	1029	395047	0.311	0.010
228	Hemangioma and lymphangioma, any site	neoplasms	rs189447480	-0.197	0.335	0.822	5.572E-01	logistic	421655	529	421126	0.010	0.010
714.1	Rheumatoid arthritis	musculoskeletal	rs189447480	0.064	0.110	1.066	5.589E-01	logistic	412357	3850	408507	0.026	0.010
599	Other symptoms/disorders or the urinary system	genitourinary	rs189447480	-0.047	0.080	0.955	5.608E-01	logistic	398281	8175	390106	0.023	0.010
433.3	Cerebral ischemia	circulatory system	rs189447480	0.060	0.105	1.062	5.651E-01	logistic	407003	4318	402685	0.075	0.010
578.8	Hemorrhage of rectum and anus	digestive	rs189447480	0.080	0.140	1.084	5.663E-01	logistic	385630	2327	383303	0.270	0.010
727.1	Synovitis and tenosynovitis	musculoskeletal	rs189447480	-0.257	0.449	0.773	5.669E-01	logistic	389486	312	389174	0.321	0.010
290	Delirium dementia and amnestic and other cognitive disorders	mental disorders	rs189447480	0.047	0.082	1.048	5.709E-01	logistic	412158	7185	404973	0.336	0.010
804	Fracture of hand or wrist	injuries & poisonings	rs189447480	-0.180	0.318	0.835	5.717E-01	logistic	396955	577	396378	0.055	0.010
594.3	Calculus of ureter	genitourinary	rs189447480	0.094	0.166	1.098	5.721E-01	logistic	414010	1634	412376	0.079	0.010
741.4	Joint effusions	musculoskeletal	rs189447480	-0.163	0.290	0.849	5.731E-01	logistic	415693	683	415010	0.039	0.010
500.2	Pneumoconiosis	respiratory	rs189447480	-0.231	0.411	0.794	5.742E-01	logistic	391432	375	391057	0.452	0.010
297	Suicidal ideation or attempt	mental disorders	rs189447480	-0.231	0.410	0.794	5.744E-01	logistic	381982	360	381622	0.464	0.010

ST31; PheWAS results for the lead cis-pQTLs of the 4 proteins prioritized (ACOX1, FGF5, FURIN, and MST1).

SNP = Single Nucleotide Polymorphism; SE = Standard error HWE_p = P-value for Hardy-Weinberg equilibrium test

Phenotype	Description	Group	SNP	Beta	SE	Odds tatio	P-value	Type	n_total	n_cases	n_controls	HWE_p	allele_freq
597.1	Urethral stricture (not specified as infectious)	genitourinary	rs189447480	0.088	0.158	1.092	5.767E-01	logistic	403370	1816	401554	0.027	0.010
750.2	Lower gastrointestinal congenital anomalies	congenital anomalies	rs189447480	-0.320	0.579	0.726	5.809E-01	logistic	420453	200	420253	0.033	0.010
735.23	Hallux rigidus	musculoskeletal	rs189447480	0.196	0.356	1.217	5.817E-01	logistic	402973	319	402654	0.242	0.010
501	Pneumonitis due to inhalation of food or vomitus	respiratory	rs189447480	0.088	0.160	1.092	5.817E-01	logistic	392843	1786	391057	0.471	0.010
556.1	Ulceration of intestine	digestive	rs189447480	-0.318	0.580	0.727	5.830E-01	logistic	334685	200	334485	0.446	0.010
184.11	Malignant neoplasm of ovary	neoplasms	rs189447480	0.071	0.131	1.074	5.852E-01	logistic	207552	2715	204837	0.364	0.010
292.1	Aphasia/speech disturbance	mental disorders	rs189447480	-0.126	0.231	0.882	5.861E-01	logistic	406020	1047	404973	0.253	0.010
185	Cancer of prostate	neoplasms	rs189447480	0.038	0.069	1.038	5.870E-01	logistic	167540	11079	156461	0.218	0.010
496.2	Chronic bronchitis	respiratory	rs189447480	0.051	0.093	1.052	5.872E-01	logistic	404328	5519	398809	0.344	0.010
531.1	Hemorrhage from gastrointestinal ulcer	digestive	rs189447480	-0.172	0.318	0.842	5.893E-01	logistic	411913	574	411339	0.136	0.010
594.1	Calculus of kidney	genitourinary	rs189447480	0.065	0.121	1.067	5.895E-01	logistic	415555	3179	412376	0.057	0.010
411	Ischemic Heart Disease	circulatory system	rs189447480	-0.021	0.038	0.980	5.904E-01	logistic	410606	40056	370550	0.121	0.010
277	Other disorders of metabolism	endocrine/metabolic	rs189447480	0.108	0.201	1.114	5.926E-01	logistic	421029	1088	419941	0.130	0.010
619.2	Disorders of uterus, NEC	genitourinary	rs189447480	-0.203	0.380	0.816	5.928E-01	logistic	217379	413	216966	0.370	0.010
540	Appendiceal conditions	digestive	rs189447480	-0.190	0.355	0.827	5.932E-01	logistic	422123	467	421656	0.013	0.010
550.4	Umbilical hernia	digestive	rs189447480	0.095	0.178	1.100	5.934E-01	logistic	366811	1417	365394	0.376	0.010
331	Other cerebral degenerations	neurological	rs189447480	0.088	0.166	1.092	5.952E-01	logistic	400699	1652	399047	0.089	0.010
579	Other symptoms involving abdomen and pelvis	digestive	rs189447480	-0.122	0.231	0.885	5.961E-01	logistic	384340	1037	383303	0.246	0.010
189	Cancer of urinary organs (incl. kidney and bladder)	neoplasms	rs189447480	0.054	0.102	1.055	5.967E-01	logistic	421760	4573	417187	0.121	0.010
276.14	Hypopotassemia	endocrine/metabolic	rs189447480	0.061	0.115	1.063	5.980E-01	logistic	399074	3502	395572	0.440	0.010
618.2	Uterine/Uterovaginal prolapse	genitourinary	rs189447480	-0.133	0.252	0.876	5.983E-01	logistic	214329	874	213455	0.204	0.010
595	Hydronephrosis	genitourinary	rs189447480	-0.124	0.237	0.883	5.998E-01	logistic	413362	986	412376	0.094	0.010
803.1	Fracture of humerus	injuries & poisonings	rs189447480	0.106	0.201	1.111	6.004E-01	logistic	397468	1090	396378	0.043	0.010
990	Effects radiation NOS	injuries & poisonings	rs189447480	0.044	0.083	1.045	6.005E-01	logistic	408877	6919	401958	0.084	0.010
585.32	End stage renal disease	genitourinary	rs189447480	-0.127	0.244	0.880	6.013E-01	logistic	381994	932	381062	0.267	0.010
802	Fracture of pelvis	injuries & poisonings	rs189447480	0.112	0.215	1.118	6.020E-01	logistic	397331	953	396378	0.045	0.010
747.12	Valvular heart disease/ heart chambers	congenital anomalies	rs189447480	0.198	0.380	1.218	6.035E-01	logistic	421875	280	421595	0.020	0.010
366.2	Senile cataract	sense organs	rs189447480	0.038	0.074	1.039	6.041E-01	logistic	380814	9136	371678	0.206	0.010
560.3	Peritoneal or intestinal adhesions	digestive	rs189447480	-0.135	0.260	0.874	6.043E-01	logistic	335317	832	334485	0.452	0.010
368	Visual disturbances	sense organs	rs189447480	-0.077	0.152	0.926	6.119E-01	logistic	417843	2298	415545	0.075	0.010
193	Thyroid cancer	neoplasms	rs189447480	0.161	0.319	1.174	6.143E-01	logistic	421300	413	420887	0.348	0.010
557.1	Celiac disease	digestive	rs189447480	-0.084	0.168	0.919	6.163E-01	logistic	336383	1898	334485	0.463	0.010
512	Other symptoms of respiratory system	respiratory	rs189447480	0.043	0.086	1.044	6.174E-01	logistic	411090	6491	404599	0.137	0.010
627.1	Postmenopausal bleeding	genitourinary	rs189447480	0.079	0.160	1.083	6.203E-01	logistic	194538	1791	192747	0.918	0.010
411.4	Coronary atherosclerosis	circulatory system	rs189447480	-0.024	0.049	0.976	6.208E-01	logistic	394337	23787	370550	0.162	0.010
803.2	Fracture of radius and ulna	injuries & poisonings	rs189447480	-0.076	0.154	0.927	6.208E-01	logistic	398618	2240	396378	0.035	0.010
717	Polymyalgia Rheumatica	musculoskeletal	rs189447480	-0.073	0.147	0.930	6.216E-01	logistic	422397	2442	419955	0.012	0.010
201	Hodgkin's disease	neoplasms	rs189447480	0.187	0.381	1.206	6.226E-01	logistic	415895	282	415613	0.052	0.010
381.1	Otitis media	sense organs	rs189447480	0.127	0.260	1.136	6.239E-01	logistic	419355	639	418716	0.016	0.010
250.42	Other abnormal glucose	endocrine/metabolic	rs189447480	-0.220	0.449	0.803	6.245E-01	logistic	386940	303	386637	0.143	0.010
79.9	Viremia, NOS	infectious diseases	rs189447480	0.131	0.269	1.140	6.264E-01	logistic	415798	594	415204	0.065	0.010
8.52	Intestinal infection due to C. difficile	infectious diseases	rs189447480	-0.141	0.290	0.868	6.269E-01	logistic	417984	668	417316	0.045	0.010
218.1	Uterine leiomyoma	neoplasms	rs189447480	0.062	0.130	1.064	6.312E-01	logistic	209756	2761	206995	0.256	0.010
348	Other conditions of brain	neurological	rs189447480	-0.139	0.290	0.870	6.314E-01	logistic	399717	670	399047	0.070	0.010
280.2	Iron deficiency anemia secondary to blood loss (chronic	hematopoietic	rs189447480	-0.215	0.449	0.806	6.317E-01	logistic	382749	300	382449	0.152	0.010
530.2	Esophageal bleeding (varices/hemorrhage	digestive	rs189447480	0.152	0.318	1.164	6.327E-01	logistic	357206	418	356788	0.096	0.010
603	Other disorders of testis	genitourinary	rs189447480	0.145	0.304	1.156	6.331E-01	logistic	162246	468	161778	0.206	0.010
560.4	Other intestinal obstruction	digestive	rs189447480	0.070	0.147	1.073	6.346E-01	logistic	336617	2132	334485	0.231	0.010
342	Hemiplegia	neurological	rs189447480	-0.071	0.149	0.932	6.347E-01	logistic	401449	2402	399047	0.091	0.010
251.1	Hypoglycemia	endocrine/metabolic	rs189447480	0.082	0.173	1.085	6.352E-01	logistic	383028	1531	381497	0.096	0.010
561	Symptoms involving digestive system	digestive	rs189447480	0.046	0.097	1.047	6.357E-01	logistic	339539	5054	334485	0.394	0.010
596.1	Bladder neck obstruction	genitourinary	rs189447480	-0.150	0.318	0.860	6.363E-01	logistic	402115	561	401554	0.127	0.010
596.5	Functional disorders of bladder	genitourinary	rs189447480	0.123	0.260	1.130	6.374E-01	logistic	402194	640	401554	0.132	0.010
252.1	Hyperparathyroidism	endocrine/metabolic	rs189447480	-0.109	0.231	0.897	6.379E-01	logistic	416271	1025	415246	0.036	0.010
344	Other paralytic syndromes	neurological	rs189447480	-0.136	0.290	0.873	6.389E-01	logistic	399714	667	399047	0.070	0.010
41.11	Methicillin sensitive Staphylococcus aureus	infectious diseases	rs189447480	-0.085	0.181	0.919	6.390E-01	logistic	398282	1635	396647	0.191	0.010
172	Skin cancer	neoplasms	rs189447480	-0.043	0.093	0.958	6.398E-01	logistic	413263	6055	407208	0.032	0.010
202.21	Nodular lymphoma	neoplasms	rs189447480	0.141	0.303	1.151	6.422E-01	logistic	416077	464	415613	0.054	0.010
740	Osteoarthrosis	musculoskeletal	rs189447480	-0.016	0.035	0.984	6.426E-01	logistic	392374	46516	345858	0.119	0.010
578.2	Blood in stool	digestive	rs189447480	0.074	0.159	1.077	6.429E-01	logistic	385103	1800	383303	0.262	0.010
610	Benign mammary dysplasias	genitourinary	rs189447480	-0.155	0.335	0.856	6.440E-01	logistic	220777	505	220272	0.282	0.010
728.7	Fasciitis	musculoskeletal	rs189447480	-0.079	0.170	0.924	6.445E-01	logistic	391018	1844	389174	0.221	0.010
851	Complications of transplants and reattached limbs	injuries & poisonings	rs189447480	-0.231	0.502	0.793	6.449E-01	logistic	416895	243	416652	0.033	0.010
481	Influenza	respiratory	rs189447480	0.139	0.304	1.150	6.460E-01	logistic	397264	464	396800	0.404	0.010
350	Abnormal movement	neurological	rs189447480	0.089	0.194	1.093	6.469E-01	logistic	421410	1200	420210	0.008	0.010
750	Digestive congenital anomalies	congenital anomalies	rs189447480	0.145	0.318	1.156	6.485E-01	logistic	420674	421	420253	0.027	0.010
208	Benign neoplasm of colon	neoplasms	rs189447480	-0.023	0.052	0.977	6.497E-01	logistic	395855	20019	375836	0.134	0.010
8.6	Viral Enteritis	infectious diseases	rs189447480	-0.152	0.335	0.859	6.506E-01	logistic	417821	505	417316	0.047	0.010
574.3	Cholecystitis without cholelithiasis	digestive	rs189447480	0.087	0.194	1.091	6.529E-01	logistic	396244	1197	395047	0.196	0.010
420.3	Endocarditis	circulatory system	rs189447480	-0.136	0.303	0.873	6.533E-01	logistic	417383	613	416770	0.153	0.010
427.2	Atrial fibrillation and flutter	circulatory system	rs189447480	0.025	0.056	1.025	6.540E-01	logistic	388316	16468	371848	0.074	0.010

ST31; PheWAS results for the lead cis-pQTLs of the 4 proteins prioritized (ACOX1, FGF5, FURIN, and MST1).
SNP = Single Nucleotide Polymorphism; SE = Standard error HWE_p = P-value for Hardy-Weinberg equilibrium test

Phenotype	Description	Group	SNP	Beta	SE	Odds tatio	P-value	Type	n_total	n_cases	n_controls	HWE_p	allele_freq
500	Lung disease due to external agents	respiratory	rs189447480	-0.136	0.303	0.873	6.543E-01	logistic	391675	618	391057	0.454	0.010
153	Colorectal cancer	neoplasms	rs189447480	0.037	0.083	1.038	6.544E-01	logistic	377768	7048	370720	0.045	0.010
522	Diseases of pulp and periapical tissues	digestive	rs189447480	-0.201	0.449	0.818	6.544E-01	logistic	409832	296	409536	0.410	0.010
580.3	Nephritis and nephropathy without mention of glomerulonephritis	genitourinary	rs189447480	-0.200	0.449	0.819	6.563E-01	logistic	381356	294	381062	0.261	0.010
337	Disorders of the autonomic nervous system	neurological	rs189447480	-0.182	0.410	0.833	6.569E-01	logistic	399396	349	399047	0.067	0.010
153.2	Colon cancer	neoplasms	rs189447480	0.045	0.102	1.046	6.578E-01	logistic	375260	4540	370720	0.010	0.010
155	Cancer of liver and intrahepatic bile duct	neoplasms	rs189447480	0.107	0.244	1.113	6.607E-01	logistic	394604	742	393862	0.229	0.010
362.29	Macular degeneration (senile) of retina NOS	sense organs	rs189447480	-0.051	0.115	0.951	6.609E-01	logistic	401665	3908	397757	0.130	0.010
278	Overweight, obesity and other hyperalimentation	endocrine/metabolic	rs189447480	0.025	0.057	1.025	6.640E-01	logistic	405726	15173	390553	0.136	0.010
260	Protein-calorie malnutrition	endocrine/metabolic	rs189447480	0.146	0.335	1.157	6.642E-01	logistic	412473	376	412097	0.133	0.010
191.1	Cancer of brain and nervous system	neoplasms	rs189447480	0.100	0.231	1.105	6.646E-01	logistic	421229	832	420397	0.008	0.010
276.4	Acid-base balance disorder	endocrine/metabolic	rs189447480	-0.068	0.157	0.934	6.653E-01	logistic	397692	2120	395572	0.307	0.010
535.8	Other specified gastritis	digestive	rs189447480	0.056	0.129	1.057	6.659E-01	logistic	365017	2803	362214	0.007	0.010
568.1	Peritoneal adhesions (postoperative) (postinfection)	digestive	rs189447480	0.080	0.187	1.084	6.676E-01	logistic	385136	1297	383839	0.014	0.010
184	Cancer of other female genital organs	neoplasms	rs189447480	0.054	0.126	1.055	6.682E-01	logistic	207829	2992	204837	0.368	0.010
715.2	Ankylosing spondylitis	musculoskeletal	rs189447480	-0.151	0.355	0.860	6.713E-01	logistic	408956	449	408507	0.026	0.010
335	Multiple sclerosis	neurological	rs189447480	-0.080	0.190	0.923	6.728E-01	logistic	400514	1467	399047	0.080	0.010
416	Cardiomegaly	circulatory system	rs189447480	0.044	0.104	1.045	6.743E-01	logistic	417255	4417	412838	0.294	0.010
255.1	Adrenal hyperfunction	endocrine/metabolic	rs189447480	0.189	0.450	1.208	6.745E-01	logistic	415447	201	415246	0.029	0.010
70	Viral hepatitis	infectious diseases	rs189447480	-0.133	0.318	0.875	6.753E-01	logistic	415755	551	415204	0.061	0.010
580.2	Nephrotic syndrome without mention of glomerulonephritis	genitourinary	rs189447480	-0.157	0.380	0.855	6.793E-01	logistic	381457	395	381062	0.262	0.010
560	Intestinal obstruction without mention of hernia	digestive	rs189447480	-0.049	0.120	0.952	6.806E-01	logistic	338110	3625	334485	0.242	0.010
285	Other anemias	hematopoietic	rs189447480	0.027	0.066	1.027	6.830E-01	logistic	393921	11472	382449	0.118	0.010
170.2	Cancer of connective tissue	neoplasms	rs189447480	0.167	0.411	1.182	6.837E-01	logistic	423349	248	423101	0.024	0.010
714	Rheumatoid arthritis and other inflammatory polyarthropathies	musculoskeletal	rs189447480	0.044	0.108	1.045	6.838E-01	logistic	412574	4067	408507	0.027	0.010
374	Other disorders of eyelids	sense organs	rs189447480	-0.061	0.152	0.940	6.859E-01	logistic	413055	2262	410793	0.135	0.010
317	Alcohol-related disorders	mental disorders	rs189447480	0.039	0.097	1.040	6.874E-01	logistic	408346	5053	403293	0.018	0.010
411.9	Other acute and subacute forms of ischemic heart disease	circulatory system	rs189447480	0.071	0.176	1.073	6.876E-01	logistic	372042	1492	370550	0.260	0.010
720	Spinal stenosis	musculoskeletal	rs189447480	0.043	0.107	1.044	6.892E-01	logistic	412805	4135	408670	0.147	0.010
345.1	Epilepsy	neurological	rs189447480	-0.126	0.318	0.881	6.910E-01	logistic	399597	550	399047	0.069	0.010
426	Cardiac conduction disorders	circulatory system	rs189447480	-0.028	0.070	0.973	6.940E-01	logistic	382540	10692	371848	0.022	0.010
278.1	Obesity	endocrine/metabolic	rs189447480	0.023	0.058	1.023	6.949E-01	logistic	405667	15114	390553	0.138	0.010
628	Ovarian cyst	genitourinary	rs189447480	-0.083	0.211	0.921	6.952E-01	logistic	193944	1197	192747	0.915	0.010
740.12	Osteoarthrosis, localized, secondary	musculoskeletal	rs189447480	0.124	0.318	1.132	6.967E-01	logistic	346286	428	345858	0.157	0.010
625	Pain and other symptoms associated with female genital organs	genitourinary	rs189447480	0.148	0.381	1.159	6.980E-01	logistic	218575	291	218284	0.381	0.010
253.2	Pituitary hypofunction	endocrine/metabolic	rs189447480	-0.174	0.449	0.840	6.988E-01	logistic	415534	288	415246	0.028	0.010
626	Disorders of menstruation and other abnormal bleeding from female genital tract	genitourinary	rs189447480	0.035	0.090	1.035	6.999E-01	logistic	198823	6076	192747	0.933	0.010
602	Other disorders of prostate	genitourinary	rs189447480	-0.158	0.410	0.854	7.005E-01	logistic	156802	341	156461	0.123	0.010
112	Candidiasis	infectious diseases	rs189447480	-0.070	0.184	0.932	7.028E-01	logistic	419935	1555	418380	0.113	0.010
145.2	Cancer of tongue	neoplasms	rs189447480	-0.145	0.380	0.865	7.035E-01	logistic	420733	391	420342	0.003	0.010
735.2	Acquired toe deformities	musculoskeletal	rs189447480	0.059	0.156	1.061	7.051E-01	logistic	404574	1920	402654	0.144	0.010
555	Inflammatory bowel disease and other gastroenteritis and colitis	digestive	rs189447480	-0.039	0.104	0.961	7.051E-01	logistic	339234	4749	334485	0.127	0.010
724	Other and unspecified disorders of back	musculoskeletal	rs189447480	-0.119	0.318	0.888	7.078E-01	logistic	409212	542	408670	0.215	0.010
442	Other aneurysm	circulatory system	rs189447480	-0.049	0.132	0.952	7.085E-01	logistic	408493	3024	405469	0.071	0.010
446.4	Wegener's granulomatosis	circulatory system	rs189447480	-0.187	0.502	0.829	7.090E-01	logistic	405703	234	405469	0.140	0.010
371	Inflammation of the eye	sense organs	rs189447480	0.078	0.210	1.081	7.091E-01	logistic	411823	1030	410793	0.125	0.010
333	Extrapyramidal disease and abnormal movement disorders	neurological	rs189447480	0.072	0.194	1.075	7.097E-01	logistic	400270	1223	399047	0.041	0.010
729	Other disorders of soft tissues	musculoskeletal	rs189447480	0.153	0.411	1.165	7.106E-01	logistic	389423	249	389174	0.323	0.010
199	Neoplasm of uncertain behavior	neoplasms	rs189447480	0.100	0.269	1.105	7.111E-01	logistic	386620	614	386006	0.601	0.010
752.11	Spina bifida	congenital anomalies	rs189447480	0.166	0.450	1.181	7.119E-01	logistic	422743	204	422539	0.144	0.010
473.4	Voice disturbance	respiratory	rs189447480	-0.165	0.449	0.848	7.133E-01	logistic	396801	284	396517	0.176	0.010
430	Intracranial hemorrhage	circulatory system	rs189447480	-0.061	0.166	0.941	7.146E-01	logistic	404595	1910	402685	0.006	0.010
514	Abnormal findings examination of lungs	respiratory	rs189447480	-0.057	0.155	0.945	7.150E-01	logistic	419804	2147	417657	0.013	0.010
227	Benign neoplasm of other endocrine glands and related structures	neoplasms	rs189447480	0.091	0.252	1.095	7.180E-01	logistic	421596	709	420887	0.240	0.010
722	Intervertebral disc disorders	musculoskeletal	rs189447480	0.091	0.260	1.096	7.251E-01	logistic	409333	663	408670	0.220	0.010
198.1	Secondary malignancy of lymph nodes	neoplasms	rs189447480	-0.028	0.080	0.972	7.263E-01	logistic	394015	8009	386006	0.477	0.010
244.4	Hypothyroidism NOS	endocrine/metabolic	rs189447480	0.019	0.053	1.019	7.266E-01	logistic	410296	18066	392230	0.423	0.010
508	Pulmonary collapse; interstitial and compensatory emphysema	respiratory	rs189447480	-0.040	0.116	0.960	7.272E-01	logistic	394890	3833	391057	0.267	0.010
41	Bacterial infection NOS	infectious diseases	rs189447480	0.025	0.072	1.025	7.290E-01	logistic	406056	9409	396647	0.177	0.010
604	Disorders of penis	genitourinary	rs189447480	0.105	0.304	1.111	7.296E-01	logistic	162266	488	161778	0.205	0.010
737	Curvature of spine	musculoskeletal	rs189447480	-0.084	0.244	0.919	7.297E-01	logistic	403550	896	402654	0.245	0.010
289.5	Diseases of spleen	hematopoietic	rs189447480	0.084	0.244	1.088	7.307E-01	logistic	409070	761	408309	0.177	0.010
742	Derangement of joint, non-traumatic	musculoskeletal	rs189447480	0.108	0.318	1.115	7.335E-01	logistic	415444	434	415010	0.081	0.010
401.2	Hypertensive heart and/or renal disease	circulatory system	rs189447480	0.068	0.202	1.071	7.347E-01	logistic	291807	1144	290663	0.409	0.010
573.7	Abnormal results of function study of liver	digestive	rs189447480	0.038	0.112	1.038	7.357E-01	logistic	404090	3816	400274	0.066	0.010
174.1	Breast cancer [female]	neoplasms	rs189447480	0.024	0.070	1.024	7.363E-01	logistic	213896	10137	203759	0.348	0.010
153.3	Malignant neoplasm of rectum, rectosigmoid junction, and anus	neoplasms	rs189447480	-0.045	0.134	0.956	7.370E-01	logistic	373609	2889	370720	0.106	0.010
528.11	Stomatitis and mucositis (ulcerative)	digestive	rs189447480	0.151	0.450	1.162	7.379E-01	logistic	417919	209	417710	0.058	0.010
575.9	Nonspecific abnormal findings on radiological and other examination of biliary tract	digestive	rs189447480	-0.167	0.502	0.846	7.394E-01	logistic	395275	228	395047	0.298	0.010
381.11	Suppurative and unspecified otitis media	sense organs	rs189447480	0.136	0.411	1.146	7.407E-01	logistic	418969	253	418716	0.023	0.010
351	Other peripheral nerve disorders	neurological	rs189447480	0.028	0.085	1.028	7.423E-01	logistic	409126	6711	402415	0.063	0.010

ST31; PheWAS results for the lead cis-pQTLs of the 4 proteins prioritized (ACOX1, FGF5, FURIN, and MST1).

SNP = Single Nucleotide Polymorphism; SE = Standard error HWE_p = P-value for Hardy-Weinberg equilibrium test

Phenotype	Description	Group	SNP	Beta	SE	Odds tatio	P-value	Type	n_total	n_cases	n_controls	HWE_p	allele_freq
600	Hyperplasia of prostate	genitourinary	rs189447480	0.022	0.066	1.022	7.423E-01	logistic	169018	12557	156461	0.365	0.010
451.2	Phlebitis and thrombophlebitis of lower extremities	circulatory system	rs189447480	-0.110	0.335	0.896	7.426E-01	logistic	381453	487	380966	0.040	0.010
536	Disorders of function of stomach	digestive	rs189447480	-0.058	0.178	0.943	7.427E-01	logistic	363858	1644	362214	0.226	0.010
585.3	Chronic renal failure [CKD]	genitourinary	rs189447480	-0.021	0.064	0.979	7.463E-01	logistic	393739	12677	381062	0.290	0.010
529	Diseases and other conditions of the tongue	digestive	rs189447480	-0.145	0.449	0.865	7.465E-01	logistic	417990	280	417710	0.057	0.010
250.23	Type 2 diabetes with ophthalmic manifestations	endocrine/metabolic	rs189447480	-0.053	0.163	0.949	7.469E-01	logistic	388590	1953	386637	0.163	0.010
947	Urticaria	dermatologic	rs189447480	0.145	0.450	1.156	7.469E-01	logistic	415196	209	414987	0.043	0.010
324	Other CNS infection and poliomyelitis	neurological	rs189447480	0.114	0.356	1.121	7.486E-01	logistic	422418	347	422071	0.003	0.010
218	Benign neoplasm of uterus	neoplasms	rs189447480	0.041	0.129	1.042	7.493E-01	logistic	209861	2866	206995	0.256	0.010
285.2	Anemia of chronic disease	hematopoietic	rs189447480	-0.082	0.259	0.921	7.516E-01	logistic	383237	788	382449	0.146	0.010
614.5	Inflammatory disease of cervix, vagina, and vulva	genitourinary	rs189447480	-0.101	0.319	0.904	7.519E-01	logistic	217969	531	217438	0.638	0.010
568	Other disorders of peritoneum	digestive	rs189447480	0.056	0.178	1.058	7.519E-01	logistic	385305	1466	383839	0.015	0.010
618.1	Prolapse of vaginal walls	genitourinary	rs189447480	-0.036	0.114	0.965	7.533E-01	logistic	217426	3971	213455	0.255	0.010
569.2	Gastrointestinal complications	digestive	rs189447480	-0.141	0.449	0.869	7.544E-01	logistic	384117	278	383839	0.004	0.010
333.4	Torsion dystonia	neurological	rs189447480	0.119	0.380	1.126	7.550E-01	logistic	399349	302	399047	0.055	0.010
117.4	Aspergillosis	infectious diseases	rs189447480	0.139	0.450	1.149	7.574E-01	logistic	418590	210	418380	0.100	0.010
270.33	Amyloidosis	endocrine/metabolic	rs189447480	-0.126	0.410	0.882	7.588E-01	logistic	422032	330	421702	0.110	0.010
253.7	Other disorders of neurohypophysis	endocrine/metabolic	rs189447480	0.109	0.355	1.115	7.592E-01	logistic	415594	348	415246	0.031	0.010
575.1	Cholangitis	digestive	rs189447480	-0.075	0.244	0.928	7.598E-01	logistic	395932	885	395047	0.305	0.010
523	Gingival and periodontal diseases	digestive	rs189447480	0.102	0.336	1.108	7.603E-01	logistic	409931	395	409536	0.413	0.010
536.8	Dyspepsia and other specified disorders of function of stomach	digestive	rs189447480	-0.054	0.178	0.947	7.606E-01	logistic	363851	1637	362214	0.226	0.010
687.1	Rash and other nonspecific skin eruption	dermatologic	rs189447480	-0.067	0.220	0.935	7.613E-01	logistic	413803	1087	412716	0.382	0.010
496.21	Obstructive chronic bronchitis	respiratory	rs189447480	0.029	0.096	1.029	7.614E-01	logistic	404163	5354	398809	0.339	0.010
320	Meningitis	neurological	rs189447480	0.114	0.380	1.121	7.645E-01	logistic	422374	303	422071	0.002	0.010
540.1	Appendicitis	digestive	rs189447480	-0.123	0.410	0.884	7.647E-01	logistic	421984	328	421656	0.012	0.010
187.2	Malignant neoplasm of testis	neoplasms	rs189447480	0.031	0.104	1.031	7.658E-01	logistic	184014	4620	179394	0.085	0.010
603.1	Hydrocele	genitourinary	rs189447480	0.095	0.319	1.099	7.659E-01	logistic	162225	447	161778	0.204	0.010
427.7	Tachycardia NOS	circulatory system	rs189447480	0.046	0.158	1.047	7.689E-01	logistic	373741	1893	371848	0.246	0.010
395	Heart valve disorders	circulatory system	rs189447480	-0.021	0.073	0.979	7.696E-01	logistic	411117	9703	401414	0.047	0.010
386	Vertiginous syndromes and other disorders of vestibular system	sense organs	rs189447480	-0.051	0.175	0.950	7.699E-01	logistic	411346	1681	409665	0.333	0.010
701.3	Circumscribed scleroderma	dermatologic	rs189447480	-0.146	0.502	0.864	7.712E-01	logistic	415410	224	415186	0.029	0.010
352.2	Facial nerve disorders [CN7]	neurological	rs189447480	0.075	0.260	1.078	7.717E-01	logistic	403092	677	402415	0.022	0.010
510	Other diseases of lung	respiratory	rs189447480	0.052	0.181	1.053	7.734E-01	logistic	421711	1427	420284	0.002	0.010
580.32	Nephritis and nephropathy with pathological lesion	genitourinary	rs189447480	-0.129	0.449	0.879	7.734E-01	logistic	381336	274	381062	0.261	0.010
967	Adverse effects of sedatives or other central nervous system depressants and anesthetics	injuries & poisonings	rs189447480	-0.144	0.502	0.866	7.740E-01	logistic	367398	225	367173	0.033	0.010
149.4	Cancer of larynx	neoplasms	rs189447480	-0.129	0.449	0.879	7.743E-01	logistic	420618	276	420342	0.004	0.010
378	Strabismus and other disorders of binocular eye movements	sense organs	rs189447480	0.063	0.220	1.065	7.745E-01	logistic	412884	956	411928	0.139	0.010
300	Anxiety disorders	mental disorders	rs189447480	0.020	0.071	1.020	7.752E-01	logistic	391438	9816	381622	0.002	0.010
78	Viral warts & HPV	infectious diseases	rs189447480	-0.144	0.503	0.866	7.752E-01	logistic	415426	222	415204	0.058	0.010
555.2	Ulcerative colitis	digestive	rs189447480	0.035	0.123	1.036	7.760E-01	logistic	337679	3194	334485	0.369	0.010
427.8	Sinoatrial node dysfunction (Bradycardia)	circulatory system	rs189447480	0.079	0.279	1.083	7.762E-01	logistic	372427	579	371848	0.228	0.010
634	Miscarriage; stillbirth	pregnancy complications	rs189447480	0.072	0.254	1.074	7.779E-01	logistic	223299	707	222592	0.305	0.010
535.6	Duodenitis	digestive	rs189447480	-0.048	0.170	0.953	7.783E-01	logistic	363993	1779	362214	0.228	0.010
348.2	Cerebral edema and compression of brain	neurological	rs189447480	-0.105	0.380	0.901	7.830E-01	logistic	399425	378	399047	0.068	0.010
695.7	Prurigo and Lichen	dermatologic	rs189447480	0.104	0.380	1.110	7.838E-01	logistic	405510	304	405206	0.041	0.010
690	Erythematosquamous dermatosis	dermatologic	rs189447480	-0.079	0.290	0.924	7.848E-01	logistic	405831	625	405206	0.039	0.010
117	Mycoses	infectious diseases	rs189447480	-0.120	0.449	0.887	7.887E-01	logistic	418652	272	418380	0.099	0.010
636	Early or threatened labor; hemorrhage in early pregnancy	pregnancy complications	rs189447480	0.077	0.293	1.080	7.926E-01	logistic	223122	530	222592	0.302	0.010
573.9	Abnormal serum enzyme levels	digestive	rs189447480	0.118	0.450	1.125	7.936E-01	logistic	400489	215	400274	0.138	0.010
452	Other venous embolism and thrombosis	circulatory system	rs189447480	-0.107	0.410	0.899	7.941E-01	logistic	381290	324	380966	0.041	0.010
38	Septicemia	infectious diseases	rs189447480	-0.022	0.084	0.978	7.956E-01	logistic	403876	7229	396647	0.140	0.010
707	Chronic ulcer of skin	dermatologic	rs189447480	0.026	0.101	1.026	7.961E-01	logistic	420904	4774	416130	0.005	0.010
707.2	Chronic ulcer of leg or foot	dermatologic	rs189447480	0.039	0.150	1.039	7.972E-01	logistic	418232	2102	416130	0.041	0.010
368.2	Diplopia and disorders of binocular vision	sense organs	rs189447480	-0.081	0.318	0.922	7.981E-01	logistic	416069	524	415545	0.175	0.010
702.2	Seborrheic keratosis	dermatologic	rs189447480	0.074	0.290	1.077	7.984E-01	logistic	413611	539	413072	0.034	0.010
261.4	Vitamin D deficiency	endocrine/metabolic	rs189447480	-0.038	0.150	0.963	8.009E-01	logistic	414357	2260	412097	0.033	0.010
593	Hematuria	genitourinary	rs189447480	-0.025	0.100	0.975	8.035E-01	logistic	381996	5069	376927	0.114	0.010
479	Other upper respiratory disease	respiratory	rs189447480	-0.088	0.355	0.916	8.041E-01	logistic	396937	420	396517	0.174	0.010
385	Other disorders of middle ear and mastoid	sense organs	rs189447480	0.094	0.380	1.099	8.042E-01	logistic	419023	307	418716	0.022	0.010
174	Breast cancer	neoplasms	rs189447480	0.017	0.070	1.017	8.058E-01	logistic	401305	10248	391057	0.008	0.010
145	Cancer of mouth	neoplasms	rs189447480	-0.068	0.279	0.934	8.061E-01	logistic	421016	674	420342	0.001	0.010
480.1	Bacterial pneumonia	respiratory	rs189447480	0.017	0.070	1.017	8.064E-01	logistic	407095	10295	396800	0.297	0.010
696.41	Psoriasis vulgaris	dermatologic	rs189447480	-0.037	0.155	0.963	8.095E-01	logistic	401395	2105	399290	0.001	0.010
371.3	Inflammation of eyelids	sense organs	rs189447480	-0.067	0.279	0.935	8.110E-01	logistic	411465	672	410793	0.236	0.010
279	Disorders involving the immune mechanism	endocrine/metabolic	rs189447480	-0.090	0.380	0.914	8.118E-01	logistic	423247	371	422876	0.032	0.010
368.1	Amblyopia	sense organs	rs189447480	0.072	0.303	1.075	8.120E-01	logistic	416041	496	415545	0.057	0.010
260.6	Anorexia	endocrine/metabolic	rs189447480	0.054	0.231	1.056	8.140E-01	logistic	412968	871	412097	0.139	0.010
427.41	Ventricular fibrillation and flutter	circulatory system	rs189447480	0.096	0.411	1.100	8.160E-01	logistic	372113	265	371848	0.223	0.010
709.3	Systemic sclerosis	dermatologic	rs189447480	0.102	0.450	1.108	8.202E-01	logistic	404118	219	403899	0.058	0.010
291.8	Alteration of consciousness	mental disorders	rs189447480	-0.085	0.380	0.918	8.224E-01	logistic	405343	370	404973	0.361	0.010
70.3	Viral hepatitis C	infectious diseases	rs189447480	0.091	0.411	1.095	8.255E-01	logistic	415468	264	415204	0.060	0.010

ST31; PheWAS results for the lead cis-pQTLs of the 4 proteins prioritized (ACOX1, FGF5, FURIN, and MST1).

SNP = Single Nucleotide Polymorphism; SE = Standard error HWE_p = P-value for Hardy-Weinberg equilibrium test

Phenotype	Description	Group	SNP	Beta	SE	Odds tatio	P-value	Type	n_total	n_cases	n_controls	HWE_p	allele_freq
242.1	Graves' disease	endocrine/metabolic	rs189447480	-0.083	0.380	0.920	8.275E-01	logistic	392598	368	392230	0.437	0.010
317.11	Alcoholic liver damage	mental disorders	rs189447480	0.042	0.198	1.043	8.301E-01	logistic	404493	1200	403293	0.170	0.010
282	Hereditary hemolytic anemias	hematopoietic	rs189447480	-0.107	0.503	0.899	8.322E-01	logistic	382667	218	382449	0.153	0.010
579.2	Splenomegaly	digestive	rs189447480	-0.061	0.290	0.940	8.325E-01	logistic	383919	616	383303	0.243	0.010
440	Atherosclerosis	circulatory system	rs189447480	-0.046	0.220	0.955	8.332E-01	logistic	406545	1076	405469	0.151	0.010
604.3	Peyronie's disease	genitourinary	rs189447480	0.094	0.450	1.099	8.344E-01	logistic	162002	224	161778	0.199	0.010
440.2	Atherosclerosis of the extremities	circulatory system	rs189447480	-0.050	0.244	0.951	8.384E-01	logistic	406343	874	405469	0.148	0.010
250.1	Type 1 diabetes	endocrine/metabolic	rs189447480	-0.030	0.146	0.971	8.385E-01	logistic	389041	2404	386637	0.170	0.010
426.3	Bundle branch block	circulatory system	rs189447480	0.024	0.117	1.024	8.397E-01	logistic	375377	3529	371848	0.144	0.010
636.3	Hemorrhage in early pregnancy	pregnancy complications	rs189447480	0.091	0.451	1.095	8.406E-01	logistic	222810	218	222592	0.296	0.010
690.1	Seborrheic dermatitis	dermatologic	rs189447480	-0.058	0.290	0.944	8.417E-01	logistic	405818	612	405206	0.038	0.010
433.21	Cerebral artery occlusion, with cerebral infarction	circulatory system	rs189447480	0.022	0.113	1.023	8.427E-01	logistic	406495	3810	402685	0.087	0.010
389	Hearing loss	sense organs	rs189447480	-0.017	0.084	0.984	8.439E-01	logistic	415421	7124	408297	0.049	0.010
801	Fracture of ankle and foot	injuries & poisonings	rs189447480	-0.055	0.279	0.947	8.445E-01	logistic	397040	662	396378	0.052	0.010
198.4	Secondary malignant neoplasm of liver	neoplasms	rs189447480	-0.019	0.096	0.981	8.447E-01	logistic	391482	5476	386006	0.552	0.010
803	Fracture of upper limb	injuries & poisonings	rs189447480	0.021	0.107	1.021	8.450E-01	logistic	400593	4215	396378	0.002	0.010
756	Other congenital musculoskeletal anomalies	congenital anomalies	rs189447480	0.043	0.220	1.043	8.464E-01	logistic	422256	974	421282	0.141	0.010
333.8	Other degenerative diseases of the basal ganglia	neurological	rs189447480	0.087	0.449	1.091	8.467E-01	logistic	399271	224	399047	0.067	0.010
687.4	Disturbance of skin sensation	dermatologic	rs189447480	0.035	0.184	1.036	8.476E-01	logistic	414113	1397	412716	0.389	0.010
292.3	Memory loss	mental disorders	rs189447480	0.058	0.303	1.060	8.477E-01	logistic	405478	505	404973	0.364	0.010
577.3	Cyst and pseudocyst of pancreas	digestive	rs189447480	-0.061	0.318	0.941	8.481E-01	logistic	418522	514	418008	0.013	0.010
159.4	Malignant neoplasm of retroperitoneum and peritoneum	neoplasms	rs189447480	-0.086	0.449	0.918	8.486E-01	logistic	394127	265	393862	0.116	0.010
527	Diseases of the salivary glands	digestive	rs189447480	0.086	0.450	1.090	8.488E-01	logistic	417933	223	417710	0.058	0.010
737.3	Kyphoscoliosis and scoliosis	musculoskeletal	rs189447480	0.045	0.244	1.046	8.543E-01	logistic	403443	789	402654	0.247	0.010
276	Disorders of fluid, electrolyte, and acid-base balance	endocrine/metabolic	rs189447480	0.010	0.057	1.011	8.546E-01	logistic	411277	15705	395572	0.008	0.010
241	Nontoxic nodular goiter	endocrine/metabolic	rs189447480	-0.047	0.260	0.954	8.557E-01	logistic	392996	766	392230	0.441	0.010
362.31	Separation of retinal layers	sense organs	rs189447480	-0.057	0.318	0.944	8.564E-01	logistic	398271	514	397757	0.090	0.010
770	Myalgia and myositis unspecified	symptoms	rs189447480	-0.030	0.166	0.971	8.573E-01	logistic	422525	1839	420686	0.014	0.010
569.1	Toxic gastroenteritis and colitis	digestive	rs189447480	0.066	0.380	1.068	8.618E-01	logistic	384157	318	383839	0.002	0.010
516	Abnormal sputum	respiratory	rs189447480	0.038	0.220	1.038	8.638E-01	logistic	421370	981	420389	0.001	0.010
427.4	Cardiac arrest and ventricular fibrillation	circulatory system	rs189447480	-0.040	0.237	0.961	8.654E-01	logistic	372755	907	371848	0.230	0.010
760	Back pain	symptoms	rs189447480	0.016	0.097	1.016	8.677E-01	logistic	414925	5193	409732	0.069	0.010
426.91	Cardiac pacemaker in situ	circulatory system	rs189447480	0.016	0.099	1.017	8.686E-01	logistic	376826	4978	371848	0.037	0.010
191.11	Cancer of brain	neoplasms	rs189447480	0.040	0.244	1.041	8.693E-01	logistic	421187	790	420397	0.006	0.010
715	Other inflammatory spondylopathies	musculoskeletal	rs189447480	-0.032	0.197	0.968	8.705E-01	logistic	409805	1298	408507	0.015	0.010
415.2	Chronic pulmonary heart disease	circulatory system	rs189447480	0.028	0.170	1.028	8.706E-01	logistic	414491	1653	412838	0.258	0.010
459.9	Circulatory disease NEC	circulatory system	rs189447480	0.008	0.048	1.008	8.710E-01	logistic	395548	22976	372572	0.336	0.010
728.71	Contracture of palmar fascia [Dupuytren's disease]	musculoskeletal	rs189447480	-0.028	0.173	0.973	8.721E-01	logistic	390879	1705	389174	0.221	0.010
686	Other local infections of skin and subcutaneous tissue	dermatologic	rs189447480	-0.035	0.220	0.965	8.721E-01	logistic	419554	1057	418497	0.200	0.010
362.3	Other nondiabetic retinopathy	sense organs	rs189447480	0.046	0.290	1.047	8.733E-01	logistic	398314	557	397757	0.092	0.010
227.3	Benign neoplasm of pituitary gland and craniopharyngeal duct (pouch	neoplasms	rs189447480	-0.060	0.380	0.941	8.738E-01	logistic	421248	361	420887	0.346	0.010
575	Other biliary tract disease	digestive	rs189447480	-0.018	0.117	0.982	8.741E-01	logistic	398734	3687	395047	0.338	0.010
276.1	Electrolyte imbalance	endocrine/metabolic	rs189447480	-0.011	0.070	0.989	8.756E-01	logistic	406083	10511	395572	0.176	0.010
530.1	Esophagitis, GERD and related diseases	digestive	rs189447480	-0.007	0.043	0.993	8.768E-01	logistic	385890	29102	356788	0.033	0.010
727	Other disorders of synovium, tendon, and bursa	musculoskeletal	rs189447480	-0.021	0.137	0.979	8.771E-01	logistic	391842	2668	389174	0.349	0.010
253	Disorders of the pituitary gland and its hypothalamic control	endocrine/metabolic	rs189447480	0.035	0.231	1.036	8.790E-01	logistic	416136	890	415246	0.037	0.010
599.4	Urinary incontinence	genitourinary	rs189447480	-0.021	0.136	0.980	8.793E-01	logistic	392806	2700	390106	0.037	0.010
433.2	Occlusion of cerebral arteries	circulatory system	rs189447480	0.017	0.112	1.017	8.801E-01	logistic	406563	3878	402685	0.086	0.010
695.9	Unspecified erythematous condition	dermatologic	rs189447480	0.057	0.380	1.059	8.806E-01	logistic	405524	318	405206	0.041	0.010
425.12	Other hypertrophic cardiomyopathy	circulatory system	rs189447480	0.061	0.411	1.063	8.818E-01	logistic	417044	274	416770	0.151	0.010
359	Muscular dystrophies and other myopathies	neurological	rs189447480	0.059	0.411	1.061	8.849E-01	logistic	419442	274	419168	0.089	0.010
275	Disorders of mineral metabolism	endocrine/metabolic	rs189447480	0.015	0.101	1.015	8.857E-01	logistic	420958	4773	416185	0.016	0.010
427.3	Other specified cardiac dysrhythmias	circulatory system	rs189447480	0.020	0.136	1.020	8.861E-01	logistic	374456	2608	371848	0.130	0.010
635.2	Antepartum hemorrhage, abruptio placentae, and placenta previa	pregnancy complications	rs189447480	-0.072	0.504	0.931	8.869E-01	logistic	222797	205	222592	0.295	0.010
736	Other acquired deformities of limbs	musculoskeletal	rs189447480	-0.033	0.231	0.968	8.870E-01	logistic	403605	951	402654	0.247	0.010
615	Endometriosis	genitourinary	rs189447480	-0.028	0.203	0.972	8.901E-01	logistic	218669	1231	217438	0.646	0.010
585.4	Chronic kidney disease, Stage I or II	genitourinary	rs189447480	-0.034	0.251	0.966	8.910E-01	logistic	381861	799	381062	0.267	0.010
458	Hypotension	circulatory system	rs189447480	0.011	0.078	1.011	8.919E-01	logistic	380778	8206	372572	0.491	0.010
394.7	Disease of tricuspid valve	circulatory system	rs189447480	0.036	0.269	1.037	8.932E-01	logistic	402072	658	401414	0.087	0.010
374.1	Ectropion or entropion	sense organs	rs189447480	0.035	0.260	1.035	8.941E-01	logistic	411493	700	410793	0.238	0.010
459	Other disorders of circulatory system	circulatory system	rs189447480	0.006	0.048	1.006	8.944E-01	logistic	395720	23148	372572	0.337	0.010
279.1	Immunity deficiency	endocrine/metabolic	rs189447480	0.050	0.380	1.051	8.958E-01	logistic	423199	323	422876	0.032	0.010
396	Abnormal heart sounds	circulatory system	rs189447480	0.029	0.225	1.030	8.965E-01	logistic	402357	943	401414	0.032	0.010
635	Hemorrhage during pregnancy; childbirth and postpartum	pregnancy complications	rs189447480	0.044	0.338	1.044	8.975E-01	logistic	223005	413	222592	0.299	0.010
272.1	Hyperlipidemia	endocrine/metabolic	rs189447480	-0.005	0.039	0.995	8.982E-01	logistic	394381	38616	355765	0.104	0.010
626.14	Irregular menstrual bleeding	genitourinary	rs189447480	-0.045	0.357	0.956	9.003E-01	logistic	193146	399	192747	0.912	0.010
530.11	GERD	digestive	rs189447480	-0.007	0.059	0.993	9.050E-01	logistic	371656	14868	356788	0.030	0.010
819	Skull and face fracture and other intercranial injury	injuries & poisonings	rs189447480	-0.037	0.318	0.963	9.062E-01	logistic	422666	501	422165	0.023	0.010
149	Cancer of larynx, pharynx, nasal cavities	neoplasms	rs189447480	0.028	0.237	1.028	9.067E-01	logistic	421191	849	420342	0.005	0.010
189.4	Malignant neoplasm of other urinary organs	neoplasms	rs189447480	-0.059	0.502	0.943	9.072E-01	logistic	417393	206	417187	0.179	0.010
626.1	Irregular menstrual cycle/bleeding	genitourinary	rs189447480	0.013	0.109	1.013	9.089E-01	logistic	196900	4153	192747	0.926	0.010

ST31; PheWAS results for the lead cis-pQTLs of the 4 proteins prioritized (ACOX1, FGF5, FURIN, and MST1).

SNP = Single Nucleotide Polymorphism; SE = Standard error HWE_p = P-value for Hardy-Weinberg equilibrium test

Phenotype	Description	Group	SNP	Beta	SE	Odds tatio	P-value	Type	n_total	n_cases	n_controls	HWE_p	allele_freq
738	Other acquired musculoskeletal deformity	musculoskeletal	rs189447480	0.021	0.190	1.022	9.102E-01	logistic	403982	1328	402654	0.253	0.010
195.1	Malignant neoplasm, other	neoplasms	rs189447480	-0.009	0.082	0.991	9.110E-01	logistic	393640	7634	386006	0.572	0.010
41.4	E. coli	infectious diseases	rs189447480	0.013	0.121	1.013	9.118E-01	logistic	399991	3344	396647	0.216	0.010
371.1	Uveitis, noninfectious or NOS	sense organs	rs189447480	-0.048	0.450	0.953	9.147E-01	logistic	411047	254	410793	0.232	0.010
577.1	Acute pancreatitis	digestive	rs189447480	0.017	0.161	1.017	9.148E-01	logistic	419858	1850	418008	0.006	0.010
550.2	Diaphragmatic hernia	digestive	rs189447480	-0.005	0.048	0.995	9.158E-01	logistic	388258	22864	365394	0.224	0.010
187	Cancer of other male genital organs	neoplasms	rs189447480	0.009	0.083	1.009	9.182E-01	logistic	186921	7527	179394	0.045	0.010
287.31	Primary thrombocytopenia	hematopoietic	rs189447480	0.030	0.303	1.031	9.203E-01	logistic	418072	519	417553	0.059	0.010
414	Other forms of chronic heart disease	circulatory system	rs189447480	0.011	0.109	1.011	9.229E-01	logistic	374670	4120	370550	0.044	0.010
965	Poisoning by analgesics, antipyretics, and antirheumatics	injuries & poisonings	rs189447480	-0.010	0.103	0.990	9.230E-01	logistic	371918	4745	367173	0.028	0.010
446	Polyarteritis nodosa and allied conditions	circulatory system	rs189447480	-0.020	0.205	0.980	9.233E-01	logistic	406656	1187	405469	0.153	0.010
509.8	Dependence on respirator [Ventilator] or supplemental oxygen	respiratory	rs189447480	-0.026	0.269	0.975	9.240E-01	logistic	391755	698	391057	0.456	0.010
272	Disorders of lipoid metabolism	endocrine/metabolic	rs189447480	-0.004	0.038	0.996	9.259E-01	logistic	394625	38860	355765	0.100	0.010
530.3	Stricture and stenosis of esophagus	digestive	rs189447480	-0.019	0.206	0.981	9.260E-01	logistic	357976	1188	356788	0.088	0.010
707.1	Decubitus ulcer	dermatologic	rs189447480	-0.012	0.129	0.988	9.266E-01	logistic	419126	2996	416130	0.088	0.010
594	Urinary calculus	genitourinary	rs189447480	0.008	0.092	1.008	9.282E-01	logistic	418195	5819	412376	0.028	0.010
803.3	Fracture of clavicle or scapula	injuries & poisonings	rs189447480	0.023	0.260	1.023	9.289E-01	logistic	397087	709	396378	0.050	0.010
198	Secondary malignant neoplasm	neoplasms	rs189447480	0.005	0.054	1.005	9.308E-01	logistic	403287	17281	386006	0.260	0.010
381	Otitis media and Eustachian tube disorders	sense organs	rs189447480	0.020	0.237	1.021	9.311E-01	logistic	419568	852	418716	0.015	0.010
939	Atopic/contact dermatitis due to other or unspecified	dermatologic	rs189447480	-0.014	0.159	0.986	9.317E-01	logistic	416950	1963	414987	0.057	0.010
586.2	Cyst of kidney, acquired	genitourinary	rs189447480	0.014	0.162	1.014	9.320E-01	logistic	382916	1854	381062	0.282	0.010
694	Dyschromia and Vitiligo	dermatologic	rs189447480	-0.034	0.410	0.966	9.330E-01	logistic	405505	299	405206	0.042	0.010
272.11	Hypercholesterolemia	endocrine/metabolic	rs189447480	-0.003	0.040	0.997	9.342E-01	logistic	390444	34679	355765	0.155	0.010
695.3	Rosacea	dermatologic	rs189447480	-0.037	0.449	0.964	9.342E-01	logistic	405456	250	405206	0.042	0.010
738.4	Acquired spondylolisthesis	musculoskeletal	rs189447480	0.017	0.201	1.017	9.343E-01	logistic	403846	1192	402654	0.251	0.010
523.3	Periodontitis (acute or chronic)	digestive	rs189447480	0.037	0.450	1.038	9.347E-01	logistic	409770	234	409536	0.410	0.010
740.9	Osteoarthrosis NOS	musculoskeletal	rs189447480	-0.005	0.058	0.995	9.360E-01	logistic	361594	15736	345858	0.024	0.010
195	Cancer, suspected or other	neoplasms	rs189447480	-0.006	0.082	0.994	9.368E-01	logistic	393669	7663	386006	0.573	0.010
386.1	Meniere's disease	sense organs	rs189447480	-0.022	0.279	0.978	9.375E-01	logistic	410308	643	409665	0.323	0.010
300.12	Agorophobia, social phobia, and panic disorder	mental disorders	rs189447480	0.021	0.269	1.021	9.378E-01	logistic	382286	664	381622	0.361	0.010
276.5	Hypovolemia	endocrine/metabolic	rs189447480	0.007	0.097	1.008	9.383E-01	logistic	400853	5281	395572	0.348	0.010
426.32	Left bundle branch block	circulatory system	rs189447480	-0.010	0.127	0.990	9.389E-01	logistic	374914	3066	371848	0.135	0.010
395.6	Heart valve replaced	circulatory system	rs189447480	0.011	0.140	1.011	9.394E-01	logistic	403924	2510	401414	0.134	0.010
721.8	Other allied disorders of spine	musculoskeletal	rs189447480	-0.038	0.502	0.963	9.400E-01	logistic	408871	201	408670	0.212	0.010
709	Diffuse diseases of connective tissue	dermatologic	rs189447480	-0.015	0.210	0.985	9.434E-01	logistic	405028	1129	403899	0.053	0.010
255.2	Adrenal hypofunction	endocrine/metabolic	rs189447480	-0.027	0.380	0.974	9.442E-01	logistic	415595	349	415246	0.029	0.010
255.21	Glucocorticoid deficiency	endocrine/metabolic	rs189447480	-0.027	0.380	0.974	9.442E-01	logistic	415595	349	415246	0.029	0.010
418.1	Precordial pain	circulatory system	rs189447480	-0.011	0.163	0.989	9.446E-01	logistic	417539	1859	415680	0.034	0.010
531.4	Peptic ulcer, site unspecified	digestive	rs189447480	-13.025	188.456	0.000	9.449E-01	logistic	411565	226	411339	0.129	0.010
252.2	Hypoparathyroidism	endocrine/metabolic	rs189447480	-13.028	189.066	0.000	9.451E-01	logistic	415471	225	415246	0.022	0.010
291	Other specified nonpsychotic and/or transient mental disorders	mental disorders	rs189447480	-0.024	0.356	0.976	9.451E-01	logistic	405371	398	404973	0.361	0.010
198.3	Secondary malignant neoplasm of digestive systems	neoplasms	rs189447480	0.009	0.134	1.009	9.452E-01	logistic	388741	2735	386006	0.528	0.010
427.21	Atrial fibrillation	circulatory system	rs189447480	-0.007	0.108	0.993	9.456E-01	logistic	376121	4273	371848	0.024	0.010
531.3	Duodenal ulcer	digestive	rs189447480	-0.014	0.210	0.986	9.458E-01	logistic	412469	1130	411339	0.145	0.010
276.11	Hyperosmolality and/or hypernatremia	endocrine/metabolic	rs189447480	0.017	0.260	1.018	9.465E-01	logistic	396286	714	395572	0.405	0.010
172.2	Other non-epithelial cancer of skin	neoplasms	rs189447480	-0.008	0.118	0.992	9.470E-01	logistic	410817	3609	407208	0.006	0.010
281.11	Pernicious anemia	hematopoietic	rs189447480	-0.017	0.260	0.983	9.476E-01	logistic	383186	737	382449	0.145	0.010
530.9	Heartburn	digestive	rs189447480	-13.290	204.330	0.000	9.481E-01	logistic	357038	250	356788	0.107	0.010
345	Epilepsy, recurrent seizures, convulsions	neurological	rs189447480	-0.007	0.110	0.993	9.500E-01	logistic	403151	4104	399047	0.116	0.010
726	Peripheral enthesopathies and allied syndromes	musculoskeletal	rs189447480	-0.004	0.069	0.996	9.522E-01	logistic	399760	10586	389174	0.438	0.010
159.3	Malignant neoplasm of gallbladder and extrahepatic bile ducts	neoplasms	rs189447480	0.023	0.380	1.023	9.522E-01	logistic	394194	332	393862	0.115	0.010
594.2	Calculus of lower urinary tract	genitourinary	rs189447480	0.017	0.290	1.017	9.539E-01	logistic	412949	573	412376	0.096	0.010
502	Postinflammatory pulmonary fibrosis	respiratory	rs189447480	-0.009	0.158	0.991	9.550E-01	logistic	393073	2016	391057	0.363	0.010
994	Sepsis and SIRS	injuries & poisonings	rs189447480	-0.005	0.086	0.995	9.551E-01	logistic	418535	6755	411780	0.023	0.010
994.2	Sepsis	injuries & poisonings	rs189447480	-0.005	0.086	0.995	9.551E-01	logistic	418535	6755	411780	0.023	0.010
386.3	Labyrinthitis	sense organs	rs189447480	-0.021	0.380	0.979	9.560E-01	logistic	410012	347	409665	0.319	0.010
597	Other disorders of urethra and urinary tract	genitourinary	rs189447480	-0.008	0.156	0.992	9.565E-01	logistic	403600	2046	401554	0.026	0.010
110	Dermatophytosis / Dermatomycosis	infectious diseases	rs189447480	0.024	0.450	1.025	9.569E-01	logistic	418617	237	418380	0.100	0.010
38.2	Gram positive septicemia	infectious diseases	rs189447480	0.015	0.279	1.015	9.576E-01	logistic	397268	621	396647	0.181	0.010
365.1	Open-angle glaucoma	sense organs	rs189447480	-0.009	0.178	0.991	9.578E-01	logistic	399325	1562	397763	0.104	0.010
528.1	Stomatitis and mucositis	digestive	rs189447480	0.023	0.450	1.023	9.591E-01	logistic	417947	237	417710	0.058	0.010
696	Psoriasis and related disorders	dermatologic	rs189447480	-0.007	0.130	0.993	9.594E-01	logistic	402206	2916	399290	0.013	0.010
722.9	Other and unspecified disc disorder	musculoskeletal	rs189447480	0.015	0.303	1.015	9.596E-01	logistic	409194	524	408670	0.216	0.010
204.12	Lymphoid leukemia, chronic	neoplasms	rs189447480	0.012	0.244	1.012	9.597E-01	logistic	416428	815	415613	0.057	0.010
384.4	Perforation of tympanic membrane	sense organs	rs189447480	-0.022	0.450	0.978	9.603E-01	logistic	418963	247	418716	0.024	0.010
250.11	Type 1 diabetes with ketoacidosis	endocrine/metabolic	rs189447480	-0.021	0.450	0.979	9.631E-01	logistic	386885	248	386637	0.144	0.010
516.1	Hemoptysis	respiratory	rs189447480	-0.011	0.231	0.989	9.634E-01	logistic	421320	931	420389	0.001	0.010
535	Gastritis and duodenitis	digestive	rs189447480	0.003	0.056	1.003	9.643E-01	logistic	378649	16435	362214	0.048	0.010
296.2	Depression	mental disorders	rs189447480	0.002	0.044	1.002	9.659E-01	logistic	408593	26971	381622	0.217	0.010
585.34	Chronic Kidney Disease, Stage IV	genitourinary	rs189447480	0.008	0.206	1.008	9.675E-01	logistic	382213	1151	381062	0.273	0.010
497	Bronchitis	respiratory	rs189447480	-0.016	0.410	0.985	9.697E-01	logistic	399105	296	398809	0.389	0.010

ST31; PheWAS results for the lead cis-pQTLs of the 4 proteins prioritized (ACOX1, FGF5, FURIN, and MST1).

SNP = Single Nucleotide Polymorphism; SE = Standard error HWE_p = P-value for Hardy-Weinberg equilibrium test

Phenotype	Description	Group	SNP	Beta	SE	Odds tatio	P-value	Type	n_total	n_cases	n_controls	HWE_p	allele_freq
262	Mineral deficiency NEC	endocrine/metabolic	rs189447480	-0.014	0.380	0.986	9.700E-01	logistic	412440	343	412097	0.131	0.010
559	Ileostomy status	digestive	rs189447480	-0.006	0.166	0.994	9.720E-01	logistic	336295	1810	334485	0.465	0.010
250.4	Abnormal glucose	endocrine/metabolic	rs189447480	-0.008	0.220	0.992	9.723E-01	logistic	387669	1032	386637	0.154	0.010
506	Empyema and pneumothorax	respiratory	rs189447480	-0.006	0.206	0.994	9.773E-01	logistic	392231	1174	391057	0.462	0.010
442.1	Aortic aneurysm	circulatory system	rs189447480	0.004	0.142	1.004	9.782E-01	logistic	407951	2482	405469	0.074	0.010
458.9	Hypotension NOS	circulatory system	rs189447480	0.003	0.106	1.003	9.790E-01	logistic	376953	4381	372572	0.549	0.010
696.4	Psoriasis	dermatologic	rs189447480	0.003	0.130	1.003	9.791E-01	logistic	402177	2887	399290	0.014	0.010
764	Sciatica	symptoms	rs189447480	-0.005	0.194	0.995	9.806E-01	logistic	419302	1316	417986	0.093	0.010
442.11	Abdominal aortic aneurysm	circulatory system	rs189447480	0.004	0.173	1.004	9.817E-01	logistic	407127	1658	405469	0.085	0.010
627	Menopausal and postmenopausal disorders	genitourinary	rs189447480	0.003	0.136	1.003	9.839E-01	logistic	195450	2703	192747	0.920	0.010
530.13	Barrett's esophagus	digestive	rs189447480	0.002	0.106	1.002	9.850E-01	logistic	361195	4407	356788	0.042	0.010
530.14	Reflux esophagitis	digestive	rs189447480	0.002	0.123	1.002	9.871E-01	logistic	360032	3244	356788	0.058	0.010
187.1	Malignant neoplasm of unspecified male genital organ	neoplasms	rs189447480	0.001	0.106	1.001	9.911E-01	logistic	183955	4561	179394	0.078	0.010
426.9	Cardiac pacemaker/device in situ	circulatory system	rs189447480	0.001	0.099	1.001	9.912E-01	logistic	376950	5102	371848	0.037	0.010
743.21	Pathologic fracture of vertebrae	musculoskeletal	rs189447480	0.003	0.279	1.003	9.915E-01	logistic	421861	627	421234	0.006	0.010
184.2	Cancer of other female genital organs (excluding uterus and ovary	neoplasms	rs189447480	-0.005	0.450	0.995	9.919E-01	logistic	205080	243	204837	0.319	0.010
180.3	Cervical intraepithelial neoplasia [CIN] [Cervical dysplasia	neoplasms	rs189447480	-0.003	0.319	0.997	9.928E-01	logistic	196385	481	195904	0.180	0.010
296	Mood disorders	mental disorders	rs189447480	0.000	0.044	1.000	9.934E-01	logistic	409186	27564	381622	0.223	0.010
204	Leukemia	neoplasms	rs189447480	0.001	0.128	1.001	9.949E-01	logistic	418621	3008	415613	0.085	0.010
191	Manlignant and unknown neoplasms of brain and nervous system	neoplasms	rs189447480	0.001	0.225	1.001	9.956E-01	logistic	421363	966	420397	0.008	0.010
279.11	Deficiency of humoral immunity	endocrine/metabolic	rs189447480	0.002	0.449	1.002	9.973E-01	logistic	423118	242	422876	0.031	0.010
415	Pulmonary heart disease	circulatory system	rs189447480	-0.001	0.170	0.999	9.976E-01	logistic	414538	1700	412838	0.257	0.010
8	Intestinal infection	infectious diseases	rs9823546	0.047	0.035	1.048	1.843E-01	logistic	419231	1915	417316	0.632	0.293
8.5	Bacterial enteritis	infectious diseases	rs9823546	0.042	0.044	1.043	3.373E-01	logistic	418567	1251	417316	0.664	0.292
8.52	Intestinal infection due to C. difficile	infectious diseases	rs9823546	0.029	0.060	1.029	6.290E-01	logistic	417984	668	417316	0.656	0.292
8.6	Viral Enteritis	infectious diseases	rs9823546	0.053	0.068	1.055	4.357E-01	logistic	417821	505	417316	0.649	0.292
38	Septicemia	infectious diseases	rs9823546	0.009	0.018	1.009	6.226E-01	logistic	403876	7229	396647	0.748	0.293
38.1	Gram negative septicemia	infectious diseases	rs9823546	0.130	0.086	1.139	1.280E-01	logistic	396961	314	396647	0.650	0.293
38.2	Gram positive septicemia	infectious diseases	rs9823546	0.035	0.062	1.036	5.693E-01	logistic	397268	621	396647	0.682	0.293
41	Bacterial infection NOS	infectious diseases	rs9823546	0.009	0.016	1.009	5.691E-01	logistic	406056	9409	396647	0.625	0.293
41.1	Staphylococcus infections	infectious diseases	rs9823546	0.022	0.032	1.022	5.002E-01	logistic	398981	2334	396647	0.665	0.293
41.11	Methicillin sensitive Staphylococcus aureus	infectious diseases	rs9823546	0.019	0.038	1.020	6.147E-01	logistic	398282	1635	396647	0.667	0.293
41.2	Streptococcus infection	infectious diseases	rs9823546	-0.047	0.040	0.954	2.473E-01	logistic	398155	1508	396647	0.658	0.292
41.4	E. coli	infectious diseases	rs9823546	0.074	0.027	1.077	5.162E-03	logistic	399991	3344	396647	0.635	0.293
53	Herpes zoster	infectious diseases	rs9823546	0.171	0.079	1.186	3.183E-02	logistic	415563	359	415204	0.692	0.293
70	Viral hepatitis	infectious diseases	rs9823546	-0.010	0.066	0.990	8.744E-01	logistic	415755	551	415204	0.710	0.292
70.3	Viral hepatitis C	infectious diseases	rs9823546	0.081	0.094	1.085	3.883E-01	logistic	415468	264	415204	0.701	0.292
70.4	Chronic hepatitis	infectious diseases	rs9823546	0.141	0.089	1.152	1.139E-01	logistic	415491	287	415204	0.698	0.293
78	Viral warts & HPV	infectious diseases	rs9823546	-0.037	0.105	0.964	7.258E-01	logistic	415426	222	415204	0.690	0.292
79	Viral infection	infectious diseases	rs9823546	-0.030	0.049	0.971	5.473E-01	logistic	416221	1017	415204	0.681	0.292
79.9	Viremia, NOS	infectious diseases	rs9823546	-0.111	0.065	0.895	9.039E-02	logistic	415798	594	415204	0.683	0.292
80	Postoperative infection	infectious diseases	rs9823546	-0.042	0.036	0.959	2.402E-01	logistic	414741	1897	412844	0.608	0.292
81	Infection/inflammation of internal prosthetic device; implant; and graft	infectious diseases	rs9823546	-0.014	0.035	0.986	6.957E-01	logistic	414783	1939	412844	0.561	0.293
110	Dermatophytosis / Dermatomycosis	infectious diseases	rs9823546	0.172	0.098	1.187	7.916E-02	logistic	418617	237	418380	0.603	0.292
112	Candidiasis	infectious diseases	rs9823546	0.041	0.039	1.042	2.930E-01	logistic	419935	1555	418380	0.612	0.292
117	Mycoses	infectious diseases	rs9823546	0.047	0.093	1.048	6.148E-01	logistic	418652	272	418380	0.563	0.292
117.4	Aspergillosis	infectious diseases	rs9823546	0.087	0.106	1.091	4.105E-01	logistic	418590	210	418380	0.562	0.292
145	Cancer of mouth	neoplasms	rs9823546	0.030	0.060	1.031	6.087E-01	logistic	421016	674	420342	0.688	0.292
145.2	Cancer of tongue	neoplasms	rs9823546	0.050	0.078	1.051	5.246E-01	logistic	420733	391	420342	0.690	0.292
149	Cancer of larynx, pharynx, nasal cavities	neoplasms	rs9823546	0.010	0.053	1.010	8.572E-01	logistic	421191	849	420342	0.656	0.292
149.1	Cancer of oropharynx	neoplasms	rs9823546	0.004	0.080	1.004	9.599E-01	logistic	420725	383	420342	0.672	0.292
149.4	Cancer of larynx	neoplasms	rs9823546	0.006	0.094	1.006	9.506E-01	logistic	420618	276	420342	0.679	0.292
150	Cancer of esophagus	neoplasms	rs9823546	-0.087	0.042	0.916	3.767E-02	logistic	395291	1429	393862	0.679	0.292
151	Cancer of stomach	neoplasms	rs9823546	-0.173	0.052	0.841	8.991E-04	logistic	394829	967	393862	0.732	0.292
153	Colorectal cancer	neoplasms	rs9823546	0.020	0.019	1.020	2.827E-01	logistic	377768	7048	370720	0.575	0.293
153.2	Colon cancer	neoplasms	rs9823546	0.027	0.023	1.028	2.376E-01	logistic	375260	4540	370720	0.498	0.293
153.3	Malignant neoplasm of rectum, rectosigmoid junction, and anus	neoplasms	rs9823546	0.013	0.029	1.013	6.501E-01	logistic	373609	2889	370720	0.636	0.293
155	Cancer of liver and intrahepatic bile duct	neoplasms	rs9823546	0.074	0.056	1.076	1.911E-01	logistic	394604	742	393862	0.728	0.292
155.1	Malignant neoplasm of liver, primary	neoplasms	rs9823546	0.154	0.084	1.166	6.664E-02	logistic	394187	325	393862	0.704	0.292
157	Pancreatic cancer	neoplasms	rs9823546	-0.010	0.042	0.990	8.050E-01	logistic	395256	1394	393862	0.740	0.292
158	Neoplasm of unspecified nature of digestive system	neoplasms	rs9823546	-0.122	0.082	0.886	1.403E-01	logistic	394238	376	393862	0.719	0.292
159	Malignant neoplasm of other and ill-defined sites within the digestive organs and peritoneum	neoplasms	rs9823546	0.106	0.052	1.111	4.043E-02	logistic	394736	874	393862	0.731	0.292
159.2	Malignant neoplasm of small intestine, including duodenum	neoplasms	rs9823546	0.150	0.095	1.161	1.138E-01	logistic	394117	255	393862	0.723	0.292
159.3	Malignant neoplasm of gallbladder and extrahepatic bile ducts	neoplasms	rs9823546	-0.016	0.086	0.984	8.538E-01	logistic	394194	332	393862	0.735	0.292
159.4	Malignant neoplasm of retroperitoneum and peritoneum	neoplasms	rs9823546	0.145	0.093	1.156	1.181E-01	logistic	394127	265	393862	0.716	0.292
165	Cancer within the respiratory system	neoplasms	rs9823546	-0.042	0.022	0.959	5.297E-02	logistic	422554	5325	417229	0.628	0.292
165.1	Cancer of bronchus; lung	neoplasms	rs9823546	-0.030	0.024	0.970	2.088E-01	logistic	421452	4223	417229	0.611	0.292
170	Cancer of bone and connective tissue	neoplasms	rs9823546	0.052	0.081	1.054	5.214E-01	logistic	423460	359	423101	0.645	0.292
170.2	Cancer of connective tissue	neoplasms	rs9823546	0.071	0.097	1.073	4.689E-01	logistic	423349	248	423101	0.658	0.292
172	Skin cancer	neoplasms	rs9823546	0.027	0.020	1.028	1.725E-01	logistic	413263	6055	407208	0.724	0.292
172.1	Melanomas of skin, dx or hx	neoplasms	rs9823546	0.011	0.036	1.011	7.660E-01	logistic	409038	1830	407208	0.752	0.292

ST31; PheWAS results for the lead cis-pQTLs of the 4 proteins prioritized (ACOX1, FGF5, FURIN, and MST1).

SNP = Single Nucleotide Polymorphism; SE = Standard error HWE_p = P-value for Hardy-Weinberg equilibrium test

Phenotype	Description	Group	SNP	Beta	SE	Odds tatio	P-value	Type	n_total	n_cases	n_controls	HWE_p	allele_freq
172.11	Melanomas of skin	neoplasms	rs9823546	0.011	0.036	1.011	7.660E-01	logistic	409038	1830	407208	0.752	0.292
172.2	Other non-epithelial cancer of skin	neoplasms	rs9823546	0.031	0.026	1.031	2.347E-01	logistic	410817	3609	407208	0.731	0.292
172.3	Carcinoma in situ of skin	neoplasms	rs9823546	0.101	0.075	1.107	1.794E-01	logistic	407616	408	407208	0.758	0.292
174	Breast cancer	neoplasms	rs9823546	-0.022	0.016	0.978	1.595E-01	logistic	401305	10248	391057	0.643	0.292
174.1	Breast cancer [female]	neoplasms	rs9823546	-0.023	0.016	0.977	1.443E-01	logistic	213896	10137	203759	0.756	0.293
174.11	Malignant neoplasm of female breast	neoplasms	rs9823546	-0.023	0.017	0.977	1.700E-01	logistic	212959	9200	203759	0.785	0.293
175	Acquired absence of breast	neoplasms	rs9823546	0.008	0.025	1.008	7.333E-01	logistic	206502	3992	202510	0.752	0.294
180	Cervical cancer and dysplasia	neoplasms	rs9823546	-0.022	0.031	0.978	4.864E-01	logistic	198422	2518	195904	0.811	0.294
180.1	Cervical cancer	neoplasms	rs9823546	-0.026	0.035	0.974	4.493E-01	logistic	197916	2012	195904	0.794	0.294
180.3	Cervical intraepithelial neoplasia [CIN] [Cervical dysplasia]	neoplasms	rs9823546	-0.008	0.071	0.992	9.113E-01	logistic	196385	481	195904	0.824	0.294
182	Malignant neoplasm of uterus	neoplasms	rs9823546	-0.040	0.044	0.961	3.674E-01	logistic	197217	1245	195972	0.770	0.294
184	Cancer of other female genital organs	neoplasms	rs9823546	-0.027	0.029	0.973	3.447E-01	logistic	207829	2992	204837	0.763	0.294
184.1	Malignant neoplasm of ovary and other uterine adnexa	neoplasms	rs9823546	-0.032	0.030	0.969	2.879E-01	logistic	207626	2789	204837	0.769	0.294
184.11	Malignant neoplasm of ovary	neoplasms	rs9823546	-0.041	0.030	0.960	1.742E-01	logistic	207552	2715	204837	0.757	0.294
184.2	Cancer of other female genital organs (excluding uterus and ovary)	neoplasms	rs9823546	-0.129	0.102	0.879	2.081E-01	logistic	205080	243	204837	0.759	0.294
185	Cancer of prostate	neoplasms	rs9823546	-0.007	0.016	0.993	6.429E-01	logistic	167540	11079	156461	0.178	0.291
187	Cancer of other male genital organs	neoplasms	rs9823546	-0.020	0.019	0.981	2.919E-01	logistic	186921	7527	179394	0.182	0.291
187.1	Malignant neoplasm of unspecified male genital organ	neoplasms	rs9823546	-0.012	0.024	0.989	6.234E-01	logistic	183955	4561	179394	0.021	0.291
187.2	Malignant neoplasm of testis	neoplasms	rs9823546	-0.015	0.023	0.985	5.229E-01	logistic	184014	4620	179394	0.004	0.291
189	Cancer of urinary organs (incl. kidney and bladder)	neoplasms	rs9823546	0.002	0.023	1.002	9.434E-01	logistic	421760	4573	417187	0.645	0.292
189.1	Cancer of kidney and renal pelvis	neoplasms	rs9823546	-0.037	0.045	0.964	4.124E-01	logistic	418392	1205	417187	0.682	0.292
189.11	Malignant neoplasm of kidney, except pelvis	neoplasms	rs9823546	-0.014	0.046	0.986	7.687E-01	logistic	418324	1137	417187	0.684	0.292
189.2	Cancer of bladder	neoplasms	rs9823546	0.010	0.027	1.010	7.142E-01	logistic	420532	3345	417187	0.646	0.292
189.21	Malignant neoplasm of bladder	neoplasms	rs9823546	0.013	0.028	1.013	6.415E-01	logistic	420210	3023	417187	0.664	0.292
189.4	Malignant neoplasm of other urinary organs	neoplasms	rs9823546	0.040	0.107	1.041	7.088E-01	logistic	417393	206	417187	0.673	0.292
191	Manlignant and unknown neoplasms of brain and nervous system	neoplasms	rs9823546	0.035	0.050	1.035	4.854E-01	logistic	421363	966	420397	0.579	0.292
191.1	Cancer of brain and nervous system	neoplasms	rs9823546	0.026	0.054	1.027	6.233E-01	logistic	421229	832	420397	0.584	0.292
191.11	Cancer of brain	neoplasms	rs9823546	0.032	0.055	1.032	5.654E-01	logistic	421187	790	420397	0.589	0.292
193	Thyroid cancer	neoplasms	rs9823546	-0.216	0.080	0.806	7.140E-03	logistic	421300	413	420887	0.649	0.292
195	Cancer, suspected or other	neoplasms	rs9823546	-0.013	0.018	0.988	4.892E-01	logistic	393669	7663	386006	0.536	0.292
195.1	Malignant neoplasm, other	neoplasms	rs9823546	-0.013	0.018	0.988	4.886E-01	logistic	393640	7634	386006	0.538	0.292
196	Radiotherapy	neoplasms	rs9823546	-0.001	0.039	0.999	9.795E-01	logistic	387589	1583	386006	0.604	0.292
197	Chemotherapy	neoplasms	rs9823546	0.003	0.015	1.003	8.599E-01	logistic	397532	11526	386006	0.594	0.292
198	Secondary malignant neoplasm	neoplasms	rs9823546	0.006	0.012	1.006	6.222E-01	logistic	403287	17281	386006	0.629	0.293
198.1	Secondary malignancy of lymph nodes	neoplasms	rs9823546	0.034	0.017	1.034	5.467E-02	logistic	394015	8009	386006	0.500	0.293
198.2	Secondary malignancy of respiratory organs	neoplasms	rs9823546	-0.012	0.023	0.988	5.931E-01	logistic	390707	4701	386006	0.685	0.292
198.3	Secondary malignant neoplasm of digestive systems	neoplasms	rs9823546	-0.011	0.030	0.989	7.196E-01	logistic	388741	2735	386006	0.645	0.292
198.4	Secondary malignant neoplasm of liver	neoplasms	rs9823546	-0.014	0.021	0.986	4.991E-01	logistic	391482	5476	386006	0.640	0.292
198.6	Secondary malignancy of bone	neoplasms	rs9823546	-0.041	0.023	0.959	7.044E-02	logistic	390762	4756	386006	0.628	0.292
198.7	Secondary malignant neoplasm of skin	neoplasms	rs9823546	0.088	0.094	1.092	3.502E-01	logistic	386268	262	386006	0.598	0.292
199	Neoplasm of uncertain behavior	neoplasms	rs9823546	-0.076	0.064	0.927	2.344E-01	logistic	386620	614	386006	0.559	0.292
200	Myeloproliferative disease	neoplasms	rs9823546	-0.029	0.041	0.971	4.752E-01	logistic	417103	1490	415613	0.536	0.292
200.1	Polycythemia vera	neoplasms	rs9823546	-0.057	0.069	0.945	4.107E-01	logistic	408705	523	408182	0.380	0.293
201	Hodgkin's disease	neoplasms	rs9823546	0.169	0.090	1.184	6.001E-02	logistic	415895	282	415613	0.553	0.292
202	Cancer of other lymphoid, histiocytic tissue	neoplasms	rs9823546	0.007	0.030	1.007	8.242E-01	logistic	418277	2664	415613	0.534	0.292
202.2	Non-Hodgkins lymphoma	neoplasms	rs9823546	0.007	0.030	1.007	8.242E-01	logistic	418277	2664	415613	0.534	0.292
202.21	Nodular lymphoma	neoplasms	rs9823546	0.018	0.072	1.018	7.993E-01	logistic	416077	464	415613	0.532	0.292
202.24	Large cell lymphoma	neoplasms	rs9823546	-0.002	0.047	0.998	9.710E-01	logistic	416717	1104	415613	0.528	0.292
204	Leukemia	neoplasms	rs9823546	-0.003	0.028	0.997	9.051E-01	logistic	418621	3008	415613	0.624	0.292
204.1	Lymphoid leukemia	neoplasms	rs9823546	-0.054	0.052	0.948	3.035E-01	logistic	416514	901	415613	0.589	0.292
204.12	Lymphoid leukemia, chronic	neoplasms	rs9823546	-0.082	0.055	0.921	1.387E-01	logistic	416428	815	415613	0.592	0.292
204.2	Myeloid leukemia	neoplasms	rs9823546	-0.026	0.057	0.974	6.440E-01	logistic	416360	747	415613	0.551	0.292
204.21	Myeloid leukemia, acute	neoplasms	rs9823546	0.000	0.064	1.000	9.958E-01	logistic	416195	582	415613	0.558	0.292
204.4	Multiple myeloma	neoplasms	rs9823546	0.040	0.046	1.041	3.825E-01	logistic	416757	1144	415613	0.559	0.292
208	Benign neoplasm of colon	neoplasms	rs9823546	-0.009	0.011	0.991	4.126E-01	logistic	395855	20019	375836	0.604	0.292
211	Benign neoplasm of other parts of digestive system	neoplasms	rs9823546	0.034	0.026	1.035	1.947E-01	logistic	400517	3467	397050	0.672	0.292
214	Lipoma	neoplasms	rs9823546	0.007	0.047	1.007	8.809E-01	logistic	415339	1112	414227	0.646	0.292
214.1	Lipoma of skin and subcutaneous tissue	neoplasms	rs9823546	0.002	0.057	1.002	9.664E-01	logistic	414958	731	414227	0.644	0.292
216	Benign neoplasm of skin	neoplasms	rs9823546	0.041	0.046	1.042	3.756E-01	logistic	415074	1116	413958	0.713	0.292
217	Vascular hamartomas and non-neoplastic nevi	neoplasms	rs9823546	-0.207	0.094	0.813	2.793E-02	logistic	414259	301	413958	0.654	0.292
217.1	Nevus, non-neoplastic	neoplasms	rs9823546	-0.198	0.098	0.820	4.357E-02	logistic	414233	275	413958	0.651	0.292
218	Benign neoplasm of uterus	neoplasms	rs9823546	0.045	0.029	1.046	1.234E-01	logistic	209861	2866	206995	0.781	0.294
218.1	Uterine leiomyoma	neoplasms	rs9823546	0.048	0.029	1.049	1.060E-01	logistic	209756	2761	206995	0.783	0.294
225	Benign neoplasm of brain and other parts of nervous system	neoplasms	rs9823546	0.000	0.054	1.000	9.985E-01	logistic	421233	836	420397	0.599	0.292
225.1	Benign neoplasm of brain, cranial nerves, meninges	neoplasms	rs9823546	-0.027	0.055	0.974	6.255E-01	logistic	421204	807	420397	0.595	0.292
227	Benign neoplasm of other endocrine glands and related structures	neoplasms	rs9823546	-0.004	0.058	0.996	9.467E-01	logistic	421596	709	420887	0.628	0.292
227.1	Benign neoplasm of adrenal gland	neoplasms	rs9823546	-0.052	0.098	0.949	5.930E-01	logistic	421144	257	420887	0.637	0.292
227.3	Benign neoplasm of pituitary gland and craniopharyngeal duct (pouch)	neoplasms	rs9823546	-0.002	0.082	0.998	9.809E-01	logistic	421248	361	420887	0.651	0.292
228	Hemangioma and lymphangioma, any site	neoplasms	rs9823546	0.018	0.067	1.018	7.880E-01	logistic	421655	529	421126	0.673	0.292
241	Nontoxic nodular goiter	endocrine/metabolic	rs9823546	0.037	0.056	1.037	5.089E-01	logistic	392996	766	392230	0.543	0.293
241.1	Nontoxic uninodular goiter	endocrine/metabolic	rs9823546	0.010	0.103	1.010	9.204E-01	logistic	392455	225	392230	0.533	0.293

ST31; PheWAS results for the lead cis-pQTLs of the 4 proteins prioritized (ACOX1, FGF5, FURIN, and MST1).

SNP = Single Nucleotide Polymorphism; SE = Standard error HWE_p = P-value for Hardy-Weinberg equilibrium test

Phenotype	Description	Group	SNP	Beta	SE	Odds tatio	P-value	Type	n_total	n_cases	n_controls	HWE_p	allele_freq
241.2	Nontoxic multinodular goiter	endocrine/metabolic	rs9823546	0.018	0.100	1.018	8.600E-01	logistic	392467	237	392230	0.513	0.293
242	Thyrotoxicosis with or without goiter	endocrine/metabolic	rs9823546	-0.075	0.040	0.928	6.049E-02	logistic	393812	1582	392230	0.478	0.293
242.1	Graves' disease	endocrine/metabolic	rs9823546	-0.151	0.084	0.860	7.159E-02	logistic	392598	368	392230	0.507	0.293
244	Hypothyroidism	endocrine/metabolic	rs9823546	-0.015	0.012	0.985	1.849E-01	logistic	411296	19066	392230	0.669	0.293
244.1	Secondary hypothyroidism	endocrine/metabolic	rs9823546	-0.013	0.045	0.987	7.642E-01	logistic	393443	1213	392230	0.520	0.293
244.4	Hypothyroidism NOS	endocrine/metabolic	rs9823546	-0.016	0.012	0.984	1.809E-01	logistic	410296	18066	392230	0.666	0.293
246	Other disorders of thyroid	endocrine/metabolic	rs9823546	0.074	0.088	1.077	3.986E-01	logistic	392532	302	392230	0.531	0.293
250	Diabetes mellitus	endocrine/metabolic	rs9823546	-0.007	0.010	0.993	4.549E-01	logistic	414239	27602	386637	0.701	0.293
250.1	Type 1 diabetes	endocrine/metabolic	rs9823546	-0.008	0.032	0.992	8.009E-01	logistic	389041	2404	386637	0.778	0.293
250.11	Type 1 diabetes with ketoacidosis	endocrine/metabolic	rs9823546	-0.027	0.099	0.974	7.875E-01	logistic	386885	248	386637	0.769	0.293
250.2	Type 2 diabetes	endocrine/metabolic	rs9823546	-0.006	0.010	0.994	5.288E-01	logistic	413206	26569	386637	0.698	0.293
250.23	Type 2 diabetes with ophthalmic manifestations	endocrine/metabolic	rs9823546	-0.028	0.035	0.972	4.236E-01	logistic	388590	1953	386637	0.785	0.293
250.4	Abnormal glucose	endocrine/metabolic	rs9823546	0.020	0.048	1.020	6.738E-01	logistic	387669	1032	386637	0.756	0.293
250.42	Other abnormal glucose	endocrine/metabolic	rs9823546	0.103	0.087	1.108	2.388E-01	logistic	386940	303	386637	0.751	0.293
251.1	Hypoglycemia	endocrine/metabolic	rs9823546	-0.009	0.040	0.991	8.207E-01	logistic	383028	1531	381497	0.724	0.293
252	Disorders of parathyroid gland	endocrine/metabolic	rs9823546	-0.001	0.044	0.999	9.749E-01	logistic	416485	1239	415246	0.692	0.292
252.1	Hyperparathyroidism	endocrine/metabolic	rs9823546	0.004	0.048	1.004	9.328E-01	logistic	416271	1025	415246	0.688	0.292
252.2	Hypoparathyroidism	endocrine/metabolic	rs9823546	-0.016	0.104	0.984	8.770E-01	logistic	415471	225	415246	0.686	0.292
253	Disorders of the pituitary gland and its hypothalamic contro	endocrine/metabolic	rs9823546	0.001	0.052	1.001	9.859E-01	logistic	416136	890	415246	0.621	0.292
253.2	Pituitary hypofunction	endocrine/metabolic	rs9823546	-0.041	0.093	0.960	6.586E-01	logistic	415534	288	415246	0.664	0.292
253.7	Other disorders of neurohypophysis	endocrine/metabolic	rs9823546	-0.036	0.084	0.965	6.706E-01	logistic	415594	348	415246	0.644	0.292
255	Disorders of adrenal glands	endocrine/metabolic	rs9823546	-0.030	0.056	0.970	5.900E-01	logistic	416034	788	415246	0.636	0.292
255.1	Adrenal hyperfunction	endocrine/metabolic	rs9823546	-0.063	0.111	0.939	5.707E-01	logistic	415447	201	415246	0.661	0.292
255.2	Adrenal hypofunction	endocrine/metabolic	rs9823546	0.004	0.083	1.004	9.592E-01	logistic	415595	349	415246	0.644	0.292
255.21	Glucocorticoid deficiency	endocrine/metabolic	rs9823546	0.004	0.083	1.004	9.592E-01	logistic	415595	349	415246	0.644	0.292
260	Protein-calorie malnutrition	endocrine/metabolic	rs9823546	0.109	0.079	1.115	1.660E-01	logistic	412473	376	412097	0.527	0.292
260.6	Anorexia	endocrine/metabolic	rs9823546	0.032	0.052	1.033	5.398E-01	logistic	412968	871	412097	0.530	0.292
261	Vitamin deficiency	endocrine/metabolic	rs9823546	-0.001	0.025	0.999	9.624E-01	logistic	416032	3935	412097	0.552	0.292
261.2	Vitamin B-complex deficiencies	endocrine/metabolic	rs9823546	0.011	0.038	1.011	7.765E-01	logistic	413809	1712	412097	0.519	0.292
261.4	Vitamin D deficiency	endocrine/metabolic	rs9823546	0.010	0.033	1.010	7.685E-01	logistic	414357	2260	412097	0.543	0.292
262	Mineral deficiency NEC	endocrine/metabolic	rs9823546	0.055	0.083	1.056	5.082E-01	logistic	412440	343	412097	0.527	0.292
270	Disorders of protein plasma/amino-acid transport and metabolism	endocrine/metabolic	rs9823546	0.030	0.055	1.030	5.903E-01	logistic	422490	788	421702	0.648	0.292
270.3	Disorders of plasma protein metabolism	endocrine/metabolic	rs9823546	0.035	0.056	1.036	5.334E-01	logistic	422449	747	421702	0.649	0.292
270.32	Paraproteinemia	endocrine/metabolic	rs9823546	0.026	0.062	1.027	6.728E-01	logistic	422319	617	421702	0.651	0.292
270.33	Amyloidosis	endocrine/metabolic	rs9823546	-0.068	0.087	0.934	4.337E-01	logistic	422032	330	421702	0.672	0.292
272	Disorders of lipoid metabolism	endocrine/metabolic	rs9823546	-0.020	0.009	0.980	1.704E-02	logistic	394625	38860	355765	0.534	0.293
272.1	Hyperlipidemia	endocrine/metabolic	rs9823546	-0.020	0.009	0.980	1.796E-02	logistic	394381	38616	355765	0.519	0.293
272.11	Hypercholesterolemia	endocrine/metabolic	rs9823546	-0.017	0.009	0.983	5.307E-02	logistic	390444	34679	355765	0.465	0.293
272.9	Unspecified disorder of lipoid metabolism	endocrine/metabolic	rs9823546	0.067	0.086	1.069	4.340E-01	logistic	356085	320	355765	0.509	0.293
274	Gout and other crystal arthropathies	endocrine/metabolic	rs9823546	-0.007	0.023	0.993	7.679E-01	logistic	420276	4486	415790	0.676	0.292
274.1	Gout	endocrine/metabolic	rs9823546	-0.005	0.024	0.995	8.421E-01	logistic	420024	4234	415790	0.666	0.292
274.2	Crystal arthropathies	endocrine/metabolic	rs9823546	-0.013	0.100	0.988	9.001E-01	logistic	416033	243	415790	0.674	0.292
275	Disorders of mineral metabolism	endocrine/metabolic	rs9823546	-0.030	0.023	0.970	1.865E-01	logistic	420958	4773	416185	0.674	0.292
275.1	Disorders of iron metabolism	hematopoietic	rs9823546	-0.071	0.048	0.932	1.406E-01	logistic	417274	1089	416185	0.599	0.292
275.3	Disorders of magnesium metabolism	endocrine/metabolic	rs9823546	-0.057	0.040	0.944	1.536E-01	logistic	417729	1544	416185	0.607	0.292
275.5	Disorders of calcium/phosphorus metabolism	endocrine/metabolic	rs9823546	-0.020	0.033	0.980	5.440E-01	logistic	418502	2317	416185	0.671	0.292
276	Disorders of fluid, electrolyte, and acid-base balance	endocrine/metabolic	rs9823546	0.000	0.013	1.000	9.887E-01	logistic	411277	15705	395572	0.434	0.293
276.1	Electrolyte imbalance	endocrine/metabolic	rs9823546	0.002	0.015	1.002	9.092E-01	logistic	406083	10511	395572	0.353	0.293
276.11	Hyperosmolality and/or hypernatremia	endocrine/metabolic	rs9823546	-0.104	0.060	0.902	8.209E-02	logistic	396286	714	395572	0.356	0.293
276.12	Hyposmolality and/or hyponatremia	endocrine/metabolic	rs9823546	0.011	0.022	1.011	6.285E-01	logistic	400551	4979	395572	0.381	0.293
276.13	Hyperpotassemia	endocrine/metabolic	rs9823546	-0.029	0.038	0.972	4.499E-01	logistic	397299	1727	395572	0.306	0.293
276.14	Hypopotassemia	endocrine/metabolic	rs9823546	-0.023	0.027	0.977	3.909E-01	logistic	399074	3502	395572	0.313	0.293
276.4	Acid-base balance disorder	endocrine/metabolic	rs9823546	-0.009	0.034	0.991	7.833E-01	logistic	397692	2120	395572	0.166	0.293
276.41	Acidosis	endocrine/metabolic	rs9823546	-0.017	0.037	0.984	6.529E-01	logistic	397376	1804	395572	0.203	0.293
276.42	Alkalosis	endocrine/metabolic	rs9823546	0.041	0.088	1.041	6.448E-01	logistic	395878	306	395572	0.276	0.293
276.5	Hypovolemia	endocrine/metabolic	rs9823546	-0.002	0.022	0.998	9.367E-01	logistic	400853	5281	395572	0.429	0.293
276.6	Fluid overload	endocrine/metabolic	rs9823546	0.028	0.038	1.028	4.614E-01	logistic	397239	1667	395572	0.328	0.293
277	Other disorders of metabolism	endocrine/metabolic	rs9823546	0.039	0.047	1.040	4.042E-01	logistic	421029	1088	419941	0.609	0.292
277.4	Disorders of bilirubin excretion	endocrine/metabolic	rs9823546	-0.047	0.073	0.954	5.159E-01	logistic	420403	462	419941	0.600	0.292
278	Overweight, obesity and other hyperalimentation	endocrine/metabolic	rs9823546	-0.030	0.013	0.970	1.881E-02	logistic	405726	15173	390553	0.583	0.293
278.1	Obesity	endocrine/metabolic	rs9823546	-0.029	0.013	0.971	2.371E-02	logistic	405667	15114	390553	0.591	0.293
279	Disorders involving the immune mechanism	endocrine/metabolic	rs9823546	0.144	0.078	1.155	6.638E-02	logistic	423247	371	422876	0.638	0.292
279.1	Immunity deficiency	endocrine/metabolic	rs9823546	0.129	0.084	1.138	1.247E-01	logistic	423199	323	422876	0.640	0.292
279.11	Deficiency of humoral immunity	endocrine/metabolic	rs9823546	0.158	0.097	1.171	1.025E-01	logistic	423118	242	422876	0.638	0.292
280	Iron deficiency anemias	hematopoietic	rs9823546	0.037	0.016	1.038	2.238E-02	logistic	391534	9085	382449	0.243	0.292
280.1	Iron deficiency anemias, unspecified or not due to blood loss	hematopoietic	rs9823546	0.039	0.017	1.040	1.863E-02	logistic	391238	8789	382449	0.265	0.292
280.2	Iron deficiency anemia secondary to blood loss (chronic	hematopoietic	rs9823546	-0.008	0.090	0.992	9.305E-01	logistic	382749	300	382449	0.025	0.292
281	Other deficiency anemia	hematopoietic	rs9823546	-0.024	0.043	0.976	5.808E-01	logistic	383766	1317	382449	0.038	0.292
281.1	Megaloblastic anemia	hematopoietic	rs9823546	-0.026	0.044	0.974	5.540E-01	logistic	383709	1260	382449	0.032	0.292
281.11	Pernicious anemia	hematopoietic	rs9823546	-0.042	0.058	0.959	4.717E-01	logistic	383186	737	382449	0.046	0.292
281.13	Folate-deficiency anemia	hematopoietic	rs9823546	-0.035	0.080	0.966	6.667E-01	logistic	382829	380	382449	0.023	0.292

ST31; PheWAS results for the lead cis-pQTLs of the 4 proteins prioritized (ACOX1, FGF5, FURIN, and MST1).

SNP = Single Nucleotide Polymorphism; SE = Standard error HWE_p = P-value for Hardy-Weinberg equilibrium test

Phenotype	Description	Group	SNP	Beta	SE	Odds tatio	P-value	Type	n_total	n_cases	n_controls	HWE_p	allele_freq
282	Hereditary hemolytic anemias	hematopoietic	rs9823546	-0.082	0.106	0.921	4.382E-01	logistic	382667	218	382449	0.065	0.292
283	Acquired hemolytic anemias	hematopoietic	rs9823546	0.124	0.107	1.132	2.472E-01	logistic	382649	200	382449	0.037	0.292
284	Aplastic anemia	hematopoietic	rs9823546	0.024	0.057	1.024	6.780E-01	logistic	383179	730	382449	0.030	0.292
285	Other anemias	hematopoietic	rs9823546	0.024	0.015	1.024	1.006E-01	logistic	393921	11472	382449	0.136	0.292
285.2	Anemia of chronic disease	hematopoietic	rs9823546	0.069	0.055	1.071	2.076E-01	logistic	383237	788	382449	0.010	0.292
285.22	Anemia in neoplastic disease	hematopoietic	rs9823546	0.126	0.083	1.135	1.300E-01	logistic	382780	331	382449	0.006	0.292
286	Coagulation defects	hematopoietic	rs9823546	-0.094	0.052	0.910	7.058E-02	logistic	418481	928	417553	0.620	0.293
286.1	Congenital coagulation defects	hematopoietic	rs9823546	-0.085	0.077	0.919	2.698E-01	logistic	417976	423	417553	0.589	0.293
286.12	Congenital deficiency of other clotting factors (including factor VII	hematopoietic	rs9823546	-0.010	0.102	0.990	9.237E-01	logistic	417788	235	417553	0.586	0.293
286.7	Other and unspecified coagulation defects	hematopoietic	rs9823546	-0.142	0.098	0.868	1.497E-01	logistic	417818	265	417553	0.588	0.293
287	Purpura and other hemorrhagic conditions	hematopoietic	rs9823546	-0.079	0.033	0.924	1.611E-02	logistic	419896	2343	417553	0.612	0.292
287.3	Thrombocytopenia	hematopoietic	rs9823546	-0.070	0.034	0.932	3.710E-02	logistic	419777	2224	417553	0.599	0.292
287.31	Primary thrombocytopenia	hematopoietic	rs9823546	-0.001	0.068	0.999	9.928E-01	logistic	418072	519	417553	0.574	0.293
288	Diseases of white blood cells	hematopoietic	rs9823546	-0.096	0.078	0.908	2.168E-01	logistic	408723	414	408309	0.517	0.292
288.1	Decreased white blood cell count	hematopoietic	rs9823546	-0.007	0.028	0.993	8.068E-01	logistic	411502	3193	408309	0.526	0.292
288.11	Neutropenia	hematopoietic	rs9823546	-0.007	0.028	0.993	8.068E-01	logistic	411502	3193	408309	0.526	0.292
289	Other diseases of blood and blood-forming organs	hematopoietic	rs9823546	-0.036	0.055	0.965	5.159E-01	logistic	409120	811	408309	0.511	0.292
289.3	Personal history of diseases of blood and blood-forming organs	hematopoietic	rs9823546	-0.015	0.091	0.985	8.685E-01	logistic	408604	295	408309	0.509	0.292
289.4	Lymphadenitis	hematopoietic	rs9823546	-0.082	0.038	0.921	3.304E-02	logistic	410013	1704	408309	0.540	0.292
289.5	Diseases of spleen	hematopoietic	rs9823546	0.033	0.056	1.033	5.586E-01	logistic	409070	761	408309	0.490	0.293
289.8	Polycythemia, secondary	hematopoietic	rs9823546	-0.070	0.073	0.932	3.367E-01	logistic	407605	466	407139	0.522	0.293
290	Delirium dementia and amnestic and other cognitive disorders	mental disorders	rs9823546	-0.018	0.019	0.982	3.258E-01	logistic	412158	7185	404973	0.661	0.292
290.1	Dementias	mental disorders	rs9823546	-0.008	0.024	0.992	7.493E-01	logistic	409256	4283	404973	0.623	0.292
290.11	Alzheimer's disease	mental disorders	rs9823546	-0.033	0.032	0.968	3.063E-01	logistic	407367	2394	404973	0.594	0.292
290.13	Senile dementia	mental disorders	rs9823546	-0.001	0.033	0.999	9.675E-01	logistic	407253	2280	404973	0.625	0.293
291	Other specified nonpsychotic and/or transient mental disorders	mental disorders	rs9823546	0.096	0.076	1.101	2.069E-01	logistic	405371	398	404973	0.574	0.293
291.8	Alteration of consciousness	mental disorders	rs9823546	0.111	0.079	1.118	1.598E-01	logistic	405343	370	404973	0.578	0.293
292	Neurological disorders	mental disorders	rs9823546	0.030	0.021	1.030	1.582E-01	logistic	410372	5399	404973	0.639	0.293
292.1	Aphasia/speech disturbance	mental disorders	rs9823546	0.106	0.047	1.112	2.398E-02	logistic	406020	1047	404973	0.626	0.293
292.3	Memory loss	mental disorders	rs9823546	0.046	0.069	1.047	5.065E-01	logistic	405478	505	404973	0.602	0.293
292.4	Altered mental status	mental disorders	rs9823546	0.011	0.027	1.011	6.923E-01	logistic	408434	3461	404973	0.589	0.293
292.6	Hallucinations	mental disorders	rs9823546	-0.020	0.084	0.980	8.144E-01	logistic	405317	344	404973	0.610	0.293
293	Symptoms involving head and neck	mental disorders	rs9823546	-0.012	0.072	0.988	8.728E-01	logistic	420175	464	419711	0.656	0.292
293.1	Swelling, mass, or lump in head and neck [Space-occupying lesion, intracranial NOS]	mental disorders	rs9823546	0.107	0.086	1.113	2.107E-01	logistic	420026	315	419711	0.674	0.292
295	Schizophrenia and other psychotic disorders	mental disorders	rs9823546	0.040	0.053	1.041	4.564E-01	logistic	382460	838	381622	0.559	0.293
295.1	Schizophrenia	mental disorders	rs9823546	0.067	0.059	1.069	2.613E-01	logistic	382292	670	381622	0.563	0.293
296	Mood disorders	mental disorders	rs9823546	-0.016	0.010	0.984	1.040E-01	logistic	409186	27564	381622	0.628	0.293
296.1	Bipolar	mental disorders	rs9823546	0.056	0.045	1.058	2.107E-01	logistic	382786	1164	381622	0.542	0.293
296.2	Depression	mental disorders	rs9823546	-0.019	0.010	0.981	5.347E-02	logistic	408593	26971	381622	0.615	0.293
296.22	Major depressive disorder	mental disorders	rs9823546	-0.024	0.013	0.977	6.890E-02	logistic	396820	15198	381622	0.491	0.293
297	Suicidal ideation or attempt	mental disorders	rs9823546	0.037	0.082	1.038	6.460E-01	logistic	381982	360	381622	0.565	0.293
297.2	Suicide or self-inflicted injury	mental disorders	rs9823546	-0.013	0.087	0.987	8.828E-01	logistic	381948	326	381622	0.556	0.293
300	Anxiety disorders	mental disorders	rs9823546	-0.032	0.016	0.969	4.650E-02	logistic	391438	9816	381622	0.465	0.293
300.1	Anxiety disorder	mental disorders	rs9823546	-0.030	0.017	0.970	6.911E-02	logistic	390805	9183	381622	0.477	0.293
300.12	Agorophobia, social phobia, and panic disorder	mental disorders	rs9823546	-0.070	0.061	0.932	2.507E-01	logistic	382286	664	381622	0.531	0.293
300.13	Phobia	mental disorders	rs9823546	0.025	0.063	1.025	6.962E-01	logistic	382219	597	381622	0.564	0.293
300.9	Posttraumatic stress disorder	mental disorders	rs9823546	-0.009	0.097	0.991	9.265E-01	logistic	381881	259	381622	0.549	0.293
301	Personality disorders	mental disorders	rs9823546	-0.010	0.084	0.990	9.072E-01	logistic	381968	346	381622	0.518	0.293
315	Develomental delays and disorders	mental disorders	rs9823546	0.051	0.059	1.052	3.895E-01	logistic	422717	680	422037	0.675	0.292
316	Substance addiction and disorders	mental disorders	rs9823546	0.105	0.081	1.111	1.944E-01	logistic	403645	352	403293	0.622	0.293
317	Alcohol-related disorders	mental disorders	rs9823546	-0.020	0.022	0.980	3.658E-01	logistic	408346	5053	403293	0.619	0.293
317.1	Alcoholism	mental disorders	rs9823546	-0.021	0.026	0.979	4.149E-01	logistic	407082	3789	403293	0.595	0.293
317.11	Alcoholic liver damage	mental disorders	rs9823546	-0.006	0.045	0.994	8.974E-01	logistic	404493	1200	403293	0.608	0.293
318	Tobacco use disorder	mental disorders	rs9823546	-0.009	0.022	0.991	6.873E-01	logistic	408298	5005	403293	0.659	0.293
320	Meningitis	neurological	rs9823546	-0.009	0.090	0.991	9.230E-01	logistic	422374	303	422071	0.638	0.292
323	Encephalitis	neurological	rs9823546	-0.059	0.084	0.942	4.826E-01	logistic	422420	349	422071	0.655	0.292
324	Other CNS infection and poliomyelitis	neurological	rs9823546	0.227	0.080	1.255	4.551E-03	logistic	422418	347	422071	0.637	0.292
327	Sleep disorders	neurological	rs9823546	-0.087	0.067	0.916	1.906E-01	logistic	413949	565	413384	0.756	0.292
327.3	Sleep apnea	neurological	rs9823546	0.006	0.021	1.006	7.737E-01	logistic	418773	5389	413384	0.693	0.292
331	Other cerebral degenerations	neurological	rs9823546	-0.001	0.038	0.999	9.767E-01	logistic	400699	1652	399047	0.563	0.293
331.1	Hydrocephalus	neurological	rs9823546	-0.008	0.062	0.992	8.986E-01	logistic	399668	621	399047	0.532	0.292
331.9	Cerebral degeneration, unspecified	neurological	rs9823546	0.013	0.050	1.013	8.005E-01	logistic	400009	962	399047	0.587	0.293
332	Parkinson's disease	neurological	rs9823546	0.029	0.029	1.029	3.144E-01	logistic	401966	2919	399047	0.503	0.293
333	Extrapyramidal disease and abnormal movement disorders	neurological	rs9823546	0.004	0.044	1.004	9.359E-01	logistic	400270	1223	399047	0.552	0.293
333.1	Essential tremor	neurological	rs9823546	0.006	0.067	1.006	9.256E-01	logistic	399579	532	399047	0.554	0.292
333.4	Torsion dystonia	neurological	rs9823546	-0.138	0.092	0.871	1.343E-01	logistic	399349	302	399047	0.536	0.292
333.8	Other degenerative diseases of the basal ganglia	neurological	rs9823546	0.096	0.102	1.101	3.456E-01	logistic	399271	224	399047	0.578	0.293
334	Degenerative disease of the spinal cord	neurological	rs9823546	-0.026	0.038	0.974	4.846E-01	logistic	400789	1742	399047	0.547	0.292
334.2	Anterior horn cell disease	neurological	rs9823546	-0.078	0.075	0.925	2.940E-01	logistic	399497	450	399047	0.544	0.292
335	Multiple sclerosis	neurological	rs9823546	0.022	0.040	1.022	5.828E-01	logistic	400514	1467	399047	0.564	0.293
337	Disorders of the autonomic nervous system	neurological	rs9823546	-0.111	0.085	0.895	1.943E-01	logistic	399396	349	399047	0.557	0.292

ST31; PheWAS results for the lead cis-pQTLs of the 4 proteins prioritized (ACOX1, FGF5, FURIN, and MST1).

SNP = Single Nucleotide Polymorphism; SE = Standard error HWE_p = P-value for Hardy-Weinberg equilibrium test

Phenotype	Description	Group	SNP	Beta	SE	Odds tatio	P-value	Type	n_total	n_cases	n_controls	HWE_p	allele_freq
339	Other headache syndromes	neurological	rs9823546	-0.031	0.024	0.969	2.010E-01	logistic	409356	4169	405187	0.564	0.293
340	Migraine	neurological	rs9823546	-0.017	0.031	0.983	5.811E-01	logistic	407732	2545	405187	0.537	0.293
342	Hemiplegia	neurological	rs9823546	-0.026	0.032	0.974	4.080E-01	logistic	401449	2402	399047	0.638	0.292
344	Other paralytic syndromes	neurological	rs9823546	0.014	0.060	1.014	8.204E-01	logistic	399714	667	399047	0.615	0.293
345	Epilepsy, recurrent seizures, convulsions	neurological	rs9823546	-0.024	0.025	0.976	3.296E-01	logistic	403151	4104	399047	0.627	0.292
345.1	Epilepsy	neurological	rs9823546	0.108	0.065	1.114	9.662E-02	logistic	399597	550	399047	0.576	0.293
345.11	Generalized convulsive epilepsy	neurological	rs9823546	0.049	0.078	1.050	5.279E-01	logistic	399441	394	399047	0.568	0.293
348	Other conditions of brain	neurological	rs9823546	0.048	0.060	1.049	4.174E-01	logistic	399717	670	399047	0.510	0.293
348.2	Cerebral edema and compression of brain	neurological	rs9823546	0.051	0.079	1.052	5.204E-01	logistic	399425	378	399047	0.555	0.293
350	Abnormal movement	neurological	rs9823546	-0.041	0.045	0.960	3.663E-01	logistic	421410	1200	420210	0.683	0.292
350.1	Abnormal involuntary movements	neurological	rs9823546	0.021	0.055	1.021	7.059E-01	logistic	421010	800	420210	0.659	0.293
350.3	Lack of coordination	neurological	rs9823546	-0.158	0.095	0.854	9.464E-02	logistic	420499	289	420210	0.672	0.292
351	Other peripheral nerve disorders	neurological	rs9823546	-0.058	0.019	0.944	2.770E-03	logistic	409126	6711	402415	0.566	0.293
352	Disorders of other cranial nerves	neurological	rs9823546	0.060	0.044	1.062	1.744E-01	logistic	403637	1222	402415	0.618	0.293
352.1	Trigeminal nerve disorders [CN5]	neurological	rs9823546	0.092	0.068	1.096	1.802E-01	logistic	402912	497	402415	0.637	0.293
352.2	Facial nerve disorders [CN7]	neurological	rs9823546	0.030	0.059	1.031	6.082E-01	logistic	403092	677	402415	0.591	0.293
353	Nerve root and plexus disorders	neurological	rs9823546	-0.131	0.092	0.877	1.513E-01	logistic	402720	305	402415	0.616	0.293
357	Inflammatory and toxic neuropathy	neurological	rs9823546	0.021	0.035	1.021	5.494E-01	logistic	421163	1995	419168	0.665	0.292
358	Myoneural disorders	neurological	rs9823546	0.043	0.087	1.044	6.208E-01	logistic	419485	317	419168	0.669	0.292
358.1	Myasthenia gravis	neurological	rs9823546	0.036	0.090	1.037	6.874E-01	logistic	419463	295	419168	0.667	0.292
359	Muscular dystrophies and other myopathies	neurological	rs9823546	-0.144	0.097	0.866	1.383E-01	logistic	419442	274	419168	0.658	0.292
361	Retinal detachments and defects	sense organs	rs9823546	-0.008	0.069	0.992	9.037E-01	logistic	398276	513	397763	0.635	0.292
362	Other retinal disorders	sense organs	rs9823546	0.005	0.016	1.005	7.483E-01	logistic	407698	9941	397757	0.625	0.292
362.2	Degeneration of macula and posterior pole of retina	sense organs	rs9823546	0.010	0.017	1.010	5.486E-01	logistic	406927	9170	397757	0.622	0.292
362.29	Macular degeneration (senile) of retina NOS	sense organs	rs9823546	0.043	0.025	1.044	8.224E-02	logistic	401665	3908	397757	0.606	0.292
362.3	Other nondiabetic retinopathy	sense organs	rs9823546	-0.036	0.067	0.965	5.871E-01	logistic	398314	557	397757	0.624	0.292
362.31	Separation of retinal layers	sense organs	rs9823546	-0.026	0.069	0.974	7.078E-01	logistic	398271	514	397757	0.615	0.292
363	Chorioretinal inflammations, scars, and other disorders of choroid	sense organs	rs9823546	0.071	0.101	1.074	4.800E-01	logistic	397993	230	397763	0.628	0.292
364	Corneal opacity and other disorders of cornea	sense organs	rs9823546	0.024	0.037	1.025	5.110E-01	logistic	399513	1750	397763	0.629	0.292
364.4	Corneal degenerations	sense organs	rs9823546	0.007	0.046	1.007	8.797E-01	logistic	398917	1154	397763	0.635	0.292
364.5	Corneal dystrophy	sense organs	rs9823546	0.067	0.078	1.069	3.900E-01	logistic	398152	389	397763	0.604	0.292
365	Glaucoma	sense organs	rs9823546	0.024	0.018	1.024	1.854E-01	logistic	405209	7446	397763	0.607	0.292
365.1	Open-angle glaucoma	sense organs	rs9823546	0.107	0.039	1.113	5.491E-03	logistic	399325	1562	397763	0.644	0.292
365.2	Primary angle-closure glaucoma	sense organs	rs9823546	0.005	0.060	1.005	9.389E-01	logistic	398441	678	397763	0.623	0.292
366	Cataract	sense organs	rs9823546	0.023	0.009	1.023	1.162E-02	logistic	406937	35259	371678	0.507	0.292
366.2	Senile cataract	sense organs	rs9823546	0.044	0.017	1.045	7.332E-03	logistic	380814	9136	371678	0.001	0.292
367	Disorders of refraction and accommodation; blindness and low vision	sense organs	rs9823546	0.018	0.020	1.018	3.656E-01	logistic	417170	6164	411006	0.636	0.292
367.1	Myopia	sense organs	rs9823546	0.093	0.042	1.098	2.590E-02	logistic	412331	1325	411006	0.612	0.292
367.2	Astigmatism	sense organs	rs9823546	-0.008	0.027	0.992	7.517E-01	logistic	414418	3412	411006	0.579	0.292
367.8	Hypermetropia	sense organs	rs9823546	-0.005	0.093	0.995	9.580E-01	logistic	411285	279	411006	0.618	0.292
367.9	Blindness and low vision	sense organs	rs9823546	-0.007	0.053	0.993	8.944E-01	logistic	411881	875	411006	0.583	0.292
368	Visual disturbances	sense organs	rs9823546	0.034	0.032	1.034	2.961E-01	logistic	417843	2298	415545	0.593	0.292
368.1	Amblyopia	sense organs	rs9823546	0.079	0.069	1.083	2.483E-01	logistic	416041	496	415545	0.563	0.292
368.2	Diplopia and disorders of binocular vision	sense organs	rs9823546	0.060	0.067	1.062	3.685E-01	logistic	416069	524	415545	0.587	0.292
368.4	Visual field defects	sense organs	rs9823546	-0.049	0.075	0.953	5.154E-01	logistic	415988	443	415545	0.531	0.292
368.9	Subjective visual disturbances	sense organs	rs9823546	-0.005	0.069	0.995	9.438E-01	logistic	416057	512	415545	0.532	0.292
368.91	Psychophysical visual disturbances	sense organs	rs9823546	0.059	0.095	1.061	5.325E-01	logistic	415808	263	415545	0.537	0.292
369	Infection of the eye	sense organs	rs9823546	-0.119	0.078	0.888	1.285E-01	logistic	411210	417	410793	0.658	0.292
369.5	Conjunctivitis, infectious	sense organs	rs9823546	-0.215	0.114	0.806	5.856E-02	logistic	410999	206	410793	0.659	0.292
371	Inflammation of the eye	sense organs	rs9823546	0.015	0.048	1.015	7.561E-01	logistic	411823	1030	410793	0.683	0.292
371.1	Uveitis, noninfectious or NOS	sense organs	rs9823546	0.054	0.097	1.056	5.726E-01	logistic	411047	254	410793	0.668	0.292
371.3	Inflammation of eyelids	sense organs	rs9823546	-0.024	0.060	0.976	6.863E-01	logistic	411465	672	410793	0.671	0.292
374	Other disorders of eyelids	sense organs	rs9823546	0.019	0.033	1.019	5.574E-01	logistic	413055	2262	410793	0.679	0.292
374.1	Ectropion or entropion	sense organs	rs9823546	0.091	0.058	1.095	1.158E-01	logistic	411493	700	410793	0.677	0.292
374.3	Ptosis of eyelid	sense organs	rs9823546	-0.018	0.045	0.982	6.831E-01	logistic	411997	1204	410793	0.688	0.292
375	Disorders of lacrimal system	sense organs	rs9823546	0.013	0.041	1.013	7.426E-01	logistic	413379	1451	411928	0.563	0.293
375.2	Epiphora	sense organs	rs9823546	0.057	0.085	1.059	4.973E-01	logistic	412257	329	411928	0.599	0.293
377	Disorders of optic nerve and visual pathways	sense organs	rs9823546	-0.195	0.085	0.823	2.167E-02	logistic	412293	365	411928	0.565	0.292
378	Strabismus and other disorders of binocular eye movements	sense organs	rs9823546	0.076	0.050	1.079	1.267E-01	logistic	412884	956	411928	0.595	0.293
[truncated: 86,381 more chars]
